# Elucidating the Causal Impact of Dietary Factors on Rheumatoid Arthritis: Insights From Multivariable Mendelian Randomization Analysis

**DOI:** 10.1002/fsn3.4630

**Published:** 2024-12-01

**Authors:** Yun Lu, Qiang Su, Zhongbo Xie, Jiang Liang

**Affiliations:** ^1^ Guizhou University of Traditional Chinese Medicine Guiyang Guizhou China; ^2^ Department of Rheumatology and Hematology First Affiliated Hospital of Guizhou University of Traditional Chinese Medicine Guiyang Guizhou China; ^3^ Shandong Academy of Chinese Medicine Jinan Shandong China

**Keywords:** causal relationships, dietary, Mendelian randomization, multivariable Mendelian randomization, rheumatoid arthritis

## Abstract

Rheumatoid arthritis (RA) is a common chronic autoimmune disorder with an incompletely elucidated pathogenesis. Emerging research indicates that dietary factors may significantly influence the onset and progression of RA. Nevertheless, the causal relationship between dietary habits and RA remains ambiguous. This investigation employed a multivariable Mendelian randomization (MVMR) methodology to rigorously assess the potential causal effects of various dietary factors on RA risk. This study utilized genome‐wide association study (GWAS) summary‐level data, encompassing dietary preferences (*n* = 113,425–159,579), and RA data (*n* = 302,614) from the most recent FinnGen database. The primary causal analysis was conducted using inverse‐variance weighting (IVW), complemented by MR‐Egger, weighted median, Bayesian weighted Mendelian randomization, and Robust Adjusted Profile Score (RAPS) methodologies. Sensitivity analyses incorporated Cochran's *Q* test, MR‐Egger intercept test, MR‐PRESSO, and leave‐one‐out analysis. Steiger tests were employed to evaluate the identified dietary preferences. MVMR was utilized to assess the direct impact of dietary factors on RA. The study identified significant associations between RA and nine dietary preferences: F‐lentils/beans liking (Fle), chips liking (Chips), coffee difference liking (CD), coffee with sugar liking (CWS), milk chocolate liking (MC), coriander liking (Coriander), pollock liking (Pollock), soft cheese liking (SC), and blue cheese liking (BC). The MVMR analysis indicated that genetically predicted coriander and MC have a direct impact on RA, independent of other dietary factors. This study presents novel causal evidence suggesting that dietary preferences may impact the risk of RA. Specifically, a reduction in the consumption of milk chocolate and coriander may contribute to the prevention and alleviation of RA. However, additional research is required to elucidate the underlying mechanisms and to validate these findings across diverse populations.

## Graphical abstract

This Mendelian randomization study investigated causal relationships between dietary preferences and rheumatoid arthritis (RA) risk using genome‐wide association study data. Nine dietary preferences were significantly associated with RA risk. Notably, multivariable Mendelian randomization analysis revealed that genetically predicted preferences for coriander and milk chocolate independently increased RA risk, suggesting their potential role in RA pathogenesis and prevention.

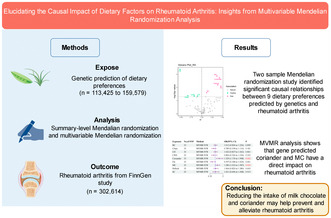

## 1 Introduction

Rheumatoid arthritis (RA) is a persistent autoimmune disorder marked by disturbances in various metabolic pathways (Di Matteo, Bathon, and Emery 2023). Key features of RA encompass immune cell infiltration, systemic inflammatory responses, neovascularization, and synovial hyperplasia (Hanlon et al. 2022). These pathological processes culminate in the erosion and degradation of bone and cartilage, ultimately leading to functional impairment and disability in affected individuals (Aletaha and Smolen 2018). Globally, approximately 0.46% of the population is affected by RA annually, with women being three times more likely to develop the condition compared with men (Almutairi et al. 2021; Finckh et al. 2022). The disease imposes a substantial economic burden and adversely affects patients' mental health and quality of life. These factors underscore the critical importance of advancing research focused on the early diagnosis, treatment, and intervention of RA. Recent research has indicated a significant association between dietary habits and the onset and progression of RA (Asoudeh et al. 2022). For example, a survey revealed that dietary factors impacted symptoms in approximately 25% of individuals with RA (Jiménez‐Sánchez et al. 2022). Additionally, the Mediterranean diet, which is widely recognized as a healthful eating pattern, has been reported to confer protective effects against RA (Bahrampour and Clark 2022).

Mendelian randomization (MR) studies have become prevalent in investigations of disease etiology (Smith and Ebrahim 2003). In the absence of randomized controlled trials (RCTs), MR stands out as a particularly persuasive approach for investigating causal associations between exposures and outcomes (Zuccolo and Holmes 2017). Through the utilization of genetic variants as instrumental variables (IVs) for exposures, such as specific dietary preferences, MR analysis can enhance the robustness of causal inference by mitigating unobserved confounding and attenuating reverse causation (Davies, Holmes, and Davey Smith 2018; Skrivankova et al. 2021). The IVs approach employed in this study closely resembles RCTs by randomly assigning genetic variations at conception. Under specific assumptions, the MR framework also emulates a randomized controlled trial (Richmond and Davey Smith 2022). In contrast to traditional epidemiological methods, this approach minimizes the impact of confounding factors such as gender and age, thereby facilitating more reliable causal inference (Burgess, Butterworth, and Thompson 2013). Additionally, the risk of reverse causation is reduced in MR studies due to the formation of genotypes before the onset of disease.

Understanding the impact of diet on RA holds significant potential for enhancing clinical treatment strategies and promoting early preventive measures. However, there is a notable paucity of MR studies that explore the causal relationship between dietary patterns and RA. Consequently, we undertook this MR analysis to investigate the potential association between diet and RA risk. The causal relationship between dietary preferences and RA warrants further elucidation. In this study, we utilized MR analysis, leveraging summary data from genome‐wide association studies (GWAS), to conduct a comprehensive investigation into the causal effects of 187 dietary factors on RA. Furthermore, we employed multivariable Mendelian randomization (MVMR) to evaluate the independent contributions of specific exposure factors, distinct from other dietary variables. The principal objective of this study was to identify dietary preference factors associated with RA and to elucidate the underlying biological mechanisms involved.

## 2 Materials and Methods

### 2.1 Study Design

**FIGURE 1:**
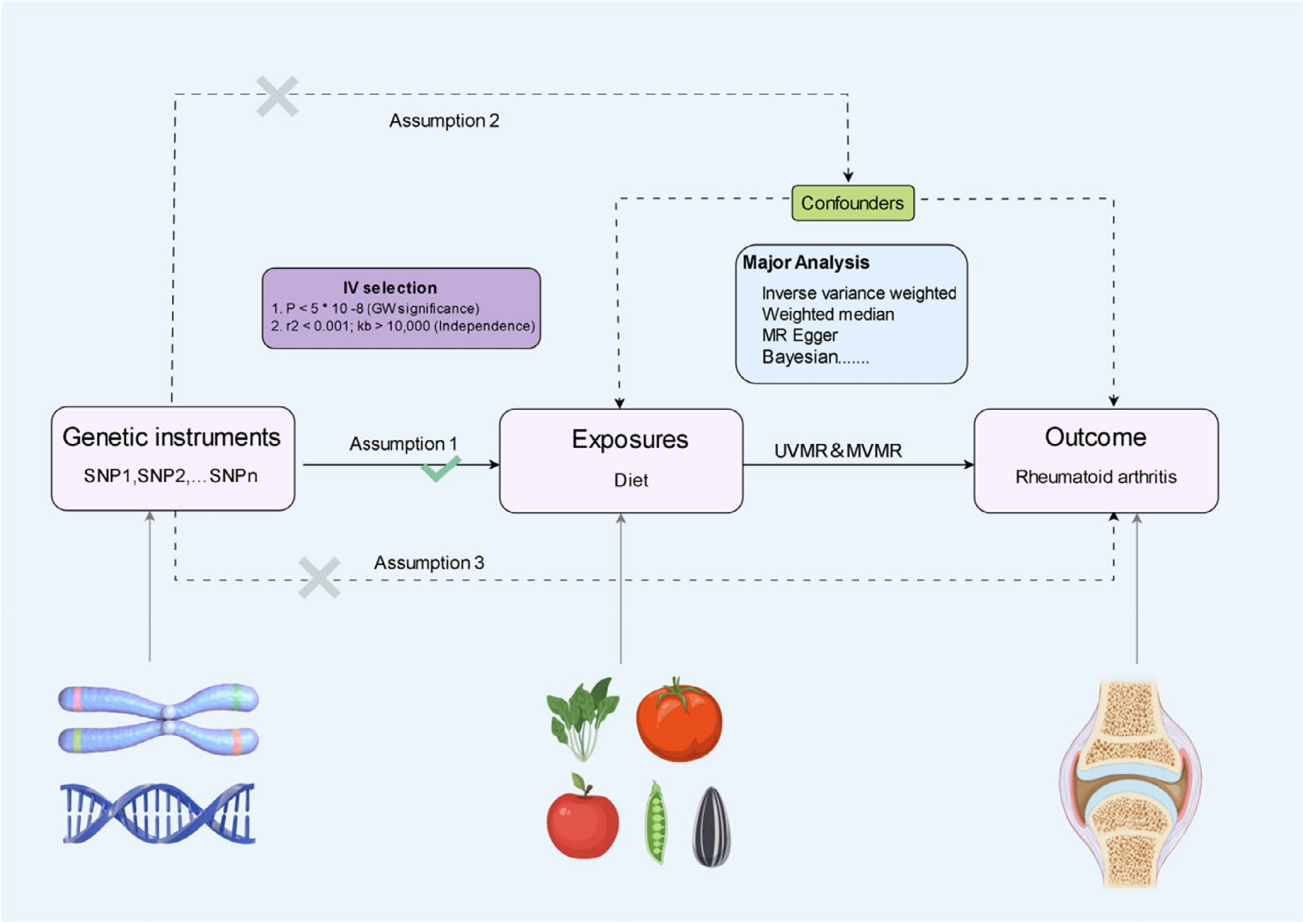
Flowchart presented in this study delineates the core assumptions underpinning the MR analysis. The principal aim of performing two‐sample MR and MVMR analyses was to examine the potential causal relationship between dietary preferences and the susceptibility to RA.

In this study, we utilized an integrated methodology incorporating two‐sample MR and MVMR analyses to investigate the potential causal relationship between dietary preferences and RA. The MR analytical framework and study design are depicted in Figure 1. To ensure the validity of the MR analysis, it is imperative to adhere to three fundamental assumptions. Firstly, the chosen IVs, specifically single nucleotide polymorphisms (SNPs), must exhibit a significant association with the dietary factors under investigation. Secondly, the IVs should remain independent of any confounding variables. Lastly, the IVs must exert their influence on the outcome exclusively through the exposure of interest, rather than through any direct effects on the outcome (Boef, Dekkers, and le Cessie 2015). Given that this study utilized publicly accessible GWAS data, no further ethical approval was necessary. The MR analyses conducted in this study were executed using the R software platform (version 4.2.1), specifically employing the two‐sample MR and MR‐PRESSO packages.

### 2.2 GWAS Data of 187 Dietary Preferences

Recent GWAS have identified 187 factors associated with dietary preferences (May‐Wilson et al. 2022). This extensive dataset provides an unprecedented opportunity to investigate the relationships between dietary preferences and RA. To compile this comprehensive, large‐scale dataset on dietary preferences, the GWAS utilized data from 11 cohorts, encompassing a combined sample of 161,625 individuals of European ancestry. Comprehensive details regarding the aggregated GWAS data are available in the primary publication and the GWAS Catalog. Table S1 presents an exhaustive enumeration of the 187 dietary preferences, including their respective names, GWAS Catalog identifiers, and abbreviations.

### 2.3 GWAS Data for RA

Initiated in 2017, the FinnGen study (https://finngen.gitbook.io/documentation/) represents a substantial national cohort endeavor established through a public–private partnership. This initiative integrates genetic data from the Finnish Biobank with digital health records from the Finnish Health Register, thereby providing a distinctive opportunity to examine genetic variations linked to disease trajectories within isolated populations (Kurki et al. 2023). We acquired summary‐level estimates of genetic associations with RA from the most recent publicly available R11 data release of the FinnGen study (https://storage.googleapis.com/finngen‐public‐data‐r11/summary_stats/finngen_R11_M13_RHEUMA.gz). RA diagnoses were classified according to the International Classification of Diseases, Tenth Revision (ICD‐10) code M06, encompassing 14,818 cases and 287,796 controls. Genome‐wide association analyses for each trait were adjusted for variables including sex, age, genetic ancestry, and genotyping batch. Although GWAS findings for RA were also available from the UK Biobank, we opted not to include this dataset due to overlapping study populations.

### 2.4 IVs Selection

In order to test hypothesis (1), we employed a rigorous selection process to identify instrumental variables (IVs) associated with 187 dietary preferences from various perspectives. In order to obtain more robust results, we adjusted the significance threshold to *p* < 8 × 10^−6^ for the selection of relevant SNPs. Subsequently, we pruned the SNPs by excluding those in linkage disequilibrium (LD), defined as *R* ^2^ > 0.001 within a 10,000 kb range, a criterion commonly utilized in prior research (Choi et al. 2019; Yang et al. 2020). To address potential bias stemming from weak IVs, we computed the *R* ^2^ and *F*‐statistics for each SNP (Lv et al. 2023). SNPs with *F* < 10 were defined as weak IVs and were subsequently excluded from the analysis (Burgess, Butterworth, and Thompson 2013). Subsequently, SNPs linked to diet were isolated from the findings while SNPs correlated with the outcomes (*p* < 1 × 10^−5^) were excluded. The SNPs were then standardized for both exposures and outcomes by eliminating palindromic SNPs and those with allele discrepancies. In accordance with hypothesis (3), SNPs associated with outcomes (*p* < 1 × 10^−5^) were omitted from the IVs. Ultimately, MR analysis was performed on metabolites with a minimum of two SNPs (Gill et al. 2019).

### 2.5 Statistical Analysis and Secondary Analysis

The principal MR analysis employed the inverse‐variance weighted (IVW) method. Specifically, a fixed‐effect model IVW was applied in the absence of significant heterogeneity, whereas a random‐effect model IVW was utilized when heterogeneity was detected (Zhang et al. 2023). The IVW method synthesizes the Wald estimates of genetic causal associations for individual SNPs to quantify the effect of the exposure on the outcome, contingent upon the assumption that all selected SNPs are valid IVs (Burgess, Dudbridge, and Thompson 2016; Yavorska and Burgess 2017). This methodology produces accurate estimates and constitutes the primary statistical technique for evaluating causal relationships (Burgess, Butterworth, and Thompson 2013; Burgess et al. 2015). In conjunction with conventional MR techniques, we utilized an array of innovative methodologies, including MR‐Egger, weighted median (WM), robust adjusted profile score (RAPS), debiased inverse‐variance weighted method (dIVW), constrained maximum likelihood (cML), and Bayesian weighted Mendelian randomization (BWMR), to enhance the robustness and comprehensiveness of the MR findings. The implementation of these advanced methodologies enhances the robustness and credibility of the MR results. Under the assumption that more than 50% of the SNPs are valid, the WM method provides a consistent estimate of the final effect (Bowden et al. 2016; Xu et al. 2022). Within the MR‐Egger framework, an intercept term is incorporated to address pleiotropy, based on the assumption that all SNPs may be invalid (Bowden, Davey Smith, and Burgess 2015). The RAPS method addresses specific pleiotropy and yields reliable inferences for MR analyses that involve a large number of weak instruments (Yu et al. 2023). The dIVW method addresses the issue of weak instrument bias inherent in the IVW approach, thereby enhancing robustness in scenarios characterized by the presence of numerous weak instruments (Ye, Shao, and Kang 2021). The cML method addresses biases arising from both related and unrelated pleiotropy (Yin and Zhu 2024). Lastly, BWMR facilitates the determination of causal relationships between exposures and outcomes (Zhao et al. 2020).

Secondary analyses in MR included evaluations of heterogeneity, pleiotropy, and sensitivity. Heterogeneity among SNPs associated with the exposure was assessed using Cochran's *Q* test (Burgess, Butterworth, and Thompson 2013). The *Q* statistic and the *I* ^2^ (%) value were calculated to quantitatively assess heterogeneity, where *I* ^2^ is defined by the formula *I* ^2^ = [*Q*—(*K*—1)]/*Q*. In this context, *K* denotes the number of single nucleotide polymorphisms (SNPs), and *Q* represents the *Q* statistic. Horizontal pleiotropy was evaluated through the MR‐Egger intercept method (Bowden, Davey Smith, and Burgess 2015) and the Global test in MR‐PRESSO (Verbanck et al. 2018). A near‐zero intercept indicates a diminished likelihood of horizontal pleiotropy. For sensitivity analysis, the leave‐one‐out (LOO) method was employed, systematically excluding each SNP to evaluate the influence of individual SNPs on the overall causal estimate (Flatby et al. 2023). Furthermore, a sensitivity analysis was conducted by comparing outcomes across multiple MR methods to ensure the robustness and reliability of the conclusions (Mbutiwi, Dessy, and Sylvestre 2022).

To reinforce the exclusion restriction assumption in MR analyses, the Steiger test was implemented. This test is designed to verify that the genetic variants used as IVs demonstrate a stronger association with the exposure variable than with the outcome variable, thereby maintaining the validity of the instrumental variable assumptions. This additional validation procedure enhances the robustness of our MR investigations by confirming the directionality of the causal inference (Hemani, Tilling, and Davey Smith 2017).

### 2.6 Confounding Analysis and Multivariable MR Analysis

We performed sensitivity analyses to assess the horizontal pleiotropy of MR results, aiming to identify SNPs that might violate the assumptions of MR. Despite these efforts, the presence of a limited number of residual confounding SNPs cannot be entirely ruled out. We investigated instrumental variables associated with dietary preferences using the GWAS Catalog (https://www.ebi.ac.uk/gwas/) to ascertain their relationships with established risk factors for RA (Di Matteo, Bathon, and Emery 2023), including smoking, body weight and body fat, anxiety, viral infections, and the diseases themselves. If any SNPs are identified as being associated with the aforementioned confounding variables and the outcome (*p* < 1 × 10^−5^), MR analyses will be repeated after excluding these SNPs to ensure the robustness of the findings.

To comply with assumptions 2 and 3 of MR, it is essential to verify that genetic variants are associated with a single risk factor. However, some genetic variants are linked to multiple risk factors, a phenomenon known as pleiotropy. In cases of pleiotropy, MVMR can effectively address the interactions among genetic variants associated with various exposures that may influence each other (Sanderson 2021). MVMR provides a robust solution to the issues of independence, dominance, and comparability that single‐variable MR cannot adequately address. Specifically, while single‐variable MR assesses the aggregate effect of an exposure on the outcome, MVMR disaggregates this analysis to evaluate the distinct impact of each exposure on the outcome, independent of other exposures. In this study, we employed MVMR to account for the interactions among the identified dietary preferences. MVMR was implemented through the IVW (Burgess and Thompson 2015), MR‐PRESSO (Verbanck et al. 2018), and LASSO regression (Grant and Burgess 2021; Xiao et al. 2022) techniques. In the context of MVMR, the IVW method involves regressing SNPs associated with all exposures against the outcome, with weights assigned based on the inverse variance of the outcome. To mitigate pleiotropy among IVs, the MR‐PRESSO method was applied to detect and exclude outliers. Additionally, LASSO regression was used to remove exposures demonstrating collinearity.

## 3 Results

### 3.1 Preliminary Analysis

#### 3.1.1 Identification of SNPs and Initial Screening

Employing a significance threshold of *p* < 5 × 10^−8^, this study identified 747 SNPs associated with dietary preferences. The F‐statistics for these SNPs ranged from 29.72 to 426.22, thereby confirming the statistical robustness of the selected dietary preferences as IVs in the MR analysis. Detailed information regarding the IVs is presented in Table S2. Prior to the formal MR analysis, all outliers were identified and excluded through a screening process for confounding factors and the application of MR‐PRESSO, as outlined in Table S3.

#### 3.1.2 Mendelian Randomization Analysis and Sensitivity Tests

**FIGURE 2:**
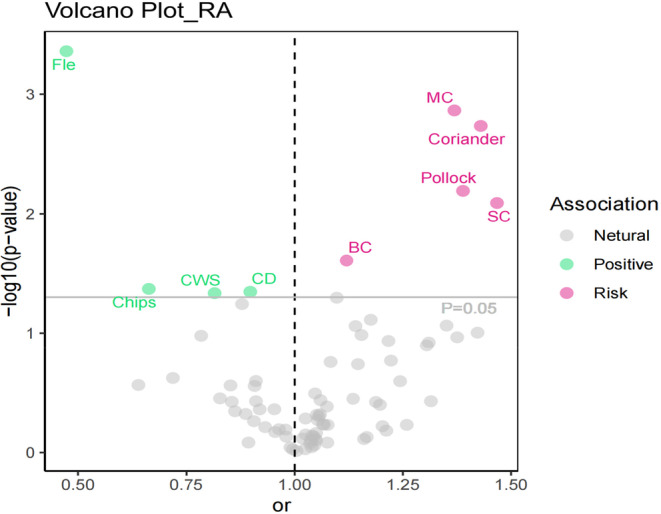
Volcano plot illustrating the association between dietary preferences and the risk of developing RA.

**FIGURE 3:**
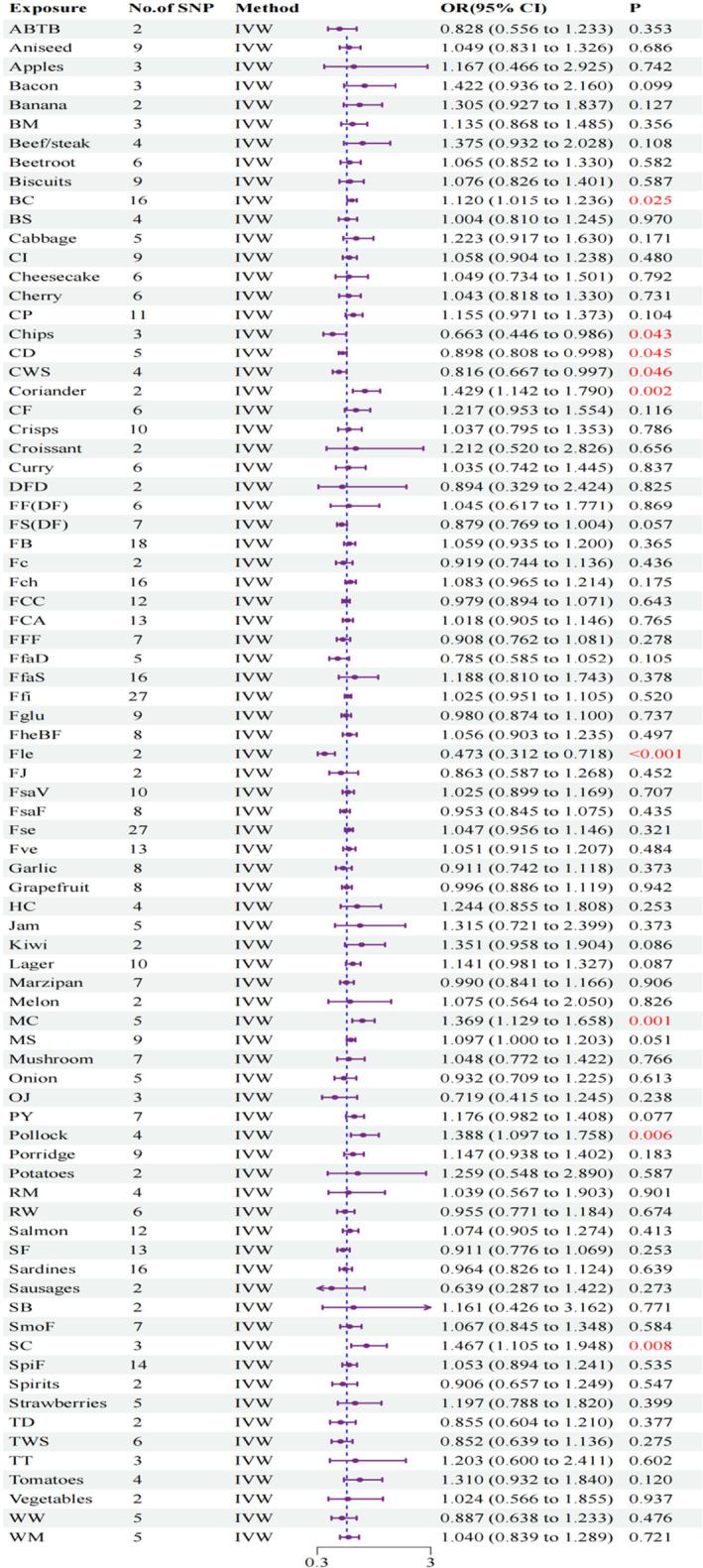
Forest plot depicting the association between dietary preferences and the risk of RA.

In this comprehensive MR analysis, we explored the association between dietary preferences and RA utilizing the IVW method. The analysis revealed significant correlations between nine dietary preference factors and RA, as detailed in Table S4 and illustrated in Figures 2 and 3. Notable results included a significant inverse association for Fle (odds ratio [OR]: 0.473, 95% confidence interval [CI]: 0.312–0.718, *p* < 0.001), chips (OR: 0.663, 95% CI: 0.446–0.986, *p* = 0.043), CD (OR: 0.898, 95% CI: 0.808–0.998, *p* = 0.045), and CWS (OR: 0.816, 95% CI: 0.667–0.997, *p* = 0.046), MC (OR: 1.369, 95% CI: 1.129–1.658, *p* = 0.001), coriander (OR: 1.429, 95% CI: 1.142–1.790, *p* = 0.002), pollock (OR: 1.388, 95% CI: 1.097–1.758, *p* = 0.006), SC (OR: 1.476, 95% CI: 1.105–1.948, *p* = 0.008), and BC (OR: 1.120, 95% CI: 1.015–1.236, *p* = 0.025). The observed consistency across multiple methodologies, such as MR‐Egger, WM, RAPS, dIVW, cML, and BWMR, highlights the robustness of these findings. Repeated sensitivity analyses revealed no SNPs demonstrating pleiotropy, as evidenced by both horizontal pleiotropy assessments and MR‐PRESSO analyses (see Table S5). This absence of pleiotropic SNPs further corroborates the reliability of our instrumental variables. Moreover, heterogeneity tests indicated that most MR results showed no evidence of heterogeneity, except for the findings related to Fle (refer to Table S6), thereby reinforcing the validity of our results. Additionally, the LOO analysis did not reveal any significant sources of bias (see Figure S1). All evaluations successfully passed the Steiger test, affirming the absence of reverse causation among the instrumental variables (refer to Table S7). Further visual data are presented in Figures S2–S4.

### 3.2 MVMR Analysis

To address potential interactions and confounding effects among dietary factors, we performed a MVMR analysis. This approach facilitates a more thorough investigation of the independent effects of various dietary preferences on the risk of RA. The MVMR analysis utilized three robust methodologies: IVW, MR‐PRESSO test, and LASSO regression. These methods were selected to ensure the reliability and consistency of our findings while effectively accounting for potential biases and confounding variables.

**FIGURE 4:**
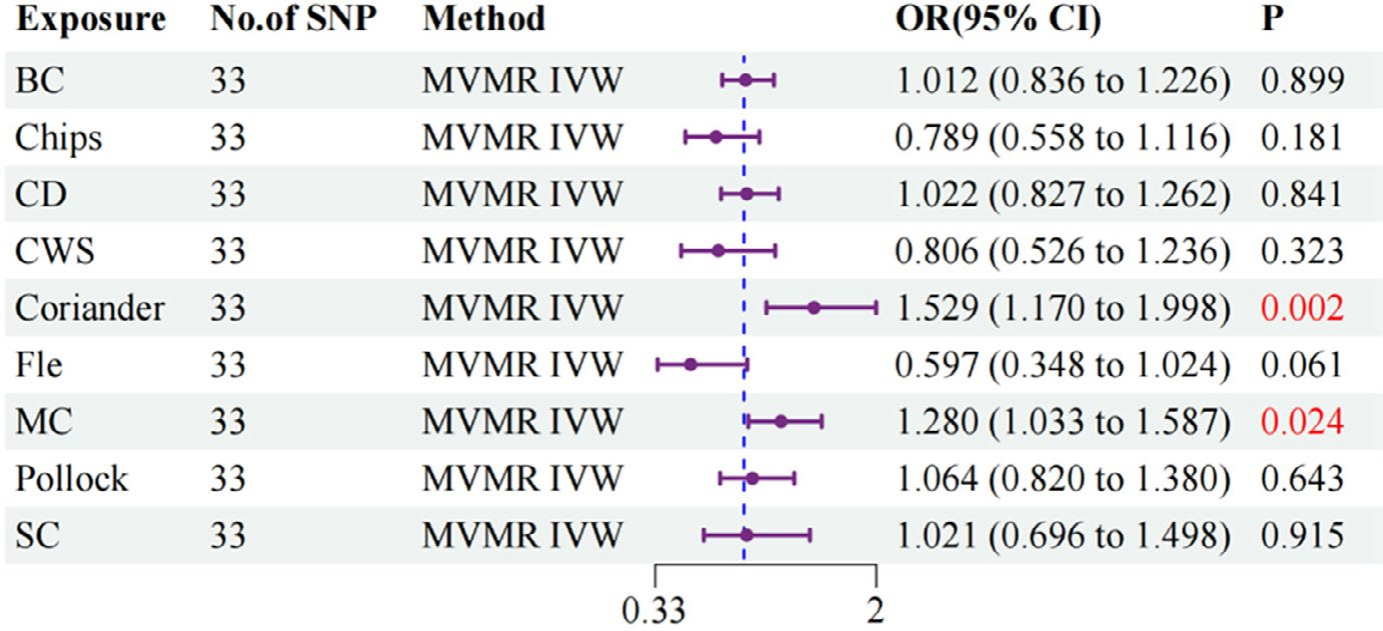
Forest plot illustrating the outcomes of the MVMR analysis.

Our analysis identified two dietary factors that remained significantly associated with the risk of RA, even after controlling for other dietary preferences: coriander (OR: 1.529, 95% CI: 1.170–1.998, *p* = 0.002) and MC (OR: 1.280, 95% CI: 1.033–1.587, *p* = 0.024). These findings indicate that a genetic predisposition to preferences for coriander and MC may independently affect the likelihood of developing RA. The positive odds ratios suggest that individuals with a genetic predisposition to favor these foods may exhibit an elevated risk of developing RA. Notably, these findings remained consistent across all three analytical methods utilized in the MVMR analysis. This consistency enhances the reliability of our results and indicates that the observed associations are robust across various statistical approaches (see Table S8, Figure 4).

## 4 Discussion

### 4.1 Dietary Factors and RA Risk: Insights From MR Analysis

RA is a complex autoimmune disorder characterized by multifaceted and intricate mechanisms. Emerging evidence underscores the substantial influence of dietary factors on the pathogenesis of RA (Gioia et al. 2020); however, the precise causal relationships and underlying mechanisms remain largely elusive. In this study, we employed SNPs strongly associated with dietary preferences, alongside comprehensive site examination and data from the FinnGen database, to evaluate the potential causal effects of dietary preferences on RA through MR analysis.

This comprehensive MR analysis rigorously examined the causal relationship between genetically predicted dietary preferences and the incidence of RA. The results suggest that elevated preferences for Fle, chips, CD, and CWS are associated with a decreased risk of RA. Conversely, increased preferences for MC, coriander, pollock, SC, and BC are correlated with a heightened risk of developing RA. The MVMR analysis notably indicates a potential association between coriander and MC with RA risk. Specifically, a one standard unit (SD) increase in coriander consumption is associated with a 52.9% increase in RA risk, whereas a similar increase in MC consumption correlates with a 28.0% increase in RA risk. Further investigation into the underlying mechanisms of these dietary factors could provide new insights and potential therapeutic targets for RA management. This study introduces a novel approach to examining the potential causal relationship between RA and dietary preferences through the application of MVMR.

### 4.2 Detailed Analysis of Dietary Factors and Their Implications

A proteomic analysis utilizing a RA rat model demonstrated that coriander could partially ameliorate RA‐induced molecular disturbances, including impaired carbon metabolism, compromised mitochondrial function (tricarboxylic acid cycle and oxidative phosphorylation), and alterations in muscle fiber types, which are relevant in combating cachexia in RA patients (Jia et al. 2021). However, these findings are inconsistent with the results of our study, which suggest that coriander may elevate RA risk through specific mechanisms, such as modulating the immune system, inflammatory responses, and gut microbiota. Additionally, our MR study may have identified a genetic correlation between coriander consumption and an elevated risk of RA. Crucially, we are unable to establish a temporal relationship between coriander intake and the onset of RA. Consequently, the association between coriander consumption and RA necessitates further investigation. Existing research indicates that coriander intake may influence the composition of gut microbiota (Ali et al. 2024), which is intimately connected to immune responses. Alterations in the abundance of specific bacterial taxa could potentially promote inflammation, thereby augmenting the risk of RA. Therefore, we hypothesize that the consumption of coriander is significantly associated with an increased risk of developing RA.

No other research has demonstrated a direct relationship between MC and RA. This study may constitute a pioneering effort to elucidate the causal relationship between these variables through the application of MR methods. Nonetheless, the underlying mechanisms remain obscure. MC generally contains elevated levels of sugar and saturated fat, which could potentially contribute to increased inflammatory markers (Khawaja, Gaziano, and Djoussé 2011), weight gain (Halib et al. 2020), and fluctuations in blood glucose levels (Oliveira, Falkenhain, and Little 2022). Consequently, a predilection for milk chocolate may influence the immune system and heighten the risk of chronic diseases, including RA.

No existing research has demonstrated a direct relationship between SC, BC, and RA. Consequently, this study may represent a pioneering effort to elucidate the causal relationship between these variables using MR methods. Nonetheless, the underlying mechanisms remain unclear. Cheese, a significant component of the human diet, provides essential nutrients including amino acids, calcium, and vitamins. However, it typically contains saturated fats, which are associated with elevated blood lipid levels, weight gain, and metabolic syndrome (Natrella et al. 2024). Therefore, the preference for cheese, including varieties such as soft cheese and blue cheese, may influence the incidence and progression of RA. Our preliminary two‐sample MR study indicates a positive correlation between a preference for Pollock and the incidence of RA. However, no direct causal relationship has been established. This observation may be attributable to the presence of specific components in Pollock, such as particular fatty acids or proteins, which could potentially provoke or intensify inflammatory responses in certain individuals, thereby elevating the risk of RA among Pollock enthusiasts. Furthermore, individuals who exhibit a preference for Pollock may possess distinct dietary habits or lifestyle choices that could potentially correlate with the onset of RA.

While the MVMR study did not demonstrate significant associations with Fle, chips, CD, and CWS, our preliminary two‐sample MR analysis identified a negative correlation between these factors and RA, suggesting their potential role as protective factors against RA. The observed inverse relationship between lentil/bean preference and RA may be attributable to the high levels of fiber, antioxidants, and phytochemicals present in these foods, which are well‐documented for their anti‐inflammatory properties (Hou et al. 2014; Johnson et al. 2021; Souza et al. 2021). The intake of legumes has been associated with a reduction in systemic inflammation, potentially decreasing the risk of developing RA (Schmidt et al. 2022). Furthermore, the substantial fiber content in legumes promotes enhanced gut health, which is intricately connected to the functioning of the immune system (Azzeh et al. 2017). Although a preference for chips demonstrated a negative correlation with RA, it is crucial to recognize that chips generally contain elevated levels of sodium and unhealthy fats, which may be linked to various lifestyle factors. Individuals who favor chips might exhibit different dietary choices and lifestyle habits, potentially influencing their risk of developing RA. Consequently, future research should investigate the comprehensive dietary patterns of individuals who consume chips to elucidate their impact on RA risk more thoroughly. Concerning the findings related to coffee consumption, research indicates that coffee may exhibit anti‐inflammatory properties (Khan et al. 2019). Previous MR studies and prospective cohort studies have suggested a positive correlation between coffee consumption and RA (Ascione et al. 2023; Wang et al. 2023). However, contrasting evidence exists, as another study found no association (Pu et al. 2022), while case–control studies have indicated that coffee may act as a protective factor against RA (Rambod, Nazarinia, and Raieskarimian 2018). These findings are not quite consistent with our research conclusions, which specifically indicate that the intake of unsweetened coffee is associated with a reduced risk of RA. This protective effect may be mediated through mechanisms that enhance metabolism and attenuate inflammatory responses (Antonietti et al. 2022). In contrast, the observed negative correlation with sweetened coffee suggests that sugar intake may exacerbate inflammation to a certain degree. However, the impact of sugar on rheumatoid arthritis seems to be moderated by individual variability. Additionally, the strict inclusion criteria of our study and the differences in GWAS research may contribute to this ongoing debate. This controversy requires resolution through subsequent MR‐based meta‐analyses and comprehensive mechanistic studies.

Nonetheless, additional research is required to rigorously investigate the specific effects of the previously mentioned dietary preferences on RA within a controlled experimental framework.

### 4.3 Strengths, Limitations, and Future Directions

This MR study presents several notable advantages. Firstly, it represents the most extensive and systematic investigation to date, exploring the causal relationship between dietary preferences and RA, encompassing a range of dietary patterns. Secondly, it utilizes robust and comprehensive MR analysis to address inherent limitations, including reverse causation and confounding variables. To augment the reliability of our findings, we employed a range of methodologies to substantiate the assumptions underlying MR and to mitigate potential biases. The uniform directionality observed across multiple MR estimates, coupled with comprehensive sensitivity analyses, underscores the robustness of our results. Furthermore, the validity of our conclusions is reinforced through MVMR analysis.

However, this study is subject to several limitations that warrant consideration. Firstly, the study population is confined to individuals of European ancestry, which may constrain the generalizability of the findings to other ethnic groups. Secondly, the possibility of unobserved pleiotropy in the MR analysis poses a risk of bias in the results. Nonetheless, the F‐statistic values for all selected SNPs exceed 10, indicating the robustness of our instrumental variables. Thirdly, the screening of data for dietary preferences was limited. Moreover, there exists a notable deficiency in foundational research and high‐quality, large‐sample clinical randomized controlled trials to substantiate the associations with RA. Consequently, further validation is imperative to ascertain the robustness of these findings.

## 5 Conclusion

In conclusion, the present MR study elucidates genetic predispositions to dietary preferences that exhibit a causal relationship with RA. Specifically, the consumption of milk chocolate and coriander, identified as risk factors via MVMR, merits further investigation. The identification of these dietary preference factors is pivotal for the early detection, prevention, and management of RA and also holds significant implications for the design of future clinical studies. Furthermore, this analysis provides a foundational framework for the exploration of potential etiologies and mechanisms underlying RA.

## Author Contributions

**Yun Lu:** conceptualization (equal), methodology (equal), resources (equal), visualization (equal), writing – original draft (equal). **Qiang Su:** conceptualization (equal), methodology (equal), resources (equal), software (equal), visualization (equal), writing – original draft (equal). **Zhongbo Xie:** data curation (equal), formal analysis (equal), investigation (equal). **Jiang Liang:** project administration (lead), supervision (lead), validation (lead), writing – review and editing (lead).

## Conflicts of Interest

The authors declare no conflicts of interest.

## Supporting information


Figures S1–S4.

Tables S1–S8.

## Data Availability

All data generated or analyzed during this study are included in this published article (and its Supporting Information files).
