# Supplementary material for: Elucidating the Causal Impact of Dietary Factors on Rheumatoid Arthritis: Insights From Multivariable Mendelian Randomization Analysis
Source: Food Sci Nutr. 2024 Dec 1;12(12):10903–12. doi: 10.1002/fsn3.4630 (PMC11666914; doi:10.1002/fsn3.4630)
Supplement: Supplementary file 1 — Figures S1–S4. [file FSN3-12-10903-s001.pdf]

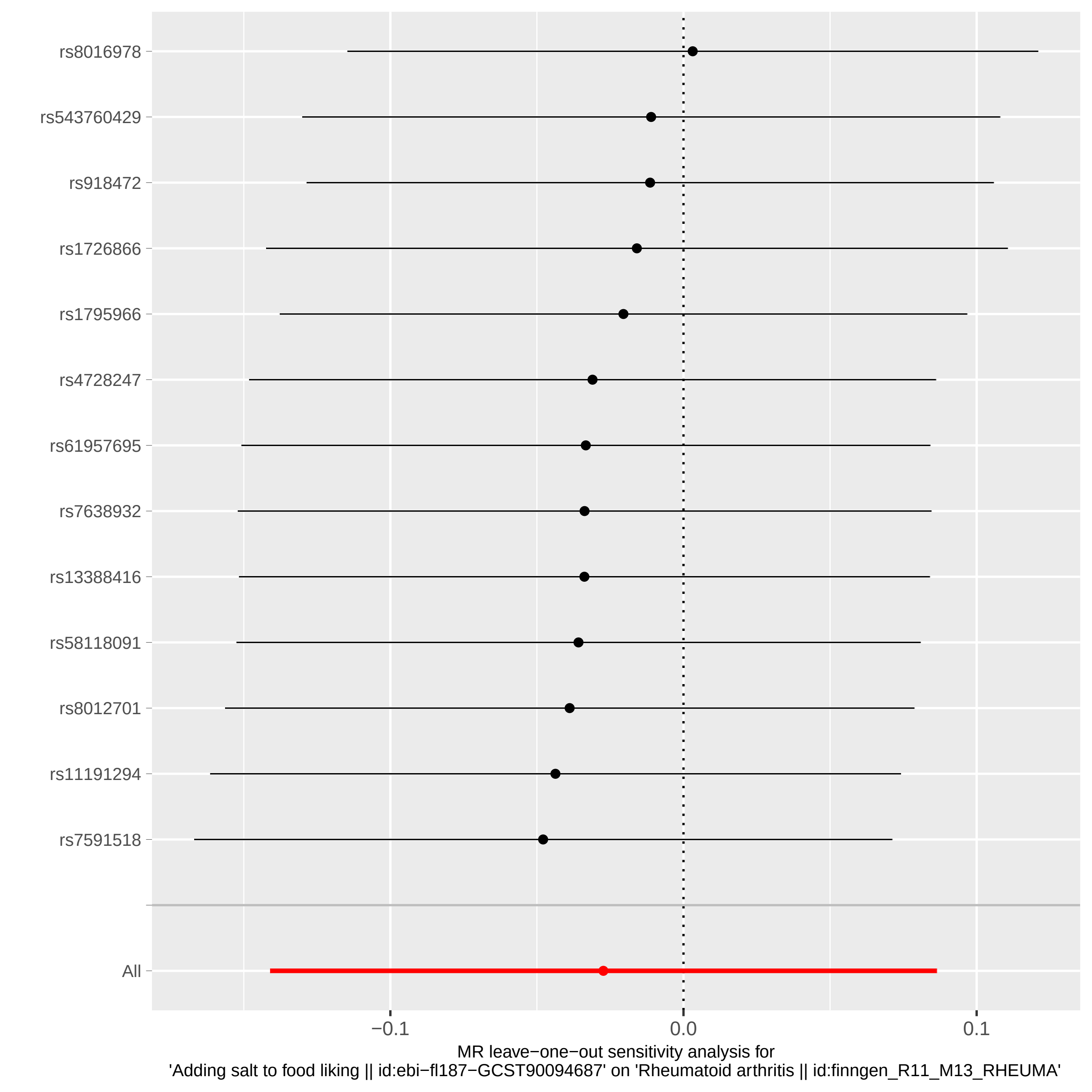
rs8016978
rs543760429
rs918472
rs1726866
rs1795966
rs4728247
rs61957695
rs7638932
rs13388416
rs58118091
rs8012701
rs11191294
rs7591518
All
−0.1
0.0
0.1
MR leave−one−out sensitivity analysis for
'Adding salt to food liking || id:ebi−fl187−GCST90094687' on 'Rheumatoid arthritis || id:finngen_R11_M13_RHEUMA'

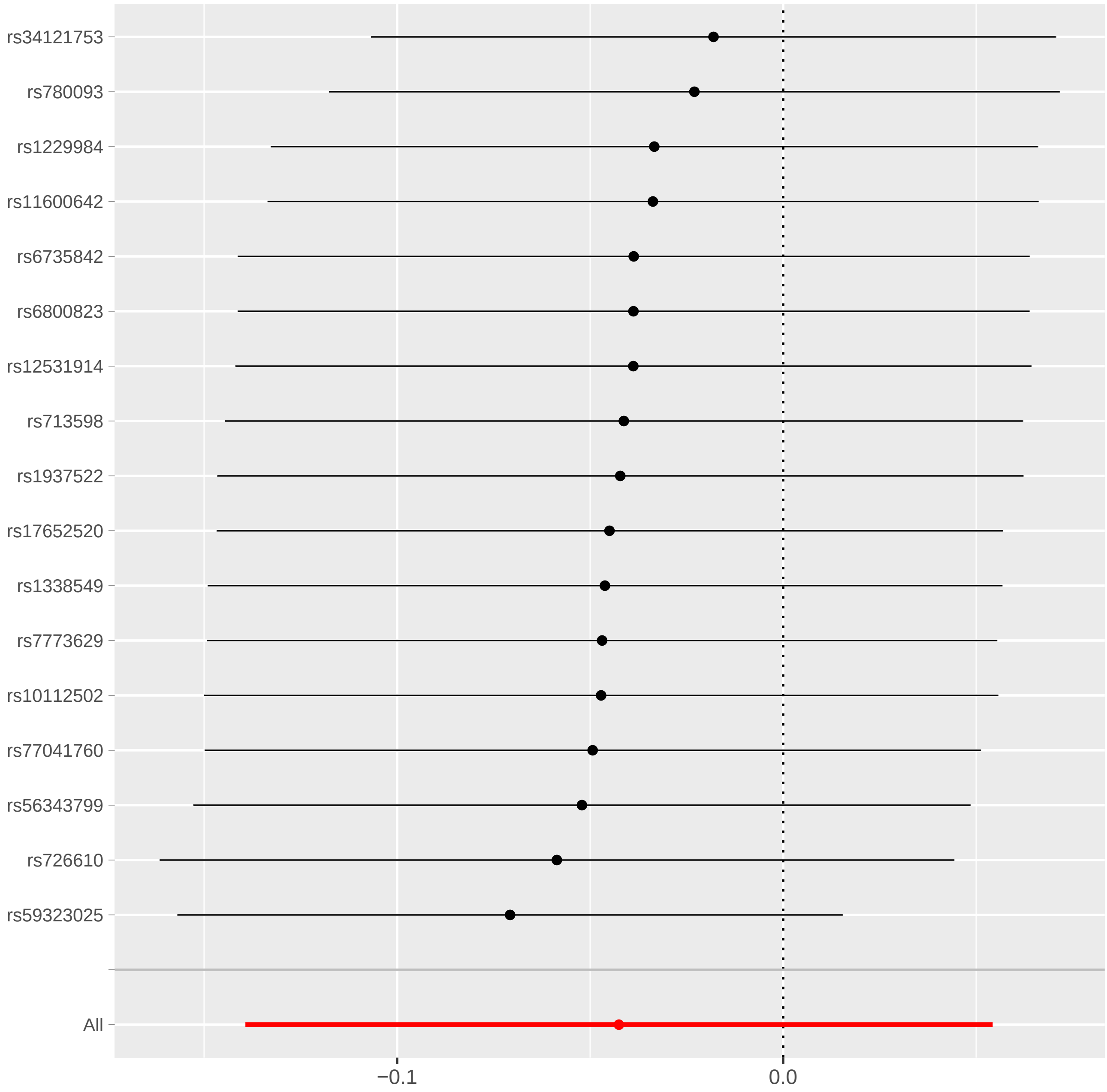

MR leave−one−out sensitivity analysis for
'Alcohol liking || id:ebi−fl187−GCST90094688' on 'Rheumatoid arthritis || id:finngen_R11_M13_RHEUMA'

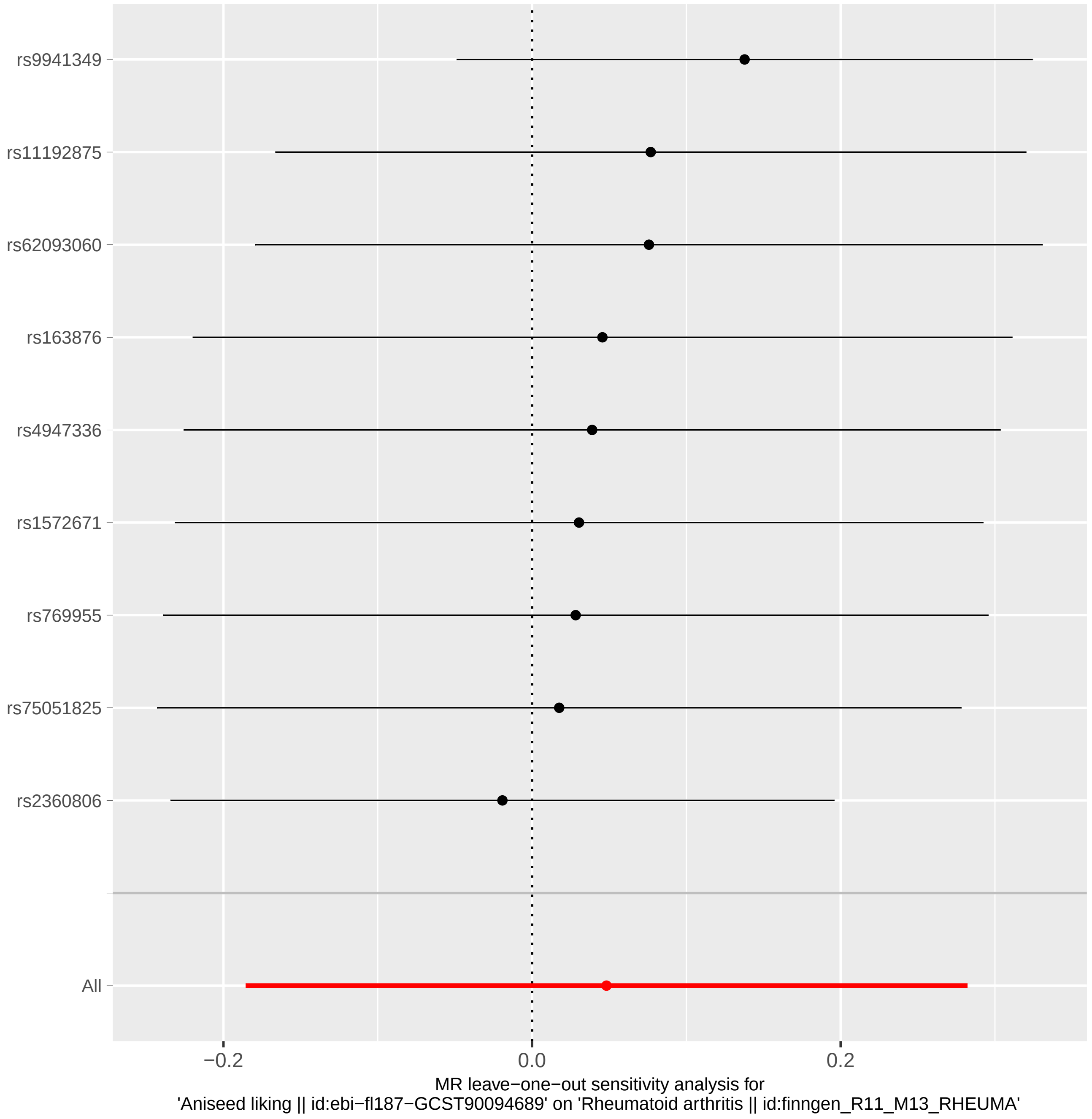
rs9941349
rs11192875
rs62093060
rs163876
rs4947336
rs1572671
rs769955
rs75051825
rs2360806
All
−0.2
0.0
0.2
MR leave−one−out sensitivity analysis for
'Aniseed liking || id:ebi−fl187−GCST90094689' on 'Rheumatoid arthritis || id:finngen_R11_M13_RHEUMA'

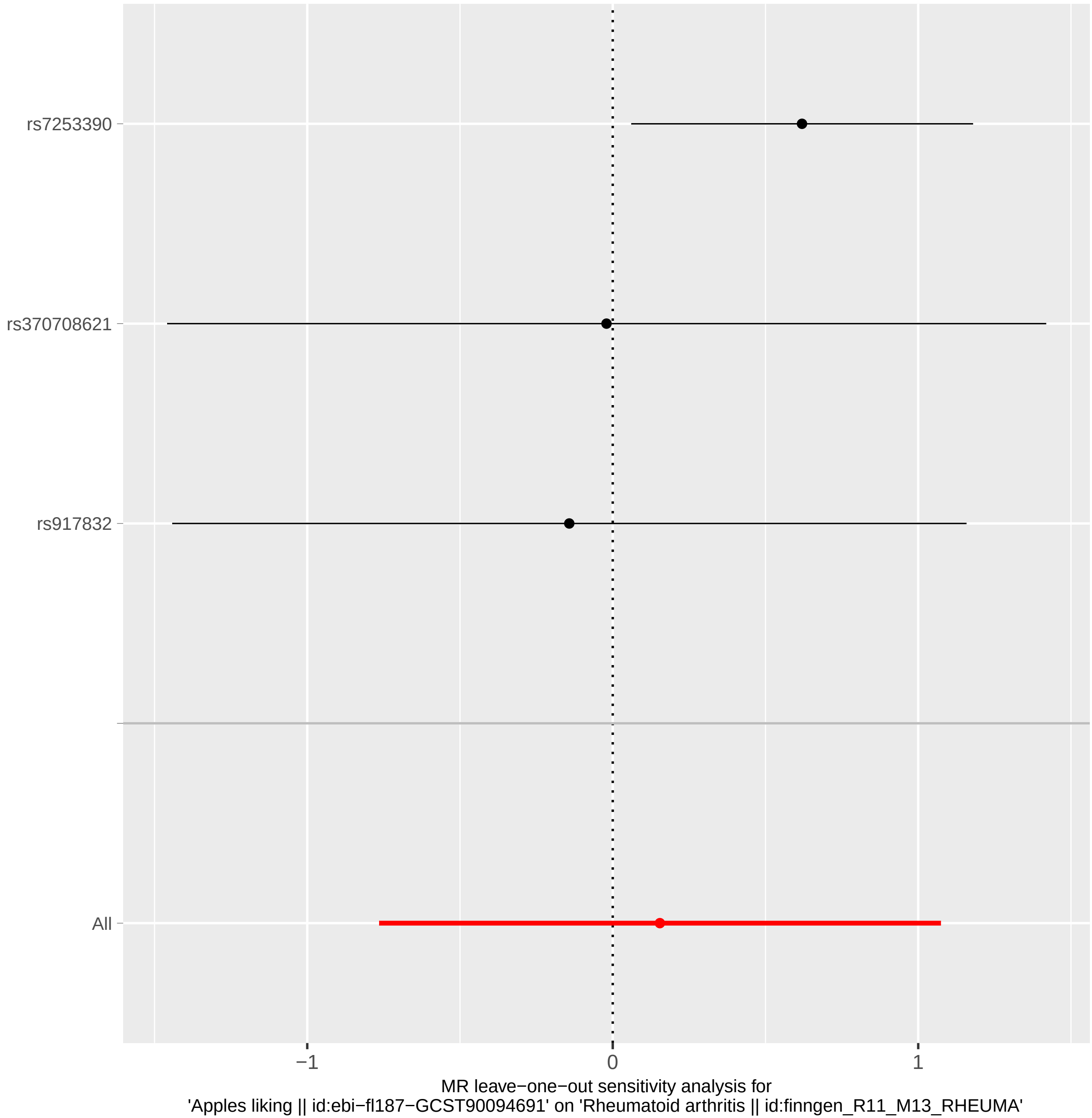
rs7253390
rs370708621
rs917832
All
−1
0
1
MR leave−one−out sensitivity analysis for
'Apples liking || id:ebi−fl187−GCST90094691' on 'Rheumatoid arthritis || id:finngen_R11_M13_RHEUMA'

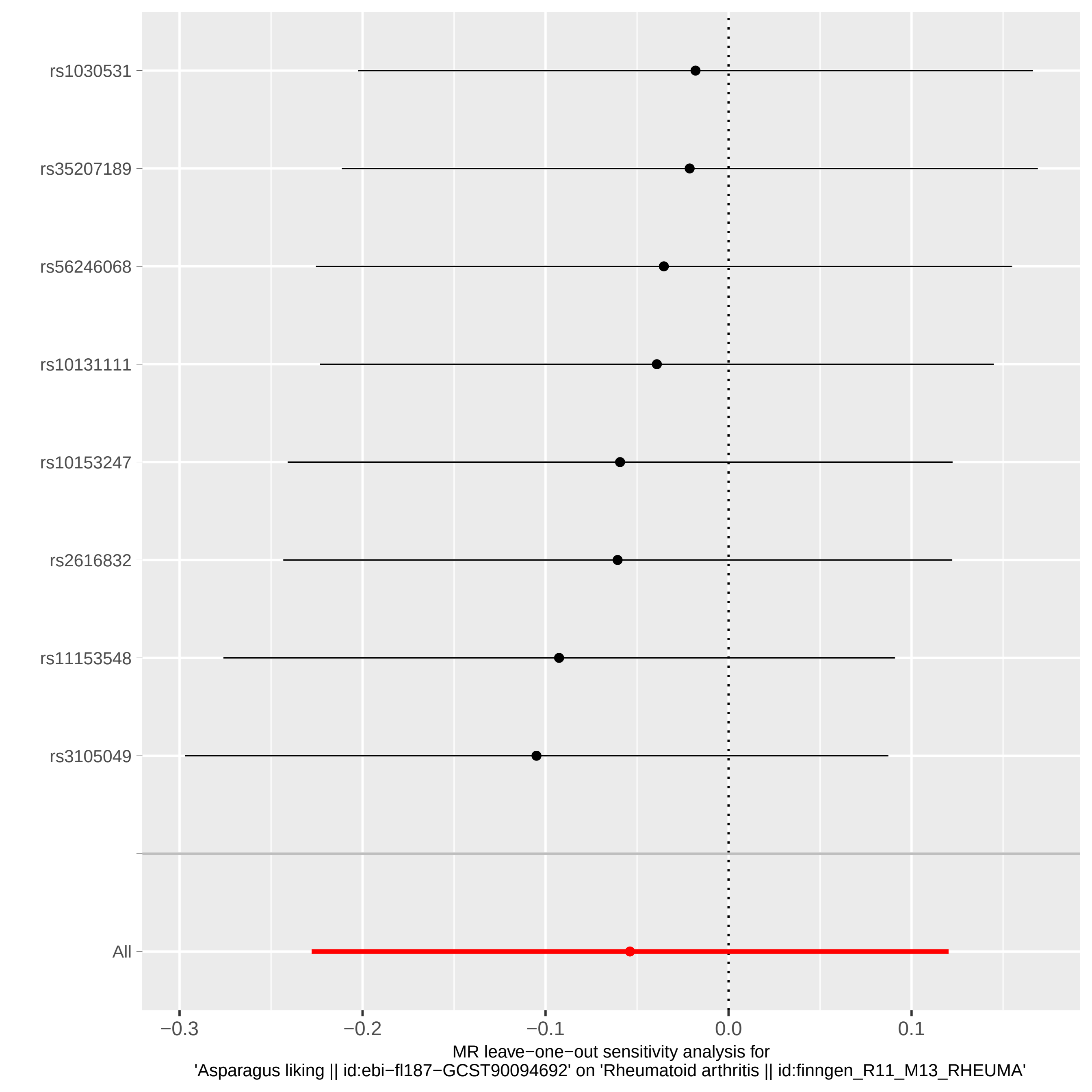

rs1030531
rs35207189
rs56246068
rs10131111
rs10153247
rs2616832
rs11153548
rs3105049
All
−0.3
−0.2
−0.1
0.0
0.1
MR leave−one−out sensitivity analysis for
'Asparagus liking || id:ebi−fl187−GCST90094692' on 'Rheumatoid arthritis || id:finngen_R11_M13_RHEUMA'

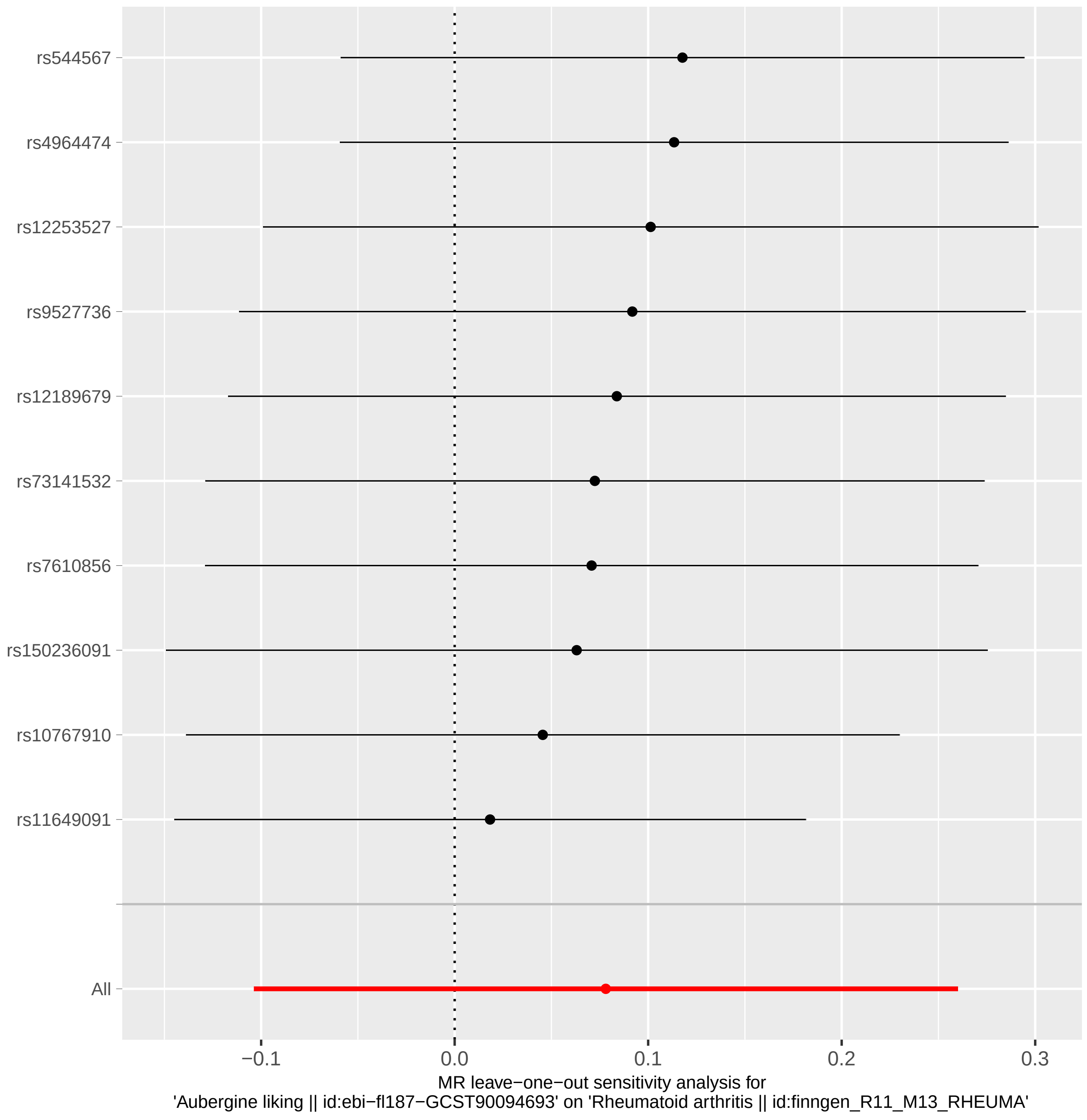
rs544567
rs4964474
rs12253527
rs9527736
rs12189679
rs73141532
rs7610856
rs150236091
rs10767910
rs11649091
All
−0.1
0.0
0.1
0.2
0.3
MR leave−one−out sensitivity analysis for
'Aubergine liking || id:ebi−fl187−GCST90094693' on 'Rheumatoid arthritis || id:finngen_R11_M13_RHEUMA'

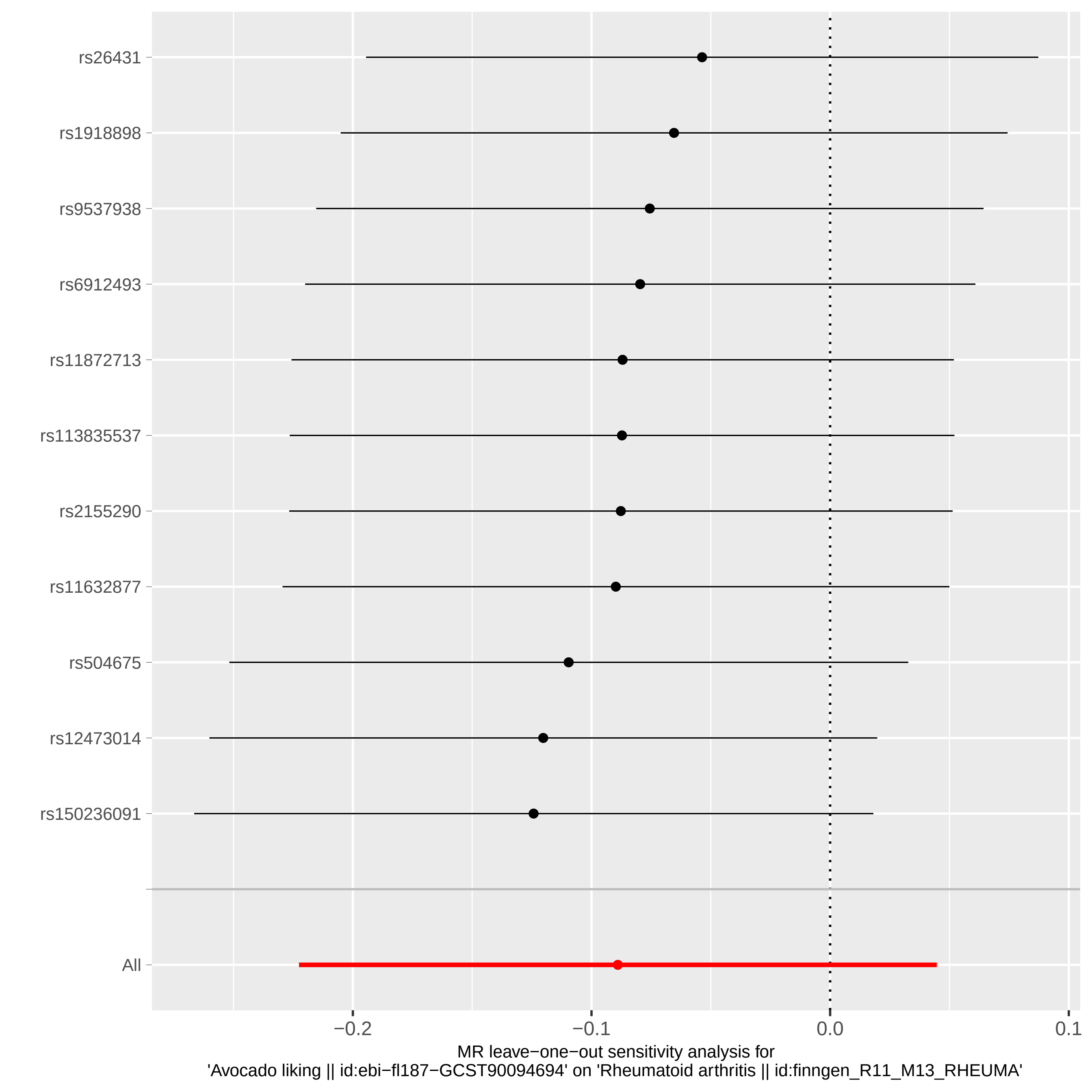
rs26431
rs1918898
rs9537938
rs6912493
rs11872713
rs113835537
rs2155290
rs11632877
rs504675
rs12473014
rs150236091
All
−0.2
−0.1
0.0
0.1
MR leave−one−out sensitivity analysis for
'Avocado liking || id:ebi−fl187−GCST90094694' on 'Rheumatoid arthritis || id:finngen_R11_M13_RHEUMA'

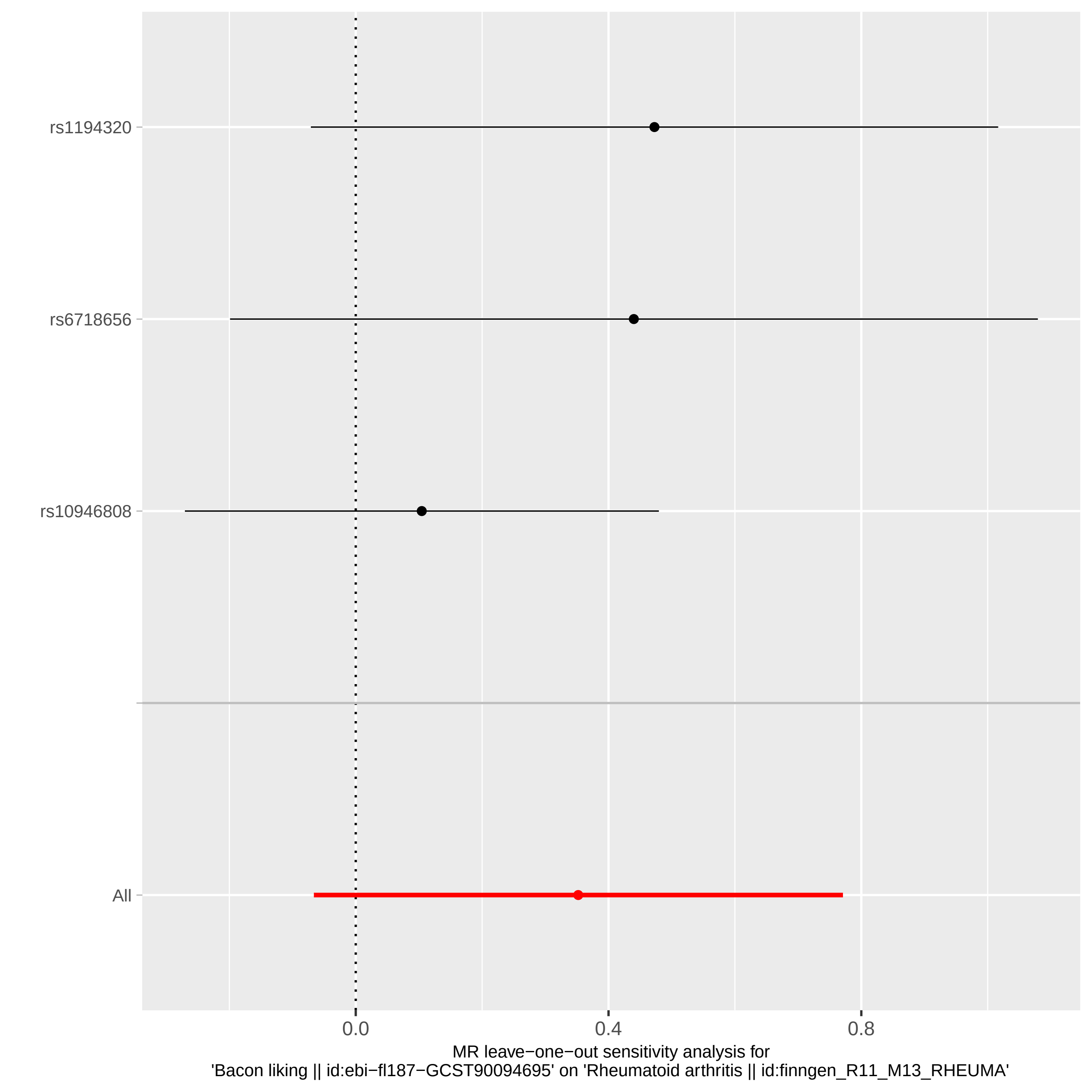
rs1194320
rs6718656
rs10946808
All
0.0
0.4
0.8
MR leave−one−out sensitivity analysis for
'Bacon liking || id:ebi−fl187−GCST90094695' on 'Rheumatoid arthritis || id:finngen_R11_M13_RHEUMA'

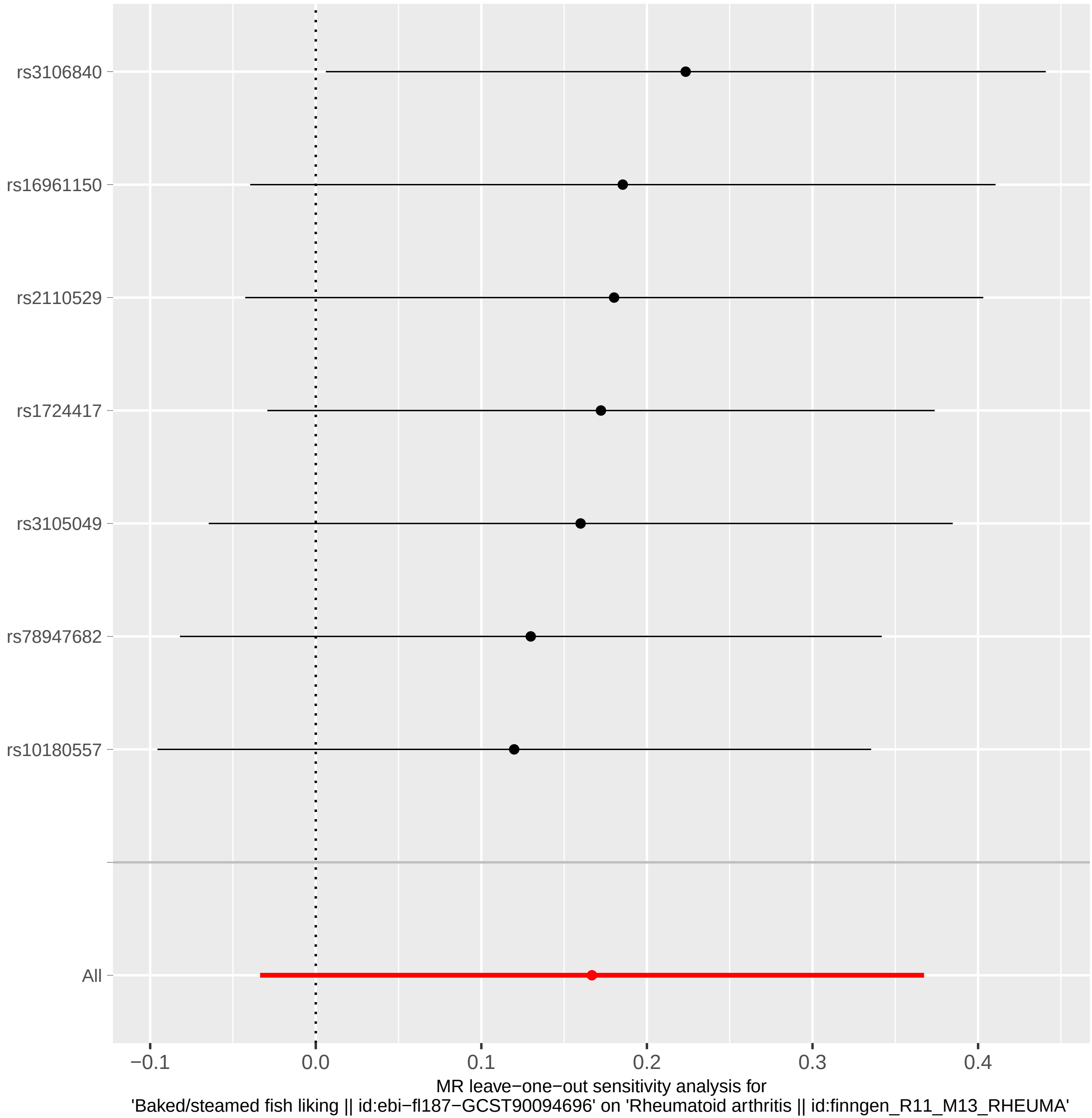
rs3106840
rs16961150
rs2110529
rs1724417
rs3105049
rs78947682
rs10180557
All
−0.1
0.0
0.1
0.2
0.3
0.4
MR leave−one−out sensitivity analysis for
'Baked/steamed fish liking || id:ebi−fl187−GCST90094696' on 'Rheumatoid arthritis || id:finngen_R11_M13_RHEUMA'

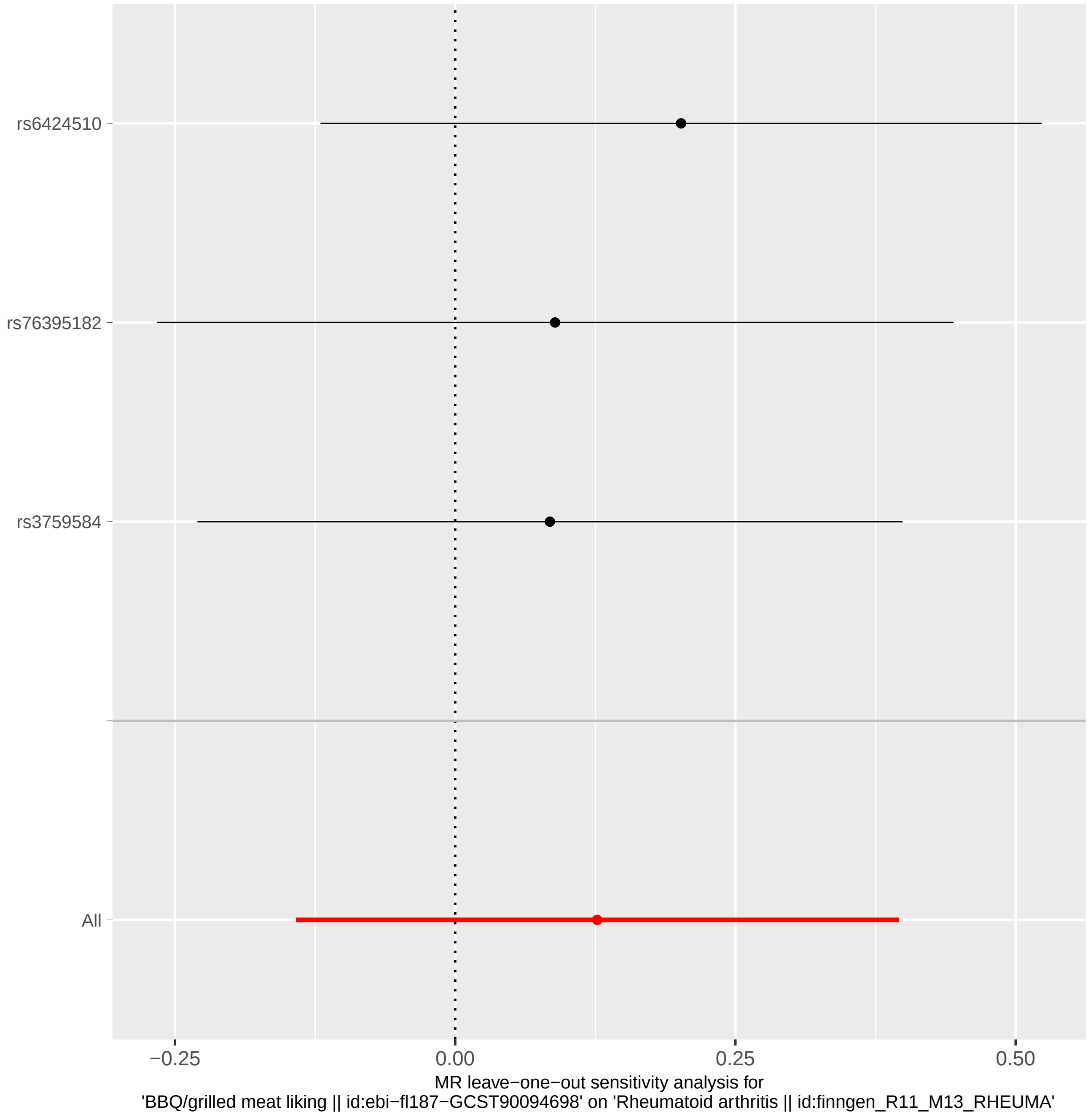
rs6424510
rs76395182
rs3759584
All
−0.25
0.00
0.25
0.50
MR leave−one−out sensitivity analysis for
'BBQ/grilled meat liking || id:ebi−fl187−GCST90094698' on 'Rheumatoid arthritis || id:finngen_R11_M13_RHEUMA'

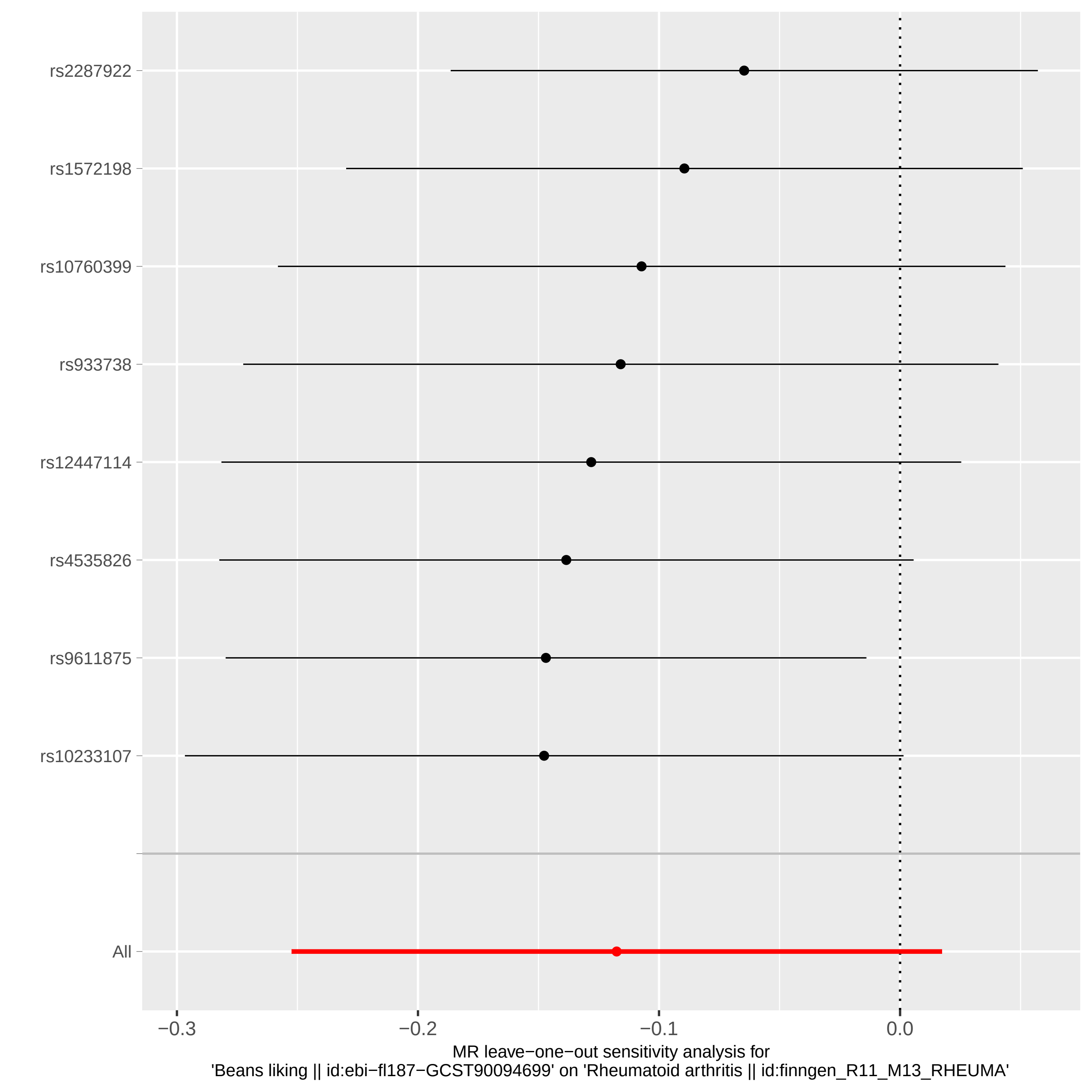
rs2287922
rs1572198
rs10760399
rs933738
rs12447114
rs4535826
rs9611875
rs10233107
All
−0.3
−0.2
−0.1
0.0
MR leave−one−out sensitivity analysis for
'Beans liking || id:ebi−fl187−GCST90094699' on 'Rheumatoid arthritis || id:finngen_R11_M13_RHEUMA'

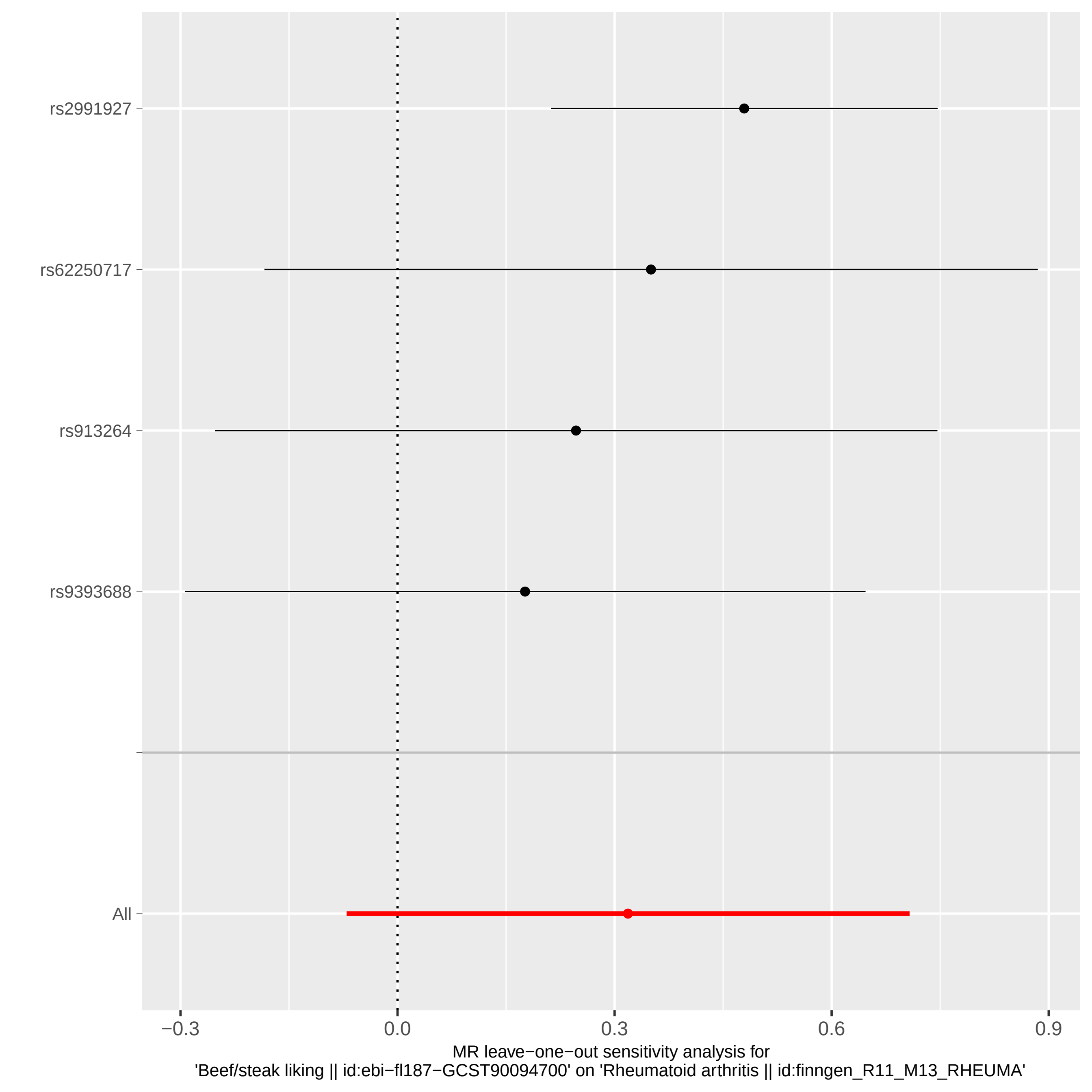
rs2991927
rs62250717
rs913264
rs9393688
All
−0.3
0.0
0.3
0.6
0.9
MR leave−one−out sensitivity analysis for
'Beef/steak liking || id:ebi−fl187−GCST90094700' on 'Rheumatoid arthritis || id:finngen_R11_M13_RHEUMA'

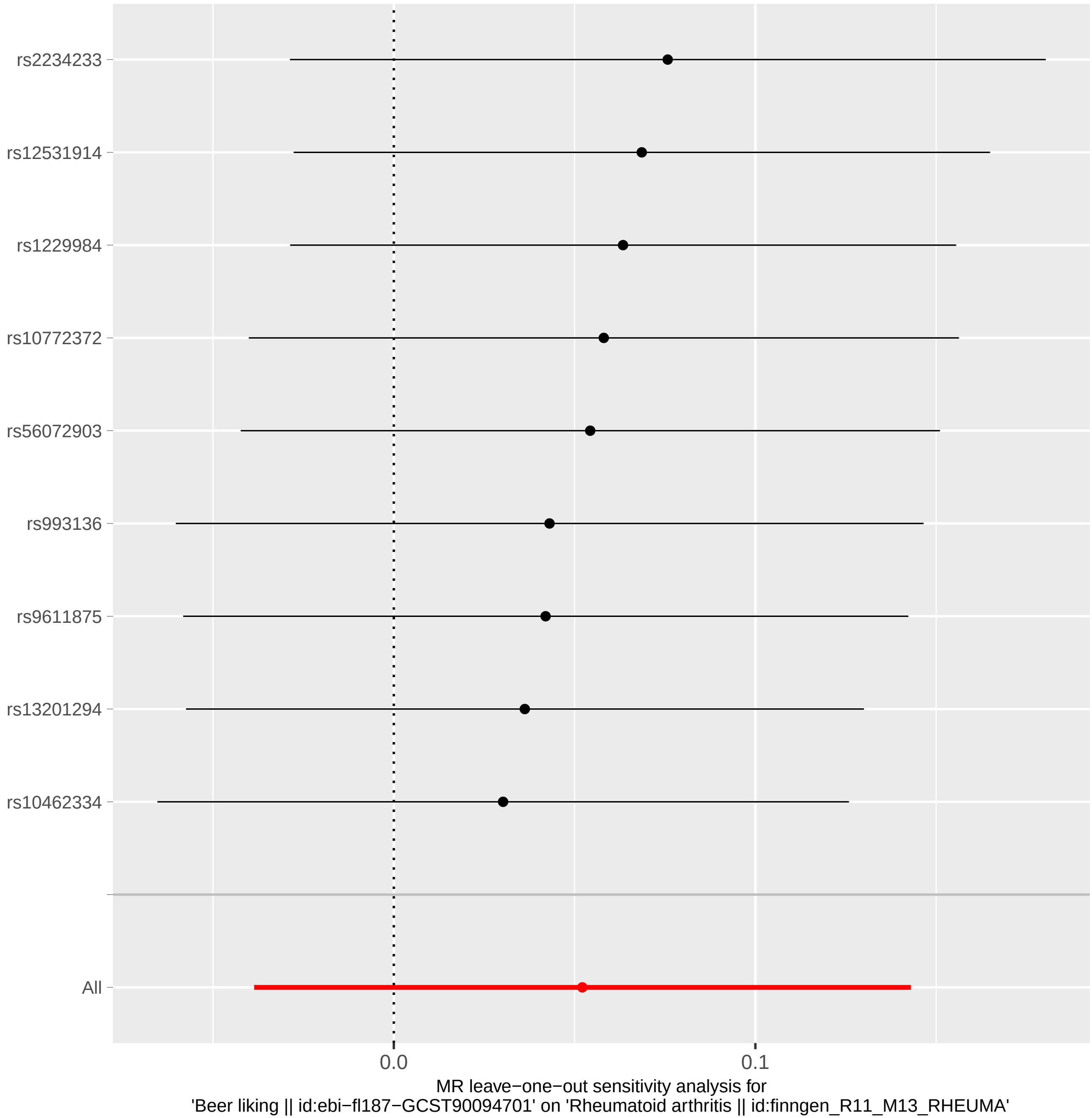

rs2234233
rs12531914
rs1229984
rs10772372
rs56072903
rs993136
rs9611875
rs13201294
rs10462334
All
0.0
0.1
MR leave−one−out sensitivity analysis for
'Beer liking || id:ebi−fl187−GCST90094701' on 'Rheumatoid arthritis || id:finngen_R11_M13_RHEUMA'

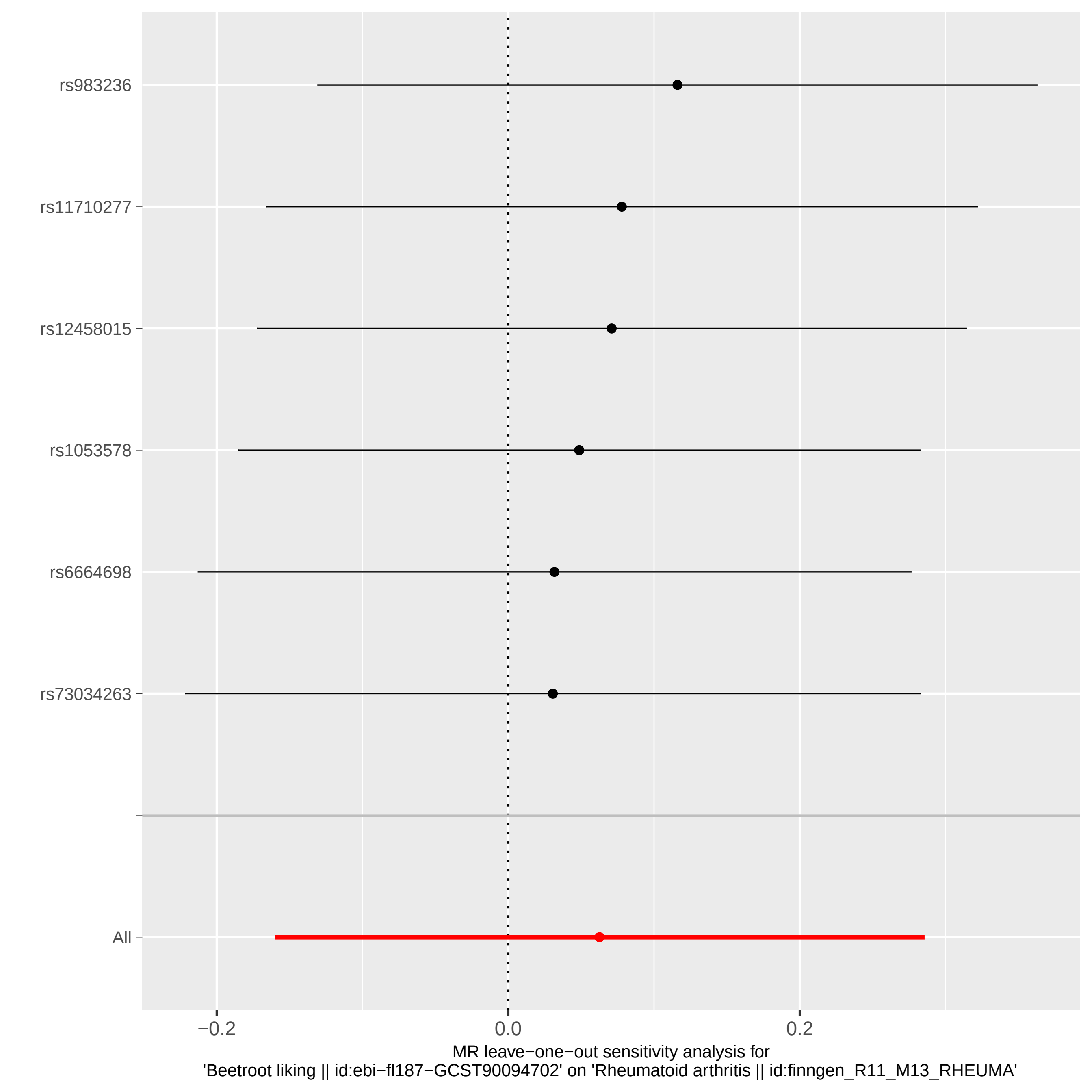
rs983236
rs11710277
rs12458015
rs1053578
rs6664698
rs73034263
All
−0.2
0.0
0.2
MR leave−one−out sensitivity analysis for
'Beetroot liking || id:ebi−fl187−GCST90094702' on 'Rheumatoid arthritis || id:finngen_R11_M13_RHEUMA'

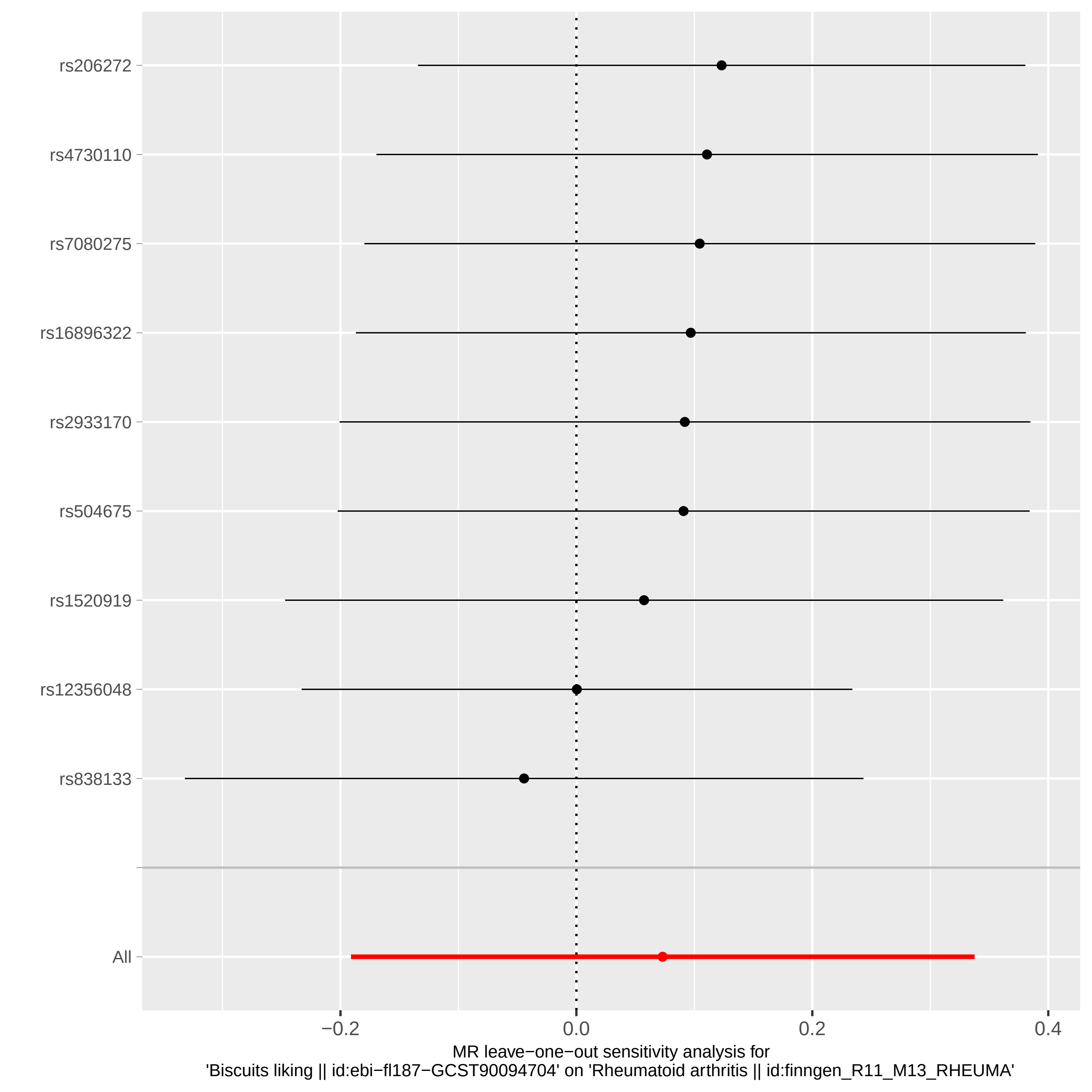

rs206272
rs4730110
rs7080275
rs16896322
rs2933170
rs504675
rs1520919
rs12356048
rs838133
All
−0.2
0.0
0.2
0.4
MR leave−one−out sensitivity analysis for
'Biscuits liking || id:ebi−fl187−GCST90094704' on 'Rheumatoid arthritis || id:finngen_R11_M13_RHEUMA'

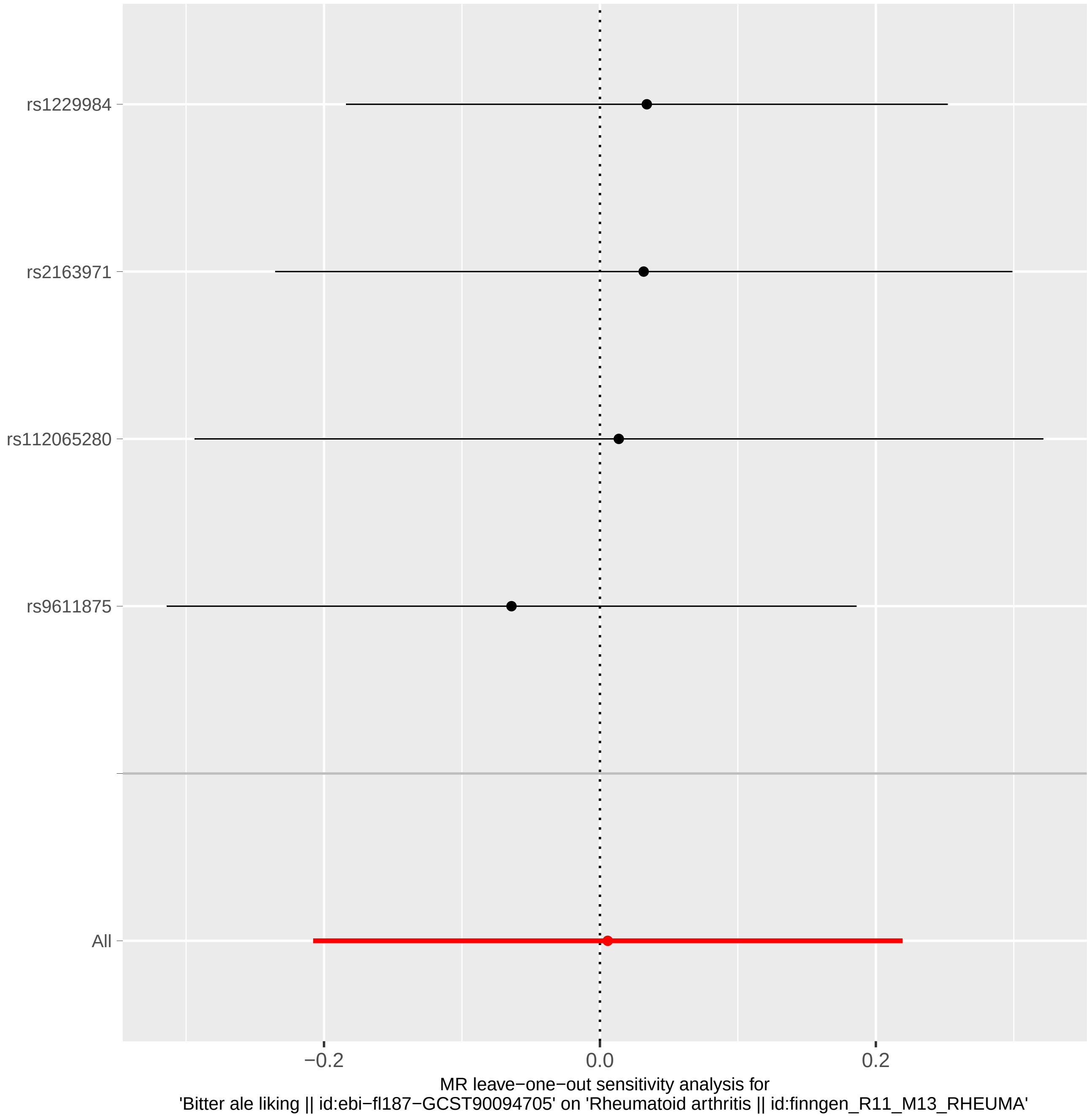
rs1229984
rs2163971
rs112065280
rs9611875
All
−0.2
0.0
0.2
MR leave−one−out sensitivity analysis for
'Bitter ale liking || id:ebi−fl187−GCST90094705' on 'Rheumatoid arthritis || id:finngen_R11_M13_RHEUMA'

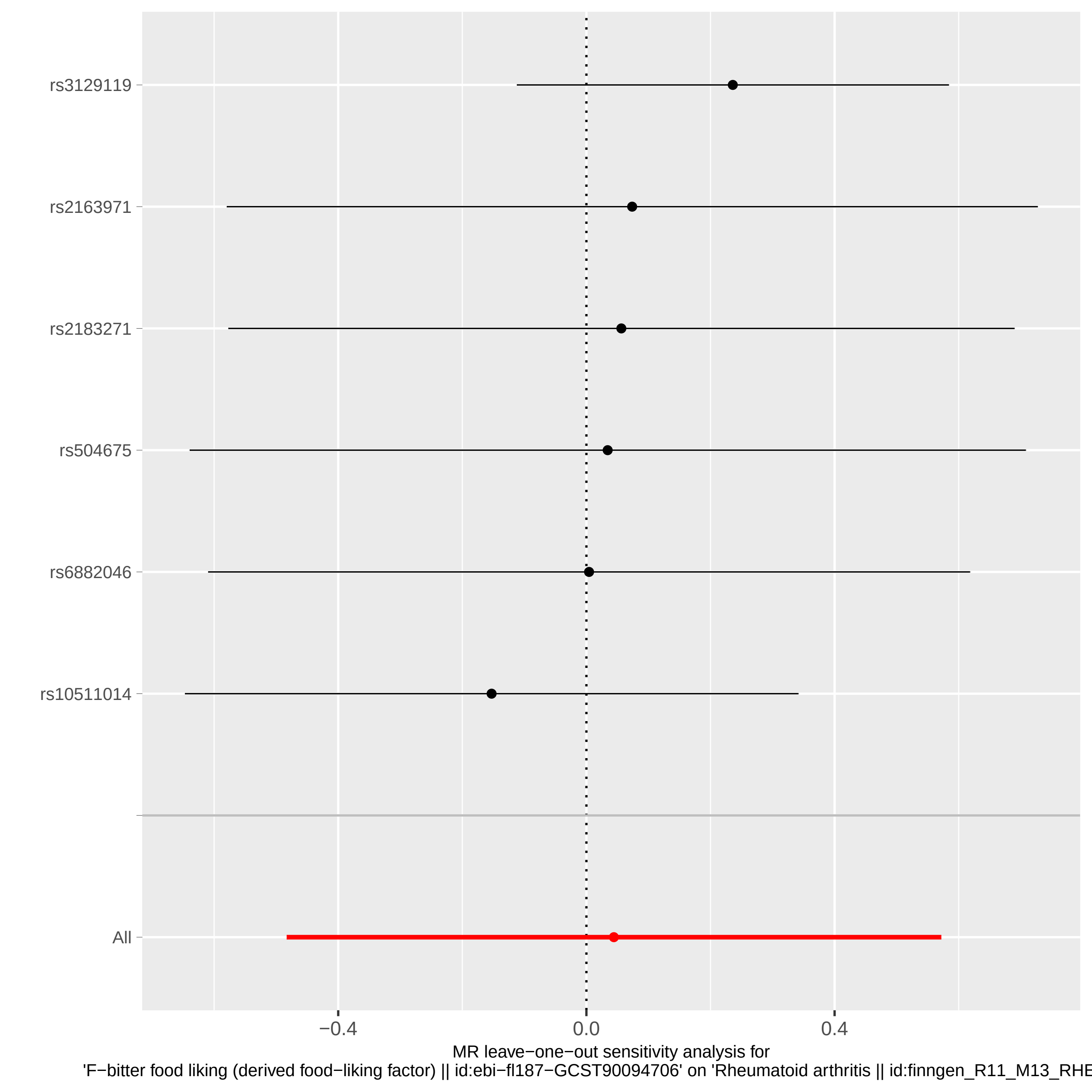
rs3129119
rs2163971
rs2183271
rs504675
rs6882046
rs10511014
All
−0.4
0.0
0.4
MR leave−one−out sensitivity analysis for
'F−bitter food liking (derived food−liking factor) || id:ebi−fl187−GCST90094706' on 'Rheumatoid arthritis || id:finngen_R11_M13_RHE

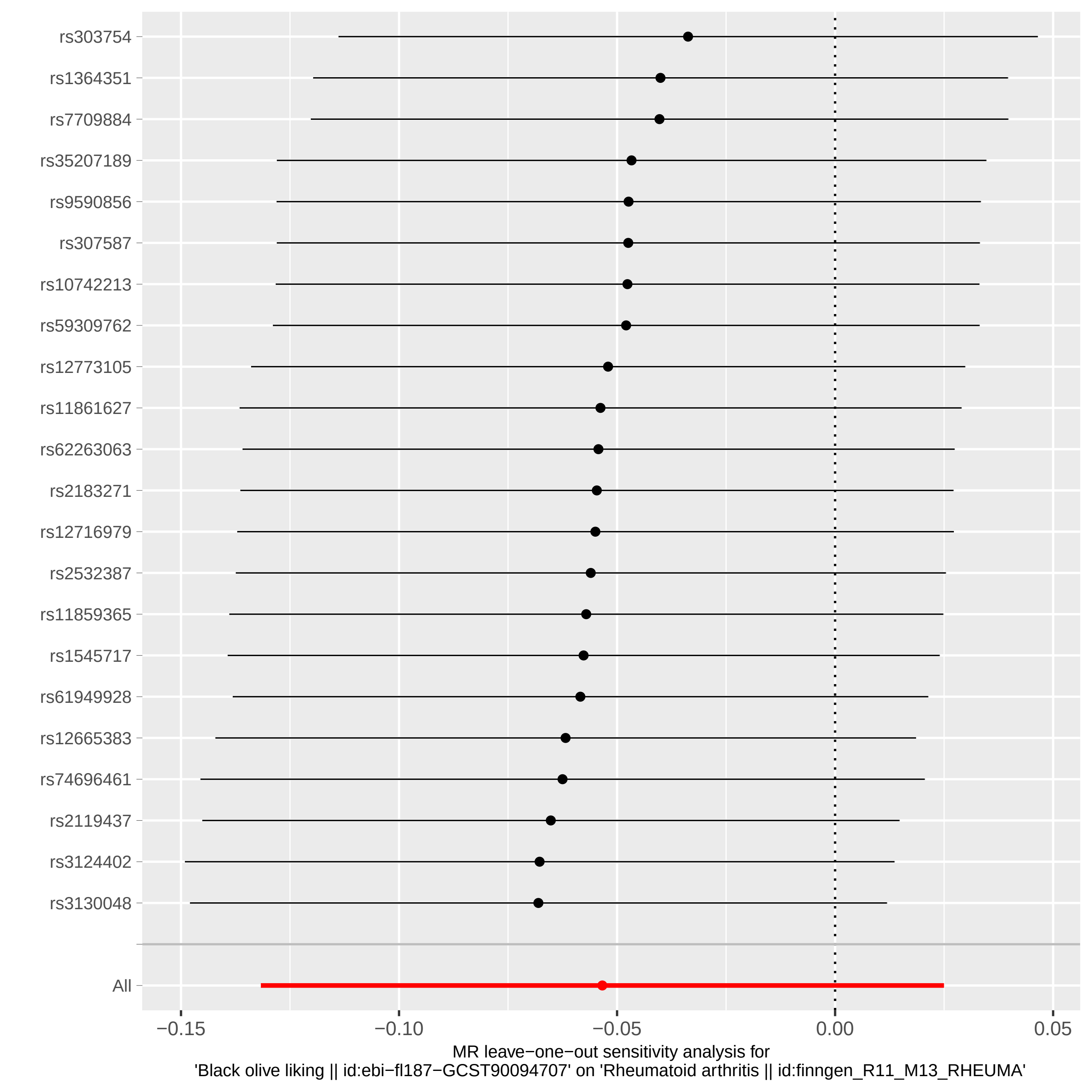

rs303754
rs1364351
rs7709884
rs35207189
rs9590856
rs307587
rs10742213
rs59309762
rs12773105
rs11861627
rs62263063
rs2183271
rs12716979
rs2532387
rs11859365
rs1545717
rs61949928
rs12665383
rs74696461
rs2119437
rs3124402
rs3130048
All
−0.15
−0.10
−0.05
0.00
0.05
MR leave−one−out sensitivity analysis for
'Black olive liking || id:ebi−fl187−GCST90094707' on 'Rheumatoid arthritis || id:finngen_R11_M13_RHEUMA'

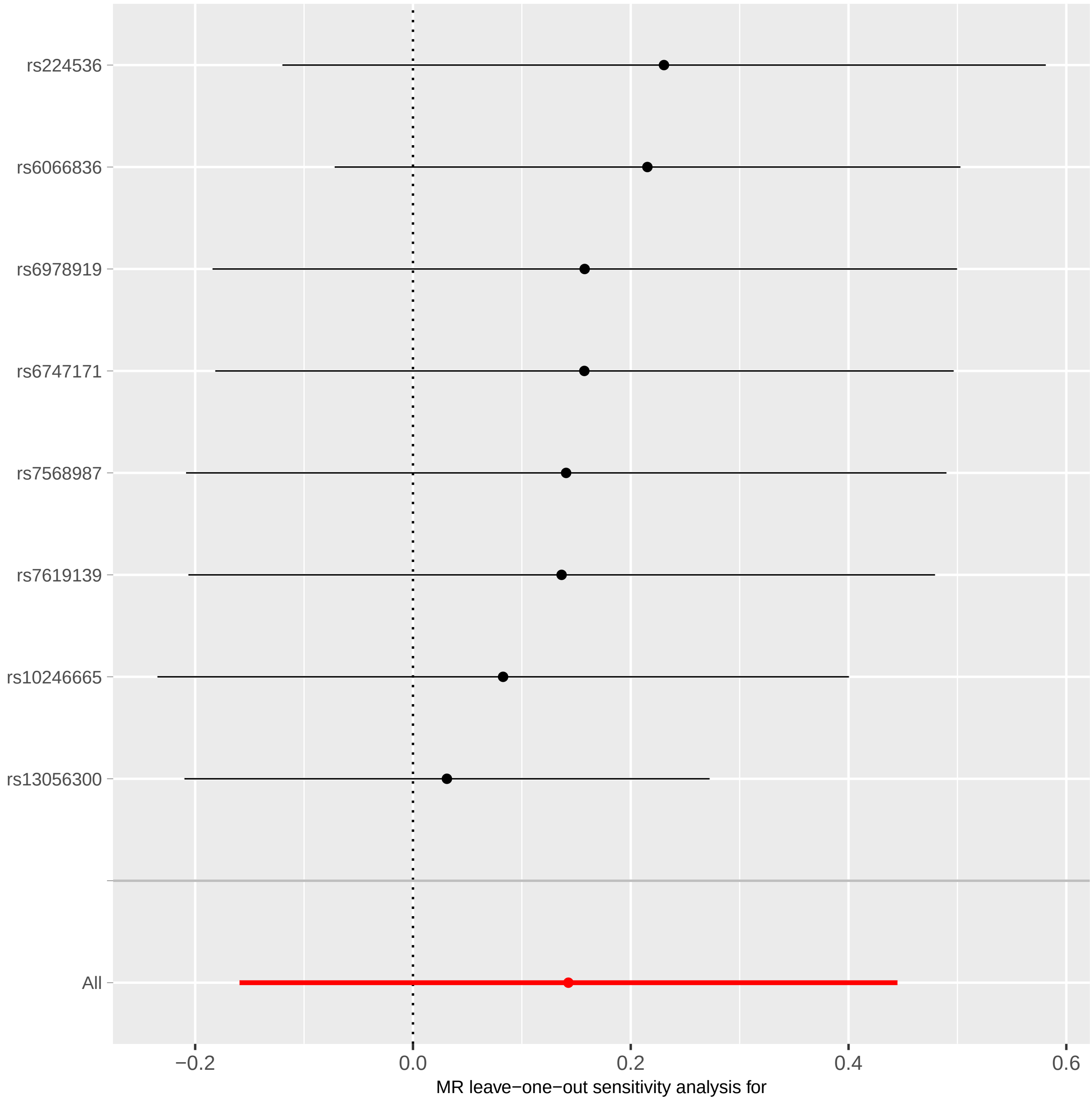

MR leave−one−out sensitivity analysis for
'Black pepper liking || id:ebi−fl187−GCST90094708' on 'Rheumatoid arthritis || id:finngen_R11_M13_RHEUMA'

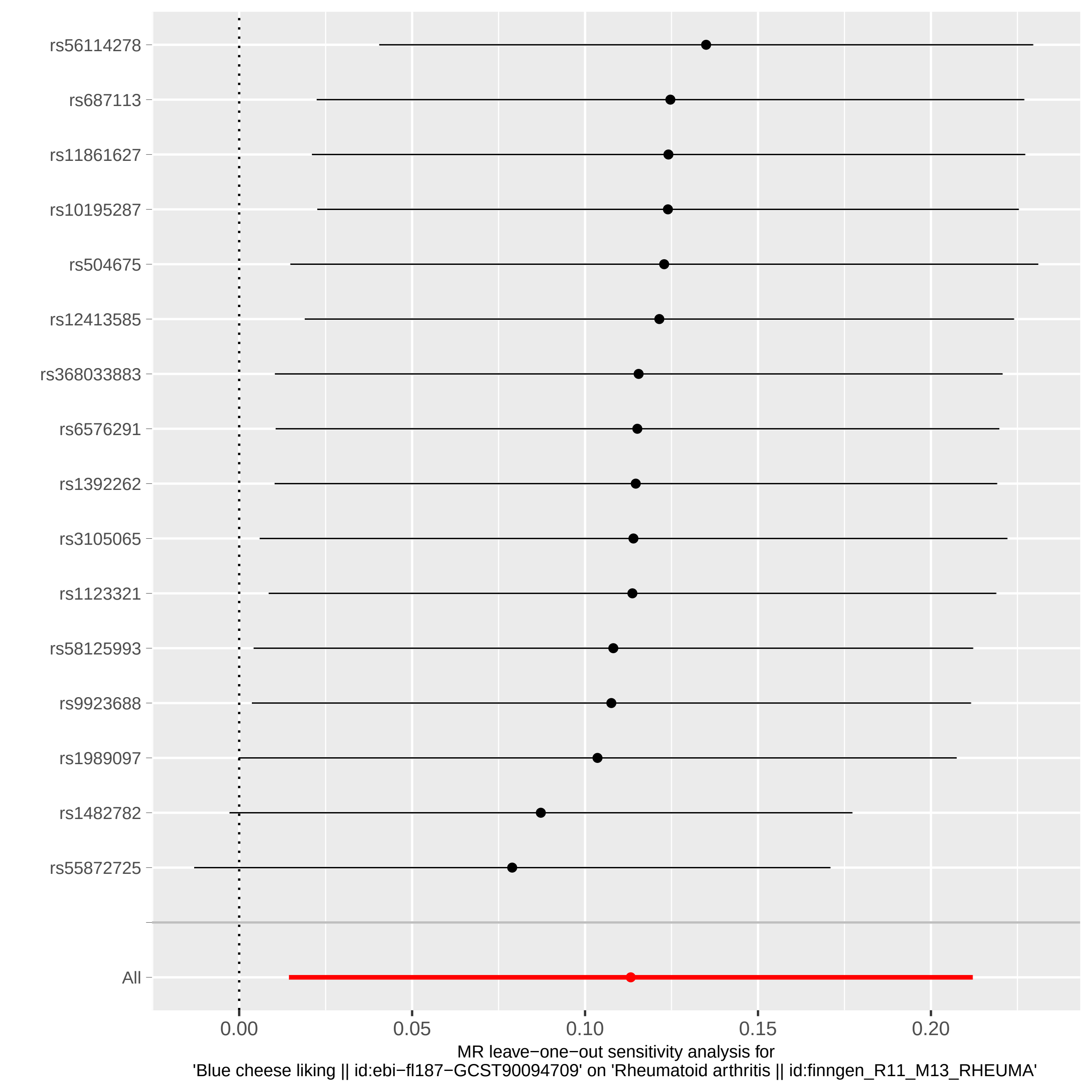
rs56114278
rs687113
rs11861627
rs10195287
rs504675
rs12413585
rs368033883
rs6576291
rs1392262
rs3105065
rs1123321
rs58125993
rs9923688
rs1989097
rs1482782
rs55872725
All
0.00
0.05
0.10
0.15
0.20
MR leave−one−out sensitivity analysis for
'Blue cheese liking || id:ebi−fl187−GCST90094709' on 'Rheumatoid arthritis || id:finngen_R11_M13_RHEUMA'

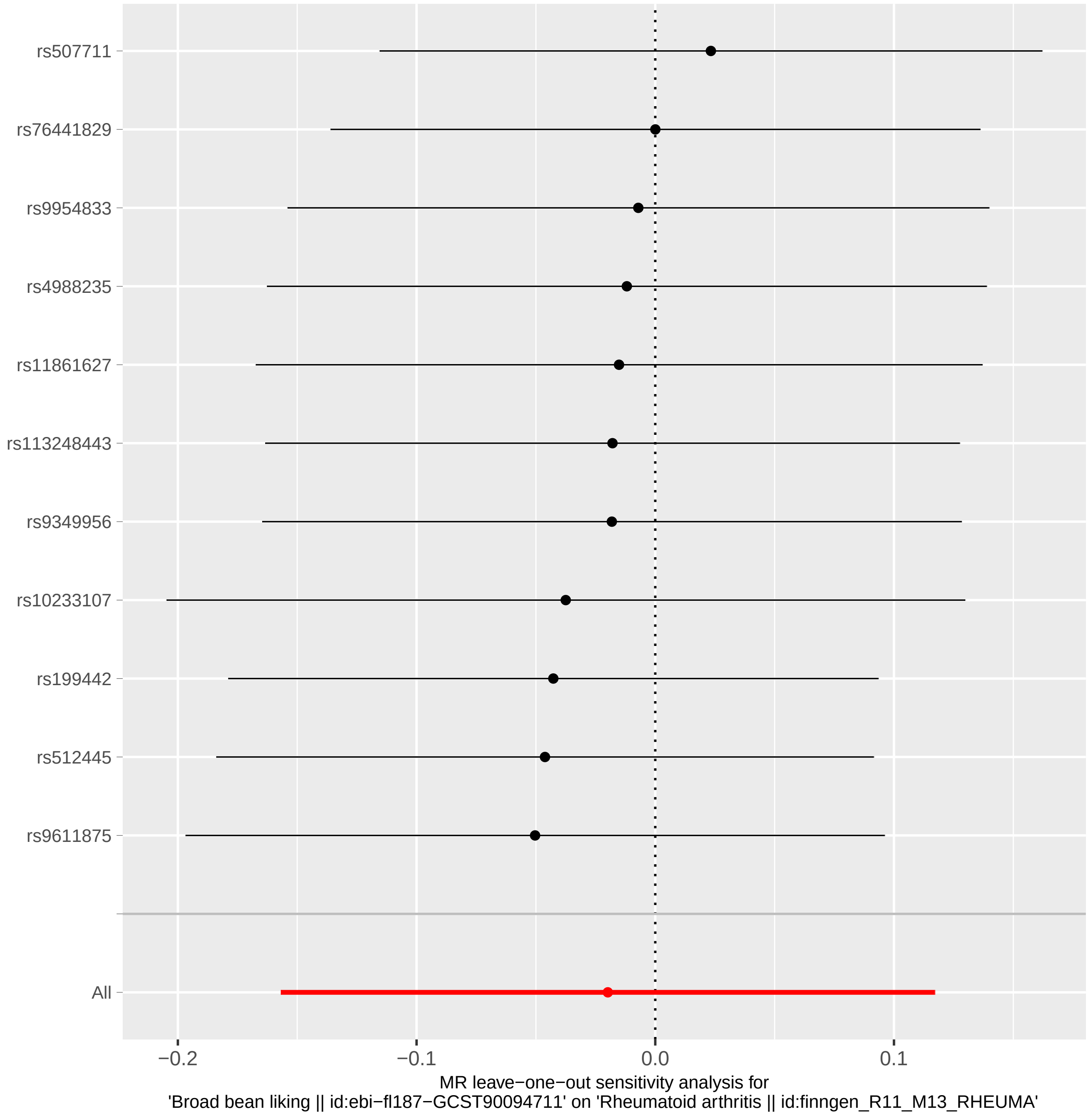

rs507711
rs76441829
rs9954833
rs4988235
rs11861627
rs113248443
rs9349956
rs10233107
rs199442
rs512445
rs9611875
All
−0.2
−0.1
0.0
0.1
MR leave−one−out sensitivity analysis for
'Broad bean liking || id:ebi−fl187−GCST90094711' on 'Rheumatoid arthritis || id:finngen_R11_M13_RHEUMA'

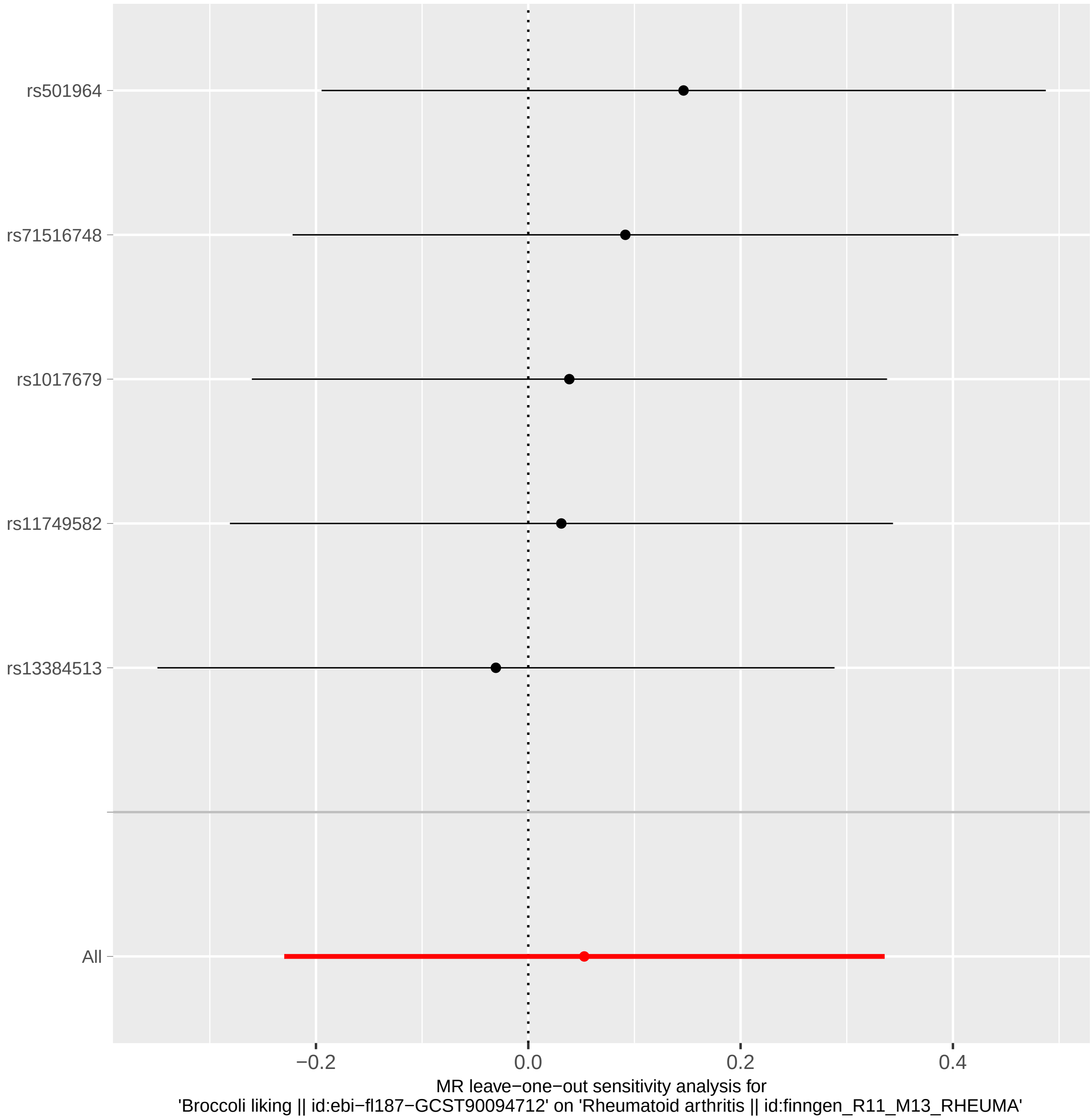
rs501964
rs71516748
rs1017679
rs11749582
rs13384513
All
−0.2
0.0
0.2
0.4
MR leave−one−out sensitivity analysis for
'Broccoli liking || id:ebi−fl187−GCST90094712' on 'Rheumatoid arthritis || id:finngen_R11_M13_RHEUMA'

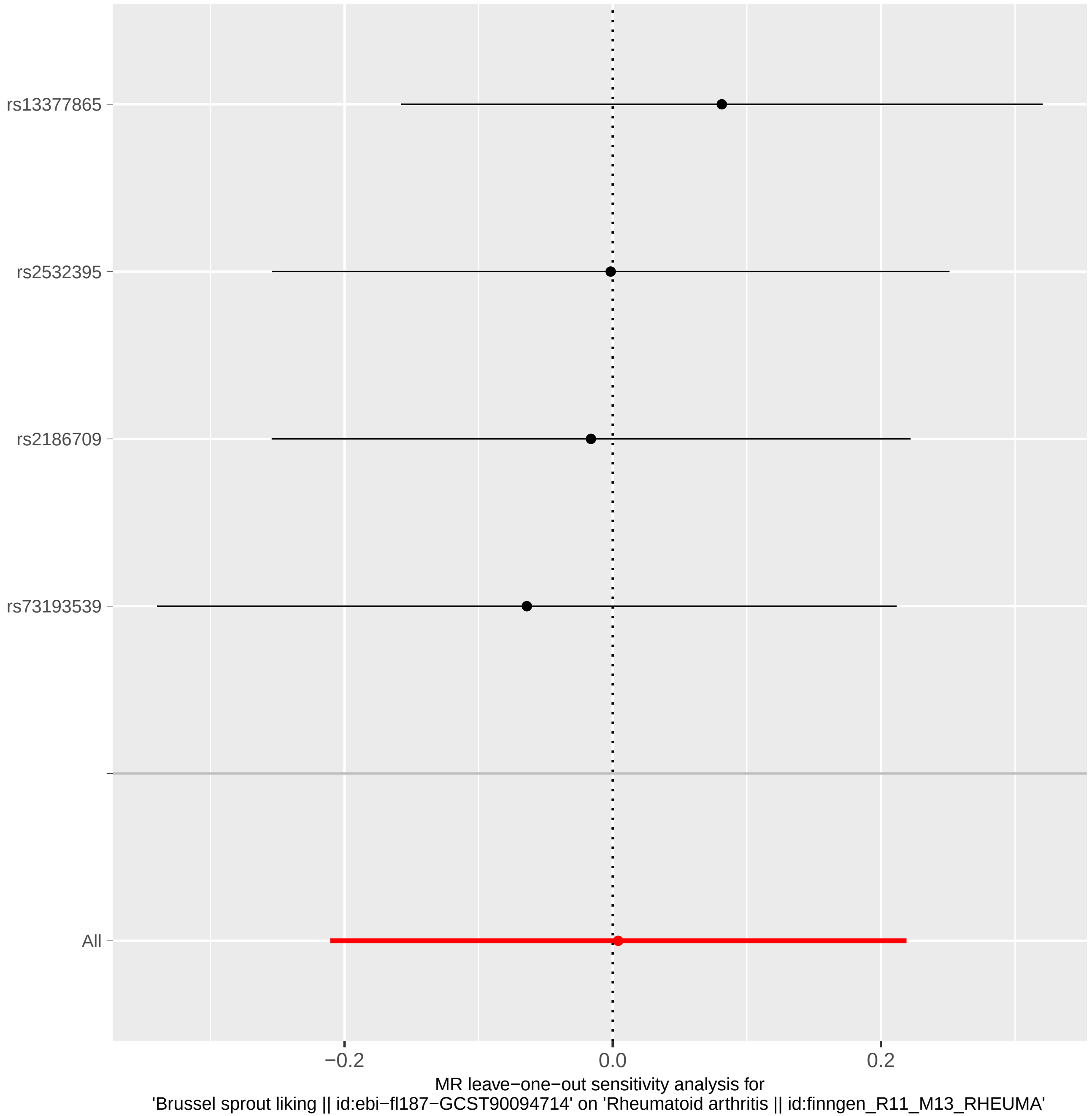
rs13377865
rs2532395
rs2186709
rs73193539
All
−0.2
0.0
0.2
MR leave−one−out sensitivity analysis for
'Brussel sprout liking || id:ebi−fl187−GCST90094714' on 'Rheumatoid arthritis || id:finngen_R11_M13_RHEUMA'

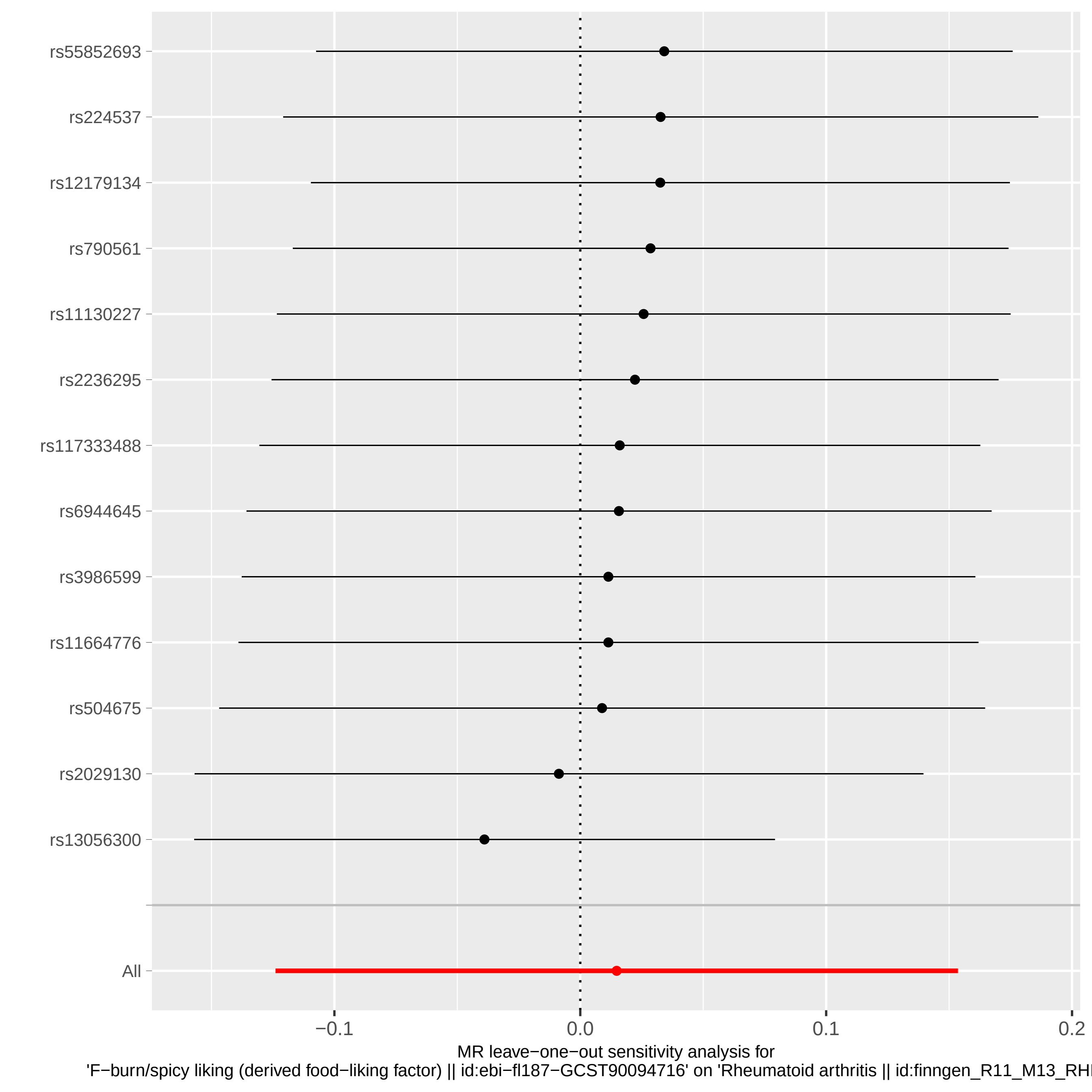

rs55852693
rs224537
rs12179134
rs790561
rs11130227
rs2236295
rs117333488
rs6944645
rs3986599
rs11664776
rs504675
rs2029130
rs13056300
All
−0.1
0.0
0.1
0.2
MR leave−one−out sensitivity analysis for
'F−burn/spicy liking (derived food−liking factor) || id:ebi−fl187−GCST90094716' on 'Rheumatoid arthritis || id:finngen_R11_M13_RH

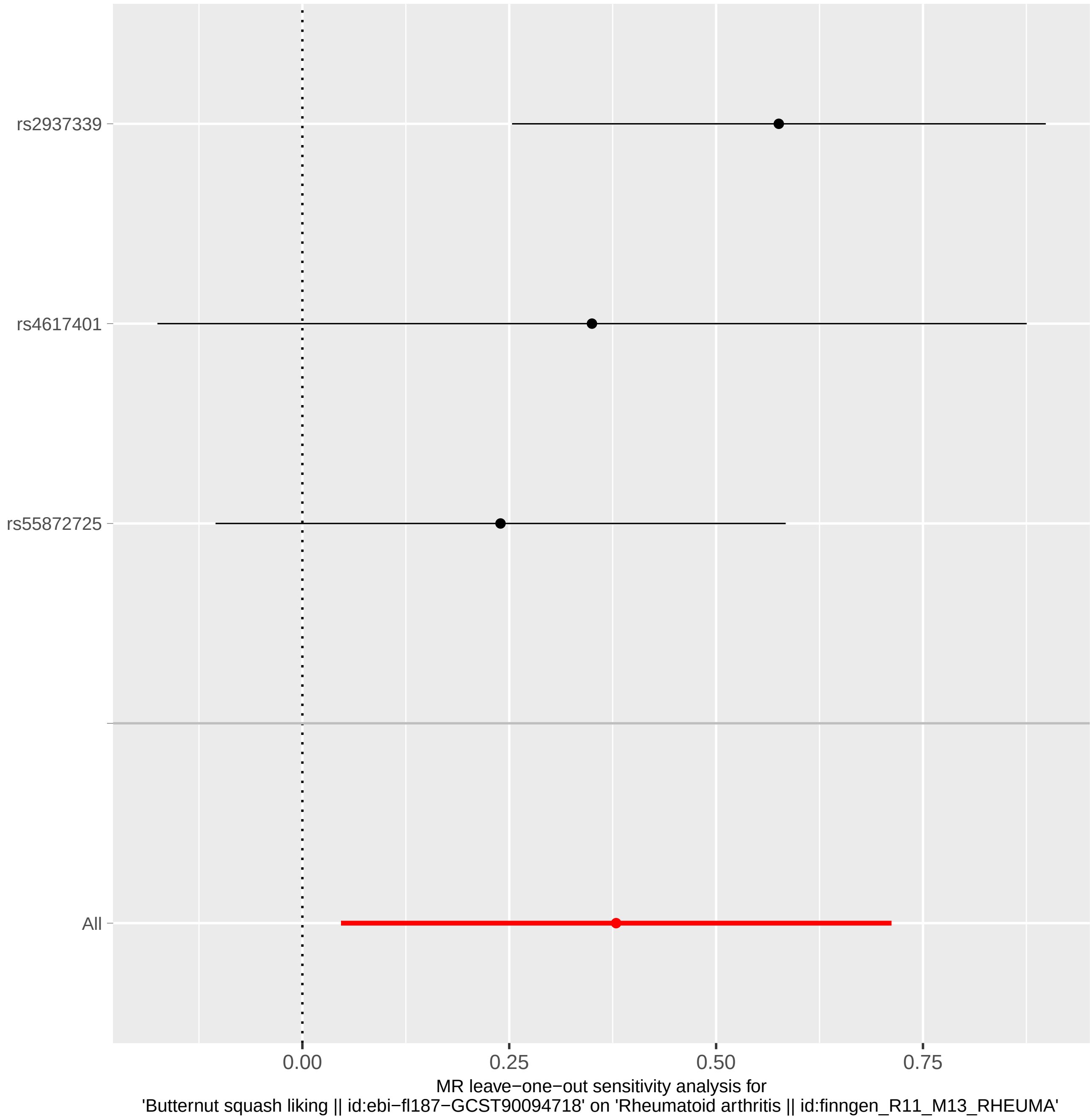
rs2937339
rs4617401
rs55872725
All
0.00
0.25
0.50
0.75
MR leave−one−out sensitivity analysis for
'Butternut squash liking || id:ebi−fl187−GCST90094718' on 'Rheumatoid arthritis || id:finngen_R11_M13_RHEUMA'

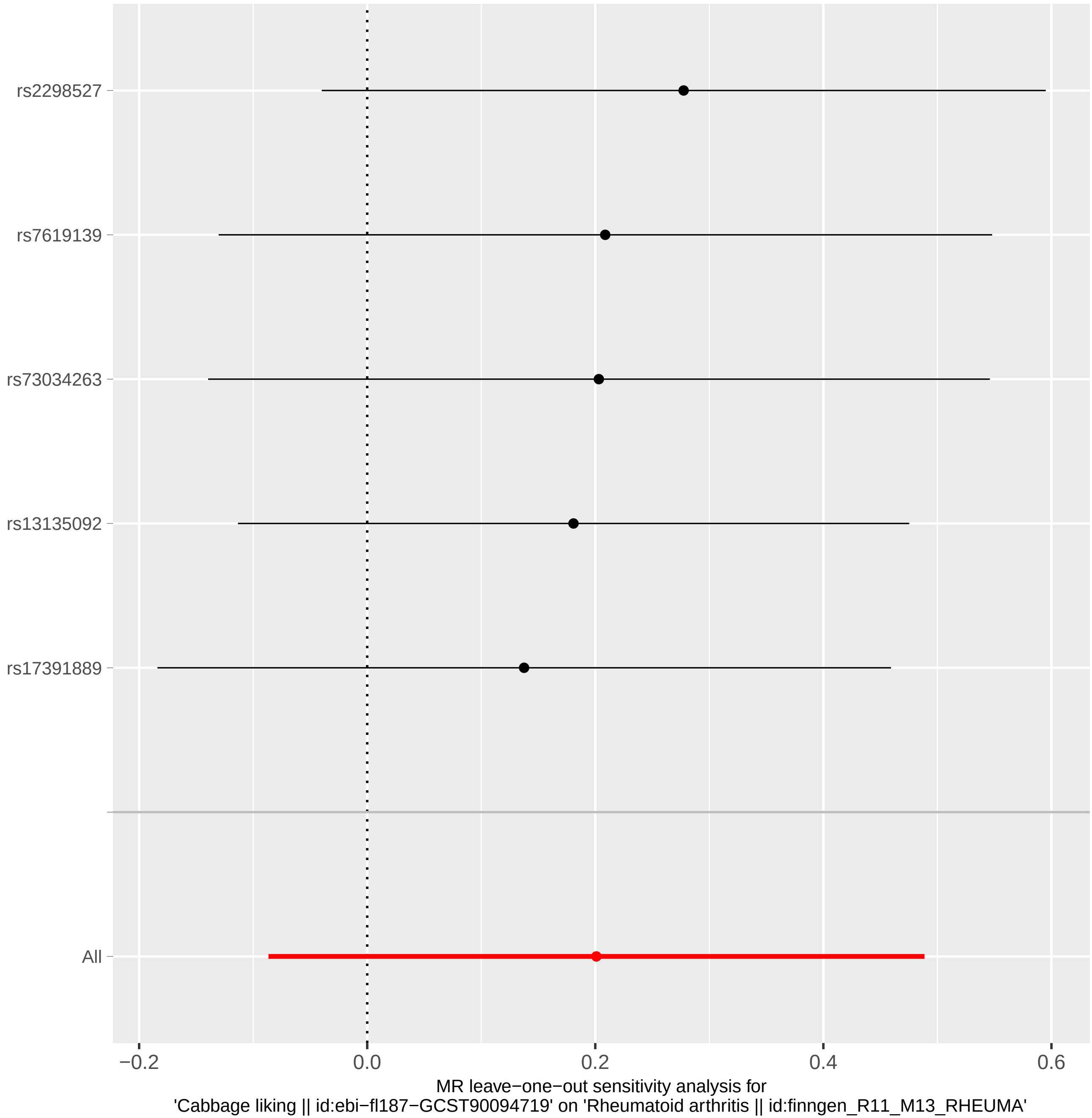

rs2298527
rs7619139
rs73034263
rs13135092
rs17391889
All
−0.2
0.0
0.2
0.4
0.6
MR leave−one−out sensitivity analysis for
'Cabbage liking || id:ebi−fl187−GCST90094719' on 'Rheumatoid arthritis || id:finngen_R11_M13_RHEUMA'

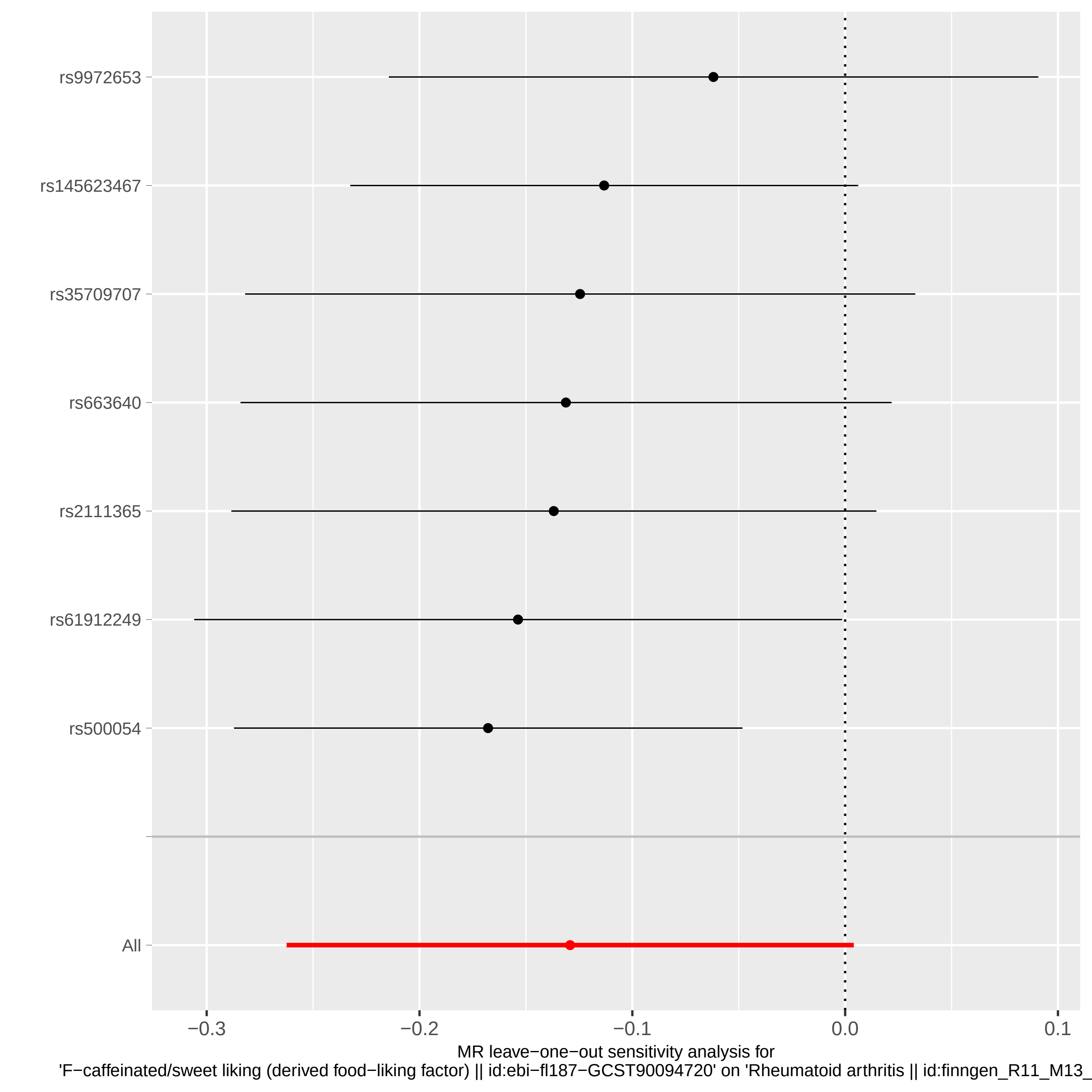

rs9972653
rs145623467
rs35709707
rs663640
rs2111365
rs61912249
rs500054
All
−0.3
−0.2
−0.1
0.0
0.1
MR leave−one−out sensitivity analysis for
'F−caffeinated/sweet liking (derived food−liking factor) || id:ebi−fl187−GCST90094720' on 'Rheumatoid arthritis || id:finngen_R11_M13_

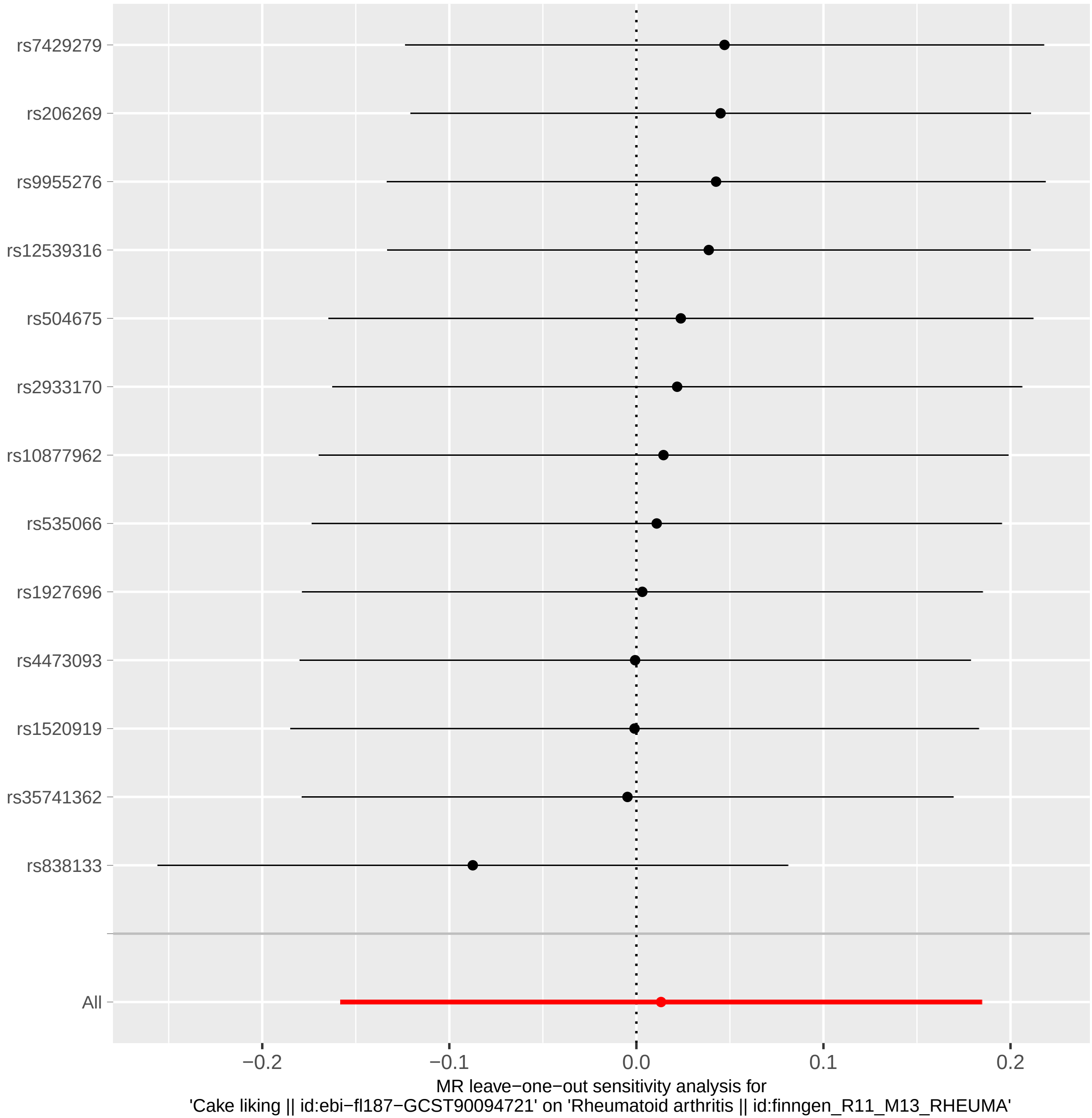
rs7429279
rs206269
rs9955276
rs12539316
rs504675
rs2933170
rs10877962
rs535066
rs1927696
rs4473093
rs1520919
rs35741362
rs838133
All
−0.2
−0.1
0.0
0.1
0.2
MR leave−one−out sensitivity analysis for
'Cake liking || id:ebi−fl187−GCST90094721' on 'Rheumatoid arthritis || id:finngen_R11_M13_RHEUMA'

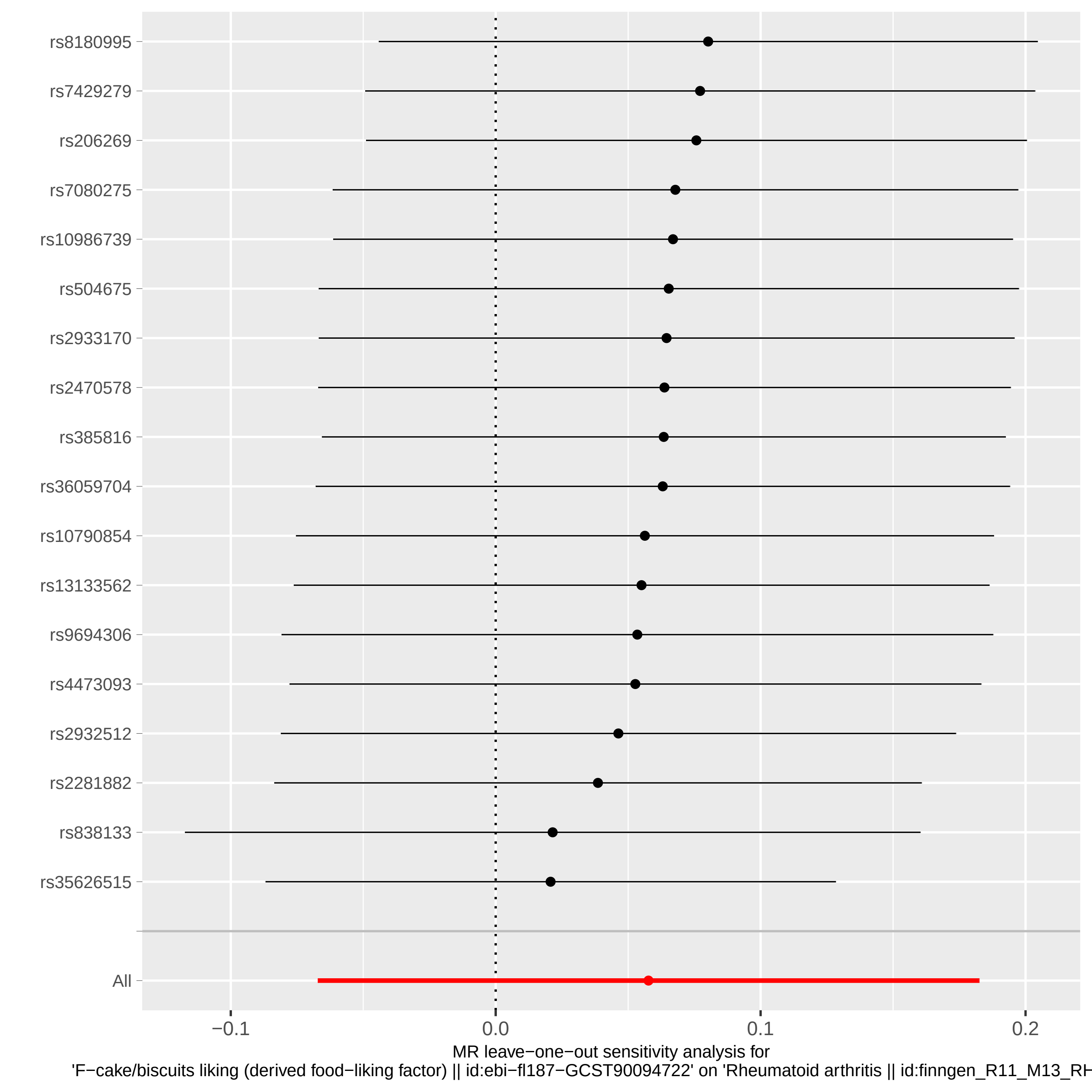
rs8180995
rs7429279
rs206269
rs7080275
rs10986739
rs504675
rs2933170
rs2470578
rs385816
rs36059704
rs10790854
rs13133562
rs9694306
rs4473093
rs2932512
rs2281882
rs838133
rs35626515
All
−0.1
0.0
0.1
0.2
MR leave−one−out sensitivity analysis for
'F−cake/biscuits liking (derived food−liking factor) || id:ebi−fl187−GCST90094722' on 'Rheumatoid arthritis || id:finngen_R11_M13_RH

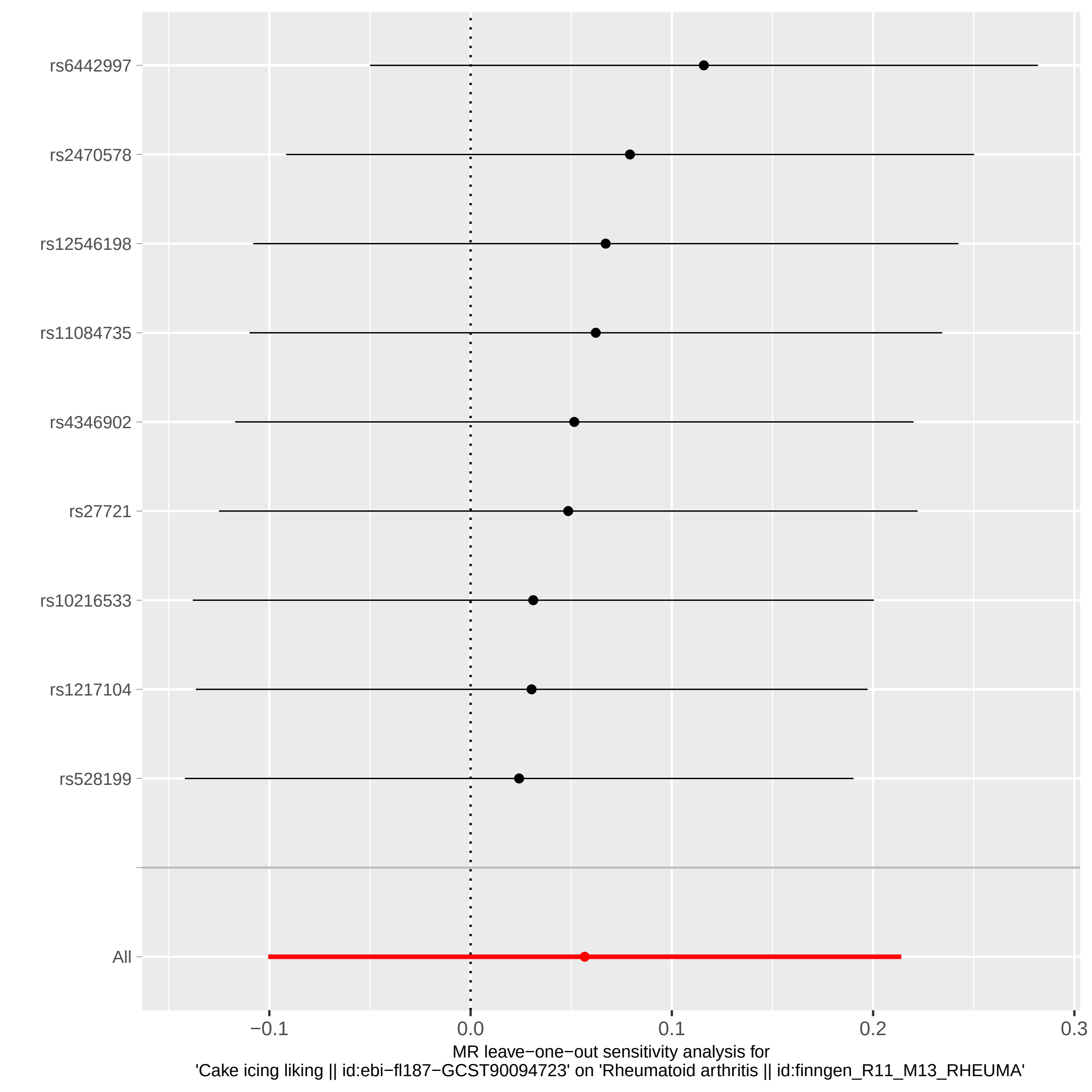
rs6442997
rs2470578
rs12546198
rs11084735
rs4346902
rs27721
rs10216533
rs1217104
rs528199
All
−0.1
0.0
0.1
0.2
0.3
MR leave−one−out sensitivity analysis for
'Cake icing liking || id:ebi−fl187−GCST90094723' on 'Rheumatoid arthritis || id:finngen_R11_M13_RHEUMA'

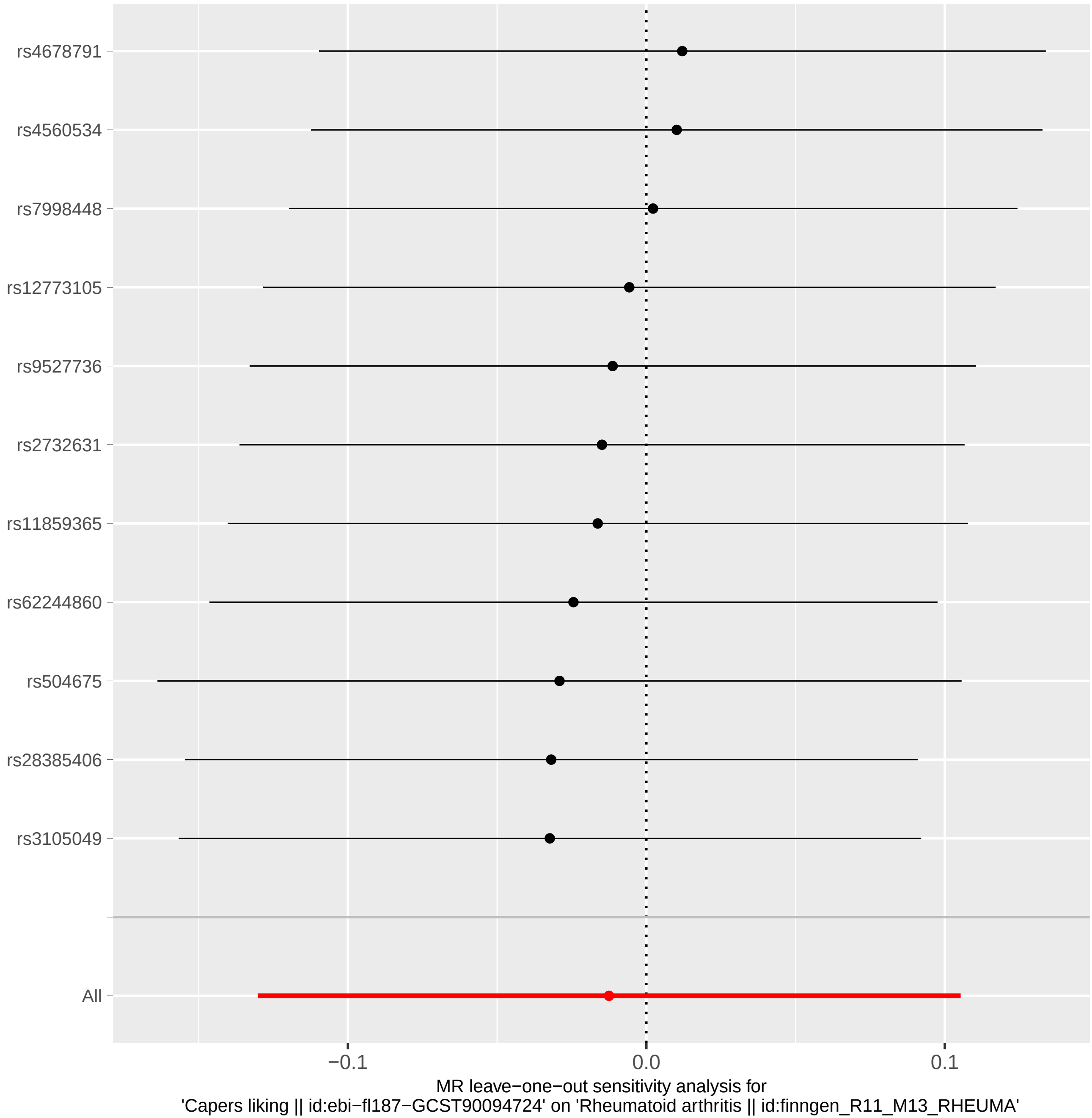

rs4678791
rs4560534
rs7998448
rs12773105
rs9527736
rs2732631
rs11859365
rs62244860
rs504675
rs28385406
rs3105049
All
−0.1
0.0
0.1
MR leave−one−out sensitivity analysis for
'Capers liking || id:ebi−fl187−GCST90094724' on 'Rheumatoid arthritis || id:finngen_R11_M13_RHEUMA'

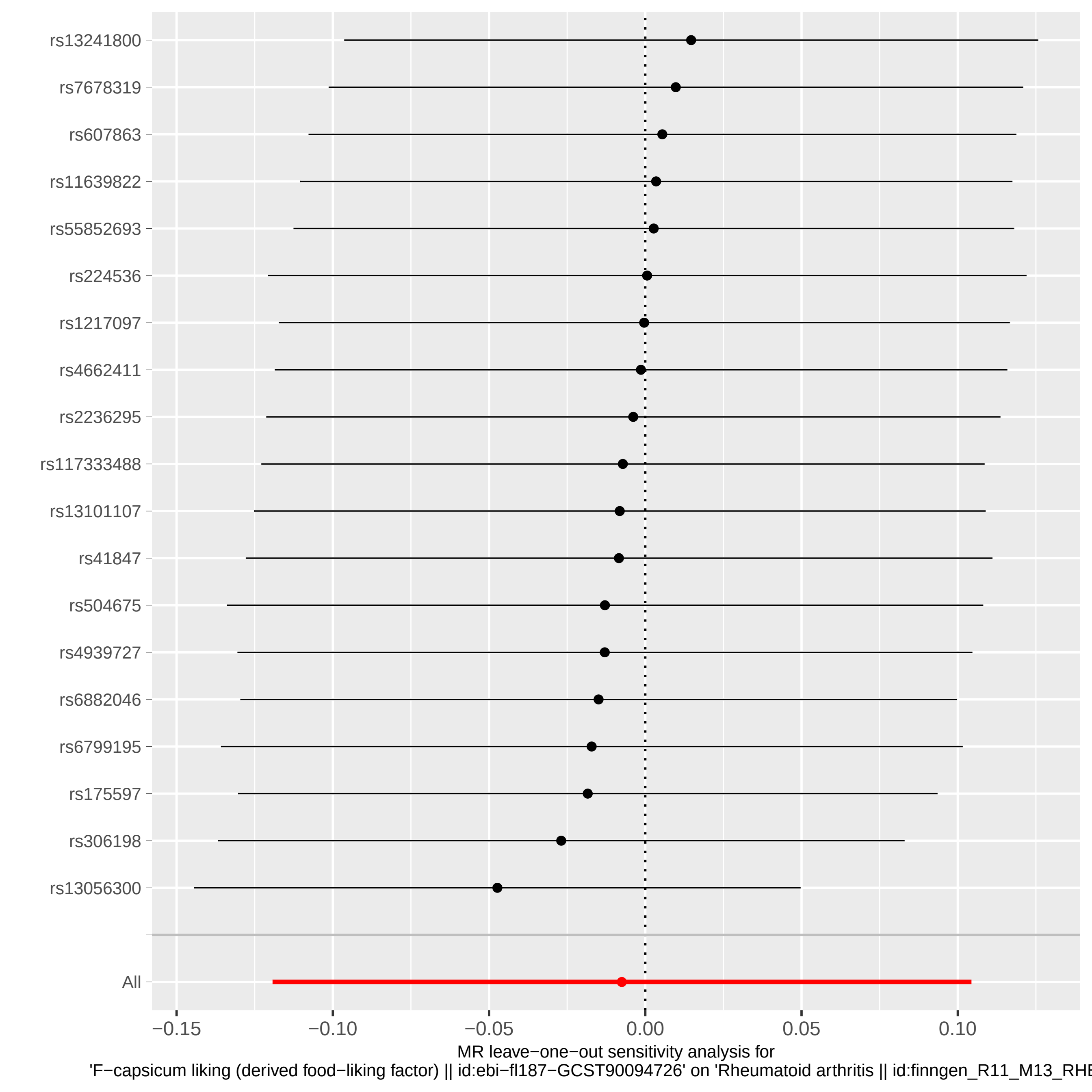

rs13241800
rs7678319
rs607863
rs11639822
rs55852693
rs224536
rs1217097
rs4662411
rs2236295
rs117333488
rs13101107
rs41847
rs504675
rs4939727
rs6882046
rs6799195
rs175597
rs306198
rs13056300
All
−0.15
−0.10
−0.05
0.00
0.05
0.10
MR leave−one−out sensitivity analysis for
'F−capsicum liking (derived food−liking factor) || id:ebi−fl187−GCST90094726' on 'Rheumatoid arthritis || id:finngen_R11_M13_RHE

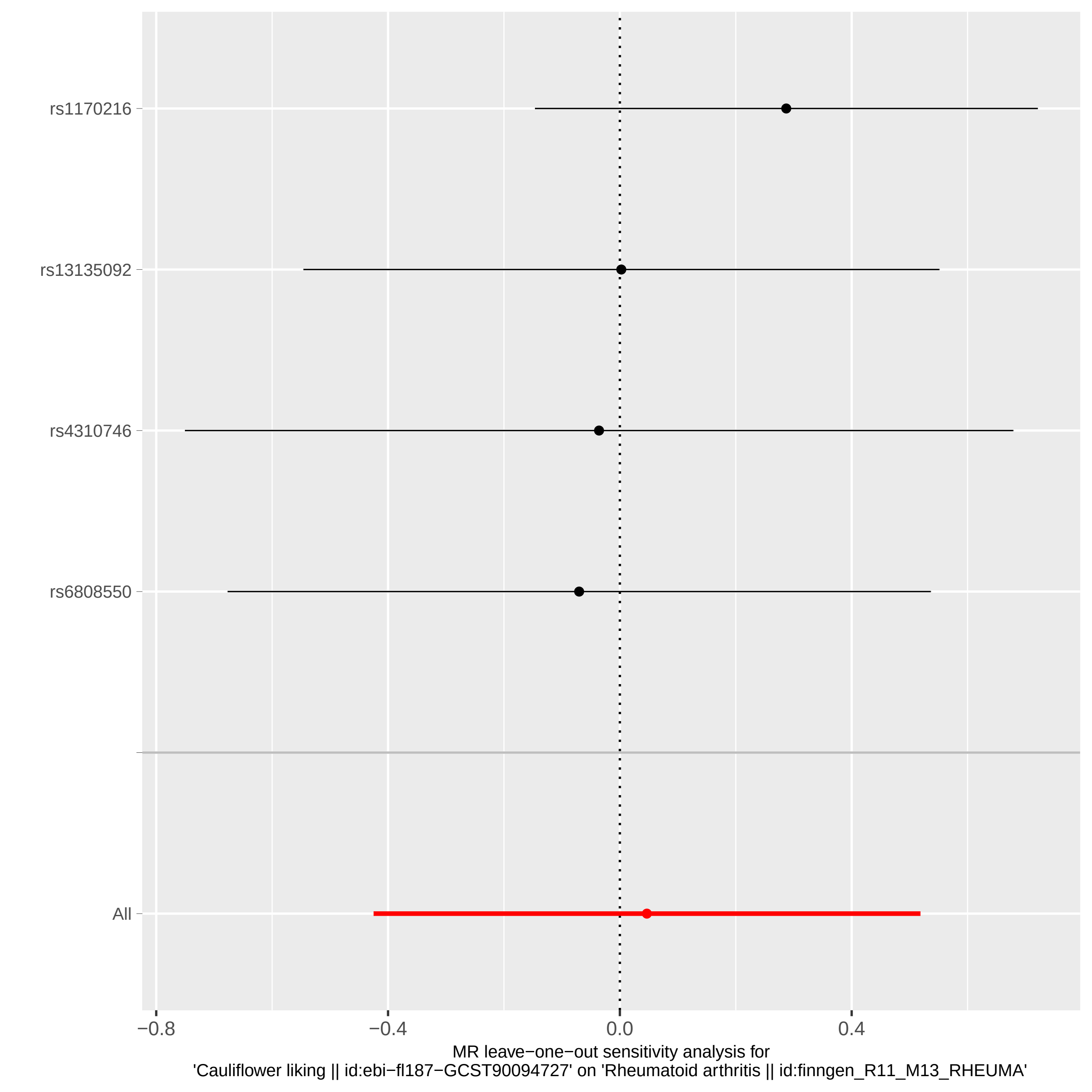
rs1170216
rs13135092
rs4310746
rs6808550
All
−0.8
−0.4
0.0
0.4
MR leave−one−out sensitivity analysis for
'Cauliflower liking || id:ebi−fl187−GCST90094727' on 'Rheumatoid arthritis || id:finngen_R11_M13_RHEUMA'

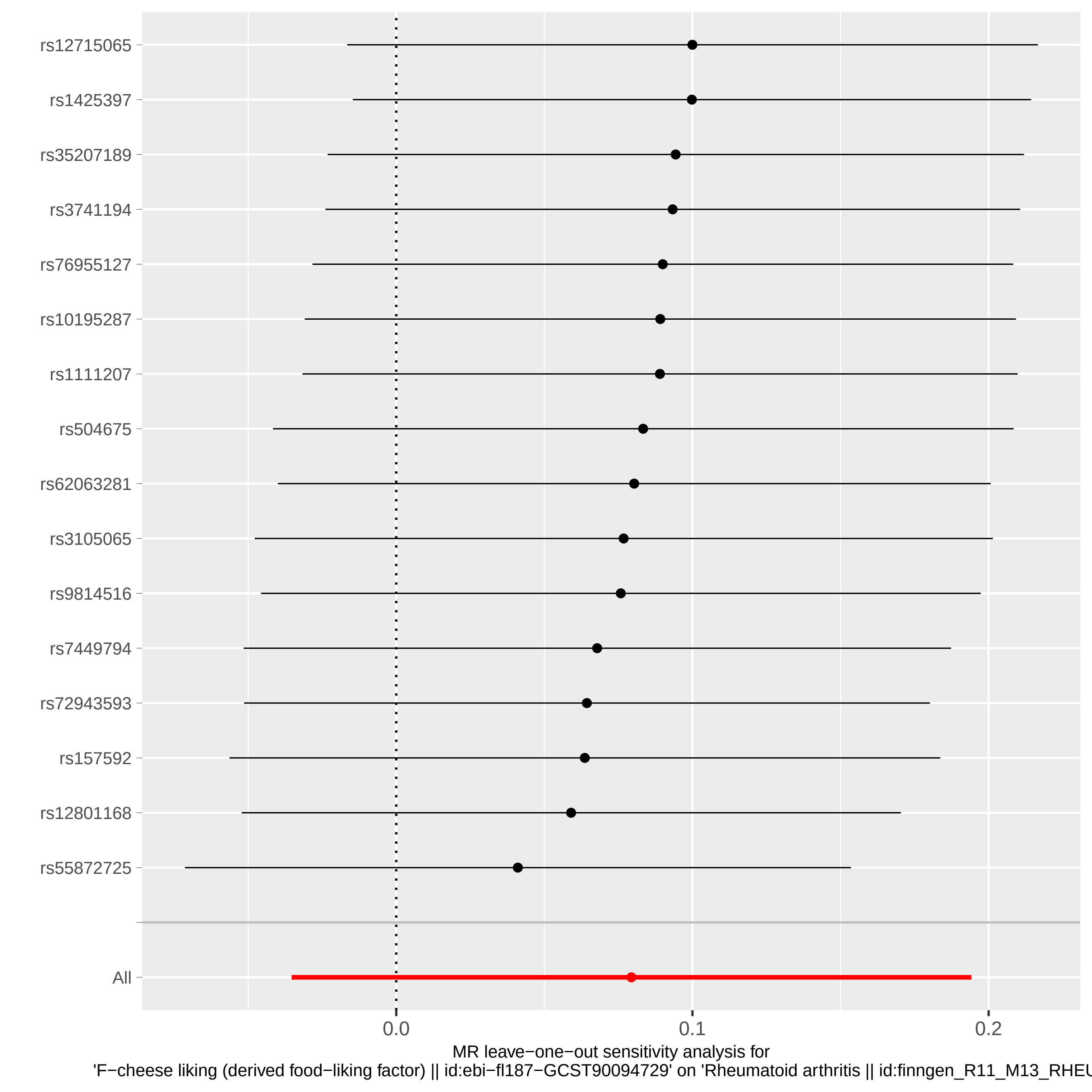
rs12715065
rs1425397
rs35207189
rs3741194
rs76955127
rs10195287
rs1111207
rs504675
rs62063281
rs3105065
rs9814516
rs7449794
rs72943593
rs157592
rs12801168
rs55872725
All
0.0
0.1
0.2
MR leave−one−out sensitivity analysis for
'F−cheese liking (derived food−liking factor) || id:ebi−fl187−GCST90094729' on 'Rheumatoid arthritis || id:finngen_R11_M13_RHEU

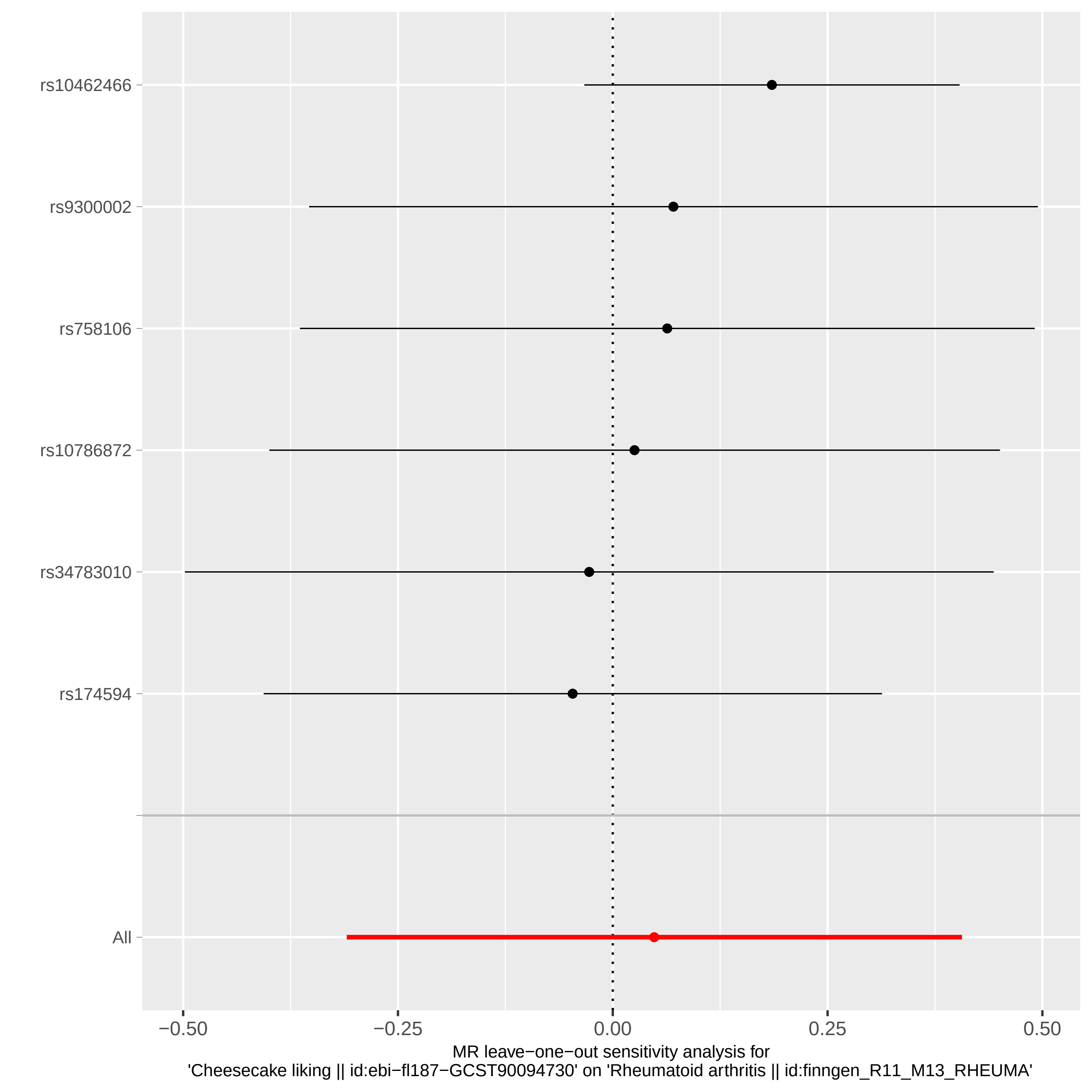

rs10462466
rs9300002
rs758106
rs10786872
rs34783010
rs174594
All
−0.50
−0.25
0.00
0.25
0.50
MR leave−one−out sensitivity analysis for
'Cheesecake liking || id:ebi−fl187−GCST90094730' on 'Rheumatoid arthritis || id:finngen_R11_M13_RHEUMA'

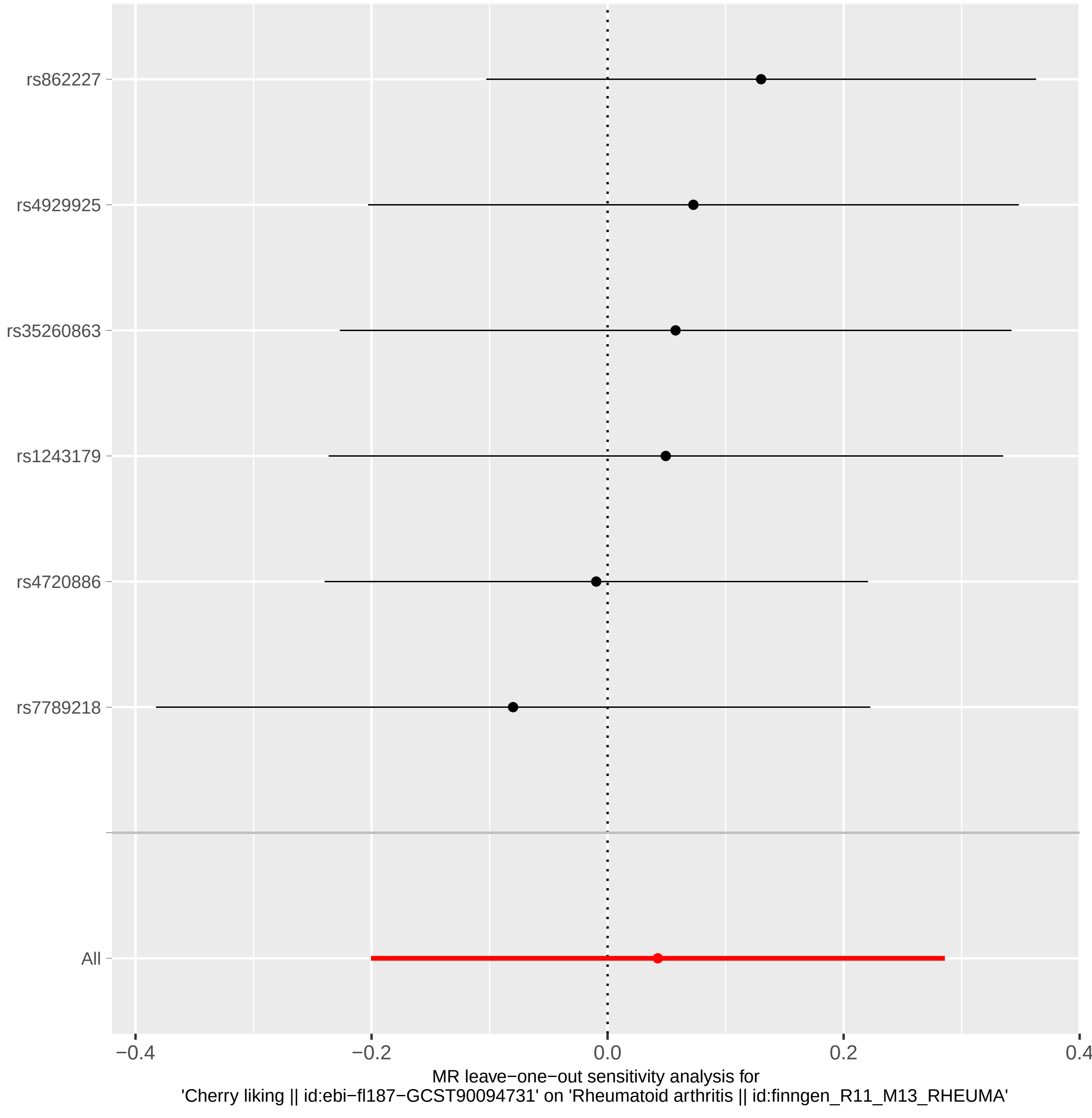
rs862227
rs4929925
rs35260863
rs1243179
rs4720886
rs7789218
All
−0.4
−0.2
0.0
0.2
0.4
MR leave−one−out sensitivity analysis for
'Cherry liking || id:ebi−fl187−GCST90094731' on 'Rheumatoid arthritis || id:finngen_R11_M13_RHEUMA'

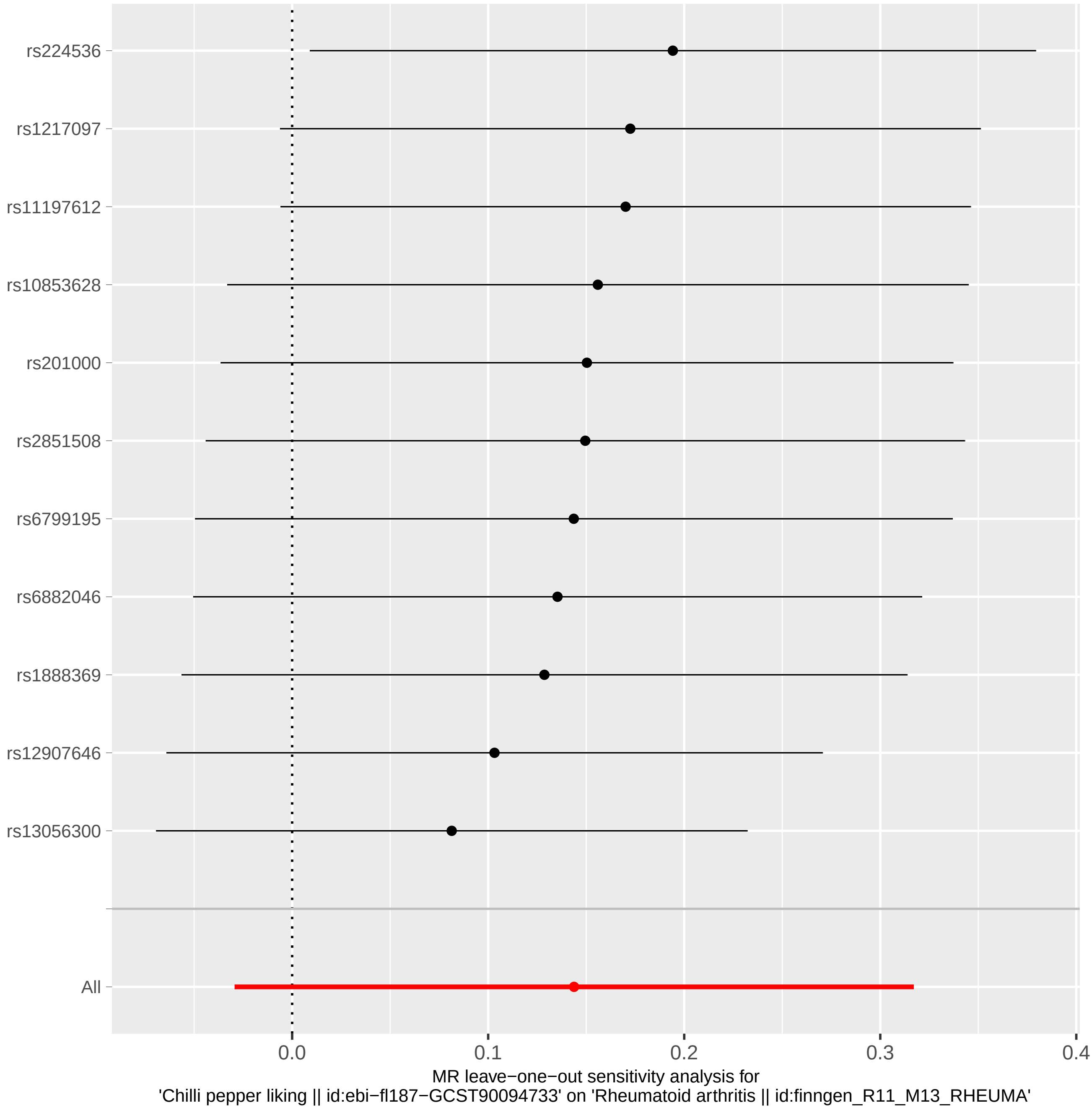
rs224536
rs1217097
rs11197612
rs10853628
rs201000
rs2851508
rs6799195
rs6882046
rs1888369
rs12907646
rs13056300
All
0.0
0.1
0.2
0.3
0.4
MR leave−one−out sensitivity analysis for
'Chilli pepper liking || id:ebi−fl187−GCST90094733' on 'Rheumatoid arthritis || id:finngen_R11_M13_RHEUMA'

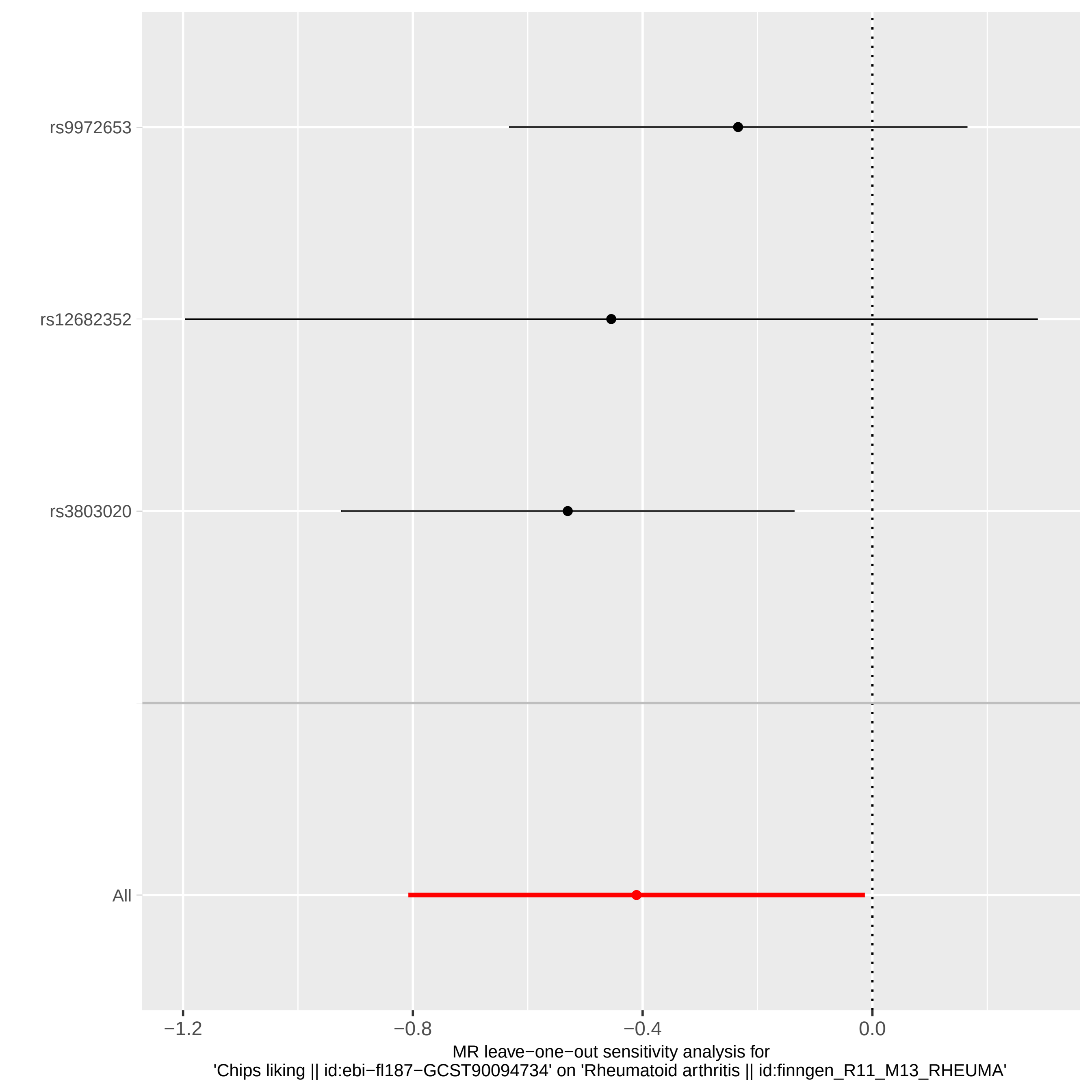

rs9972653
rs12682352
rs3803020
All
−1.2
−0.8
−0.4
0.0
MR leave−one−out sensitivity analysis for
'Chips liking || id:ebi−fl187−GCST90094734' on 'Rheumatoid arthritis || id:finngen_R11_M13_RHEUMA'

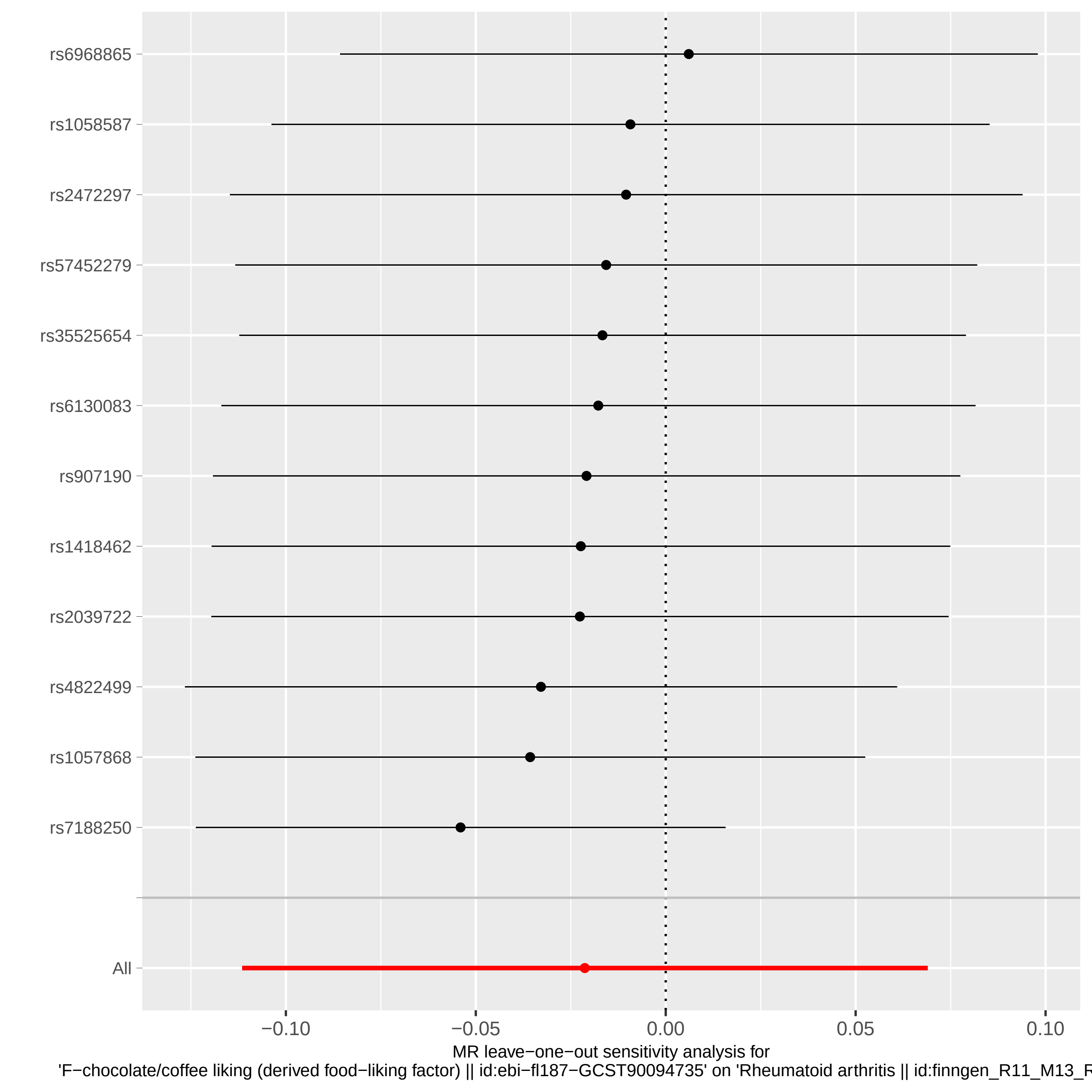
rs6968865
rs1058587
rs2472297
rs57452279
rs35525654
rs6130083
rs907190
rs1418462
rs2039722
rs4822499
rs1057868
rs7188250
All
−0.10
−0.05
0.00
0.05
0.10
MR leave−one−out sensitivity analysis for
'F−chocolate/coffee liking (derived food−liking factor) || id:ebi−fl187−GCST90094735' on 'Rheumatoid arthritis || id:finngen_R11_M13_R

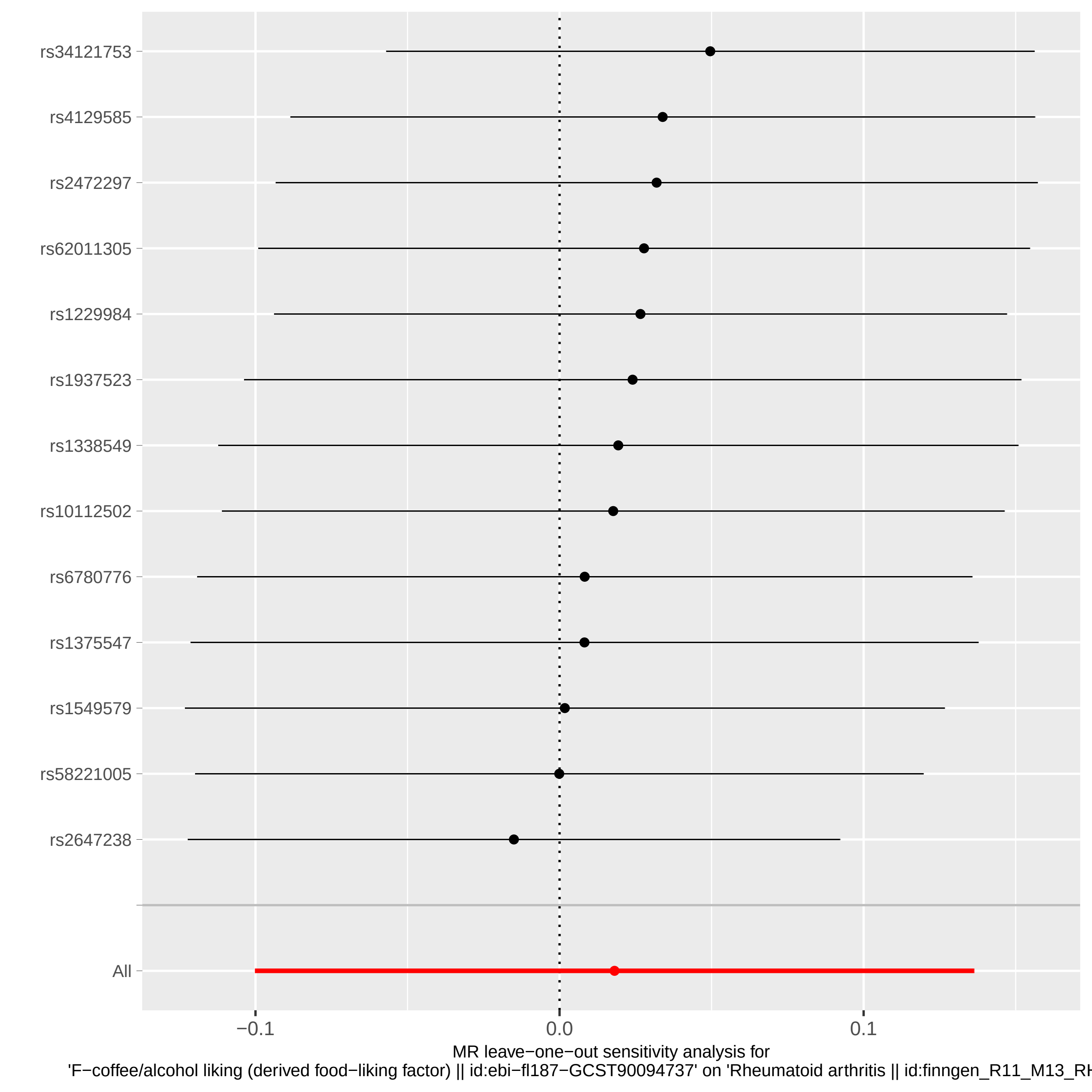
rs34121753
rs4129585
rs2472297
rs62011305
rs1229984
rs1937523
rs1338549
rs10112502
rs6780776
rs1375547
rs1549579
rs58221005
rs2647238
All
−0.1
0.0
0.1
MR leave−one−out sensitivity analysis for
'F−coffee/alcohol liking (derived food−liking factor) || id:ebi−fl187−GCST90094737' on 'Rheumatoid arthritis || id:finngen_R11_M13_RH

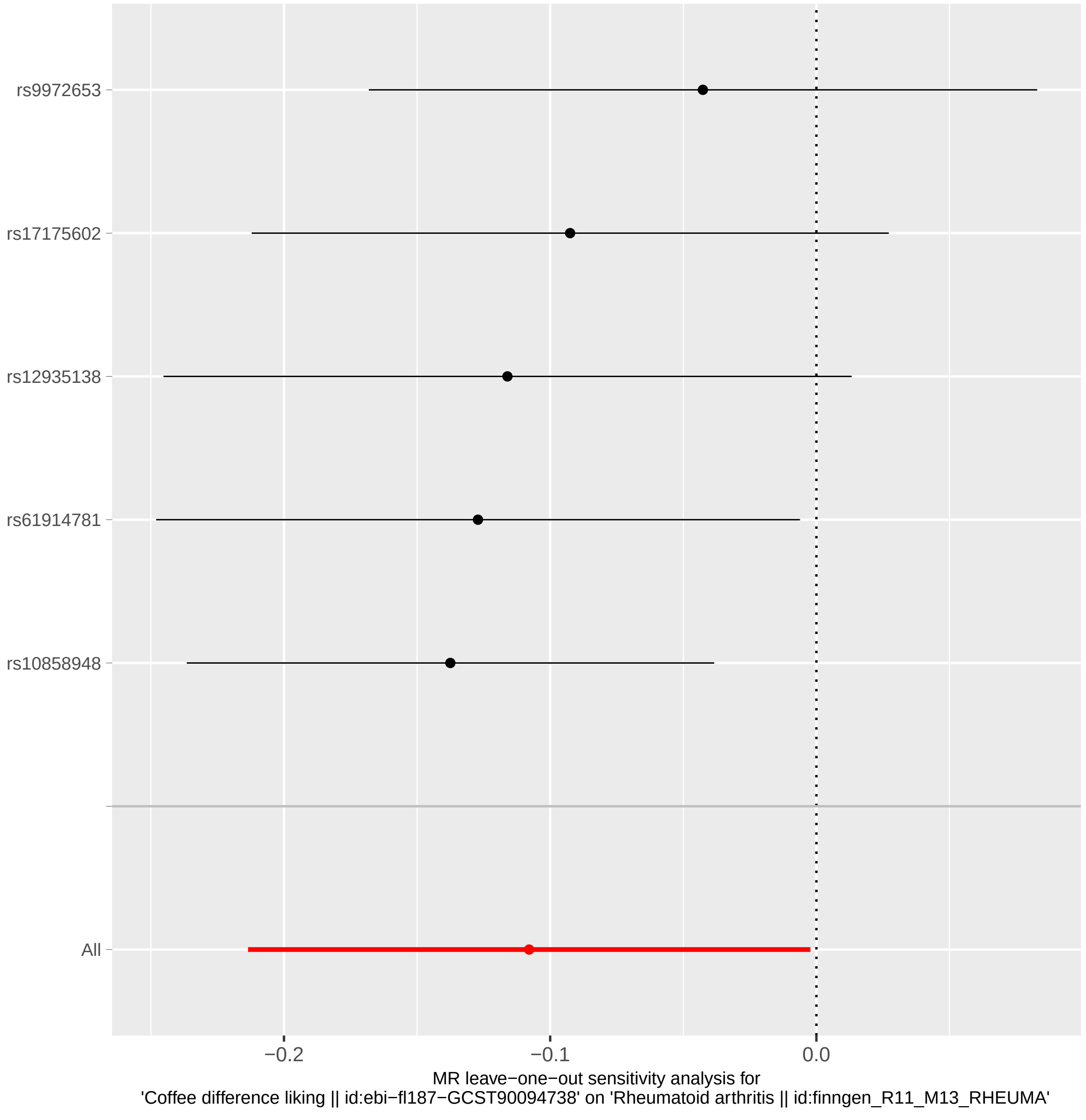
rs9972653
rs17175602
rs12935138
rs61914781
rs10858948
All
−0.2
−0.1
0.0
MR leave−one−out sensitivity analysis for
'Coffee difference liking || id:ebi−fl187−GCST90094738' on 'Rheumatoid arthritis || id:finngen_R11_M13_RHEUMA'

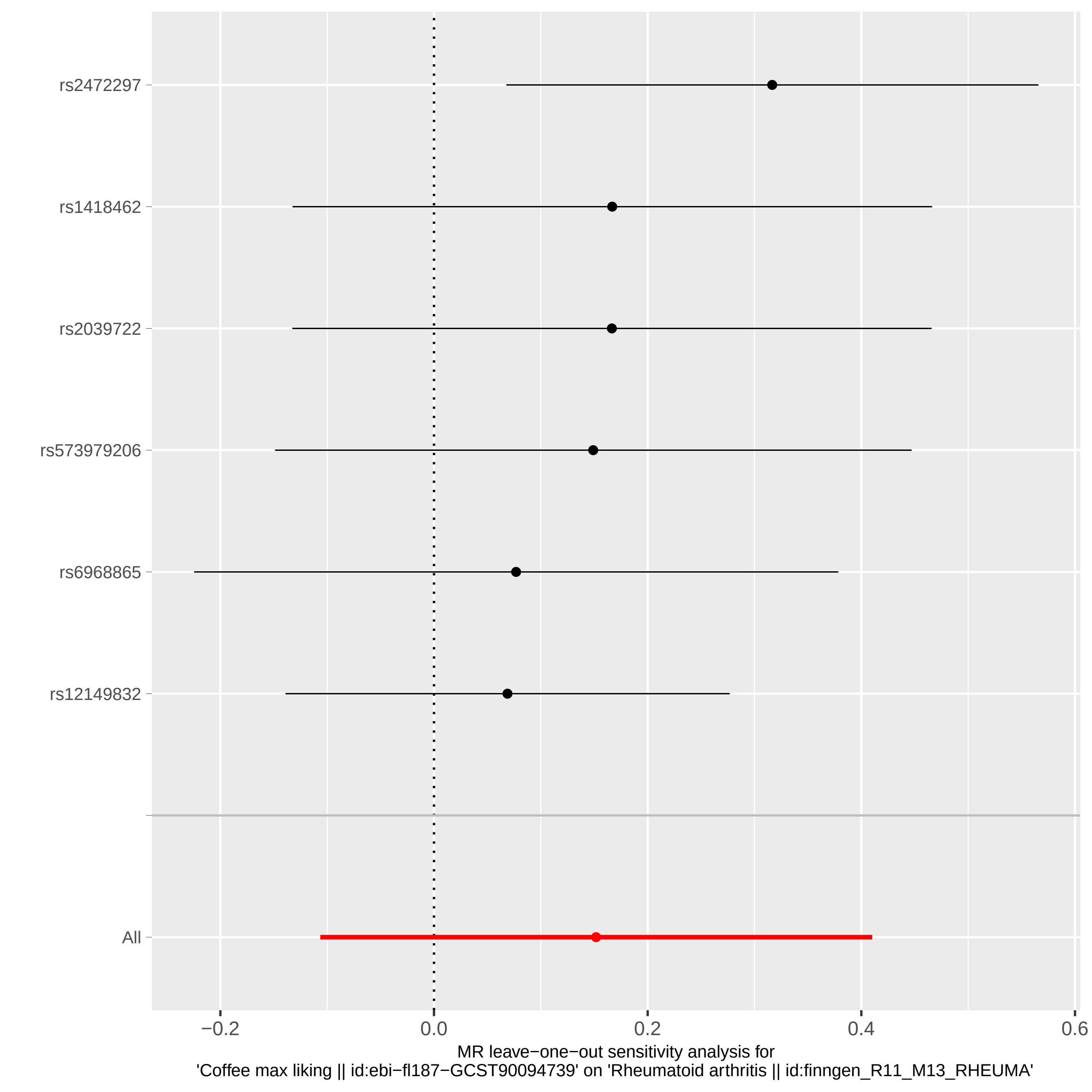
rs2472297
rs1418462
rs2039722
rs573979206
rs6968865
rs12149832
All
−0.2
0.0
0.2
0.4
0.6
MR leave−one−out sensitivity analysis for
'Coffee max liking || id:ebi−fl187−GCST90094739' on 'Rheumatoid arthritis || id:finngen_R11_M13_RHEUMA'

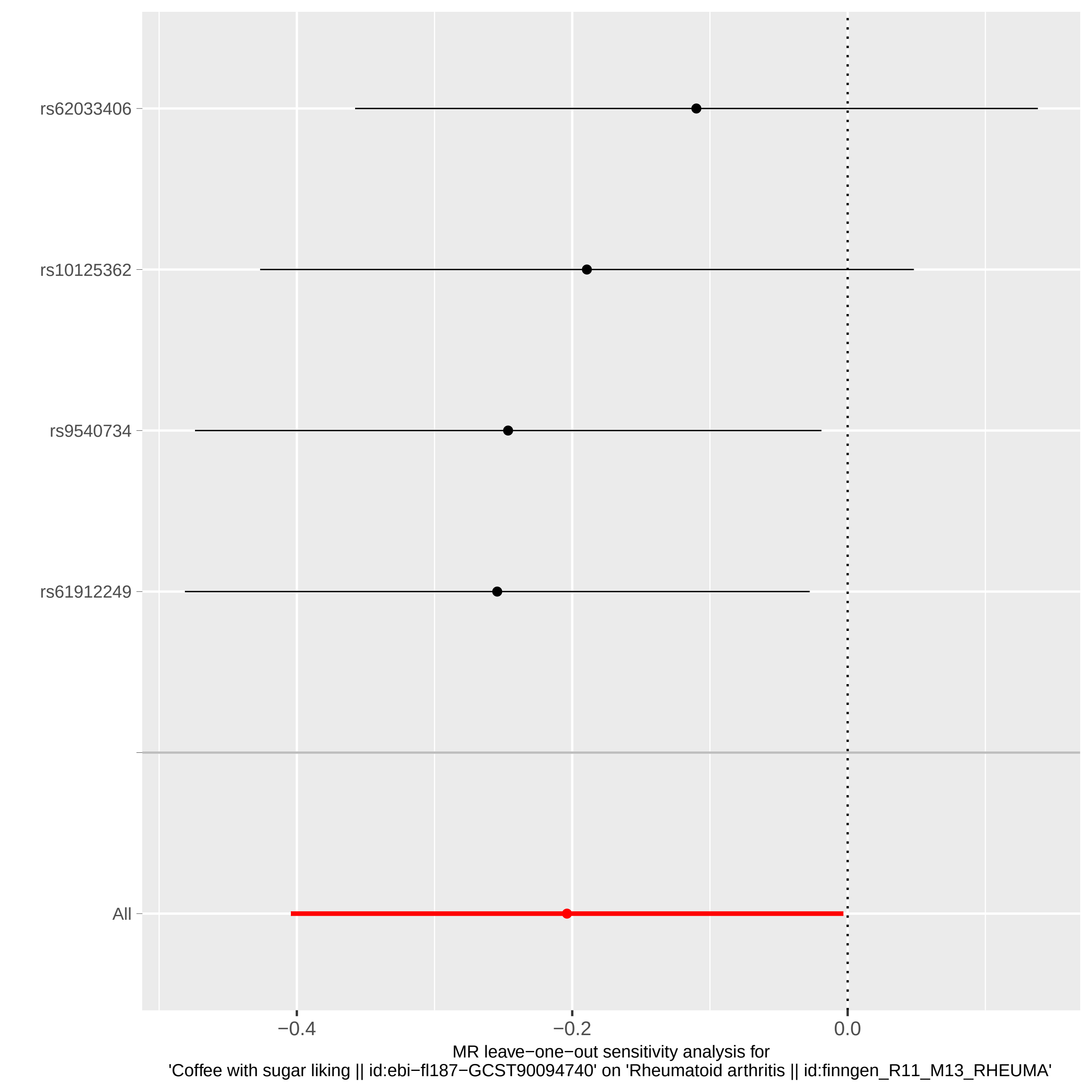

rs62033406
rs10125362
rs9540734
rs61912249
All
−0.4
−0.2
0.0
MR leave−one−out sensitivity analysis for
'Coffee with sugar liking || id:ebi−fl187−GCST90094740' on 'Rheumatoid arthritis || id:finngen_R11_M13_RHEUMA'

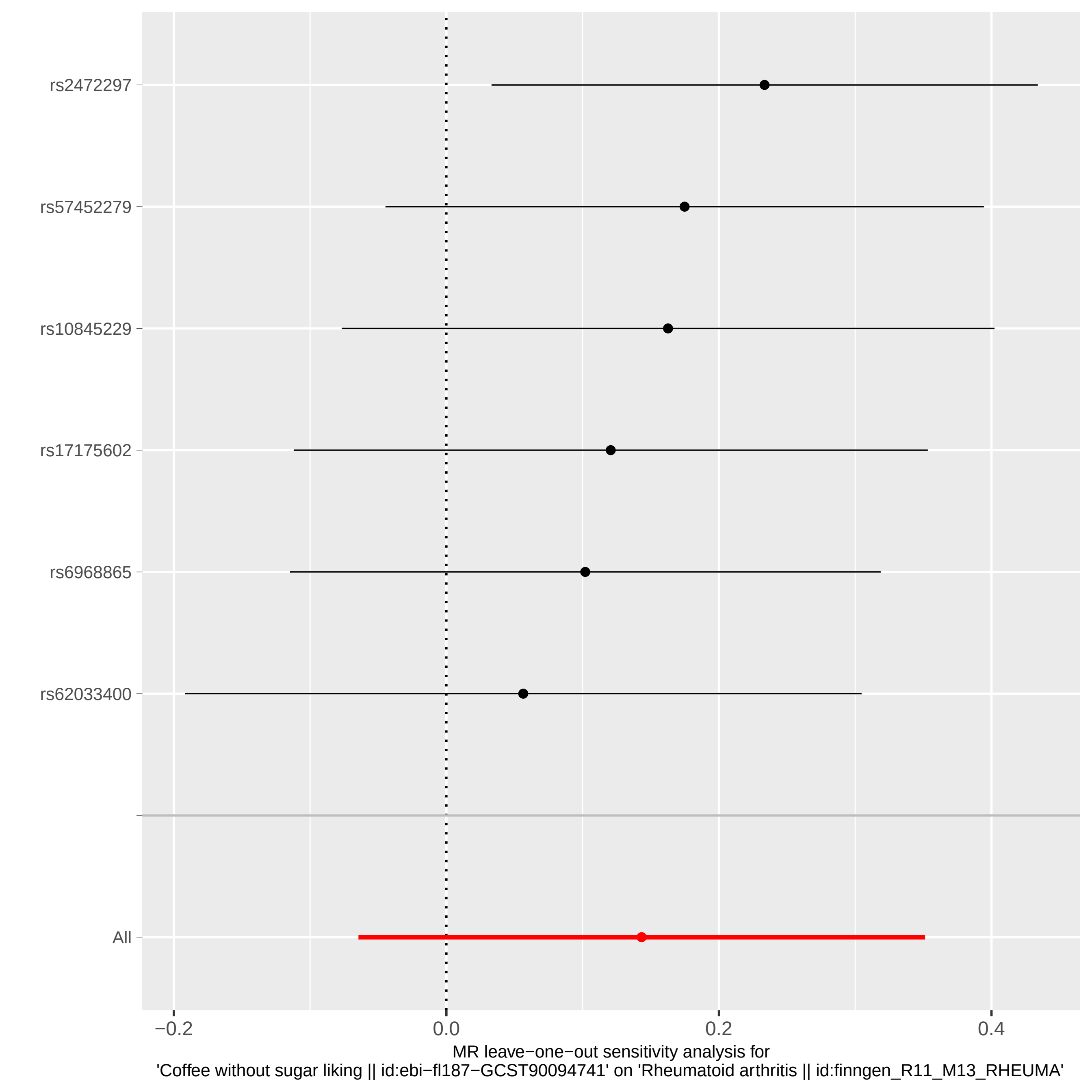
rs2472297
rs57452279
rs10845229
rs17175602
rs6968865
rs62033400
All
−0.2
0.0
0.2
0.4
MR leave−one−out sensitivity analysis for
'Coffee without sugar liking || id:ebi−fl187−GCST90094741' on 'Rheumatoid arthritis || id:finngen_R11_M13_RHEUMA'

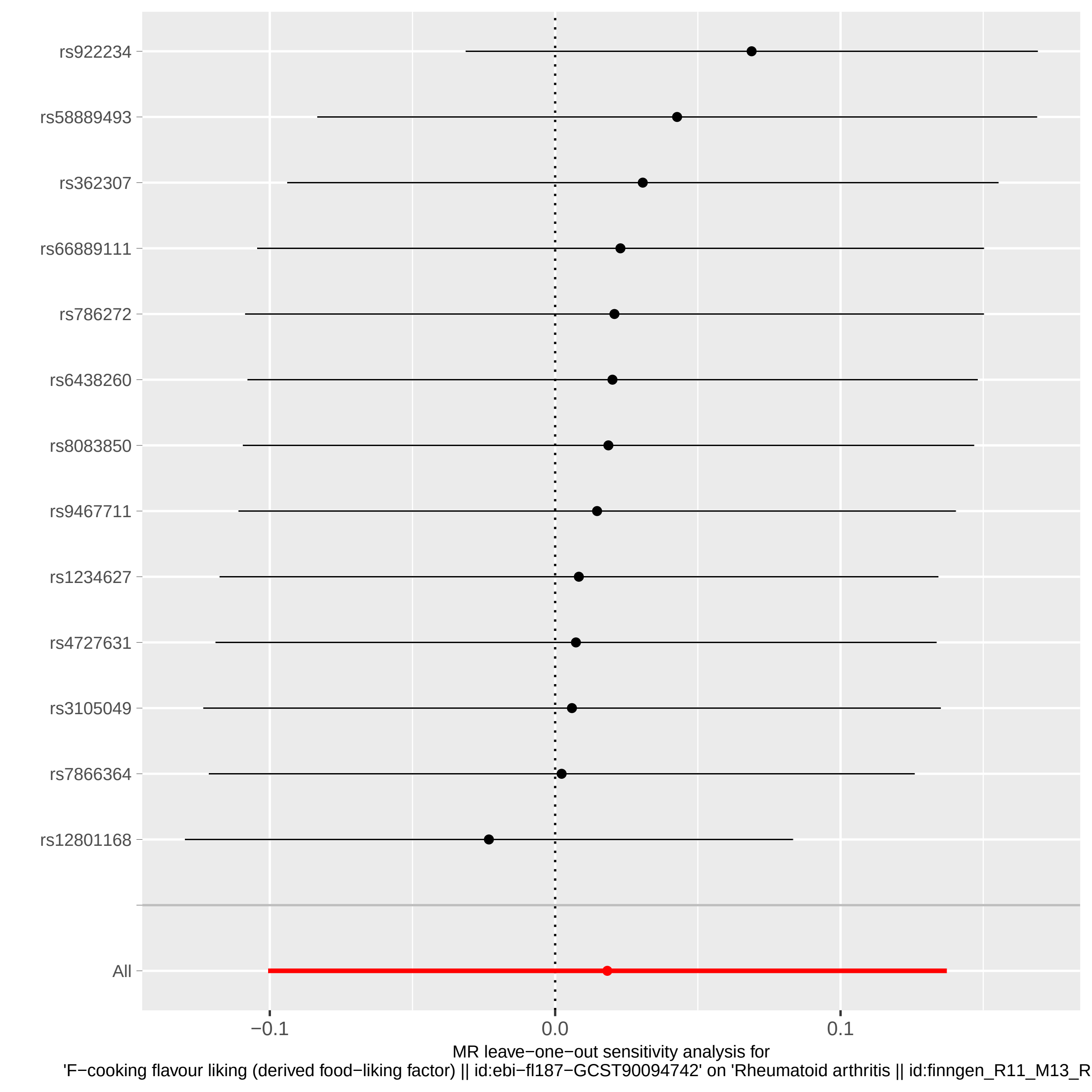
rs922234
rs58889493
rs362307
rs66889111
rs786272
rs6438260
rs8083850
rs9467711
rs1234627
rs4727631
rs3105049
rs7866364
rs12801168
All
−0.1
0.0
0.1
MR leave−one−out sensitivity analysis for
'F−cooking flavour liking (derived food−liking factor) || id:ebi−fl187−GCST90094742' on 'Rheumatoid arthritis || id:finngen_R11_M13_R

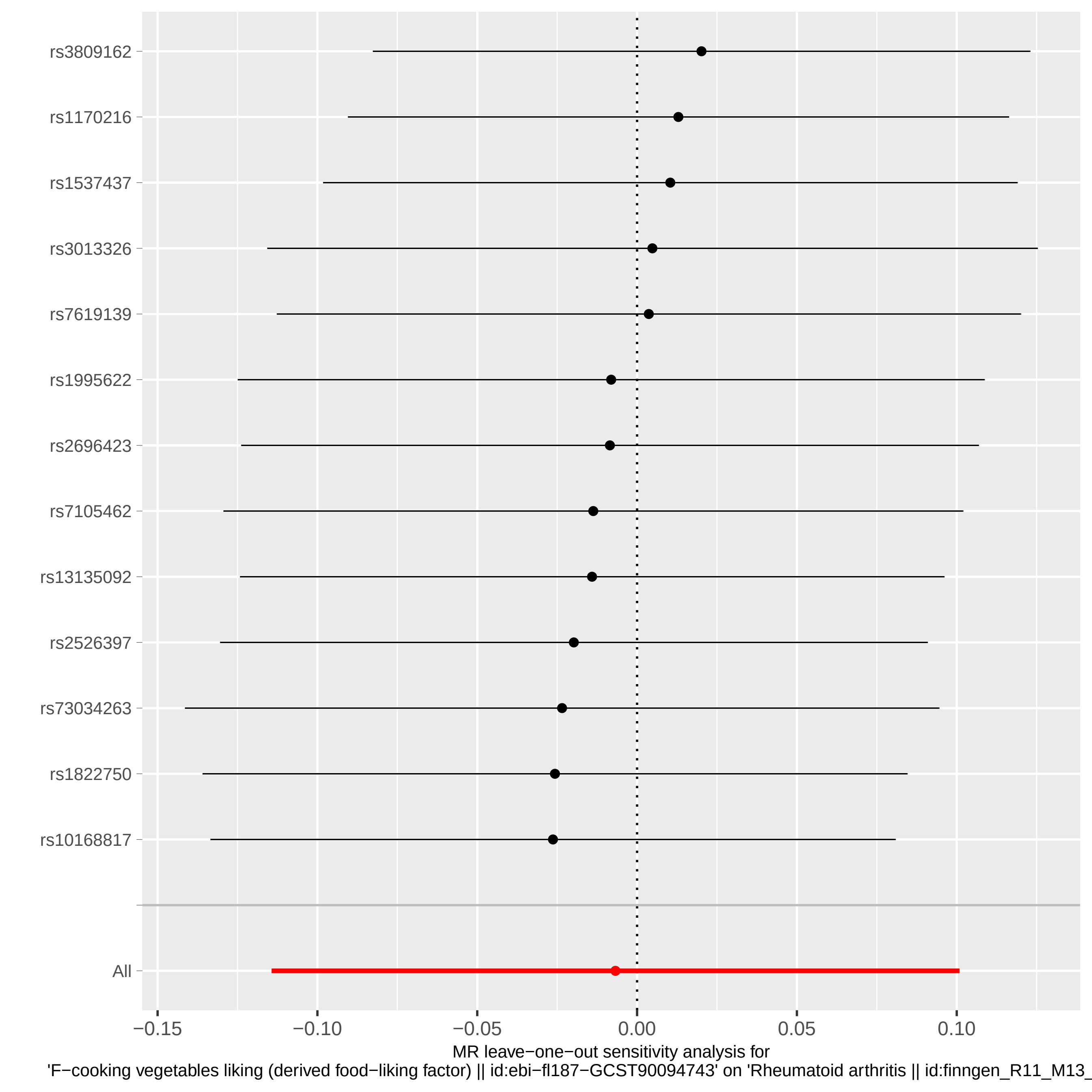

rs3809162
rs1170216
rs1537437
rs3013326
rs7619139
rs1995622
rs2696423
rs7105462
rs13135092
rs2526397
rs73034263
rs1822750
rs10168817
All
−0.15
−0.10
−0.05
0.00
0.05
0.10
MR leave−one−out sensitivity analysis for
'F−cooking vegetables liking (derived food−liking factor) || id:ebi−fl187−GCST90094743' on 'Rheumatoid arthritis || id:finngen_R11_M13_

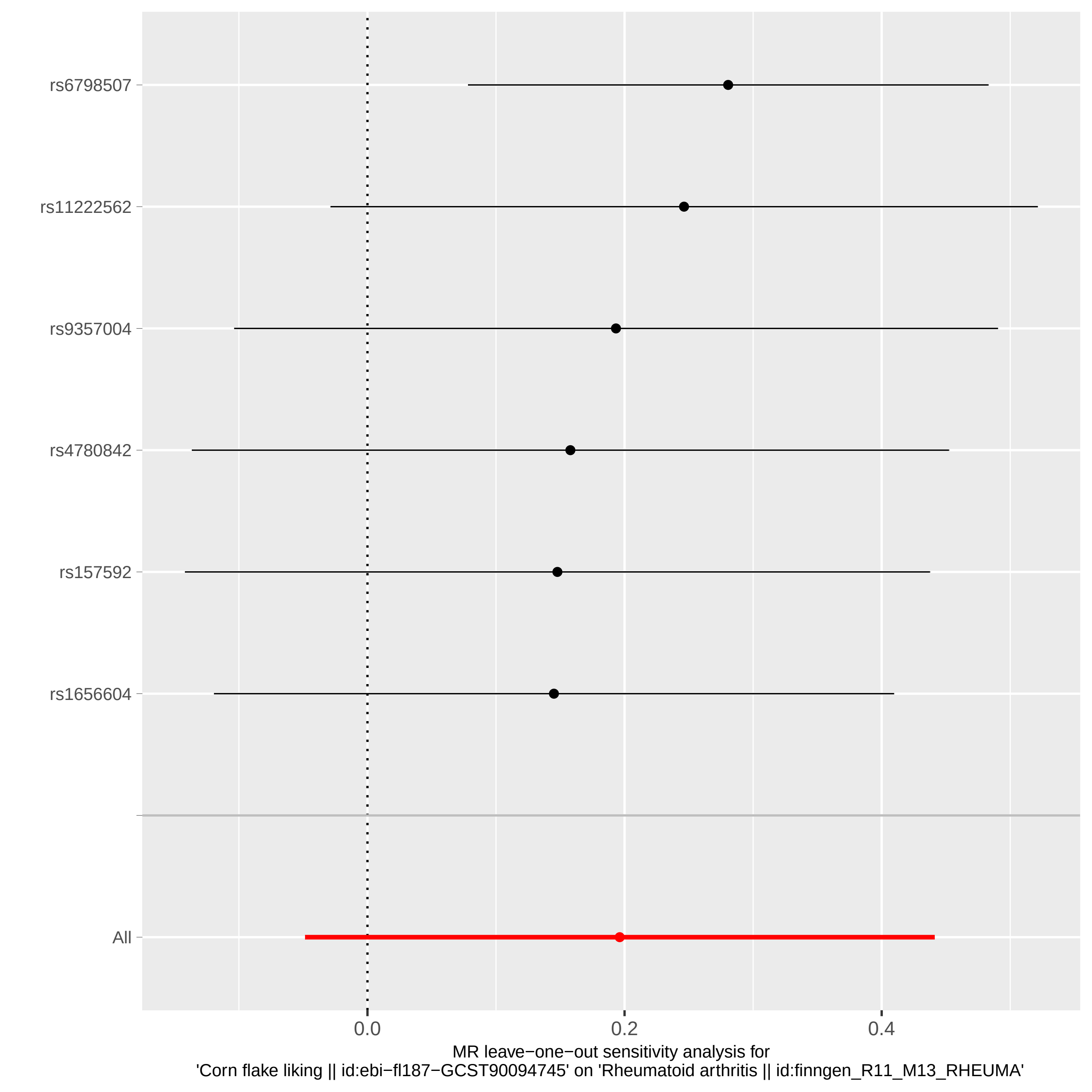
rs6798507
rs11222562
rs9357004
rs4780842
rs157592
rs1656604
All
0.0
0.2
0.4
MR leave−one−out sensitivity analysis for
'Corn flake liking || id:ebi−fl187−GCST90094745' on 'Rheumatoid arthritis || id:finngen_R11_M13_RHEUMA'

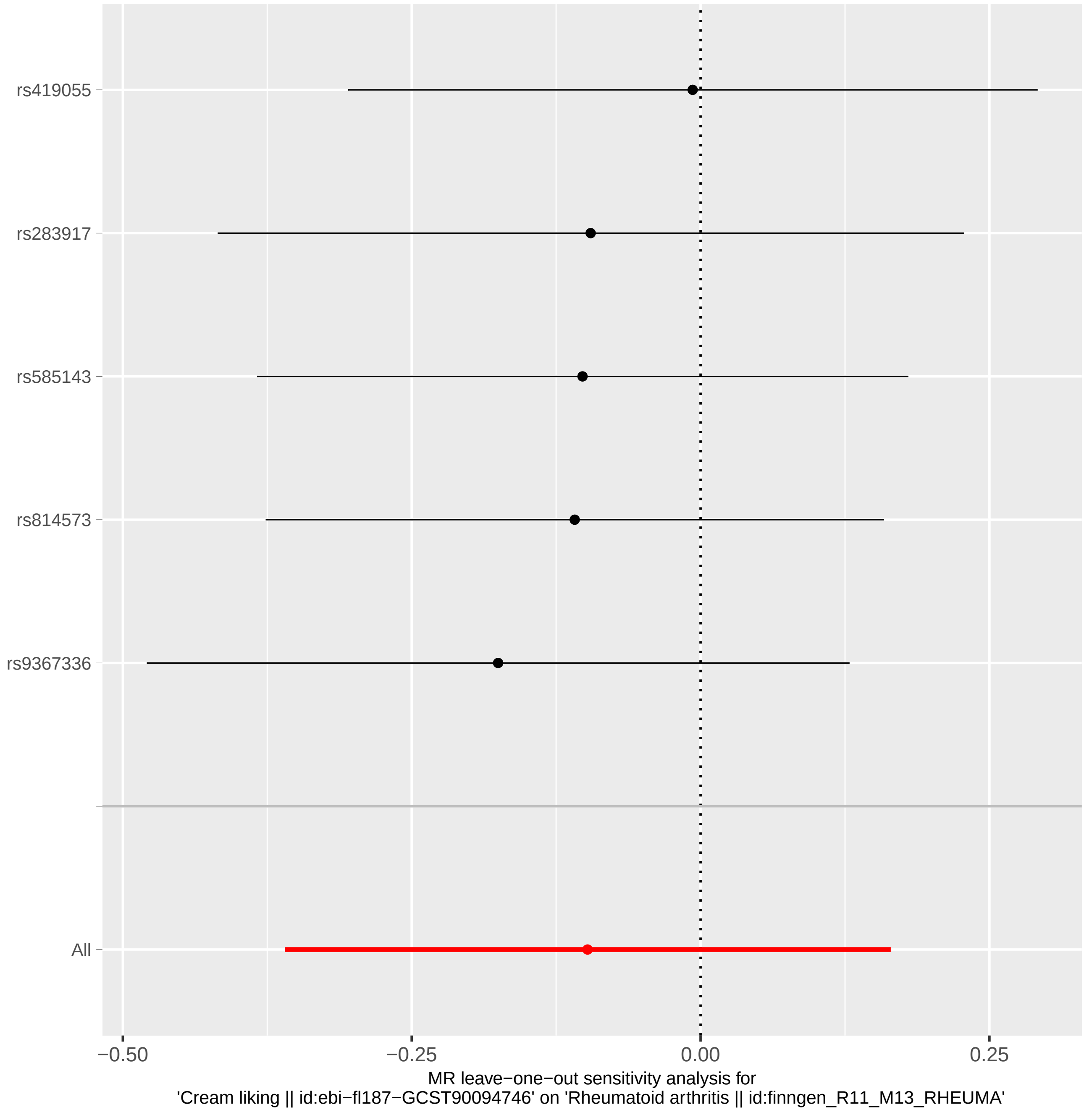
rs419055
rs283917
rs585143
rs814573
rs9367336
All
−0.50
−0.25
0.00
0.25
MR leave−one−out sensitivity analysis for
'Cream liking || id:ebi−fl187−GCST90094746' on 'Rheumatoid arthritis || id:finngen_R11_M13_RHEUMA'

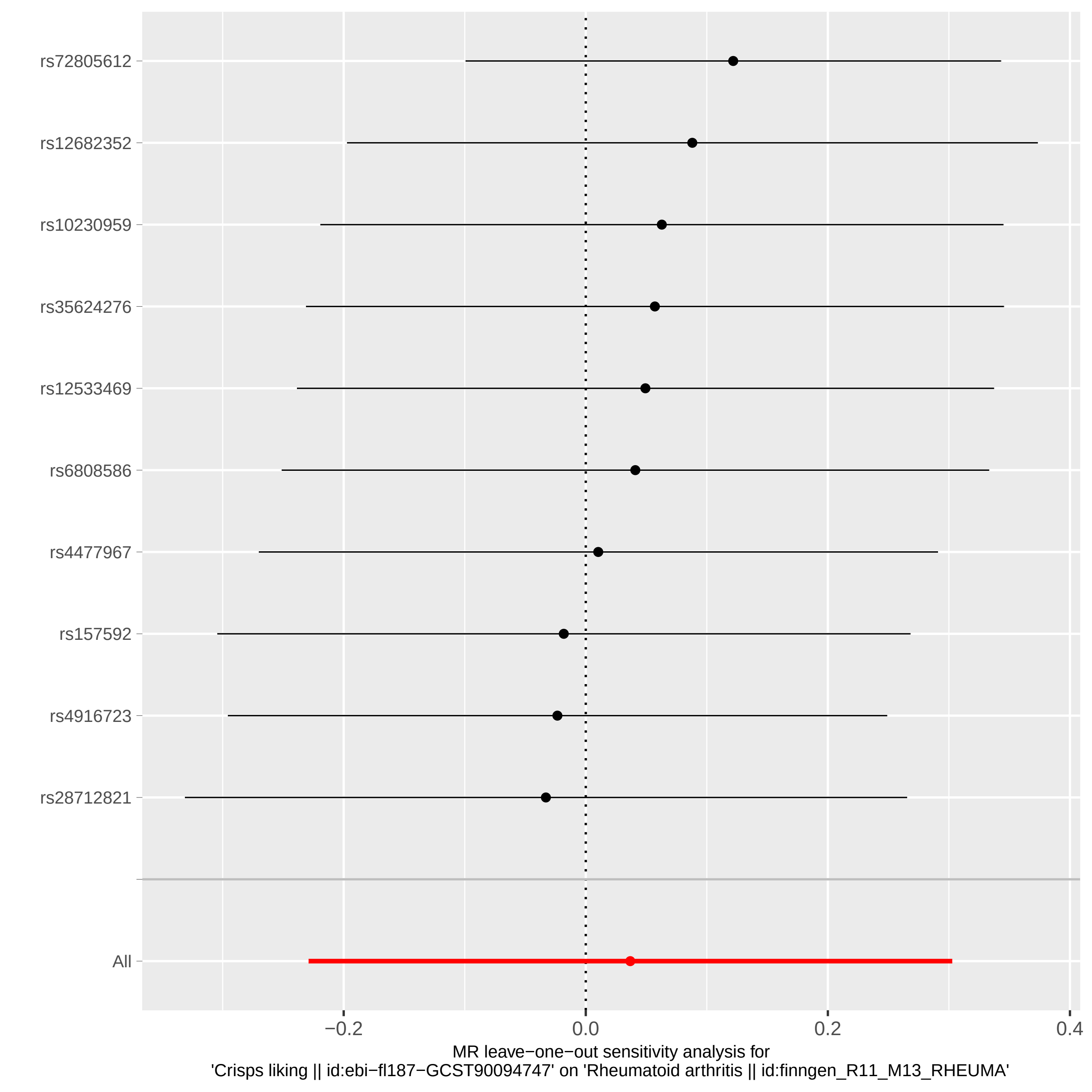

rs72805612
rs12682352
rs10230959
rs35624276
rs12533469
rs6808586
rs4477967
rs157592
rs4916723
rs28712821
All
−0.2
0.0
0.2
0.4
MR leave−one−out sensitivity analysis for
'Crisps liking || id:ebi−fl187−GCST90094747' on 'Rheumatoid arthritis || id:finngen_R11_M13_RHEUMA'

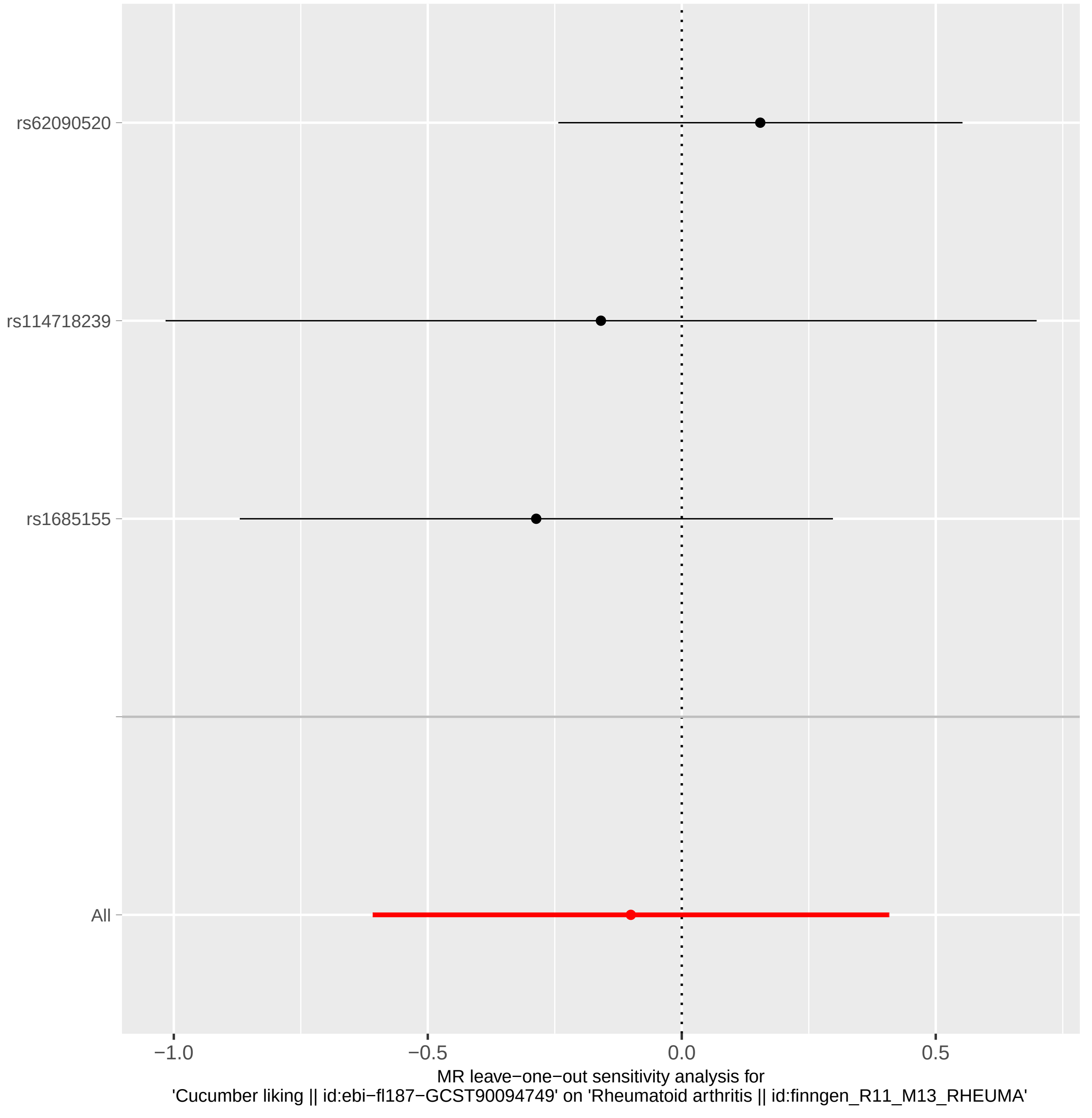
rs62090520
rs114718239
rs1685155
All
−1.0
−0.5
0.0
0.5
MR leave−one−out sensitivity analysis for
'Cucumber liking || id:ebi−fl187−GCST90094749' on 'Rheumatoid arthritis || id:finngen_R11_M13_RHEUMA'

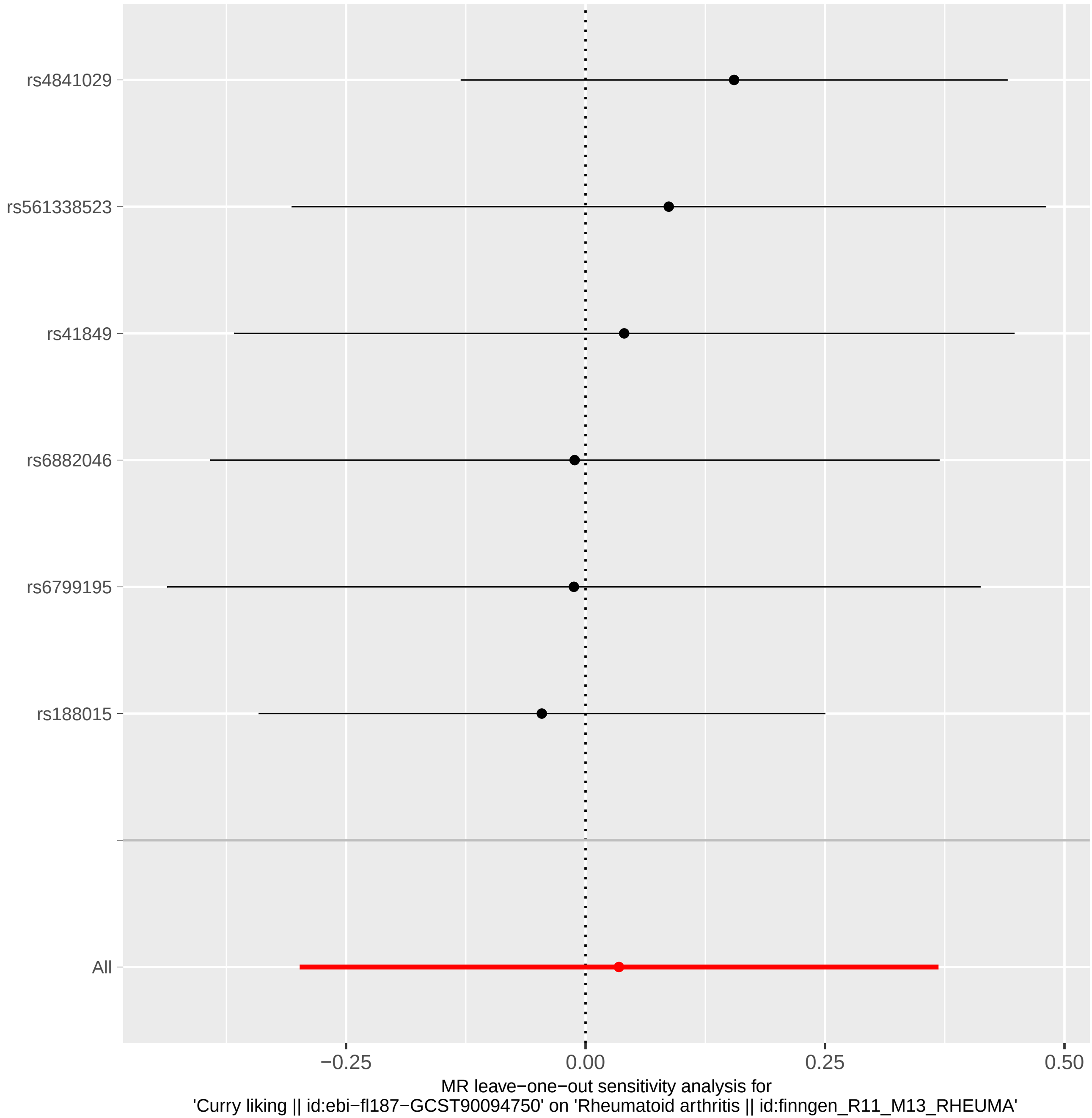
rs4841029
rs561338523
rs41849
rs6882046
rs6799195
rs188015
All
−0.25
0.00
0.25
0.50
MR leave−one−out sensitivity analysis for
'Curry liking || id:ebi−fl187−GCST90094750' on 'Rheumatoid arthritis || id:finngen_R11_M13_RHEUMA'

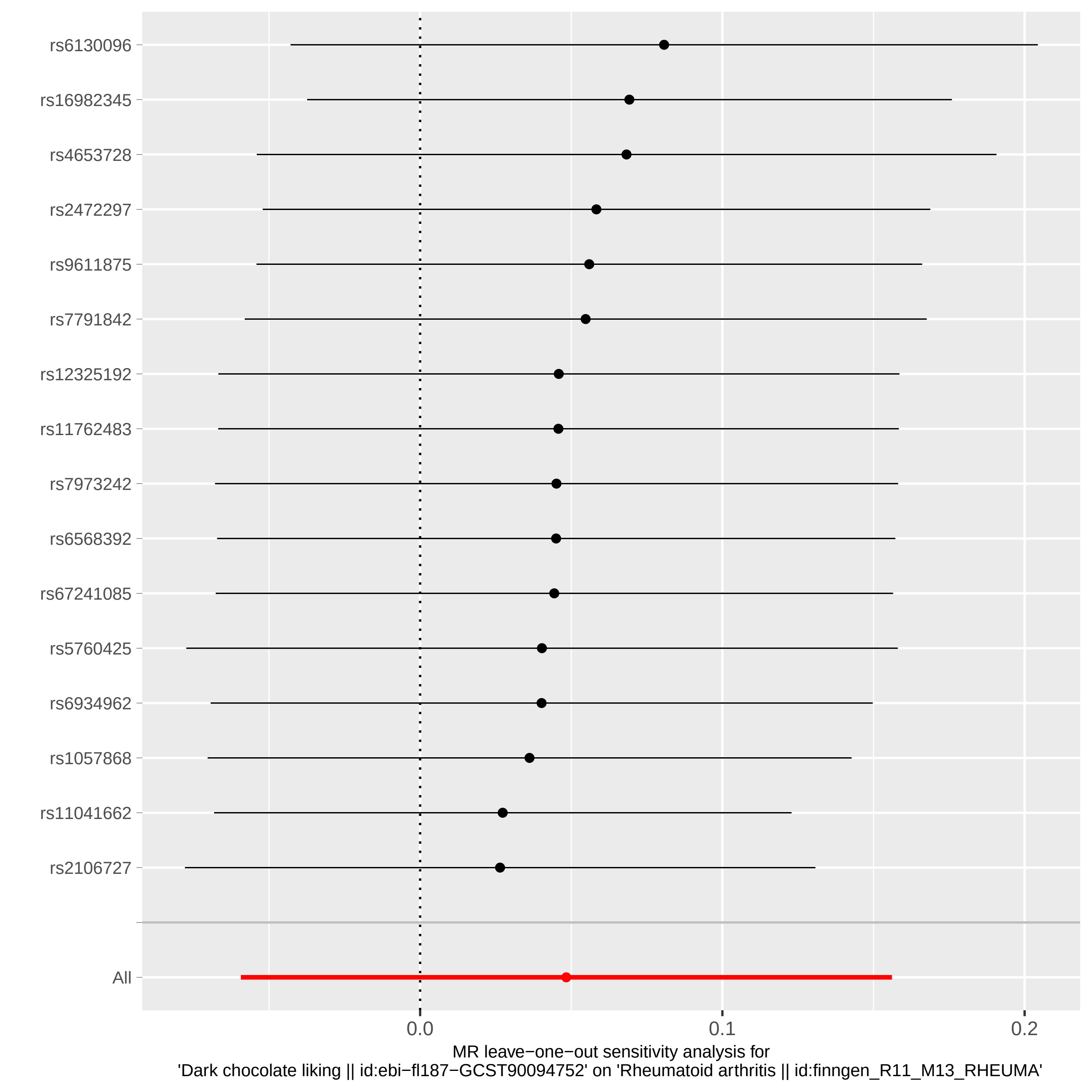

rs6130096
rs16982345
rs4653728
rs2472297
rs9611875
rs7791842
rs12325192
rs11762483
rs7973242
rs6568392
rs67241085
rs5760425
rs6934962
rs1057868
rs11041662
rs2106727
All
0.0
0.1
0.2
MR leave−one−out sensitivity analysis for
'Dark chocolate liking || id:ebi−fl187−GCST90094752' on 'Rheumatoid arthritis || id:finngen_R11_M13_RHEUMA'

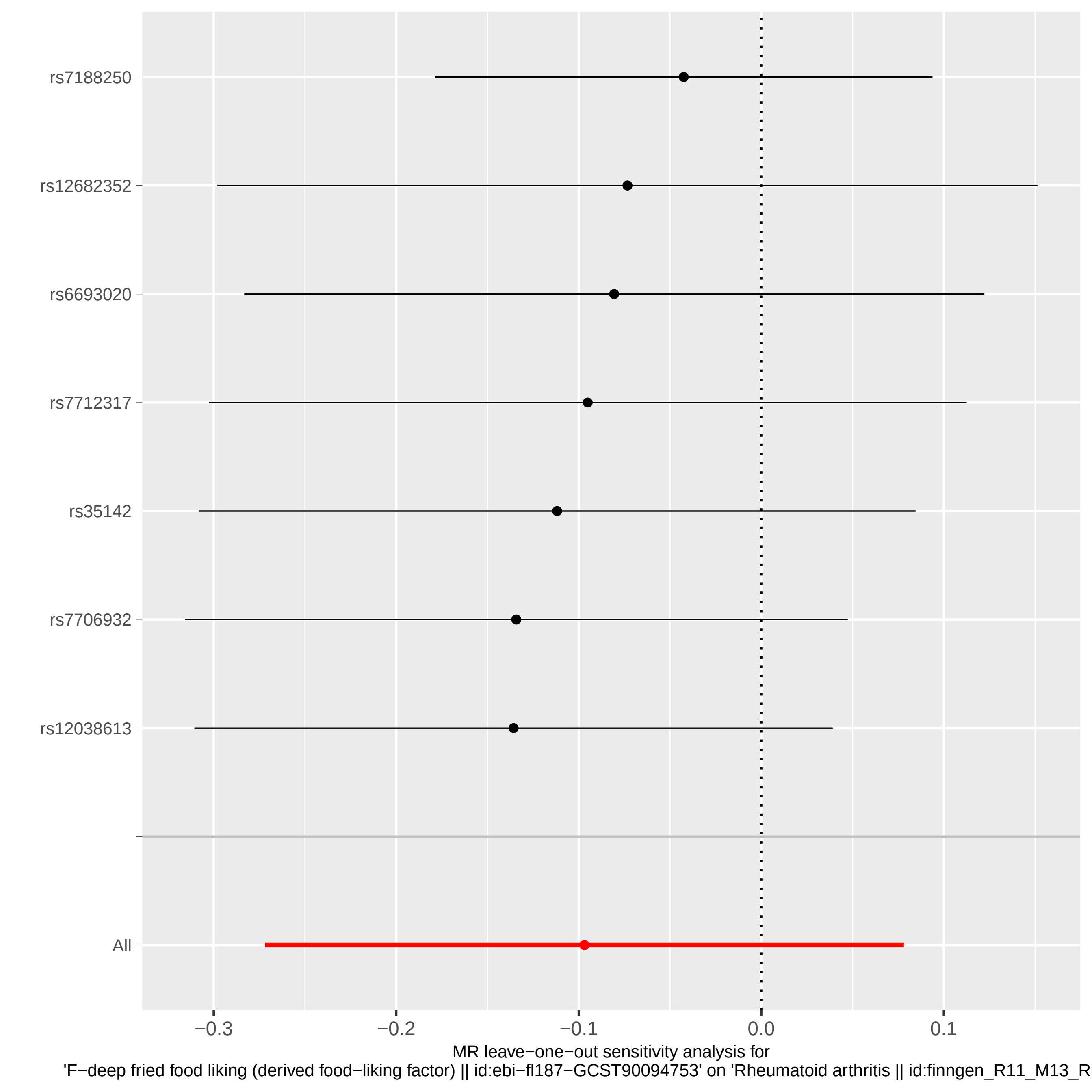
rs7188250
rs12682352
rs6693020
rs7712317
rs35142
rs7706932
rs12038613
All
−0.3
−0.2
−0.1
0.0
0.1
MR leave−one−out sensitivity analysis for
'F−deep fried food liking (derived food−liking factor) || id:ebi−fl187−GCST90094753' on 'Rheumatoid arthritis || id:finngen_R11_M13_R

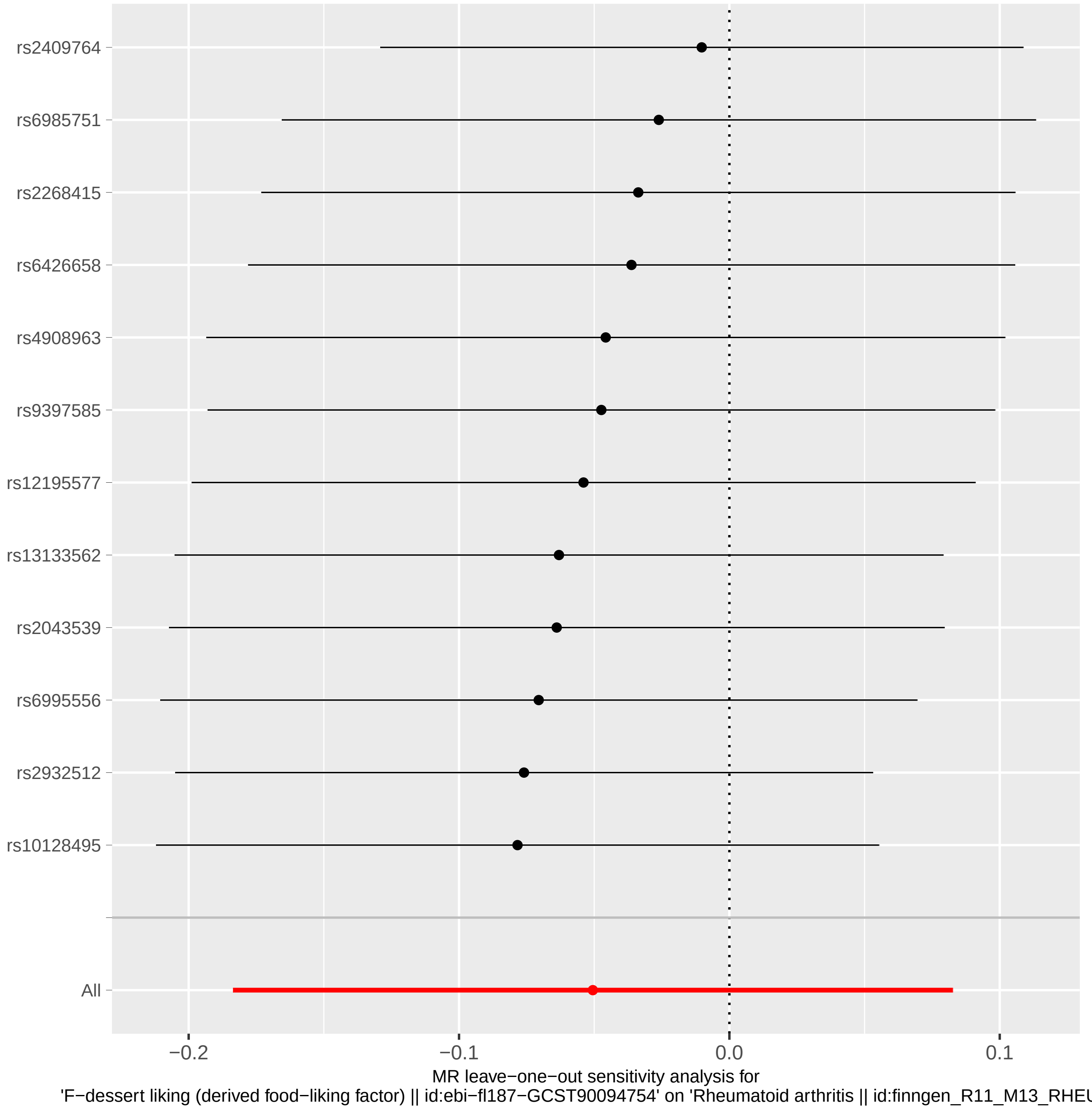
rs2409764
rs6985751
rs2268415
rs6426658
rs4908963
rs9397585
rs12195577
rs13133562
rs2043539
rs6995556
rs2932512
rs10128495
All
−0.2
−0.1
0.0
0.1
MR leave−one−out sensitivity analysis for
'F−dessert liking (derived food−liking factor) || id:ebi−fl187−GCST90094754' on 'Rheumatoid arthritis || id:finngen_R11_M13_RHEU

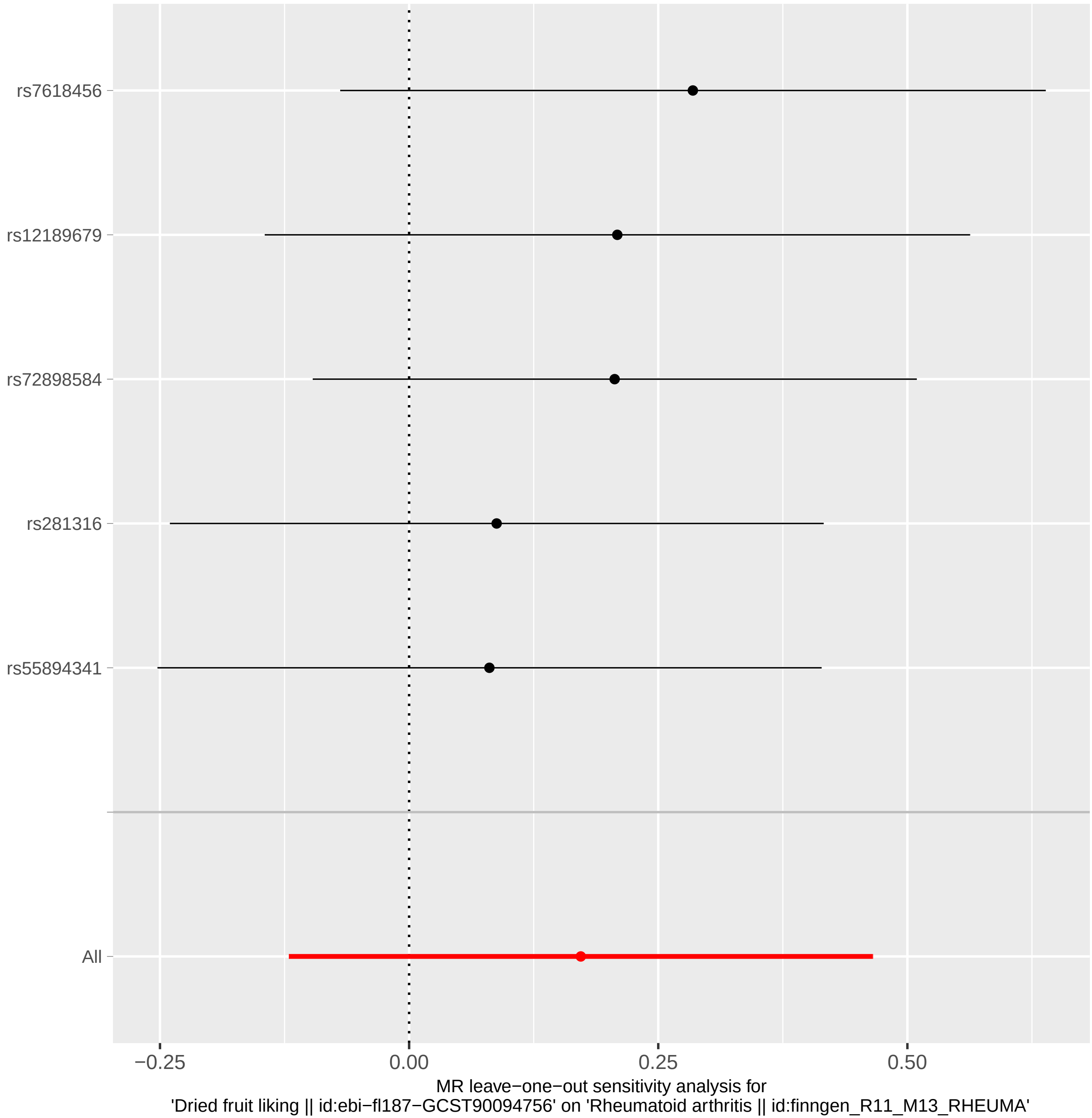

rs7618456
rs12189679
rs72898584
rs281316
rs55894341
All
−0.25
0.00
0.25
0.50
MR leave−one−out sensitivity analysis for
'Dried fruit liking || id:ebi−fl187−GCST90094756' on 'Rheumatoid arthritis || id:finngen_R11_M13_RHEUMA'

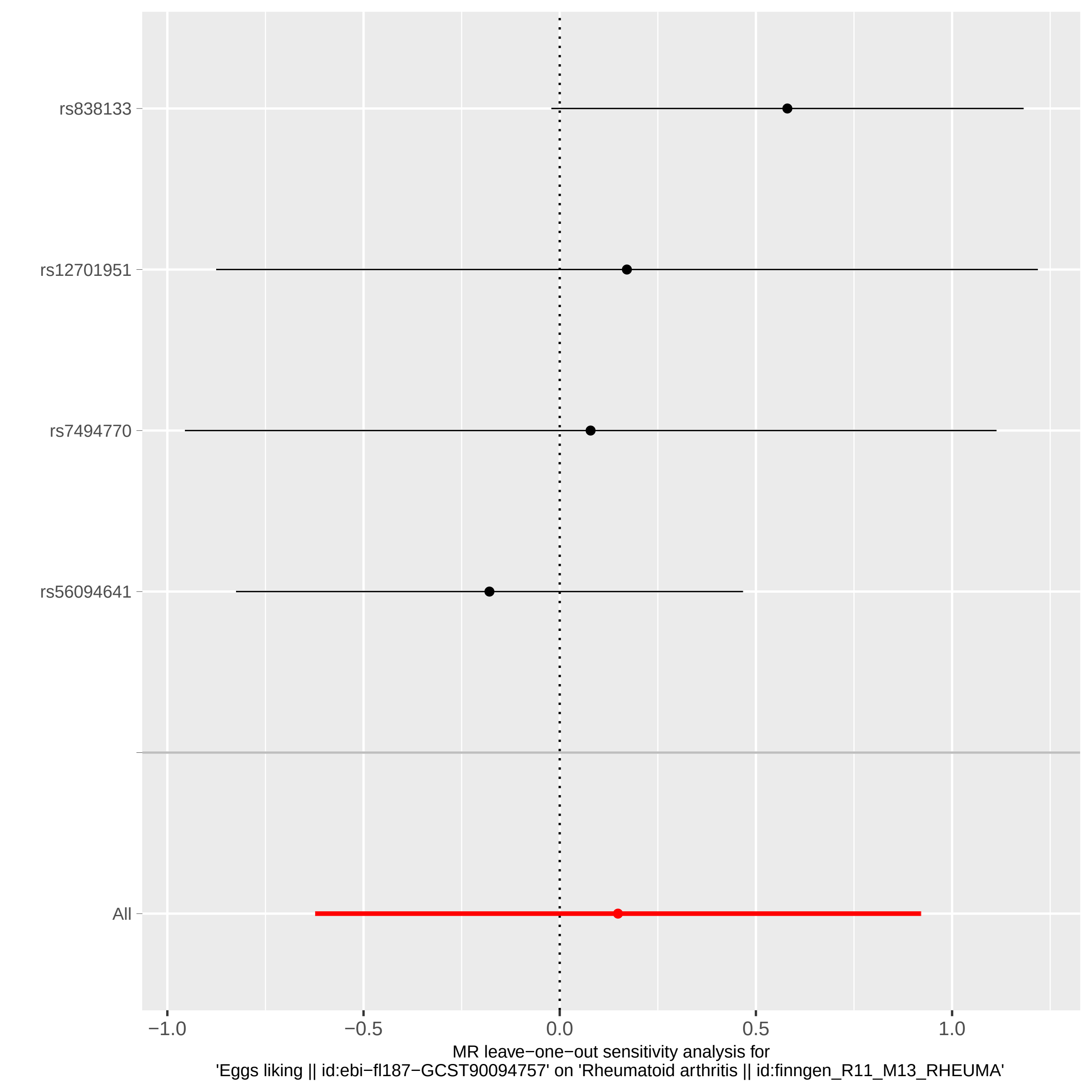
rs838133
rs12701951
rs7494770
rs56094641
All
−1.0
−0.5
0.0
0.5
1.0
MR leave−one−out sensitivity analysis for
'Eggs liking || id:ebi−fl187−GCST90094757' on 'Rheumatoid arthritis || id:finngen_R11_M13_RHEUMA'

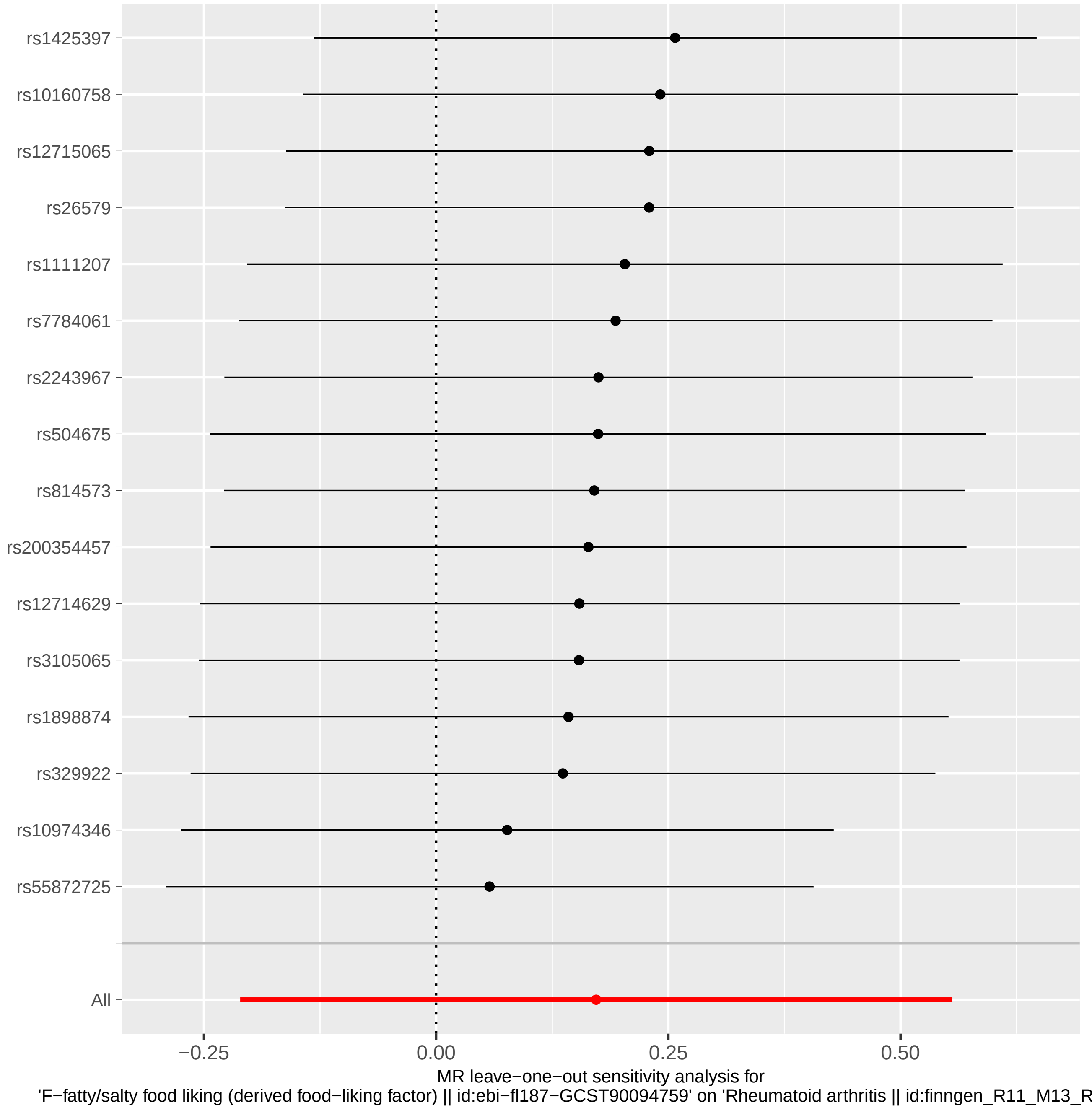
rs1425397
rs10160758
rs12715065
rs26579
rs1111207
rs7784061
rs2243967
rs504675
rs814573
rs200354457
rs12714629
rs3105065
rs1898874
rs329922
rs10974346
rs55872725
All
−0.25
0.00
0.25
0.50
MR leave−one−out sensitivity analysis for
'F−fatty/salty food liking (derived food−liking factor) || id:ebi−fl187−GCST90094759' on 'Rheumatoid arthritis || id:finngen_R11_M13_R

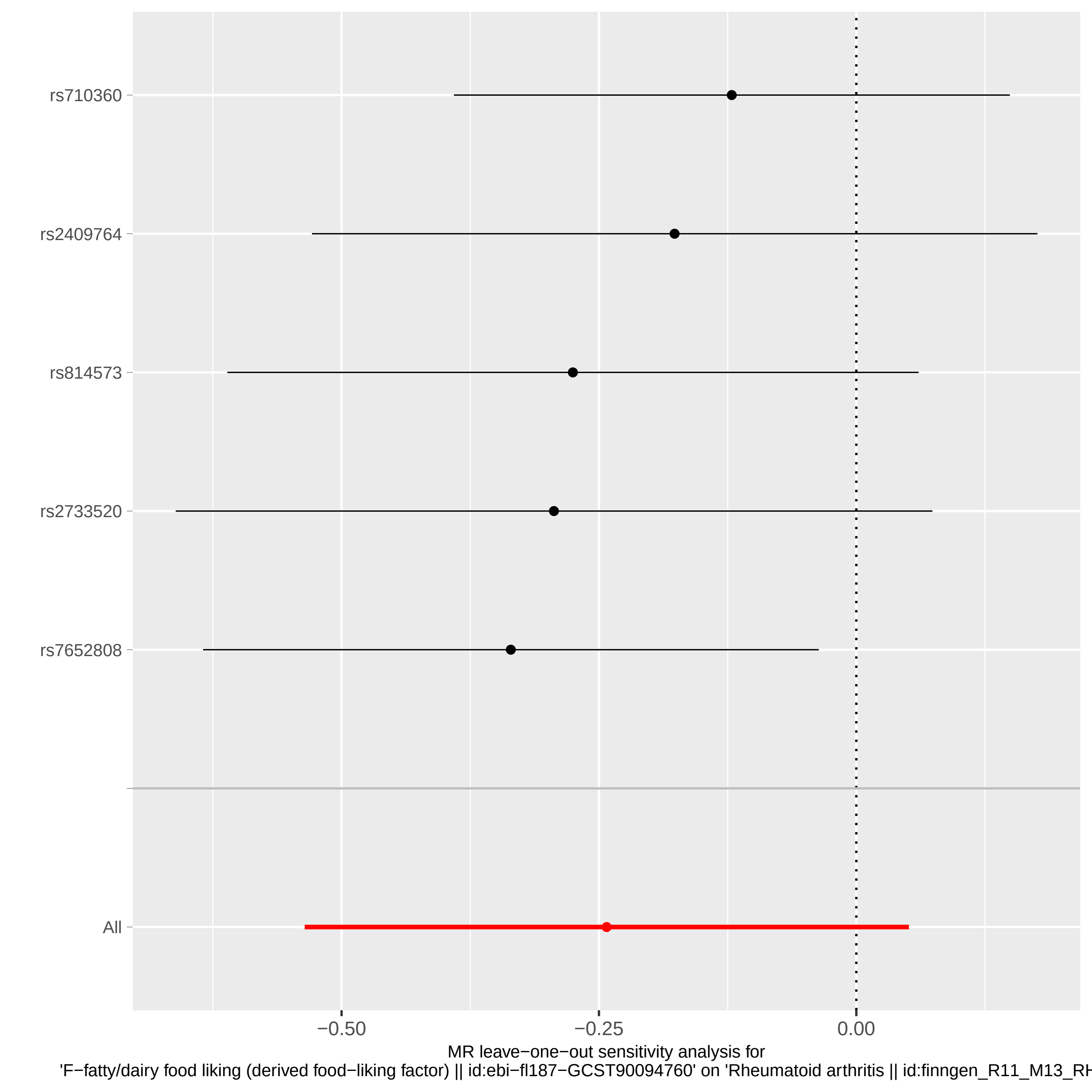

rs710360
rs2409764
rs814573
rs2733520
rs7652808
All
−0.50
−0.25
0.00
MR leave−one−out sensitivity analysis for
'F−fatty/dairy food liking (derived food−liking factor) || id:ebi−fl187−GCST90094760' on 'Rheumatoid arthritis || id:finngen_R11_M13_RH

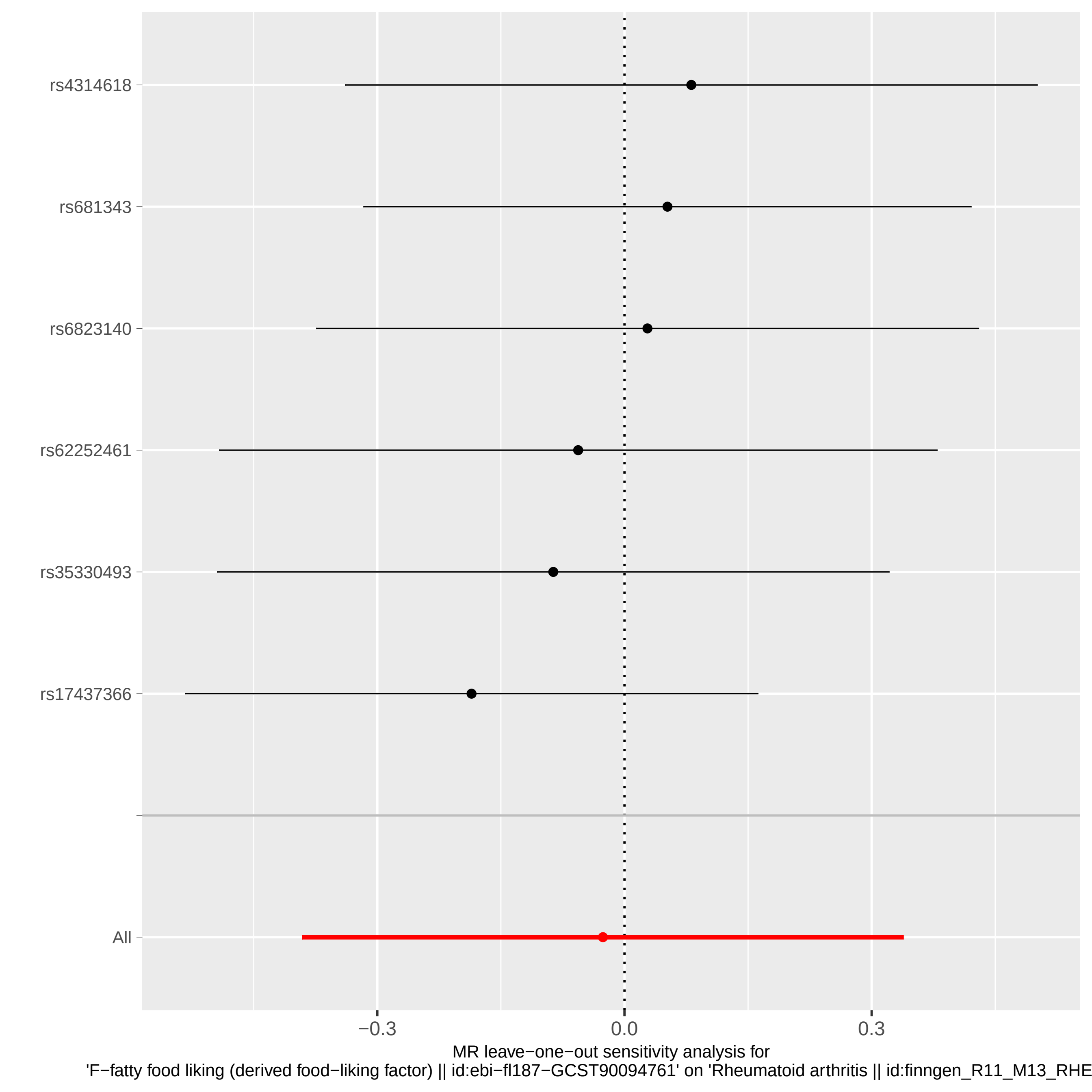

rs4314618
rs681343
rs6823140
rs62252461
rs35330493
rs17437366
All
−0.3
0.0
0.3
MR leave−one−out sensitivity analysis for
'F−fatty food liking (derived food−liking factor) || id:ebi−fl187−GCST90094761' on 'Rheumatoid arthritis || id:finngen_R11_M13_RHE

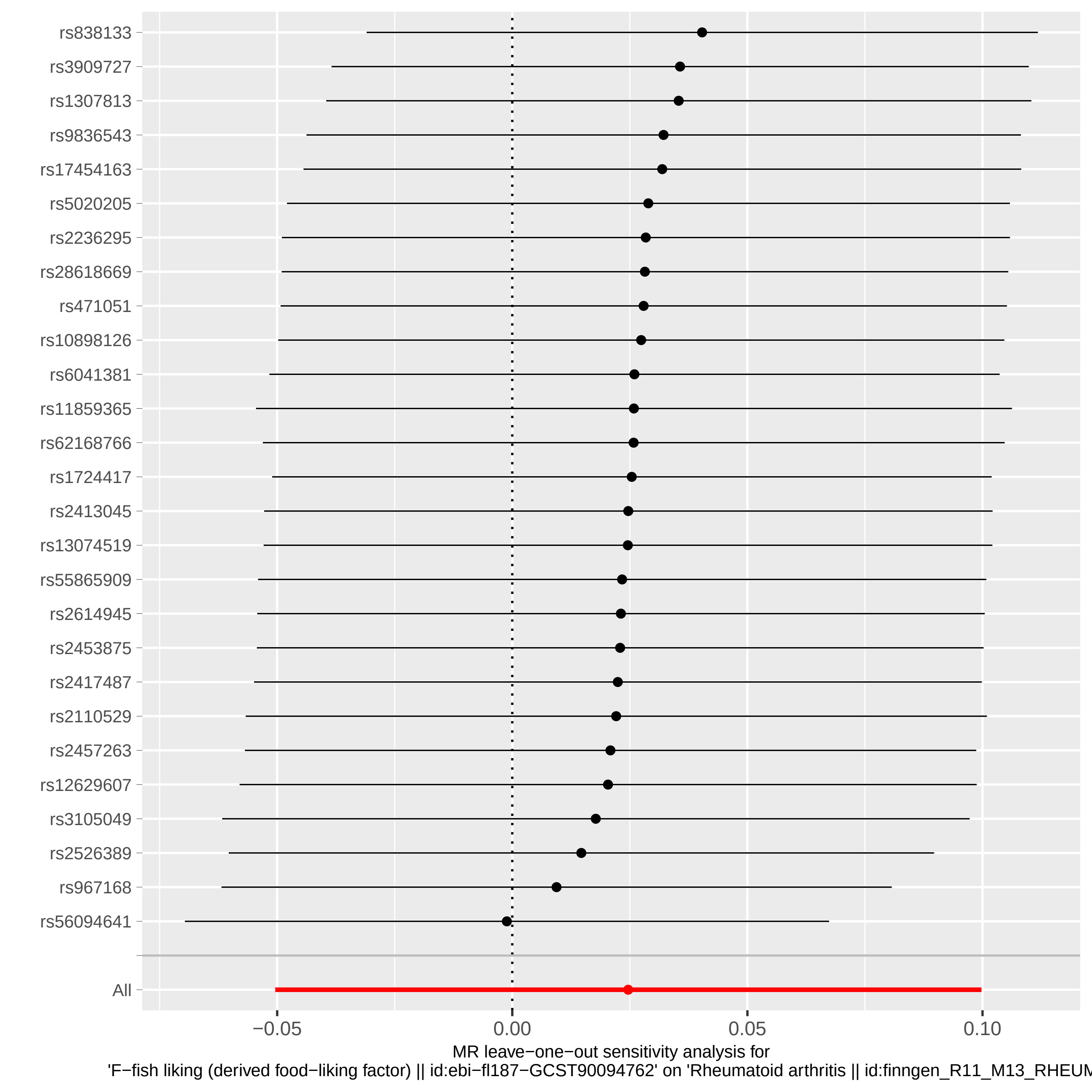
rs838133
rs3909727
rs1307813
rs9836543
rs17454163
rs5020205
rs2236295
rs28618669
rs471051
rs10898126
rs6041381
rs11859365
rs62168766
rs1724417
rs2413045
rs13074519
rs55865909
rs2614945
rs2453875
rs2417487
rs2110529
rs2457263
rs12629607
rs3105049
rs2526389
rs967168
rs56094641
All
−0.05
0.00
0.05
0.10
MR leave−one−out sensitivity analysis for
'F−fish liking (derived food−liking factor) || id:ebi−fl187−GCST90094762' on 'Rheumatoid arthritis || id:finngen_R11_M13_RHEUM

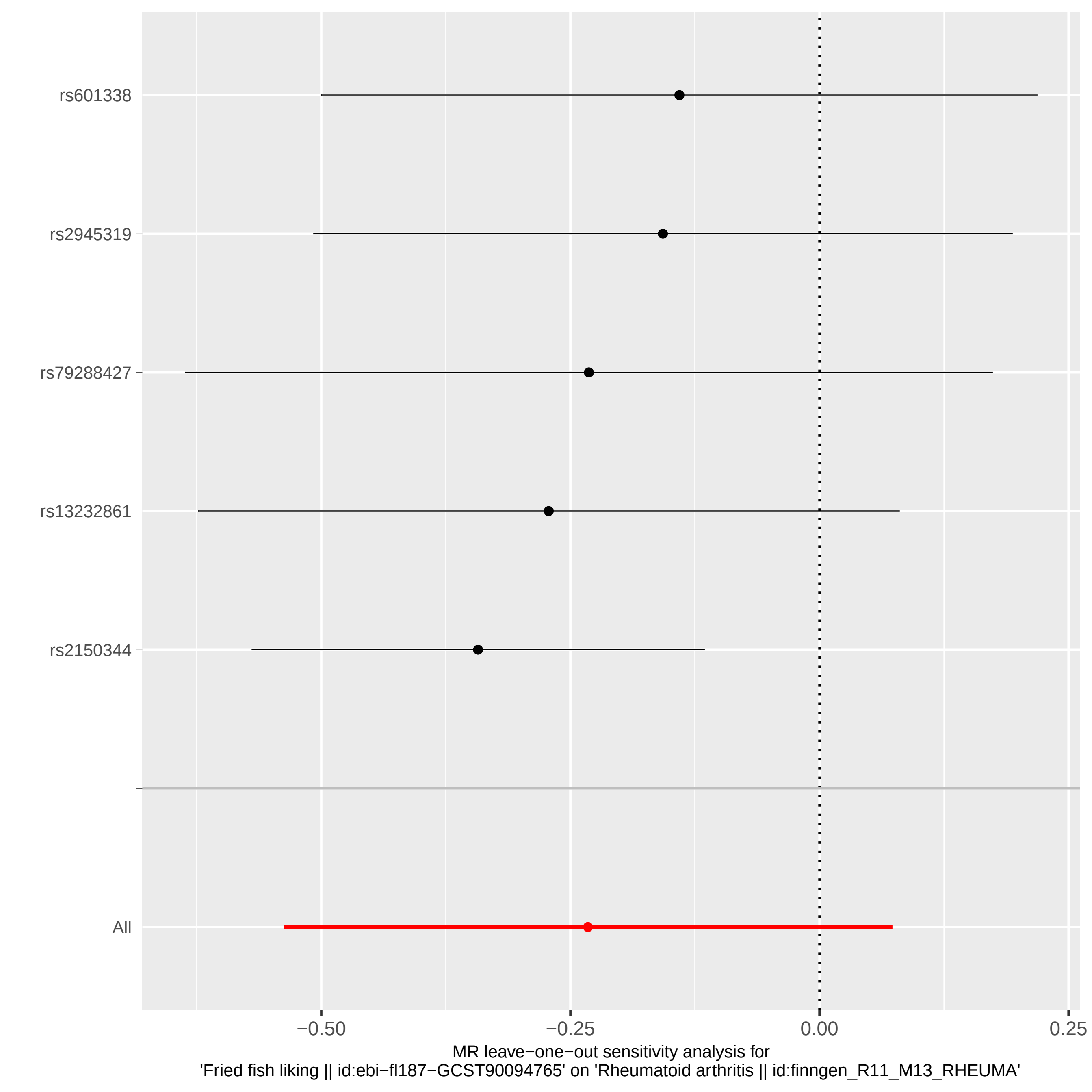
rs601338
rs2945319
rs79288427
rs13232861
rs2150344
All
−0.50
−0.25
0.00
0.25
MR leave−one−out sensitivity analysis for
'Fried fish liking || id:ebi−fl187−GCST90094765' on 'Rheumatoid arthritis || id:finngen_R11_M13_RHEUMA'

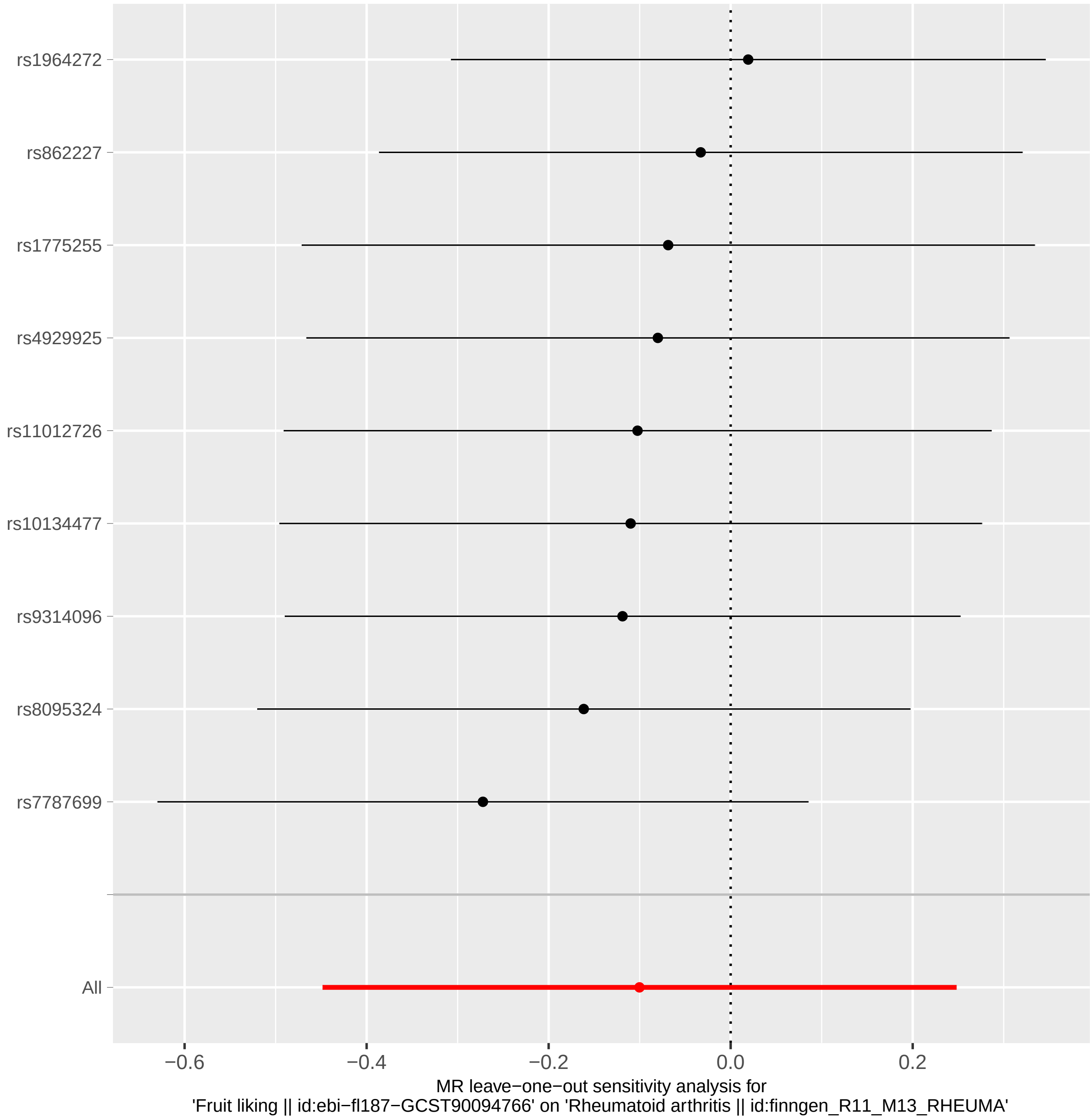

rs1964272
rs862227
rs1775255
rs4929925
rs11012726
rs10134477
rs9314096
rs8095324
rs7787699
All
−0.6
−0.4
−0.2
0.0
0.2
MR leave−one−out sensitivity analysis for
'Fruit liking || id:ebi−fl187−GCST90094766' on 'Rheumatoid arthritis || id:finngen_R11_M13_RHEUMA'

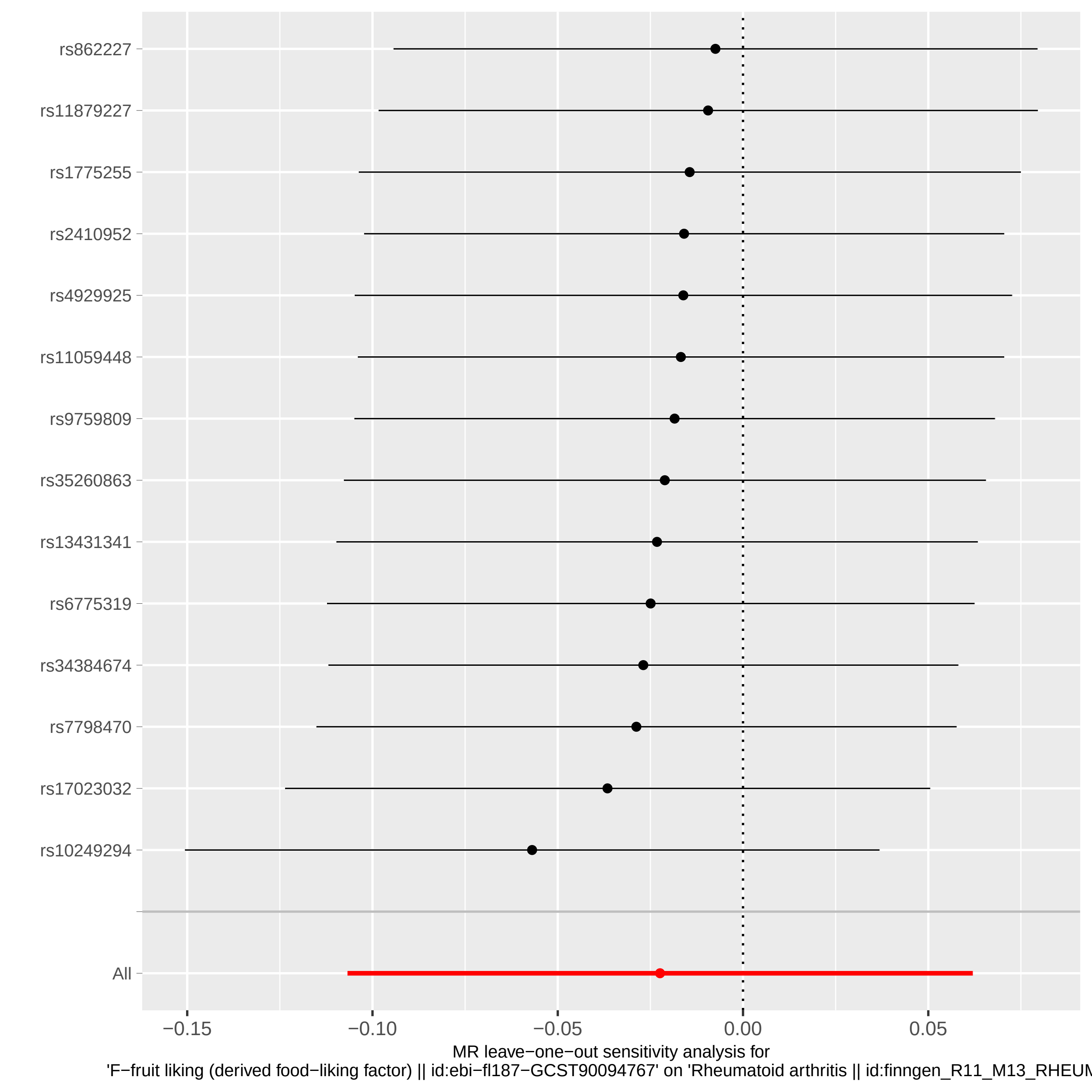
rs862227
rs11879227
rs1775255
rs2410952
rs4929925
rs11059448
rs9759809
rs35260863
rs13431341
rs6775319
rs34384674
rs7798470
rs17023032
rs10249294
All
−0.15
−0.10
−0.05
0.00
0.05
MR leave−one−out sensitivity analysis for
'F−fruit liking (derived food−liking factor) || id:ebi−fl187−GCST90094767' on 'Rheumatoid arthritis || id:finngen_R11_M13_RHEUM

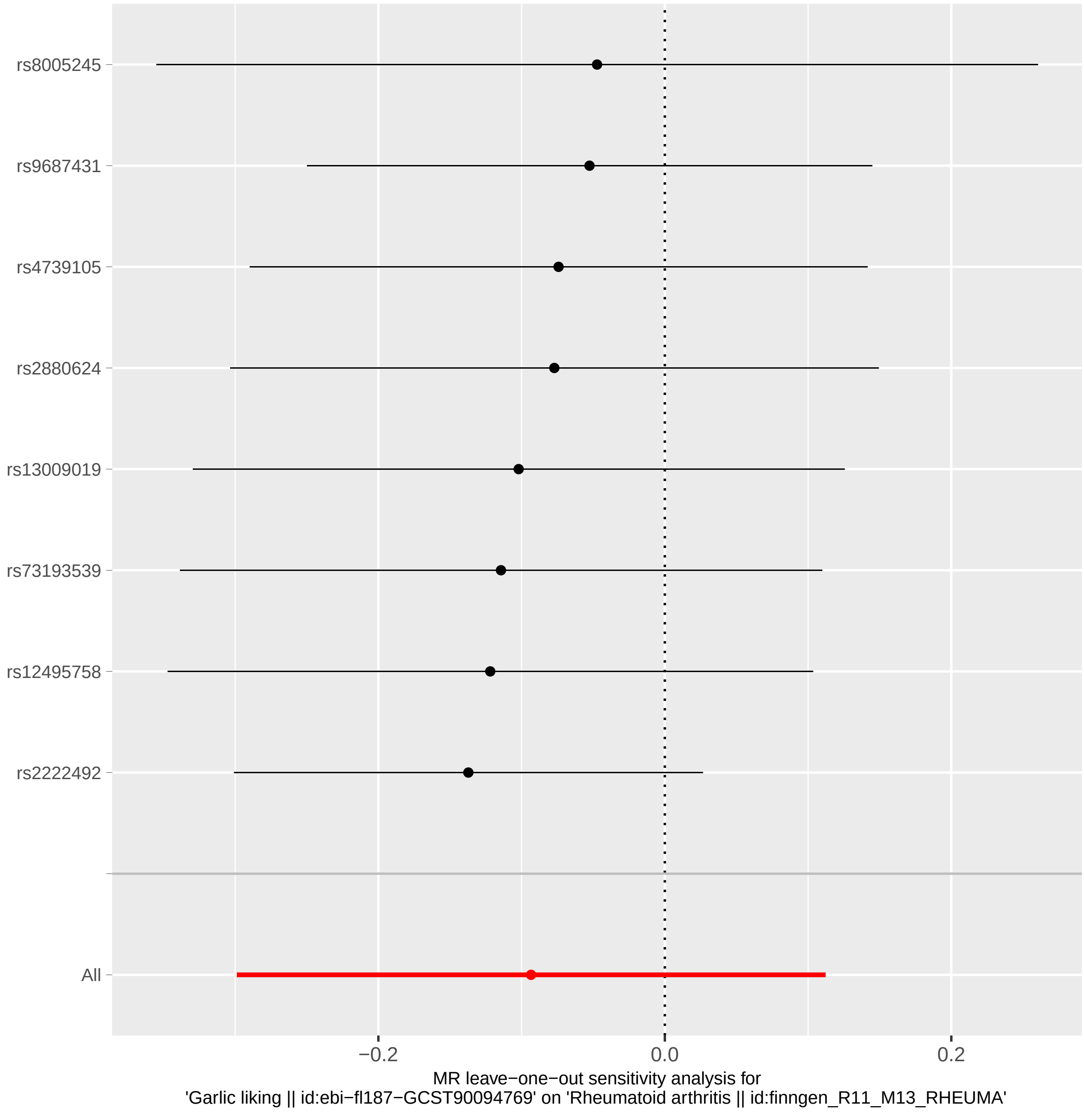
rs8005245
rs9687431
rs4739105
rs2880624
rs13009019
rs73193539
rs12495758
rs2222492
All
−0.2
0.0
0.2
MR leave−one−out sensitivity analysis for
'Garlic liking || id:ebi−fl187−GCST90094769' on 'Rheumatoid arthritis || id:finngen_R11_M13_RHEUMA'

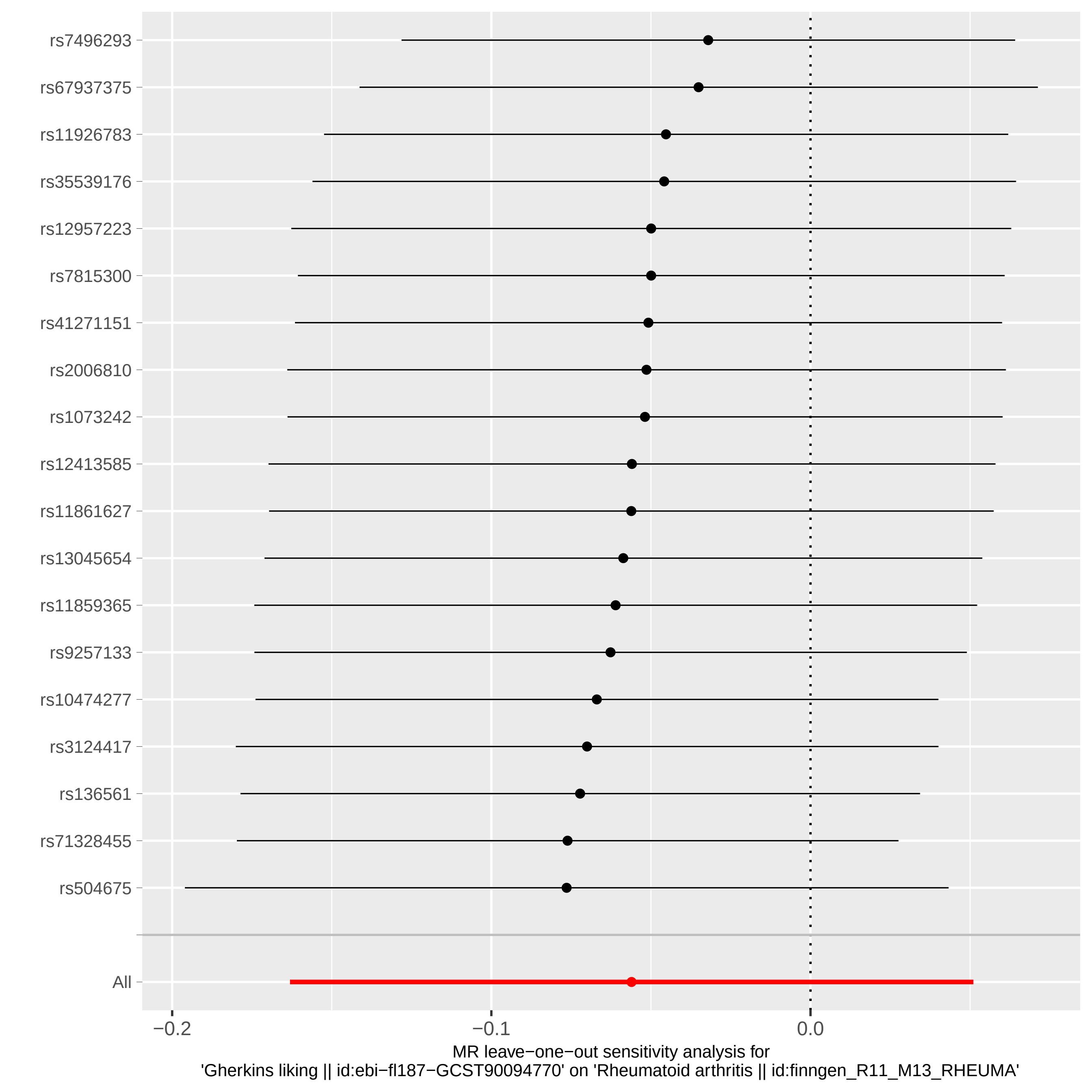
rs7496293
rs67937375
rs11926783
rs35539176
rs12957223
rs7815300
rs41271151
rs2006810
rs1073242
rs12413585
rs11861627
rs13045654
rs11859365
rs9257133
rs10474277
rs3124417
rs136561
rs71328455
rs504675
All
−0.2
−0.1
0.0
MR leave−one−out sensitivity analysis for
'Gherkins liking || id:ebi−fl187−GCST90094770' on 'Rheumatoid arthritis || id:finngen_R11_M13_RHEUMA'

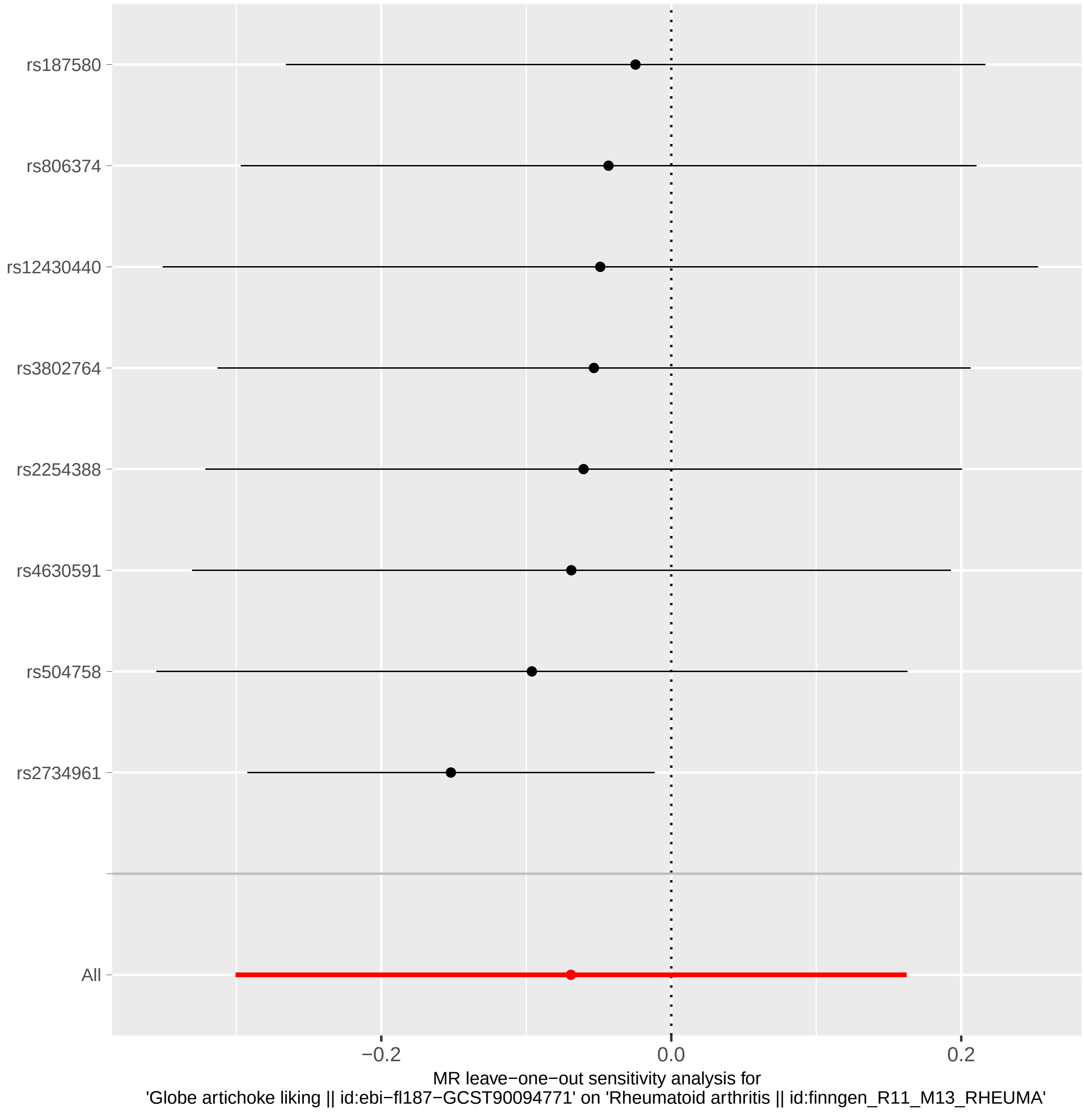
rs187580
rs806374
rs12430440
rs3802764
rs2254388
rs4630591
rs504758
rs2734961
All
−0.2
0.0
0.2
MR leave−one−out sensitivity analysis for
'Globe artichoke liking || id:ebi−fl187−GCST90094771' on 'Rheumatoid arthritis || id:finngen_R11_M13_RHEUMA'

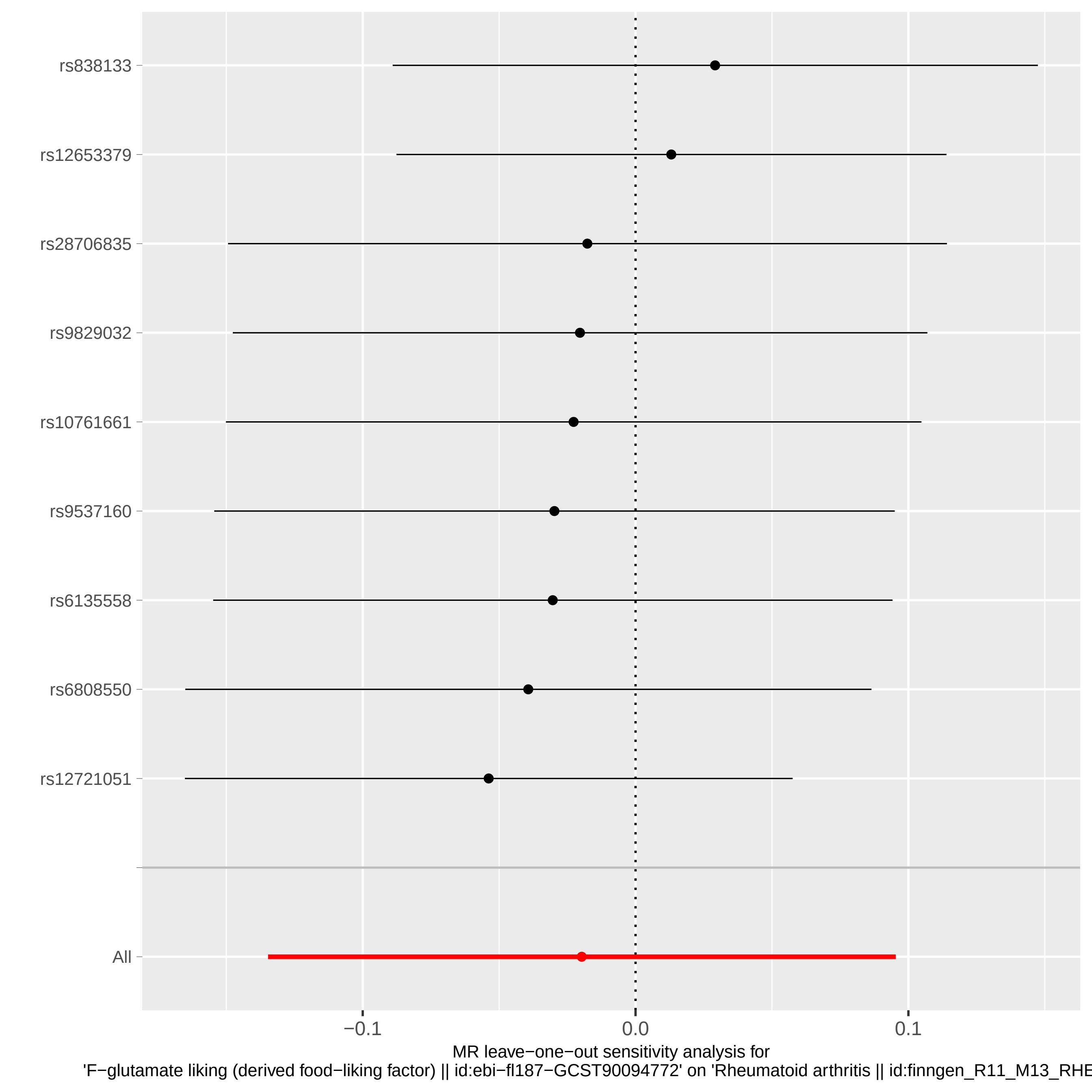

rs838133
rs12653379
rs28706835
rs9829032
rs10761661
rs9537160
rs6135558
rs6808550
rs12721051
All
−0.1
0.0
0.1
MR leave−one−out sensitivity analysis for
'F−glutamate liking (derived food−liking factor) || id:ebi−fl187−GCST90094772' on 'Rheumatoid arthritis || id:finngen_R11_M13_RHE

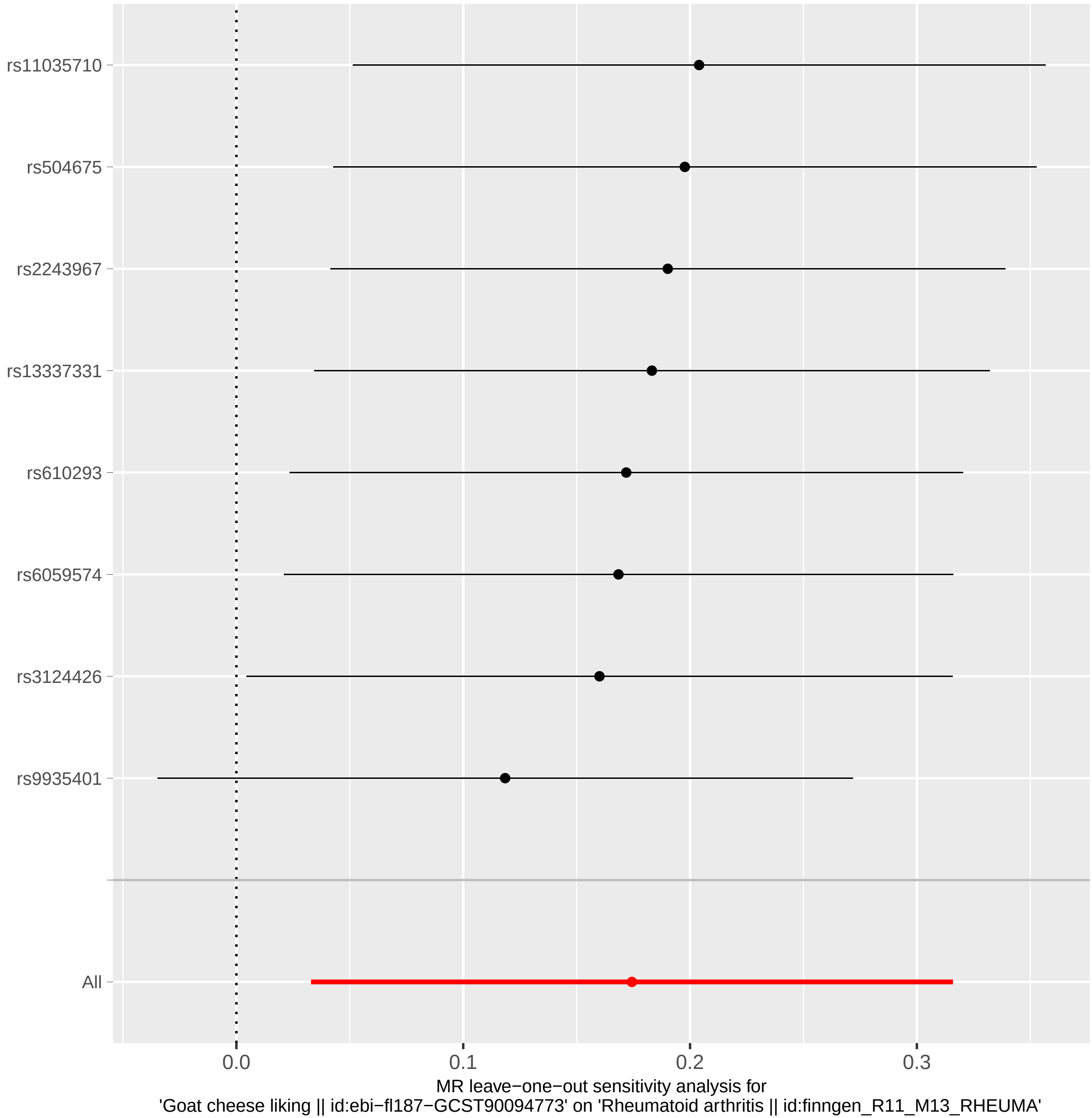

rs11035710
rs504675
rs2243967
rs13337331
rs610293
rs6059574
rs3124426
rs9935401
All
0.0
0.1
0.2
0.3
MR leave−one−out sensitivity analysis for
'Goat cheese liking || id:ebi−fl187−GCST90094773' on 'Rheumatoid arthritis || id:finngen_R11_M13_RHEUMA'

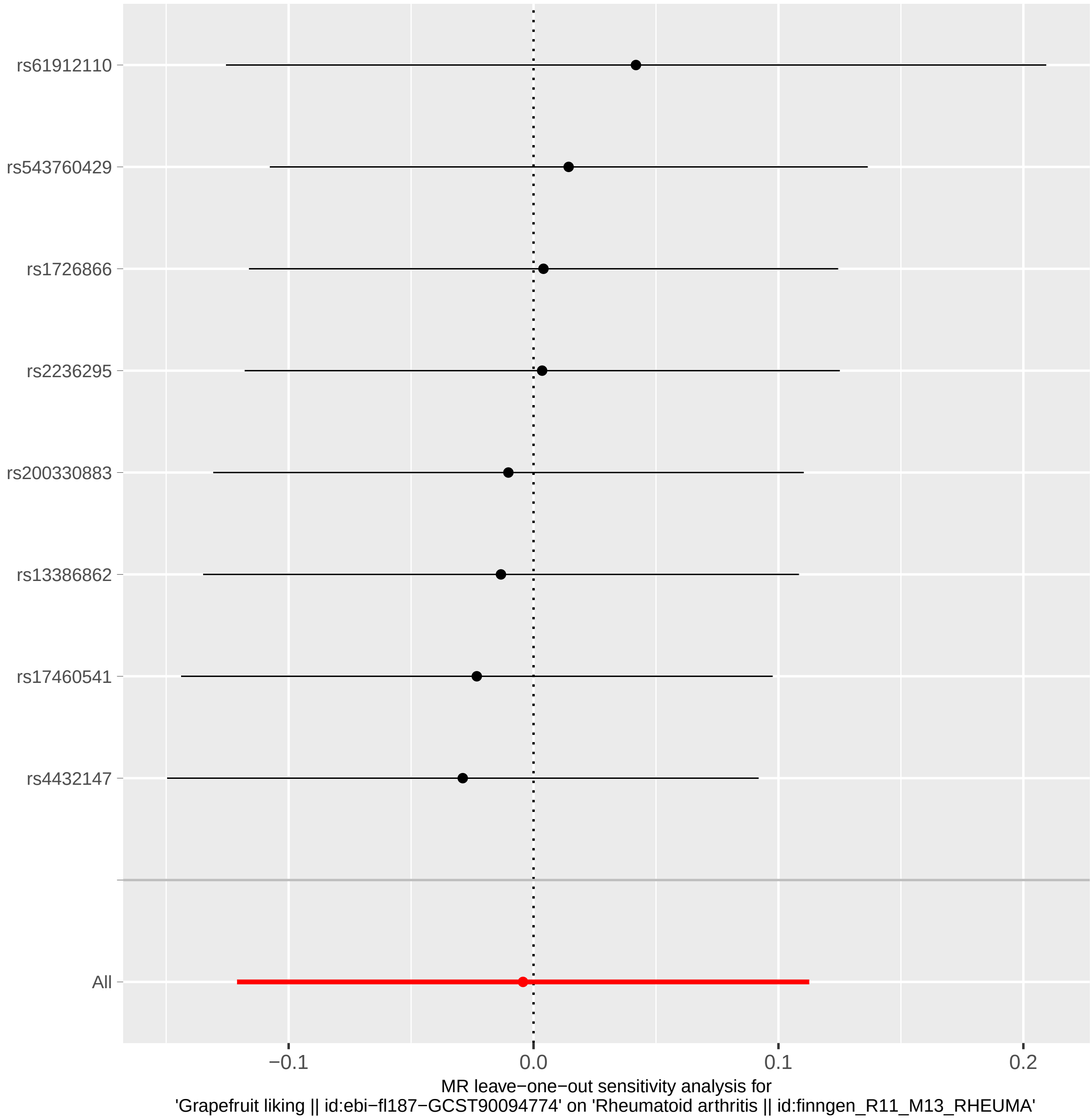
rs61912110
rs543760429
rs1726866
rs2236295
rs200330883
rs13386862
rs17460541
rs4432147
All
−0.1
0.0
0.1
0.2
MR leave−one−out sensitivity analysis for
'Grapefruit liking || id:ebi−fl187−GCST90094774' on 'Rheumatoid arthritis || id:finngen_R11_M13_RHEUMA'

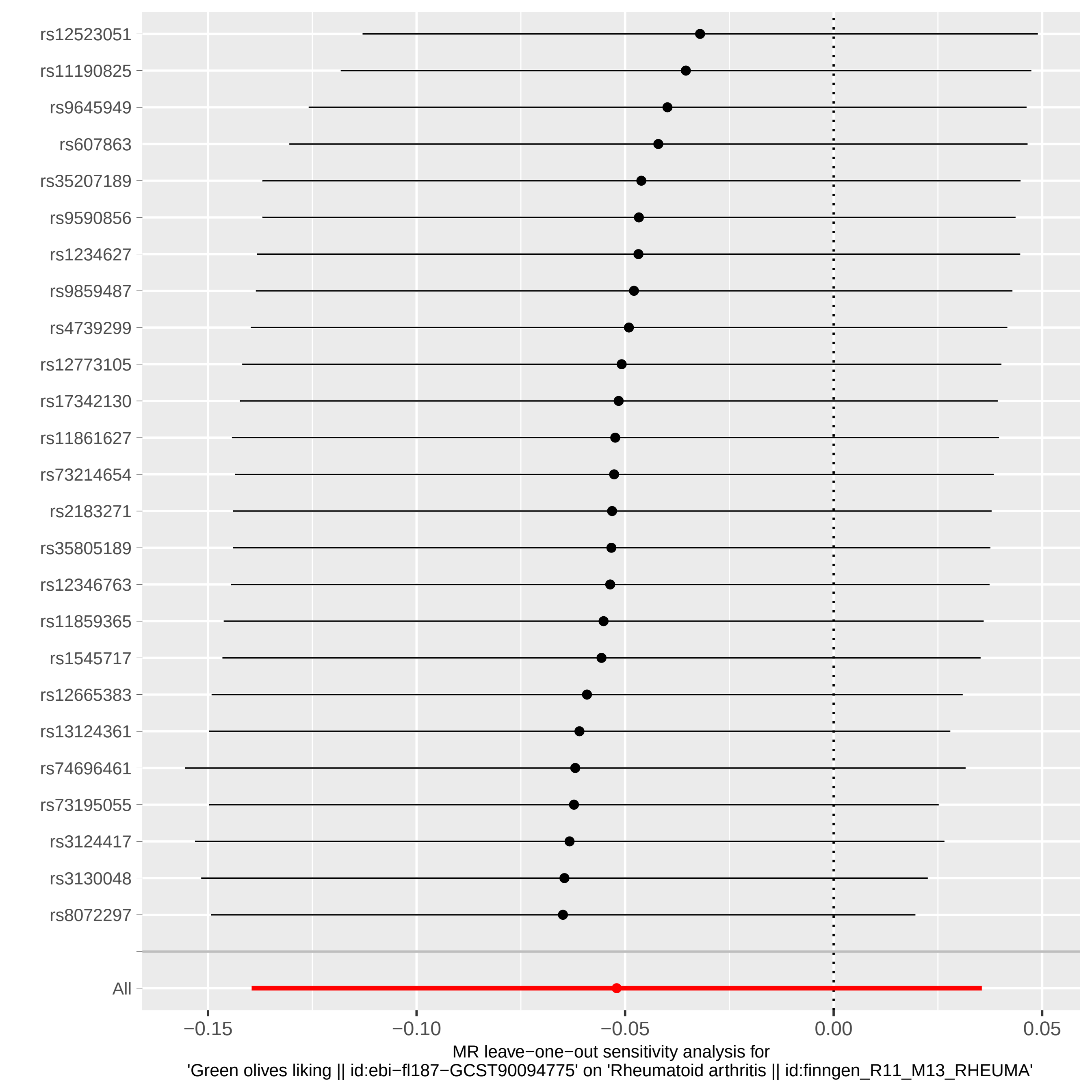
rs12523051
rs11190825
rs9645949
rs607863
rs35207189
rs9590856
rs1234627
rs9859487
rs4739299
rs12773105
rs17342130
rs11861627
rs73214654
rs2183271
rs35805189
rs12346763
rs11859365
rs1545717
rs12665383
rs13124361
rs74696461
rs73195055
rs3124417
rs3130048
rs8072297
All
−0.15
−0.10
−0.05
0.00
0.05
MR leave−one−out sensitivity analysis for
'Green olives liking || id:ebi−fl187−GCST90094775' on 'Rheumatoid arthritis || id:finngen_R11_M13_RHEUMA'

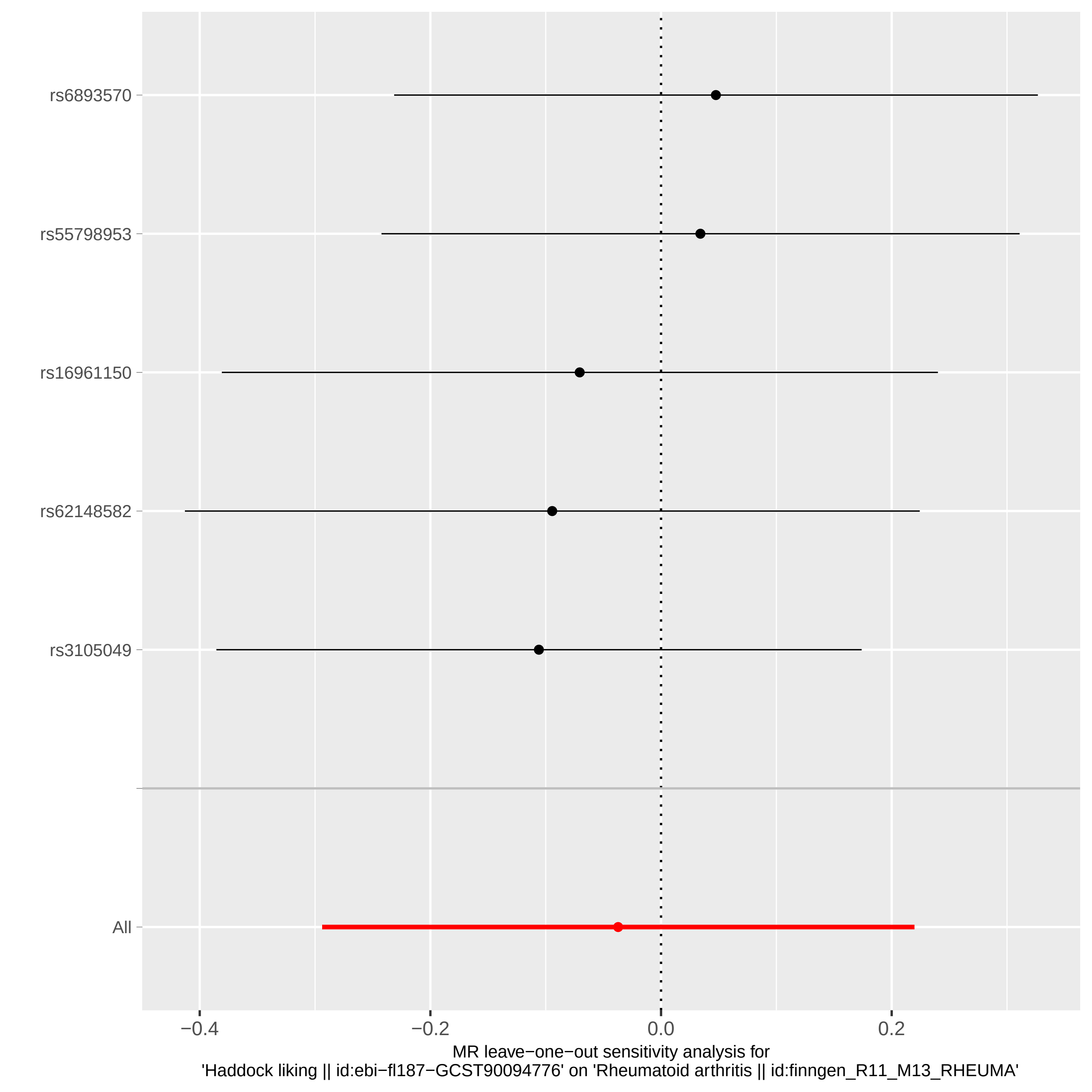
rs6893570
rs55798953
rs16961150
rs62148582
rs3105049
All
−0.4
−0.2
0.0
0.2
MR leave−one−out sensitivity analysis for
'Haddock liking || id:ebi−fl187−GCST90094776' on 'Rheumatoid arthritis || id:finngen_R11_M13_RHEUMA'

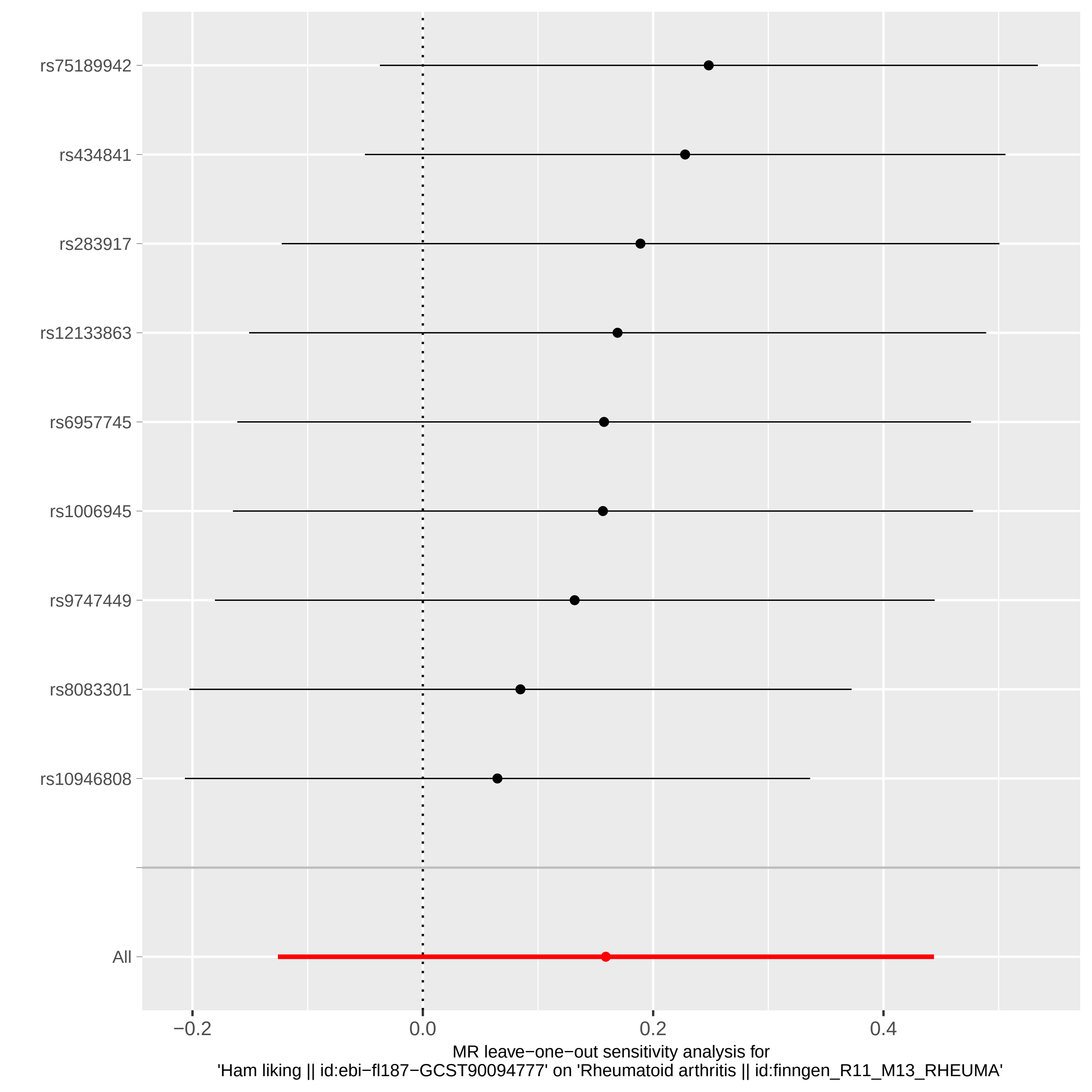
rs75189942
rs434841
rs283917
rs12133863
rs6957745
rs1006945
rs9747449
rs8083301
rs10946808
All
−0.2
0.0
0.2
0.4
MR leave−one−out sensitivity analysis for
'Ham liking || id:ebi−fl187−GCST90094777' on 'Rheumatoid arthritis || id:finngen_R11_M13_RHEUMA'

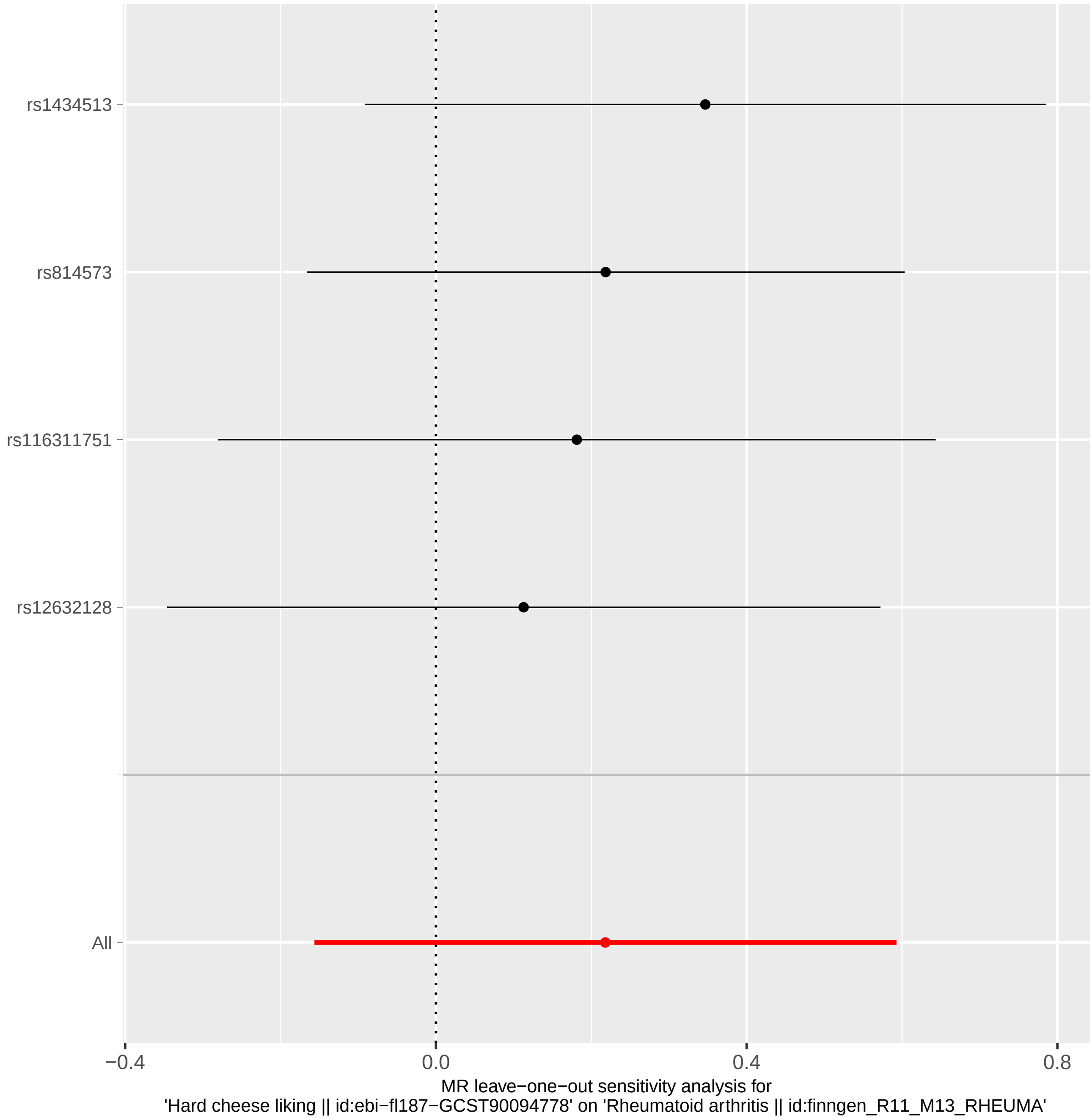
rs1434513
rs814573
rs116311751
rs12632128
All
−0.4
0.0
0.4
0.8
MR leave−one−out sensitivity analysis for
'Hard cheese liking || id:ebi−fl187−GCST90094778' on 'Rheumatoid arthritis || id:finngen_R11_M13_RHEUMA'

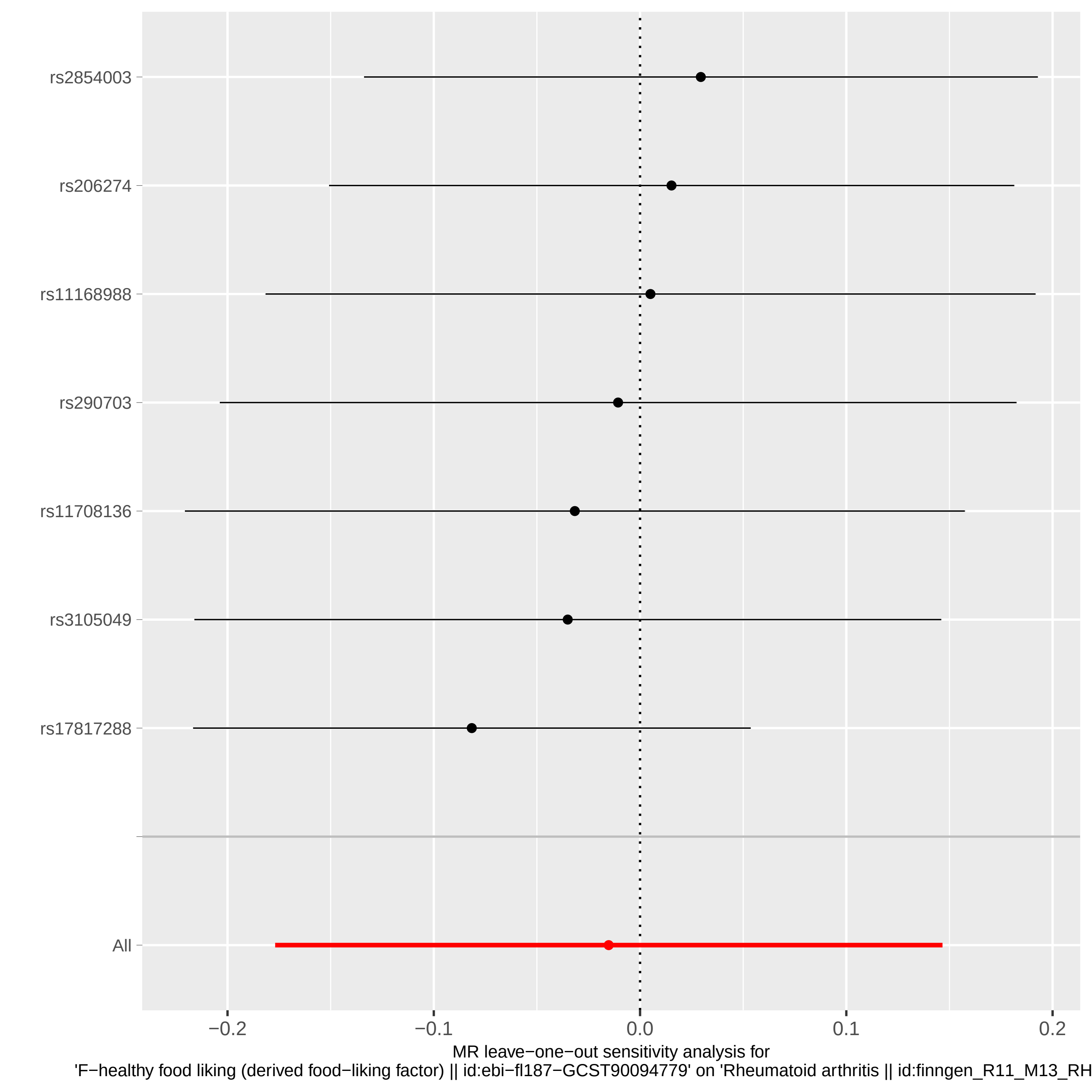

rs2854003
rs206274
rs11168988
rs290703
rs11708136
rs3105049
rs17817288
All
−0.2
−0.1
0.0
0.1
0.2
MR leave−one−out sensitivity analysis for
'F−healthy food liking (derived food−liking factor) || id:ebi−fl187−GCST90094779' on 'Rheumatoid arthritis || id:finngen_R11_M13_RH

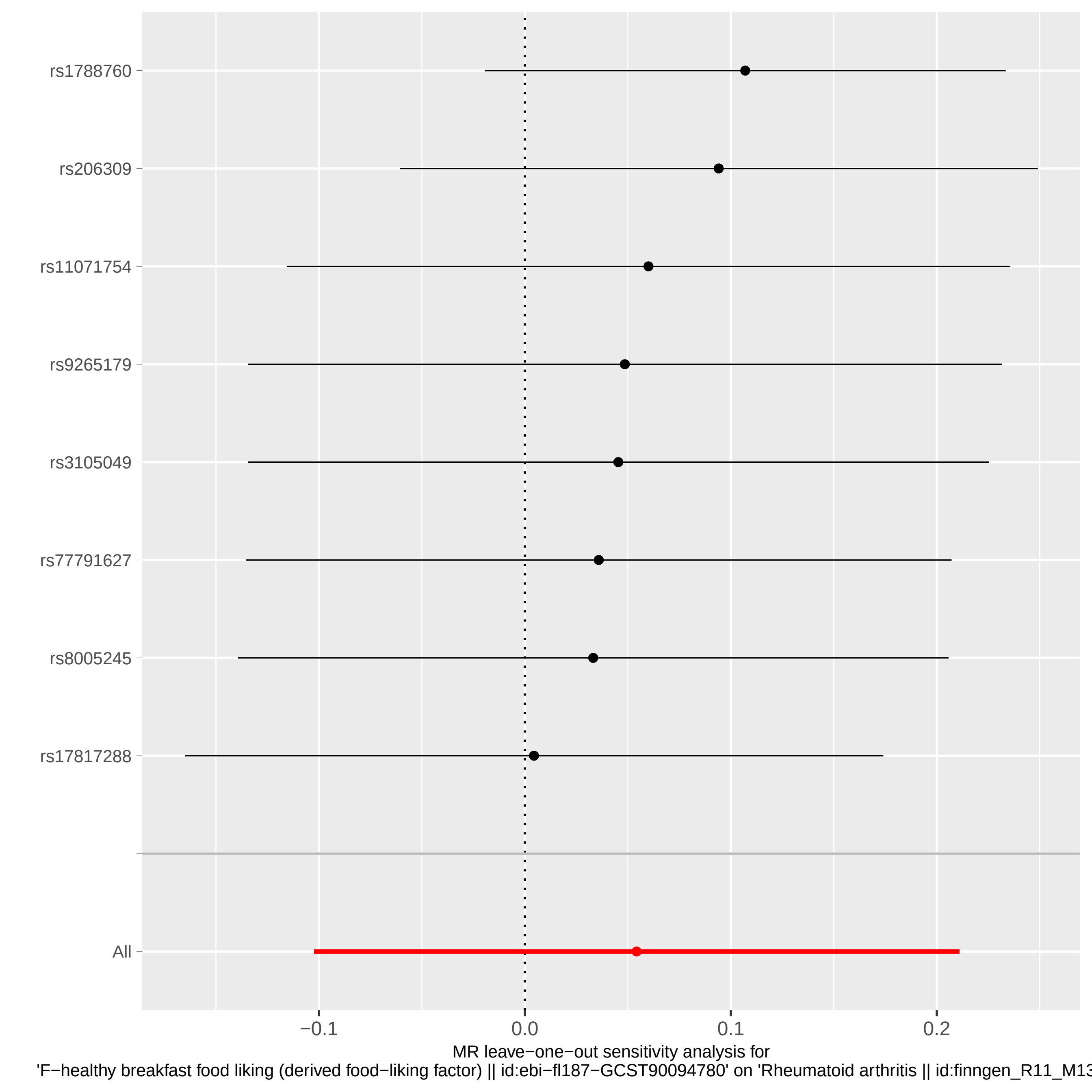
rs1788760
rs206309
rs11071754
rs9265179
rs3105049
rs77791627
rs8005245
rs17817288
All
−0.1
0.0
0.1
0.2
MR leave−one−out sensitivity analysis for
'F−healthy breakfast food liking (derived food−liking factor) || id:ebi−fl187−GCST90094780' on 'Rheumatoid arthritis || id:finngen_R11_M13

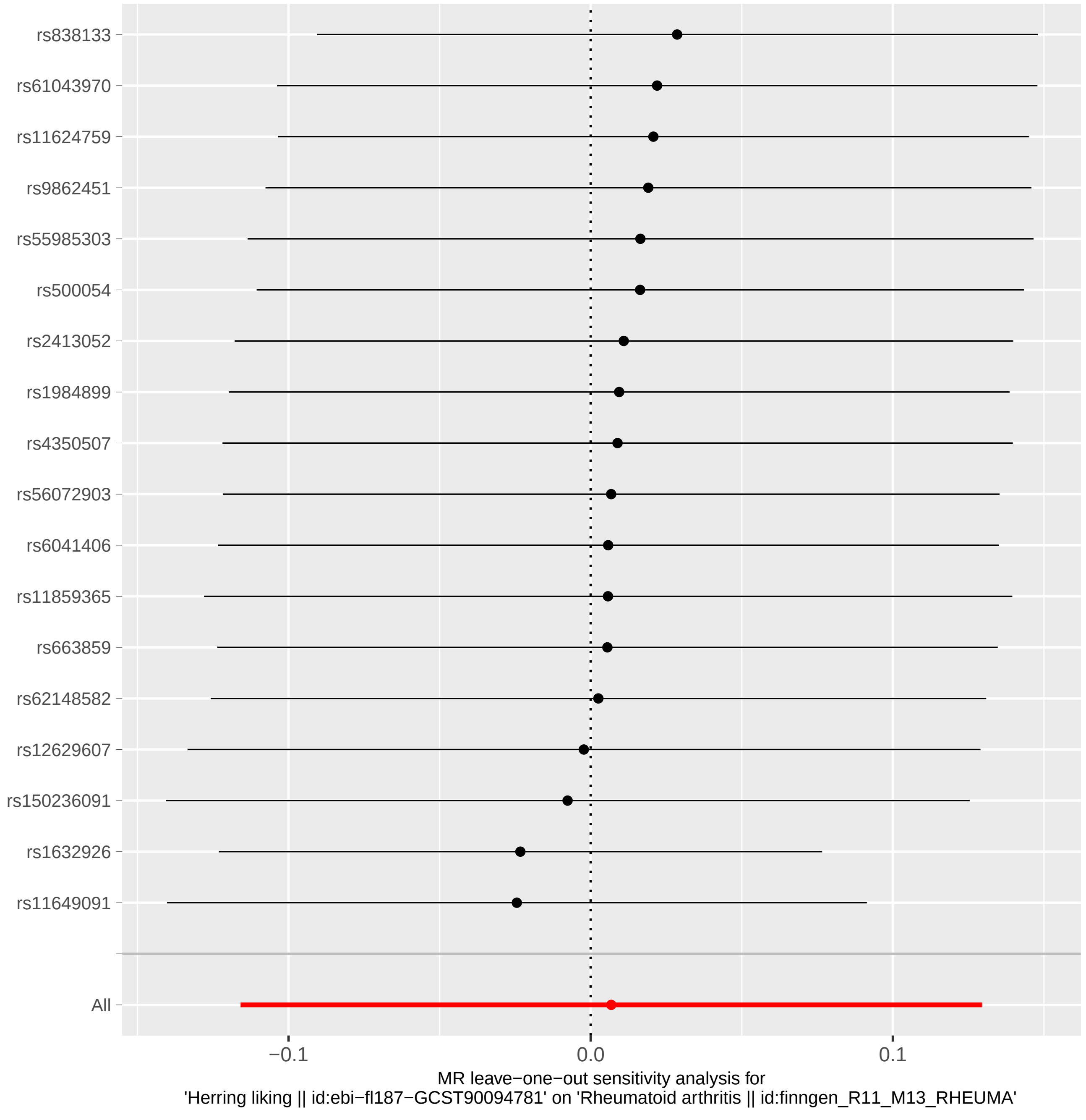
rs838133
rs61043970
rs11624759
rs9862451
rs55985303
rs500054
rs2413052
rs1984899
rs4350507
rs56072903
rs6041406
rs11859365
rs663859
rs62148582
rs12629607
rs150236091
rs1632926
rs11649091
All
−0.1
0.0
0.1
MR leave−one−out sensitivity analysis for
'Herring liking || id:ebi−fl187−GCST90094781' on 'Rheumatoid arthritis || id:finngen_R11_M13_RHEUMA'

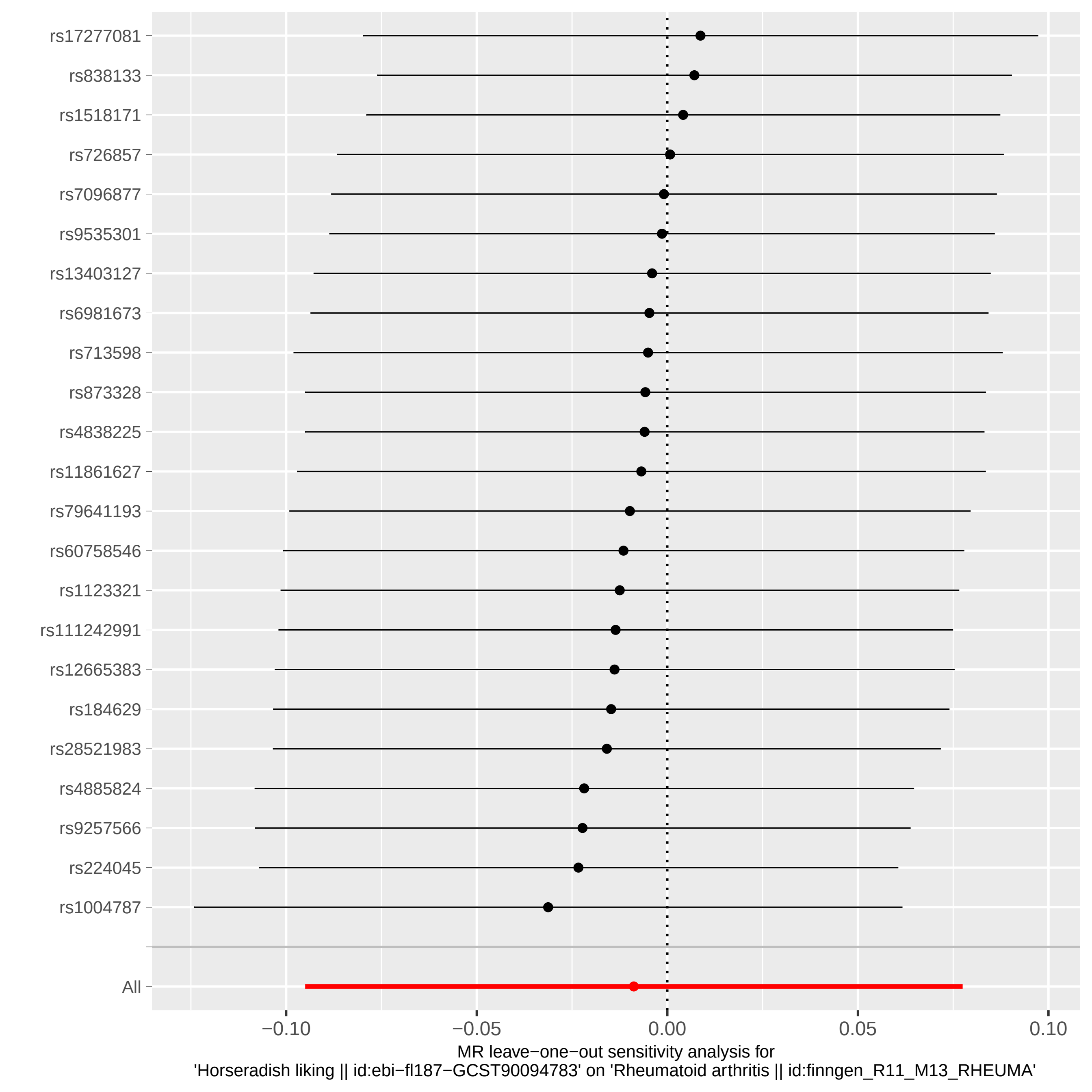
rs17277081
rs838133
rs1518171
rs726857
rs7096877
rs9535301
rs13403127
rs6981673
rs713598
rs873328
rs4838225
rs11861627
rs79641193
rs60758546
rs1123321
rs111242991
rs12665383
rs184629
rs28521983
rs4885824
rs9257566
rs224045
rs1004787
All
−0.10
−0.05
0.00
0.05
0.10
MR leave−one−out sensitivity analysis for
'Horseradish liking || id:ebi−fl187−GCST90094783' on 'Rheumatoid arthritis || id:finngen_R11_M13_RHEUMA'

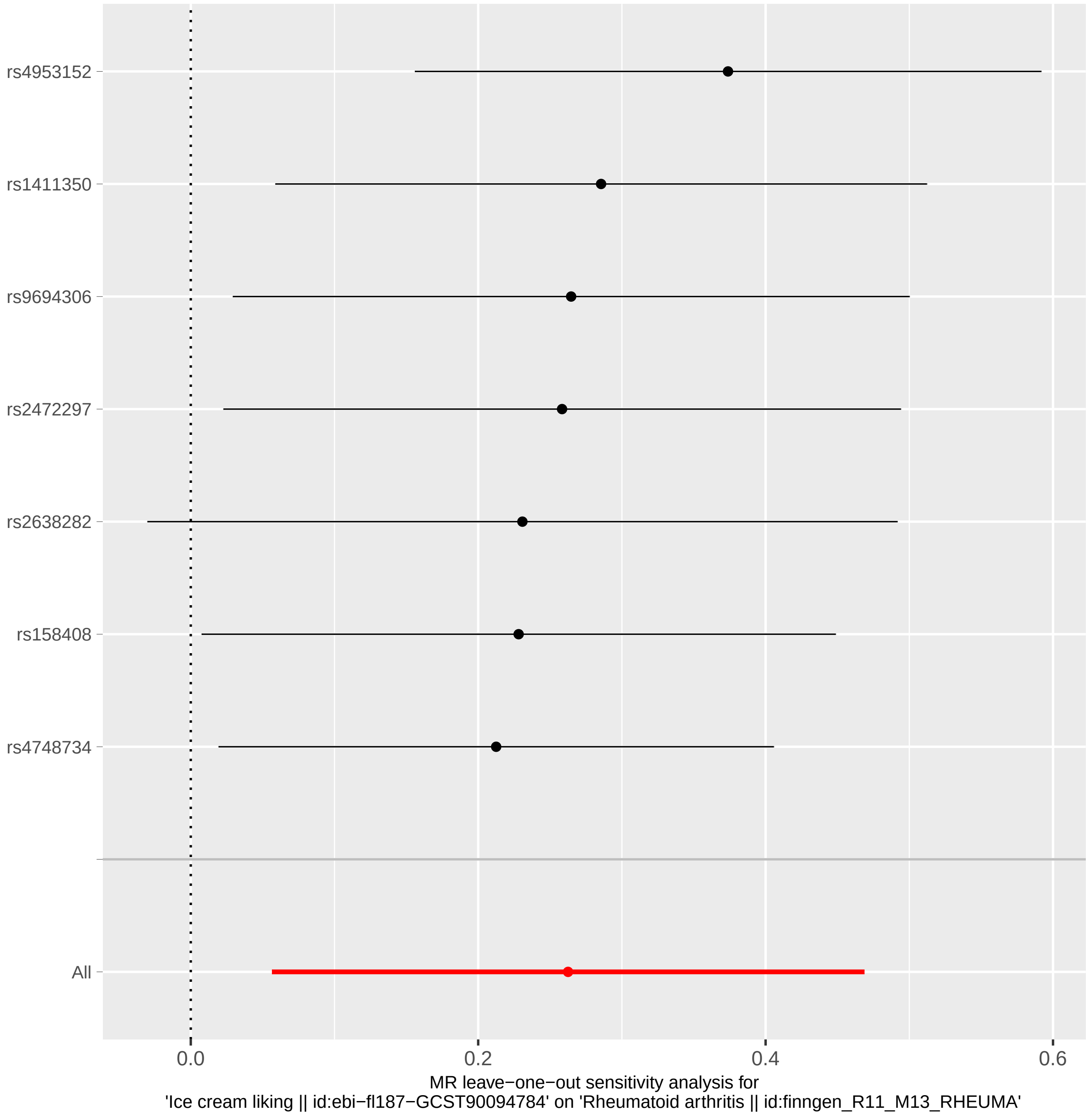
rs4953152
rs1411350
rs9694306
rs2472297
rs2638282
rs158408
rs4748734
All
0.0
0.2
0.4
0.6
MR leave−one−out sensitivity analysis for
'Ice cream liking || id:ebi−fl187−GCST90094784' on 'Rheumatoid arthritis || id:finngen_R11_M13_RHEUMA'

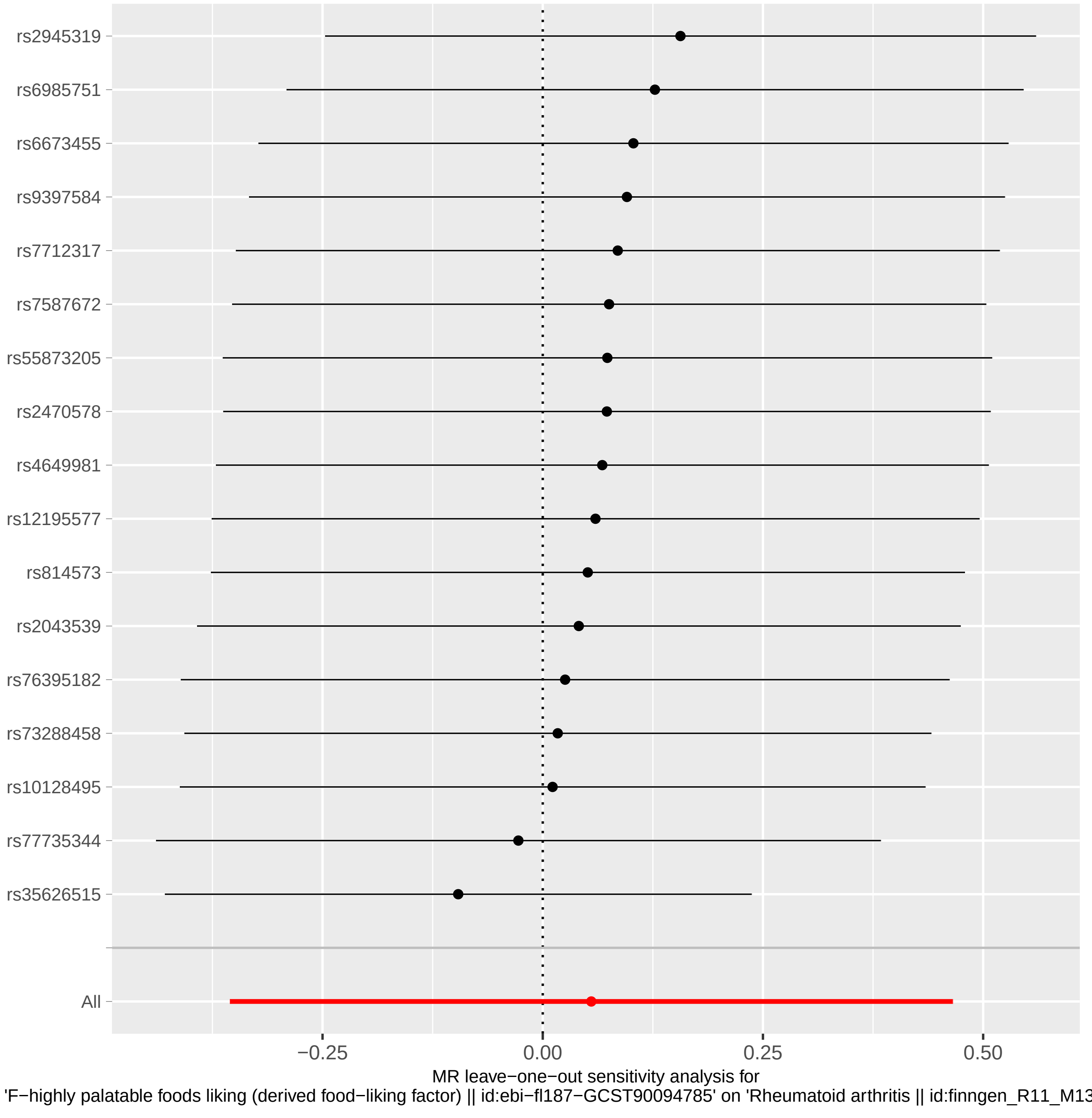
rs2945319
rs6985751
rs6673455
rs9397584
rs7712317
rs7587672
rs55873205
rs2470578
rs4649981
rs12195577
rs814573
rs2043539
rs76395182
rs73288458
rs10128495
rs77735344
rs35626515
All
−0.25
0.00
0.25
0.50
MR leave−one−out sensitivity analysis for
'F−highly palatable foods liking (derived food−liking factor) || id:ebi−fl187−GCST90094785' on 'Rheumatoid arthritis || id:finngen_R11_M13

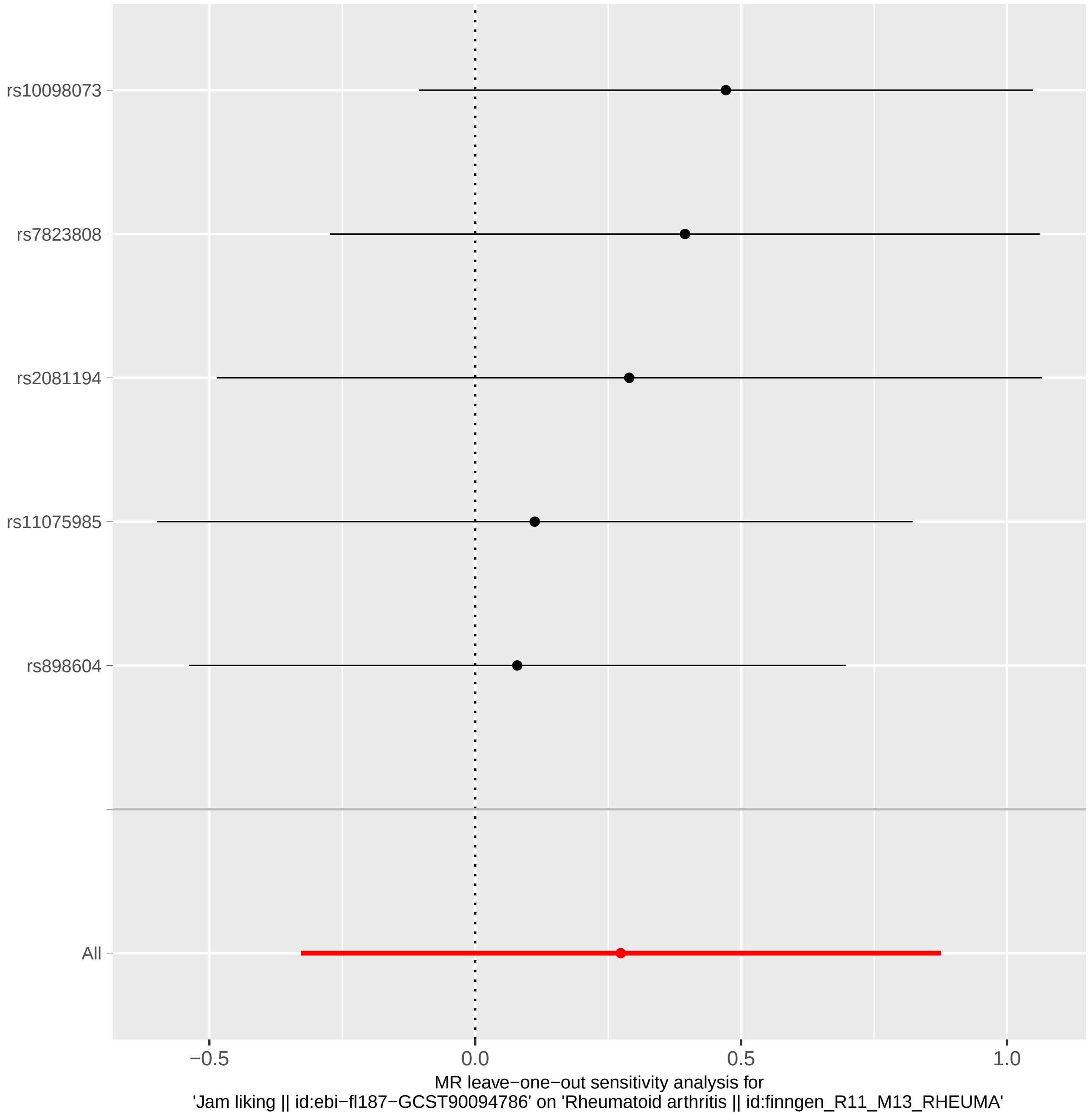
rs10098073
rs7823808
rs2081194
rs11075985
rs898604
All
−0.5
0.0
0.5
1.0
MR leave−one−out sensitivity analysis for
'Jam liking || id:ebi−fl187−GCST90094786' on 'Rheumatoid arthritis || id:finngen_R11_M13_RHEUMA'

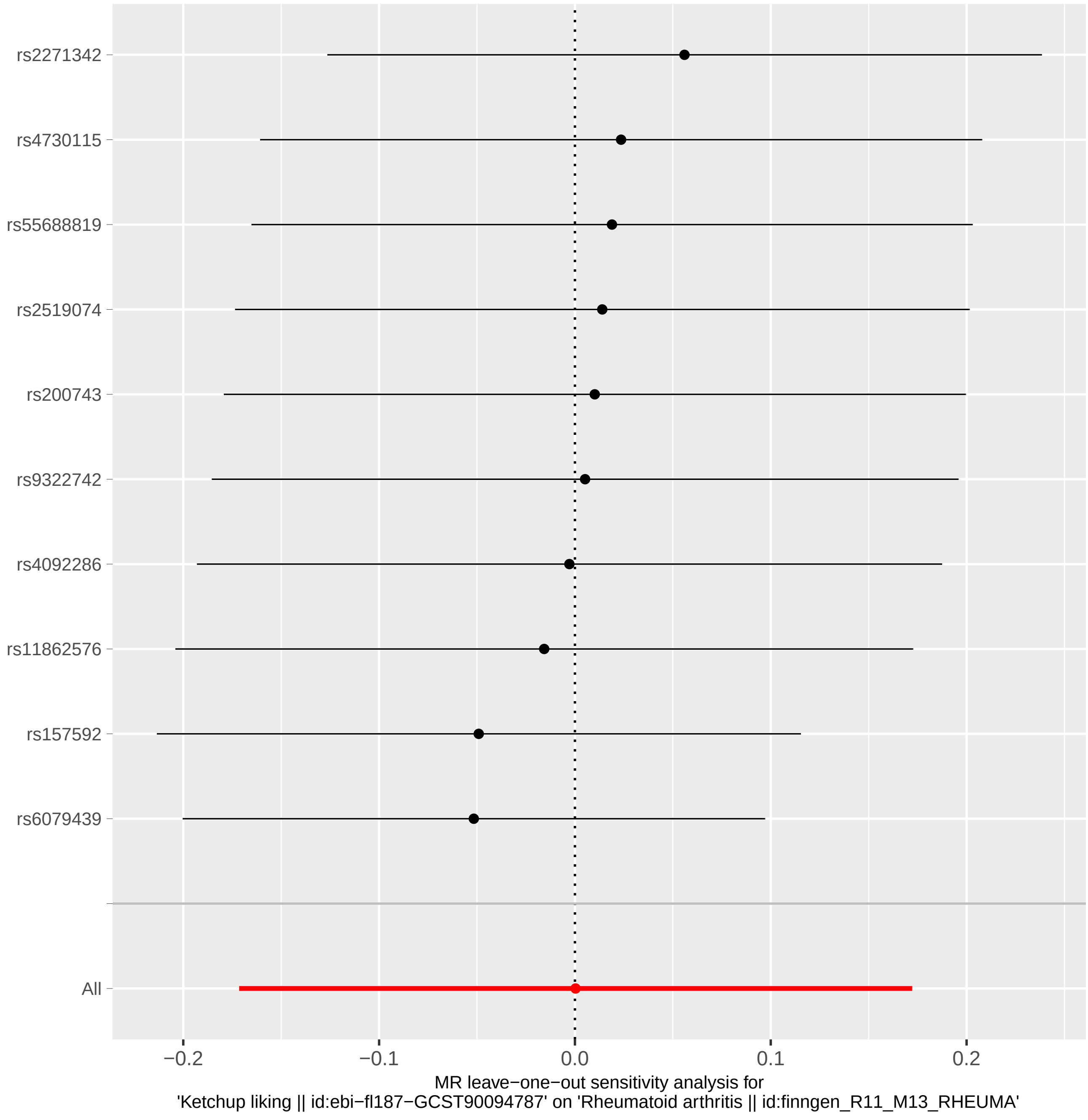
rs2271342
rs4730115
rs55688819
rs2519074
rs200743
rs9322742
rs4092286
rs11862576
rs157592
rs6079439
All
−0.2
−0.1
0.0
0.1
0.2
MR leave−one−out sensitivity analysis for
'Ketchup liking || id:ebi−fl187−GCST90094787' on 'Rheumatoid arthritis || id:finngen_R11_M13_RHEUMA'

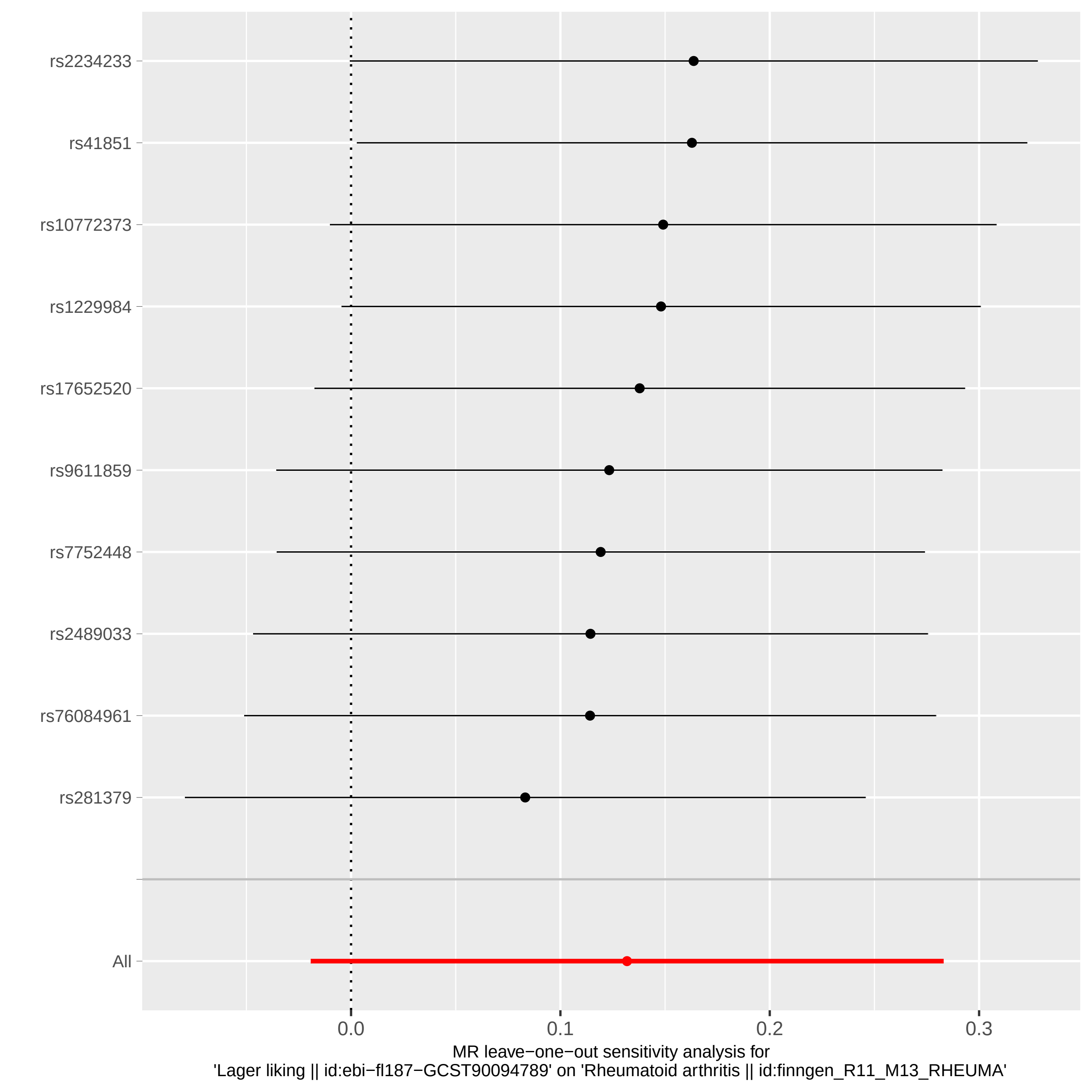

rs2234233
rs41851
rs10772373
rs1229984
rs17652520
rs9611859
rs7752448
rs2489033
rs76084961
rs281379
All
0.0
0.1
0.2
0.3
MR leave−one−out sensitivity analysis for
'Lager liking || id:ebi−fl187−GCST90094789' on 'Rheumatoid arthritis || id:finngen_R11_M13_RHEUMA'

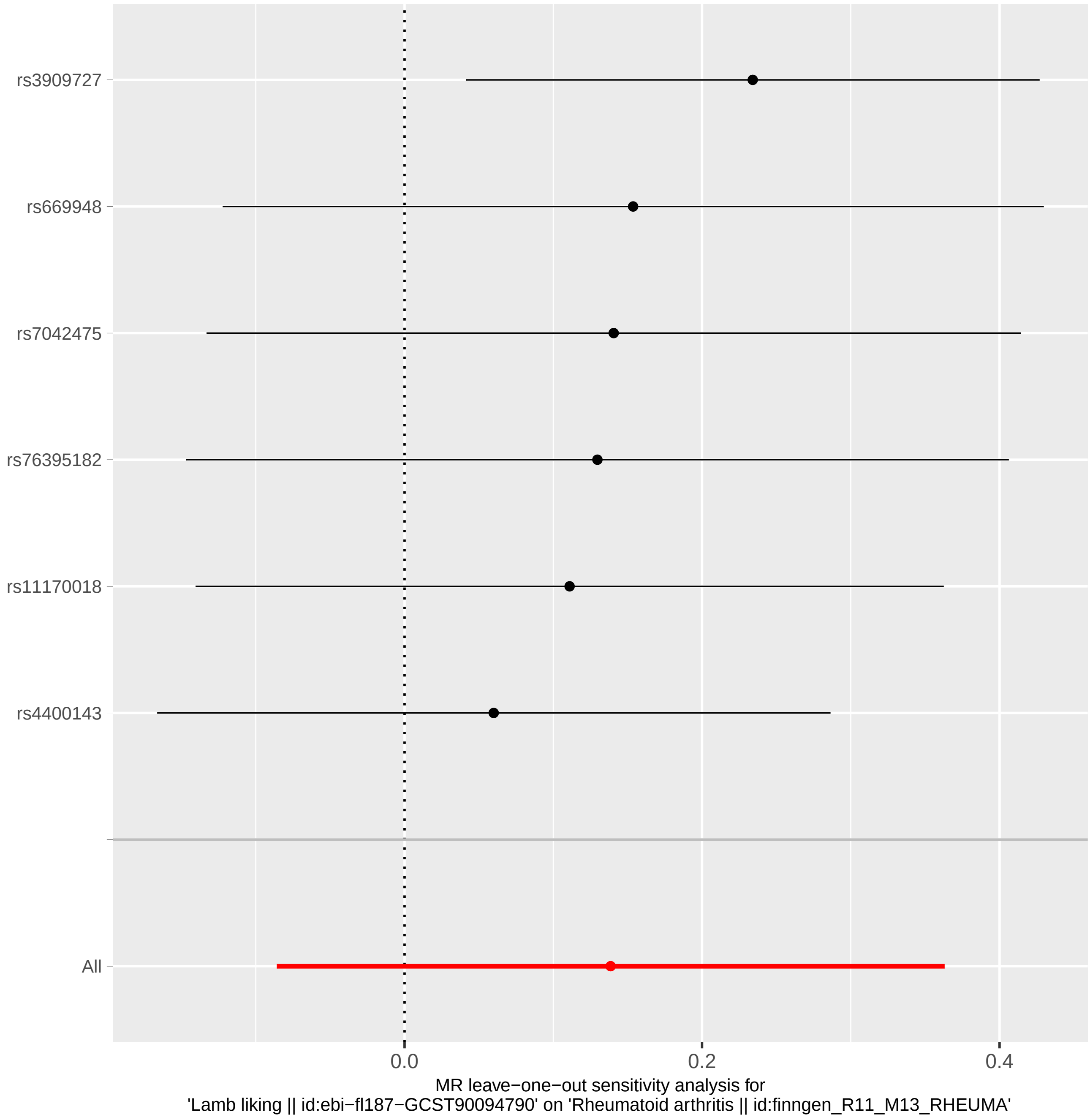

rs3909727
rs669948
rs7042475
rs76395182
rs11170018
rs4400143
All
0.0
0.2
0.4
MR leave−one−out sensitivity analysis for
'Lamb liking || id:ebi−fl187−GCST90094790' on 'Rheumatoid arthritis || id:finngen_R11_M13_RHEUMA'

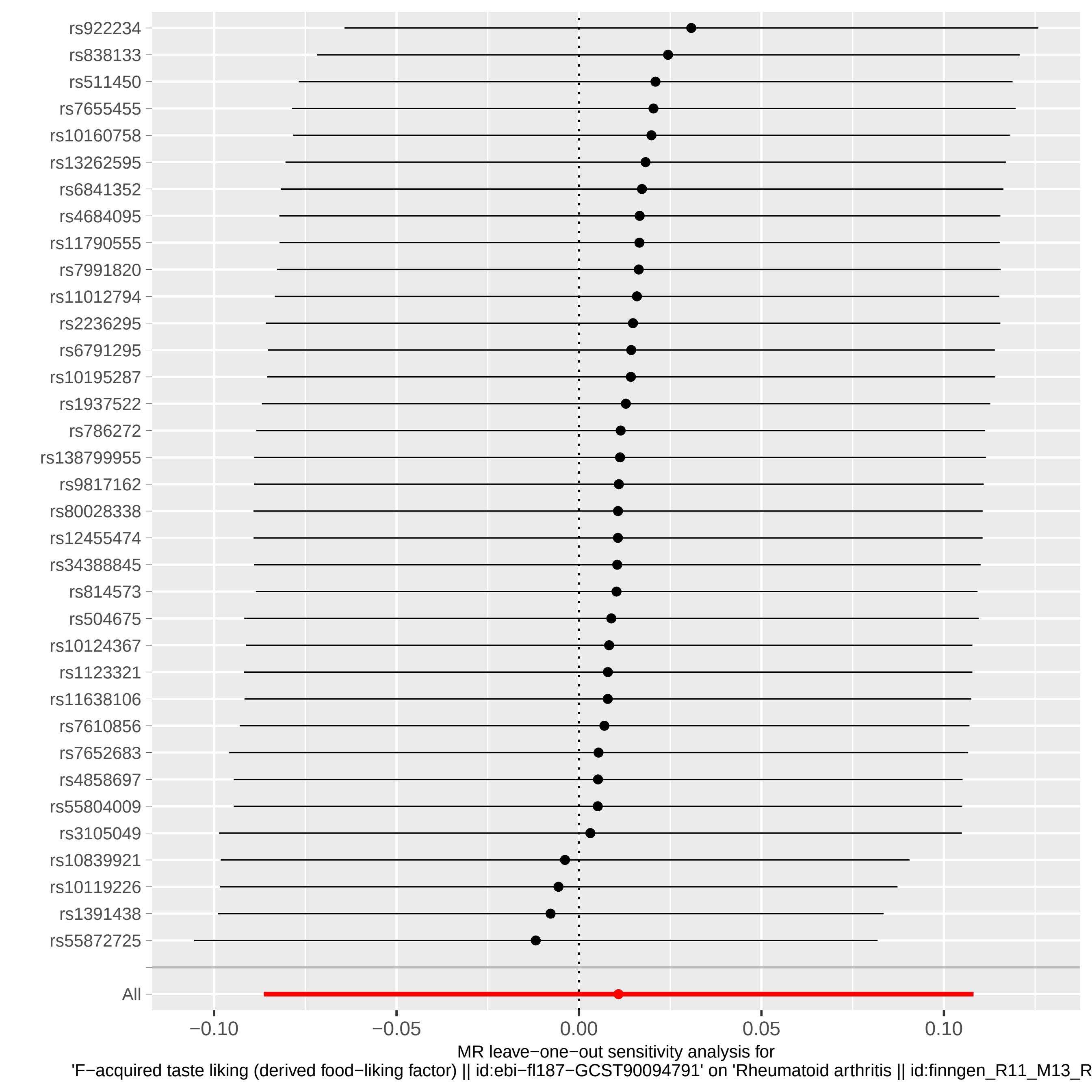
rs922234
rs838133
rs511450
rs7655455
rs10160758
rs13262595
rs6841352
rs4684095
rs11790555
rs7991820
rs11012794
rs2236295
rs6791295
rs10195287
rs1937522
rs786272
rs138799955
rs9817162
rs80028338
rs12455474
rs34388845
rs814573
rs504675
rs10124367
rs1123321
rs11638106
rs7610856
rs7652683
rs4858697
rs55804009
rs3105049
rs10839921
rs10119226
rs1391438
rs55872725
All
−0.10
−0.05
0.00
0.05
0.10
MR leave−one−out sensitivity analysis for
'F−acquired taste liking (derived food−liking factor) || id:ebi−fl187−GCST90094791' on 'Rheumatoid arthritis || id:finngen_R11_M13_R

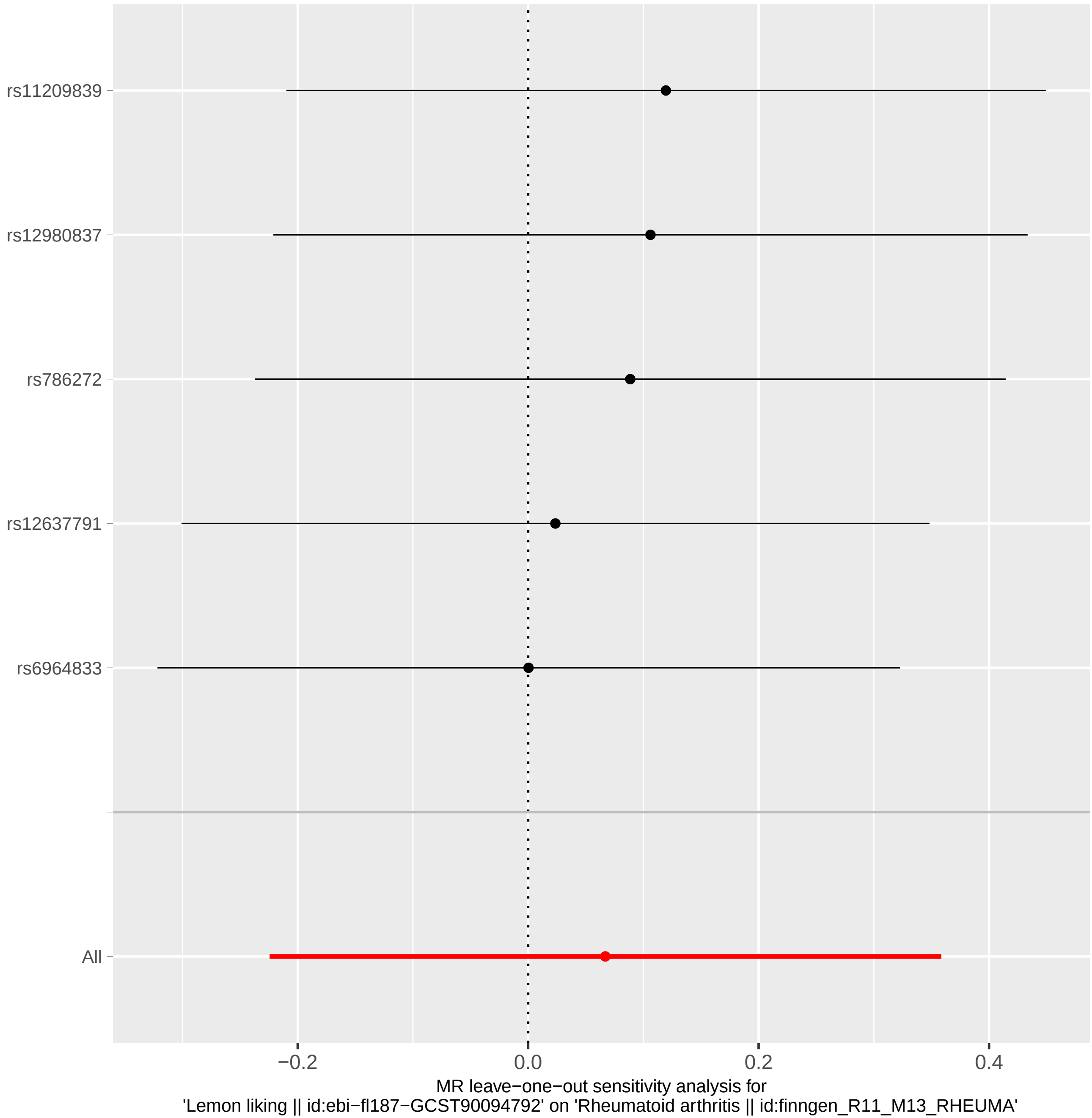
rs11209839
rs12980837
rs786272
rs12637791
rs6964833
All
−0.2
0.0
0.2
0.4
MR leave−one−out sensitivity analysis for
'Lemon liking || id:ebi−fl187−GCST90094792' on 'Rheumatoid arthritis || id:finngen_R11_M13_RHEUMA'

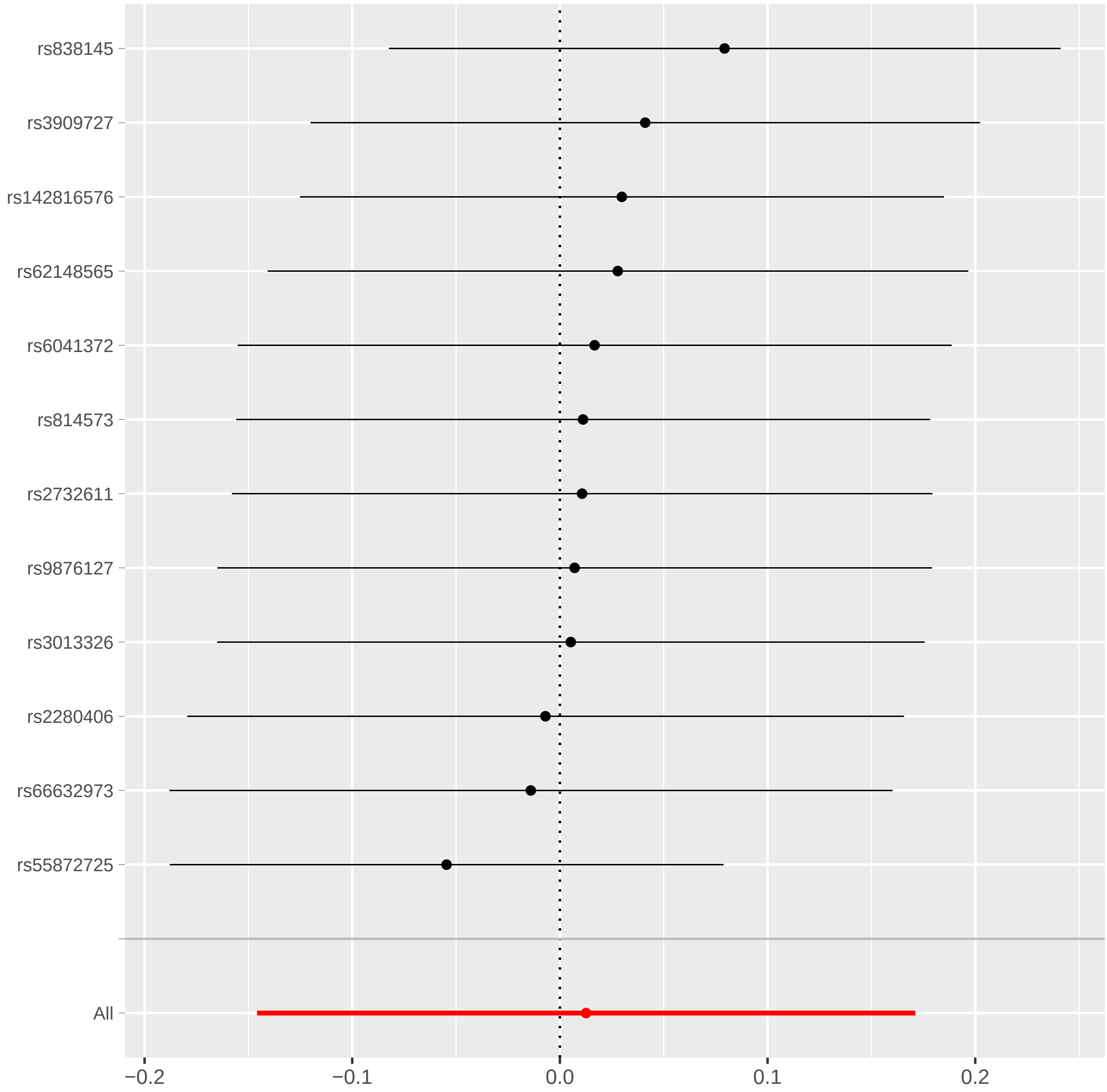

MR leave−one−out sensitivity analysis for
'Liver liking || id:ebi−fl187−GCST90094794' on 'Rheumatoid arthritis || id:finngen_R11_M13_RHEUMA'

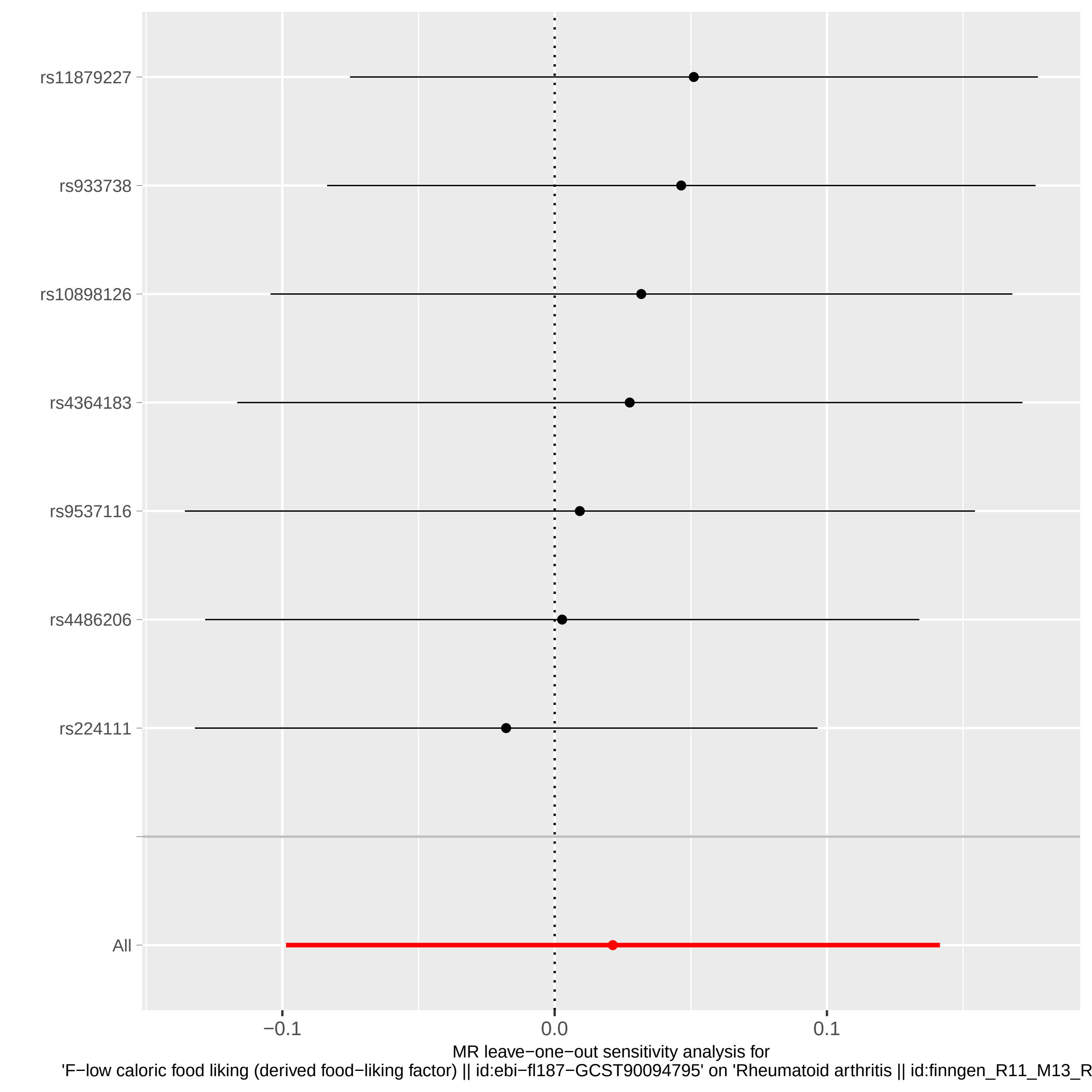
rs11879227
rs933738
rs10898126
rs4364183
rs9537116
rs4486206
rs224111
All
−0.1
0.0
0.1
MR leave−one−out sensitivity analysis for
'F−low caloric food liking (derived food−liking factor) || id:ebi−fl187−GCST90094795' on 'Rheumatoid arthritis || id:finngen_R11_M13_R

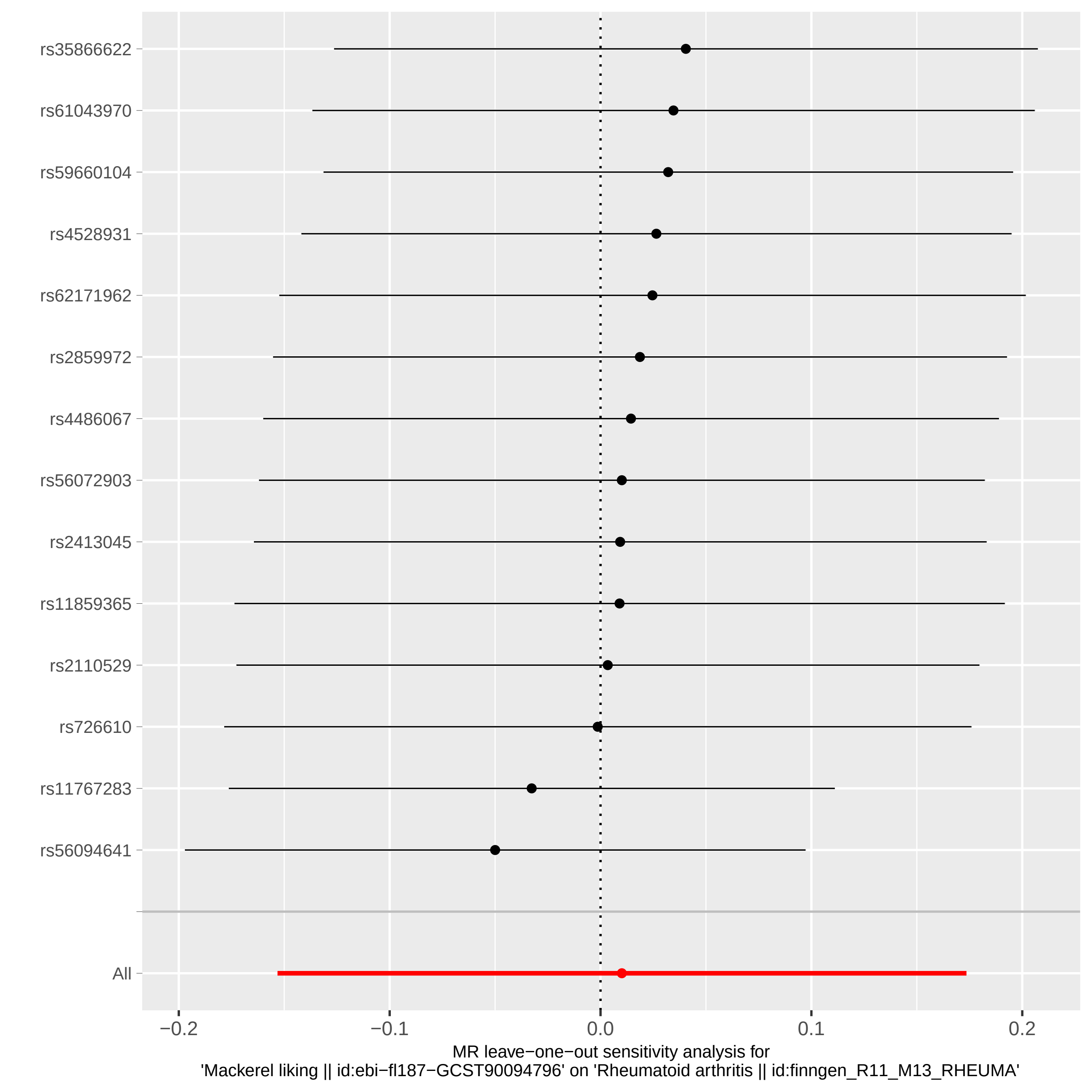
rs35866622
rs61043970
rs59660104
rs4528931
rs62171962
rs2859972
rs4486067
rs56072903
rs2413045
rs11859365
rs2110529
rs726610
rs11767283
rs56094641
All
−0.2
−0.1
0.0
0.1
0.2
MR leave−one−out sensitivity analysis for
'Mackerel liking || id:ebi−fl187−GCST90094796' on 'Rheumatoid arthritis || id:finngen_R11_M13_RHEUMA'

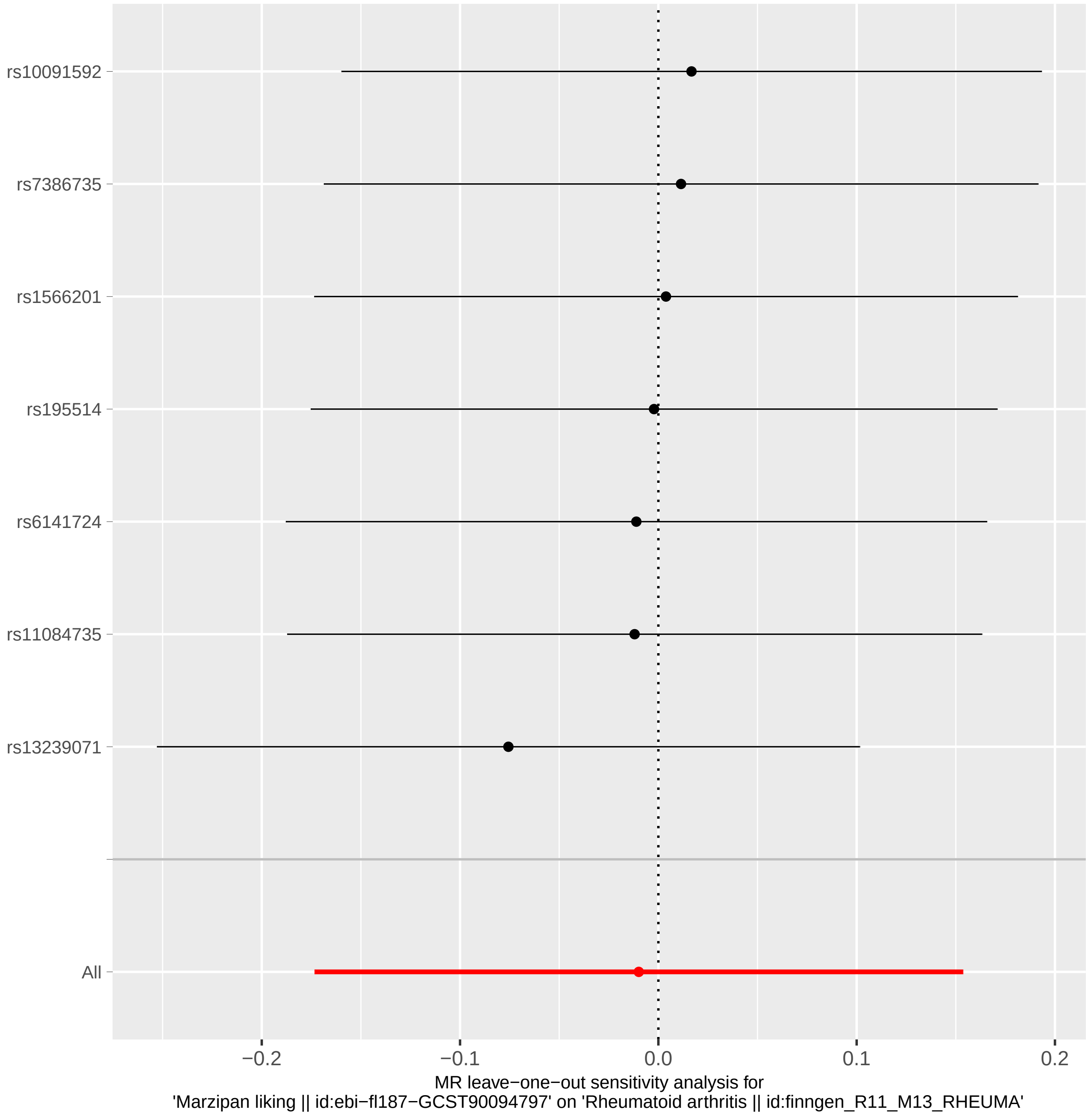
rs10091592
rs7386735
rs1566201
rs195514
rs6141724
rs11084735
rs13239071
All
−0.2
−0.1
0.0
0.1
0.2
MR leave−one−out sensitivity analysis for
'Marzipan liking || id:ebi−fl187−GCST90094797' on 'Rheumatoid arthritis || id:finngen_R11_M13_RHEUMA'

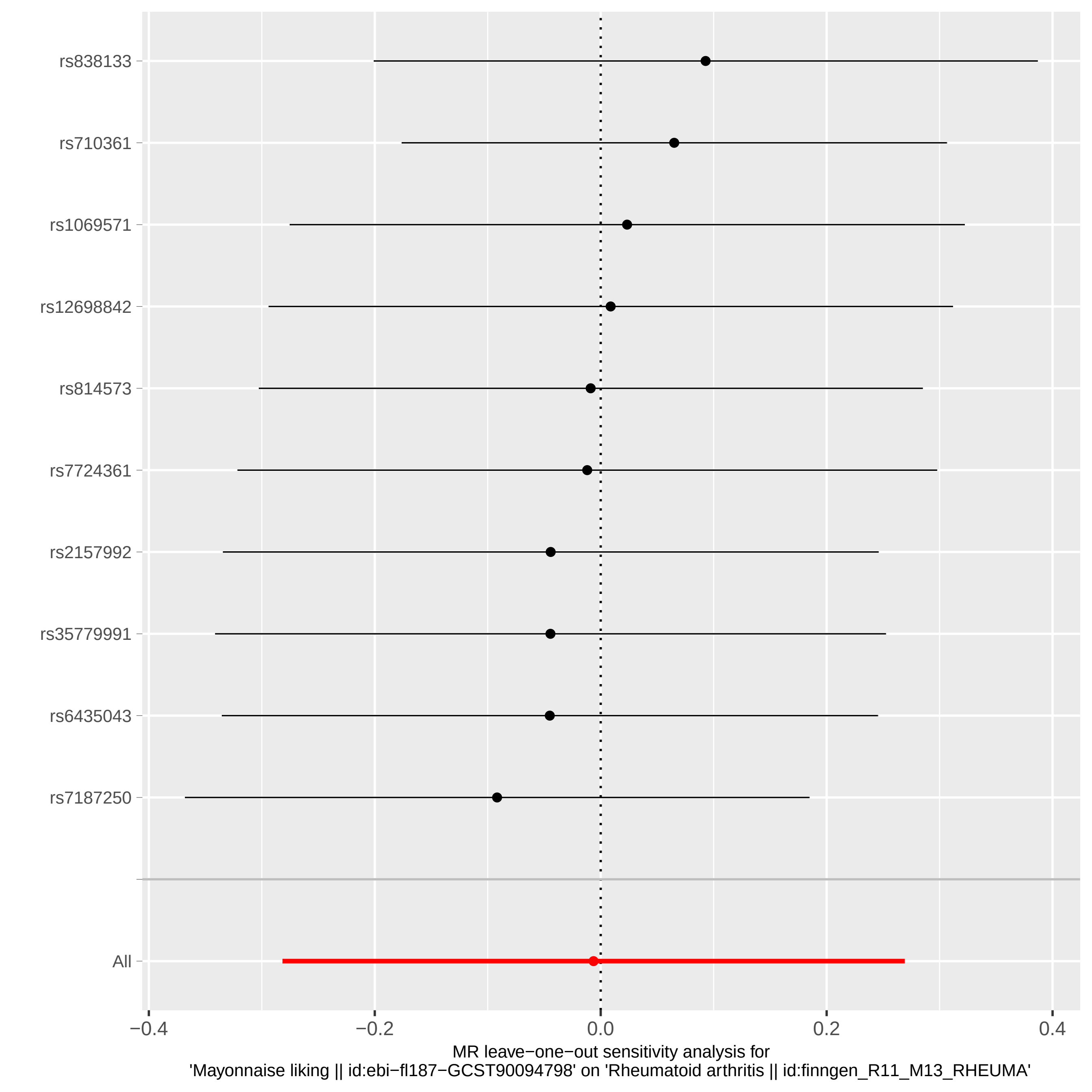
rs838133
rs710361
rs1069571
rs12698842
rs814573
rs7724361
rs2157992
rs35779991
rs6435043
rs7187250
All
−0.4
−0.2
0.0
0.2
0.4
MR leave−one−out sensitivity analysis for
'Mayonnaise liking || id:ebi−fl187−GCST90094798' on 'Rheumatoid arthritis || id:finngen_R11_M13_RHEUMA'

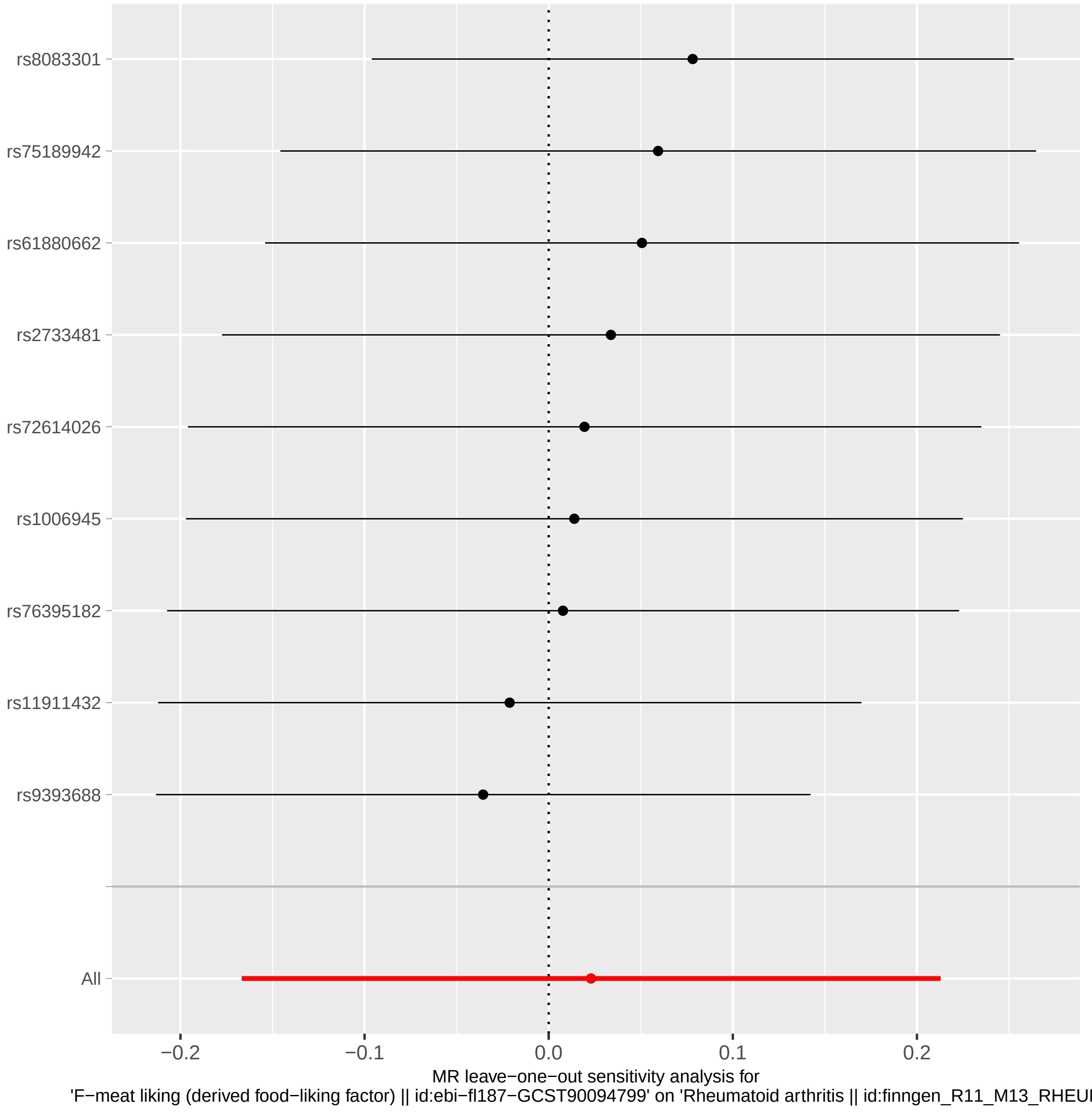
rs8083301
rs75189942
rs61880662
rs2733481
rs72614026
rs1006945
rs76395182
rs11911432
rs9393688
All
−0.2
−0.1
0.0
0.1
0.2
MR leave−one−out sensitivity analysis for
'F−meat liking (derived food−liking factor) || id:ebi−fl187−GCST90094799' on 'Rheumatoid arthritis || id:finngen_R11_M13_RHEU

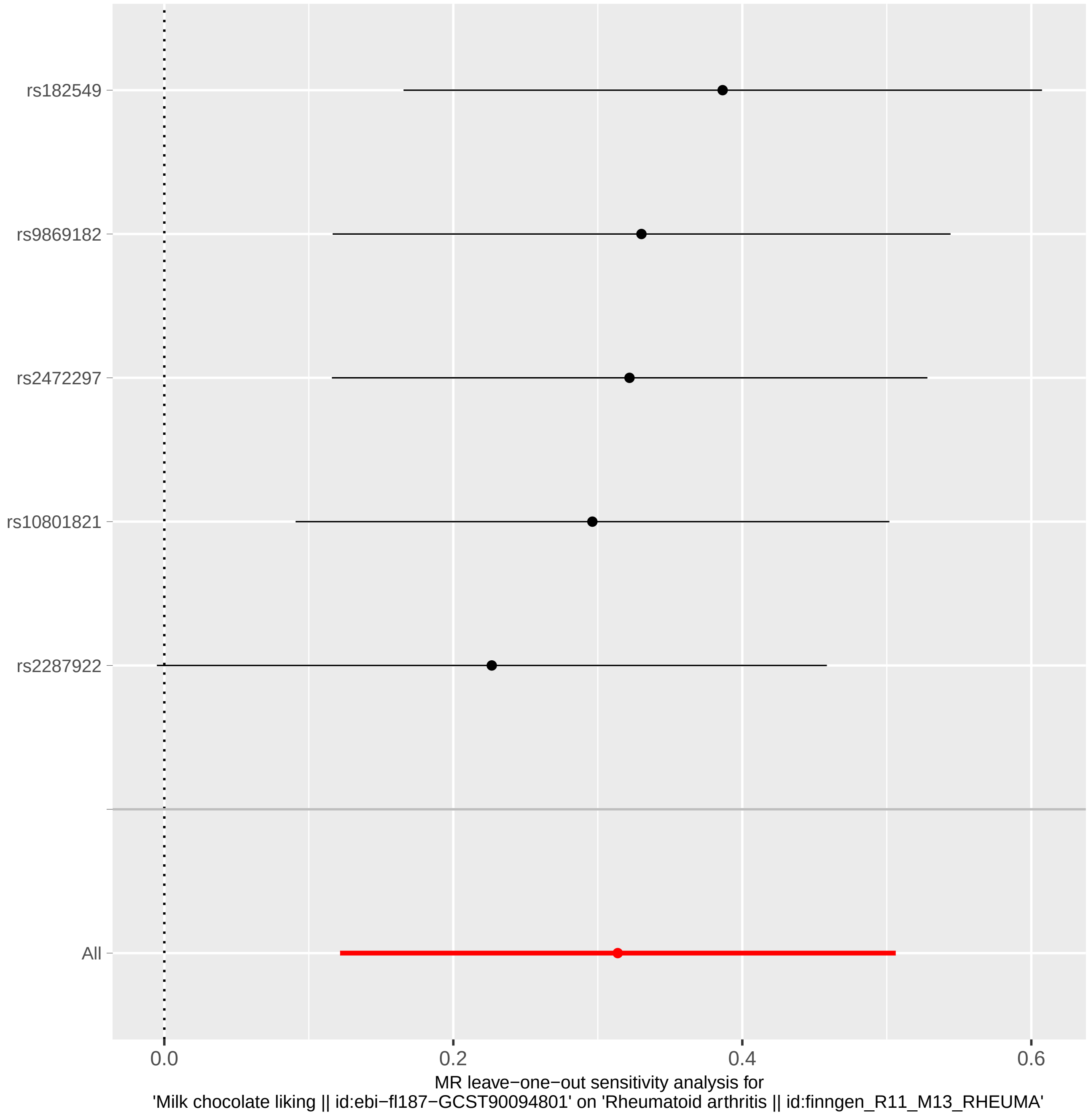
rs182549
rs9869182
rs2472297
rs10801821
rs2287922
All
0.0
0.2
0.4
0.6
MR leave−one−out sensitivity analysis for
'Milk chocolate liking || id:ebi−fl187−GCST90094801' on 'Rheumatoid arthritis || id:finngen_R11_M13_RHEUMA'

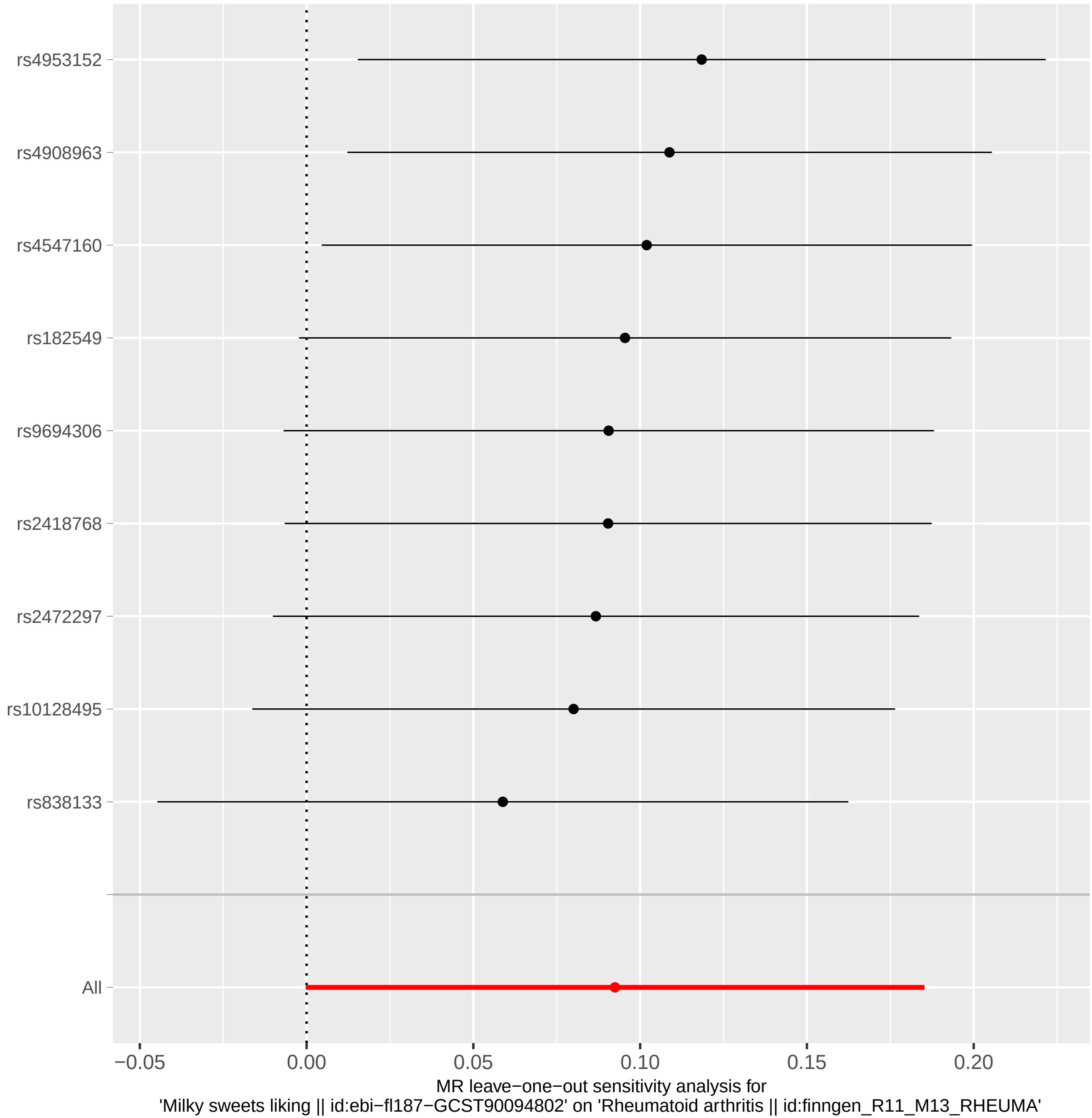
rs4953152
rs4908963
rs4547160
rs182549
rs9694306
rs2418768
rs2472297
rs10128495
rs838133
All
−0.05
0.00
0.05
0.10
0.15
0.20
MR leave−one−out sensitivity analysis for
'Milky sweets liking || id:ebi−fl187−GCST90094802' on 'Rheumatoid arthritis || id:finngen_R11_M13_RHEUMA'

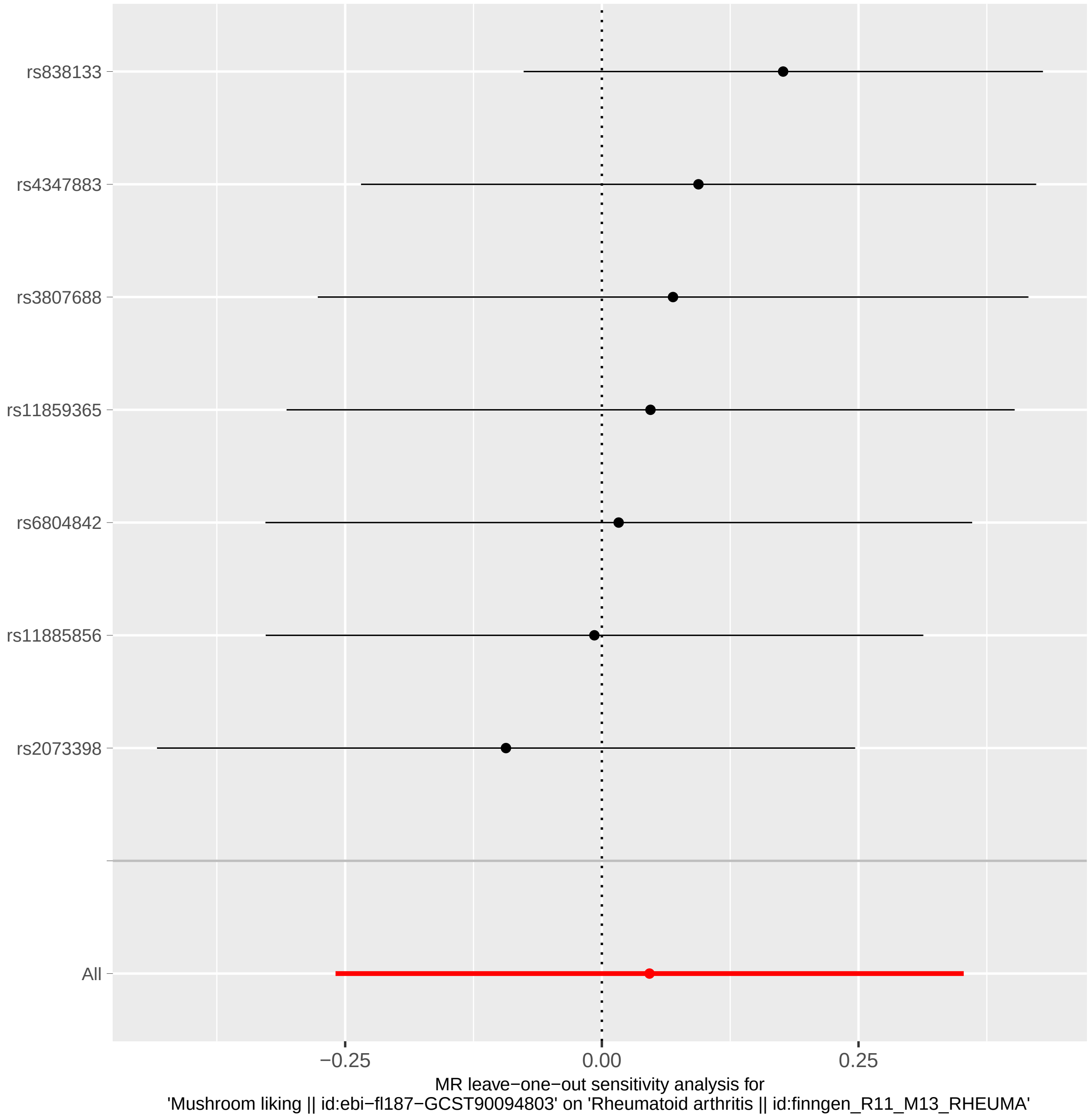
rs838133
rs4347883
rs3807688
rs11859365
rs6804842
rs11885856
rs2073398
All
−0.25
0.00
0.25
MR leave−one−out sensitivity analysis for
'Mushroom liking || id:ebi−fl187−GCST90094803' on 'Rheumatoid arthritis || id:finngen_R11_M13_RHEUMA'

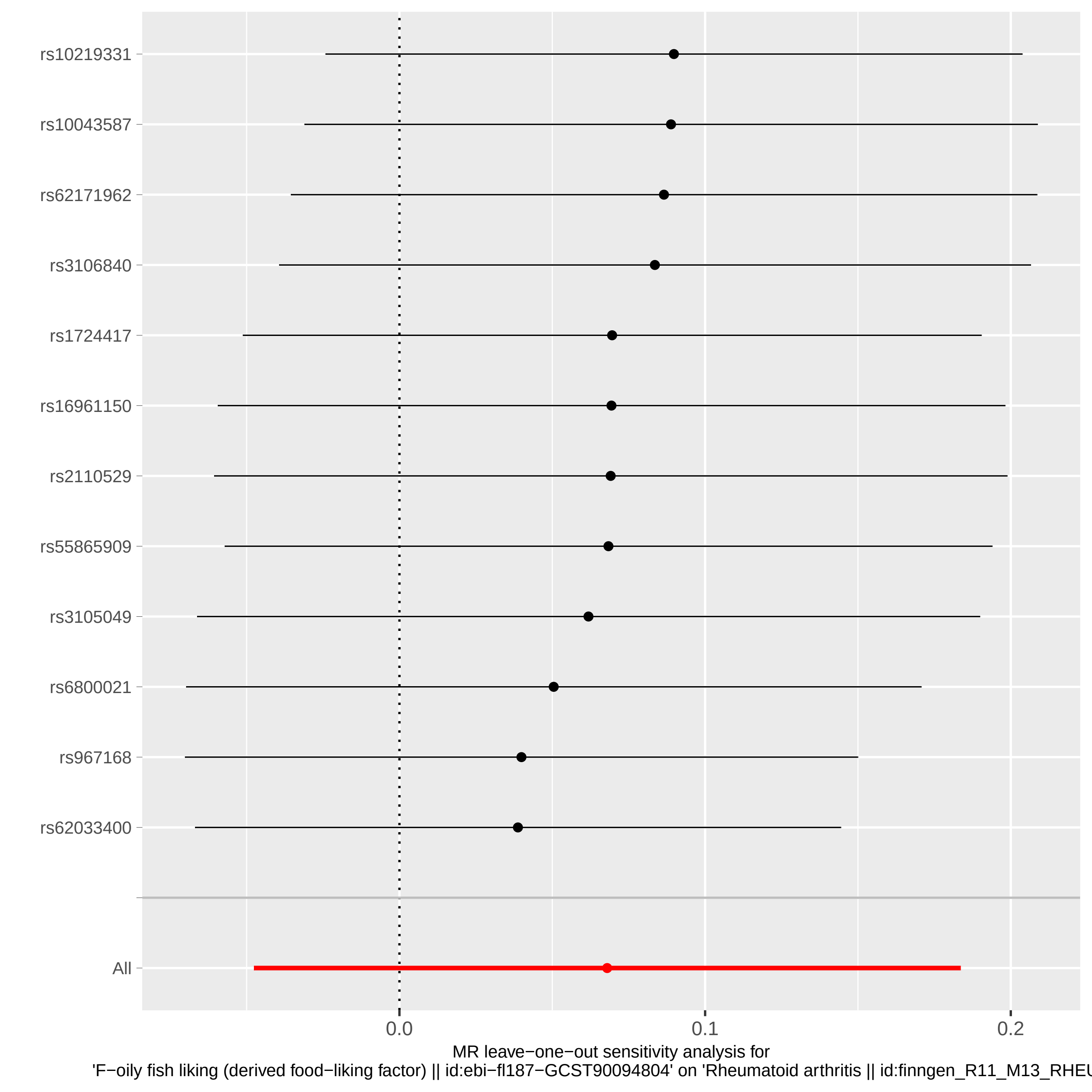
rs10219331
rs10043587
rs62171962
rs3106840
rs1724417
rs16961150
rs2110529
rs55865909
rs3105049
rs6800021
rs967168
rs62033400
All
0.0
0.1
0.2
MR leave−one−out sensitivity analysis for
'F−oily fish liking (derived food−liking factor) || id:ebi−fl187−GCST90094804' on 'Rheumatoid arthritis || id:finngen_R11_M13_RHEU

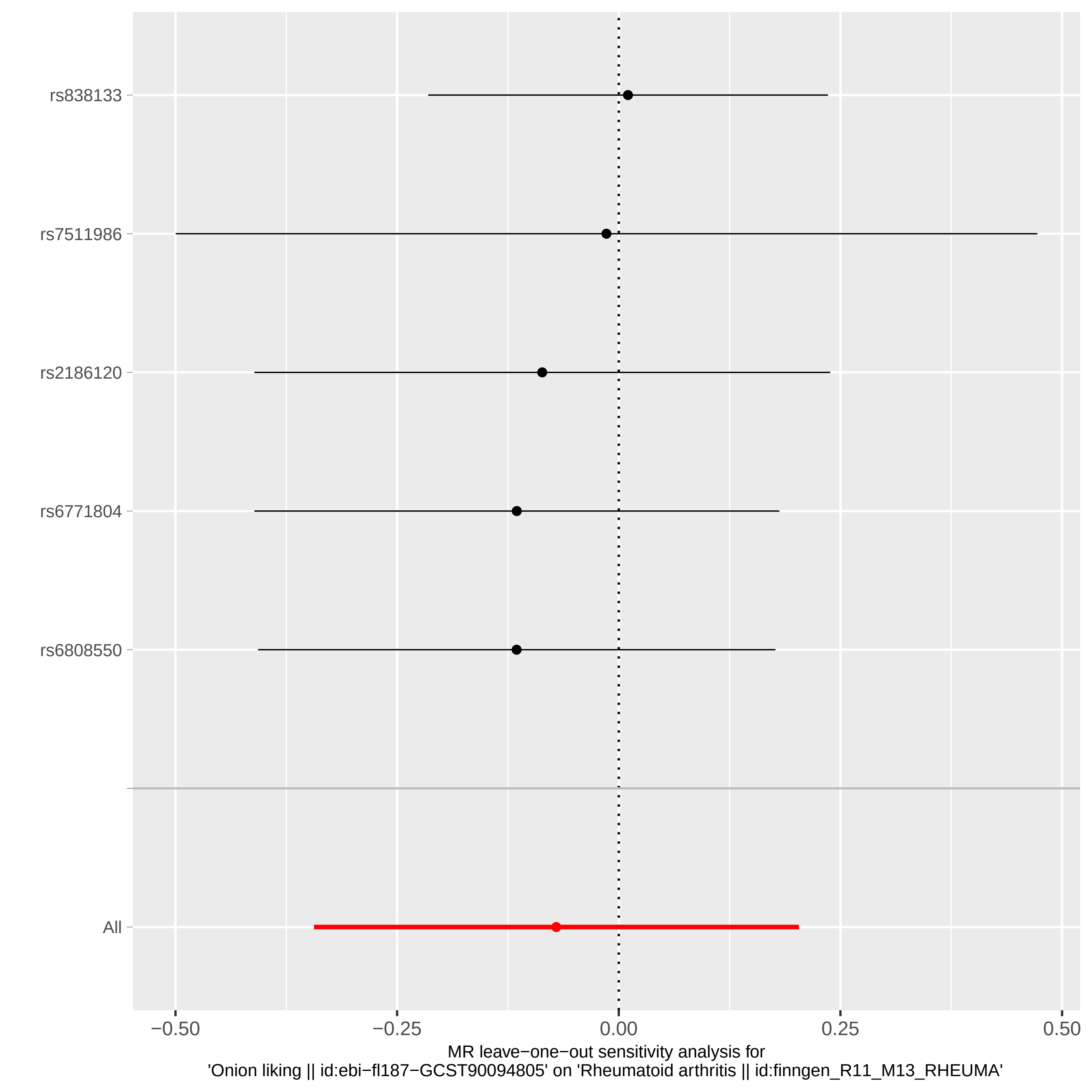
rs838133
rs7511986
rs2186120
rs6771804
rs6808550
All
−0.50
−0.25
0.00
0.25
0.50
MR leave−one−out sensitivity analysis for
'Onion liking || id:ebi−fl187−GCST90094805' on 'Rheumatoid arthritis || id:finngen_R11_M13_RHEUMA'

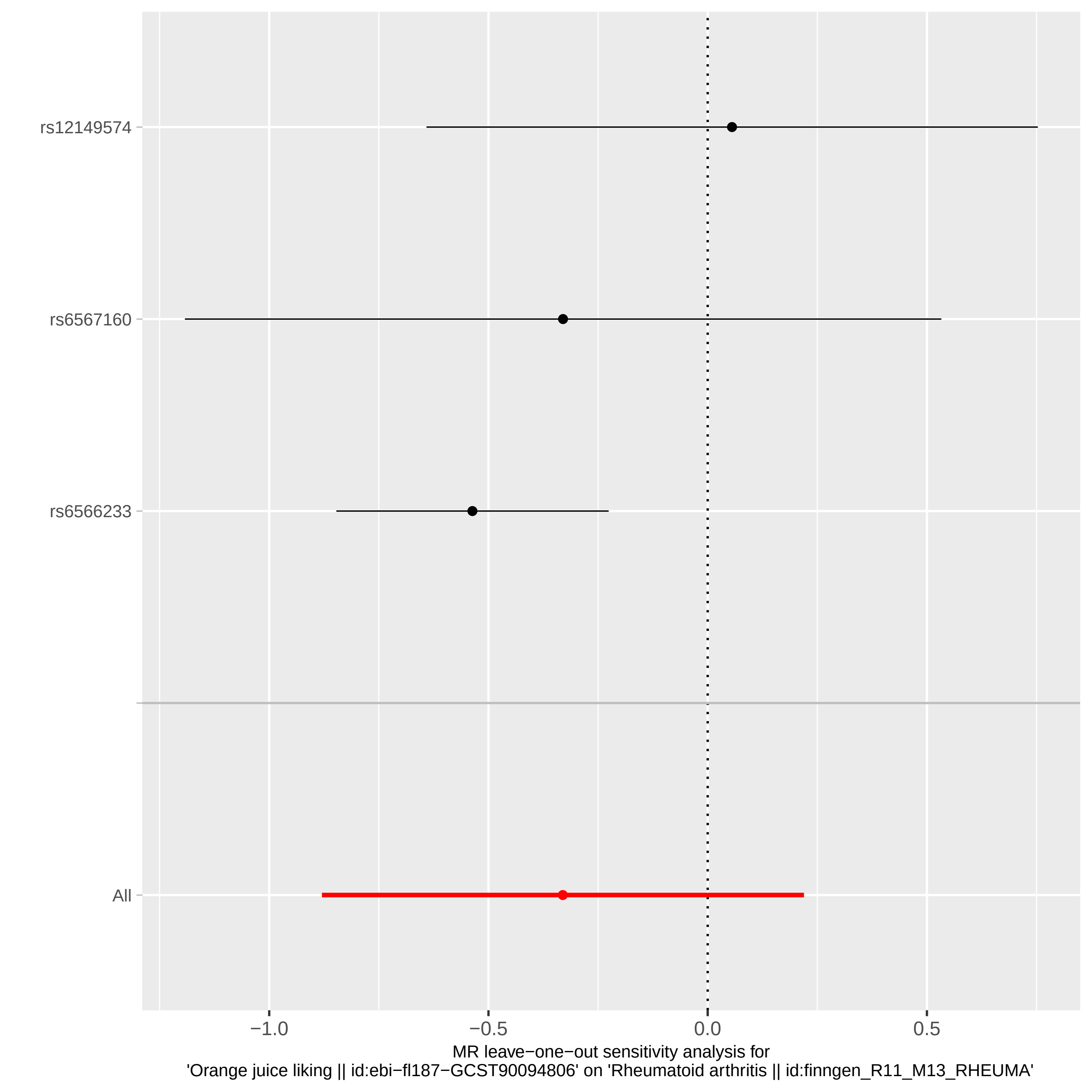
rs12149574
rs6567160
rs6566233
All
−1.0
−0.5
0.0
0.5
MR leave−one−out sensitivity analysis for
'Orange juice liking || id:ebi−fl187−GCST90094806' on 'Rheumatoid arthritis || id:finngen_R11_M13_RHEUMA'

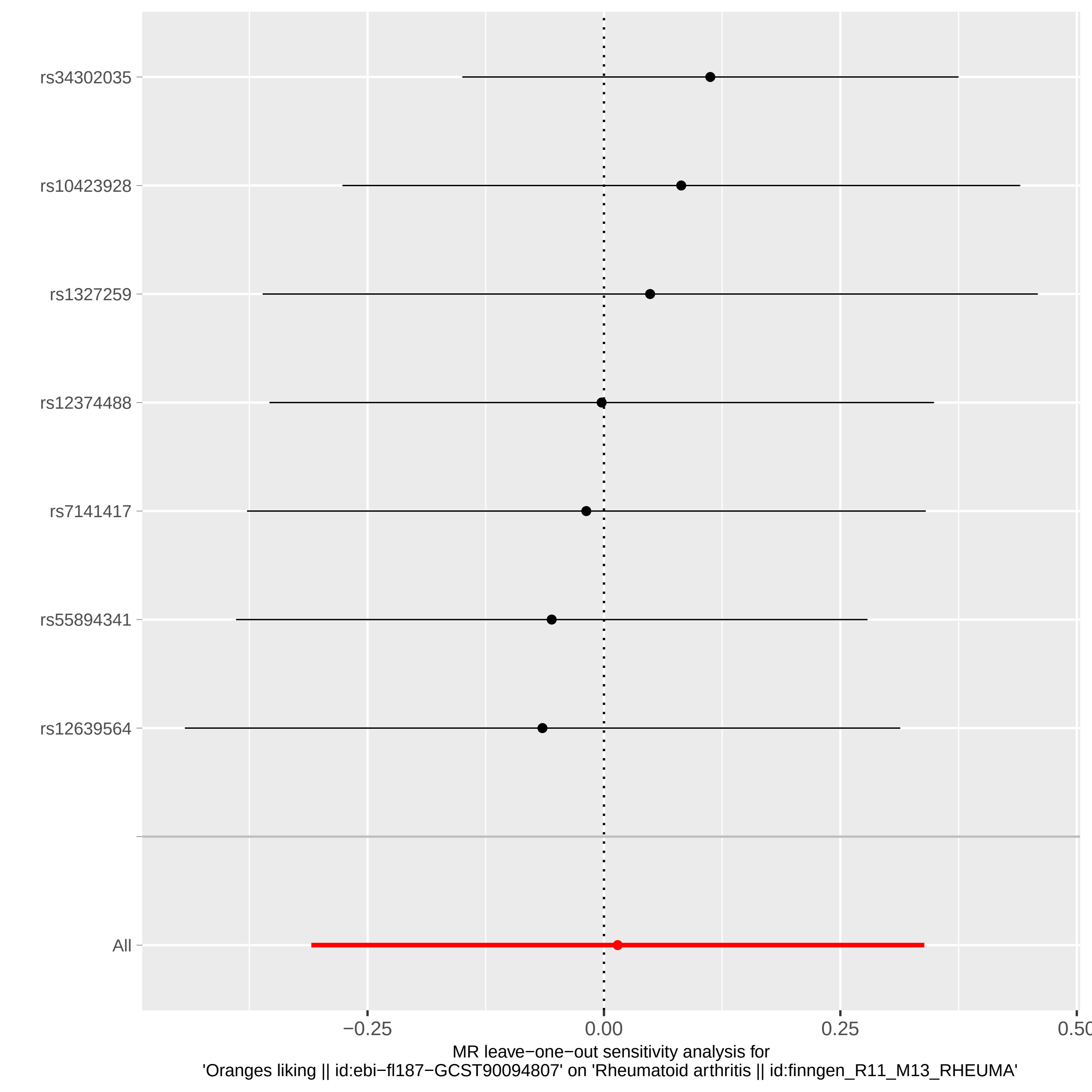
rs34302035
rs10423928
rs1327259
rs12374488
rs7141417
rs55894341
rs12639564
All
−0.25
0.00
0.25
0.50
MR leave−one−out sensitivity analysis for
'Oranges liking || id:ebi−fl187−GCST90094807' on 'Rheumatoid arthritis || id:finngen_R11_M13_RHEUMA'

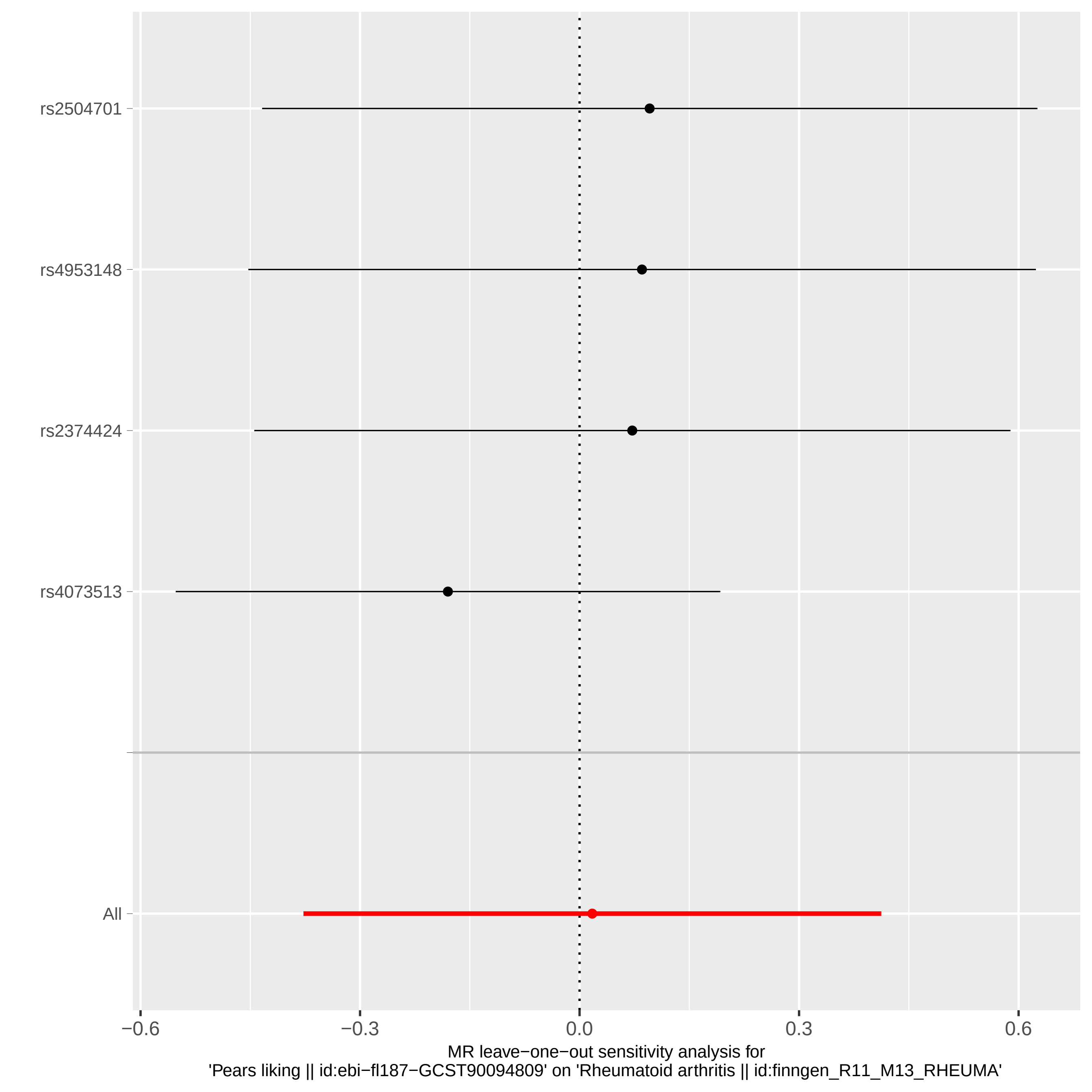
rs2504701
rs4953148
rs2374424
rs4073513
All
−0.6
−0.3
0.0
0.3
0.6
MR leave−one−out sensitivity analysis for
'Pears liking || id:ebi−fl187−GCST90094809' on 'Rheumatoid arthritis || id:finngen_R11_M13_RHEUMA'

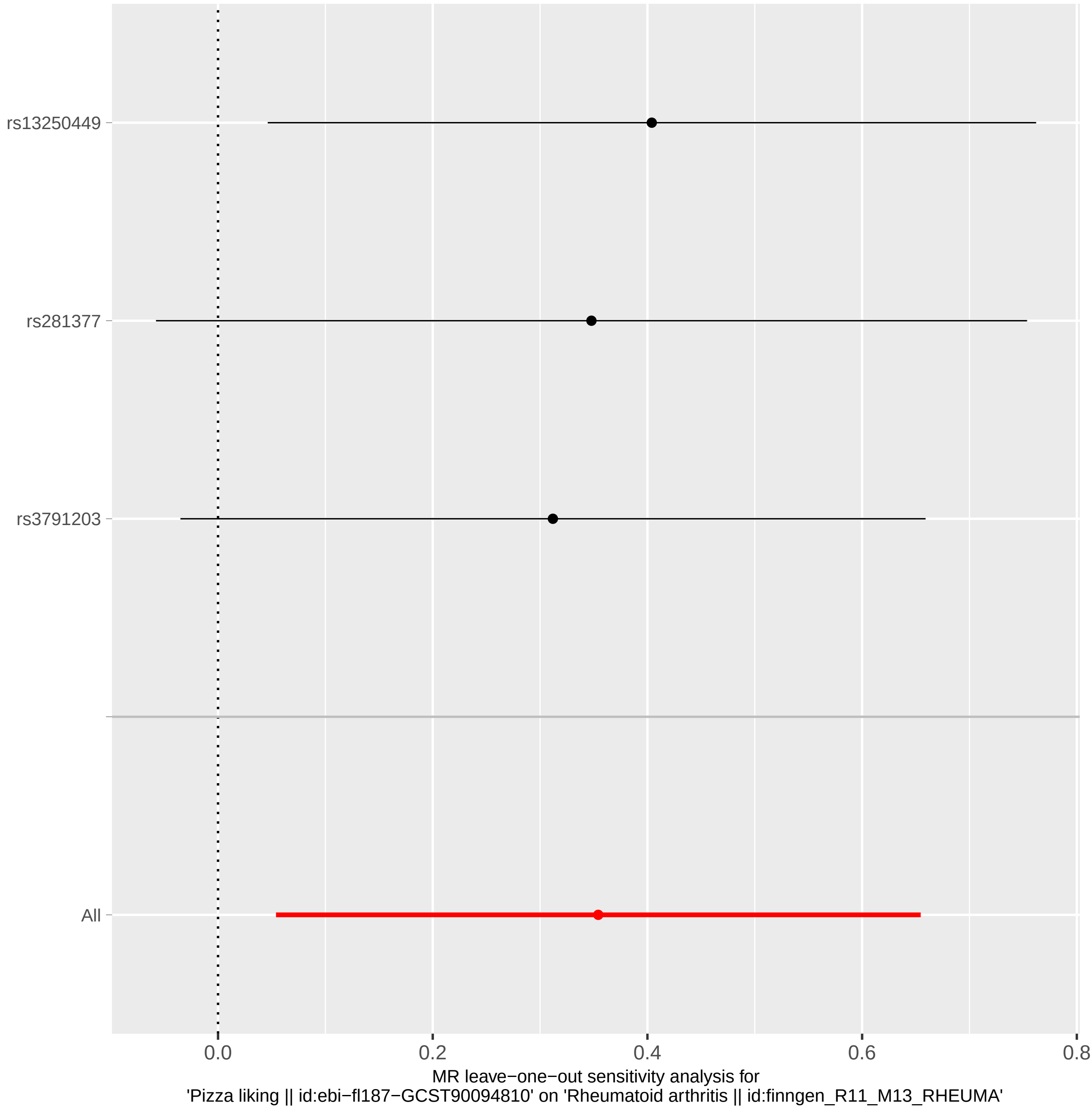
rs13250449
rs281377
rs3791203
All
0.0
0.2
0.4
0.6
0.8
MR leave−one−out sensitivity analysis for
'Pizza liking || id:ebi−fl187−GCST90094810' on 'Rheumatoid arthritis || id:finngen_R11_M13_RHEUMA'

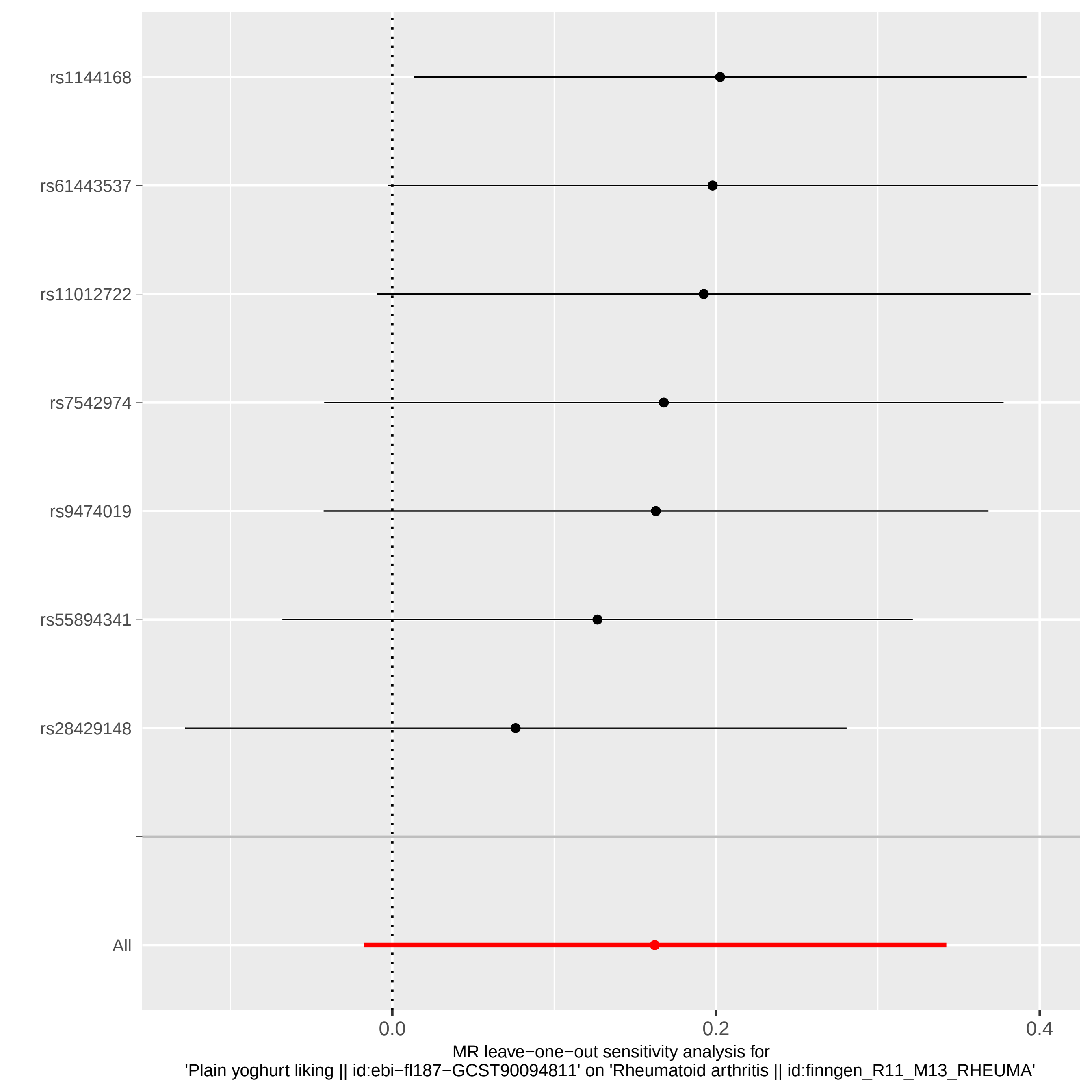
rs1144168
rs61443537
rs11012722
rs7542974
rs9474019
rs55894341
rs28429148
All
0.0
0.2
0.4
MR leave−one−out sensitivity analysis for
'Plain yoghurt liking || id:ebi−fl187−GCST90094811' on 'Rheumatoid arthritis || id:finngen_R11_M13_RHEUMA'

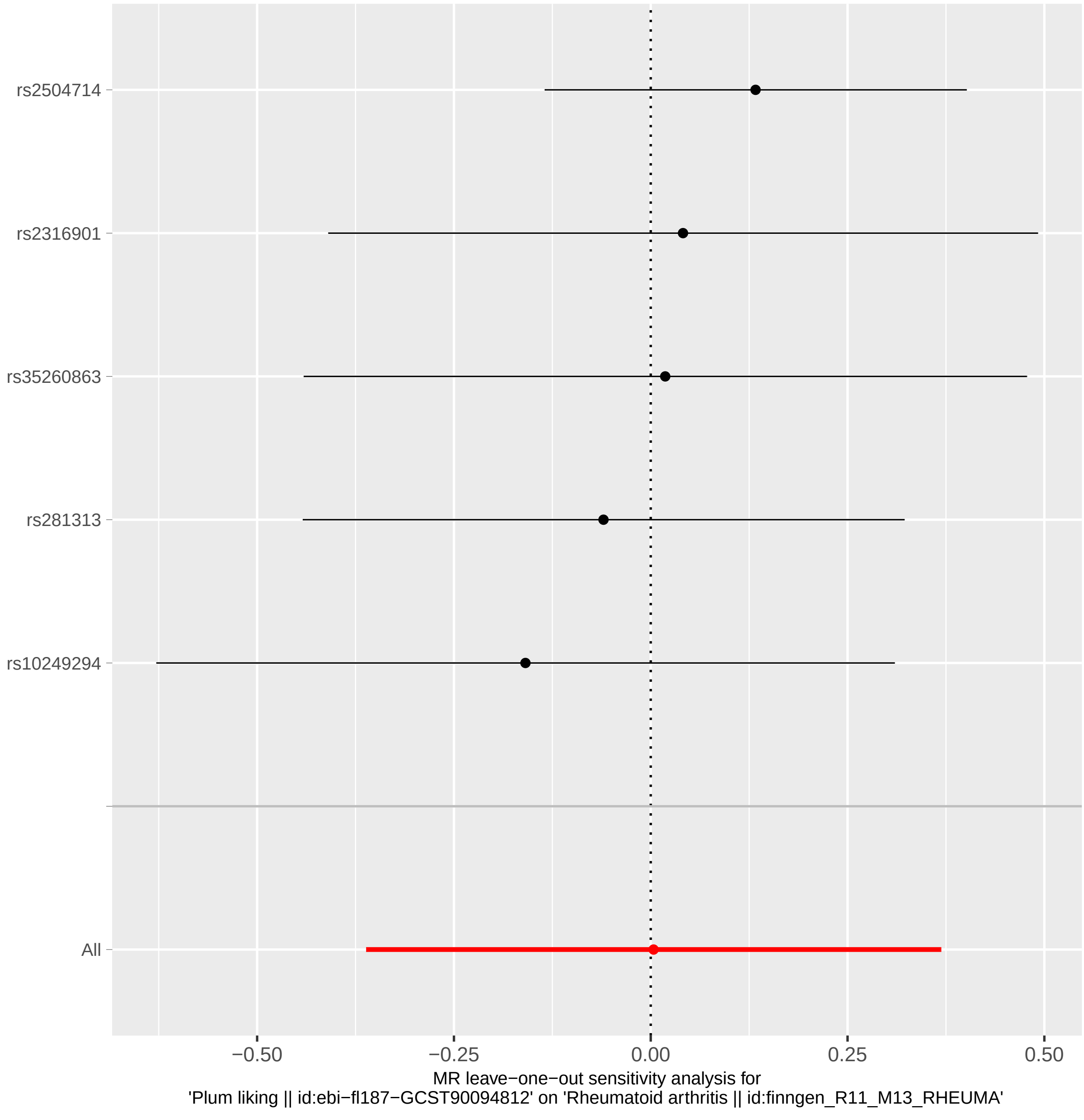
rs2504714
rs2316901
rs35260863
rs281313
rs10249294
All
−0.50
−0.25
0.00
0.25
0.50
MR leave−one−out sensitivity analysis for
'Plum liking || id:ebi−fl187−GCST90094812' on 'Rheumatoid arthritis || id:finngen_R11_M13_RHEUMA'

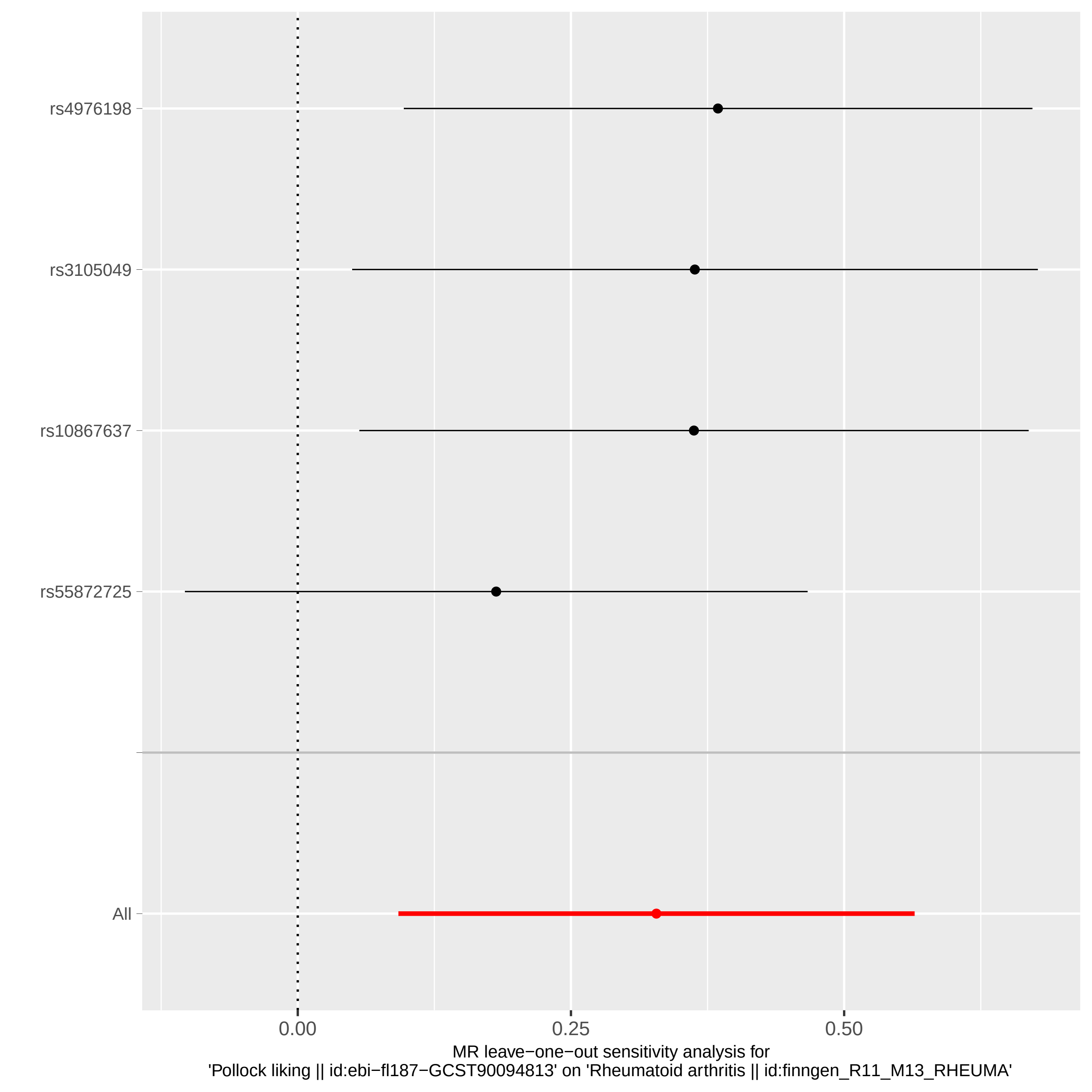
rs4976198
rs3105049
rs10867637
rs55872725
All
0.00
0.25
0.50
MR leave−one−out sensitivity analysis for
'Pollock liking || id:ebi−fl187−GCST90094813' on 'Rheumatoid arthritis || id:finngen_R11_M13_RHEUMA'

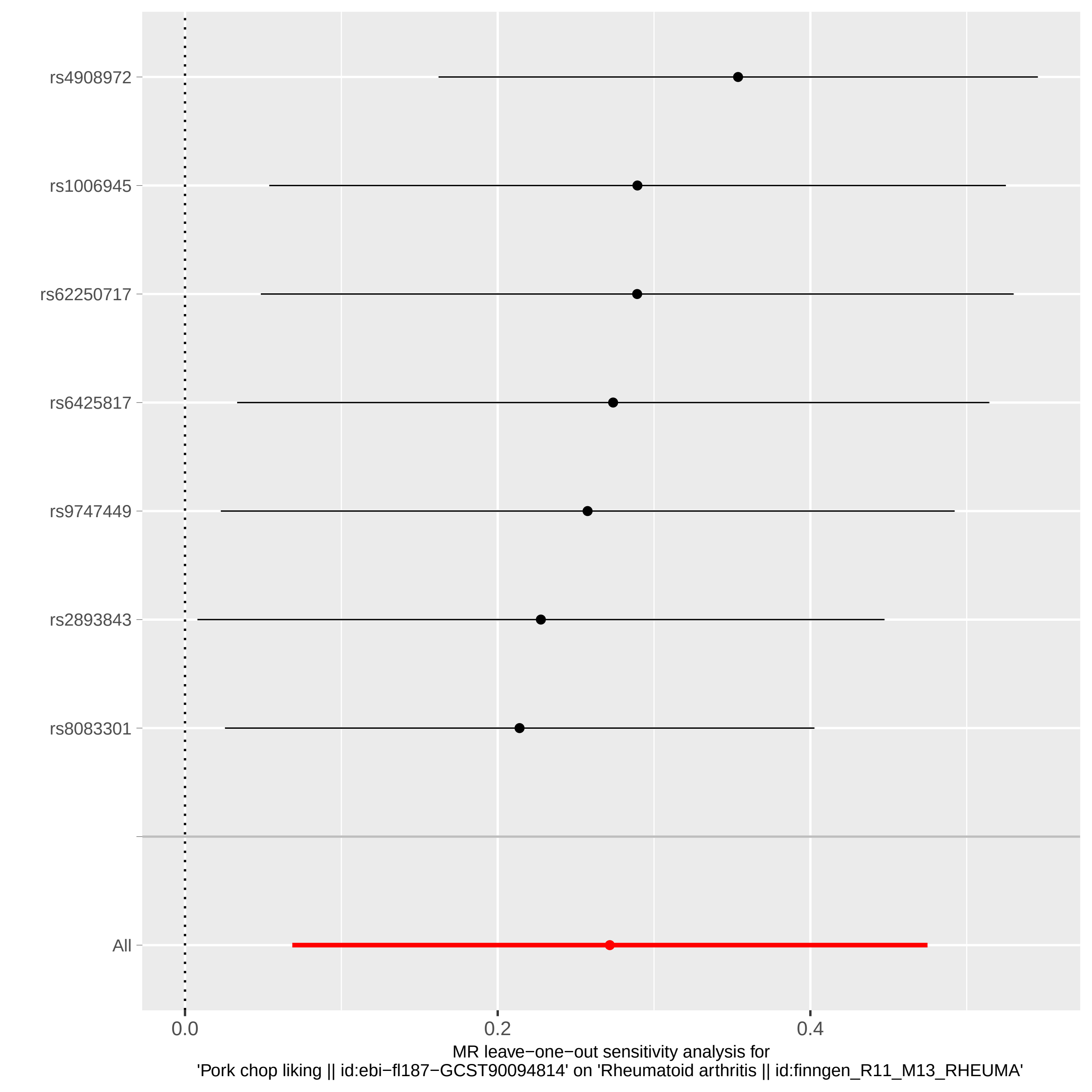
rs4908972
rs1006945
rs62250717
rs6425817
rs9747449
rs2893843
rs8083301
All
0.0
0.2
0.4
MR leave−one−out sensitivity analysis for
'Pork chop liking || id:ebi−fl187−GCST90094814' on 'Rheumatoid arthritis || id:finngen_R11_M13_RHEUMA'

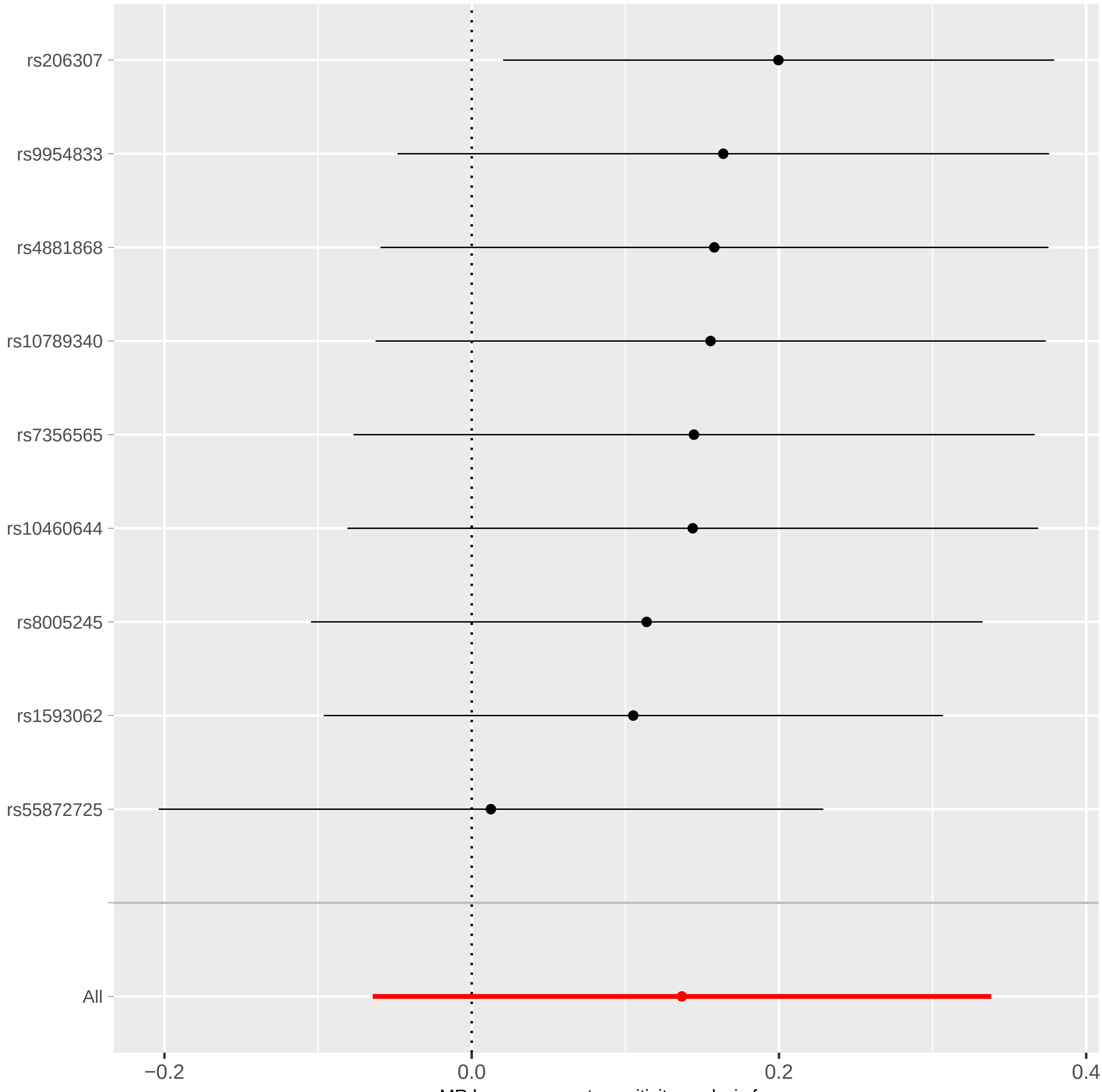

MR leave−one−out sensitivity analysis for
'Porridge liking || id:ebi−fl187−GCST90094815' on 'Rheumatoid arthritis || id:finngen_R11_M13_RHEUMA'

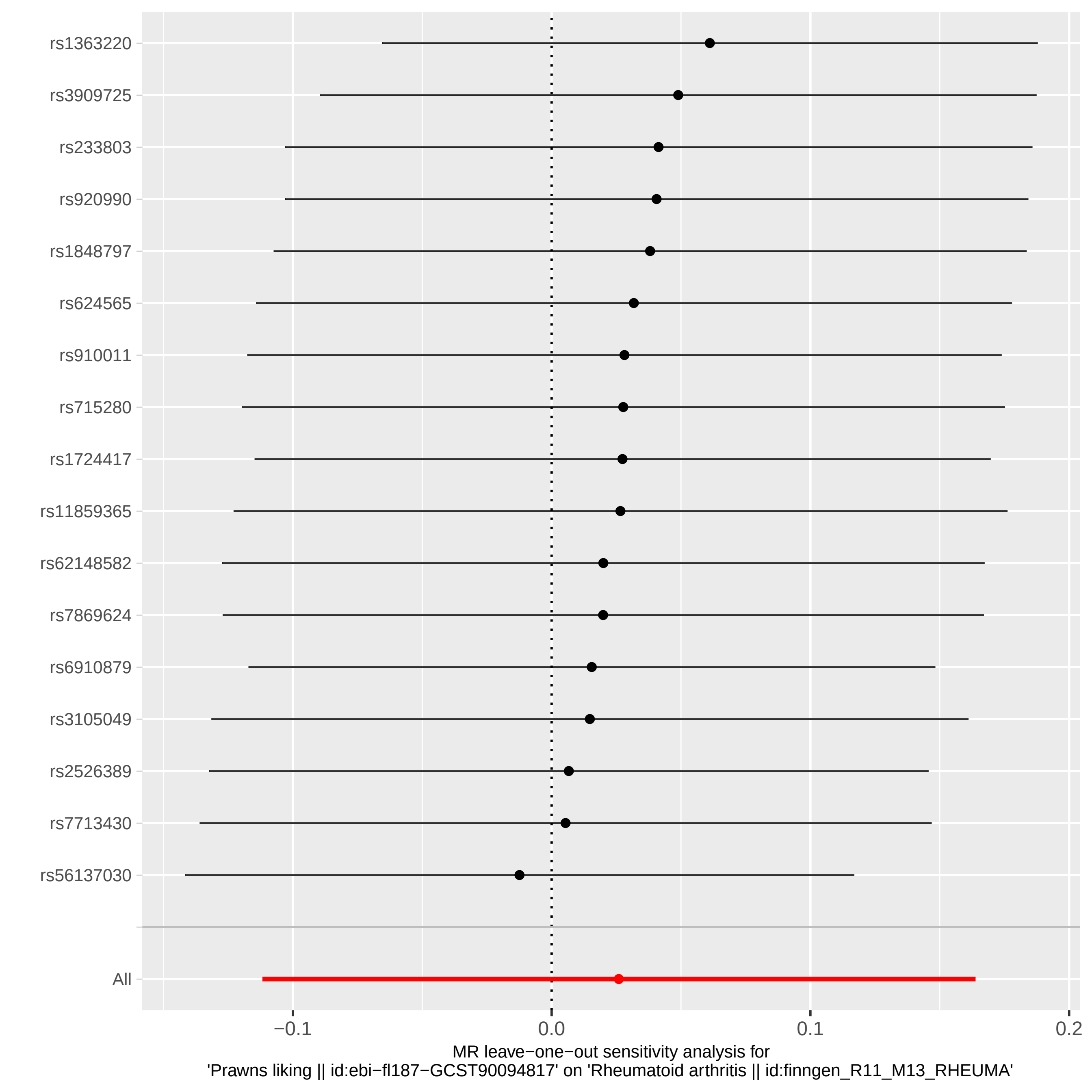
rs1363220
rs3909725
rs233803
rs920990
rs1848797
rs624565
rs910011
rs715280
rs1724417
rs11859365
rs62148582
rs7869624
rs6910879
rs3105049
rs2526389
rs7713430
rs56137030
All
−0.1
0.0
0.1
0.2
MR leave−one−out sensitivity analysis for
'Prawns liking || id:ebi−fl187−GCST90094817' on 'Rheumatoid arthritis || id:finngen_R11_M13_RHEUMA'

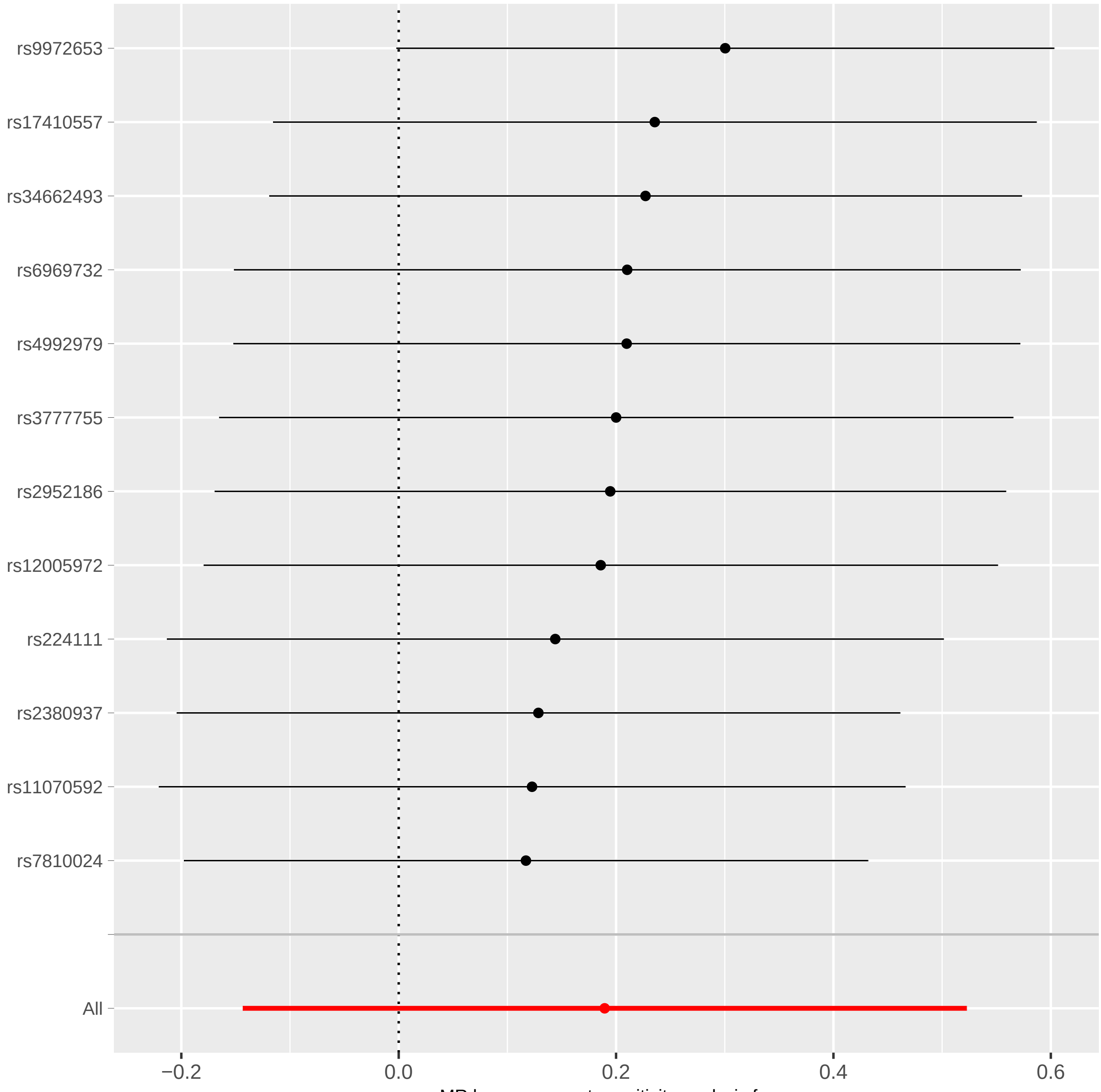

MR leave−one−out sensitivity analysis for
'Raw carrot liking || id:ebi−fl187−GCST90094818' on 'Rheumatoid arthritis || id:finngen_R11_M13_RHEUMA'

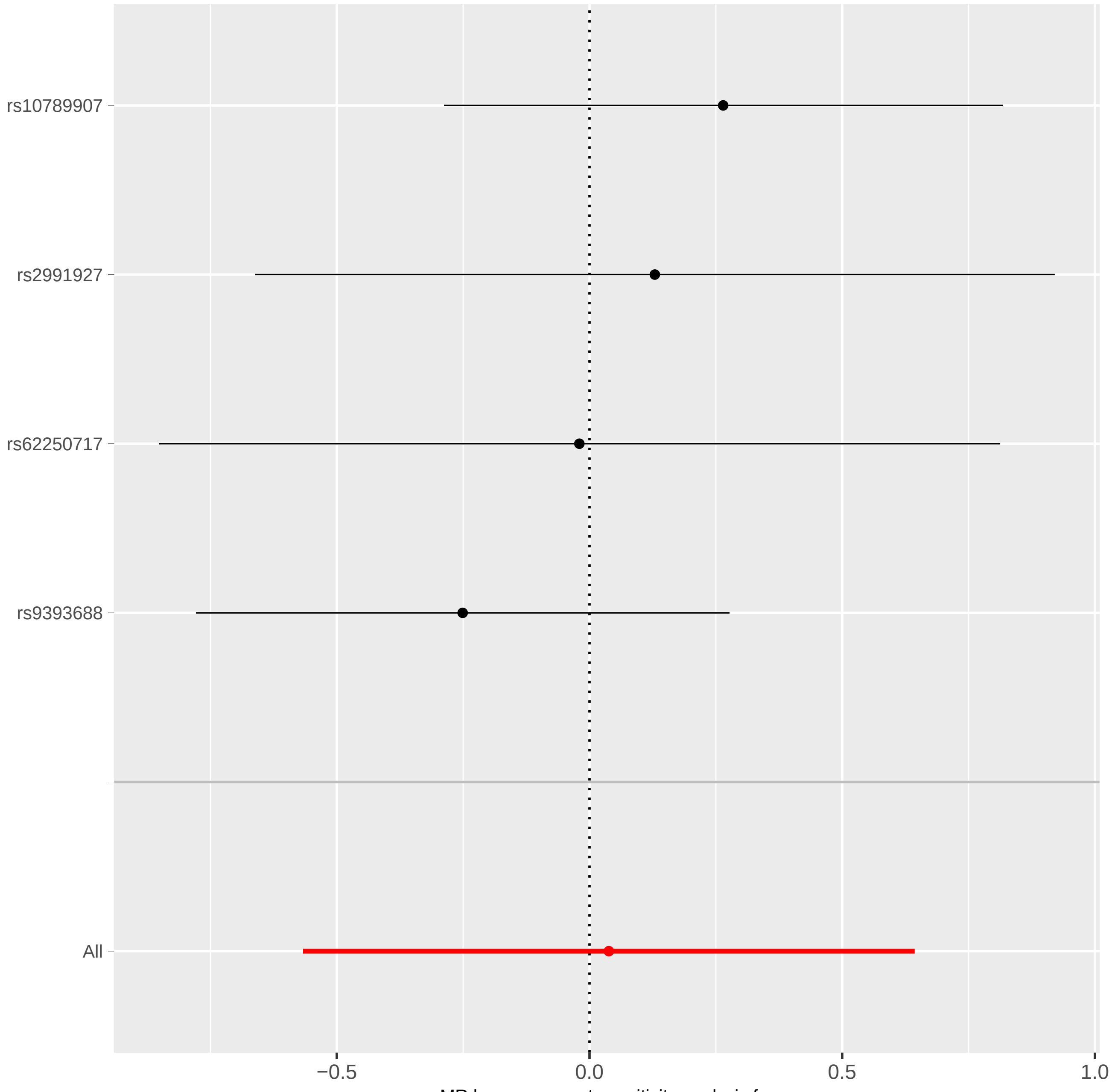

MR leave−one−out sensitivity analysis for
'Red meat liking || id:ebi−fl187−GCST90094819' on 'Rheumatoid arthritis || id:finngen_R11_M13_RHEUMA'

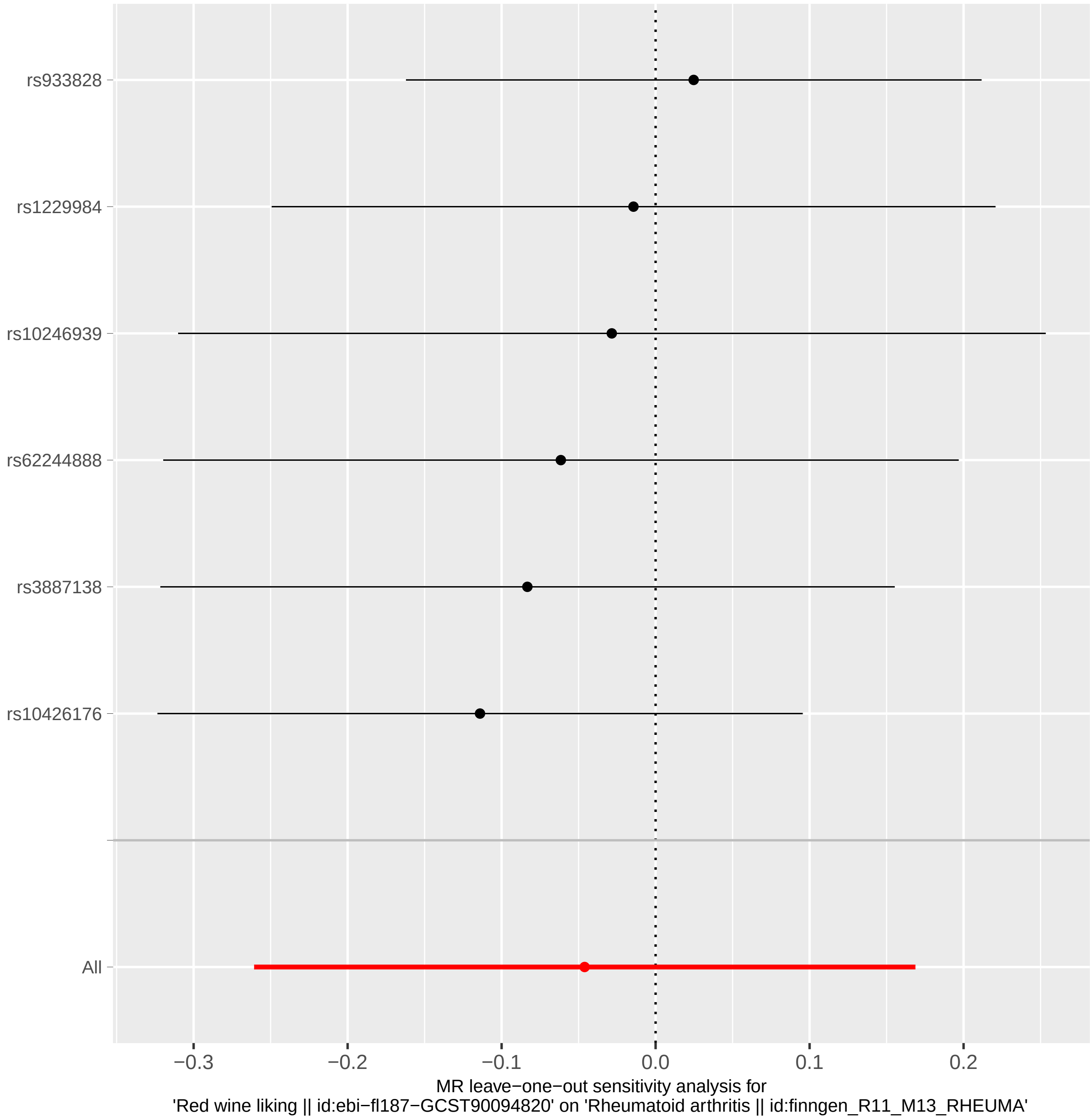
rs933828
rs1229984
rs10246939
rs62244888
rs3887138
rs10426176
All
−0.3
−0.2
−0.1
0.0
0.1
0.2
MR leave−one−out sensitivity analysis for
'Red wine liking || id:ebi−fl187−GCST90094820' on 'Rheumatoid arthritis || id:finngen_R11_M13_RHEUMA'

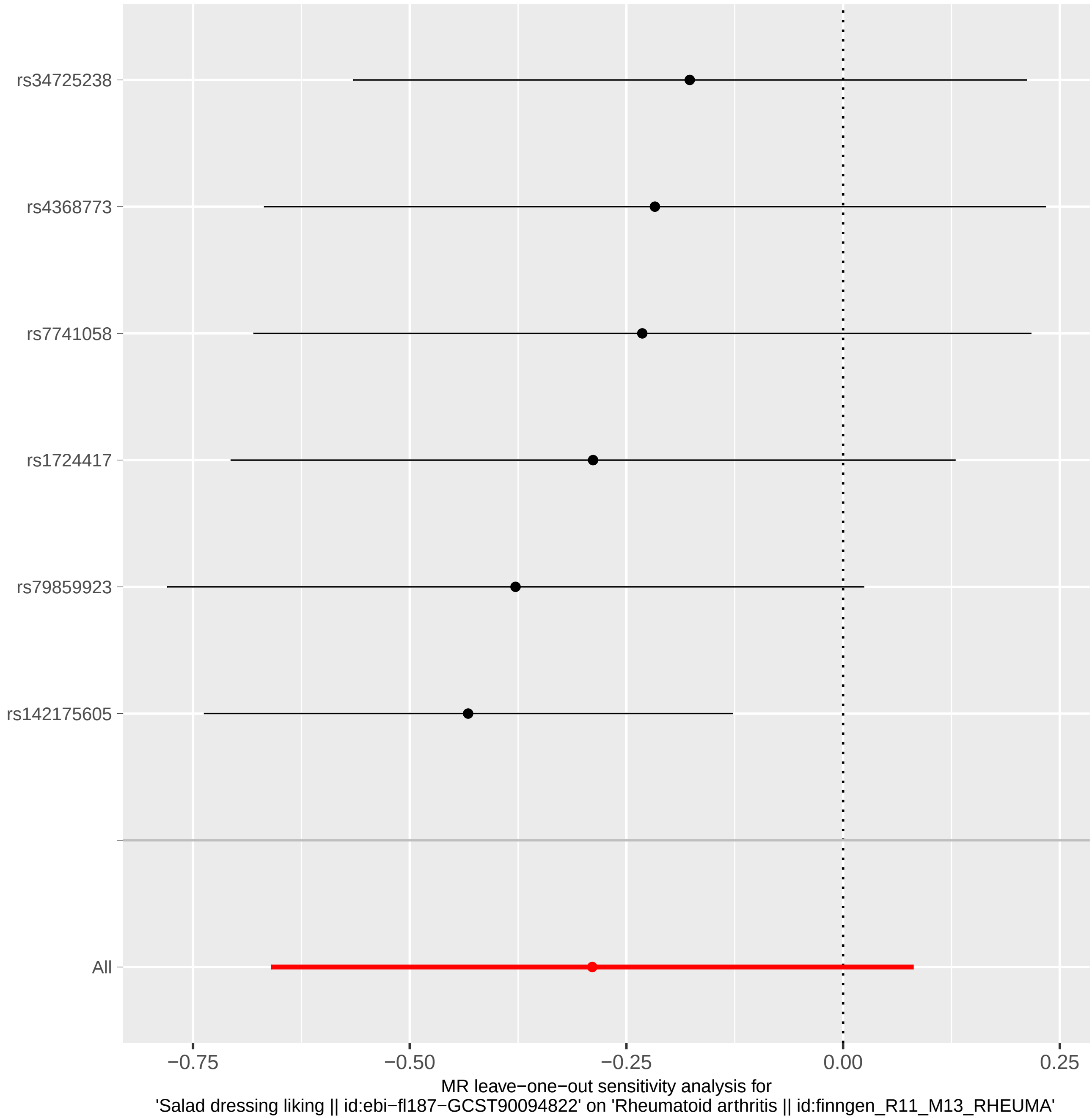
rs34725238
rs4368773
rs7741058
rs1724417
rs79859923
rs142175605
All
−0.75
−0.50
−0.25
0.00
0.25
MR leave−one−out sensitivity analysis for
'Salad dressing liking || id:ebi−fl187−GCST90094822' on 'Rheumatoid arthritis || id:finngen_R11_M13_RHEUMA'

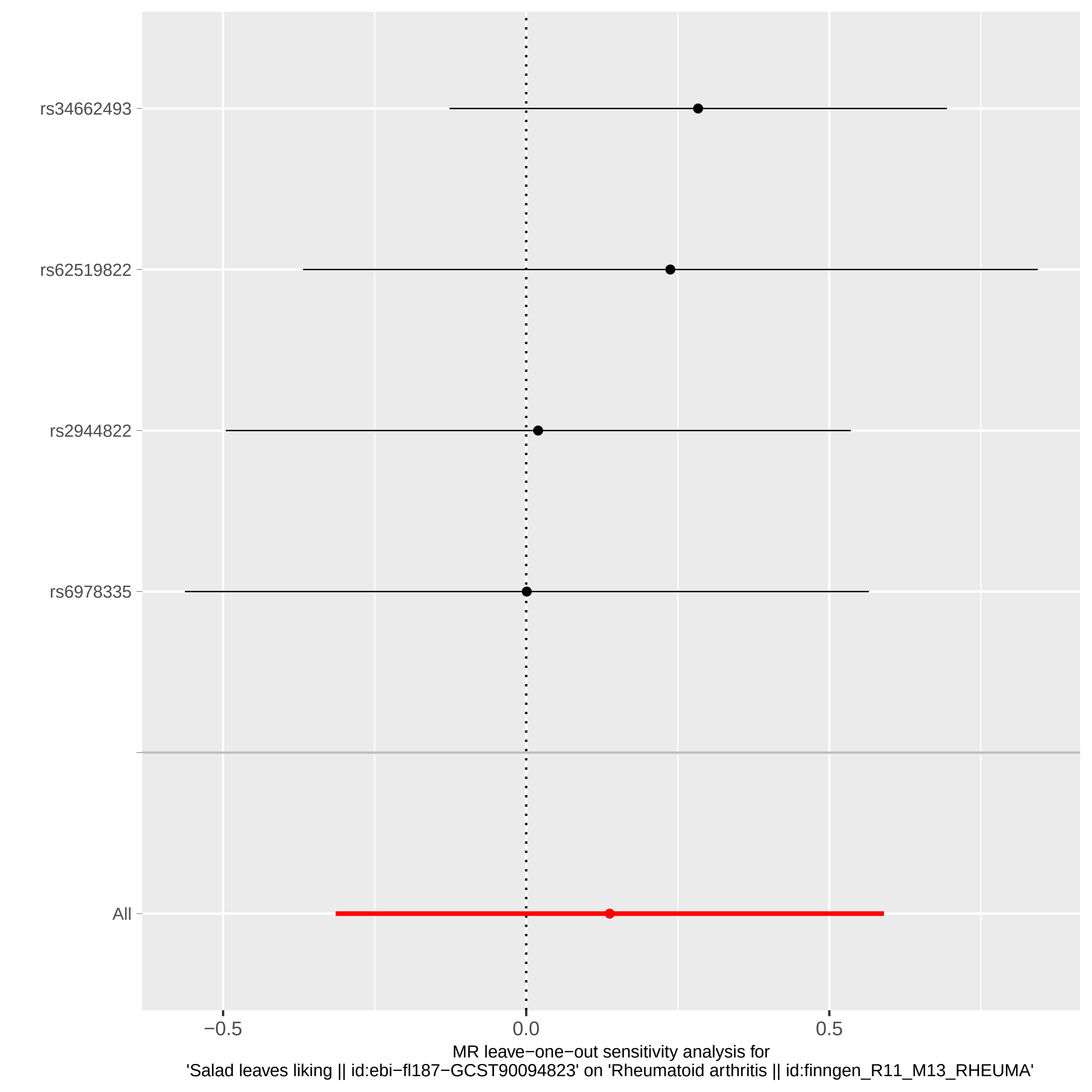
rs34662493
rs62519822
rs2944822
rs6978335
All
−0.5
0.0
0.5
MR leave−one−out sensitivity analysis for
'Salad leaves liking || id:ebi−fl187−GCST90094823' on 'Rheumatoid arthritis || id:finngen_R11_M13_RHEUMA'

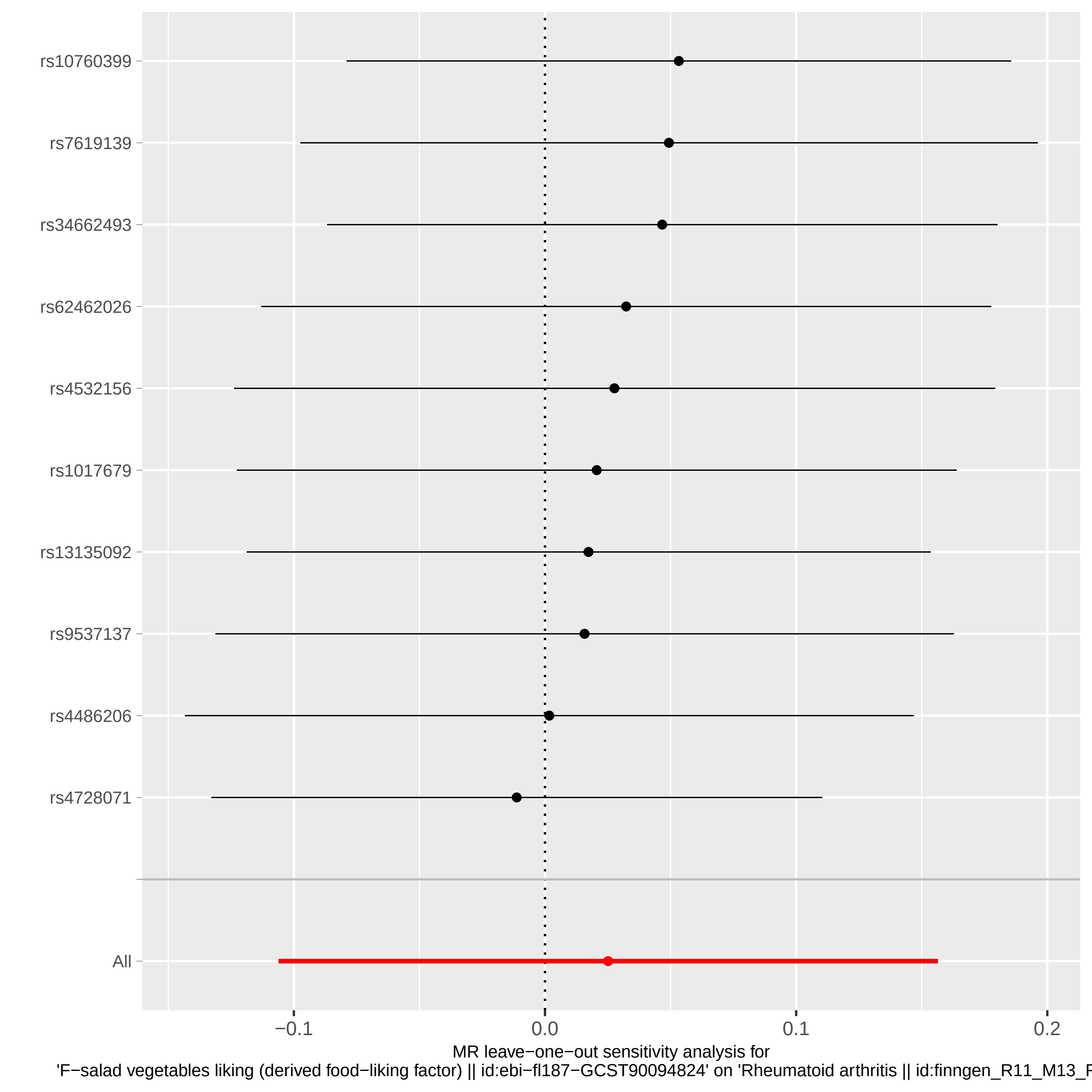
rs10760399
rs7619139
rs34662493
rs62462026
rs4532156
rs1017679
rs13135092
rs9537137
rs4486206
rs4728071
All
−0.1
0.0
0.1
0.2
MR leave−one−out sensitivity analysis for
'F−salad vegetables liking (derived food−liking factor) || id:ebi−fl187−GCST90094824' on 'Rheumatoid arthritis || id:finngen_R11_M13_F

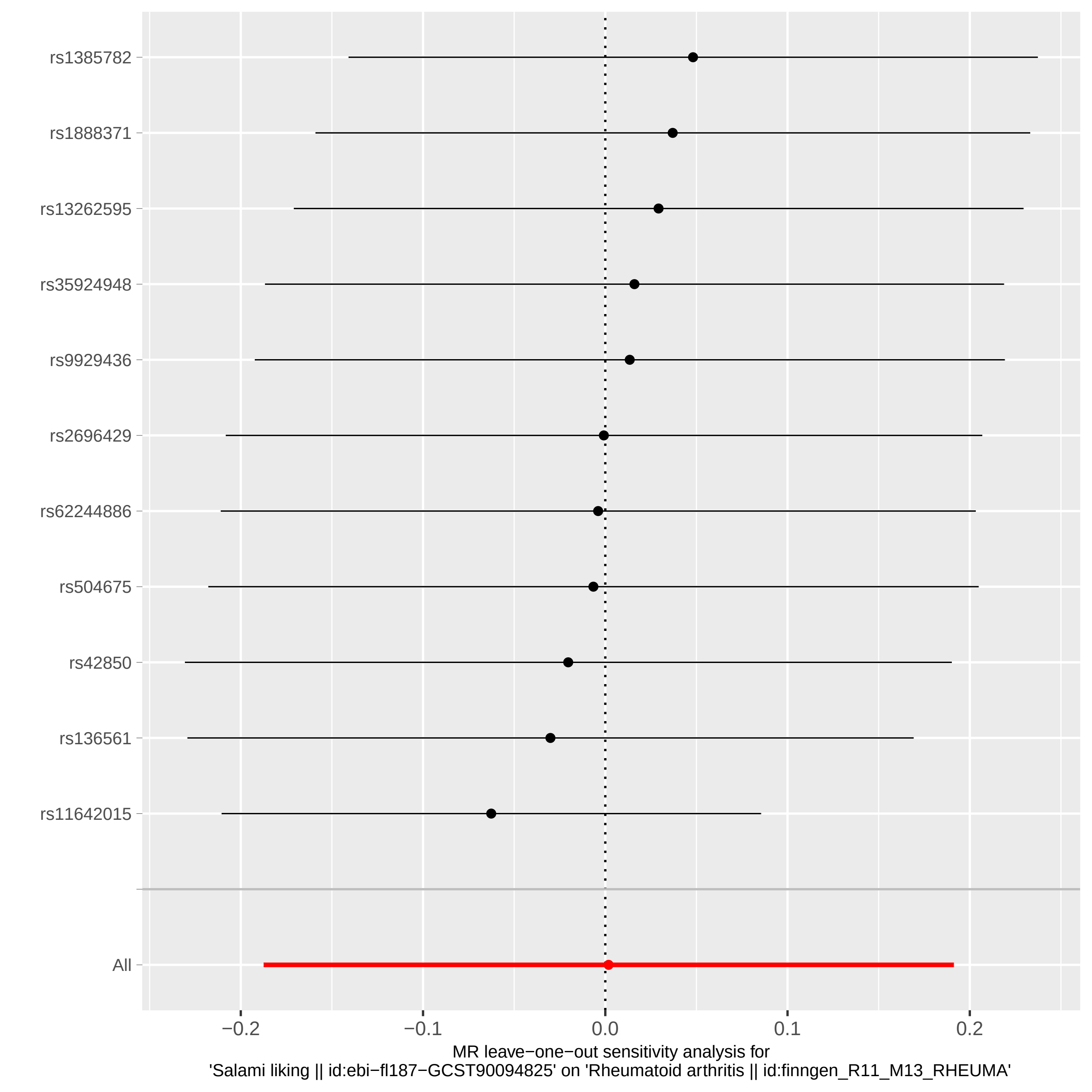
rs1385782
rs1888371
rs13262595
rs35924948
rs9929436
rs2696429
rs62244886
rs504675
rs42850
rs136561
rs11642015
All
−0.2
−0.1
0.0
0.1
0.2
MR leave−one−out sensitivity analysis for
'Salami liking || id:ebi−fl187−GCST90094825' on 'Rheumatoid arthritis || id:finngen_R11_M13_RHEUMA'

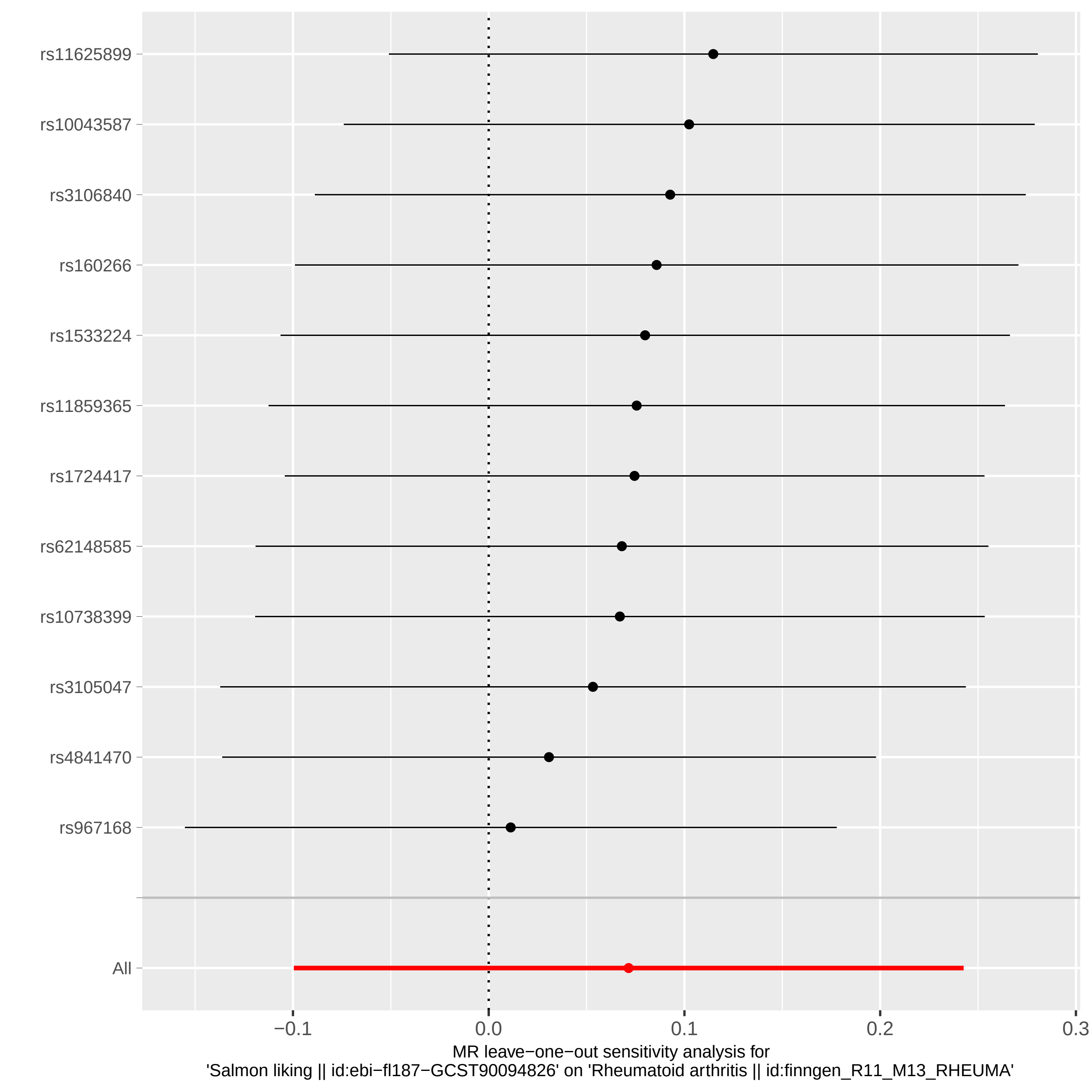
rs11625899
rs10043587
rs3106840
rs160266
rs1533224
rs11859365
rs1724417
rs62148585
rs10738399
rs3105047
rs4841470
rs967168
All
−0.1
0.0
0.1
0.2
0.3
MR leave−one−out sensitivity analysis for
'Salmon liking || id:ebi−fl187−GCST90094826' on 'Rheumatoid arthritis || id:finngen_R11_M13_RHEUMA'

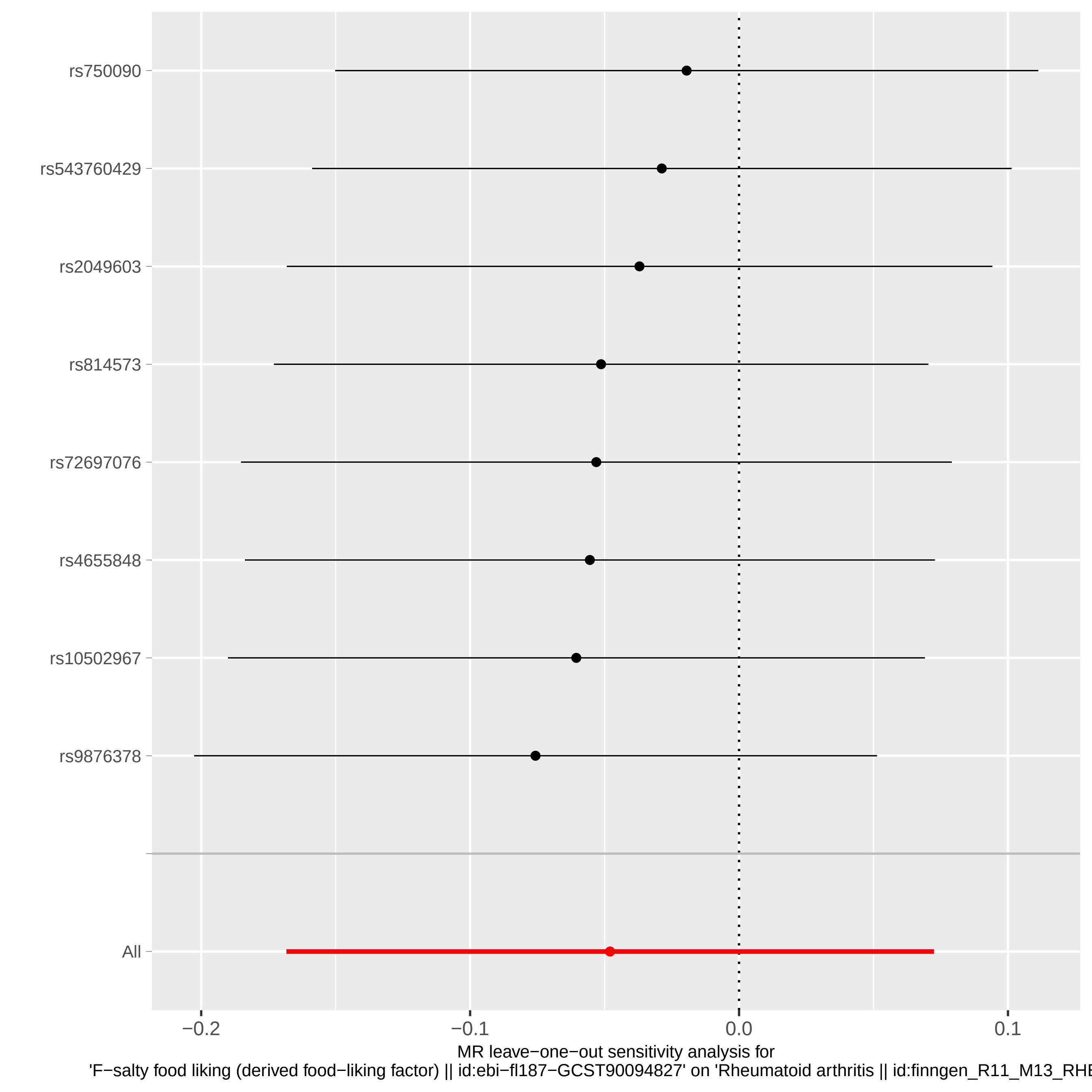
rs750090
rs543760429
rs2049603
rs814573
rs72697076
rs4655848
rs10502967
rs9876378
All
−0.2
−0.1
0.0
0.1
MR leave−one−out sensitivity analysis for
'F−salty food liking (derived food−liking factor) || id:ebi−fl187−GCST90094827' on 'Rheumatoid arthritis || id:finngen_R11_M13_RHE

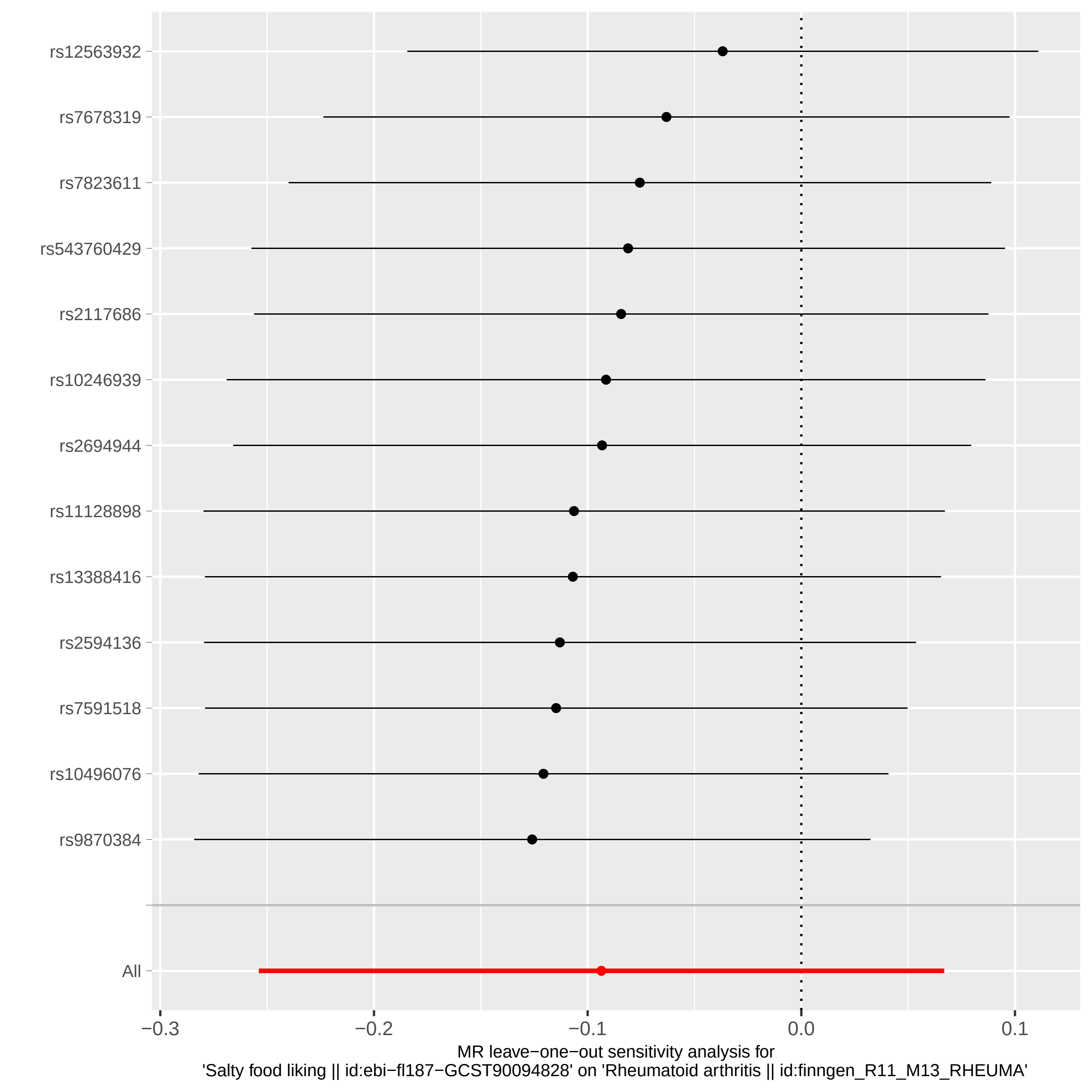
rs12563932
rs7678319
rs7823611
rs543760429
rs2117686
rs10246939
rs2694944
rs11128898
rs13388416
rs2594136
rs7591518
rs10496076
rs9870384
All
−0.3
−0.2
−0.1
0.0
0.1
MR leave−one−out sensitivity analysis for
'Salty food liking || id:ebi−fl187−GCST90094828' on 'Rheumatoid arthritis || id:finngen_R11_M13_RHEUMA'

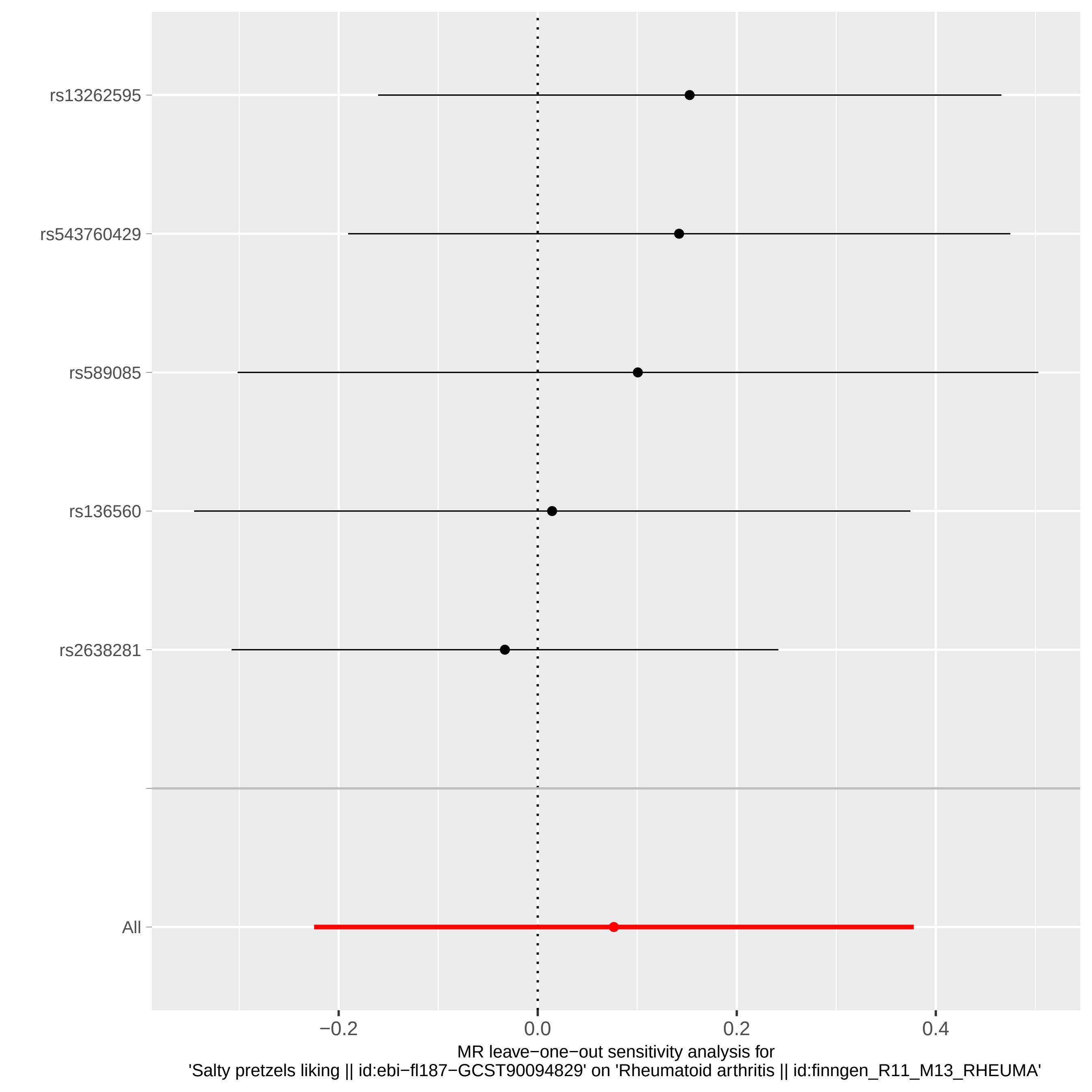
rs13262595
rs543760429
rs589085
rs136560
rs2638281
All
−0.2
0.0
0.2
0.4
MR leave−one−out sensitivity analysis for
'Salty pretzels liking || id:ebi−fl187−GCST90094829' on 'Rheumatoid arthritis || id:finngen_R11_M13_RHEUMA'

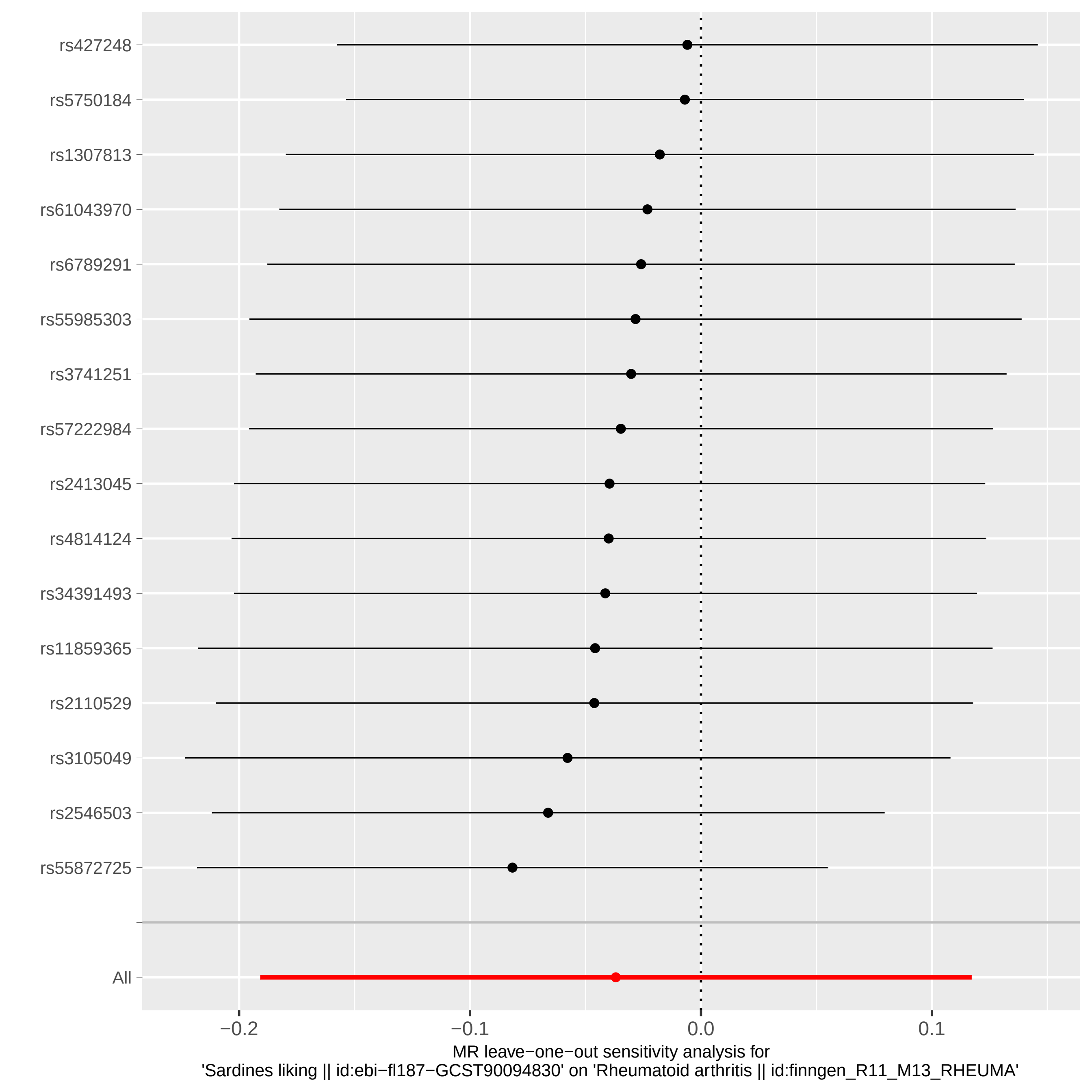
rs427248
rs5750184
rs1307813
rs61043970
rs6789291
rs55985303
rs3741251
rs57222984
rs2413045
rs4814124
rs34391493
rs11859365
rs2110529
rs3105049
rs2546503
rs55872725
All
−0.2
−0.1
0.0
0.1
MR leave−one−out sensitivity analysis for
'Sardines liking || id:ebi−fl187−GCST90094830' on 'Rheumatoid arthritis || id:finngen_R11_M13_RHEUMA'

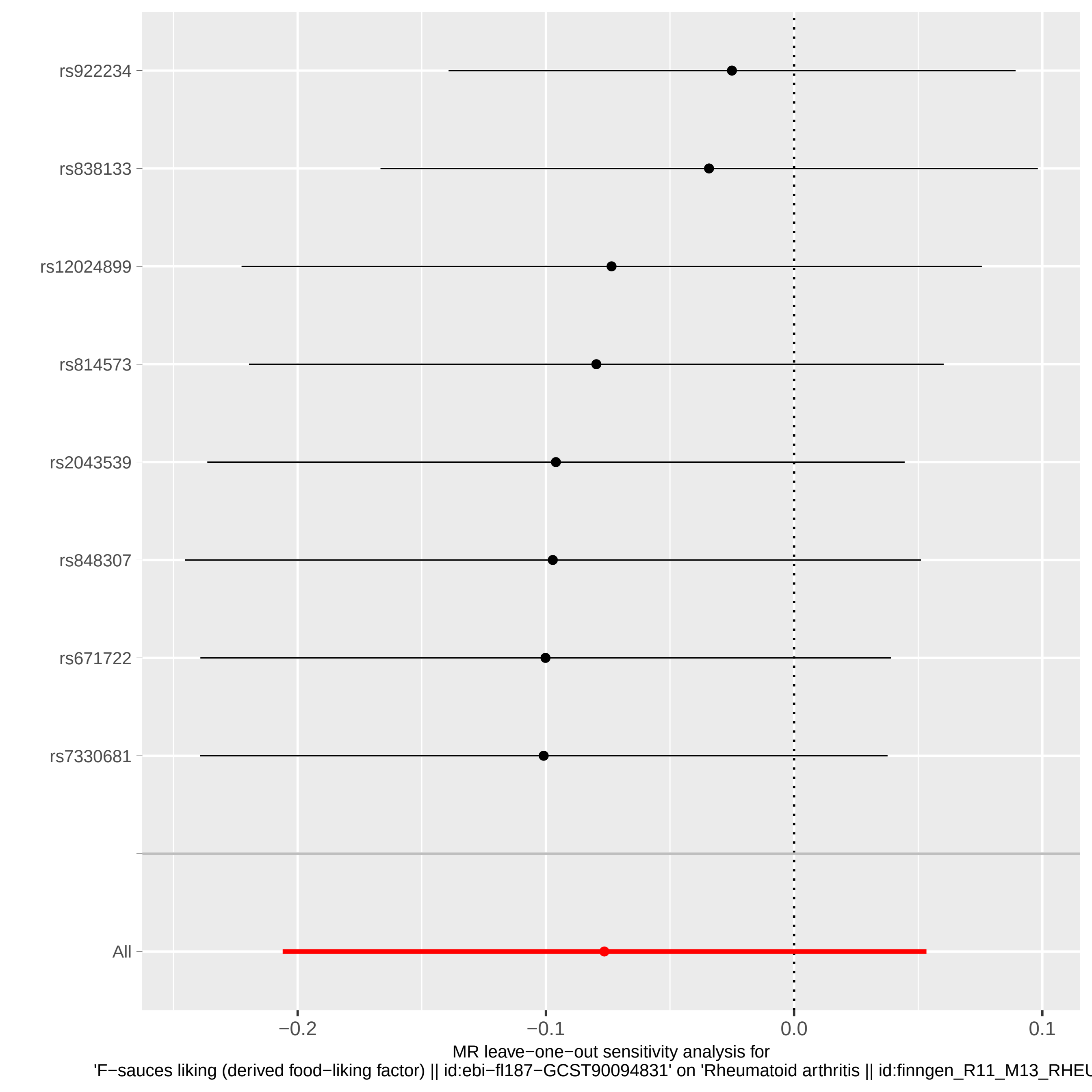

rs922234
rs838133
rs12024899
rs814573
rs2043539
rs848307
rs671722
rs7330681
All
−0.2
−0.1
0.0
0.1
MR leave−one−out sensitivity analysis for
'F−sauces liking (derived food−liking factor) || id:ebi−fl187−GCST90094831' on 'Rheumatoid arthritis || id:finngen_R11_M13_RHEU

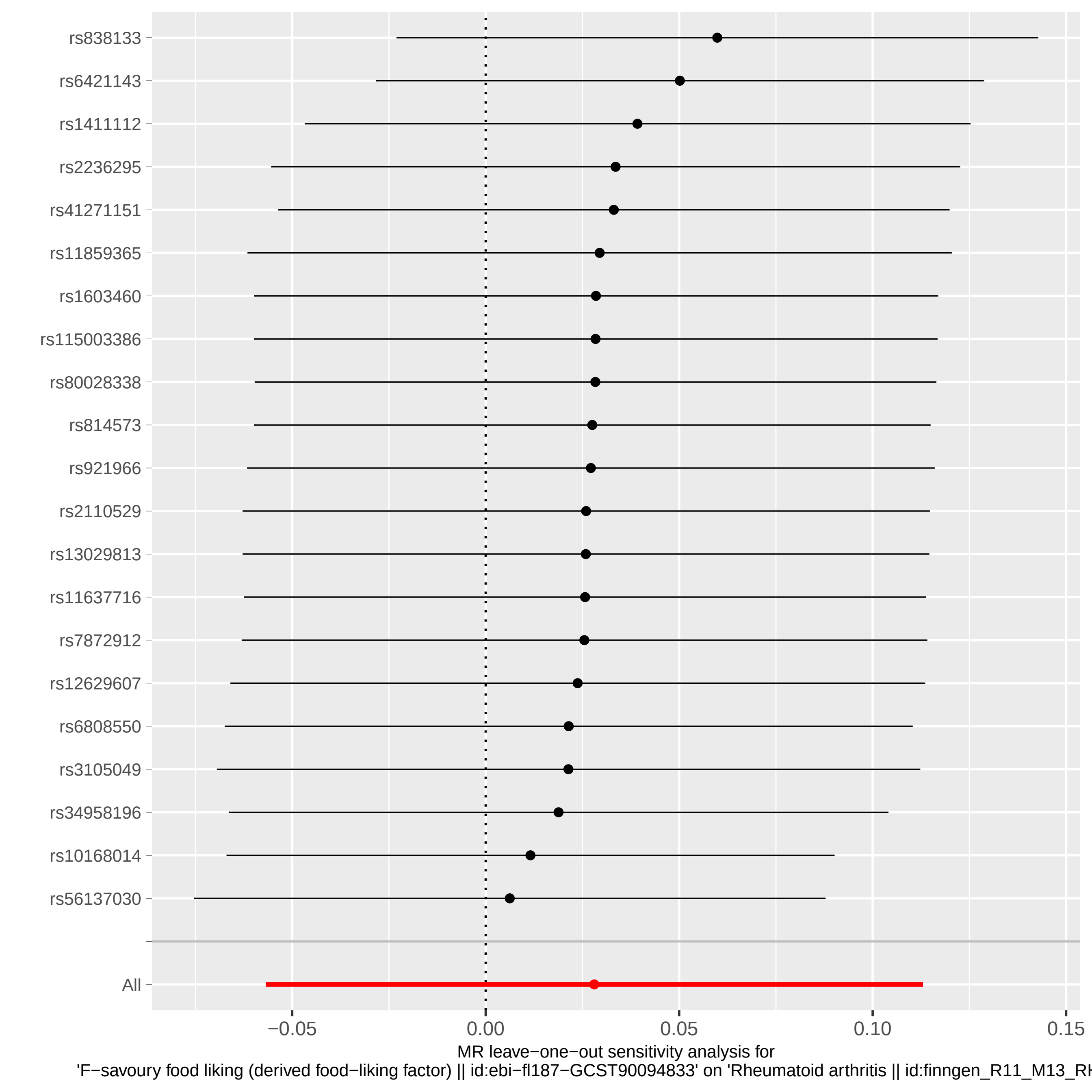

rs838133
rs6421143
rs1411112
rs2236295
rs41271151
rs11859365
rs1603460
rs115003386
rs80028338
rs814573
rs921966
rs2110529
rs13029813
rs11637716
rs7872912
rs12629607
rs6808550
rs3105049
rs34958196
rs10168014
rs56137030
All
−0.05
0.00
0.05
0.10
0.15
MR leave−one−out sensitivity analysis for
'F−savoury food liking (derived food−liking factor) || id:ebi−fl187−GCST90094833' on 'Rheumatoid arthritis || id:finngen_R11_M13_RH

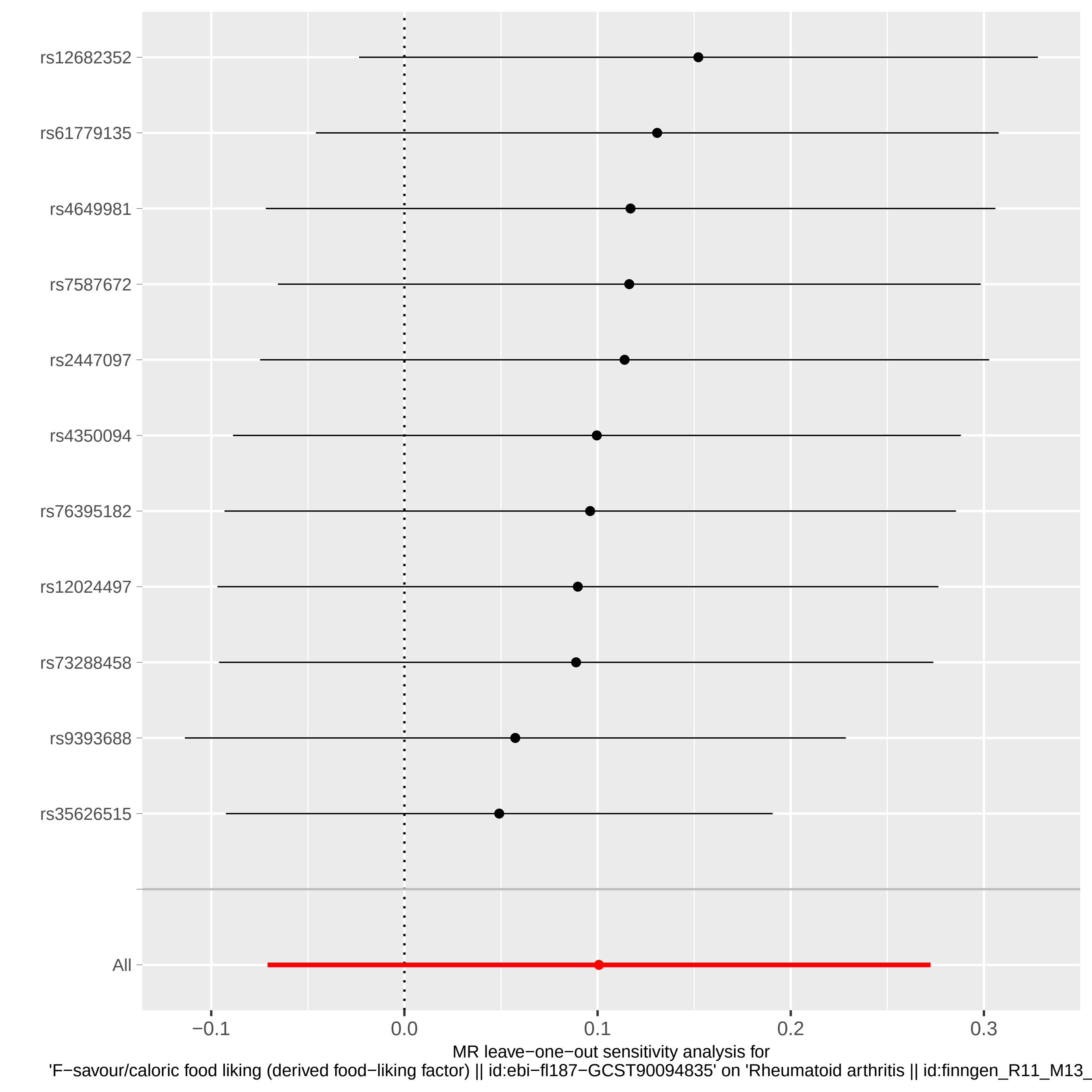

rs12682352
rs61779135
rs4649981
rs7587672
rs2447097
rs4350094
rs76395182
rs12024497
rs73288458
rs9393688
rs35626515
All
−0.1
0.0
0.1
0.2
0.3
MR leave−one−out sensitivity analysis for
'F−savour/caloric food liking (derived food−liking factor) || id:ebi−fl187−GCST90094835' on 'Rheumatoid arthritis || id:finngen_R11_M13_

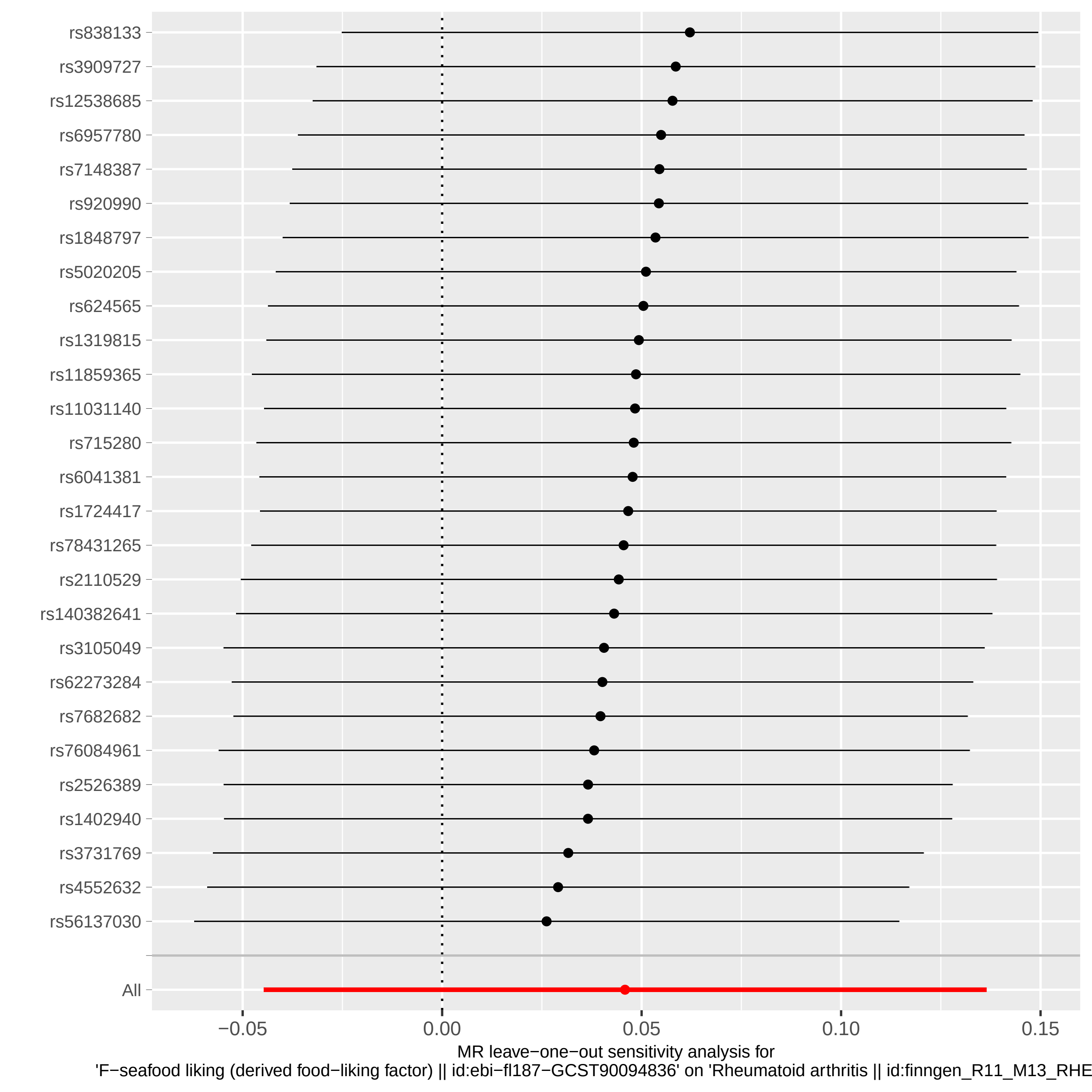
rs838133
rs3909727
rs12538685
rs6957780
rs7148387
rs920990
rs1848797
rs5020205
rs624565
rs1319815
rs11859365
rs11031140
rs715280
rs6041381
rs1724417
rs78431265
rs2110529
rs140382641
rs3105049
rs62273284
rs7682682
rs76084961
rs2526389
rs1402940
rs3731769
rs4552632
rs56137030
All
−0.05
0.00
0.05
0.10
0.15
MR leave−one−out sensitivity analysis for
'F−seafood liking (derived food−liking factor) || id:ebi−fl187−GCST90094836' on 'Rheumatoid arthritis || id:finngen_R11_M13_RHE

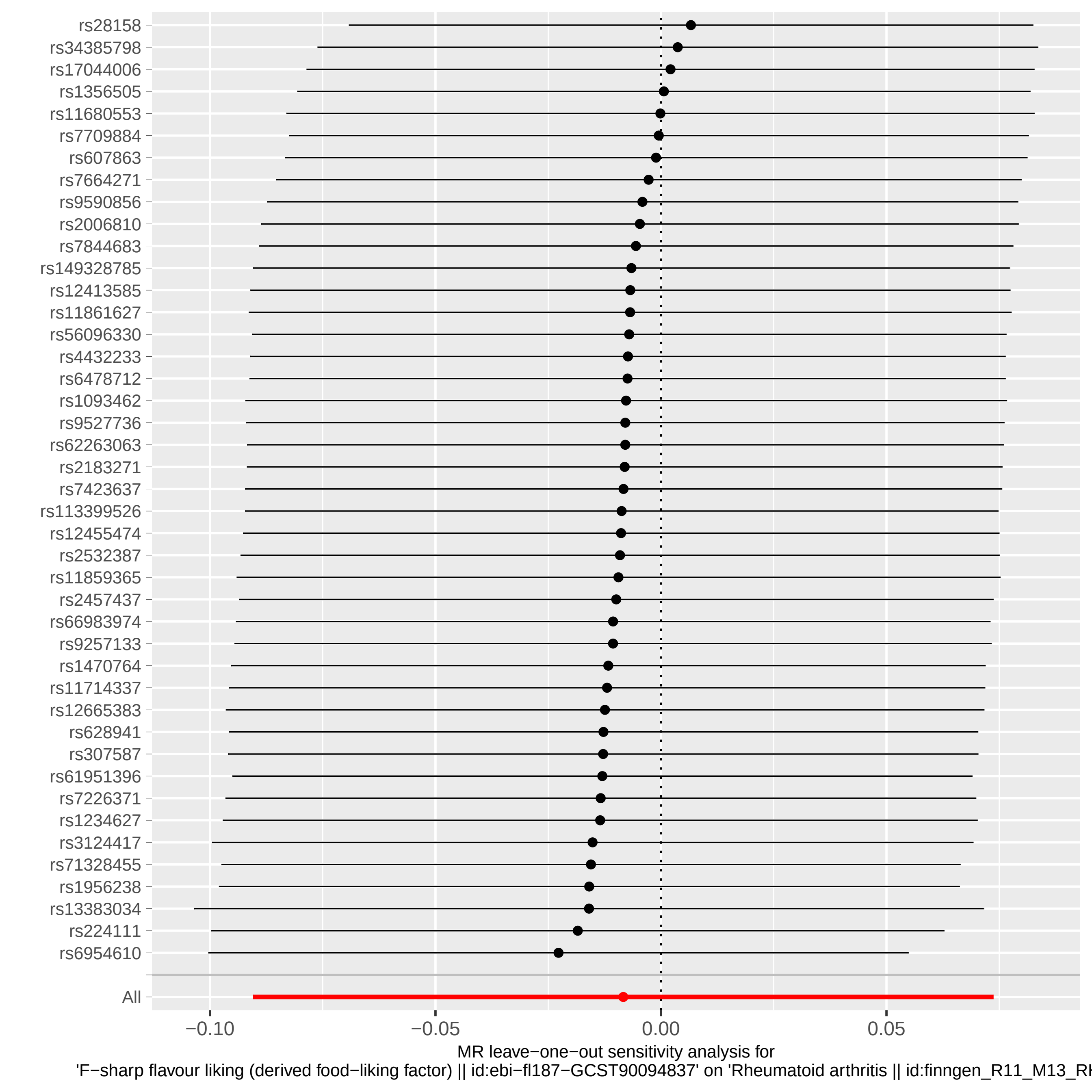

rs28158
rs34385798
rs17044006
rs1356505
rs11680553
rs7709884
rs607863
rs7664271
rs9590856
rs2006810
rs7844683
rs149328785
rs12413585
rs11861627
rs56096330
rs4432233
rs6478712
rs1093462
rs9527736
rs62263063
rs2183271
rs7423637
rs113399526
rs12455474
rs2532387
rs11859365
rs2457437
rs66983974
rs9257133
rs1470764
rs11714337
rs12665383
rs628941
rs307587
rs61951396
rs7226371
rs1234627
rs3124417
rs71328455
rs1956238
rs13383034
rs224111
rs6954610
All
−0.10
−0.05
0.00
0.05
MR leave−one−out sensitivity analysis for
'F−sharp flavour liking (derived food−liking factor) || id:ebi−fl187−GCST90094837' on 'Rheumatoid arthritis || id:finngen_R11_M13_RH

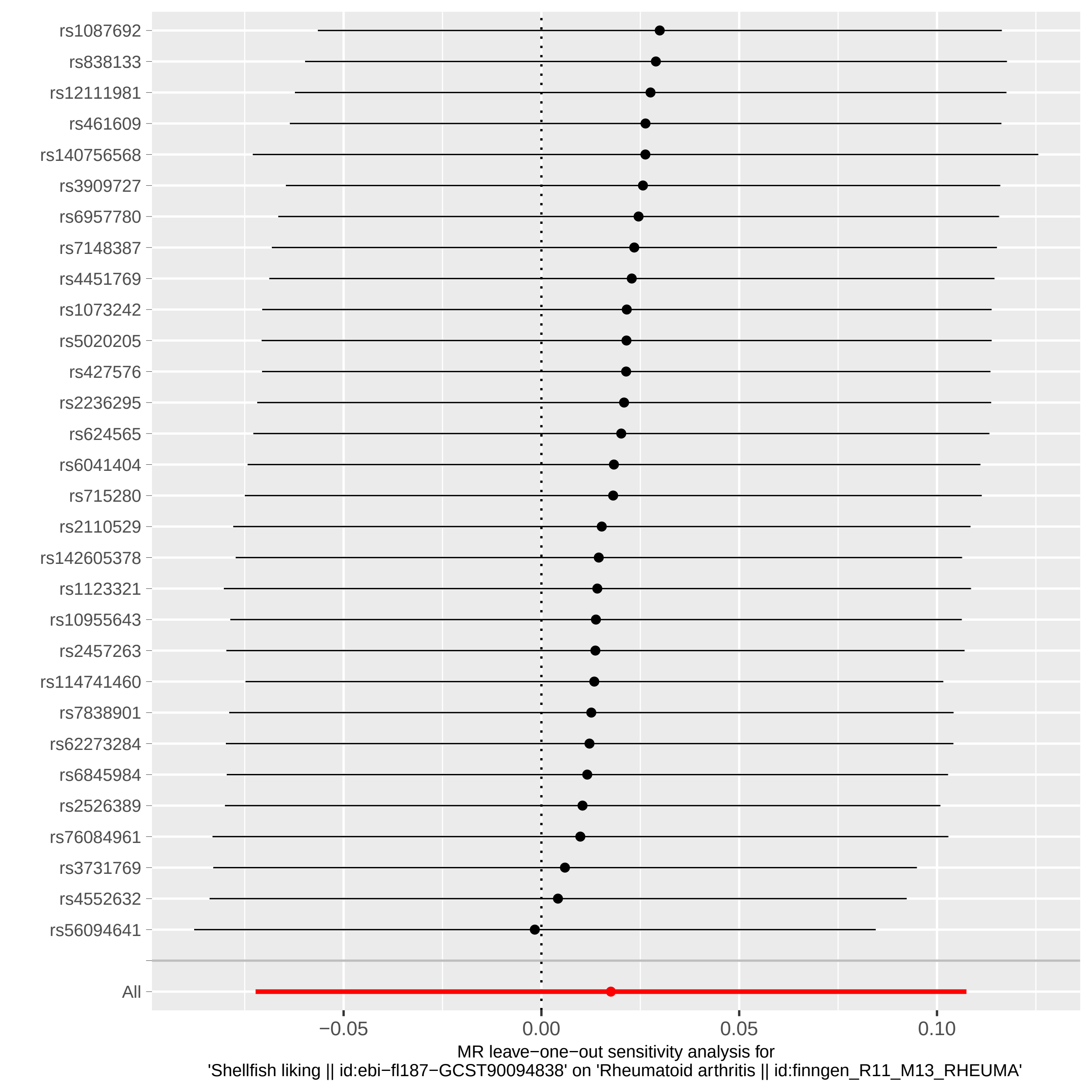

rs1087692
rs838133
rs12111981
rs461609
rs140756568
rs3909727
rs6957780
rs7148387
rs4451769
rs1073242
rs5020205
rs427576
rs2236295
rs624565
rs6041404
rs715280
rs2110529
rs142605378
rs1123321
rs10955643
rs2457263
rs114741460
rs7838901
rs62273284
rs6845984
rs2526389
rs76084961
rs3731769
rs4552632
rs56094641
All
−0.05
0.00
0.05
0.10
MR leave−one−out sensitivity analysis for
'Shellfish liking || id:ebi−fl187−GCST90094838' on 'Rheumatoid arthritis || id:finngen_R11_M13_RHEUMA'

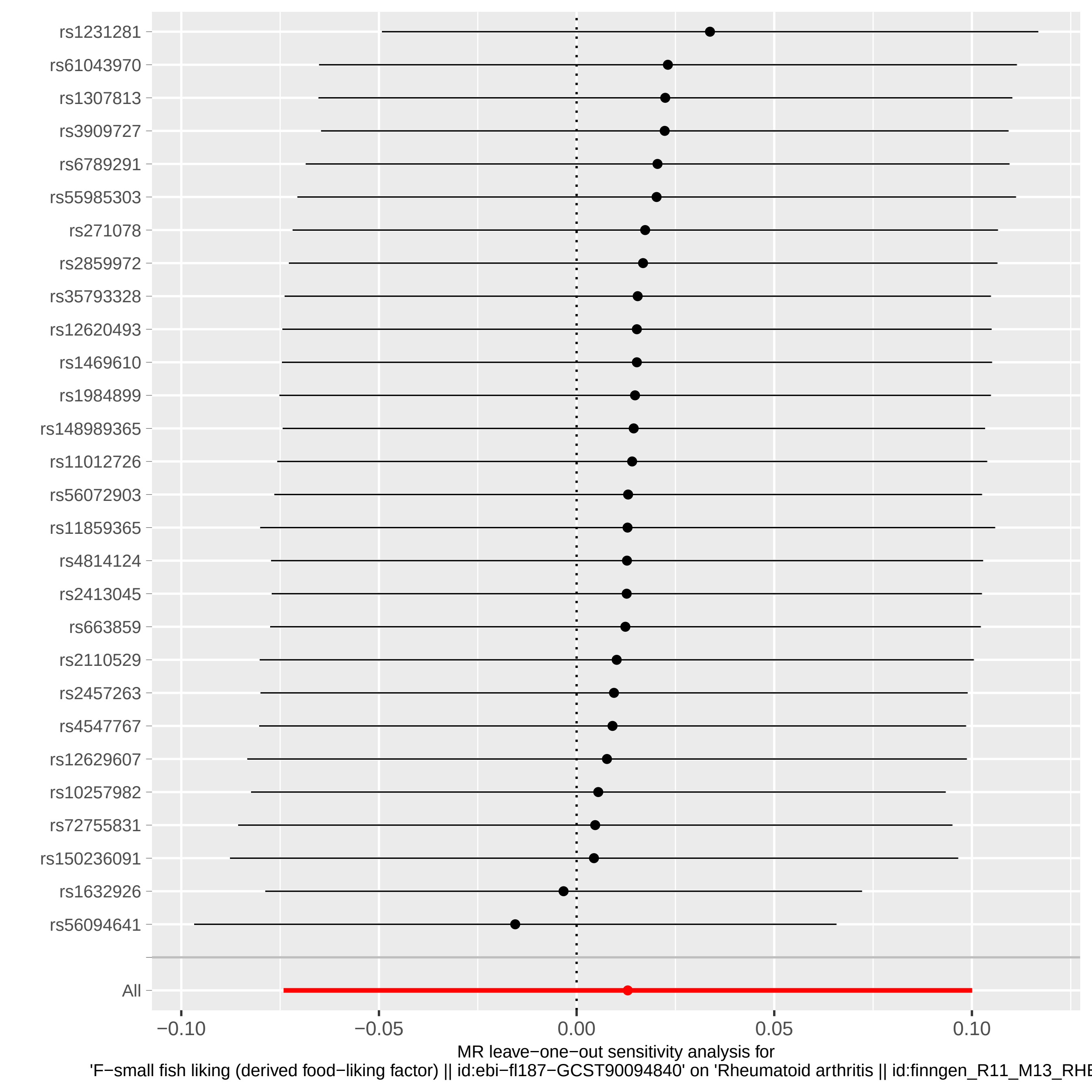
rs1231281
rs61043970
rs1307813
rs3909727
rs6789291
rs55985303
rs271078
rs2859972
rs35793328
rs12620493
rs1469610
rs1984899
rs148989365
rs11012726
rs56072903
rs11859365
rs4814124
rs2413045
rs663859
rs2110529
rs2457263
rs4547767
rs12629607
rs10257982
rs72755831
rs150236091
rs1632926
rs56094641
All
−0.10
−0.05
0.00
0.05
0.10
MR leave−one−out sensitivity analysis for
'F−small fish liking (derived food−liking factor) || id:ebi−fl187−GCST90094840' on 'Rheumatoid arthritis || id:finngen_R11_M13_RHE

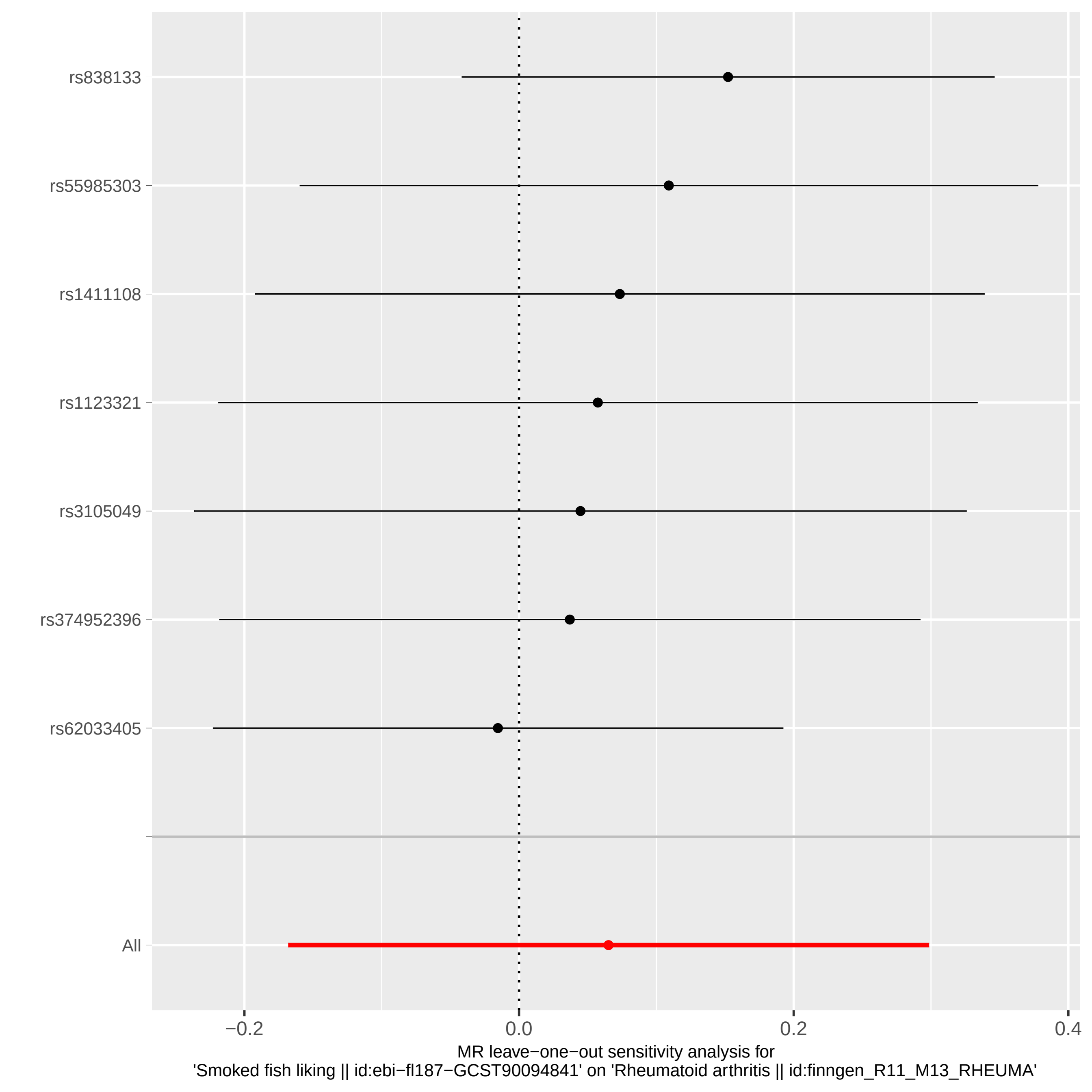
rs838133
rs55985303
rs1411108
rs1123321
rs3105049
rs374952396
rs62033405
All
−0.2
0.0
0.2
0.4
MR leave−one−out sensitivity analysis for
'Smoked fish liking || id:ebi−fl187−GCST90094841' on 'Rheumatoid arthritis || id:finngen_R11_M13_RHEUMA'

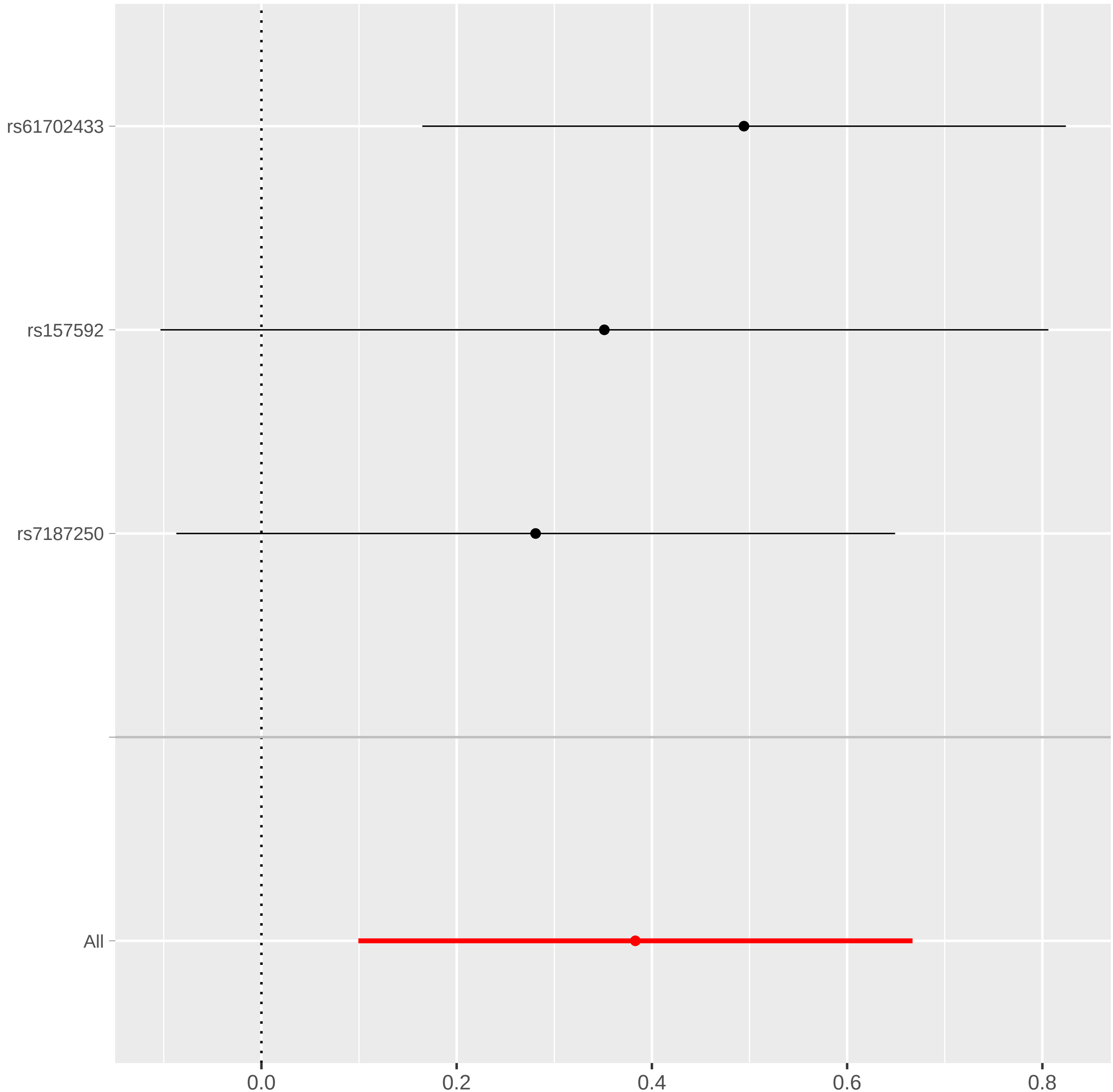

MR leave−one−out sensitivity analysis for
'Soft cheese liking || id:ebi−fl187−GCST90094843' on 'Rheumatoid arthritis || id:finngen_R11_M13_RHEUMA'

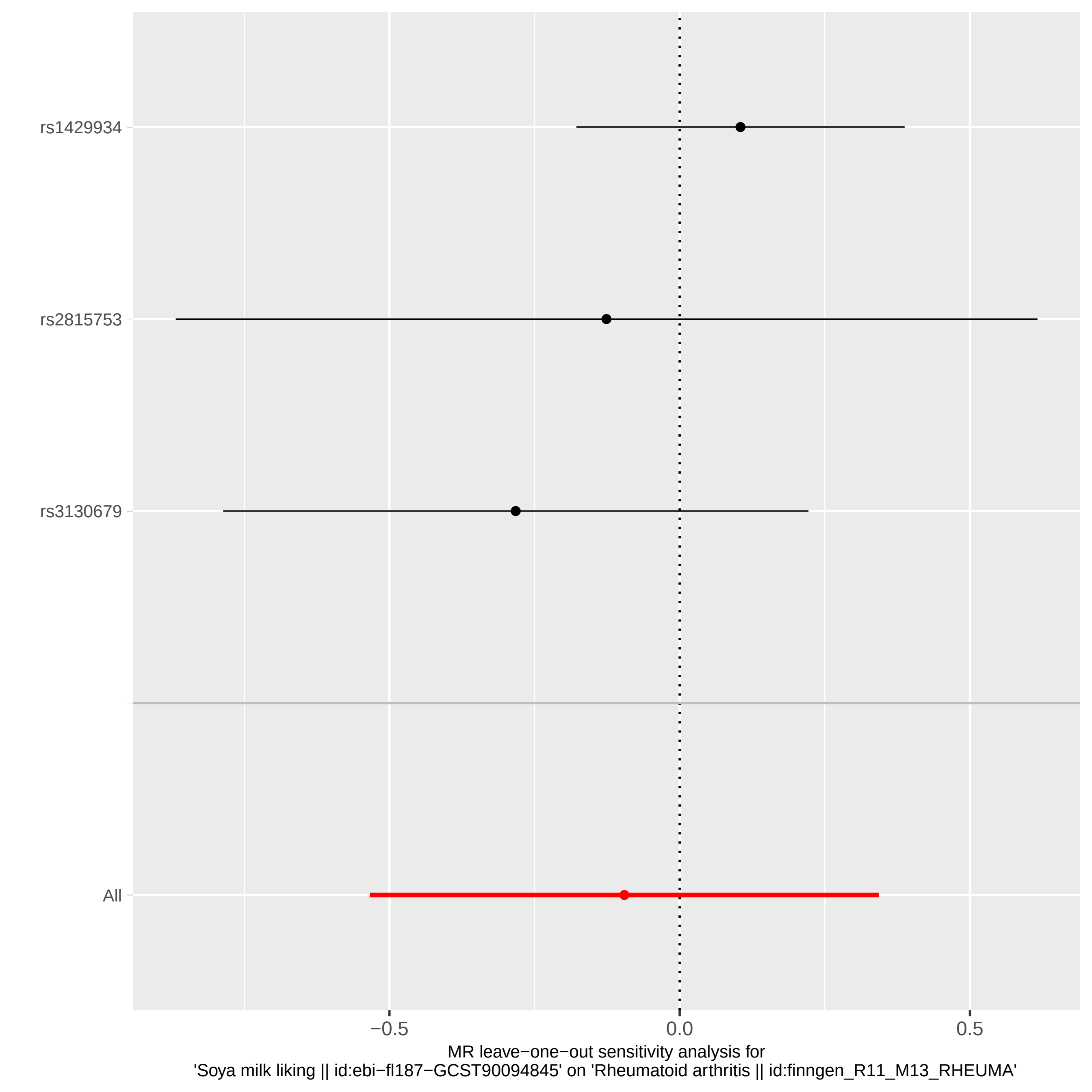

rs1429934
rs2815753
rs3130679
All
−0.5
0.0
0.5
MR leave−one−out sensitivity analysis for
'Soya milk liking || id:ebi−fl187−GCST90094845' on 'Rheumatoid arthritis || id:finngen_R11_M13_RHEUMA'

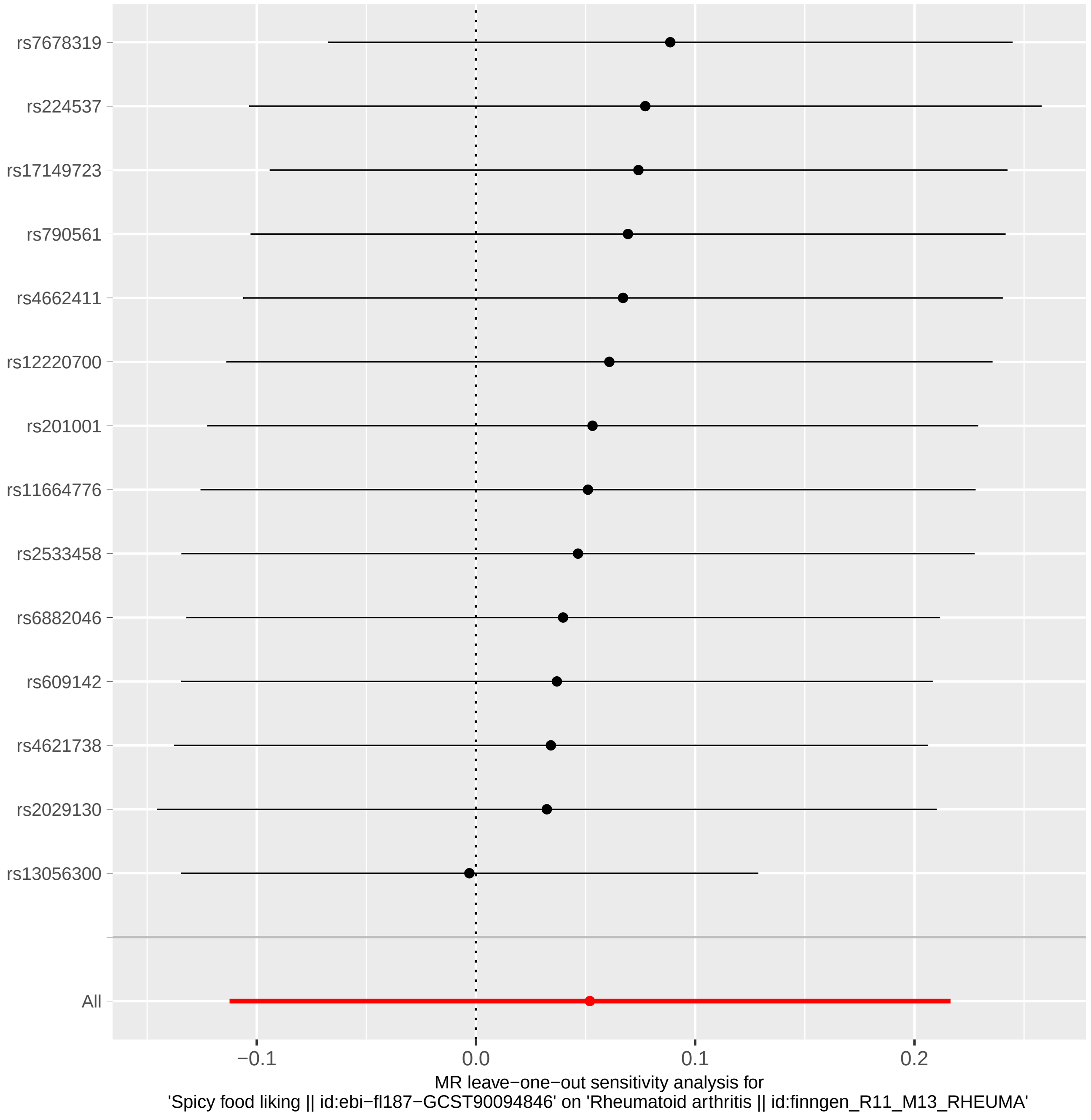
rs7678319
rs224537
rs17149723
rs790561
rs4662411
rs12220700
rs201001
rs11664776
rs2533458
rs6882046
rs609142
rs4621738
rs2029130
rs13056300
All
−0.1
0.0
0.1
0.2
MR leave−one−out sensitivity analysis for
'Spicy food liking || id:ebi−fl187−GCST90094846' on 'Rheumatoid arthritis || id:finngen_R11_M13_RHEUMA'

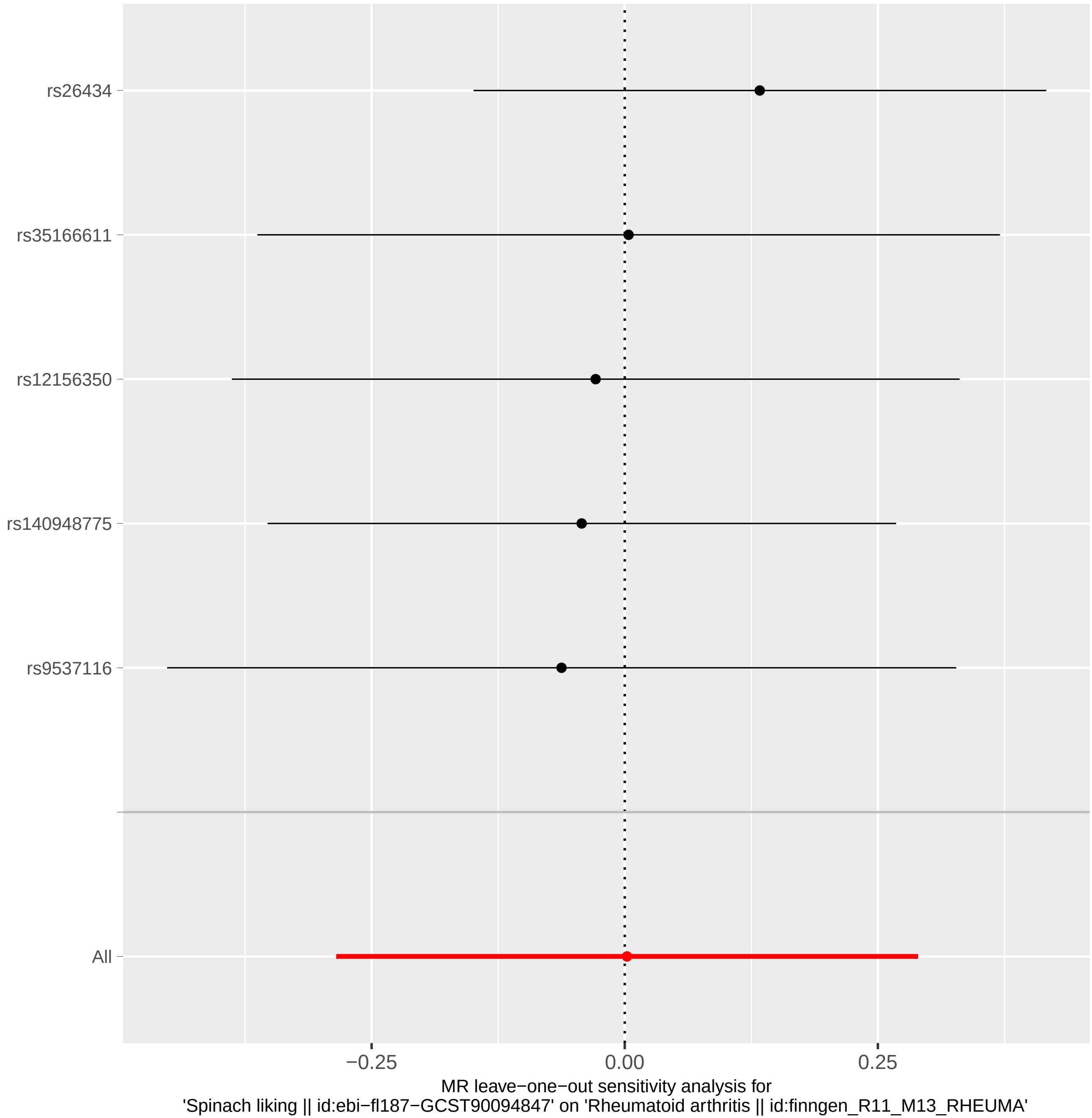
rs26434
rs35166611
rs12156350
rs140948775
rs9537116
All
−0.25
0.00
0.25
MR leave−one−out sensitivity analysis for
'Spinach liking || id:ebi−fl187−GCST90094847' on 'Rheumatoid arthritis || id:finngen_R11_M13_RHEUMA'

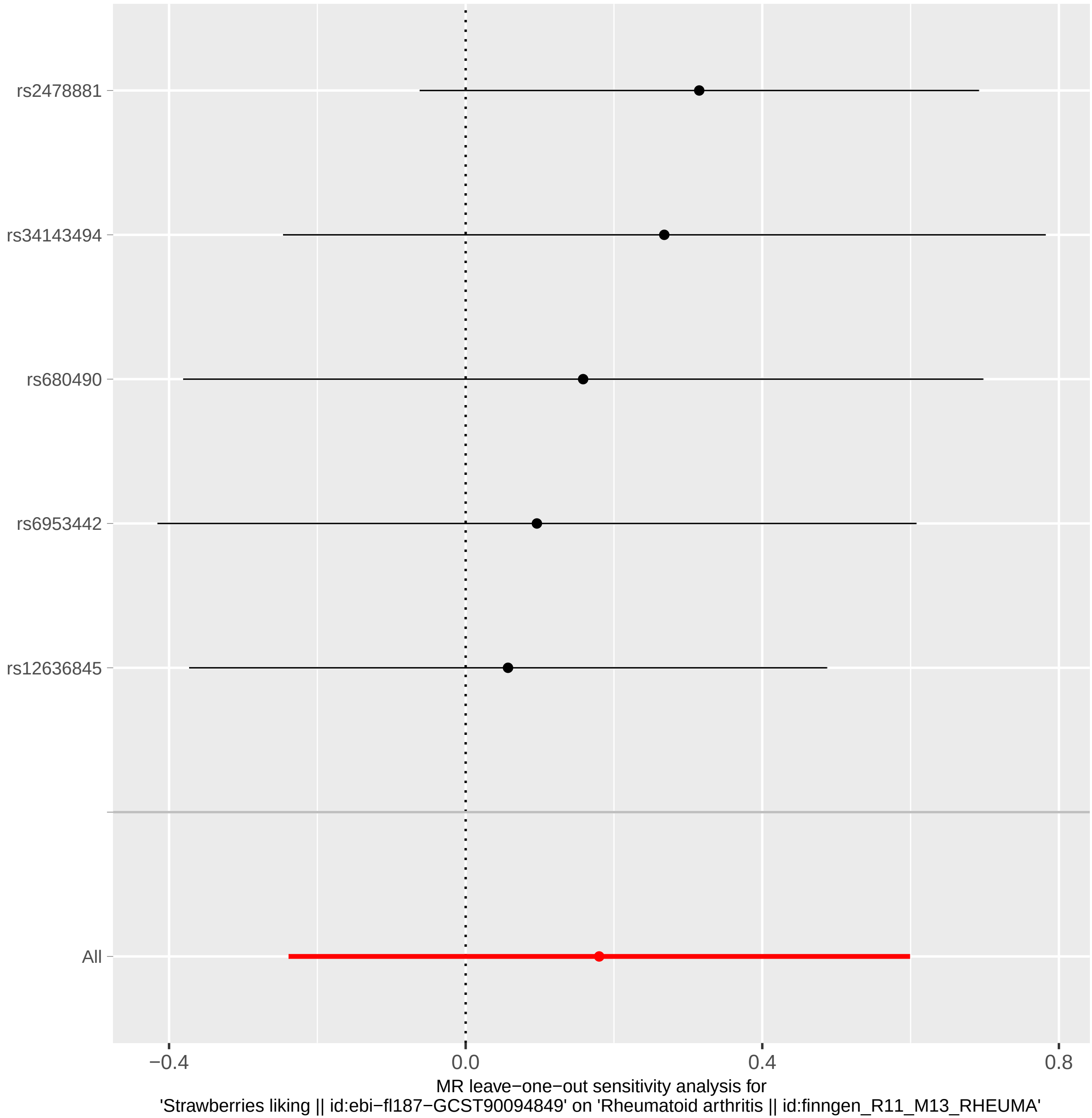

rs2478881
rs34143494
rs680490
rs6953442
rs12636845
All
−0.4
0.0
0.4
0.8
MR leave−one−out sensitivity analysis for
'Strawberries liking || id:ebi−fl187−GCST90094849' on 'Rheumatoid arthritis || id:finngen_R11_M13_RHEUMA'

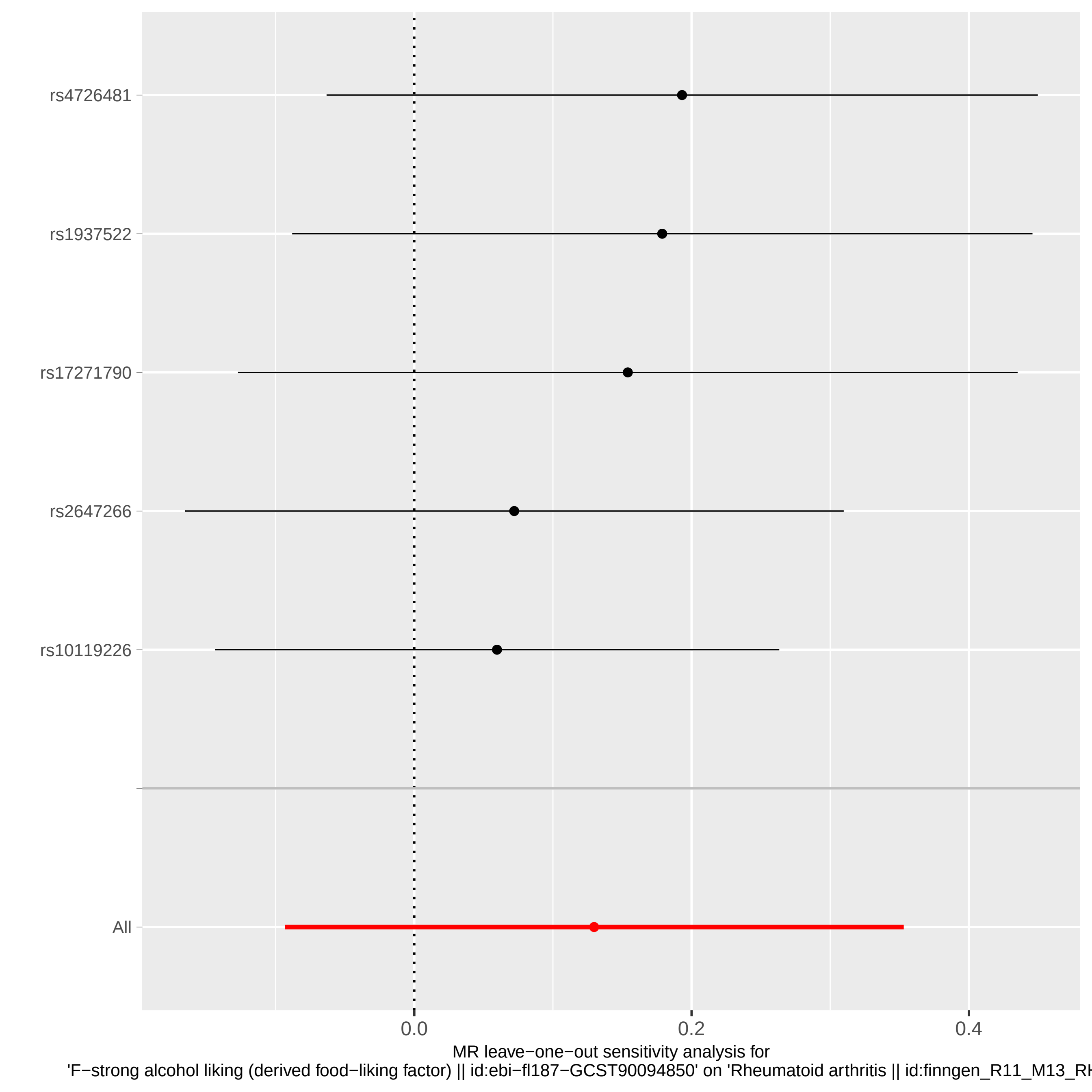

rs4726481
rs1937522
rs17271790
rs2647266
rs10119226
All
0.0
0.2
0.4
MR leave−one−out sensitivity analysis for
'F−strong alcohol liking (derived food−liking factor) || id:ebi−fl187−GCST90094850' on 'Rheumatoid arthritis || id:finngen_R11_M13_RH

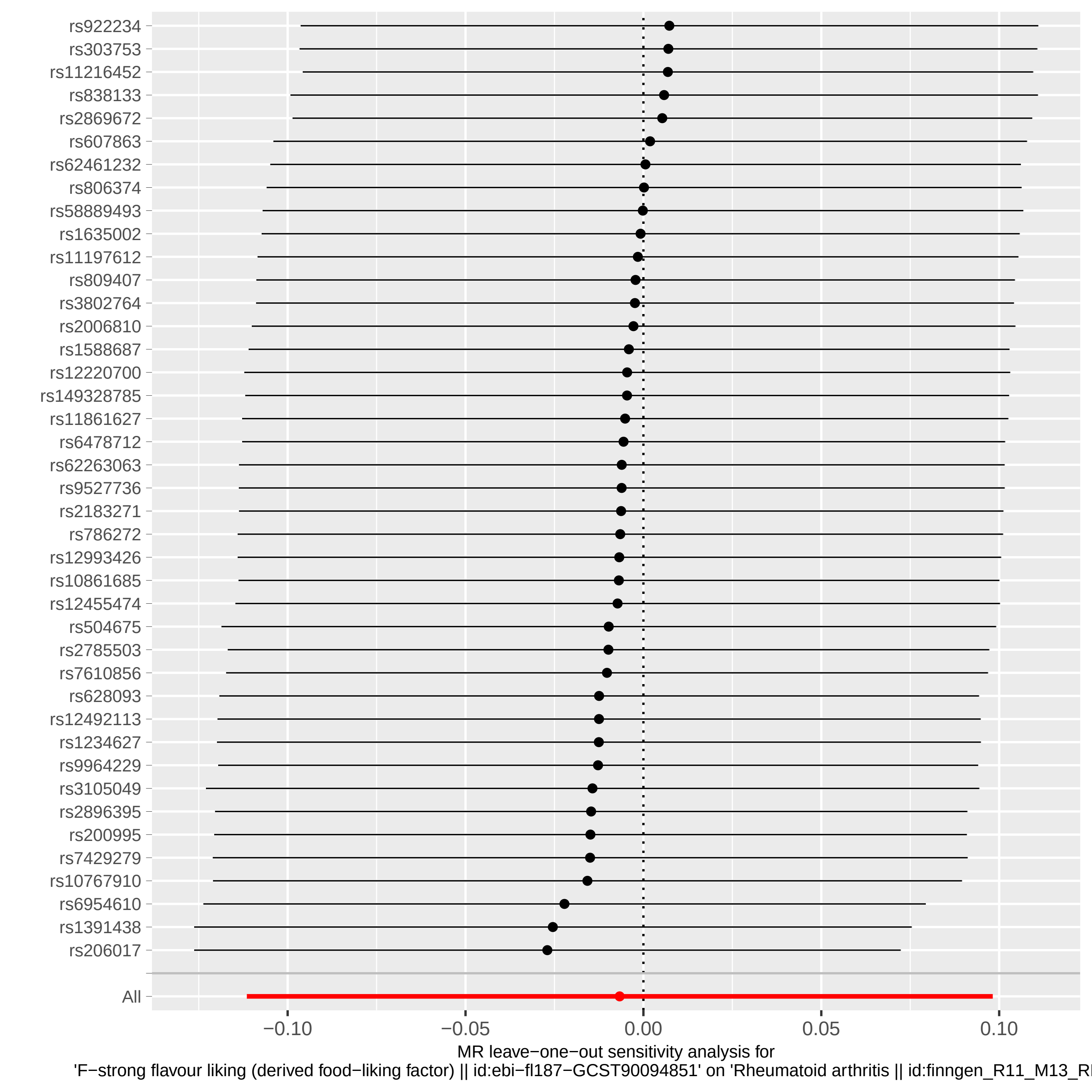
rs922234
rs303753
rs11216452
rs838133
rs2869672
rs607863
rs62461232
rs806374
rs58889493
rs1635002
rs11197612
rs809407
rs3802764
rs2006810
rs1588687
rs12220700
rs149328785
rs11861627
rs6478712
rs62263063
rs9527736
rs2183271
rs786272
rs12993426
rs10861685
rs12455474
rs504675
rs2785503
rs7610856
rs628093
rs12492113
rs1234627
rs9964229
rs3105049
rs2896395
rs200995
rs7429279
rs10767910
rs6954610
rs1391438
rs206017
All
−0.10
−0.05
0.00
0.05
0.10
MR leave−one−out sensitivity analysis for
'F−strong flavour liking (derived food−liking factor) || id:ebi−fl187−GCST90094851' on 'Rheumatoid arthritis || id:finngen_R11_M13_R

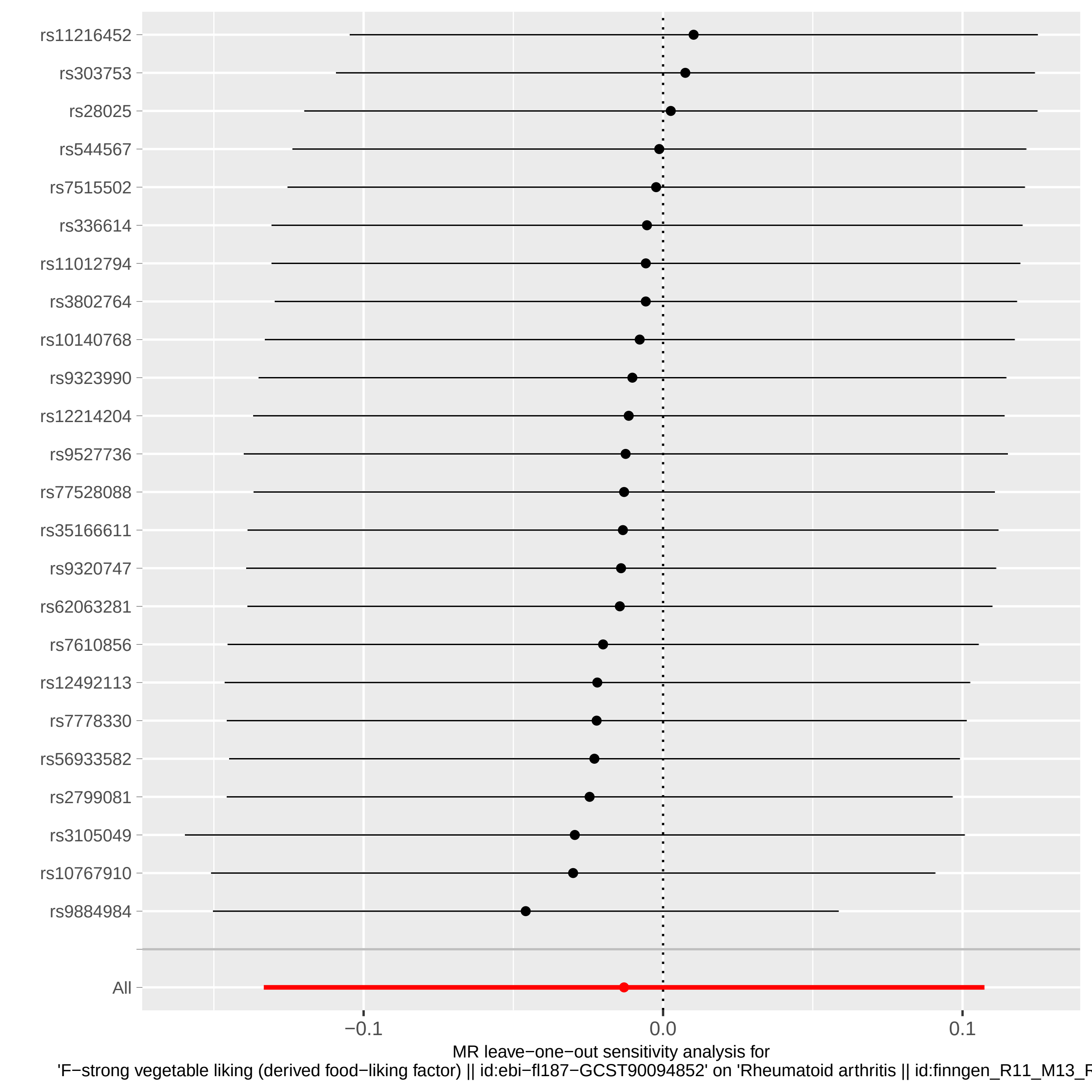
rs11216452
rs303753
rs28025
rs544567
rs7515502
rs336614
rs11012794
rs3802764
rs10140768
rs9323990
rs12214204
rs9527736
rs77528088
rs35166611
rs9320747
rs62063281
rs7610856
rs12492113
rs7778330
rs56933582
rs2799081
rs3105049
rs10767910
rs9884984
All
−0.1
0.0
0.1
MR leave−one−out sensitivity analysis for
'F−strong vegetable liking (derived food−liking factor) || id:ebi−fl187−GCST90094852' on 'Rheumatoid arthritis || id:finngen_R11_M13_F

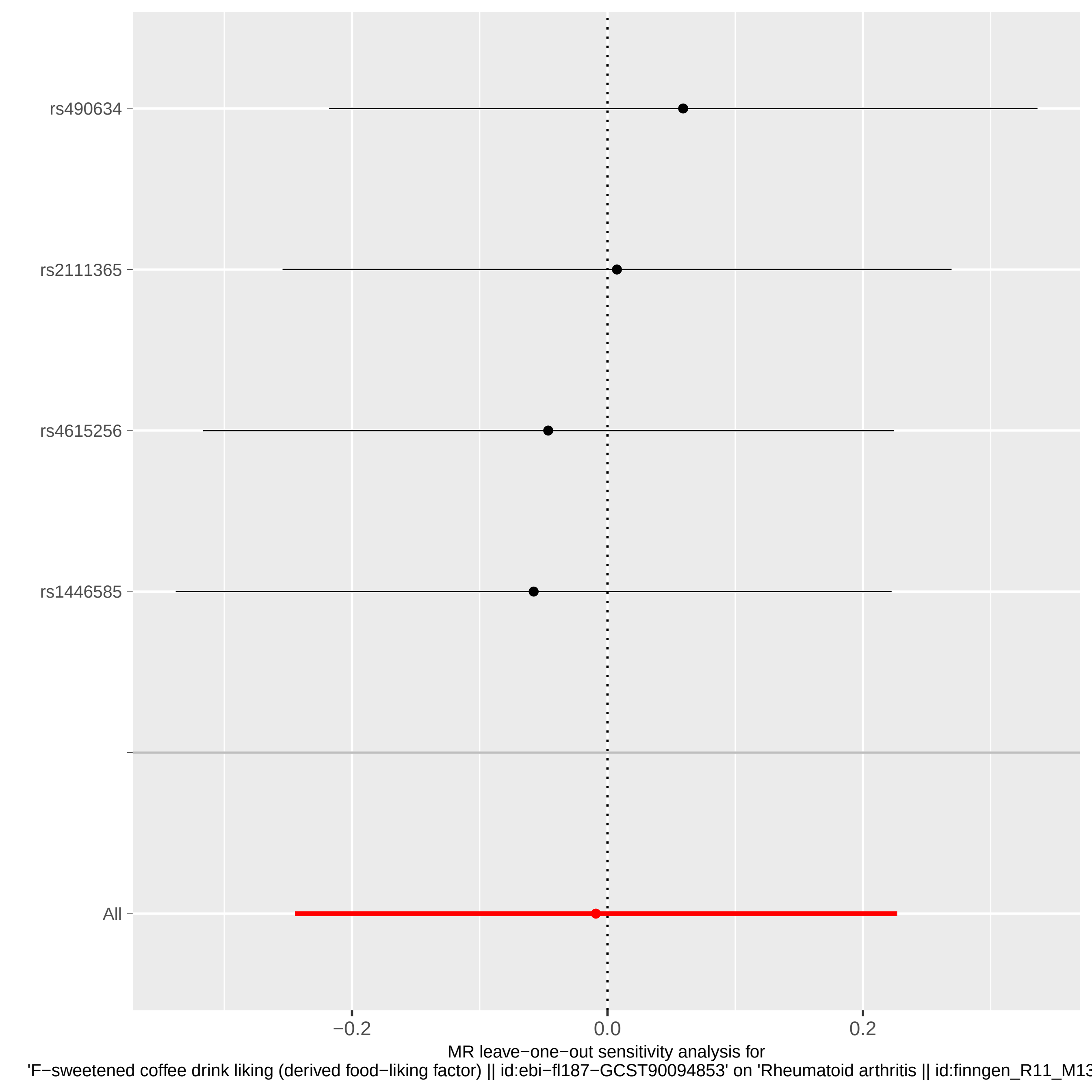
rs490634
rs2111365
rs4615256
rs1446585
All
−0.2
0.0
0.2
MR leave−one−out sensitivity analysis for
'F−sweetened coffee drink liking (derived food−liking factor) || id:ebi−fl187−GCST90094853' on 'Rheumatoid arthritis || id:finngen_R11_M13

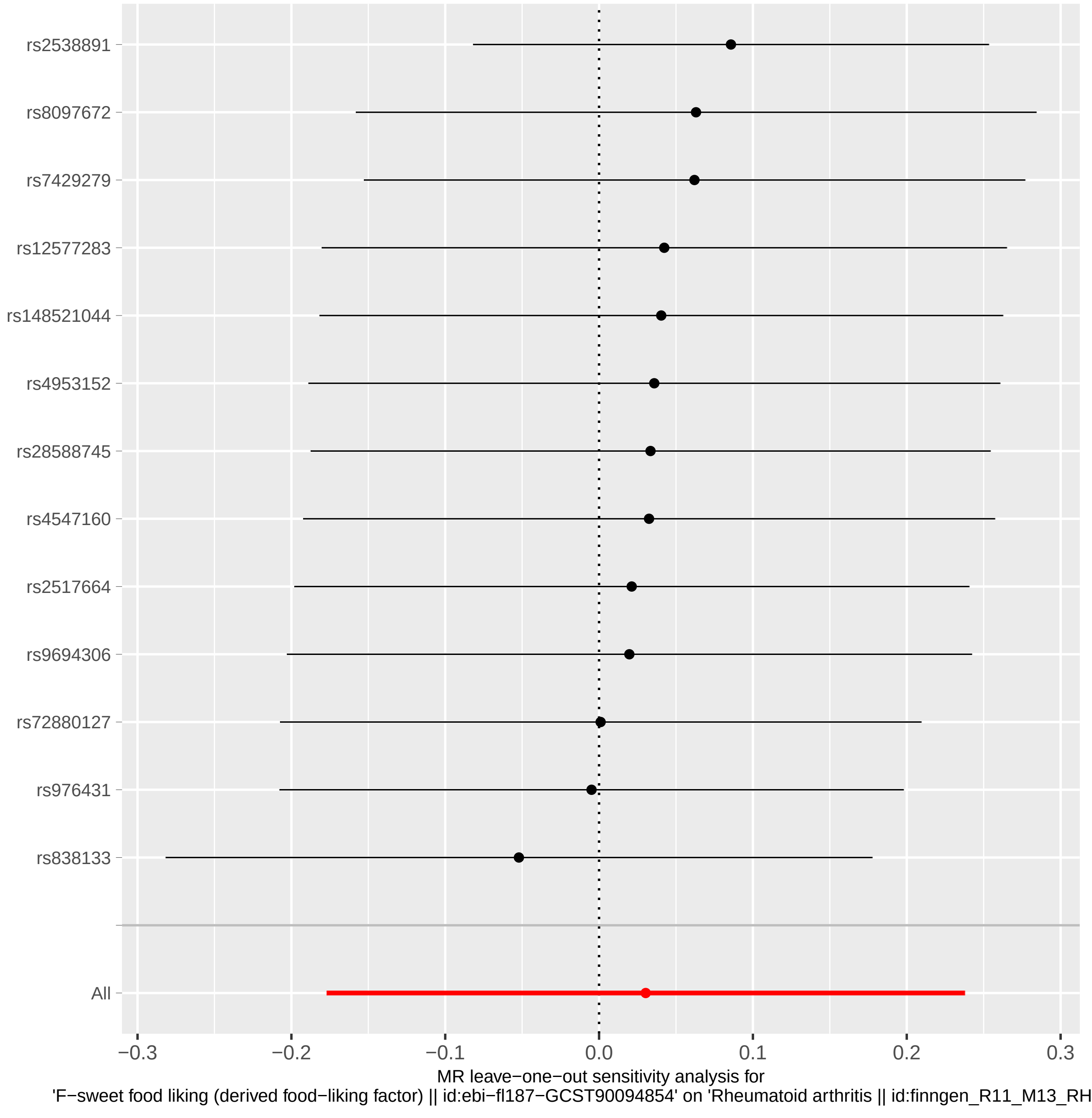
rs2538891
rs8097672
rs7429279
rs12577283
rs148521044
rs4953152
rs28588745
rs4547160
rs2517664
rs9694306
rs72880127
rs976431
rs838133
All
−0.3
−0.2
−0.1
0.0
0.1
0.2
0.3
MR leave−one−out sensitivity analysis for
'F−sweet food liking (derived food−liking factor) || id:ebi−fl187−GCST90094854' on 'Rheumatoid arthritis || id:finngen_R11_M13_RH

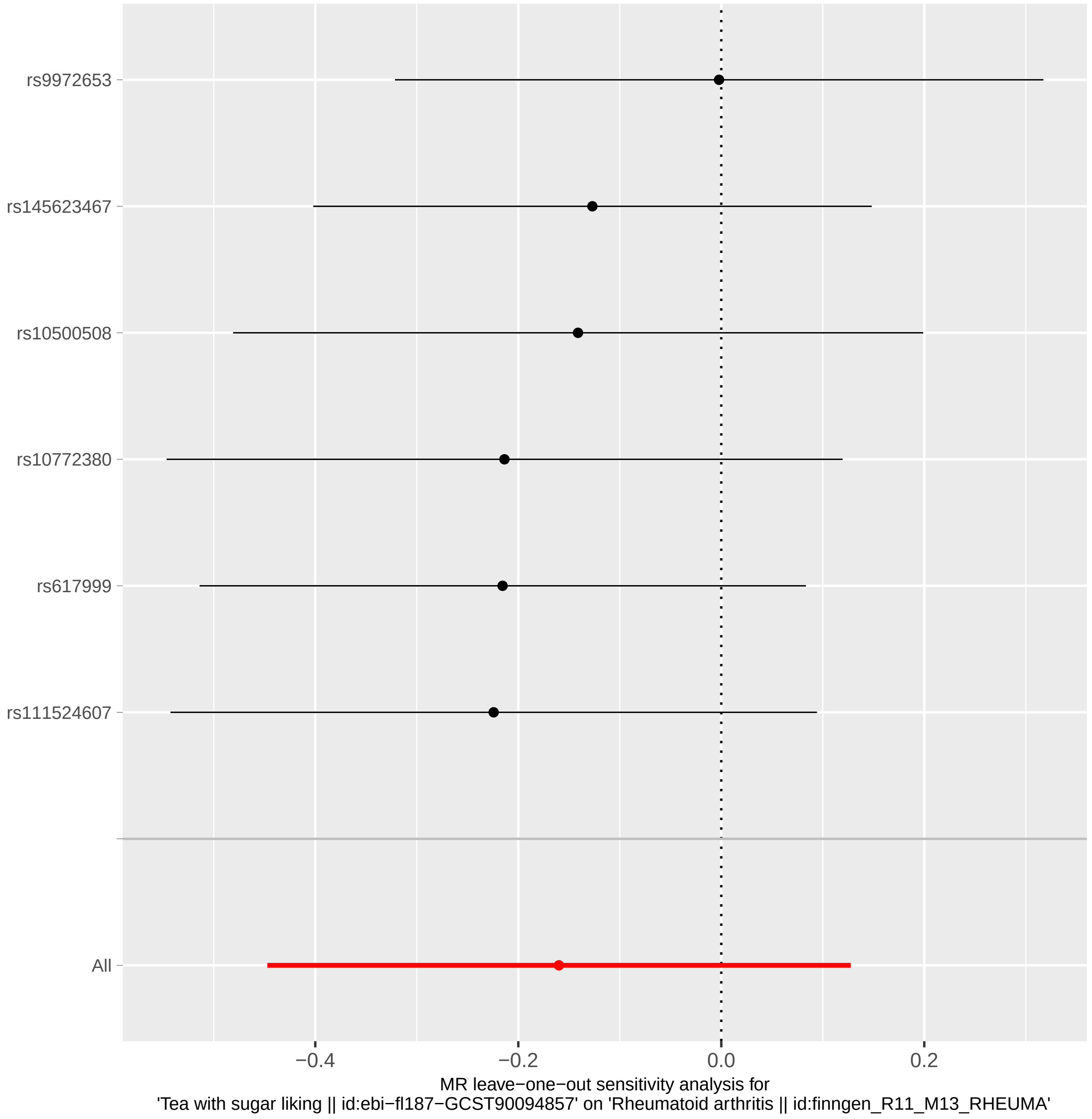

rs9972653
rs145623467
rs10500508
rs10772380
rs617999
rs111524607
All
−0.4
−0.2
0.0
0.2
MR leave−one−out sensitivity analysis for
'Tea with sugar liking || id:ebi−fl187−GCST90094857' on 'Rheumatoid arthritis || id:finngen_R11_M13_RHEUMA'

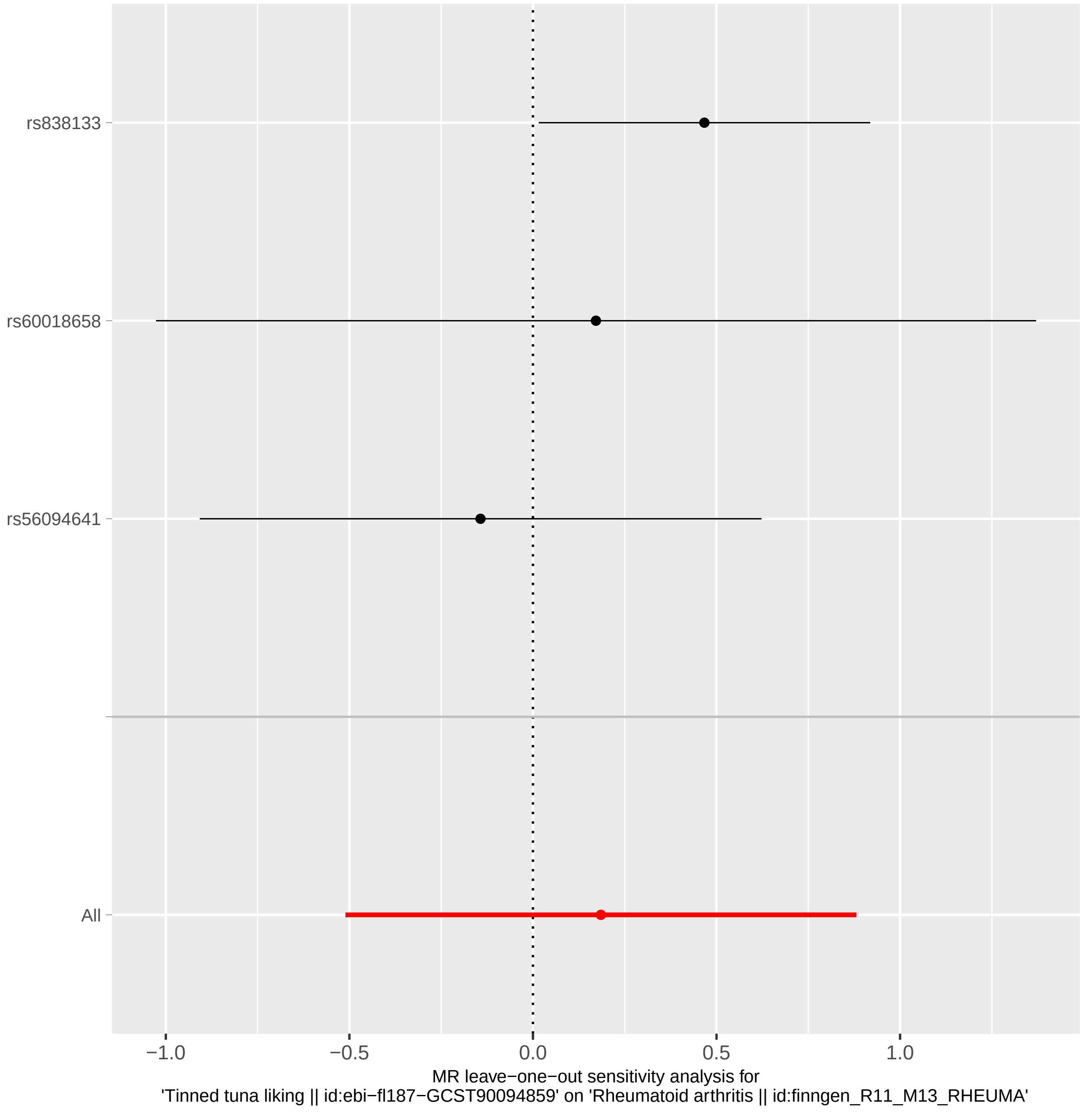

rs838133
rs60018658
rs56094641
All
−1.0
−0.5
0.0
0.5
1.0
MR leave−one−out sensitivity analysis for
'Tinned tuna liking || id:ebi−fl187−GCST90094859' on 'Rheumatoid arthritis || id:finngen_R11_M13_RHEUMA'

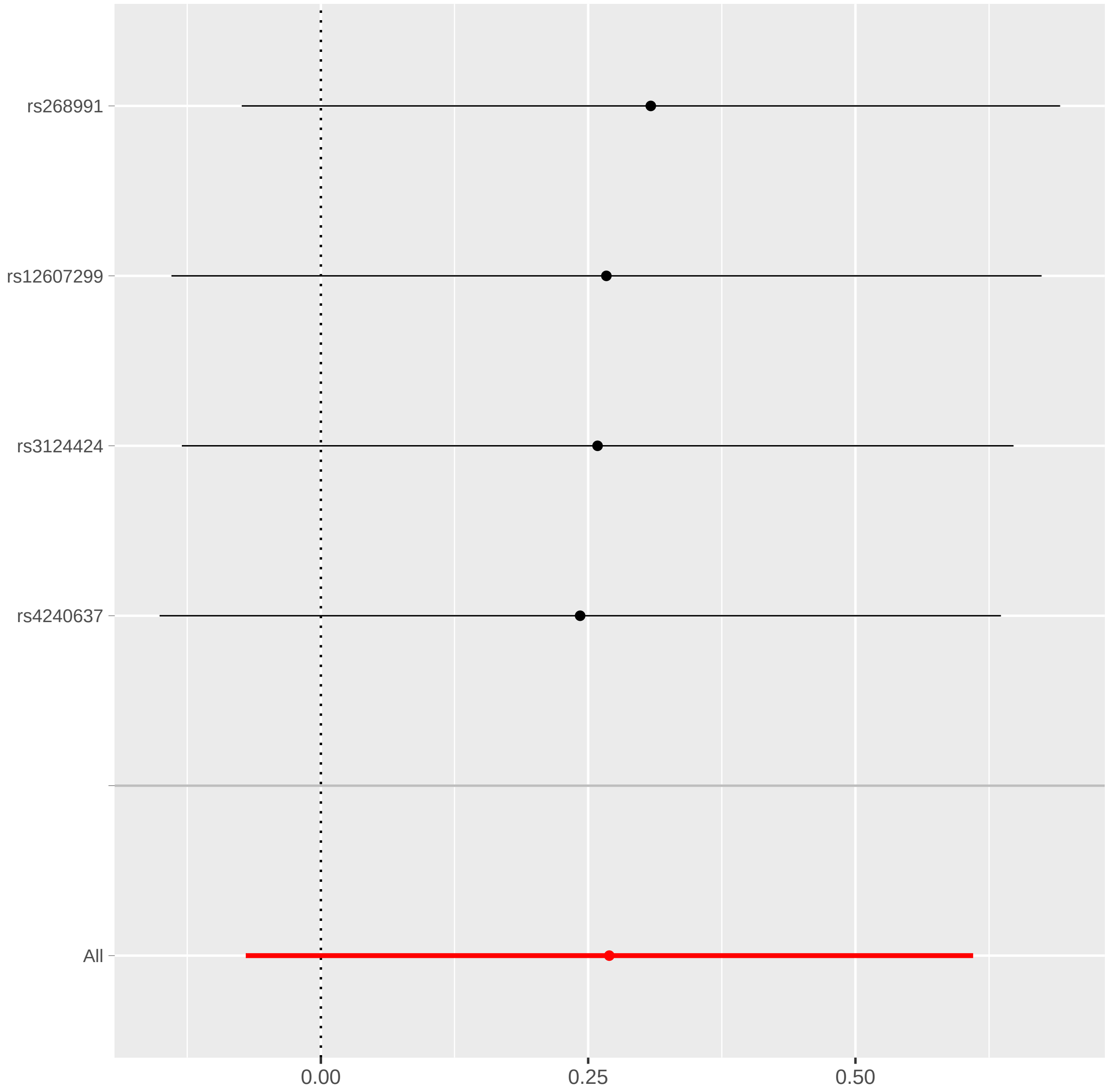

MR leave−one−out sensitivity analysis for
'Tomatoes liking || id:ebi−fl187−GCST90094860' on 'Rheumatoid arthritis || id:finngen_R11_M13_RHEUMA'

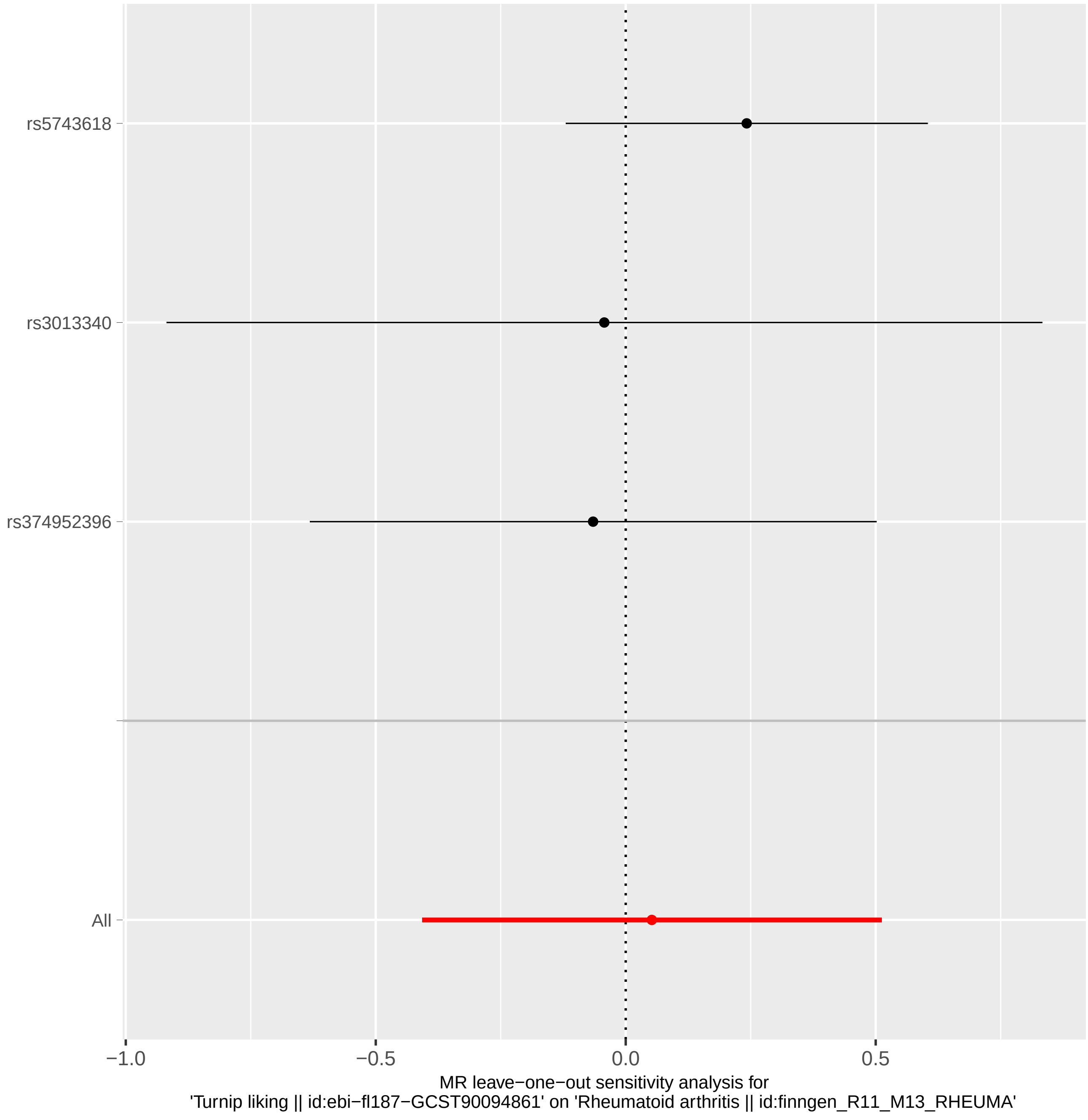
rs5743618
rs3013340
rs374952396
All
−1.0
−0.5
0.0
0.5
MR leave−one−out sensitivity analysis for
'Turnip liking || id:ebi−fl187−GCST90094861' on 'Rheumatoid arthritis || id:finngen_R11_M13_RHEUMA'

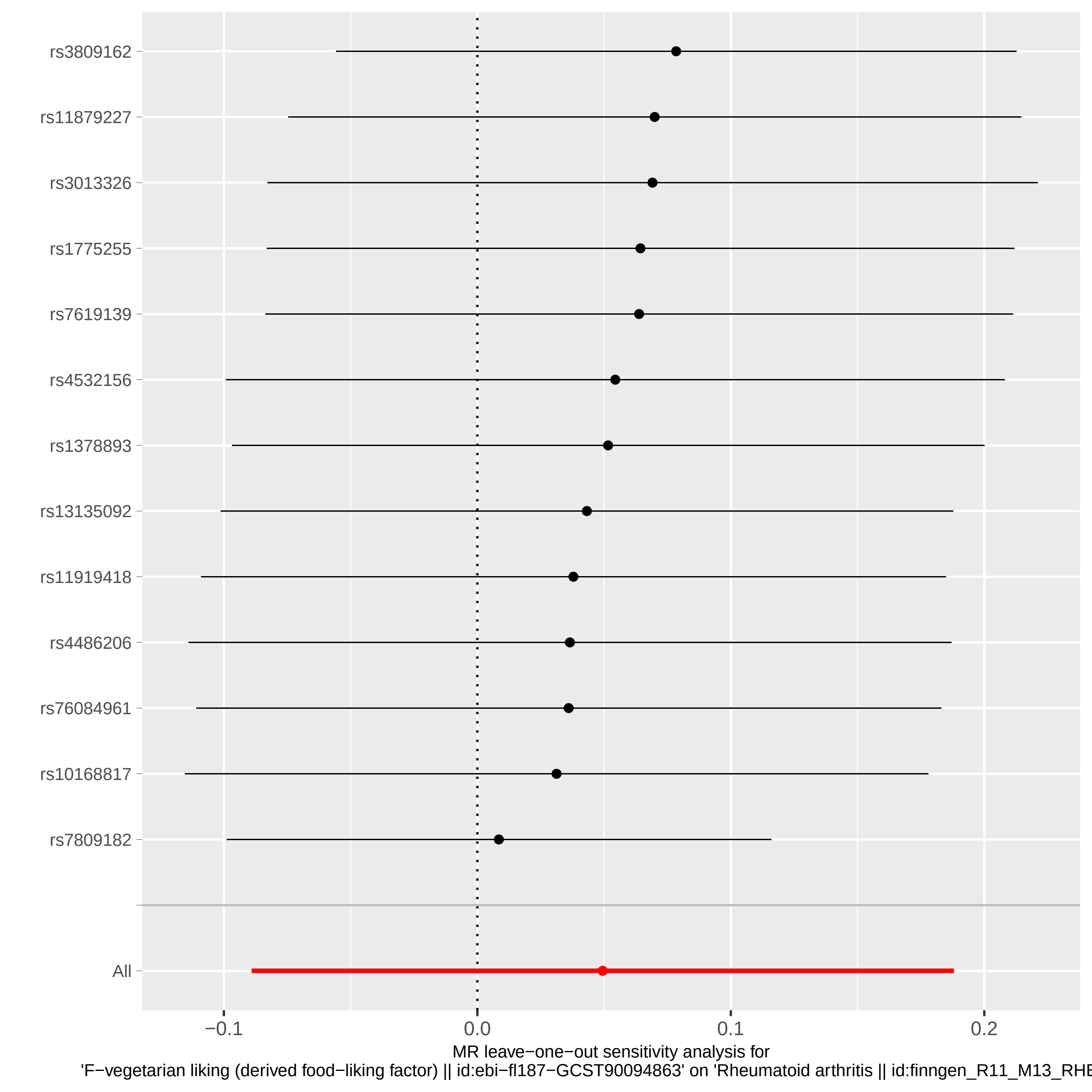
rs3809162
rs11879227
rs3013326
rs1775255
rs7619139
rs4532156
rs1378893
rs13135092
rs11919418
rs4486206
rs76084961
rs10168817
rs7809182
All
−0.1
0.0
0.1
0.2
MR leave−one−out sensitivity analysis for
'F−vegetarian liking (derived food−liking factor) || id:ebi−fl187−GCST90094863' on 'Rheumatoid arthritis || id:finngen_R11_M13_RHE

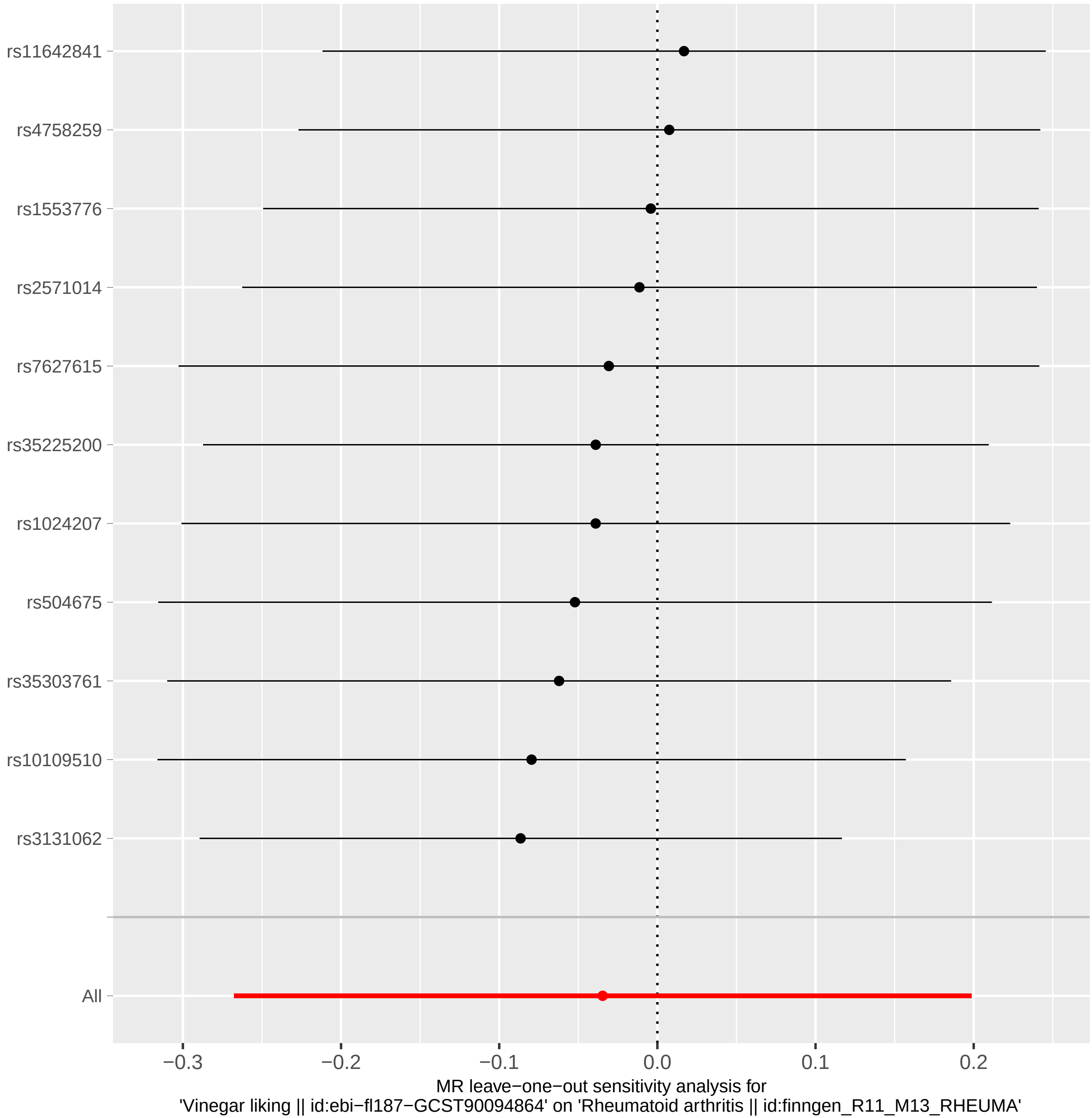
rs11642841
rs4758259
rs1553776
rs2571014
rs7627615
rs35225200
rs1024207
rs504675
rs35303761
rs10109510
rs3131062
All
−0.3
−0.2
−0.1
0.0
0.1
0.2
MR leave−one−out sensitivity analysis for
'Vinegar liking || id:ebi−fl187−GCST90094864' on 'Rheumatoid arthritis || id:finngen_R11_M13_RHEUMA'

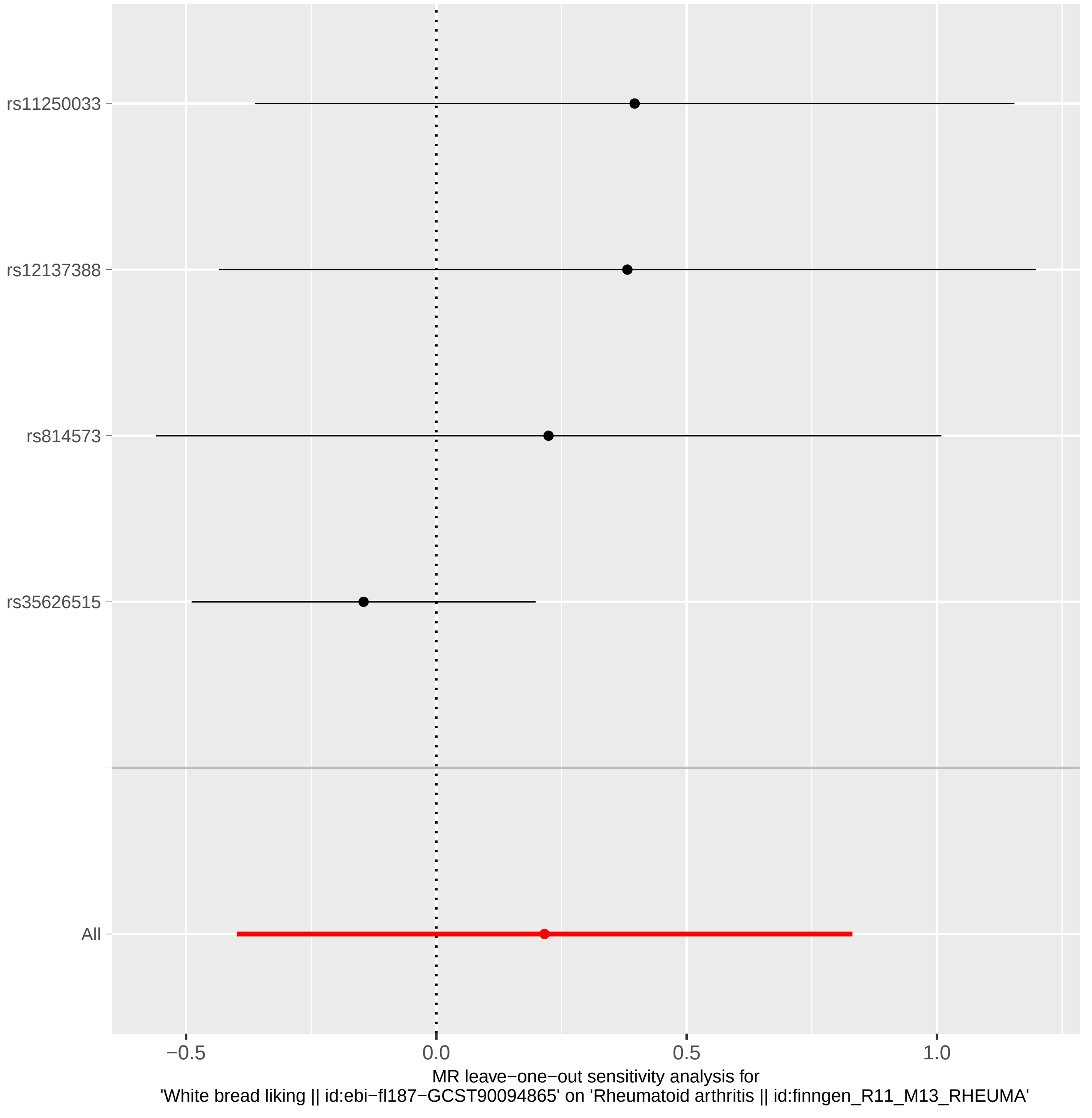
rs11250033
rs12137388
rs814573
rs35626515
All
−0.5
0.0
0.5
1.0
MR leave−one−out sensitivity analysis for
'White bread liking || id:ebi−fl187−GCST90094865' on 'Rheumatoid arthritis || id:finngen_R11_M13_RHEUMA'

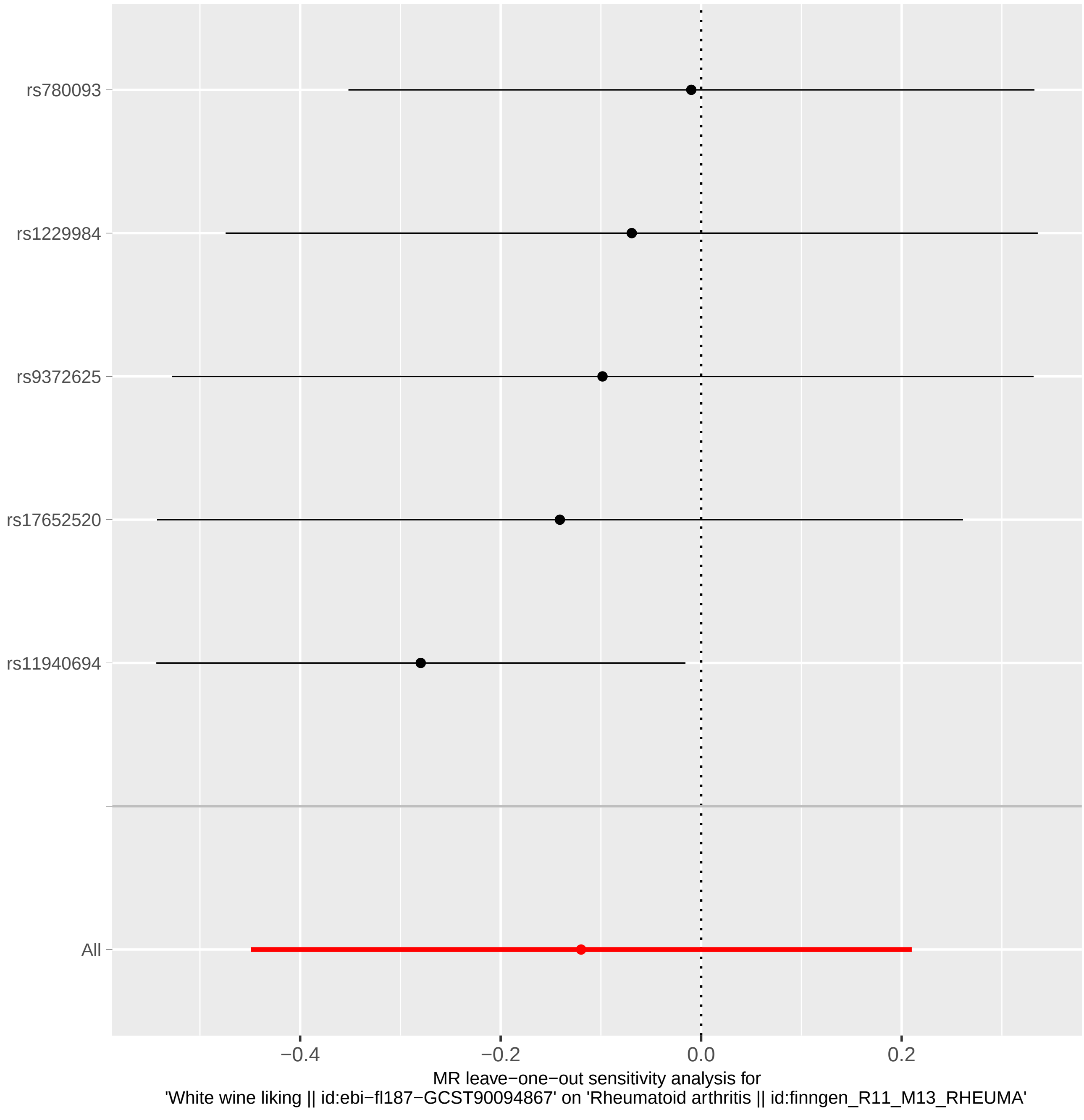
rs780093
rs1229984
rs9372625
rs17652520
rs11940694
All
−0.4
−0.2
0.0
0.2
MR leave−one−out sensitivity analysis for
'White wine liking || id:ebi−fl187−GCST90094867' on 'Rheumatoid arthritis || id:finngen_R11_M13_RHEUMA'

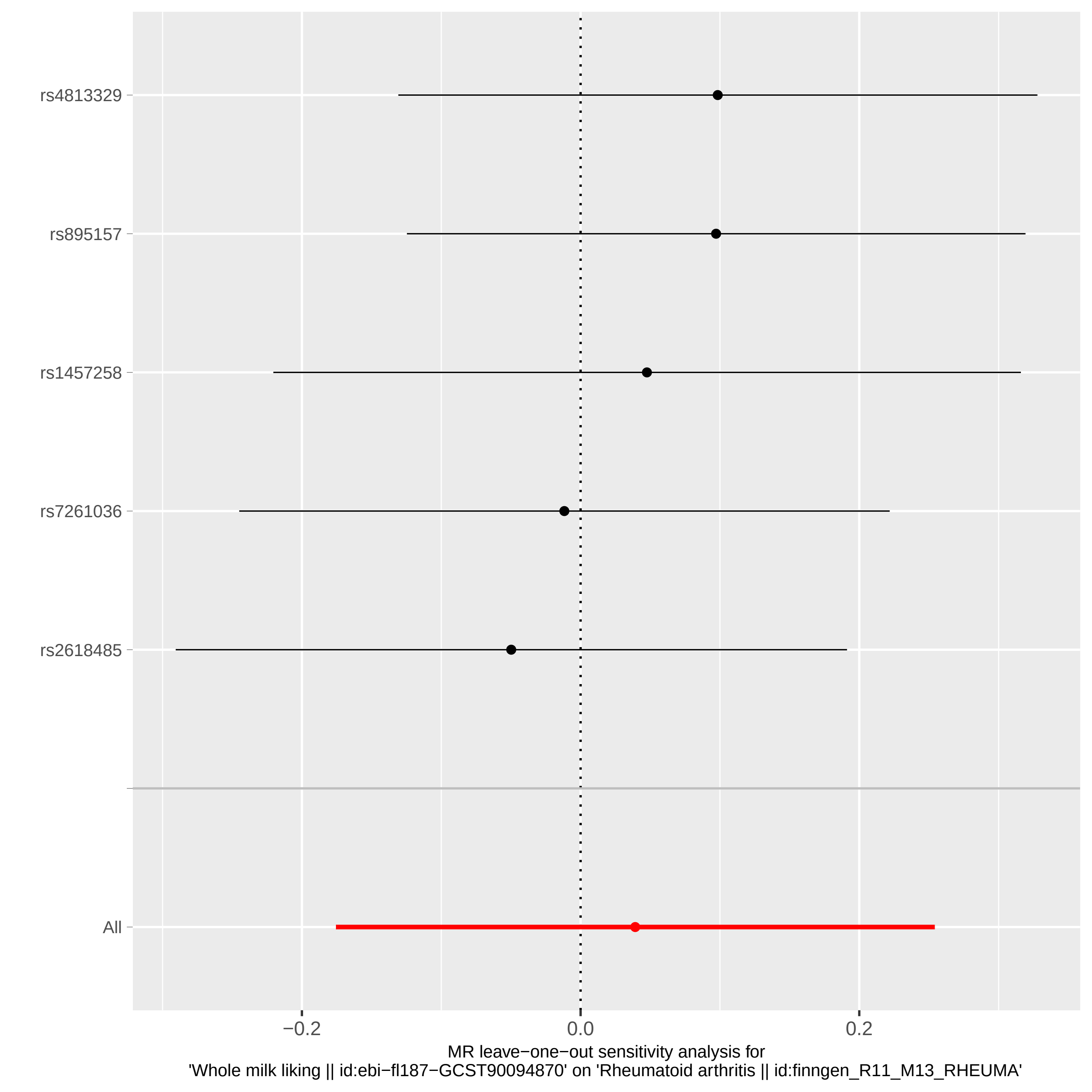
rs4813329
rs895157
rs1457258
rs7261036
rs2618485
All
−0.2
0.0
0.2
MR leave−one−out sensitivity analysis for
'Whole milk liking || id:ebi−fl187−GCST90094870' on 'Rheumatoid arthritis || id:finngen_R11_M13_RHEUMA'

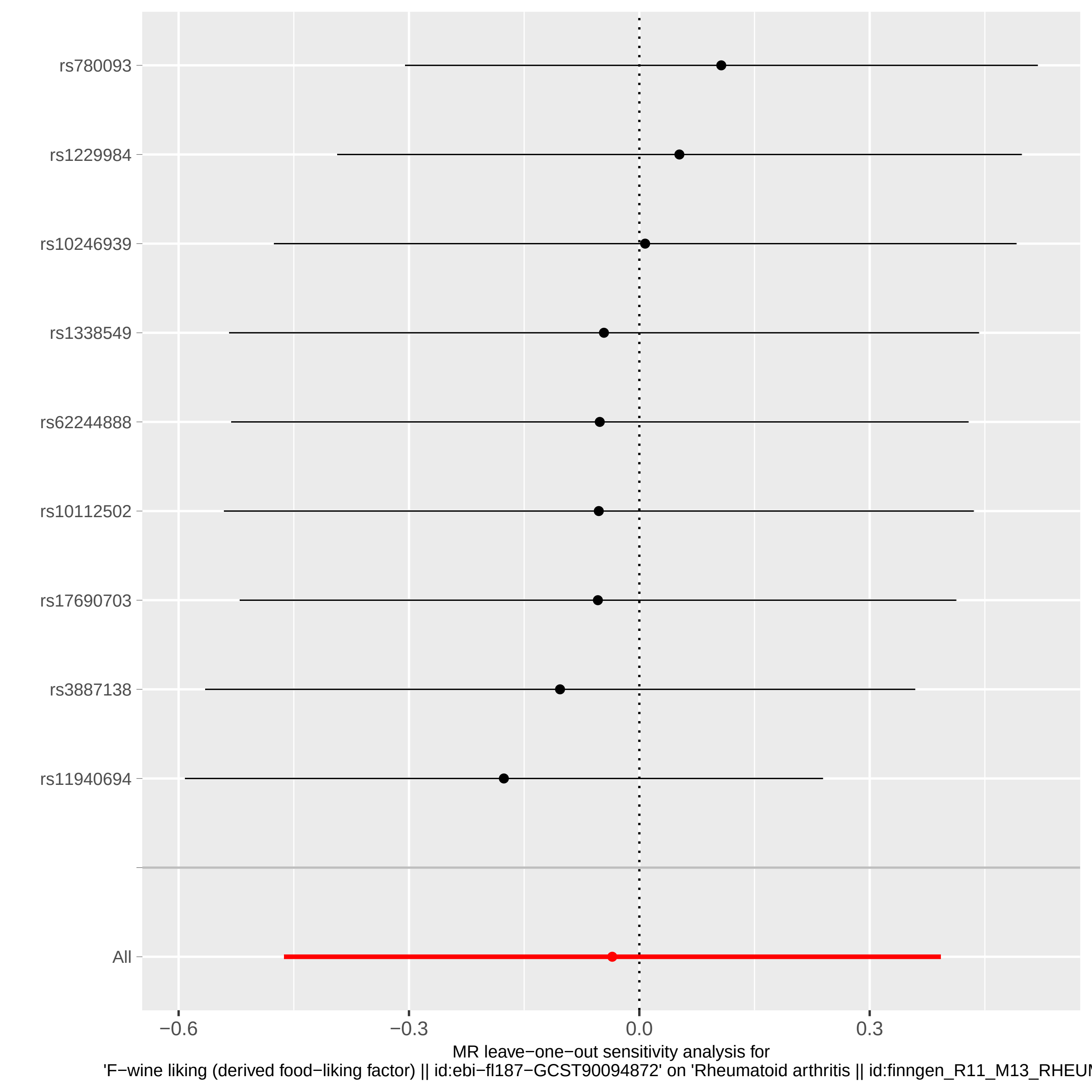
rs780093
rs1229984
rs10246939
rs1338549
rs62244888
rs10112502
rs17690703
rs3887138
rs11940694
All
−0.6
−0.3
0.0
0.3
MR leave−one−out sensitivity analysis for
'F−wine liking (derived food−liking factor) || id:ebi−fl187−GCST90094872' on 'Rheumatoid arthritis || id:finngen_R11_M13_RHEUM

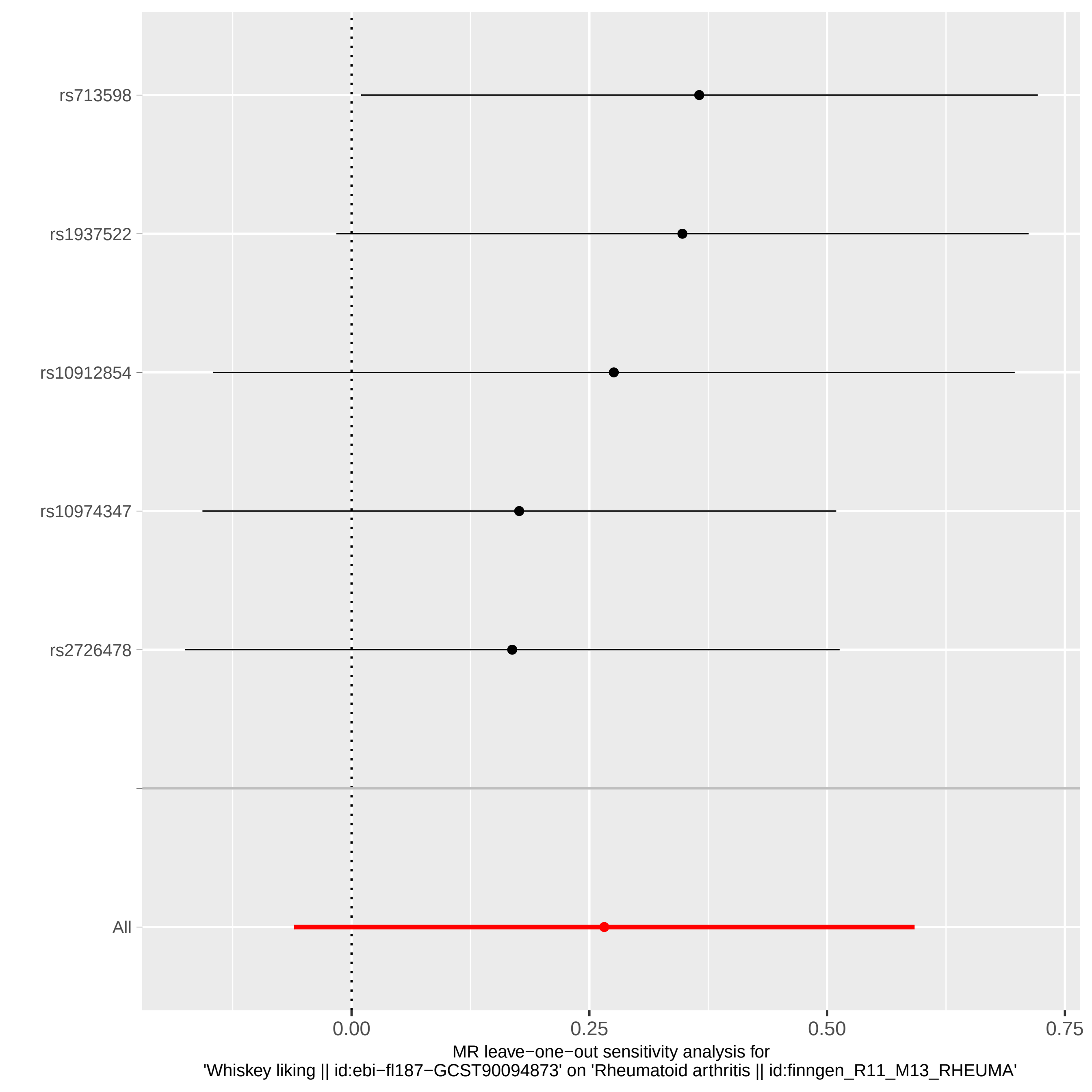

rs713598
rs1937522
rs10912854
rs10974347
rs2726478
All
0.00
0.25
0.50
0.75
MR leave−one−out sensitivity analysis for
'Whiskey liking || id:ebi−fl187−GCST90094873' on 'Rheumatoid arthritis || id:finngen_R11_M13_RHEUMA'

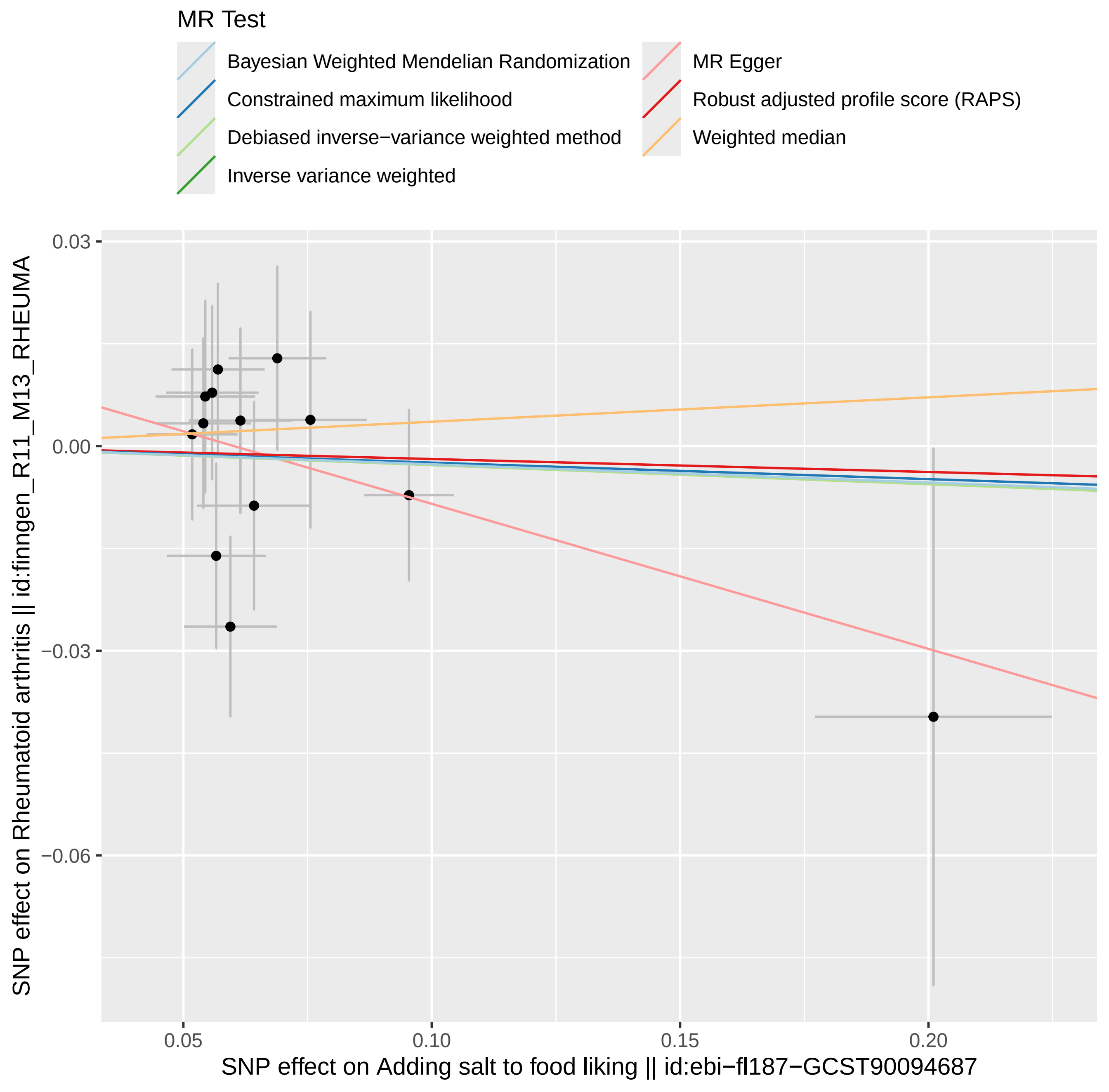
MR Test
Bayesian Weighted Mendelian Randomization
Constrained maximum likelihood
Debiased inverse−variance weighted method
Inverse variance weighted
MR Egger
Robust adjusted profile score (RAPS)
Weighted median
0.03
0.00
−0.03
−0.06
0.05
0.10
0.15
0.20
SNP effect on Rheumatoid arthritis || id:finngen_R11_M13_RHEUMA
SNP effect on Adding salt to food liking || id:ebi−fl187−GCST90094687

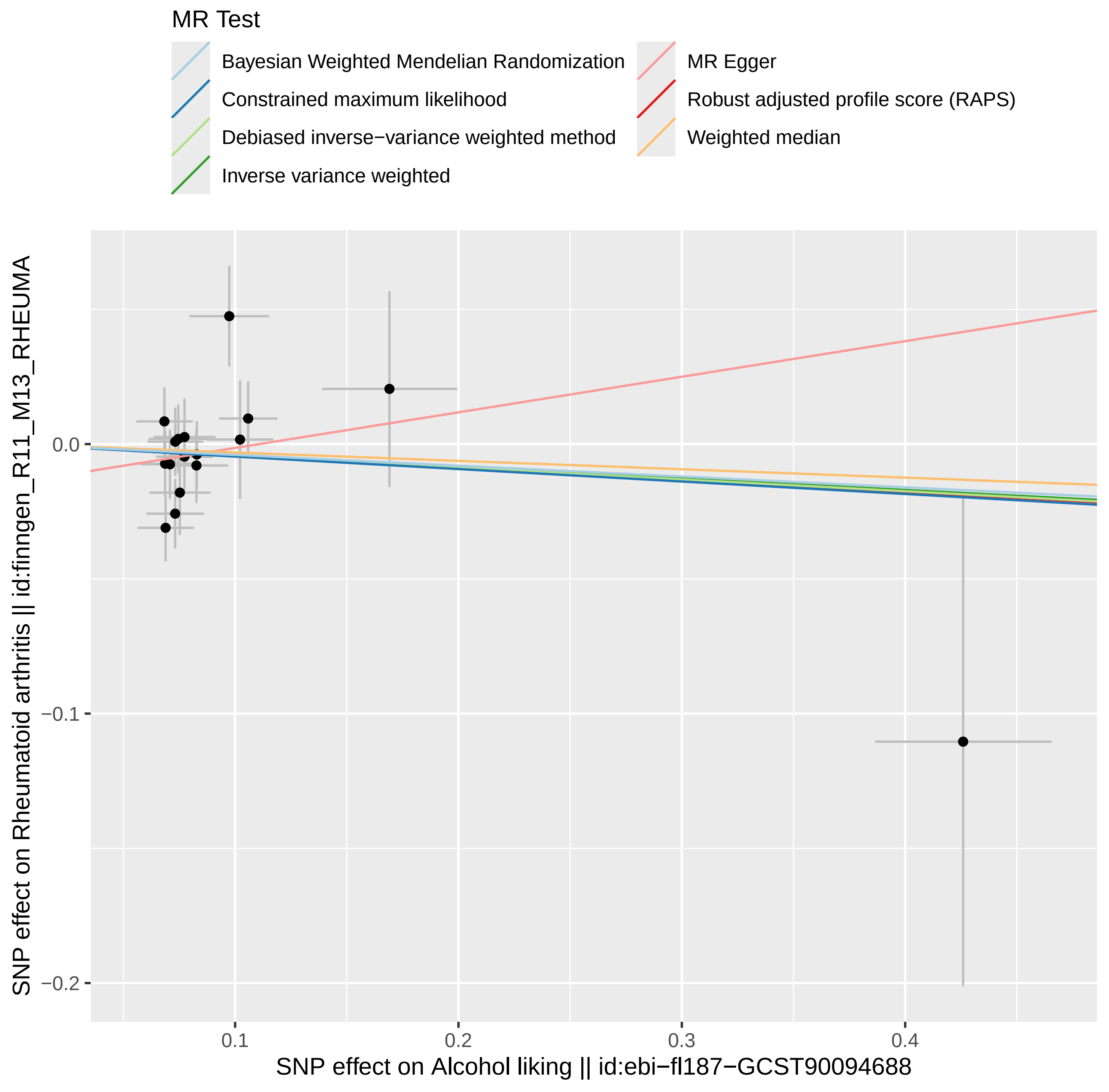

MR Test
Bayesian Weighted Mendelian Randomization
Constrained maximum likelihood
Debiased inverse−variance weighted method
Inverse variance weighted
MR Egger
Robust adjusted profile score (RAPS)
Weighted median
SNP effect on Rheumatoid arthritis || id:finngen_R11_M13_RHEUMA
0.0
−0.1
−0.2
0.1
0.2
0.3
0.4
SNP effect on Alcohol liking || id:ebi−fl187−GCST90094688

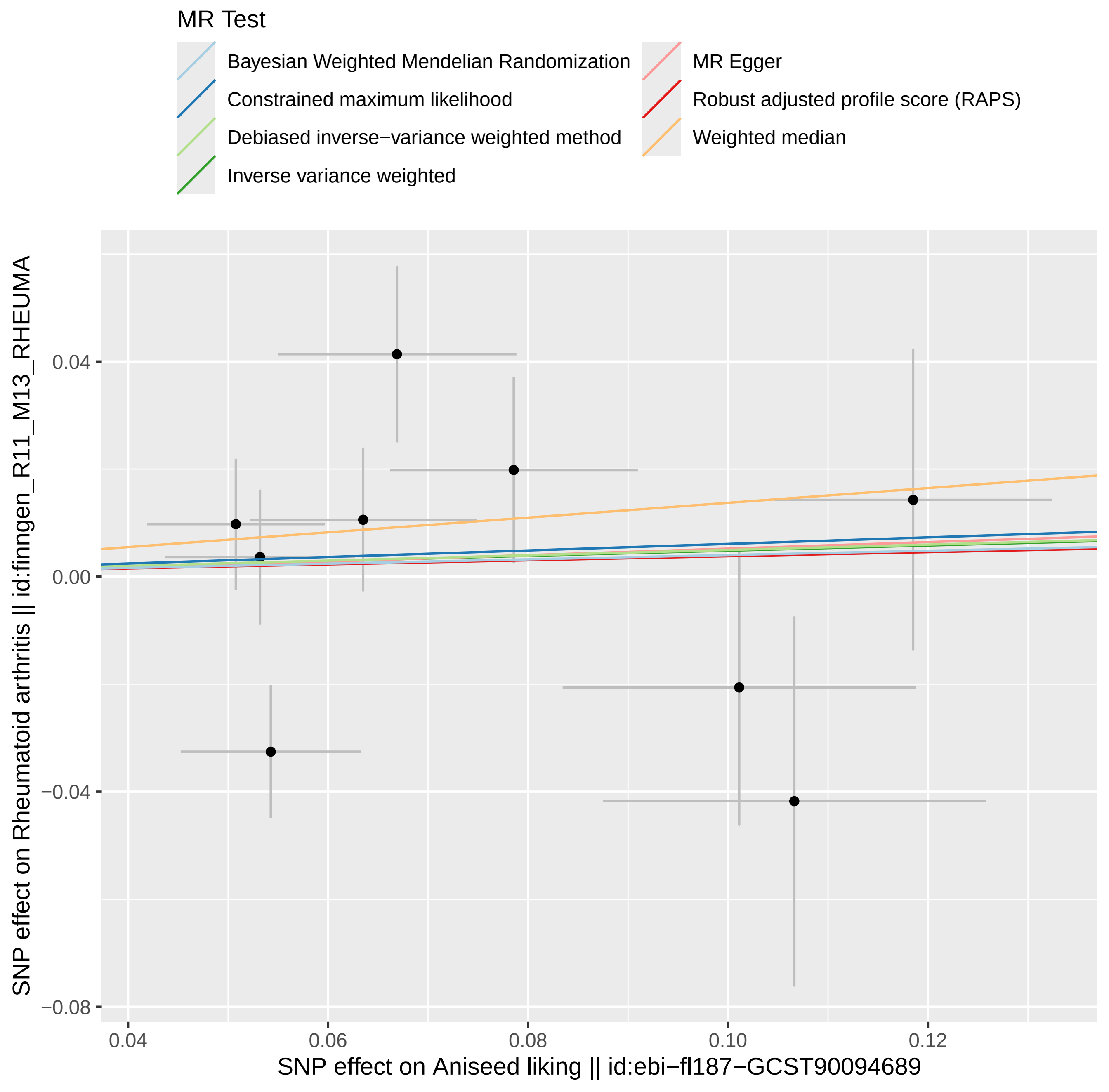

MR Test
Bayesian Weighted Mendelian Randomization
Constrained maximum likelihood
Debiased inverse−variance weighted method
Inverse variance weighted
MR Egger
Robust adjusted profile score (RAPS)
Weighted median
0.04
0.00
−0.04
−0.08
0.04
0.06
0.08
0.10
0.12
SNP effect on Rheumatoid arthritis || id:finngen_R11_M13_RHEUMA
SNP effect on Aniseed liking || id:ebi−fl187−GCST90094689

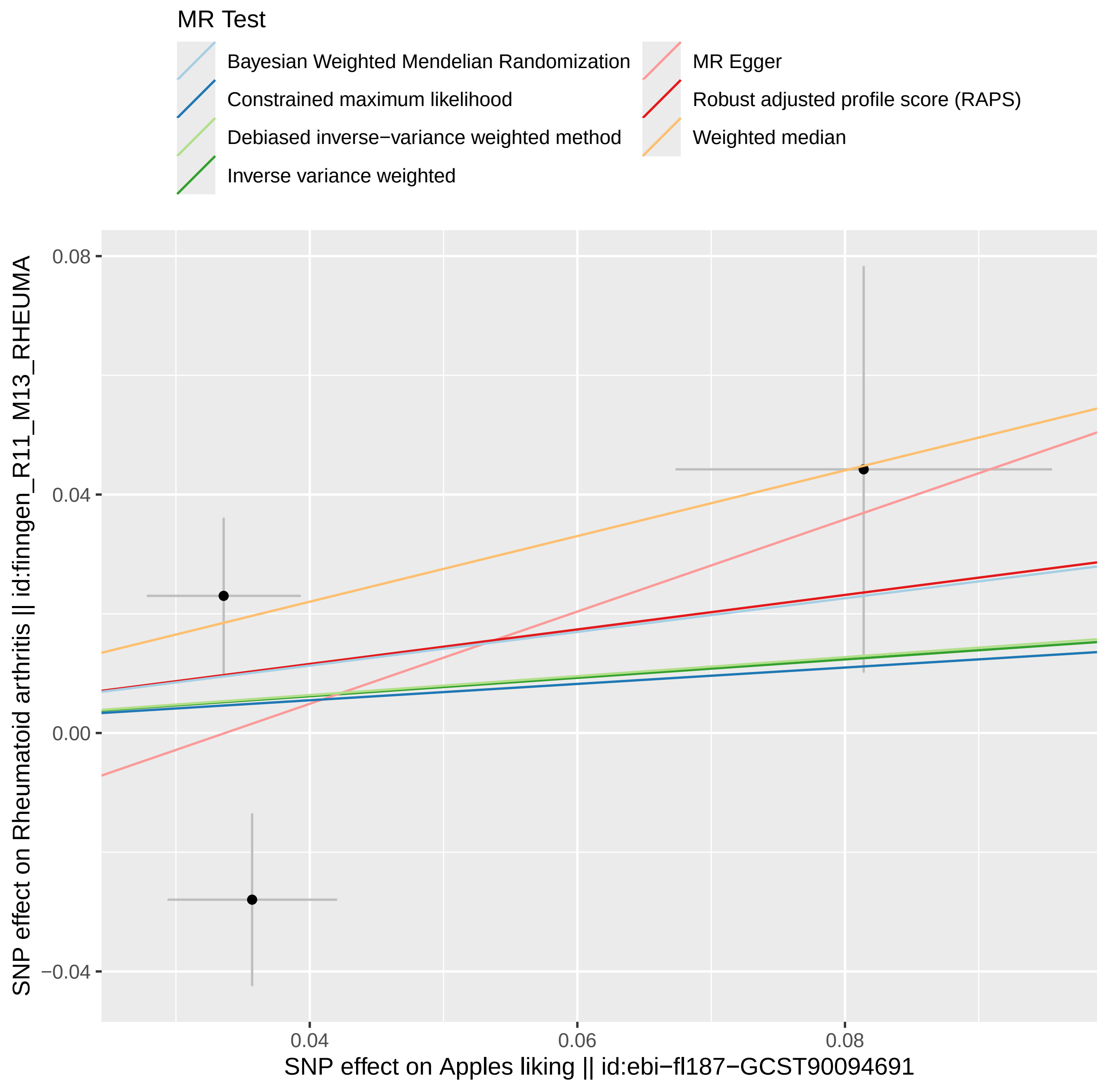

MR Test
Bayesian Weighted Mendelian Randomization
Constrained maximum likelihood
Debiased inverse−variance weighted method
Inverse variance weighted
MR Egger
Robust adjusted profile score (RAPS)
Weighted median
0.08
0.04
0.00
−0.04
0.04
0.06
0.08
SNP effect on Rheumatoid arthritis || id:finngen_R11_M13_RHEUMA
SNP effect on Apples liking || id:ebi−fl187−GCST90094691

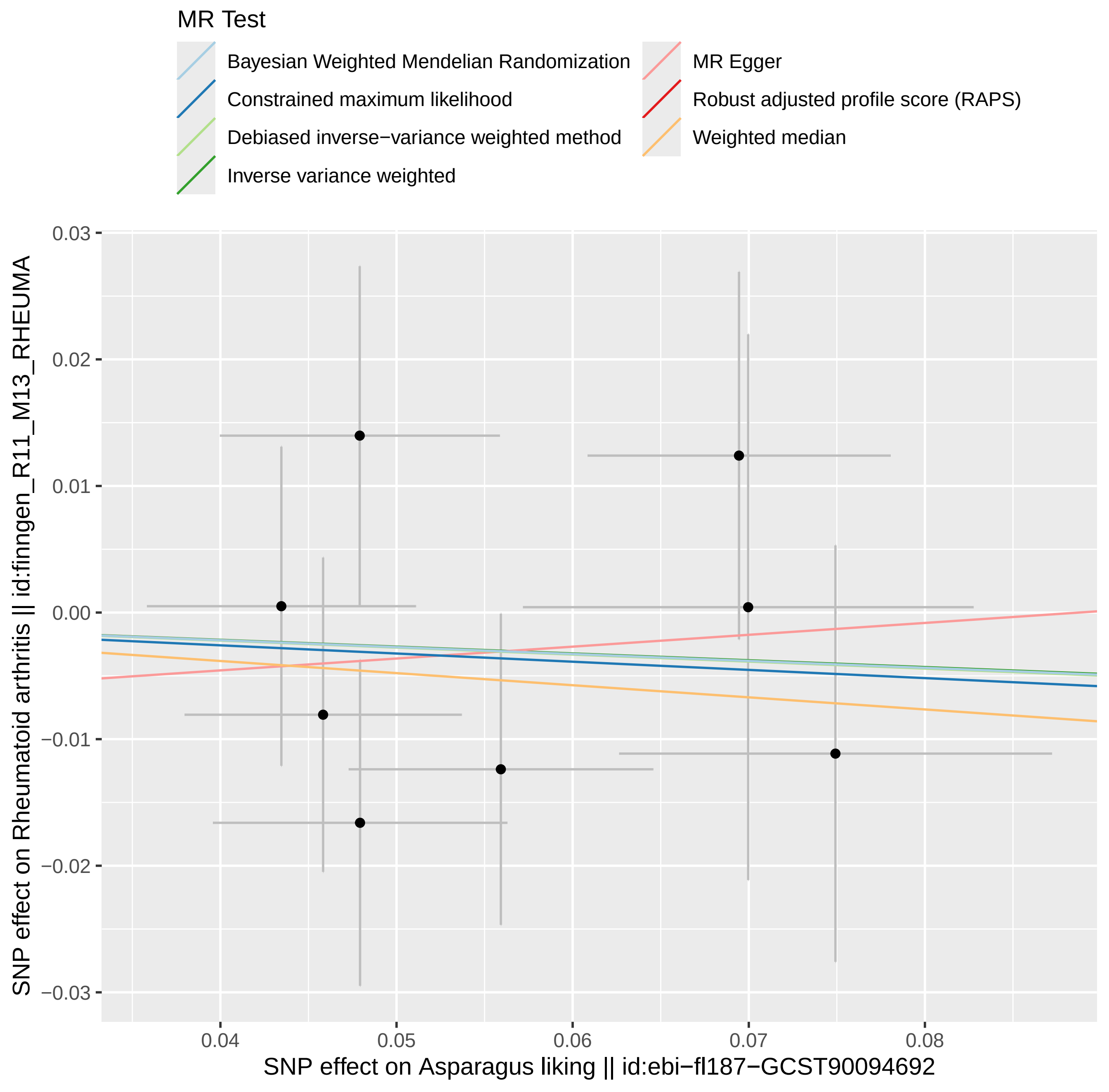
MR Test
Bayesian Weighted Mendelian Randomization
Constrained maximum likelihood
Debiased inverse−variance weighted method
Inverse variance weighted
MR Egger
Robust adjusted profile score (RAPS)
Weighted median
0.03
0.02
0.01
0.00
−0.01
−0.02
−0.03
0.04
0.05
0.06
0.07
0.08
SNP effect on Rheumatoid arthritis || id:finngen_R11_M13_RHEUMA
SNP effect on Asparagus liking || id:ebi−fl187−GCST90094692

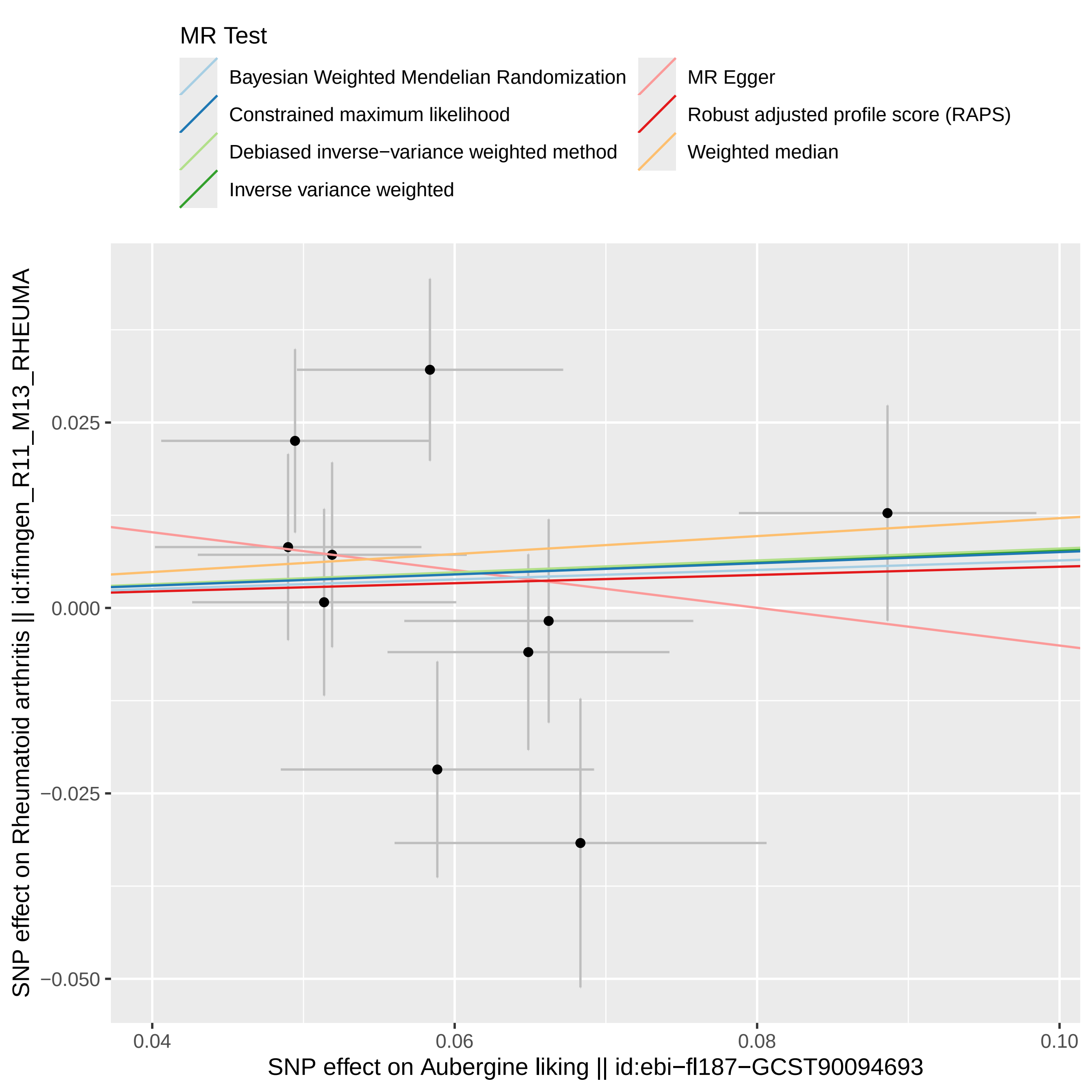

MR Test
Bayesian Weighted Mendelian Randomization
Constrained maximum likelihood
Debiased inverse−variance weighted method
Inverse variance weighted
MR Egger
Robust adjusted profile score (RAPS)
Weighted median
SNP effect on Rheumatoid arthritis || id:finngen_R11_M13_RHEUMA
0.025
0.000
−0.025
−0.050
0.04
0.06
0.08
0.10
SNP effect on Aubergine liking || id:ebi−fl187−GCST90094693

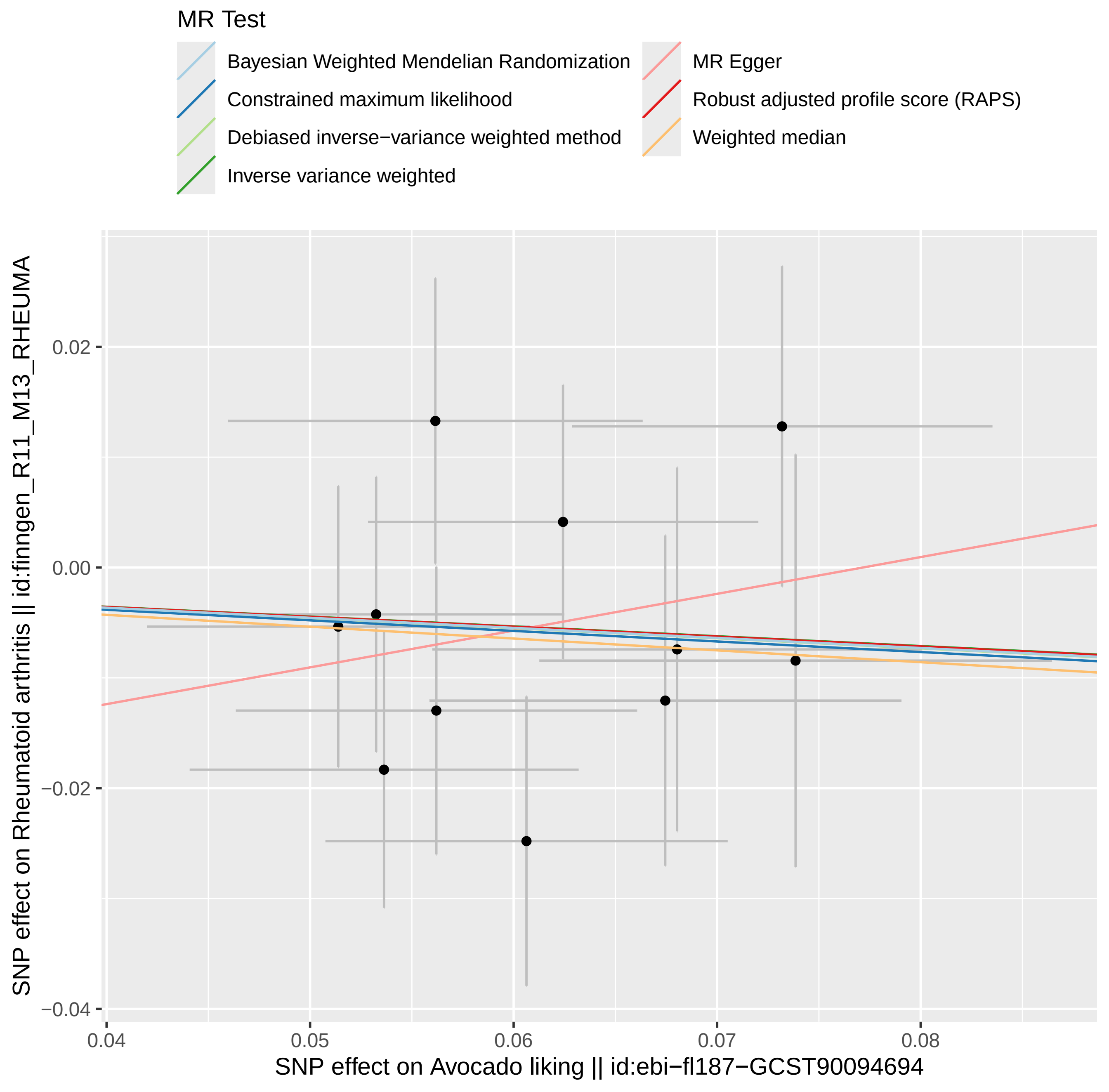

MR Test
Bayesian Weighted Mendelian Randomization
Constrained maximum likelihood
Debiased inverse−variance weighted method
Inverse variance weighted
MR Egger
Robust adjusted profile score (RAPS)
Weighted median
SNP effect on Rheumatoid arthritis || id:finngen_R11_M13_RHEUMA
0.02
0.00
−0.02
−0.04
0.04
0.05
0.06
0.07
0.08
SNP effect on Avocado liking || id:ebi−fl187−GCST90094694

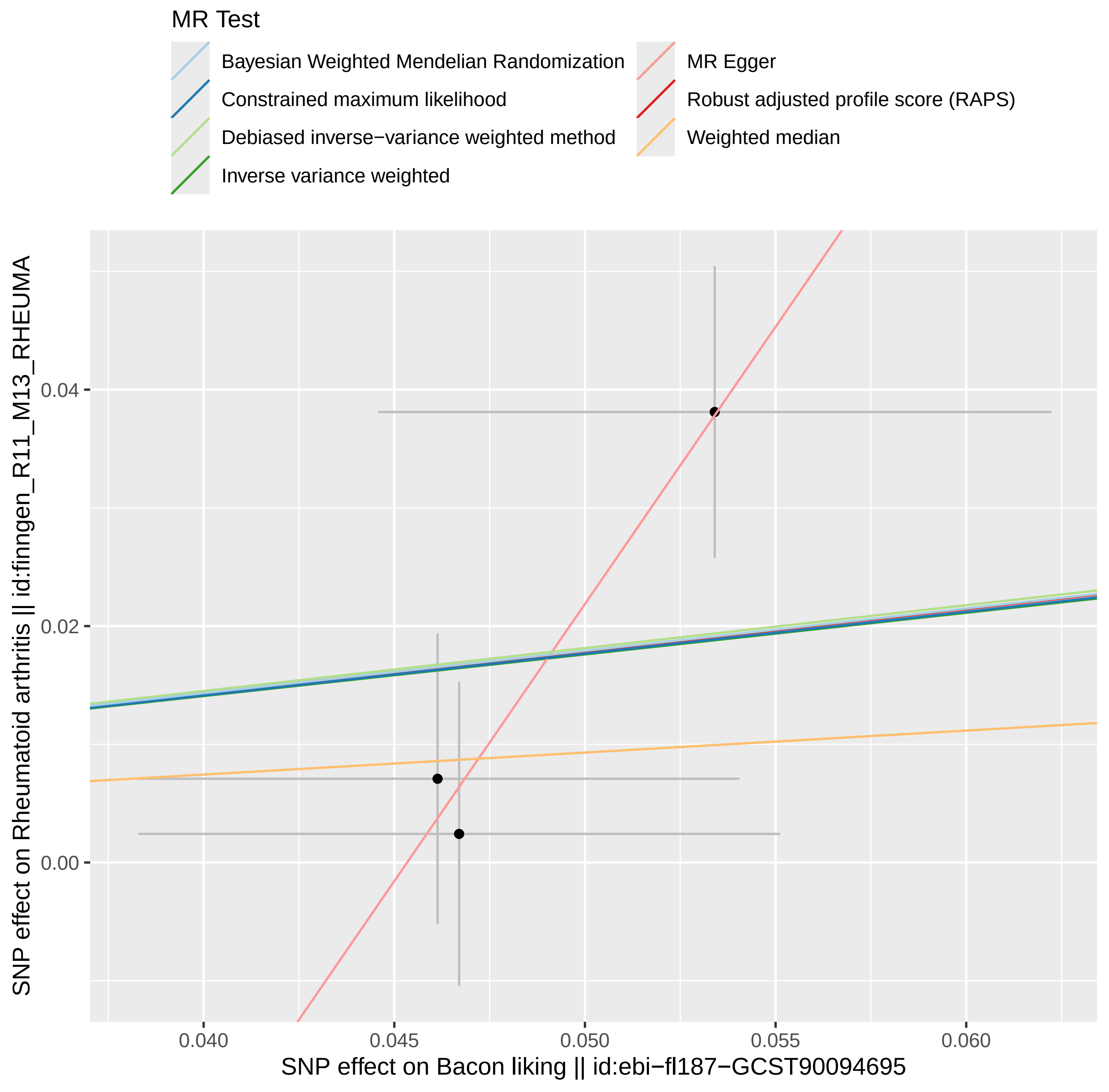

MR Test
Bayesian Weighted Mendelian Randomization
Constrained maximum likelihood
Debiased inverse−variance weighted method
Inverse variance weighted
MR Egger
Robust adjusted profile score (RAPS)
Weighted median
SNP effect on Rheumatoid arthritis || id:finngen_R11_M13_RHEUMA
0.04
0.02
0.00
0.040
0.045
0.050
0.055
0.060
SNP effect on Bacon liking || id:ebi−fl187−GCST90094695

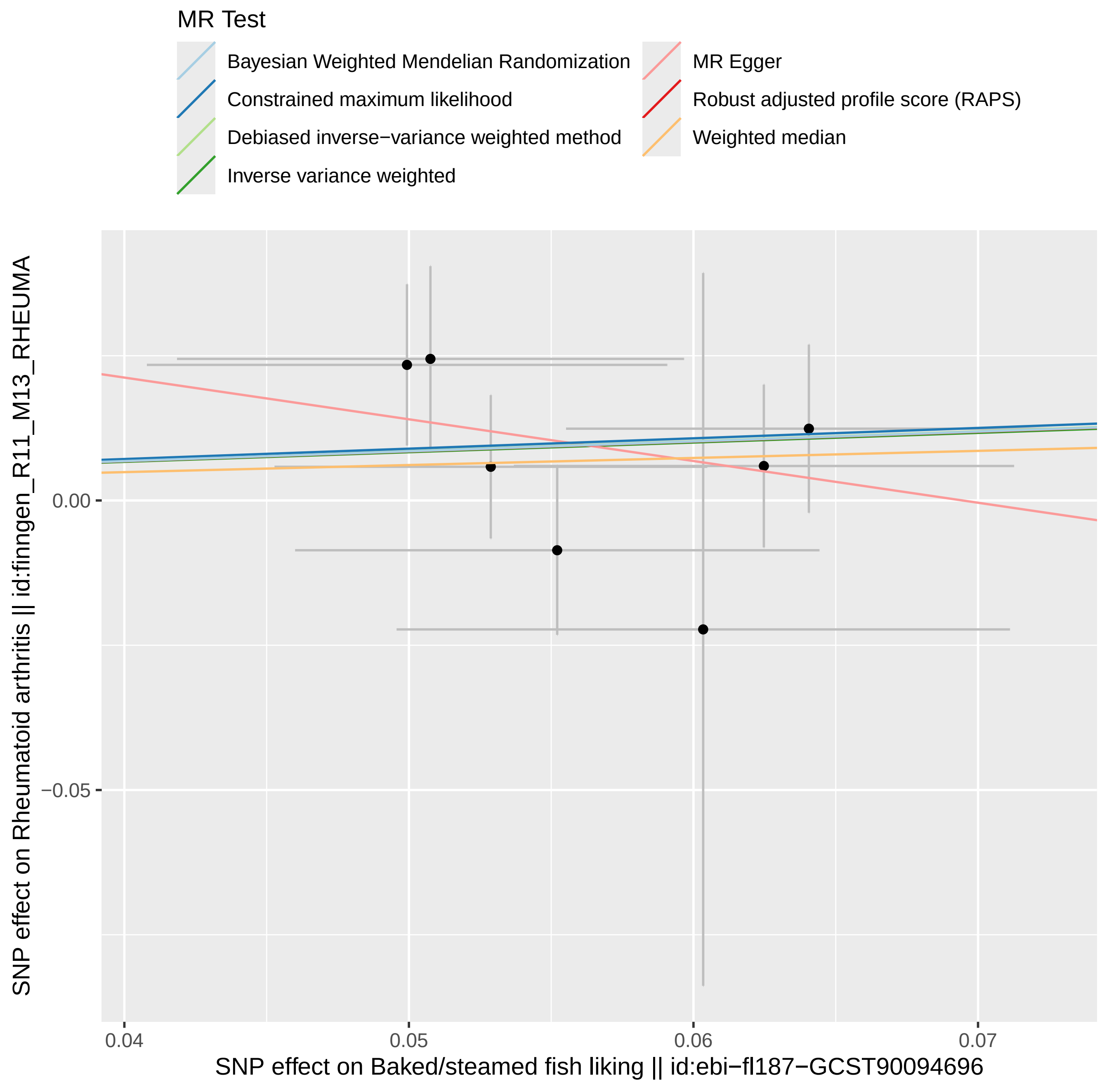
MR Test
Bayesian Weighted Mendelian Randomization
Constrained maximum likelihood
Debiased inverse−variance weighted method
Inverse variance weighted
MR Egger
Robust adjusted profile score (RAPS)
Weighted median
0.00
−0.05
0.04
0.05
0.06
0.07
SNP effect on Rheumatoid arthritis || id:finngen_R11_M13_RHEUMA
SNP effect on Baked/steamed fish liking || id:ebi−fI187−GCST90094696

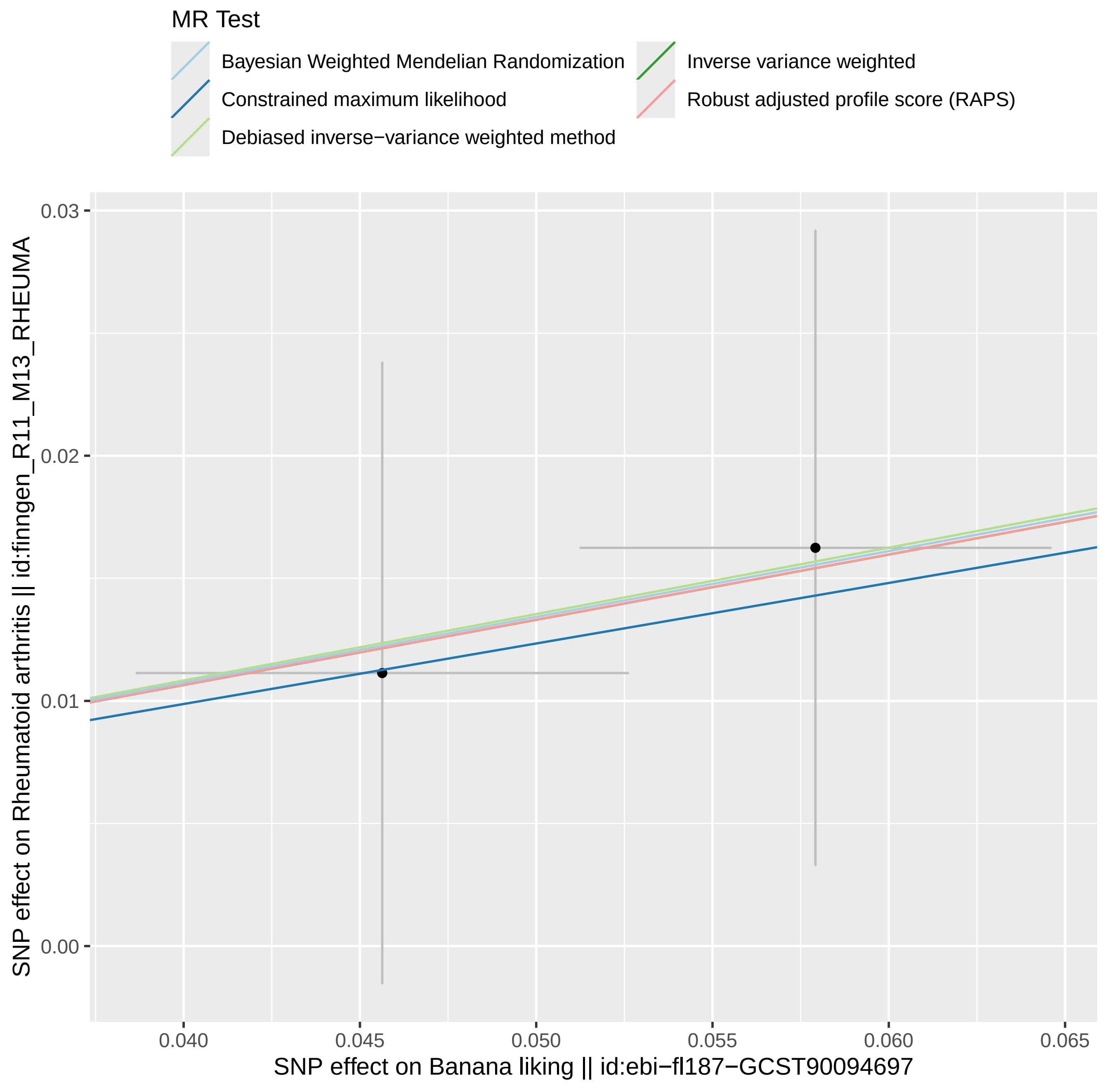
MR Test
Bayesian Weighted Mendelian Randomization
Constrained maximum likelihood
Debiased inverse−variance weighted method
Inverse variance weighted
Robust adjusted profile score (RAPS)
0.03
0.02
0.01
0.00
0.040
0.045
0.050
0.055
0.060
0.065
SNP effect on Rheumatoid arthritis || id:finngen_R11_M13_RHEUMA
SNP effect on Banana liking || id:ebi−fl187−GCST90094697

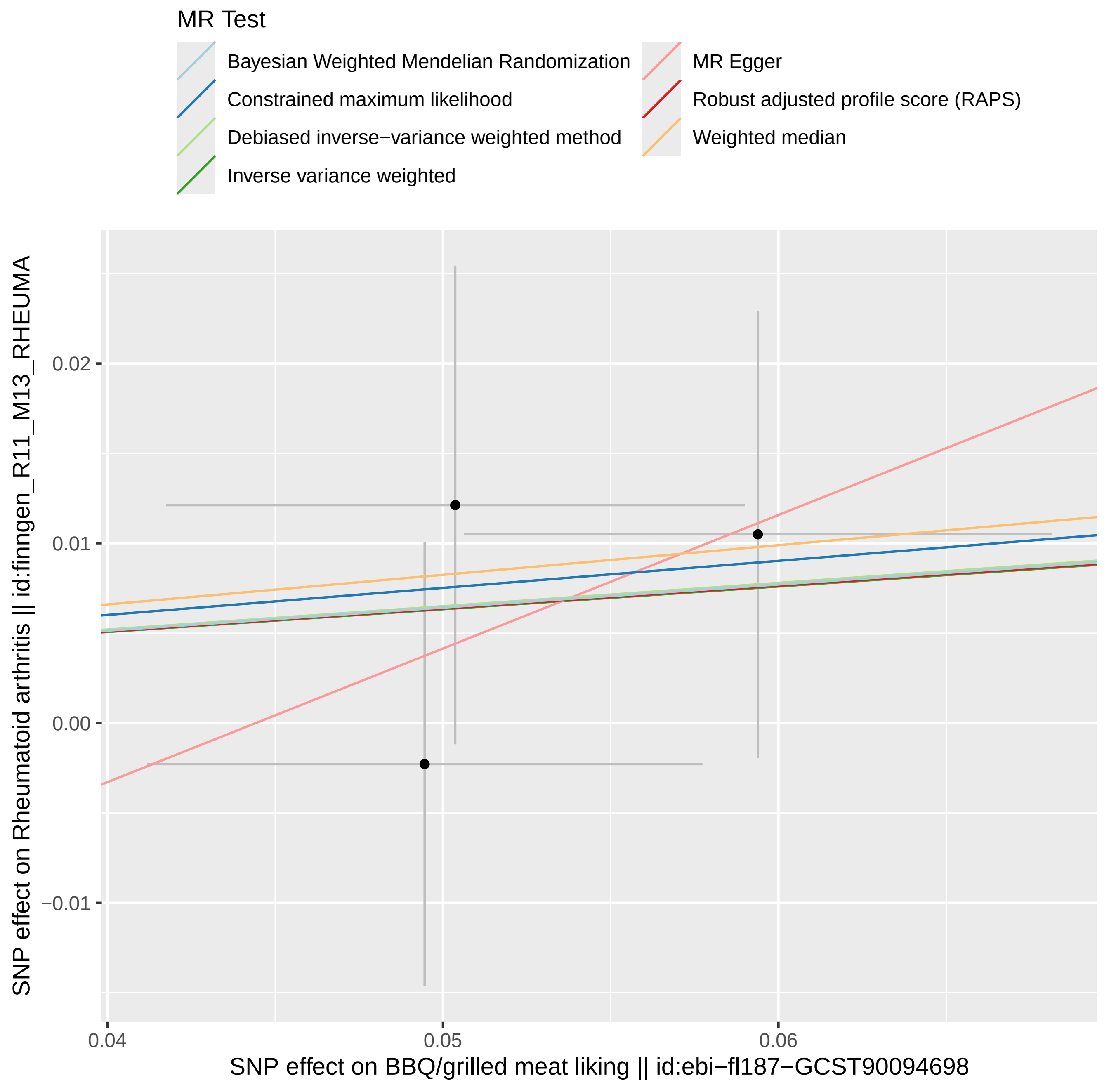
MR Test
Bayesian Weighted Mendelian Randomization
Constrained maximum likelihood
Debiased inverse−variance weighted method
Inverse variance weighted
MR Egger
Robust adjusted profile score (RAPS)
Weighted median
0.02
0.01
0.00
−0.01
0.04
0.05
0.06
SNP effect on Rheumatoid arthritis || id:finngen_R11_M13_RHEUMA
SNP effect on BBQ/grilled meat liking || id:ebi−fI187−GCST90094698

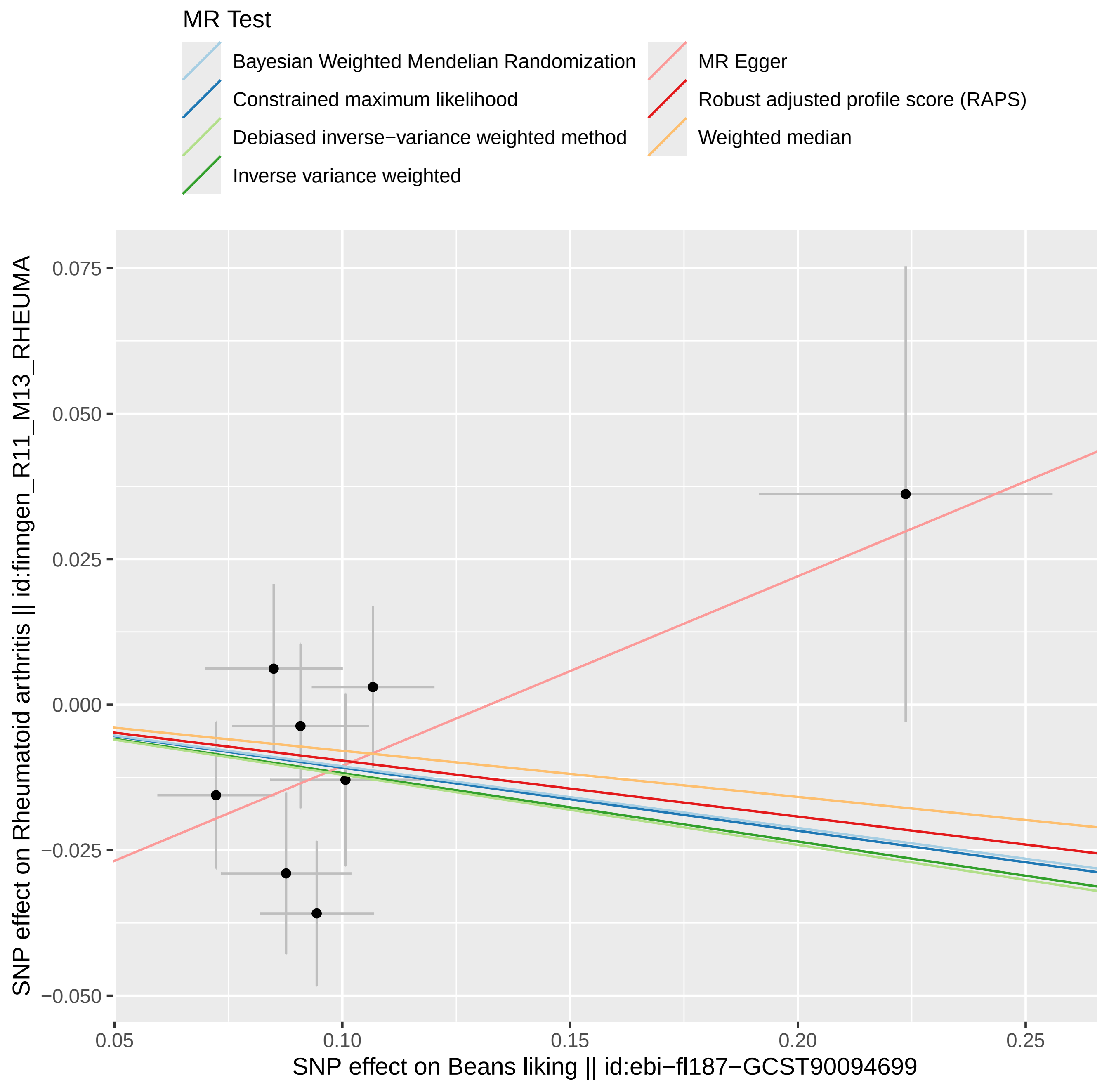
MR Test
Bayesian Weighted Mendelian Randomization
Constrained maximum likelihood
Debiased inverse−variance weighted method
Inverse variance weighted
MR Egger
Robust adjusted profile score (RAPS)
Weighted median
0.075
0.050
0.025
0.000
−0.025
−0.050
0.05
0.10
0.15
0.20
0.25
SNP effect on Rheumatoid arthritis || id:finngen_R11_M13_RHEUMA
SNP effect on Beans liking || id:ebi−fl187−GCST90094699

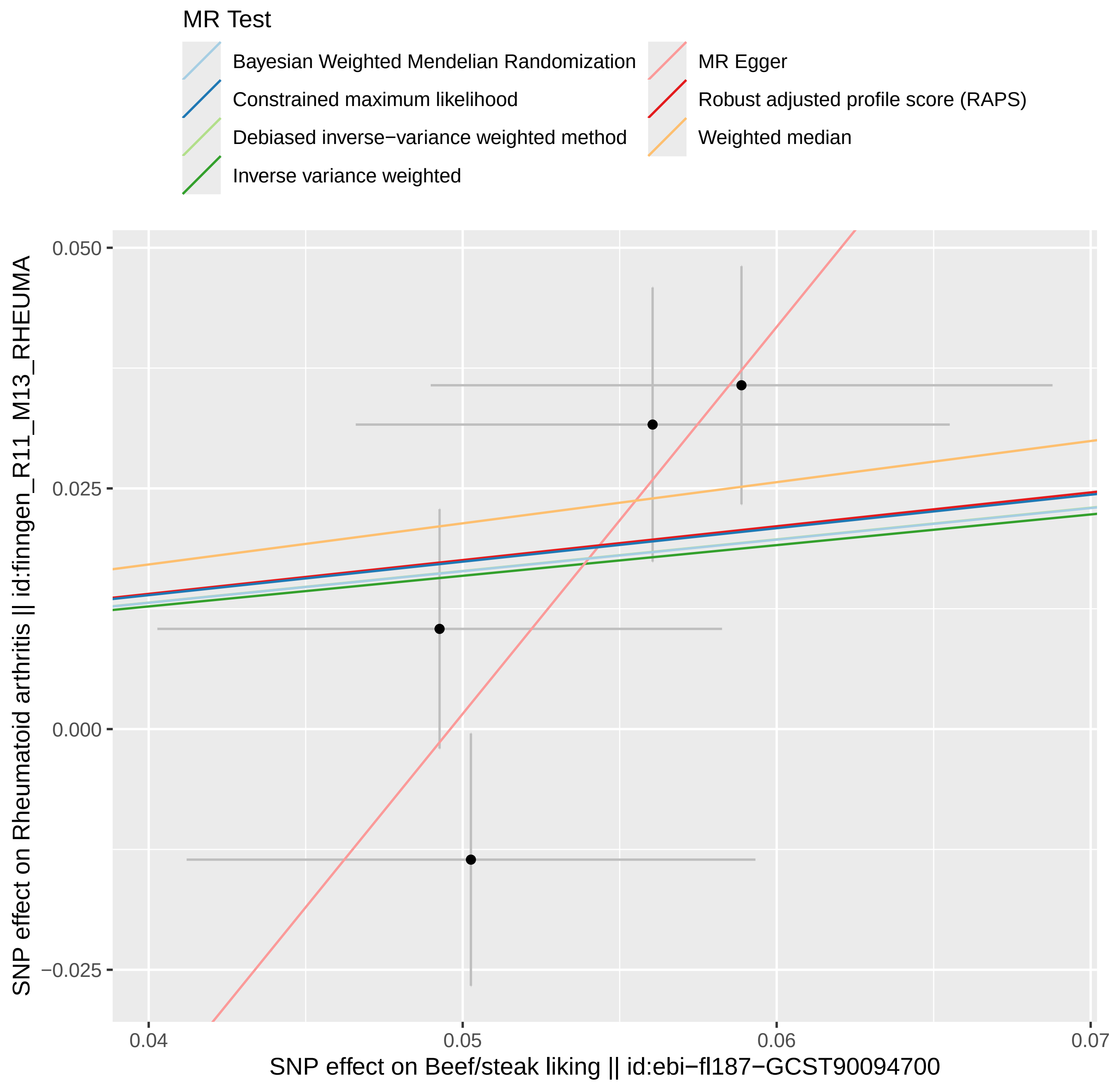

MR Test
Bayesian Weighted Mendelian Randomization
Constrained maximum likelihood
Debiased inverse−variance weighted method
Inverse variance weighted
MR Egger
Robust adjusted profile score (RAPS)
Weighted median
0.050
0.025
0.000
−0.025
0.04
0.05
0.06
0.07
SNP effect on Rheumatoid arthritis || id:finngen_R11_M13_RHEUMA
SNP effect on Beef/steak liking || id:ebi−fl187−GCST90094700

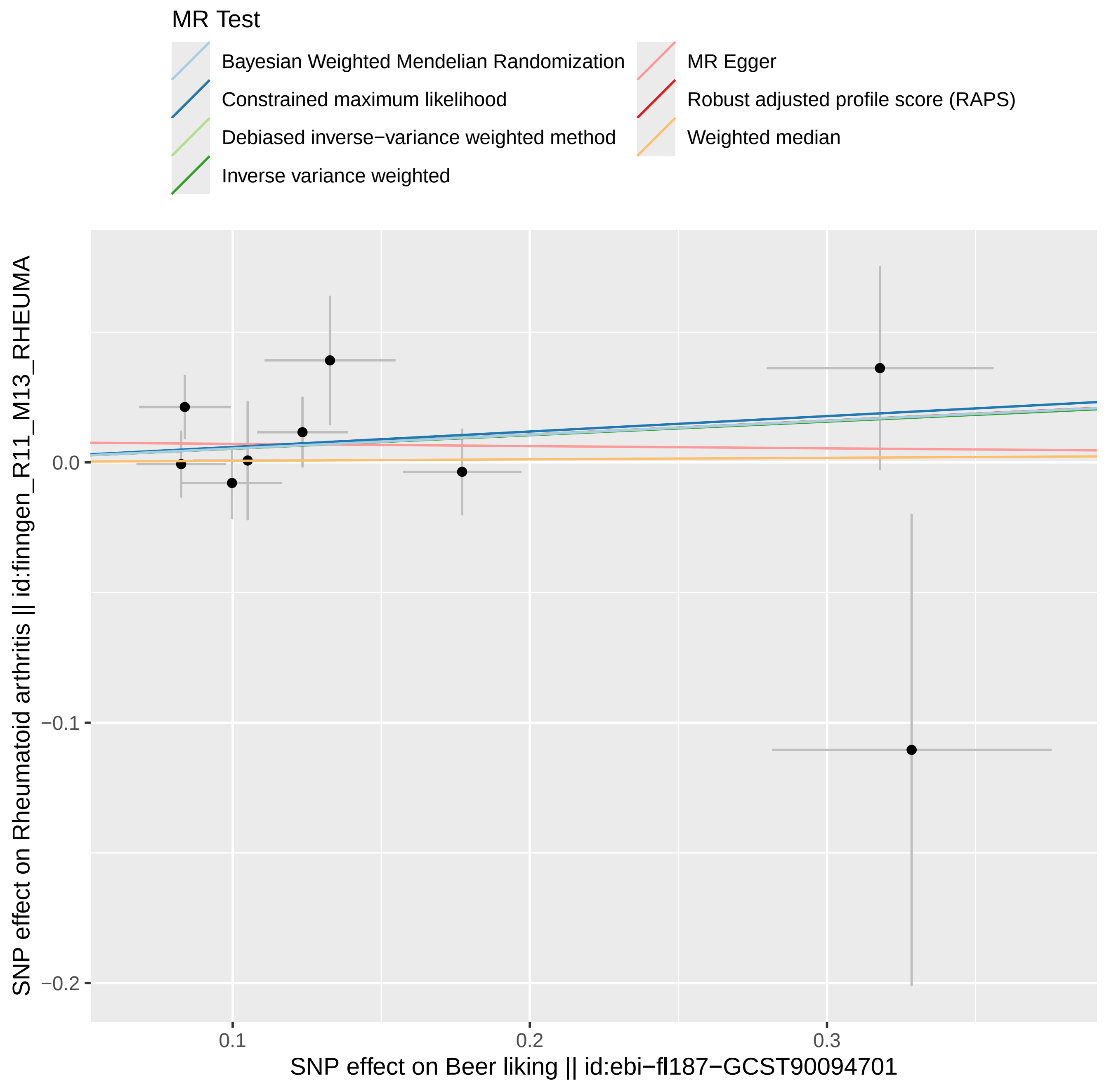

MR Test
Bayesian Weighted Mendelian Randomization
Constrained maximum likelihood
Debiased inverse−variance weighted method
Inverse variance weighted
MR Egger
Robust adjusted profile score (RAPS)
Weighted median
0.0
−0.1
−0.2
0.1
0.2
0.3
SNP effect on Rheumatoid arthritis || id:finngen_R11_M13_RHEUMA
SNP effect on Beer liking || id:ebi−fI187−GCST90094701

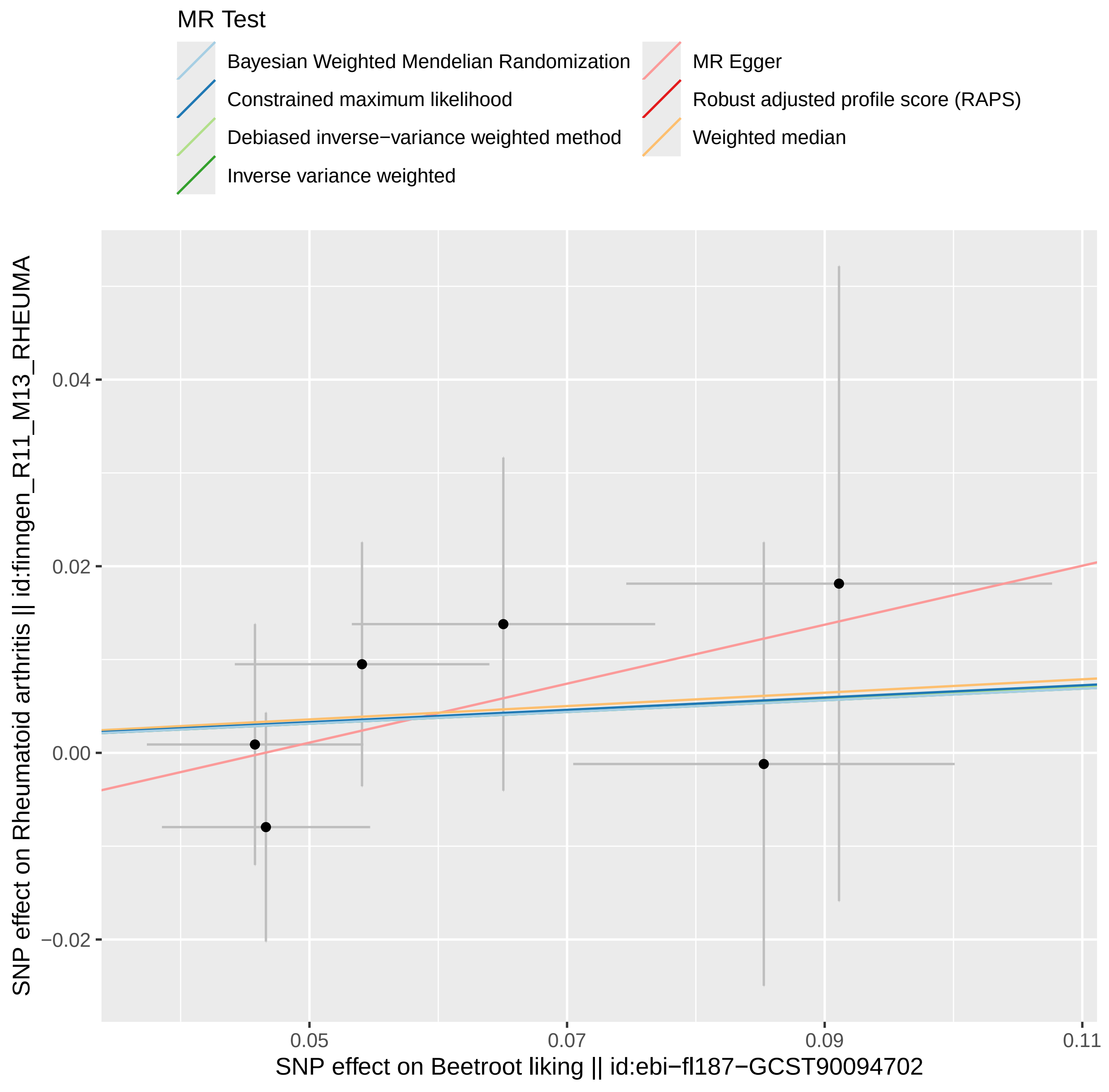
MR Test
Bayesian Weighted Mendelian Randomization
Constrained maximum likelihood
Debiased inverse−variance weighted method
Inverse variance weighted
MR Egger
Robust adjusted profile score (RAPS)
Weighted median
0.04
0.02
0.00
−0.02
0.05
0.07
0.09
0.11
SNP effect on Rheumatoid arthritis || id:finngen_R11_M13_RHEUMA
SNP effect on Beetroot liking || id:ebi−fl187−GCST90094702

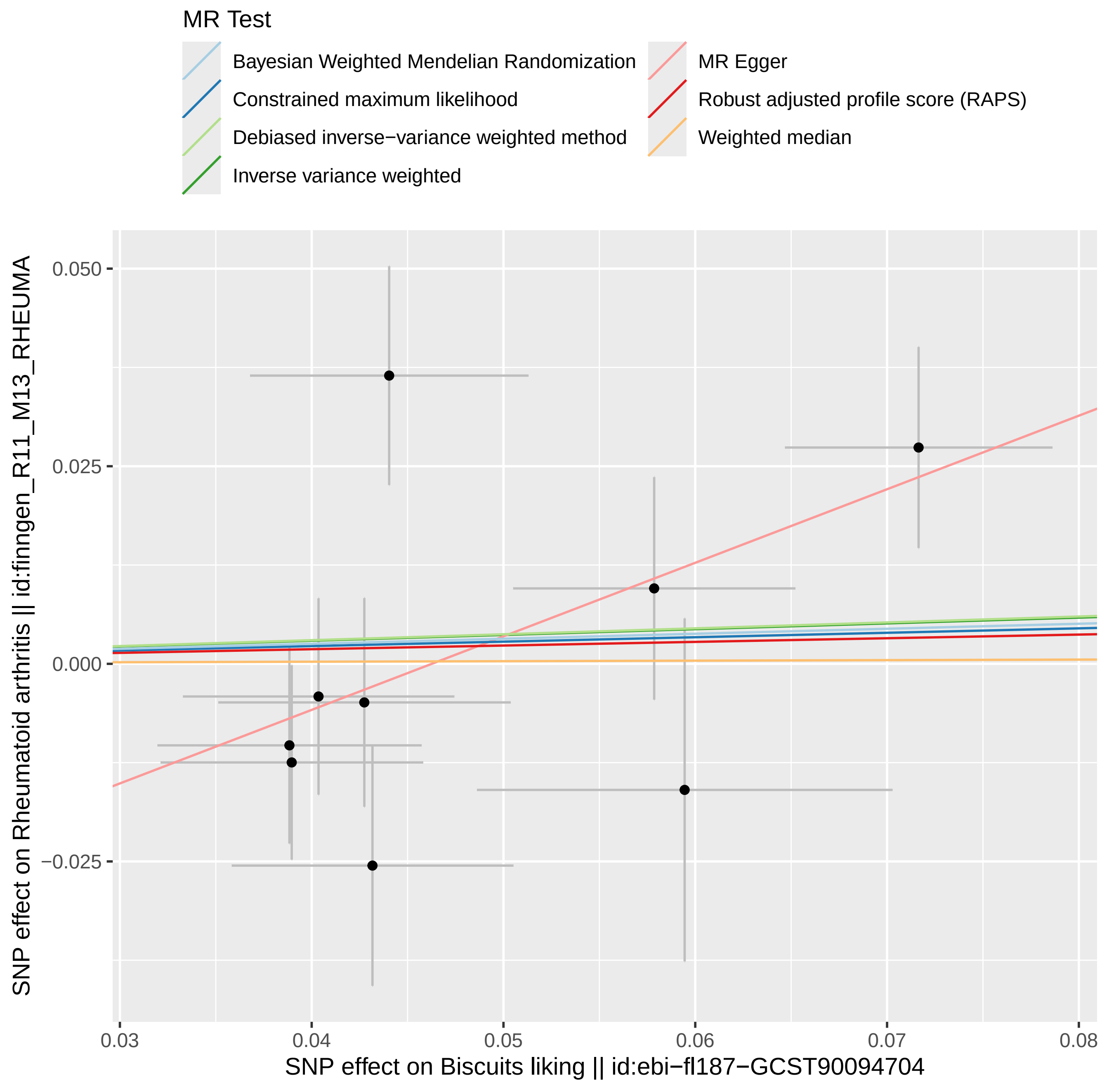
MR Test
Bayesian Weighted Mendelian Randomization
Constrained maximum likelihood
Debiased inverse−variance weighted method
Inverse variance weighted
MR Egger
Robust adjusted profile score (RAPS)
Weighted median
0.050
0.025
0.000
−0.025
0.03
0.04
0.05
0.06
0.07
0.08
SNP effect on Rheumatoid arthritis || id:finngen_R11_M13_RHEUMA
SNP effect on Biscuits liking || id:ebi−fl187−GCST90094704

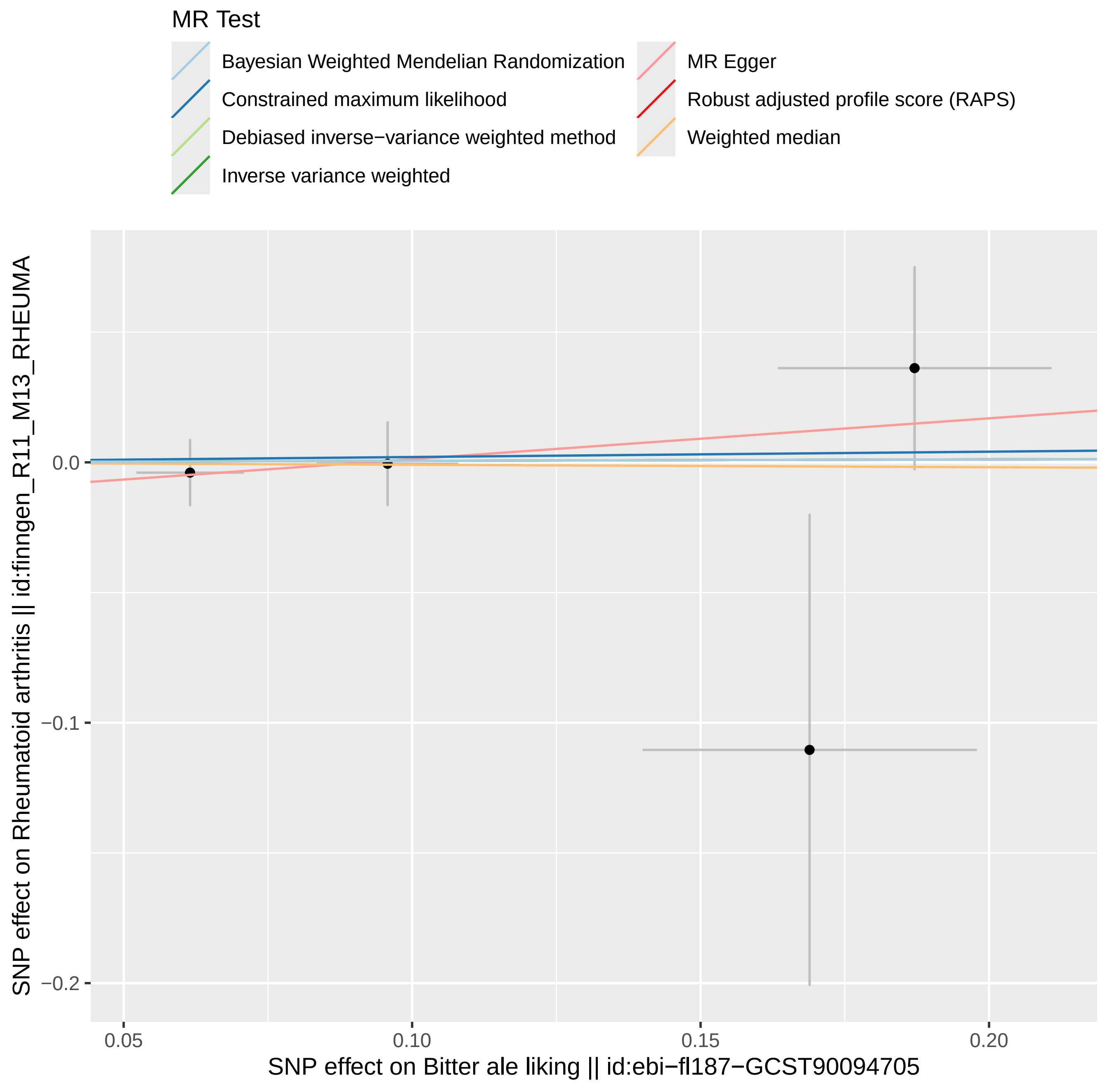
MR Test
Bayesian Weighted Mendelian Randomization
Constrained maximum likelihood
Debiased inverse−variance weighted method
Inverse variance weighted
MR Egger
Robust adjusted profile score (RAPS)
Weighted median
SNP effect on Rheumatoid arthritis || id:finngen_R11_M13_RHEUMA
0.0
−0.1
−0.2
0.05
0.10
0.15
0.20
SNP effect on Bitter ale liking || id:ebi−fl187−GCST90094705

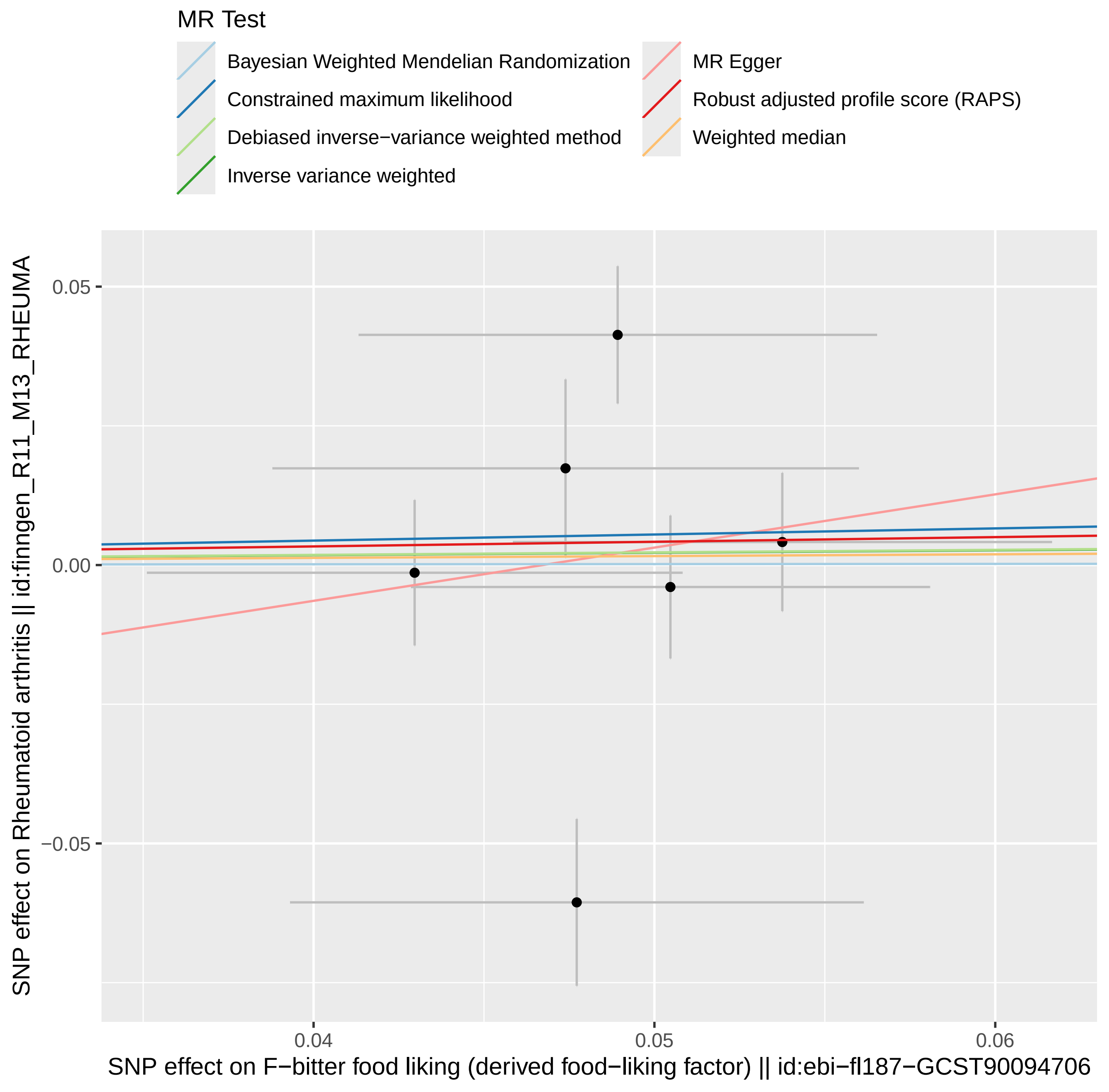
MR Test
Bayesian Weighted Mendelian Randomization
Constrained maximum likelihood
Debiased inverse−variance weighted method
Inverse variance weighted
MR Egger
Robust adjusted profile score (RAPS)
Weighted median
0.05
0.00
−0.05
0.04
0.05
0.06
SNP effect on Rheumatoid arthritis || id:finngen_R11_M13_RHEUMA
SNP effect on F−bitter food liking (derived food−liking factor) || id:ebi−fl187−GCST90094706

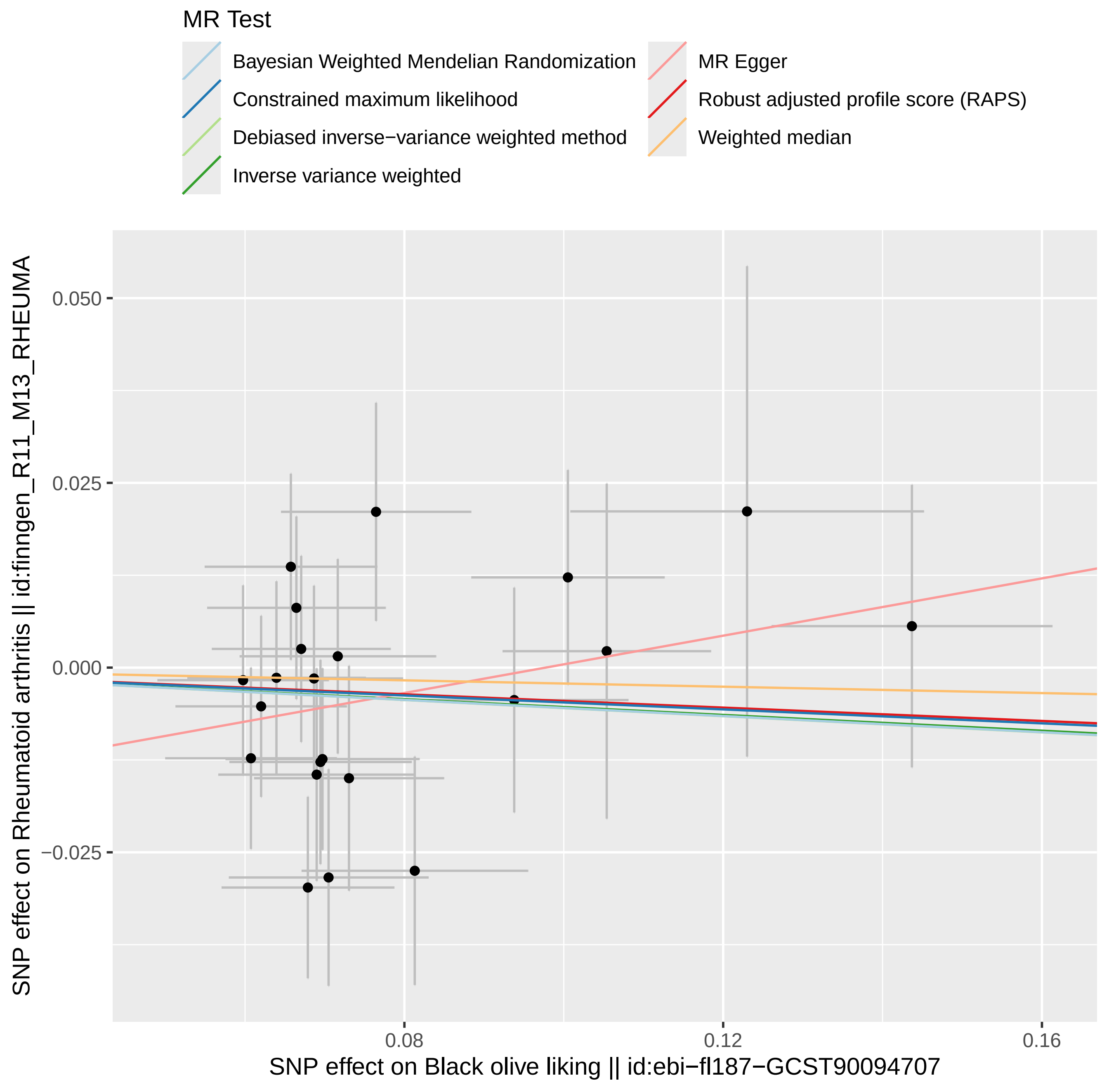

MR Test
Bayesian Weighted Mendelian Randomization
Constrained maximum likelihood
Debiased inverse−variance weighted method
Inverse variance weighted
MR Egger
Robust adjusted profile score (RAPS)
Weighted median
0.050
0.025
0.000
−0.025
0.08
0.12
0.16
SNP effect on Rheumatoid arthritis || id:finngen_R11_M13_RHEUMA
SNP effect on Black olive liking || id:ebi−fl187−GCST90094707

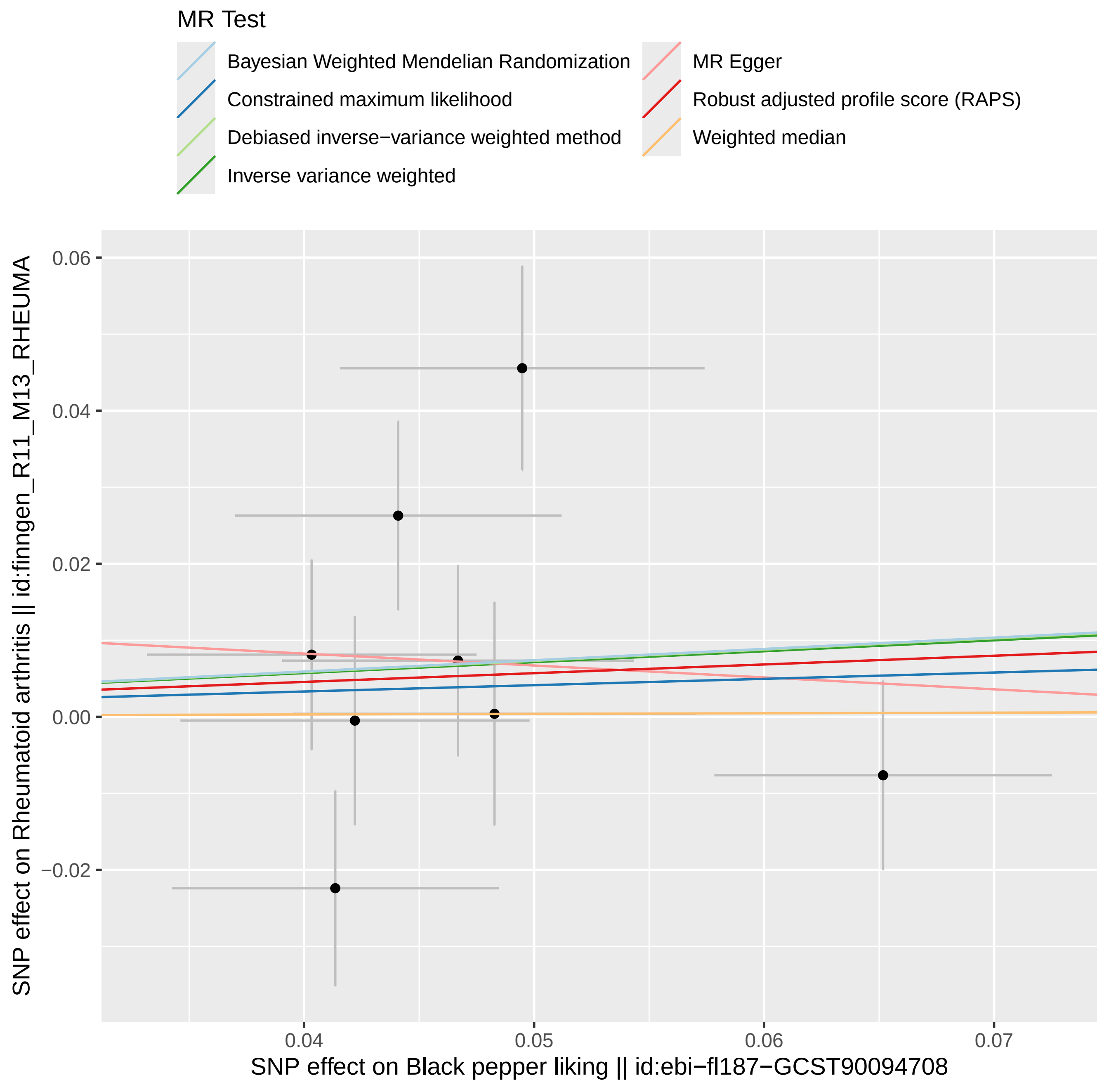
MR Test
Bayesian Weighted Mendelian Randomization
Constrained maximum likelihood
Debiased inverse−variance weighted method
Inverse variance weighted
MR Egger
Robust adjusted profile score (RAPS)
Weighted median
0.06
0.04
0.02
0.00
−0.02
0.04
0.05
0.06
0.07
SNP effect on Rheumatoid arthritis || id:finngen_R11_M13_RHEUMA
SNP effect on Black pepper liking || id:ebi−fl187−GCST90094708

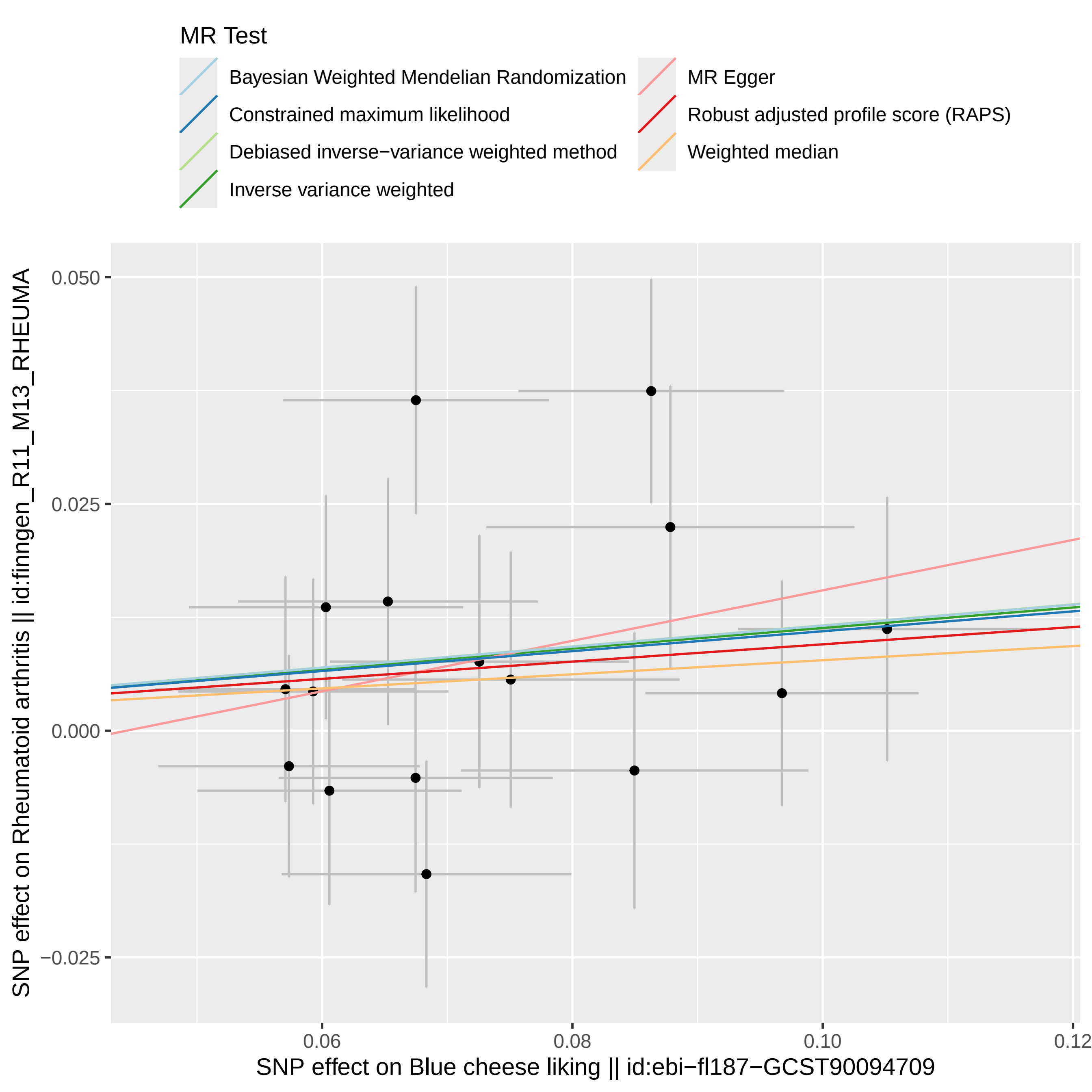
MR Test
Bayesian Weighted Mendelian Randomization
Constrained maximum likelihood
Debiased inverse−variance weighted method
Inverse variance weighted
MR Egger
Robust adjusted profile score (RAPS)
Weighted median
SNP effect on Rheumatoid arthritis || id:finngen_R11_M13_RHEUMA
0.050
0.025
0.000
−0.025
0.06
0.08
0.10
0.12
SNP effect on Blue cheese liking || id:ebi−fl187−GCST90094709

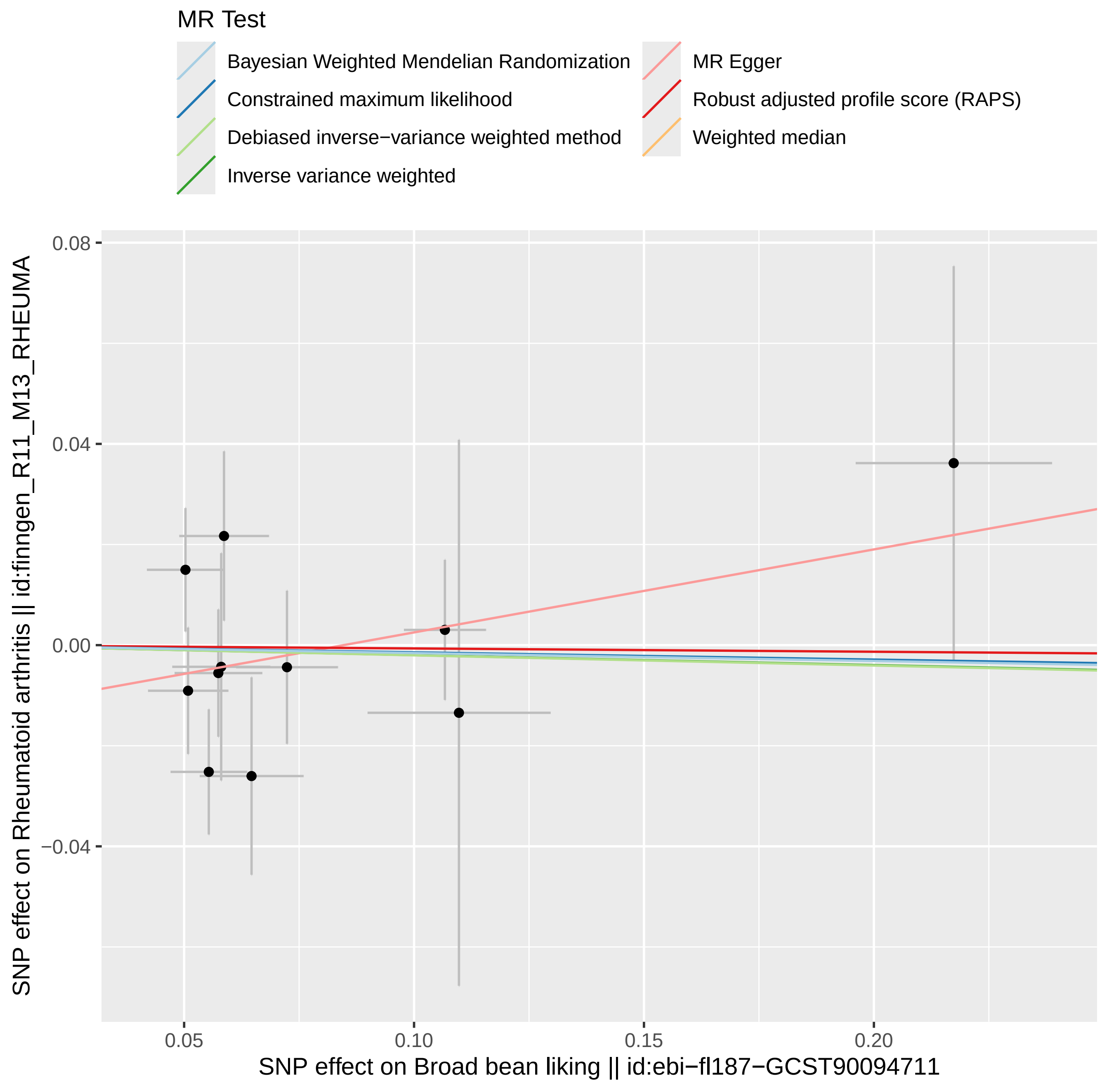
MR Test
Bayesian Weighted Mendelian Randomization
Constrained maximum likelihood
Debiased inverse−variance weighted method
Inverse variance weighted
MR Egger
Robust adjusted profile score (RAPS)
Weighted median
0.08
0.04
0.00
−0.04
0.05
0.10
0.15
0.20
SNP effect on Rheumatoid arthritis || id:finngen_R11_M13_RHEUMA
SNP effect on Broad bean liking || id:ebi−fl187−GCST90094711

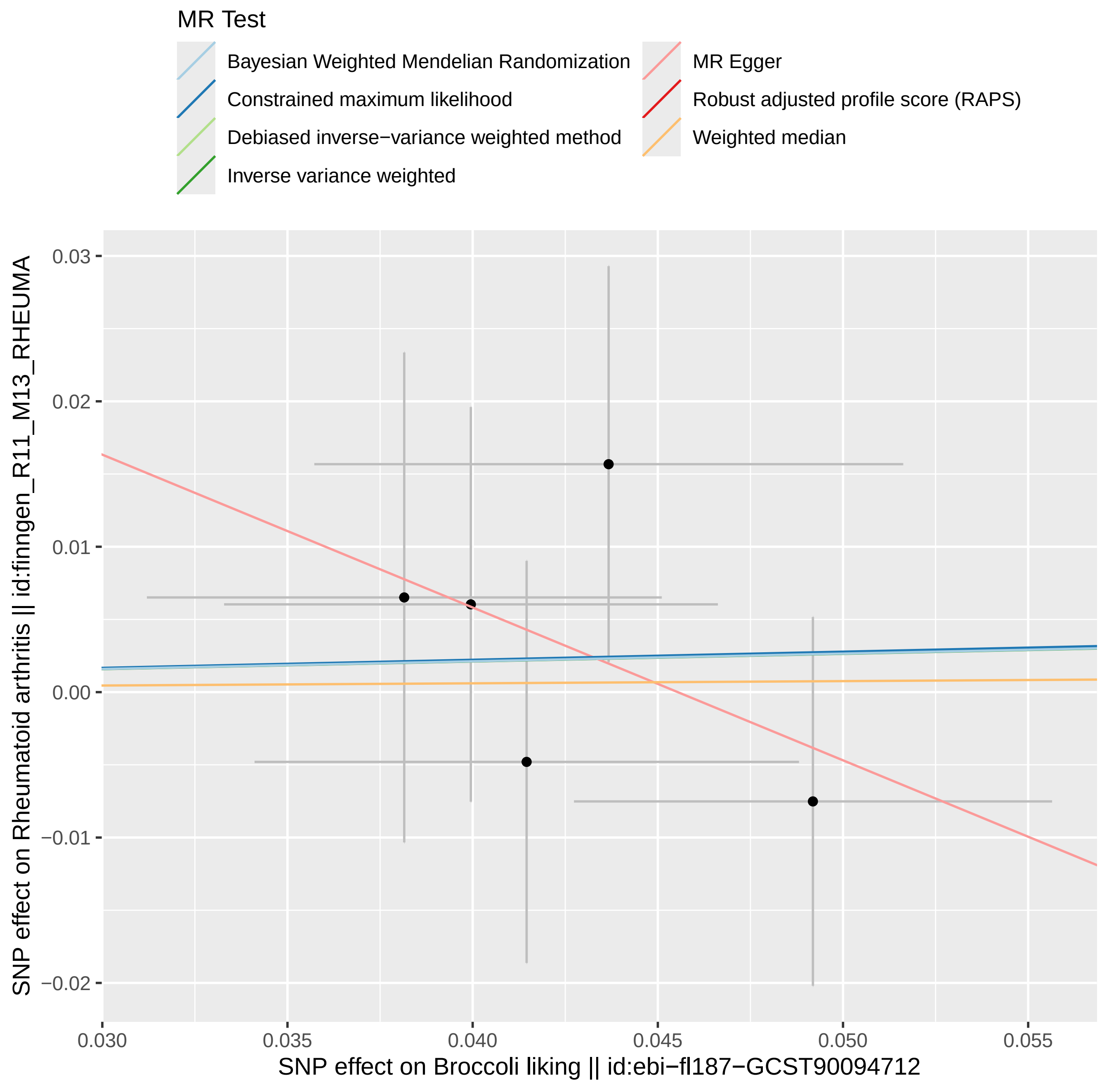
MR Test
Bayesian Weighted Mendelian Randomization
Constrained maximum likelihood
Debiased inverse−variance weighted method
Inverse variance weighted
MR Egger
Robust adjusted profile score (RAPS)
Weighted median
0.03
0.02
0.01
0.00
−0.01
−0.02
0.030
0.035
0.040
0.045
0.050
0.055
SNP effect on Rheumatoid arthritis || id:finngen_R11_M13_RHEUMA
SNP effect on Broccoli liking || id:ebi−fl187−GCST90094712

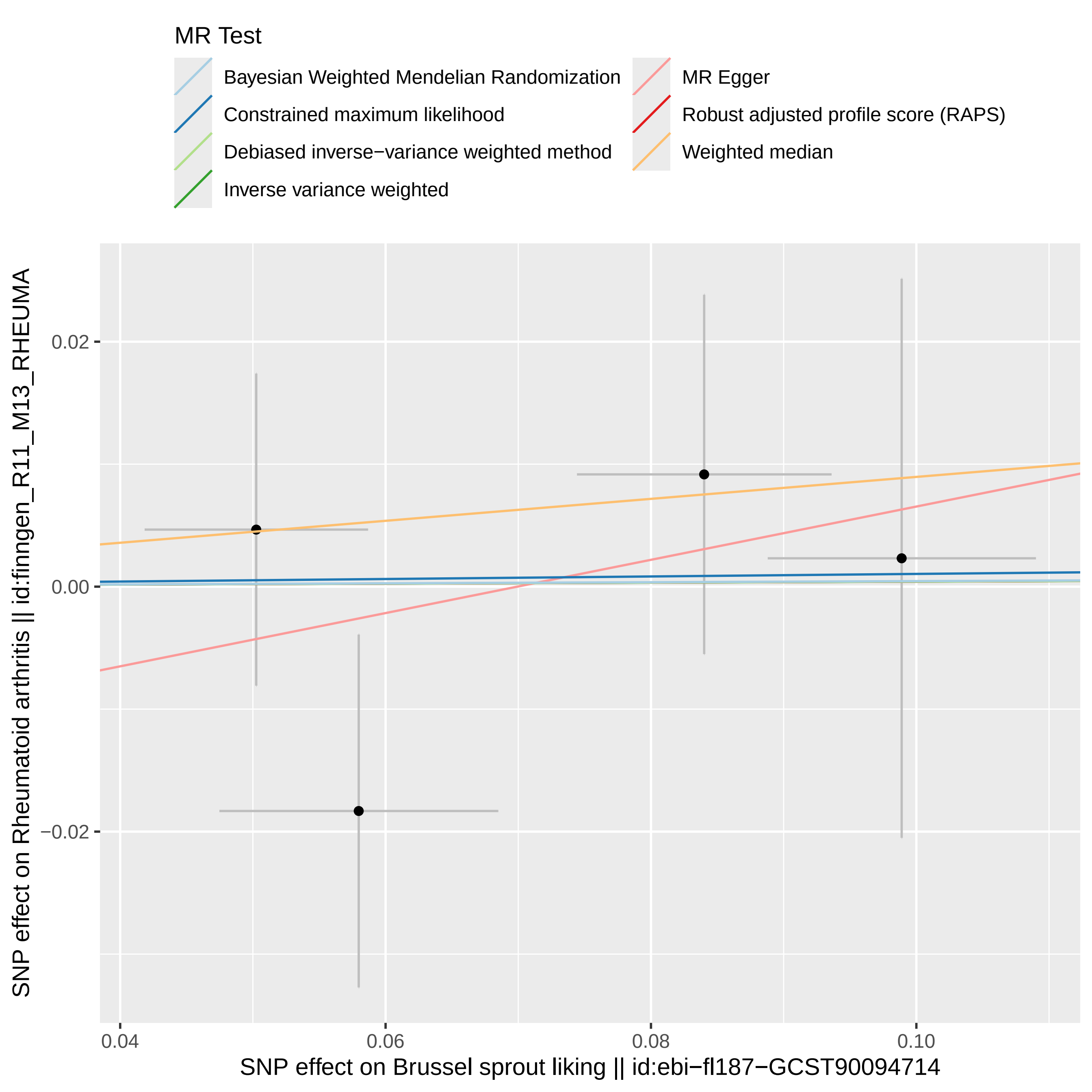
MR Test
Bayesian Weighted Mendelian Randomization
Constrained maximum likelihood
Debiased inverse−variance weighted method
Inverse variance weighted
MR Egger
Robust adjusted profile score (RAPS)
Weighted median
0.02
0.00
−0.02
0.04
0.06
0.08
0.10
SNP effect on Rheumatoid arthritis || id:finngen_R11_M13_RHEUMA
SNP effect on Brussel sprout liking || id:ebi−fl187−GCST90094714

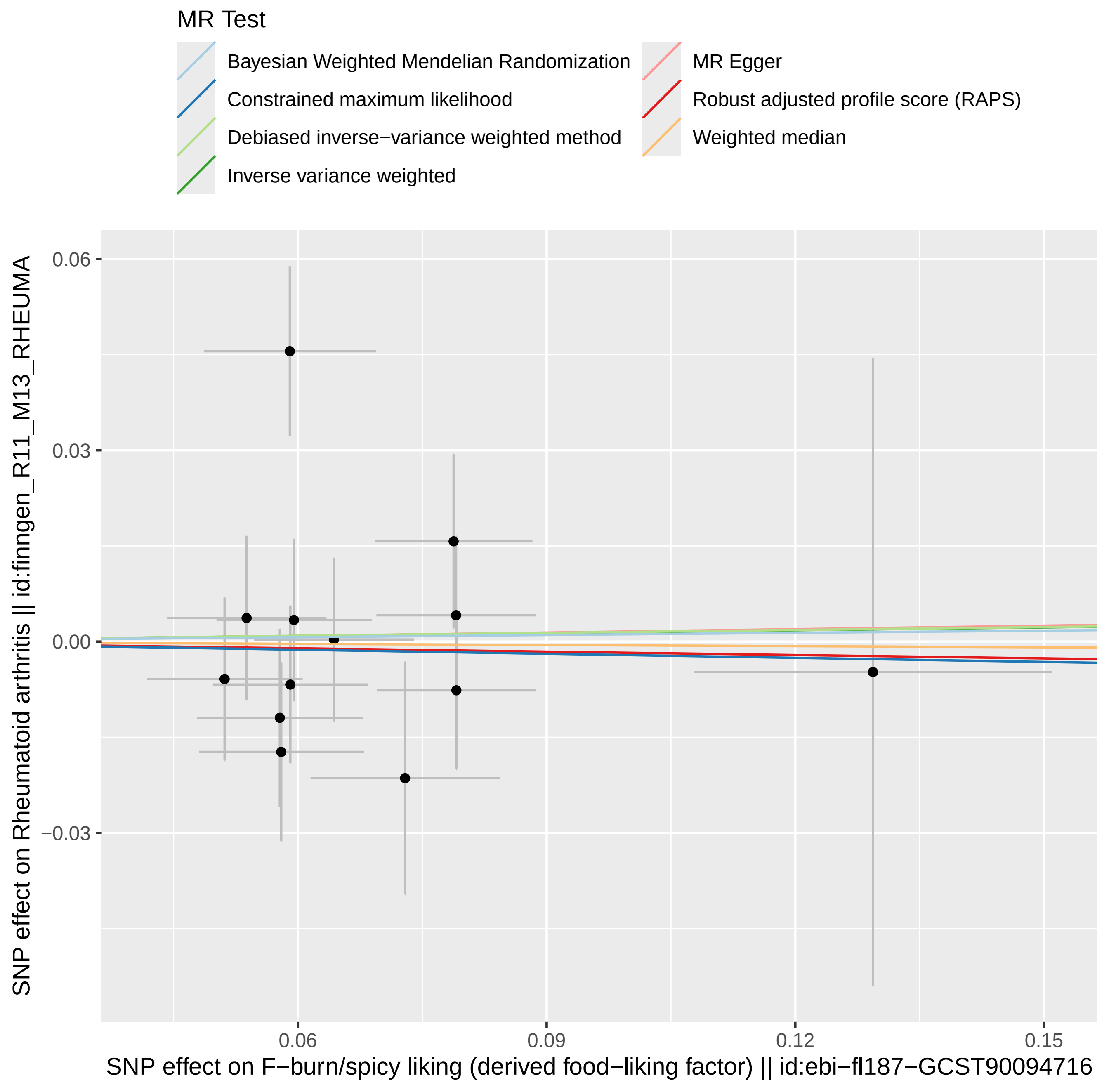
MR Test
Bayesian Weighted Mendelian Randomization
Constrained maximum likelihood
Debiased inverse−variance weighted method
Inverse variance weighted
MR Egger
Robust adjusted profile score (RAPS)
Weighted median
0.06
0.03
0.00
−0.03
0.06
0.09
0.12
0.15
SNP effect on Rheumatoid arthritis || id:finngen_R11_M13_RHEUMA
SNP effect on F−burn/spicy liking (derived food−liking factor) || id:ebi−fl187−GCST90094716

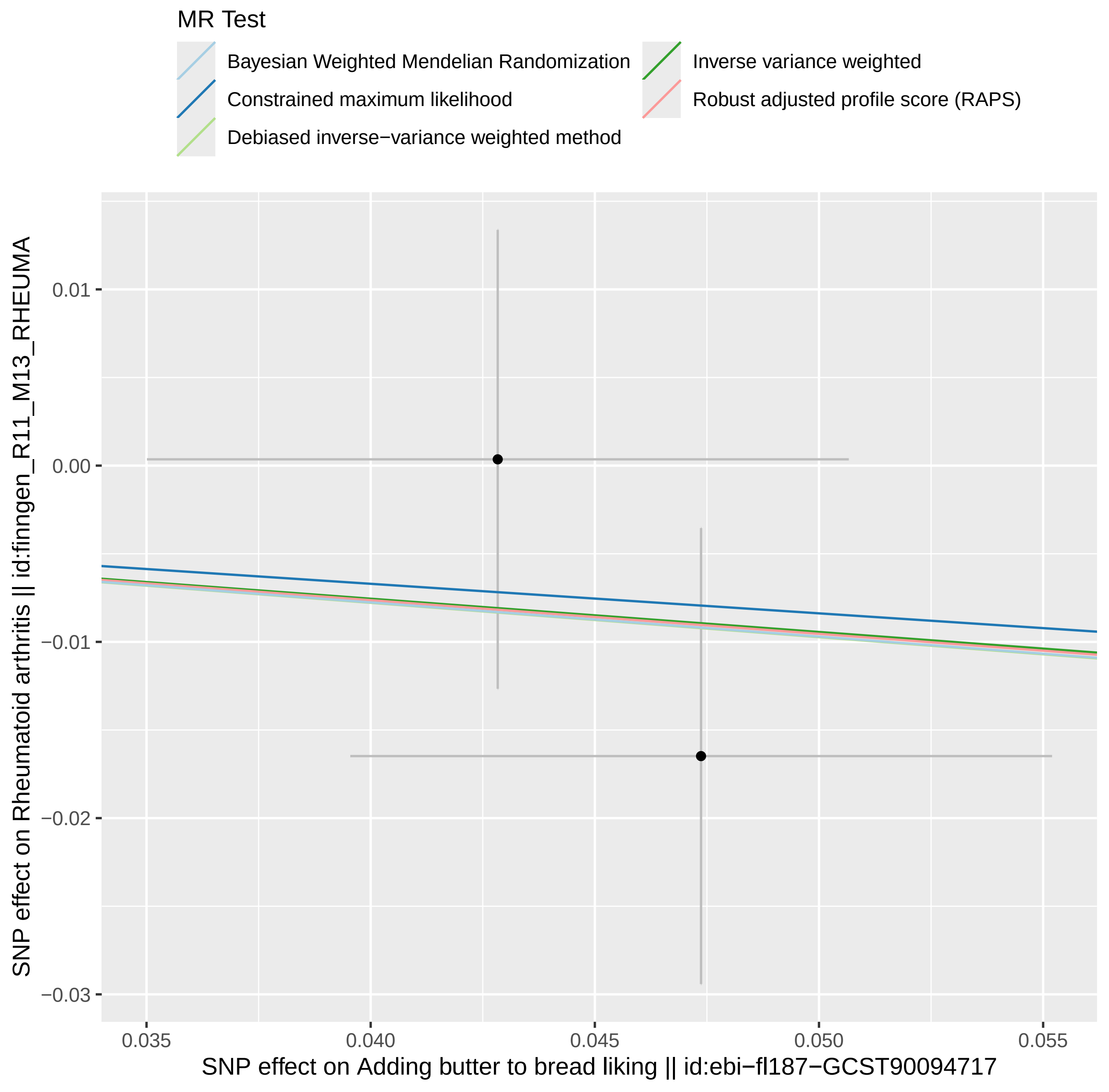
MR Test
Bayesian Weighted Mendelian Randomization
Constrained maximum likelihood
Debiased inverse−variance weighted method
Inverse variance weighted
Robust adjusted profile score (RAPS)
0.01
0.00
−0.01
−0.02
−0.03
0.035
0.040
0.045
0.050
0.055
SNP effect on Rheumatoid arthritis || id:finngen_R11_M13_RHEUMA
SNP effect on Adding butter to bread liking || id:ebi−fl187−GCST90094717

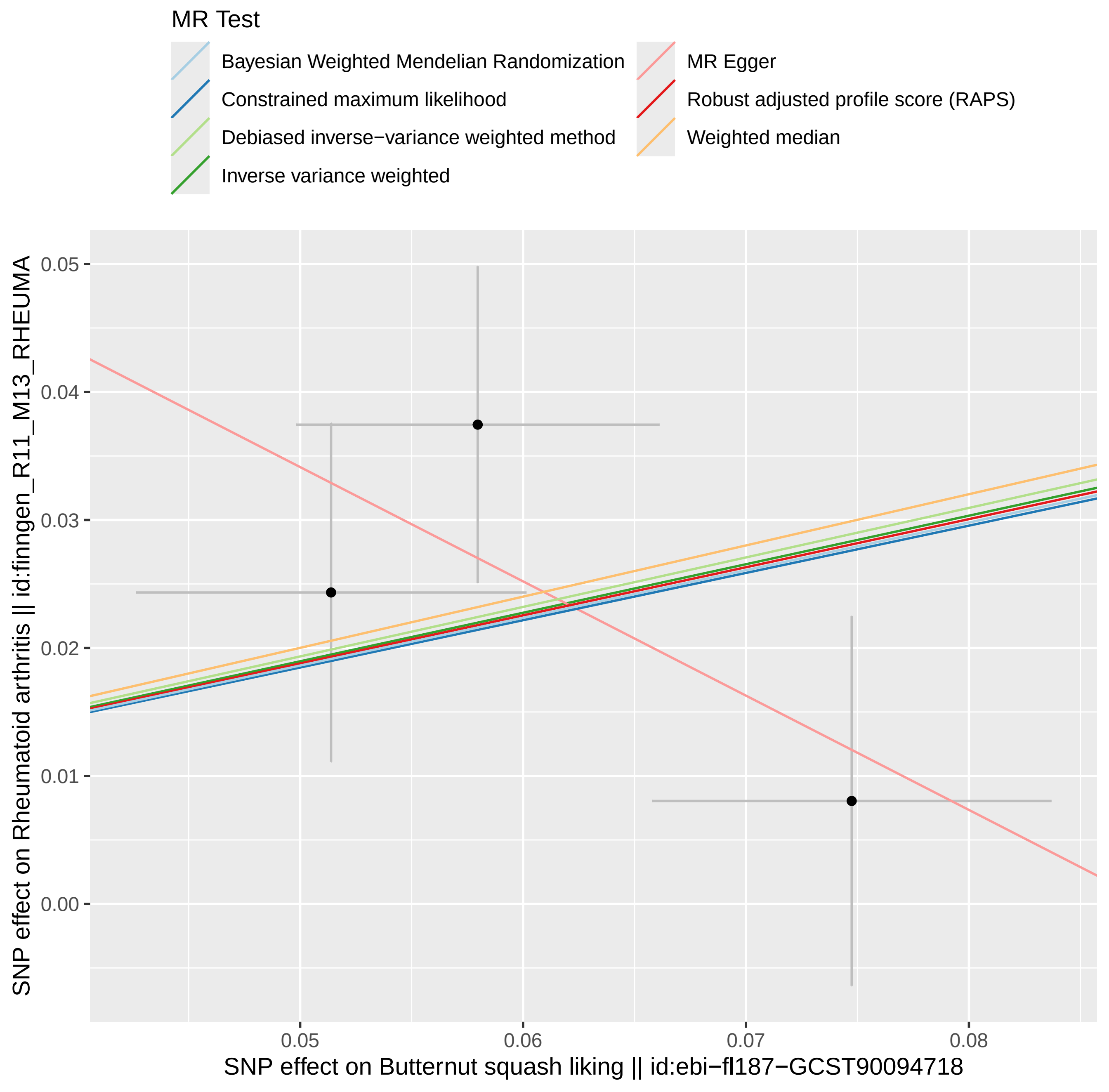

MR Test
Bayesian Weighted Mendelian Randomization
Constrained maximum likelihood
Debiased inverse−variance weighted method
Inverse variance weighted
MR Egger
Robust adjusted profile score (RAPS)
Weighted median
0.05
0.04
0.03
0.02
0.01
0.00
0.05
0.06
0.07
0.08
SNP effect on Rheumatoid arthritis || id:finngen_R11_M13_RHEUMA
SNP effect on Butternut squash liking || id:ebi−fl187−GCST90094718

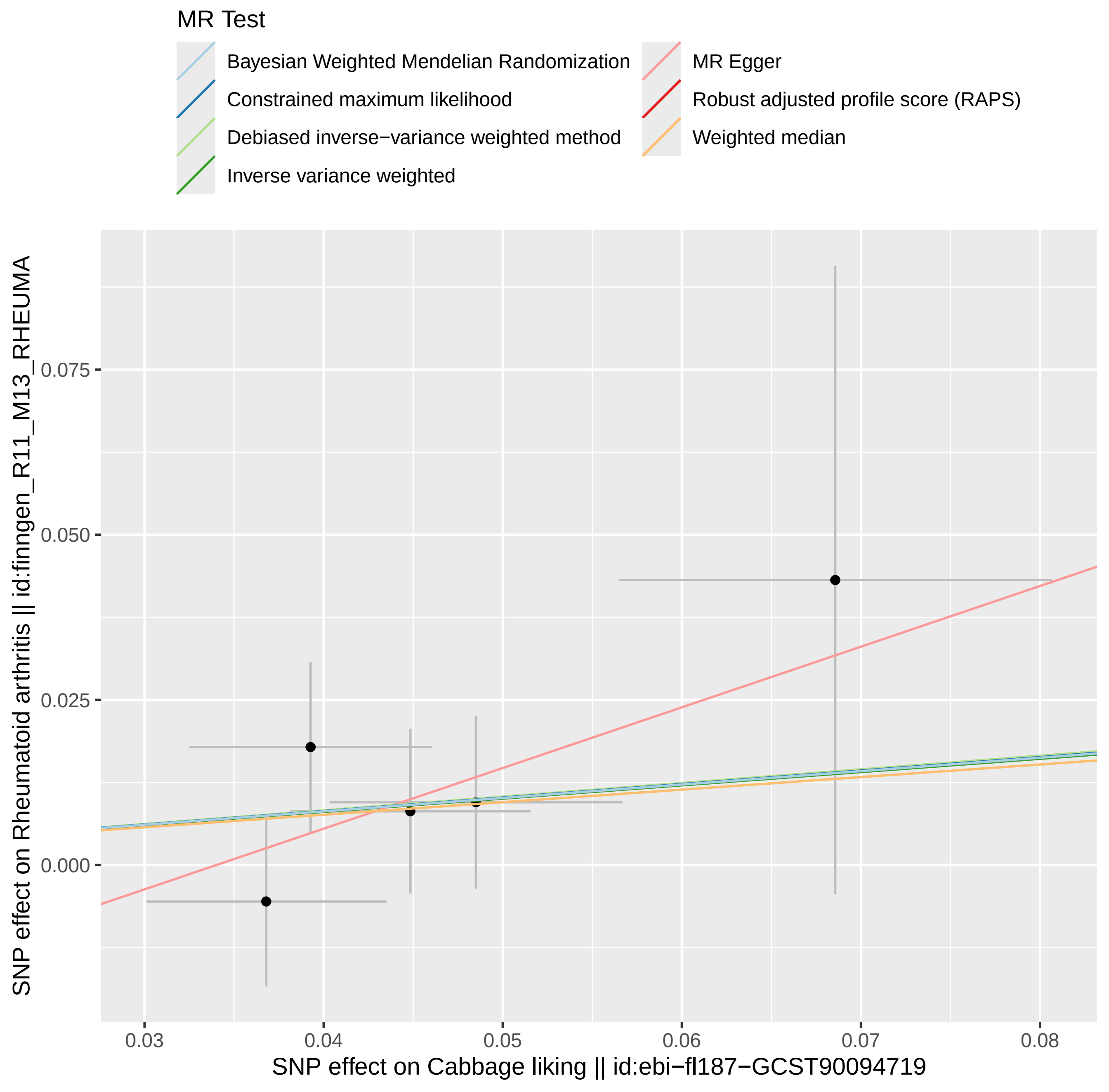

MR Test
Bayesian Weighted Mendelian Randomization
Constrained maximum likelihood
Debiased inverse−variance weighted method
Inverse variance weighted
MR Egger
Robust adjusted profile score (RAPS)
Weighted median
0.075
0.050
0.025
0.000
0.03
0.04
0.05
0.06
0.07
0.08
SNP effect on Rheumatoid arthritis || id:finngen_R11_M13_RHEUMA
SNP effect on Cabbage liking || id:ebi−fl187−GCST90094719

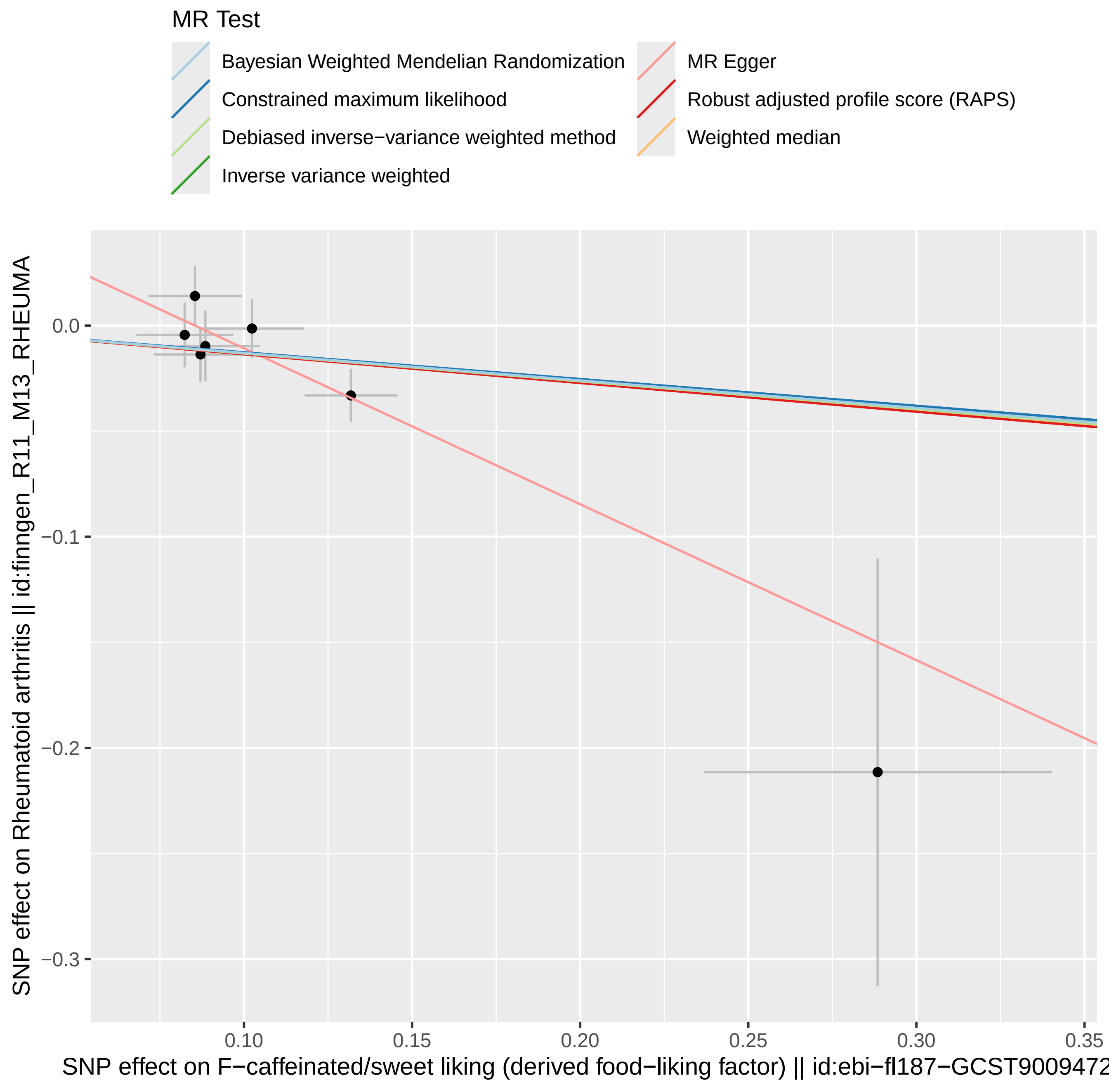

MR Test
Bayesian Weighted Mendelian Randomization
Constrained maximum likelihood
Debiased inverse−variance weighted method
Inverse variance weighted
MR Egger
Robust adjusted profile score (RAPS)
Weighted median
0.0
−0.1
−0.2
−0.3
0.10
0.15
0.20
0.25
0.30
0.35
SNP effect on Rheumatoid arthritis || id:finngen_R11_M13_RHEUMA
SNP effect on F−caffeinated/sweet liking (derived food−liking factor) || id:ebi−fl187−GCST9009472

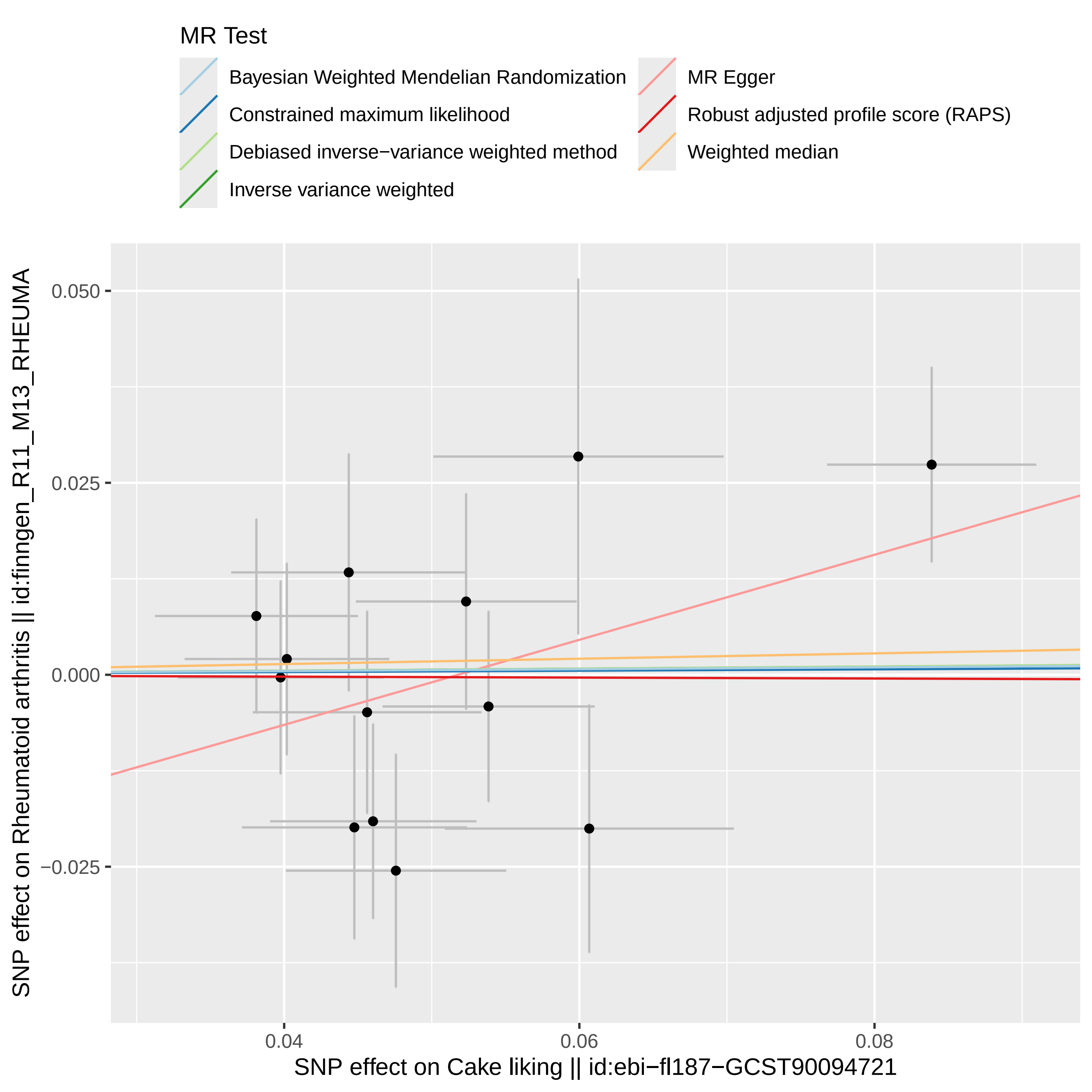
MR Test
Bayesian Weighted Mendelian Randomization
Constrained maximum likelihood
Debiased inverse−variance weighted method
Inverse variance weighted
MR Egger
Robust adjusted profile score (RAPS)
Weighted median
0.050
0.025
0.000
−0.025
0.04
0.06
0.08
SNP effect on Rheumatoid arthritis || id:finngen_R11_M13_RHEUMA
SNP effect on Cake liking || id:ebi−fl187−GCST90094721

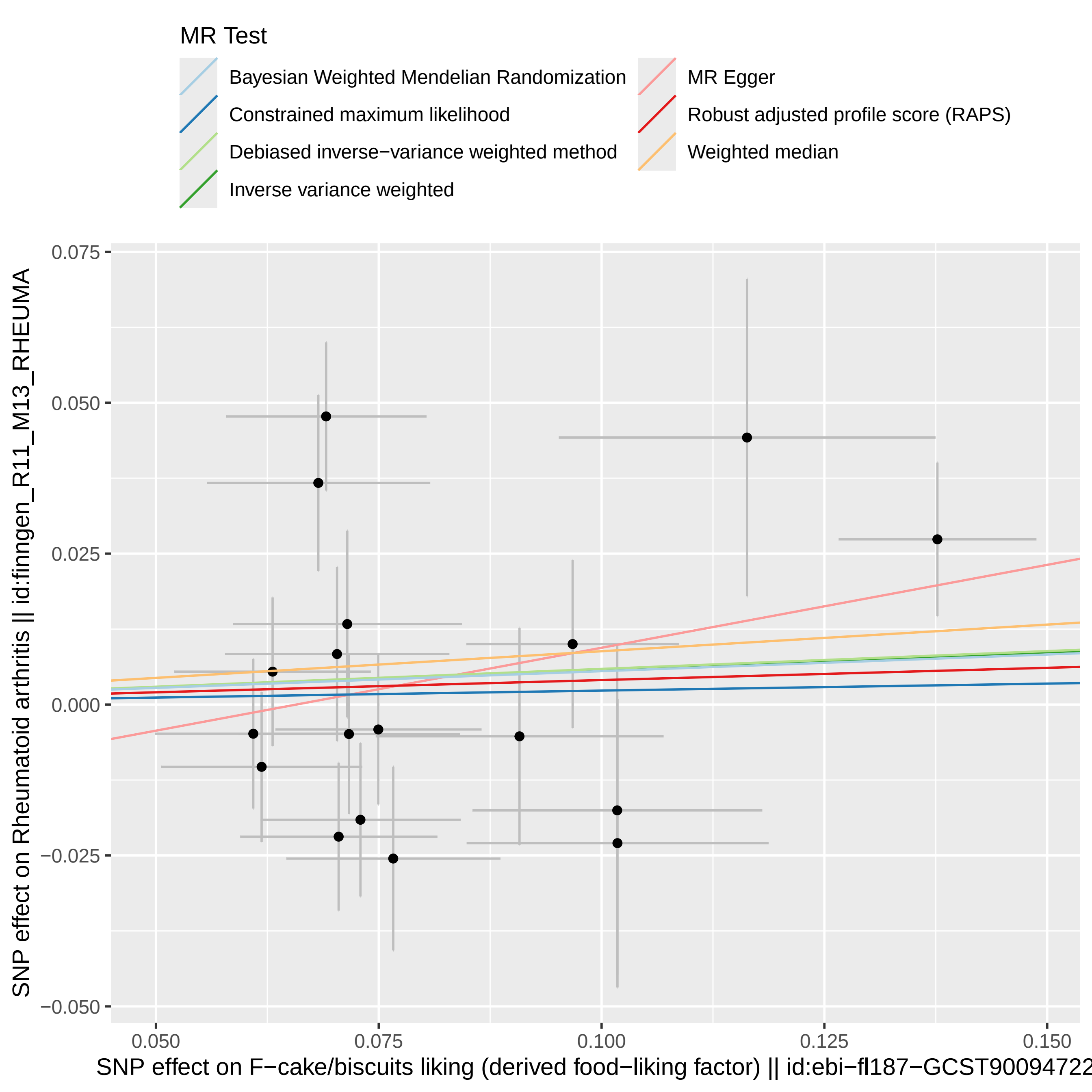

MR Test
Bayesian Weighted Mendelian Randomization
Constrained maximum likelihood
Debiased inverse−variance weighted method
Inverse variance weighted
MR Egger
Robust adjusted profile score (RAPS)
Weighted median
0.075
0.050
0.025
0.000
−0.025
−0.050
0.050
0.075
0.100
0.125
0.150
SNP effect on Rheumatoid arthritis || id:finngen_R11_M13_RHEUMA
SNP effect on F−cake/biscuits liking (derived food−liking factor) || id:ebi−fl187−GCST90094722

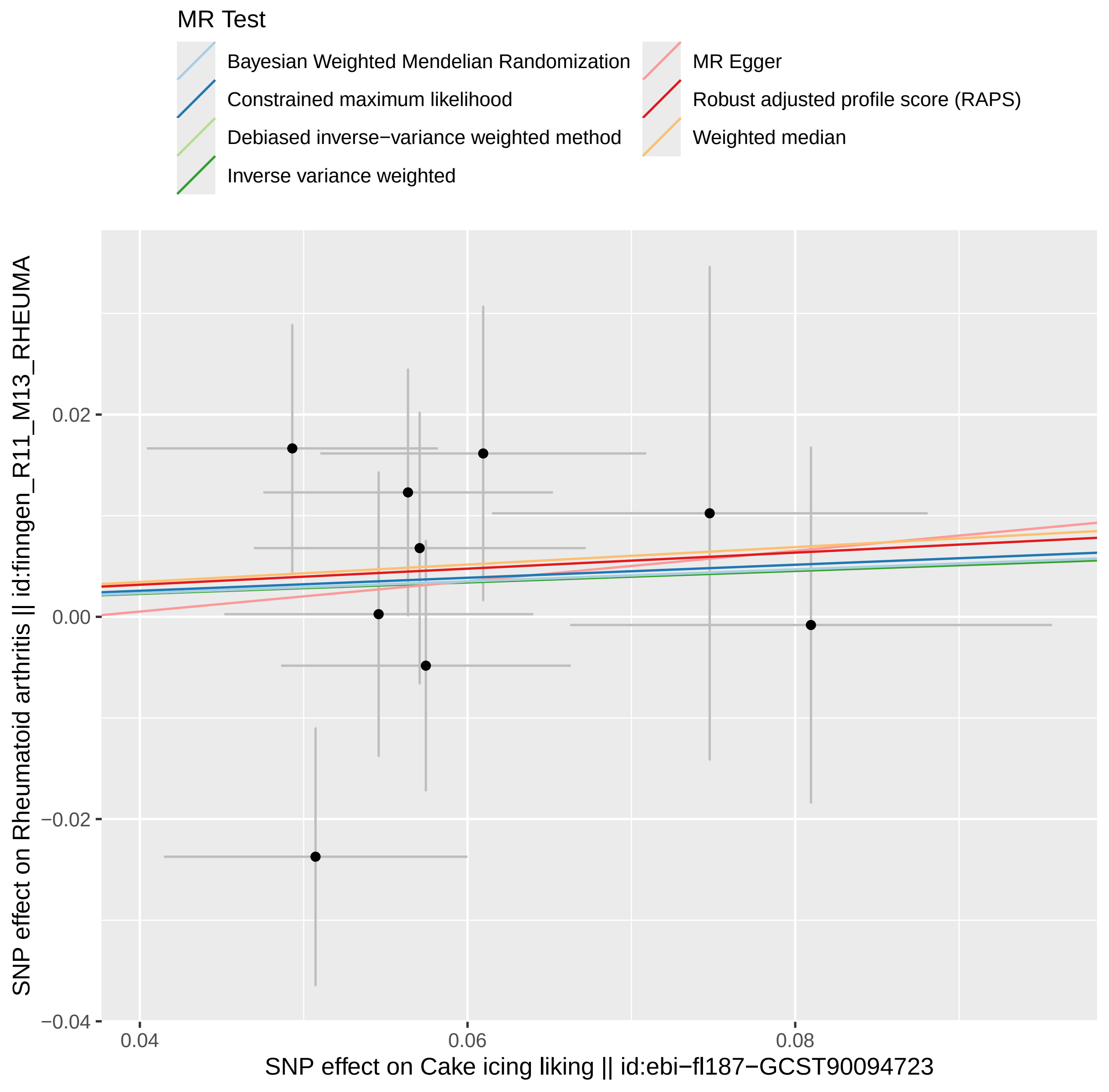
MR Test
Bayesian Weighted Mendelian Randomization
Constrained maximum likelihood
Debiased inverse−variance weighted method
Inverse variance weighted
MR Egger
Robust adjusted profile score (RAPS)
Weighted median
0.02
0.00
−0.02
−0.04
0.04
0.06
0.08
SNP effect on Rheumatoid arthritis || id:finngen_R11_M13_RHEUMA
SNP effect on Cake icing liking || id:ebi−fl187−GCST90094723

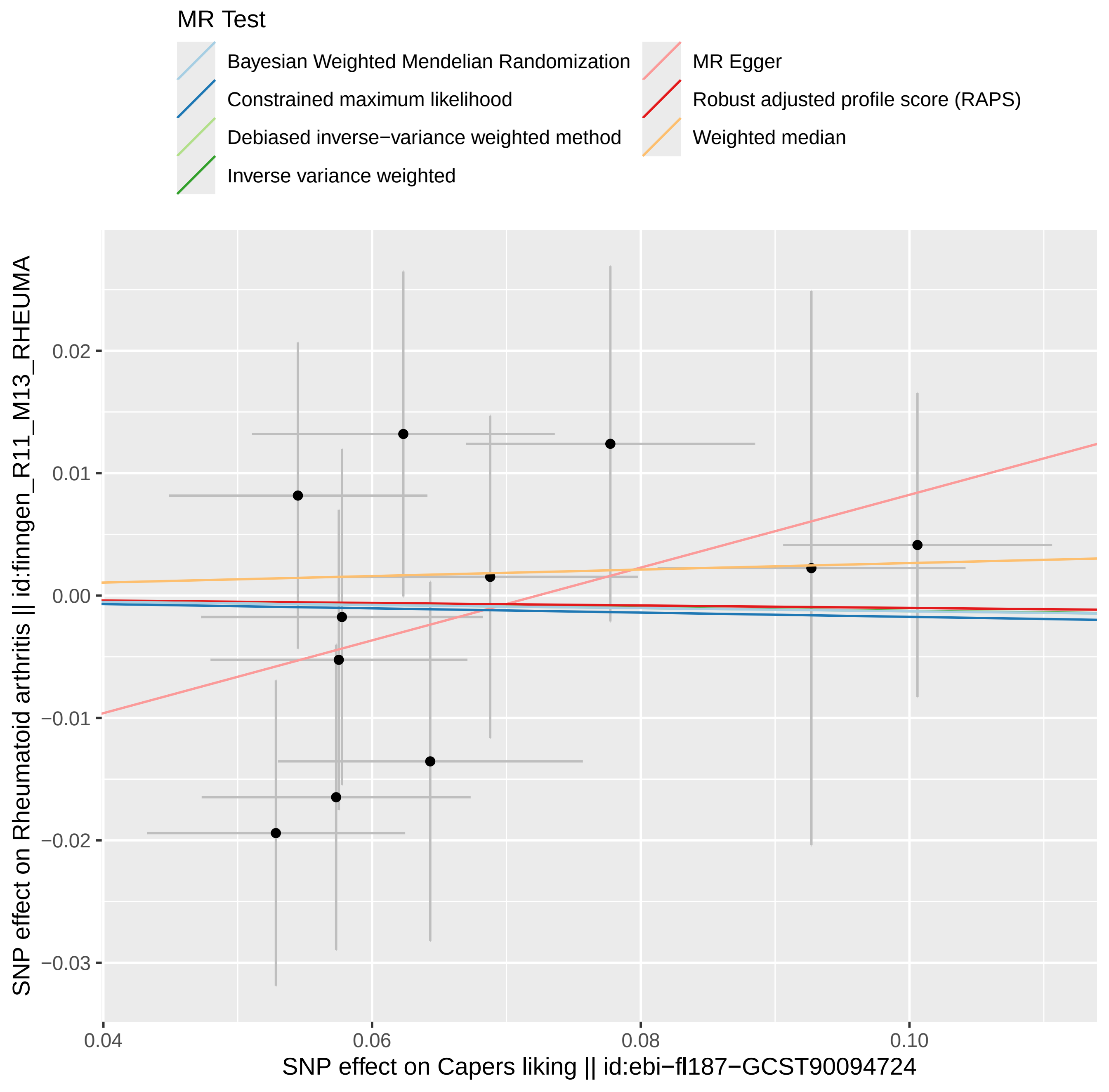
MR Test
Bayesian Weighted Mendelian Randomization
Constrained maximum likelihood
Debiased inverse−variance weighted method
Inverse variance weighted
MR Egger
Robust adjusted profile score (RAPS)
Weighted median
SNP effect on Rheumatoid arthritis || id:finngen_R11_M13_RHEUMA
0.02
0.01
0.00
−0.01
−0.02
−0.03
0.04
0.06
0.08
0.10
SNP effect on Capers liking || id:ebi−fl187−GCST90094724

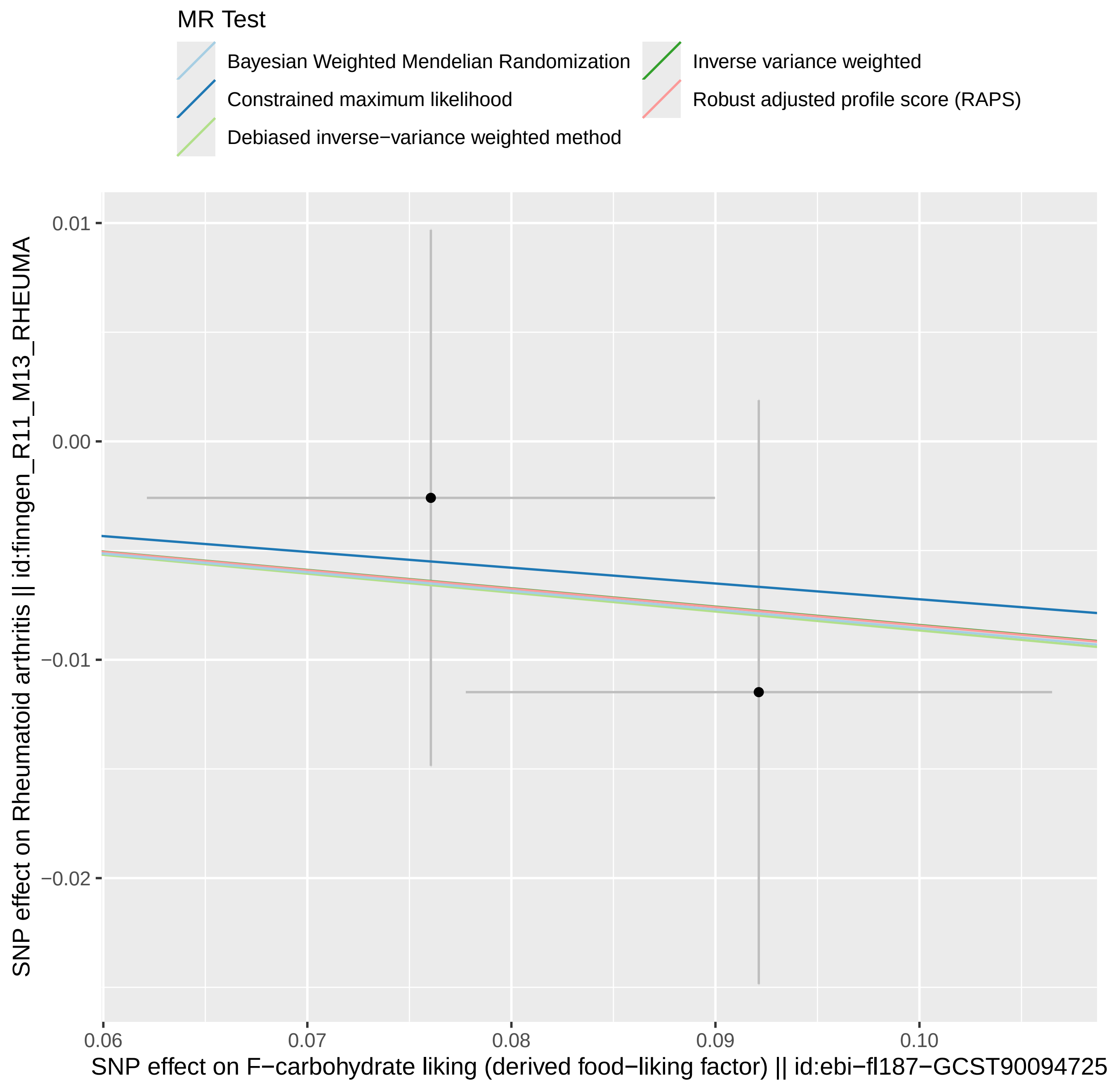
MR Test
Bayesian Weighted Mendelian Randomization
Constrained maximum likelihood
Debiased inverse−variance weighted method
Inverse variance weighted
Robust adjusted profile score (RAPS)
0.01
0.00
−0.01
−0.02
0.06
0.07
0.08
0.09
0.10
SNP effect on Rheumatoid arthritis || id:finngen_R11_M13_RHEUMA
SNP effect on F−carbohydrate liking (derived food−liking factor) || id:ebi−fl187−GCST90094725

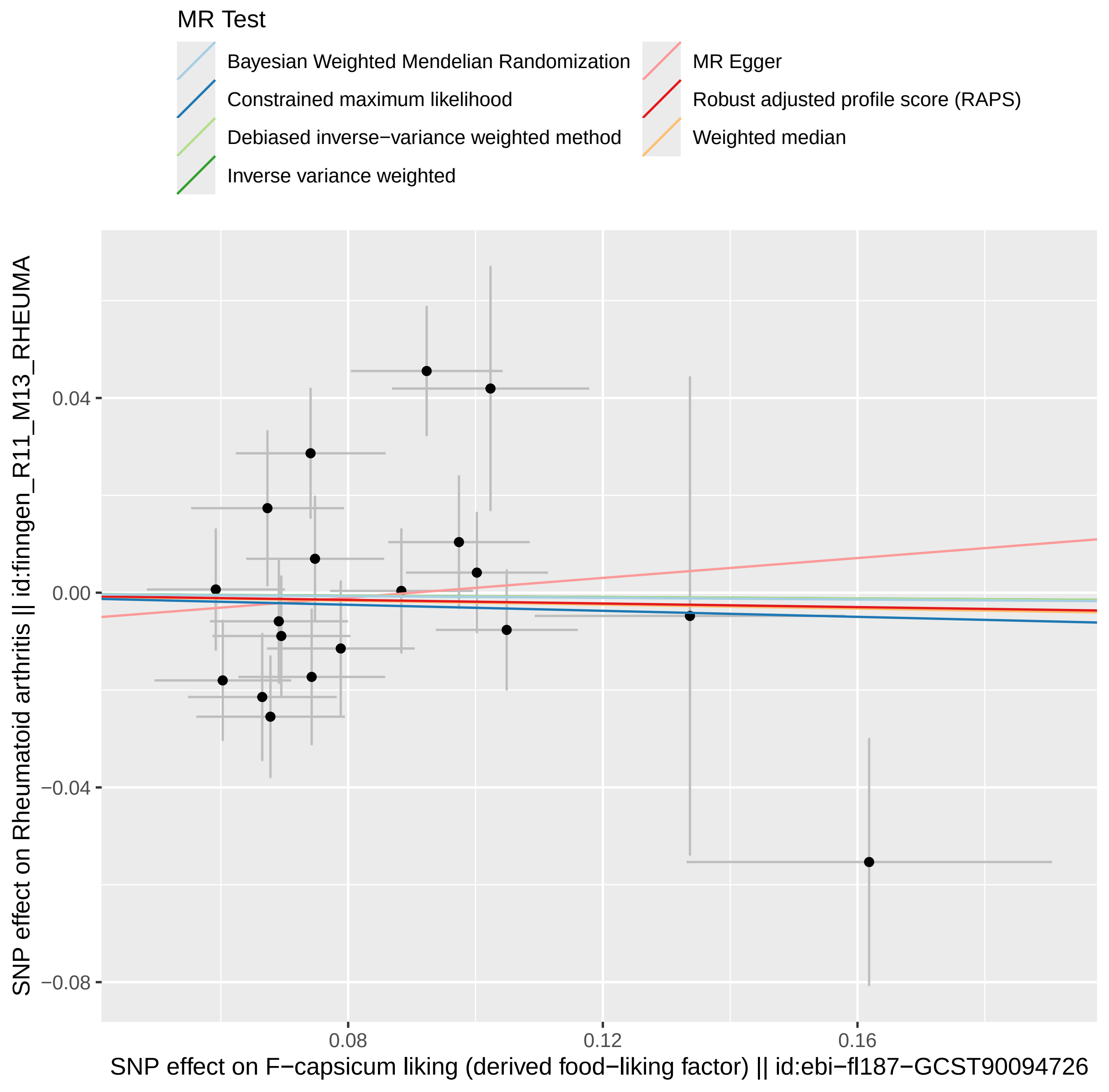

MR Test
Bayesian Weighted Mendelian Randomization
Constrained maximum likelihood
Debiased inverse−variance weighted method
Inverse variance weighted
MR Egger
Robust adjusted profile score (RAPS)
Weighted median
0.04
0.00
−0.04
−0.08
0.08
0.12
0.16
SNP effect on Rheumatoid arthritis || id:finngen_R11_M13_RHEUMA
SNP effect on F−capsicum liking (derived food−liking factor) || id:ebi−fl187−GCST90094726

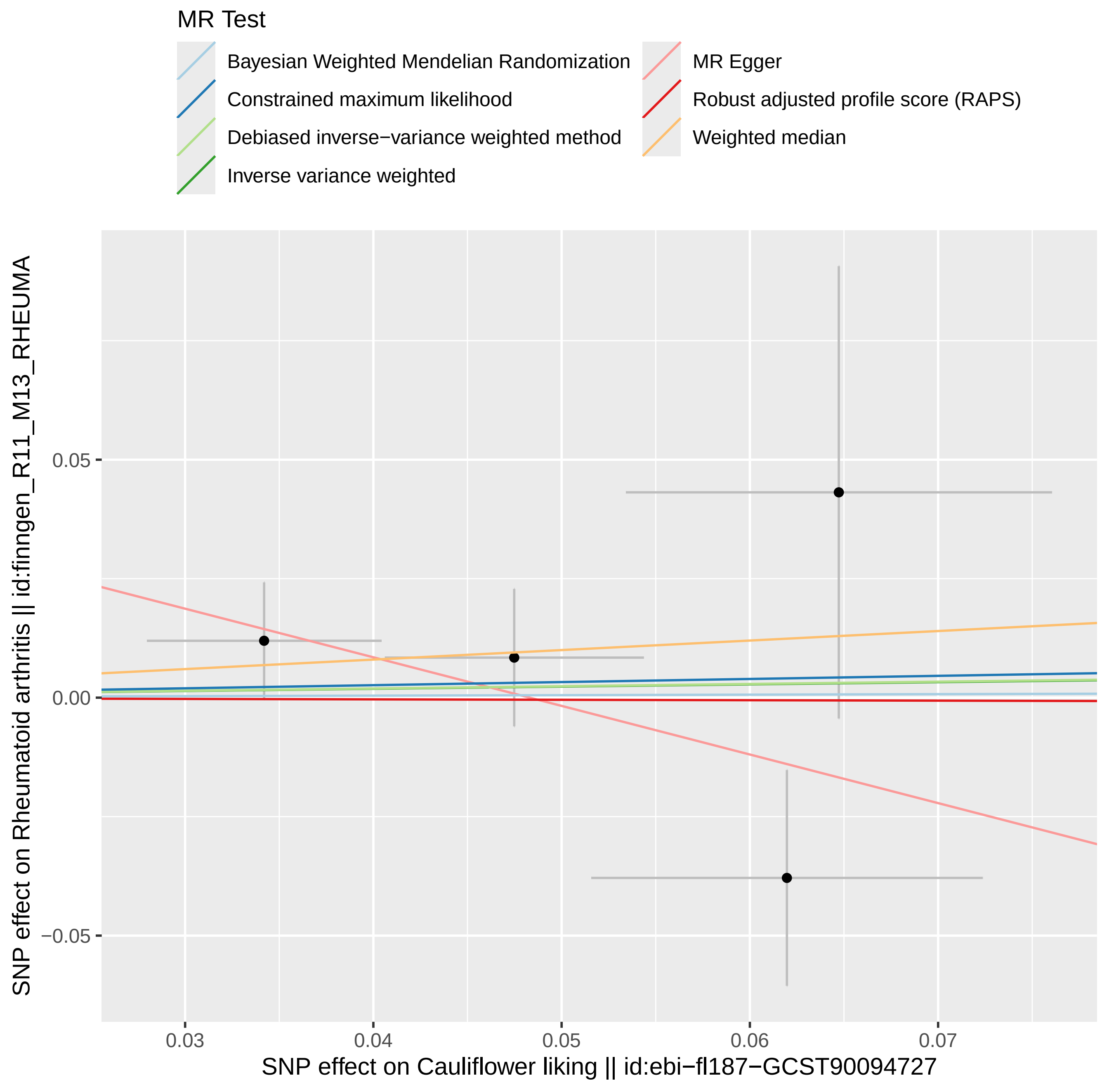
MR Test
Bayesian Weighted Mendelian Randomization
Constrained maximum likelihood
Debiased inverse−variance weighted method
Inverse variance weighted
MR Egger
Robust adjusted profile score (RAPS)
Weighted median
0.05
0.00
−0.05
0.03
0.04
0.05
0.06
0.07
SNP effect on Rheumatoid arthritis || id:finngen_R11_M13_RHEUMA
SNP effect on Cauliflower liking || id:ebi−fl187−GCST90094727

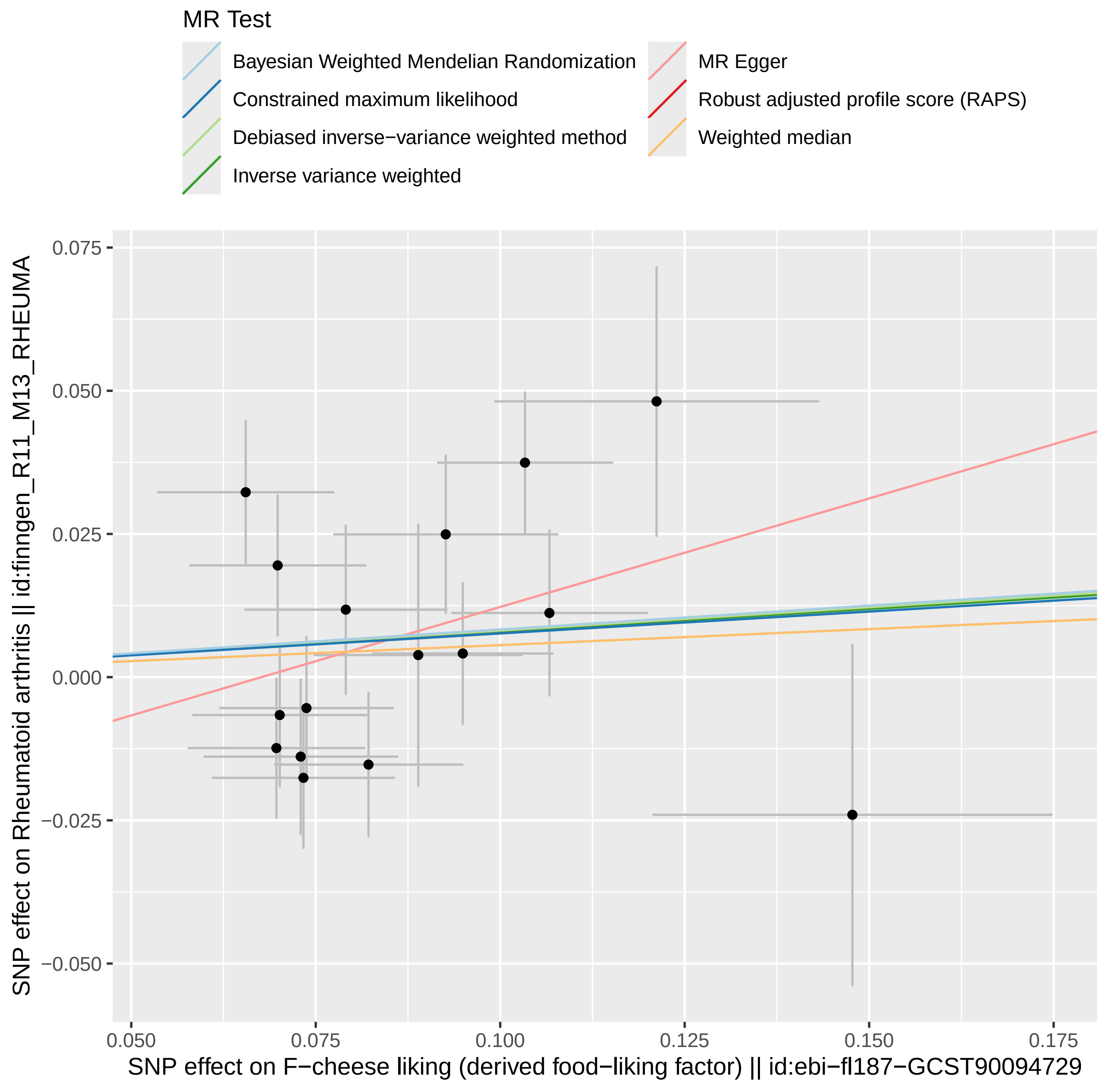

MR Test
Bayesian Weighted Mendelian Randomization
Constrained maximum likelihood
Debiased inverse−variance weighted method
Inverse variance weighted
MR Egger
Robust adjusted profile score (RAPS)
Weighted median
SNP effect on Rheumatoid arthritis || id:finngen_R11_M13_RHEUMA
0.075
0.050
0.025
0.000
−0.025
−0.050
0.050
0.075
0.100
0.125
0.150
0.175
SNP effect on F−cheese liking (derived food−liking factor) || id:ebi−fl187−GCST90094729

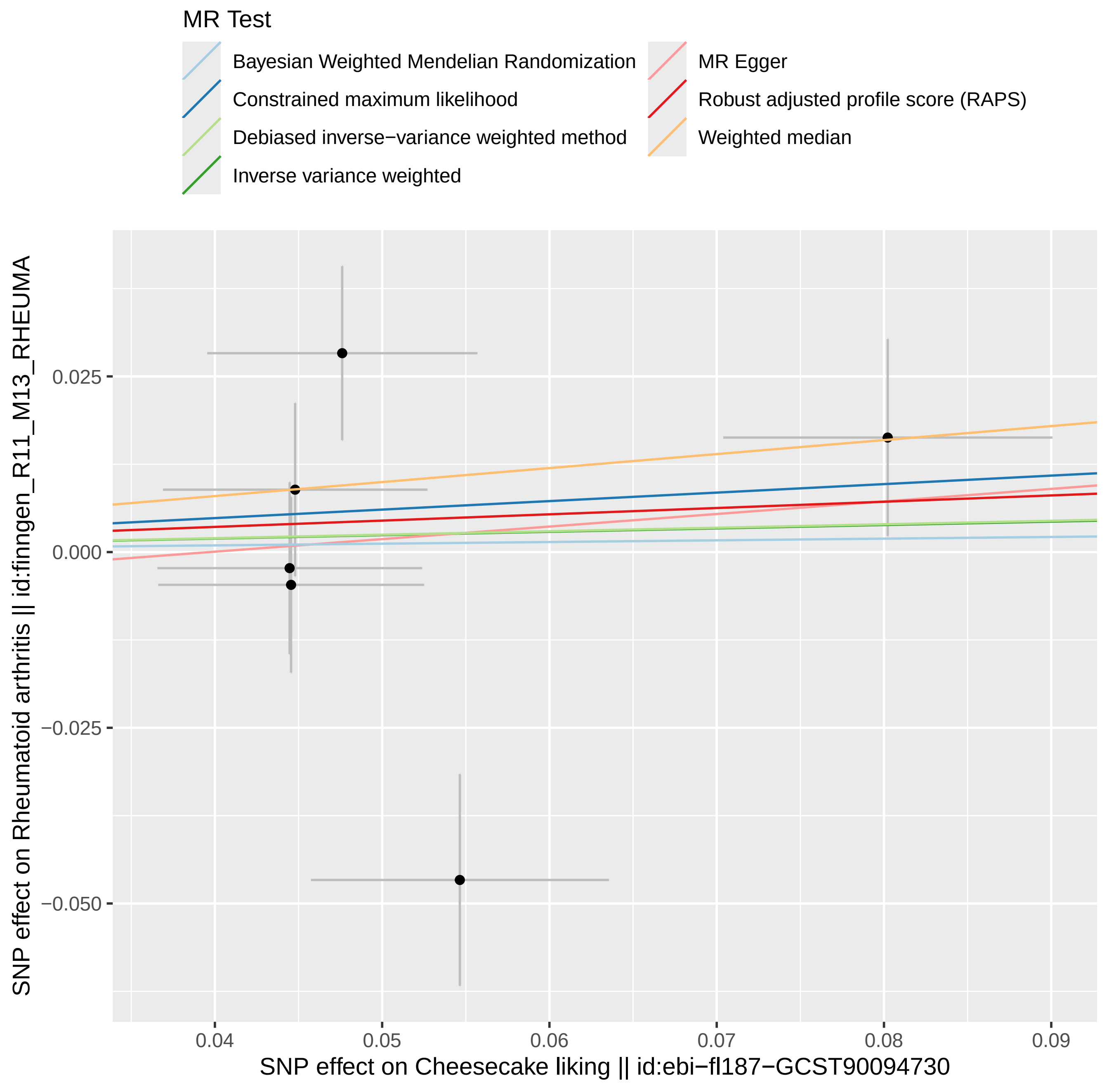

MR Test
Bayesian Weighted Mendelian Randomization
Constrained maximum likelihood
Debiased inverse−variance weighted method
Inverse variance weighted
MR Egger
Robust adjusted profile score (RAPS)
Weighted median
0.025
0.000
−0.025
−0.050
0.04
0.05
0.06
0.07
0.08
0.09
SNP effect on Cheesecake liking || id:ebi−fl187−GCST90094730
SNP effect on Rheumatoid arthritis || id:finngen_R11_M13_RHEUMA

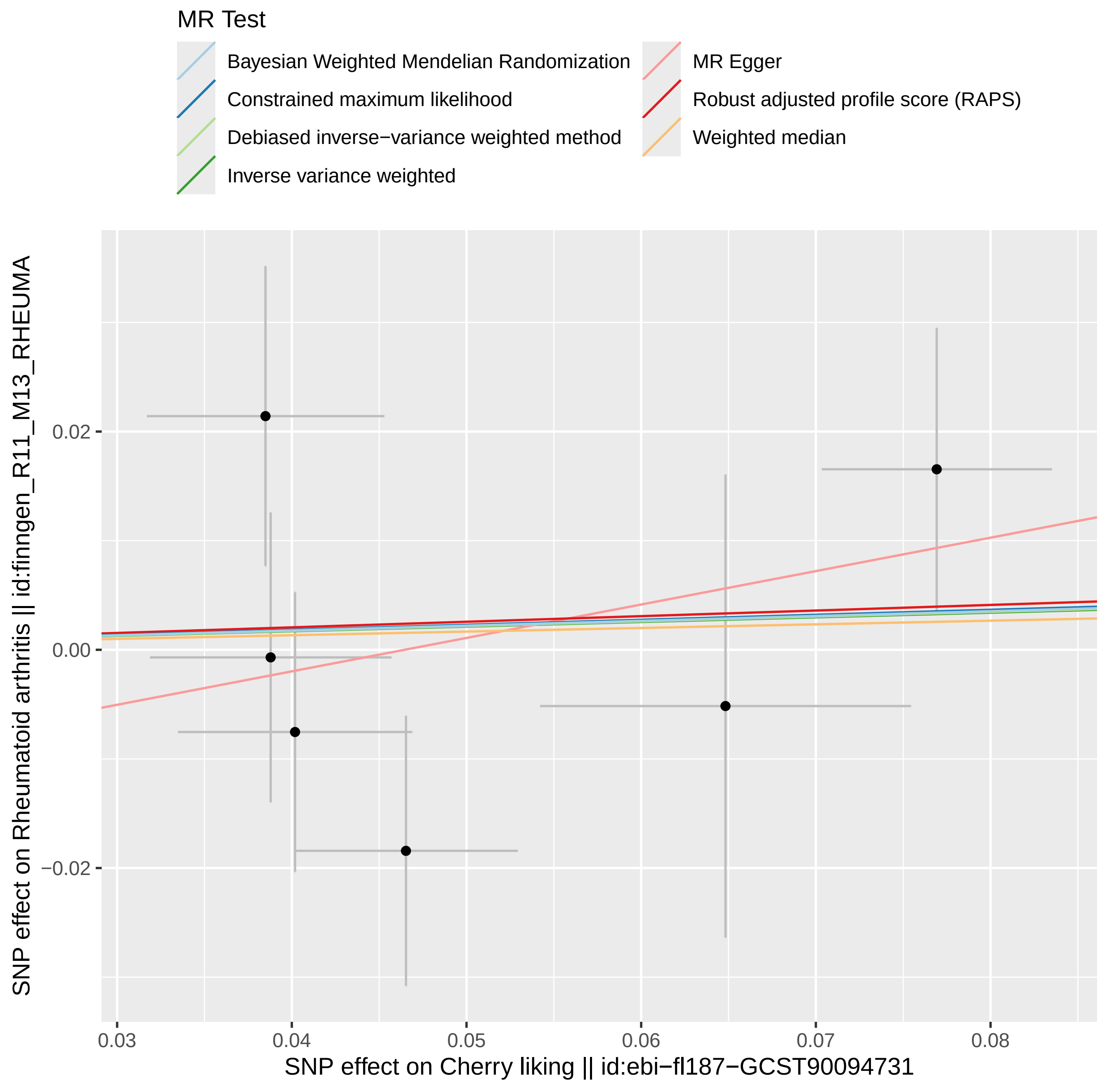

MR Test
Bayesian Weighted Mendelian Randomization
Constrained maximum likelihood
Debiased inverse−variance weighted method
Inverse variance weighted
MR Egger
Robust adjusted profile score (RAPS)
Weighted median
SNP effect on Rheumatoid arthritis || id:finngen_R11_M13_RHEUMA
0.02
0.00
−0.02
0.03
0.04
0.05
0.06
0.07
0.08
SNP effect on Cherry liking || id:ebi−fl187−GCST90094731

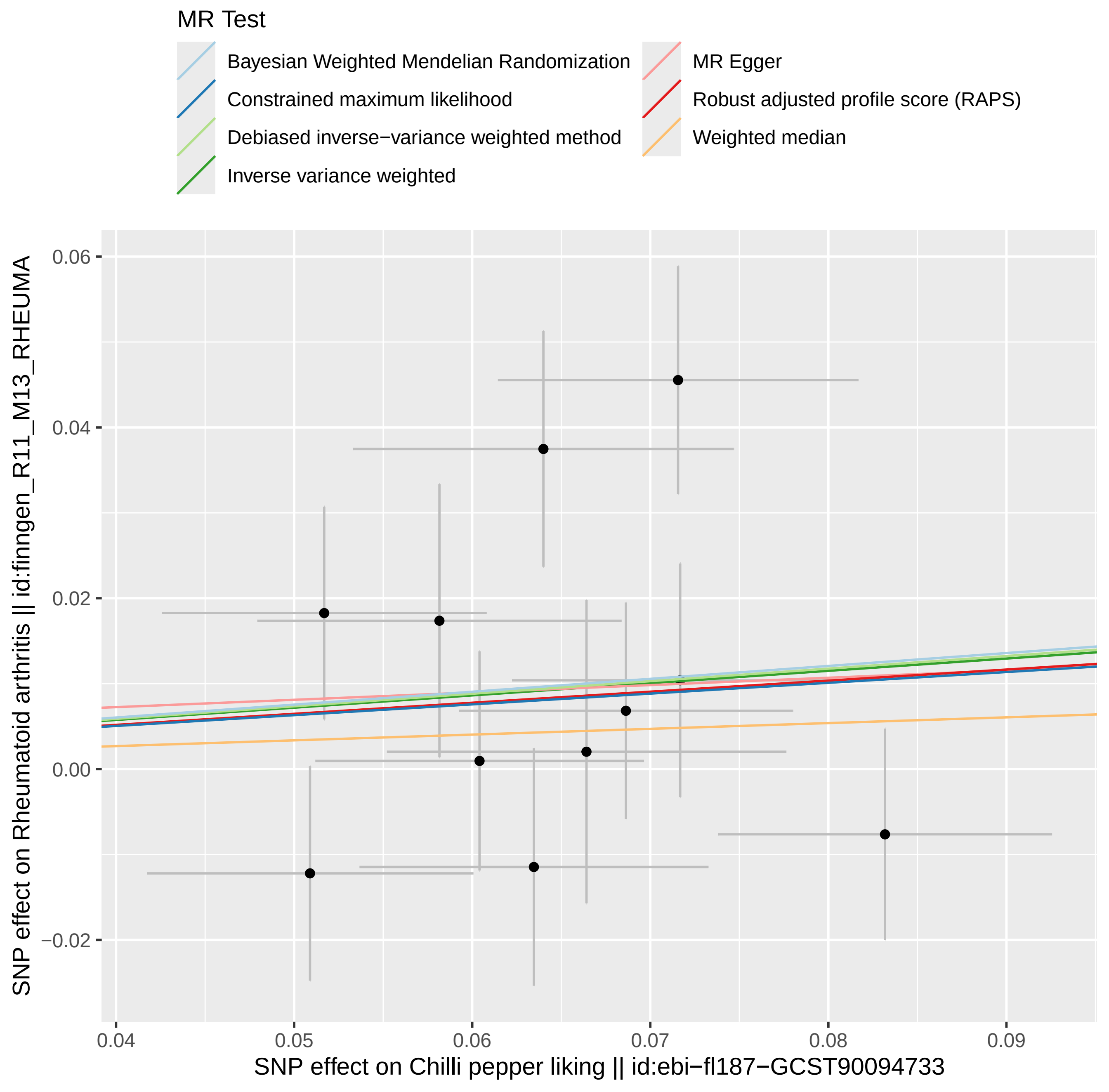
MR Test
Bayesian Weighted Mendelian Randomization
Constrained maximum likelihood
Debiased inverse−variance weighted method
Inverse variance weighted
MR Egger
Robust adjusted profile score (RAPS)
Weighted median
0.06
0.04
0.02
0.00
−0.02
0.04
0.05
0.06
0.07
0.08
0.09
SNP effect on Rheumatoid arthritis || id:finngen_R11_M13_RHEUMA
SNP effect on Chilli pepper liking || id:ebi−fl187−GCST90094733

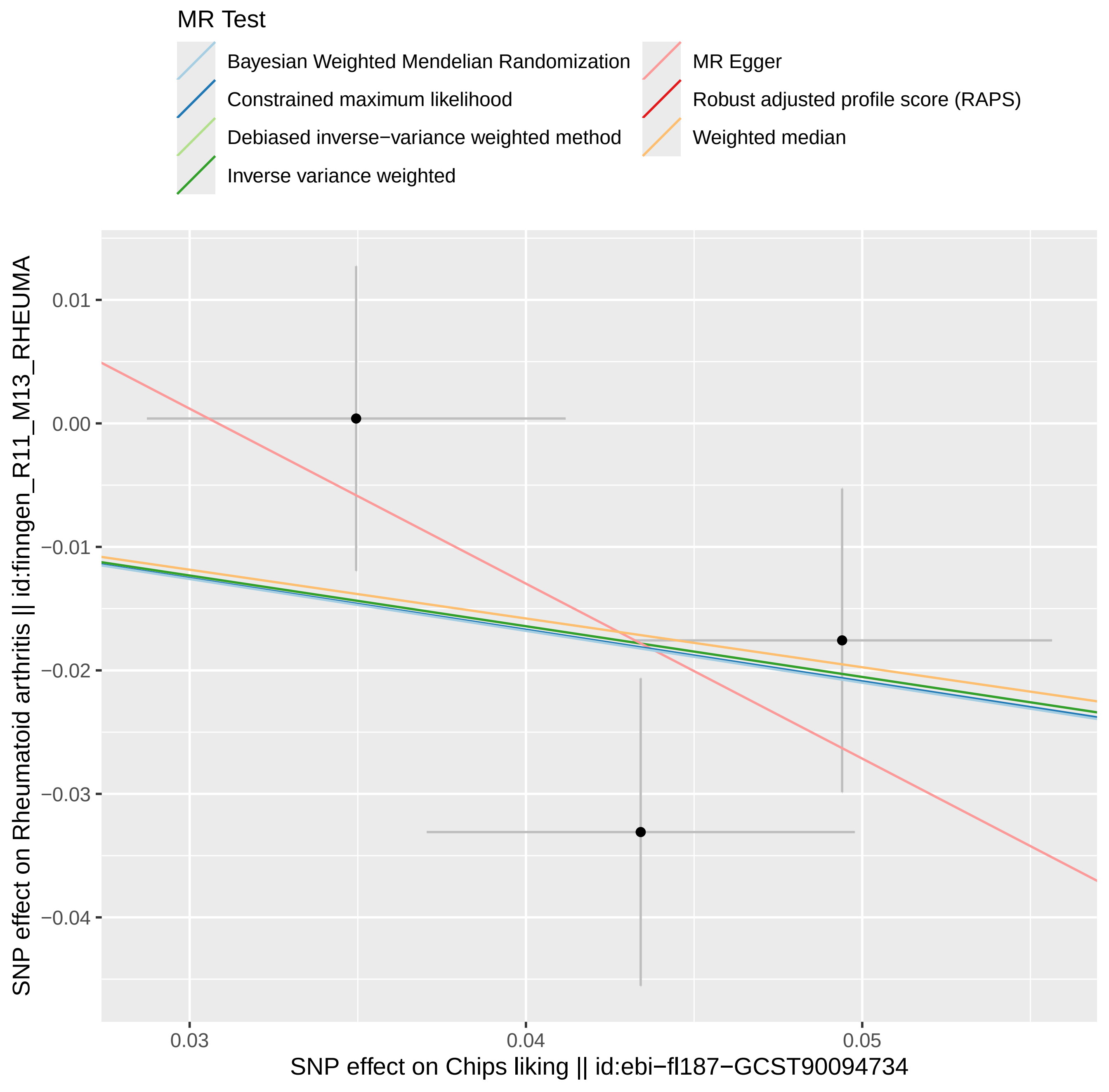
MR Test
Bayesian Weighted Mendelian Randomization
Constrained maximum likelihood
Debiased inverse−variance weighted method
Inverse variance weighted
MR Egger
Robust adjusted profile score (RAPS)
Weighted median
0.01
0.00
−0.01
−0.02
−0.03
−0.04
0.03
0.04
0.05
SNP effect on Rheumatoid arthritis || id:finngen_R11_M13_RHEUMA
SNP effect on Chips liking || id:ebi−fl187−GCST90094734

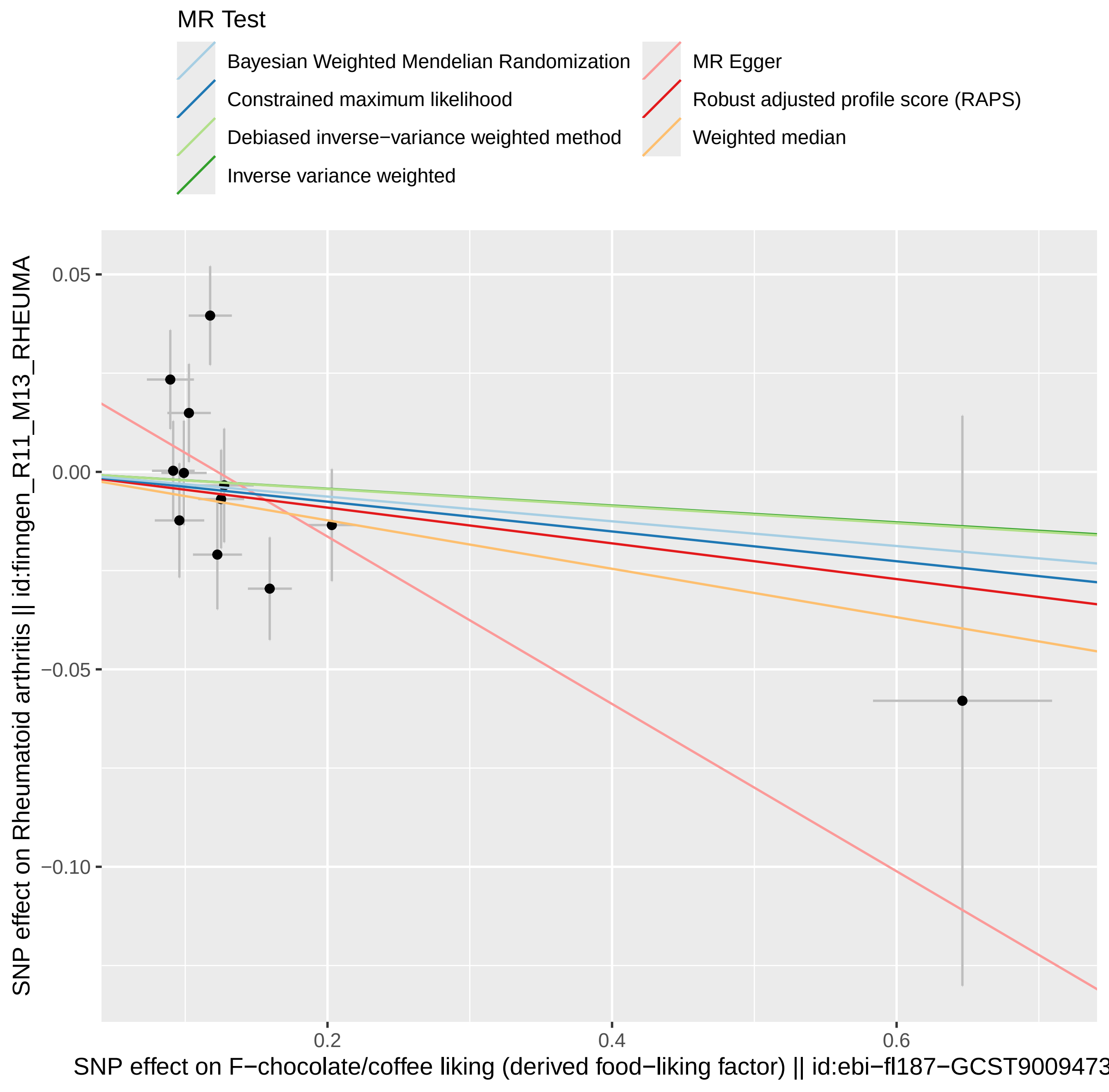
MR Test
Bayesian Weighted Mendelian Randomization
Constrained maximum likelihood
Debiased inverse−variance weighted method
Inverse variance weighted
MR Egger
Robust adjusted profile score (RAPS)
Weighted median
0.05
0.00
−0.05
−0.10
0.2
0.4
0.6
SNP effect on Rheumatoid arthritis || id:finngen_R11_M13_RHEUMA
SNP effect on F−chocolate/coffee liking (derived food−liking factor) || id:ebi−fl187−GCST9009473

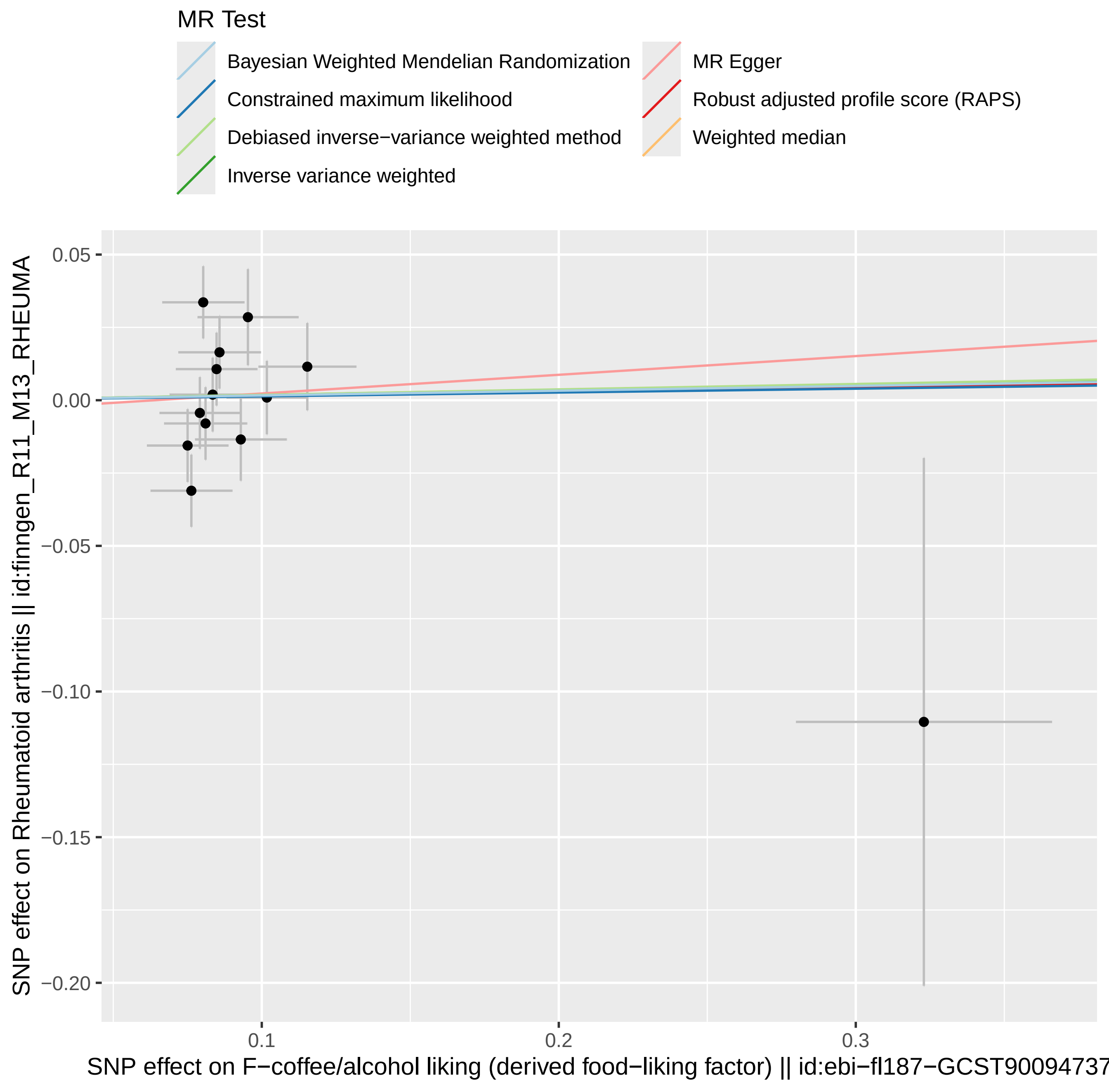
MR Test
Bayesian Weighted Mendelian Randomization
Constrained maximum likelihood
Debiased inverse−variance weighted method
Inverse variance weighted
MR Egger
Robust adjusted profile score (RAPS)
Weighted median
0.05
0.00
−0.05
−0.10
−0.15
−0.20
0.1
0.2
0.3
SNP effect on Rheumatoid arthritis || id:finngen_R11_M13_RHEUMA
SNP effect on F−coffee/alcohol liking (derived food−liking factor) || id:ebi−fl187−GCST90094737

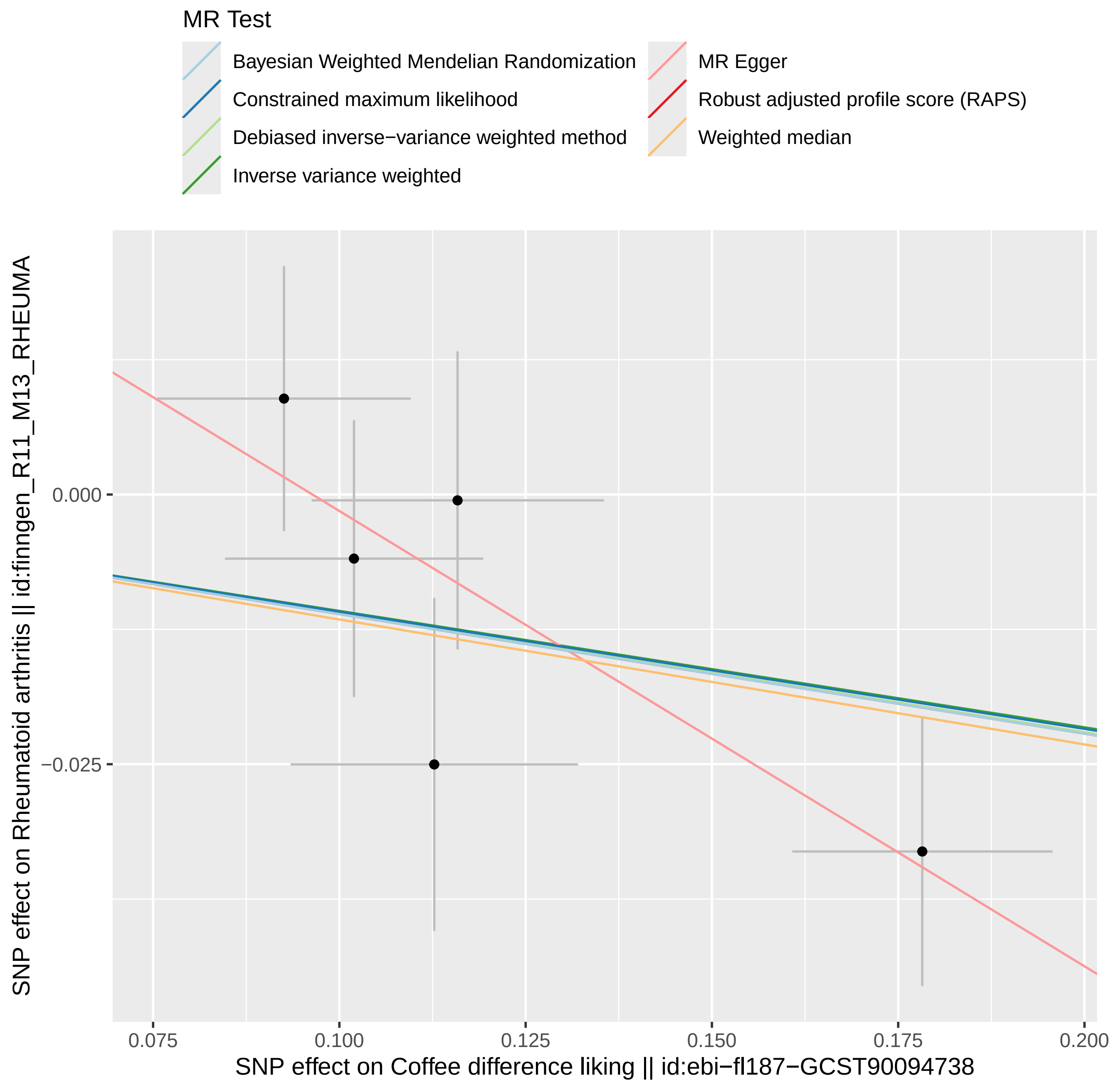

MR Test
Bayesian Weighted Mendelian Randomization
Constrained maximum likelihood
Debiased inverse−variance weighted method
Inverse variance weighted
MR Egger
Robust adjusted profile score (RAPS)
Weighted median
0.000
−0.025
0.075
0.100
0.125
0.150
0.175
0.200
SNP effect on Rheumatoid arthritis || id:finngen_R11_M13_RHEUMA
SNP effect on Coffee difference liking || id:ebi−fl187−GCST90094738

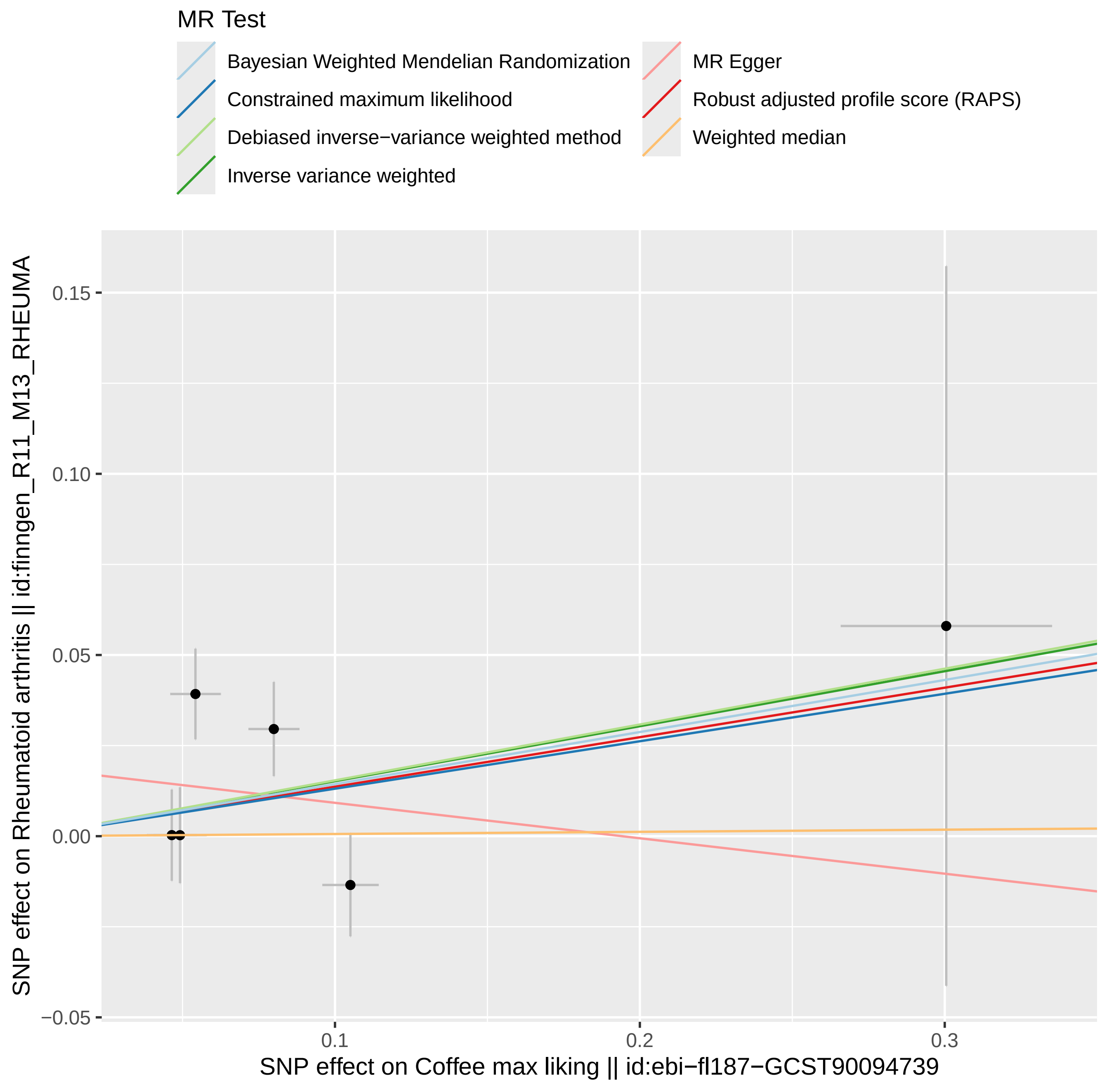
MR Test
Bayesian Weighted Mendelian Randomization
Constrained maximum likelihood
Debiased inverse−variance weighted method
Inverse variance weighted
MR Egger
Robust adjusted profile score (RAPS)
Weighted median
0.15
0.10
0.05
0.00
−0.05
0.1
0.2
0.3
SNP effect on Rheumatoid arthritis || id:finngen_R11_M13_RHEUMA
SNP effect on Coffee max liking || id:ebi−fl187−GCST90094739

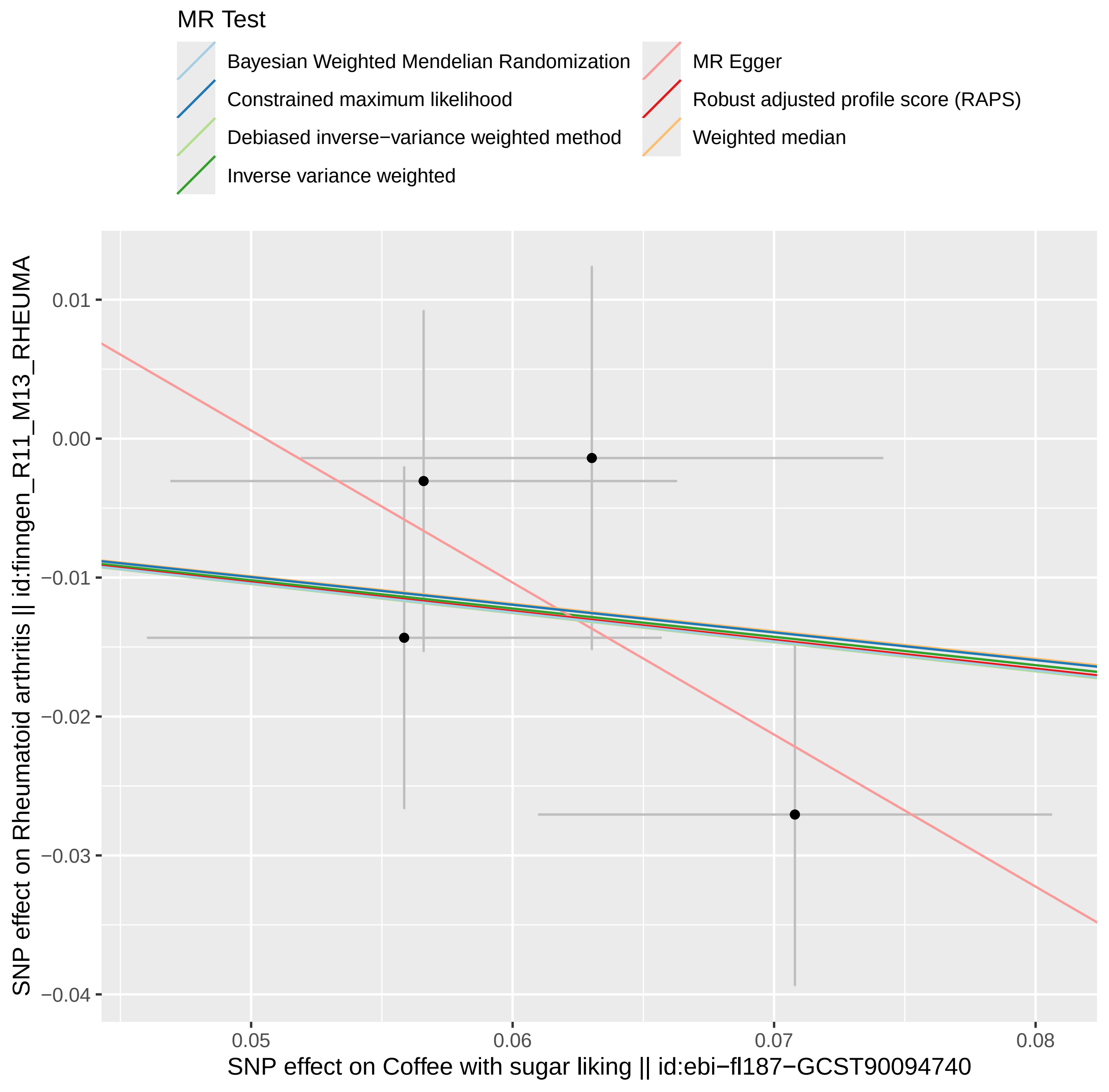

MR Test
Bayesian Weighted Mendelian Randomization
Constrained maximum likelihood
Debiased inverse−variance weighted method
Inverse variance weighted
MR Egger
Robust adjusted profile score (RAPS)
Weighted median
0.01
0.00
−0.01
−0.02
−0.03
−0.04
0.05
0.06
0.07
0.08
SNP effect on Rheumatoid arthritis || id:finngen_R11_M13_RHEUMA
SNP effect on Coffee with sugar liking || id:ebi−fl187−GCST90094740

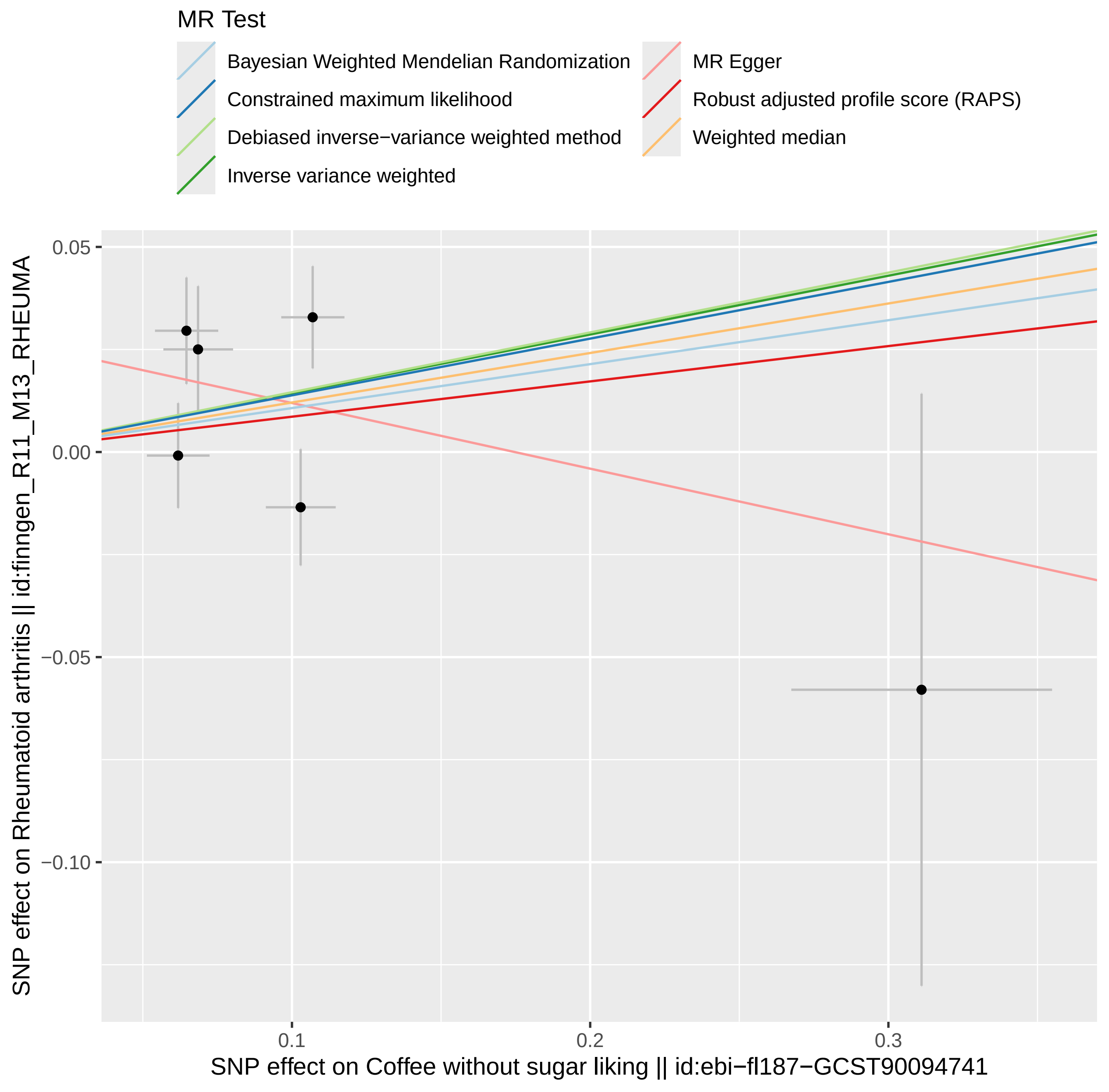
MR Test
Bayesian Weighted Mendelian Randomization
Constrained maximum likelihood
Debiased inverse−variance weighted method
Inverse variance weighted
MR Egger
Robust adjusted profile score (RAPS)
Weighted median
0.05
0.00
−0.05
−0.10
0.1
0.2
0.3
SNP effect on Rheumatoid arthritis || id:finngen_R11_M13_RHEUMA
SNP effect on Coffee without sugar liking || id:ebi−fl187−GCST90094741

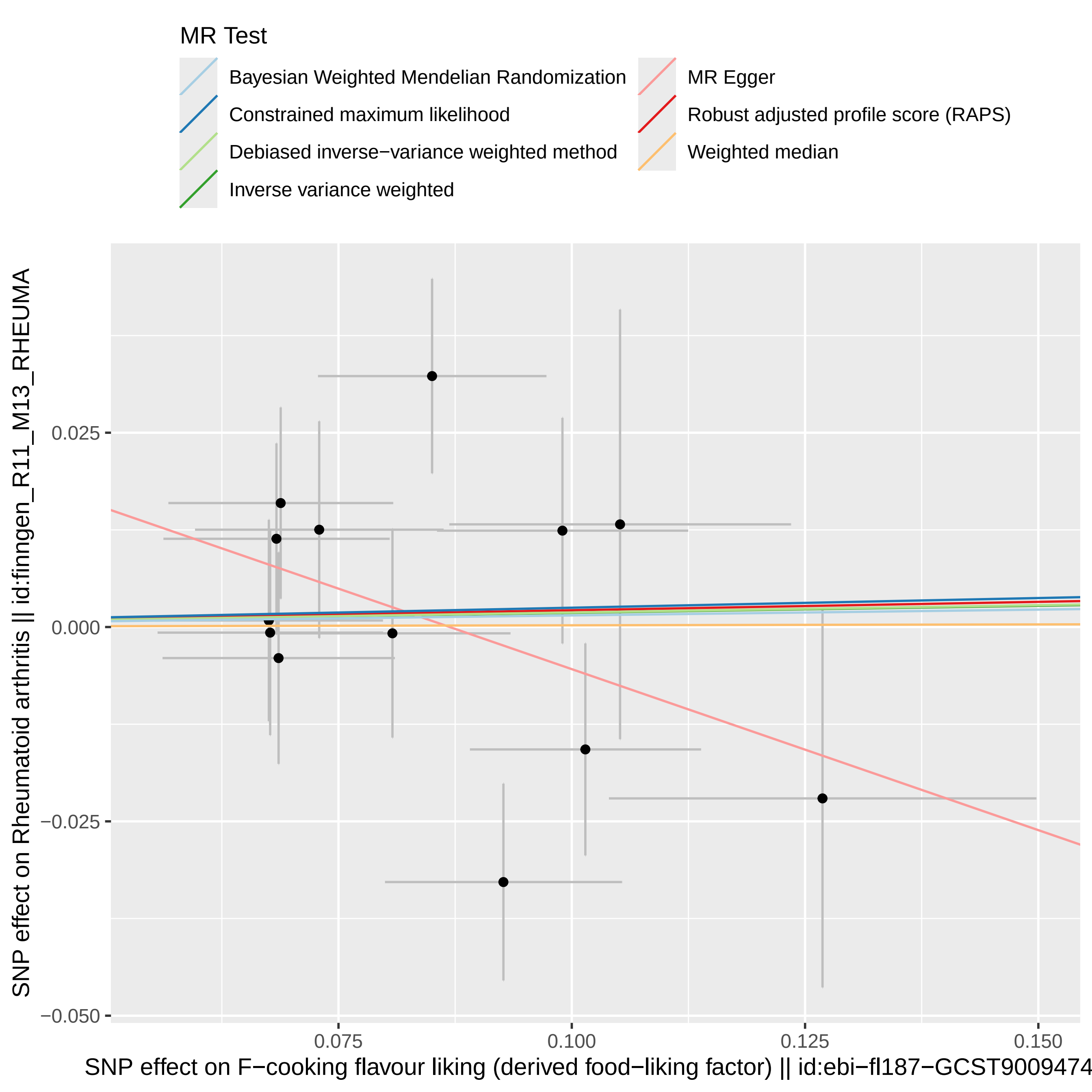

MR Test
Bayesian Weighted Mendelian Randomization
Constrained maximum likelihood
Debiased inverse−variance weighted method
Inverse variance weighted
MR Egger
Robust adjusted profile score (RAPS)
Weighted median
0.025
0.000
−0.025
−0.050
0.075
0.100
0.125
0.150
SNP effect on Rheumatoid arthritis || id:finngen_R11_M13_RHEUMA
SNP effect on F−cooking flavour liking (derived food−liking factor) || id:ebi−fl187−GCST9009474

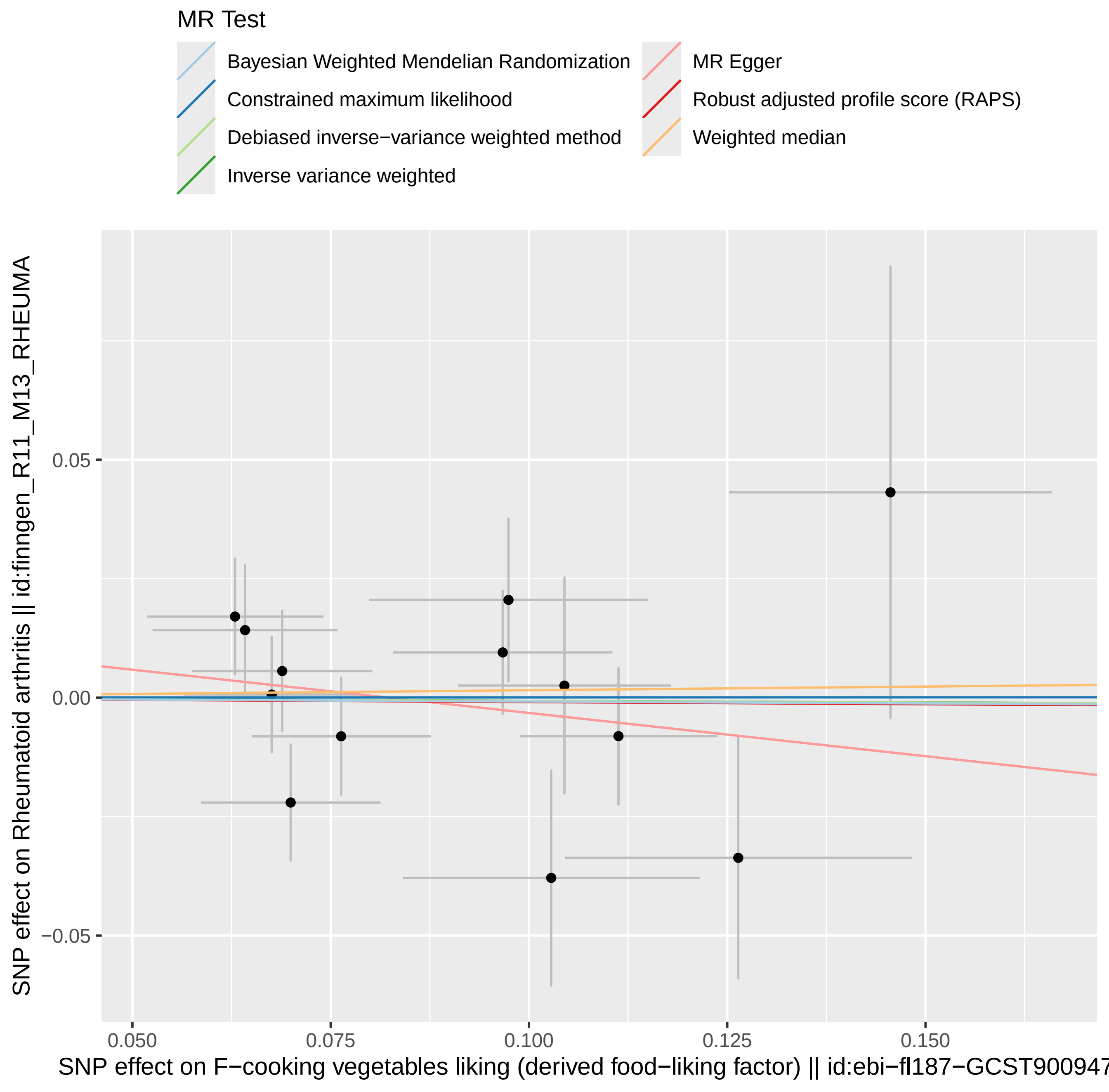
MR Test
Bayesian Weighted Mendelian Randomization
Constrained maximum likelihood
Debiased inverse−variance weighted method
Inverse variance weighted
MR Egger
Robust adjusted profile score (RAPS)
Weighted median
0.05
0.00
−0.05
0.050
0.075
0.100
0.125
0.150
SNP effect on Rheumatoid arthritis || id:finngen_R11_M13_RHEUMA
SNP effect on F−cooking vegetables liking (derived food−liking factor) || id:ebi−fl187−GCST900947

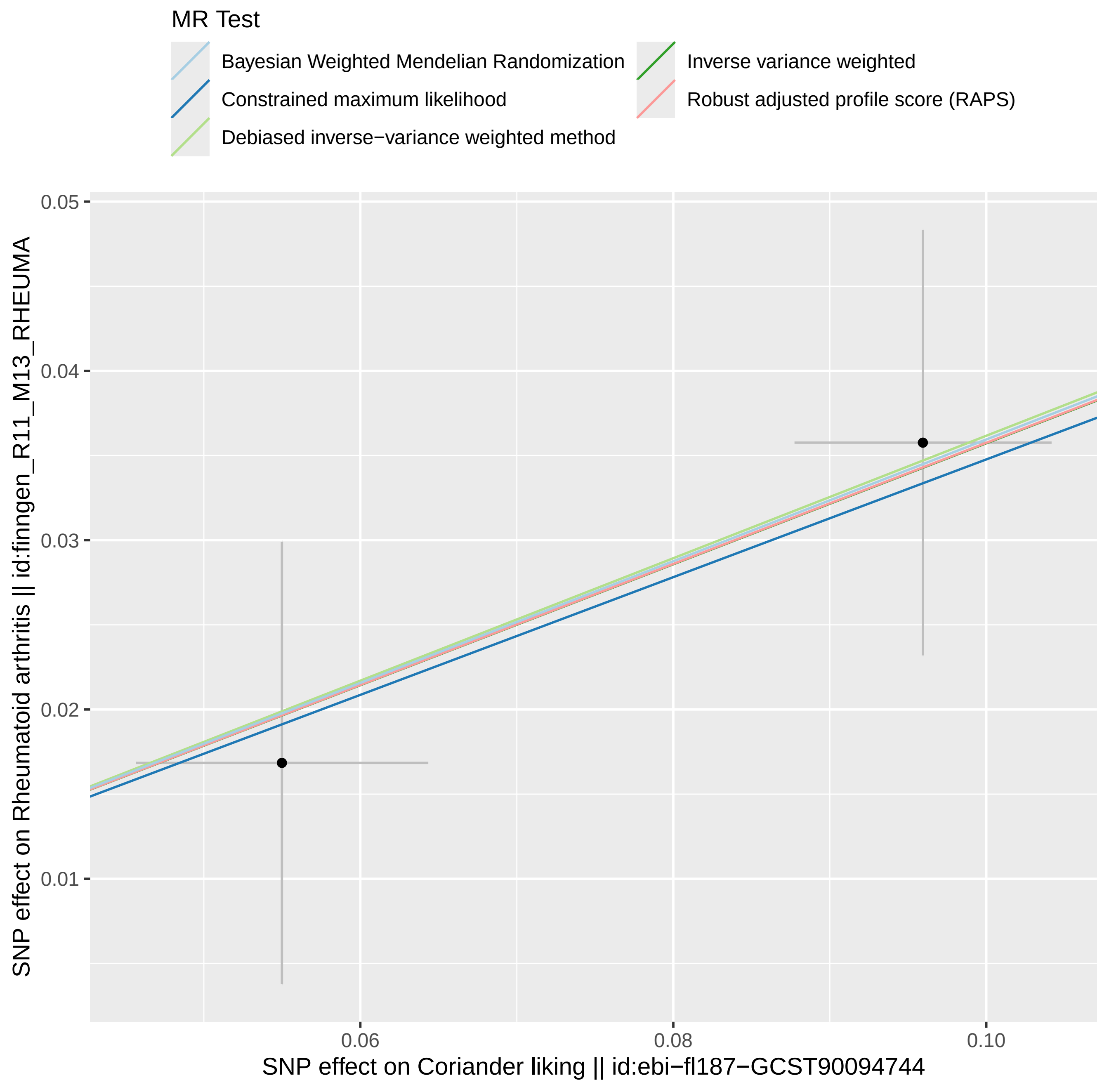

MR Test
Bayesian Weighted Mendelian Randomization
Constrained maximum likelihood
Debiased inverse−variance weighted method
Inverse variance weighted
Robust adjusted profile score (RAPS)
0.05
0.04
0.03
0.02
0.01
0.06
0.08
0.10
SNP effect on Rheumatoid arthritis || id:finngen_R11_M13_RHEUMA
SNP effect on Coriander liking || id:ebi−fl187−GCST90094744

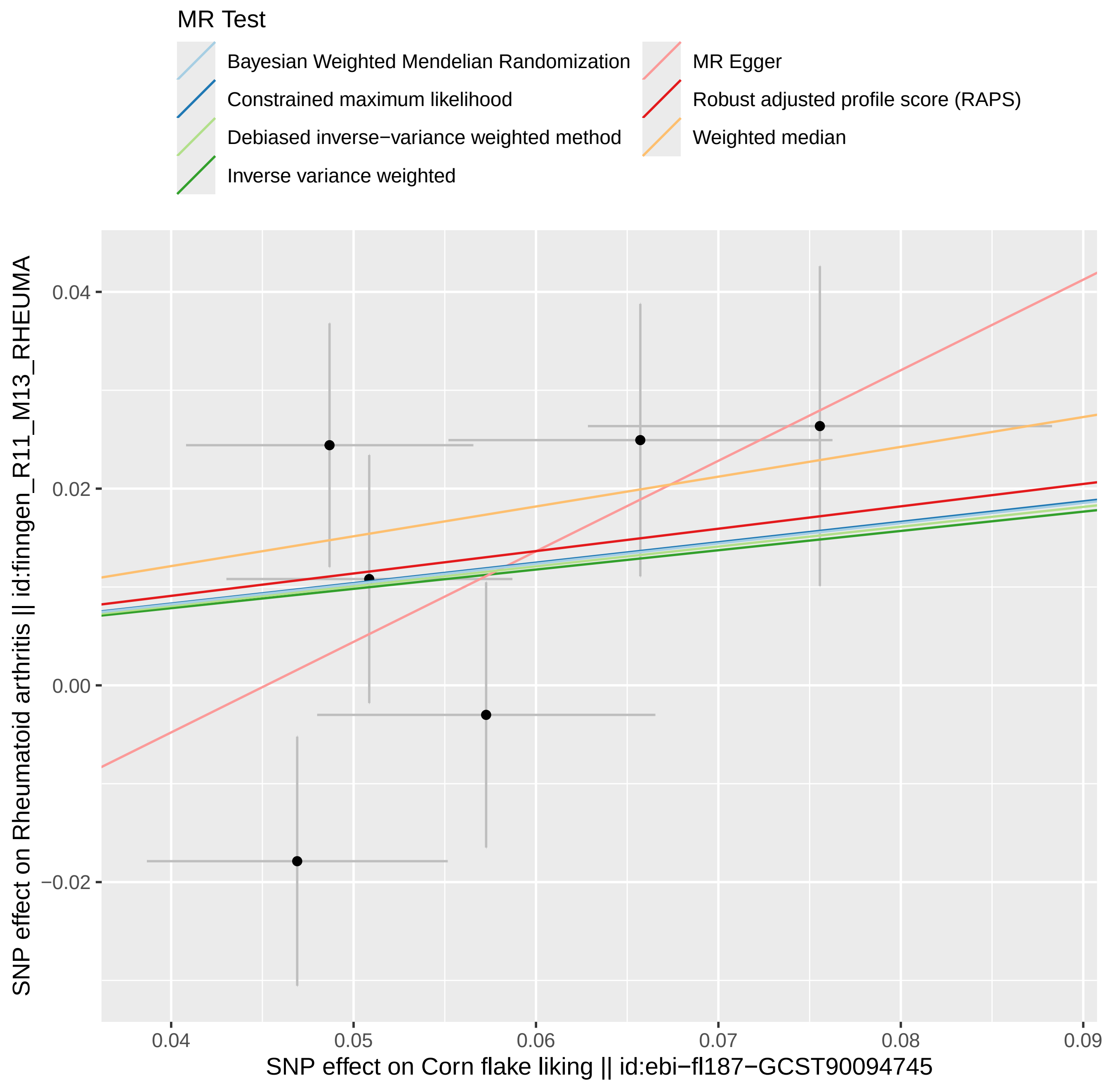
MR Test
Bayesian Weighted Mendelian Randomization
Constrained maximum likelihood
Debiased inverse−variance weighted method
Inverse variance weighted
MR Egger
Robust adjusted profile score (RAPS)
Weighted median
0.04
0.02
0.00
−0.02
0.04
0.05
0.06
0.07
0.08
0.09
SNP effect on Rheumatoid arthritis || id:finngen_R11_M13_RHEUMA
SNP effect on Corn flake liking || id:ebi−fl187−GCST90094745

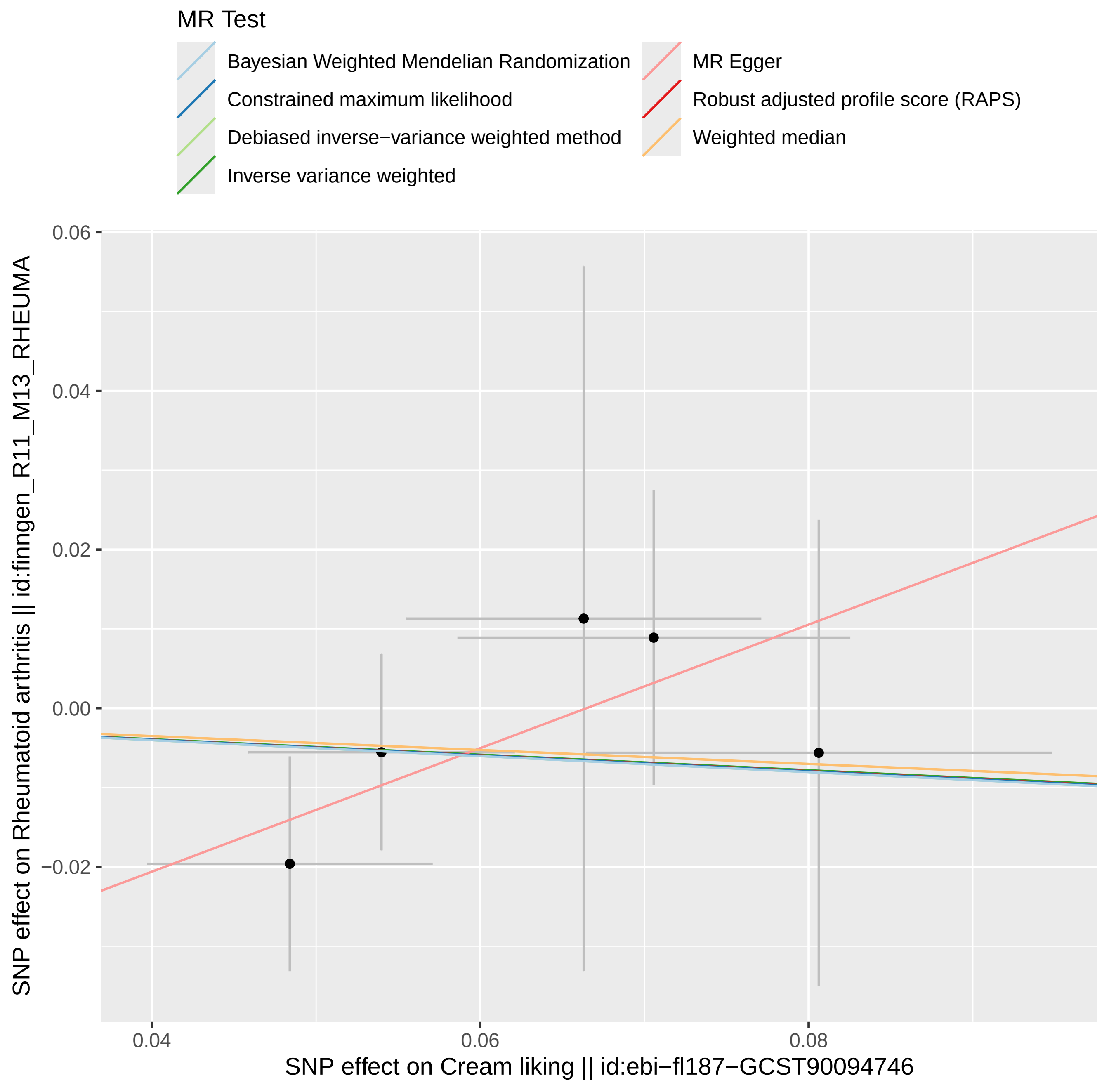

MR Test
Bayesian Weighted Mendelian Randomization
Constrained maximum likelihood
Debiased inverse−variance weighted method
Inverse variance weighted
MR Egger
Robust adjusted profile score (RAPS)
Weighted median
0.06
0.04
0.02
0.00
−0.02
0.04
0.06
0.08
SNP effect on Rheumatoid arthritis || id:finngen_R11_M13_RHEUMA
SNP effect on Cream liking || id:ebi−fl187−GCST90094746

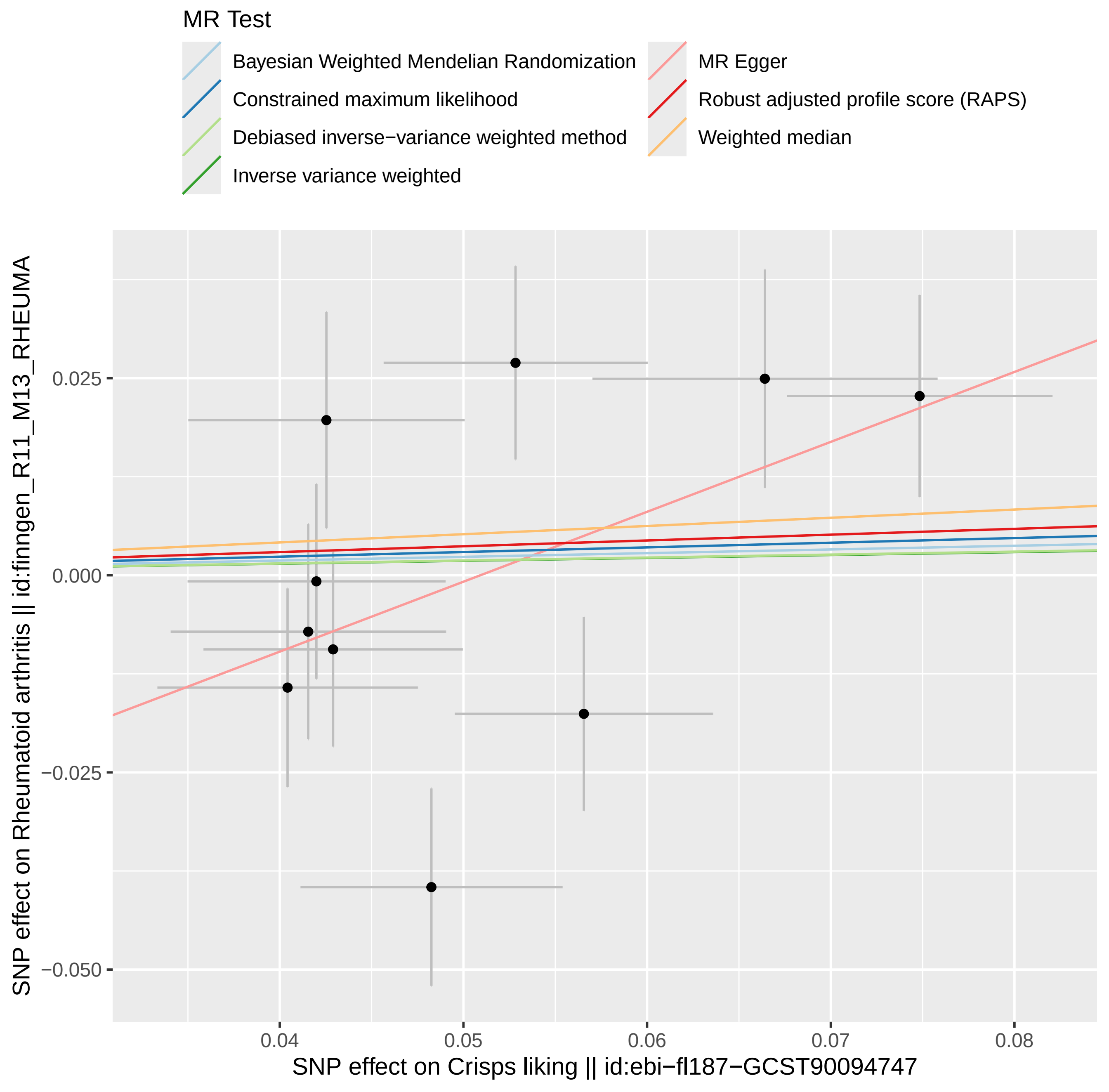
MR Test
Bayesian Weighted Mendelian Randomization
Constrained maximum likelihood
Debiased inverse−variance weighted method
Inverse variance weighted
MR Egger
Robust adjusted profile score (RAPS)
Weighted median
SNP effect on Rheumatoid arthritis || id:finngen_R11_M13_RHEUMA
0.025
0.000
−0.025
−0.050
0.04
0.05
0.06
0.07
0.08
SNP effect on Crisps liking || id:ebi−fl187−GCST90094747

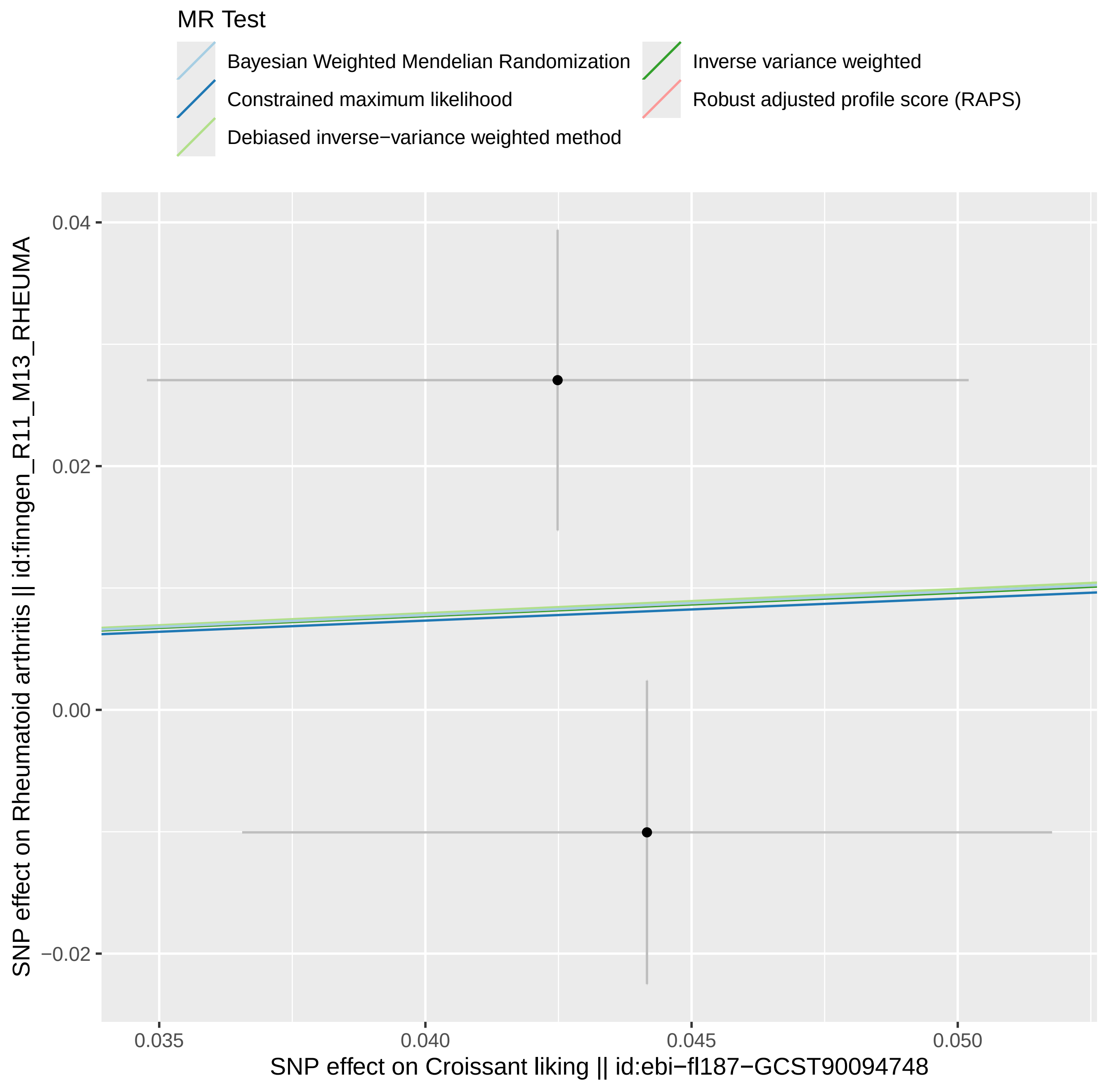
MR Test
Bayesian Weighted Mendelian Randomization
Constrained maximum likelihood
Debiased inverse−variance weighted method
Inverse variance weighted
Robust adjusted profile score (RAPS)
0.04
0.02
0.00
−0.02
0.035
0.040
0.045
0.050
SNP effect on Rheumatoid arthritis || id:finngen_R11_M13_RHEUMA
SNP effect on Croissant liking || id:ebi−fl187−GCST90094748

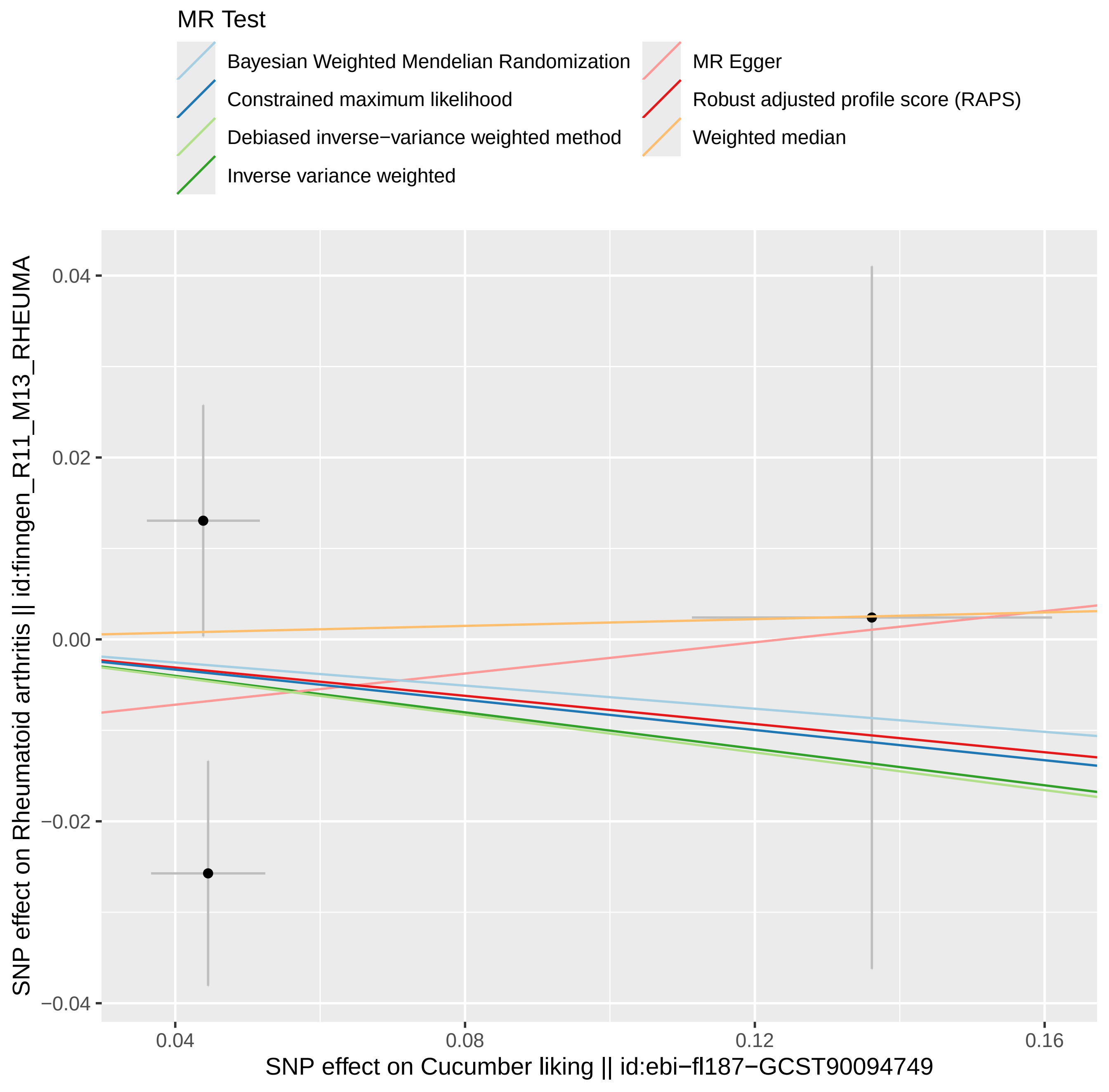
MR Test
Bayesian Weighted Mendelian Randomization
Constrained maximum likelihood
Debiased inverse−variance weighted method
Inverse variance weighted
MR Egger
Robust adjusted profile score (RAPS)
Weighted median
0.04
0.02
0.00
−0.02
−0.04
0.04
0.08
0.12
0.16
SNP effect on Rheumatoid arthritis || id:finngen_R11_M13_RHEUMA
SNP effect on Cucumber liking || id:ebi−fl187−GCST90094749

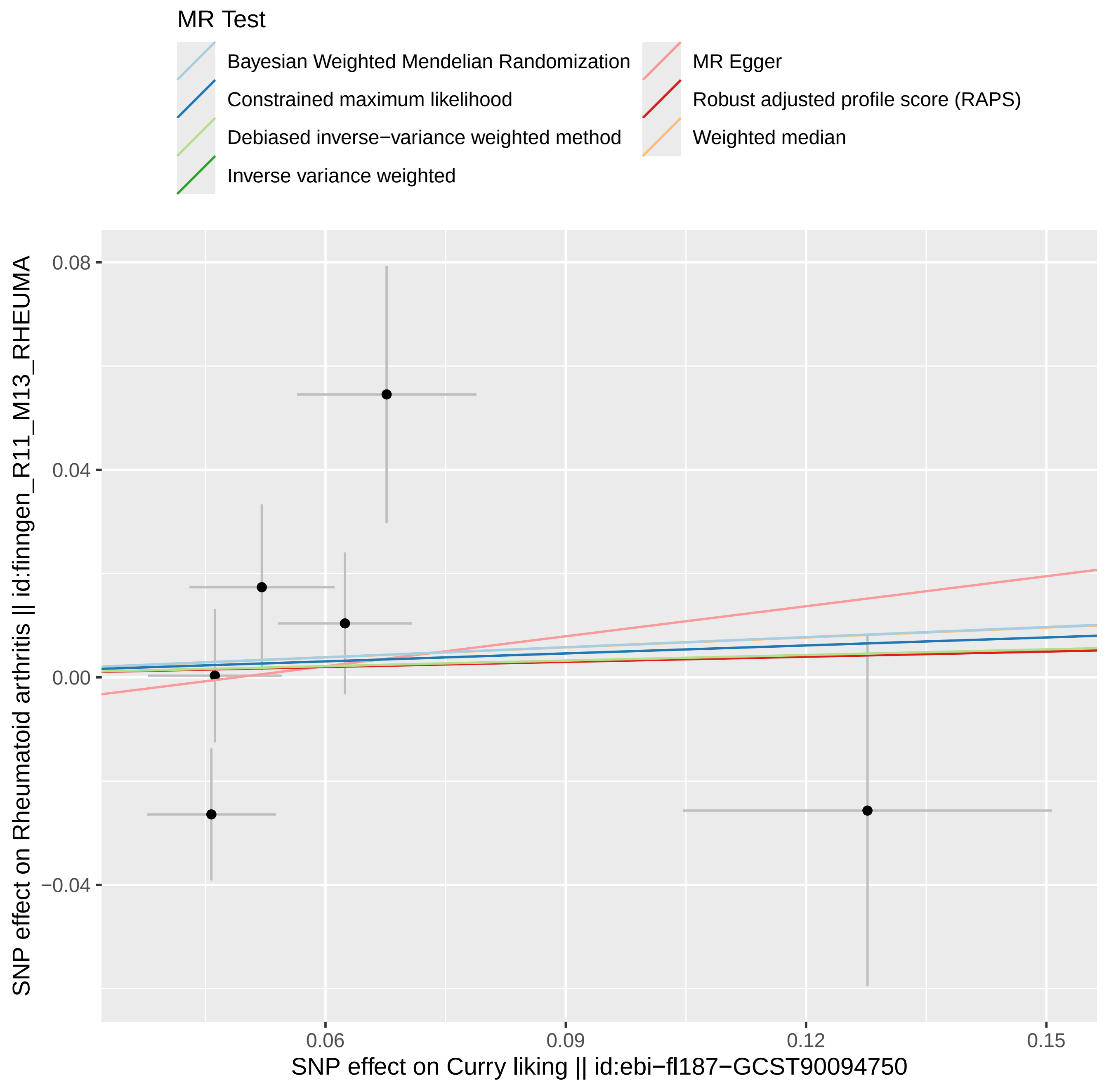

MR Test
Bayesian Weighted Mendelian Randomization
Constrained maximum likelihood
Debiased inverse−variance weighted method
Inverse variance weighted
MR Egger
Robust adjusted profile score (RAPS)
Weighted median
0.08
0.04
0.00
−0.04
0.06
0.09
0.12
0.15
SNP effect on Rheumatoid arthritis || id:finngen_R11_M13_RHEUMA
SNP effect on Curry liking || id:ebi−fl187−GCST90094750

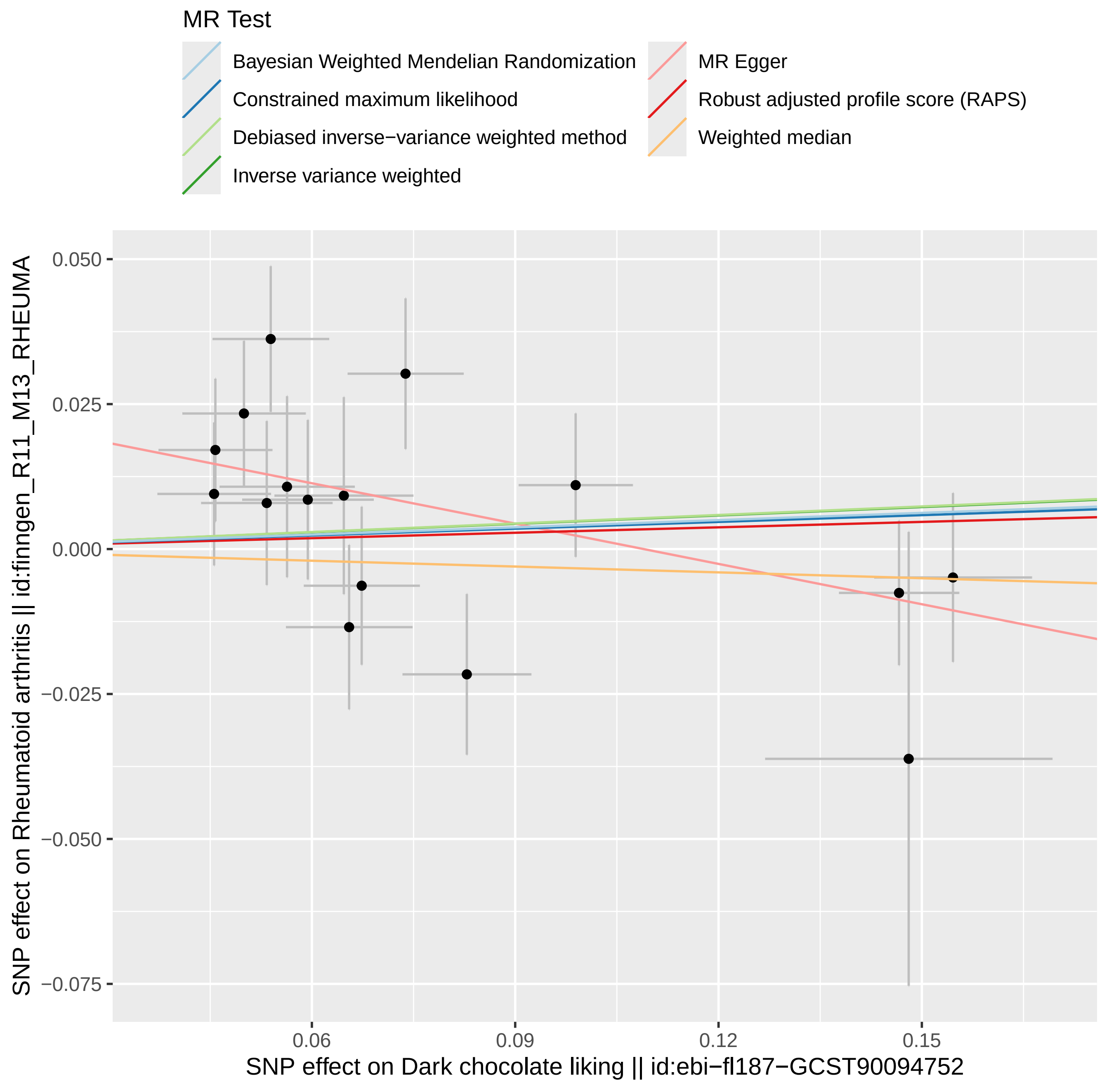
MR Test
Bayesian Weighted Mendelian Randomization
Constrained maximum likelihood
Debiased inverse−variance weighted method
Inverse variance weighted
MR Egger
Robust adjusted profile score (RAPS)
Weighted median
SNP effect on Rheumatoid arthritis || id:finngen_R11_M13_RHEUMA
0.050
0.025
0.000
−0.025
−0.050
−0.075
0.06
0.09
0.12
0.15
SNP effect on Dark chocolate liking || id:ebi−fl187−GCST90094752

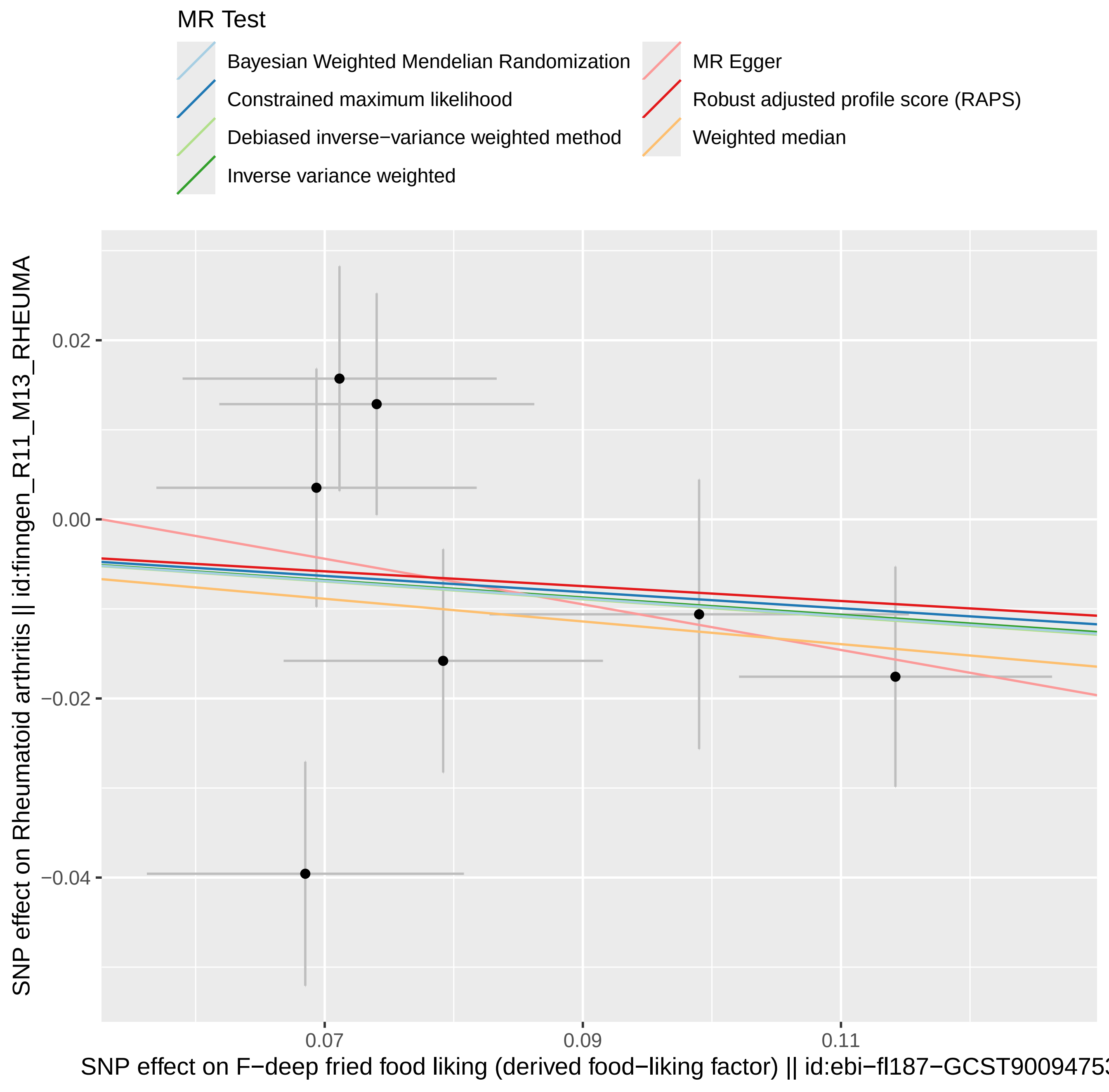
MR Test
Bayesian Weighted Mendelian Randomization
Constrained maximum likelihood
Debiased inverse−variance weighted method
Inverse variance weighted
MR Egger
Robust adjusted profile score (RAPS)
Weighted median
0.02
0.00
−0.02
−0.04
0.07
0.09
0.11
SNP effect on Rheumatoid arthritis || id:finngen_R11_M13_RHEUMA
SNP effect on F−deep fried food liking (derived food−liking factor) || id:ebi−fl187−GCST90094753

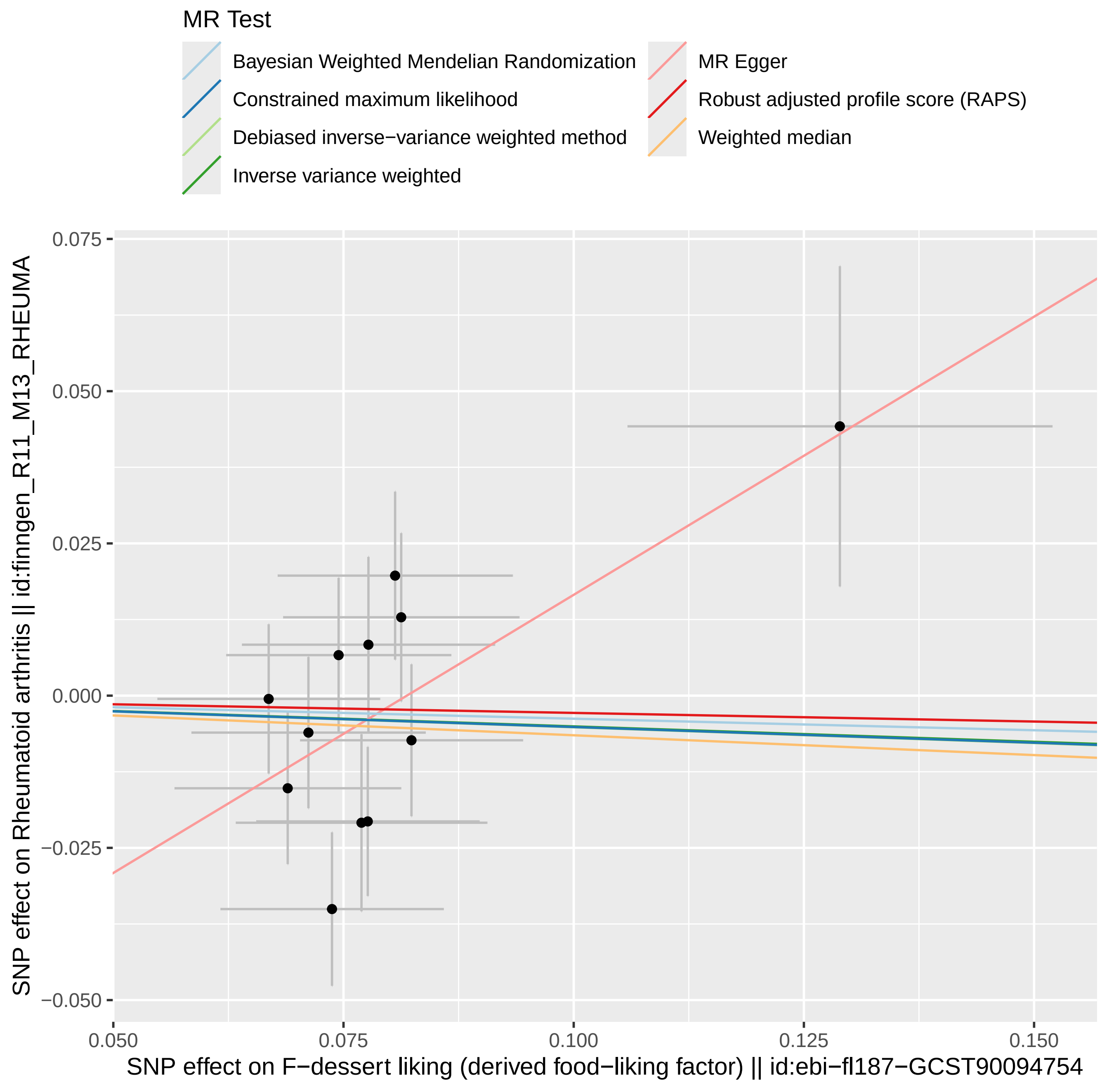
MR Test
Bayesian Weighted Mendelian Randomization
Constrained maximum likelihood
Debiased inverse−variance weighted method
Inverse variance weighted
MR Egger
Robust adjusted profile score (RAPS)
Weighted median
0.075
0.050
0.025
0.000
−0.025
−0.050
0.050
0.075
0.100
0.125
0.150
SNP effect on Rheumatoid arthritis || id:finngen_R11_M13_RHEUMA
SNP effect on F−dessert liking (derived food−liking factor) || id:ebi−fl187−GCST90094754

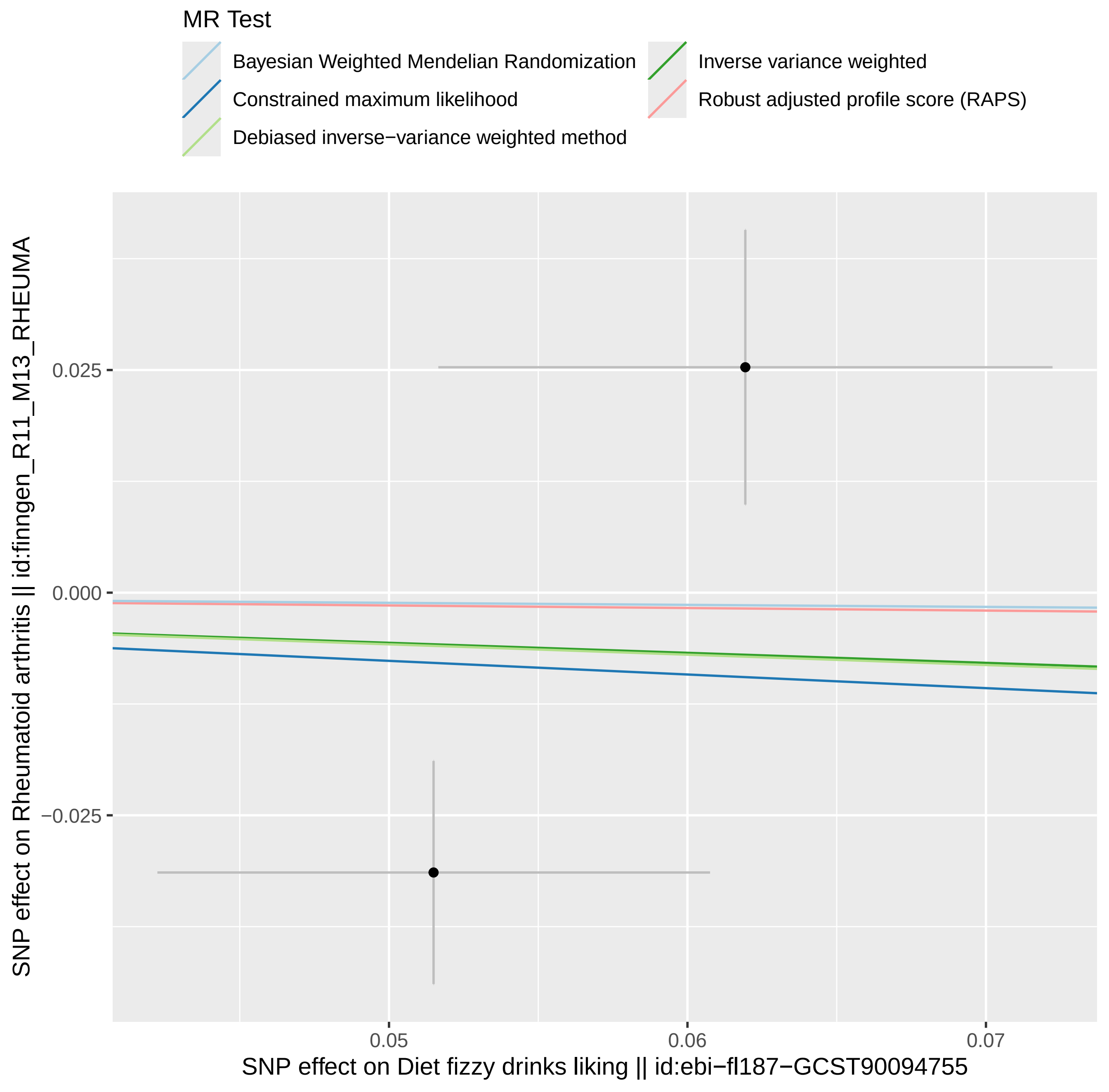

MR Test
Bayesian Weighted Mendelian Randomization
Constrained maximum likelihood
Debiased inverse−variance weighted method
Inverse variance weighted
Robust adjusted profile score (RAPS)
0.025
0.000
−0.025
0.05
0.06
0.07
SNP effect on Rheumatoid arthritis || id:finngen_R11_M13_RHEUMA
SNP effect on Diet fizzy drinks liking || id:ebi−fl187−GCST90094755

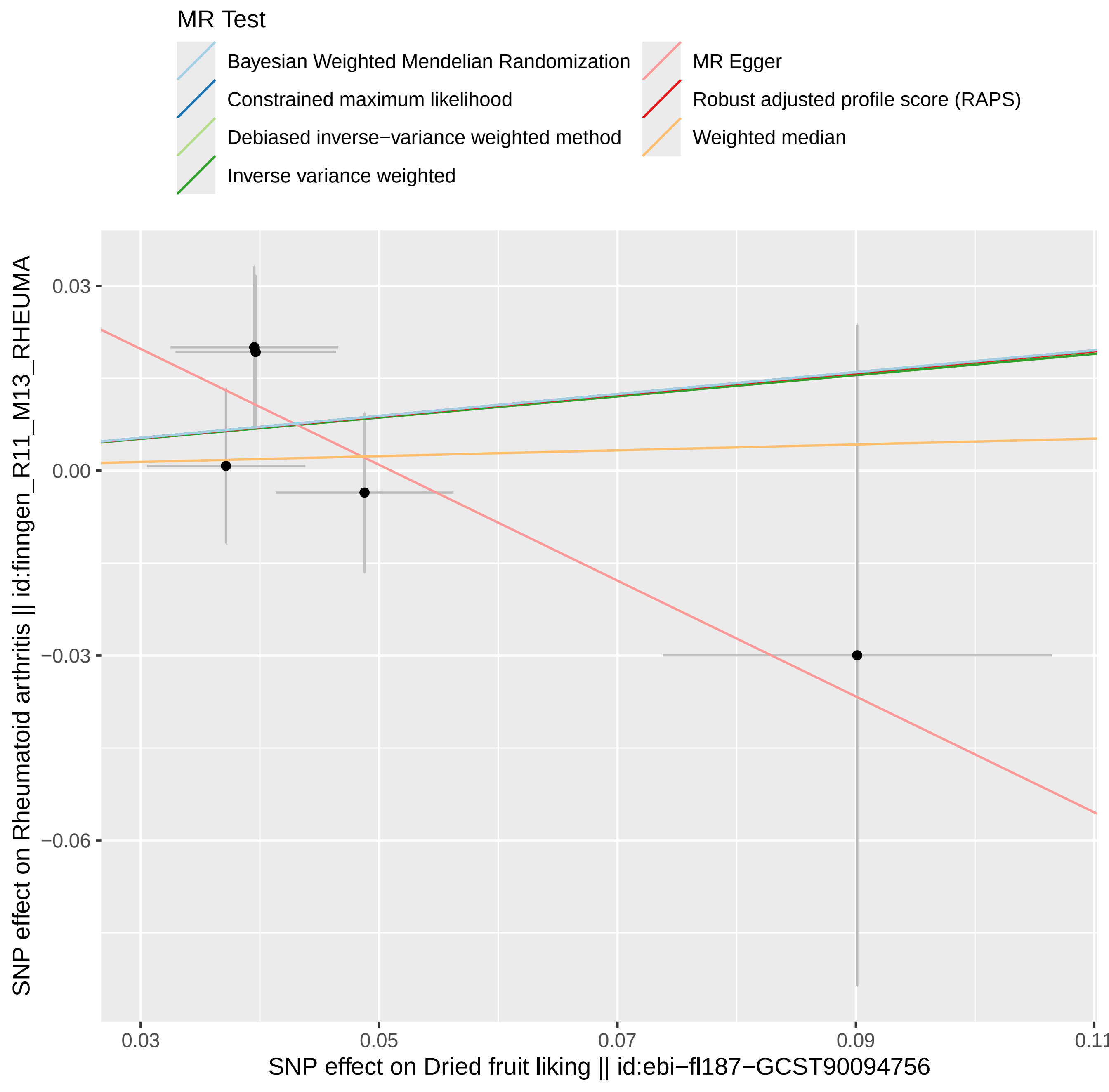
MR Test
Bayesian Weighted Mendelian Randomization
Constrained maximum likelihood
Debiased inverse−variance weighted method
Inverse variance weighted
MR Egger
Robust adjusted profile score (RAPS)
Weighted median
0.03
0.00
−0.03
−0.06
0.03
0.05
0.07
0.09
0.11
SNP effect on Rheumatoid arthritis || id:finngen_R11_M13_RHEUMA
SNP effect on Dried fruit liking || id:ebi−fl187−GCST90094756

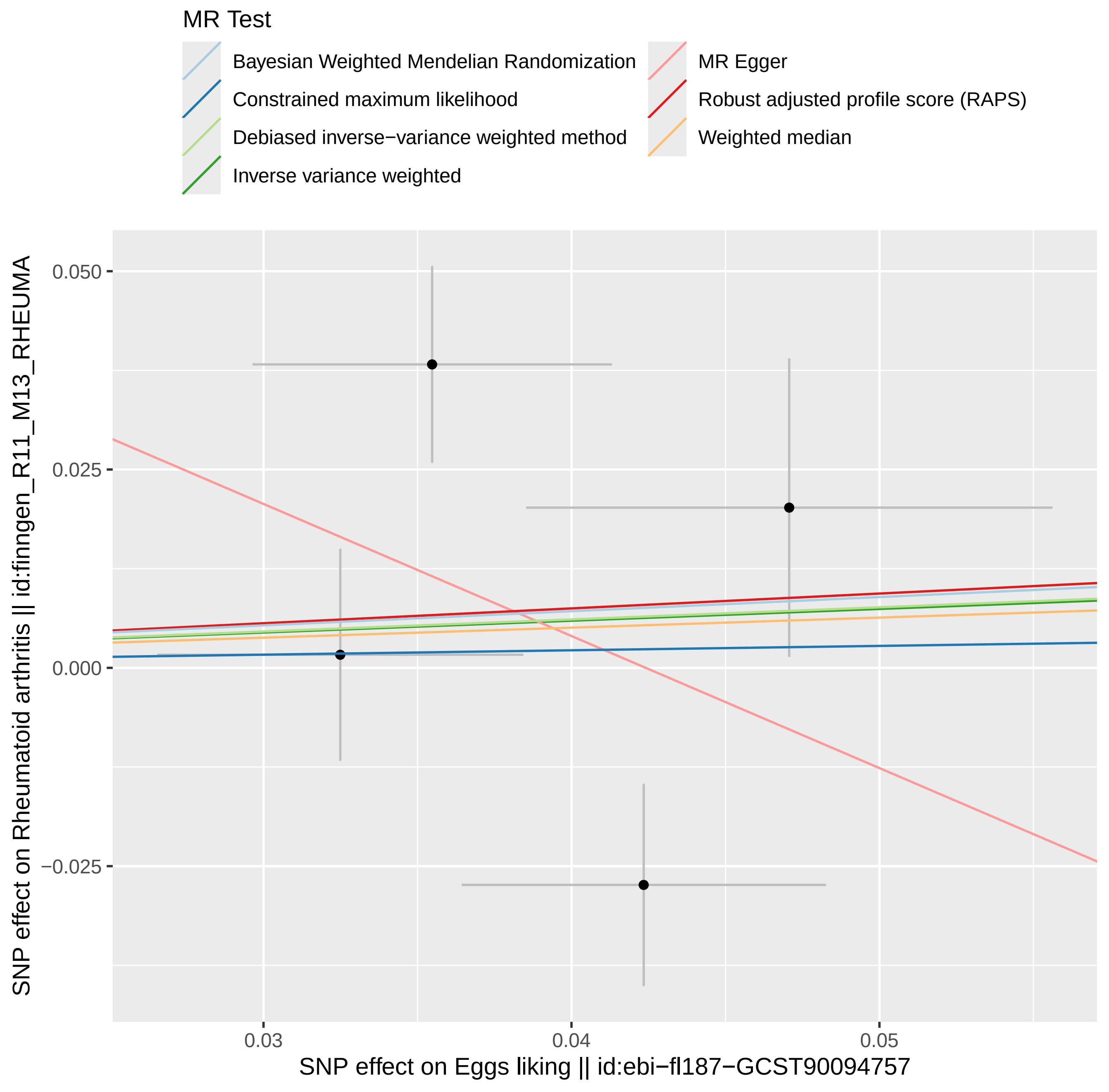

MR Test
Bayesian Weighted Mendelian Randomization
Constrained maximum likelihood
Debiased inverse−variance weighted method
Inverse variance weighted
MR Egger
Robust adjusted profile score (RAPS)
Weighted median
0.050
0.025
0.000
−0.025
0.03
0.04
0.05
SNP effect on Rheumatoid arthritis || id:finngen_R11_M13_RHEUMA
SNP effect on Eggs liking || id:ebi−fl187−GCST90094757

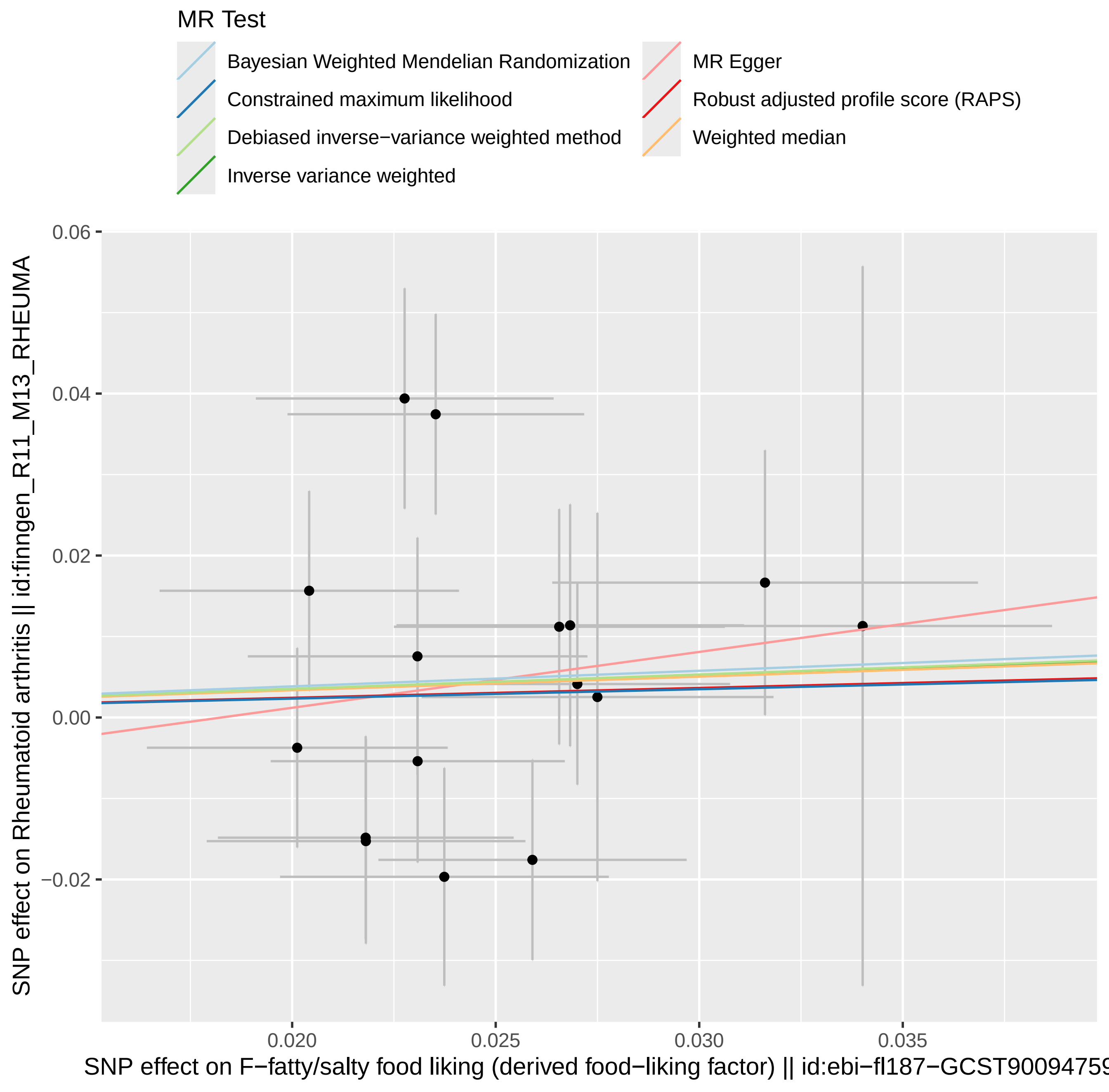

MR Test
Bayesian Weighted Mendelian Randomization
Constrained maximum likelihood
Debiased inverse−variance weighted method
Inverse variance weighted
MR Egger
Robust adjusted profile score (RAPS)
Weighted median
0.06
0.04
0.02
0.00
−0.02
0.020
0.025
0.030
0.035
SNP effect on Rheumatoid arthritis || id:finngen_R11_M13_RHEUMA
SNP effect on F−fatty/salty food liking (derived food−liking factor) || id:ebi−fl187−GCST90094759

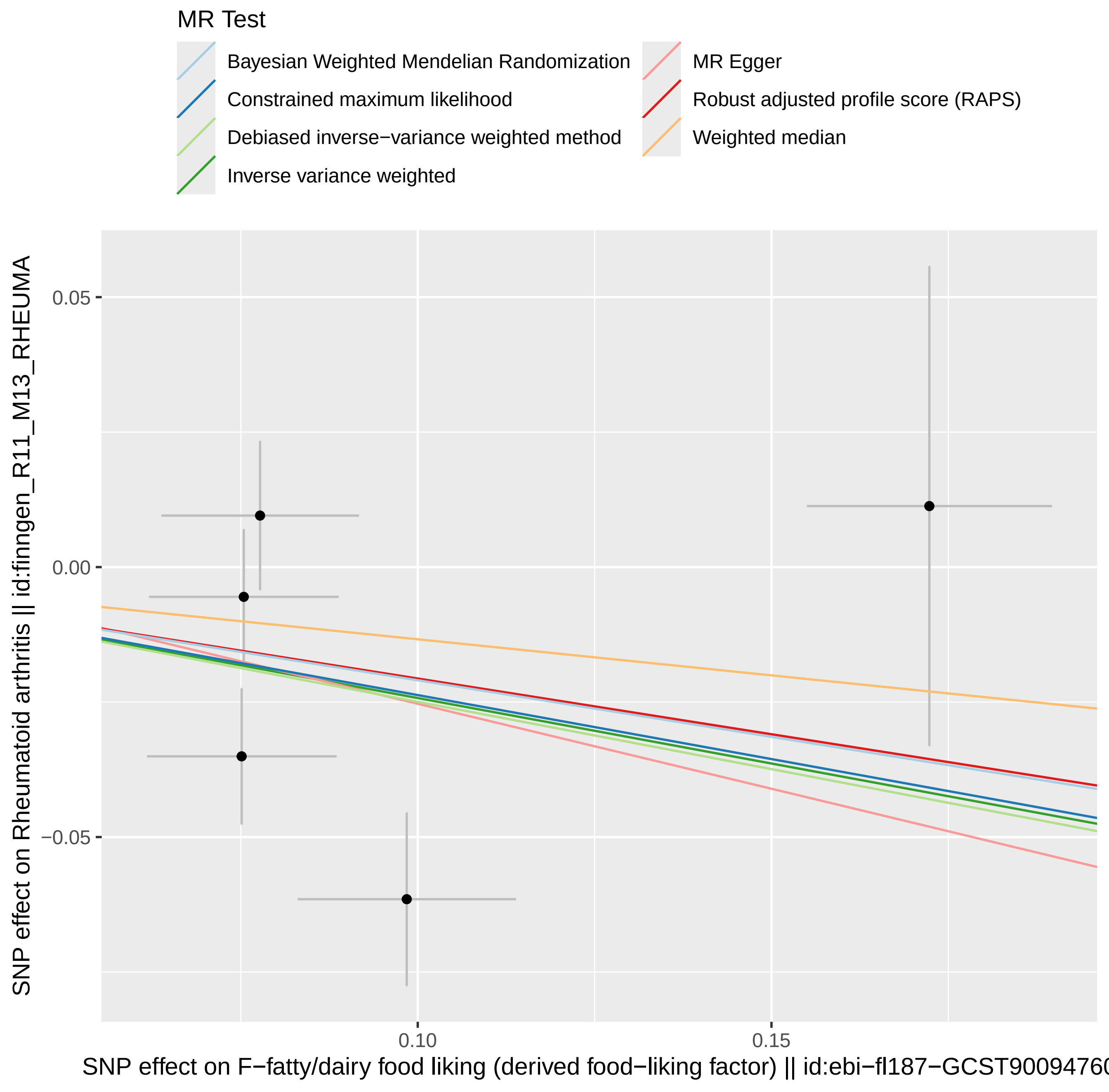

MR Test
Bayesian Weighted Mendelian Randomization
Constrained maximum likelihood
Debiased inverse−variance weighted method
Inverse variance weighted
MR Egger
Robust adjusted profile score (RAPS)
Weighted median
0.05
0.00
−0.05
0.10
0.15
SNP effect on Rheumatoid arthritis || id:finngen_R11_M13_RHEUMA
SNP effect on F−fatty/dairy food liking (derived food−liking factor) || id:ebi−fl187−GCST9009476

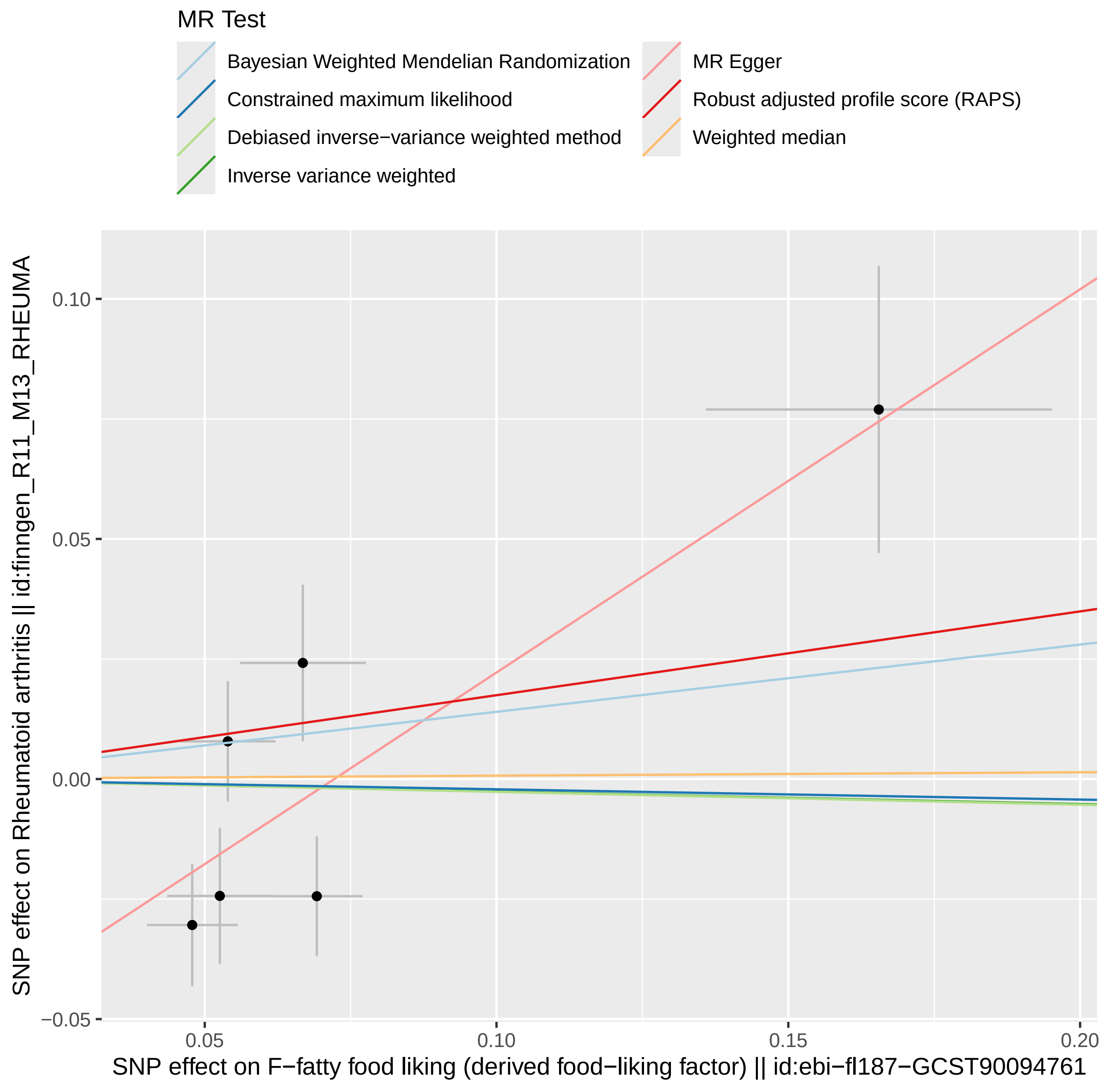
MR Test
Bayesian Weighted Mendelian Randomization
Constrained maximum likelihood
Debiased inverse−variance weighted method
Inverse variance weighted
MR Egger
Robust adjusted profile score (RAPS)
Weighted median
0.10
0.05
0.00
−0.05
0.05
0.10
0.15
0.20
SNP effect on Rheumatoid arthritis || id:finngen_R11_M13_RHEUMA
SNP effect on F−fatty food liking (derived food−liking factor) || id:ebi−fl187−GCST90094761

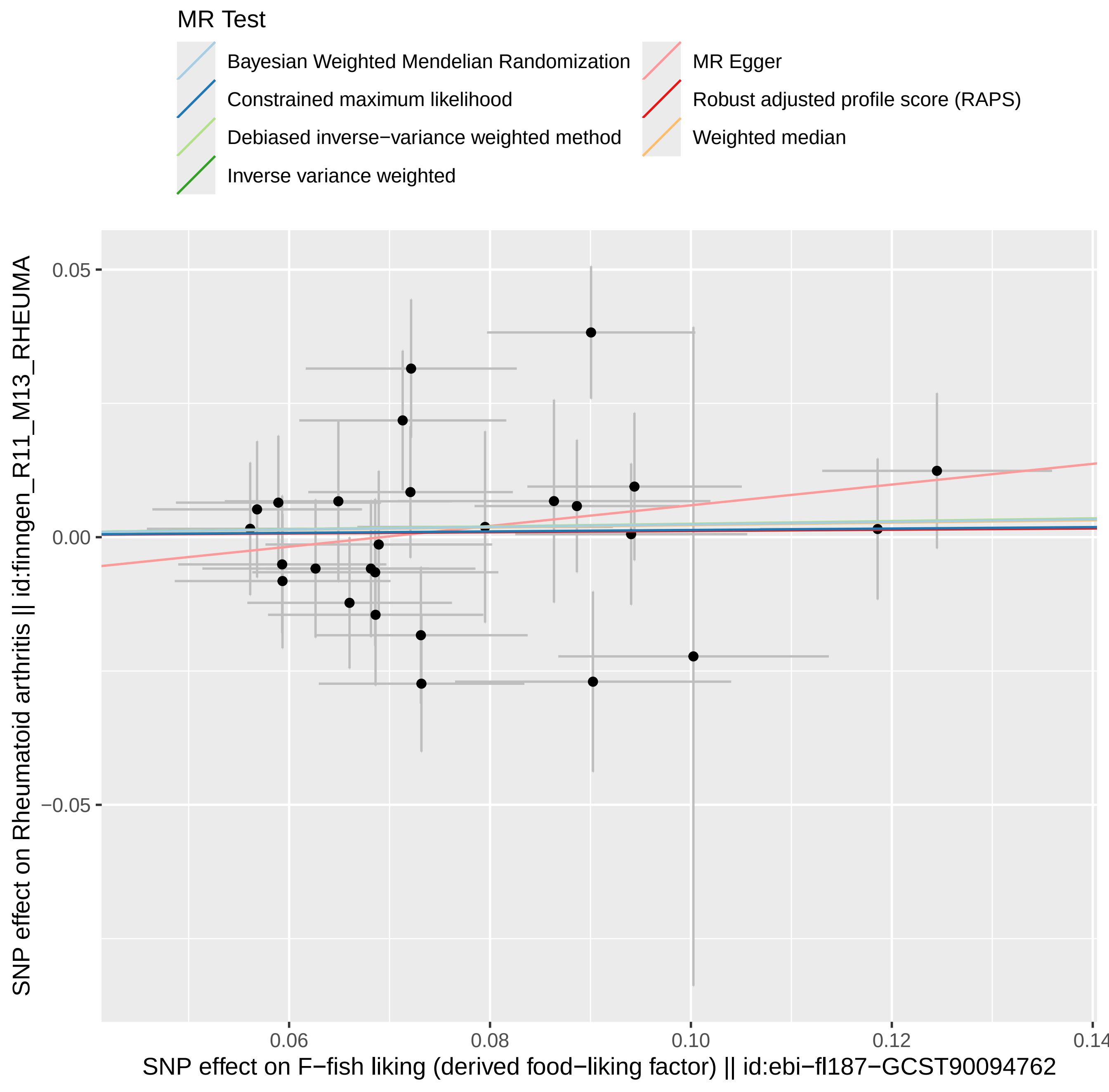
MR Test
Bayesian Weighted Mendelian Randomization
Constrained maximum likelihood
Debiased inverse−variance weighted method
Inverse variance weighted
MR Egger
Robust adjusted profile score (RAPS)
Weighted median
0.05
0.00
−0.05
0.06
0.08
0.10
0.12
0.14
SNP effect on Rheumatoid arthritis || id:finngen_R11_M13_RHEUMA
SNP effect on F−fish liking (derived food−liking factor) || id:ebi−fl187−GCST90094762

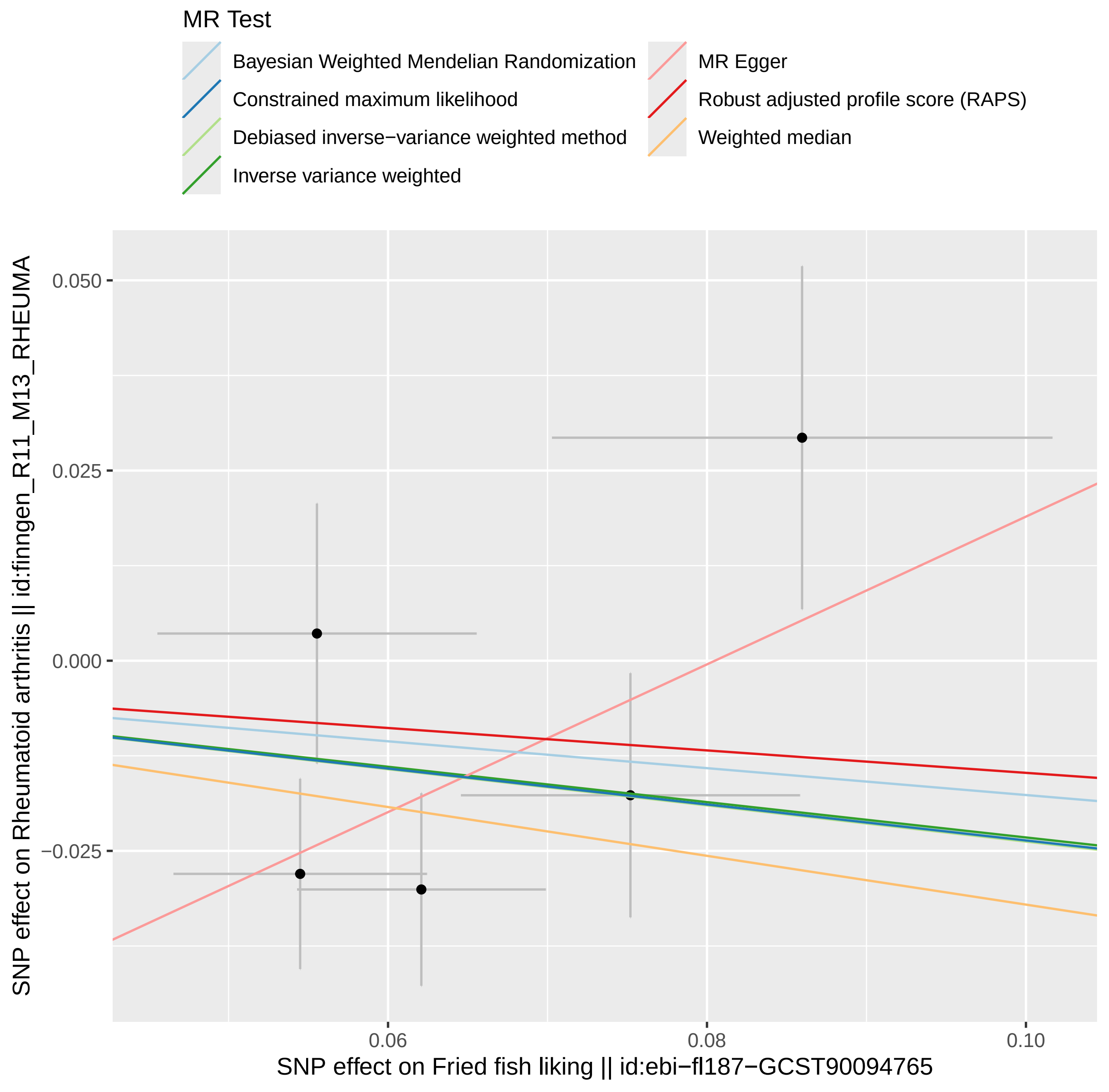
MR Test
Bayesian Weighted Mendelian Randomization
Constrained maximum likelihood
Debiased inverse−variance weighted method
Inverse variance weighted
MR Egger
Robust adjusted profile score (RAPS)
Weighted median
SNP effect on Rheumatoid arthritis || id:finngen_R11_M13_RHEUMA
0.050
0.025
0.000
−0.025
0.06
0.08
0.10
SNP effect on Fried fish liking || id:ebi−fl187−GCST90094765

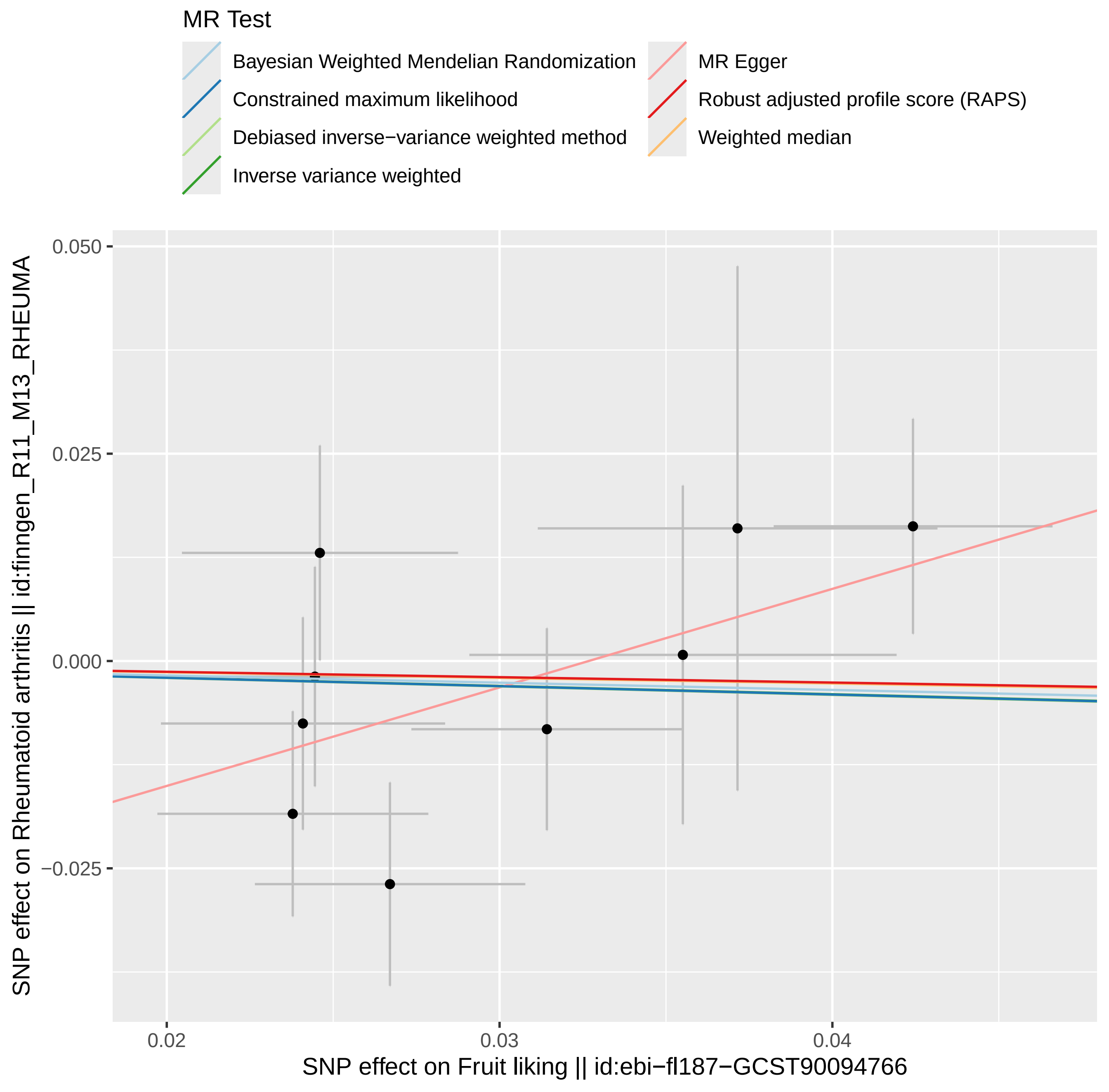

MR Test
Bayesian Weighted Mendelian Randomization
Constrained maximum likelihood
Debiased inverse−variance weighted method
Inverse variance weighted
MR Egger
Robust adjusted profile score (RAPS)
Weighted median
0.050
0.025
0.000
−0.025
0.02
0.03
0.04
SNP effect on Rheumatoid arthritis || id:finngen_R11_M13_RHEUMA
SNP effect on Fruit liking || id:ebi−fl187−GCST90094766

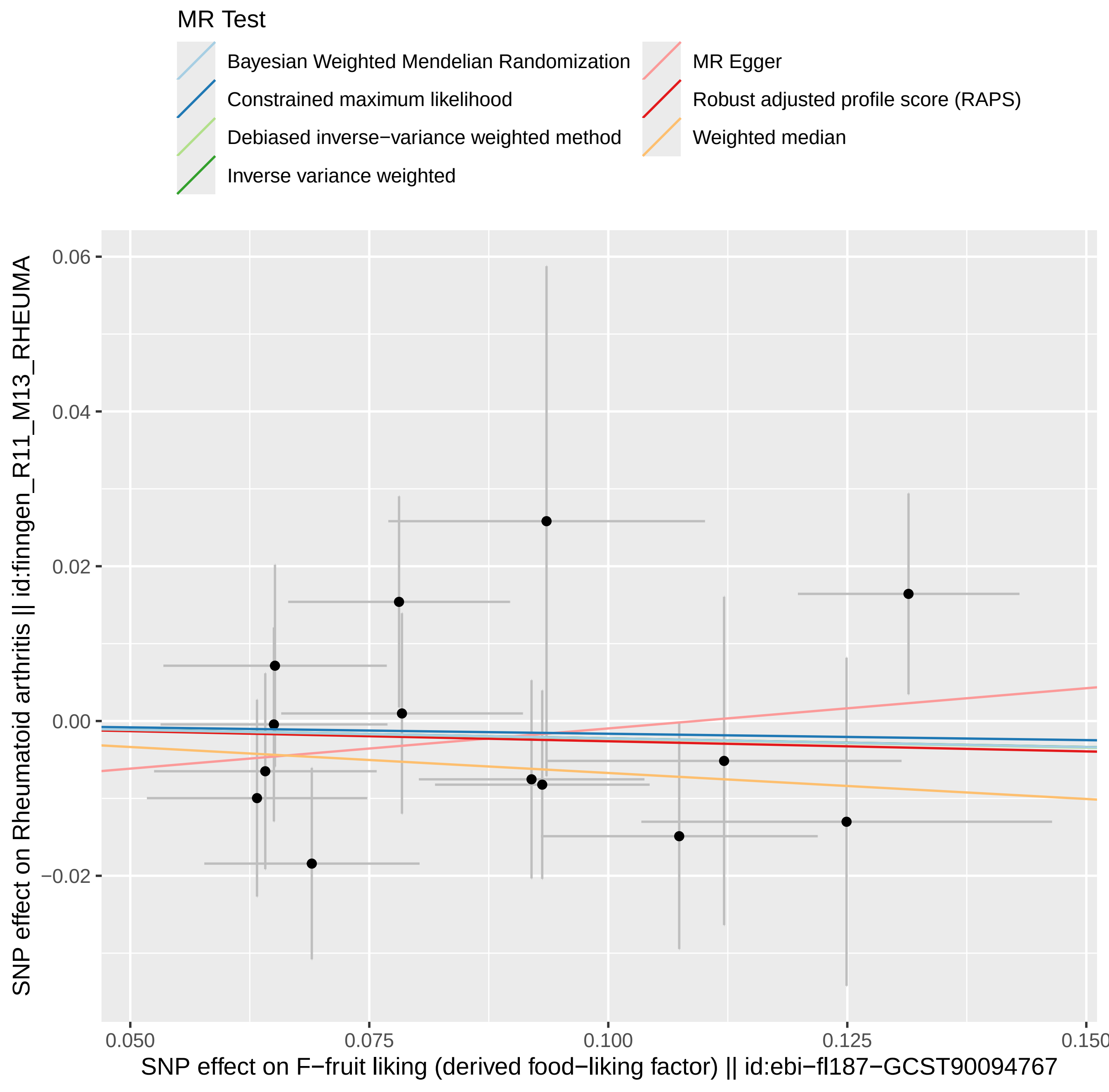

MR Test
Bayesian Weighted Mendelian Randomization
Constrained maximum likelihood
Debiased inverse−variance weighted method
Inverse variance weighted
MR Egger
Robust adjusted profile score (RAPS)
Weighted median
0.06
0.04
0.02
0.00
−0.02
0.050
0.075
0.100
0.125
0.150
SNP effect on Rheumatoid arthritis || id:finngen_R11_M13_RHEUMA
SNP effect on F−fruit liking (derived food−liking factor) || id:ebi−fl187−GCST90094767

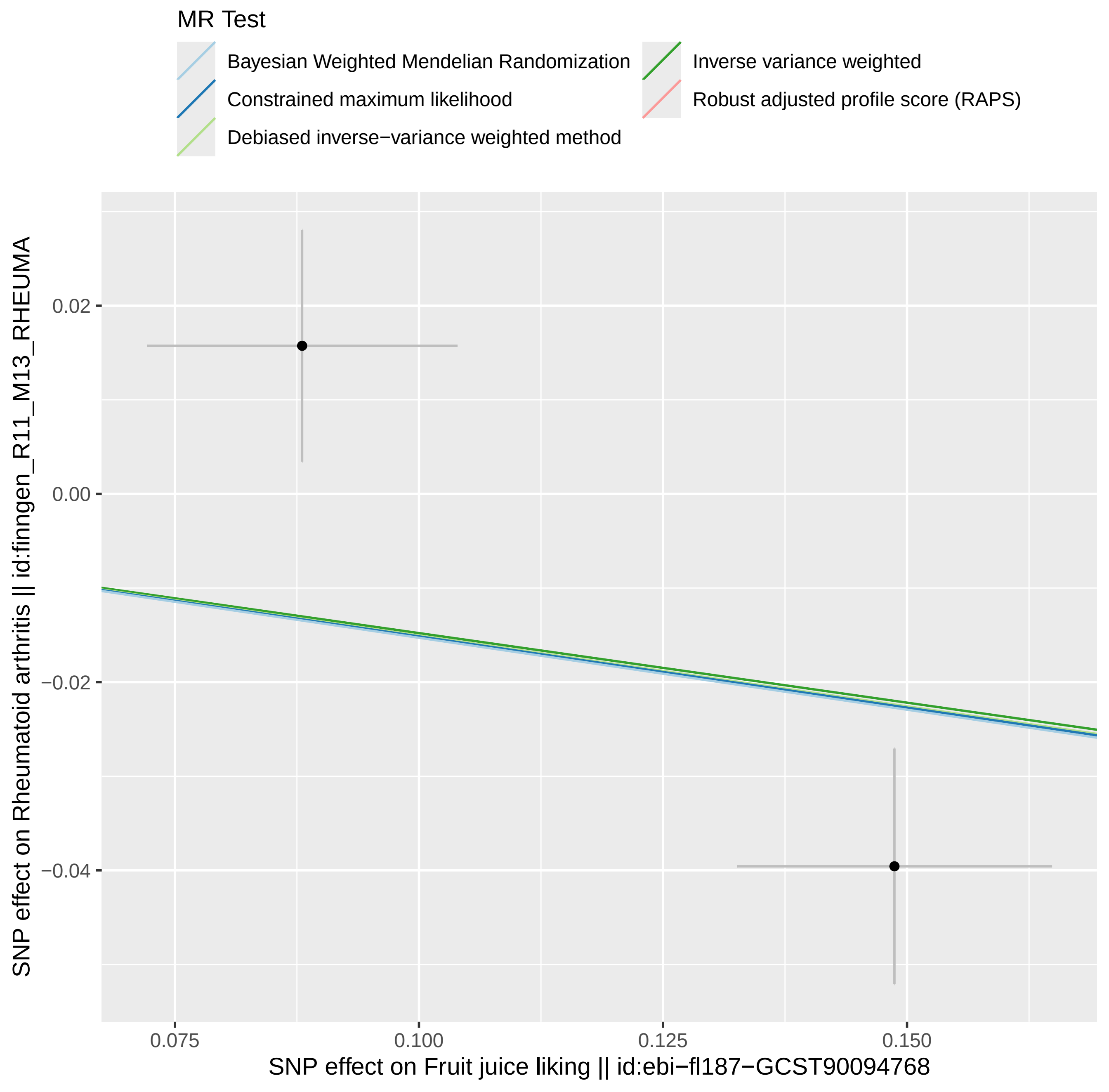
MR Test
Bayesian Weighted Mendelian Randomization
Constrained maximum likelihood
Debiased inverse−variance weighted method
Inverse variance weighted
Robust adjusted profile score (RAPS)
0.02
0.00
−0.02
−0.04
0.075
0.100
0.125
0.150
SNP effect on Fruit juice liking || id:ebi−fl187−GCST90094768
SNP effect on Rheumatoid arthritis || id:finngen_R11_M13_RHEUMA

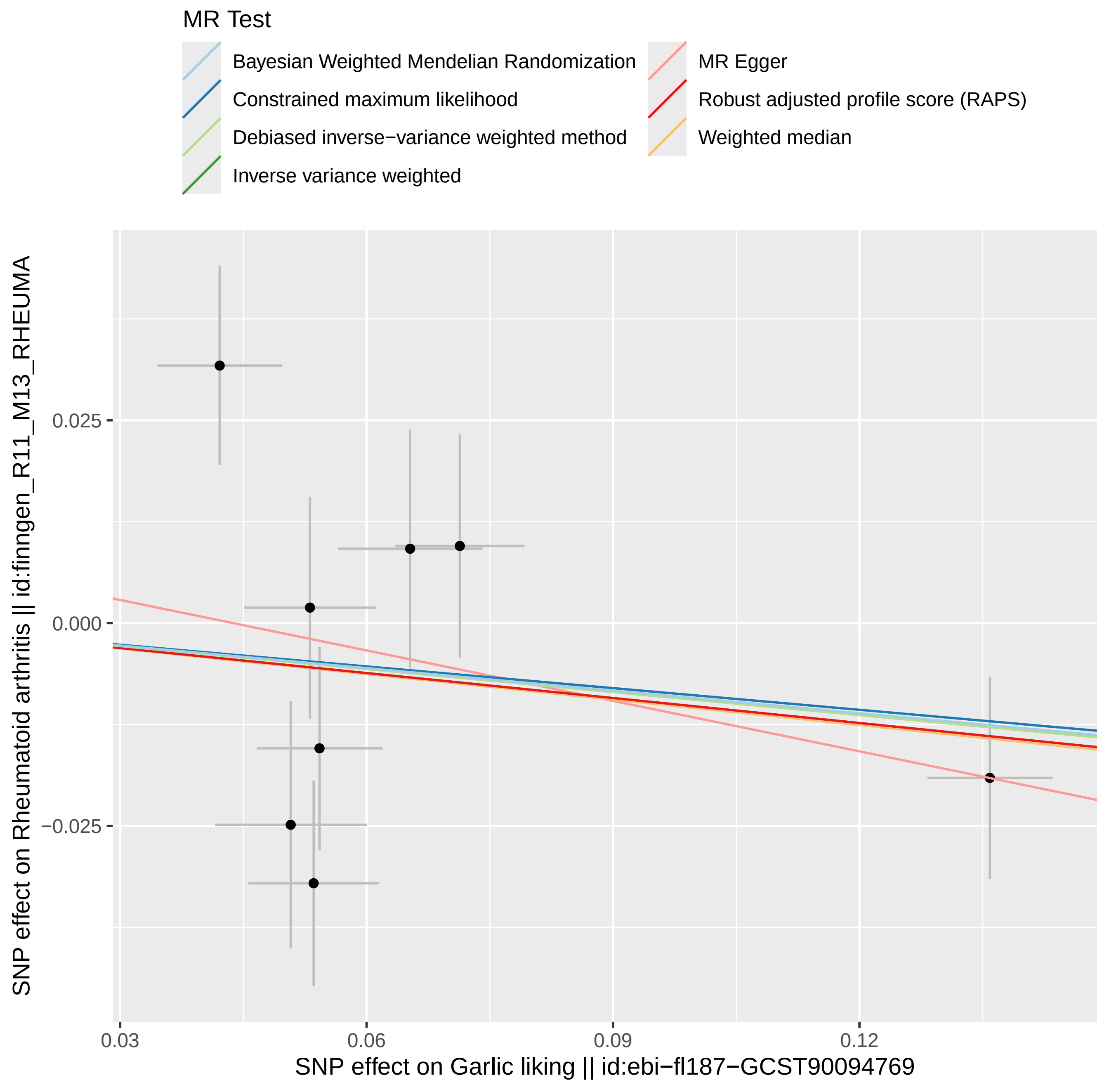
MR Test
Bayesian Weighted Mendelian Randomization
Constrained maximum likelihood
Debiased inverse−variance weighted method
Inverse variance weighted
MR Egger
Robust adjusted profile score (RAPS)
Weighted median
SNP effect on Rheumatoid arthritis || id:finngen_R11_M13_RHEUMA
0.025
0.000
−0.025
0.03
0.06
0.09
0.12
SNP effect on Garlic liking || id:ebi−fI187−GCST90094769

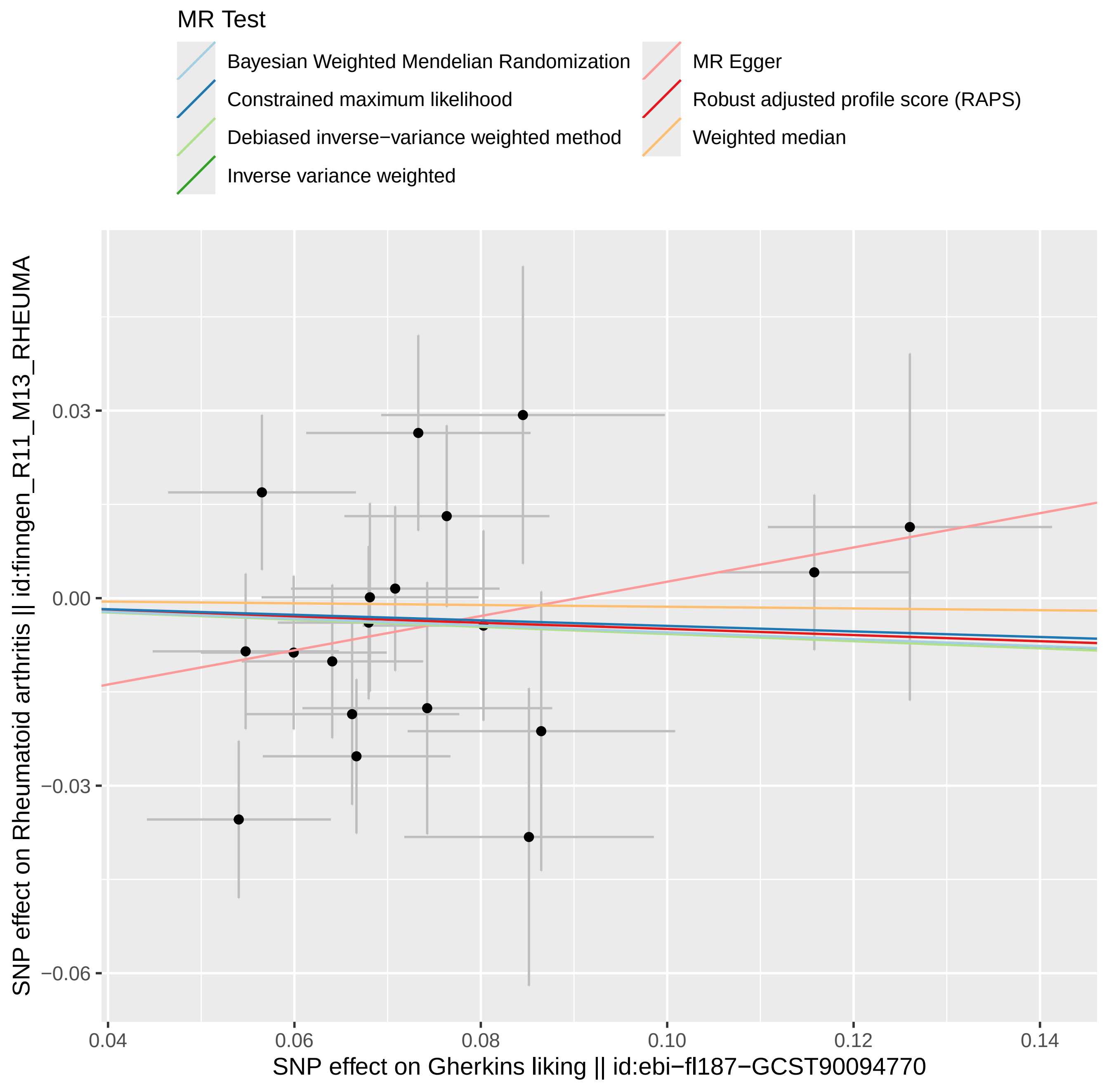

MR Test
Bayesian Weighted Mendelian Randomization
Constrained maximum likelihood
Debiased inverse−variance weighted method
Inverse variance weighted
MR Egger
Robust adjusted profile score (RAPS)
Weighted median
SNP effect on Rheumatoid arthritis || id:finngen_R11_M13_RHEUMA
0.03
0.00
−0.03
−0.06
0.04
0.06
0.08
0.10
0.12
0.14
SNP effect on Gherkins liking || id:ebi−fl187−GCST90094770

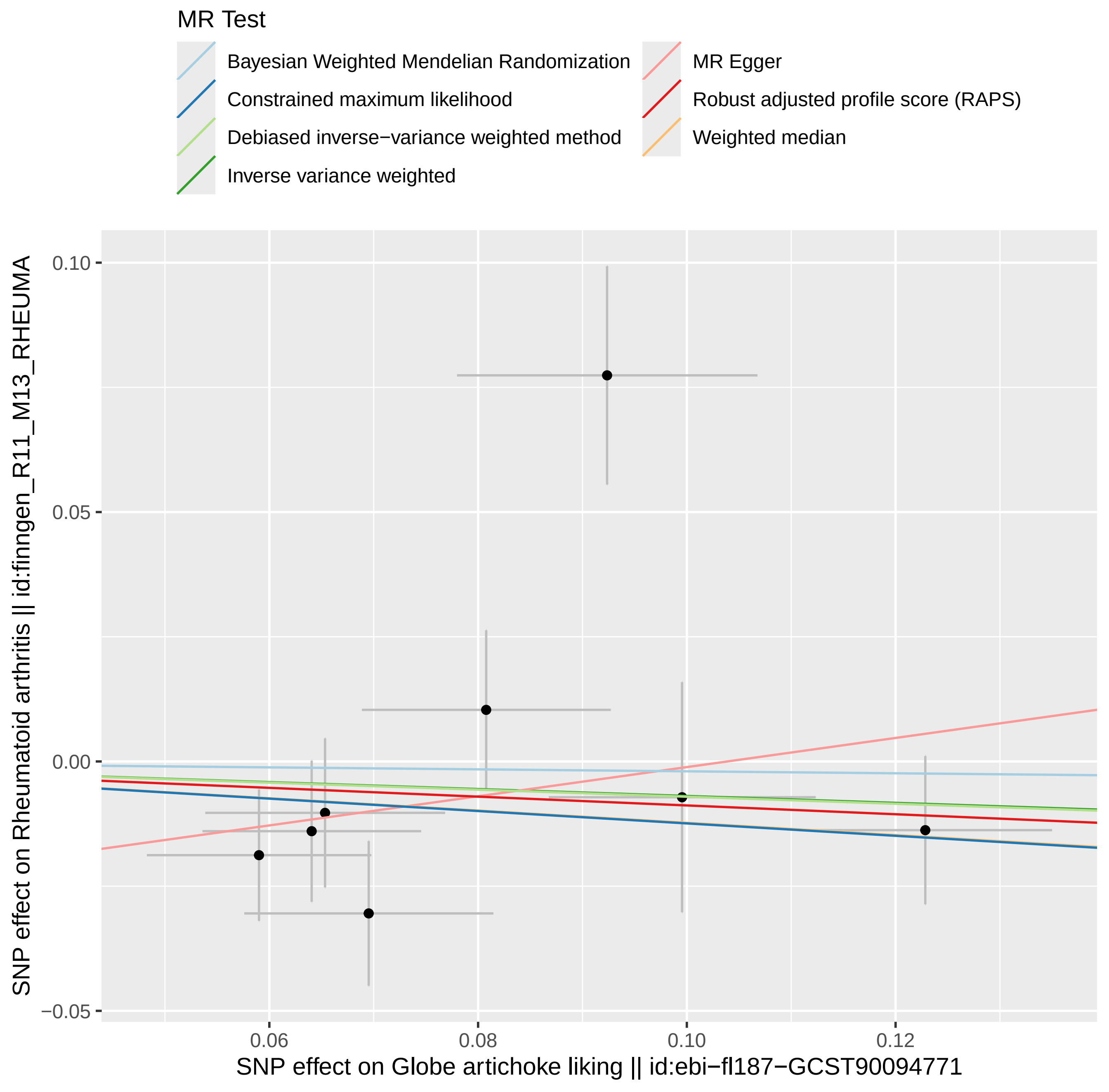
MR Test
Bayesian Weighted Mendelian Randomization
Constrained maximum likelihood
Debiased inverse−variance weighted method
Inverse variance weighted
MR Egger
Robust adjusted profile score (RAPS)
Weighted median
SNP effect on Rheumatoid arthritis || id:finngen_R11_M13_RHEUMA
0.10
0.05
0.00
−0.05
0.06
0.08
0.10
0.12
SNP effect on Globe artichoke liking || id:ebi−fl187−GCST90094771

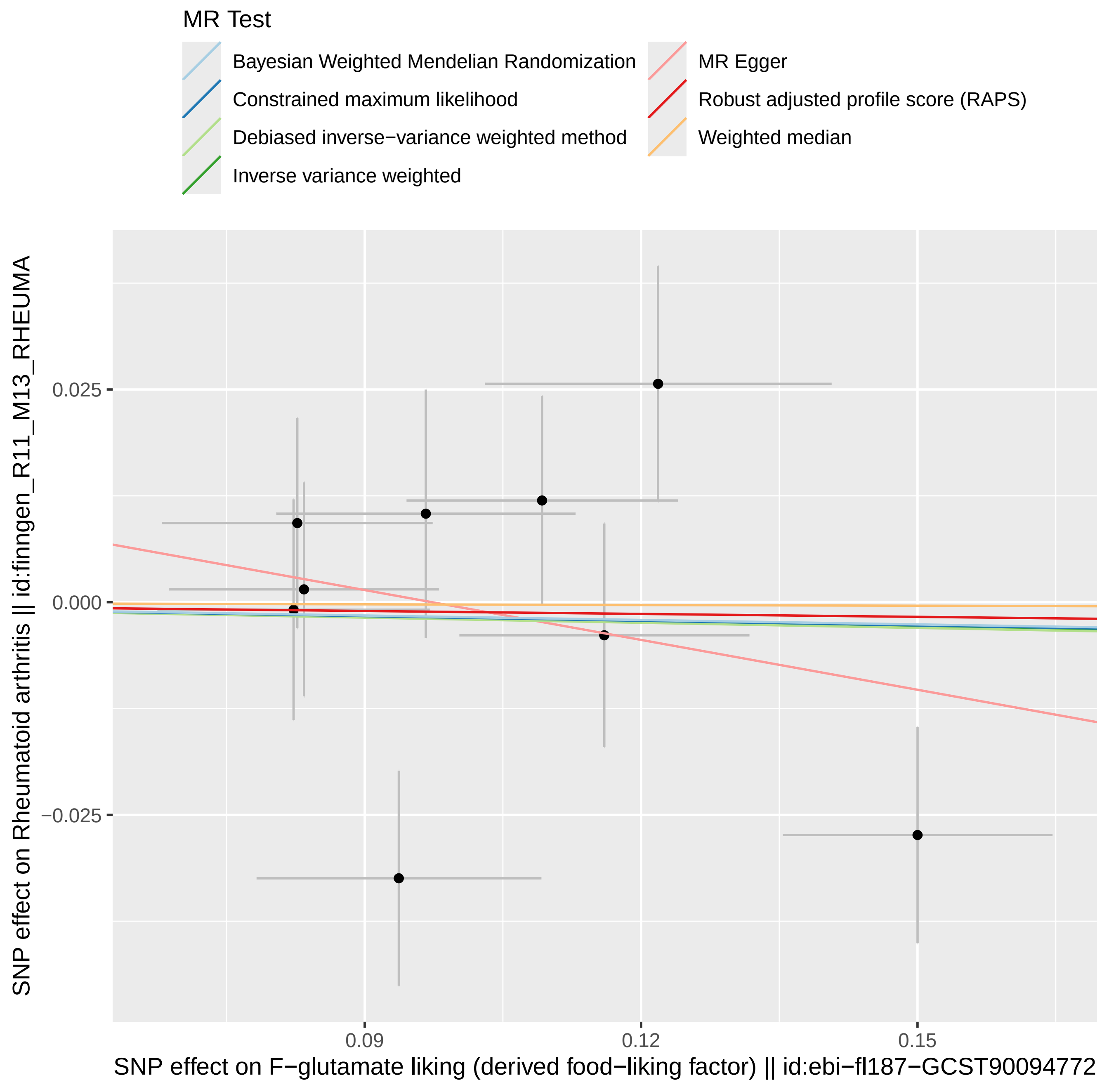

MR Test
Bayesian Weighted Mendelian Randomization
Constrained maximum likelihood
Debiased inverse−variance weighted method
Inverse variance weighted
MR Egger
Robust adjusted profile score (RAPS)
Weighted median
0.025
0.000
−0.025
0.09
0.12
0.15
SNP effect on Rheumatoid arthritis || id:finngen_R11_M13_RHEUMA
SNP effect on F−glutamate liking (derived food−liking factor) || id:ebi−fl187−GCST90094772

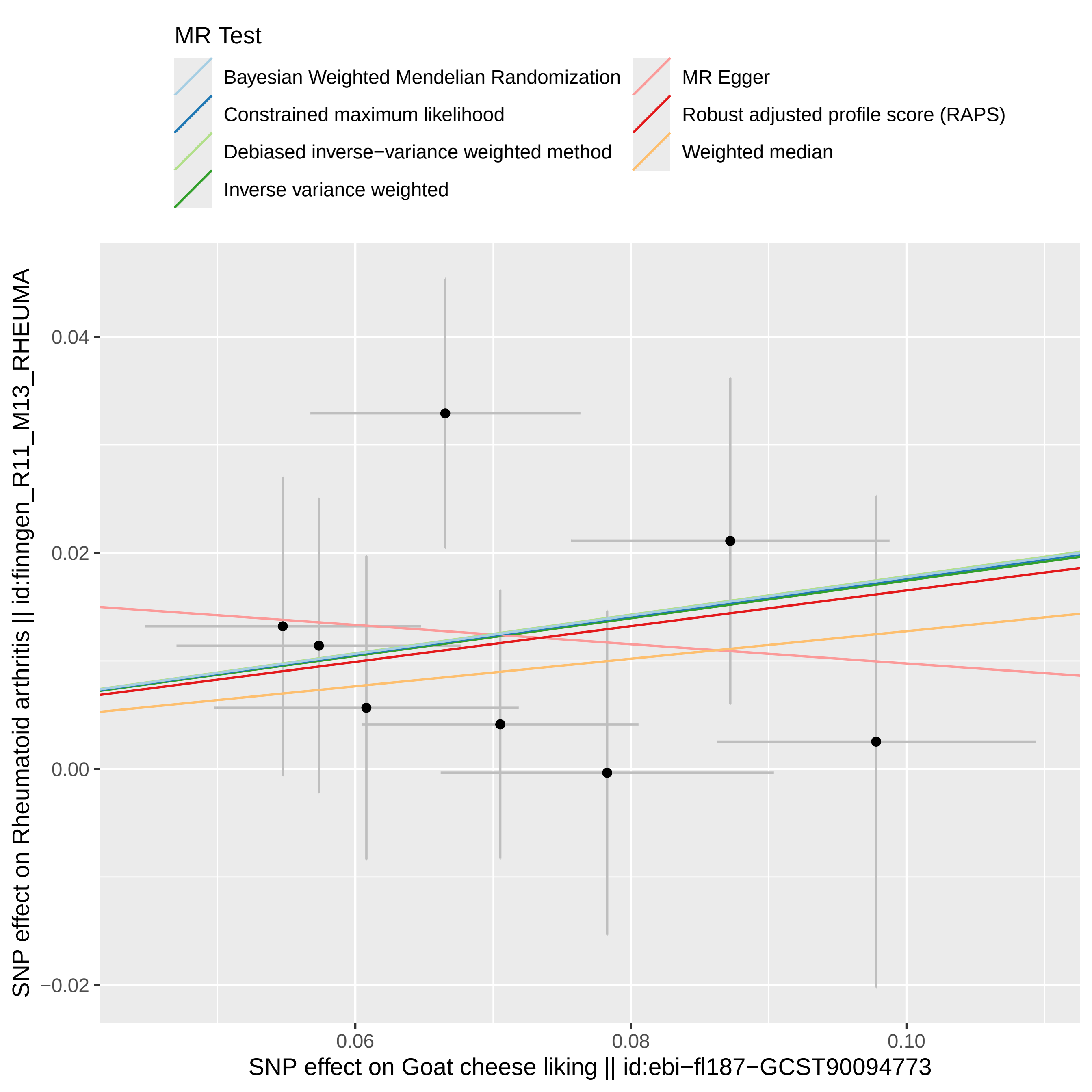

MR Test
Bayesian Weighted Mendelian Randomization
Constrained maximum likelihood
Debiased inverse−variance weighted method
Inverse variance weighted
MR Egger
Robust adjusted profile score (RAPS)
Weighted median
0.04
0.02
0.00
−0.02
0.06
0.08
0.10
SNP effect on Rheumatoid arthritis || id:finngen_R11_M13_RHEUMA
SNP effect on Goat cheese liking || id:ebi−fl187−GCST90094773

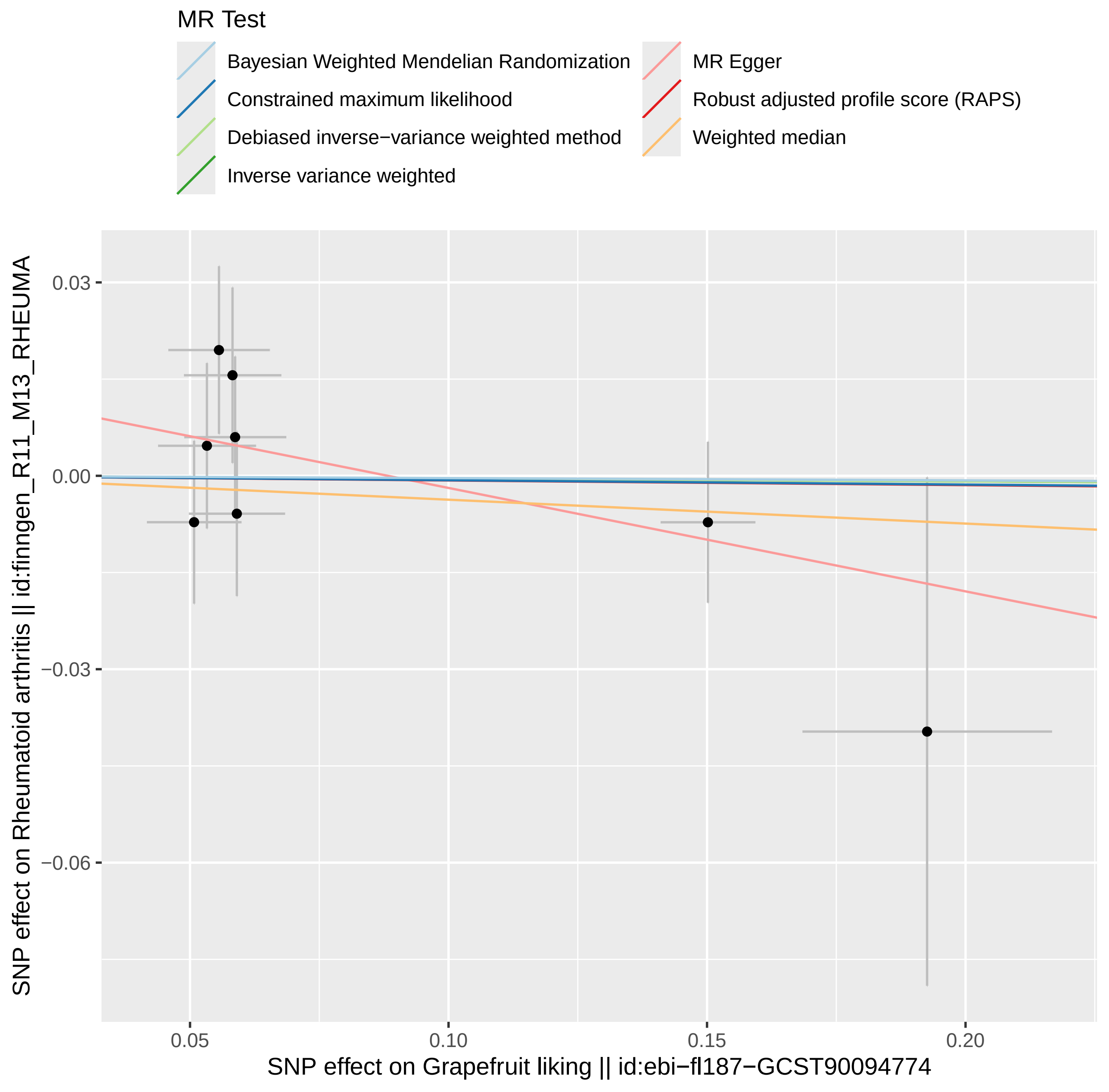

MR Test
Bayesian Weighted Mendelian Randomization
Constrained maximum likelihood
Debiased inverse−variance weighted method
Inverse variance weighted
MR Egger
Robust adjusted profile score (RAPS)
Weighted median
0.03
0.00
−0.03
−0.06
0.05
0.10
0.15
0.20
SNP effect on Grapefruit liking || id:ebi−fl187−GCST90094774
SNP effect on Rheumatoid arthritis || id:finngen_R11_M13_RHEUMA

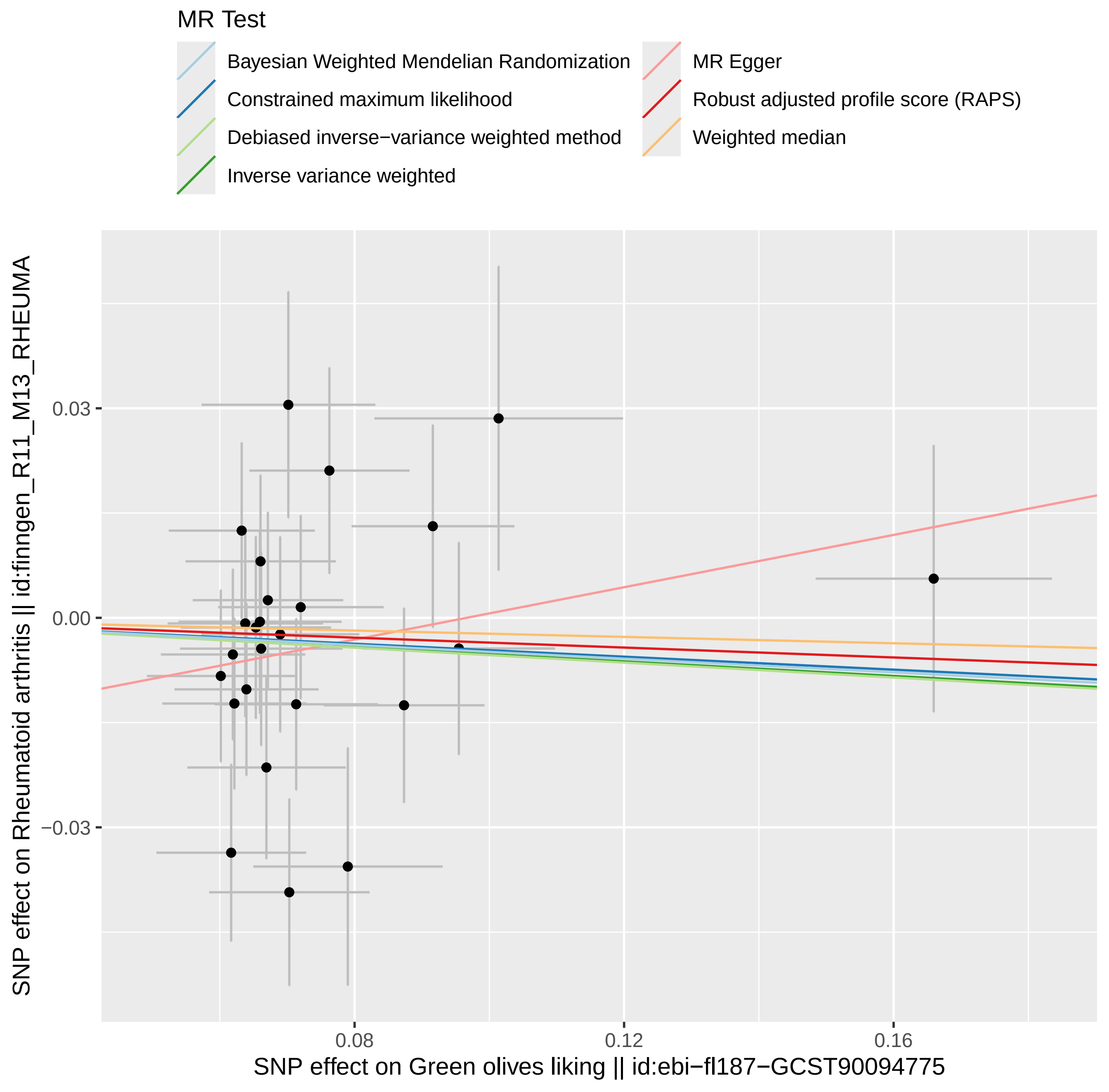

MR Test
Bayesian Weighted Mendelian Randomization
Constrained maximum likelihood
Debiased inverse−variance weighted method
Inverse variance weighted
MR Egger
Robust adjusted profile score (RAPS)
Weighted median
0.03
0.00
−0.03
0.08
0.12
0.16
SNP effect on Rheumatoid arthritis || id:finngen_R11_M13_RHEUMA
SNP effect on Green olives liking || id:ebi−fl187−GCST90094775

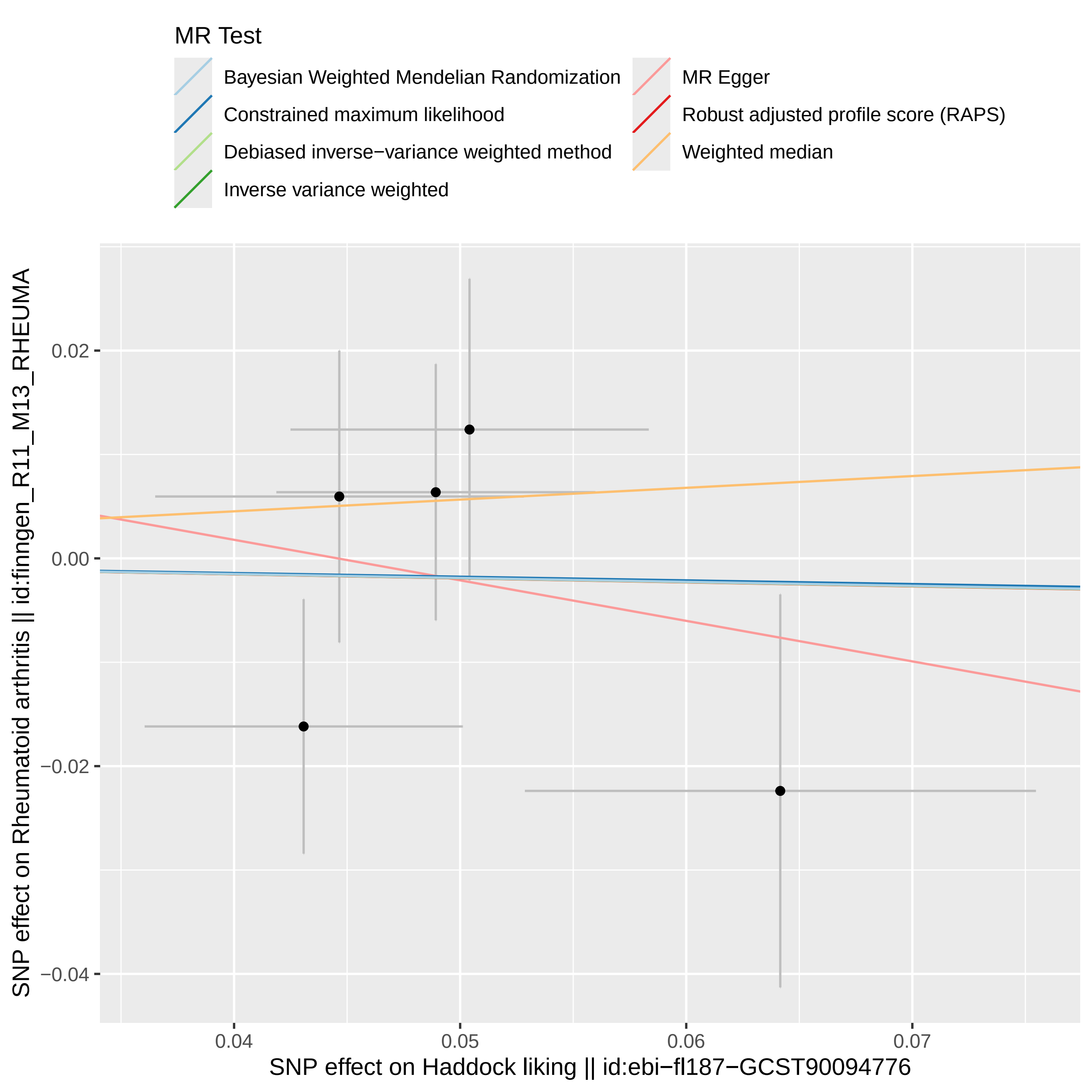

MR Test
Bayesian Weighted Mendelian Randomization
Constrained maximum likelihood
Debiased inverse−variance weighted method
Inverse variance weighted
MR Egger
Robust adjusted profile score (RAPS)
Weighted median
0.02
0.00
−0.02
−0.04
0.04
0.05
0.06
0.07
SNP effect on Haddock liking || id:ebi−fl187−GCST90094776
SNP effect on Rheumatoid arthritis || id:finngen_R11_M13_RHEUMA

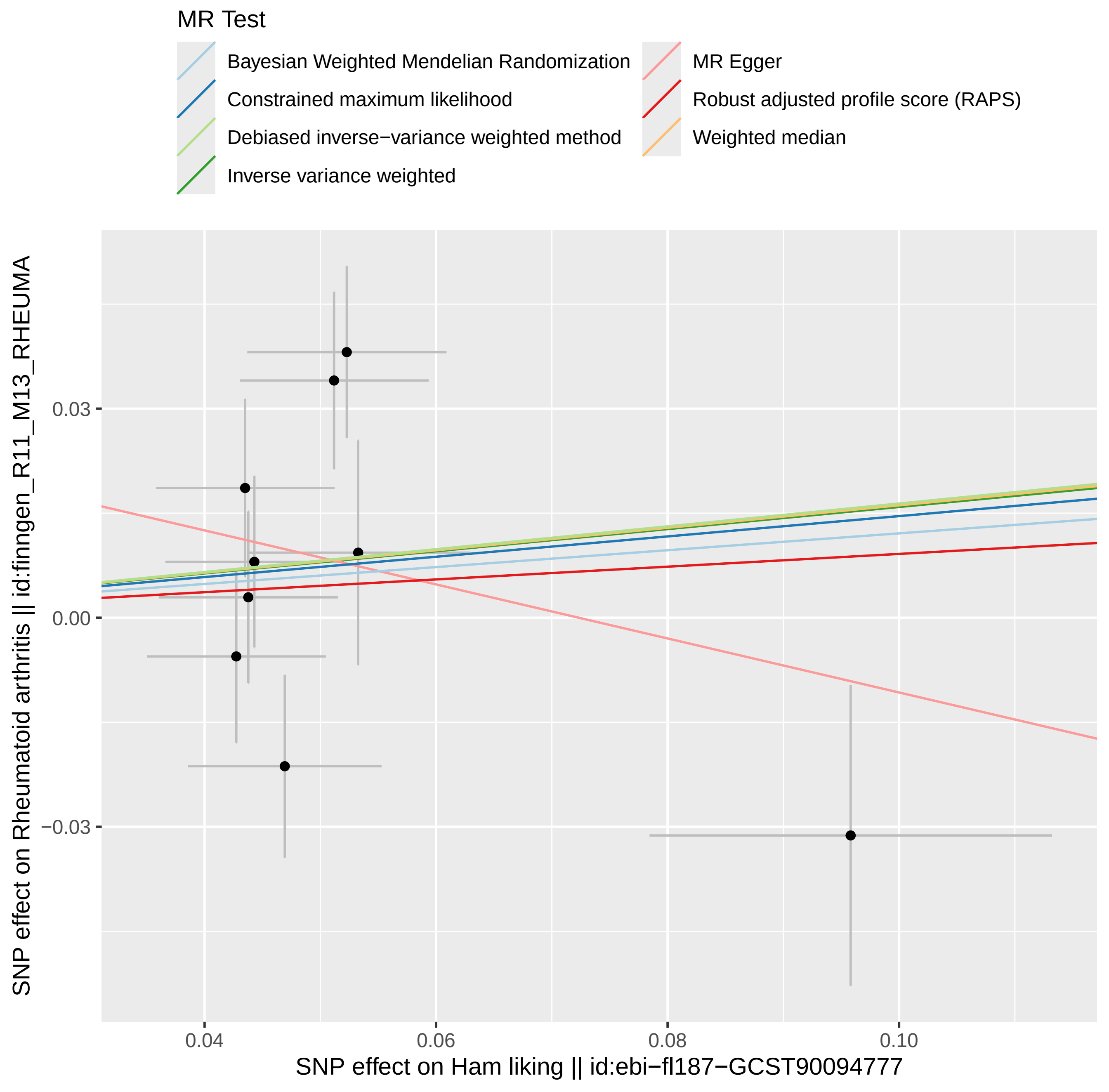
MR Test
Bayesian Weighted Mendelian Randomization
Constrained maximum likelihood
Debiased inverse−variance weighted method
Inverse variance weighted
MR Egger
Robust adjusted profile score (RAPS)
Weighted median
0.03
0.00
−0.03
0.04
0.06
0.08
0.10
SNP effect on Rheumatoid arthritis || id:finngen_R11_M13_RHEUMA
SNP effect on Ham liking || id:ebi−fl187−GCST90094777

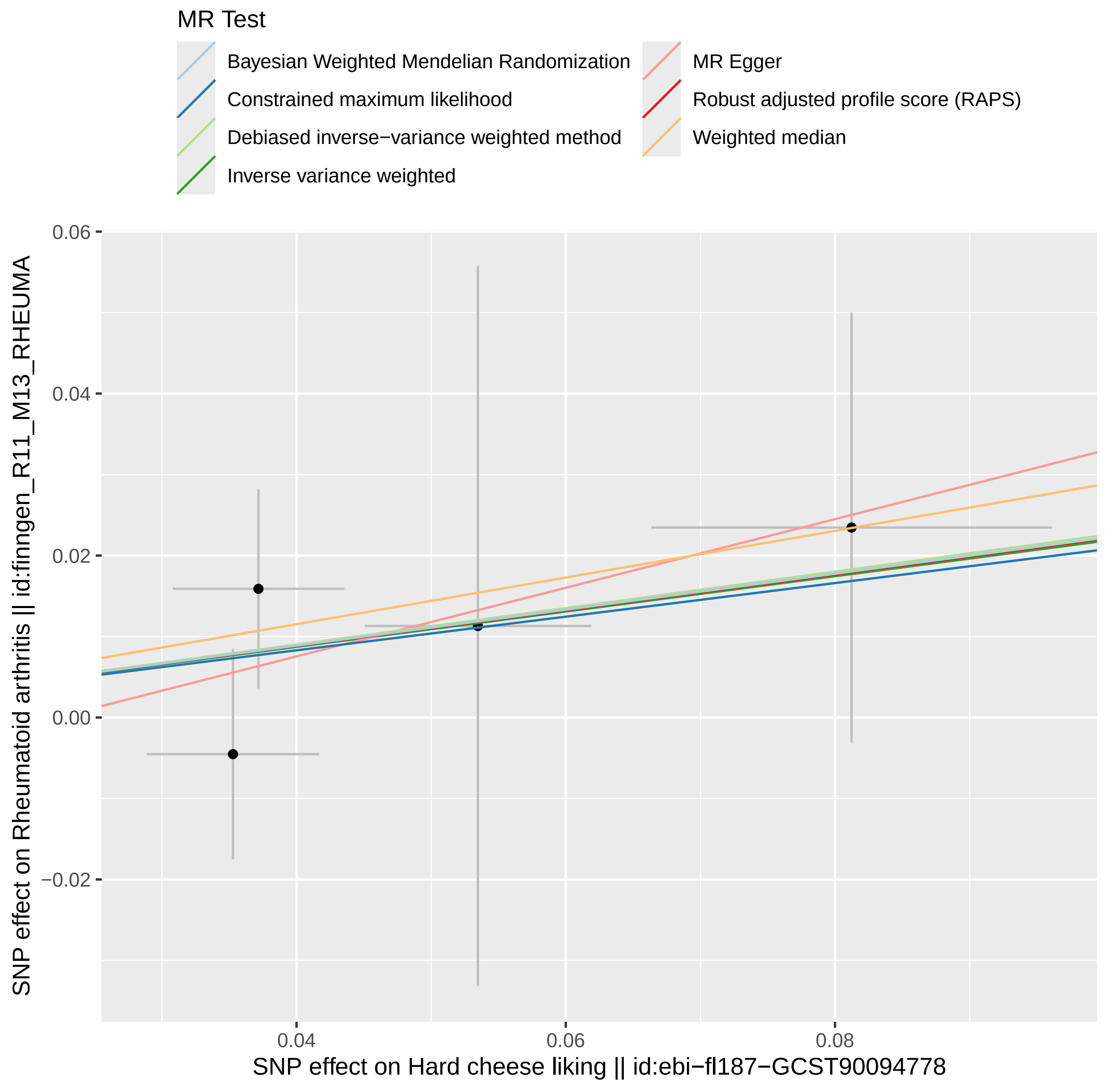
MR Test
Bayesian Weighted Mendelian Randomization
Constrained maximum likelihood
Debiased inverse−variance weighted method
Inverse variance weighted
MR Egger
Robust adjusted profile score (RAPS)
Weighted median
0.06
0.04
0.02
0.00
−0.02
0.04
0.06
0.08
SNP effect on Rheumatoid arthritis || id:finngen_R11_M13_RHEUMA
SNP effect on Hard cheese liking || id:ebi−fl187−GCST90094778

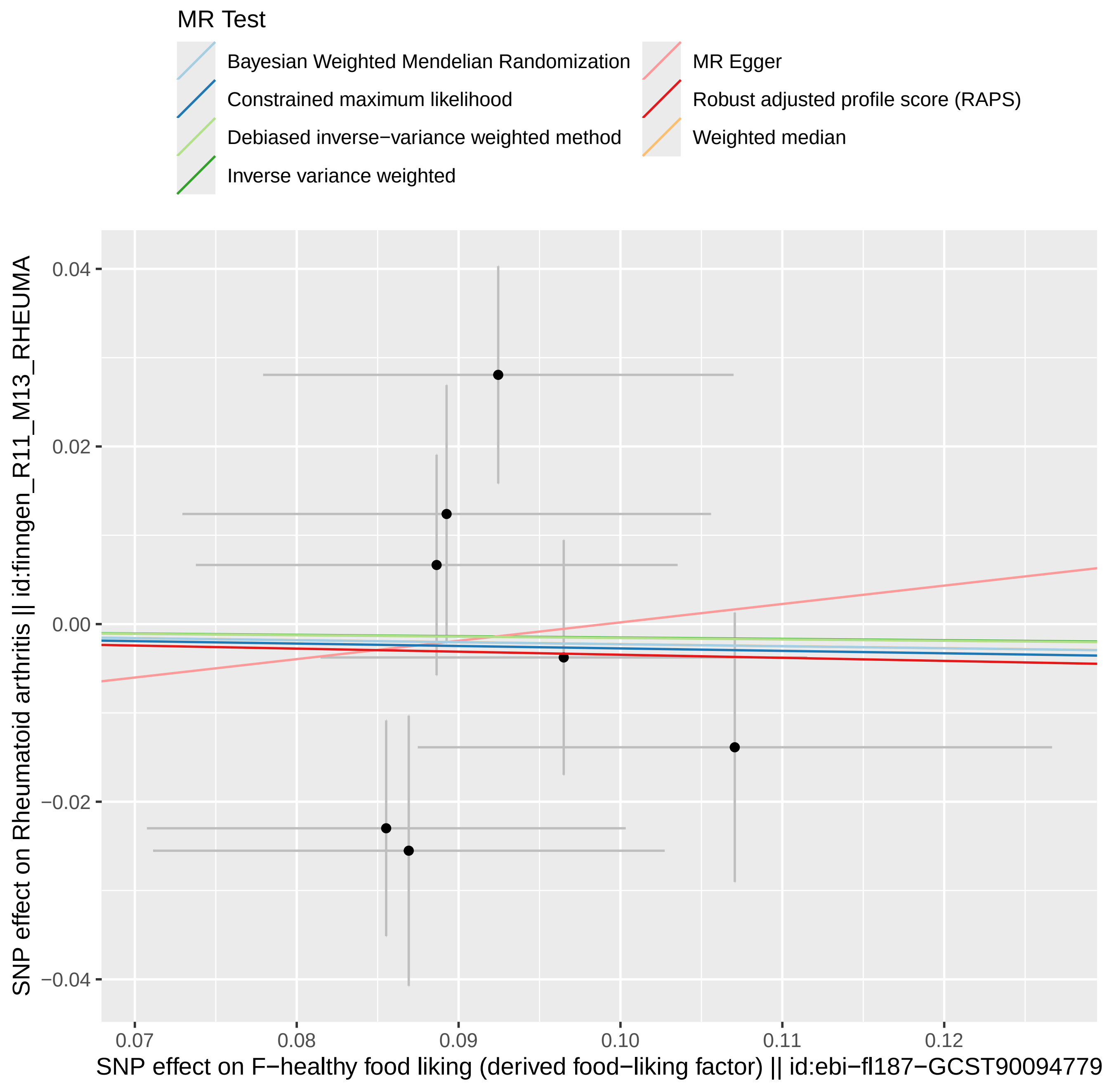
MR Test
Bayesian Weighted Mendelian Randomization
Constrained maximum likelihood
Debiased inverse−variance weighted method
Inverse variance weighted
MR Egger
Robust adjusted profile score (RAPS)
Weighted median
0.04
0.02
0.00
−0.02
−0.04
0.07
0.08
0.09
0.10
0.11
0.12
SNP effect on Rheumatoid arthritis || id:finngen_R11_M13_RHEUMA
SNP effect on F−healthy food liking (derived food−liking factor) || id:ebi−fl187−GCST90094779

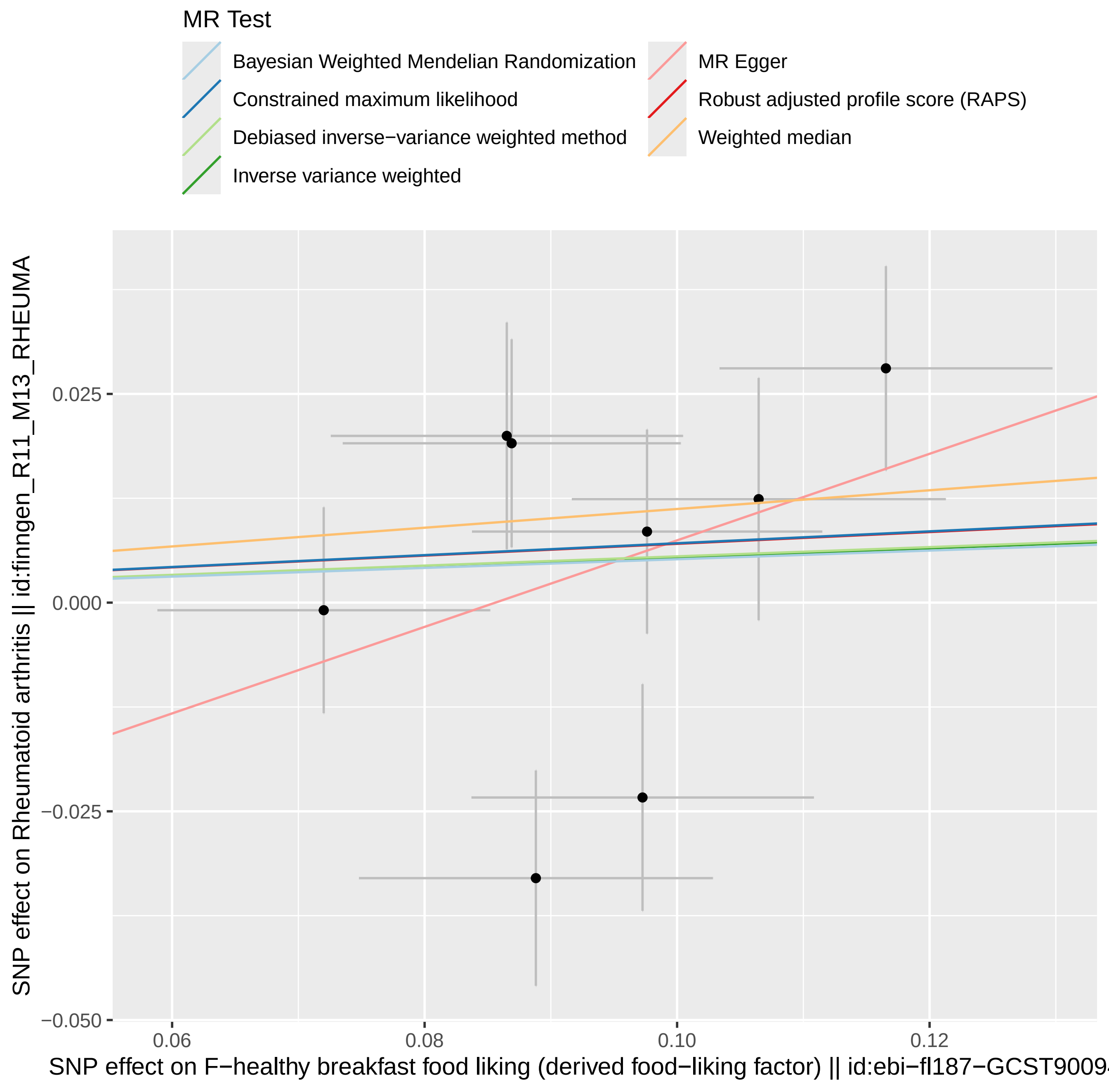

MR Test
Bayesian Weighted Mendelian Randomization
Constrained maximum likelihood
Debiased inverse−variance weighted method
Inverse variance weighted
MR Egger
Robust adjusted profile score (RAPS)
Weighted median
SNP effect on Rheumatoid arthritis || id:finngen_R11_M13_RHEUMA
0.025
0.000
−0.025
−0.050
0.06
0.08
0.10
0.12
SNP effect on F−healthy breakfast food liking (derived food−liking factor) || id:ebi−fl187−GCST9009

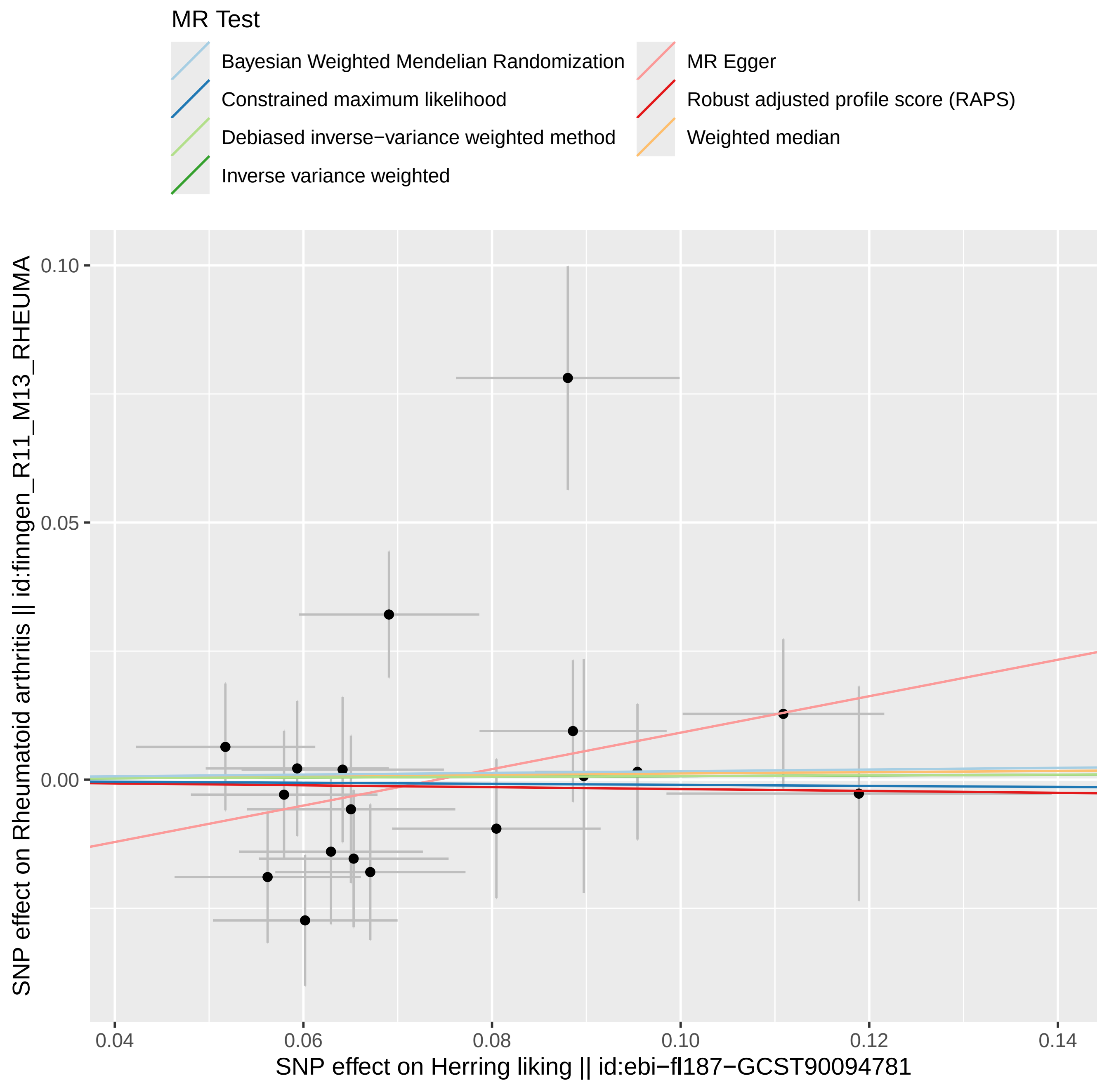

MR Test
Bayesian Weighted Mendelian Randomization
Constrained maximum likelihood
Debiased inverse−variance weighted method
Inverse variance weighted
MR Egger
Robust adjusted profile score (RAPS)
Weighted median
0.10
0.05
0.00
0.04
0.06
0.08
0.10
0.12
0.14
SNP effect on Rheumatoid arthritis || id:finngen_R11_M13_RHEUMA
SNP effect on Herring liking || id:ebi−fl187−GCST90094781

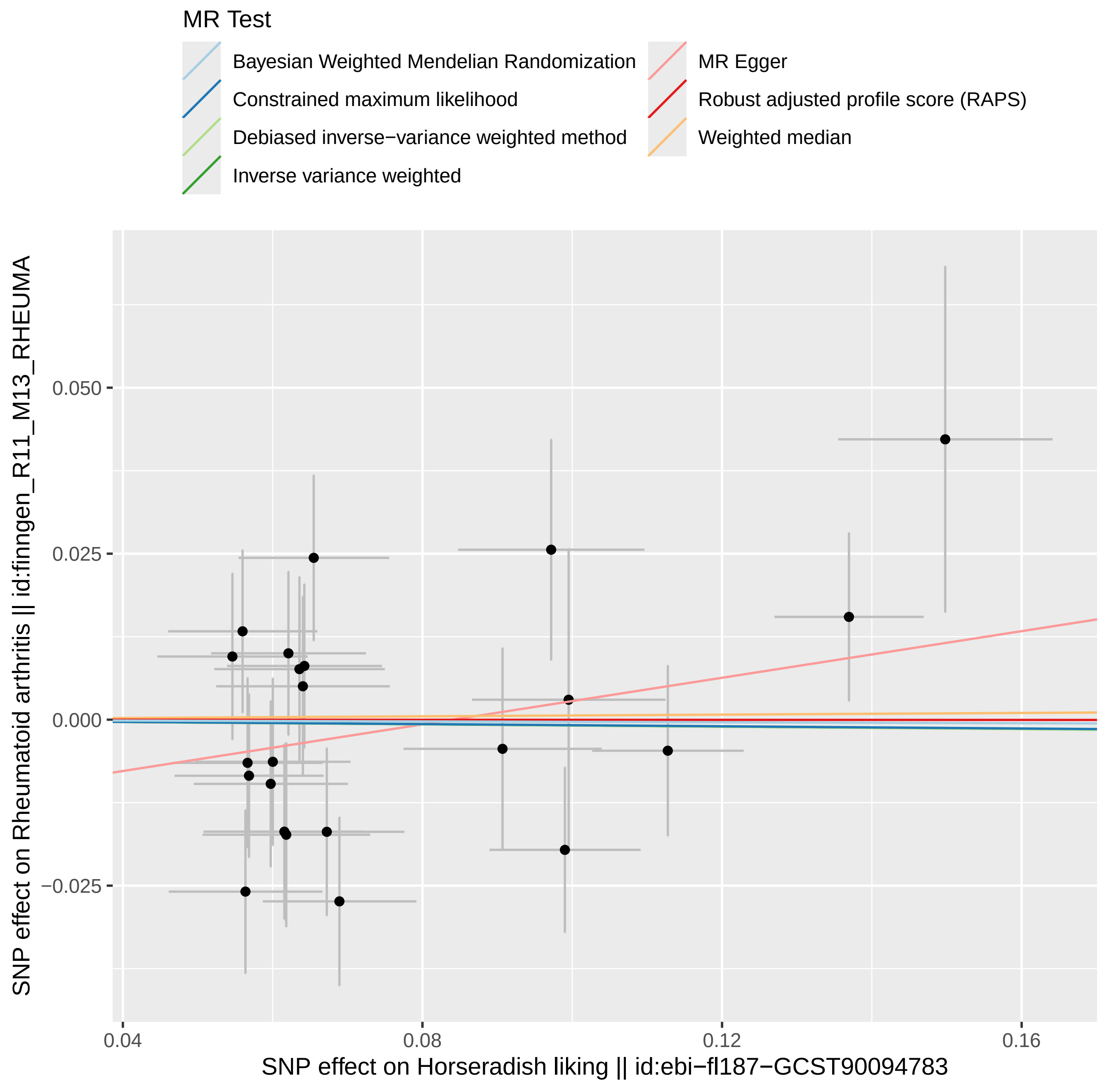
MR Test
Bayesian Weighted Mendelian Randomization
Constrained maximum likelihood
Debiased inverse−variance weighted method
Inverse variance weighted
MR Egger
Robust adjusted profile score (RAPS)
Weighted median
SNP effect on Rheumatoid arthritis || id:finngen_R11_M13_RHEUMA
0.050
0.025
0.000
−0.025
0.04
0.08
0.12
0.16
SNP effect on Horseradish liking || id:ebi−fl187−GCST90094783

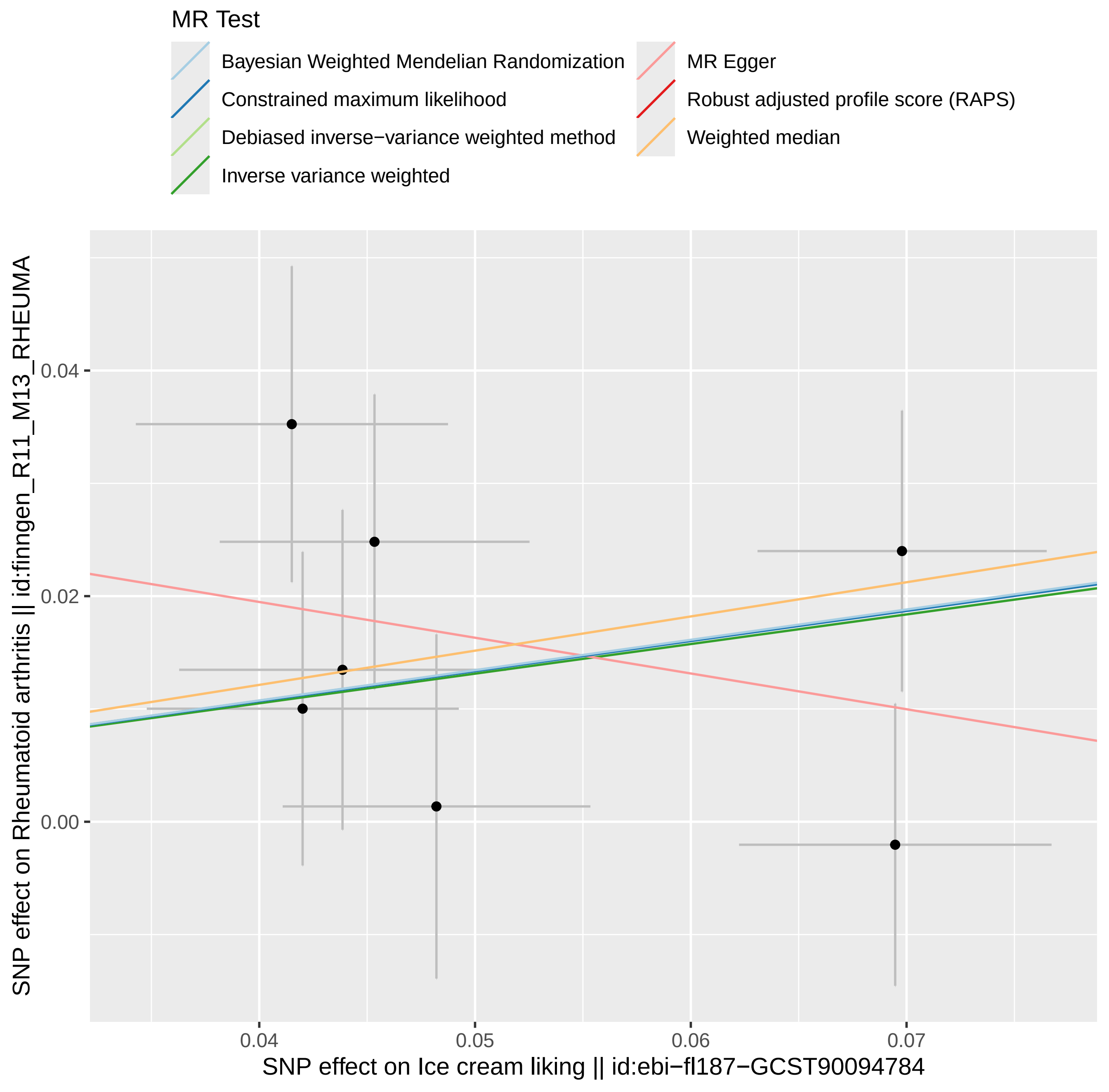

MR Test
Bayesian Weighted Mendelian Randomization
Constrained maximum likelihood
Debiased inverse−variance weighted method
Inverse variance weighted
MR Egger
Robust adjusted profile score (RAPS)
Weighted median
SNP effect on Rheumatoid arthritis || id:finngen_R11_M13_RHEUMA
0.04
0.02
0.00
0.04
0.05
0.06
0.07
SNP effect on Ice cream liking || id:ebi−fl187−GCST90094784

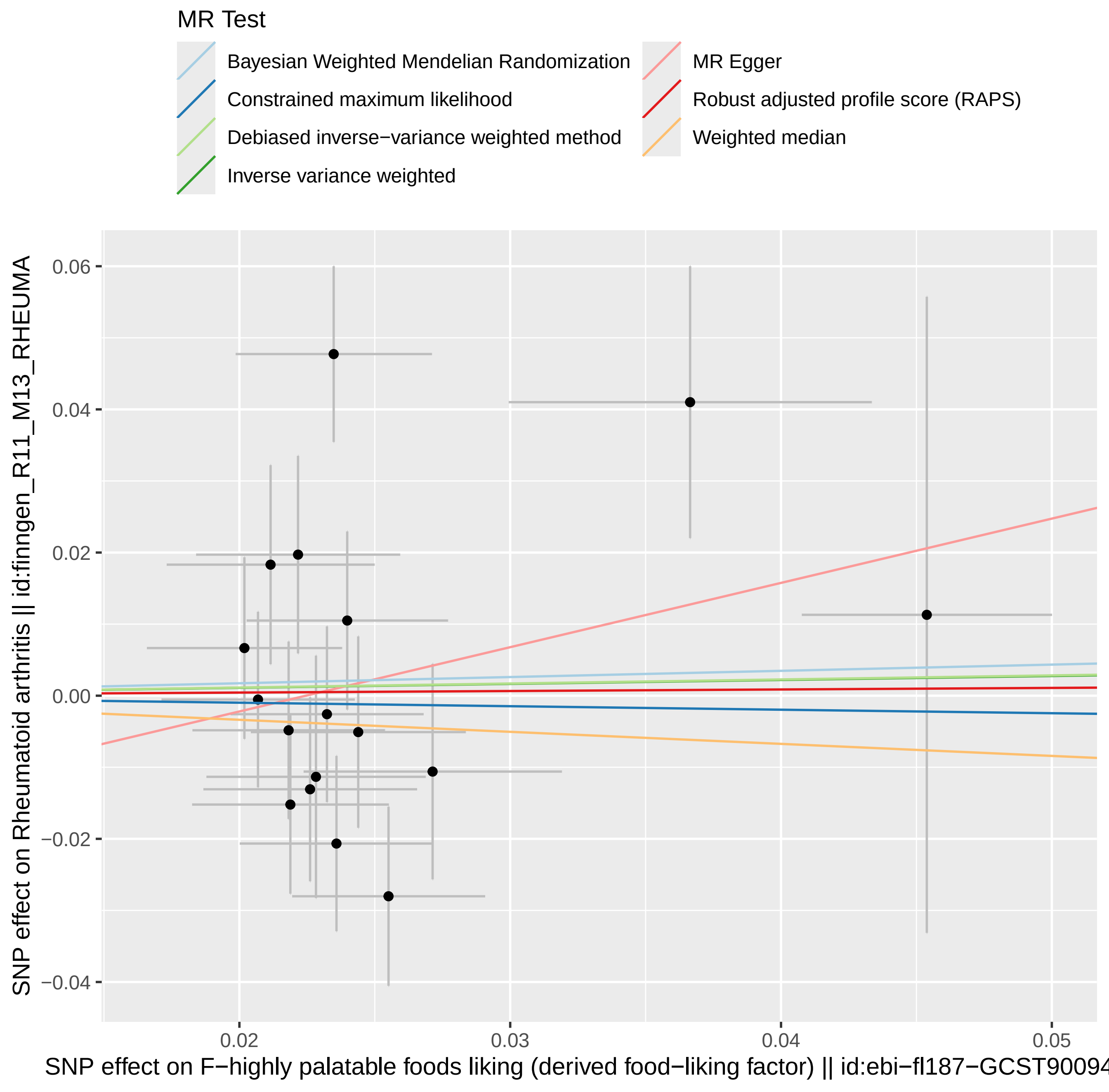

MR Test
Bayesian Weighted Mendelian Randomization
Constrained maximum likelihood
Debiased inverse−variance weighted method
Inverse variance weighted
MR Egger
Robust adjusted profile score (RAPS)
Weighted median
0.06
0.04
0.02
0.00
−0.02
−0.04
0.02
0.03
0.04
0.05
SNP effect on Rheumatoid arthritis || id:finngen_R11_M13_RHEUMA
SNP effect on F−highly palatable foods liking (derived food−liking factor) || id:ebi−fl187−GCST90094

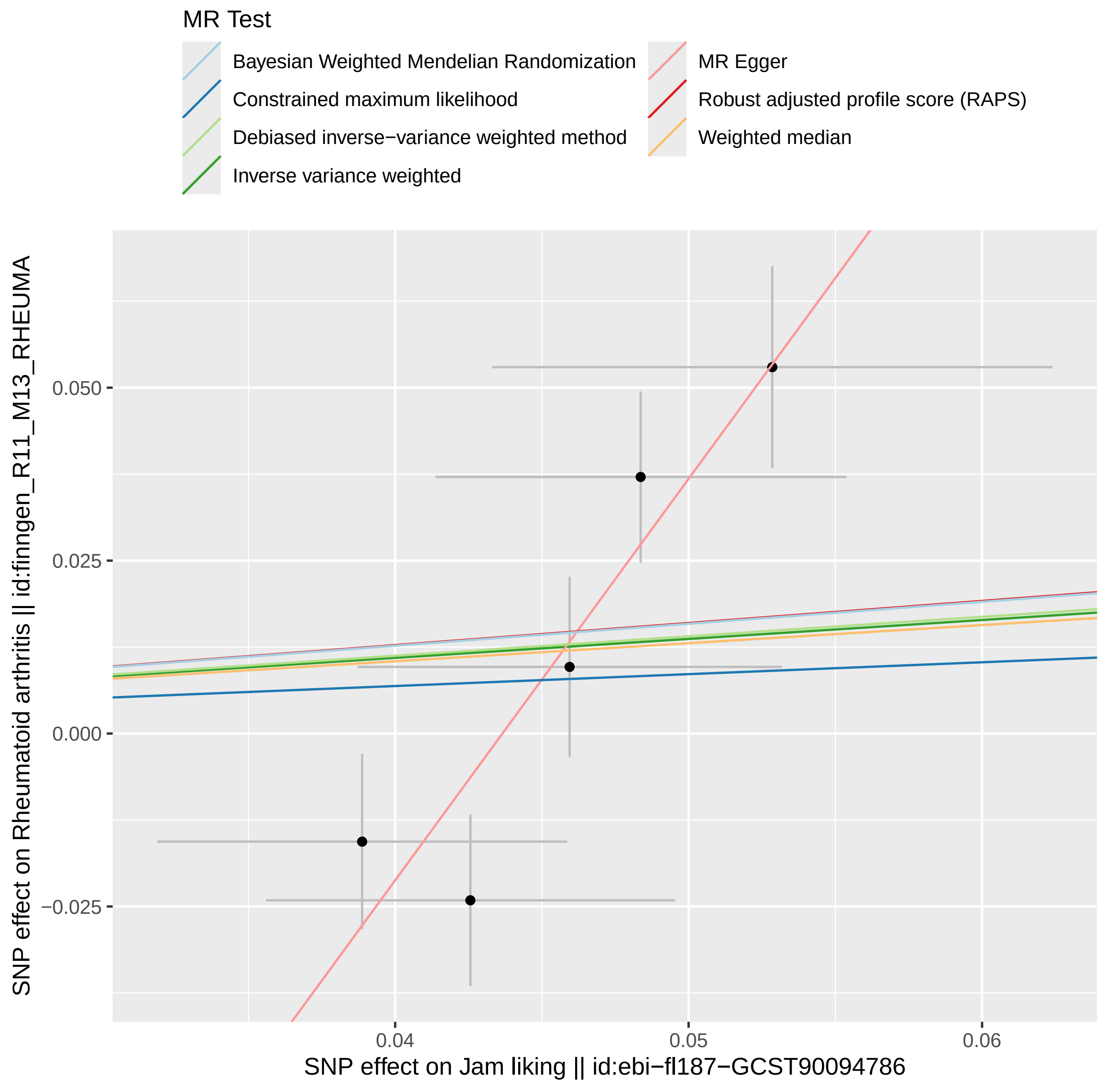
MR Test
Bayesian Weighted Mendelian Randomization
Constrained maximum likelihood
Debiased inverse−variance weighted method
Inverse variance weighted
MR Egger
Robust adjusted profile score (RAPS)
Weighted median
0.050
0.025
0.000
−0.025
0.04
0.05
0.06
SNP effect on Rheumatoid arthritis || id:finngen_R11_M13_RHEUMA
SNP effect on Jam liking || id:ebi−fl187−GCST90094786

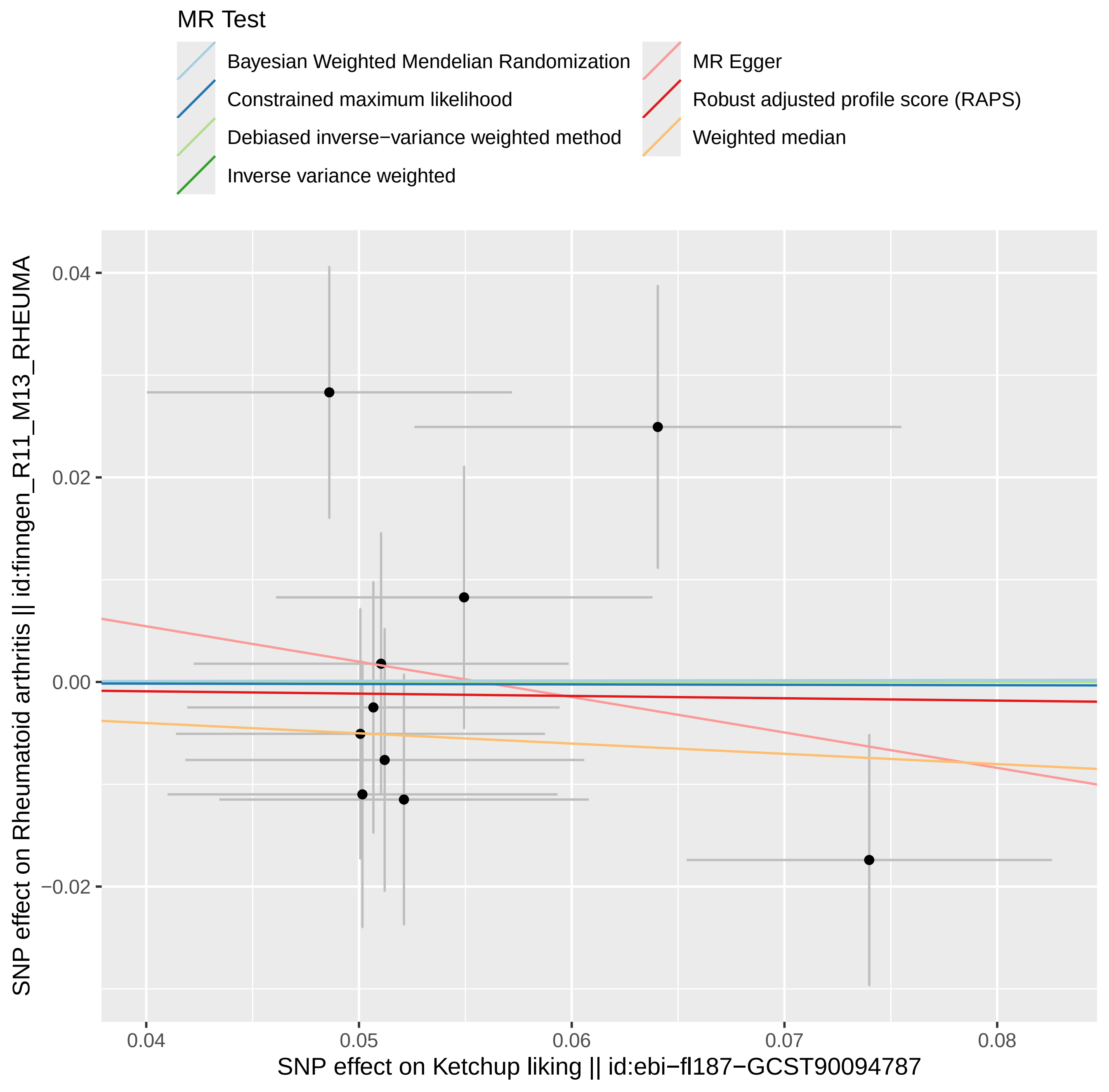
MR Test
Bayesian Weighted Mendelian Randomization
Constrained maximum likelihood
Debiased inverse−variance weighted method
Inverse variance weighted
MR Egger
Robust adjusted profile score (RAPS)
Weighted median
0.04
0.02
0.00
−0.02
0.04
0.05
0.06
0.07
0.08
SNP effect on Rheumatoid arthritis || id:finngen_R11_M13_RHEUMA
SNP effect on Ketchup liking || id:ebi−fl187−GCST90094787

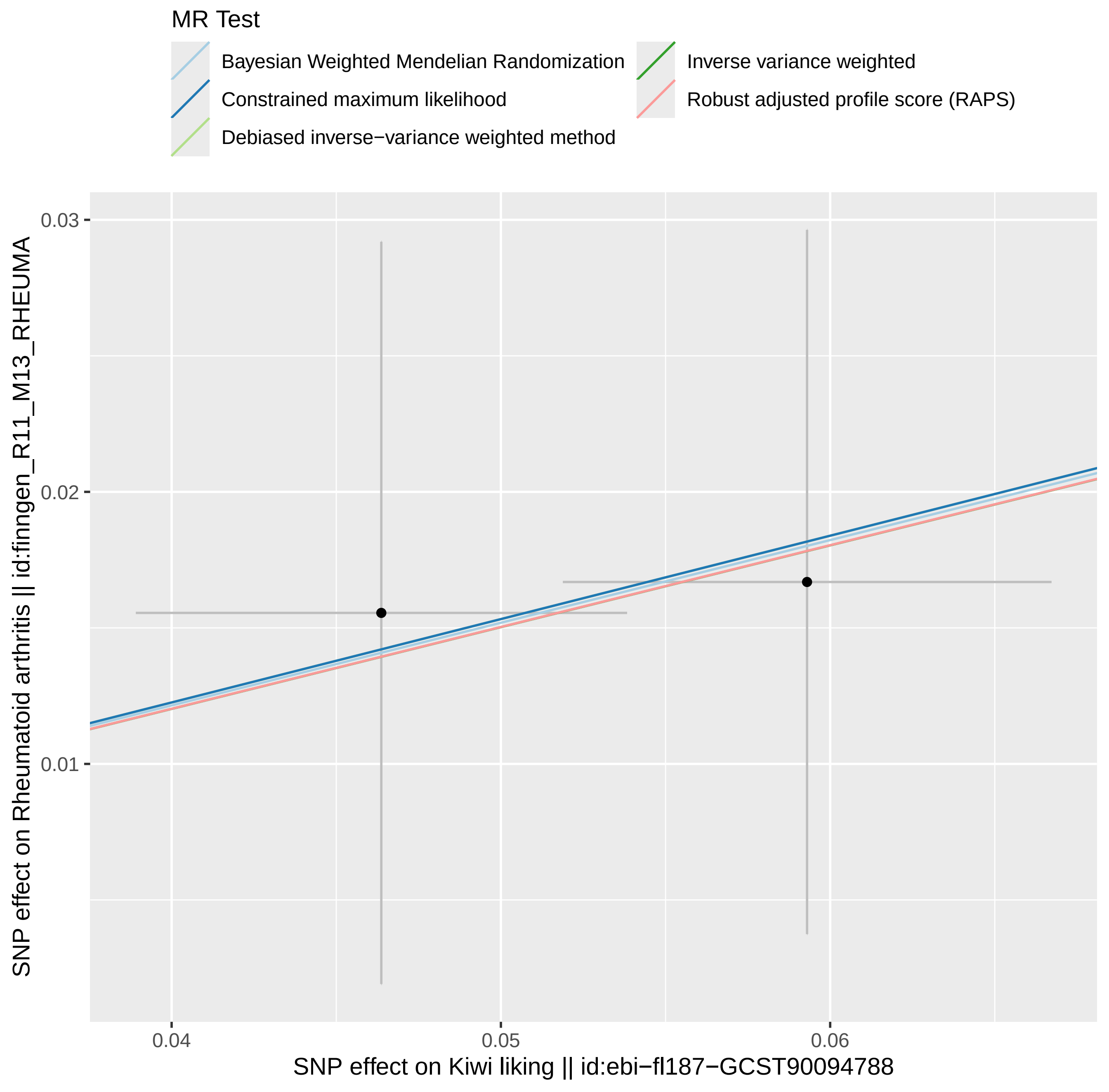
MR Test
Bayesian Weighted Mendelian Randomization
Constrained maximum likelihood
Debiased inverse−variance weighted method
Inverse variance weighted
Robust adjusted profile score (RAPS)
0.03
0.02
0.01
0.04
0.05
0.06
SNP effect on Rheumatoid arthritis || id:finngen_R11_M13_RHEUMA
SNP effect on Kiwi liking || id:ebi−fl187−GCST90094788

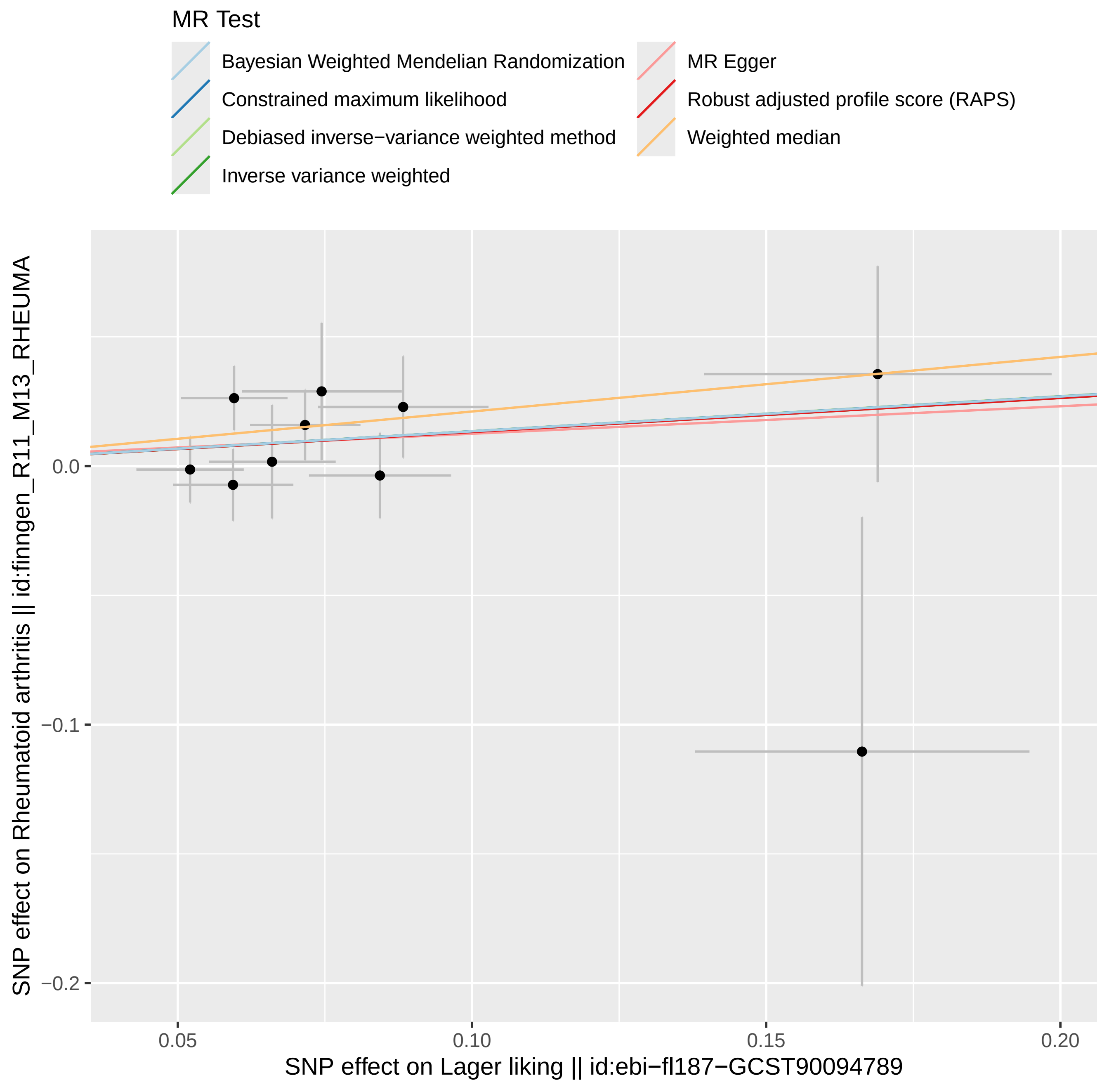

MR Test
Bayesian Weighted Mendelian Randomization
Constrained maximum likelihood
Debiased inverse−variance weighted method
Inverse variance weighted
MR Egger
Robust adjusted profile score (RAPS)
Weighted median
SNP effect on Rheumatoid arthritis || id:finngen_R11_M13_RHEUMA
0.0
−0.1
−0.2
0.05
0.10
0.15
0.20
SNP effect on Lager liking || id:ebi−fl187−GCST90094789

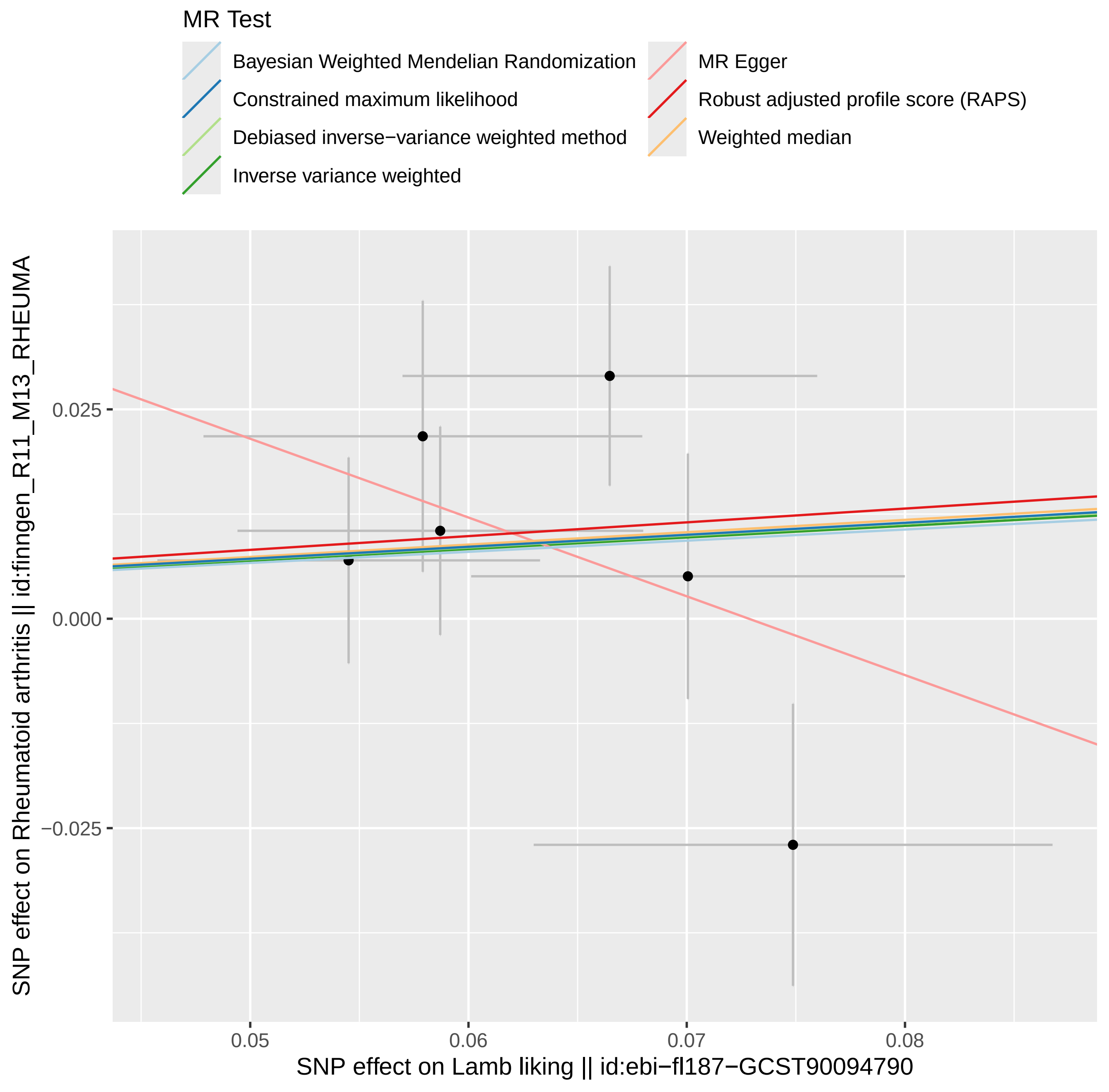
MR Test
Bayesian Weighted Mendelian Randomization
Constrained maximum likelihood
Debiased inverse−variance weighted method
Inverse variance weighted
MR Egger
Robust adjusted profile score (RAPS)
Weighted median
0.025
0.000
−0.025
0.05
0.06
0.07
0.08
SNP effect on Rheumatoid arthritis || id:finngen_R11_M13_RHEUMA
SNP effect on Lamb liking || id:ebi−fl187−GCST90094790

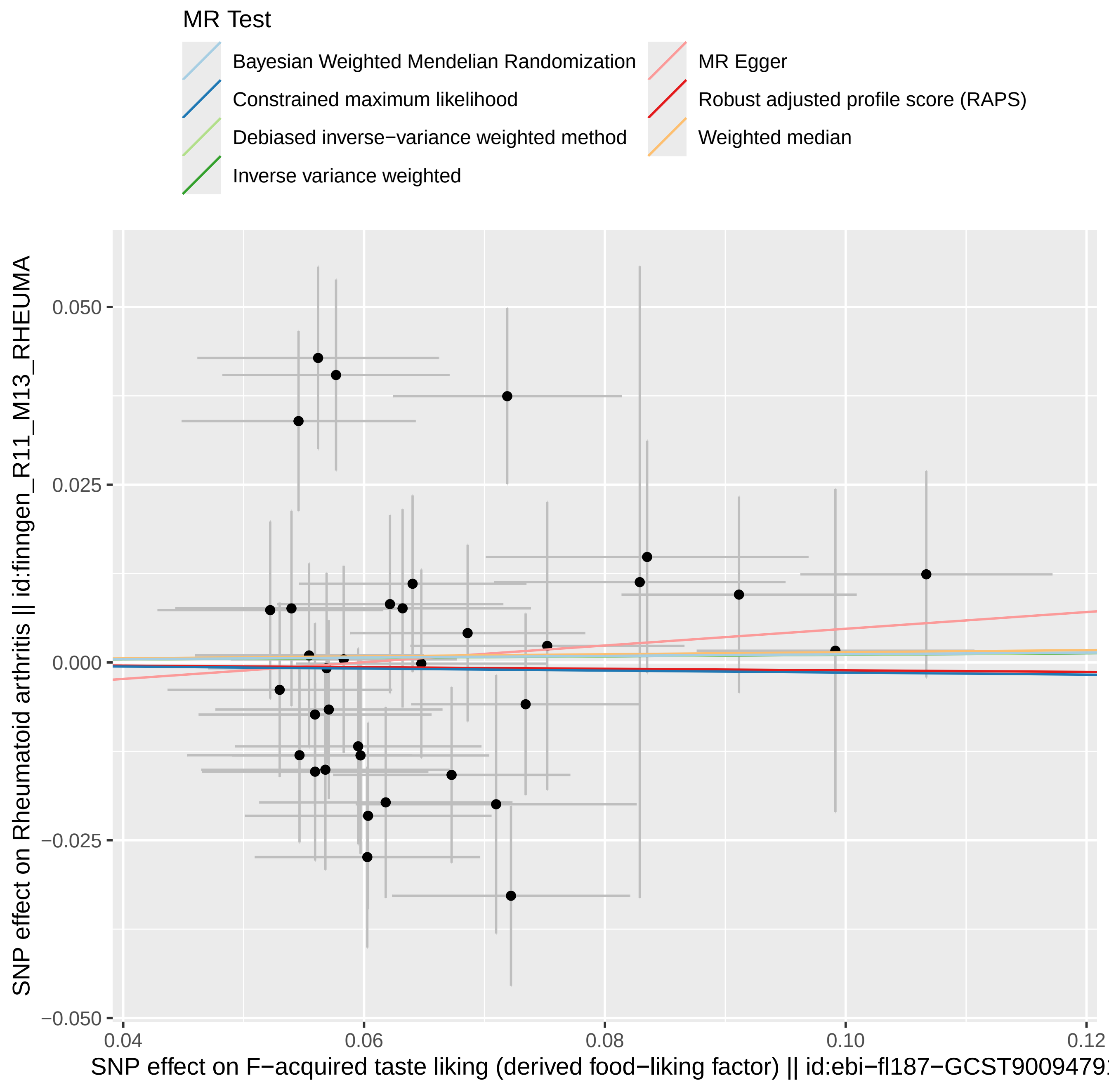
MR Test
Bayesian Weighted Mendelian Randomization
Constrained maximum likelihood
Debiased inverse−variance weighted method
Inverse variance weighted
MR Egger
Robust adjusted profile score (RAPS)
Weighted median
0.050
0.025
0.000
−0.025
−0.050
0.04
0.06
0.08
0.10
0.12
SNP effect on Rheumatoid arthritis || id:finngen_R11_M13_RHEUMA
SNP effect on F−acquired taste liking (derived food−liking factor) || id:ebi−fl187−GCST9009479

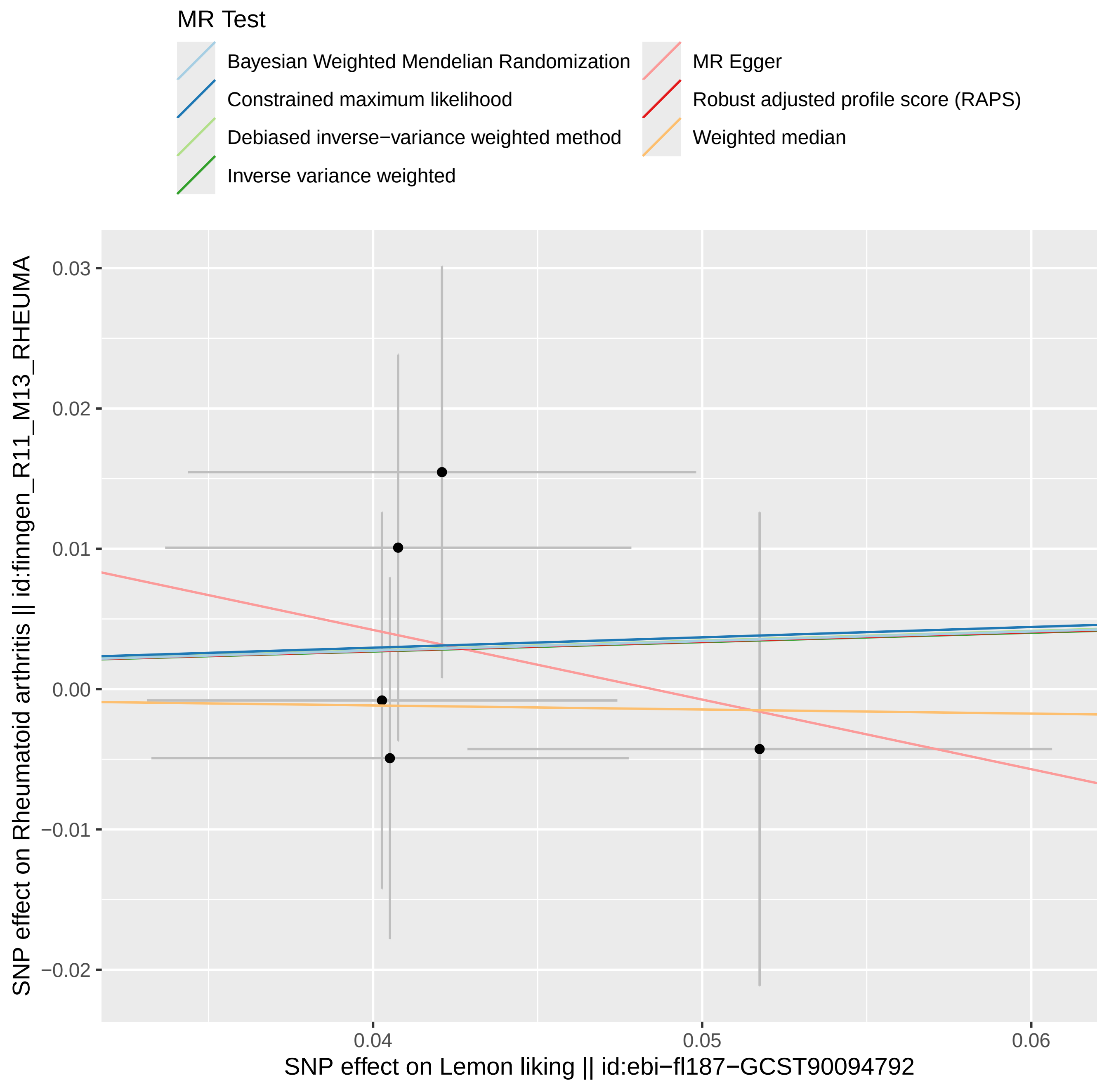
MR Test
Bayesian Weighted Mendelian Randomization
Constrained maximum likelihood
Debiased inverse−variance weighted method
Inverse variance weighted
MR Egger
Robust adjusted profile score (RAPS)
Weighted median
0.03
0.02
0.01
0.00
−0.01
−0.02
0.04
0.05
0.06
SNP effect on Rheumatoid arthritis || id:finngen_R11_M13_RHEUMA
SNP effect on Lemon liking || id:ebi−fI187−GCST90094792

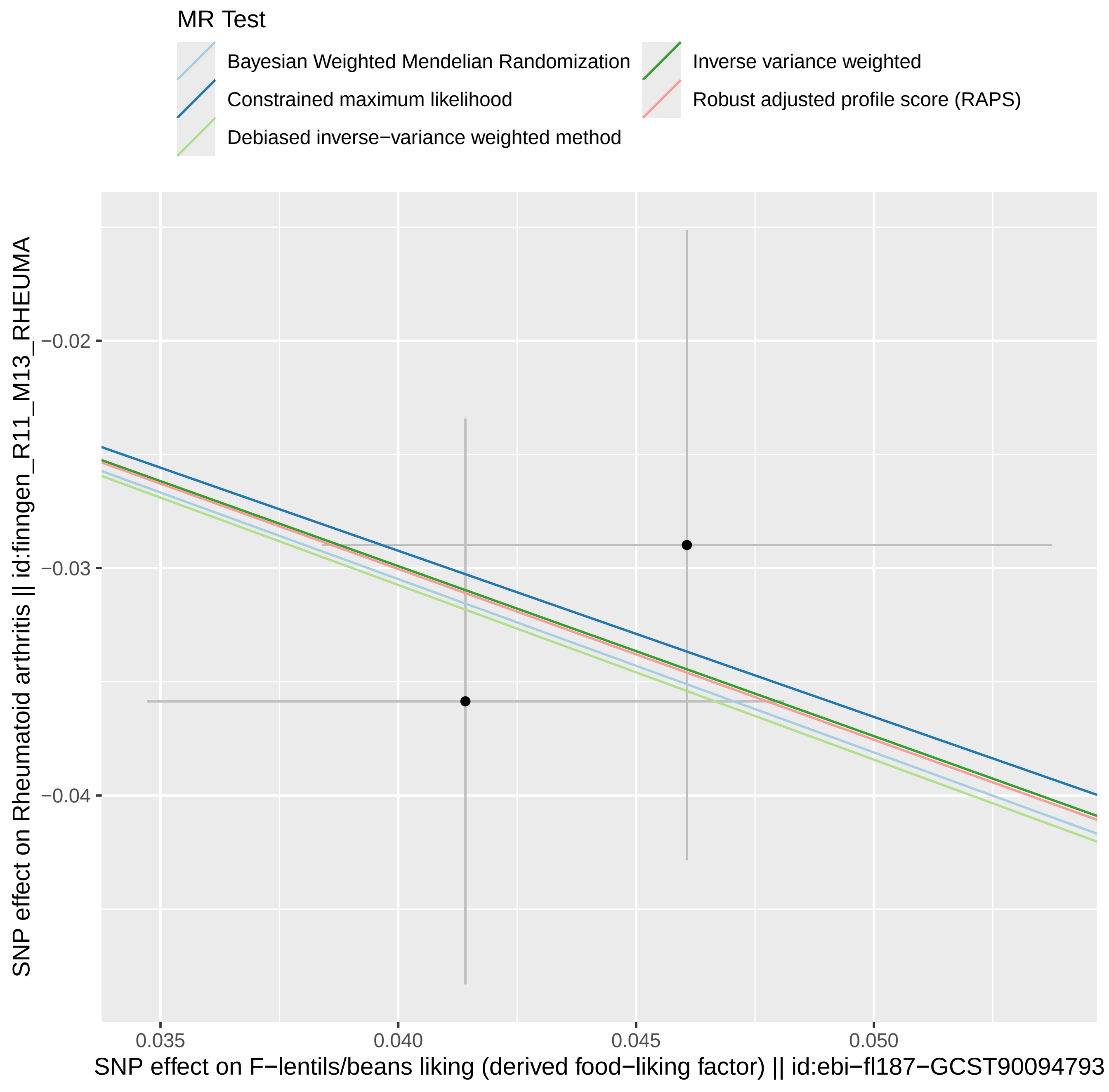
MR Test
Bayesian Weighted Mendelian Randomization
Constrained maximum likelihood
Debiased inverse−variance weighted method
Inverse variance weighted
Robust adjusted profile score (RAPS)
−0.02
−0.03
−0.04
0.035
0.040
0.045
0.050
SNP effect on Rheumatoid arthritis || id:finngen_R11_M13_RHEUMA
SNP effect on F−lentils/beans liking (derived food−liking factor) || id:ebi−fl187−GCST90094793

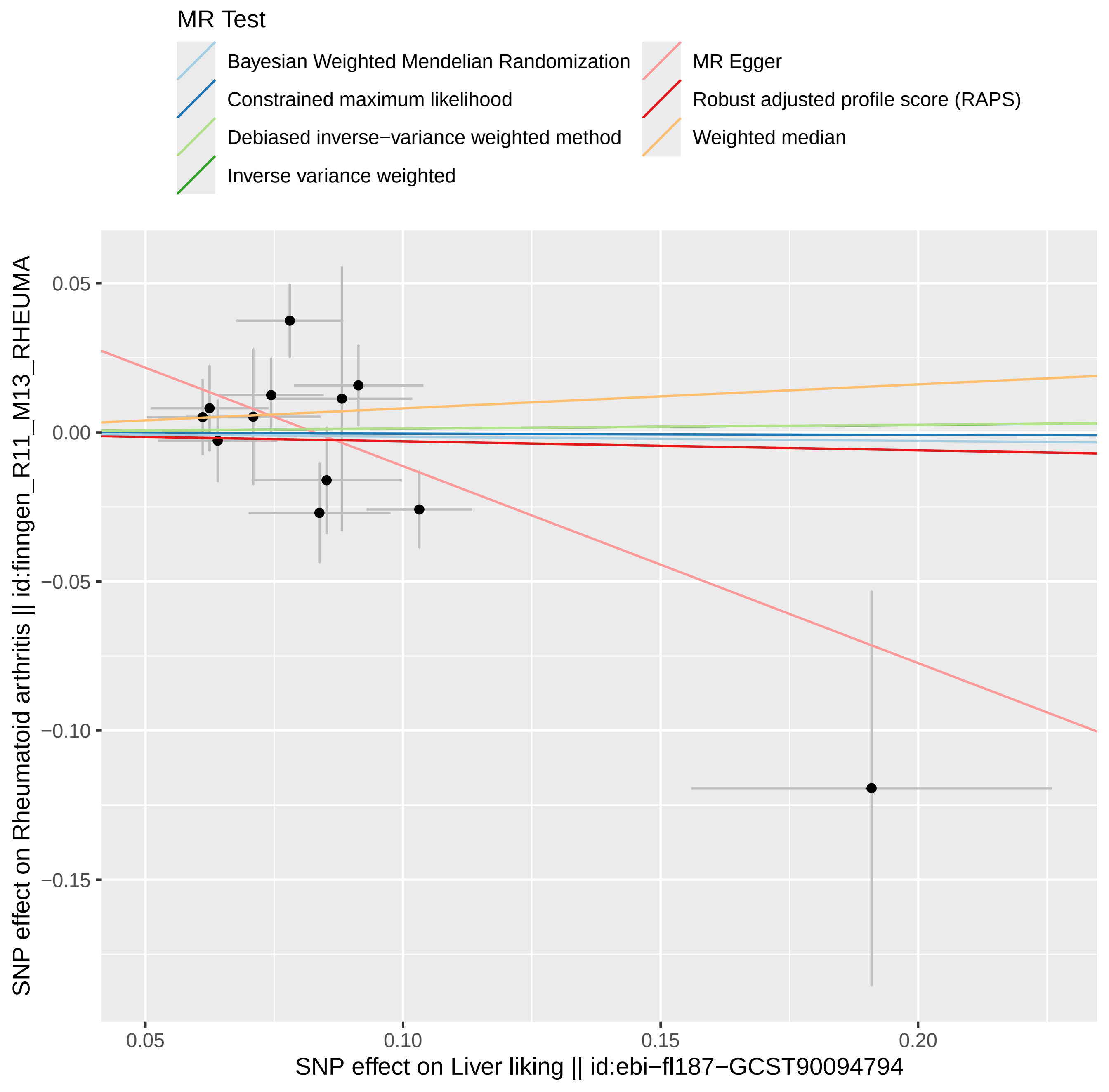
MR Test
Bayesian Weighted Mendelian Randomization
Constrained maximum likelihood
Debiased inverse−variance weighted method
Inverse variance weighted
MR Egger
Robust adjusted profile score (RAPS)
Weighted median
SNP effect on Rheumatoid arthritis || id:finngen_R11_M13_RHEUMA
0.05
0.00
−0.05
−0.10
−0.15
0.05
0.10
0.15
0.20
SNP effect on Liver liking || id:ebi−fl187−GCST90094794

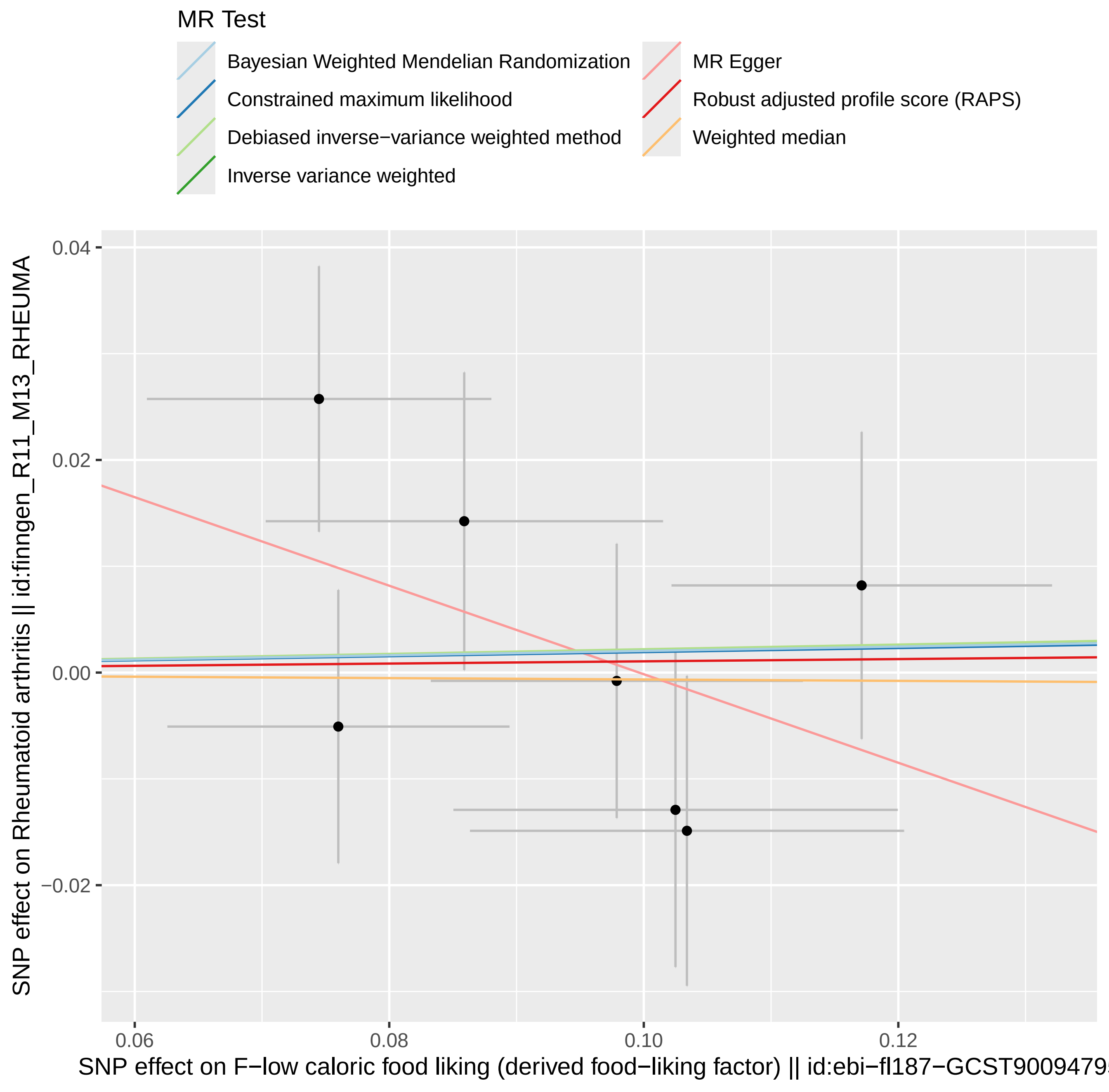

MR Test
Bayesian Weighted Mendelian Randomization
Constrained maximum likelihood
Debiased inverse−variance weighted method
Inverse variance weighted
MR Egger
Robust adjusted profile score (RAPS)
Weighted median
0.04
0.02
0.00
−0.02
0.06
0.08
0.10
0.12
SNP effect on Rheumatoid arthritis || id:finngen_R11_M13_RHEUMA
SNP effect on F−low caloric food liking (derived food−liking factor) || id:ebi−fl187−GCST9009479

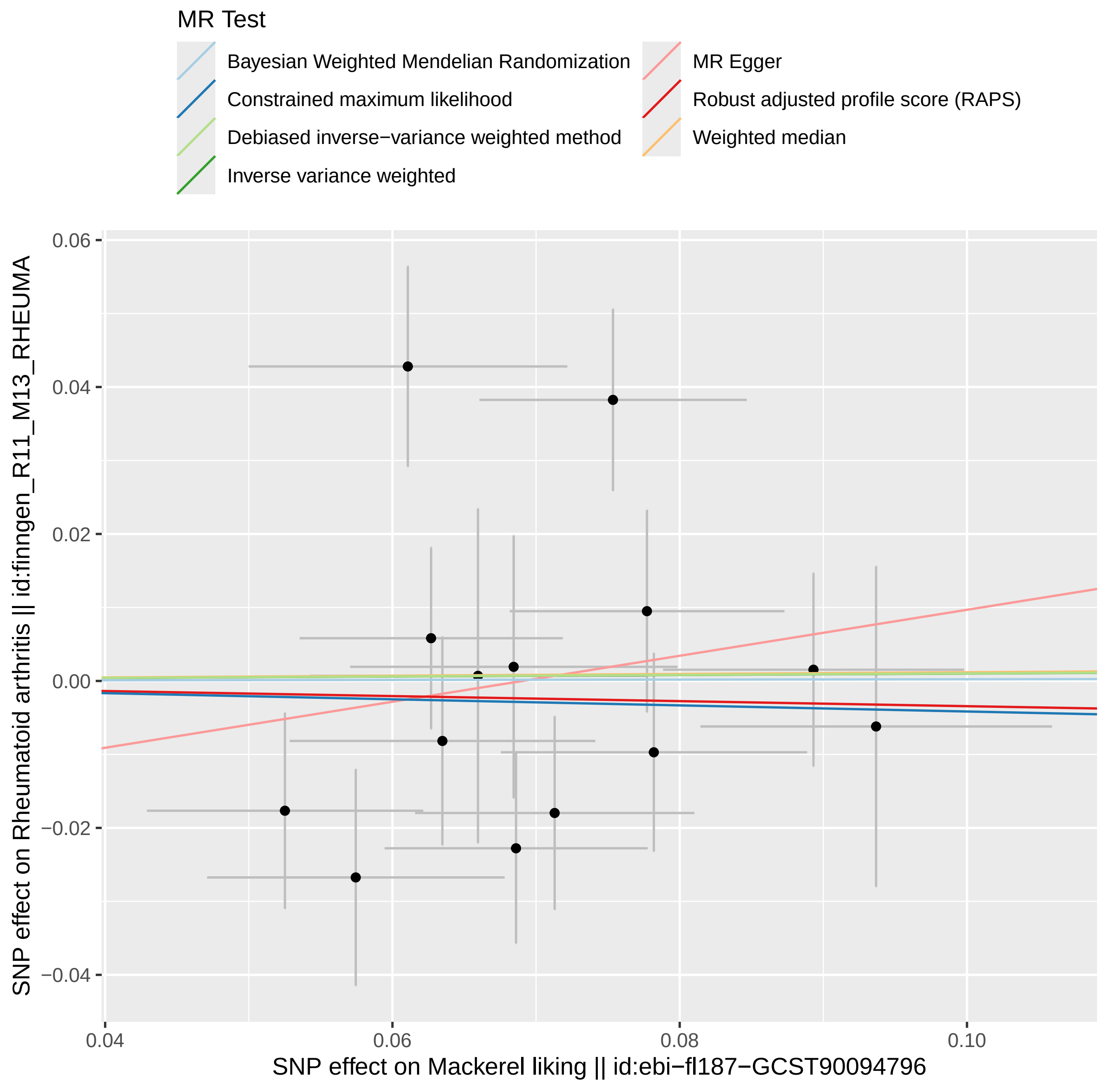
MR Test
Bayesian Weighted Mendelian Randomization
Constrained maximum likelihood
Debiased inverse−variance weighted method
Inverse variance weighted
MR Egger
Robust adjusted profile score (RAPS)
Weighted median
SNP effect on Rheumatoid arthritis || id:finngen_R11_M13_RHEUMA
SNP effect on Mackerel liking || id:ebi−fl187−GCST90094796
0.06
0.04
0.02
0.00
−0.02
−0.04
0.04
0.06
0.08
0.10

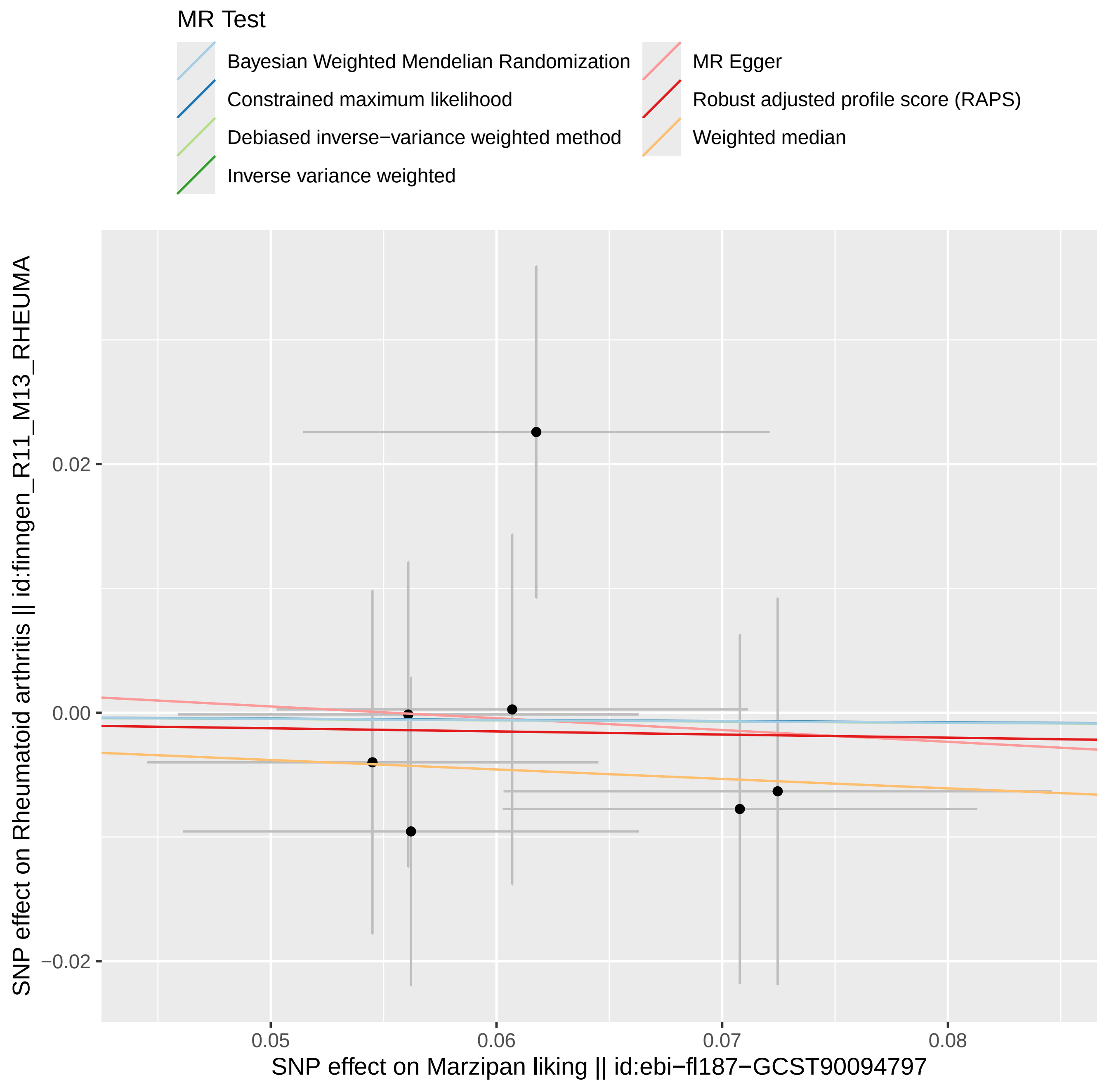

MR Test
Bayesian Weighted Mendelian Randomization
Constrained maximum likelihood
Debiased inverse−variance weighted method
Inverse variance weighted
MR Egger
Robust adjusted profile score (RAPS)
Weighted median
0.02
0.00
−0.02
0.05
0.06
0.07
0.08
SNP effect on Rheumatoid arthritis || id:finngen_R11_M13_RHEUMA
SNP effect on Marzipan liking || id:ebi−fl187−GCST90094797

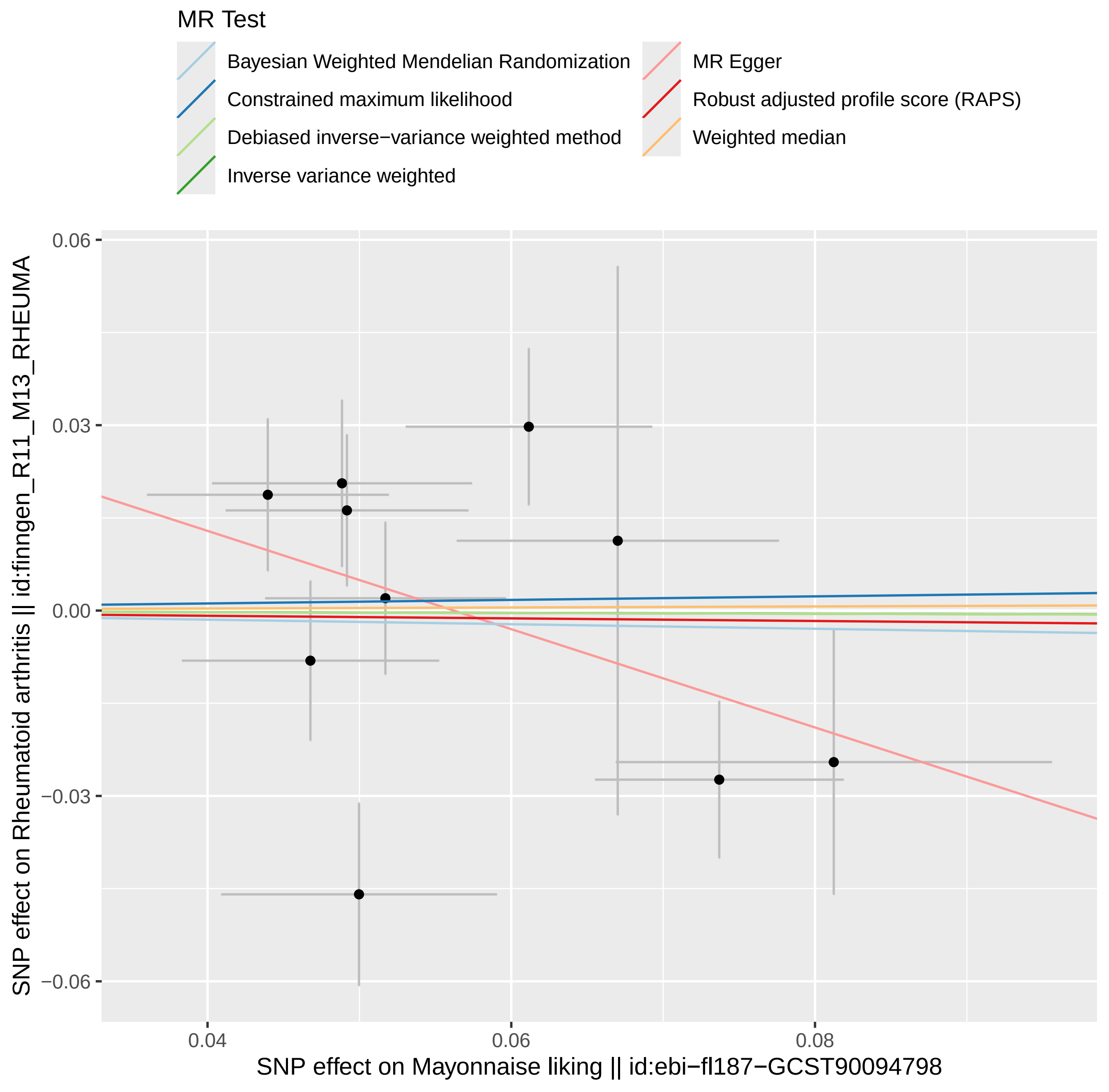
MR Test
Bayesian Weighted Mendelian Randomization
Constrained maximum likelihood
Debiased inverse−variance weighted method
Inverse variance weighted
MR Egger
Robust adjusted profile score (RAPS)
Weighted median
0.06
0.03
0.00
−0.03
−0.06
0.04
0.06
0.08
SNP effect on Rheumatoid arthritis || id:finngen_R11_M13_RHEUMA
SNP effect on Mayonnaise liking || id:ebi−fl187−GCST90094798

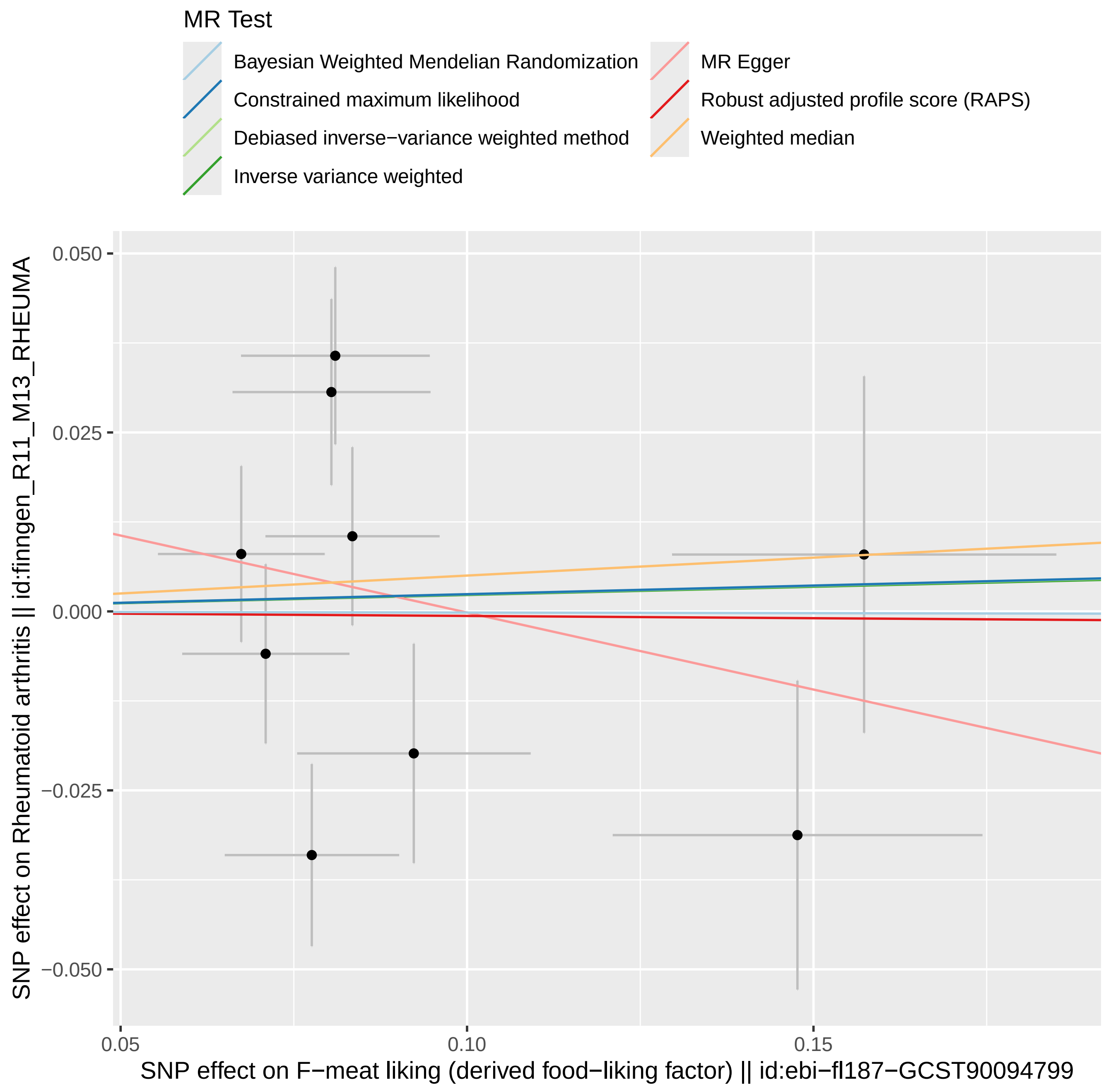

MR Test
Bayesian Weighted Mendelian Randomization
Constrained maximum likelihood
Debiased inverse−variance weighted method
Inverse variance weighted
MR Egger
Robust adjusted profile score (RAPS)
Weighted median
0.050
0.025
0.000
−0.025
−0.050
0.05
0.10
0.15
SNP effect on Rheumatoid arthritis || id:finngen_R11_M13_RHEUMA
SNP effect on F−meat liking (derived food−liking factor) || id:ebi−fl187−GCST90094799

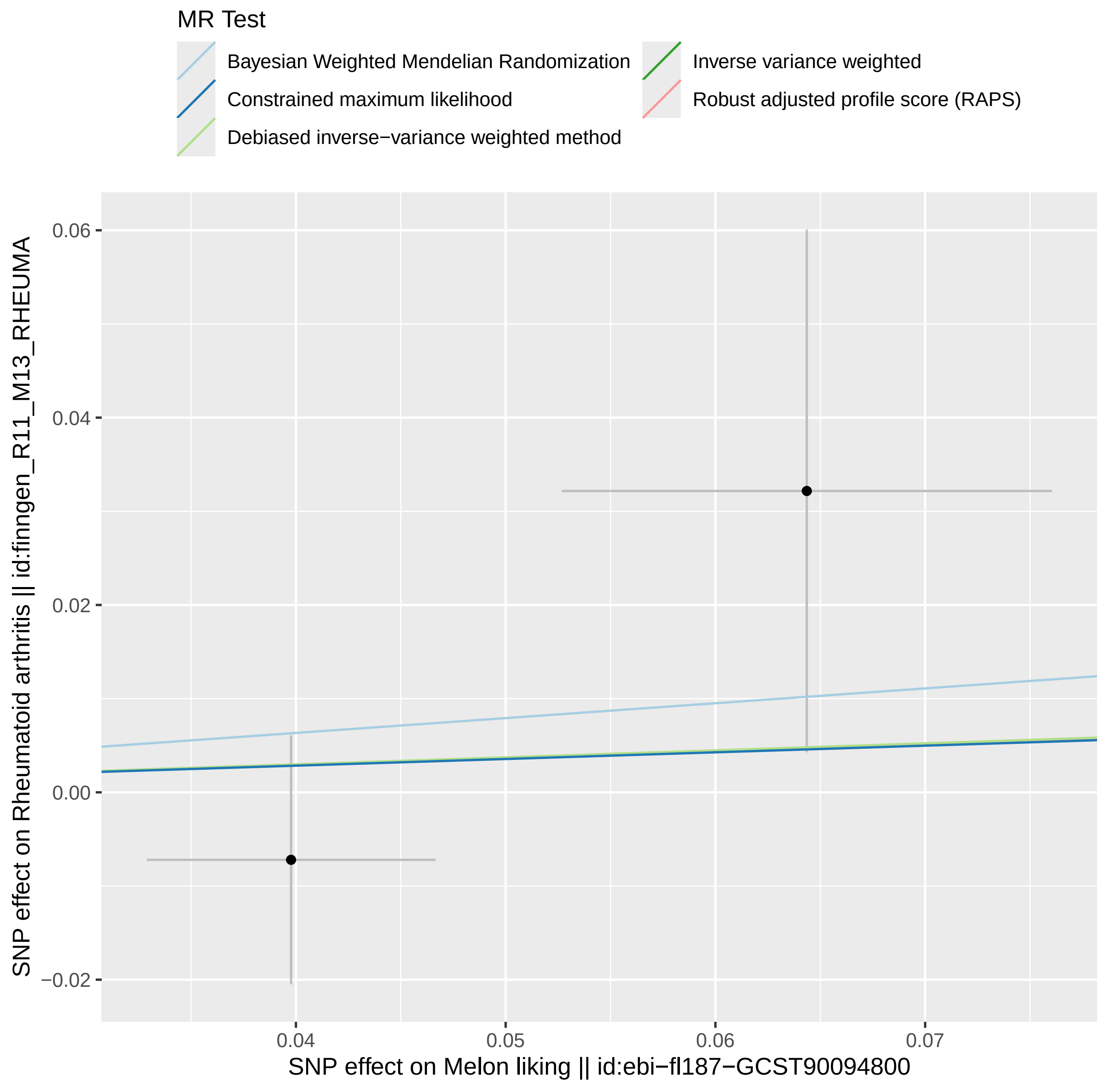
MR Test
Bayesian Weighted Mendelian Randomization
Constrained maximum likelihood
Debiased inverse−variance weighted method
Inverse variance weighted
Robust adjusted profile score (RAPS)
0.06
0.04
0.02
0.00
−0.02
0.04
0.05
0.06
0.07
SNP effect on Rheumatoid arthritis || id:finngen_R11_M13_RHEUMA
SNP effect on Melon liking || id:ebi−fl187−GCST90094800

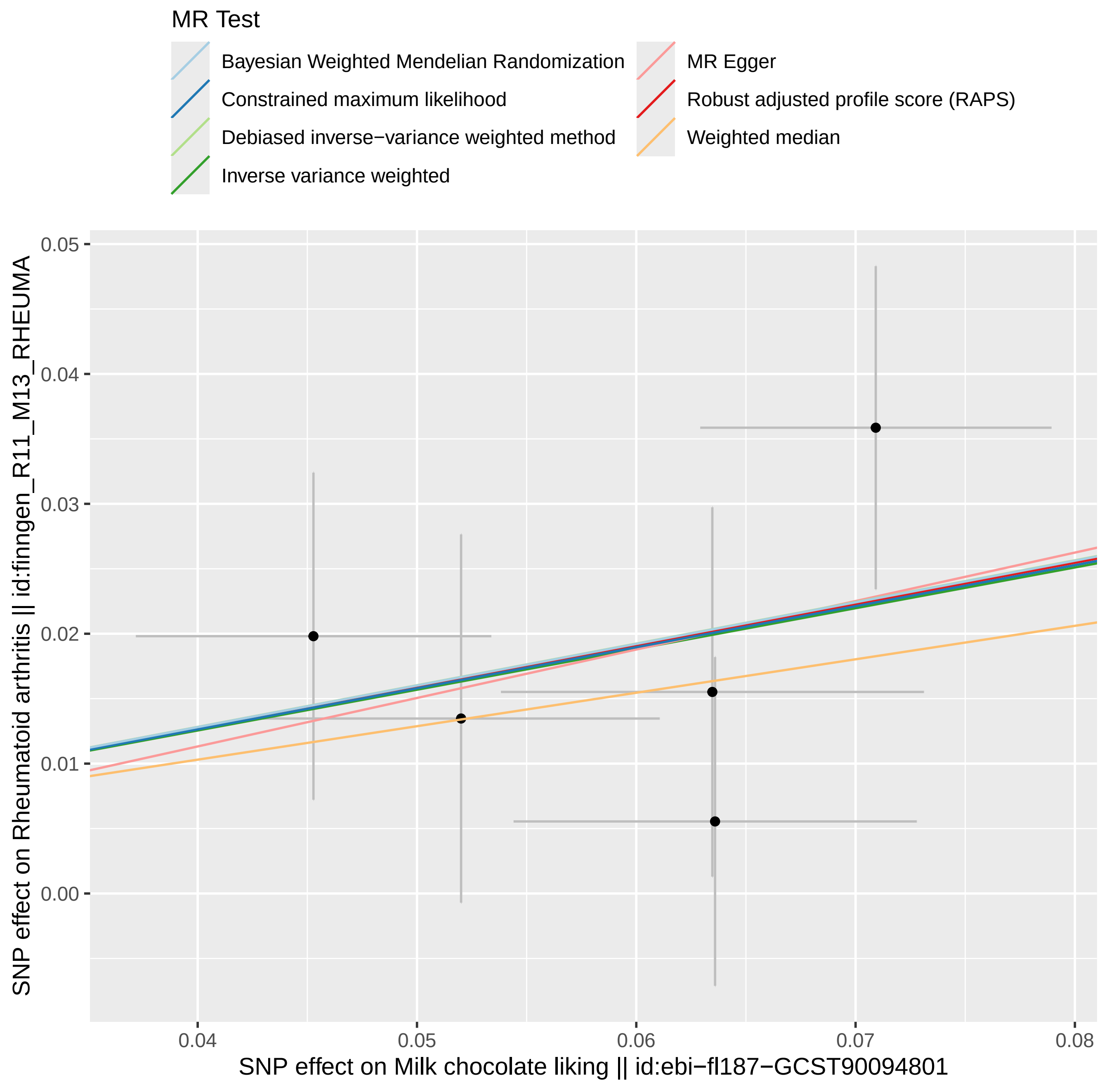
MR Test
Bayesian Weighted Mendelian Randomization
Constrained maximum likelihood
Debiased inverse−variance weighted method
Inverse variance weighted
MR Egger
Robust adjusted profile score (RAPS)
Weighted median
SNP effect on Rheumatoid arthritis || id:finngen_R11_M13_RHEUMA
0.05
0.04
0.03
0.02
0.01
0.00
0.04
0.05
0.06
0.07
0.08
SNP effect on Milk chocolate liking || id:ebi−fl187−GCST90094801

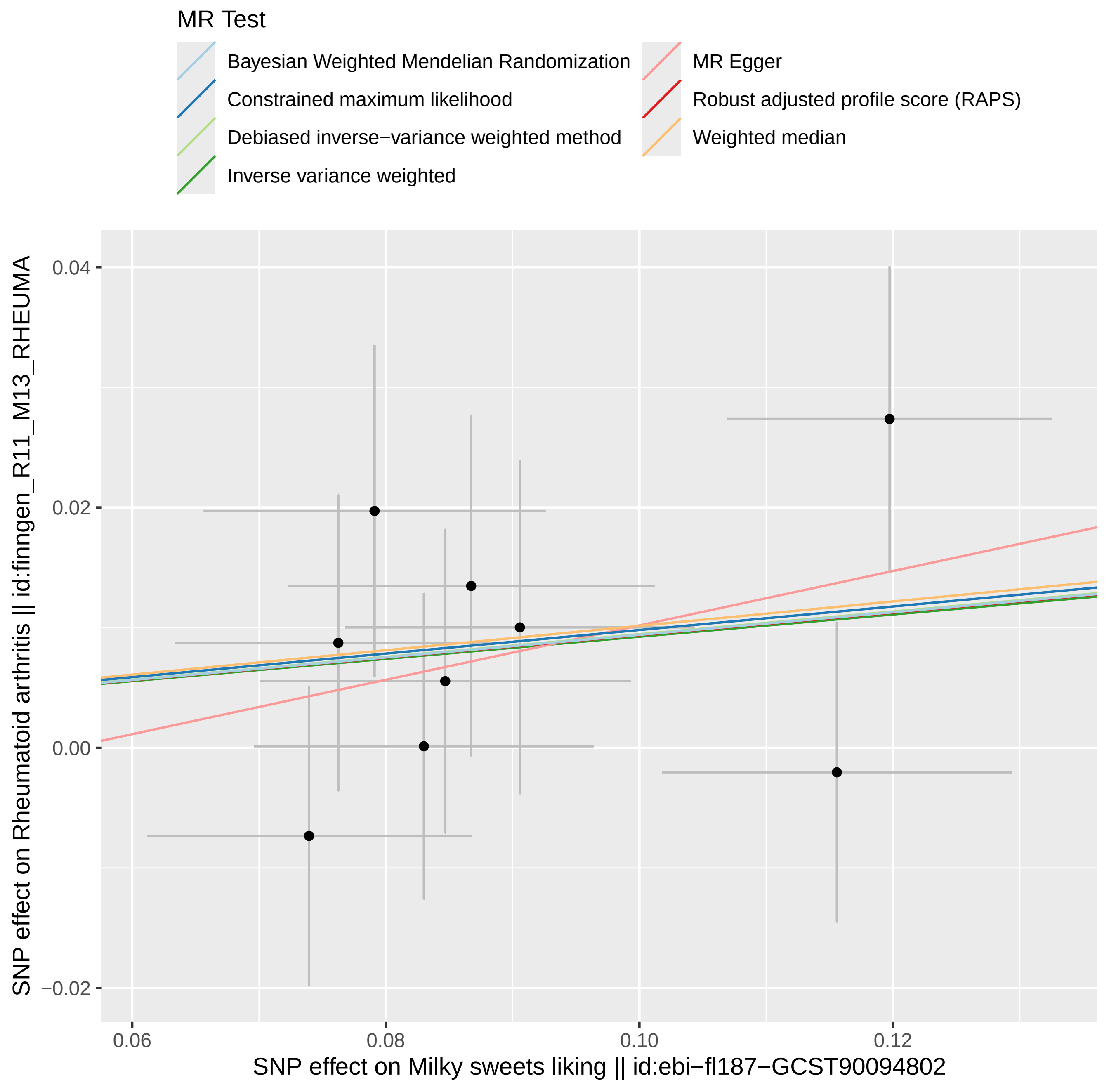
MR Test
Bayesian Weighted Mendelian Randomization
Constrained maximum likelihood
Debiased inverse−variance weighted method
Inverse variance weighted
MR Egger
Robust adjusted profile score (RAPS)
Weighted median
0.04
0.02
0.00
−0.02
0.06
0.08
0.10
0.12
SNP effect on Rheumatoid arthritis || id:finngen_R11_M13_RHEUMA
SNP effect on Milky sweets liking || id:ebi−fl187−GCST90094802

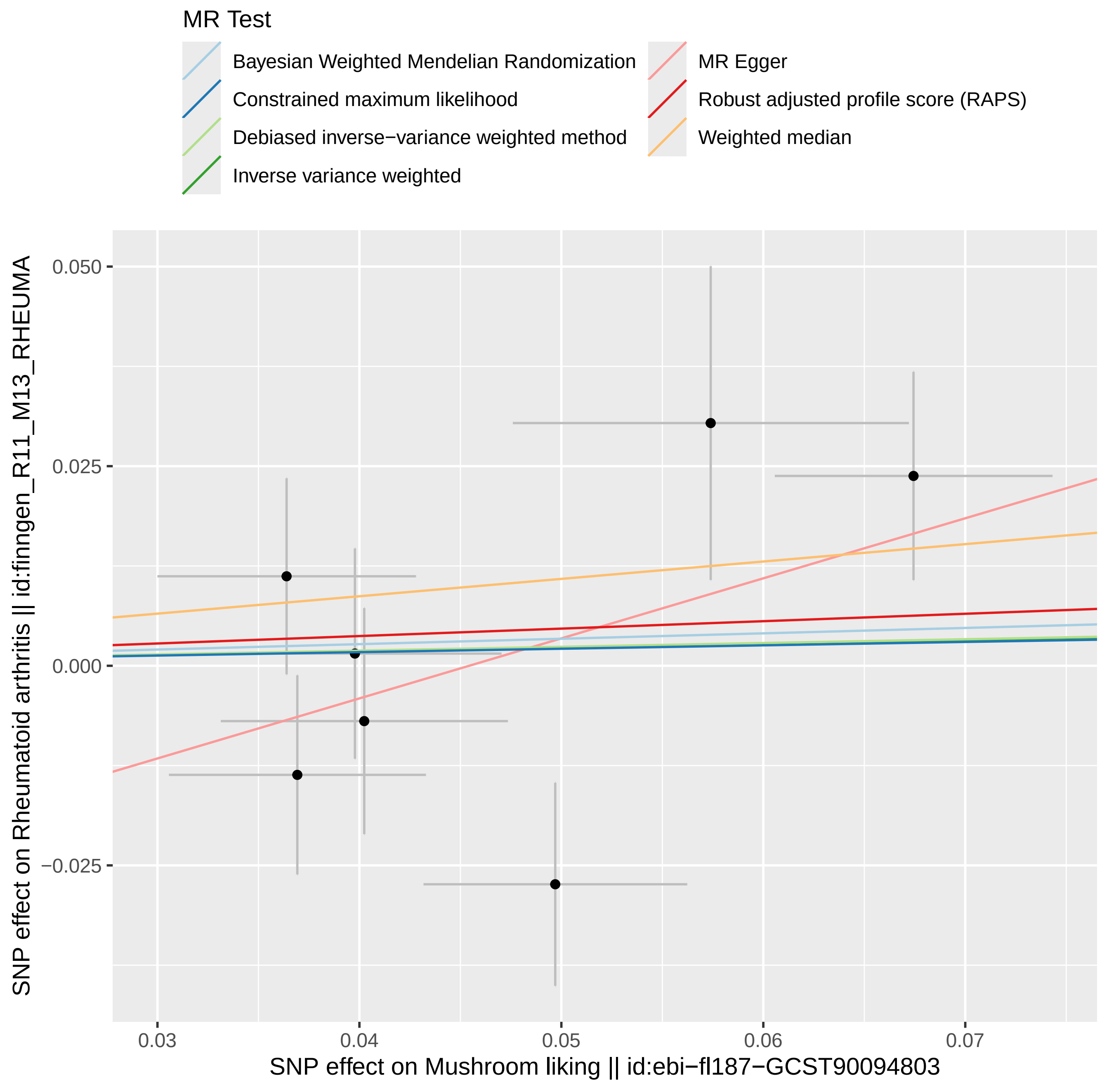
MR Test
Bayesian Weighted Mendelian Randomization
Constrained maximum likelihood
Debiased inverse−variance weighted method
Inverse variance weighted
MR Egger
Robust adjusted profile score (RAPS)
Weighted median
0.050
0.025
0.000
−0.025
0.03
0.04
0.05
0.06
0.07
SNP effect on Rheumatoid arthritis || id:finngen_R11_M13_RHEUMA
SNP effect on Mushroom liking || id:ebi−fl187−GCST90094803

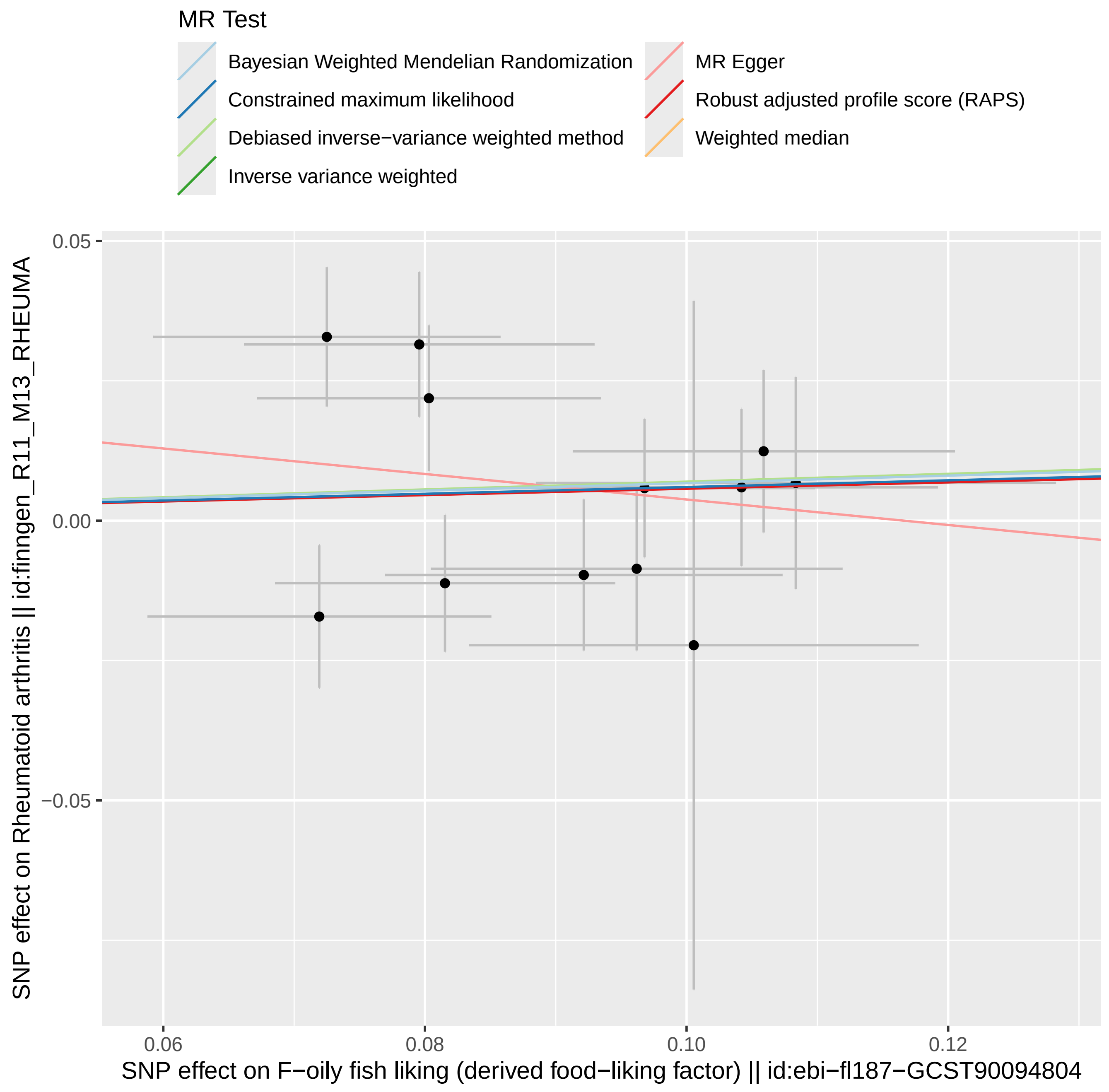
MR Test
Bayesian Weighted Mendelian Randomization
Constrained maximum likelihood
Debiased inverse−variance weighted method
Inverse variance weighted
MR Egger
Robust adjusted profile score (RAPS)
Weighted median
0.05
0.00
−0.05
0.06
0.08
0.10
0.12
SNP effect on Rheumatoid arthritis || id:finngen_R11_M13_RHEUMA
SNP effect on F−oily fish liking (derived food−liking factor) || id:ebi−fl187−GCST90094804

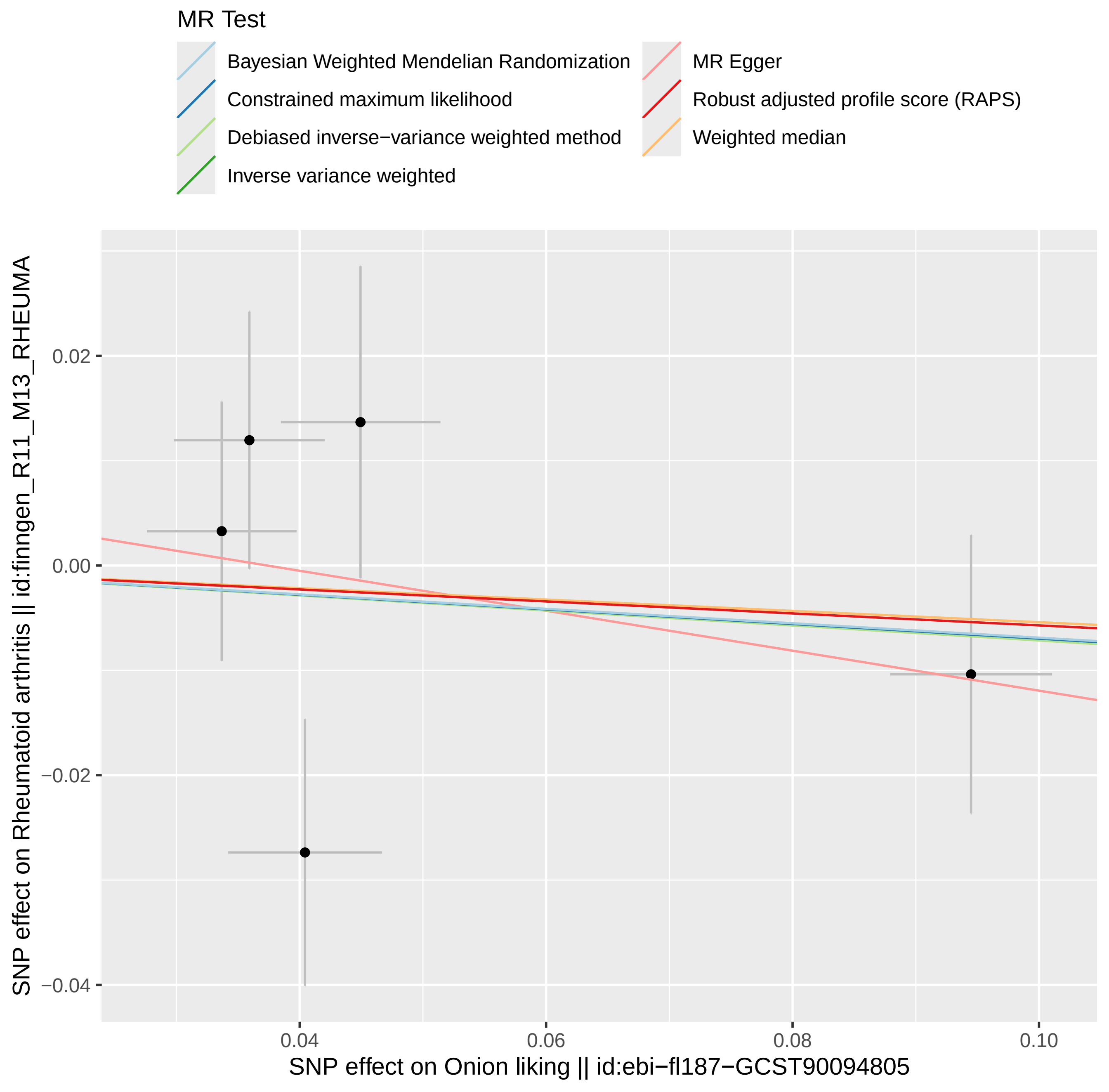
MR Test
Bayesian Weighted Mendelian Randomization
Constrained maximum likelihood
Debiased inverse−variance weighted method
Inverse variance weighted
MR Egger
Robust adjusted profile score (RAPS)
Weighted median
0.02
0.00
−0.02
−0.04
0.04
0.06
0.08
0.10
SNP effect on Rheumatoid arthritis || id:finngen_R11_M13_RHEUMA
SNP effect on Onion liking || id:ebi−fl187−GCST90094805

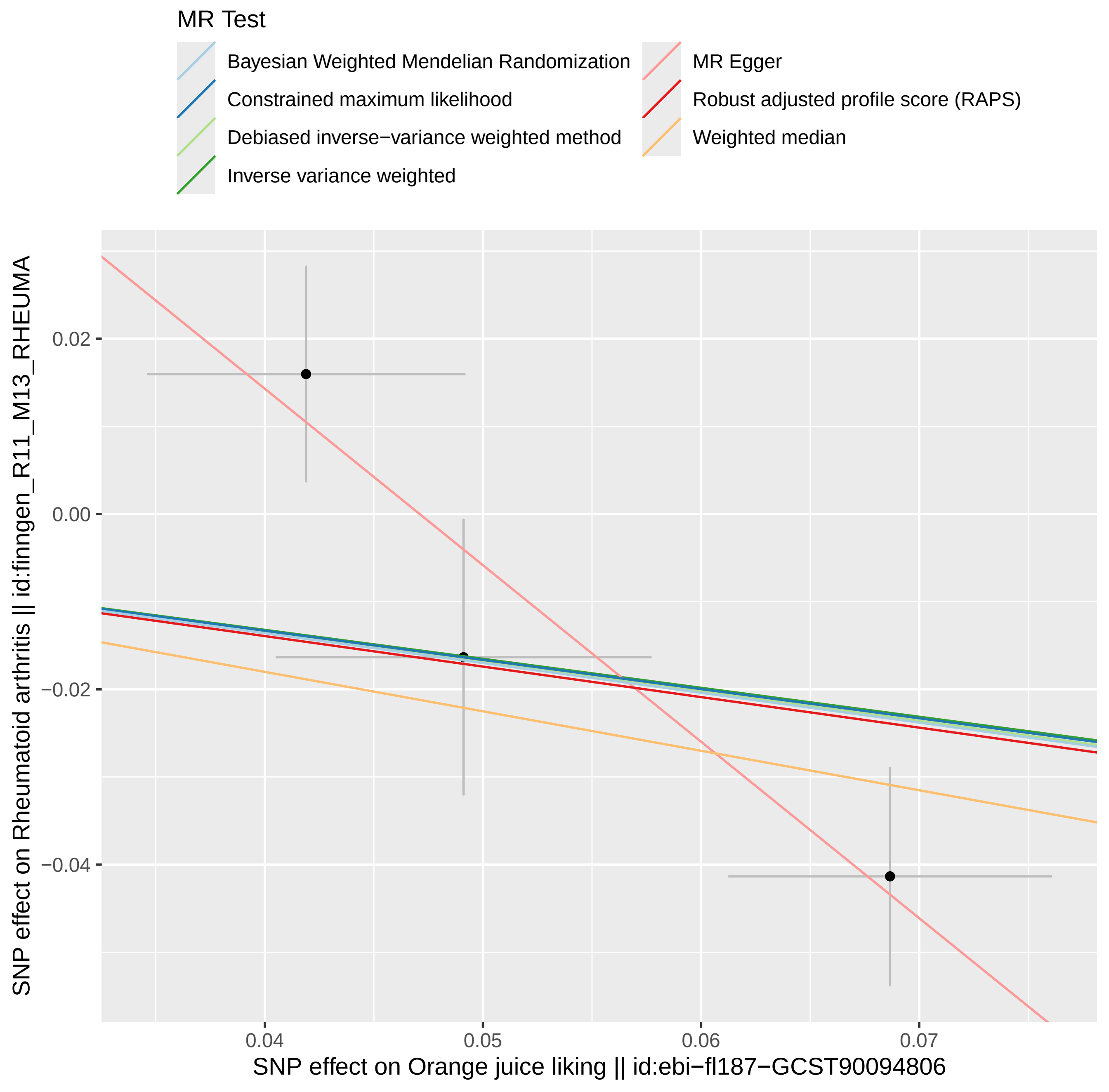
MR Test
Bayesian Weighted Mendelian Randomization
Constrained maximum likelihood
Debiased inverse−variance weighted method
Inverse variance weighted
MR Egger
Robust adjusted profile score (RAPS)
Weighted median
SNP effect on Rheumatoid arthritis || id:finngen_R11_M13_RHEUMA
0.02
0.00
−0.02
−0.04
0.04
0.05
0.06
0.07
SNP effect on Orange juice liking || id:ebi−fl187−GCST90094806

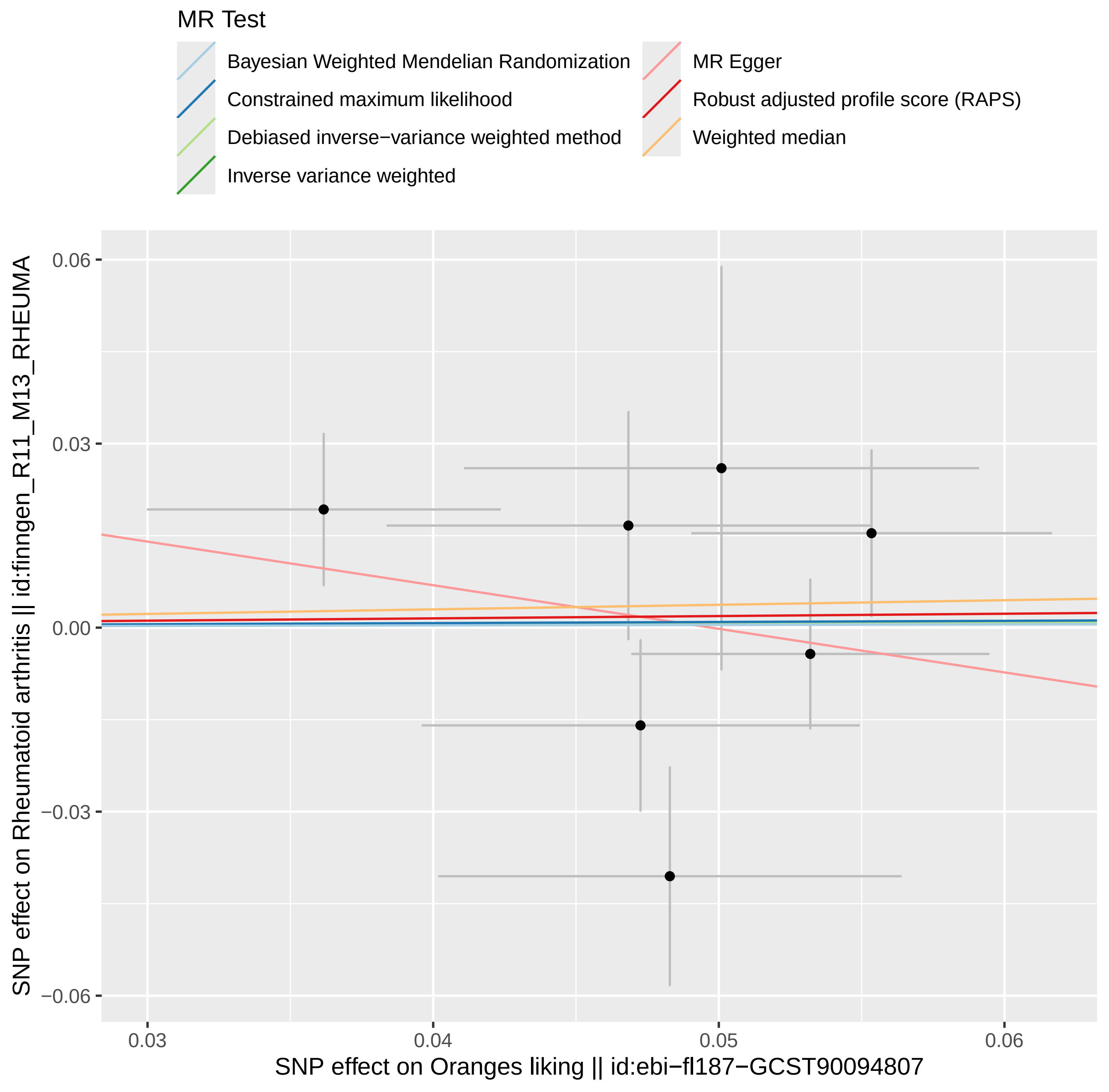
MR Test
Bayesian Weighted Mendelian Randomization
Constrained maximum likelihood
Debiased inverse−variance weighted method
Inverse variance weighted
MR Egger
Robust adjusted profile score (RAPS)
Weighted median
0.06
0.03
0.00
−0.03
−0.06
SNP effect on Rheumatoid arthritis || id:finngen_R11_M13_RHEUMA
0.03
0.04
0.05
0.06
SNP effect on Oranges liking || id:ebi−fl187−GCST90094807

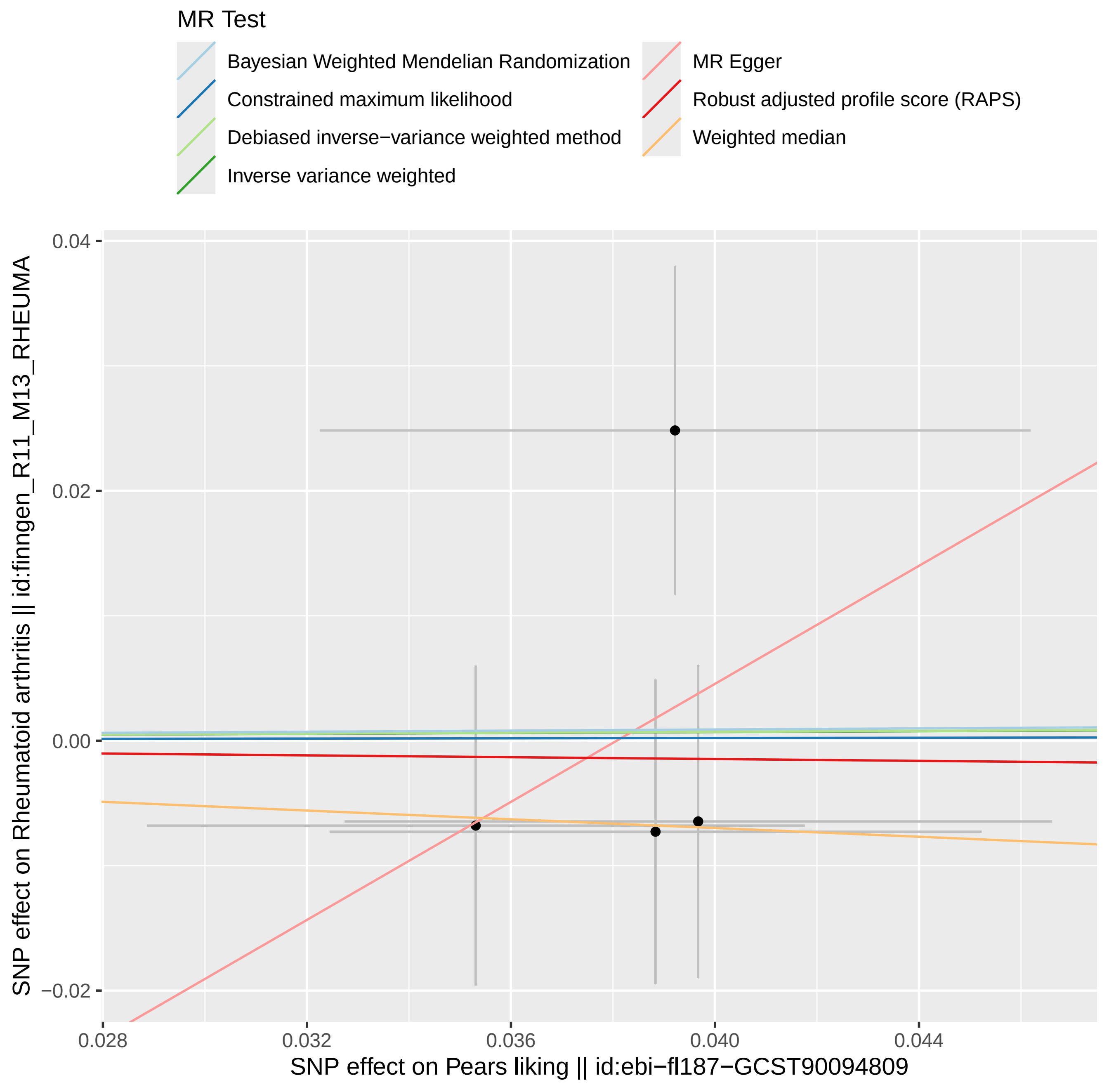
MR Test
Bayesian Weighted Mendelian Randomization
Constrained maximum likelihood
Debiased inverse−variance weighted method
Inverse variance weighted
MR Egger
Robust adjusted profile score (RAPS)
Weighted median
0.04
0.02
0.00
−0.02
0.028
0.032
0.036
0.040
0.044
SNP effect on Rheumatoid arthritis || id:finngen_R11_M13_RHEUMA
SNP effect on Pears liking || id:ebi−fl187−GCST90094809

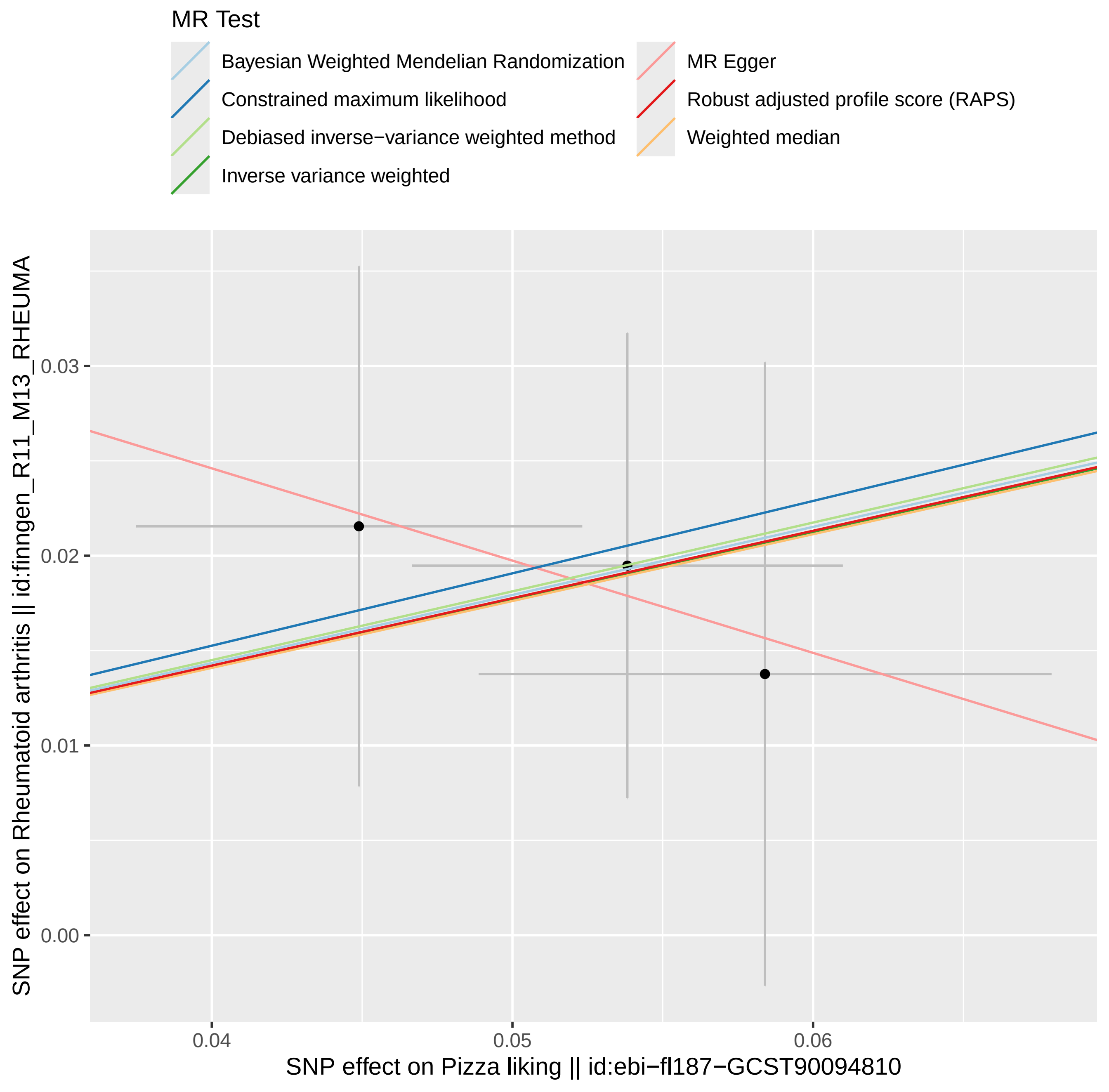
MR Test
Bayesian Weighted Mendelian Randomization
Constrained maximum likelihood
Debiased inverse−variance weighted method
Inverse variance weighted
MR Egger
Robust adjusted profile score (RAPS)
Weighted median
SNP effect on Rheumatoid arthritis || id:finngen_R11_M13_RHEUMA
0.03
0.02
0.01
0.00
0.04
0.05
0.06
SNP effect on Pizza liking || id:ebi−fl187−GCST90094810

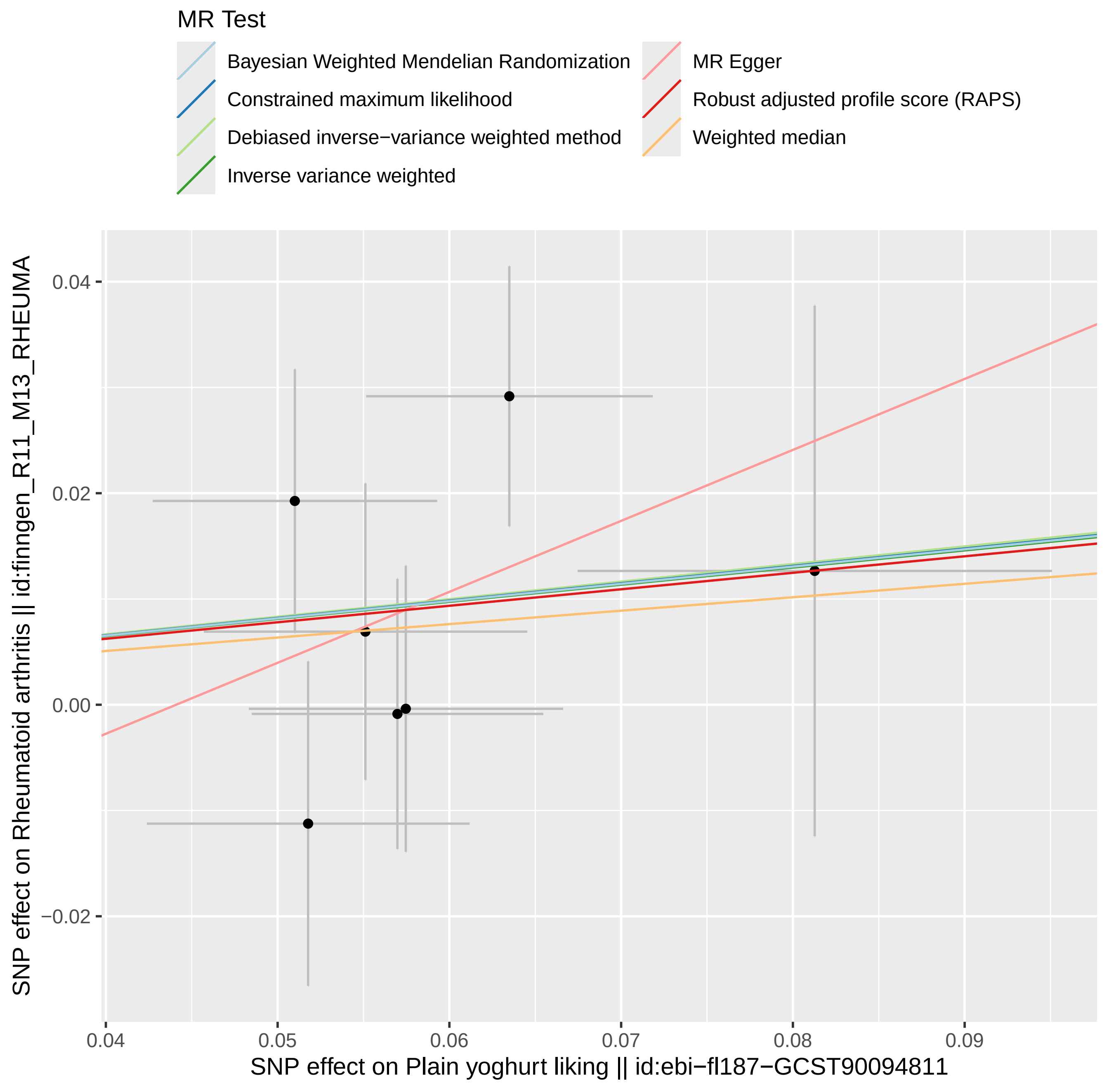
MR Test
Bayesian Weighted Mendelian Randomization
Constrained maximum likelihood
Debiased inverse−variance weighted method
Inverse variance weighted
MR Egger
Robust adjusted profile score (RAPS)
Weighted median
SNP effect on Rheumatoid arthritis || id:finngen_R11_M13_RHEUMA
SNP effect on Plain yoghurt liking || id:ebi−fl187−GCST90094811
0.04
0.02
0.00
−0.02
0.04
0.05
0.06
0.07
0.08
0.09

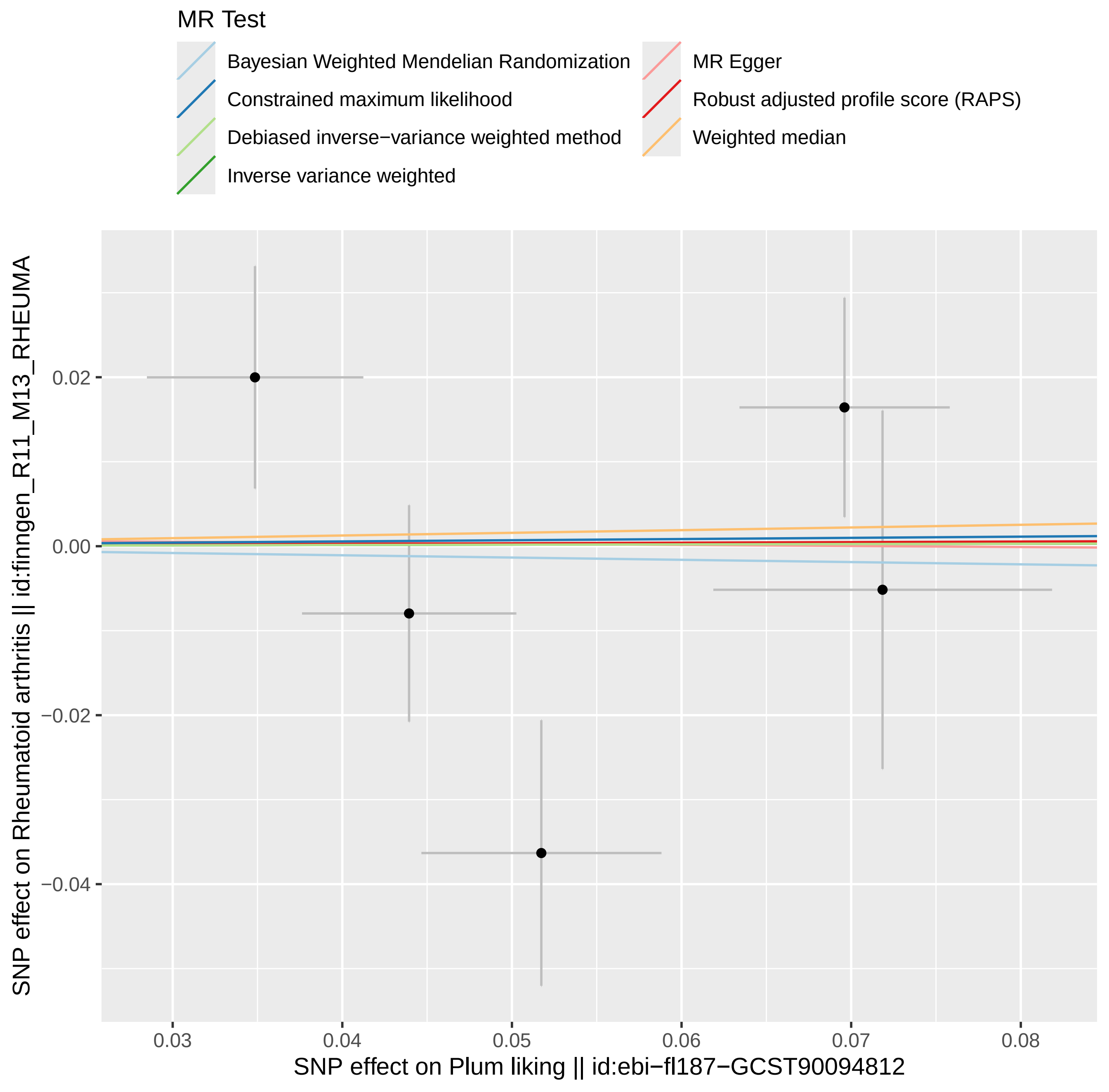
MR Test
Bayesian Weighted Mendelian Randomization
Constrained maximum likelihood
Debiased inverse−variance weighted method
Inverse variance weighted
MR Egger
Robust adjusted profile score (RAPS)
Weighted median
0.02
0.00
−0.02
−0.04
0.03
0.04
0.05
0.06
0.07
0.08
SNP effect on Rheumatoid arthritis || id:finngen_R11_M13_RHEUMA
SNP effect on Plum liking || id:ebi−fl187−GCST90094812

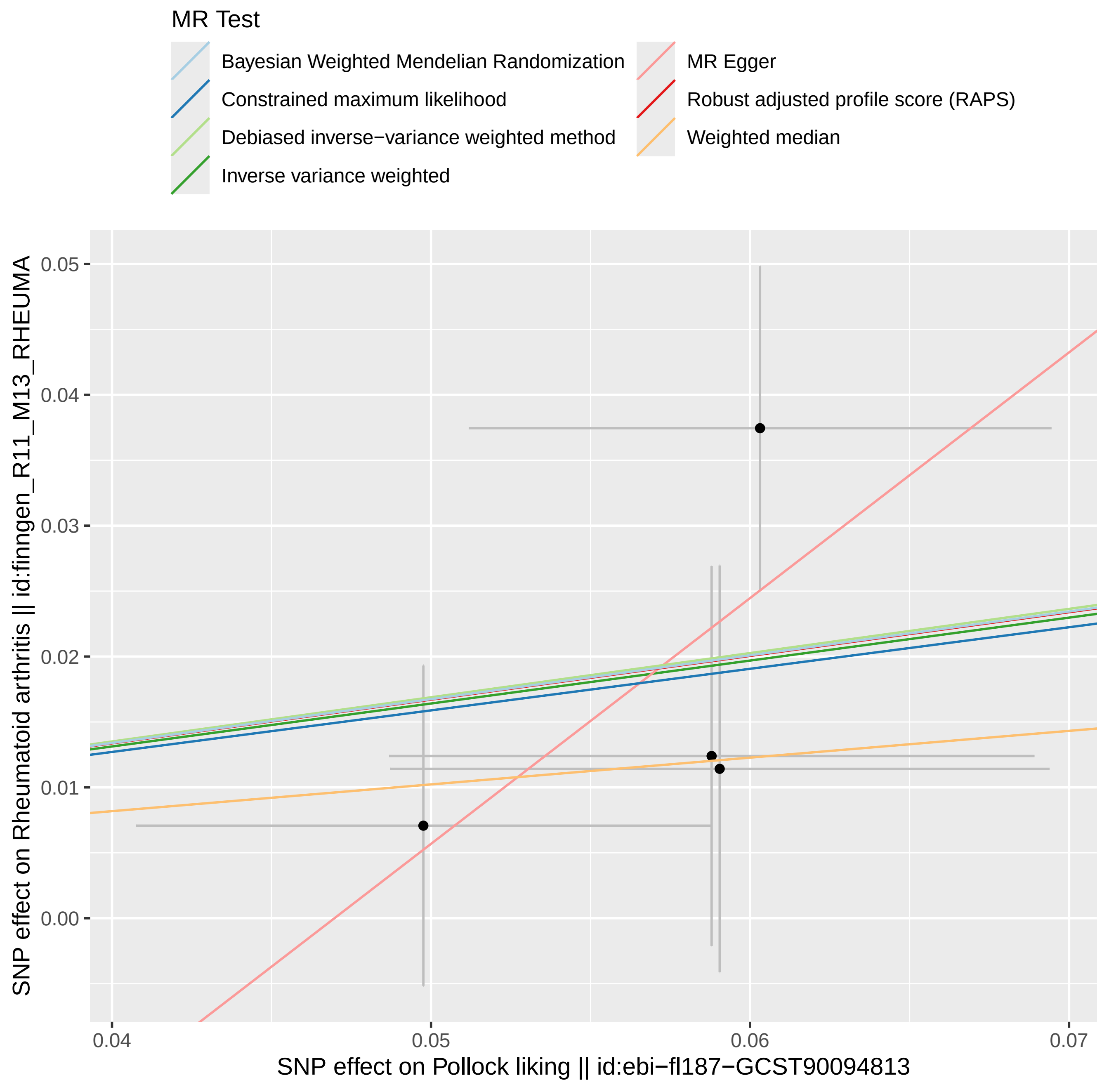
MR Test
Bayesian Weighted Mendelian Randomization
Constrained maximum likelihood
Debiased inverse−variance weighted method
Inverse variance weighted
MR Egger
Robust adjusted profile score (RAPS)
Weighted median
0.05
0.04
0.03
0.02
0.01
0.00
0.04
0.05
0.06
0.07
SNP effect on Rheumatoid arthritis || id:finngen_R11_M13_RHEUMA
SNP effect on Pollock liking || id:ebi−fl187−GCST90094813

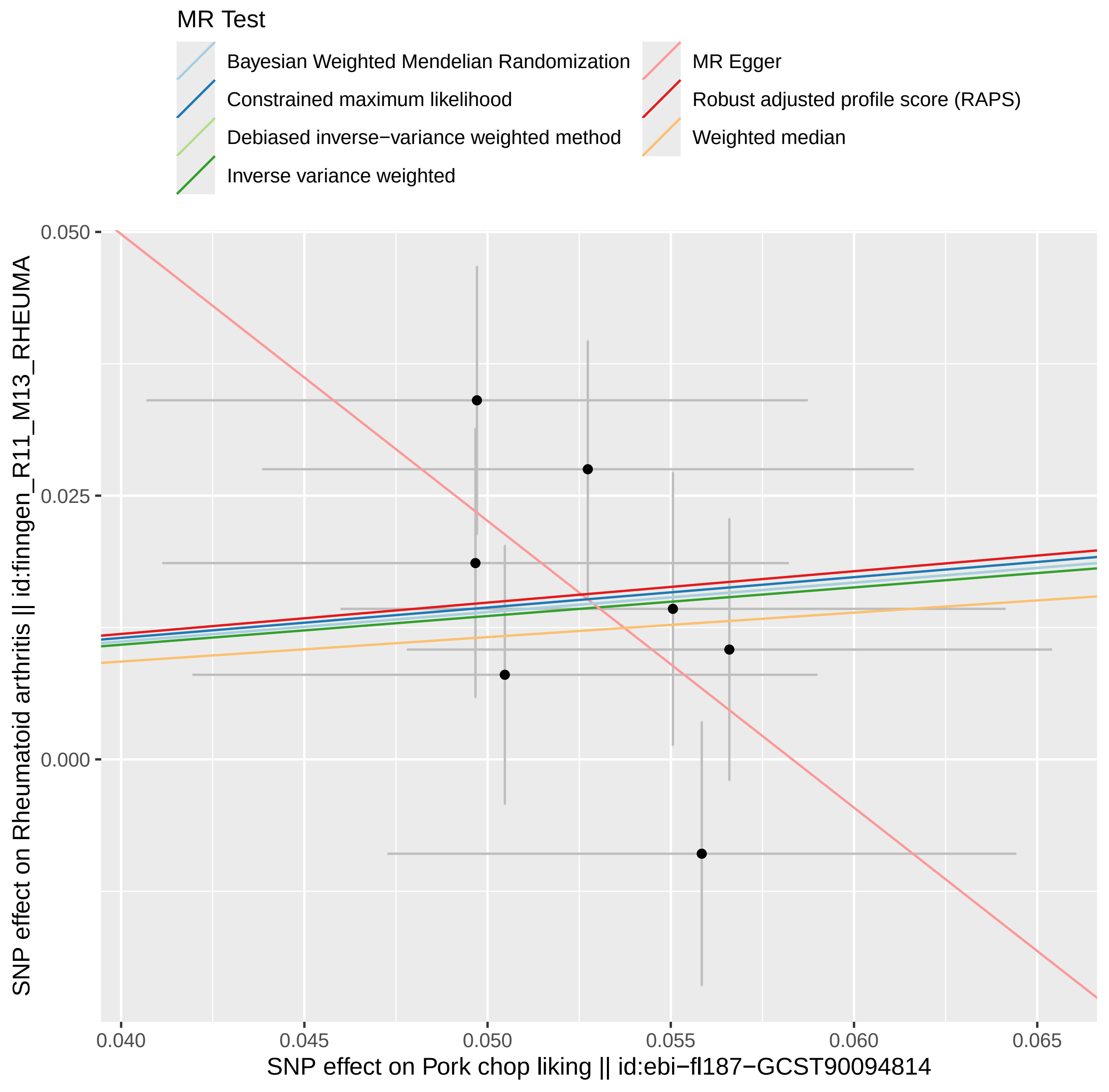
MR Test
Bayesian Weighted Mendelian Randomization
Constrained maximum likelihood
Debiased inverse−variance weighted method
Inverse variance weighted
MR Egger
Robust adjusted profile score (RAPS)
Weighted median
0.050
0.025
0.000
0.040
0.045
0.050
0.055
0.060
0.065
SNP effect on Rheumatoid arthritis || id:finngen_R11_M13_RHEUMA
SNP effect on Pork chop liking || id:ebi−fl187−GCST90094814

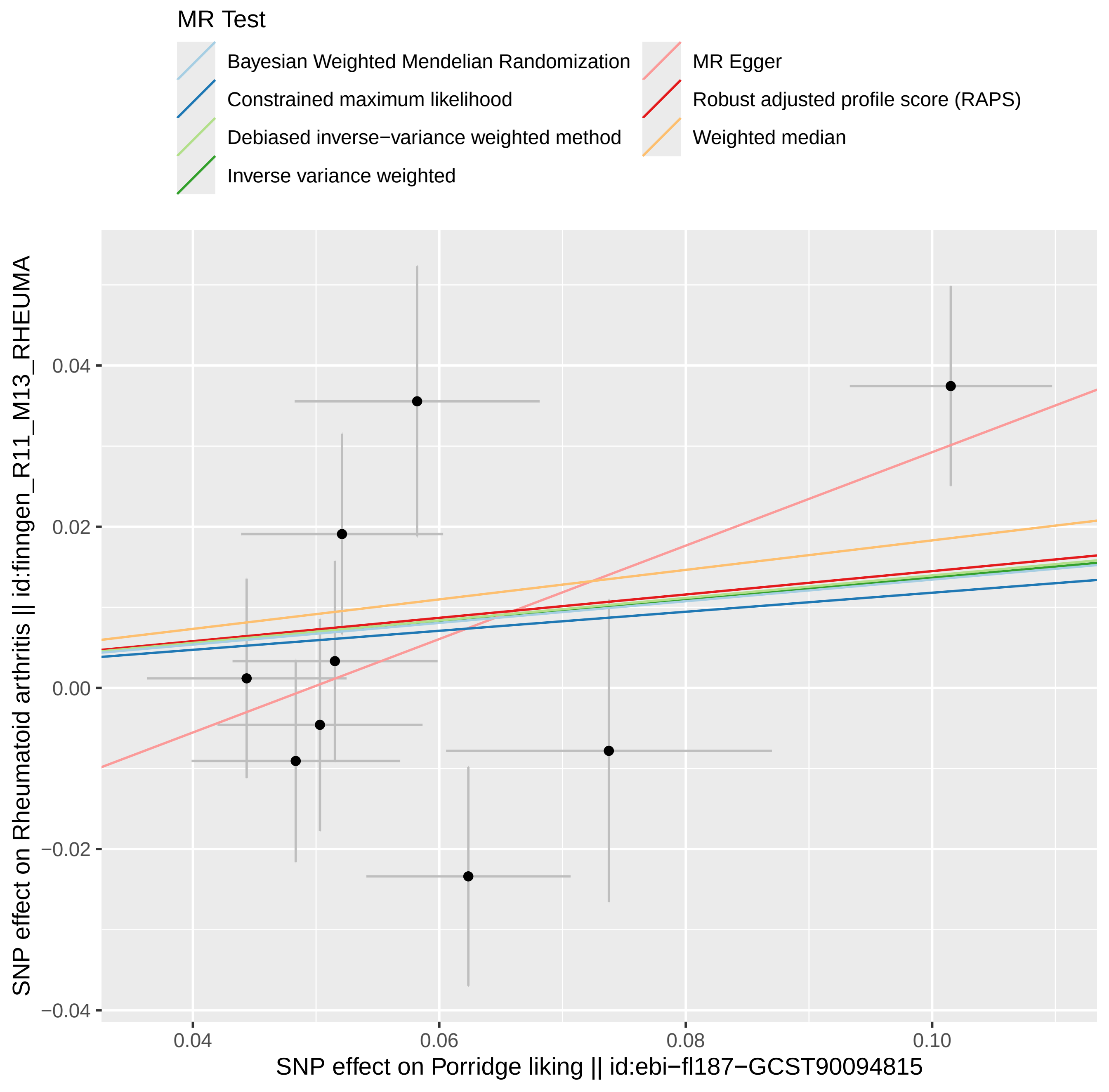
MR Test
Bayesian Weighted Mendelian Randomization
Constrained maximum likelihood
Debiased inverse−variance weighted method
Inverse variance weighted
MR Egger
Robust adjusted profile score (RAPS)
Weighted median
0.04
0.02
0.00
−0.02
−0.04
0.04
0.06
0.08
0.10
SNP effect on Rheumatoid arthritis || id:finngen_R11_M13_RHEUMA
SNP effect on Porridge liking || id:ebi−fl187−GCST90094815

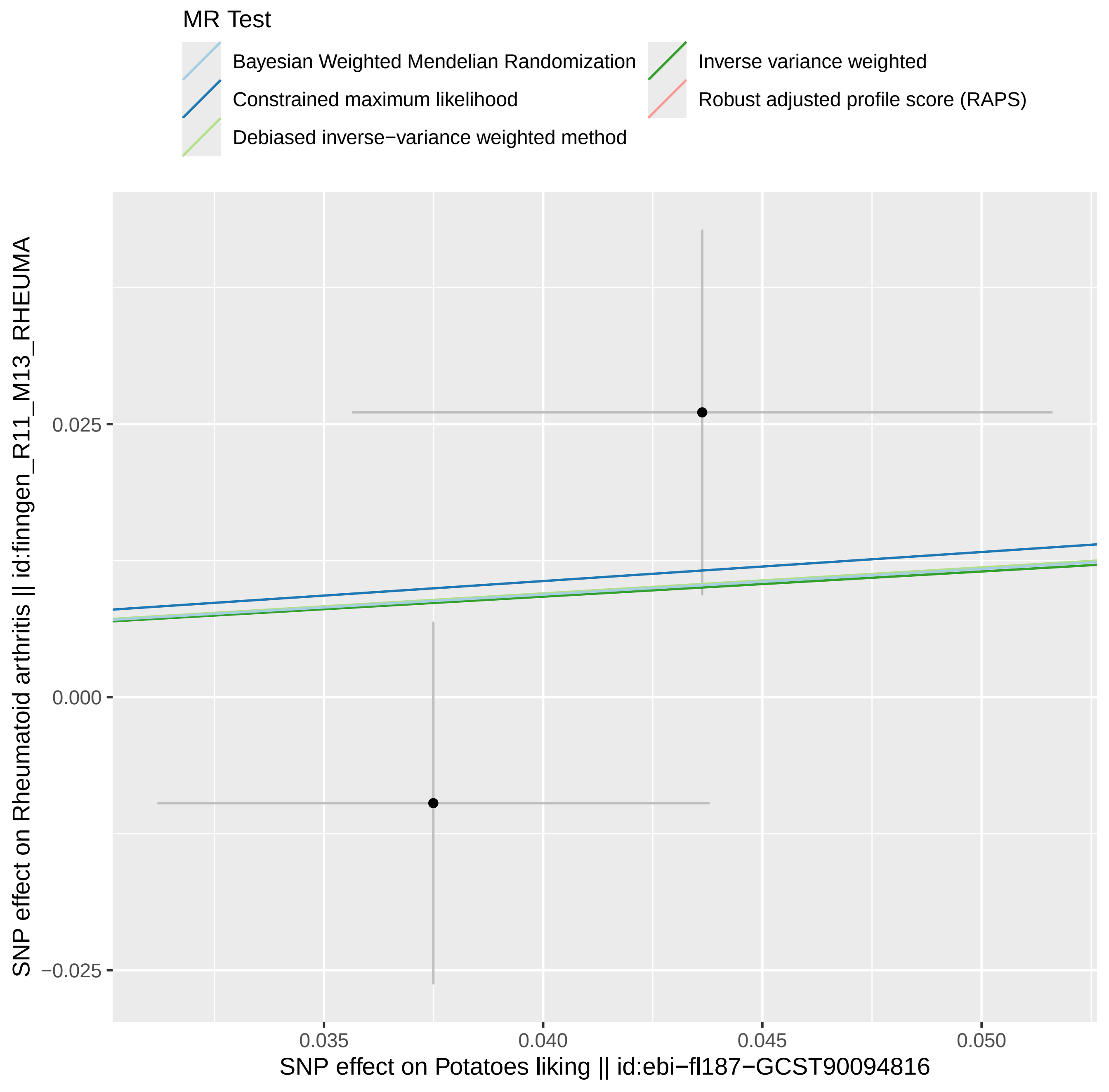
MR Test
Bayesian Weighted Mendelian Randomization
Constrained maximum likelihood
Debiased inverse−variance weighted method
Inverse variance weighted
Robust adjusted profile score (RAPS)
SNP effect on Rheumatoid arthritis || id:finngen_R11_M13_RHEUMA
0.025
0.000
−0.025
0.035
0.040
0.045
0.050
SNP effect on Potatoes liking || id:ebi−fl187−GCST90094816

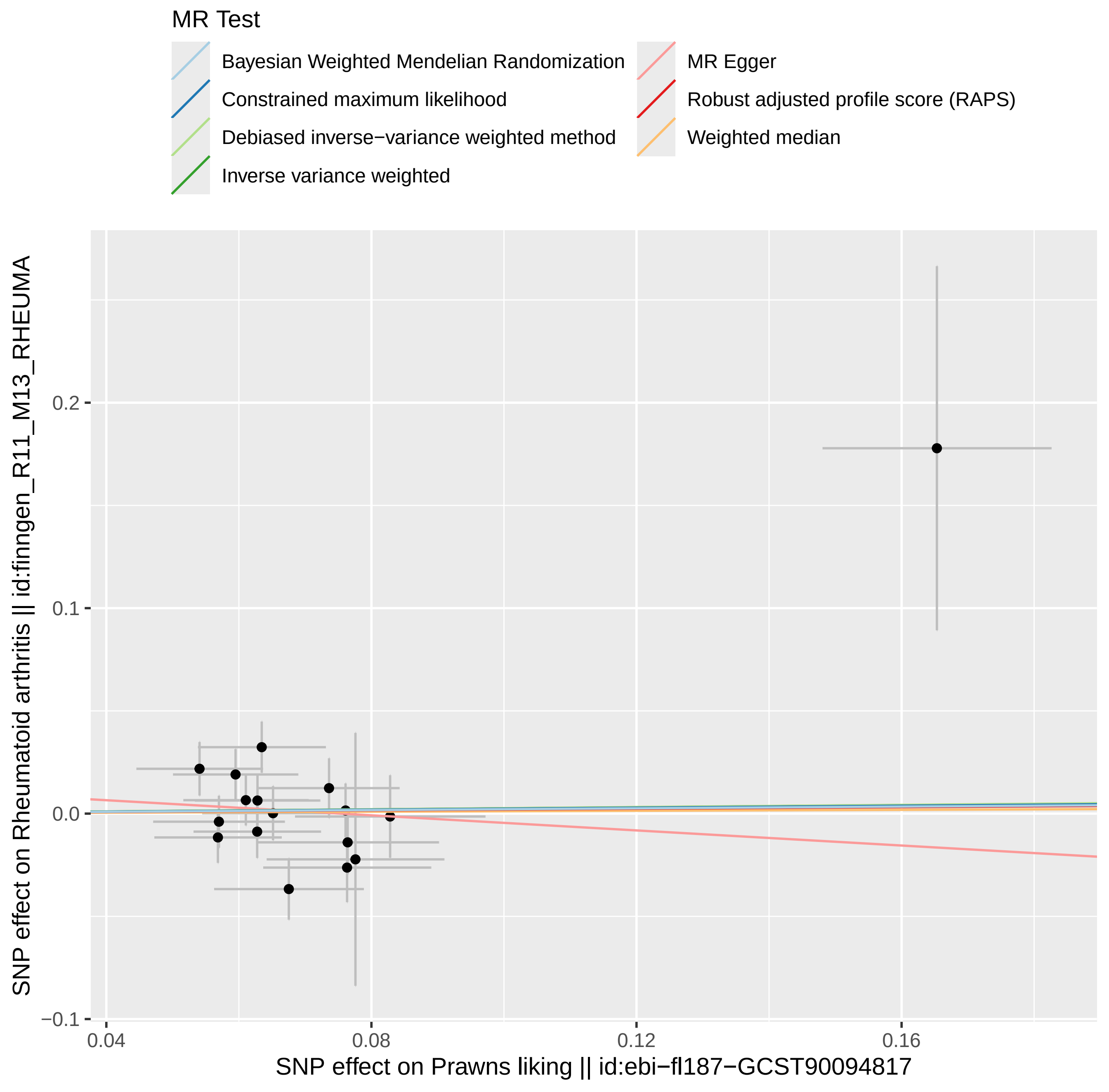
MR Test
Bayesian Weighted Mendelian Randomization
Constrained maximum likelihood
Debiased inverse−variance weighted method
Inverse variance weighted
MR Egger
Robust adjusted profile score (RAPS)
Weighted median
SNP effect on Rheumatoid arthritis || id:finngen_R11_M13_RHEUMA
0.2
0.1
0.0
−0.1
0.04
0.08
0.12
0.16
SNP effect on Prawns liking || id:ebi−fl187−GCST90094817

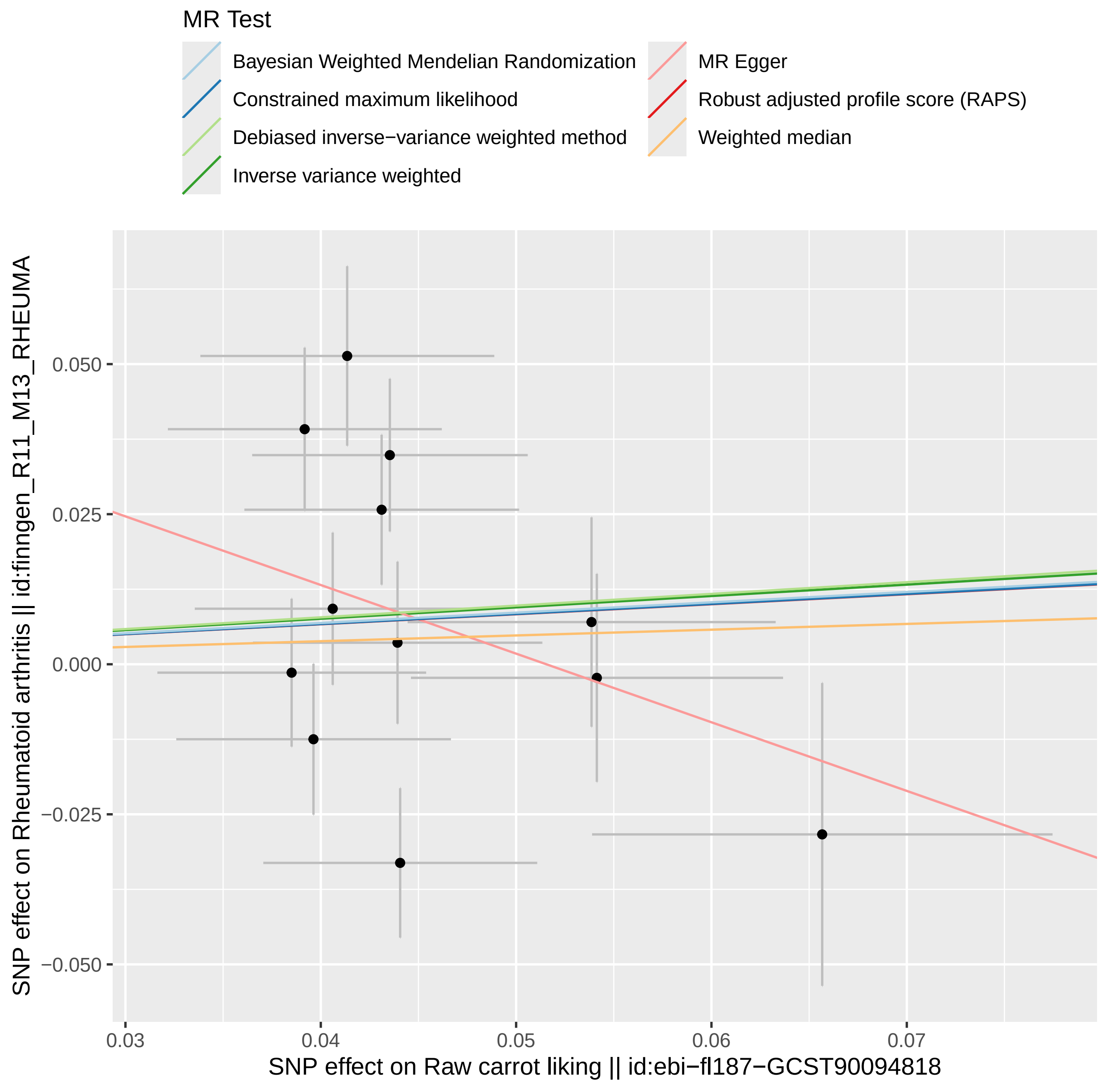
MR Test
Bayesian Weighted Mendelian Randomization
Constrained maximum likelihood
Debiased inverse−variance weighted method
Inverse variance weighted
MR Egger
Robust adjusted profile score (RAPS)
Weighted median
SNP effect on Rheumatoid arthritis || id:finngen_R11_M13_RHEUMA
0.050
0.025
0.000
−0.025
−0.050
0.03
0.04
0.05
0.06
0.07
SNP effect on Raw carrot liking || id:ebi−fl187−GCST90094818

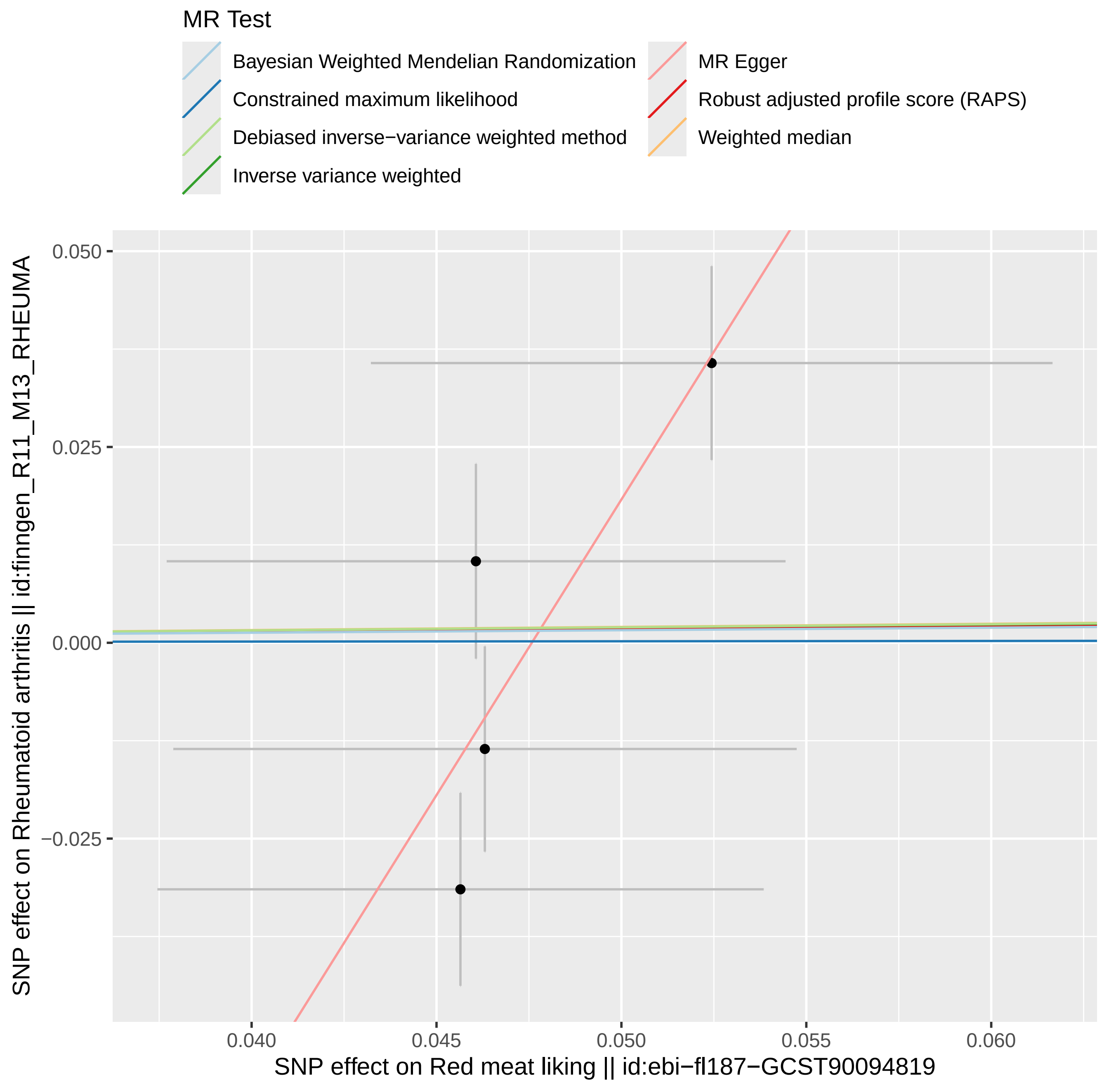

MR Test
Bayesian Weighted Mendelian Randomization
Constrained maximum likelihood
Debiased inverse−variance weighted method
Inverse variance weighted
MR Egger
Robust adjusted profile score (RAPS)
Weighted median
0.050
0.025
0.000
−0.025
0.040
0.045
0.050
0.055
0.060
SNP effect on Rheumatoid arthritis || id:finngen_R11_M13_RHEUMA
SNP effect on Red meat liking || id:ebi−fl187−GCST90094819

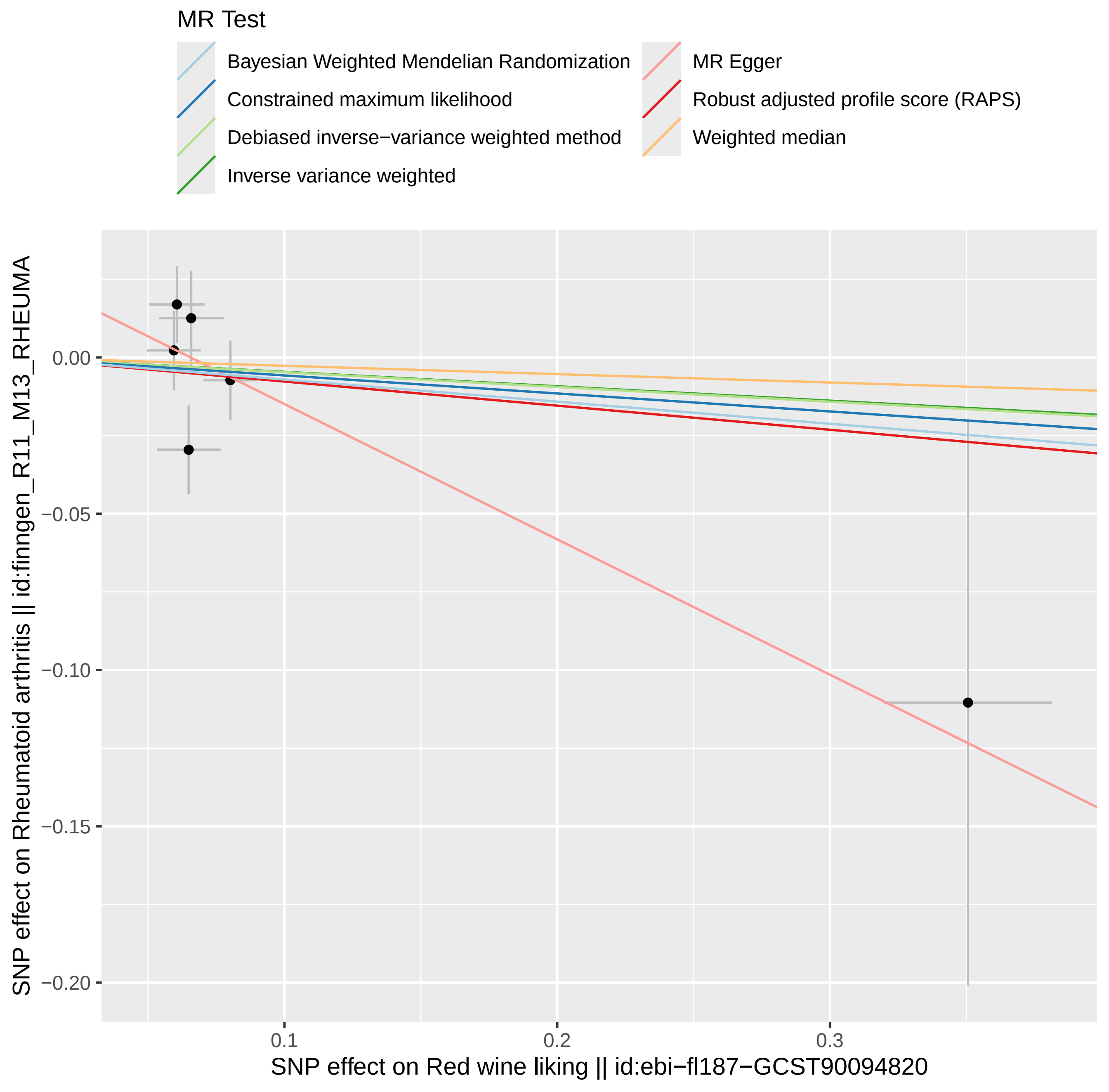
MR Test
Bayesian Weighted Mendelian Randomization
Constrained maximum likelihood
Debiased inverse−variance weighted method
Inverse variance weighted
MR Egger
Robust adjusted profile score (RAPS)
Weighted median
0.00
−0.05
−0.10
−0.15
−0.20
0.1
0.2
0.3
SNP effect on Rheumatoid arthritis || id:finngen_R11_M13_RHEUMA
SNP effect on Red wine liking || id:ebi−fI187−GCST90094820

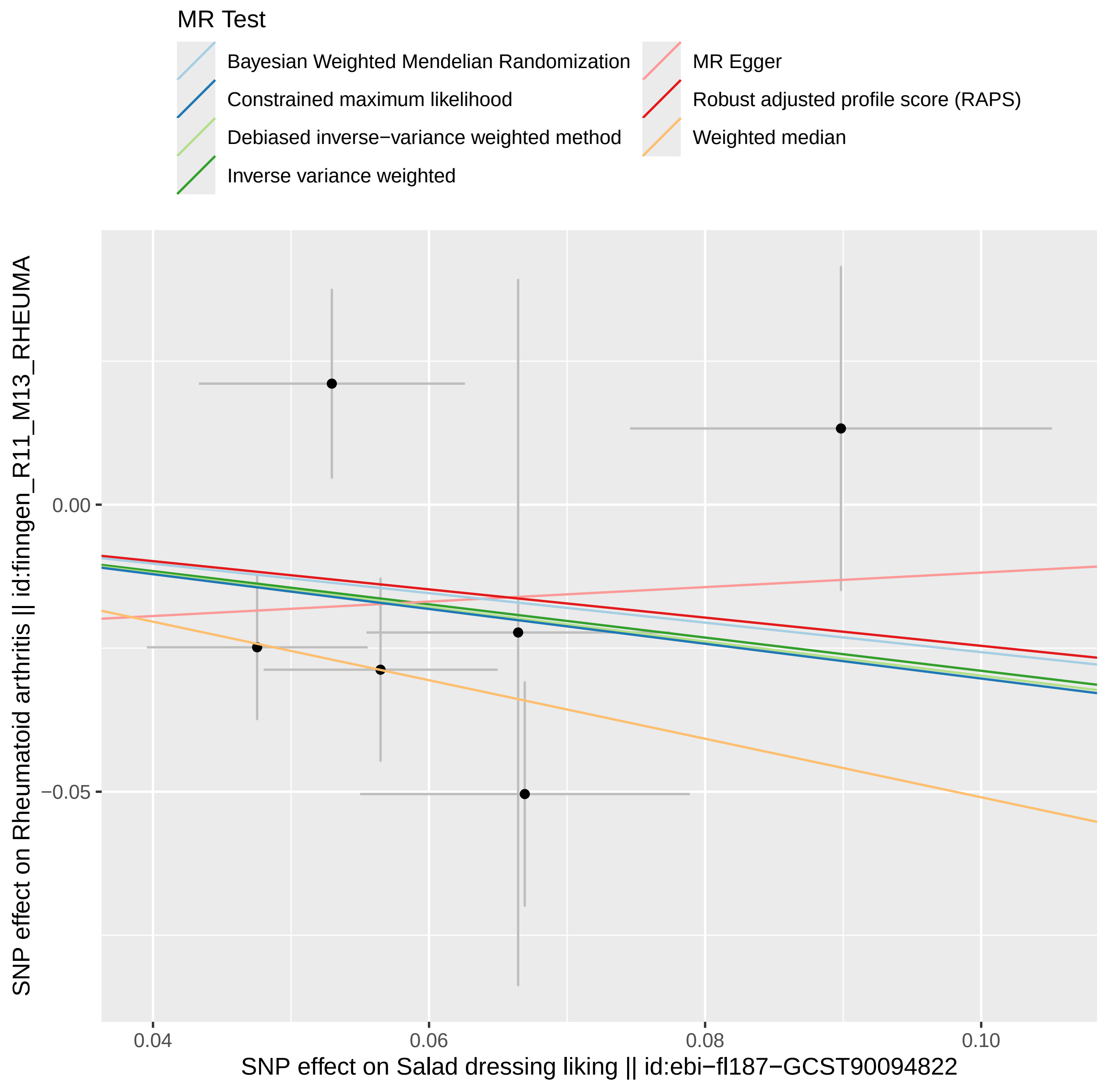
MR Test
Bayesian Weighted Mendelian Randomization
Constrained maximum likelihood
Debiased inverse−variance weighted method
Inverse variance weighted
MR Egger
Robust adjusted profile score (RAPS)
Weighted median
0.00
−0.05
0.04
0.06
0.08
0.10
SNP effect on Rheumatoid arthritis || id:finngen_R11_M13_RHEUMA
SNP effect on Salad dressing liking || id:ebi−fl187−GCST90094822

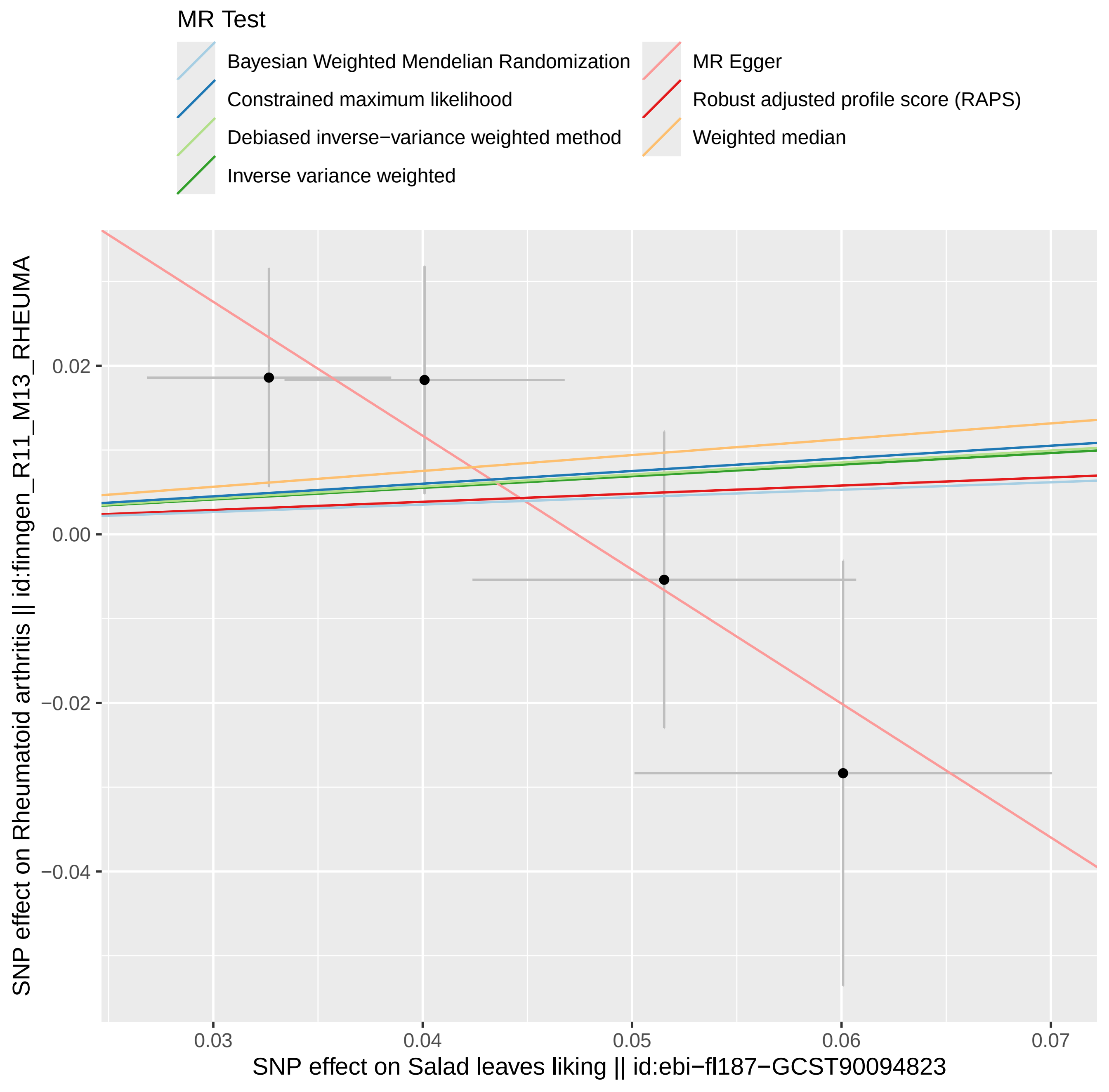
MR Test
Bayesian Weighted Mendelian Randomization
Constrained maximum likelihood
Debiased inverse−variance weighted method
Inverse variance weighted
MR Egger
Robust adjusted profile score (RAPS)
Weighted median
0.02
0.00
−0.02
−0.04
0.03
0.04
0.05
0.06
0.07
SNP effect on Rheumatoid arthritis || id:finngen_R11_M13_RHEUMA
SNP effect on Salad leaves liking || id:ebi−fl187−GCST90094823

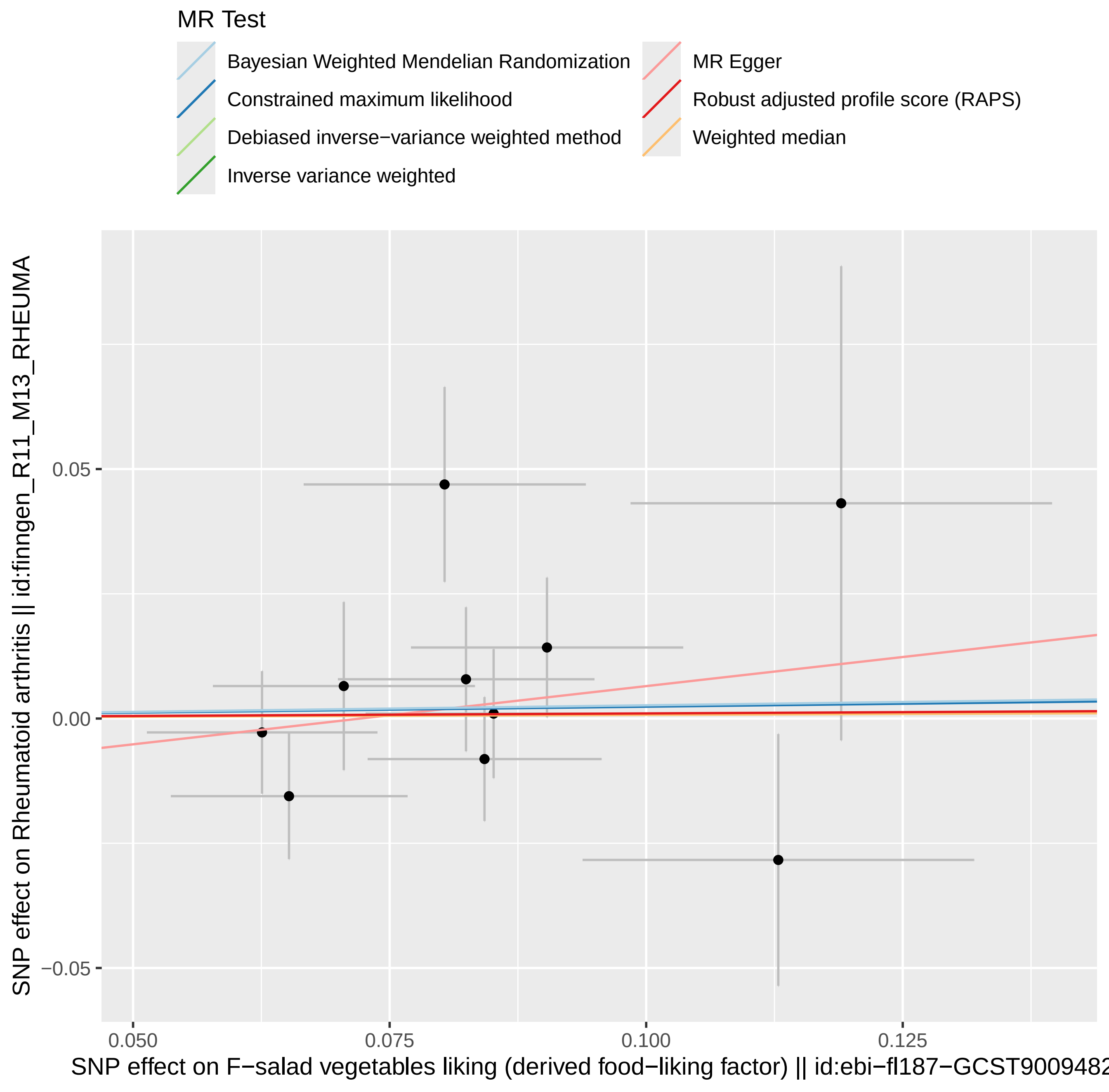

MR Test
Bayesian Weighted Mendelian Randomization
Constrained maximum likelihood
Debiased inverse−variance weighted method
Inverse variance weighted
MR Egger
Robust adjusted profile score (RAPS)
Weighted median
SNP effect on Rheumatoid arthritis || id:finngen_R11_M13_RHEUMA
0.05
0.00
−0.05
0.050
0.075
0.100
0.125
SNP effect on F−salad vegetables liking (derived food−liking factor) || id:ebi−fl187−GCST9009482

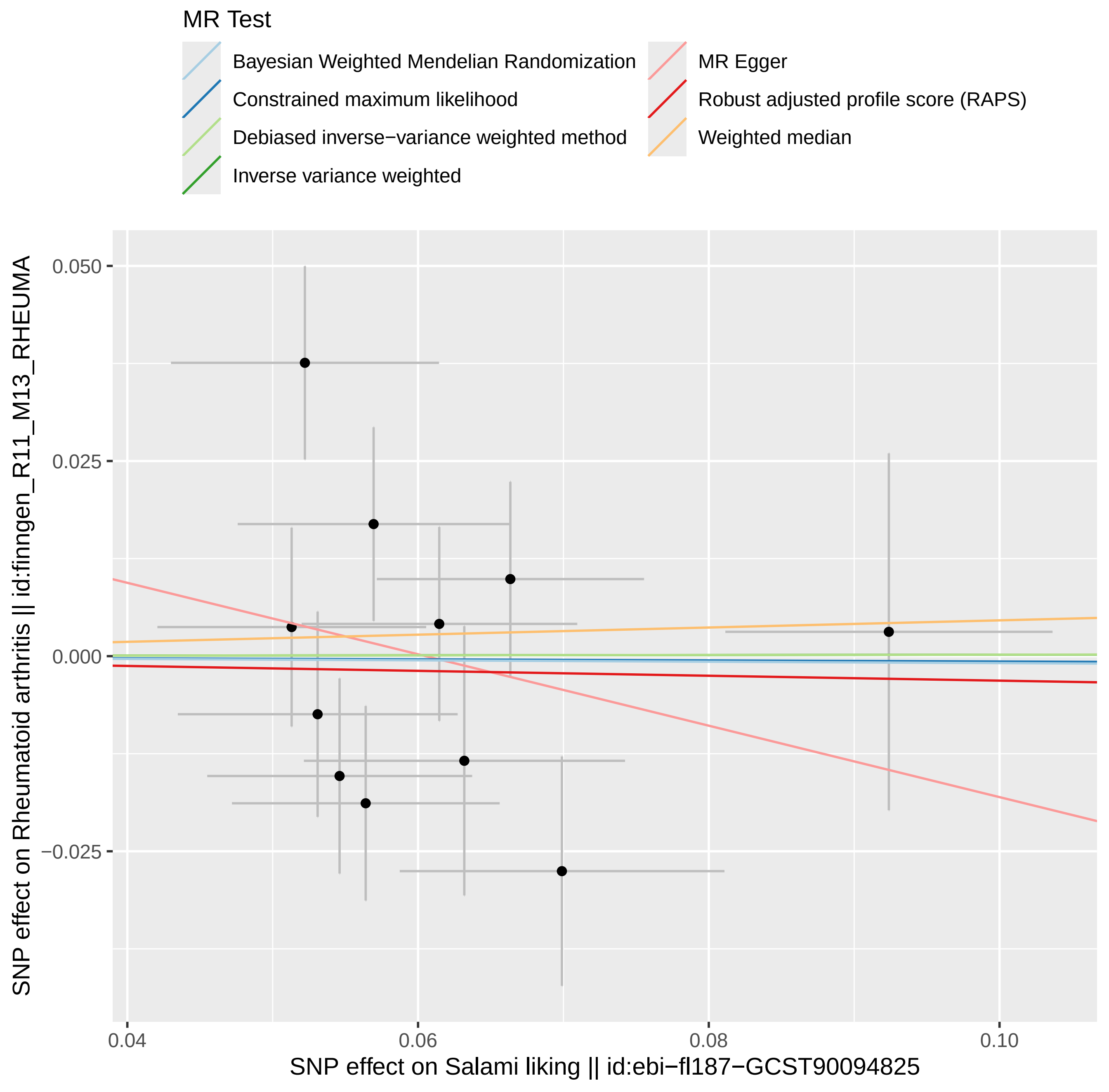

MR Test
Bayesian Weighted Mendelian Randomization
Constrained maximum likelihood
Debiased inverse−variance weighted method
Inverse variance weighted
MR Egger
Robust adjusted profile score (RAPS)
Weighted median
0.050
0.025
0.000
−0.025
0.04
0.06
0.08
0.10
SNP effect on Rheumatoid arthritis || id:finngen_R11_M13_RHEUMA
SNP effect on Salami liking || id:ebi−fl187−GCST90094825

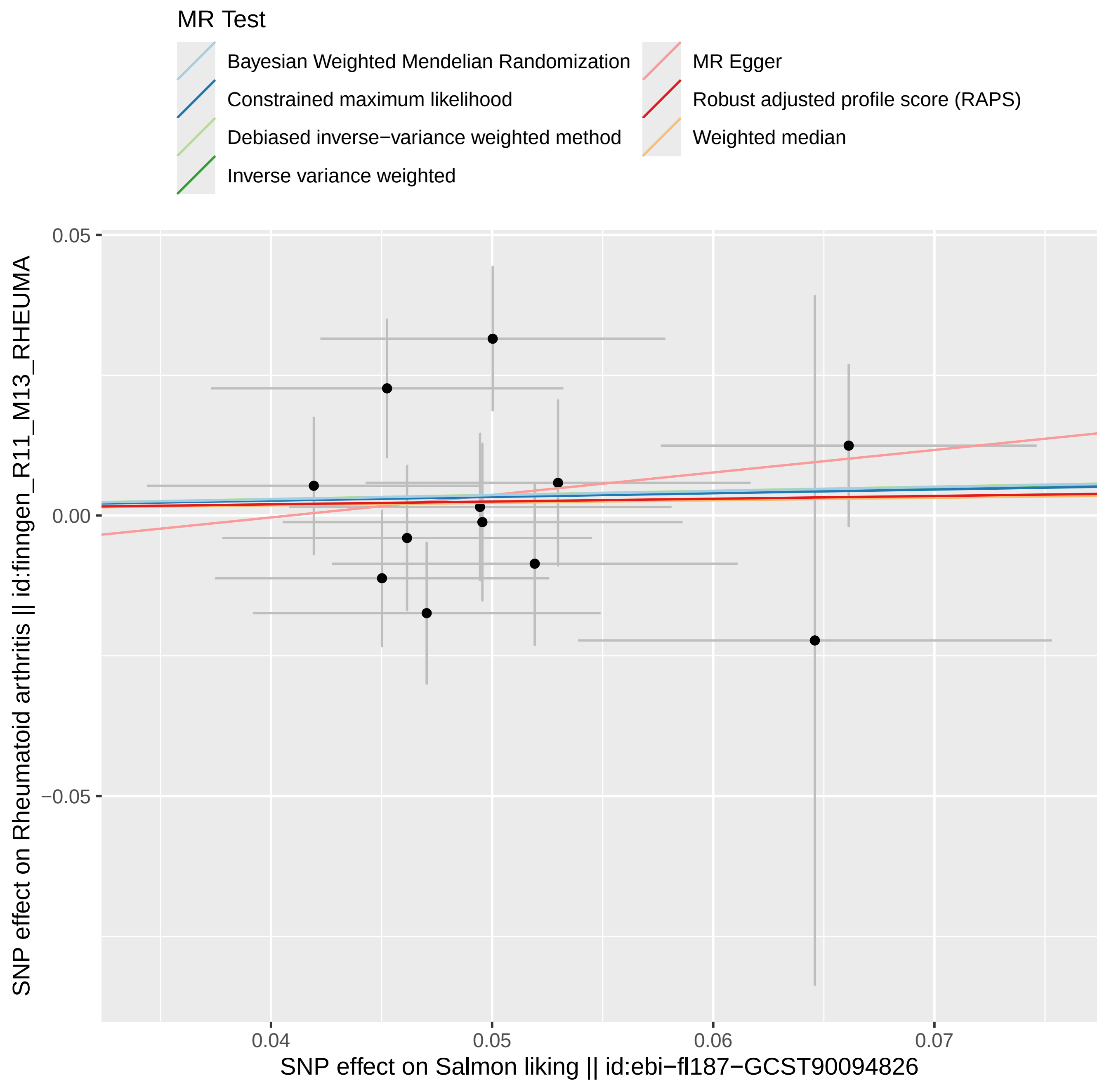

MR Test
Bayesian Weighted Mendelian Randomization
Constrained maximum likelihood
Debiased inverse−variance weighted method
Inverse variance weighted
MR Egger
Robust adjusted profile score (RAPS)
Weighted median
0.05
0.00
−0.05
0.04
0.05
0.06
0.07
SNP effect on Rheumatoid arthritis || id:finngen_R11_M13_RHEUMA
SNP effect on Salmon liking || id:ebi−fl187−GCST90094826

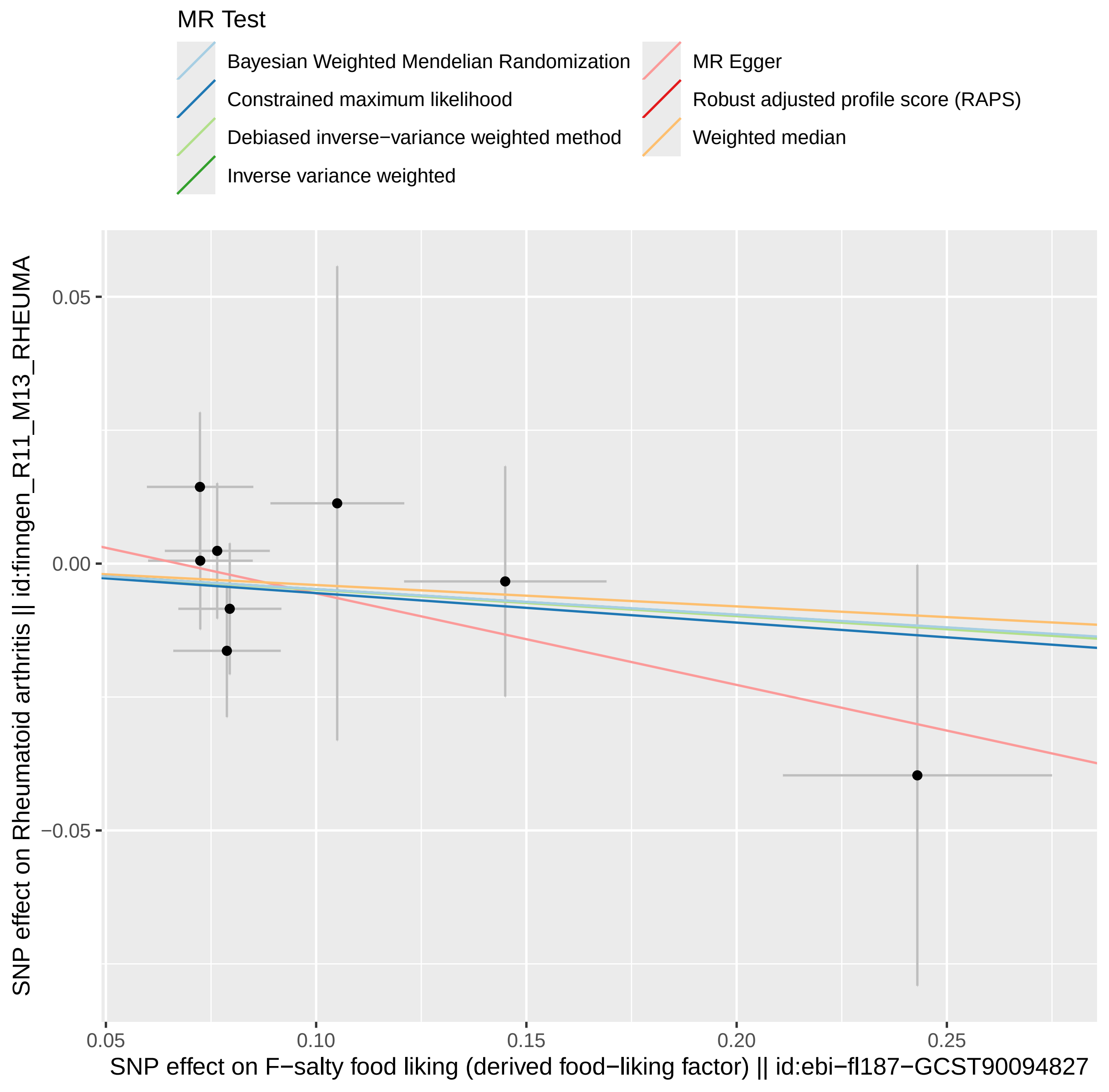
MR Test
Bayesian Weighted Mendelian Randomization
Constrained maximum likelihood
Debiased inverse−variance weighted method
Inverse variance weighted
MR Egger
Robust adjusted profile score (RAPS)
Weighted median
SNP effect on Rheumatoid arthritis || id:finngen_R11_M13_RHEUMA
SNP effect on F−salty food liking (derived food−liking factor) || id:ebi−fl187−GCST90094827
0.05
0.00
−0.05
0.05
0.10
0.15
0.20
0.25

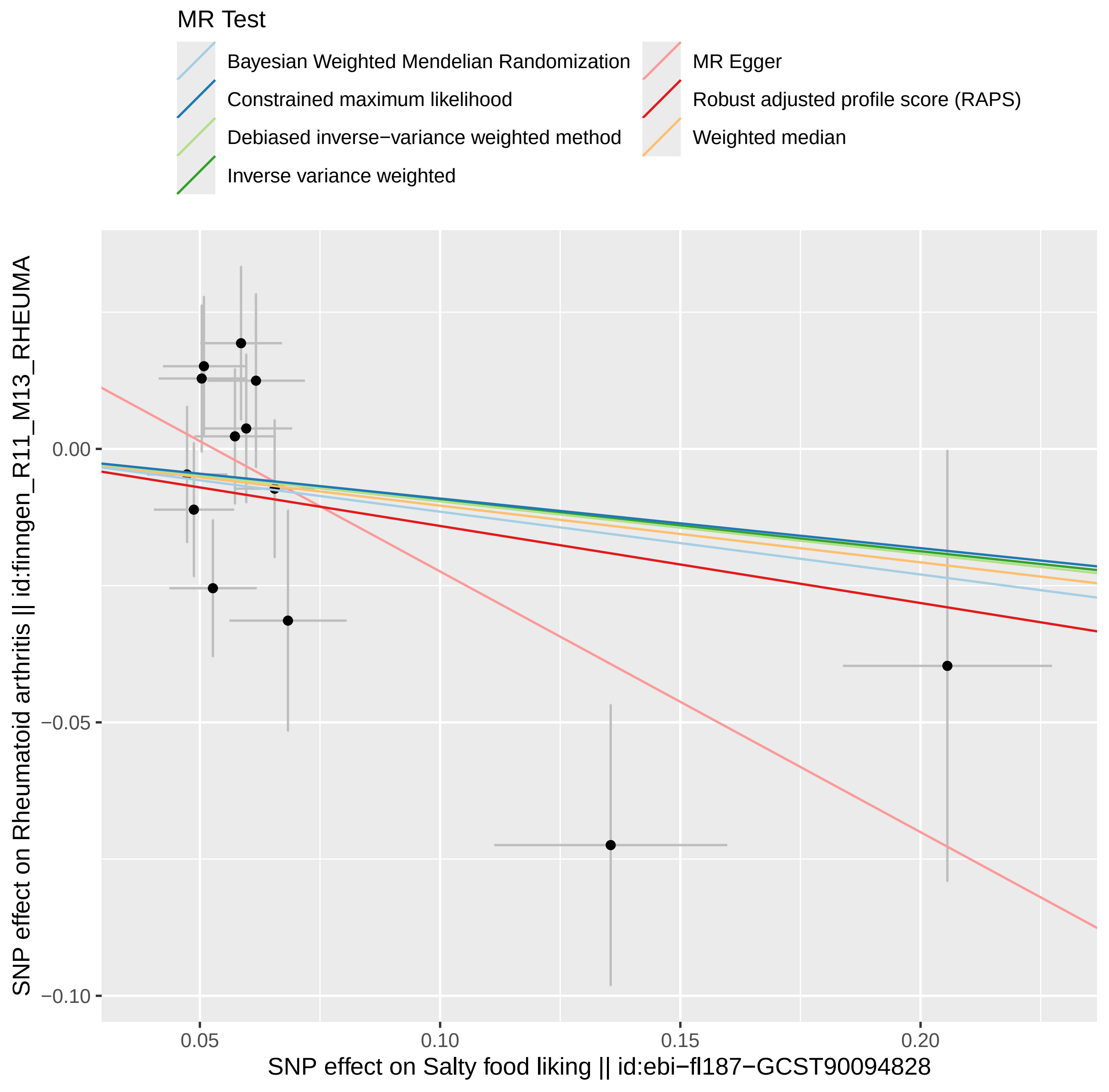

MR Test
Bayesian Weighted Mendelian Randomization
Constrained maximum likelihood
Debiased inverse−variance weighted method
Inverse variance weighted
MR Egger
Robust adjusted profile score (RAPS)
Weighted median
0.00
−0.05
−0.10
0.05
0.10
0.15
0.20
SNP effect on Rheumatoid arthritis || id:finngen_R11_M13_RHEUMA
SNP effect on Salty food liking || id:ebi−fl187−GCST90094828

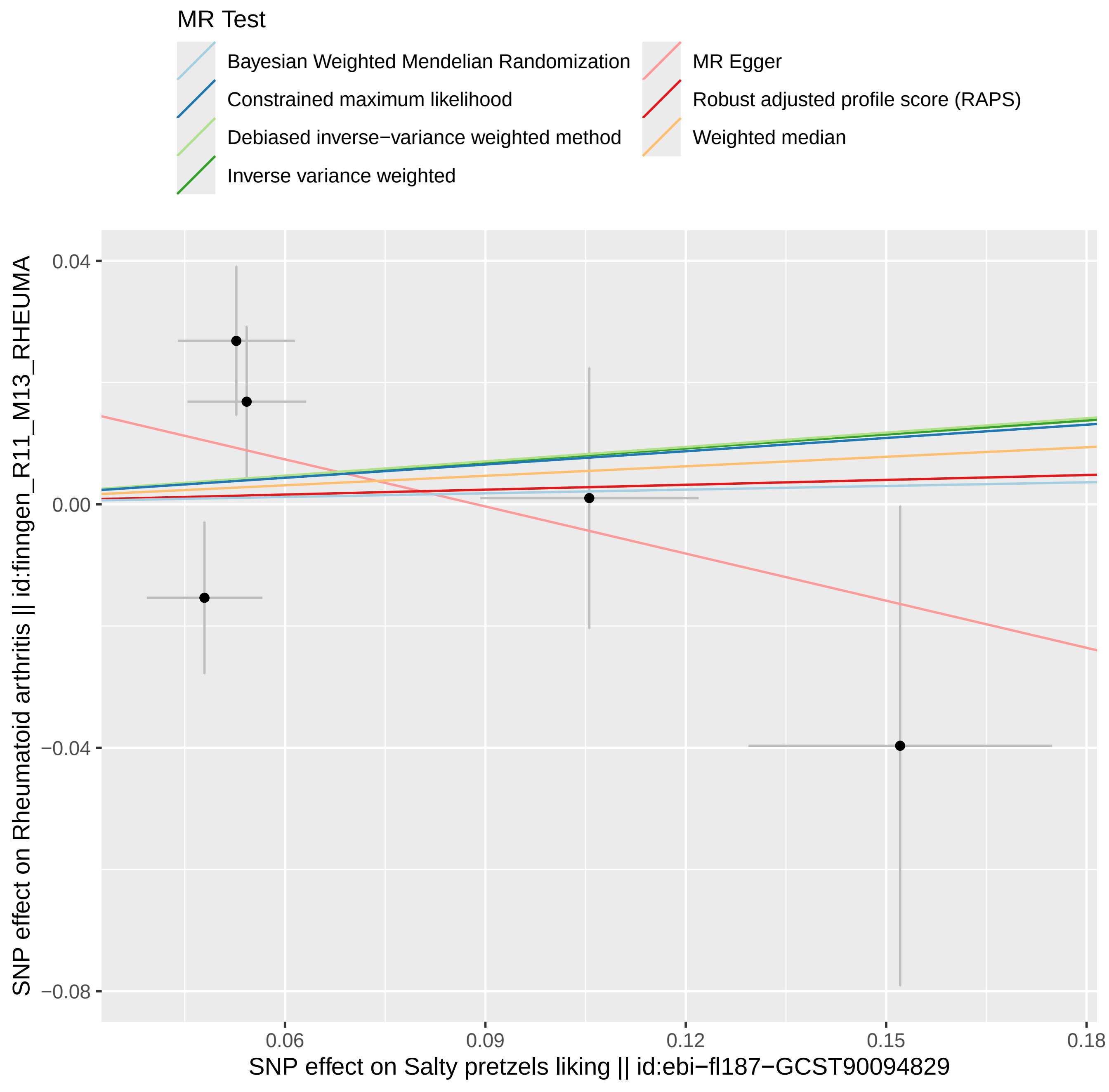

MR Test
Bayesian Weighted Mendelian Randomization
Constrained maximum likelihood
Debiased inverse−variance weighted method
Inverse variance weighted
MR Egger
Robust adjusted profile score (RAPS)
Weighted median
0.04
0.00
−0.04
−0.08
0.06
0.09
0.12
0.15
0.18
SNP effect on Rheumatoid arthritis || id:finngen_R11_M13_RHEUMA
SNP effect on Salty pretzels liking || id:ebi−fl187−GCST90094829

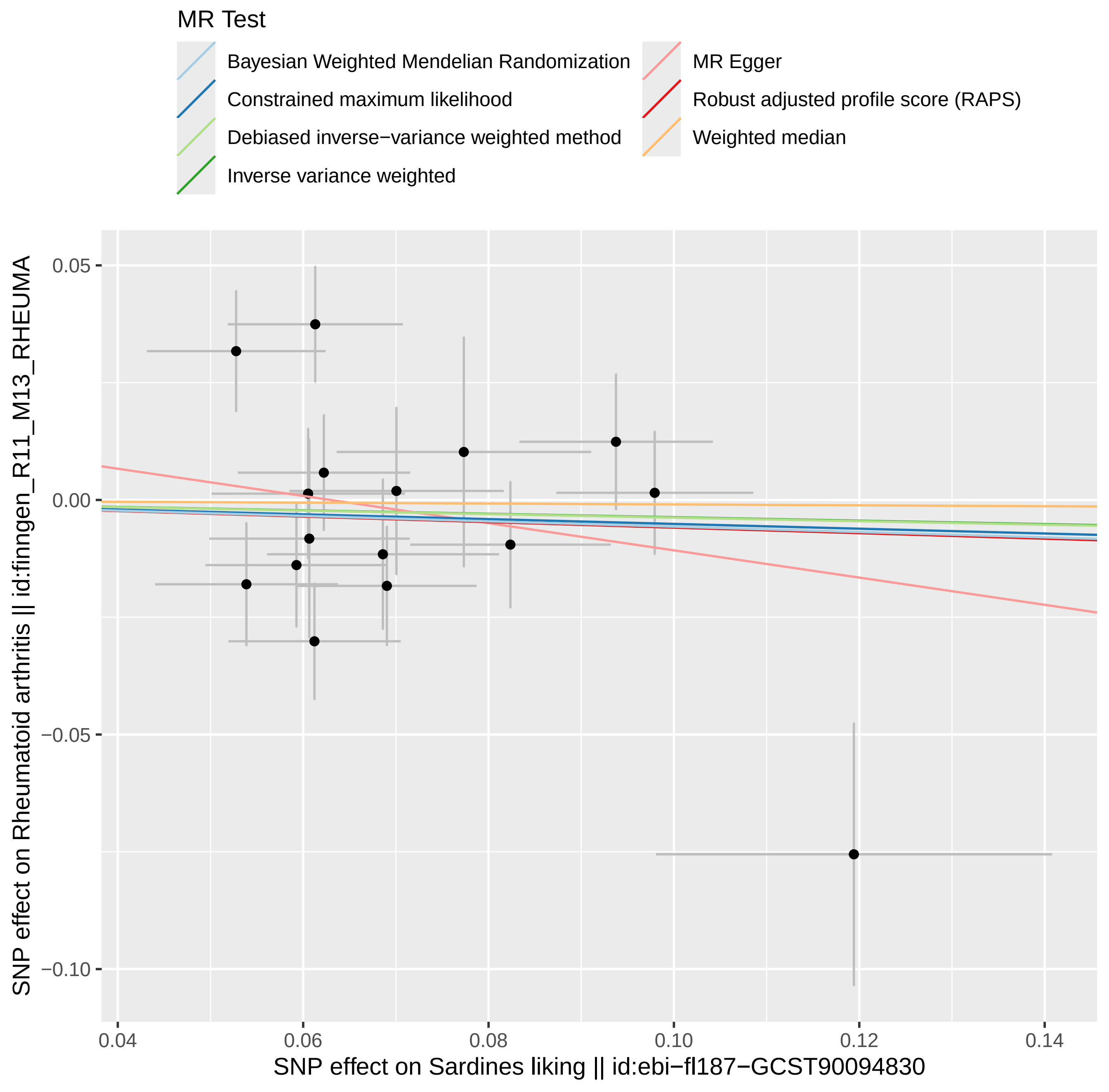

MR Test
Bayesian Weighted Mendelian Randomization
Constrained maximum likelihood
Debiased inverse−variance weighted method
Inverse variance weighted
MR Egger
Robust adjusted profile score (RAPS)
Weighted median
0.05
0.00
−0.05
−0.10
0.04
0.06
0.08
0.10
0.12
0.14
SNP effect on Rheumatoid arthritis || id:finngen_R11_M13_RHEUMA
SNP effect on Sardines liking || id:ebi−fl187−GCST90094830

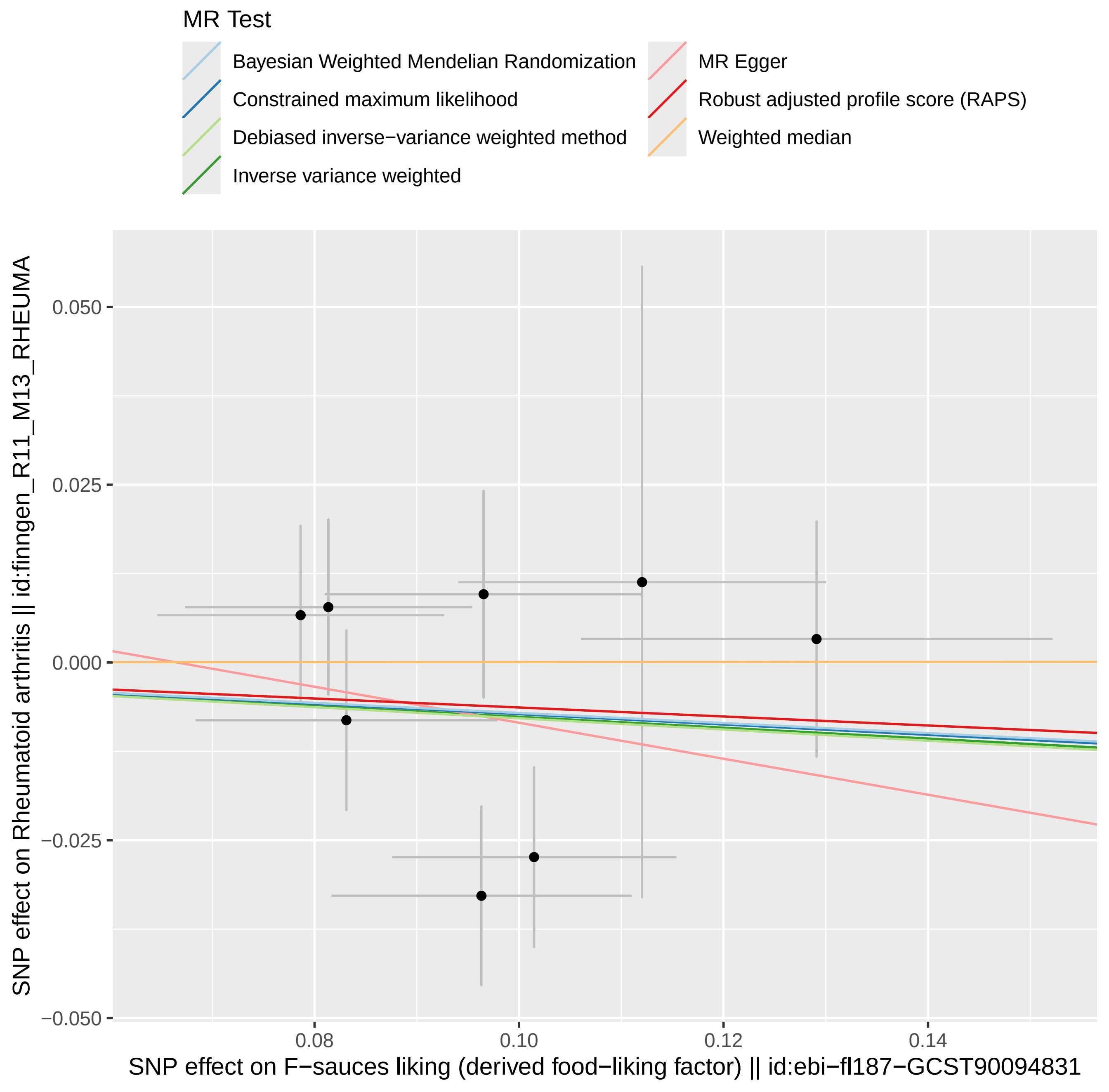
MR Test
Bayesian Weighted Mendelian Randomization
Constrained maximum likelihood
Debiased inverse−variance weighted method
Inverse variance weighted
MR Egger
Robust adjusted profile score (RAPS)
Weighted median
0.050
0.025
0.000
−0.025
−0.050
0.08
0.10
0.12
0.14
SNP effect on Rheumatoid arthritis || id:finngen_R11_M13_RHEUMA
SNP effect on F−sauces liking (derived food−liking factor) || id:ebi−fl187−GCST90094831

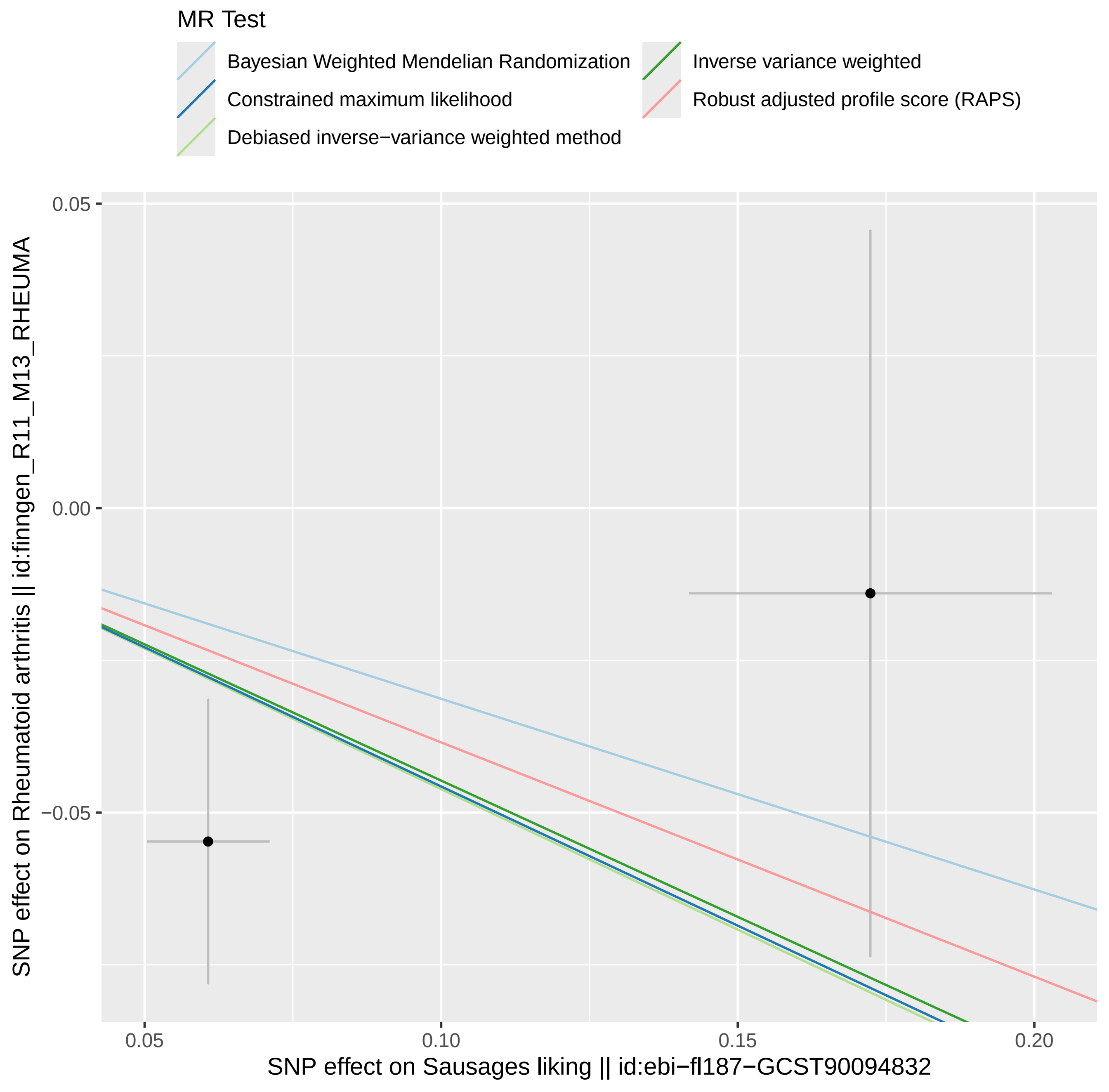
MR Test
Bayesian Weighted Mendelian Randomization
Constrained maximum likelihood
Debiased inverse−variance weighted method
Inverse variance weighted
Robust adjusted profile score (RAPS)
0.05
0.00
−0.05
0.05
0.10
0.15
0.20
SNP effect on Rheumatoid arthritis || id:finngen_R11_M13_RHEUMA
SNP effect on Sausages liking || id:ebi−fl187−GCST90094832

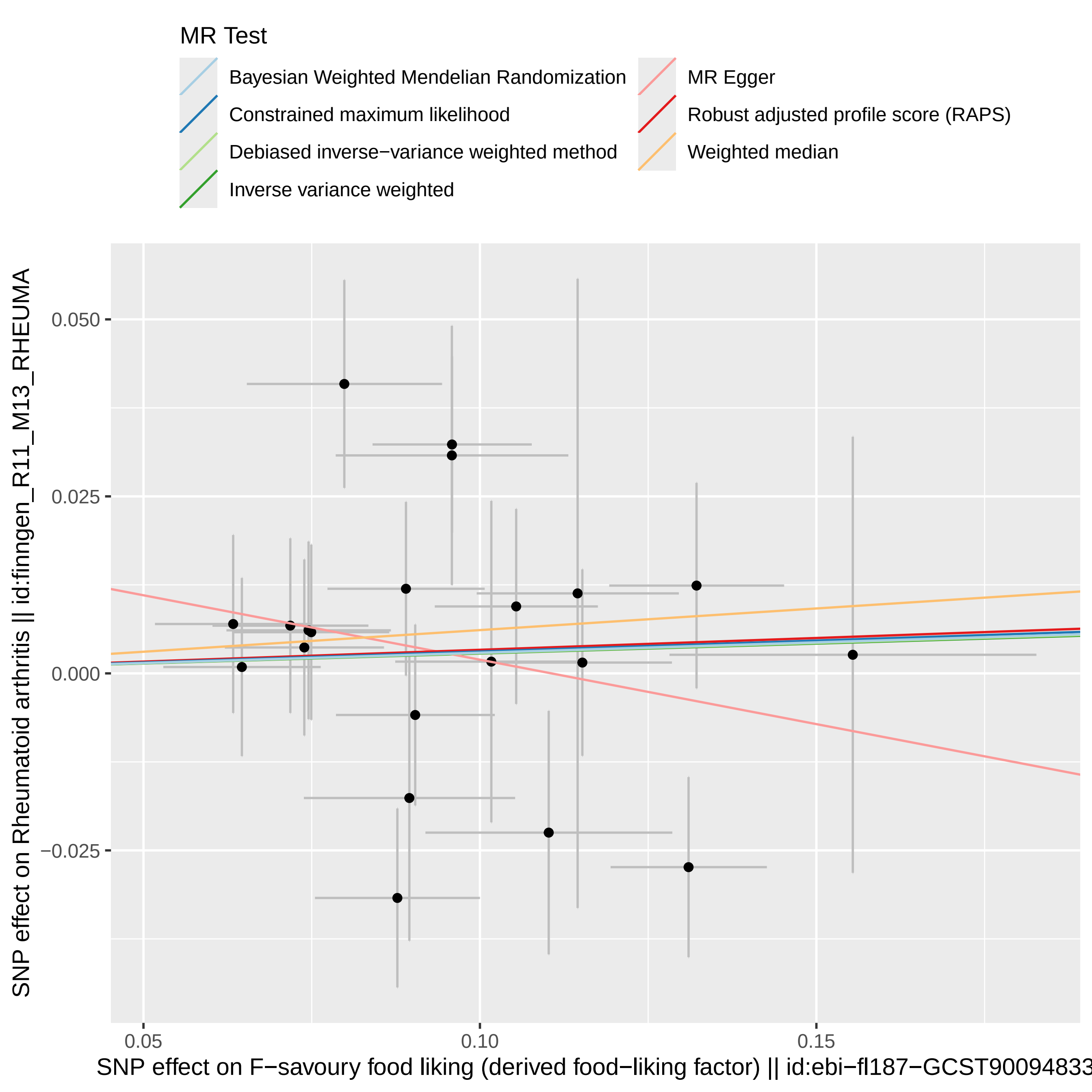
MR Test
Bayesian Weighted Mendelian Randomization
Constrained maximum likelihood
Debiased inverse−variance weighted method
Inverse variance weighted
MR Egger
Robust adjusted profile score (RAPS)
Weighted median
0.050
0.025
0.000
−0.025
0.05
0.10
0.15
SNP effect on Rheumatoid arthritis || id:finngen_R11_M13_RHEUMA
SNP effect on F−savoury food liking (derived food−liking factor) || id:ebi−fl187−GCST90094833

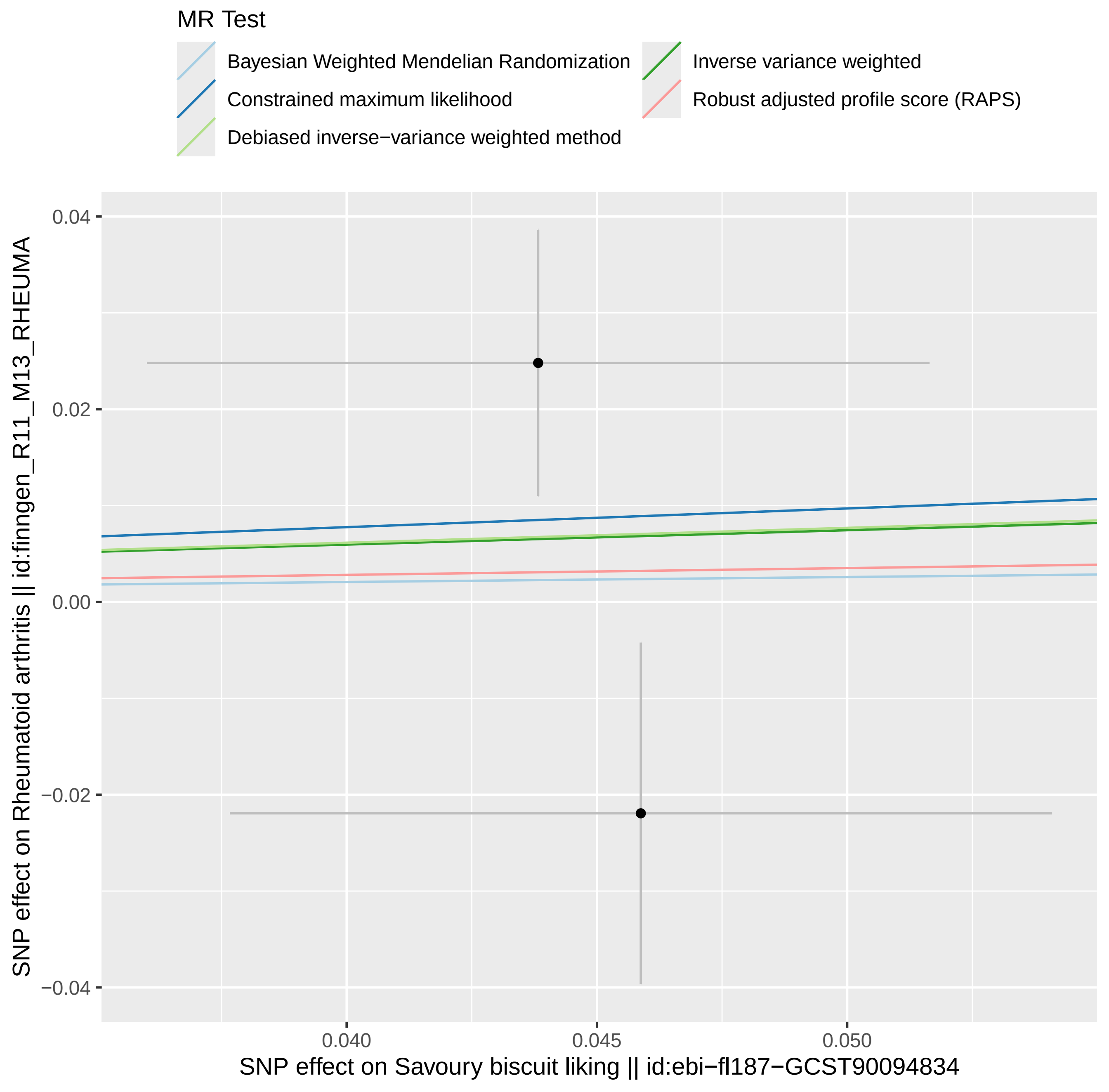
MR Test
Bayesian Weighted Mendelian Randomization
Constrained maximum likelihood
Debiased inverse−variance weighted method
Inverse variance weighted
Robust adjusted profile score (RAPS)
0.04
0.02
0.00
−0.02
−0.04
SNP effect on Rheumatoid arthritis || id:finngen_R11_M13_RHEUMA
0.040
0.045
0.050
SNP effect on Savoury biscuit liking || id:ebi−fl187−GCST90094834

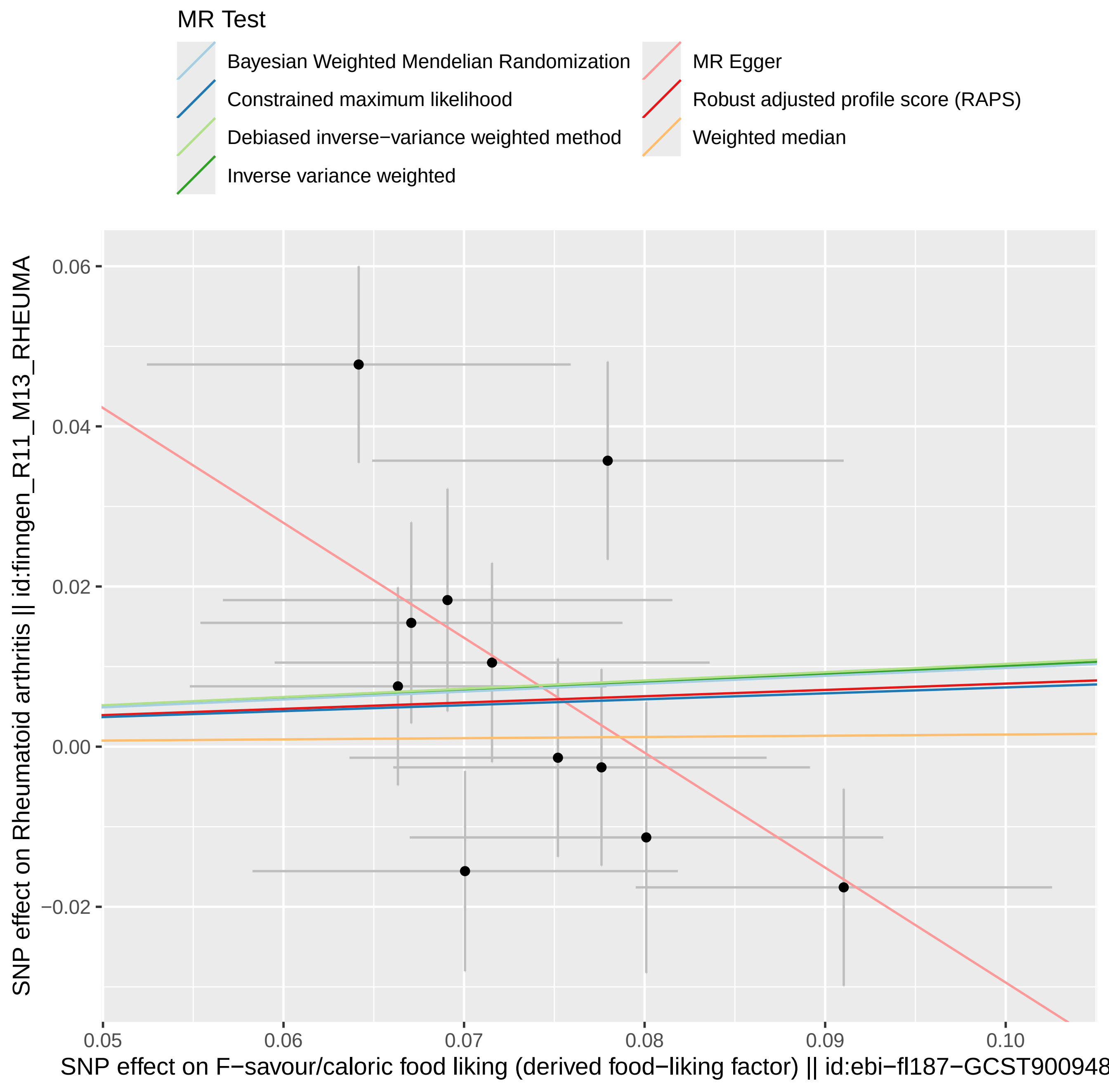

MR Test
Bayesian Weighted Mendelian Randomization
Constrained maximum likelihood
Debiased inverse−variance weighted method
Inverse variance weighted
MR Egger
Robust adjusted profile score (RAPS)
Weighted median
0.06
0.04
0.02
0.00
−0.02
0.05
0.06
0.07
0.08
0.09
0.10
SNP effect on Rheumatoid arthritis || id:finngen_R11_M13_RHEUMA
SNP effect on F−savour/caloric food liking (derived food−liking factor) || id:ebi−fl187−GCST900948

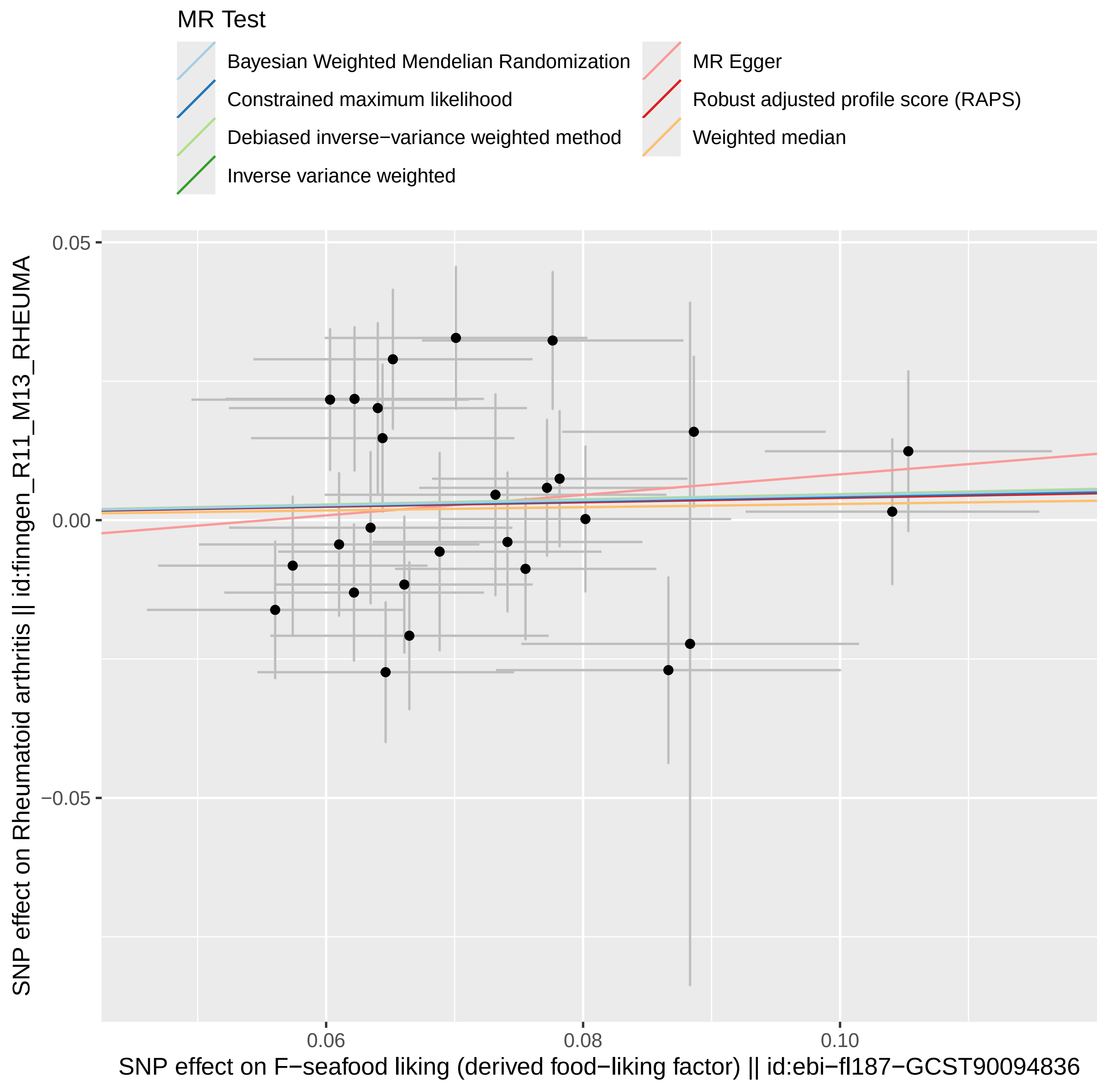
MR Test
Bayesian Weighted Mendelian Randomization
Constrained maximum likelihood
Debiased inverse−variance weighted method
Inverse variance weighted
MR Egger
Robust adjusted profile score (RAPS)
Weighted median
0.05
0.00
−0.05
0.06
0.08
0.10
SNP effect on Rheumatoid arthritis || id:finngen_R11_M13_RHEUMA
SNP effect on F−seafood liking (derived food−liking factor) || id:ebi−fl187−GCST90094836

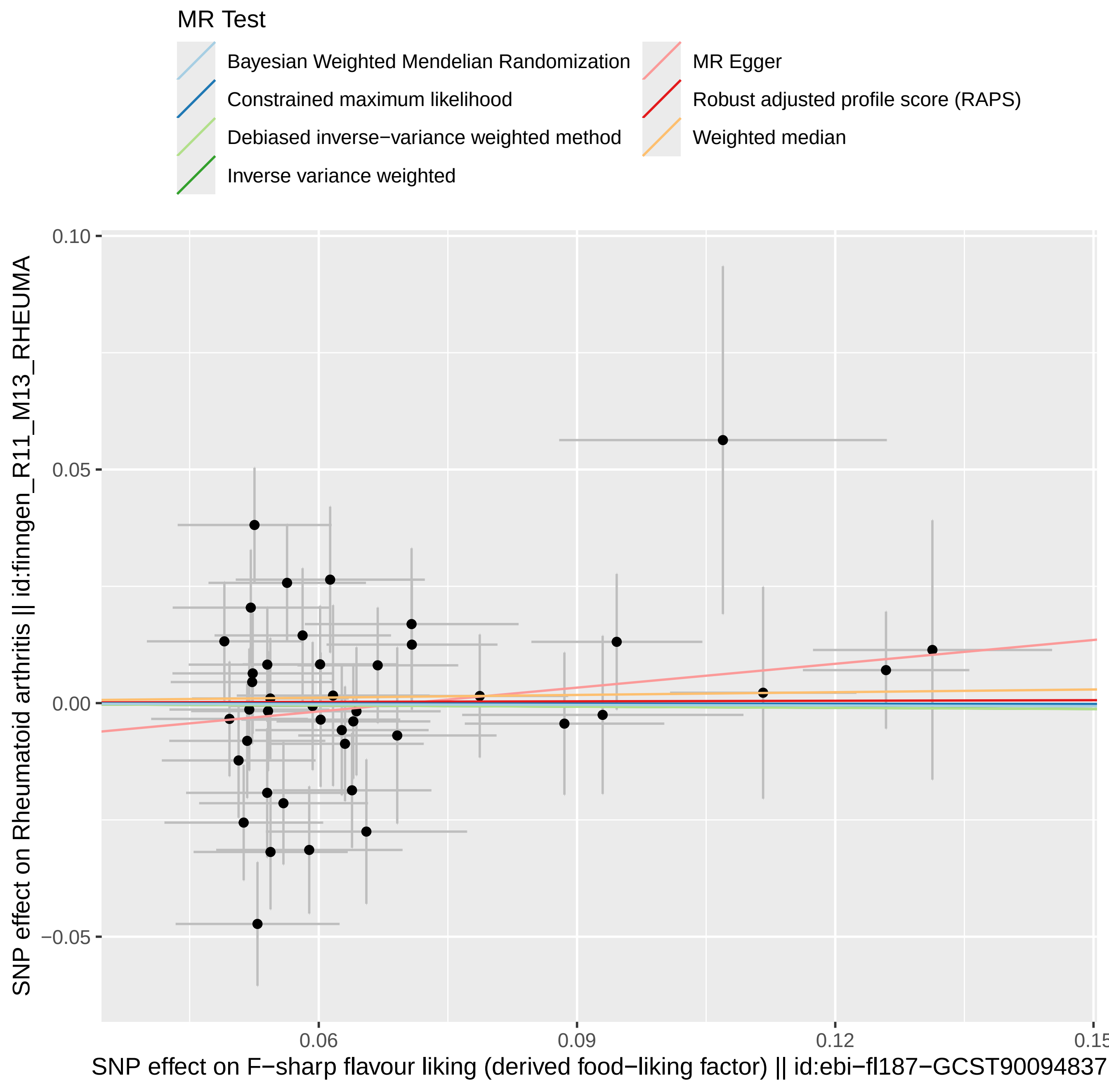

MR Test
Bayesian Weighted Mendelian Randomization
Constrained maximum likelihood
Debiased inverse−variance weighted method
Inverse variance weighted
MR Egger
Robust adjusted profile score (RAPS)
Weighted median
0.10
0.05
0.00
−0.05
0.06
0.09
0.12
0.15
SNP effect on Rheumatoid arthritis || id:finngen_R11_M13_RHEUMA
SNP effect on F−sharp flavour liking (derived food−liking factor) || id:ebi−fl187−GCST90094837

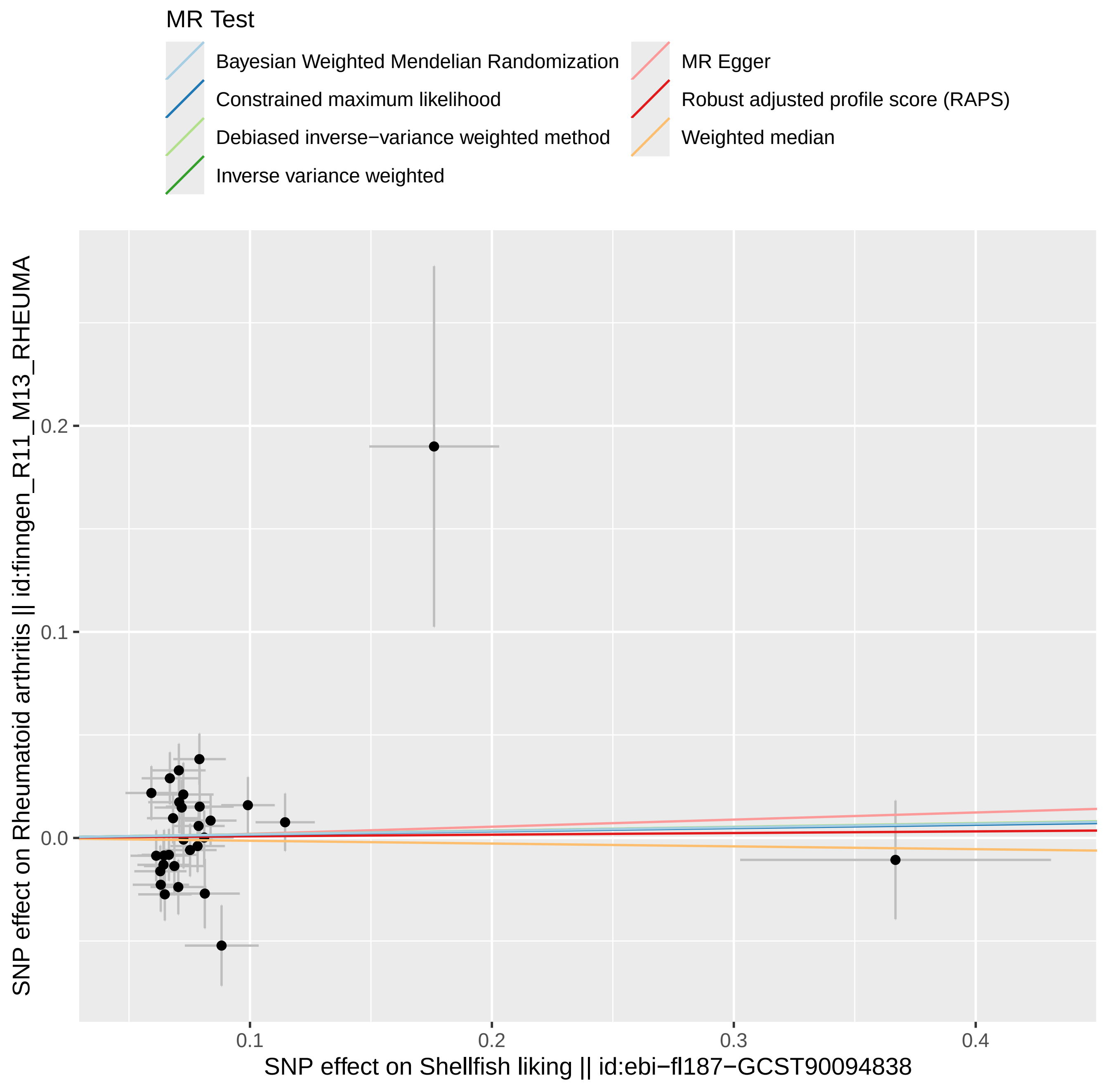

MR Test
Bayesian Weighted Mendelian Randomization
Constrained maximum likelihood
Debiased inverse−variance weighted method
Inverse variance weighted
MR Egger
Robust adjusted profile score (RAPS)
Weighted median
SNP effect on Rheumatoid arthritis || id:finngen_R11_M13_RHEUMA
0.2
0.1
0.0
0.1
0.2
0.3
0.4
SNP effect on Shellfish liking || id:ebi−fl187−GCST90094838

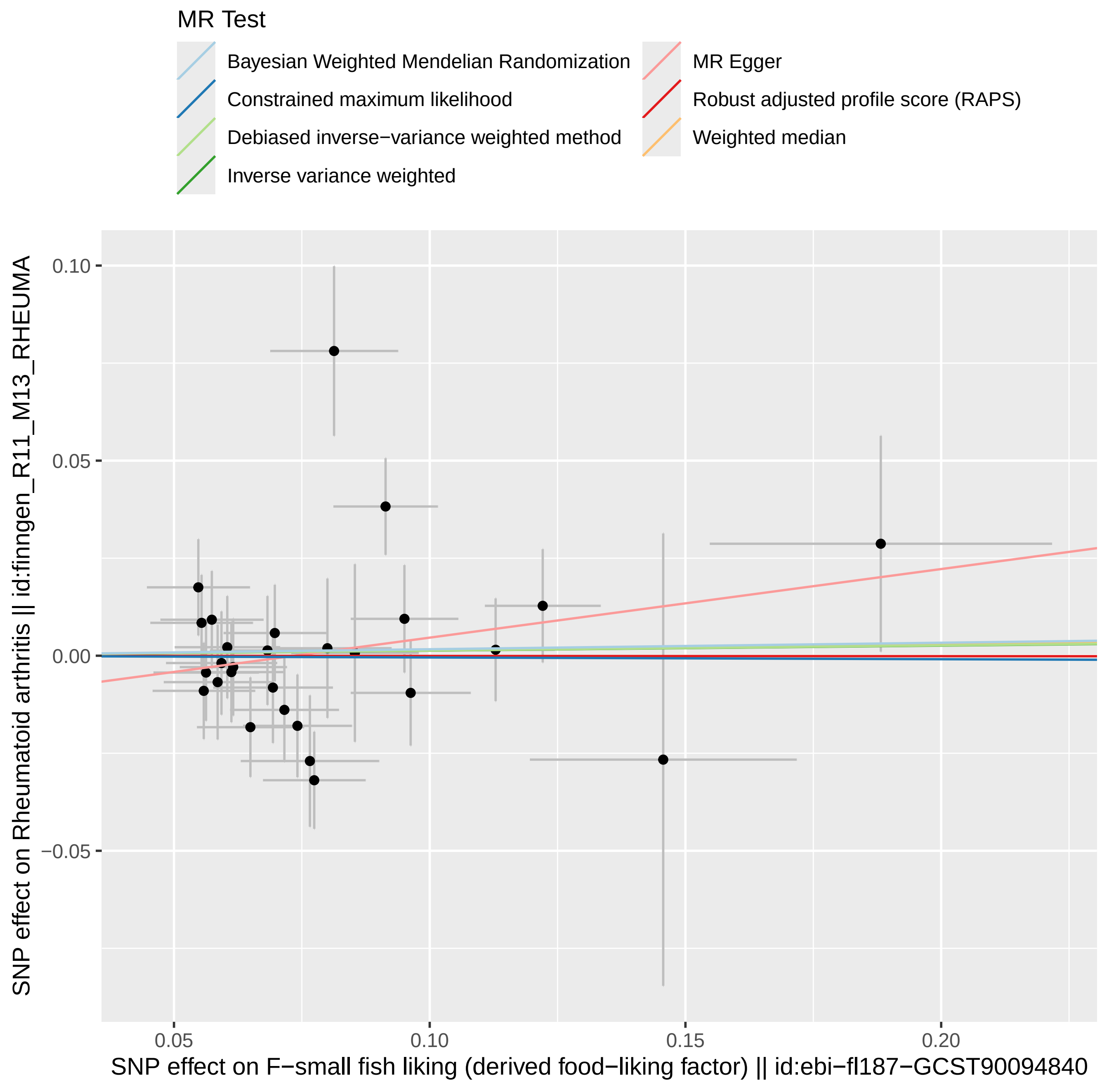

MR Test
Bayesian Weighted Mendelian Randomization
Constrained maximum likelihood
Debiased inverse−variance weighted method
Inverse variance weighted
MR Egger
Robust adjusted profile score (RAPS)
Weighted median
0.10
0.05
0.00
−0.05
0.05
0.10
0.15
0.20
SNP effect on Rheumatoid arthritis || id:finngen_R11_M13_RHEUMA
SNP effect on F−small fish liking (derived food−liking factor) || id:ebi−fl187−GCST90094840

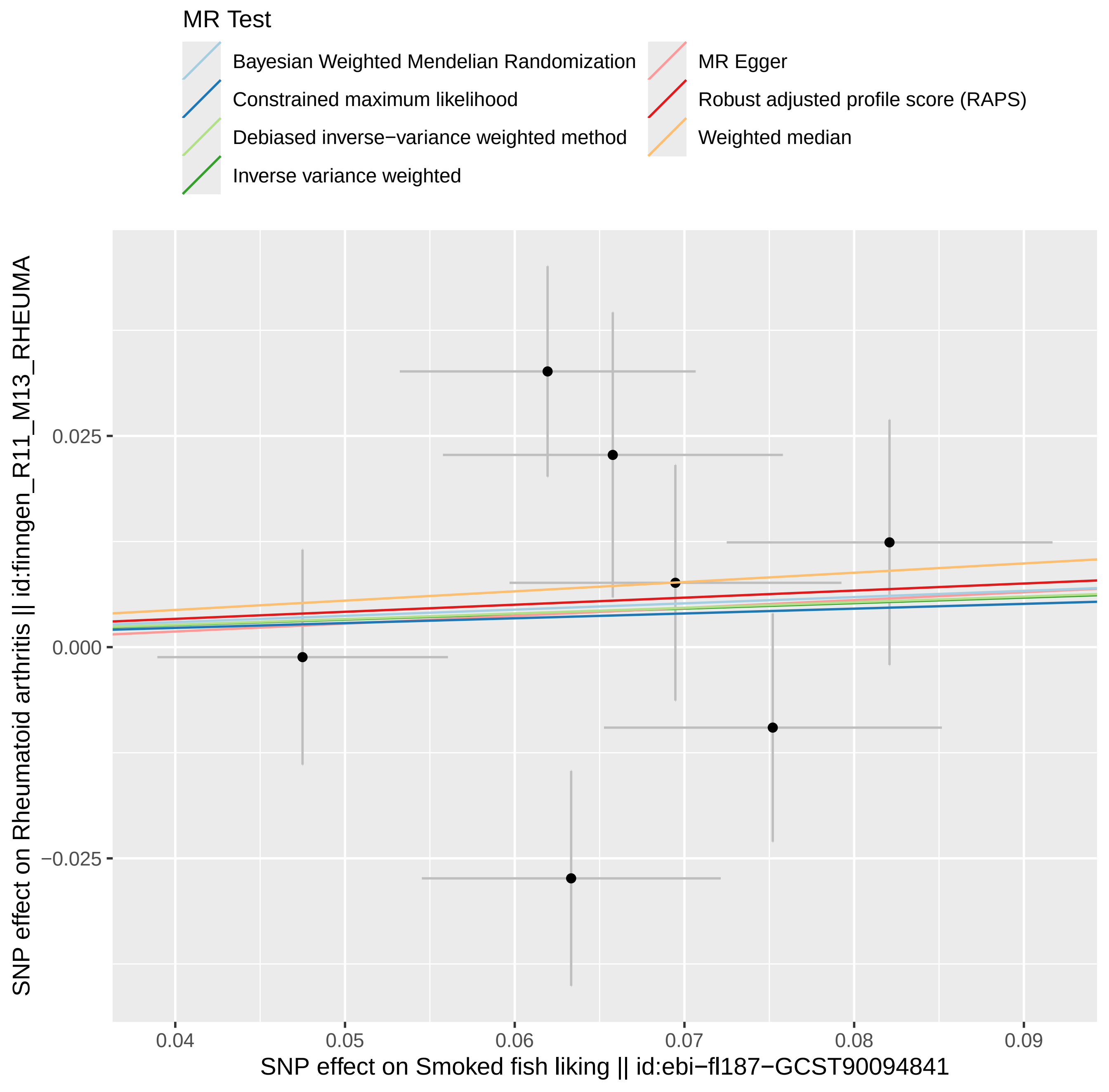

MR Test
Bayesian Weighted Mendelian Randomization
Constrained maximum likelihood
Debiased inverse−variance weighted method
Inverse variance weighted
MR Egger
Robust adjusted profile score (RAPS)
Weighted median
0.025
0.000
−0.025
0.04
0.05
0.06
0.07
0.08
0.09
SNP effect on Rheumatoid arthritis || id:finngen_R11_M13_RHEUMA
SNP effect on Smoked fish liking || id:ebi−fl187−GCST90094841

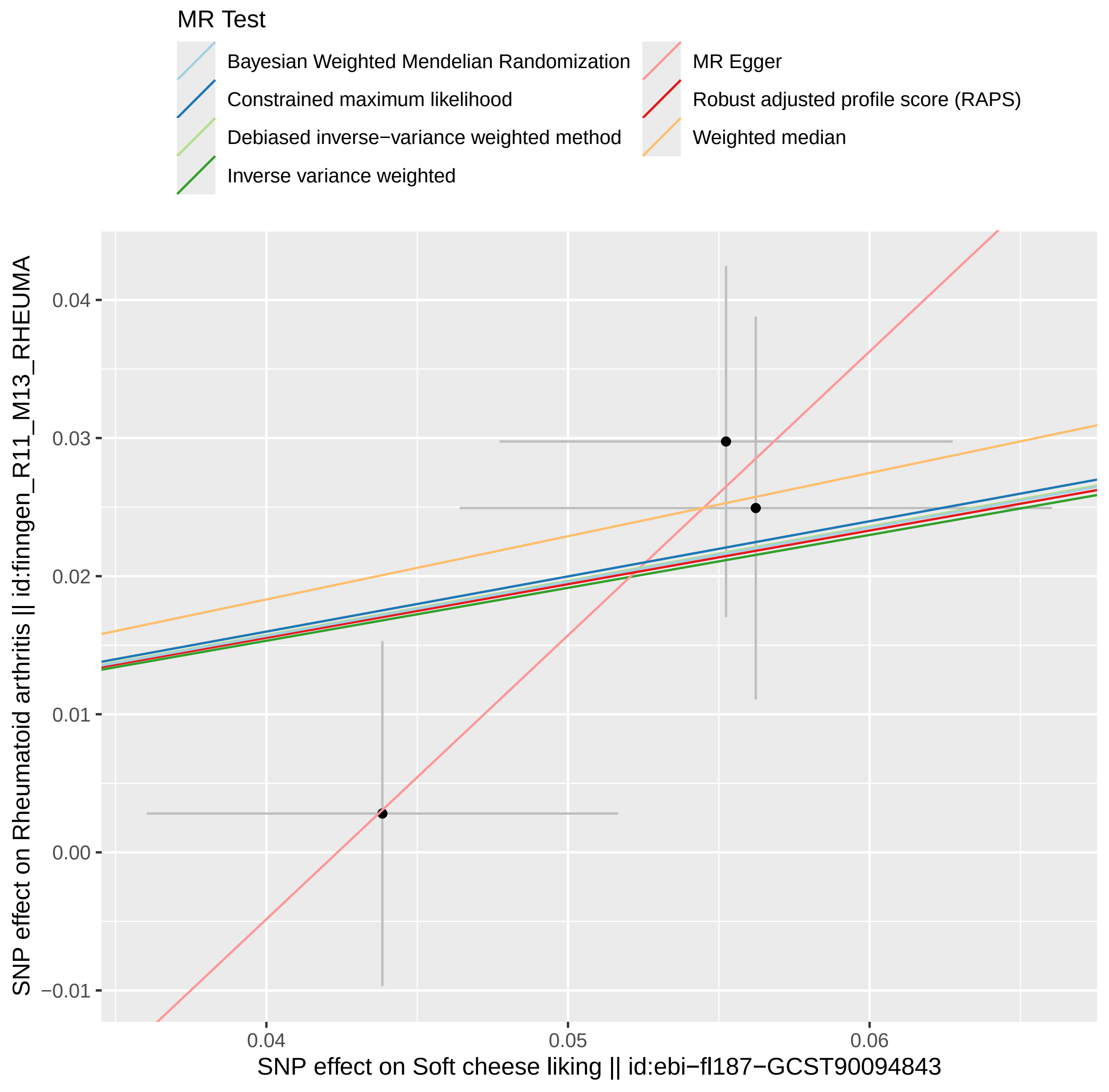
MR Test
Bayesian Weighted Mendelian Randomization
Constrained maximum likelihood
Debiased inverse−variance weighted method
Inverse variance weighted
MR Egger
Robust adjusted profile score (RAPS)
Weighted median
SNP effect on Rheumatoid arthritis || id:finngen_R11_M13_RHEUMA
SNP effect on Soft cheese liking || id:ebi−fl187−GCST90094843
0.04
0.03
0.02
0.01
0.00
−0.01
0.04
0.05
0.06

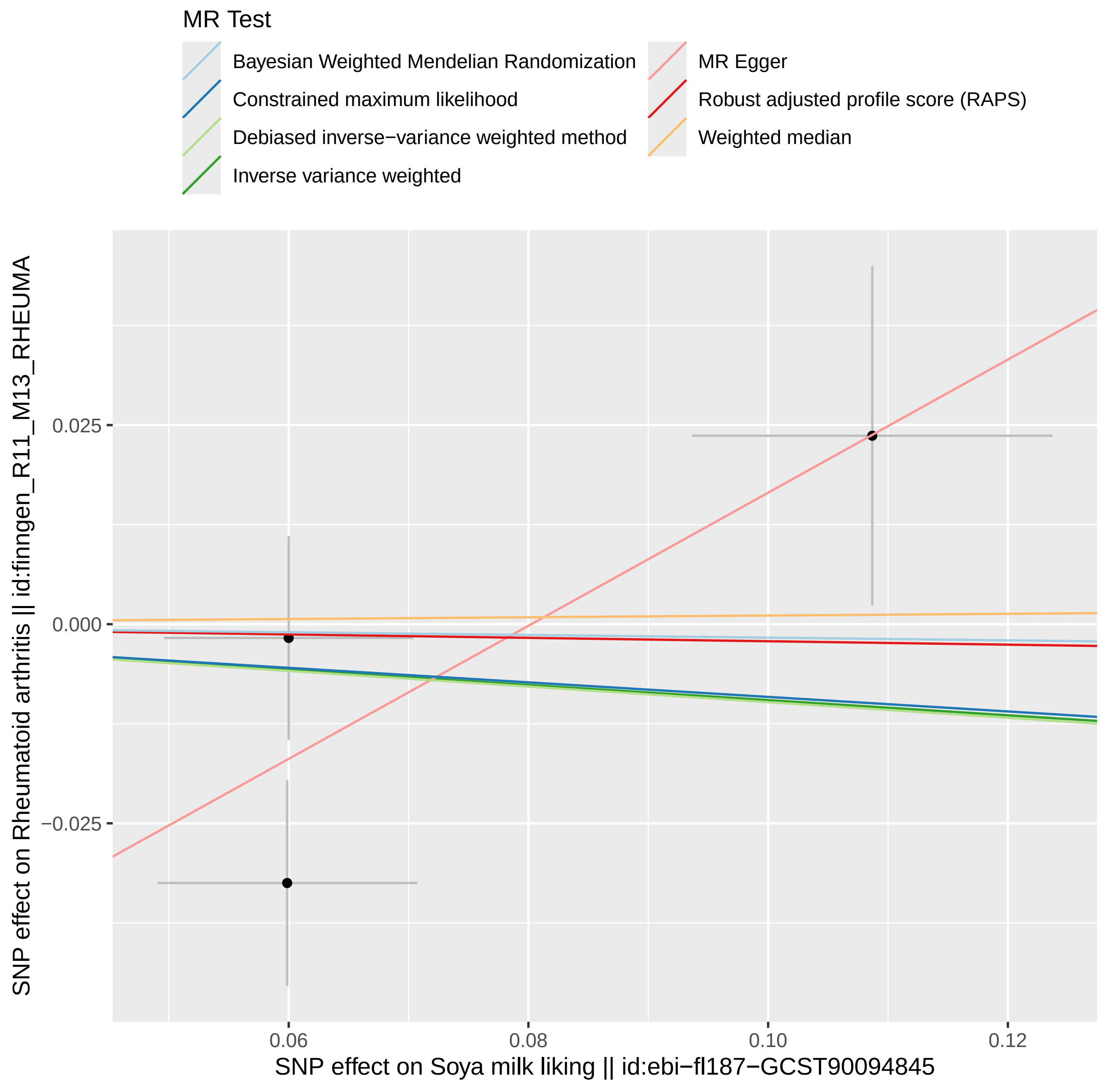
MR Test
Bayesian Weighted Mendelian Randomization
Constrained maximum likelihood
Debiased inverse−variance weighted method
Inverse variance weighted
MR Egger
Robust adjusted profile score (RAPS)
Weighted median
0.025
0.000
−0.025
0.06
0.08
0.10
0.12
SNP effect on Soya milk liking || id:ebi−fl187−GCST90094845
SNP effect on Rheumatoid arthritis || id:finngen_R11_M13_RHEUMA

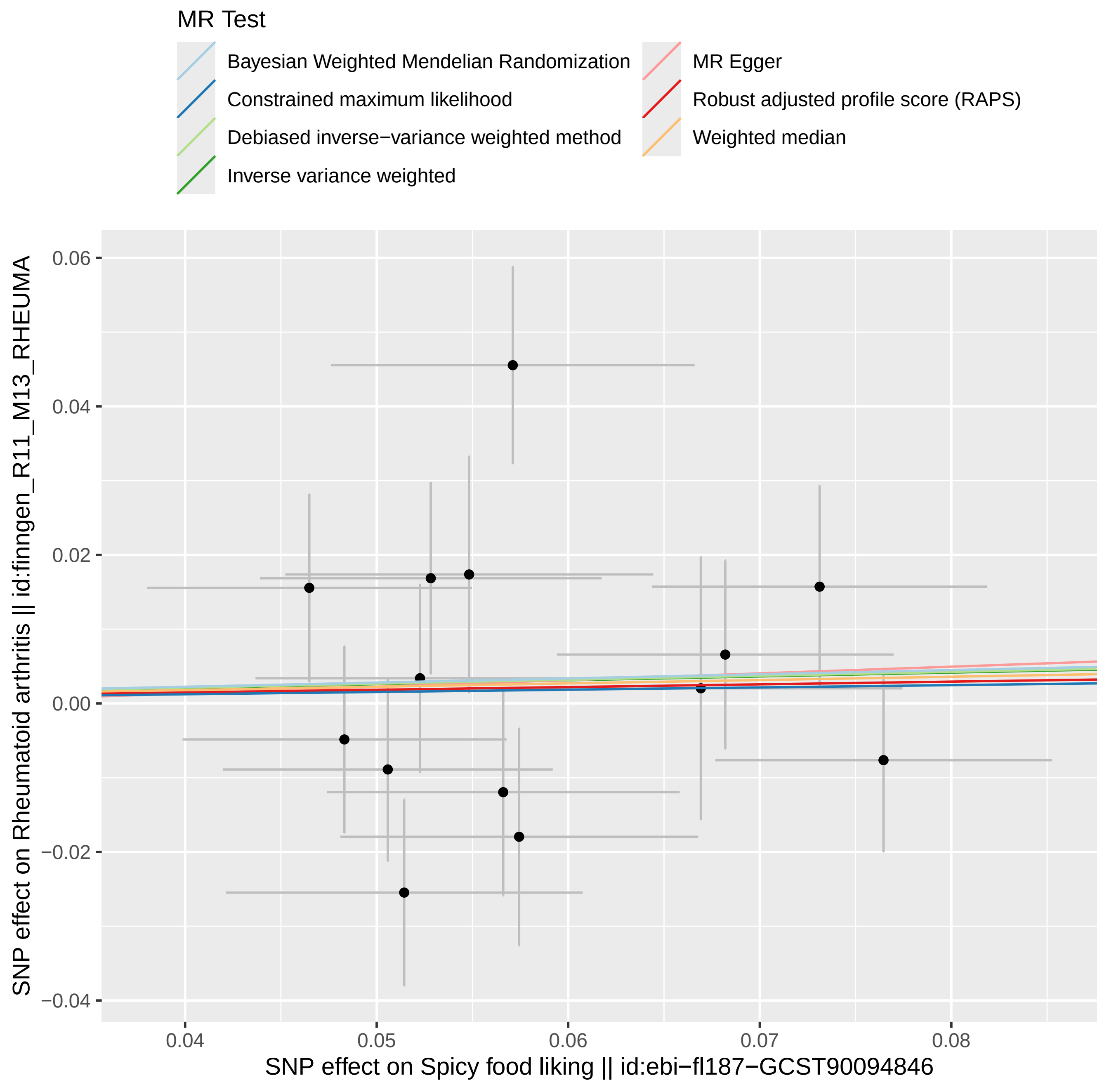
MR Test
Bayesian Weighted Mendelian Randomization
Constrained maximum likelihood
Debiased inverse−variance weighted method
Inverse variance weighted
MR Egger
Robust adjusted profile score (RAPS)
Weighted median
0.06
0.04
0.02
0.00
−0.02
−0.04
0.04
0.05
0.06
0.07
0.08
SNP effect on Rheumatoid arthritis || id:finngen_R11_M13_RHEUMA
SNP effect on Spicy food liking || id:ebi−fl187−GCST90094846

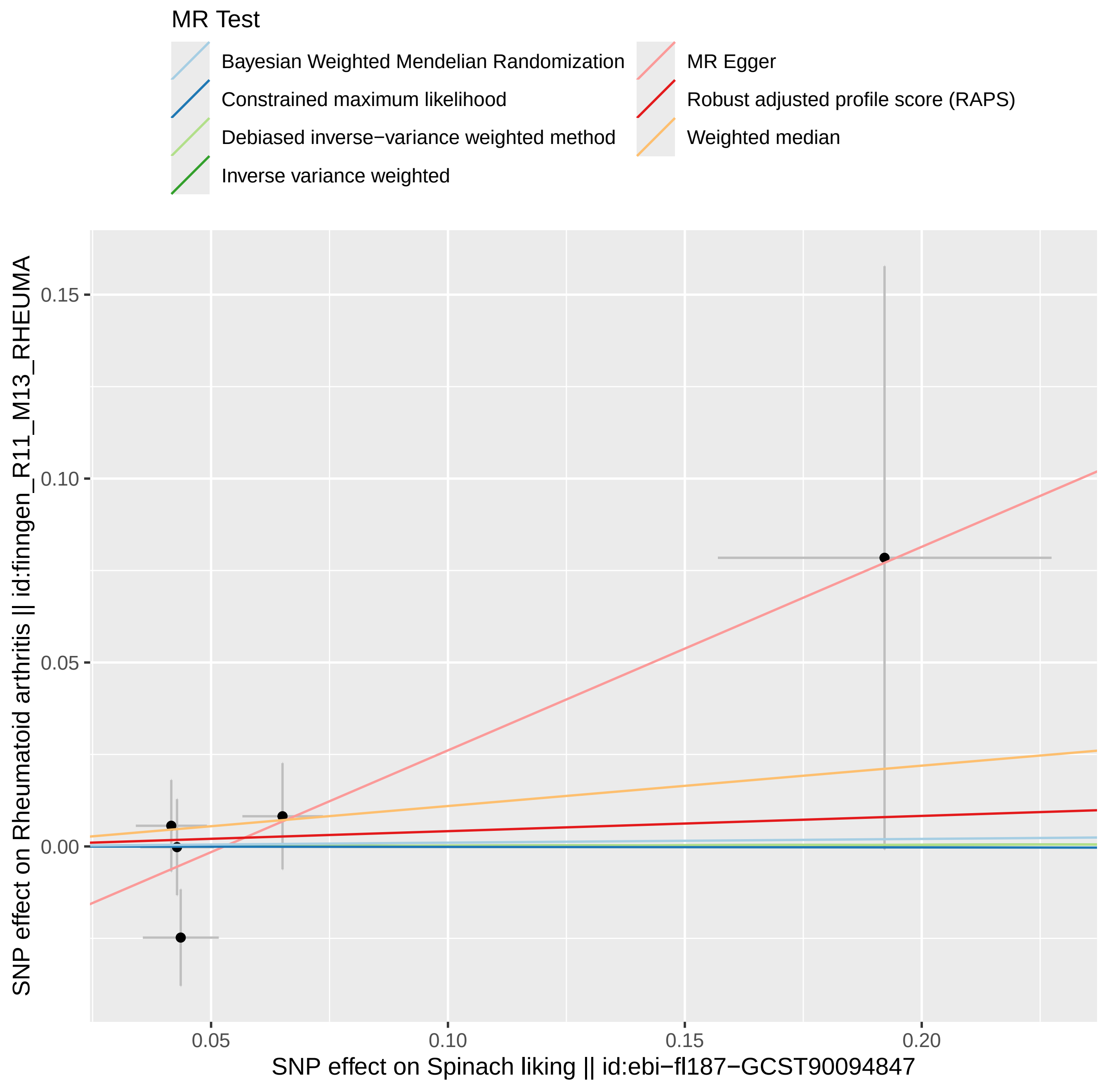

MR Test
Bayesian Weighted Mendelian Randomization
Constrained maximum likelihood
Debiased inverse−variance weighted method
Inverse variance weighted
MR Egger
Robust adjusted profile score (RAPS)
Weighted median
0.15
0.10
0.05
0.00
0.05
0.10
0.15
0.20
SNP effect on Rheumatoid arthritis || id:finngen_R11_M13_RHEUMA
SNP effect on Spinach liking || id:ebi−fl187−GCST90094847

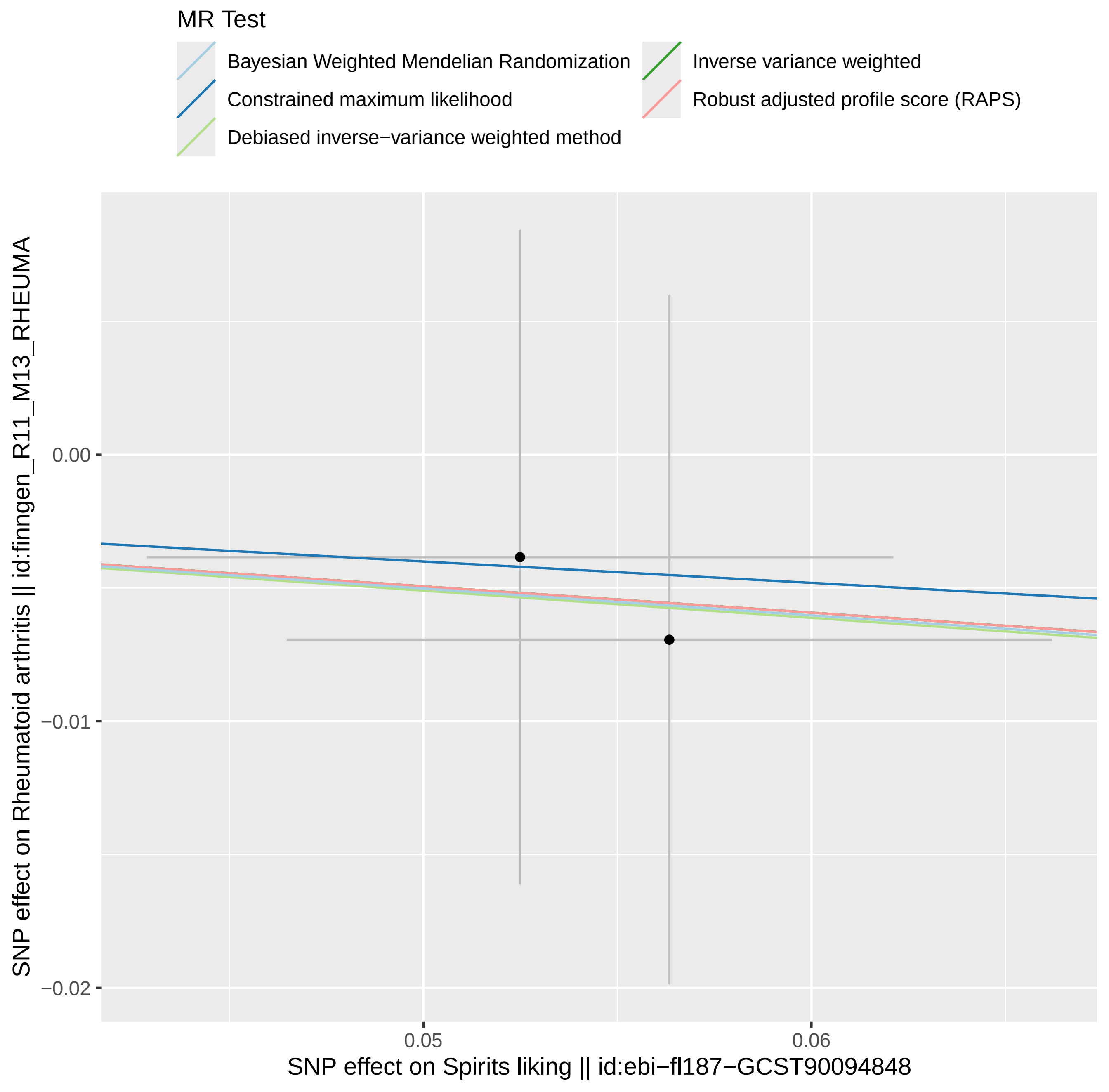
MR Test
Bayesian Weighted Mendelian Randomization
Constrained maximum likelihood
Debiased inverse−variance weighted method
Inverse variance weighted
Robust adjusted profile score (RAPS)
0.00
−0.01
−0.02
0.05
0.06
SNP effect on Rheumatoid arthritis || id:finngen_R11_M13_RHEUMA
SNP effect on Spirits liking || id:ebi−fl187−GCST90094848

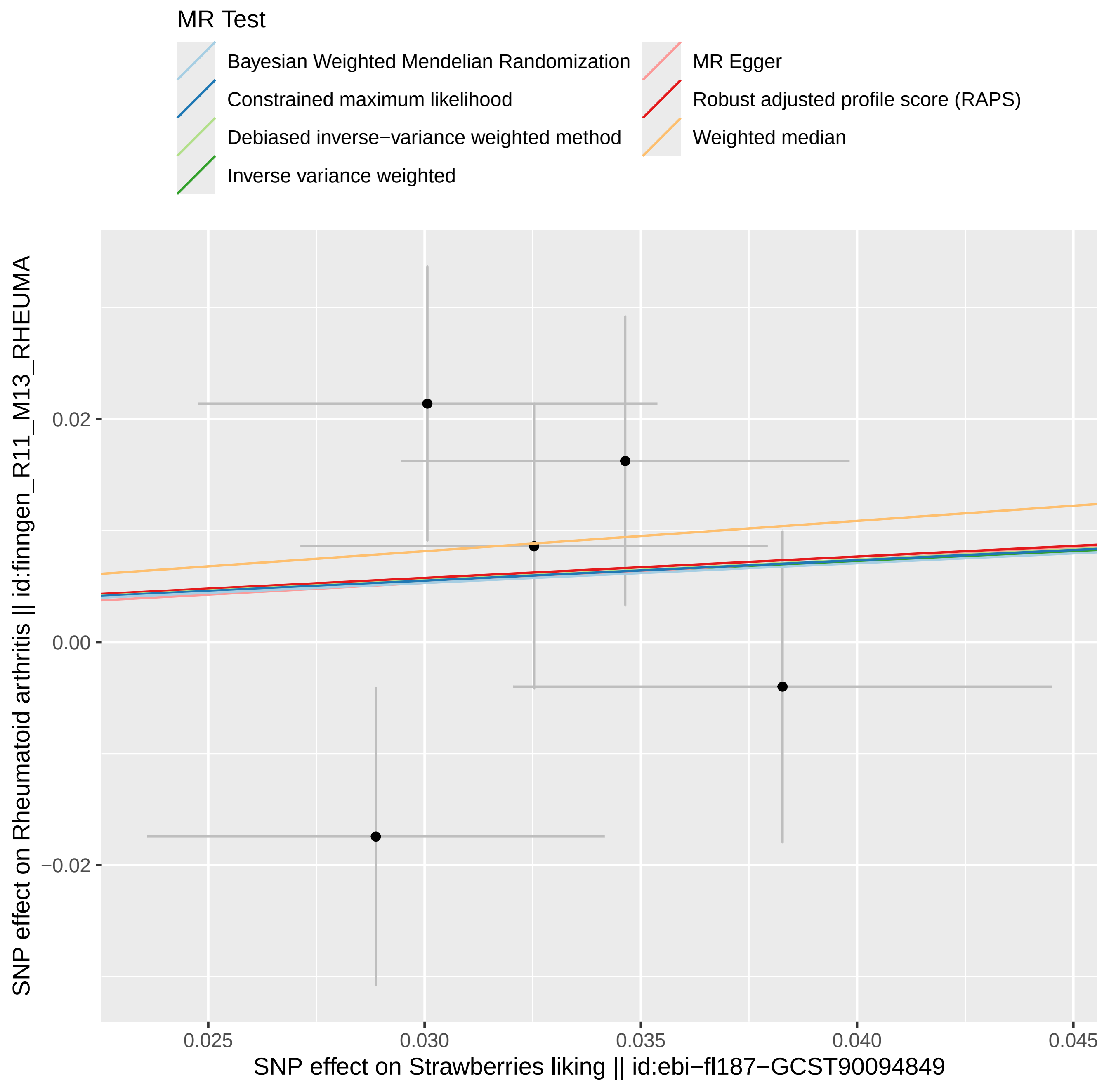
MR Test
Bayesian Weighted Mendelian Randomization
Constrained maximum likelihood
Debiased inverse−variance weighted method
Inverse variance weighted
MR Egger
Robust adjusted profile score (RAPS)
Weighted median
0.02
0.00
−0.02
0.025
0.030
0.035
0.040
0.045
SNP effect on Strawberries liking || id:ebi−fl187−GCST90094849
SNP effect on Rheumatoid arthritis || id:finngen_R11_M13_RHEUMA

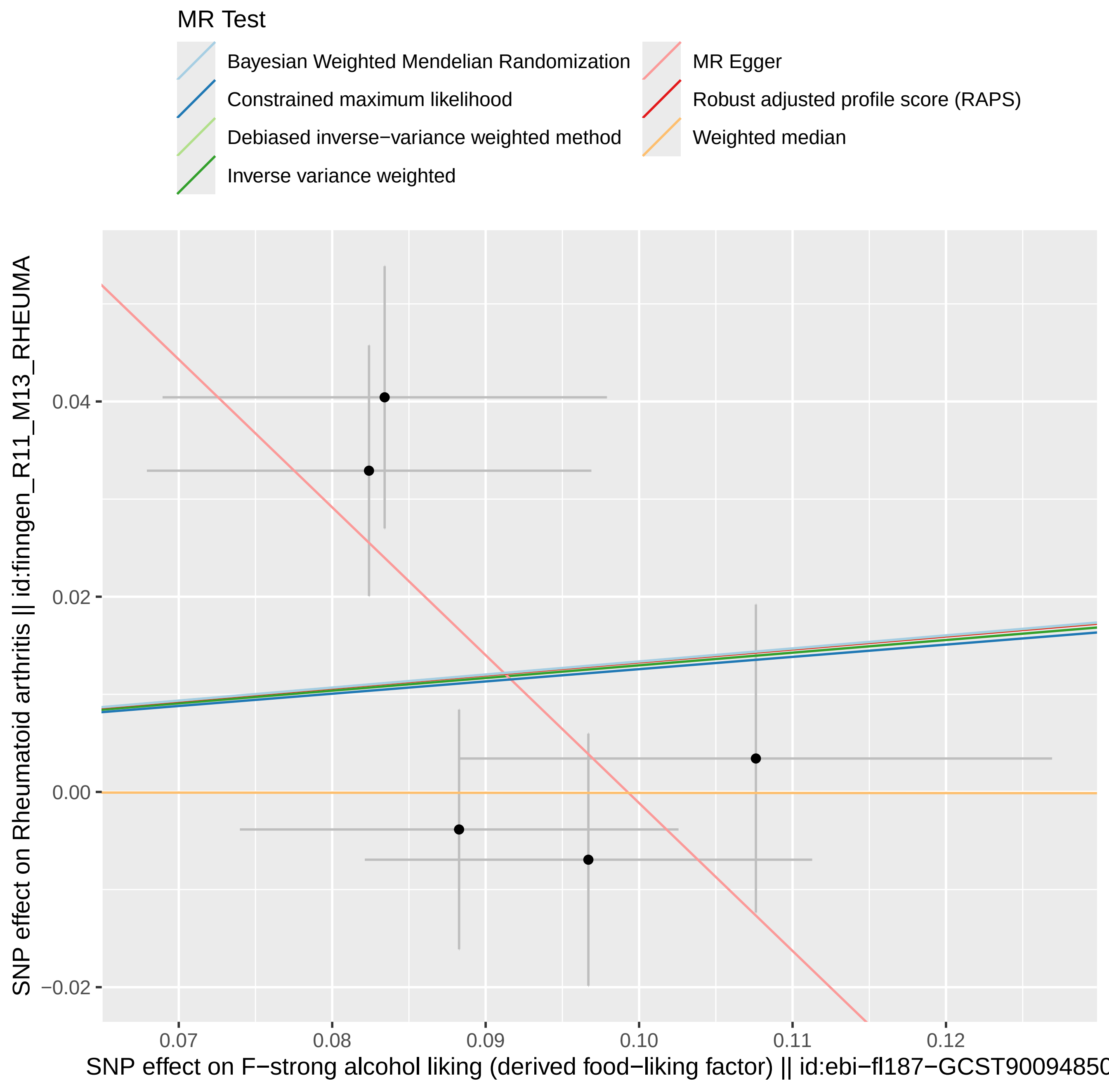
MR Test
Bayesian Weighted Mendelian Randomization
Constrained maximum likelihood
Debiased inverse−variance weighted method
Inverse variance weighted
MR Egger
Robust adjusted profile score (RAPS)
Weighted median
0.04
0.02
0.00
−0.02
0.07
0.08
0.09
0.10
0.11
0.12
SNP effect on Rheumatoid arthritis || id:finngen_R11_M13_RHEUMA
SNP effect on F−strong alcohol liking (derived food−liking factor) || id:ebi−fl187−GCST90094850

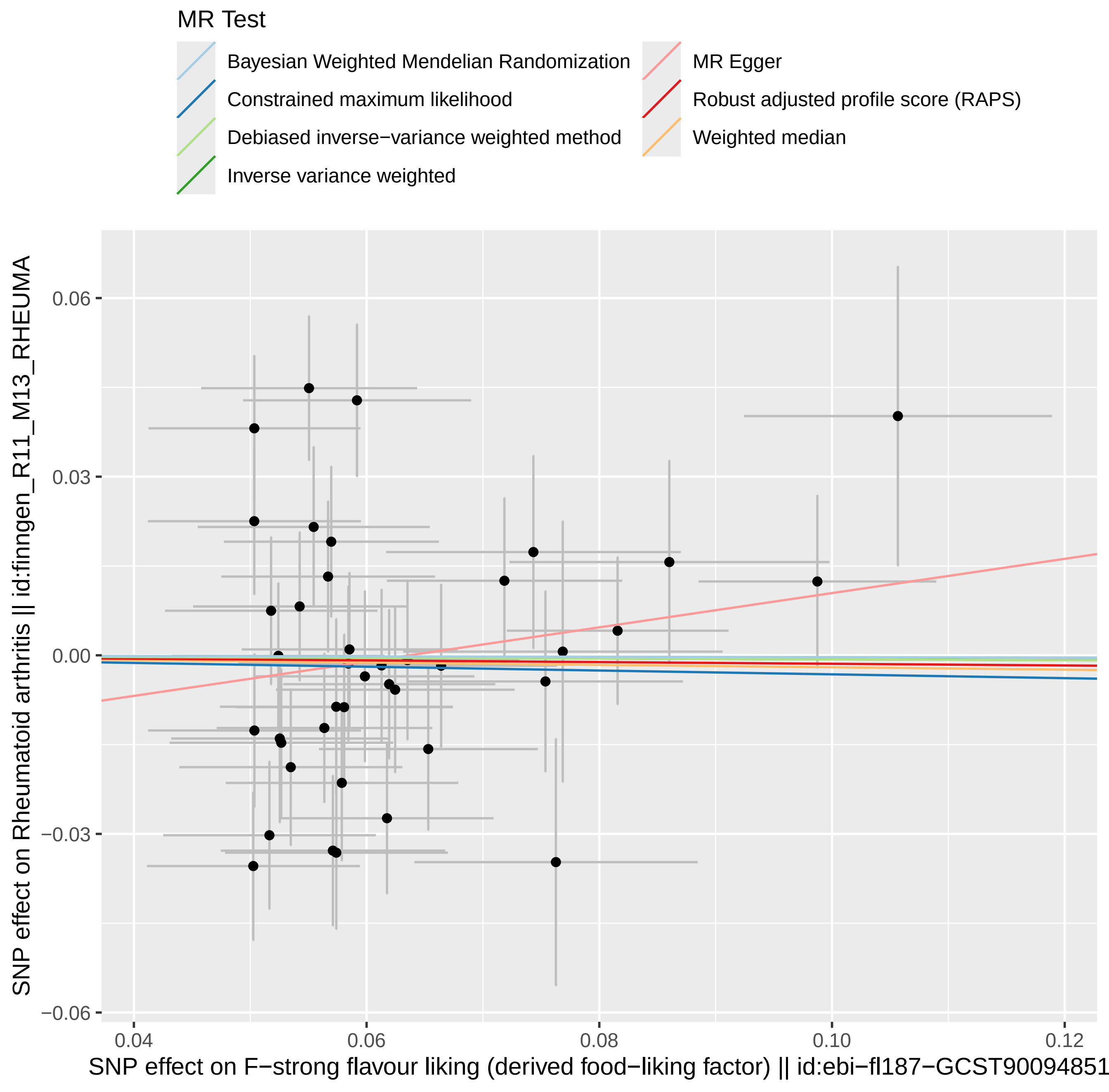

MR Test
Bayesian Weighted Mendelian Randomization
Constrained maximum likelihood
Debiased inverse−variance weighted method
Inverse variance weighted
MR Egger
Robust adjusted profile score (RAPS)
Weighted median
0.06
0.03
0.00
−0.03
−0.06
0.04
0.06
0.08
0.10
0.12
SNP effect on Rheumatoid arthritis || id:finngen_R11_M13_RHEUMA
SNP effect on F−strong flavour liking (derived food−liking factor) || id:ebi−fI187−GCST90094851

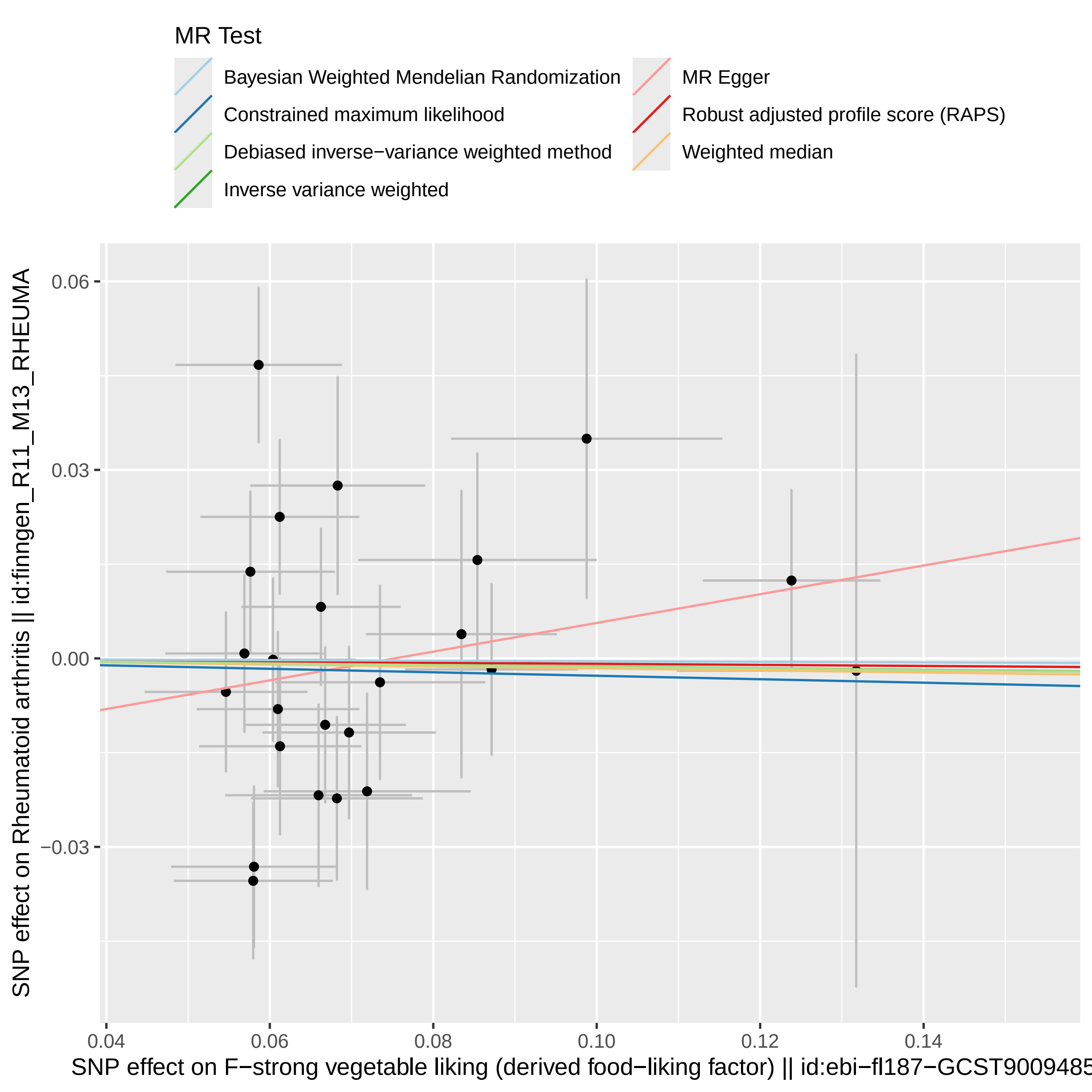
MR Test
Bayesian Weighted Mendelian Randomization
Constrained maximum likelihood
Debiased inverse−variance weighted method
Inverse variance weighted
MR Egger
Robust adjusted profile score (RAPS)
Weighted median
0.06
0.03
0.00
−0.03
0.04
0.06
0.08
0.10
0.12
0.14
SNP effect on Rheumatoid arthritis || id:finngen_R11_M13_RHEUMA
SNP effect on F−strong vegetable liking (derived food−liking factor) || id:ebi−fl187−GCST9009485

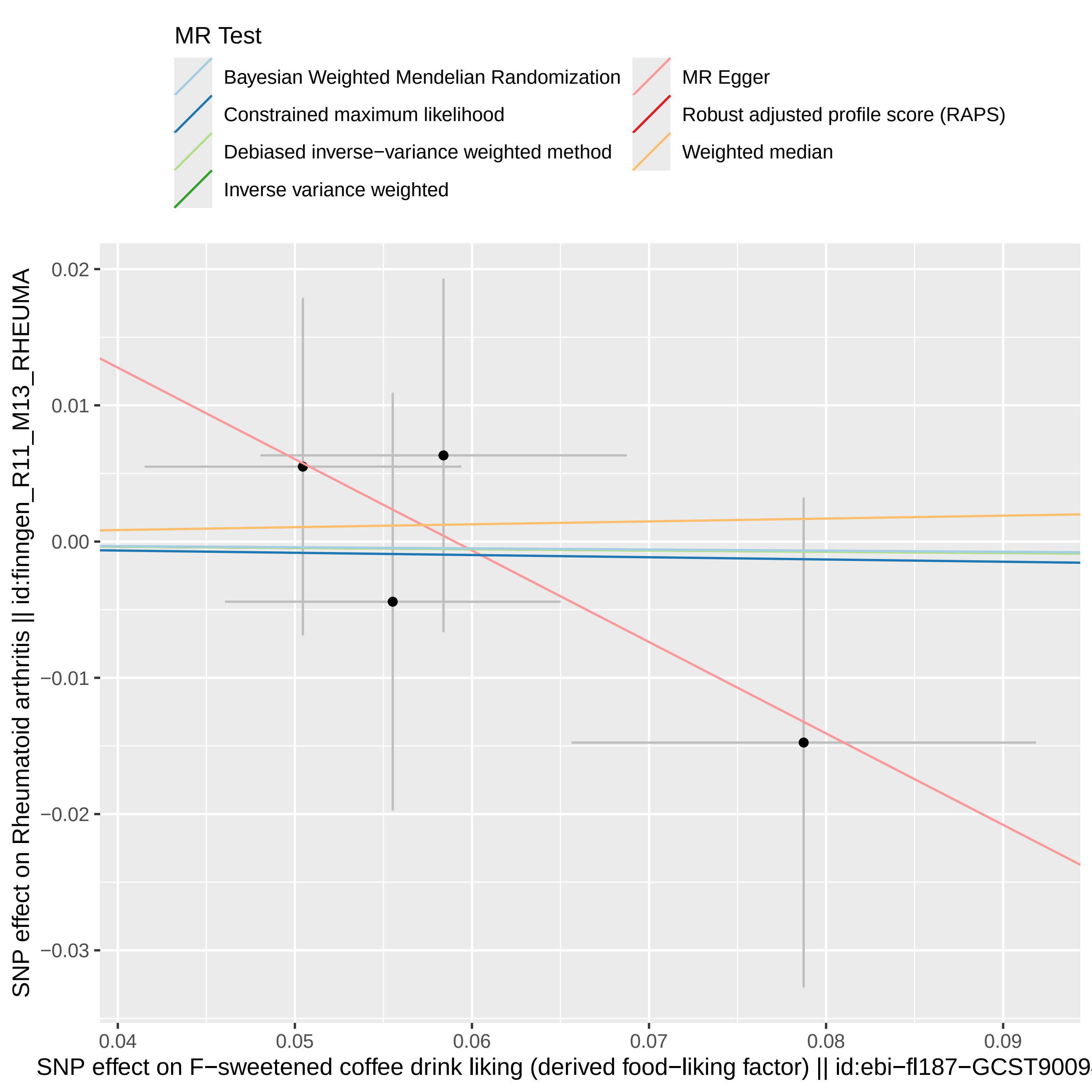

MR Test
Bayesian Weighted Mendelian Randomization
Constrained maximum likelihood
Debiased inverse−variance weighted method
Inverse variance weighted
MR Egger
Robust adjusted profile score (RAPS)
Weighted median
0.02
0.01
0.00
−0.01
−0.02
−0.03
0.04
0.05
0.06
0.07
0.08
0.09
SNP effect on Rheumatoid arthritis || id:finngen_R11_M13_RHEUMA
SNP effect on F−sweetened coffee drink liking (derived food−liking factor) || id:ebi−fl187−GCST9009

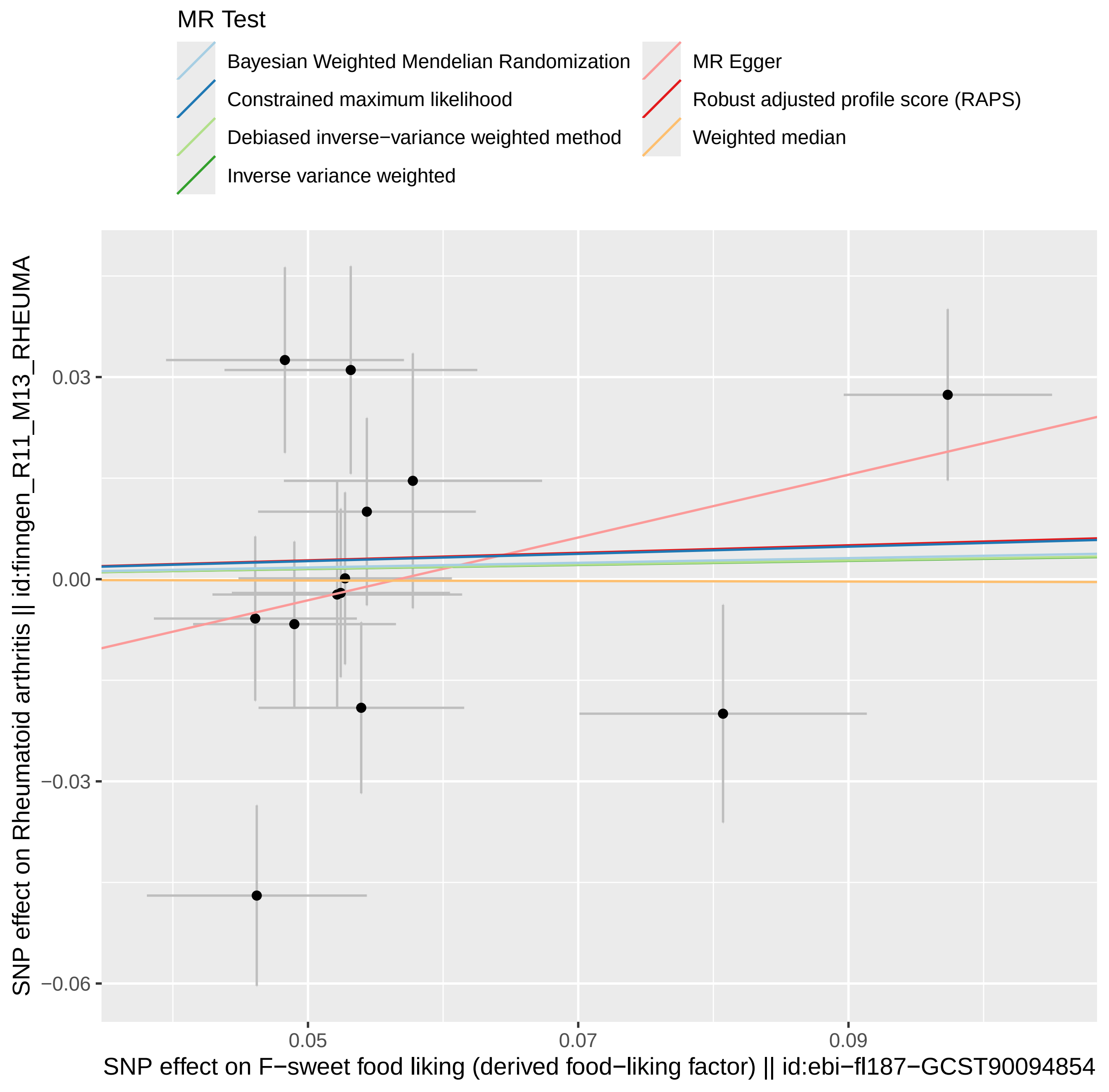
MR Test
Bayesian Weighted Mendelian Randomization
Constrained maximum likelihood
Debiased inverse−variance weighted method
Inverse variance weighted
MR Egger
Robust adjusted profile score (RAPS)
Weighted median
0.03
0.00
−0.03
−0.06
0.05
0.07
0.09
SNP effect on Rheumatoid arthritis || id:finngen_R11_M13_RHEUMA
SNP effect on F−sweet food liking (derived food−liking factor) || id:ebi−fI187−GCST90094854

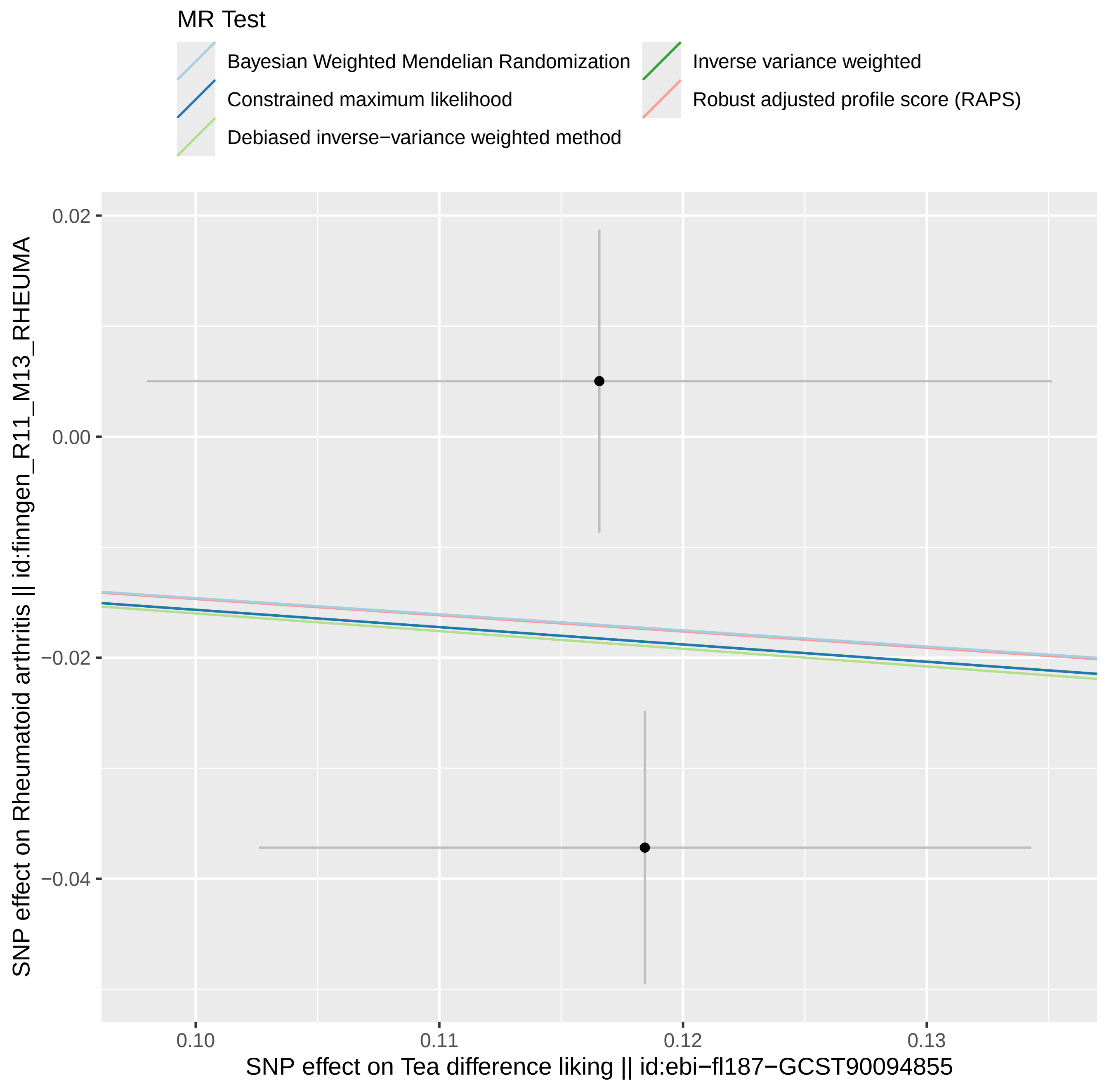
MR Test
Bayesian Weighted Mendelian Randomization
Constrained maximum likelihood
Debiased inverse−variance weighted method
Inverse variance weighted
Robust adjusted profile score (RAPS)
0.02
0.00
−0.02
−0.04
0.10
0.11
0.12
0.13
SNP effect on Rheumatoid arthritis || id:finngen_R11_M13_RHEUMA
SNP effect on Tea difference liking || id:ebi−fl187−GCST90094855

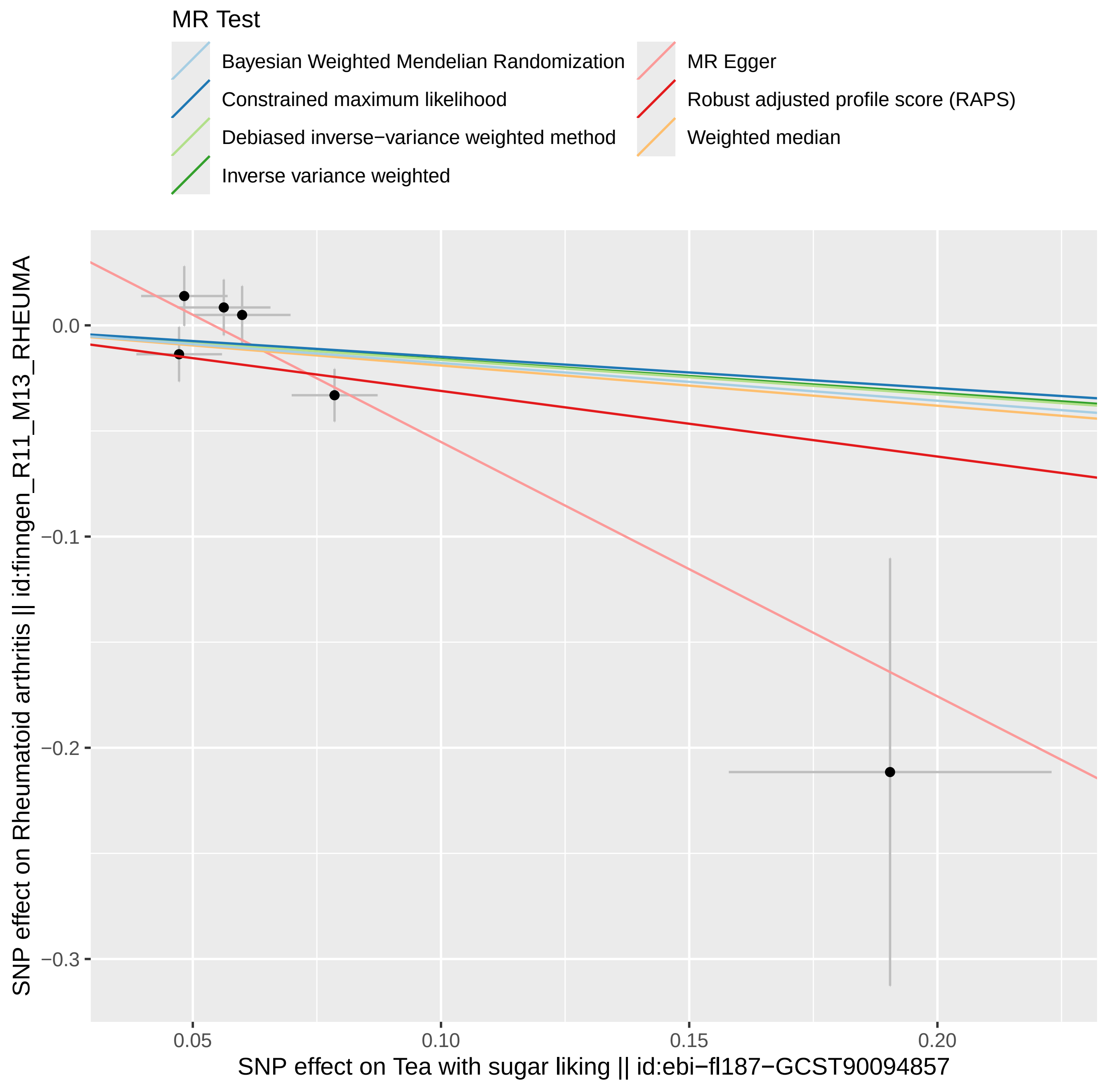
MR Test
Bayesian Weighted Mendelian Randomization
Constrained maximum likelihood
Debiased inverse−variance weighted method
Inverse variance weighted
MR Egger
Robust adjusted profile score (RAPS)
Weighted median
0.0
−0.1
−0.2
−0.3
0.05
0.10
0.15
0.20
SNP effect on Rheumatoid arthritis || id:finngen_R11_M13_RHEUMA
SNP effect on Tea with sugar liking || id:ebi−fl187−GCST90094857

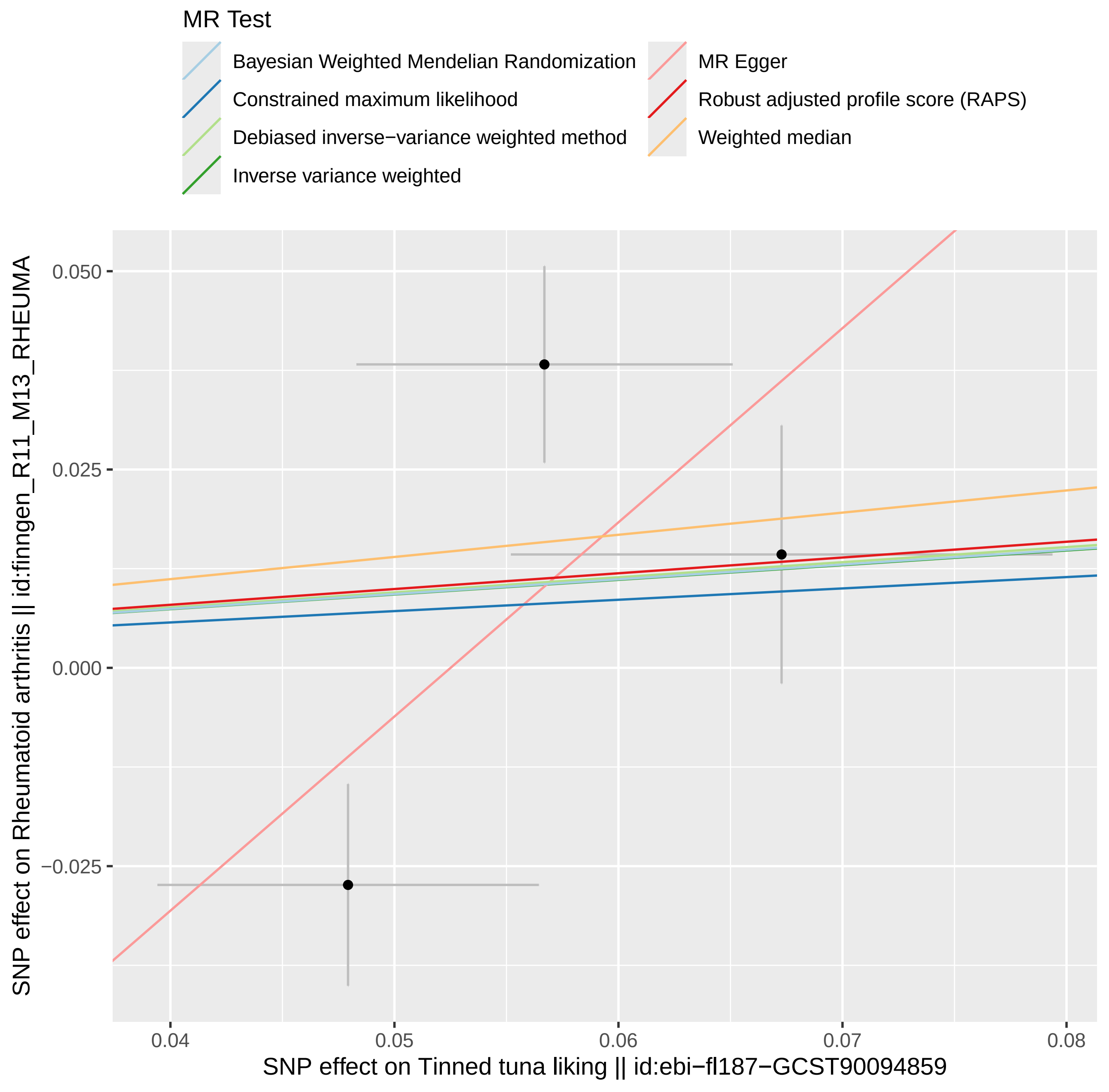
MR Test
Bayesian Weighted Mendelian Randomization
Constrained maximum likelihood
Debiased inverse−variance weighted method
Inverse variance weighted
MR Egger
Robust adjusted profile score (RAPS)
Weighted median
0.050
0.025
0.000
−0.025
0.04
0.05
0.06
0.07
0.08
SNP effect on Rheumatoid arthritis || id:finngen_R11_M13_RHEUMA
SNP effect on Tinned tuna liking || id:ebi−fI187−GCST90094859

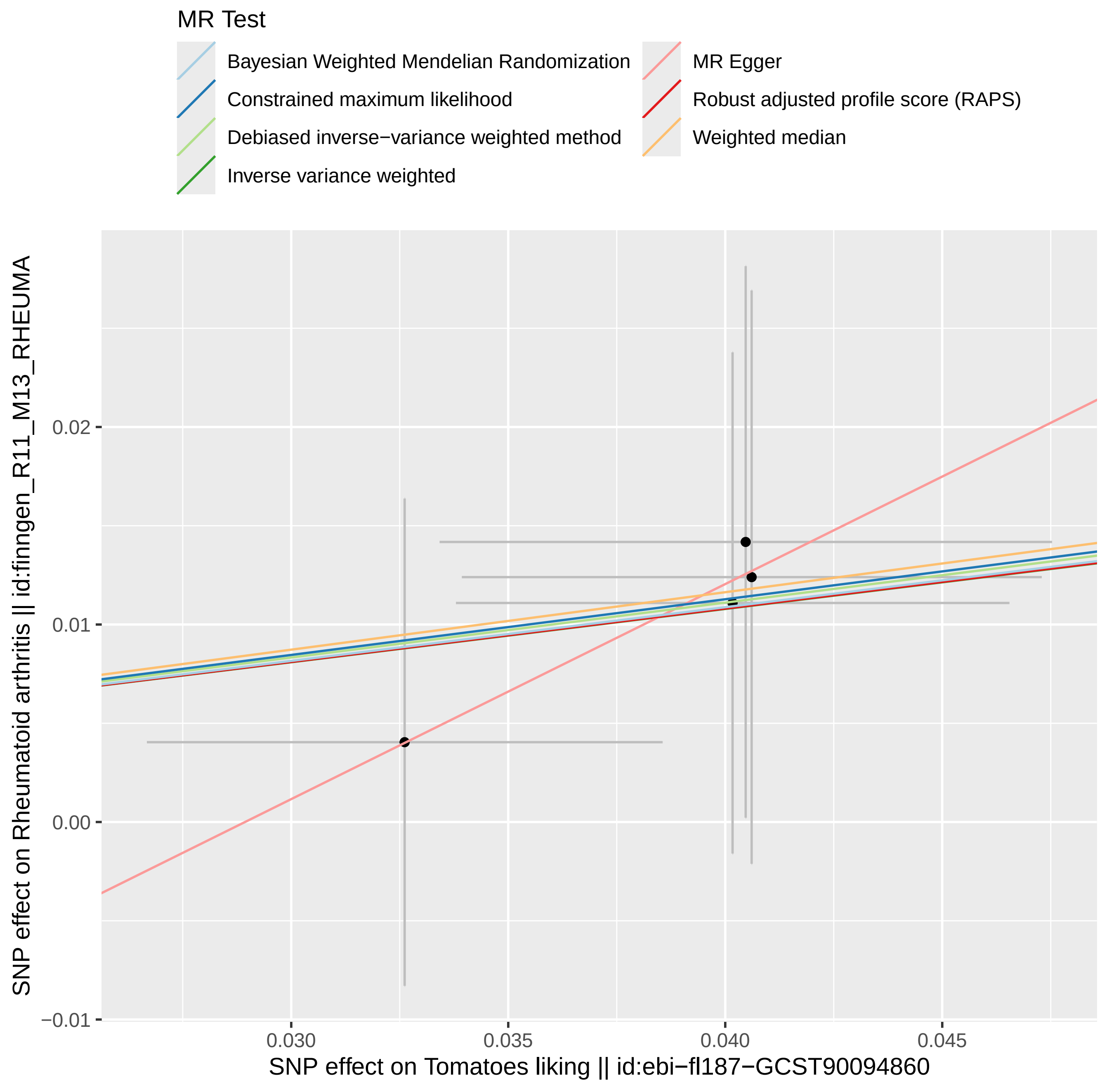
MR Test
Bayesian Weighted Mendelian Randomization
Constrained maximum likelihood
Debiased inverse−variance weighted method
Inverse variance weighted
MR Egger
Robust adjusted profile score (RAPS)
Weighted median
SNP effect on Rheumatoid arthritis || id:finngen_R11_M13_RHEUMA
0.02
0.01
0.00
−0.01
0.030
0.035
0.040
0.045
SNP effect on Tomatoes liking || id:ebi−fl187−GCST90094860

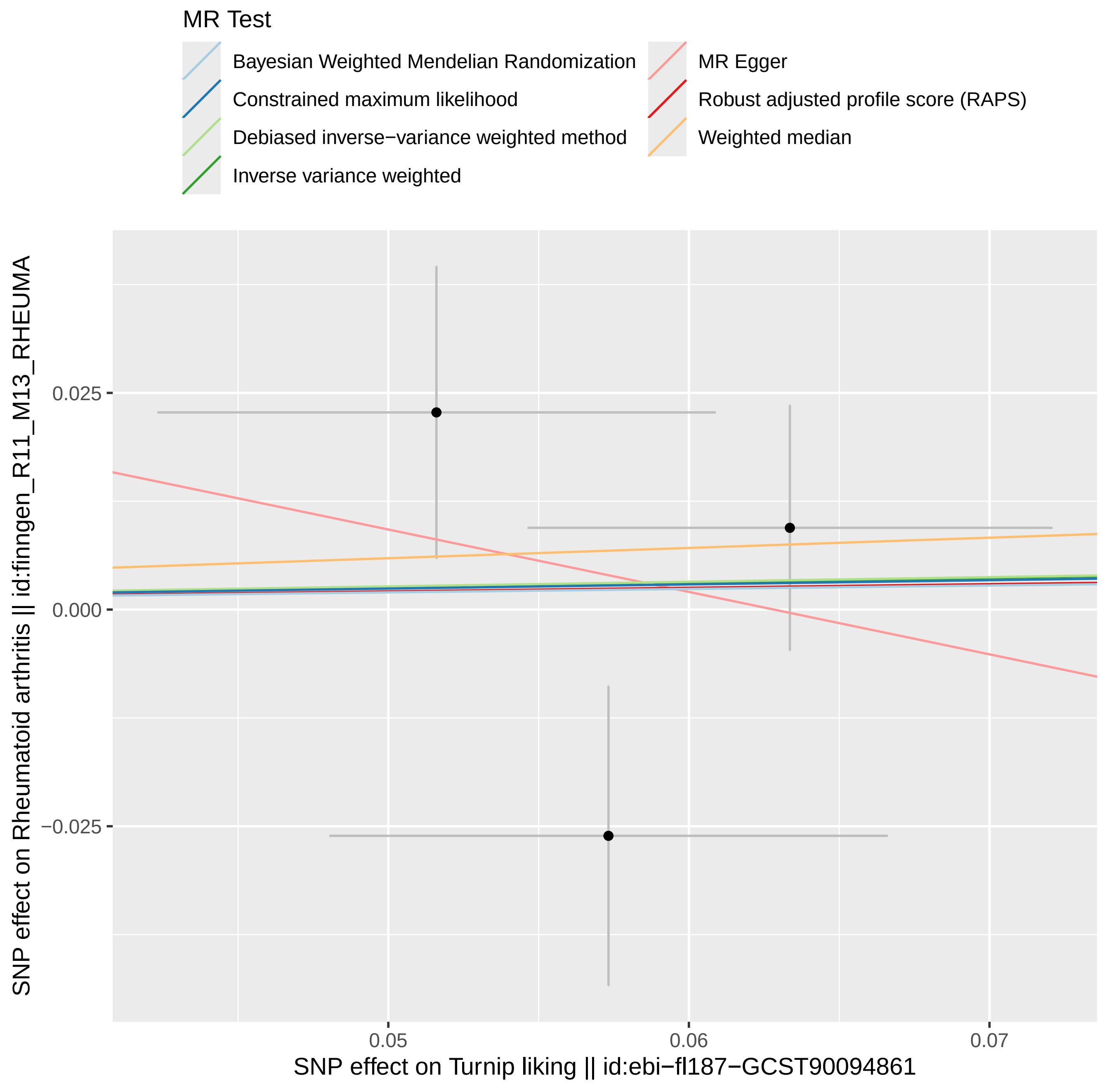
MR Test
Bayesian Weighted Mendelian Randomization
Constrained maximum likelihood
Debiased inverse−variance weighted method
Inverse variance weighted
MR Egger
Robust adjusted profile score (RAPS)
Weighted median
SNP effect on Rheumatoid arthritis || id:finngen_R11_M13_RHEUMA
0.025
0.000
−0.025
0.05
0.06
0.07
SNP effect on Turnip liking || id:ebi−fl187−GCST90094861

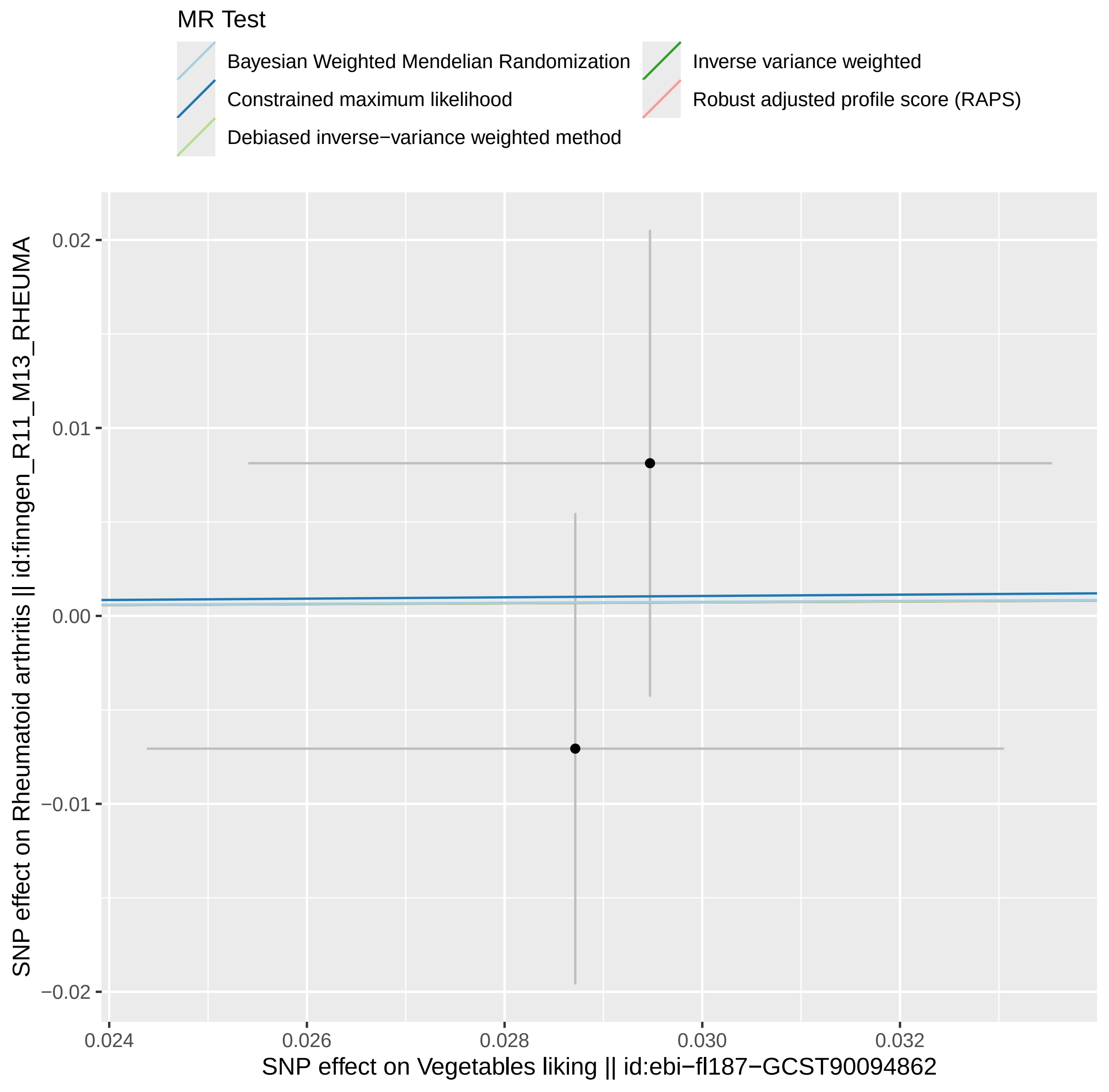
MR Test
Bayesian Weighted Mendelian Randomization
Constrained maximum likelihood
Debiased inverse−variance weighted method
Inverse variance weighted
Robust adjusted profile score (RAPS)
0.02
0.01
0.00
−0.01
−0.02
0.024
0.026
0.028
0.030
0.032
SNP effect on Rheumatoid arthritis || id:finngen_R11_M13_RHEUMA
SNP effect on Vegetables liking || id:ebi−fl187−GCST90094862

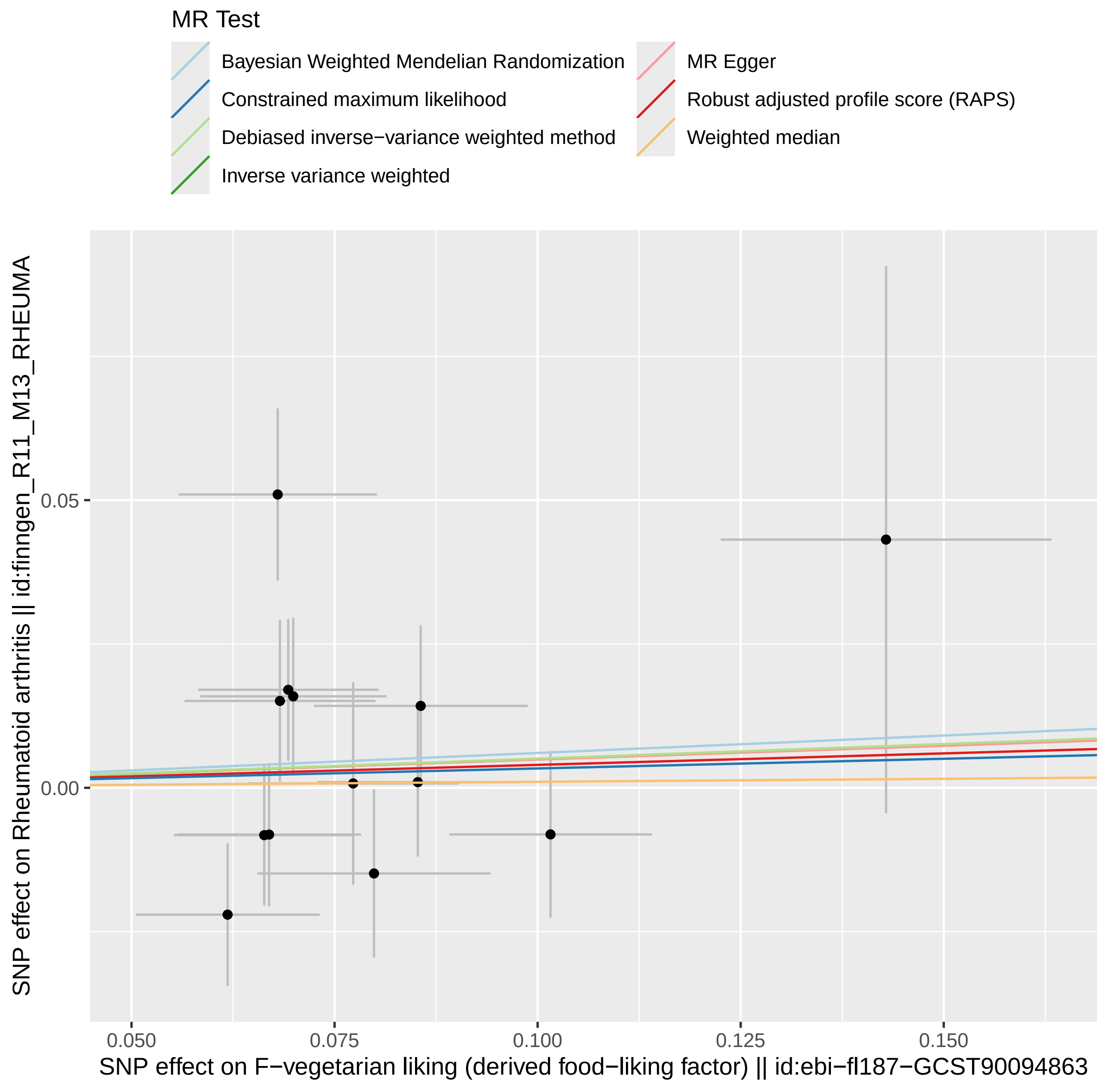
MR Test
Bayesian Weighted Mendelian Randomization
Constrained maximum likelihood
Debiased inverse−variance weighted method
Inverse variance weighted
MR Egger
Robust adjusted profile score (RAPS)
Weighted median
SNP effect on Rheumatoid arthritis || id:finngen_R11_M13_RHEUMA
0.05
0.00
0.050
0.075
0.100
0.125
0.150
SNP effect on F−vegetarian liking (derived food−liking factor) || id:ebi−fl187−GCST90094863

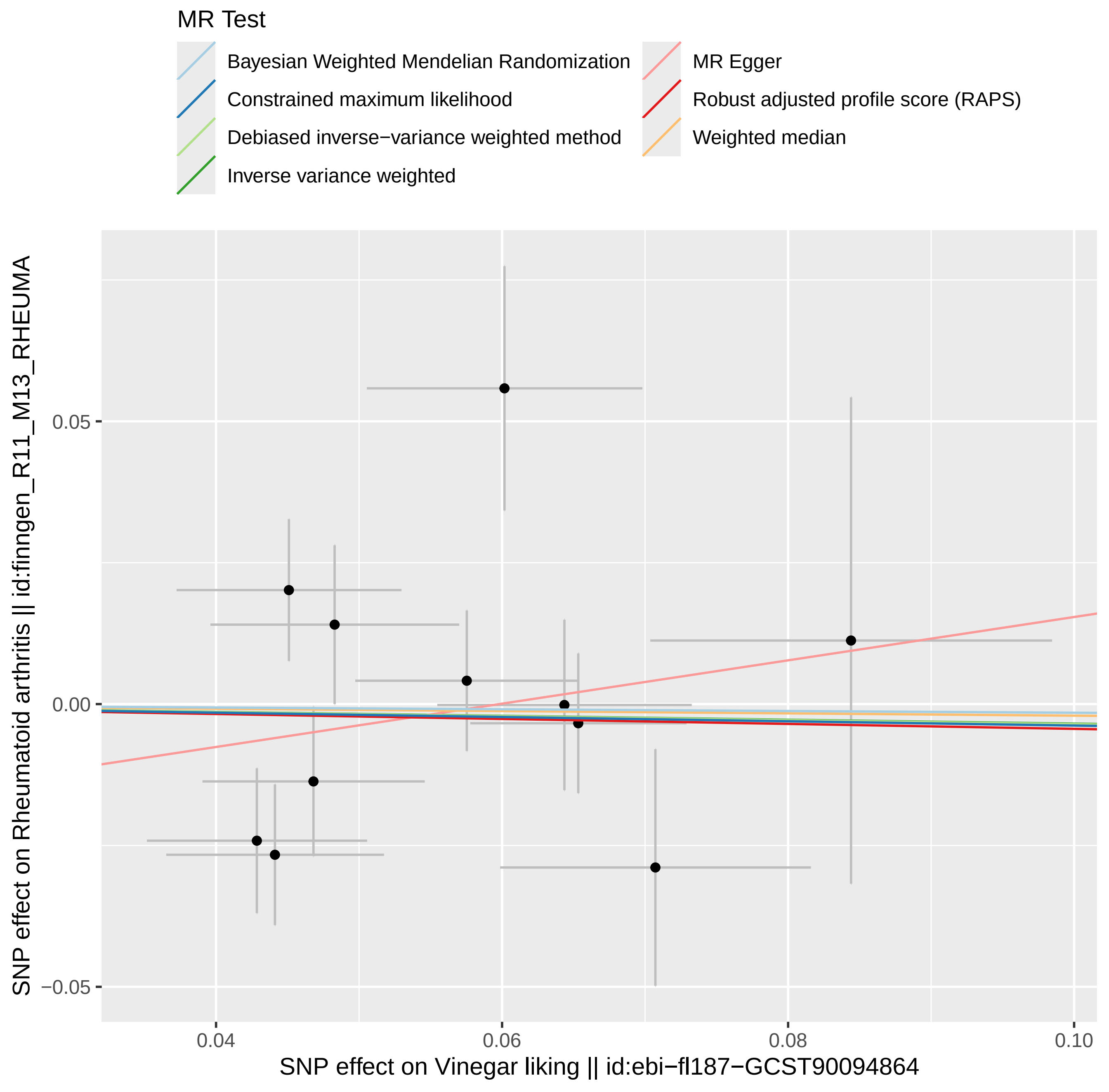
MR Test
Bayesian Weighted Mendelian Randomization
Constrained maximum likelihood
Debiased inverse−variance weighted method
Inverse variance weighted
MR Egger
Robust adjusted profile score (RAPS)
Weighted median
SNP effect on Rheumatoid arthritis || id:finngen_R11_M13_RHEUMA
0.05
0.00
−0.05
0.04
0.06
0.08
0.10
SNP effect on Vinegar liking || id:ebi−fl187−GCST90094864

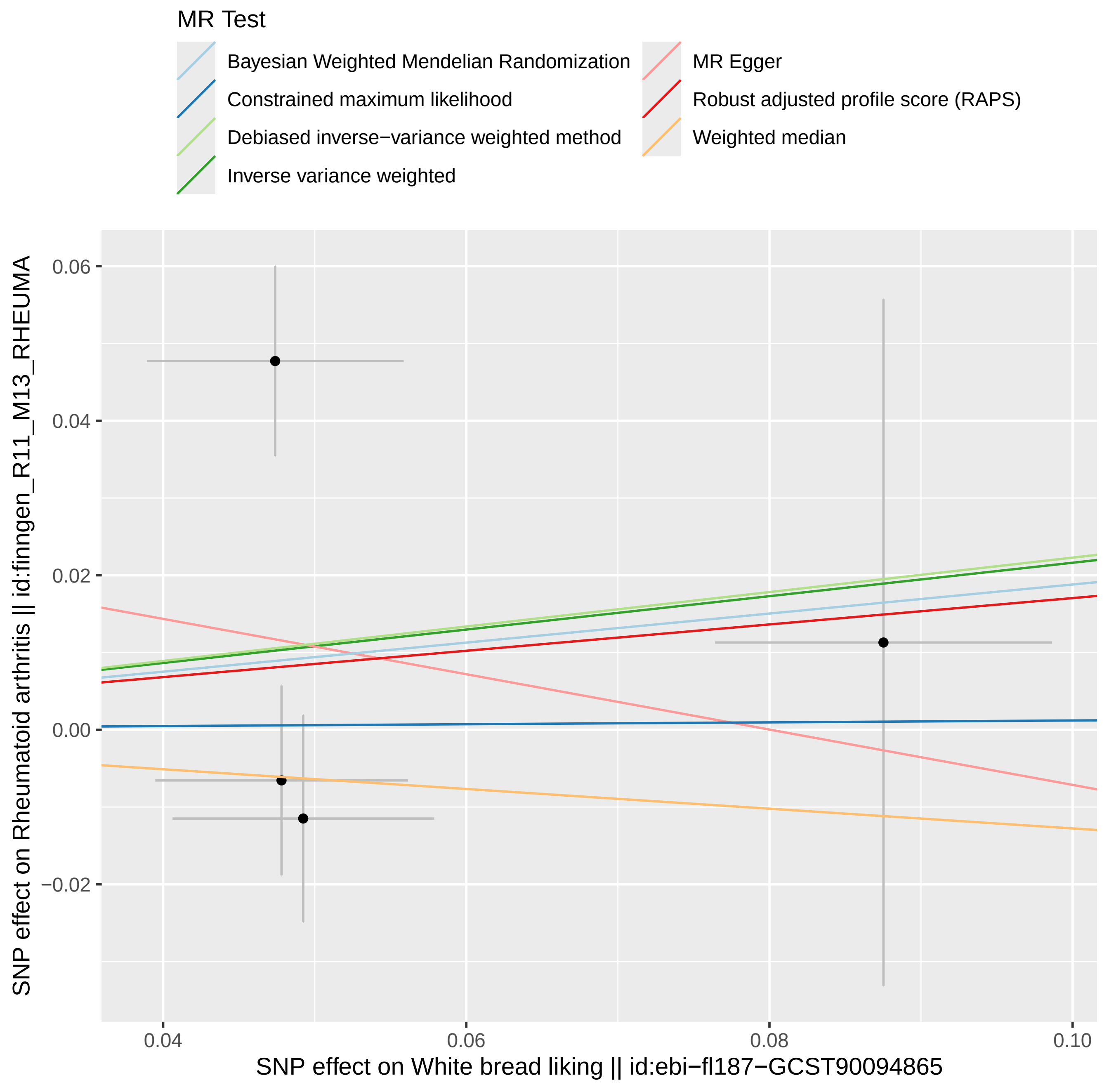

MR Test
Bayesian Weighted Mendelian Randomization
Constrained maximum likelihood
Debiased inverse−variance weighted method
Inverse variance weighted
MR Egger
Robust adjusted profile score (RAPS)
Weighted median
0.06
0.04
0.02
0.00
−0.02
0.04
0.06
0.08
0.10
SNP effect on Rheumatoid arthritis || id:finngen_R11_M13_RHEUMA
SNP effect on White bread liking || id:ebi−fI187−GCST90094865

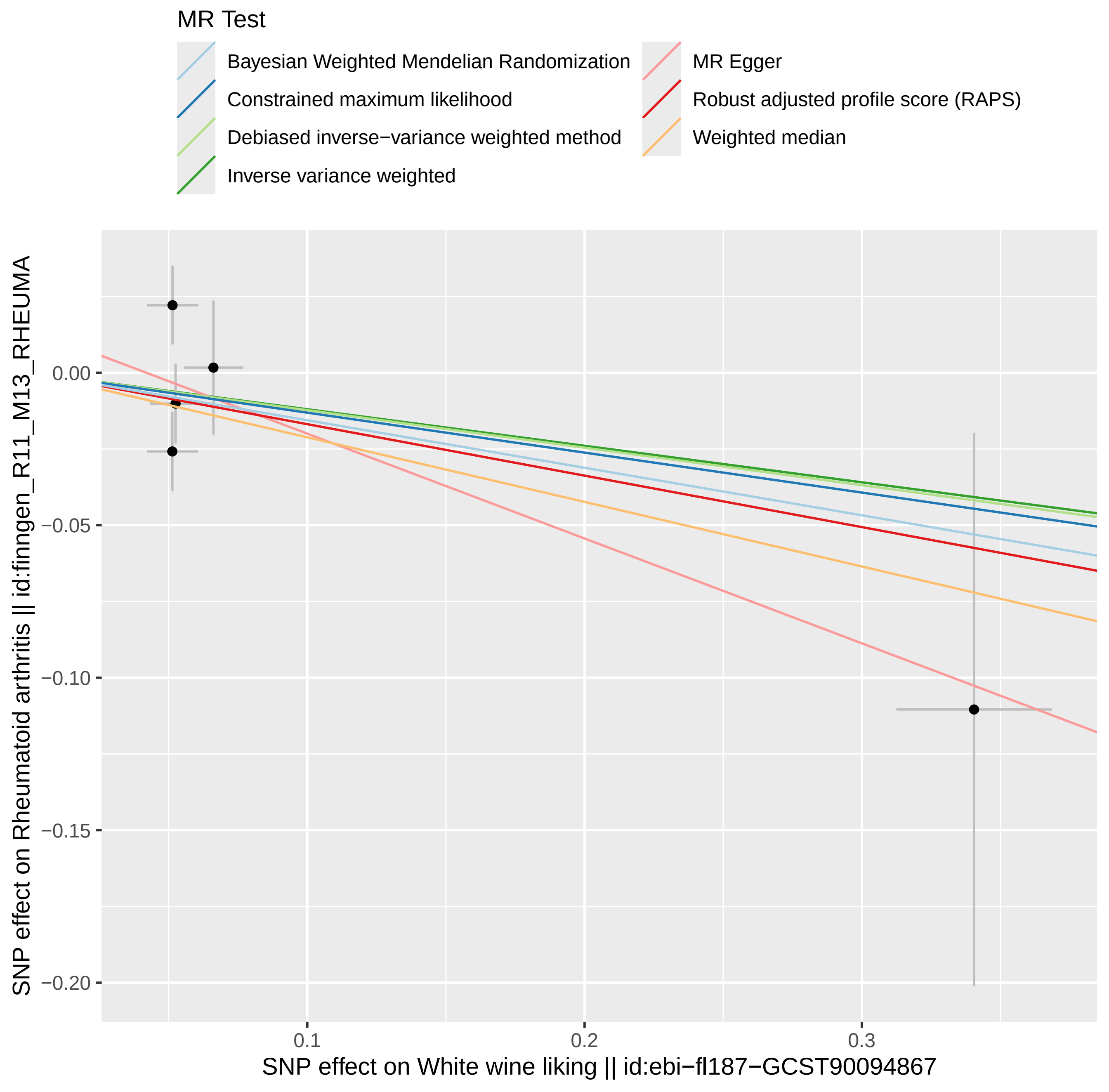

MR Test
Bayesian Weighted Mendelian Randomization
Constrained maximum likelihood
Debiased inverse−variance weighted method
Inverse variance weighted
MR Egger
Robust adjusted profile score (RAPS)
Weighted median
0.00
−0.05
−0.10
−0.15
−0.20
0.1
0.2
0.3
SNP effect on Rheumatoid arthritis || id:finngen_R11_M13_RHEUMA
SNP effect on White wine liking || id:ebi−fl187−GCST90094867

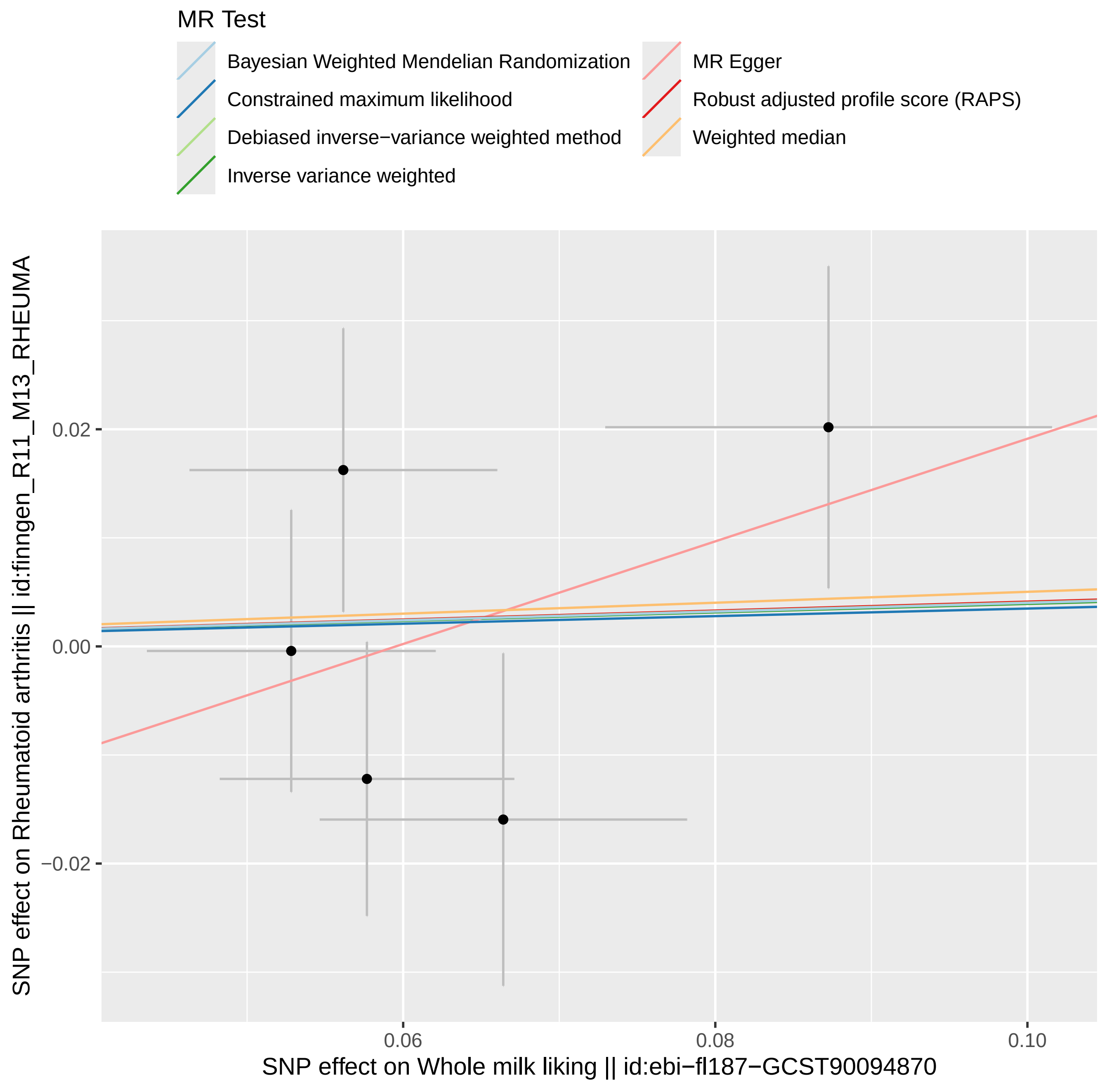

MR Test
Bayesian Weighted Mendelian Randomization
Constrained maximum likelihood
Debiased inverse−variance weighted method
Inverse variance weighted
MR Egger
Robust adjusted profile score (RAPS)
Weighted median
SNP effect on Rheumatoid arthritis || id:finngen_R11_M13_RHEUMA
0.02
0.00
−0.02
0.06
0.08
0.10
SNP effect on Whole milk liking || id:ebi−fl187−GCST90094870

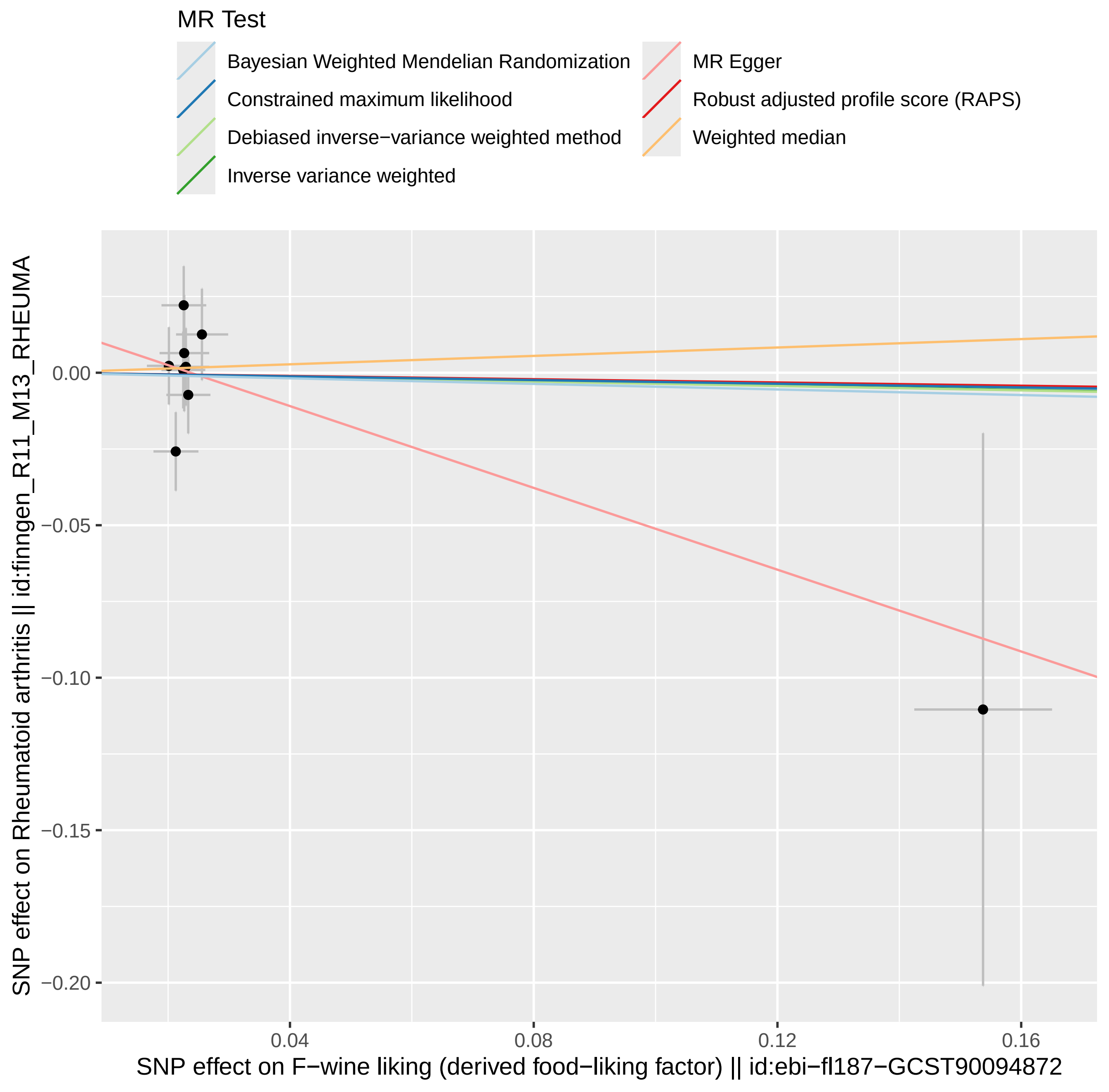

MR Test
Bayesian Weighted Mendelian Randomization
Constrained maximum likelihood
Debiased inverse−variance weighted method
Inverse variance weighted
MR Egger
Robust adjusted profile score (RAPS)
Weighted median
SNP effect on Rheumatoid arthritis || id:finngen_R11_M13_RHEUMA
0.00
−0.05
−0.10
−0.15
−0.20
0.04
0.08
0.12
0.16
SNP effect on F−wine liking (derived food−liking factor) || id:ebi−fl187−GCST90094872

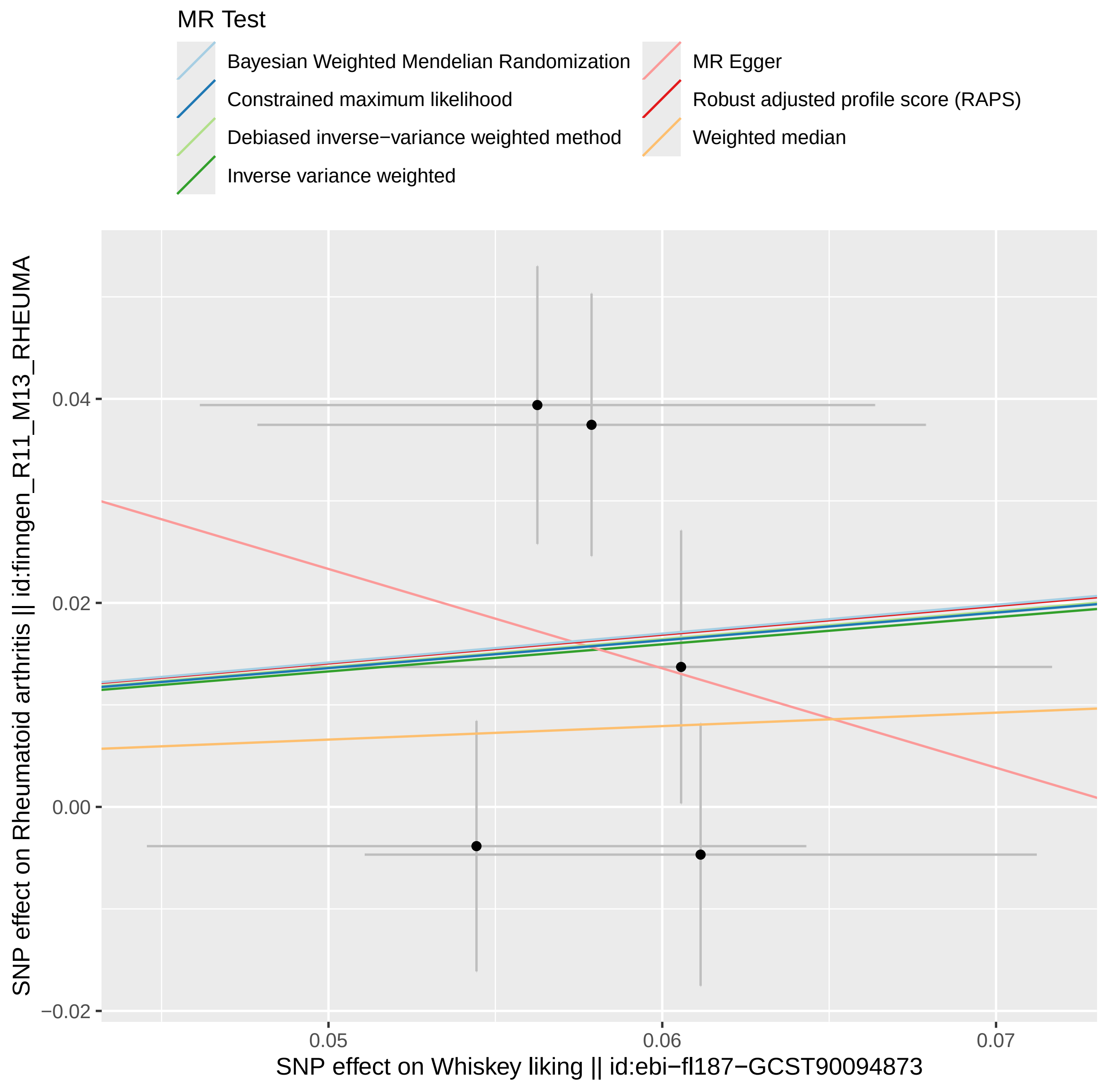
MR Test
Bayesian Weighted Mendelian Randomization
Constrained maximum likelihood
Debiased inverse−variance weighted method
Inverse variance weighted
MR Egger
Robust adjusted profile score (RAPS)
Weighted median
SNP effect on Rheumatoid arthritis || id:finngen_R11_M13_RHEUMA
0.04
0.02
0.00
−0.02
0.05
0.06
0.07
SNP effect on Whiskey liking || id:ebi−fl187−GCST90094873

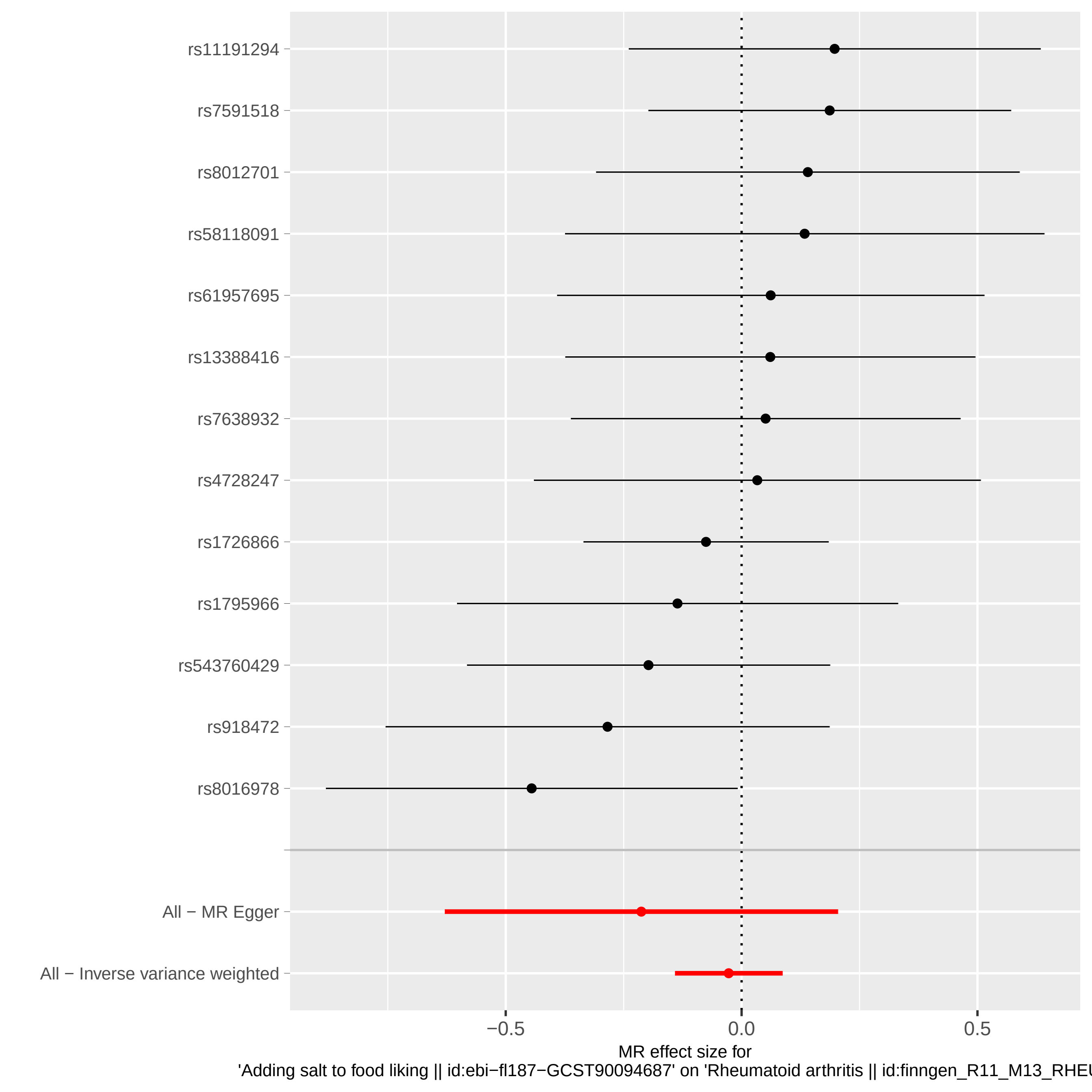
rs11191294
rs7591518
rs8012701
rs58118091
rs61957695
rs13388416
rs7638932
rs4728247
rs1726866
rs1795966
rs543760429
rs918472
rs8016978
All − MR Egger
All − Inverse variance weighted
−0.5
0.0
0.5
MR effect size for
'Adding salt to food liking || id:ebi−fl187−GCST90094687' on 'Rheumatoid arthritis || id:finngen_R11_M13_RHEU

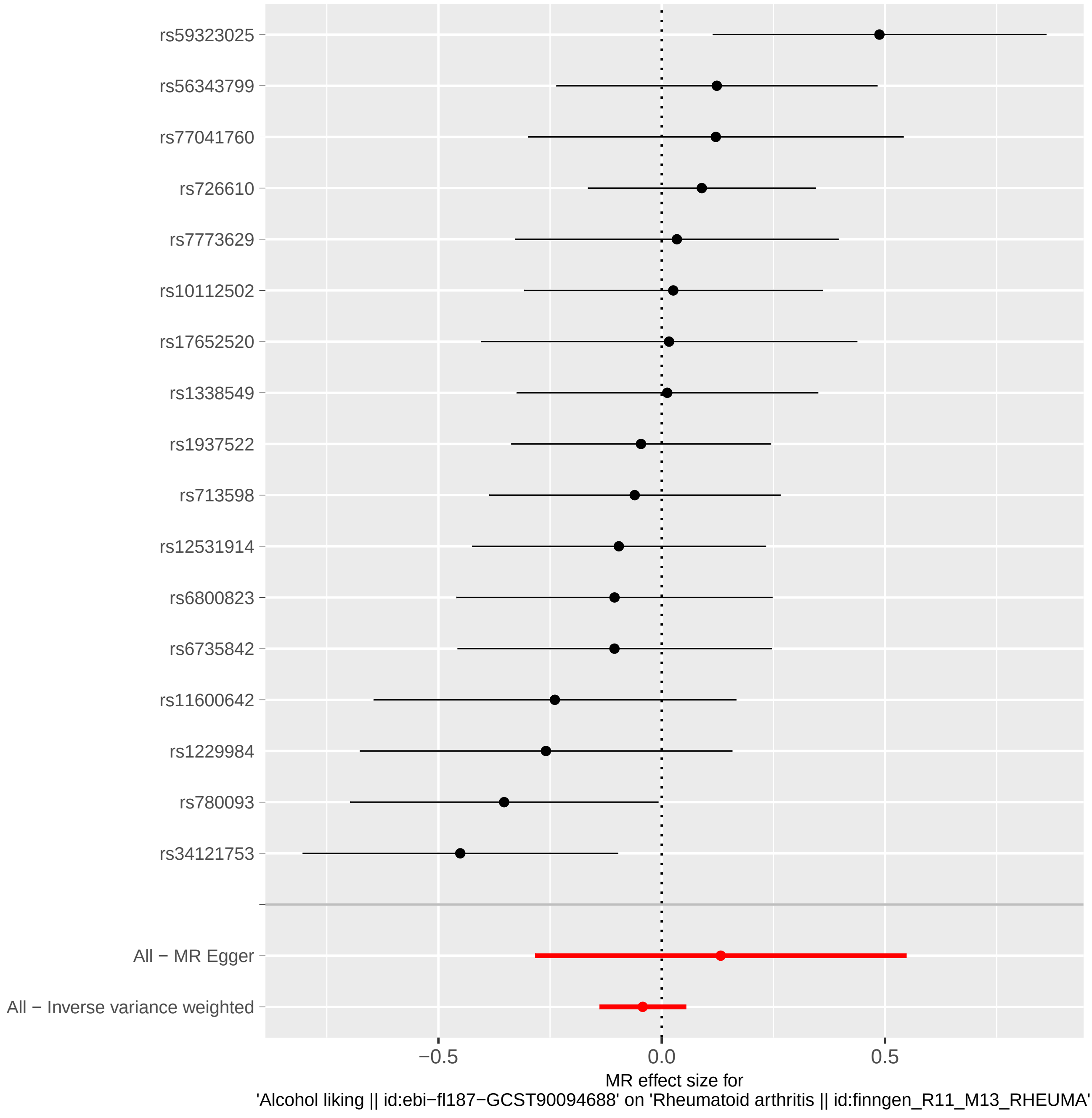
rs59323025
rs56343799
rs77041760
rs726610
rs7773629
rs10112502
rs17652520
rs1338549
rs1937522
rs713598
rs12531914
rs6800823
rs6735842
rs11600642
rs1229984
rs780093
rs34121753
All − MR Egger
All − Inverse variance weighted
−0.5
0.0
0.5
MR effect size for
'Alcohol liking || id:ebi−fl187−GCST90094688' on 'Rheumatoid arthritis || id:finngen_R11_M13_RHEUMA'

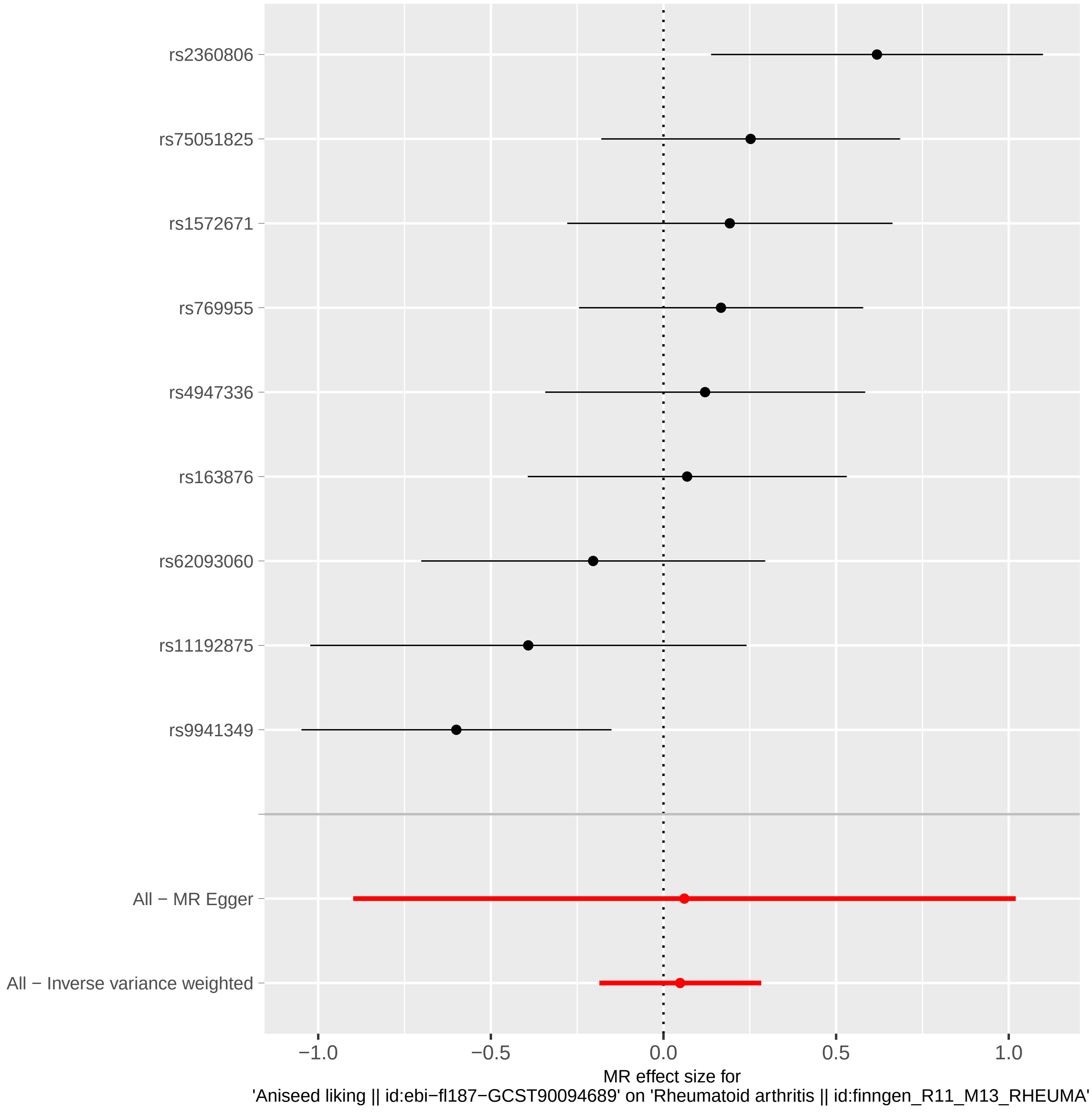
rs2360806
rs75051825
rs1572671
rs769955
rs4947336
rs163876
rs62093060
rs11192875
rs9941349
All − MR Egger
All − Inverse variance weighted
−1.0
−0.5
0.0
0.5
1.0
MR effect size for
'Aniseed liking || id:ebi−fl187−GCST90094689' on 'Rheumatoid arthritis || id:finngen_R11_M13_RHEUMA'

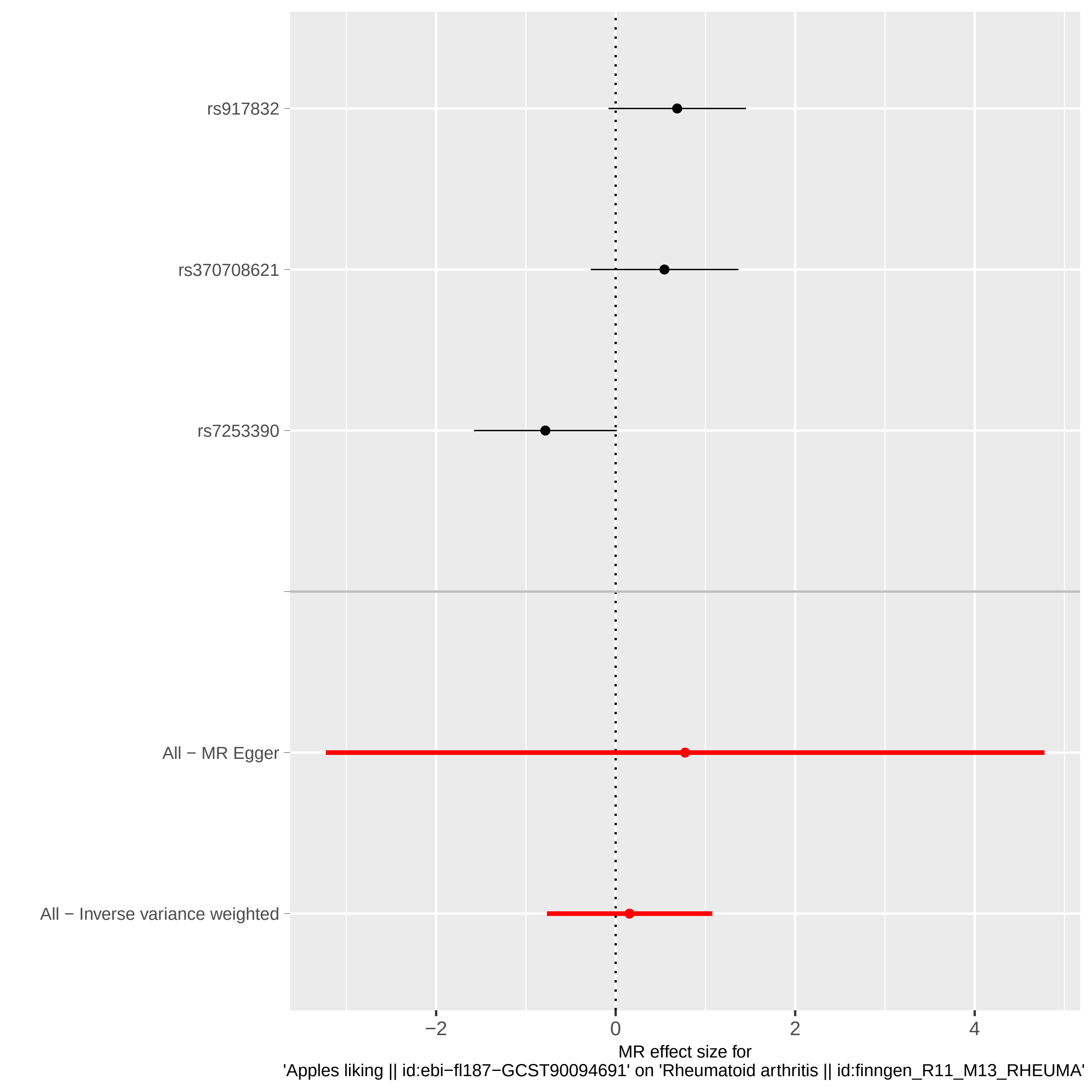

rs917832
rs370708621
rs7253390
All − MR Egger
All − Inverse variance weighted
−2
0
2
4
MR effect size for
'Apples liking || id:ebi−fl187−GCST90094691' on 'Rheumatoid arthritis || id:finngen_R11_M13_RHEUMA'

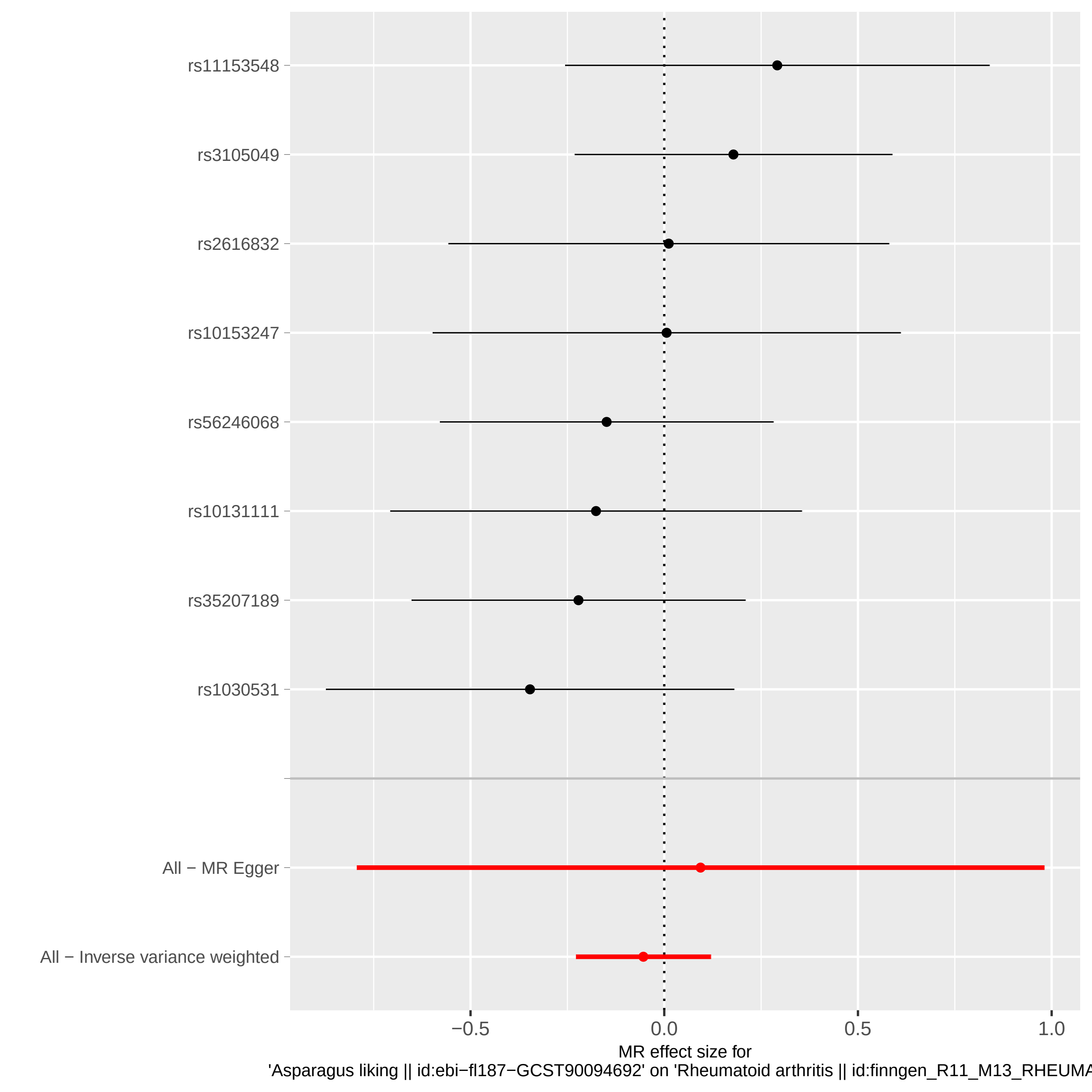

rs11153548
rs3105049
rs2616832
rs10153247
rs56246068
rs10131111
rs35207189
rs1030531
All − MR Egger
All − Inverse variance weighted
−0.5
0.0
0.5
1.0
MR effect size for
'Asparagus liking || id:ebi−fl187−GCST90094692' on 'Rheumatoid arthritis || id:finngen_R11_M13_RHEUMA

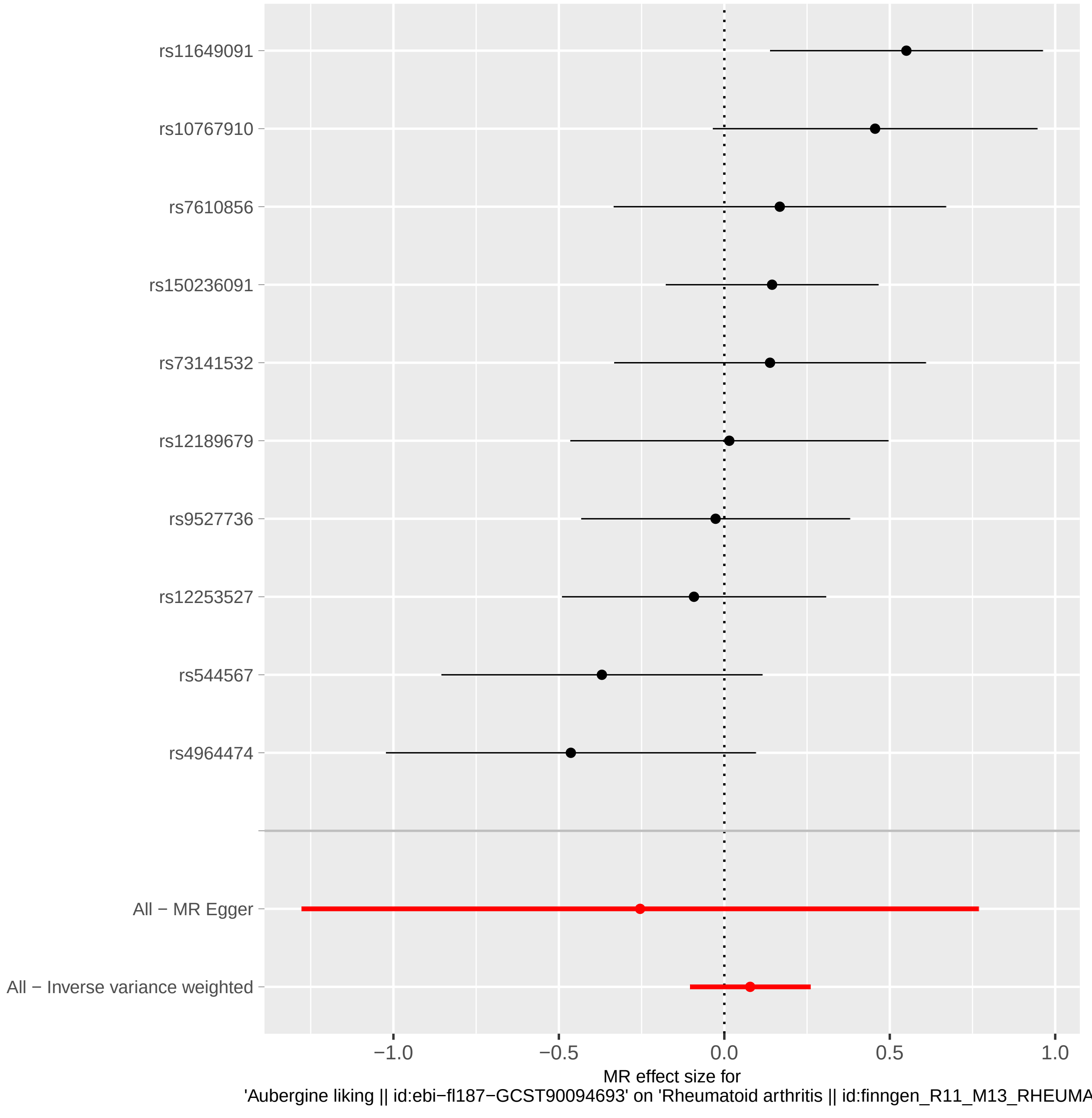
rs11649091
rs10767910
rs7610856
rs150236091
rs73141532
rs12189679
rs9527736
rs12253527
rs544567
rs4964474
All − MR Egger
All − Inverse variance weighted
−1.0
−0.5
0.0
0.5
1.0
MR effect size for
'Aubergine liking || id:ebi−fl187−GCST90094693' on 'Rheumatoid arthritis || id:finngen_R11_M13_RHEUMA

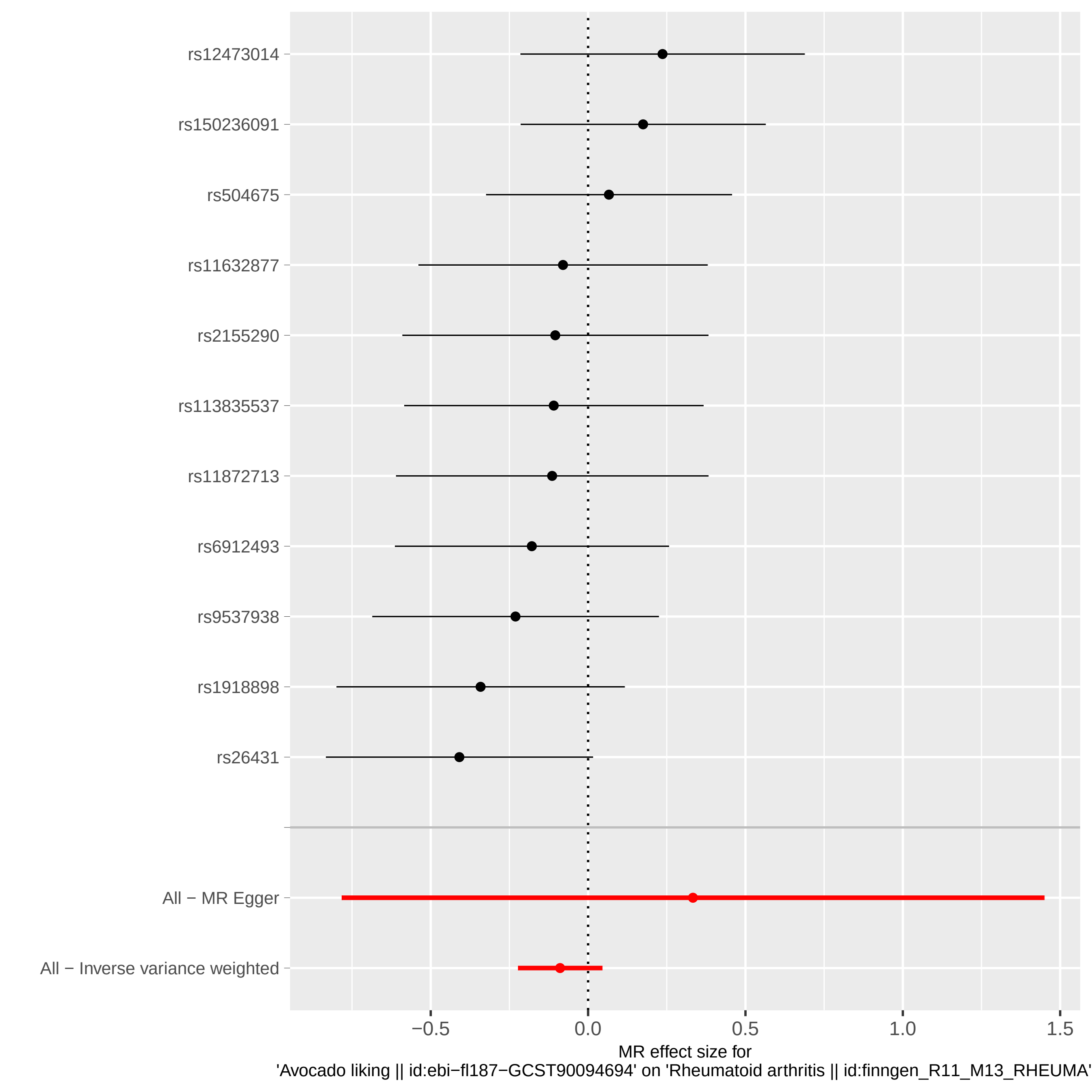
rs12473014
rs150236091
rs504675
rs11632877
rs2155290
rs113835537
rs11872713
rs6912493
rs9537938
rs1918898
rs26431
All − MR Egger
All − Inverse variance weighted
−0.5
0.0
0.5
1.0
1.5
MR effect size for
'Avocado liking || id:ebi−fl187−GCST90094694' on 'Rheumatoid arthritis || id:finngen_R11_M13_RHEUMA'

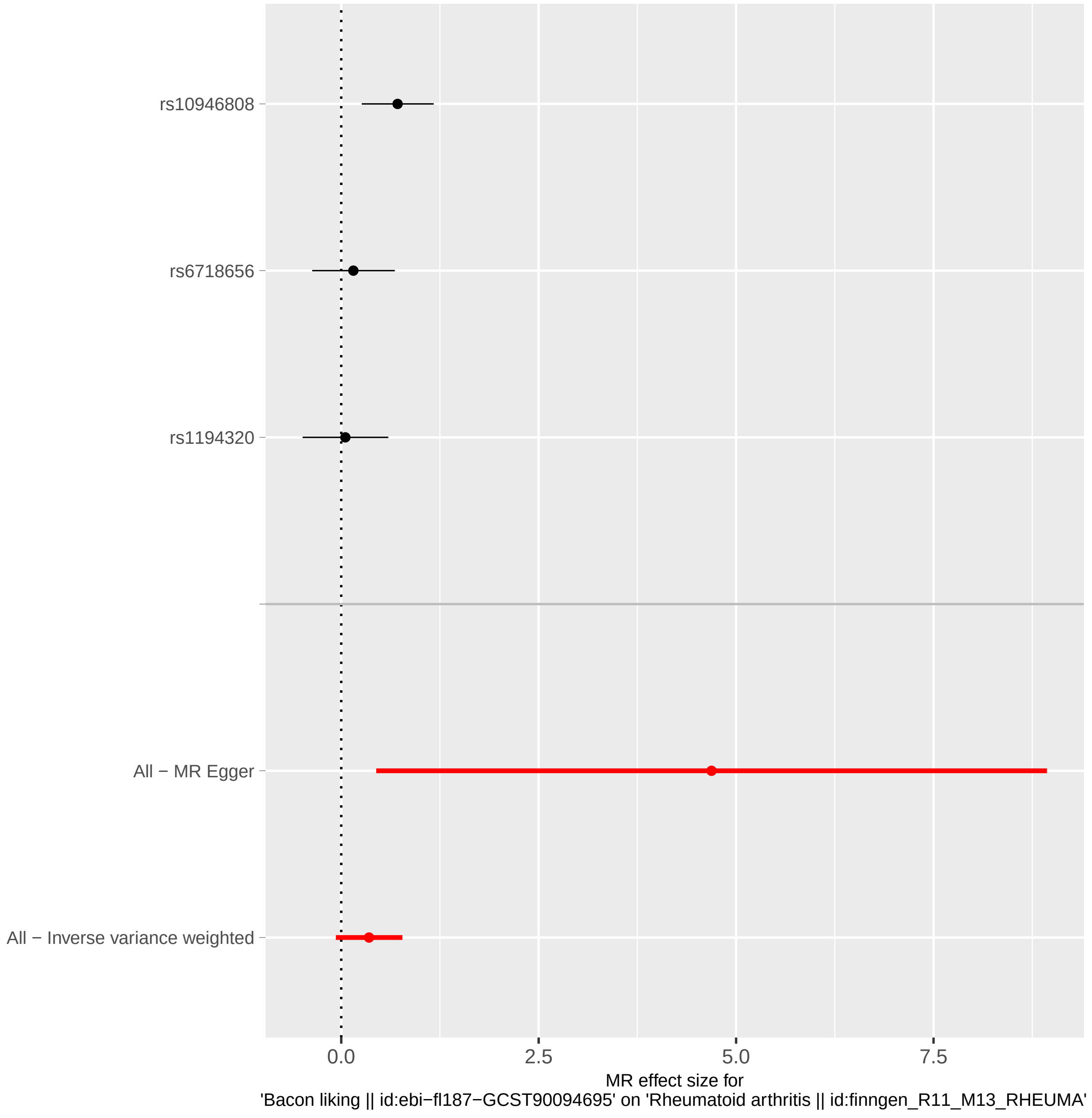
rs10946808
rs6718656
rs1194320
All − MR Egger
All − Inverse variance weighted
0.0
2.5
5.0
7.5
MR effect size for
'Bacon liking || id:ebi−fl187−GCST90094695' on 'Rheumatoid arthritis || id:finngen_R11_M13_RHEUMA'

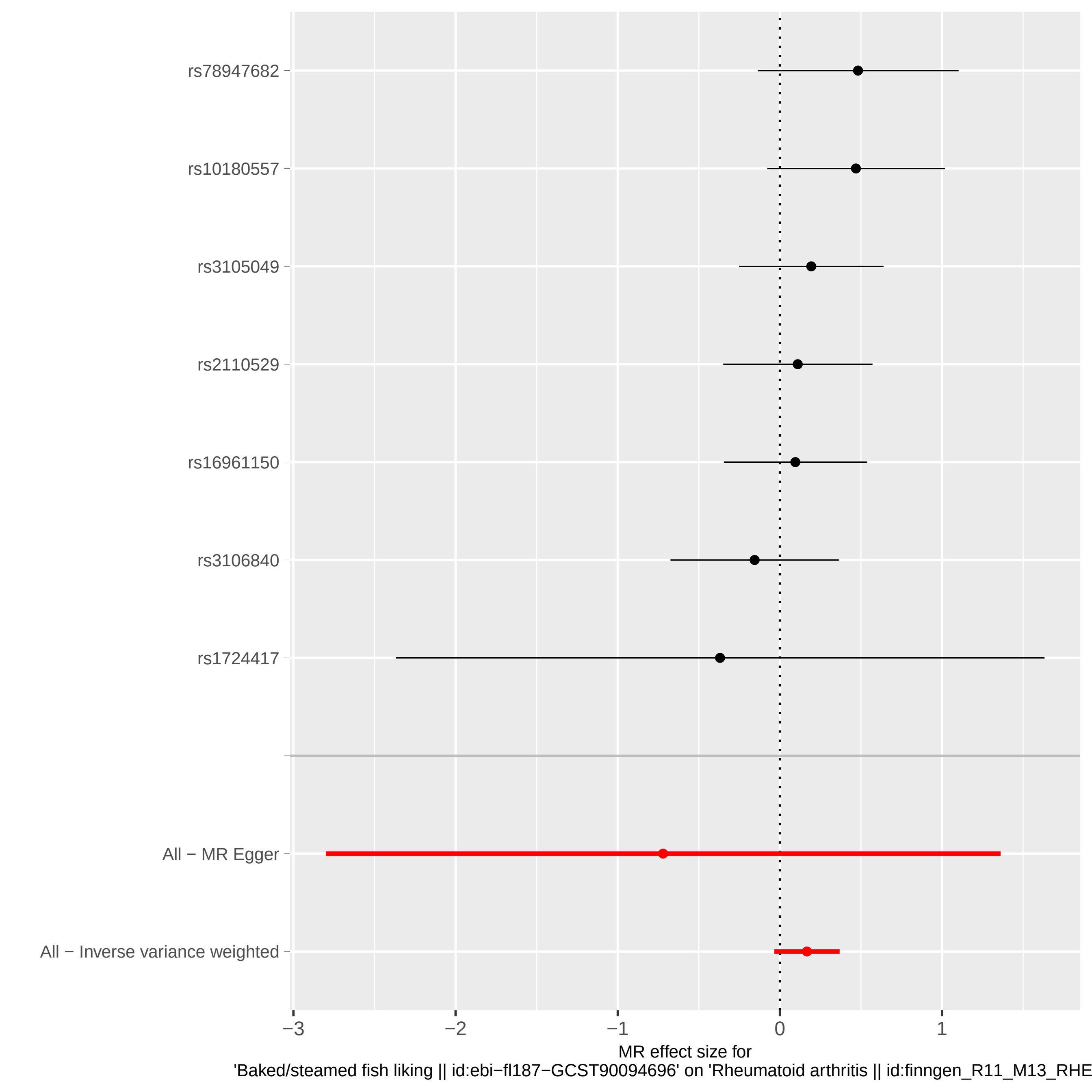
rs78947682
rs10180557
rs3105049
rs2110529
rs16961150
rs3106840
rs1724417
All − MR Egger
All − Inverse variance weighted
−3
−2
−1
0
1
MR effect size for
'Baked/steamed fish liking || id:ebi−fl187−GCST90094696' on 'Rheumatoid arthritis || id:finngen_R11_M13_RHE

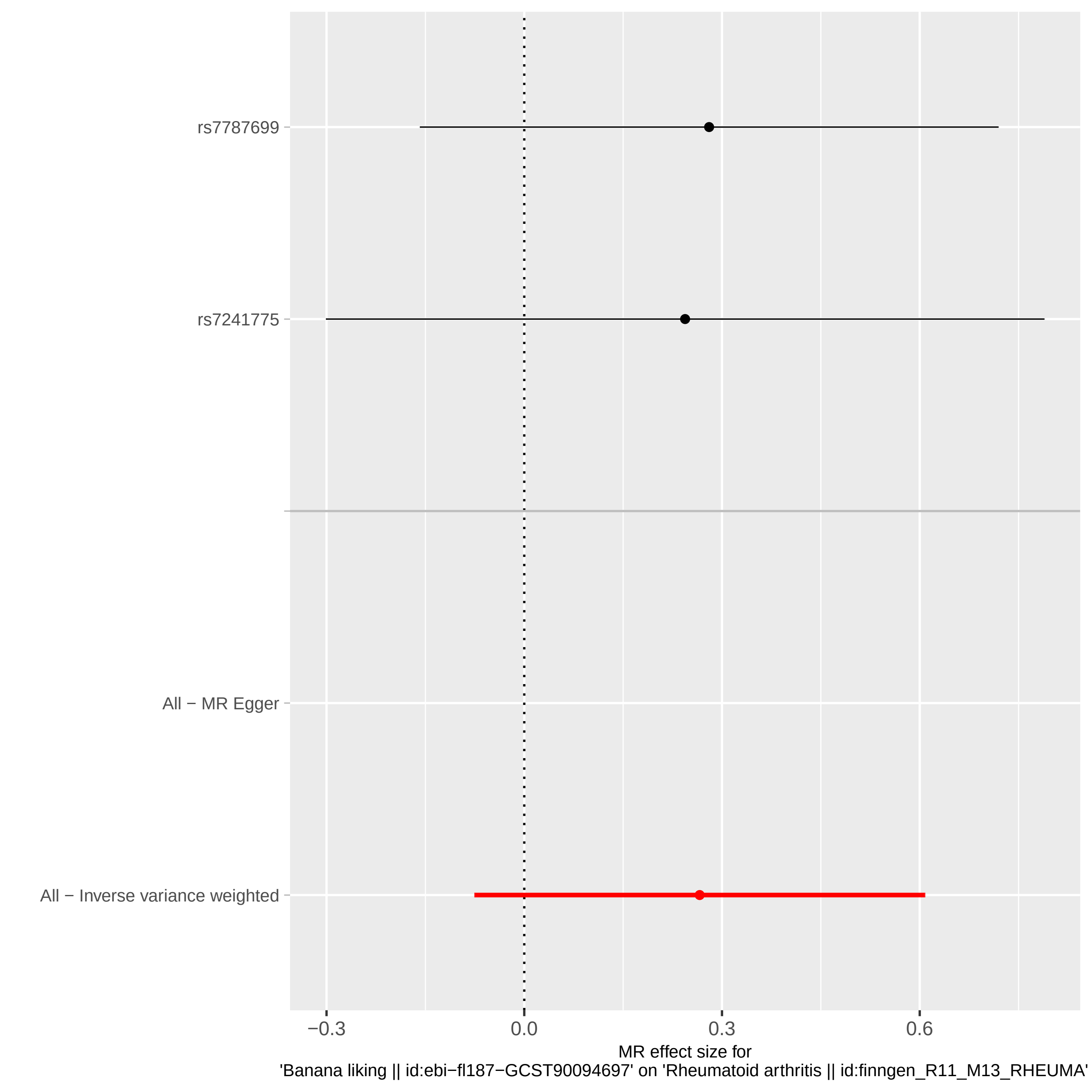
rs7787699
rs7241775
All − MR Egger
All − Inverse variance weighted
−0.3
0.0
0.3
0.6
MR effect size for
'Banana liking || id:ebi−fl187−GCST90094697' on 'Rheumatoid arthritis || id:finngen_R11_M13_RHEUMA'

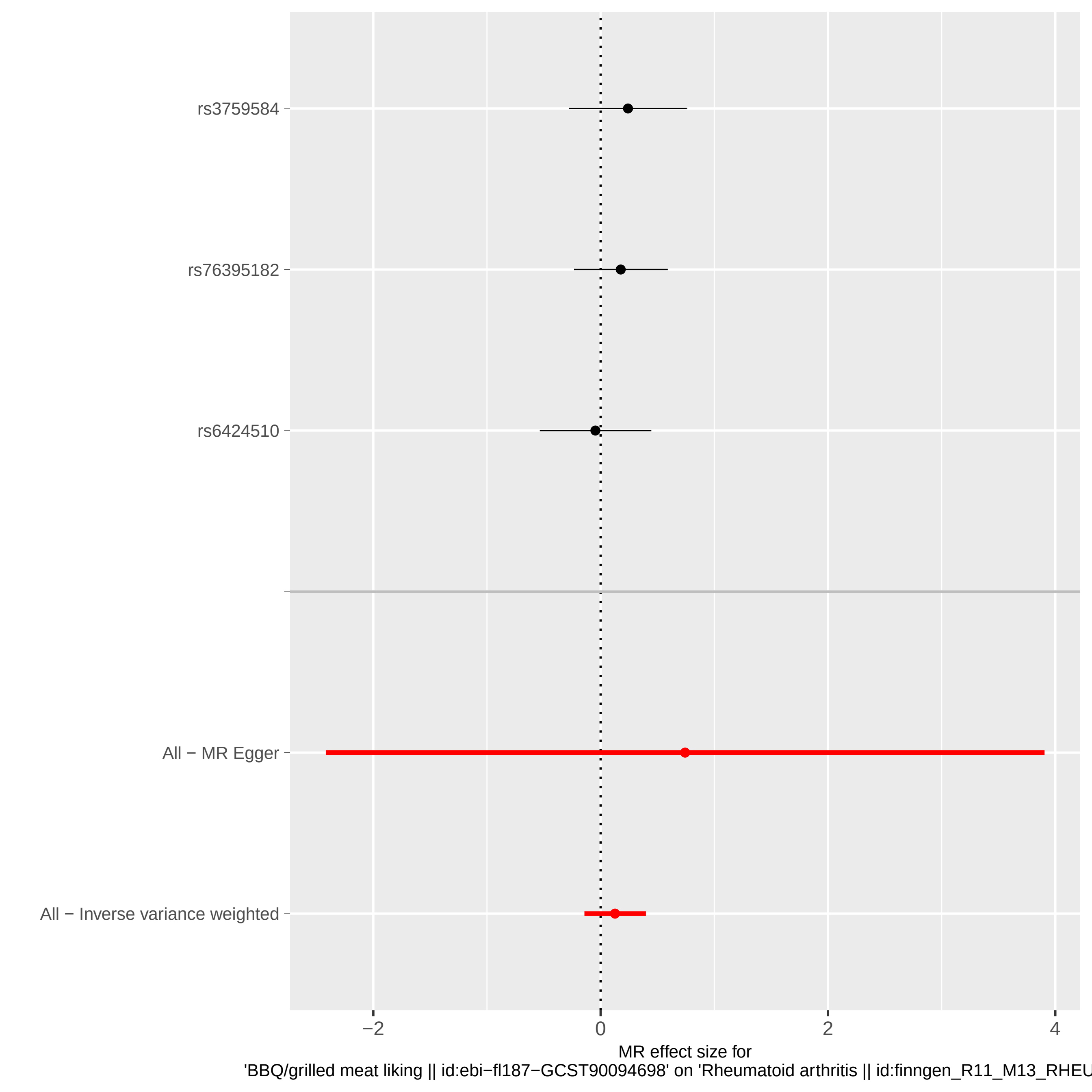
rs3759584
rs76395182
rs6424510
All − MR Egger
All − Inverse variance weighted
−2
0
2
4
MR effect size for
'BBQ/grilled meat liking || id:ebi−fl187−GCST90094698' on 'Rheumatoid arthritis || id:finngen_R11_M13_RHEU

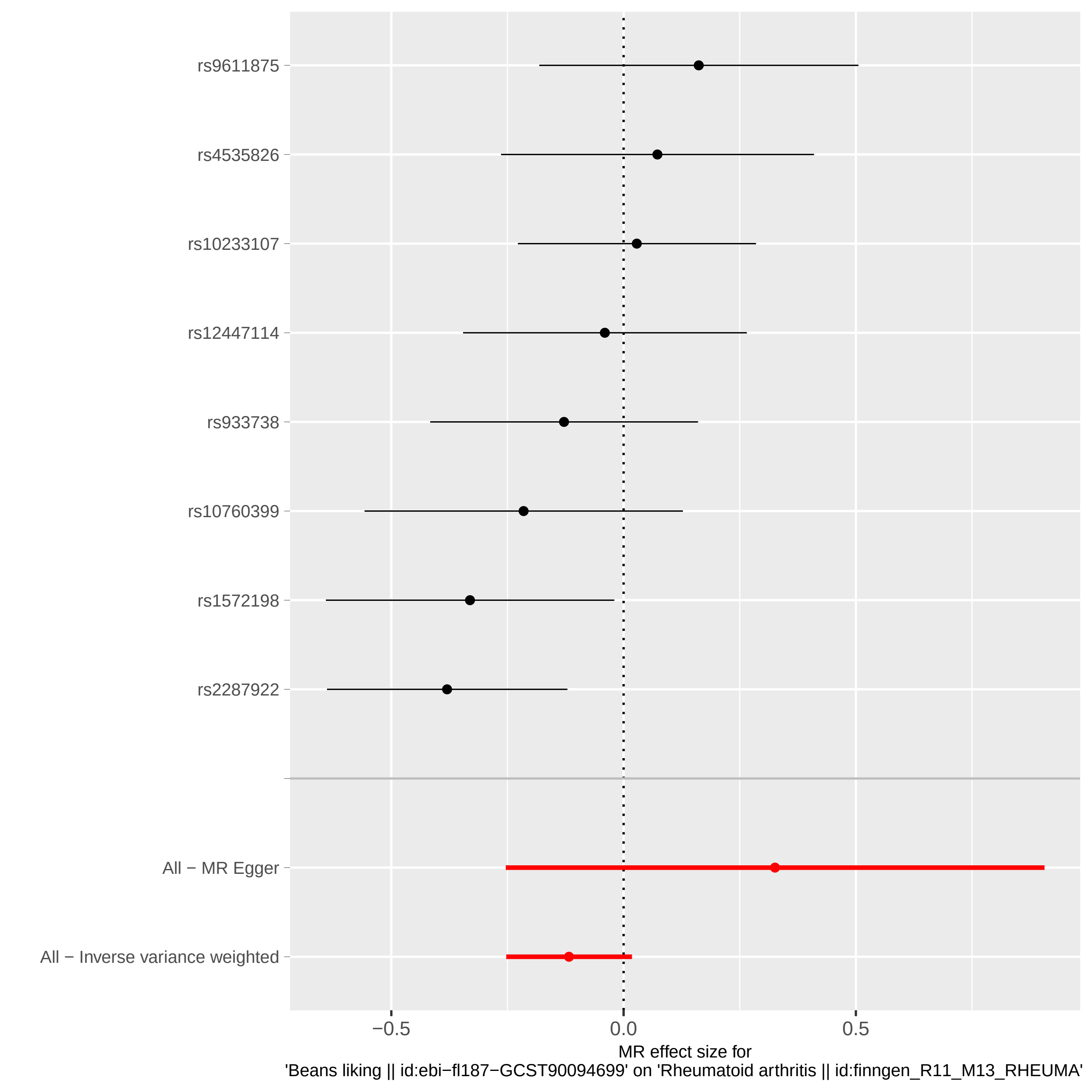
rs9611875
rs4535826
rs10233107
rs12447114
rs933738
rs10760399
rs1572198
rs2287922
All − MR Egger
All − Inverse variance weighted
−0.5
0.0
0.5
MR effect size for
'Beans liking || id:ebi−fl187−GCST90094699' on 'Rheumatoid arthritis || id:finngen_R11_M13_RHEUMA'

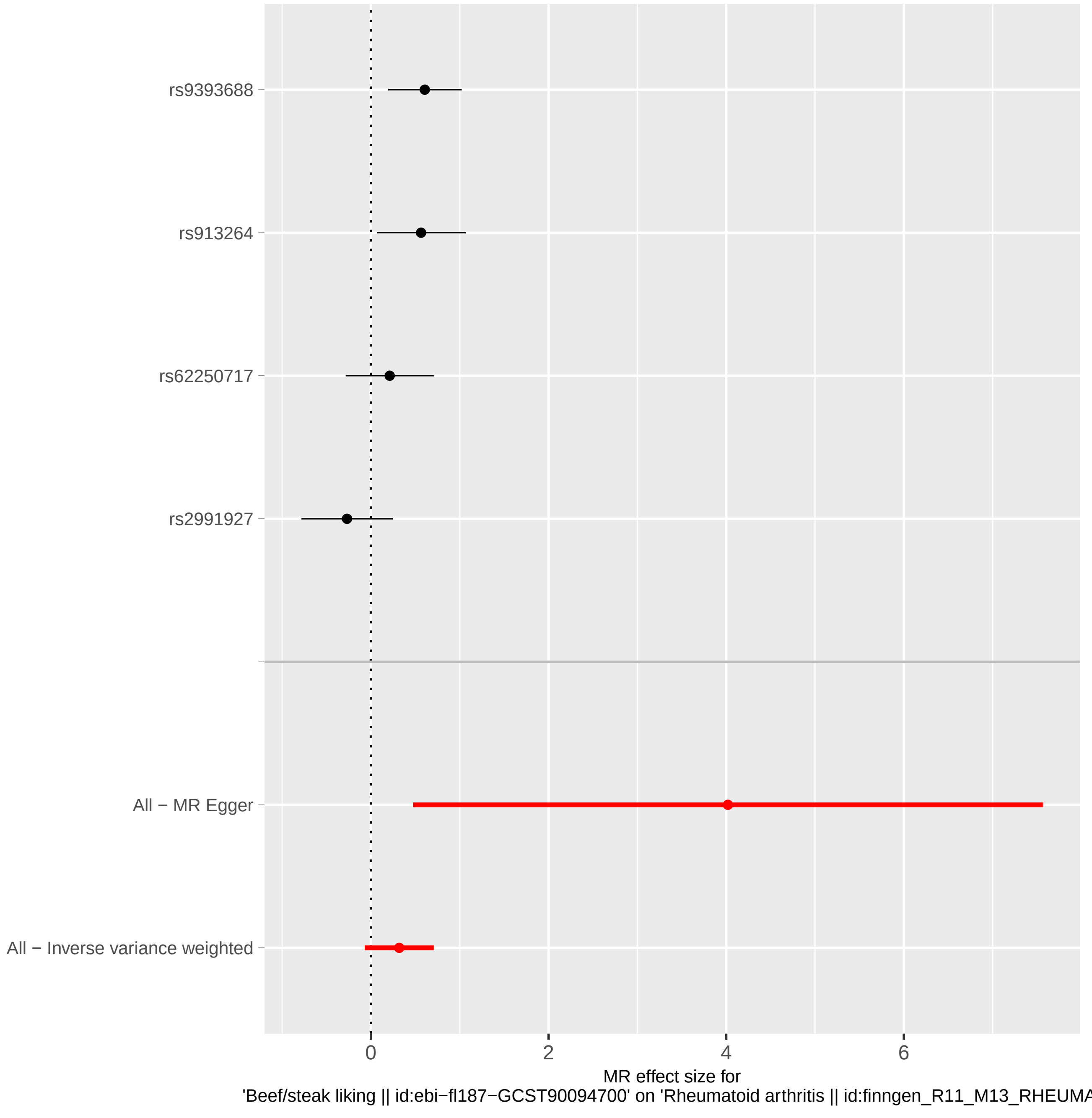
rs9393688
rs913264
rs62250717
rs2991927
All − MR Egger
All − Inverse variance weighted
0
2
4
6
MR effect size for
'Beef/steak liking || id:ebi−fl187−GCST90094700' on 'Rheumatoid arthritis || id:finngen_R11_M13_RHEUMA

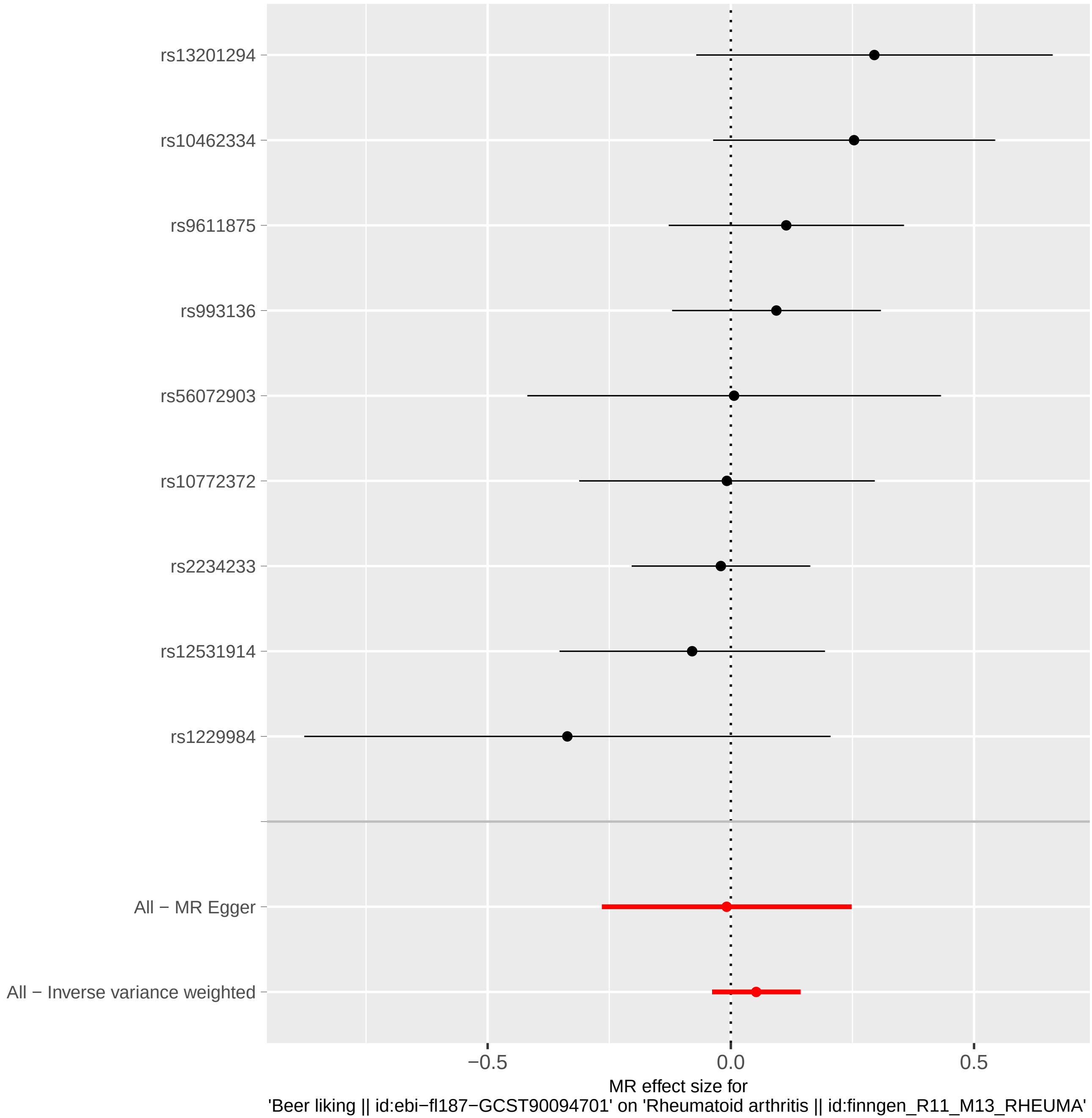
rs13201294
rs10462334
rs9611875
rs993136
rs56072903
rs10772372
rs2234233
rs12531914
rs1229984
All − MR Egger
All − Inverse variance weighted
−0.5
0.0
0.5
MR effect size for
'Beer liking || id:ebi−fl187−GCST90094701' on 'Rheumatoid arthritis || id:finngen_R11_M13_RHEUMA'

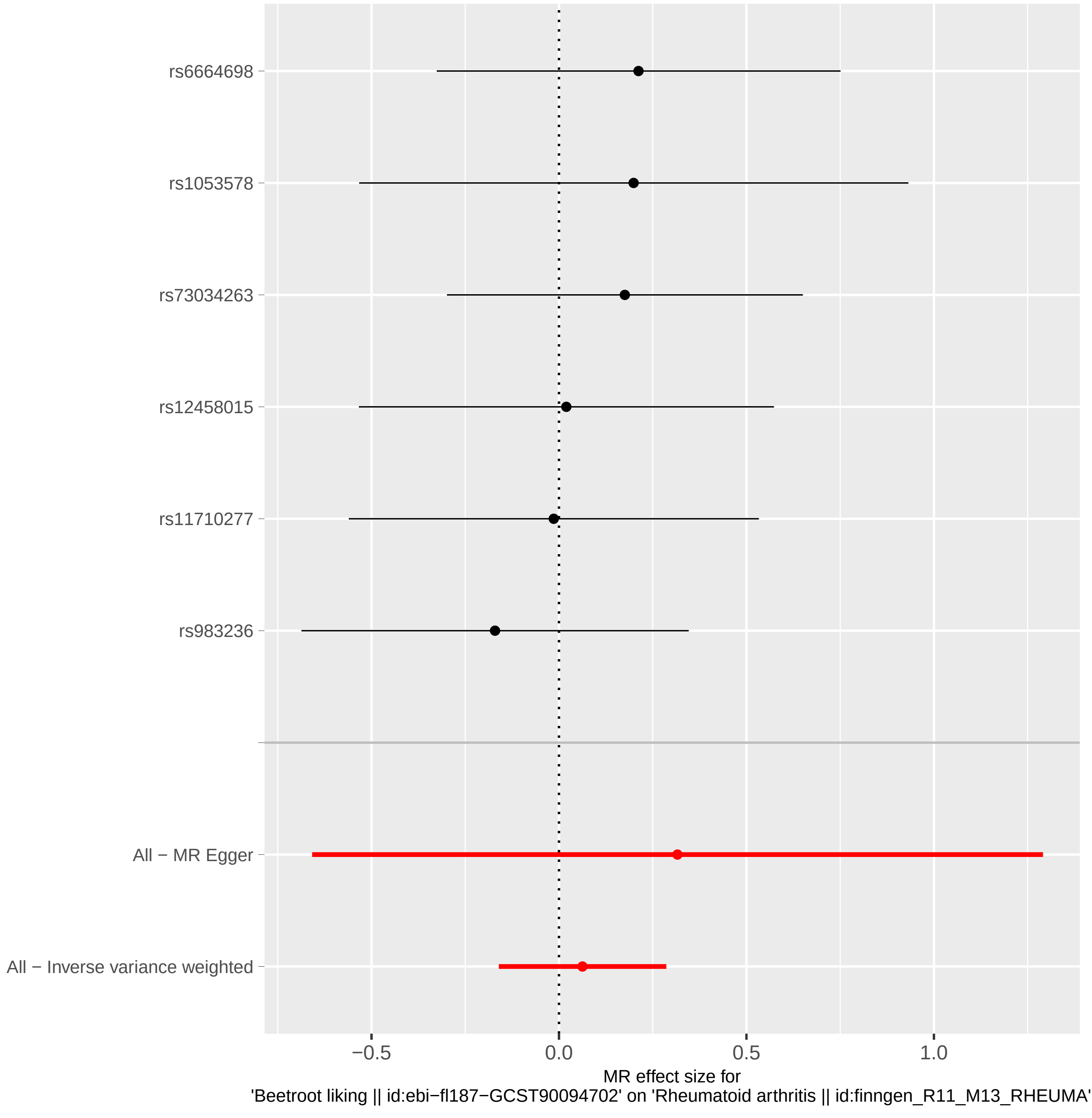
rs6664698
rs1053578
rs73034263
rs12458015
rs11710277
rs983236
All − MR Egger
All − Inverse variance weighted
−0.5
0.0
0.5
1.0
MR effect size for
'Beetroot liking || id:ebi−fl187−GCST90094702' on 'Rheumatoid arthritis || id:finngen_R11_M13_RHEUMA'

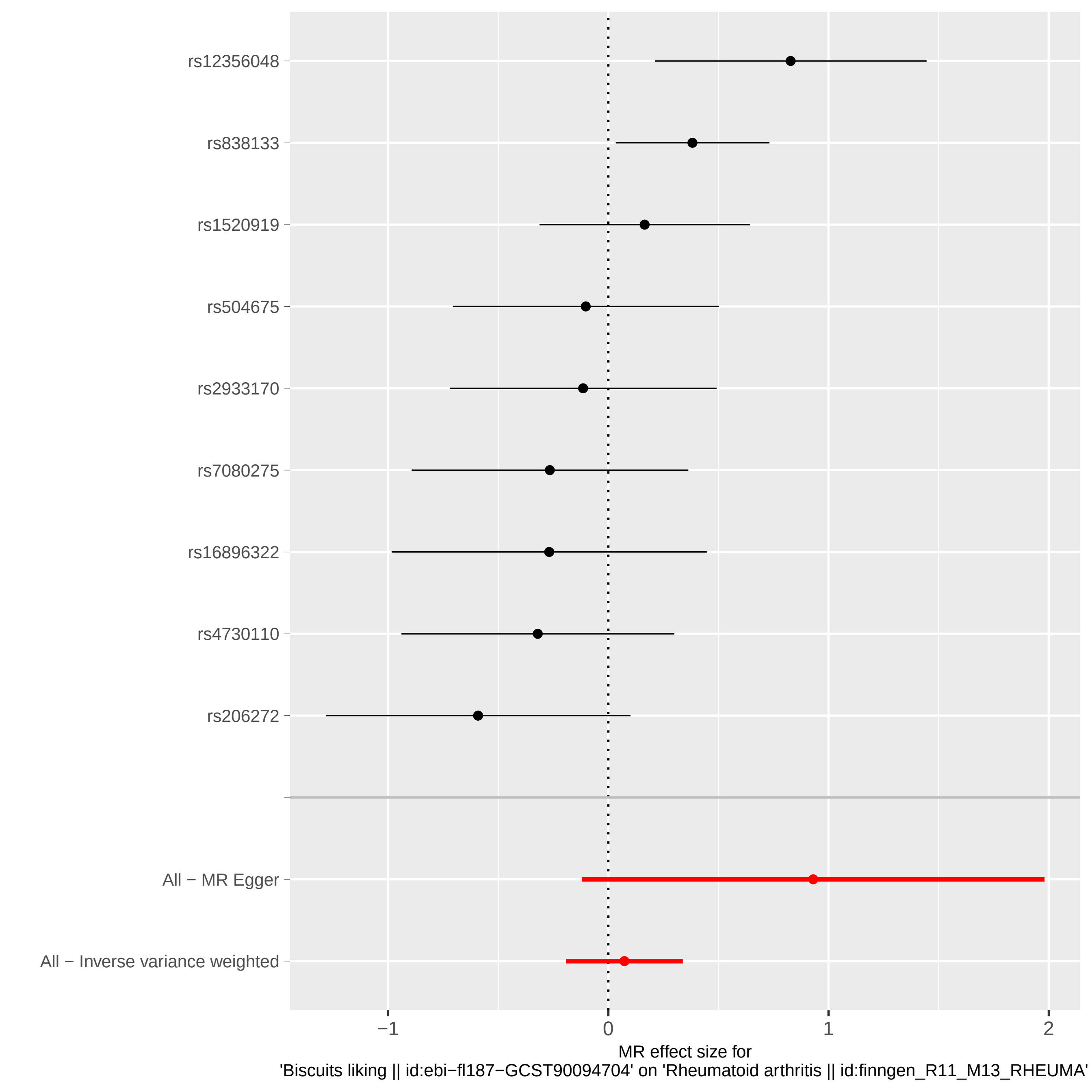

rs12356048
rs838133
rs1520919
rs504675
rs2933170
rs7080275
rs16896322
rs4730110
rs206272
All − MR Egger
All − Inverse variance weighted
−1
0
1
2
MR effect size for
'Biscuits liking || id:ebi−fl187−GCST90094704' on 'Rheumatoid arthritis || id:finngen_R11_M13_RHEUMA'

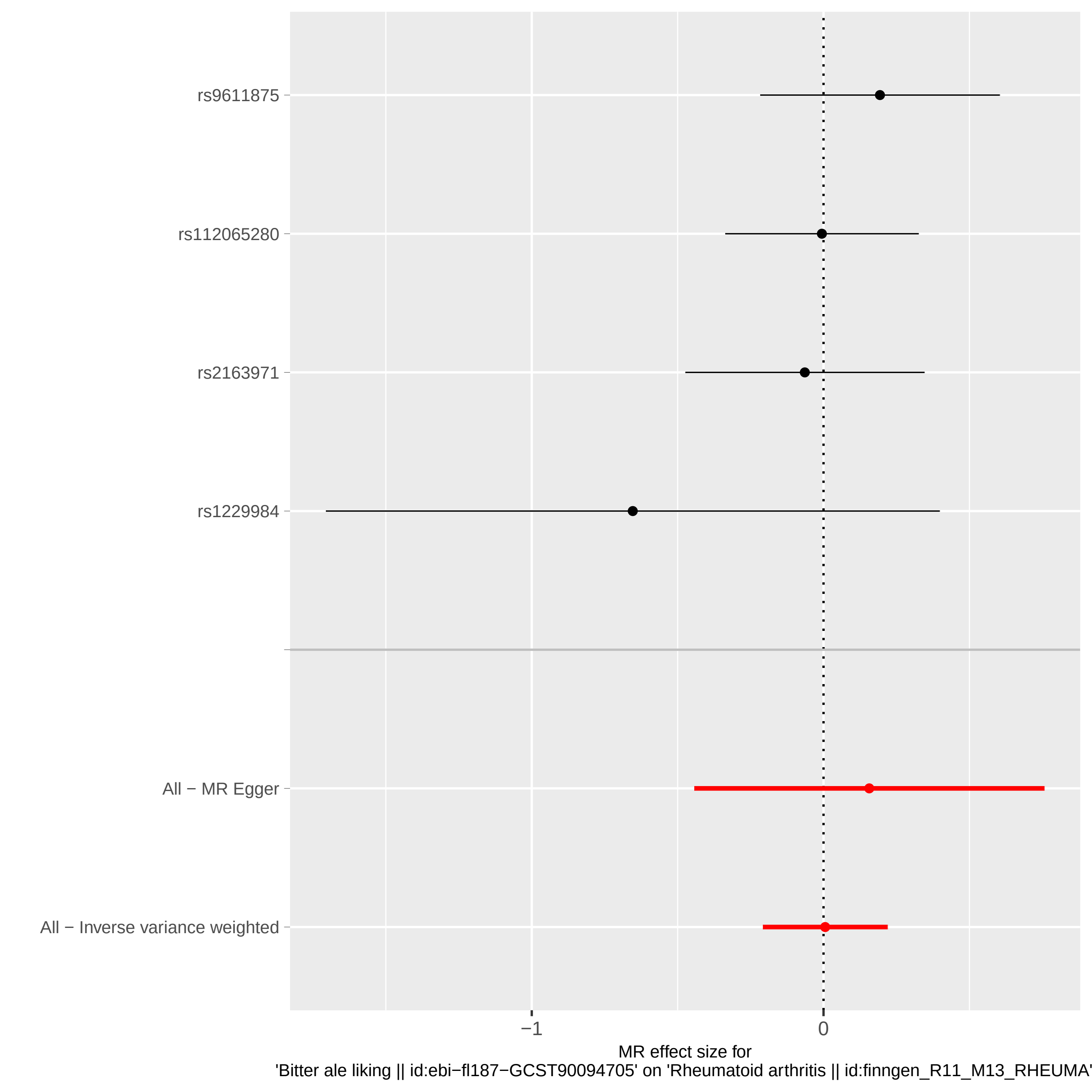
rs9611875
rs112065280
rs2163971
rs1229984
All − MR Egger
All − Inverse variance weighted
−1
0
MR effect size for
'Bitter ale liking || id:ebi−fl187−GCST90094705' on 'Rheumatoid arthritis || id:finngen_R11_M13_RHEUMA'

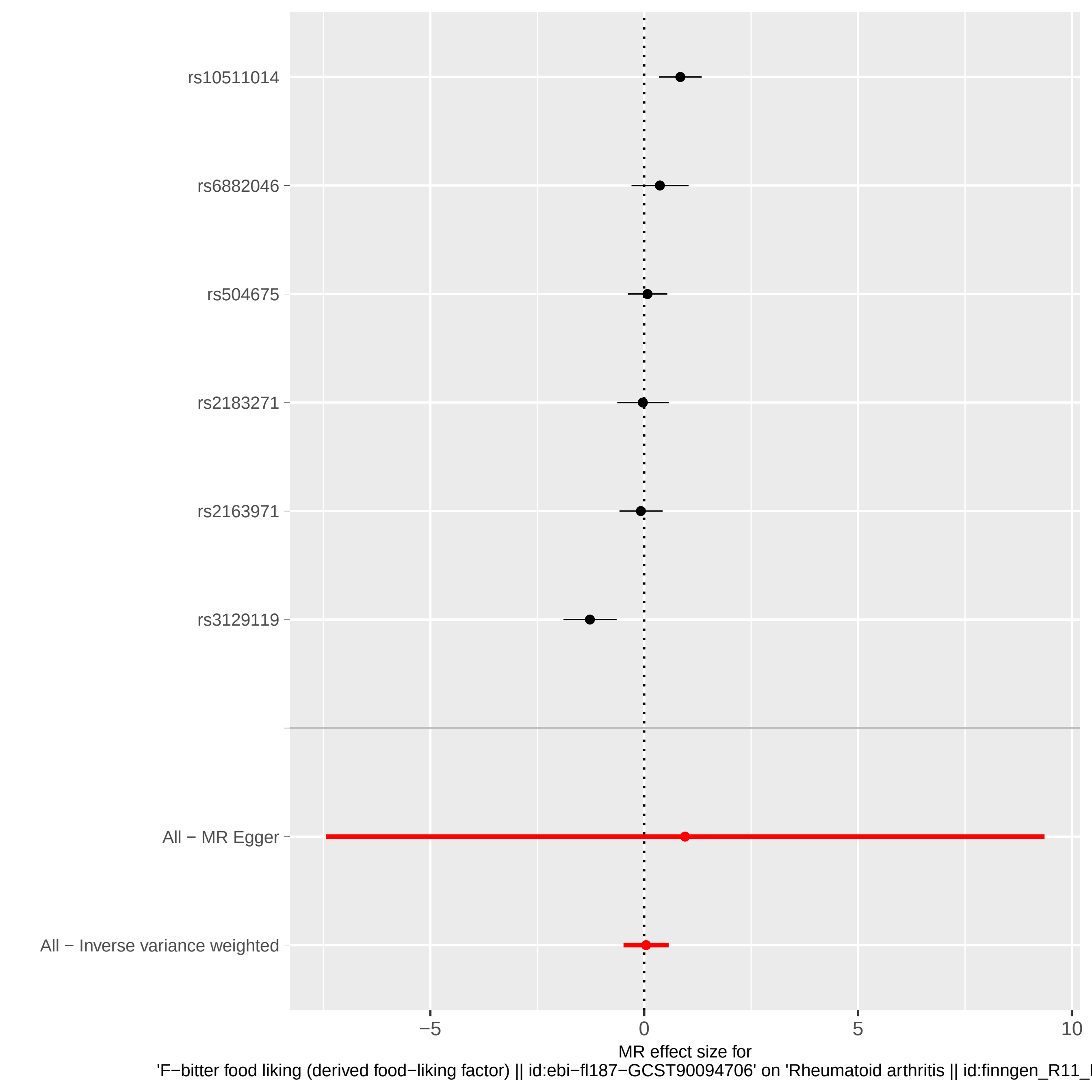

rs10511014
rs6882046
rs504675
rs2183271
rs2163971
rs3129119
All − MR Egger
All − Inverse variance weighted
−5
0
5
10
MR effect size for
'F−bitter food liking (derived food−liking factor) || id:ebi−fl187−GCST90094706' on 'Rheumatoid arthritis || id:finngen_R11_

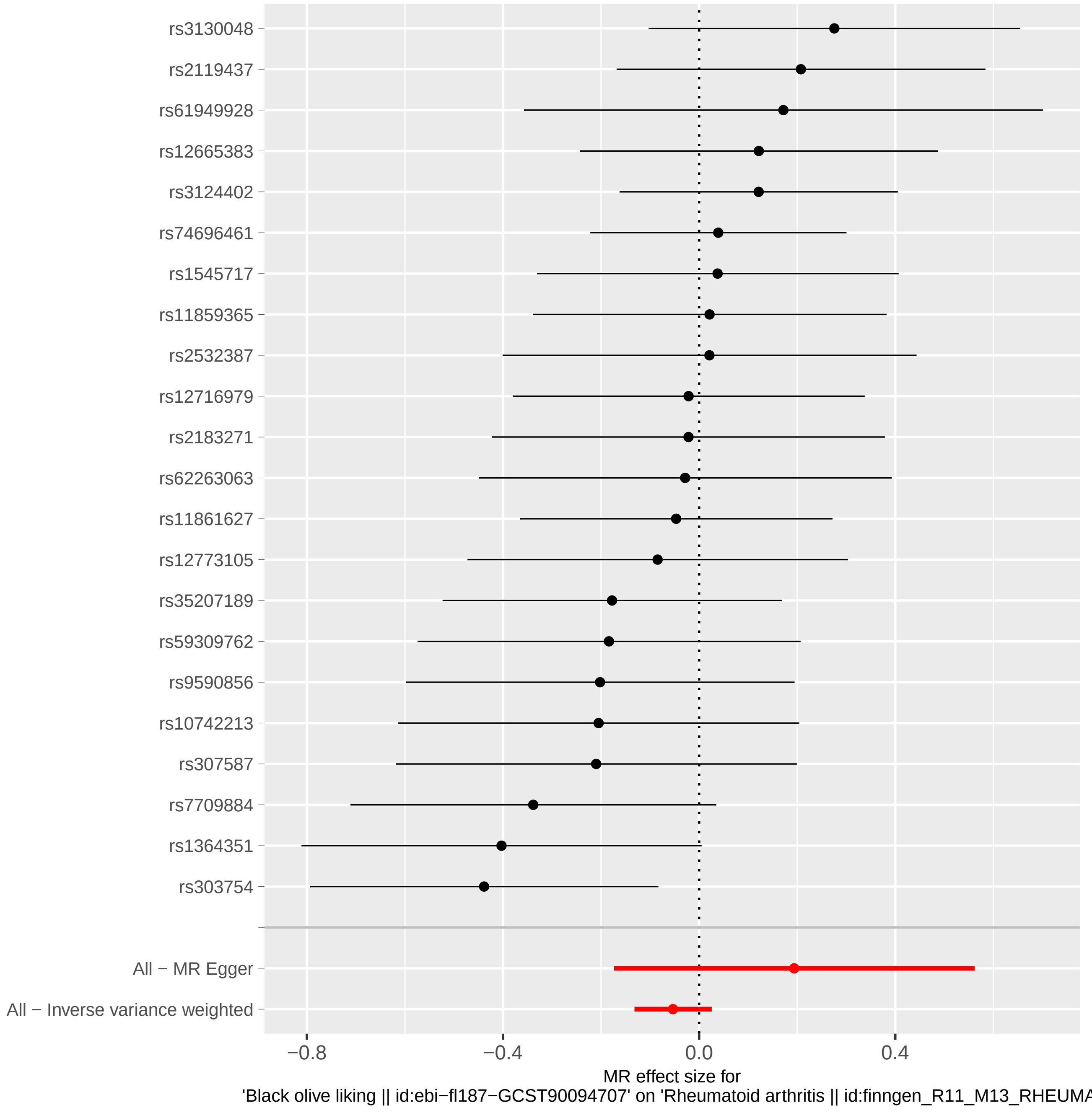
rs3130048
rs2119437
rs61949928
rs12665383
rs3124402
rs74696461
rs1545717
rs11859365
rs2532387
rs12716979
rs2183271
rs62263063
rs11861627
rs12773105
rs35207189
rs59309762
rs9590856
rs10742213
rs307587
rs7709884
rs1364351
rs303754
All − MR Egger
All − Inverse variance weighted
−0.8
−0.4
0.0
0.4
MR effect size for
'Black olive liking || id:ebi−fl187−GCST90094707' on 'Rheumatoid arthritis || id:finngen_R11_M13_RHEUMA

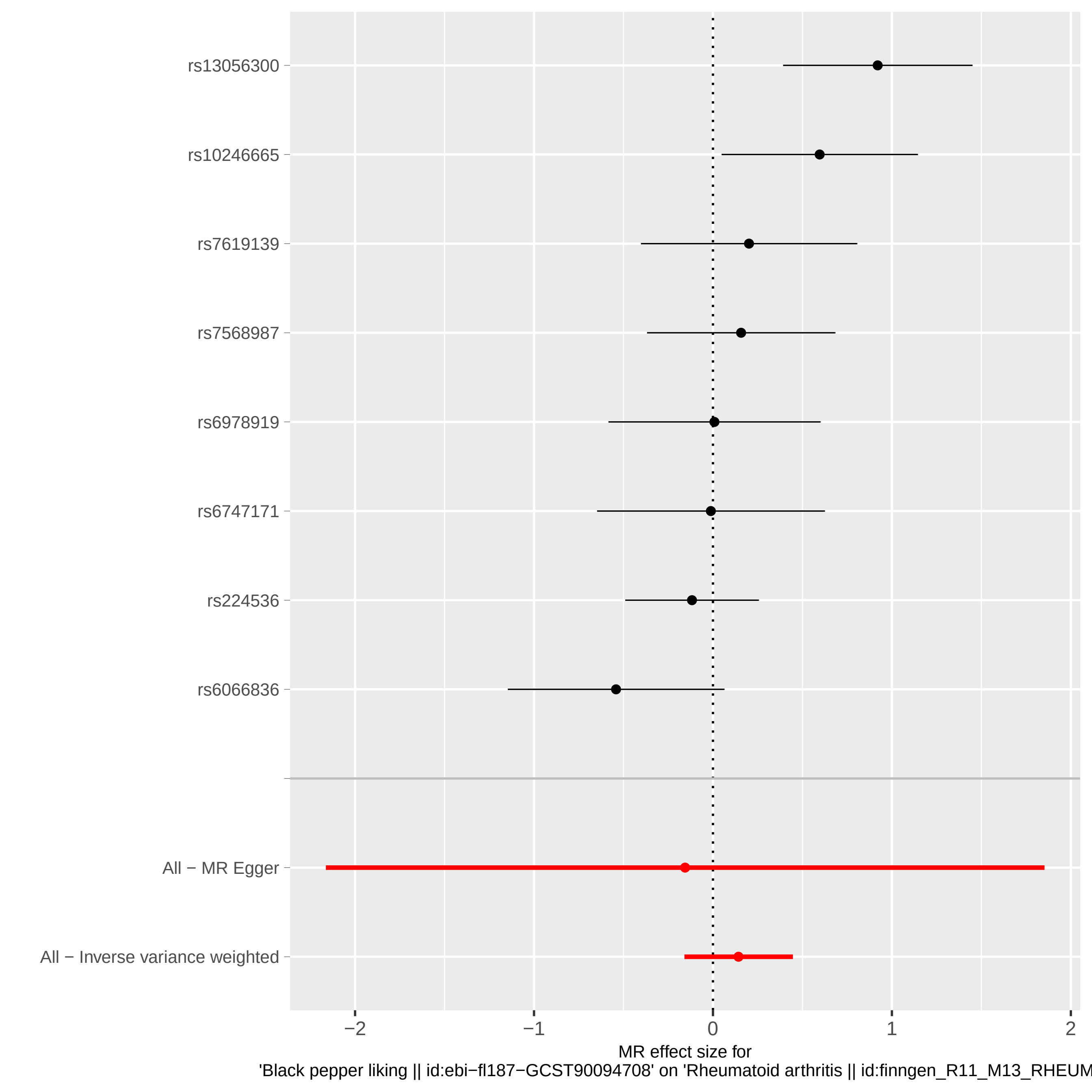
rs13056300
rs10246665
rs7619139
rs7568987
rs6978919
rs6747171
rs224536
rs6066836
All − MR Egger
All − Inverse variance weighted
−2
−1
0
1
2
MR effect size for
'Black pepper liking || id:ebi−fl187−GCST90094708' on 'Rheumatoid arthritis || id:finngen_R11_M13_RHEUM

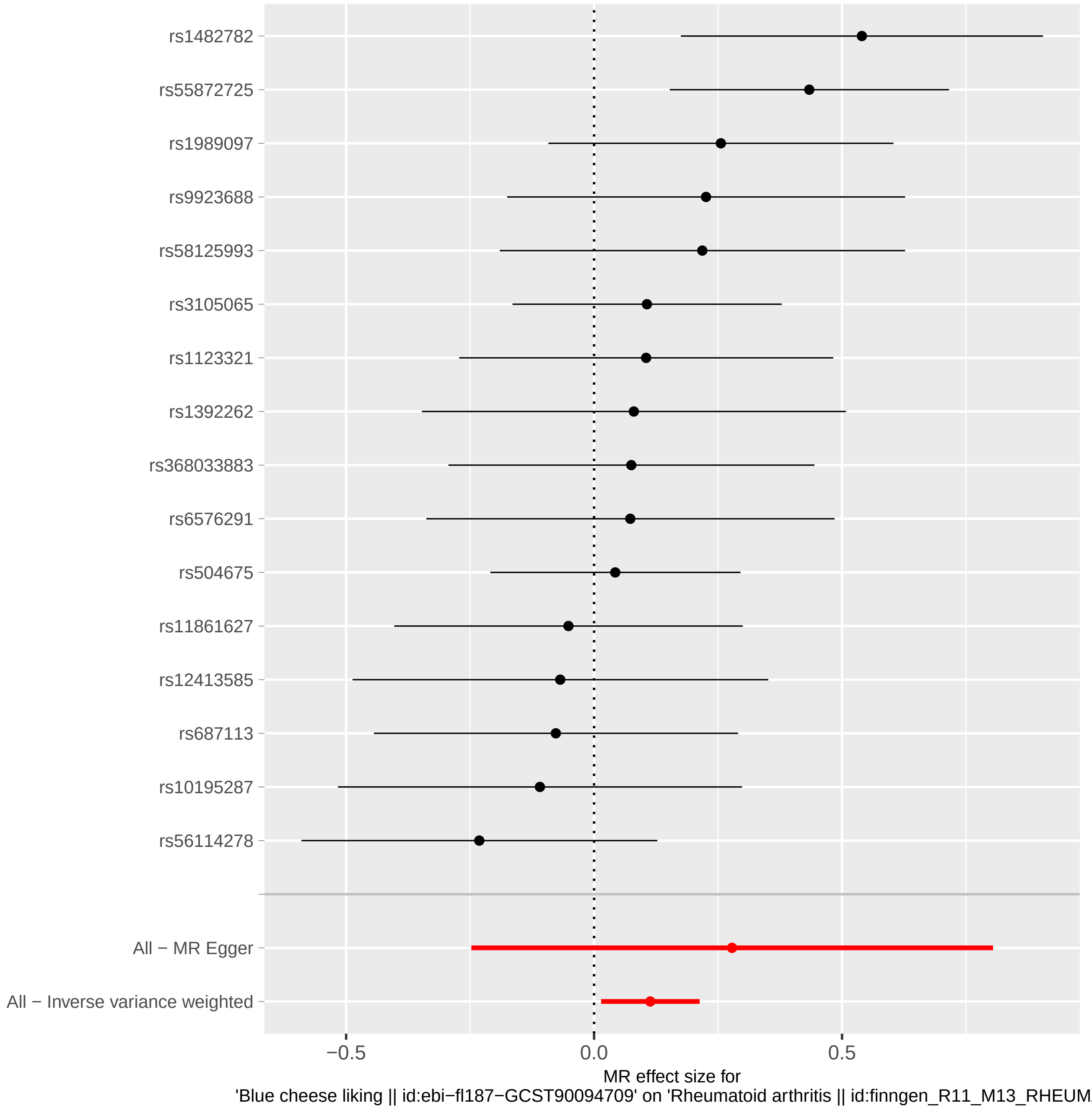
rs1482782
rs55872725
rs1989097
rs9923688
rs58125993
rs3105065
rs1123321
rs1392262
rs368033883
rs6576291
rs504675
rs11861627
rs12413585
rs687113
rs10195287
rs56114278
All − MR Egger
All − Inverse variance weighted
−0.5
0.0
0.5
MR effect size for
'Blue cheese liking || id:ebi−fl187−GCST90094709' on 'Rheumatoid arthritis || id:finngen_R11_M13_RHEUM

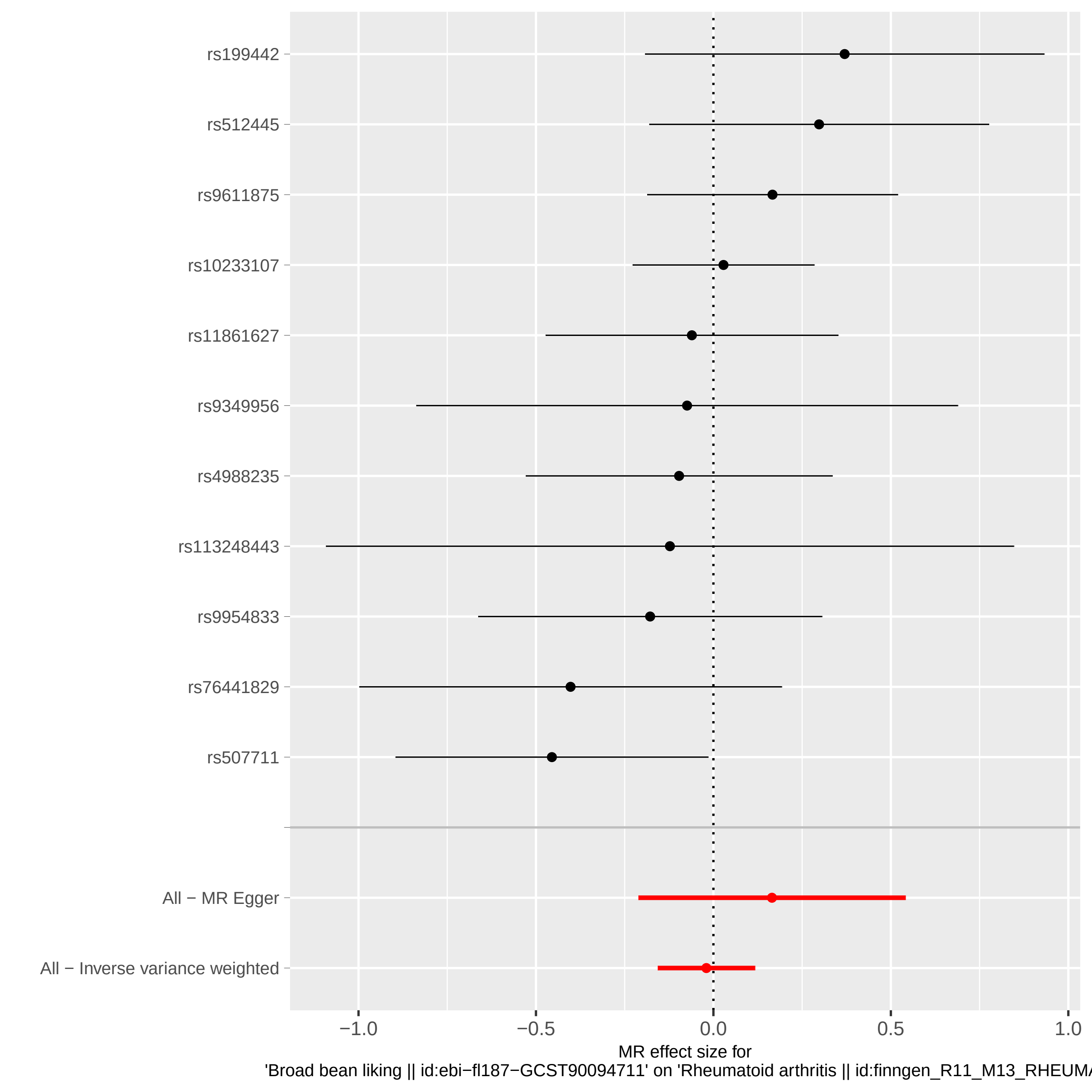

rs199442
rs512445
rs9611875
rs10233107
rs11861627
rs9349956
rs4988235
rs113248443
rs9954833
rs76441829
rs507711
All − MR Egger
All − Inverse variance weighted
−1.0
−0.5
0.0
0.5
1.0
MR effect size for
'Broad bean liking || id:ebi−fl187−GCST90094711' on 'Rheumatoid arthritis || id:finngen_R11_M13_RHEUM

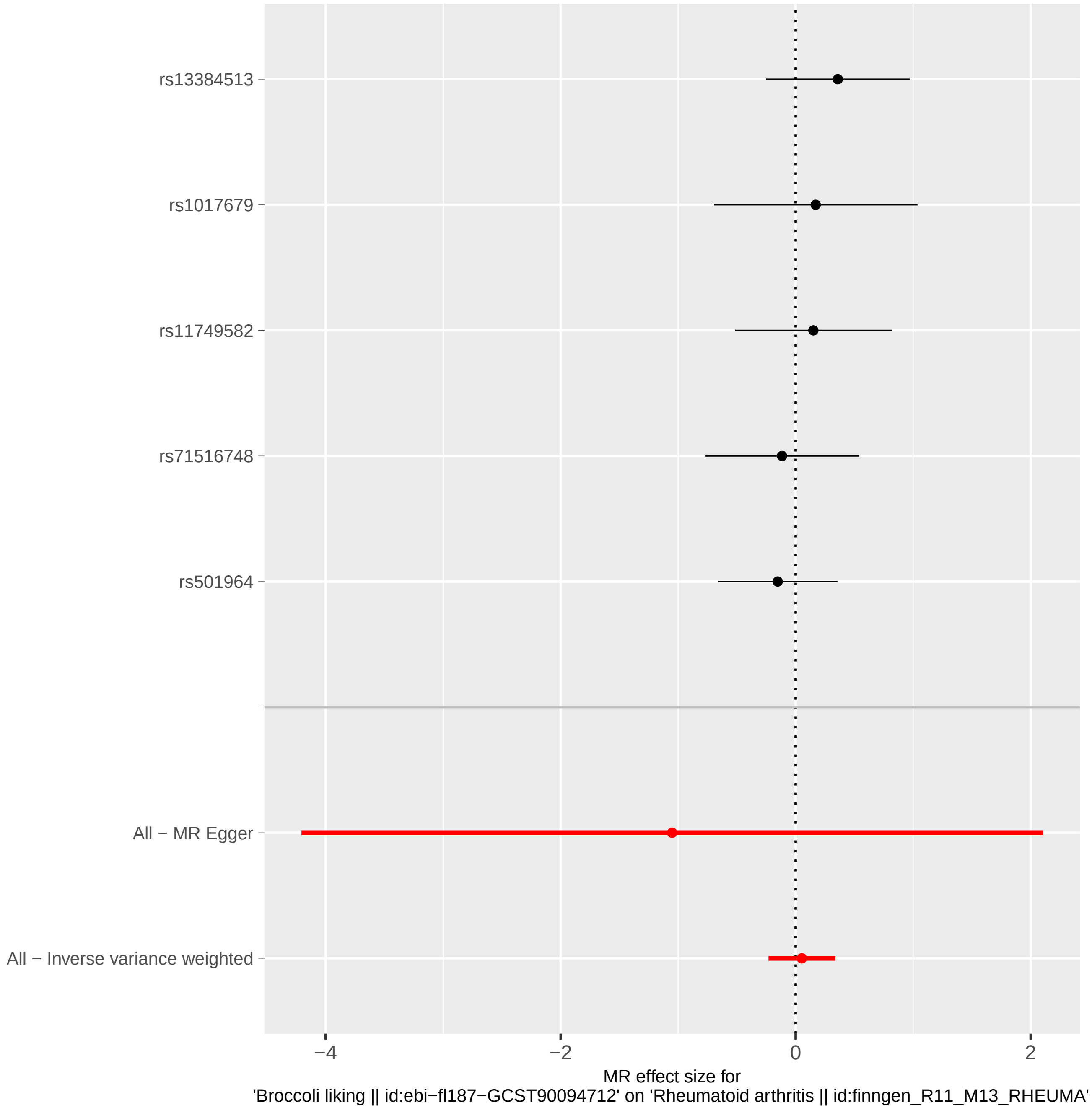

rs13384513
rs1017679
rs11749582
rs71516748
rs501964
All − MR Egger
All − Inverse variance weighted
−4
−2
0
2
MR effect size for
'Broccoli liking || id:ebi−fl187−GCST90094712' on 'Rheumatoid arthritis || id:finngen_R11_M13_RHEUMA'

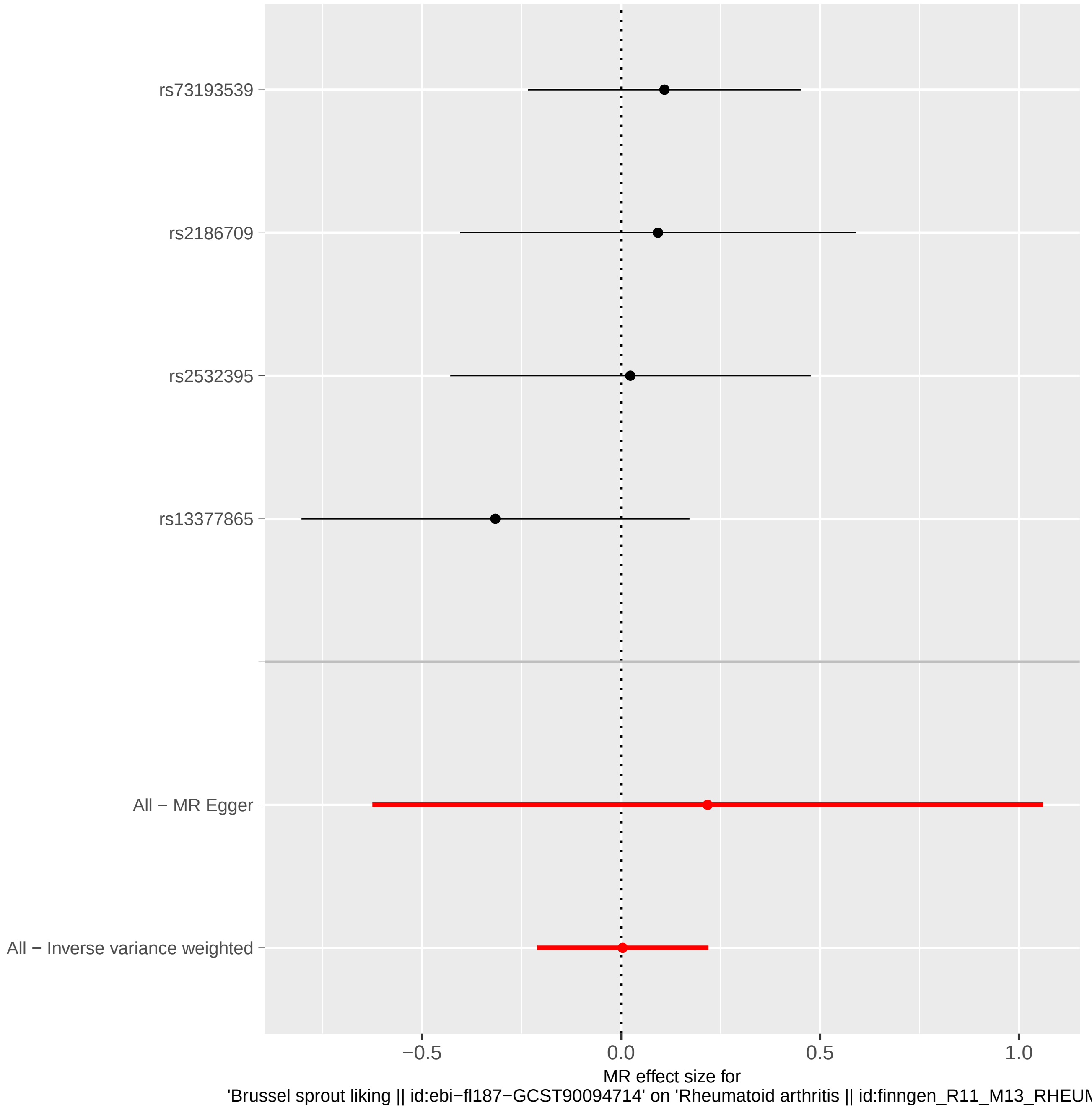
rs73193539
rs2186709
rs2532395
rs13377865
All − MR Egger
All − Inverse variance weighted
−0.5
0.0
0.5
1.0
MR effect size for
'Brussel sprout liking || id:ebi−fl187−GCST90094714' on 'Rheumatoid arthritis || id:finngen_R11_M13_RHEUM

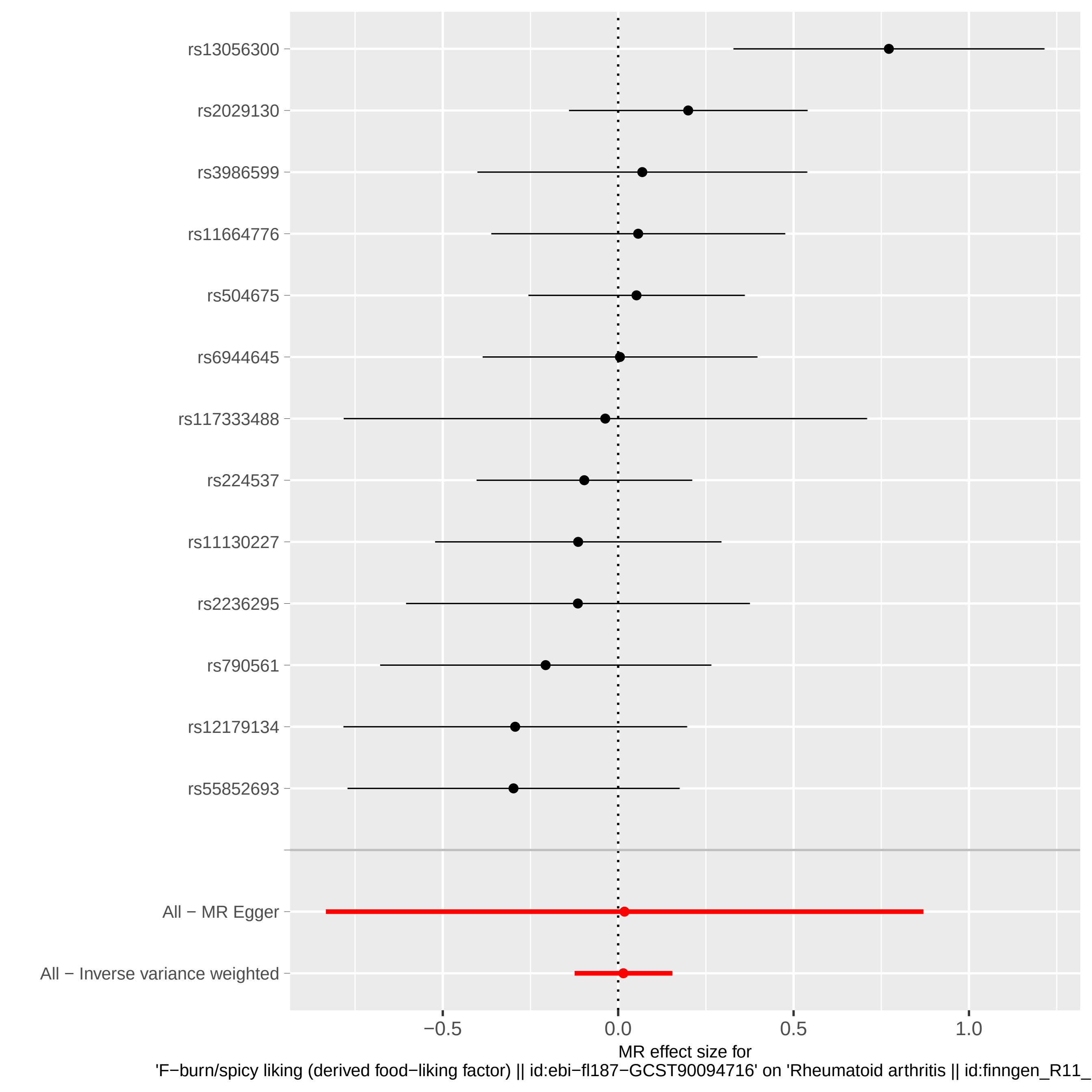
rs13056300
rs2029130
rs3986599
rs11664776
rs504675
rs6944645
rs117333488
rs224537
rs11130227
rs2236295
rs790561
rs12179134
rs55852693
All − MR Egger
All − Inverse variance weighted
−0.5
0.0
0.5
1.0
MR effect size for
'F−burn/spicy liking (derived food−liking factor) || id:ebi−fl187−GCST90094716' on 'Rheumatoid arthritis || id:finngen_R11_

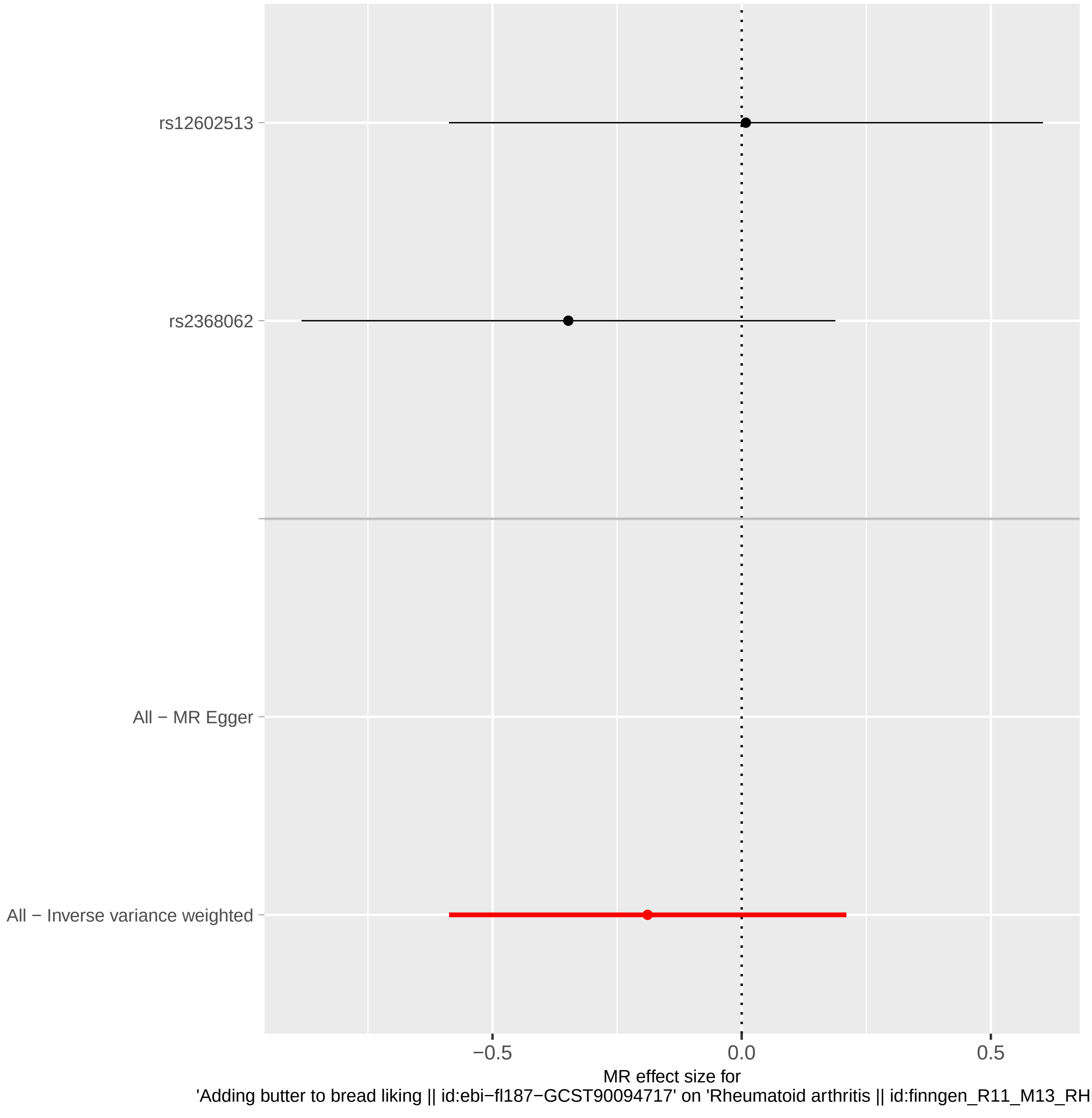
rs12602513
rs2368062
All − MR Egger
All − Inverse variance weighted
−0.5
0.0
0.5
MR effect size for
'Adding butter to bread liking || id:ebi−fl187−GCST90094717' on 'Rheumatoid arthritis || id:finngen_R11_M13_RH

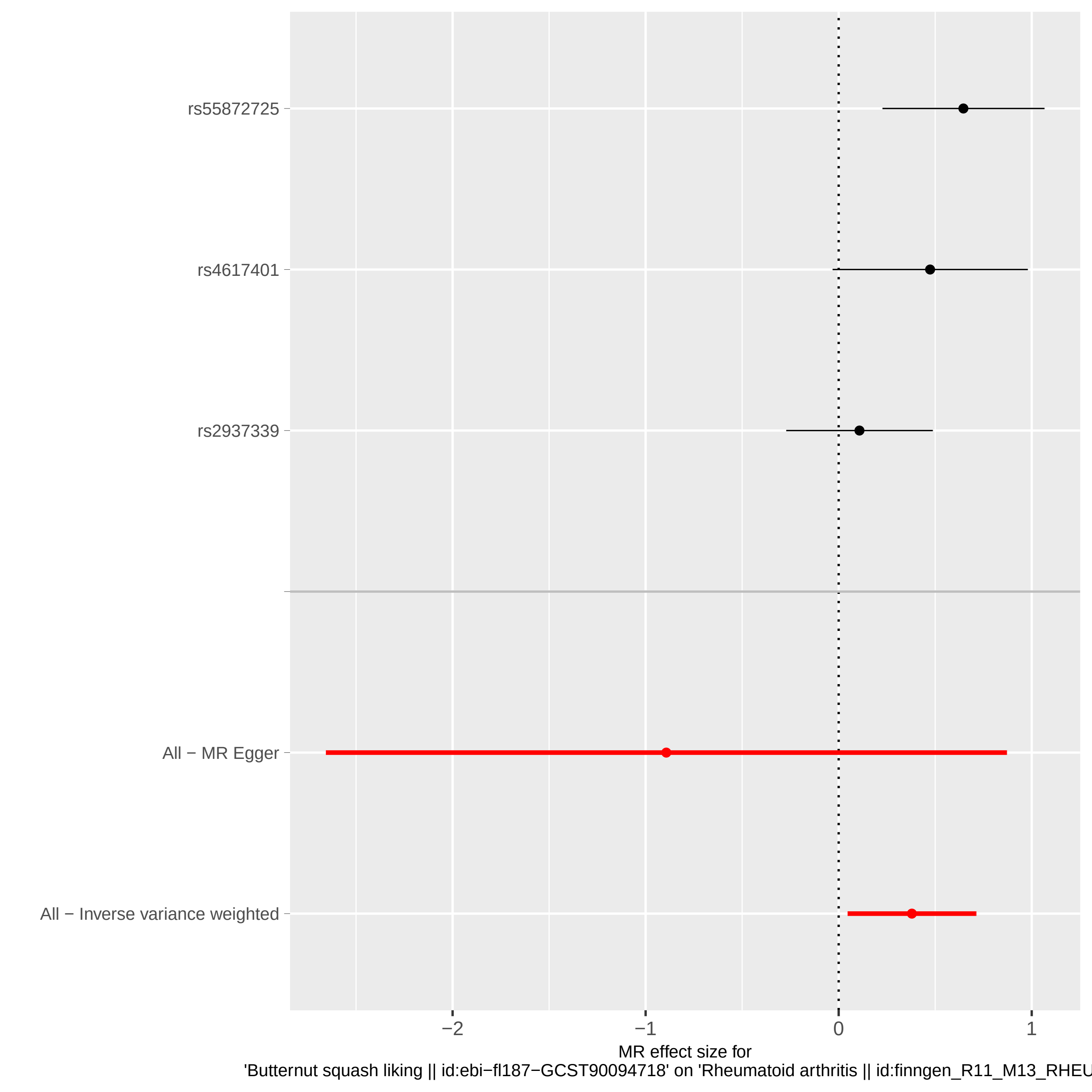
rs55872725
rs4617401
rs2937339
All − MR Egger
All − Inverse variance weighted
−2
−1
0
1
MR effect size for
'Butternut squash liking || id:ebi−fl187−GCST90094718' on 'Rheumatoid arthritis || id:finngen_R11_M13_RHEU

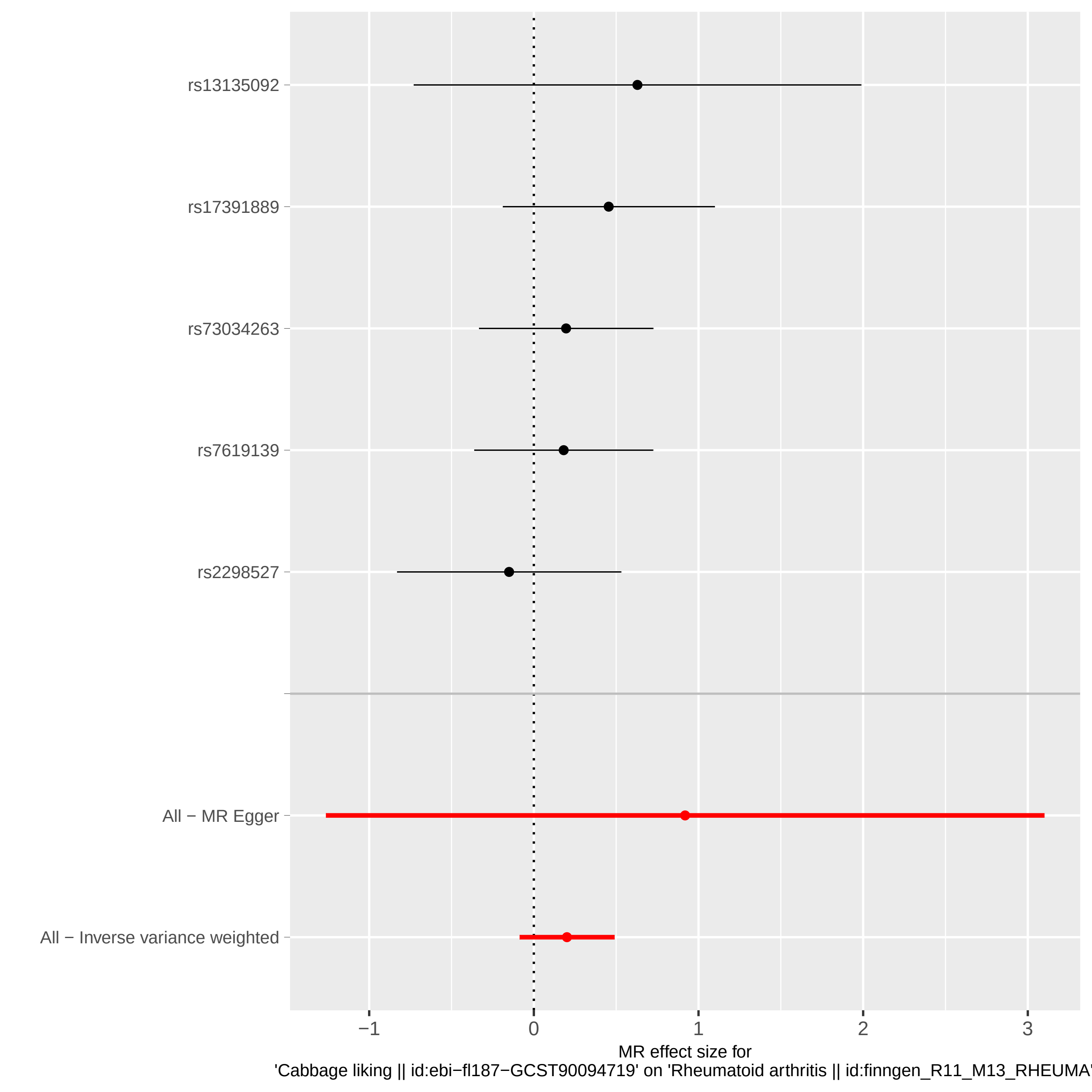
rs13135092
rs17391889
rs73034263
rs7619139
rs2298527
All − MR Egger
All − Inverse variance weighted
−1
0
1
2
3
MR effect size for
'Cabbage liking || id:ebi−fl187−GCST90094719' on 'Rheumatoid arthritis || id:finngen_R11_M13_RHEUMA'

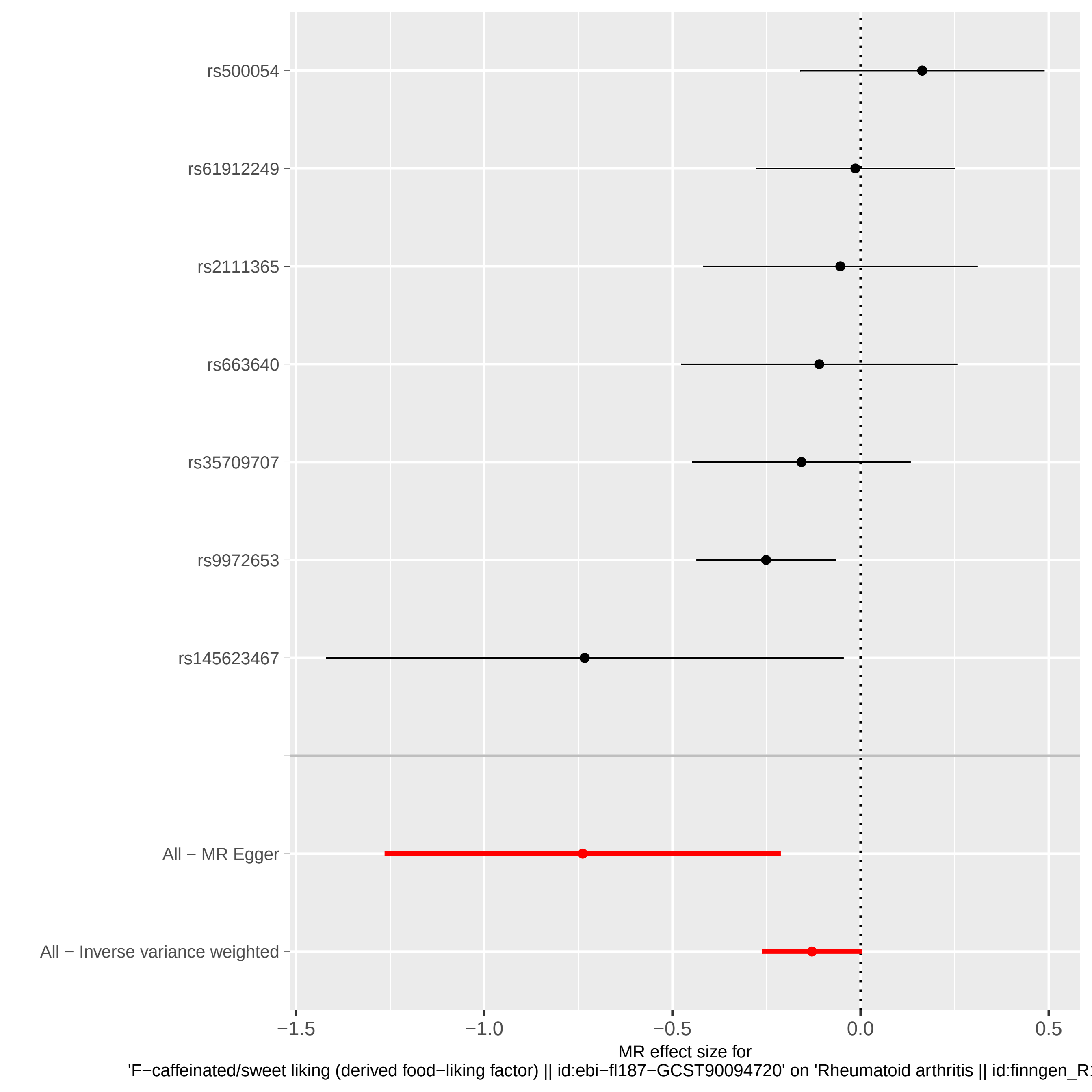

rs500054
rs61912249
rs2111365
rs663640
rs35709707
rs9972653
rs145623467
All − MR Egger
All − Inverse variance weighted
−1.5
−1.0
−0.5
0.0
0.5
MR effect size for
'F−caffeinated/sweet liking (derived food−liking factor) || id:ebi−fl187−GCST90094720' on 'Rheumatoid arthritis || id:finngen_R

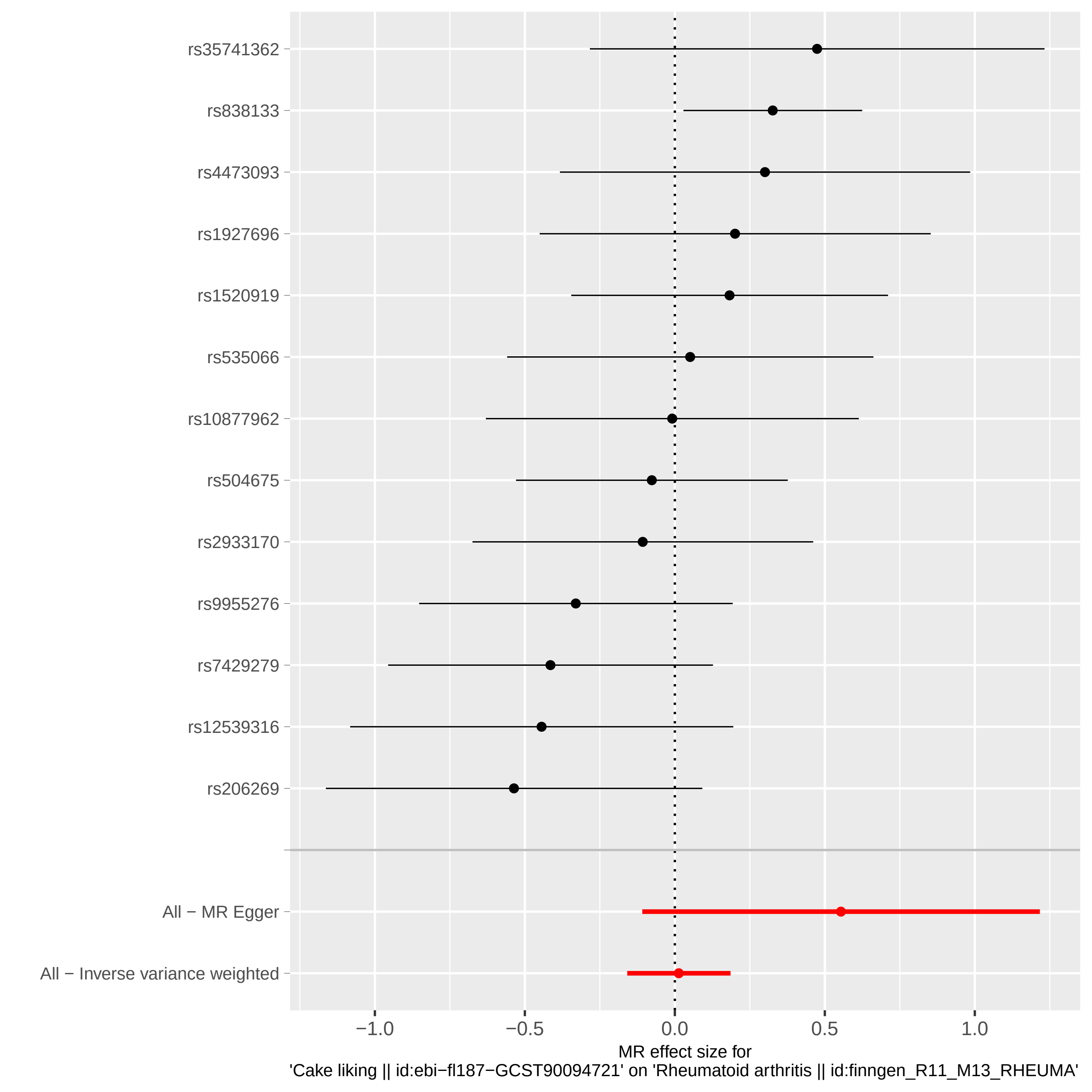

rs35741362
rs838133
rs4473093
rs1927696
rs1520919
rs535066
rs10877962
rs504675
rs2933170
rs9955276
rs7429279
rs12539316
rs206269
All − MR Egger
All − Inverse variance weighted
−1.0
−0.5
0.0
0.5
1.0
MR effect size for
'Cake liking || id:ebi−fl187−GCST90094721' on 'Rheumatoid arthritis || id:finngen_R11_M13_RHEUMA'

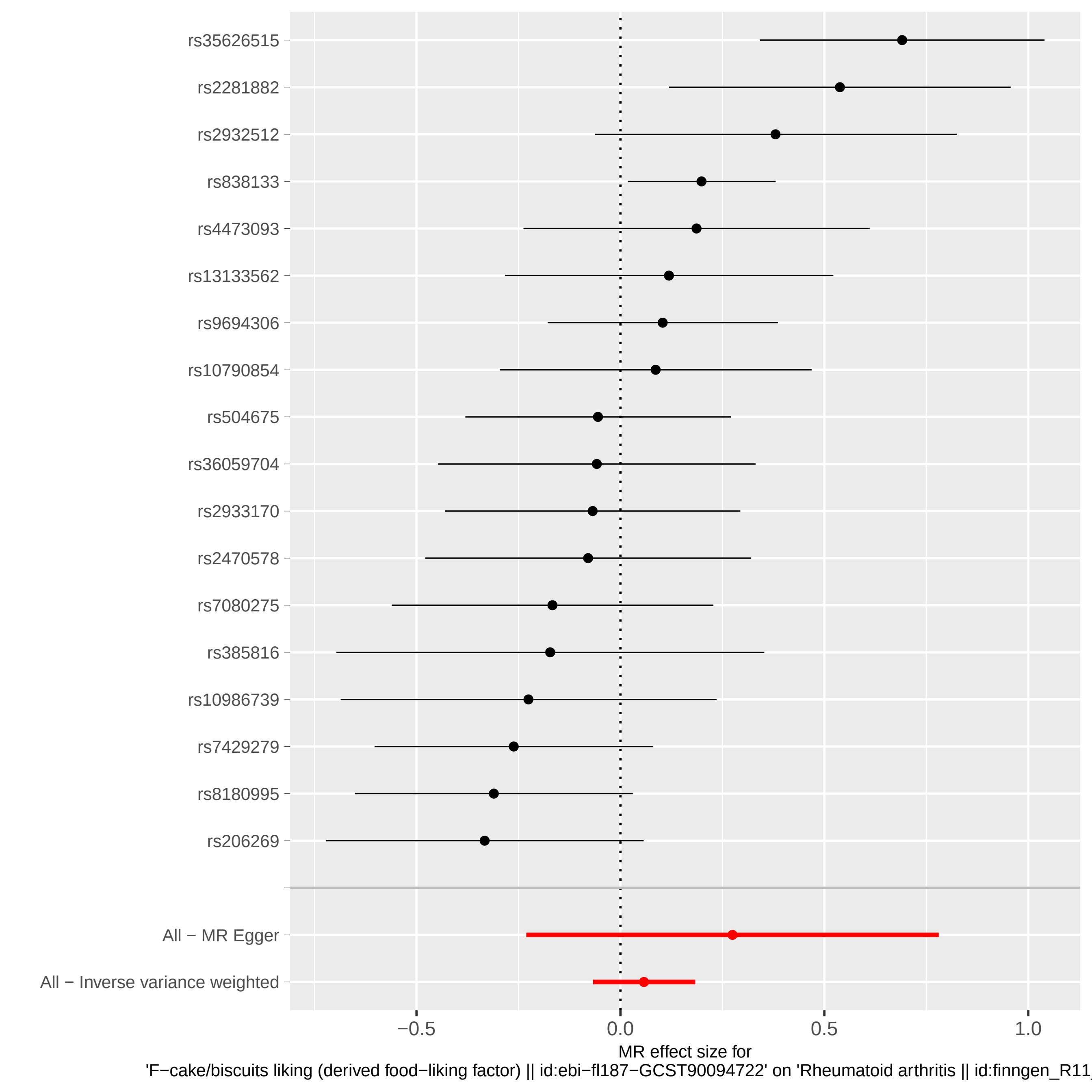

rs35626515
rs2281882
rs2932512
rs838133
rs4473093
rs13133562
rs9694306
rs10790854
rs504675
rs36059704
rs2933170
rs2470578
rs7080275
rs385816
rs10986739
rs7429279
rs8180995
rs206269
All − MR Egger
All − Inverse variance weighted
−0.5
0.0
0.5
1.0
MR effect size for
'F−cake/biscuits liking (derived food−liking factor) || id:ebi−fl187−GCST90094722' on 'Rheumatoid arthritis || id:finngen_R11_

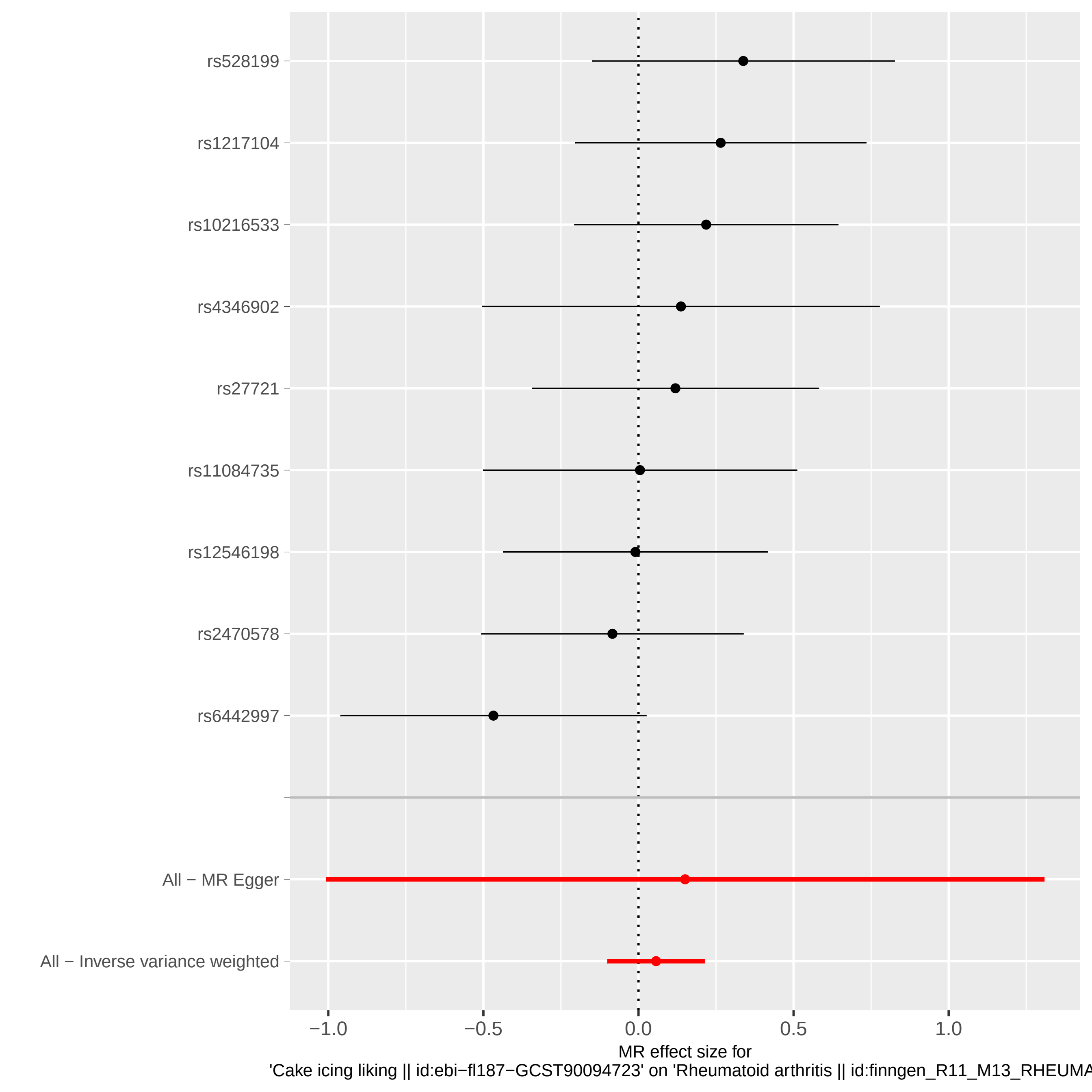
rs528199
rs1217104
rs10216533
rs4346902
rs27721
rs11084735
rs12546198
rs2470578
rs6442997
All − MR Egger
All − Inverse variance weighted
−1.0
−0.5
0.0
0.5
1.0
MR effect size for
'Cake icing liking || id:ebi−fl187−GCST90094723' on 'Rheumatoid arthritis || id:finngen_R11_M13_RHEUMA

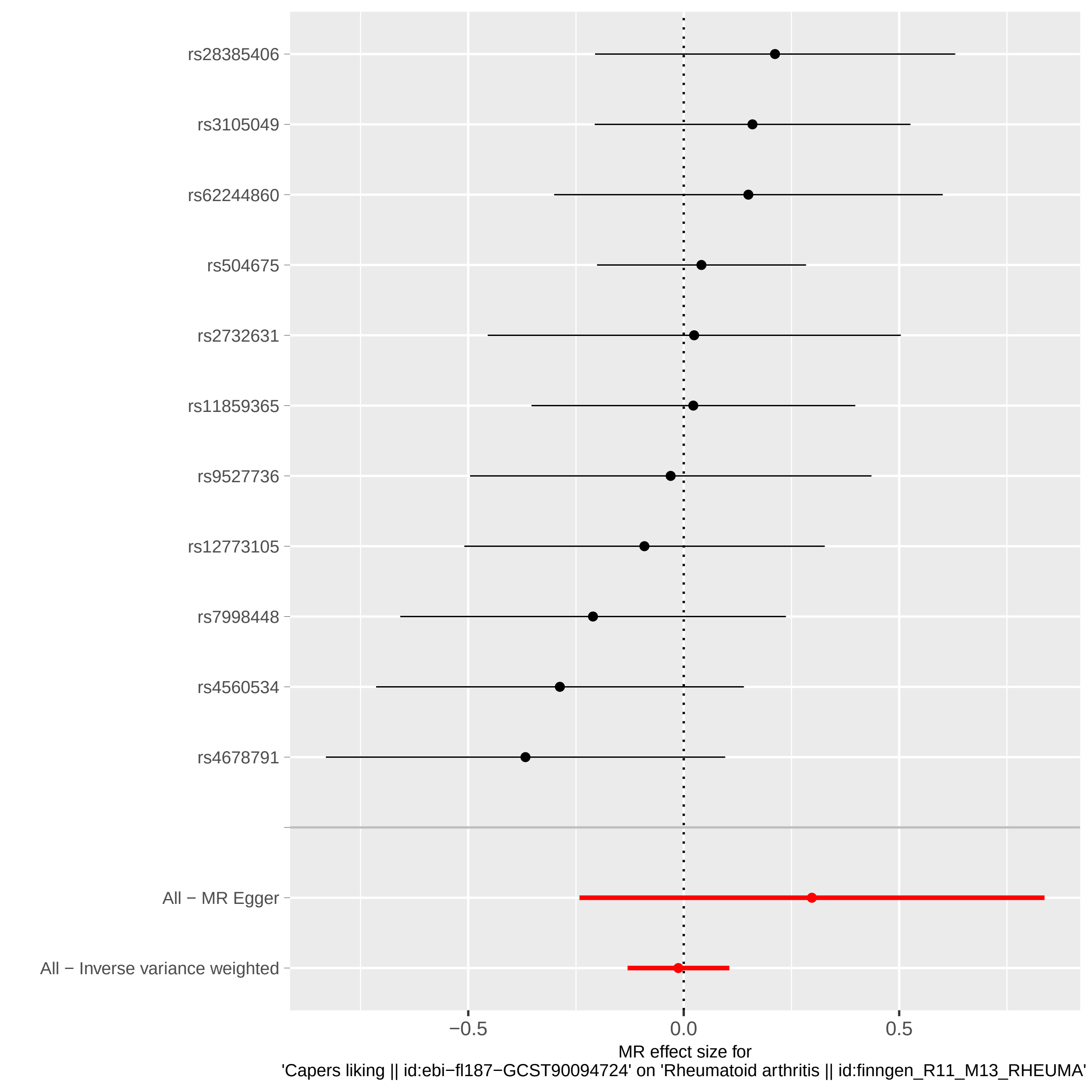
rs28385406
rs3105049
rs62244860
rs504675
rs2732631
rs11859365
rs9527736
rs12773105
rs7998448
rs4560534
rs4678791
All − MR Egger
All − Inverse variance weighted
−0.5
0.0
0.5
MR effect size for
'Capers liking || id:ebi−fl187−GCST90094724' on 'Rheumatoid arthritis || id:finngen_R11_M13_RHEUMA'

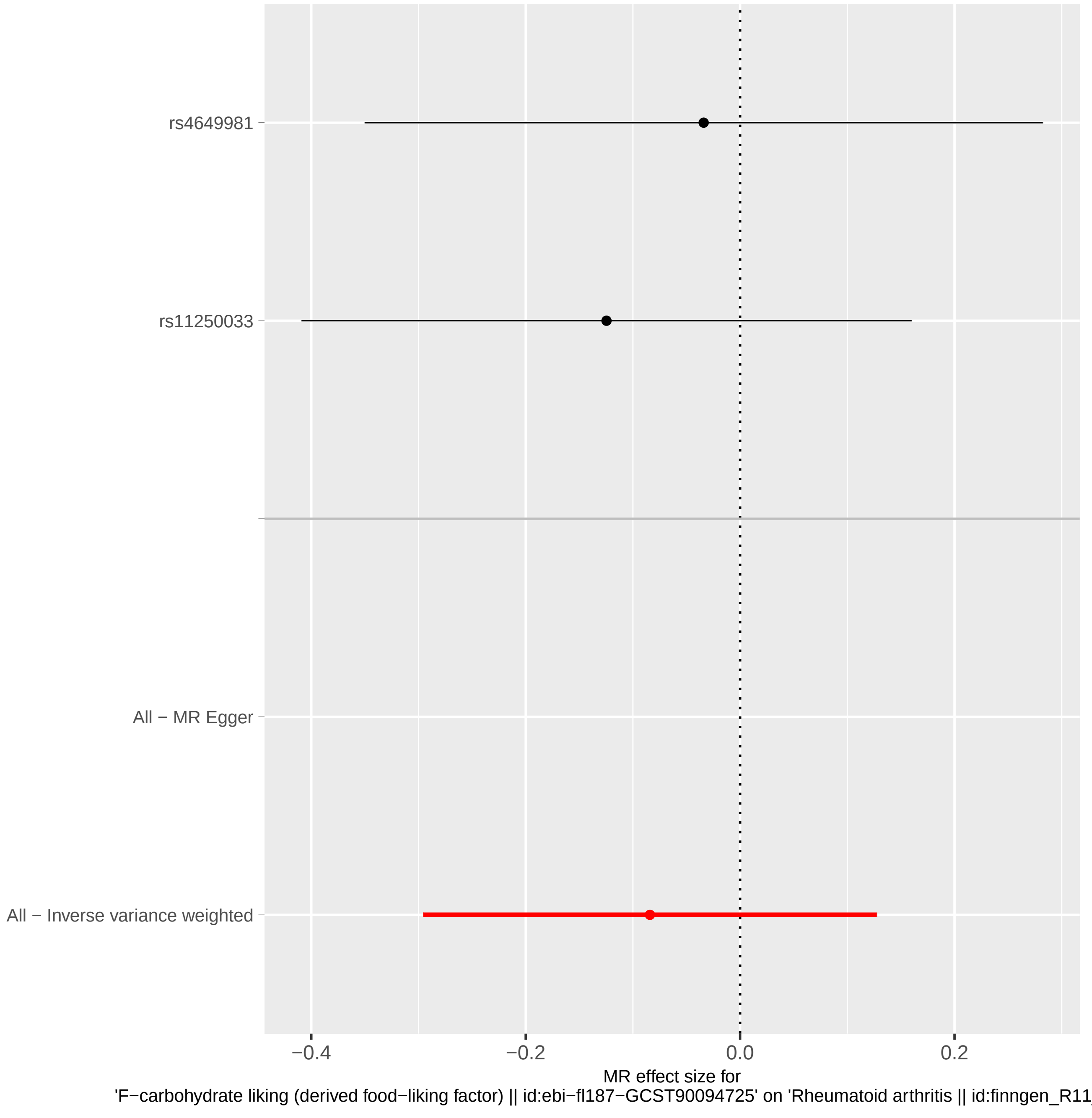

rs4649981
rs11250033
All − MR Egger
All − Inverse variance weighted
−0.4
−0.2
0.0
0.2
MR effect size for
'F−carbohydrate liking (derived food−liking factor) || id:ebi−fl187−GCST90094725' on 'Rheumatoid arthritis || id:finngen_R11

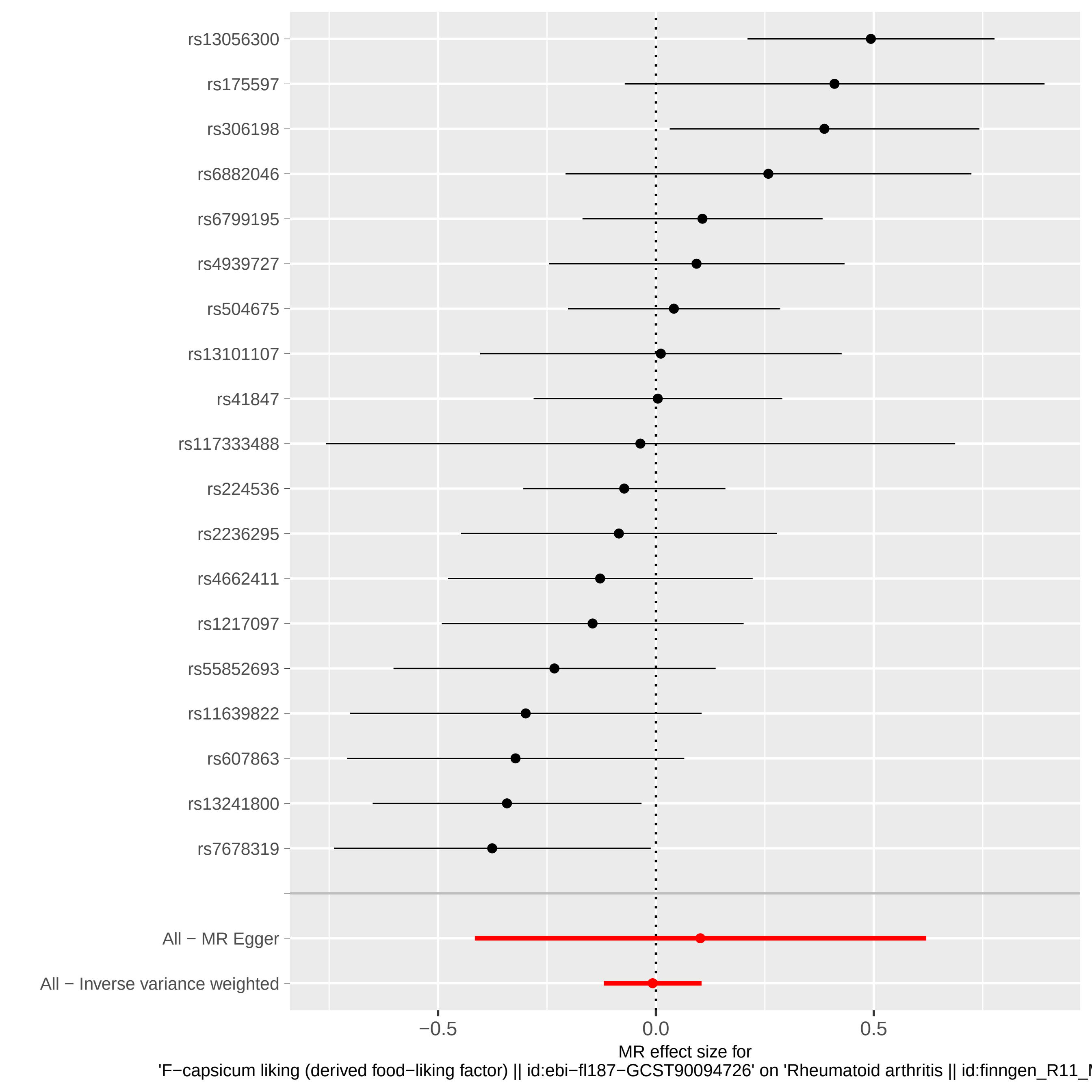
rs13056300
rs175597
rs306198
rs6882046
rs6799195
rs4939727
rs504675
rs13101107
rs41847
rs117333488
rs224536
rs2236295
rs4662411
rs1217097
rs55852693
rs11639822
rs607863
rs13241800
rs7678319
All − MR Egger
All − Inverse variance weighted
−0.5
0.0
0.5
MR effect size for
'F−capsicum liking (derived food−liking factor) || id:ebi−fl187−GCST90094726' on 'Rheumatoid arthritis || id:finngen_R11_M

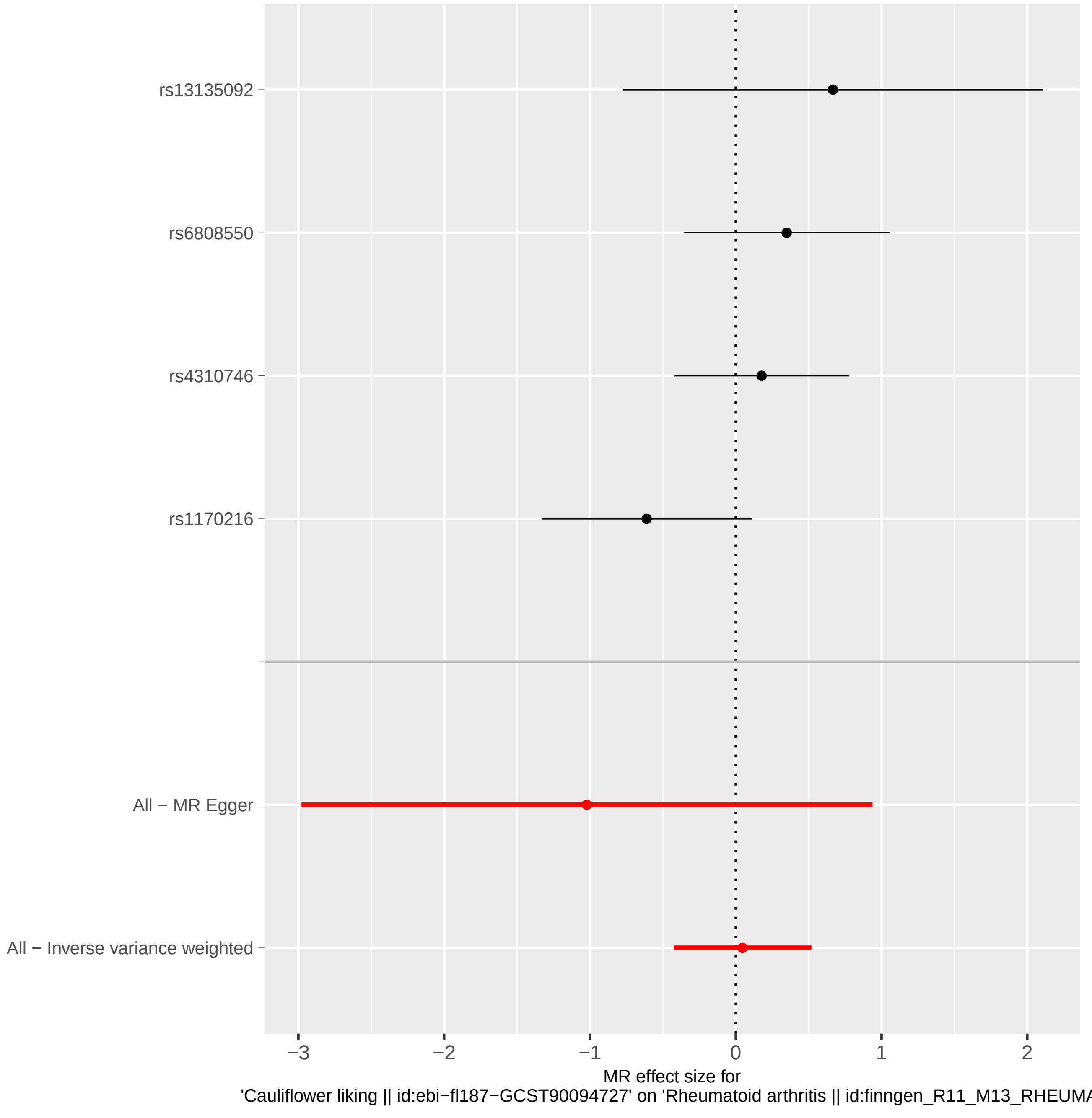

rs13135092
rs6808550
rs4310746
rs1170216
All − MR Egger
All − Inverse variance weighted
−3
−2
−1
0
1
2
MR effect size for
'Cauliflower liking || id:ebi−fl187−GCST90094727' on 'Rheumatoid arthritis || id:finngen_R11_M13_RHEUMA

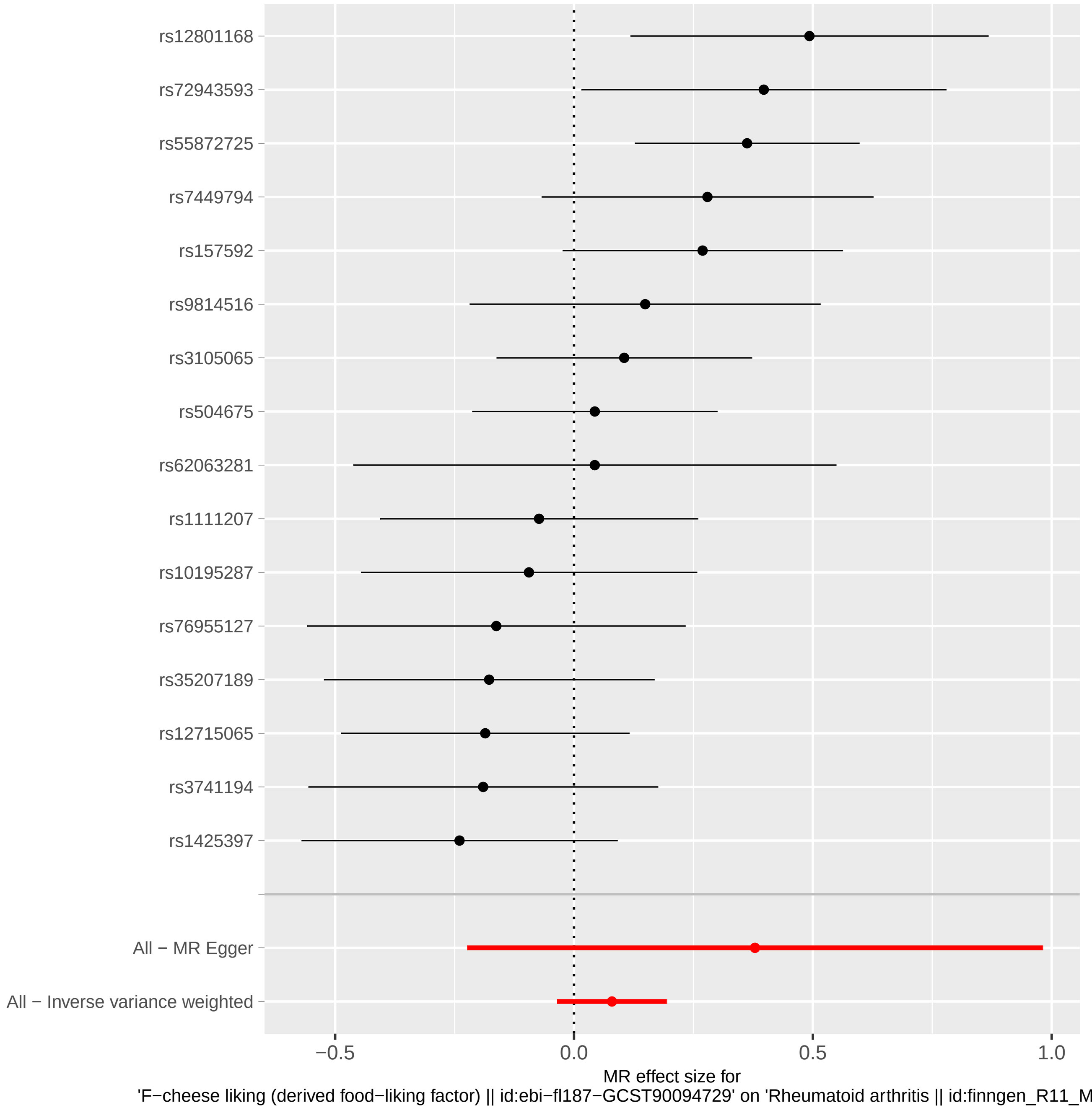
rs12801168
rs72943593
rs55872725
rs7449794
rs157592
rs9814516
rs3105065
rs504675
rs62063281
rs1111207
rs10195287
rs76955127
rs35207189
rs12715065
rs3741194
rs1425397
All − MR Egger
All − Inverse variance weighted
−0.5
0.0
0.5
1.0
MR effect size for
'F−cheese liking (derived food−liking factor) || id:ebi−fl187−GCST90094729' on 'Rheumatoid arthritis || id:finngen_R11_M

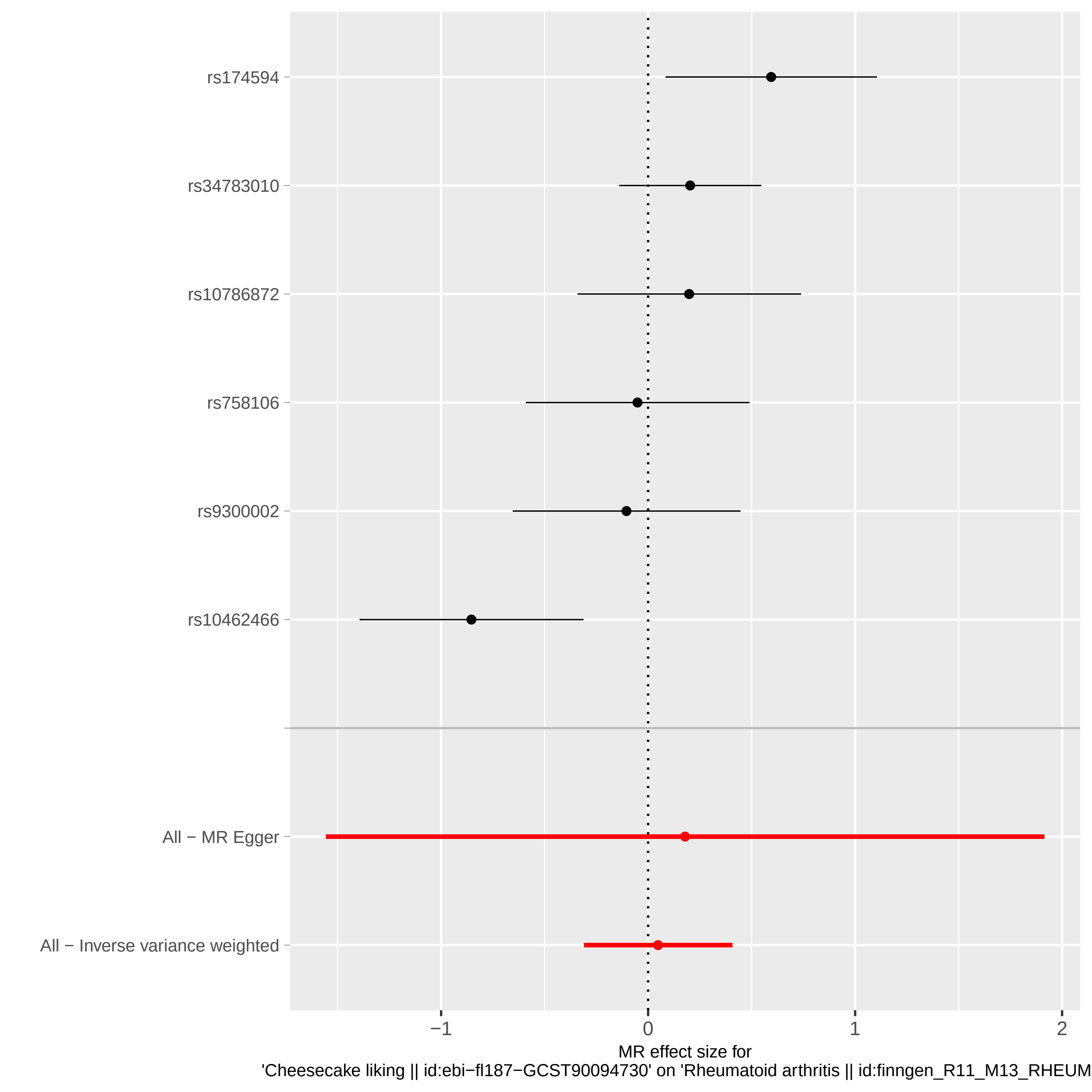

rs174594
rs34783010
rs10786872
rs758106
rs9300002
rs10462466
All − MR Egger
All − Inverse variance weighted
−1
0
1
2
MR effect size for
'Cheesecake liking || id:ebi−fl187−GCST90094730' on 'Rheumatoid arthritis || id:finngen_R11_M13_RHEUM

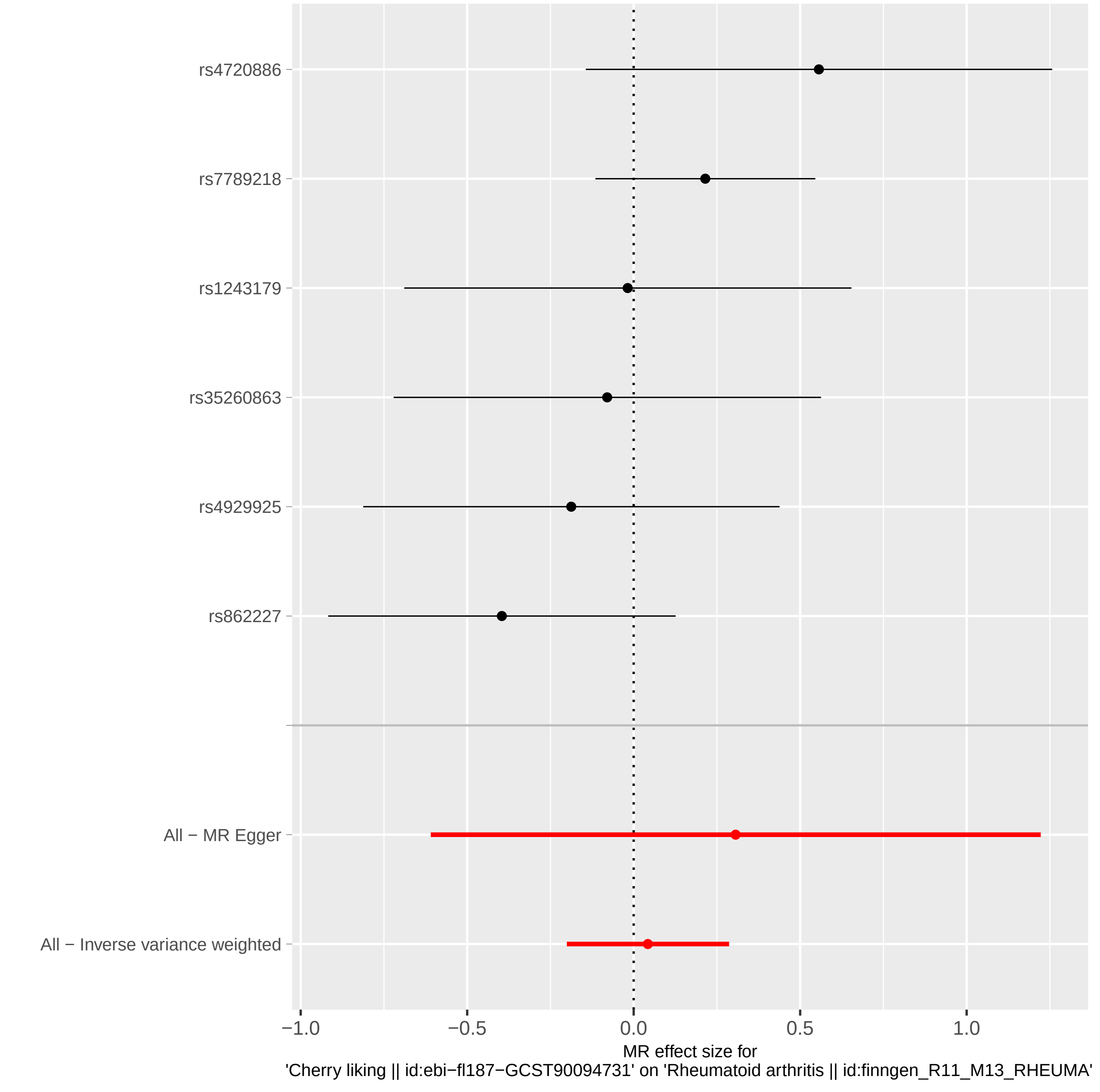
rs4720886
rs7789218
rs1243179
rs35260863
rs4929925
rs862227
All − MR Egger
All − Inverse variance weighted
−1.0
−0.5
0.0
0.5
1.0
MR effect size for
'Cherry liking || id:ebi−fl187−GCST90094731' on 'Rheumatoid arthritis || id:finngen_R11_M13_RHEUMA'

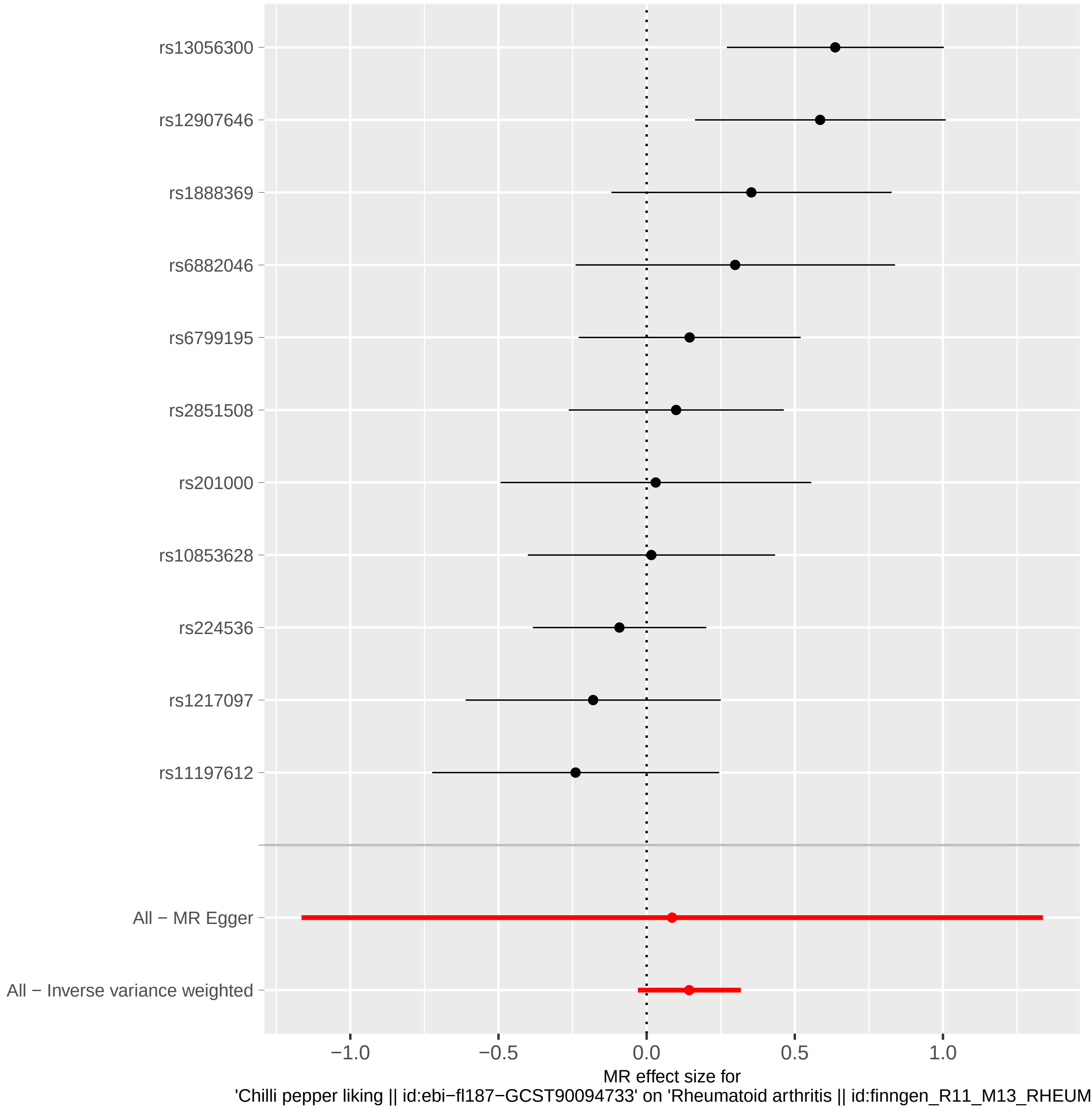

rs13056300
rs12907646
rs1888369
rs6882046
rs6799195
rs2851508
rs201000
rs10853628
rs224536
rs1217097
rs11197612
All − MR Egger
All − Inverse variance weighted
−1.0
−0.5
0.0
0.5
1.0
MR effect size for
'Chilli pepper liking || id:ebi−fl187−GCST90094733' on 'Rheumatoid arthritis || id:finngen_R11_M13_RHEUM

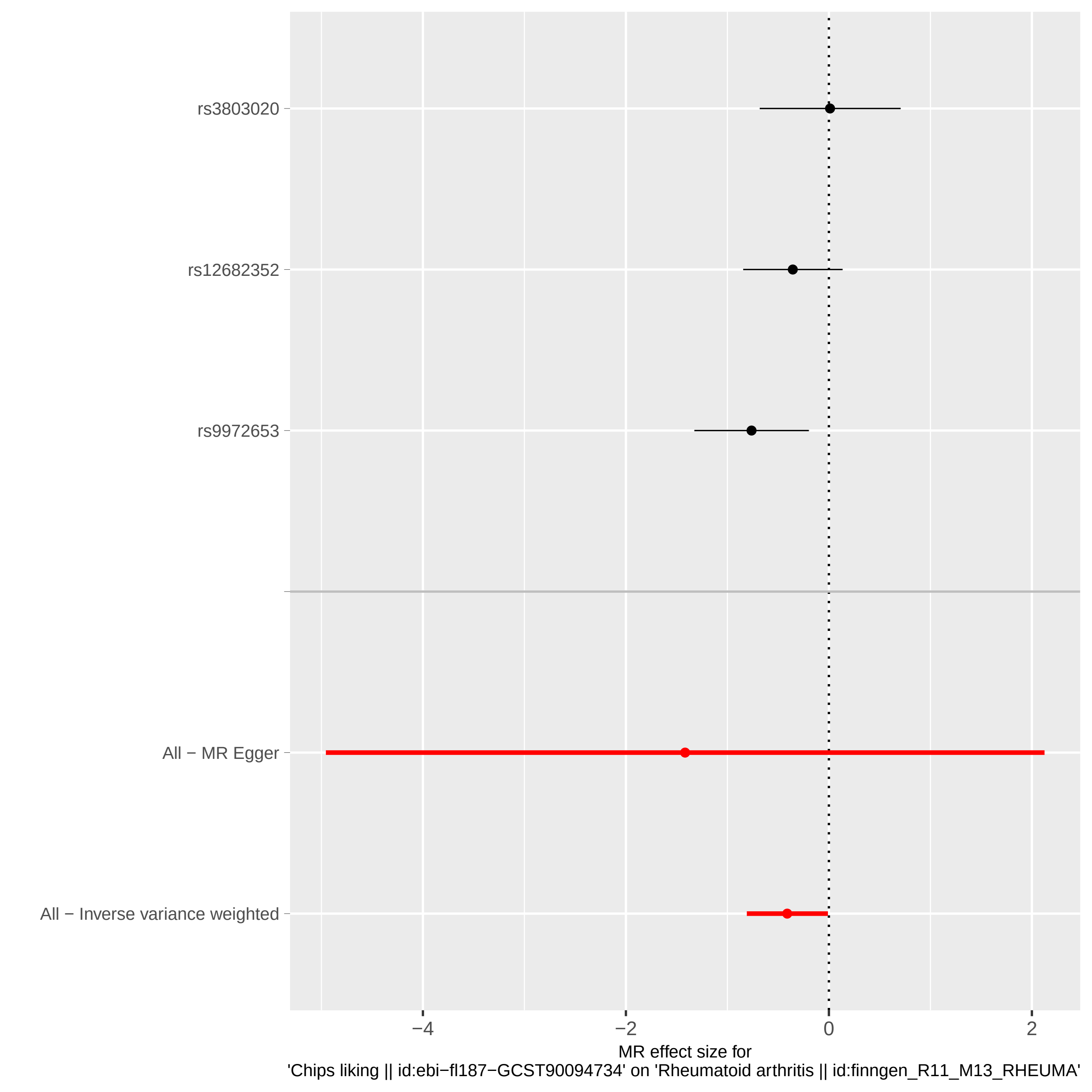

rs3803020
rs12682352
rs9972653
All − MR Egger
All − Inverse variance weighted
−4
−2
0
2
MR effect size for
'Chips liking || id:ebi−fl187−GCST90094734' on 'Rheumatoid arthritis || id:finngen_R11_M13_RHEUMA'

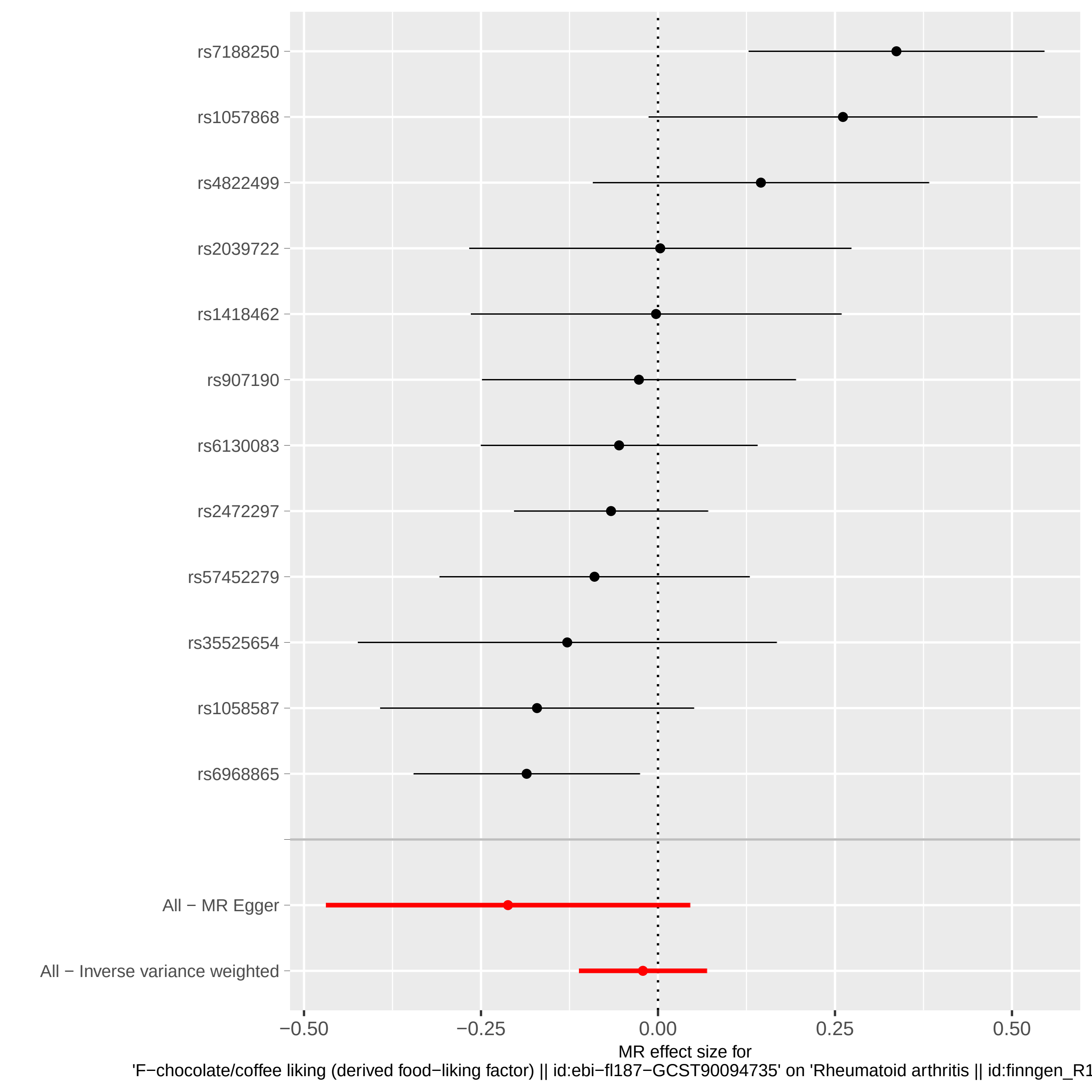

rs7188250
rs1057868
rs4822499
rs2039722
rs1418462
rs907190
rs6130083
rs2472297
rs57452279
rs35525654
rs1058587
rs6968865
All − MR Egger
All − Inverse variance weighted
−0.50
−0.25
0.00
0.25
0.50
MR effect size for
'F−chocolate/coffee liking (derived food−liking factor) || id:ebi−fl187−GCST90094735' on 'Rheumatoid arthritis || id:finngen_R1

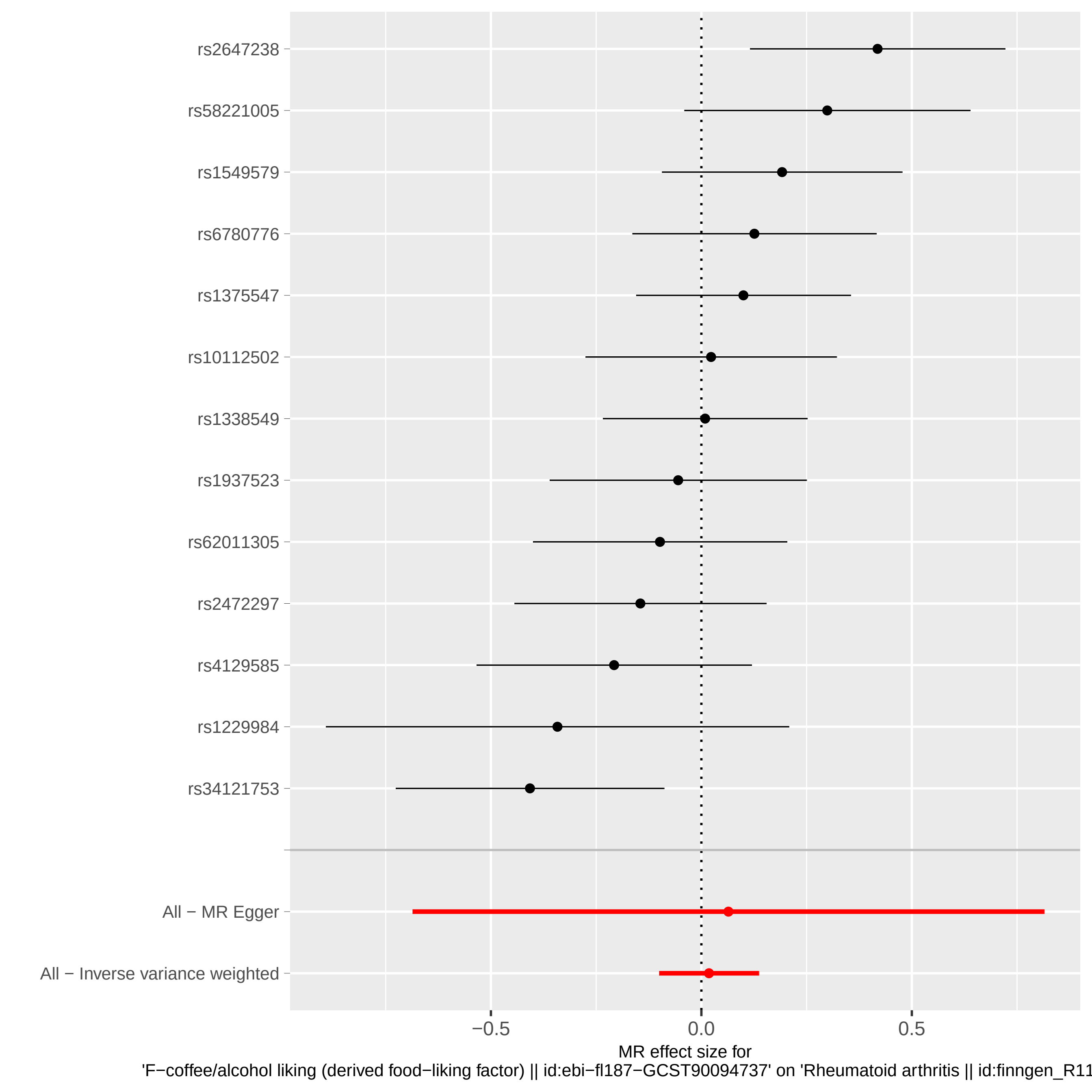
rs2647238
rs58221005
rs1549579
rs6780776
rs1375547
rs10112502
rs1338549
rs1937523
rs62011305
rs2472297
rs4129585
rs1229984
rs34121753
All − MR Egger
All − Inverse variance weighted
−0.5
0.0
0.5
MR effect size for
'F−coffee/alcohol liking (derived food−liking factor) || id:ebi−fl187−GCST90094737' on 'Rheumatoid arthritis || id:finngen_R11

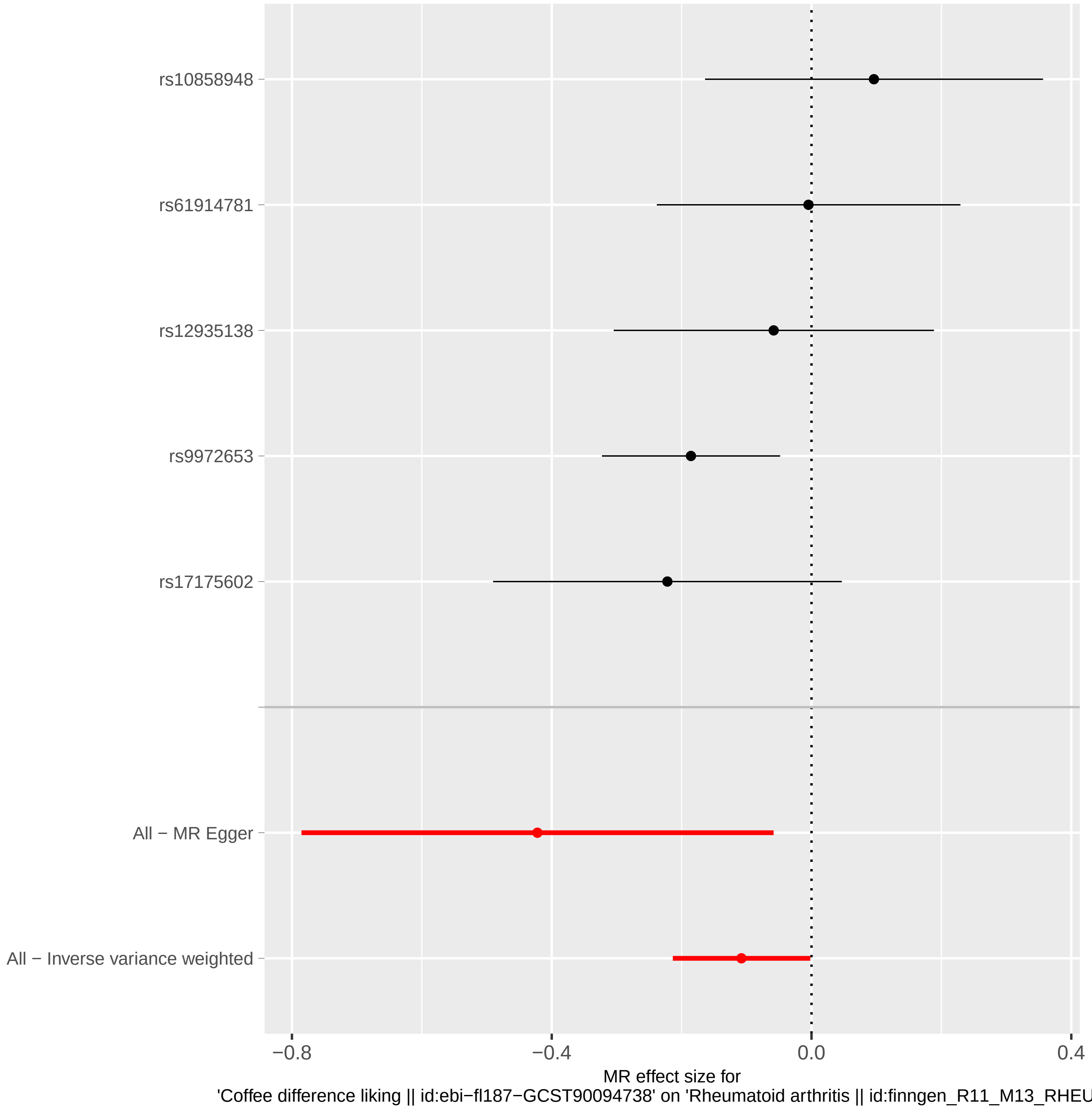
rs10858948
rs61914781
rs12935138
rs9972653
rs17175602
All − MR Egger
All − Inverse variance weighted
−0.8
−0.4
0.0
0.4
MR effect size for
'Coffee difference liking || id:ebi−fl187−GCST90094738' on 'Rheumatoid arthritis || id:finngen_R11_M13_RHEU

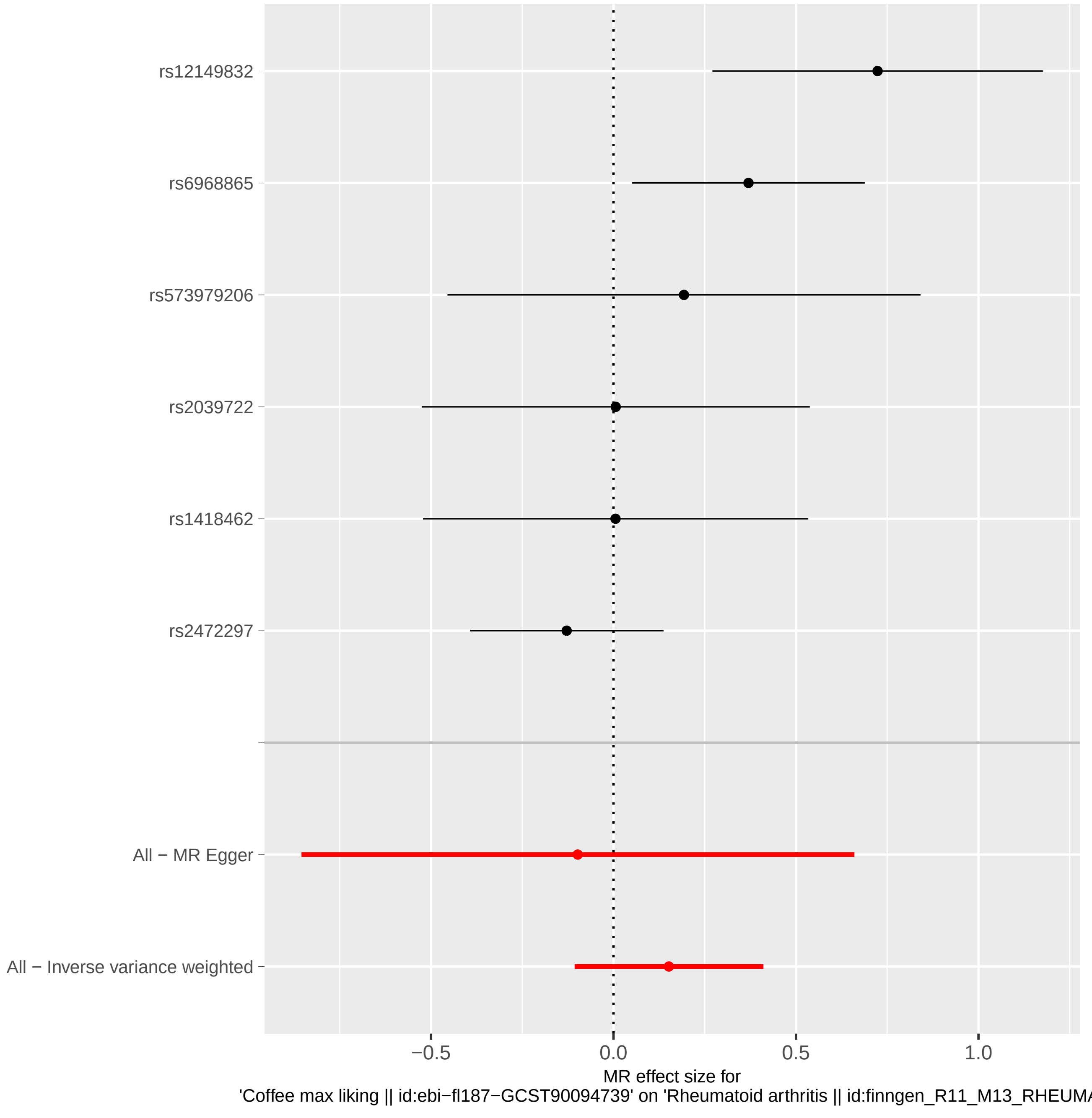
rs12149832
rs6968865
rs573979206
rs2039722
rs1418462
rs2472297
All − MR Egger
All − Inverse variance weighted
−0.5
0.0
0.5
1.0
MR effect size for
'Coffee max liking || id:ebi−fl187−GCST90094739' on 'Rheumatoid arthritis || id:finngen_R11_M13_RHEUMA

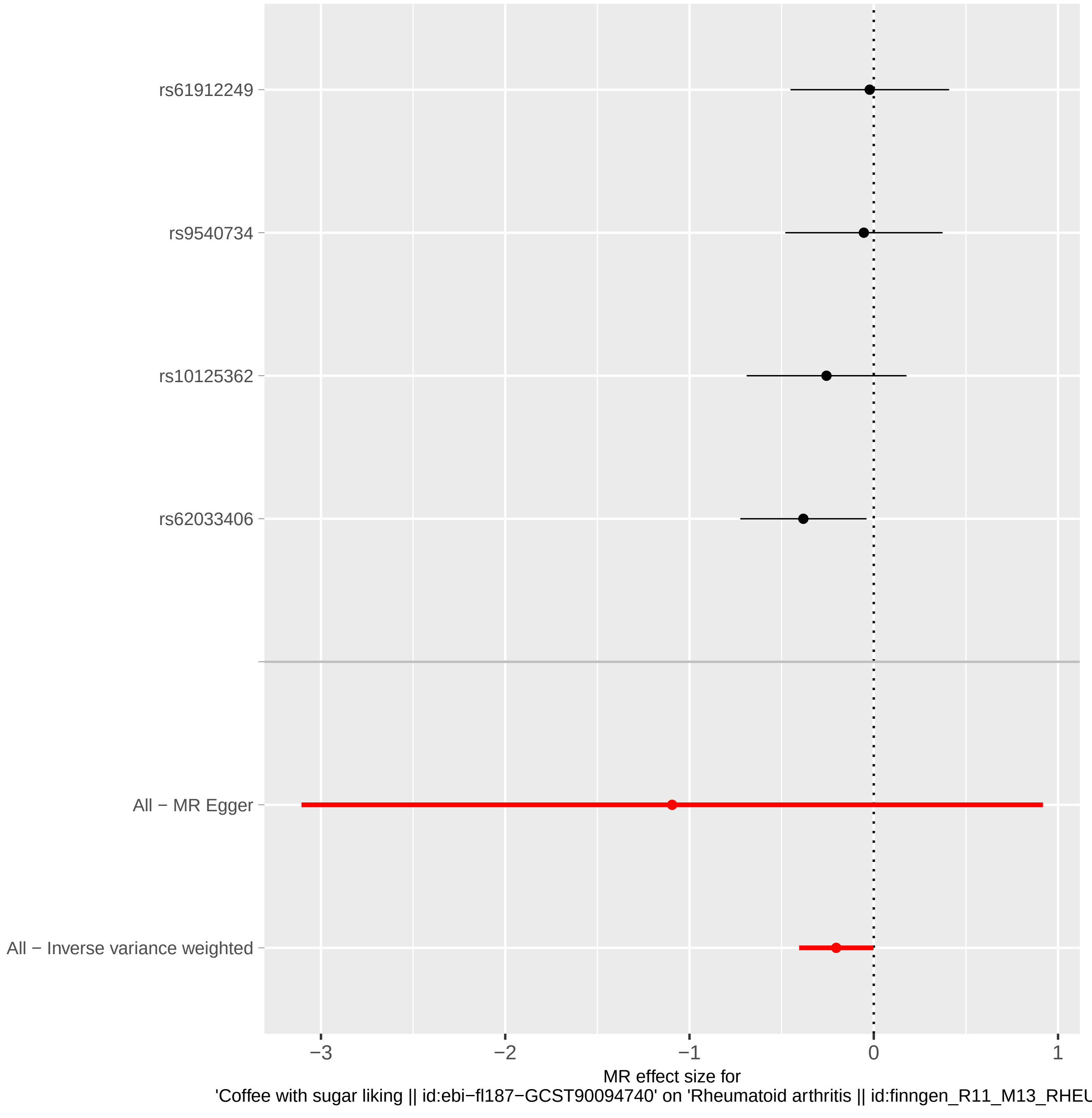

rs61912249
rs9540734
rs10125362
rs62033406
All − MR Egger
All − Inverse variance weighted
−3
−2
−1
0
1
MR effect size for
'Coffee with sugar liking || id:ebi−fl187−GCST90094740' on 'Rheumatoid arthritis || id:finngen_R11_M13_RHEU

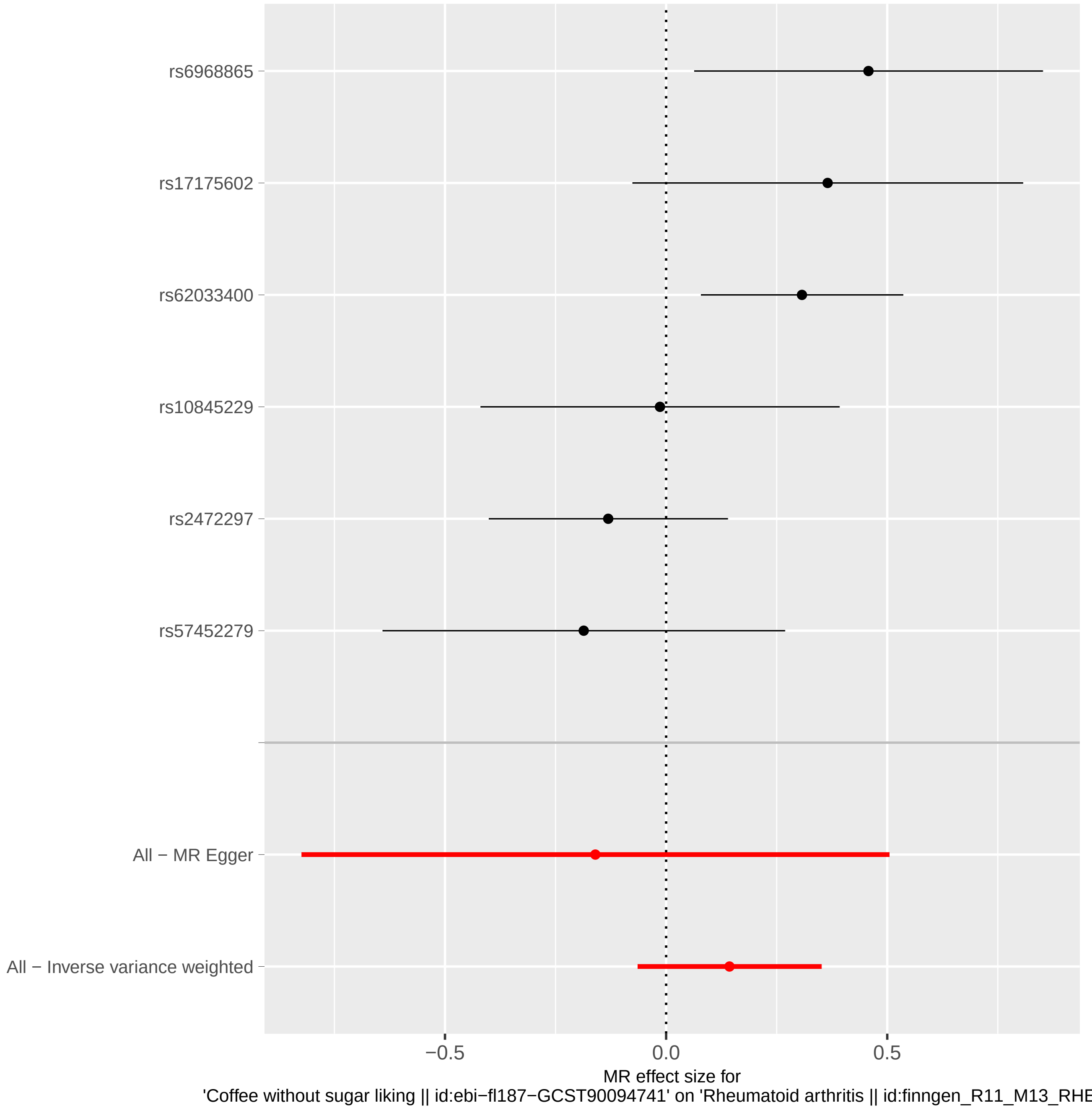
rs6968865
rs17175602
rs62033400
rs10845229
rs2472297
rs57452279
All − MR Egger
All − Inverse variance weighted
−0.5
0.0
0.5
MR effect size for
'Coffee without sugar liking || id:ebi−fl187−GCST90094741' on 'Rheumatoid arthritis || id:finngen_R11_M13_RHE

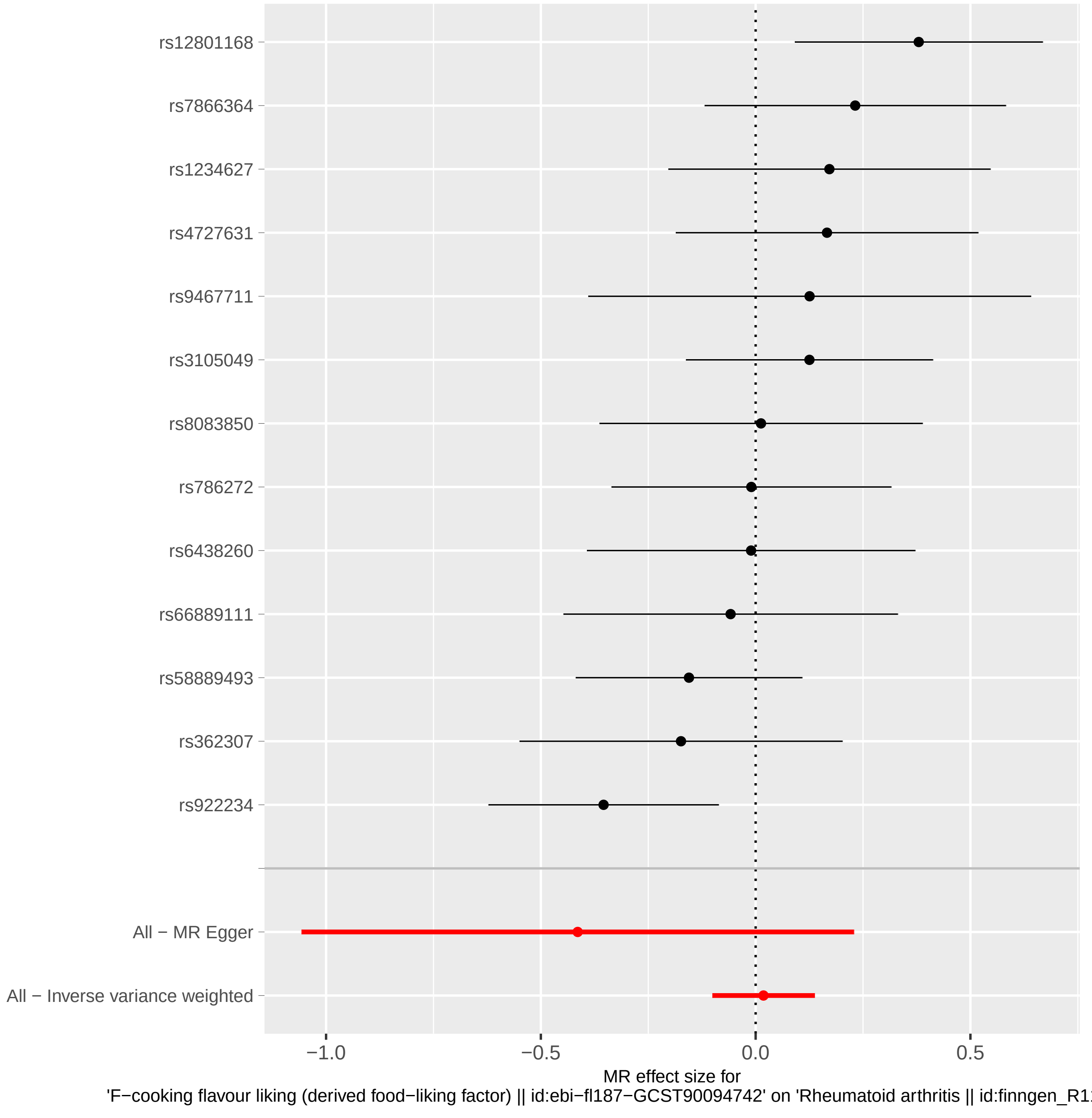
rs12801168
rs7866364
rs1234627
rs4727631
rs9467711
rs3105049
rs8083850
rs786272
rs6438260
rs66889111
rs58889493
rs362307
rs922234
All − MR Egger
All − Inverse variance weighted
−1.0
−0.5
0.0
0.5
MR effect size for
'F−cooking flavour liking (derived food−liking factor) || id:ebi−fl187−GCST90094742' on 'Rheumatoid arthritis || id:finngen_R1

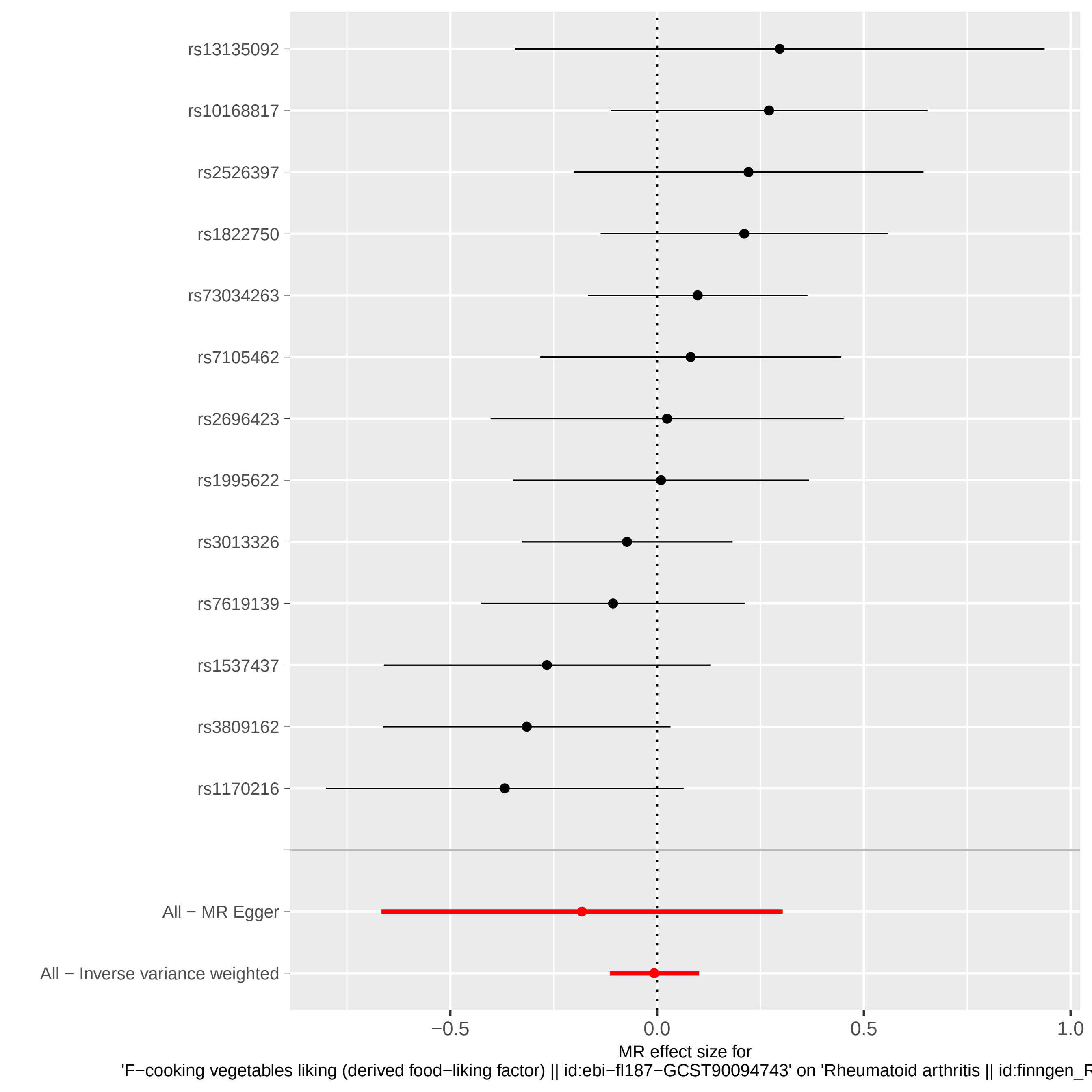
rs13135092
rs10168817
rs2526397
rs1822750
rs73034263
rs7105462
rs2696423
rs1995622
rs3013326
rs7619139
rs1537437
rs3809162
rs1170216
All − MR Egger
All − Inverse variance weighted
−0.5
0.0
0.5
1.0
MR effect size for
'F−cooking vegetables liking (derived food−liking factor) || id:ebi−fl187−GCST90094743' on 'Rheumatoid arthritis || id:finngen_R

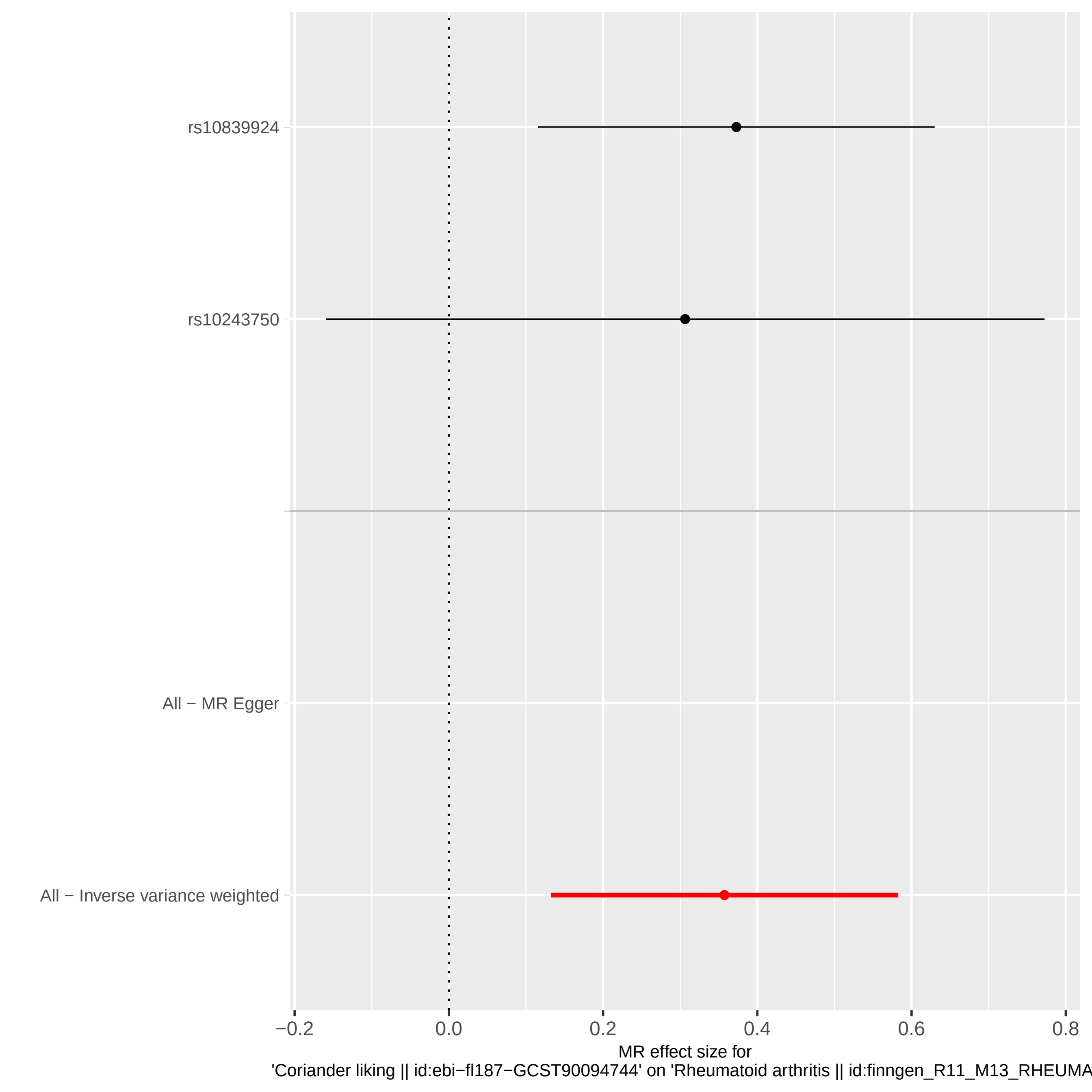
rs10839924
rs10243750
All − MR Egger
All − Inverse variance weighted
−0.2
0.0
0.2
0.4
0.6
0.8
MR effect size for
'Coriander liking || id:ebi−fl187−GCST90094744' on 'Rheumatoid arthritis || id:finngen_R11_M13_RHEUMA

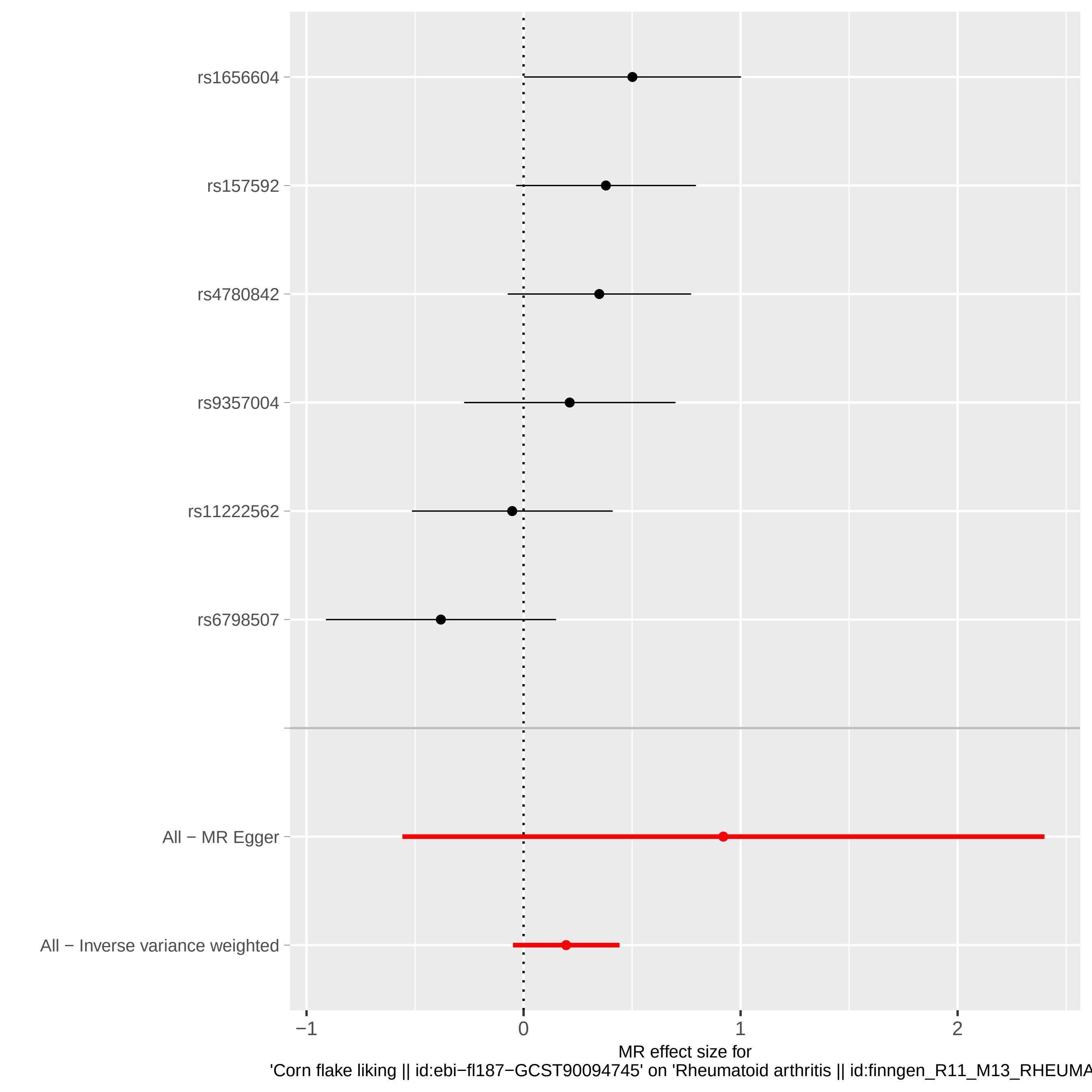
rs1656604
rs157592
rs4780842
rs9357004
rs11222562
rs6798507
All − MR Egger
All − Inverse variance weighted
−1
0
1
2
MR effect size for
'Corn flake liking || id:ebi−fl187−GCST90094745' on 'Rheumatoid arthritis || id:finngen_R11_M13_RHEUMA

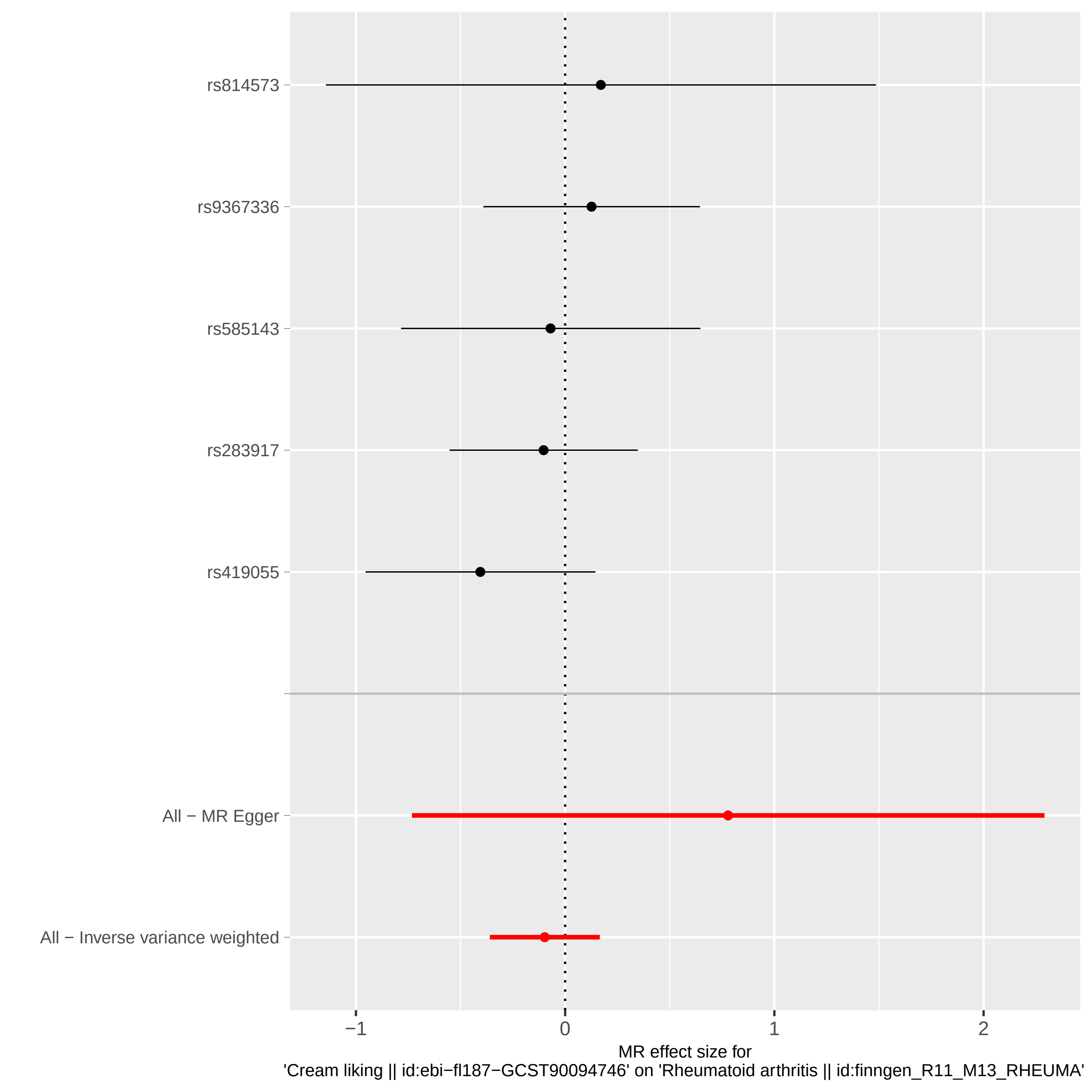
rs814573
rs9367336
rs585143
rs283917
rs419055
All − MR Egger
All − Inverse variance weighted
−1
0
1
2
MR effect size for
'Cream liking || id:ebi−fl187−GCST90094746' on 'Rheumatoid arthritis || id:finngen_R11_M13_RHEUMA'

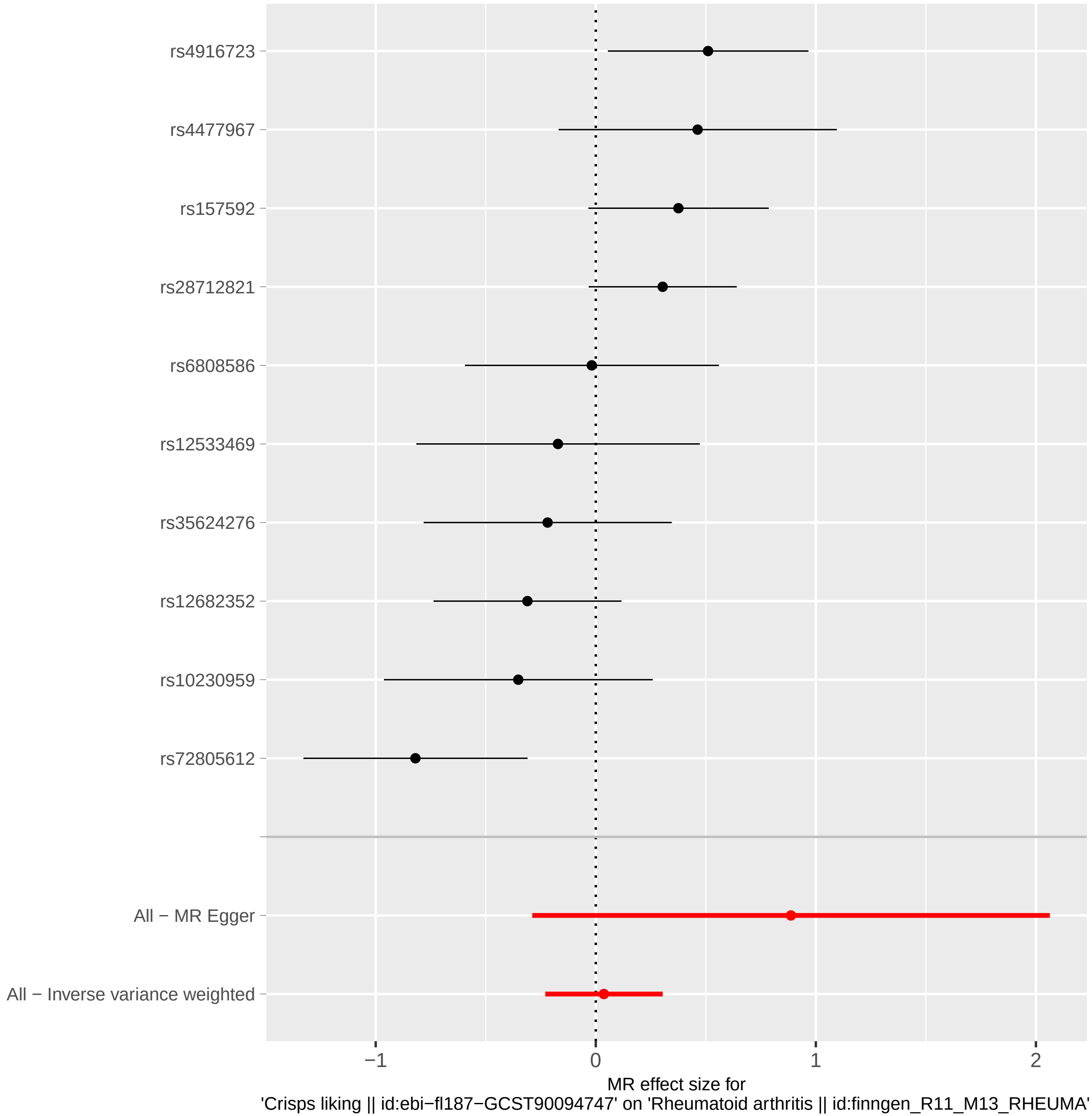
rs4916723
rs4477967
rs157592
rs28712821
rs6808586
rs12533469
rs35624276
rs12682352
rs10230959
rs72805612
All − MR Egger
All − Inverse variance weighted
−1
0
1
2
MR effect size for
'Crisps liking || id:ebi−fl187−GCST90094747' on 'Rheumatoid arthritis || id:finngen_R11_M13_RHEUMA'

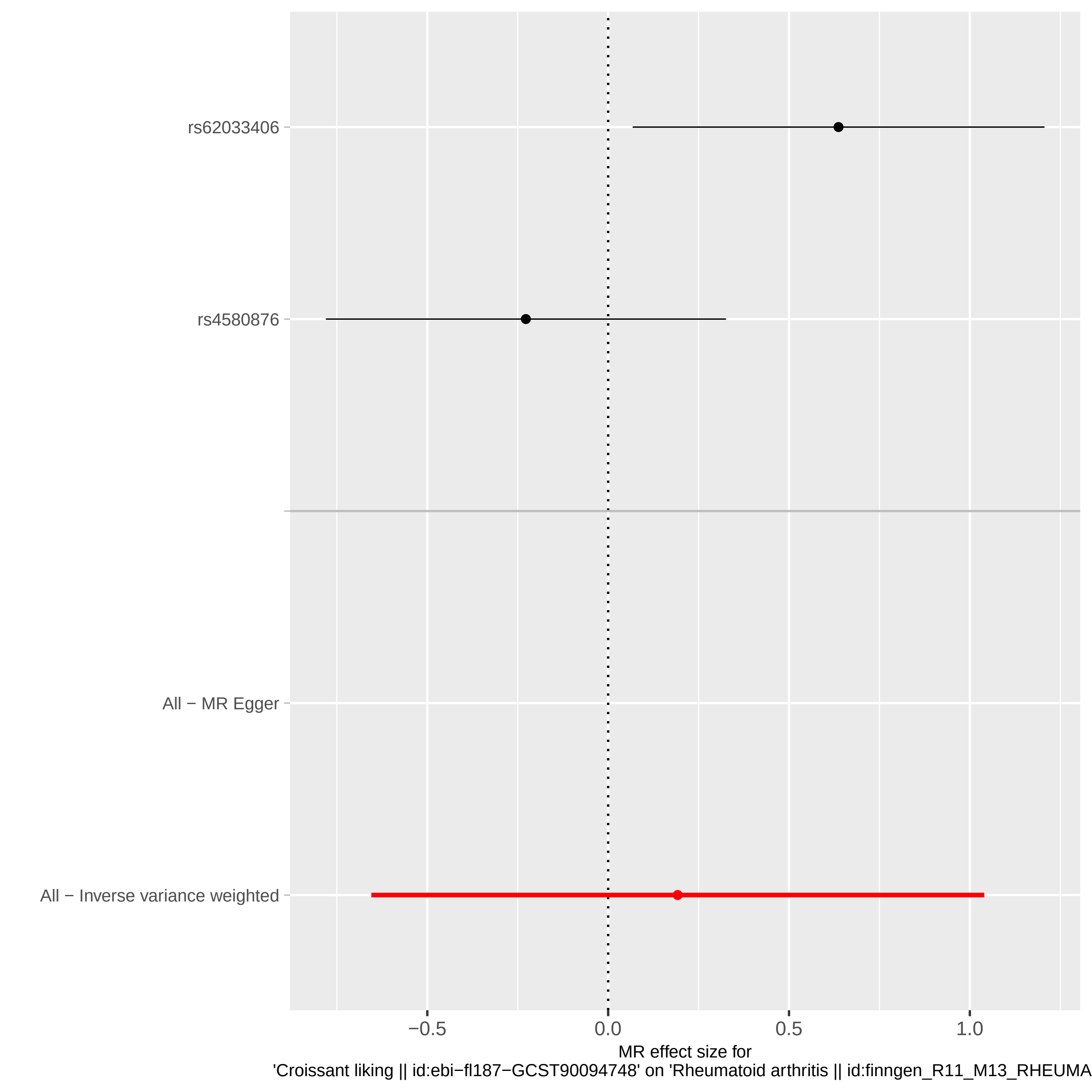

rs62033406
rs4580876
All − MR Egger
All − Inverse variance weighted
−0.5
0.0
0.5
1.0
MR effect size for
'Croissant liking || id:ebi−fl187−GCST90094748' on 'Rheumatoid arthritis || id:finngen_R11_M13_RHEUMA

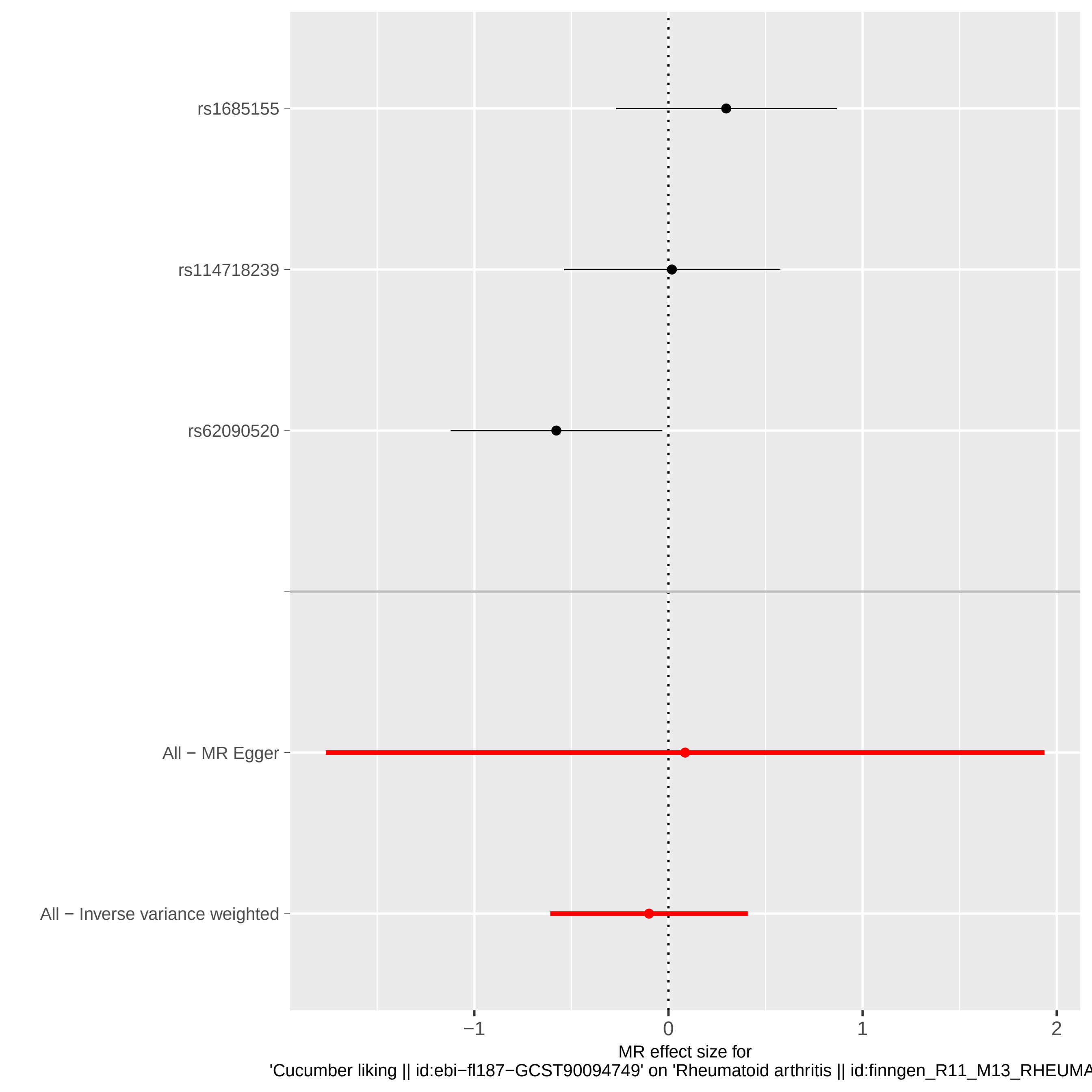
rs1685155
rs114718239
rs62090520
All − MR Egger
All − Inverse variance weighted
−1
0
1
2
MR effect size for
'Cucumber liking || id:ebi−fl187−GCST90094749' on 'Rheumatoid arthritis || id:finngen_R11_M13_RHEUMA

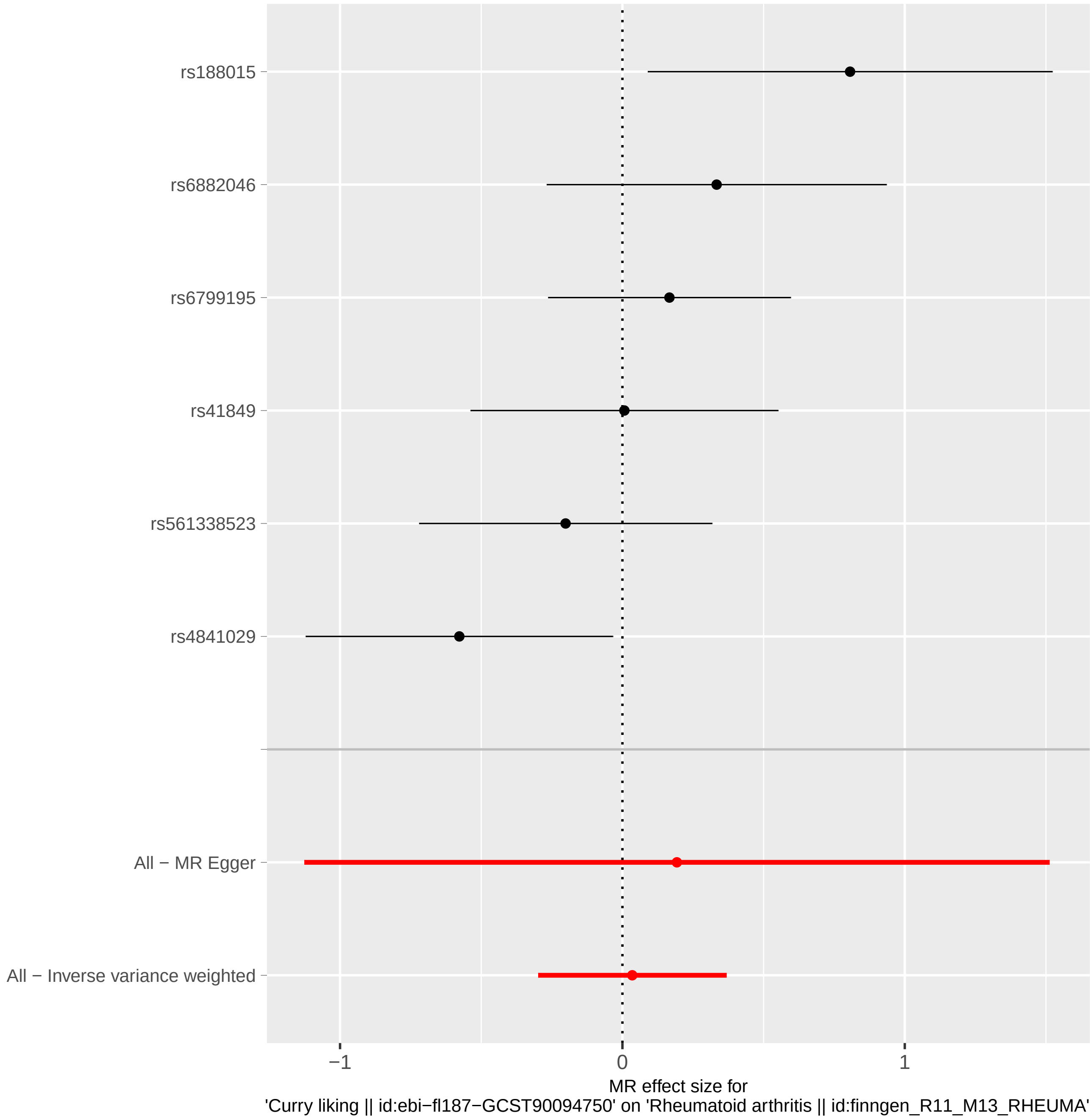

rs188015
rs6882046
rs6799195
rs41849
rs561338523
rs4841029
All − MR Egger
All − Inverse variance weighted
−1
0
1
MR effect size for
'Curry liking || id:ebi−fl187−GCST90094750' on 'Rheumatoid arthritis || id:finngen_R11_M13_RHEUMA'

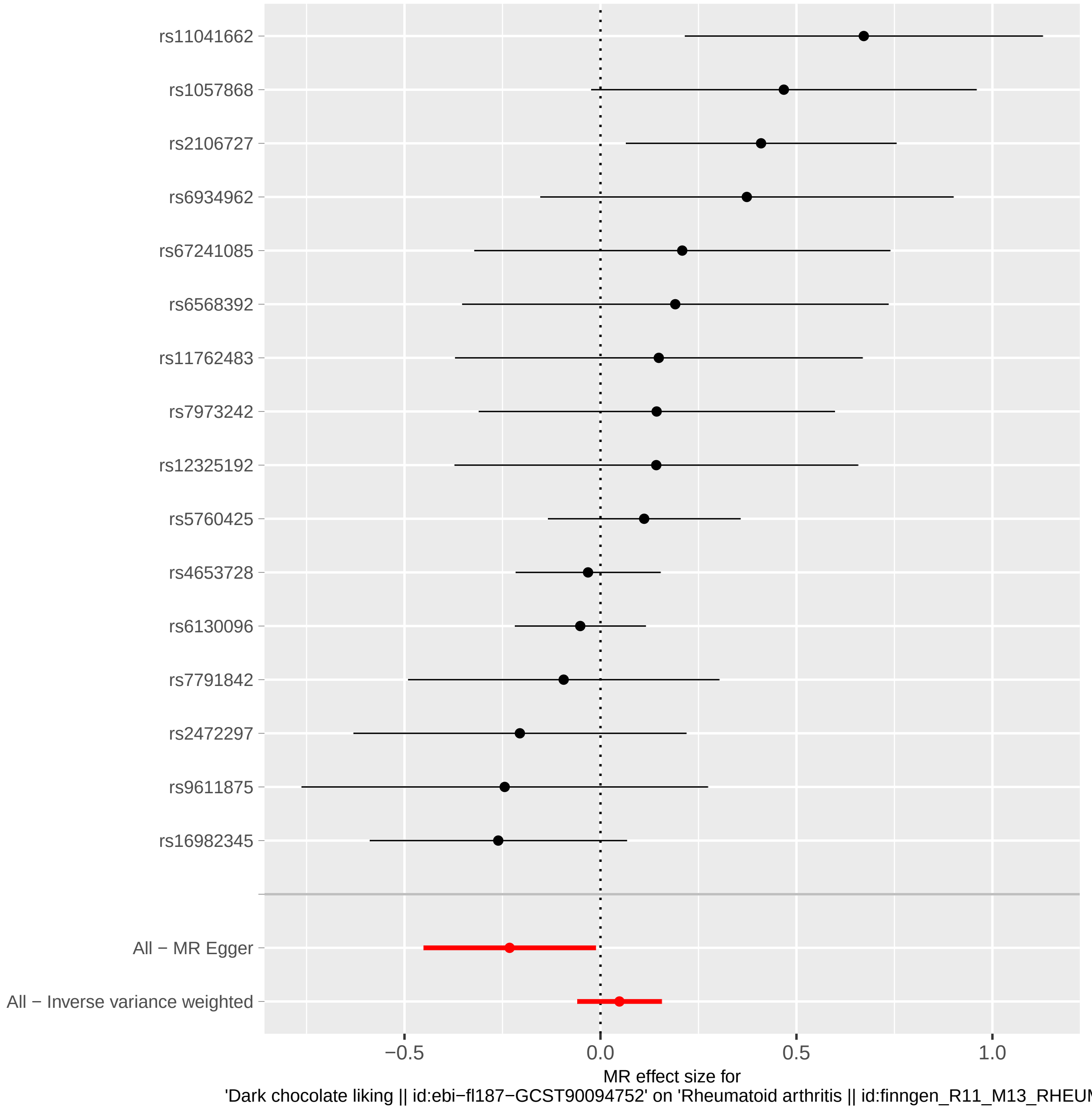
rs11041662
rs1057868
rs2106727
rs6934962
rs67241085
rs6568392
rs11762483
rs7973242
rs12325192
rs5760425
rs4653728
rs6130096
rs7791842
rs2472297
rs9611875
rs16982345
All − MR Egger
All − Inverse variance weighted
−0.5
0.0
0.5
1.0
MR effect size for
'Dark chocolate liking || id:ebi−fl187−GCST90094752' on 'Rheumatoid arthritis || id:finngen_R11_M13_RHEUM

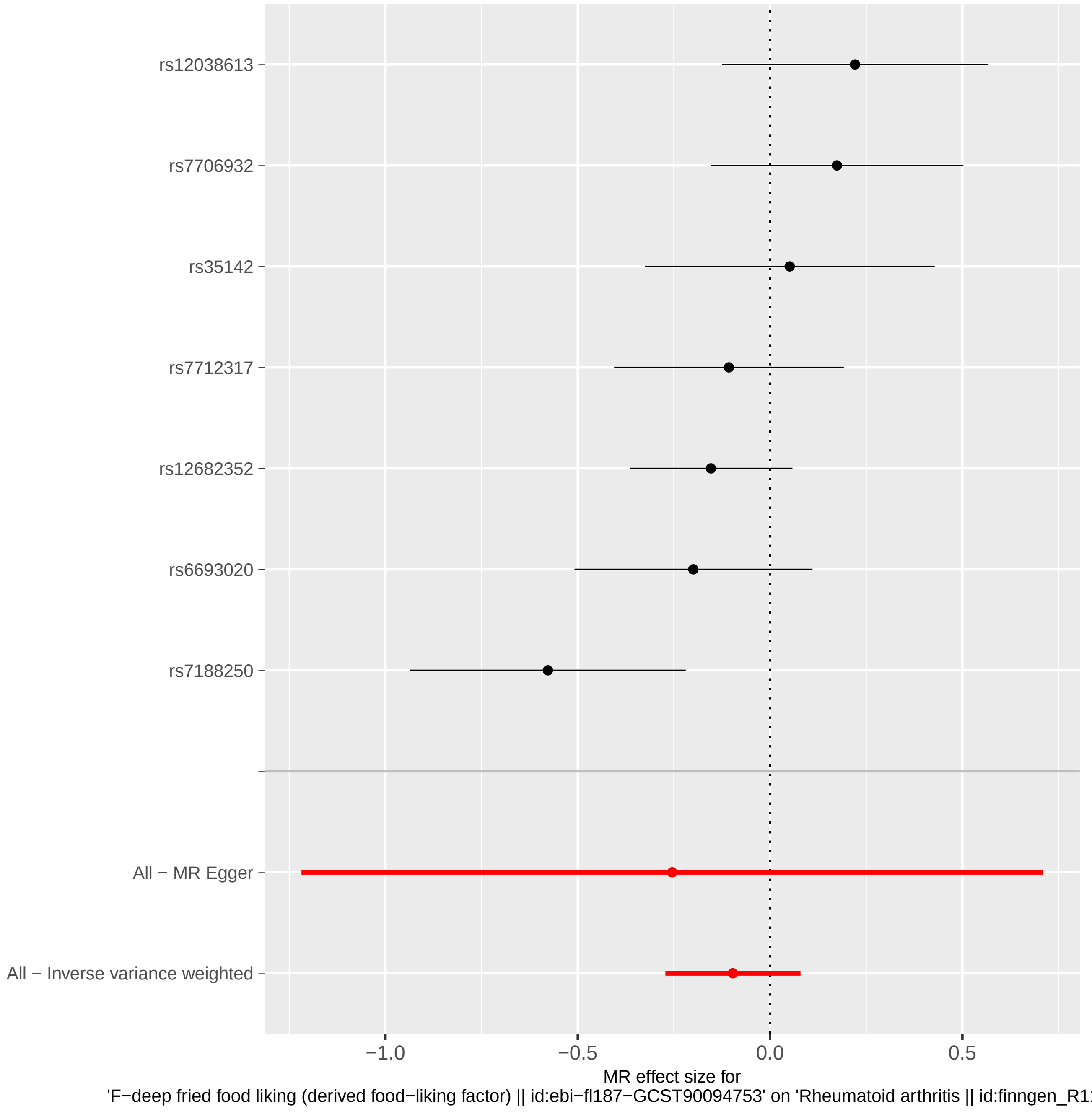

rs12038613
rs7706932
rs35142
rs7712317
rs12682352
rs6693020
rs7188250
All − MR Egger
All − Inverse variance weighted
−1.0
−0.5
0.0
0.5
MR effect size for
'F−deep fried food liking (derived food−liking factor) || id:ebi−fl187−GCST90094753' on 'Rheumatoid arthritis || id:finngen_R1

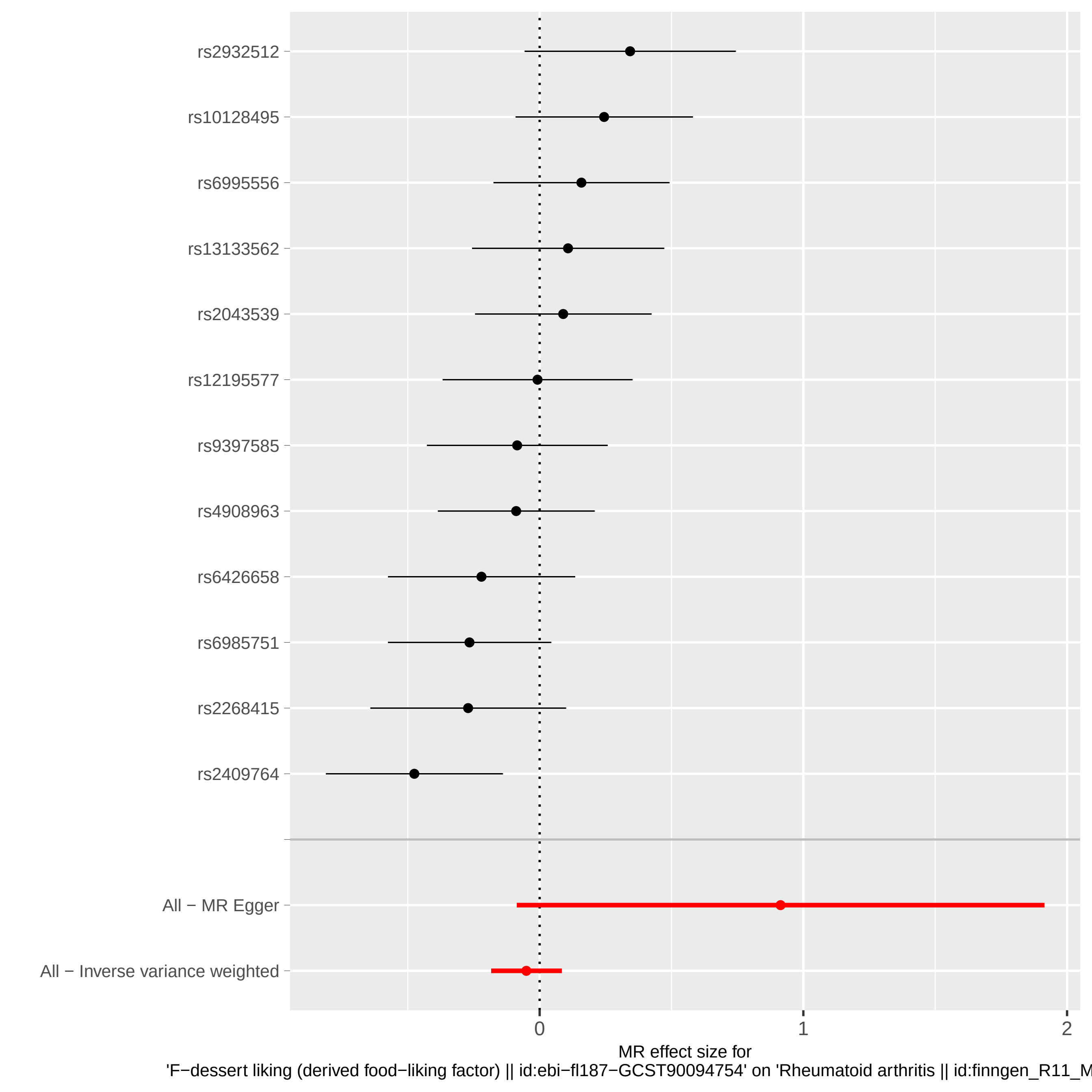
rs2932512
rs10128495
rs6995556
rs13133562
rs2043539
rs12195577
rs9397585
rs4908963
rs6426658
rs6985751
rs2268415
rs2409764
All − MR Egger
All − Inverse variance weighted
0
1
2
MR effect size for
'F−dessert liking (derived food−liking factor) || id:ebi−fl187−GCST90094754' on 'Rheumatoid arthritis || id:finngen_R11_M

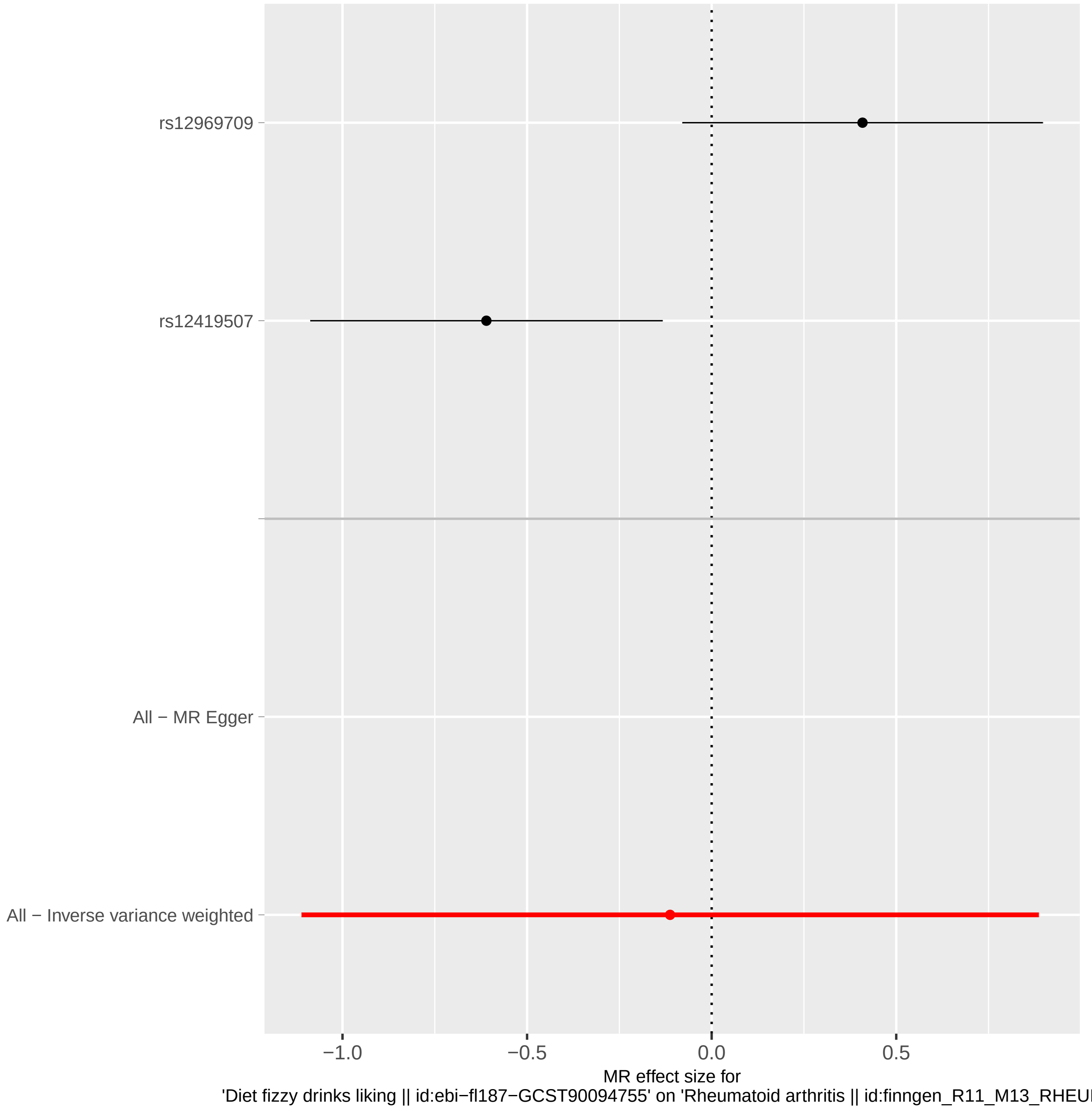
rs12969709
rs12419507
All − MR Egger
All − Inverse variance weighted
−1.0
−0.5
0.0
0.5
MR effect size for
'Diet fizzy drinks liking || id:ebi−fl187−GCST90094755' on 'Rheumatoid arthritis || id:finngen_R11_M13_RHEU

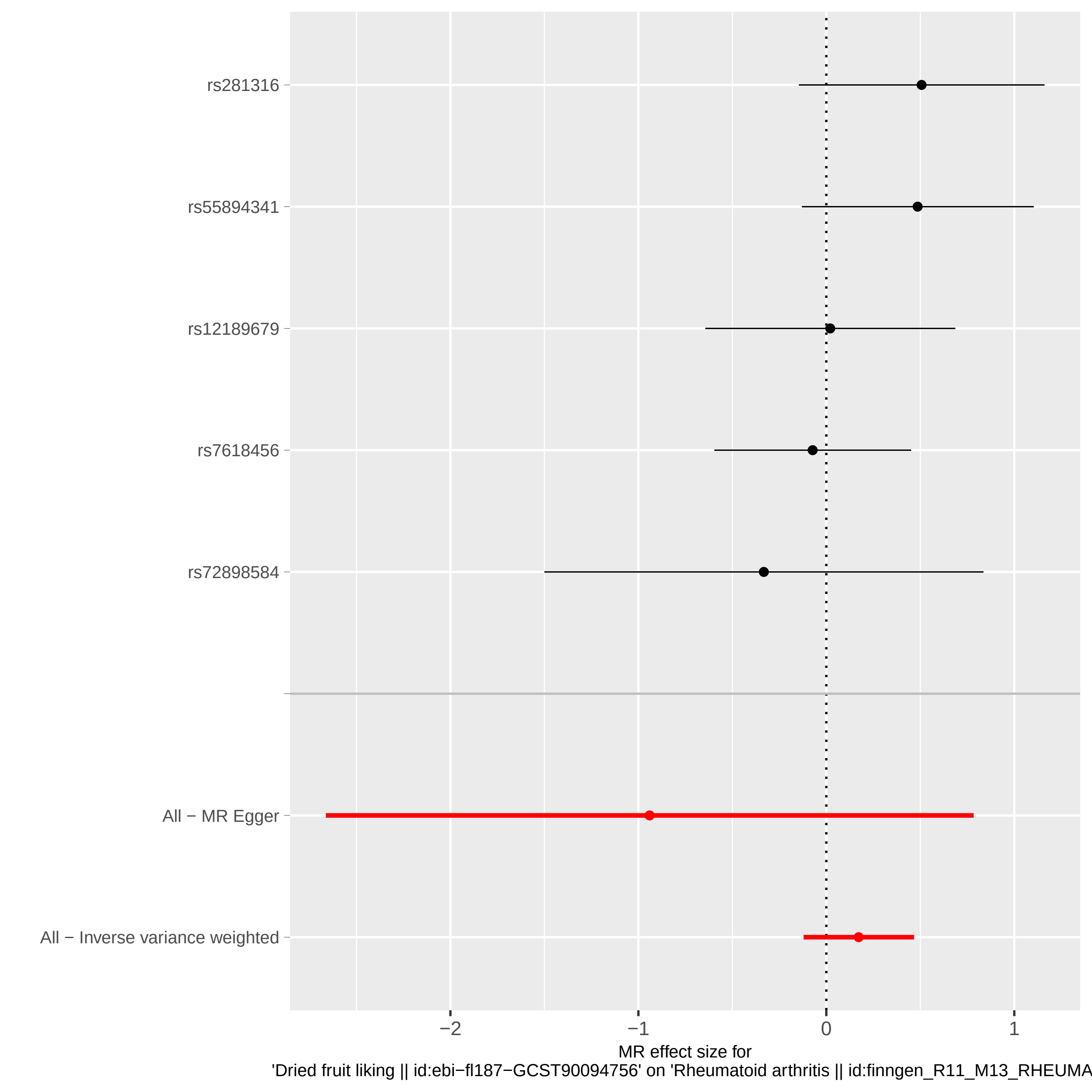
rs281316
rs55894341
rs12189679
rs7618456
rs72898584
All − MR Egger
All − Inverse variance weighted
−2
−1
0
1
MR effect size for
'Dried fruit liking || id:ebi−fl187−GCST90094756' on 'Rheumatoid arthritis || id:finngen_R11_M13_RHEUMA

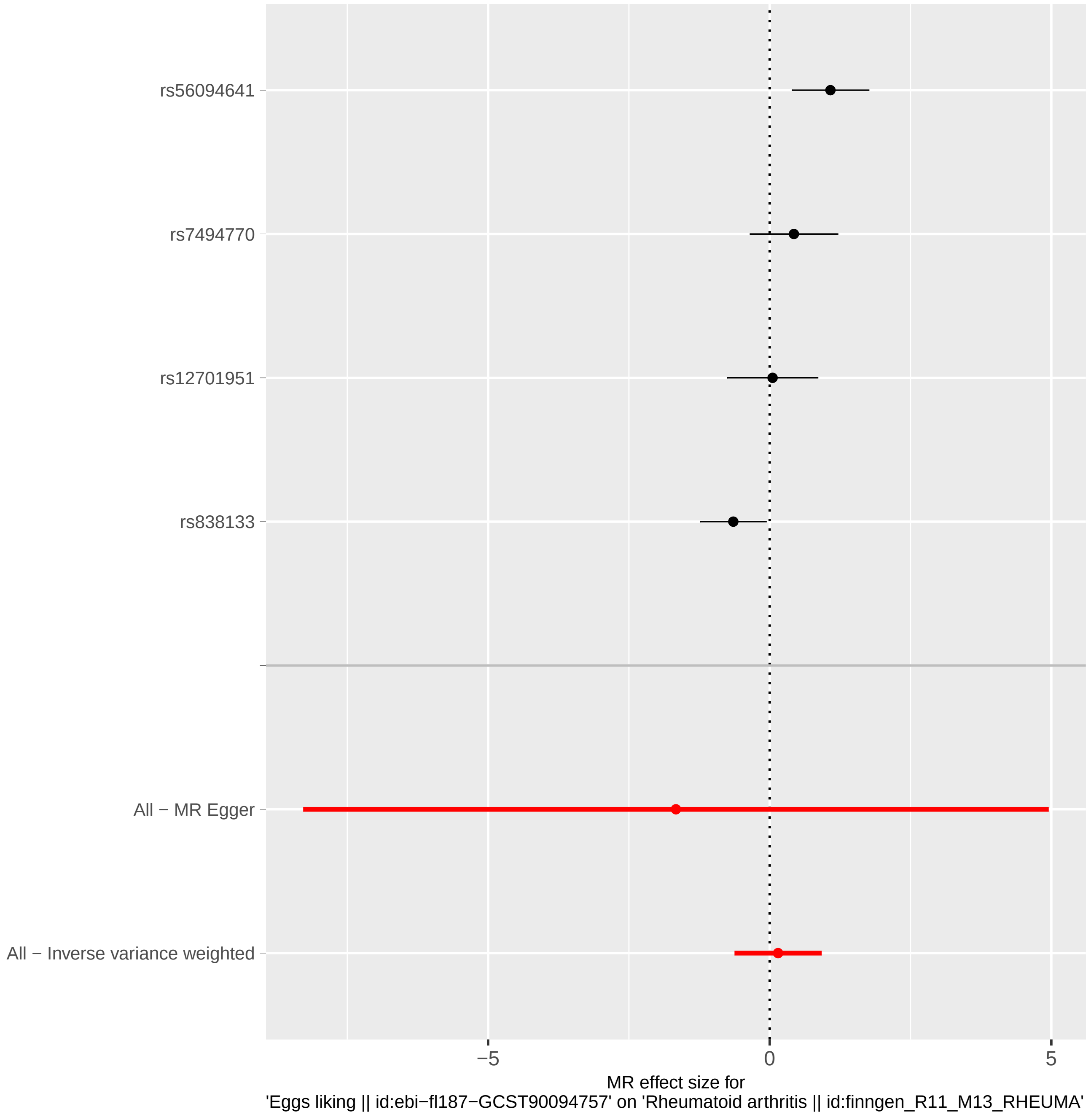
rs56094641
rs7494770
rs12701951
rs838133
All − MR Egger
All − Inverse variance weighted
−5
0
5
MR effect size for
'Eggs liking || id:ebi−fl187−GCST90094757' on 'Rheumatoid arthritis || id:finngen_R11_M13_RHEUMA'

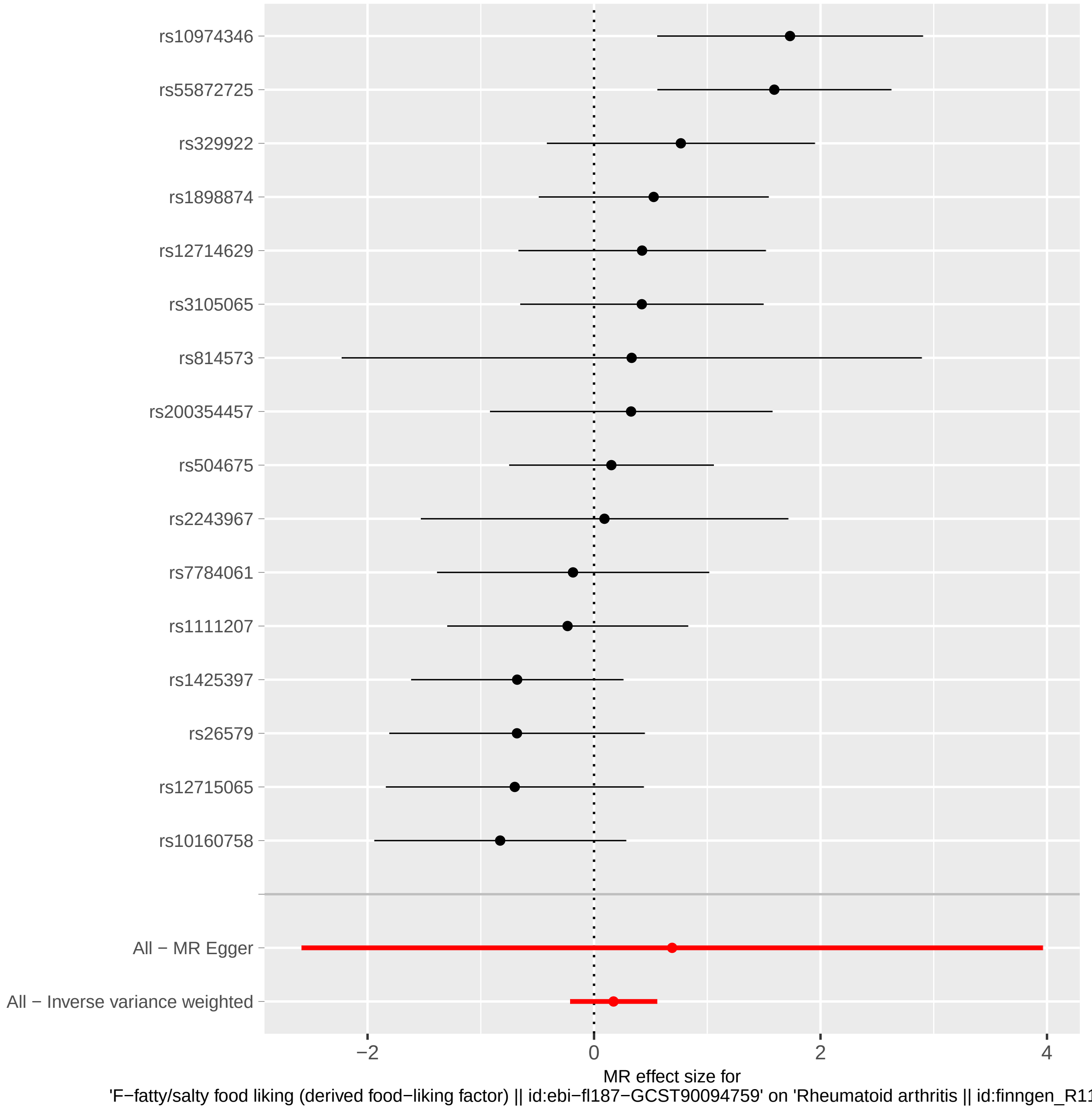

rs10974346
rs55872725
rs329922
rs1898874
rs12714629
rs3105065
rs814573
rs200354457
rs504675
rs2243967
rs7784061
rs1111207
rs1425397
rs26579
rs12715065
rs10160758
All − MR Egger
All − Inverse variance weighted
−2
0
2
4
MR effect size for
'F−fatty/salty food liking (derived food−liking factor) || id:ebi−fl187−GCST90094759' on 'Rheumatoid arthritis || id:finngen_R11

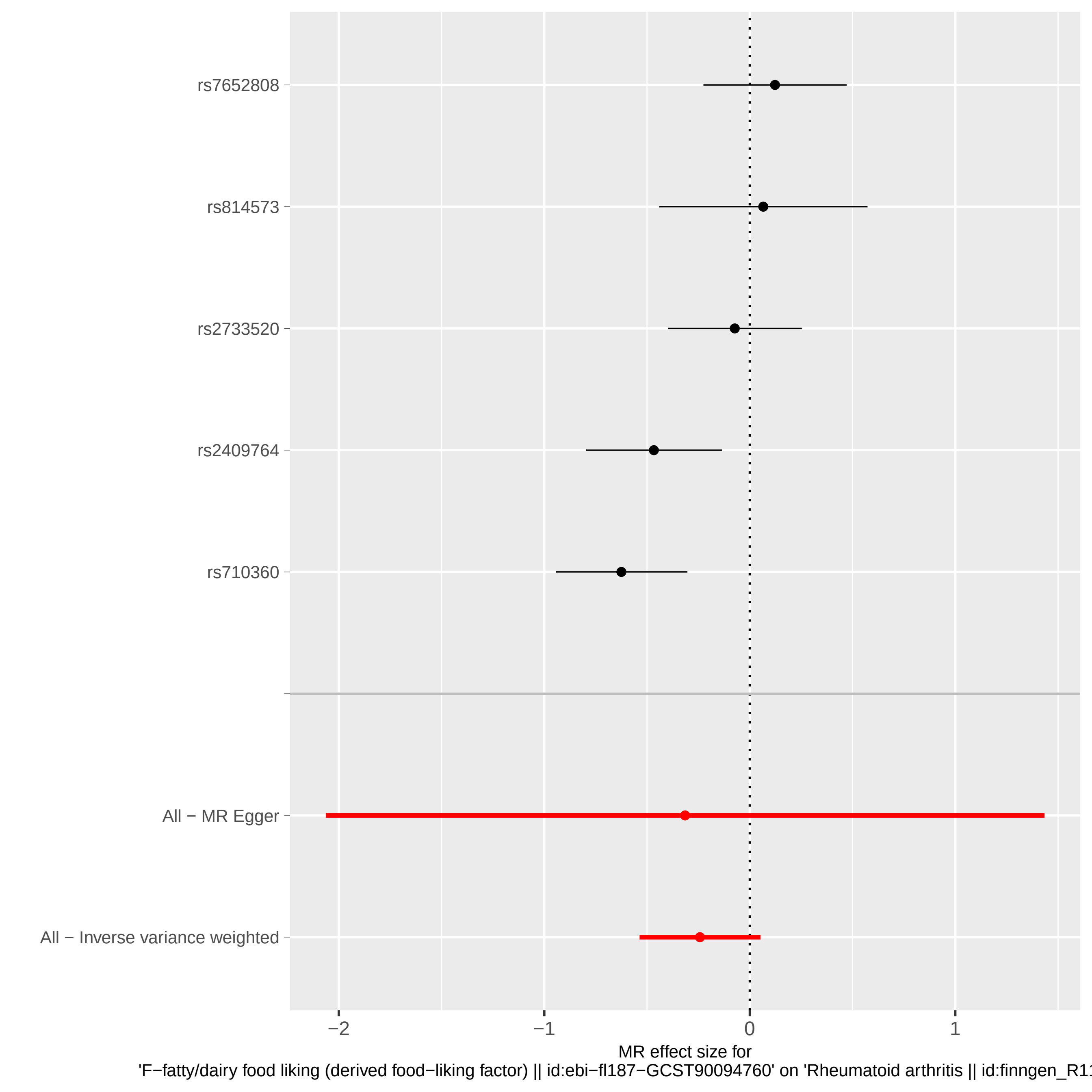
rs7652808
rs814573
rs2733520
rs2409764
rs710360
All − MR Egger
All − Inverse variance weighted
−2
−1
0
1
MR effect size for
'F−fatty/dairy food liking (derived food−liking factor) || id:ebi−fl187−GCST90094760' on 'Rheumatoid arthritis || id:finngen_R1

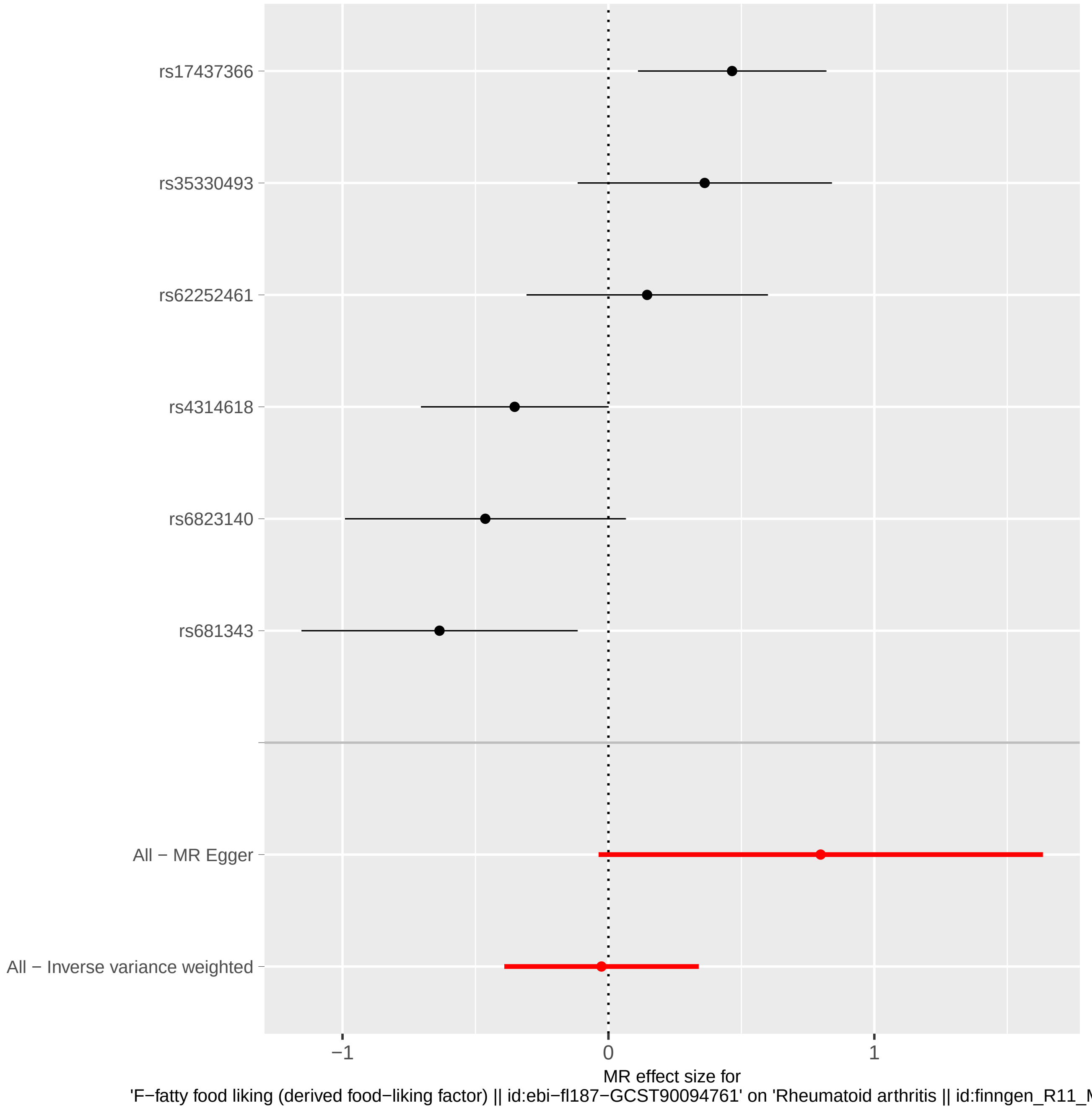
rs17437366
rs35330493
rs62252461
rs4314618
rs6823140
rs681343
All − MR Egger
All − Inverse variance weighted
−1
0
1
MR effect size for
'F−fatty food liking (derived food−liking factor) || id:ebi−fl187−GCST90094761' on 'Rheumatoid arthritis || id:finngen_R11_M

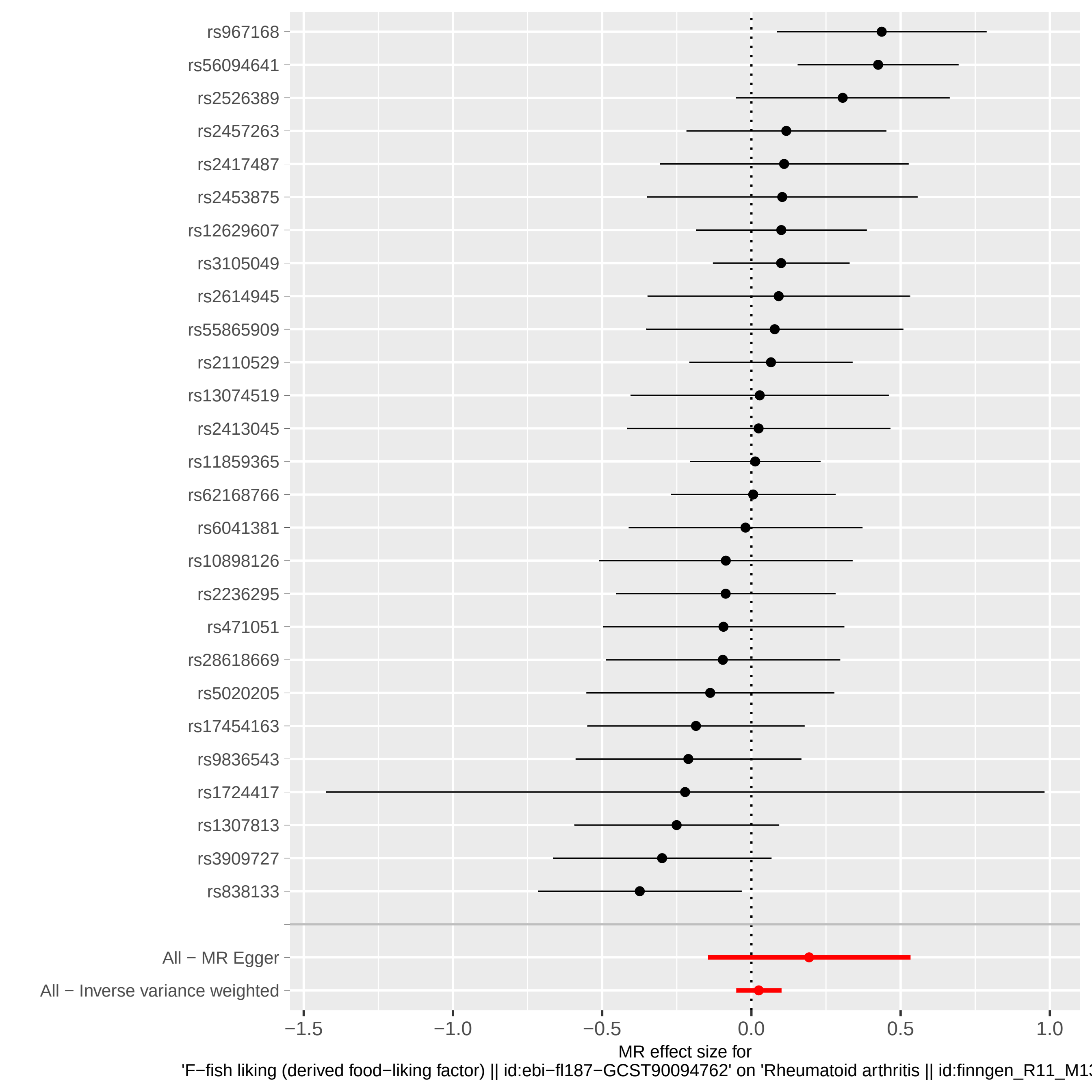
rs967168
rs56094641
rs2526389
rs2457263
rs2417487
rs2453875
rs12629607
rs3105049
rs2614945
rs55865909
rs2110529
rs13074519
rs2413045
rs11859365
rs62168766
rs6041381
rs10898126
rs2236295
rs471051
rs28618669
rs5020205
rs17454163
rs9836543
rs1724417
rs1307813
rs3909727
rs838133
All − MR Egger
All − Inverse variance weighted
−1.5
−1.0
−0.5
0.0
0.5
1.0
MR effect size for
'F−fish liking (derived food−liking factor) || id:ebi−fl187−GCST90094762' on 'Rheumatoid arthritis || id:finngen_R11_M13

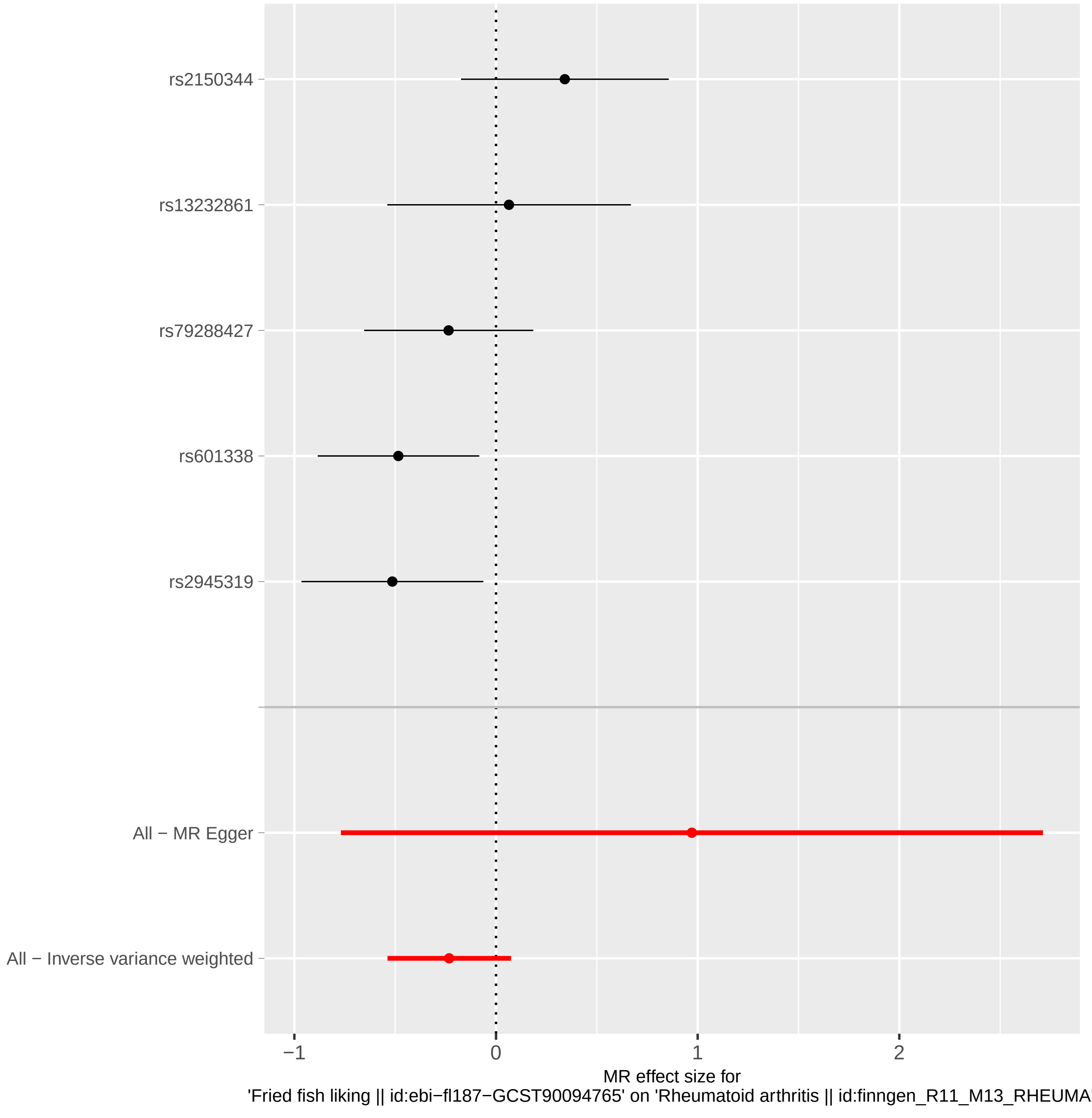
rs2150344
rs13232861
rs79288427
rs601338
rs2945319
All − MR Egger
All − Inverse variance weighted
−1
0
1
2
MR effect size for
'Fried fish liking || id:ebi−fl187−GCST90094765' on 'Rheumatoid arthritis || id:finngen_R11_M13_RHEUMA

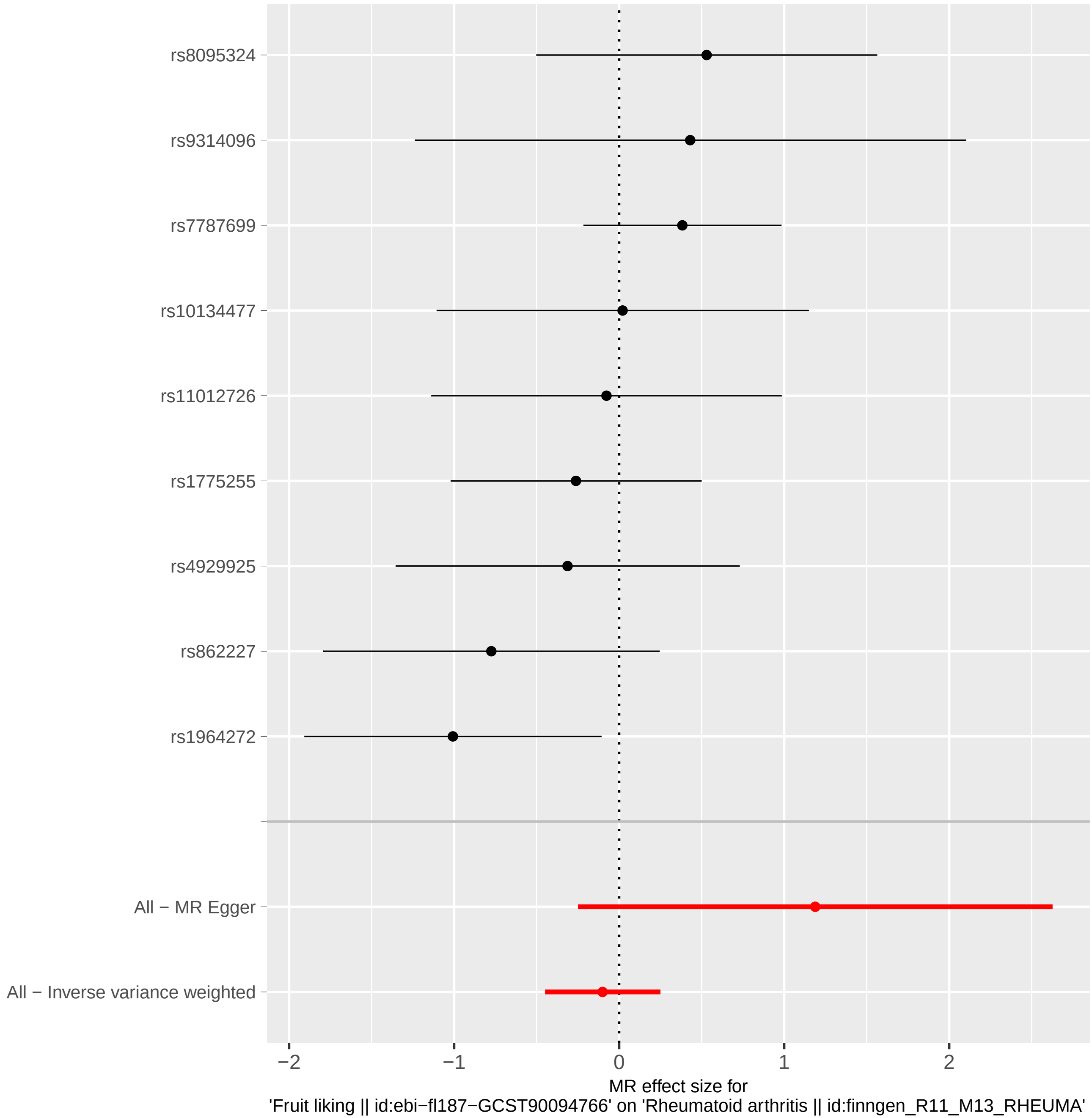

rs8095324
rs9314096
rs7787699
rs10134477
rs11012726
rs1775255
rs4929925
rs862227
rs1964272
All − MR Egger
All − Inverse variance weighted
−2
−1
0
1
2
MR effect size for
'Fruit liking || id:ebi−fl187−GCST90094766' on 'Rheumatoid arthritis || id:finngen_R11_M13_RHEUMA'

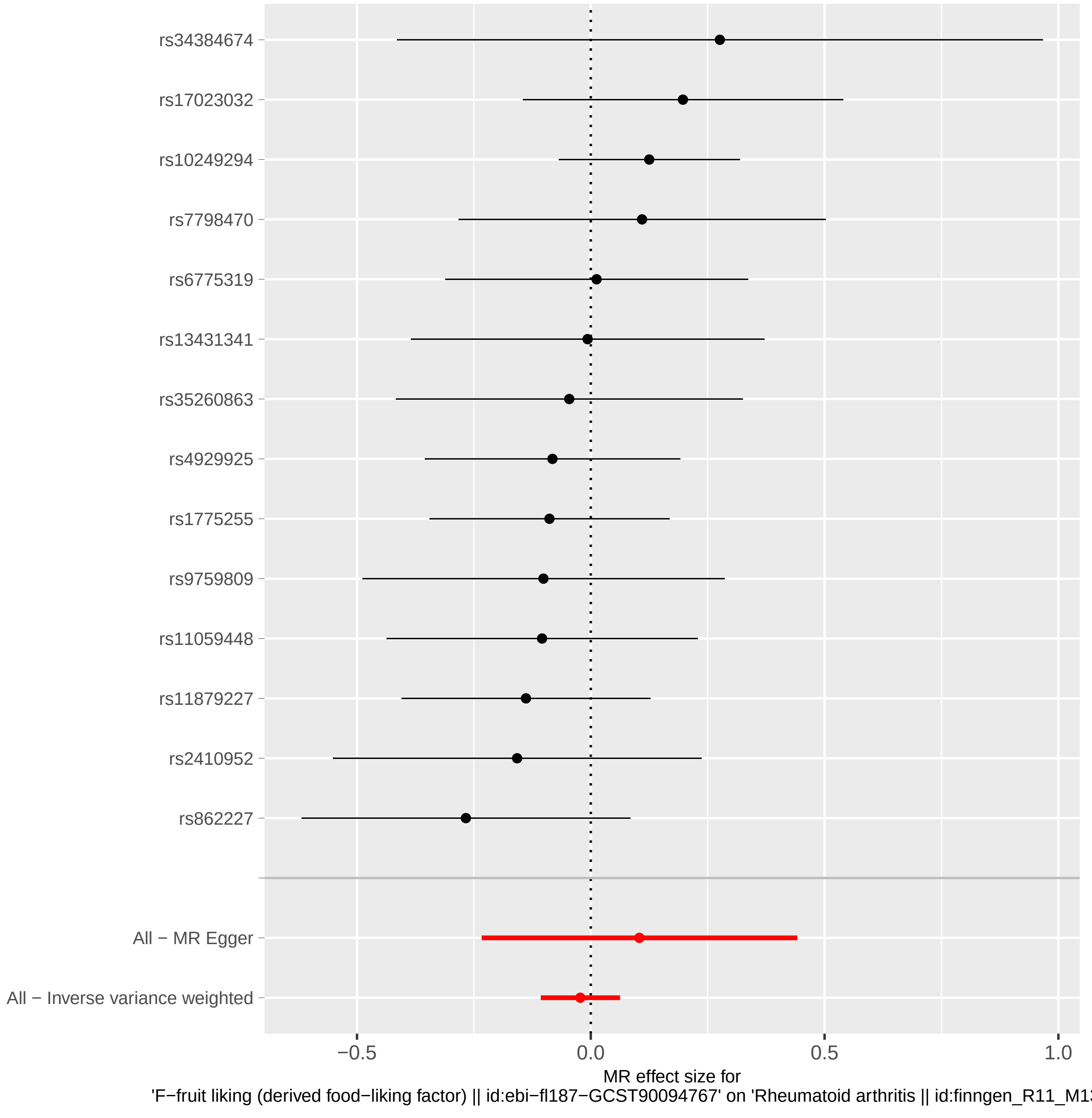

rs34384674
rs17023032
rs10249294
rs7798470
rs6775319
rs13431341
rs35260863
rs4929925
rs1775255
rs9759809
rs11059448
rs11879227
rs2410952
rs862227
All − MR Egger
All − Inverse variance weighted
−0.5
0.0
0.5
1.0
MR effect size for
'F−fruit liking (derived food−liking factor) || id:ebi−fl187−GCST90094767' on 'Rheumatoid arthritis || id:finngen_R11_M1

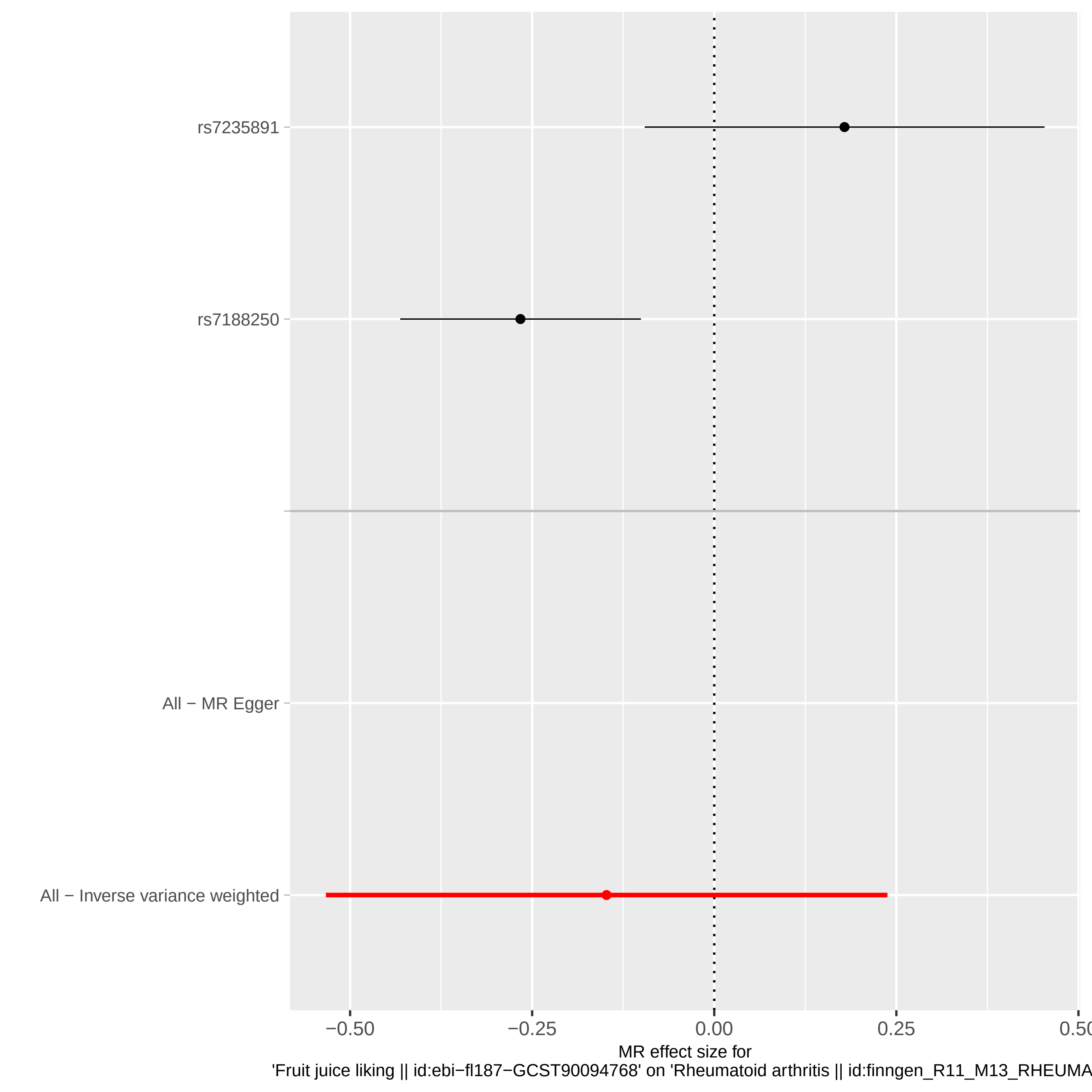
rs7235891
rs7188250
All − MR Egger
All − Inverse variance weighted
−0.50
−0.25
0.00
0.25
0.50
MR effect size for
'Fruit juice liking || id:ebi−fl187−GCST90094768' on 'Rheumatoid arthritis || id:finngen_R11_M13_RHEUMA

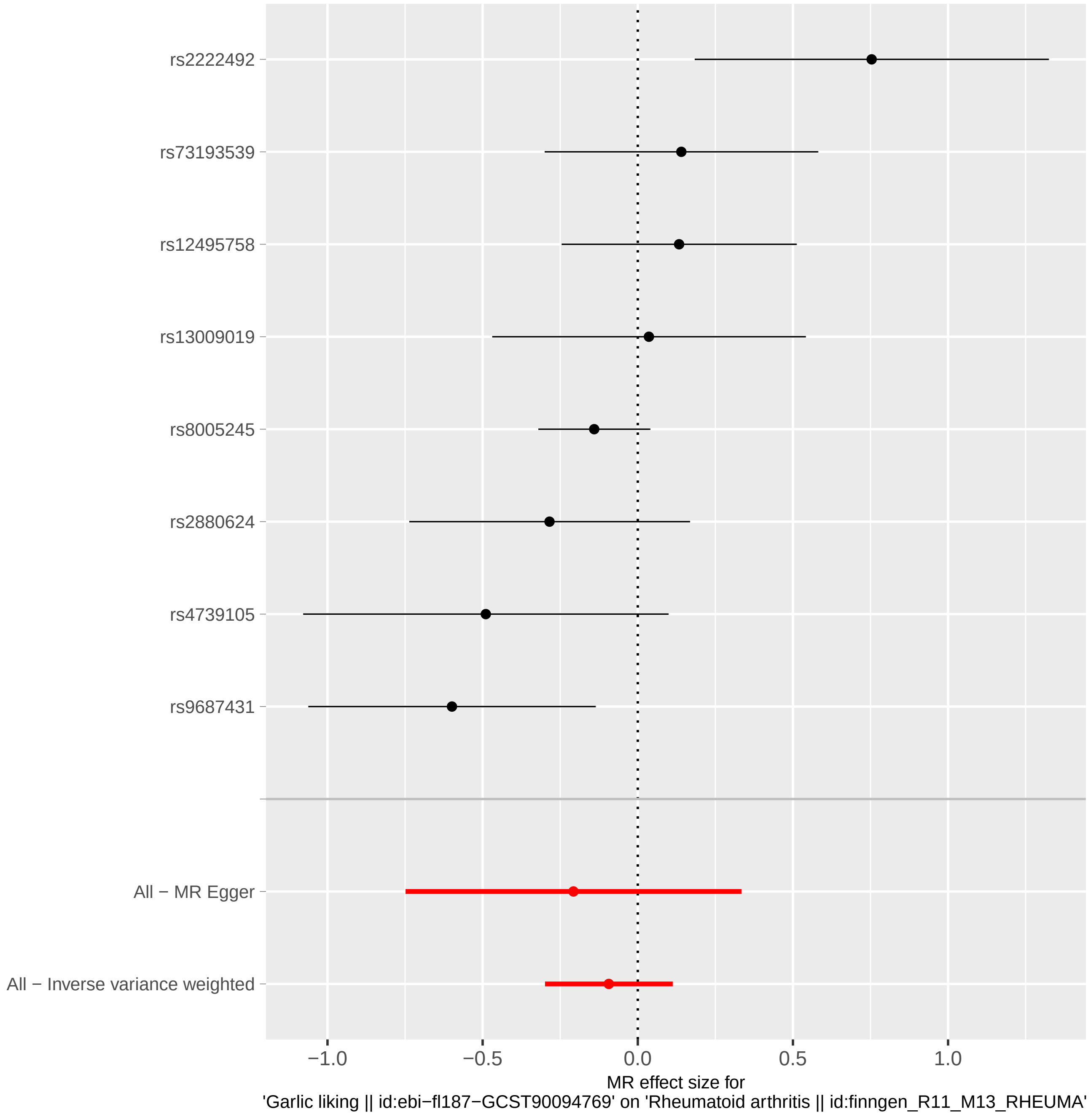
rs2222492
rs73193539
rs12495758
rs13009019
rs8005245
rs2880624
rs4739105
rs9687431
All − MR Egger
All − Inverse variance weighted
−1.0
−0.5
0.0
0.5
1.0
MR effect size for
'Garlic liking || id:ebi−fl187−GCST90094769' on 'Rheumatoid arthritis || id:finngen_R11_M13_RHEUMA'

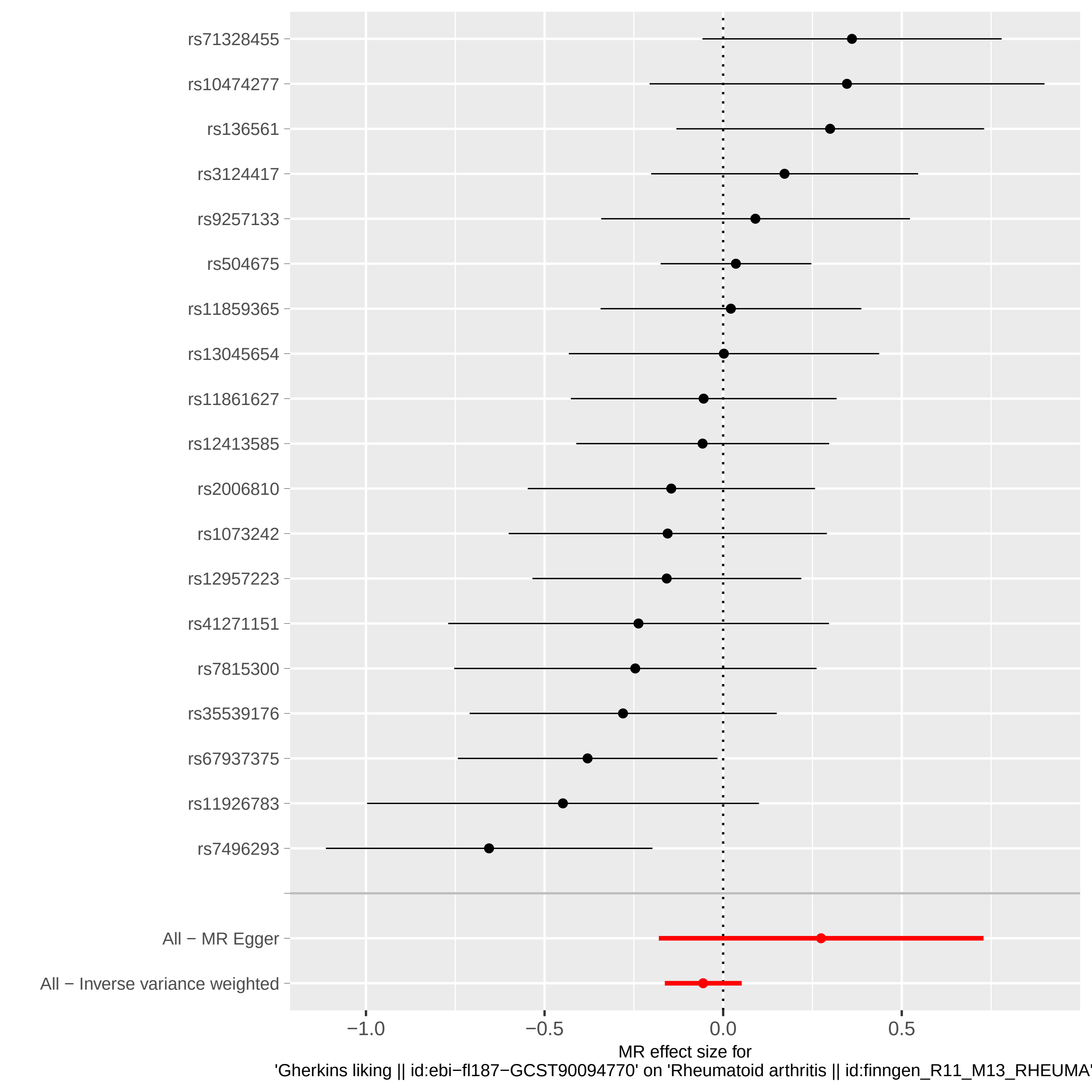
rs71328455
rs10474277
rs136561
rs3124417
rs9257133
rs504675
rs11859365
rs13045654
rs11861627
rs12413585
rs2006810
rs1073242
rs12957223
rs41271151
rs7815300
rs35539176
rs67937375
rs11926783
rs7496293
All − MR Egger
All − Inverse variance weighted
−1.0
−0.5
0.0
0.5
MR effect size for
'Gherkins liking || id:ebi−fl187−GCST90094770' on 'Rheumatoid arthritis || id:finngen_R11_M13_RHEUMA'

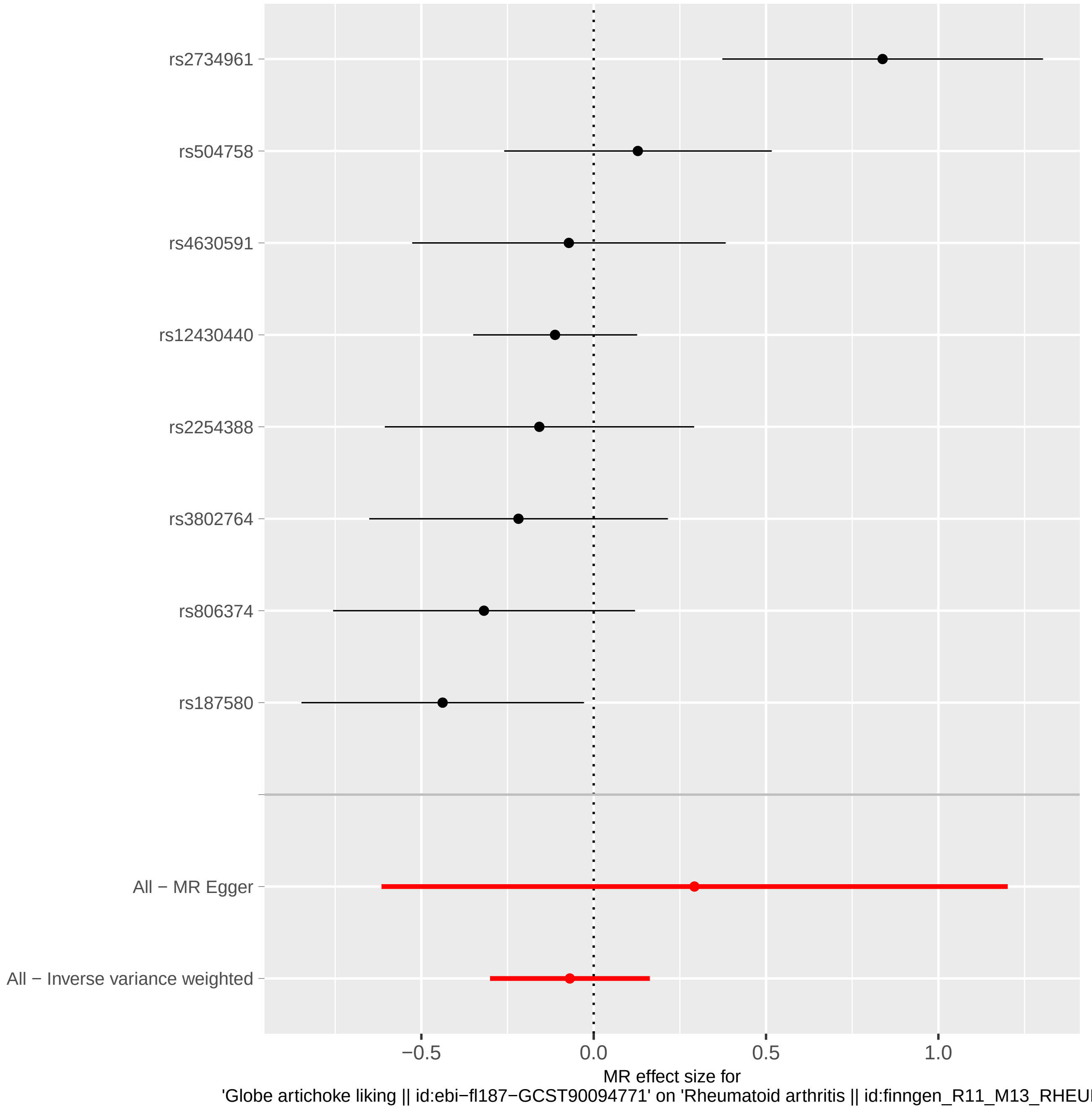
rs2734961
rs504758
rs4630591
rs12430440
rs2254388
rs3802764
rs806374
rs187580
All − MR Egger
All − Inverse variance weighted
−0.5
0.0
0.5
1.0
MR effect size for
'Globe artichoke liking || id:ebi−fl187−GCST90094771' on 'Rheumatoid arthritis || id:finngen_R11_M13_RHEU

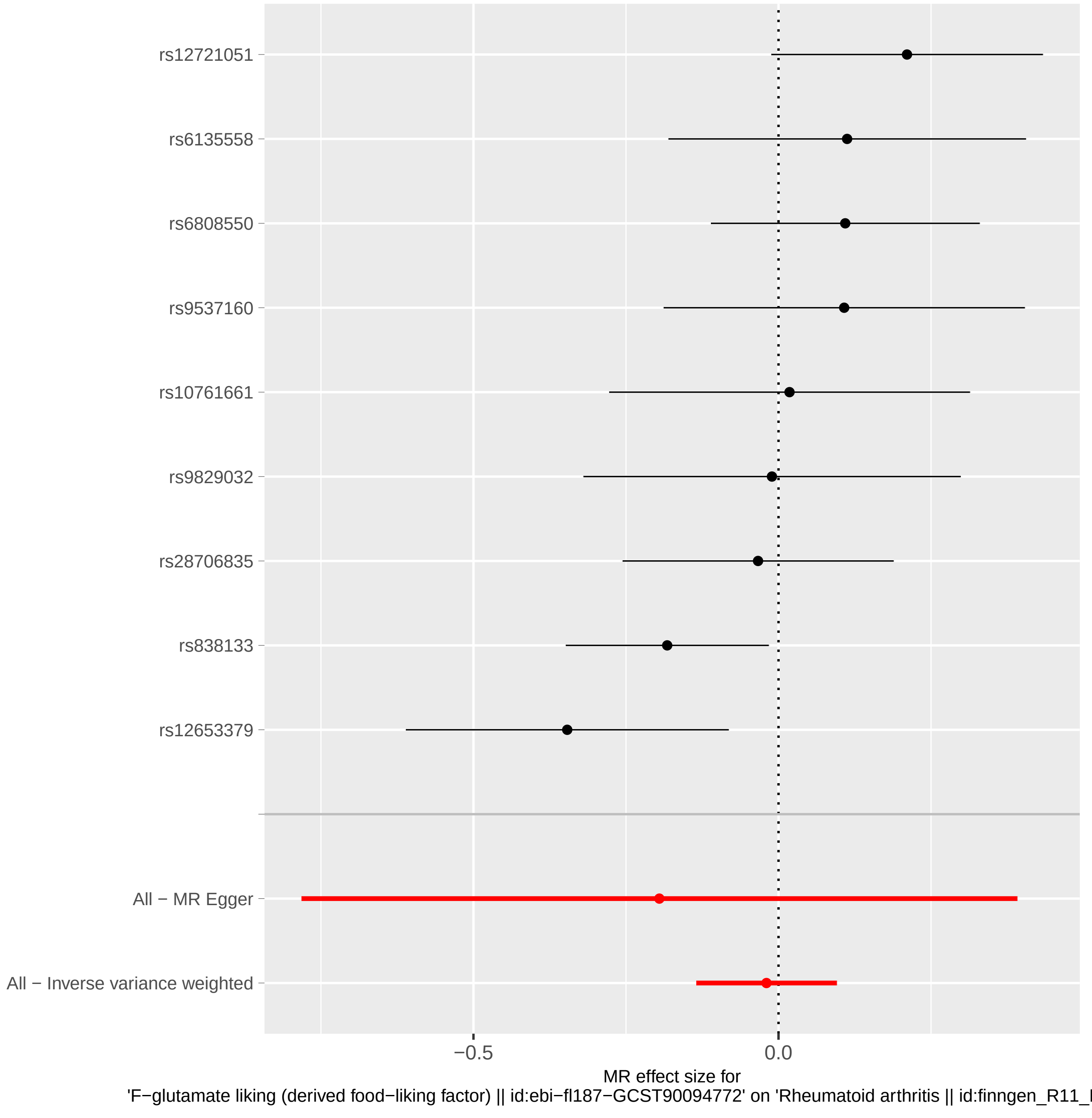

rs12721051
rs6135558
rs6808550
rs9537160
rs10761661
rs9829032
rs28706835
rs838133
rs12653379
All − MR Egger
All − Inverse variance weighted
−0.5
0.0
MR effect size for
'F−glutamate liking (derived food−liking factor) || id:ebi−fl187−GCST90094772' on 'Rheumatoid arthritis || id:finngen_R11_

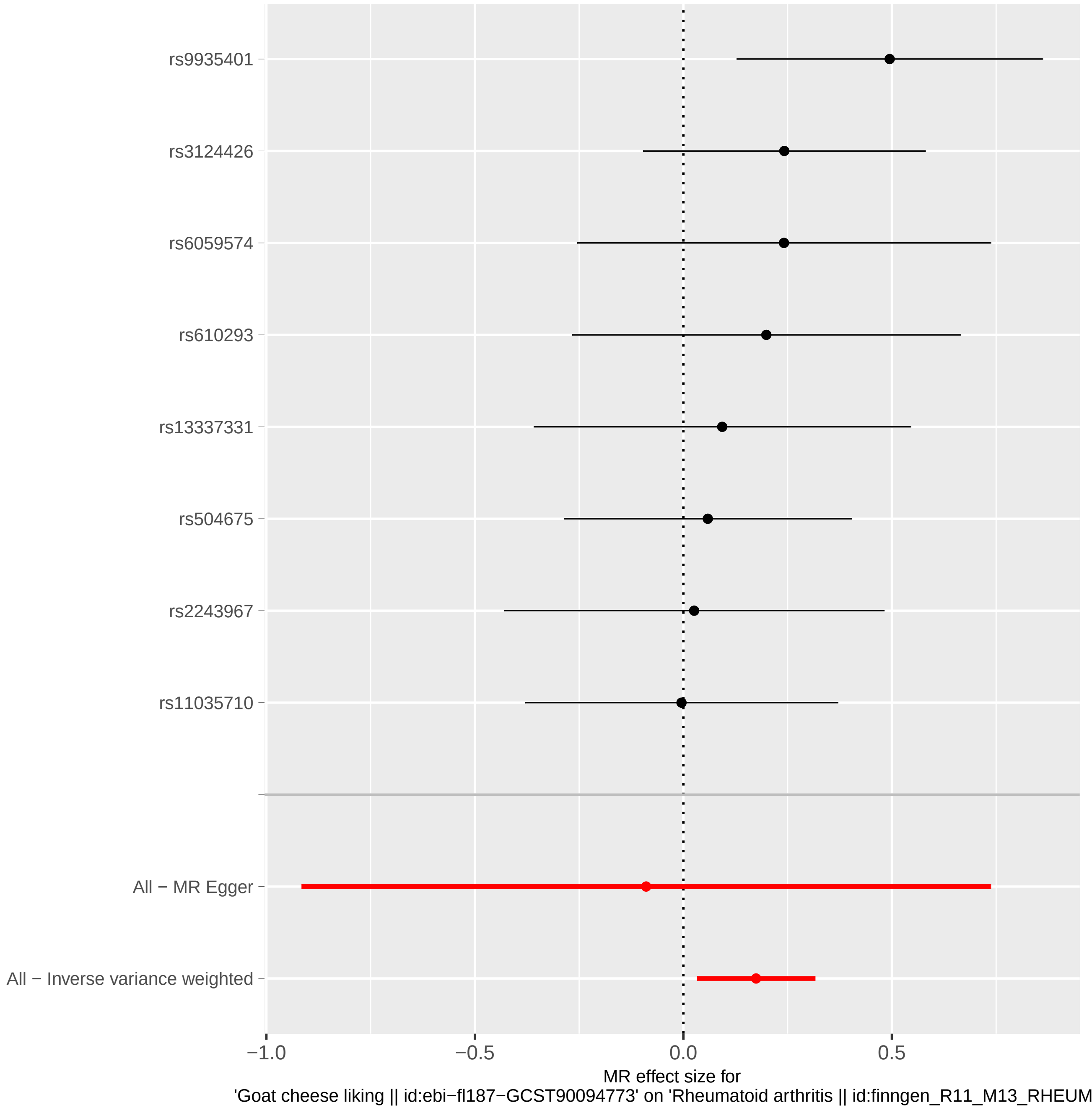

rs9935401
rs3124426
rs6059574
rs610293
rs13337331
rs504675
rs2243967
rs11035710
All − MR Egger
All − Inverse variance weighted
−1.0
−0.5
0.0
0.5
MR effect size for
'Goat cheese liking || id:ebi−fl187−GCST90094773' on 'Rheumatoid arthritis || id:finngen_R11_M13_RHEUM

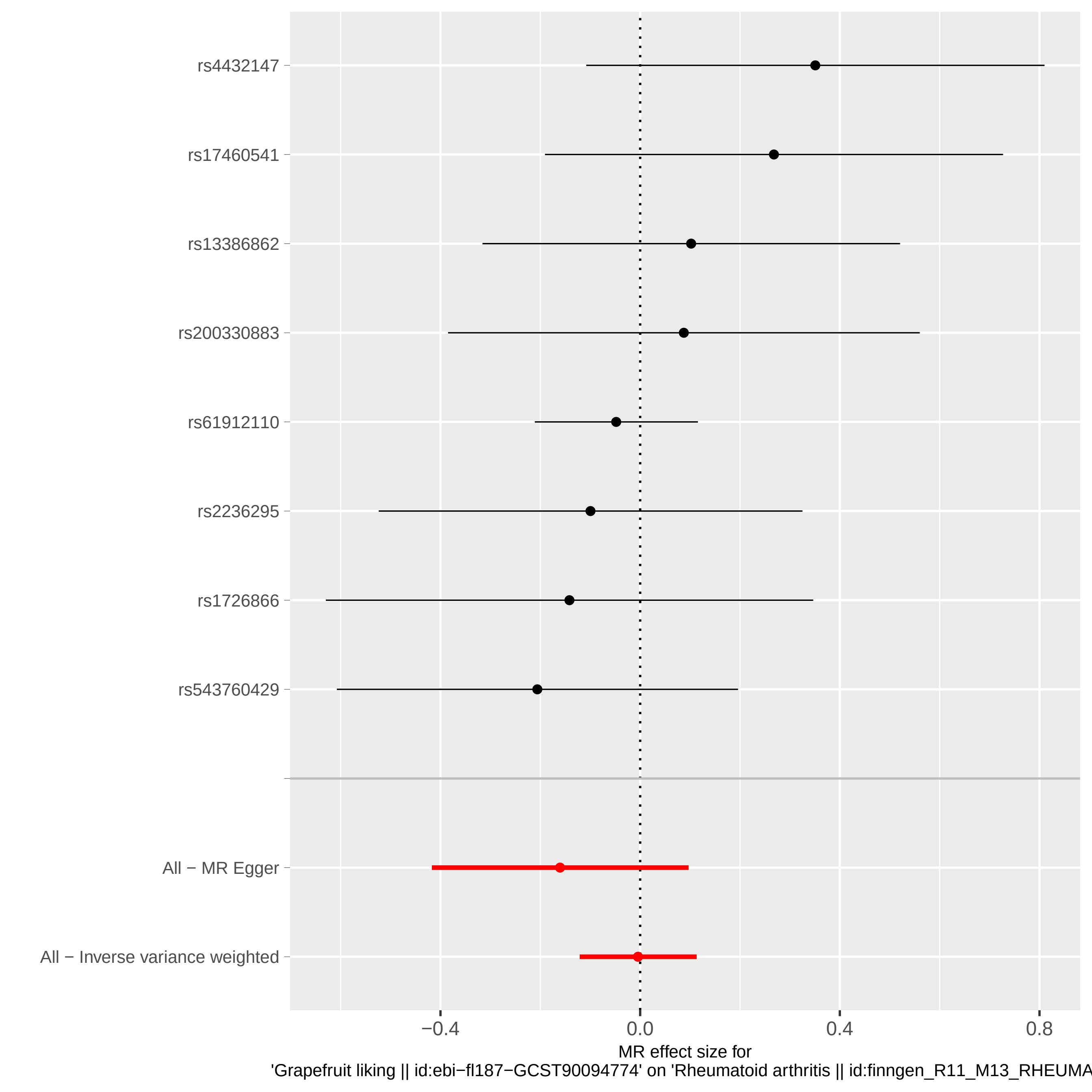

rs4432147
rs17460541
rs13386862
rs200330883
rs61912110
rs2236295
rs1726866
rs543760429
All − MR Egger
All − Inverse variance weighted
−0.4
0.0
0.4
0.8
MR effect size for
'Grapefruit liking || id:ebi−fl187−GCST90094774' on 'Rheumatoid arthritis || id:finngen_R11_M13_RHEUMA

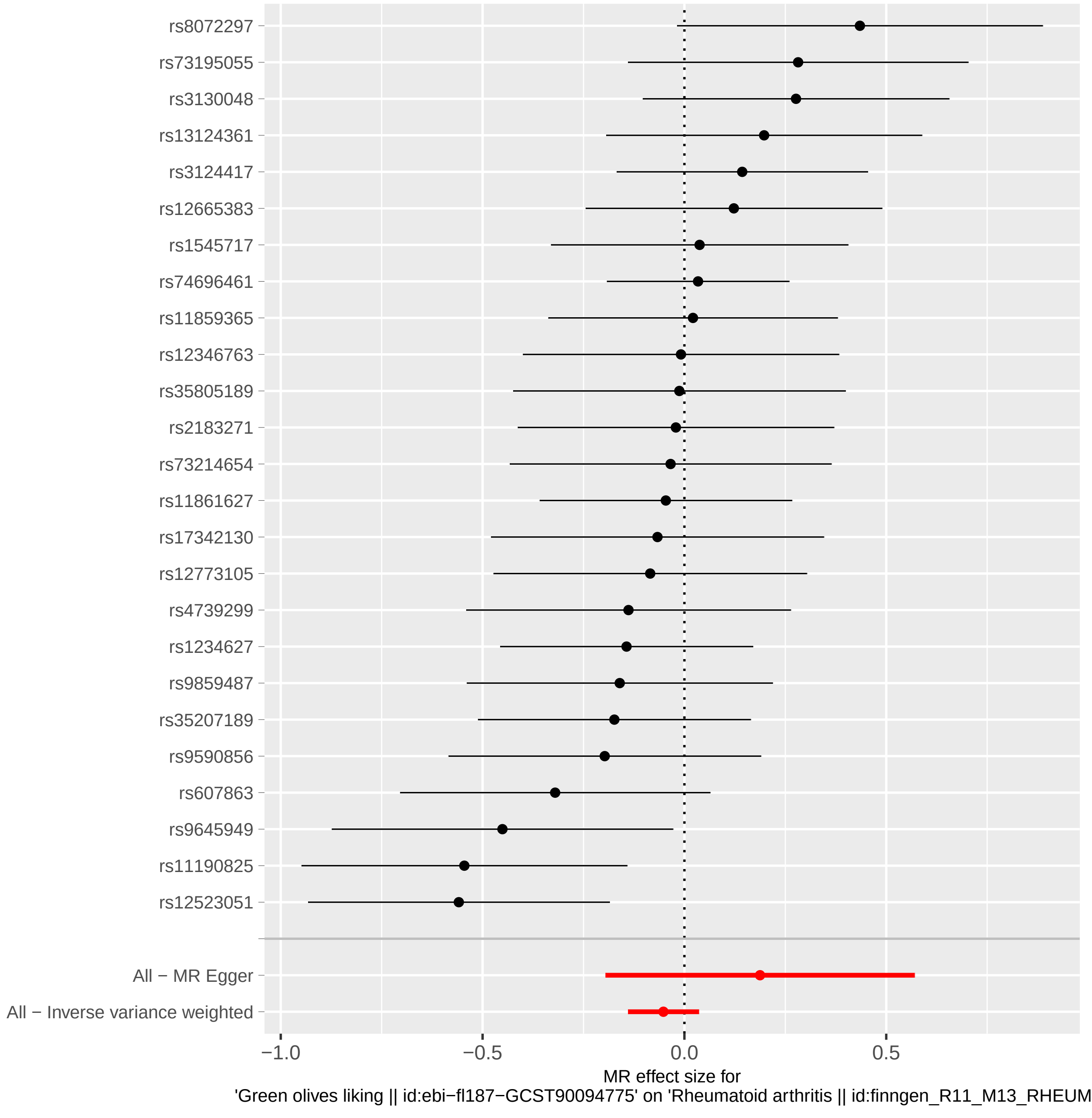
rs8072297
rs73195055
rs3130048
rs13124361
rs3124417
rs12665383
rs1545717
rs74696461
rs11859365
rs12346763
rs35805189
rs2183271
rs73214654
rs11861627
rs17342130
rs12773105
rs4739299
rs1234627
rs9859487
rs35207189
rs9590856
rs607863
rs9645949
rs11190825
rs12523051
All − MR Egger
All − Inverse variance weighted
−1.0
−0.5
0.0
0.5
MR effect size for
'Green olives liking || id:ebi−fl187−GCST90094775' on 'Rheumatoid arthritis || id:finngen_R11_M13_RHEUM

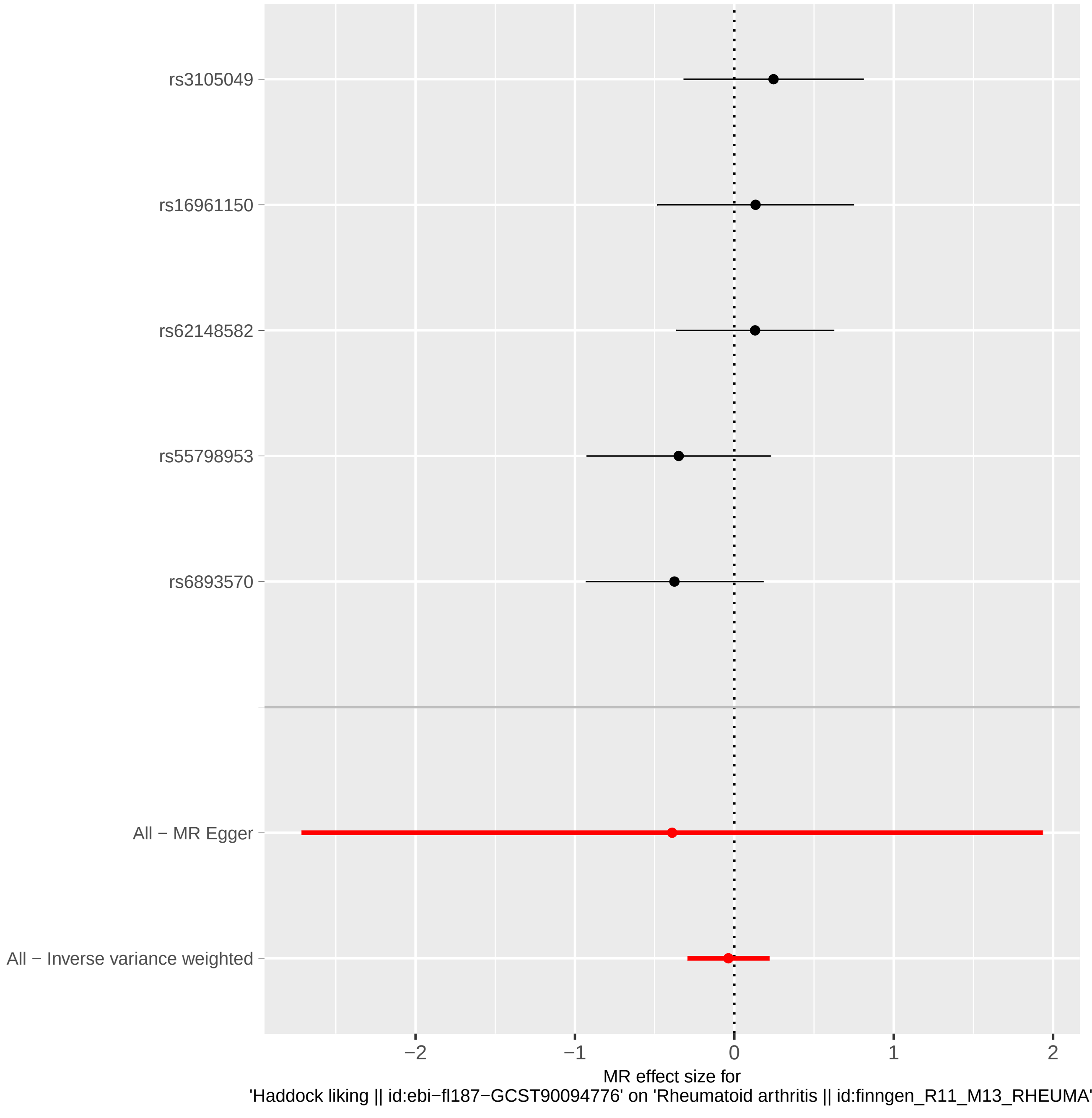
rs3105049
rs16961150
rs62148582
rs55798953
rs6893570
All − MR Egger
All − Inverse variance weighted
−2
−1
0
1
2
MR effect size for
'Haddock liking || id:ebi−fl187−GCST90094776' on 'Rheumatoid arthritis || id:finngen_R11_M13_RHEUMA'

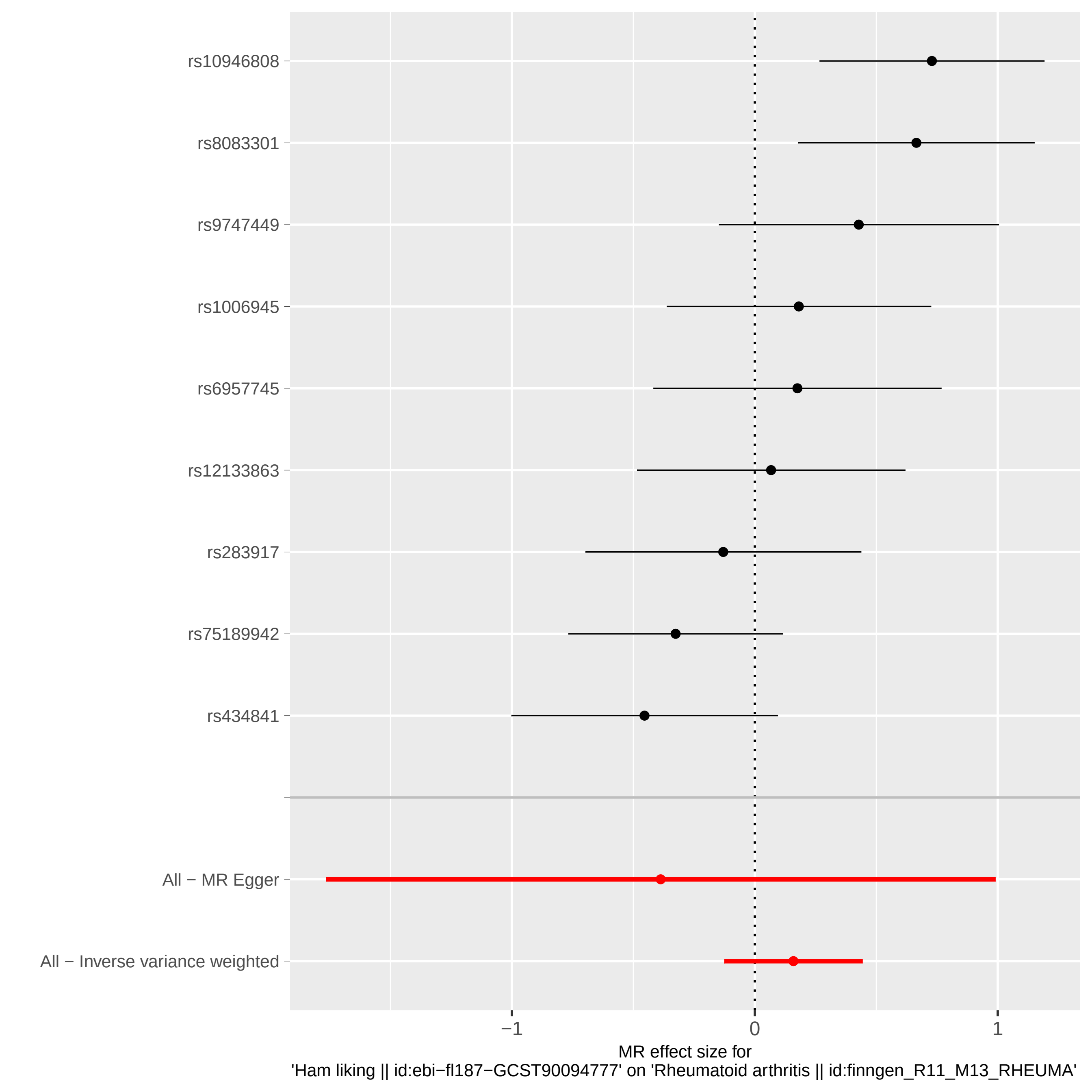
rs10946808
rs8083301
rs9747449
rs1006945
rs6957745
rs12133863
rs283917
rs75189942
rs434841
All − MR Egger
All − Inverse variance weighted
−1
0
1
MR effect size for
'Ham liking || id:ebi−fl187−GCST90094777' on 'Rheumatoid arthritis || id:finngen_R11_M13_RHEUMA'

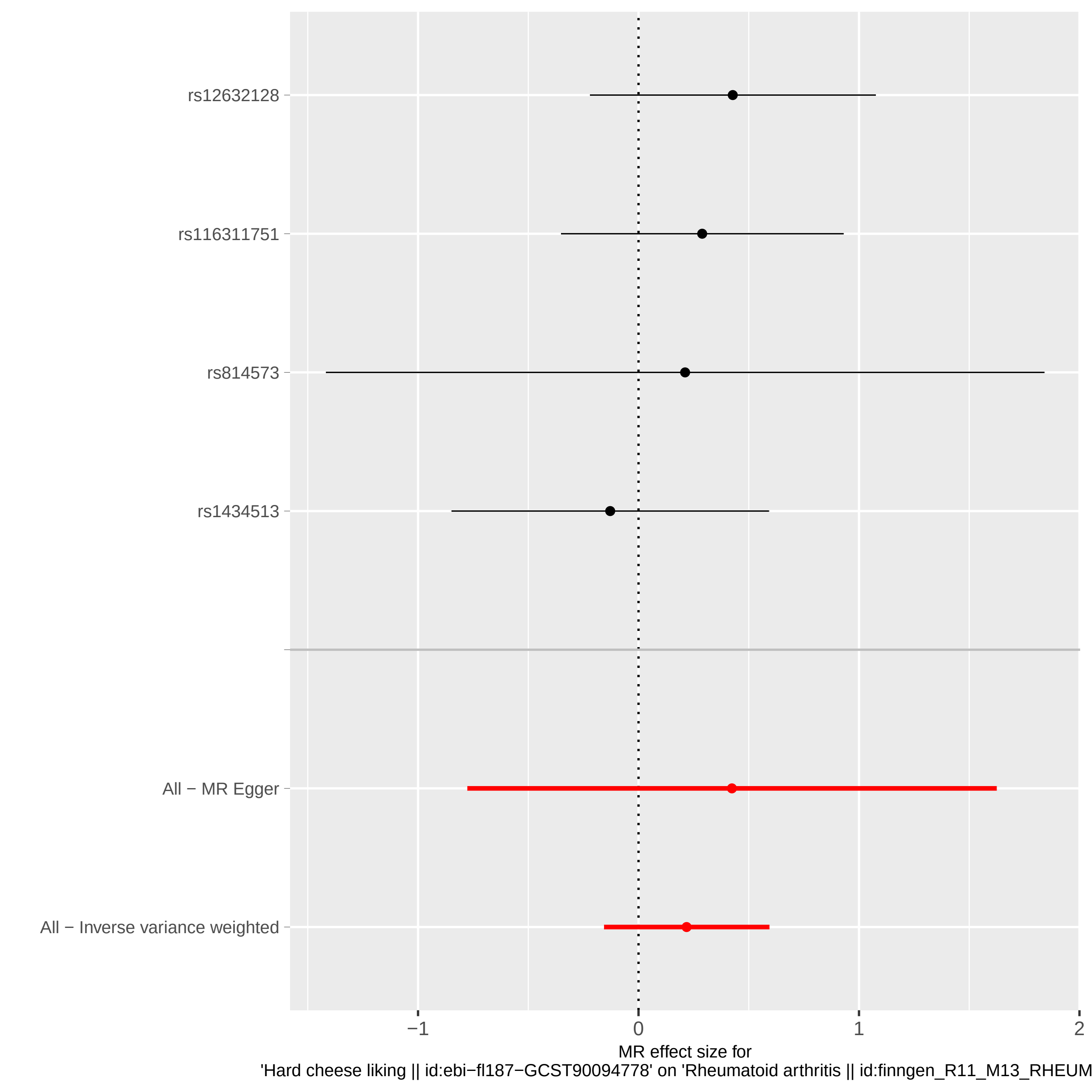

rs12632128
rs116311751
rs814573
rs1434513
All − MR Egger
All − Inverse variance weighted
−1
0
1
2
MR effect size for
'Hard cheese liking || id:ebi−fl187−GCST90094778' on 'Rheumatoid arthritis || id:finngen_R11_M13_RHEUM

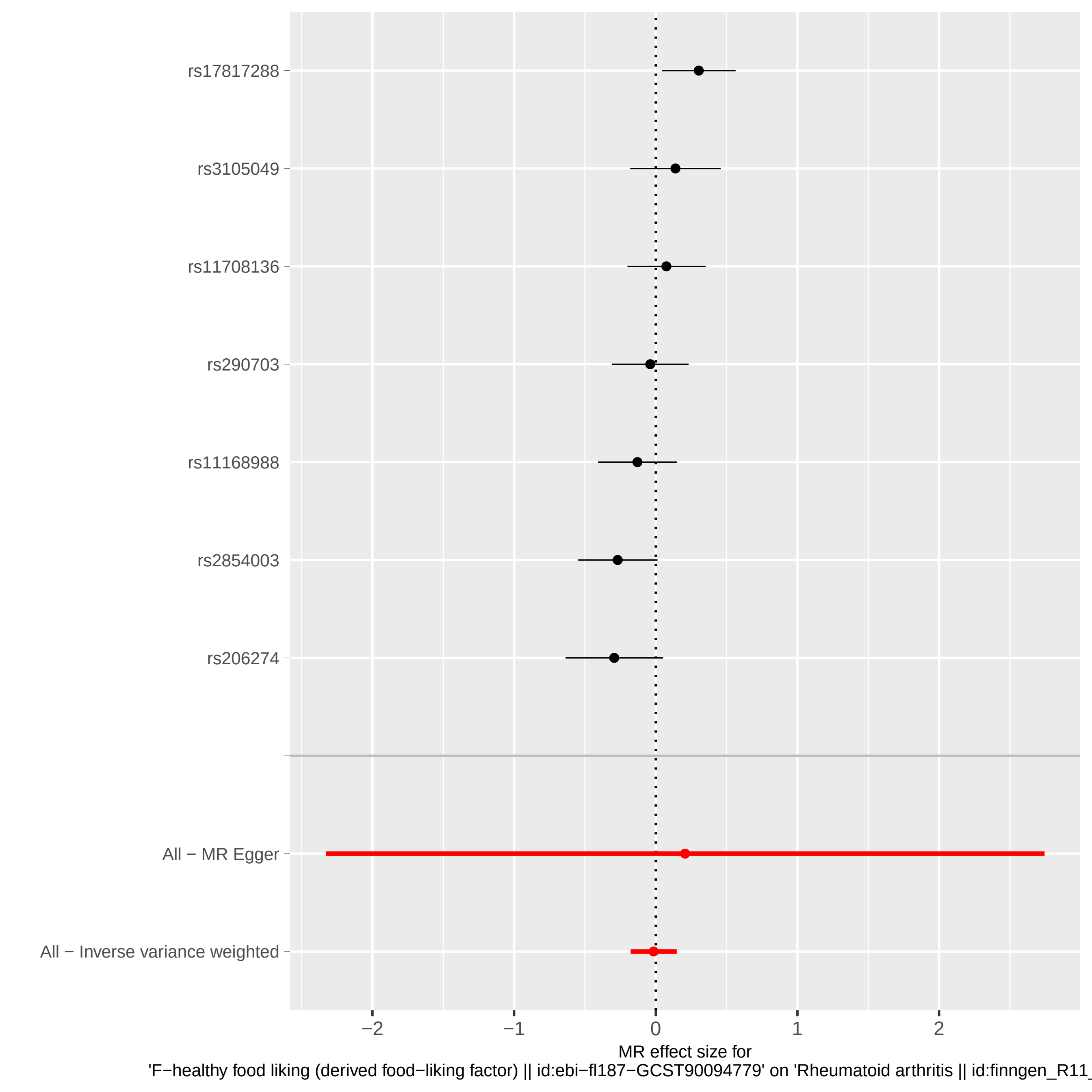
rs17817288
rs3105049
rs11708136
rs290703
rs11168988
rs2854003
rs206274
All − MR Egger
All − Inverse variance weighted
−2
−1
0
1
2
MR effect size for
'F−healthy food liking (derived food−liking factor) || id:ebi−fl187−GCST90094779' on 'Rheumatoid arthritis || id:finngen_R11_

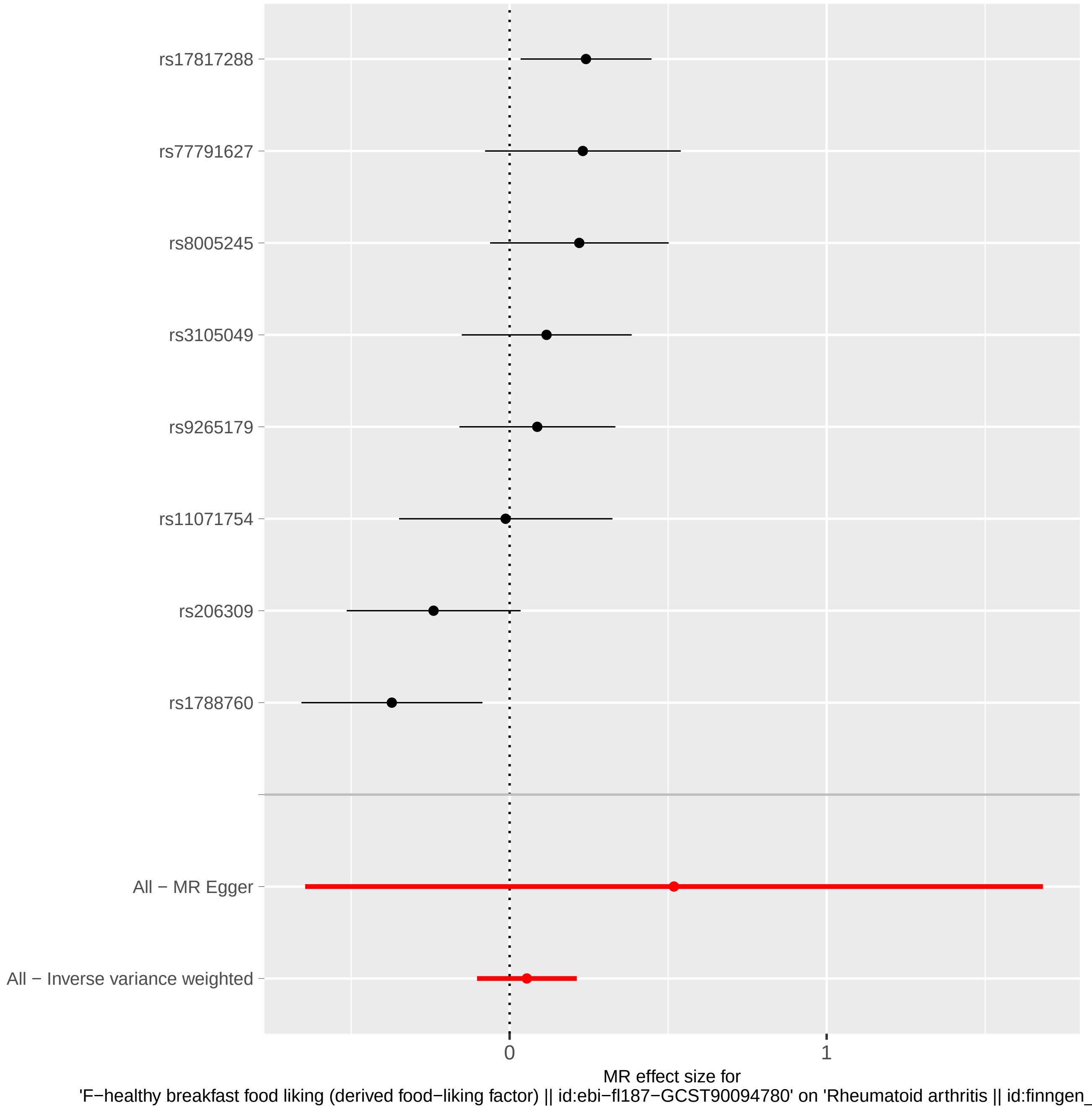
rs17817288
rs77791627
rs8005245
rs3105049
rs9265179
rs11071754
rs206309
rs1788760
All − MR Egger
All − Inverse variance weighted
0
1
MR effect size for
'F−healthy breakfast food liking (derived food−liking factor) || id:ebi−fl187−GCST90094780' on 'Rheumatoid arthritis || id:finngen_

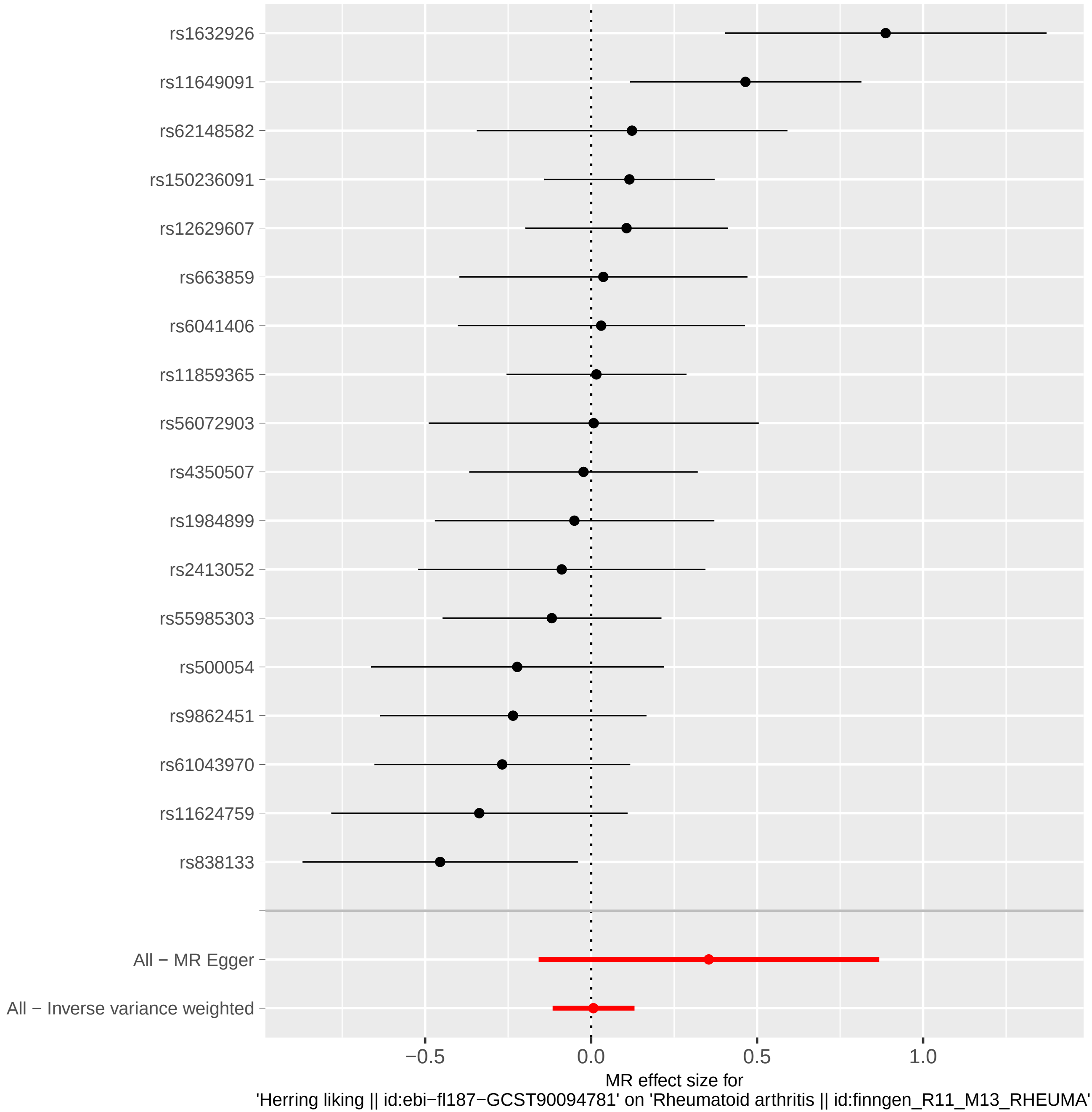
rs1632926
rs11649091
rs62148582
rs150236091
rs12629607
rs663859
rs6041406
rs11859365
rs56072903
rs4350507
rs1984899
rs2413052
rs55985303
rs500054
rs9862451
rs61043970
rs11624759
rs838133
All − MR Egger
All − Inverse variance weighted
−0.5
0.0
0.5
1.0
MR effect size for
'Herring liking || id:ebi−fl187−GCST90094781' on 'Rheumatoid arthritis || id:finngen_R11_M13_RHEUMA'

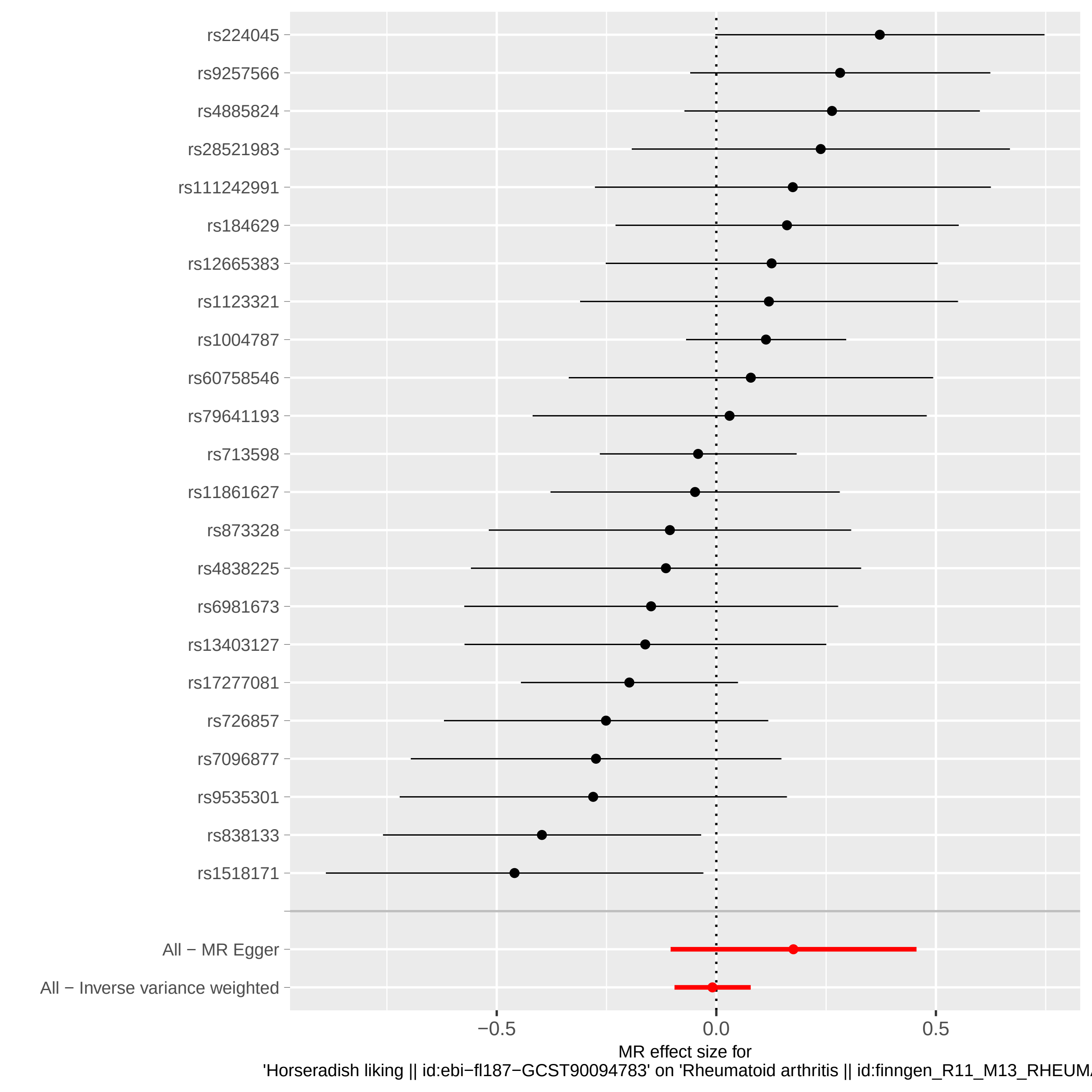
rs224045
rs9257566
rs4885824
rs28521983
rs111242991
rs184629
rs12665383
rs1123321
rs1004787
rs60758546
rs79641193
rs713598
rs11861627
rs873328
rs4838225
rs6981673
rs13403127
rs17277081
rs726857
rs7096877
rs9535301
rs838133
rs1518171
All − MR Egger
All − Inverse variance weighted
−0.5
0.0
0.5
MR effect size for
'Horseradish liking || id:ebi−fl187−GCST90094783' on 'Rheumatoid arthritis || id:finngen_R11_M13_RHEUM

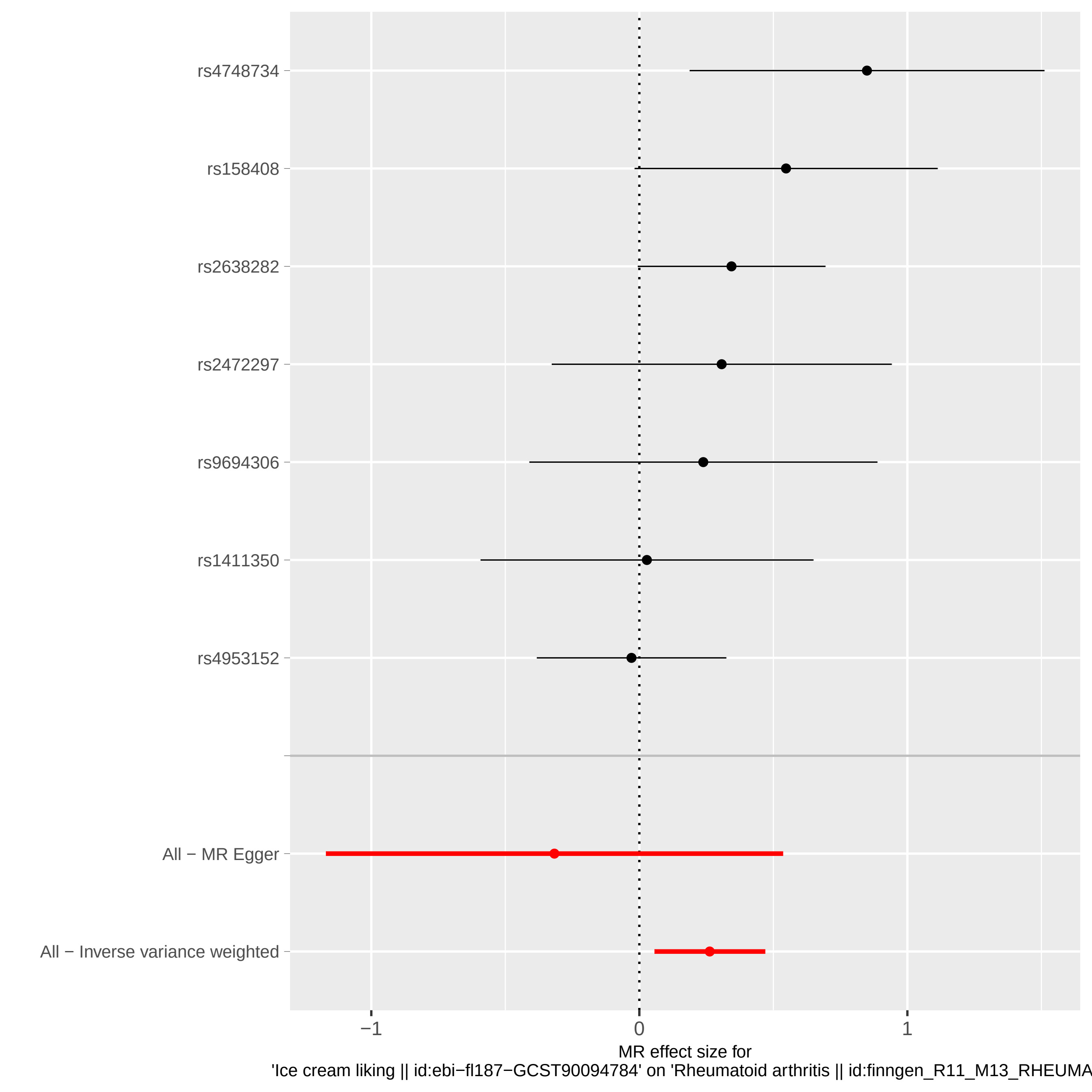

rs4748734
rs158408
rs2638282
rs2472297
rs9694306
rs1411350
rs4953152
All − MR Egger
All − Inverse variance weighted
−1
0
1
MR effect size for
'Ice cream liking || id:ebi−fl187−GCST90094784' on 'Rheumatoid arthritis || id:finngen_R11_M13_RHEUMA

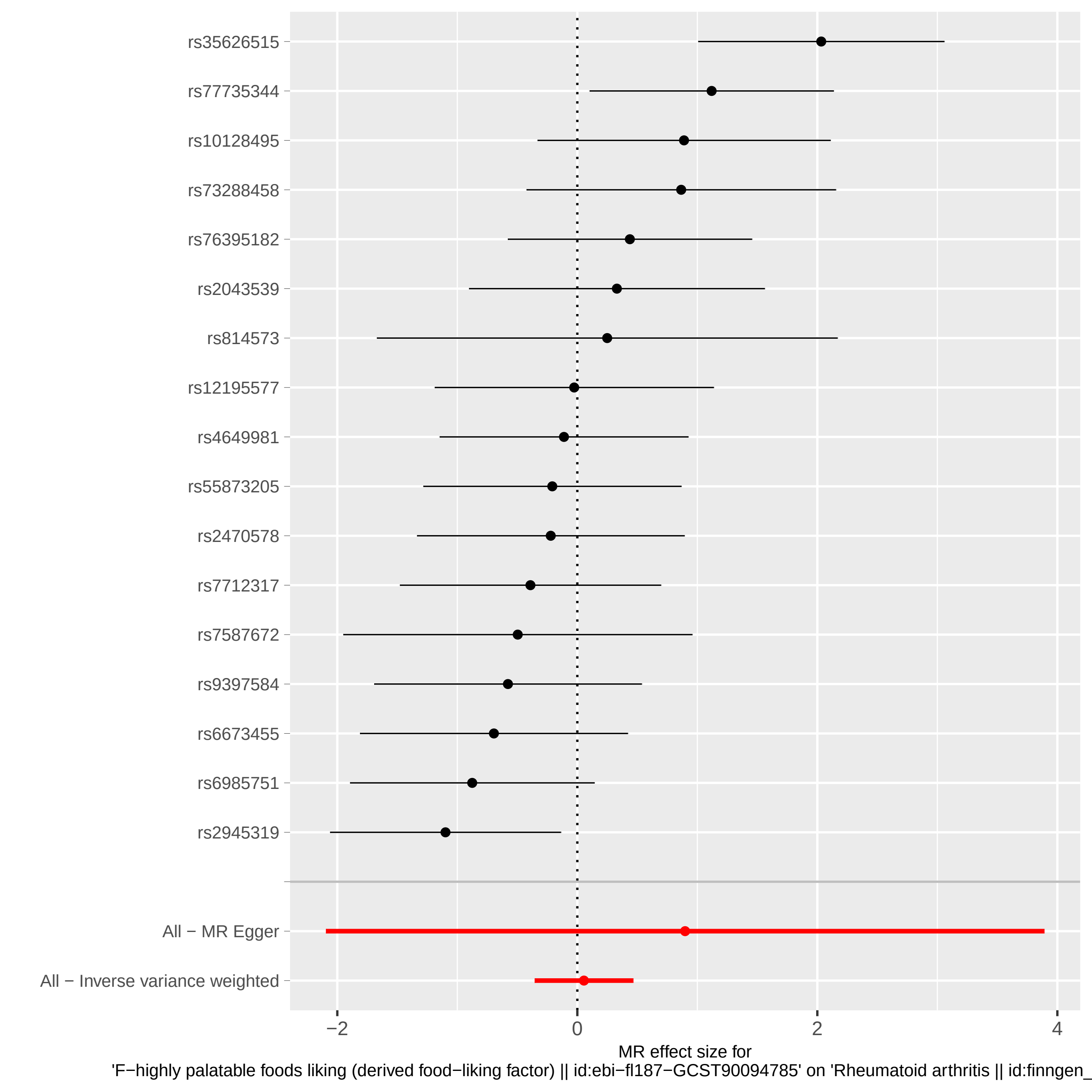
rs35626515
rs77735344
rs10128495
rs73288458
rs76395182
rs2043539
rs814573
rs12195577
rs4649981
rs55873205
rs2470578
rs7712317
rs7587672
rs9397584
rs6673455
rs6985751
rs2945319
All − MR Egger
All − Inverse variance weighted
−2
0
2
4
MR effect size for
'F−highly palatable foods liking (derived food−liking factor) || id:ebi−fl187−GCST90094785' on 'Rheumatoid arthritis || id:finngen_

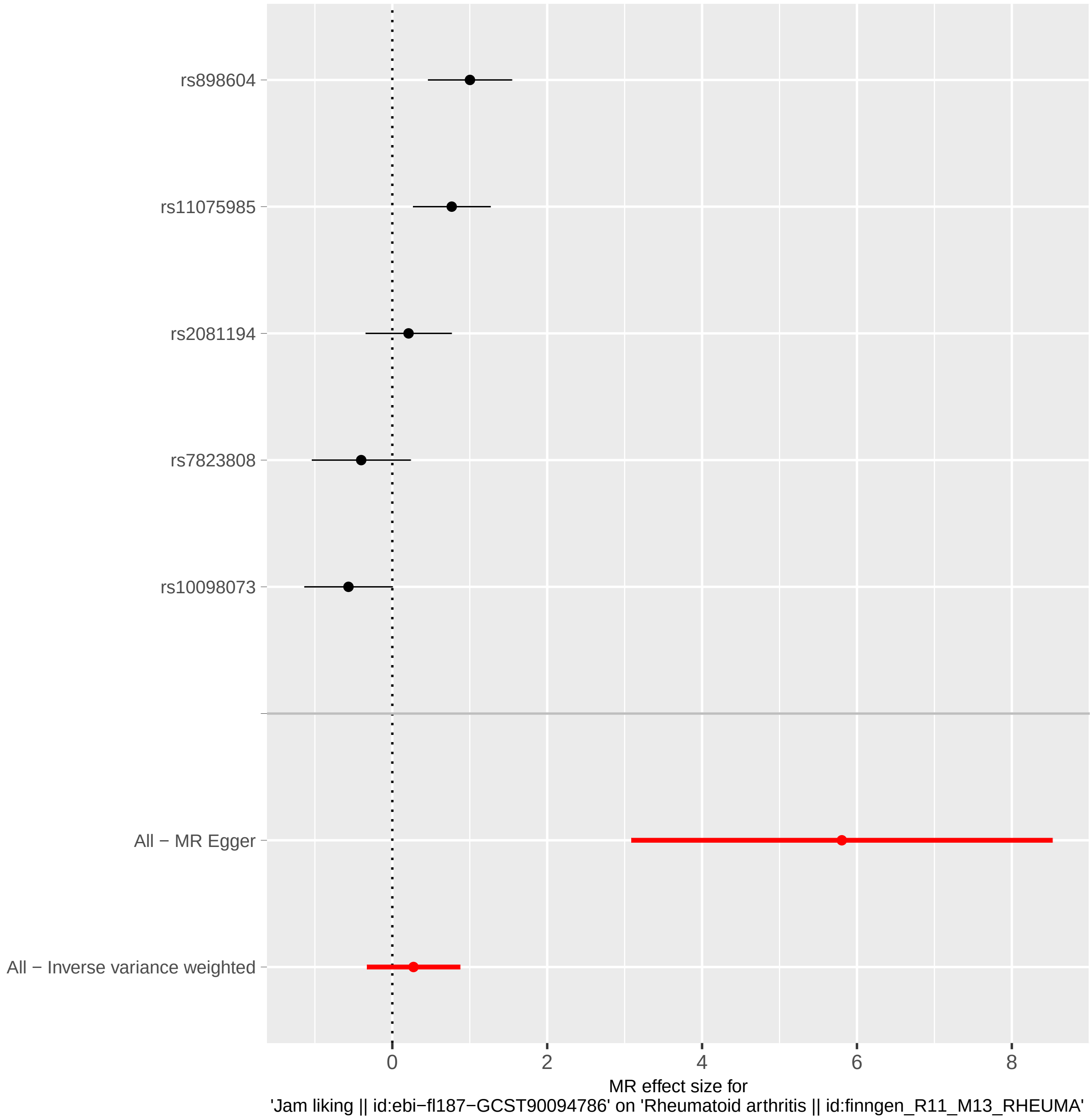
rs898604
rs11075985
rs2081194
rs7823808
rs10098073
All − MR Egger
All − Inverse variance weighted
0
2
4
6
8
MR effect size for
'Jam liking || id:ebi−fl187−GCST90094786' on 'Rheumatoid arthritis || id:finngen_R11_M13_RHEUMA'

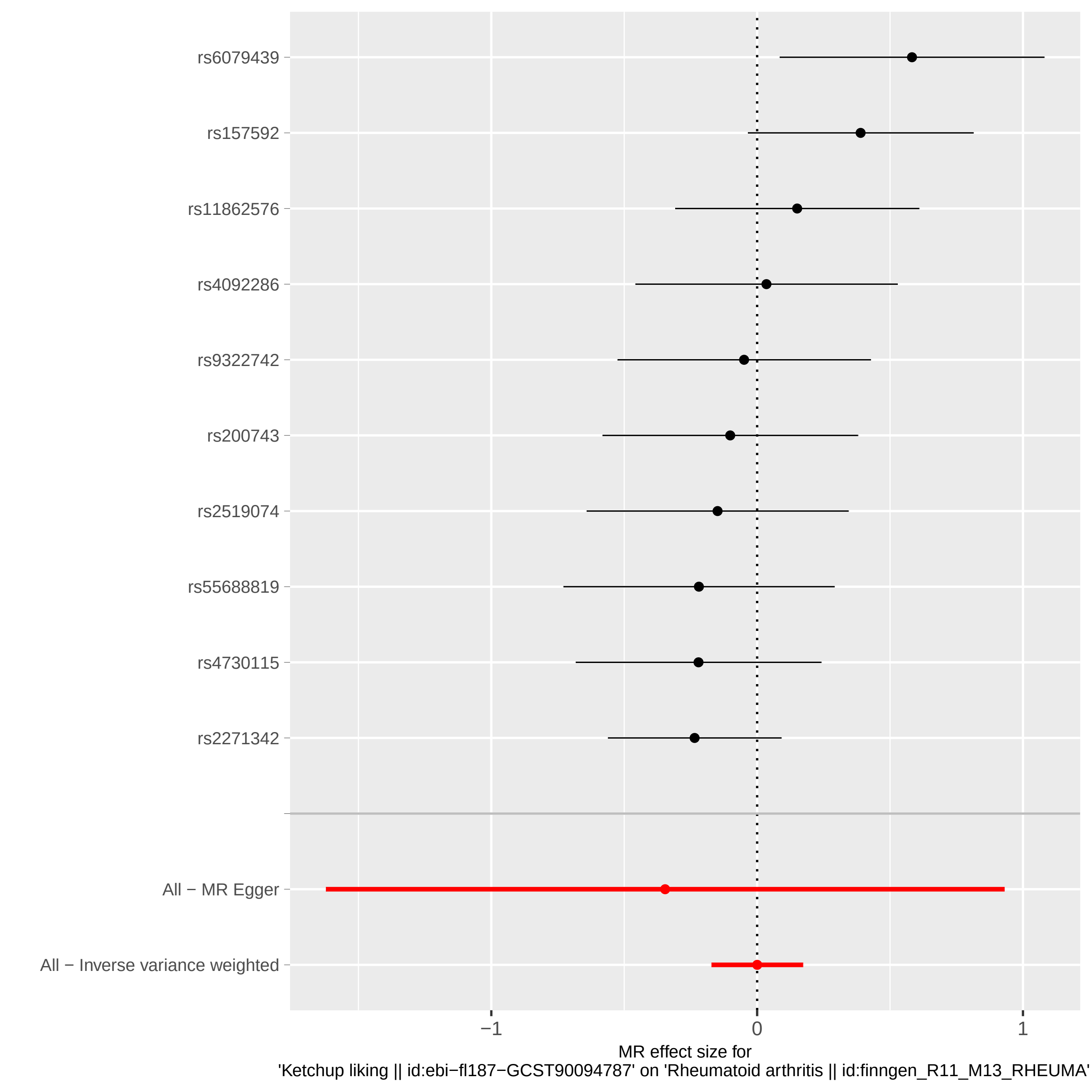
rs6079439
rs157592
rs11862576
rs4092286
rs9322742
rs200743
rs2519074
rs55688819
rs4730115
rs2271342
All − MR Egger
All − Inverse variance weighted
−1
0
1
MR effect size for
'Ketchup liking || id:ebi−fl187−GCST90094787' on 'Rheumatoid arthritis || id:finngen_R11_M13_RHEUMA'

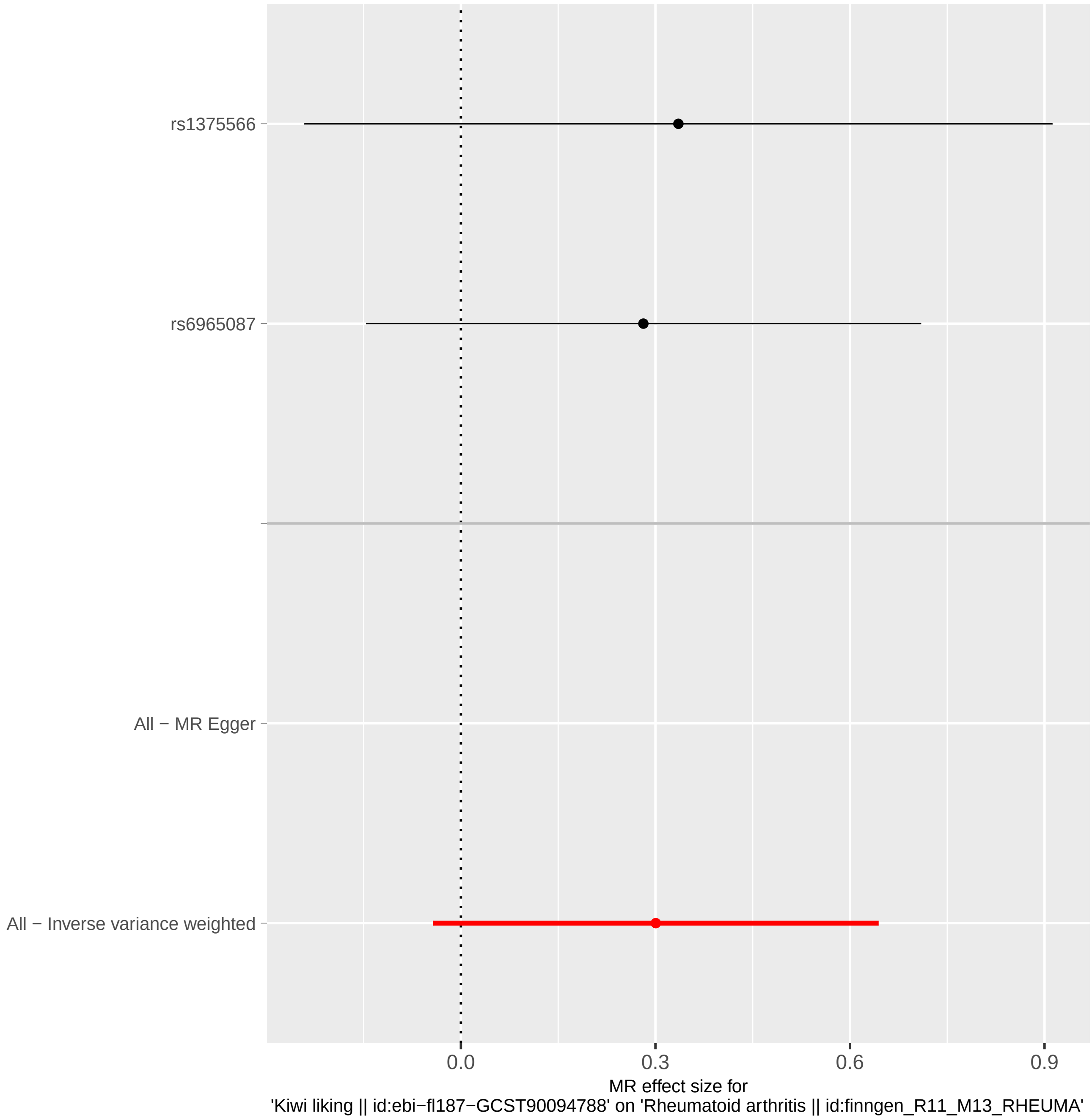
rs1375566
rs6965087
All − MR Egger
All − Inverse variance weighted
0.0
0.3
0.6
0.9
MR effect size for
'Kiwi liking || id:ebi−fl187−GCST90094788' on 'Rheumatoid arthritis || id:finngen_R11_M13_RHEUMA'

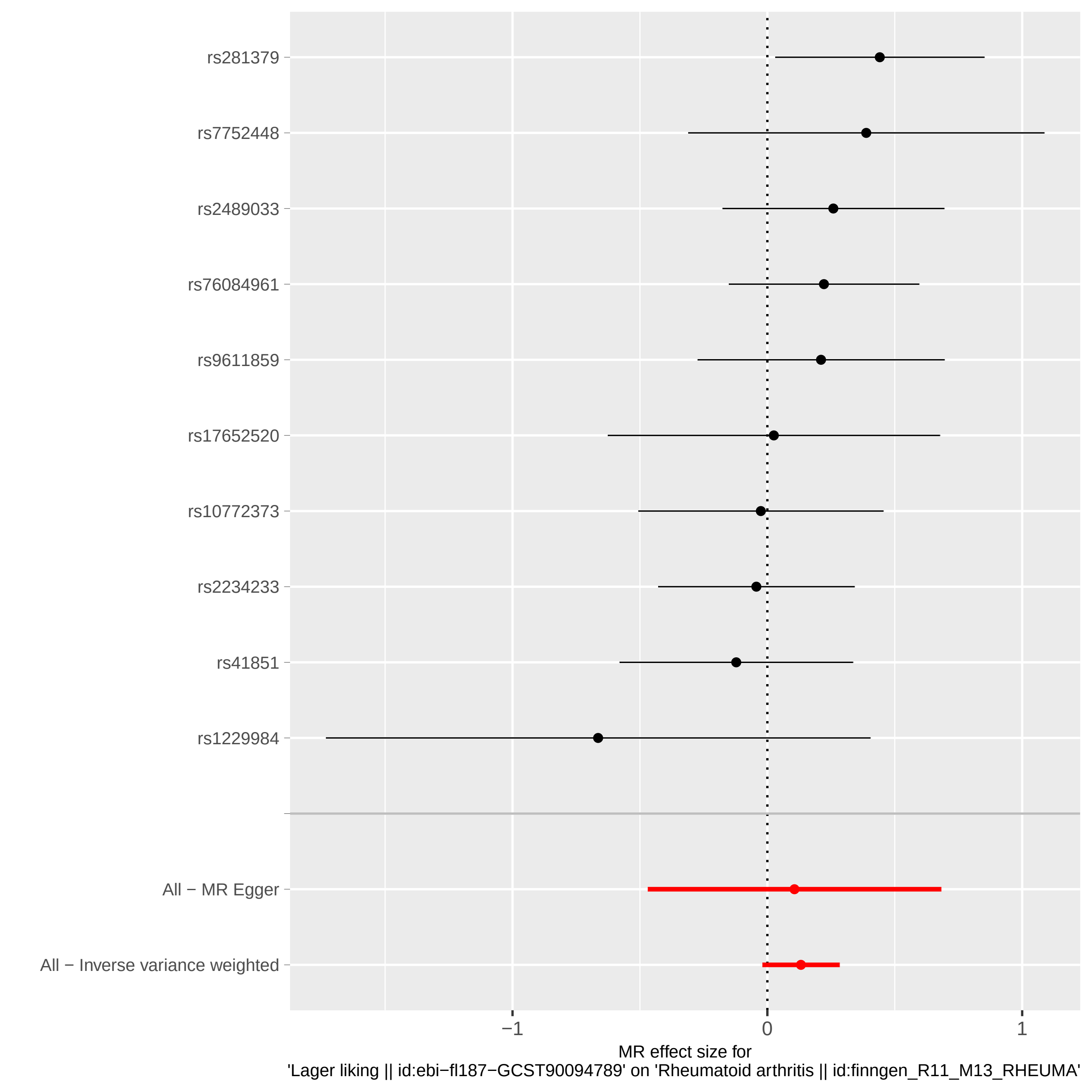
rs281379
rs7752448
rs2489033
rs76084961
rs9611859
rs17652520
rs10772373
rs2234233
rs41851
rs1229984
All − MR Egger
All − Inverse variance weighted
−1
0
1
MR effect size for
'Lager liking || id:ebi−fl187−GCST90094789' on 'Rheumatoid arthritis || id:finngen_R11_M13_RHEUMA'

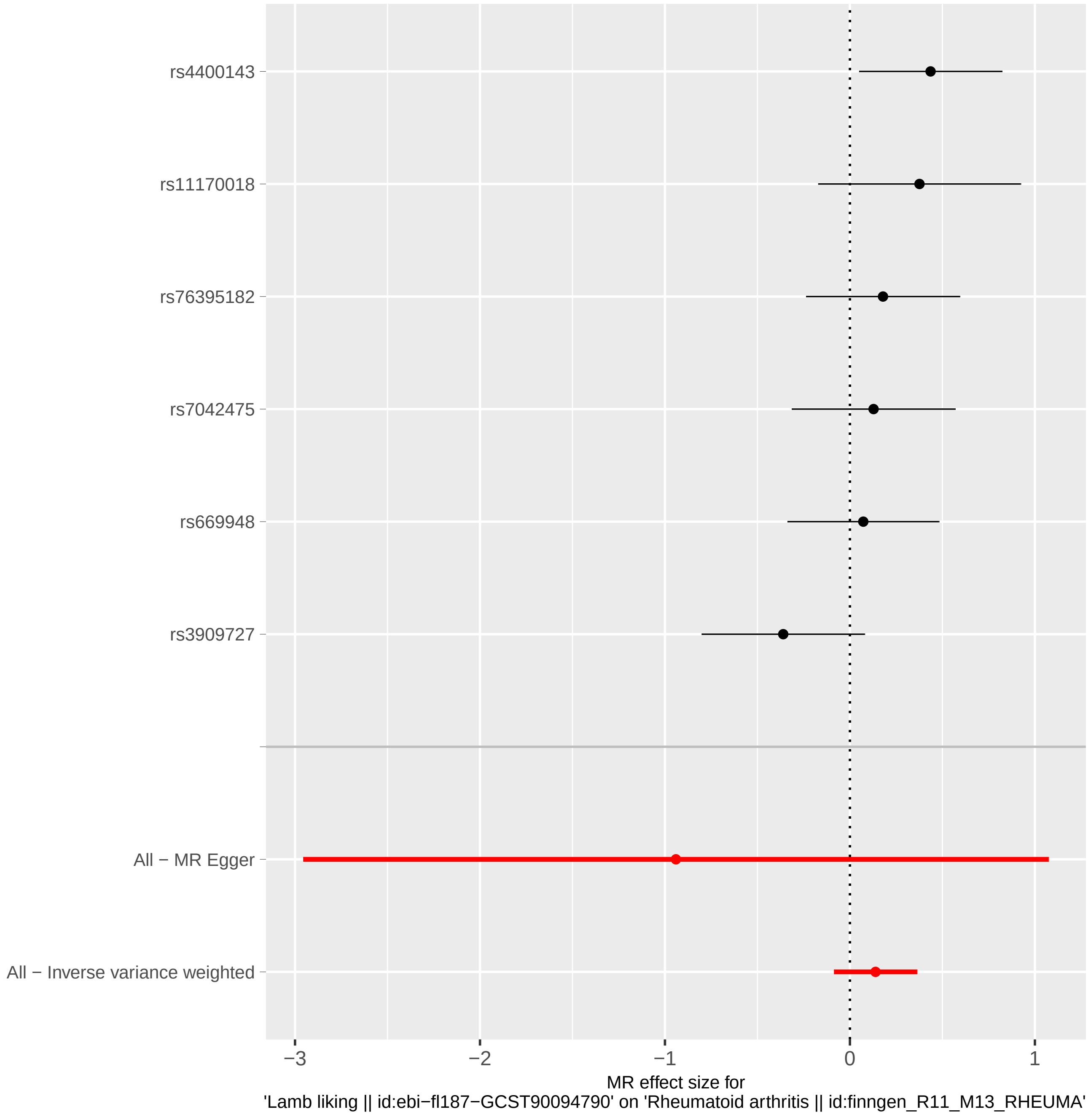
rs4400143
rs11170018
rs76395182
rs7042475
rs669948
rs3909727
All − MR Egger
All − Inverse variance weighted
−3
−2
−1
0
1
MR effect size for
'Lamb liking || id:ebi−fl187−GCST90094790' on 'Rheumatoid arthritis || id:finngen_R11_M13_RHEUMA'

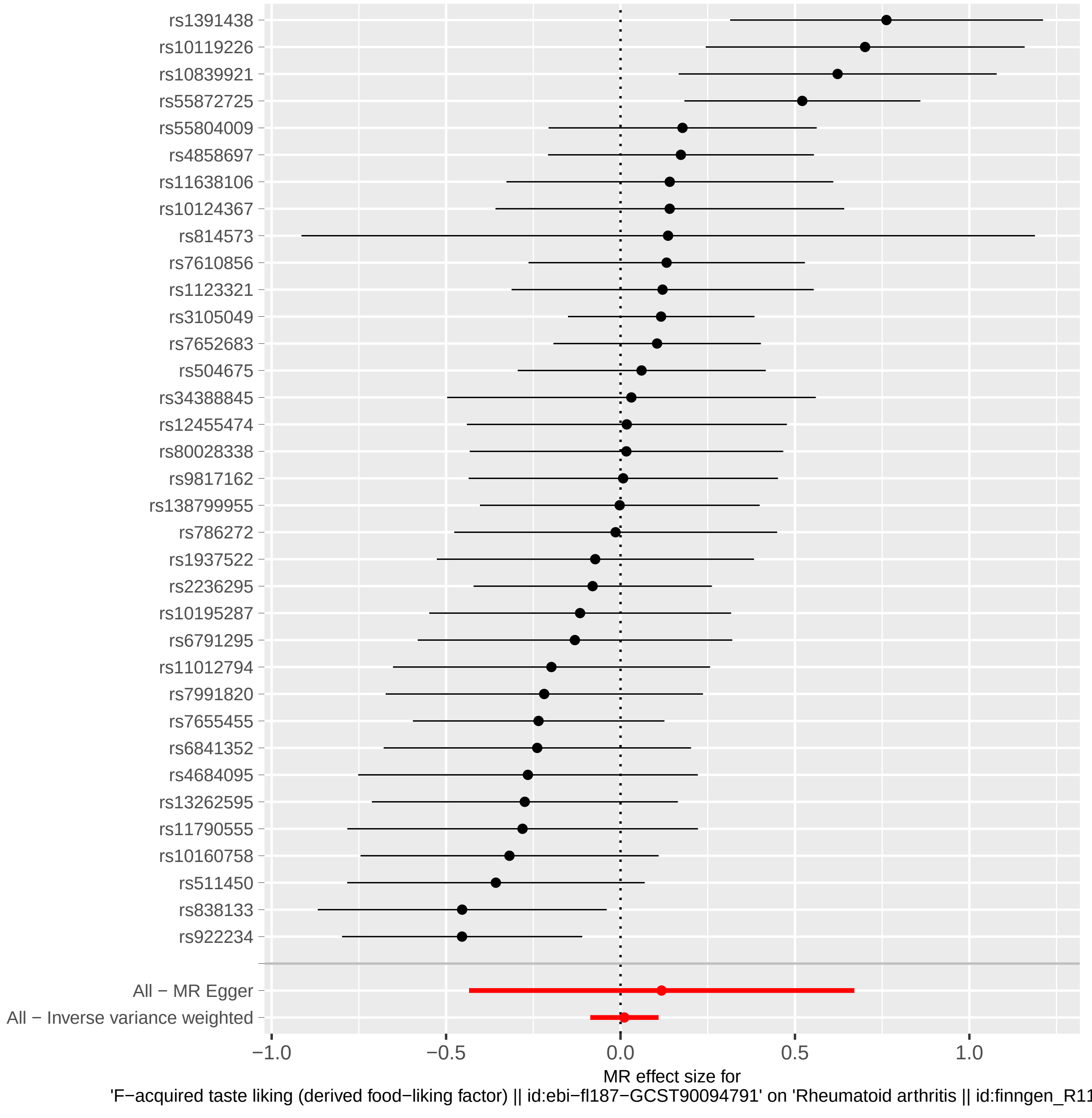
rs1391438
rs10119226
rs10839921
rs55872725
rs55804009
rs4858697
rs11638106
rs10124367
rs814573
rs7610856
rs1123321
rs3105049
rs7652683
rs504675
rs34388845
rs12455474
rs80028338
rs9817162
rs138799955
rs786272
rs1937522
rs2236295
rs10195287
rs6791295
rs11012794
rs7991820
rs7655455
rs6841352
rs4684095
rs13262595
rs11790555
rs10160758
rs511450
rs838133
rs922234
All − MR Egger
All − Inverse variance weighted
−1.0
−0.5
0.0
0.5
1.0
MR effect size for
'F−acquired taste liking (derived food−liking factor) || id:ebi−fl187−GCST90094791' on 'Rheumatoid arthritis || id:finngen_R11

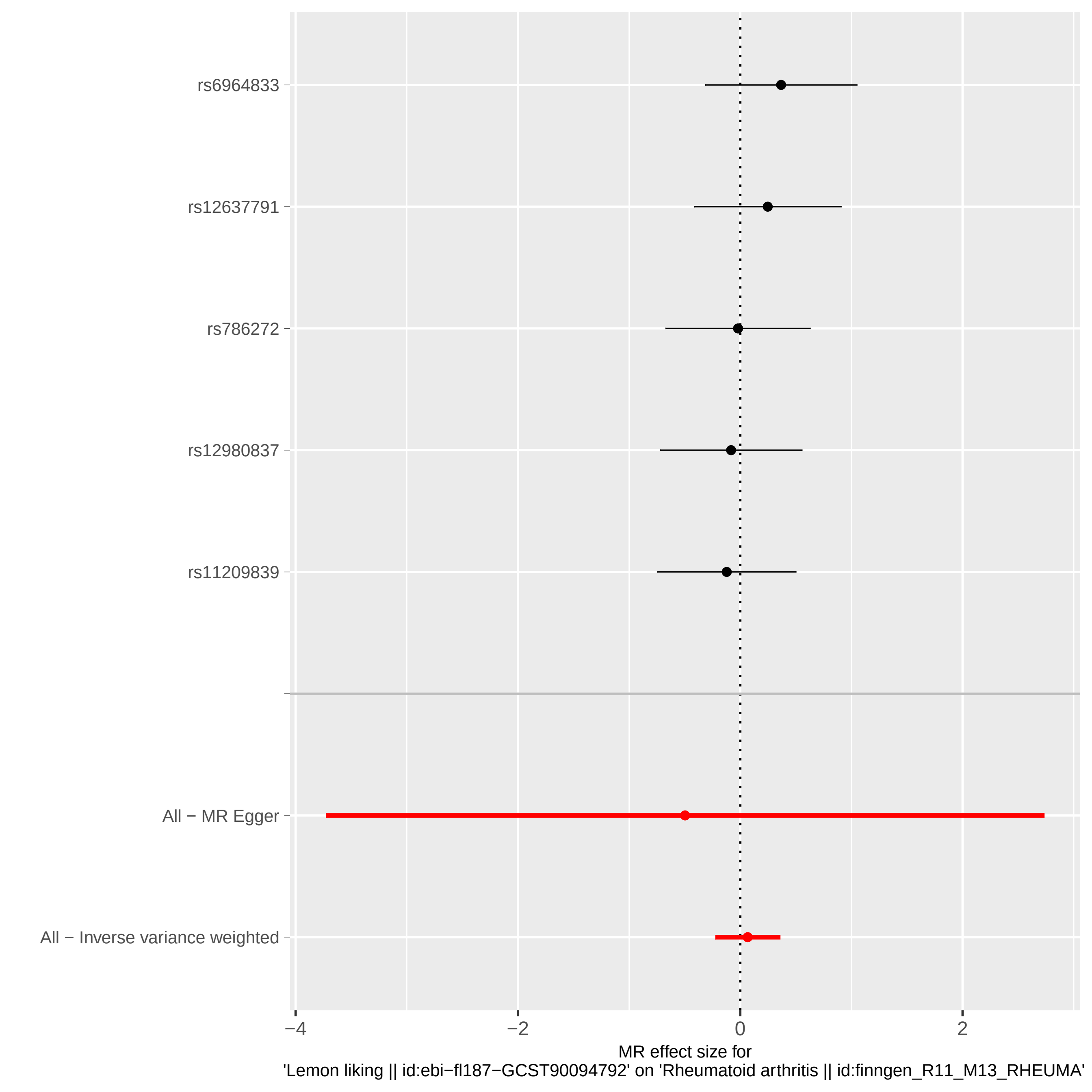
rs6964833
rs12637791
rs786272
rs12980837
rs11209839
All − MR Egger
All − Inverse variance weighted
−4
−2
0
2
MR effect size for
'Lemon liking || id:ebi−fl187−GCST90094792' on 'Rheumatoid arthritis || id:finngen_R11_M13_RHEUMA'

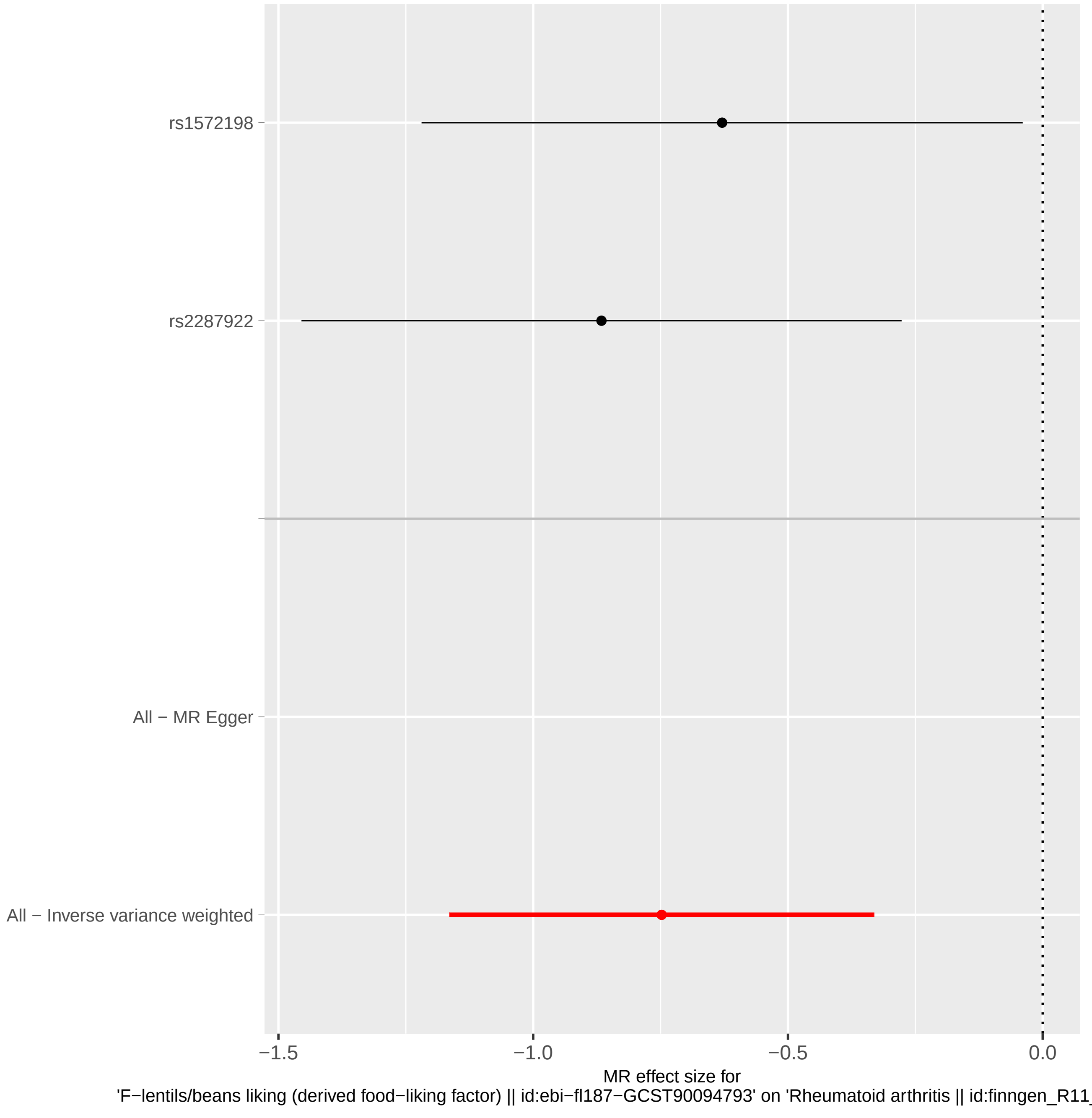

rs1572198
rs2287922
All − MR Egger
All − Inverse variance weighted
−1.5
−1.0
−0.5
0.0
MR effect size for
'F−lentils/beans liking (derived food−liking factor) || id:ebi−fl187−GCST90094793' on 'Rheumatoid arthritis || id:finngen_R11_

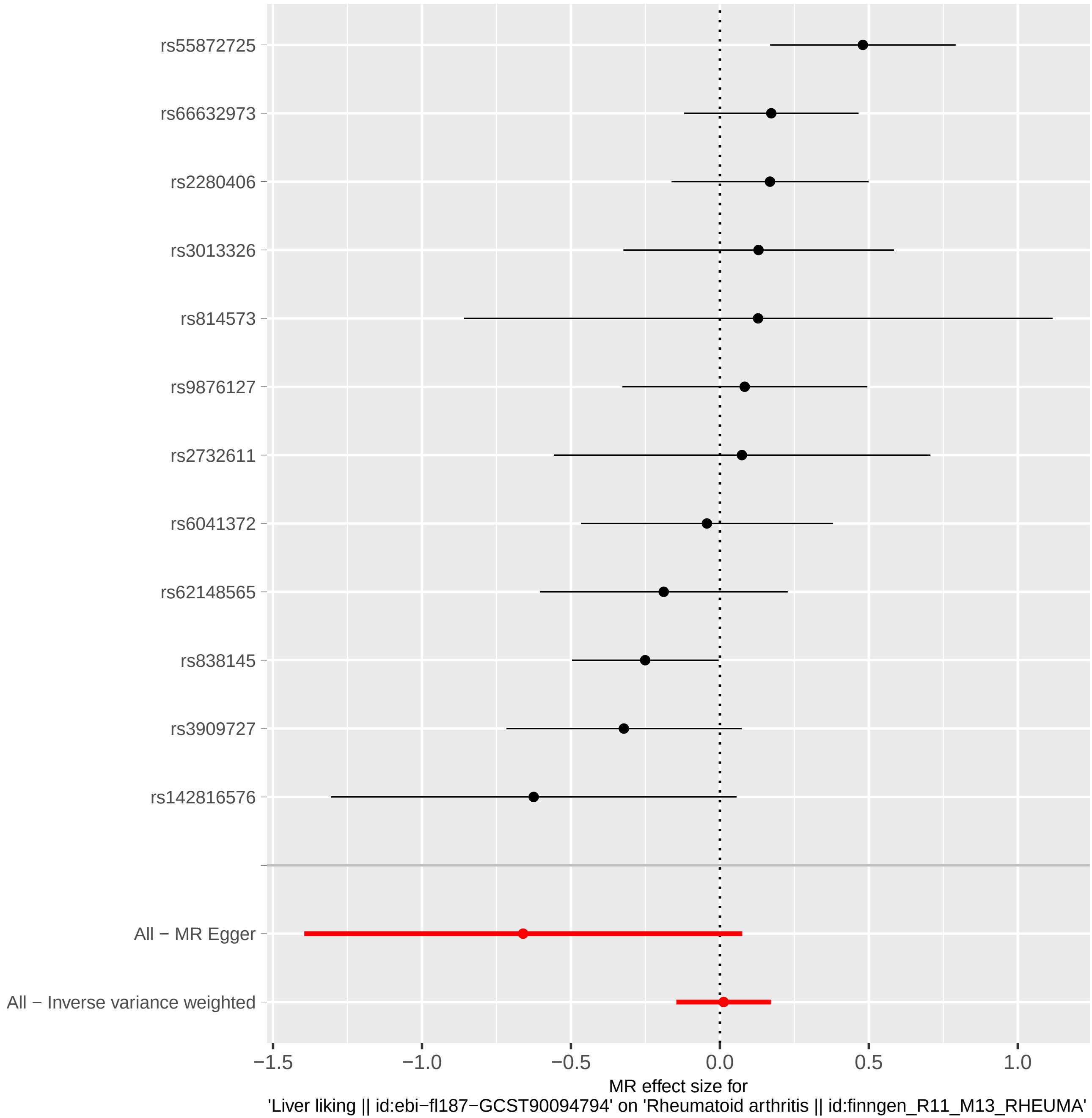

rs55872725
rs66632973
rs2280406
rs3013326
rs814573
rs9876127
rs2732611
rs6041372
rs62148565
rs838145
rs3909727
rs142816576
All − MR Egger
All − Inverse variance weighted
−1.5
−1.0
−0.5
0.0
0.5
1.0
MR effect size for
'Liver liking || id:ebi−fl187−GCST90094794' on 'Rheumatoid arthritis || id:finngen_R11_M13_RHEUMA'

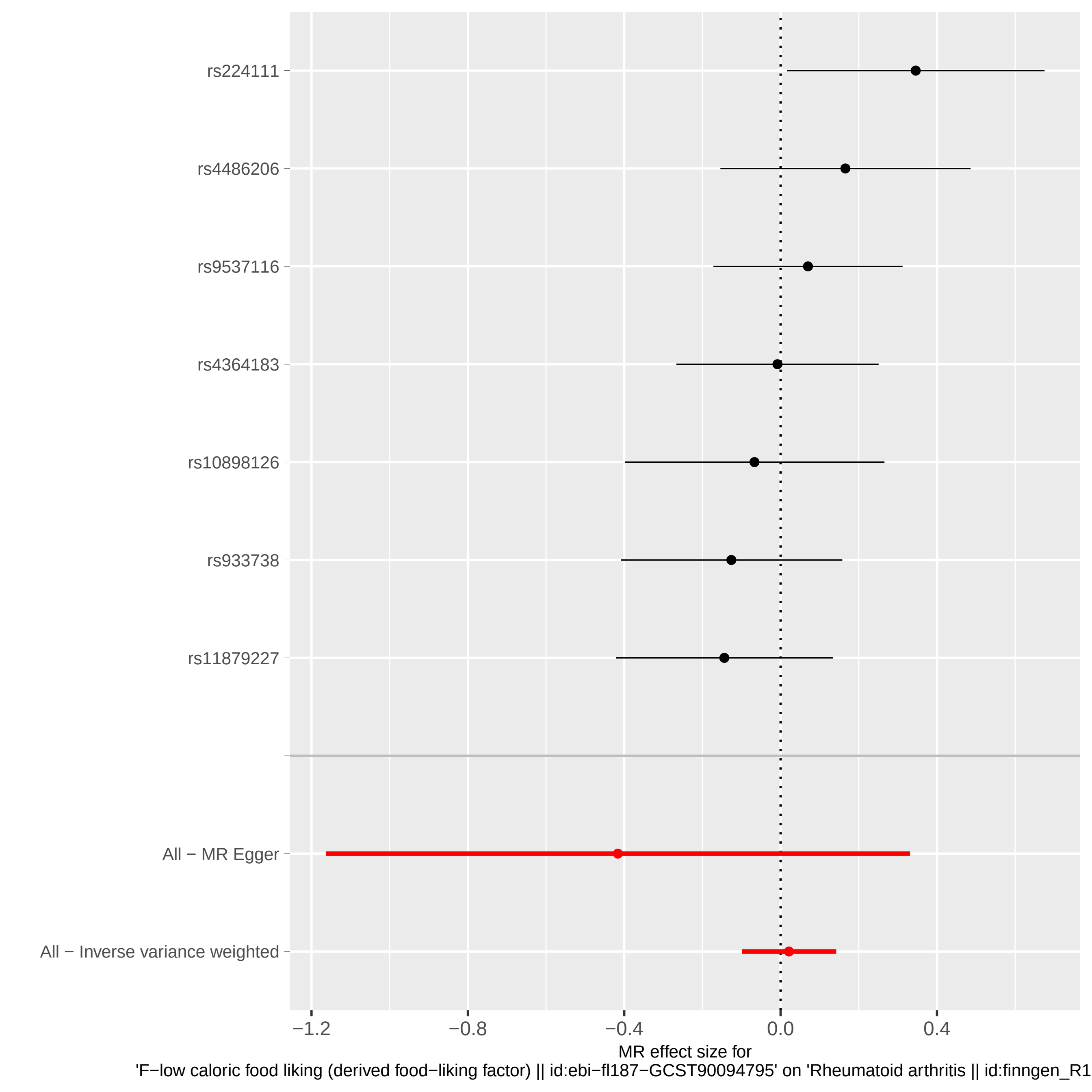
rs224111
rs4486206
rs9537116
rs4364183
rs10898126
rs933738
rs11879227
All − MR Egger
All − Inverse variance weighted
−1.2
−0.8
−0.4
0.0
0.4
MR effect size for
'F−low caloric food liking (derived food−liking factor) || id:ebi−fl187−GCST90094795' on 'Rheumatoid arthritis || id:finngen_R1

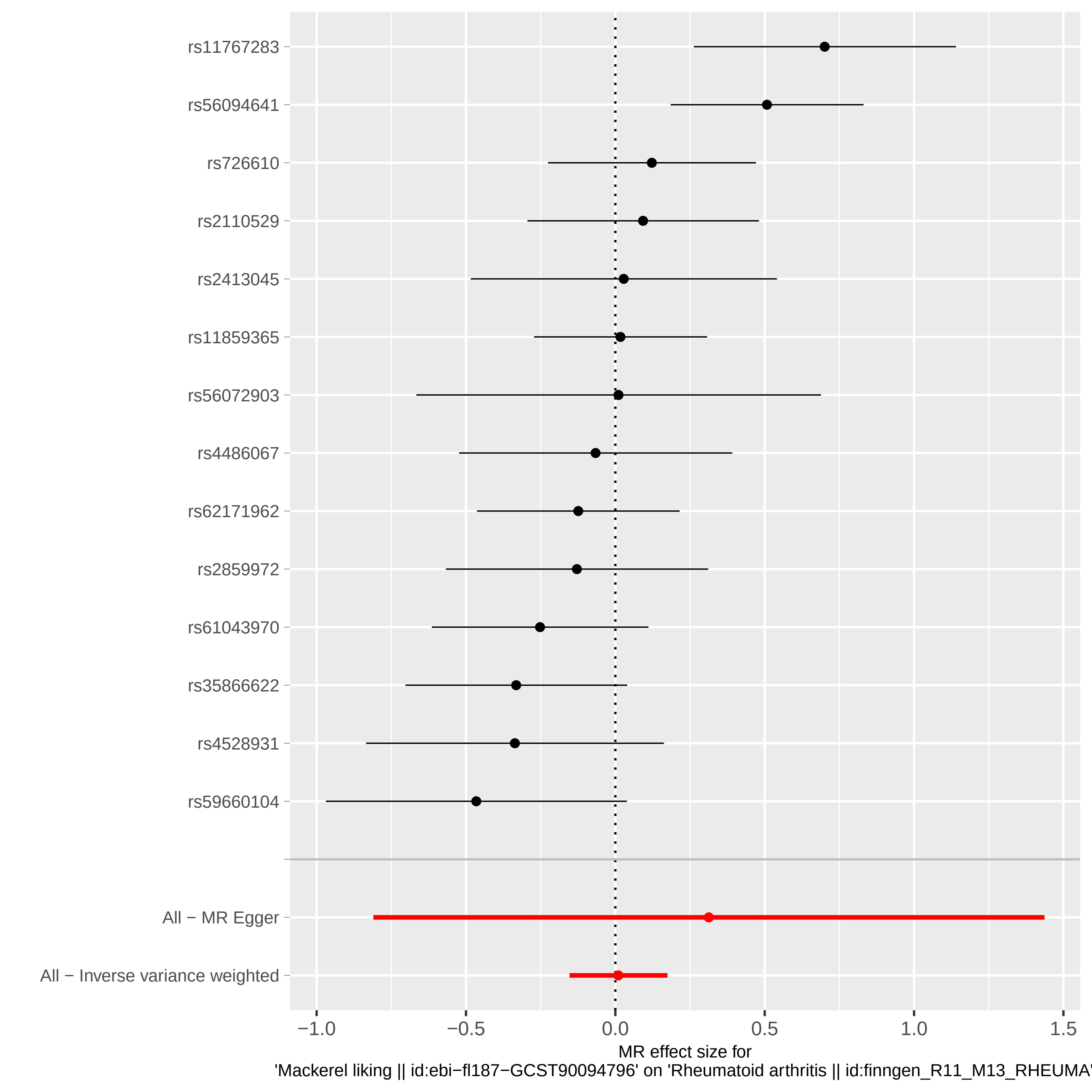
rs11767283
rs56094641
rs726610
rs2110529
rs2413045
rs11859365
rs56072903
rs4486067
rs62171962
rs2859972
rs61043970
rs35866622
rs4528931
rs59660104
All − MR Egger
All − Inverse variance weighted
−1.0
−0.5
0.0
0.5
1.0
1.5
MR effect size for
'Mackerel liking || id:ebi−fl187−GCST90094796' on 'Rheumatoid arthritis || id:finngen_R11_M13_RHEUMA'

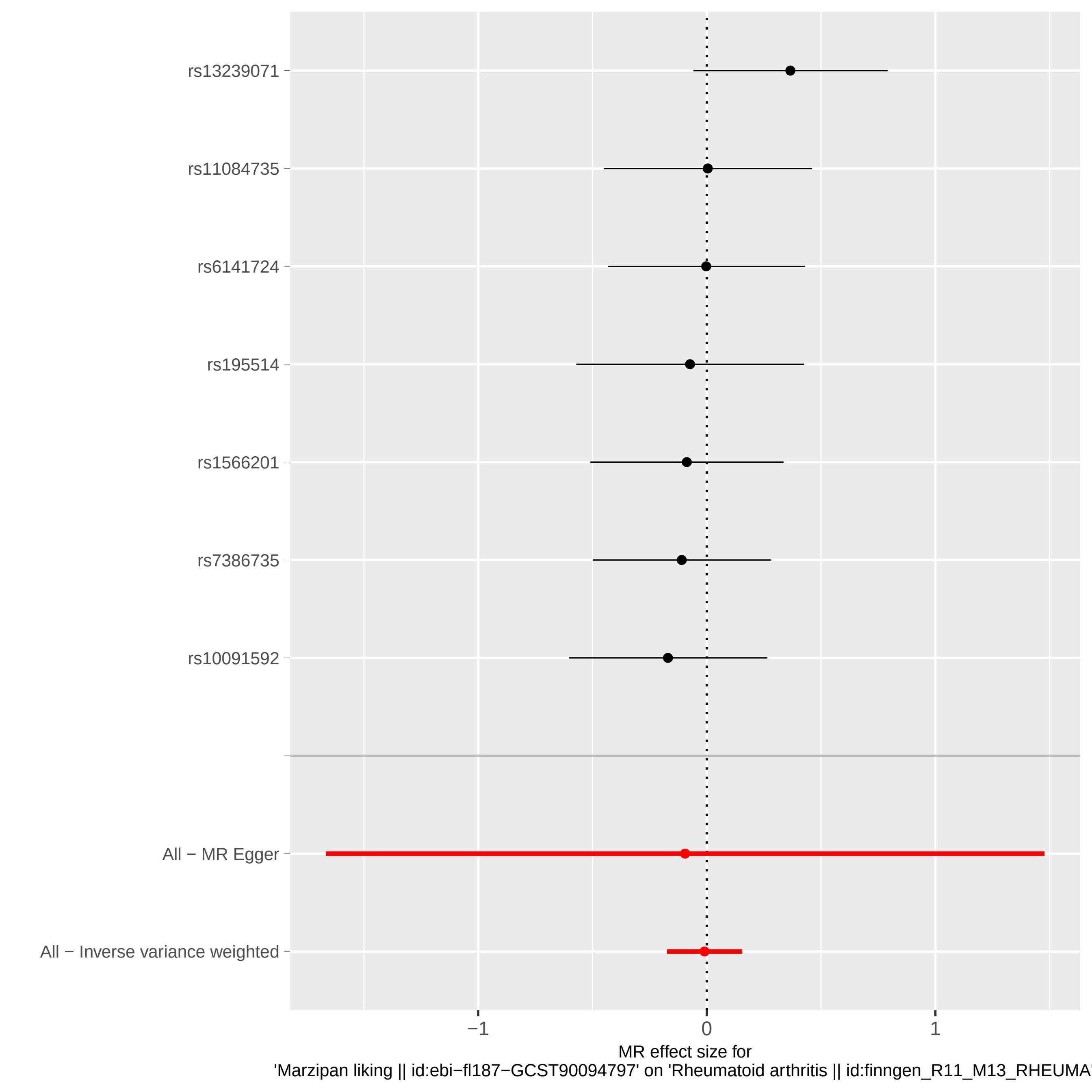

rs13239071
rs11084735
rs6141724
rs195514
rs1566201
rs7386735
rs10091592
All − MR Egger
All − Inverse variance weighted
−1
0
1
MR effect size for
'Marzipan liking || id:ebi−fl187−GCST90094797' on 'Rheumatoid arthritis || id:finngen_R11_M13_RHEUMA

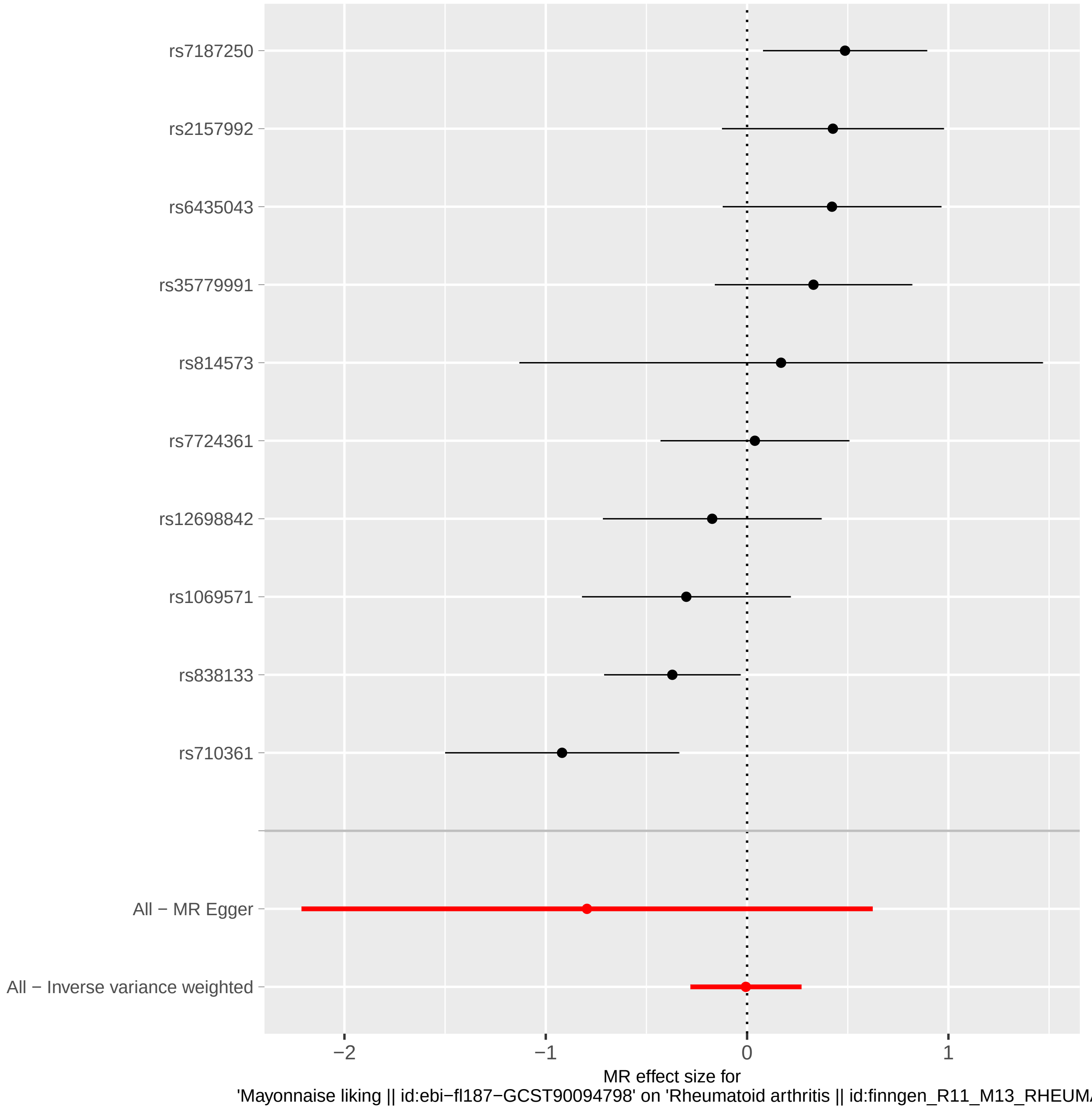
rs7187250
rs2157992
rs6435043
rs35779991
rs814573
rs7724361
rs12698842
rs1069571
rs838133
rs710361
All − MR Egger
All − Inverse variance weighted
−2
−1
0
1
MR effect size for
'Mayonnaise liking || id:ebi−fl187−GCST90094798' on 'Rheumatoid arthritis || id:finngen_R11_M13_RHEUM

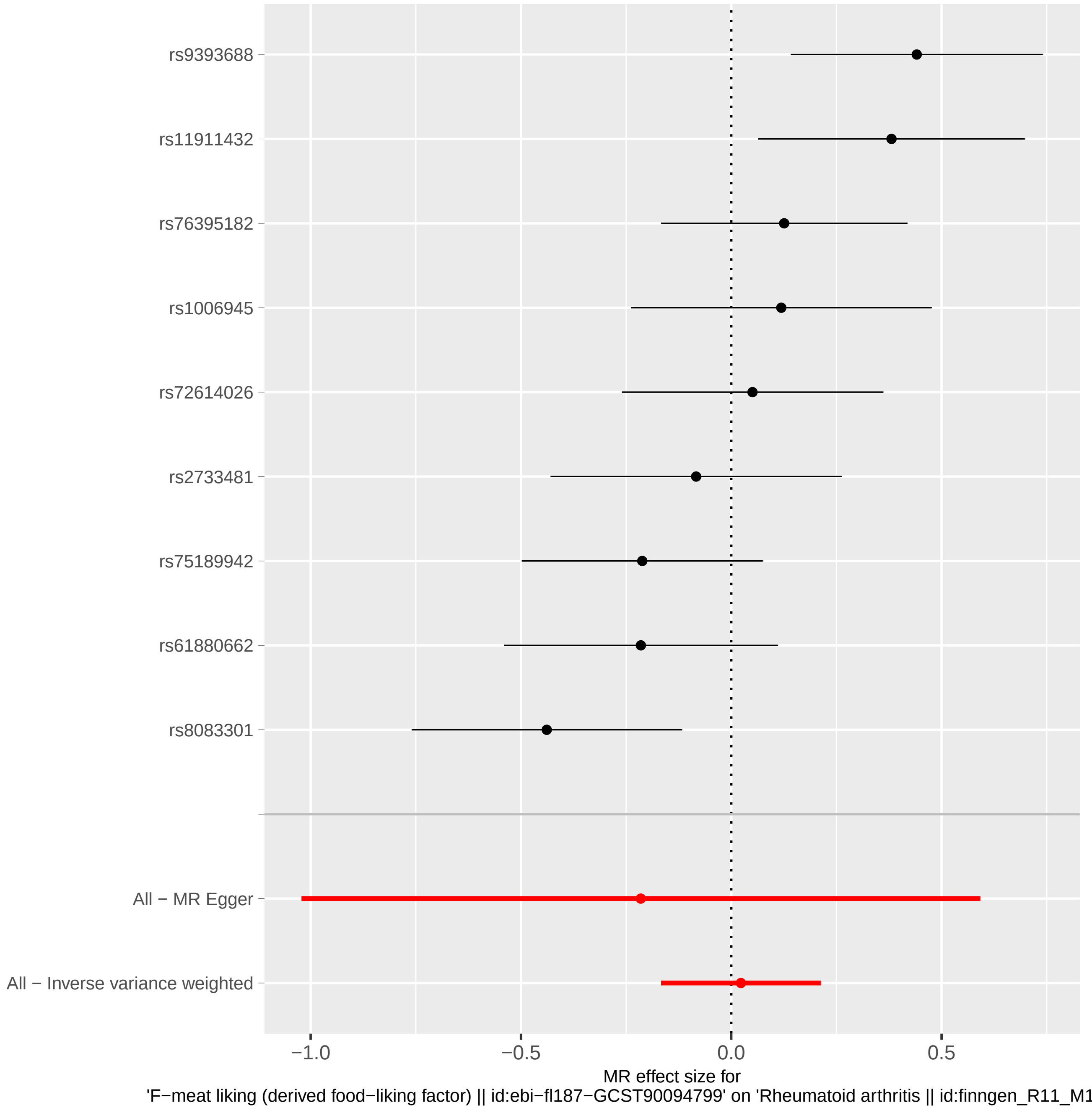
rs9393688
rs11911432
rs76395182
rs1006945
rs72614026
rs2733481
rs75189942
rs61880662
rs8083301
All − MR Egger
All − Inverse variance weighted
−1.0
−0.5
0.0
0.5
MR effect size for
'F−meat liking (derived food−liking factor) || id:ebi−fl187−GCST90094799' on 'Rheumatoid arthritis || id:finngen_R11_M1

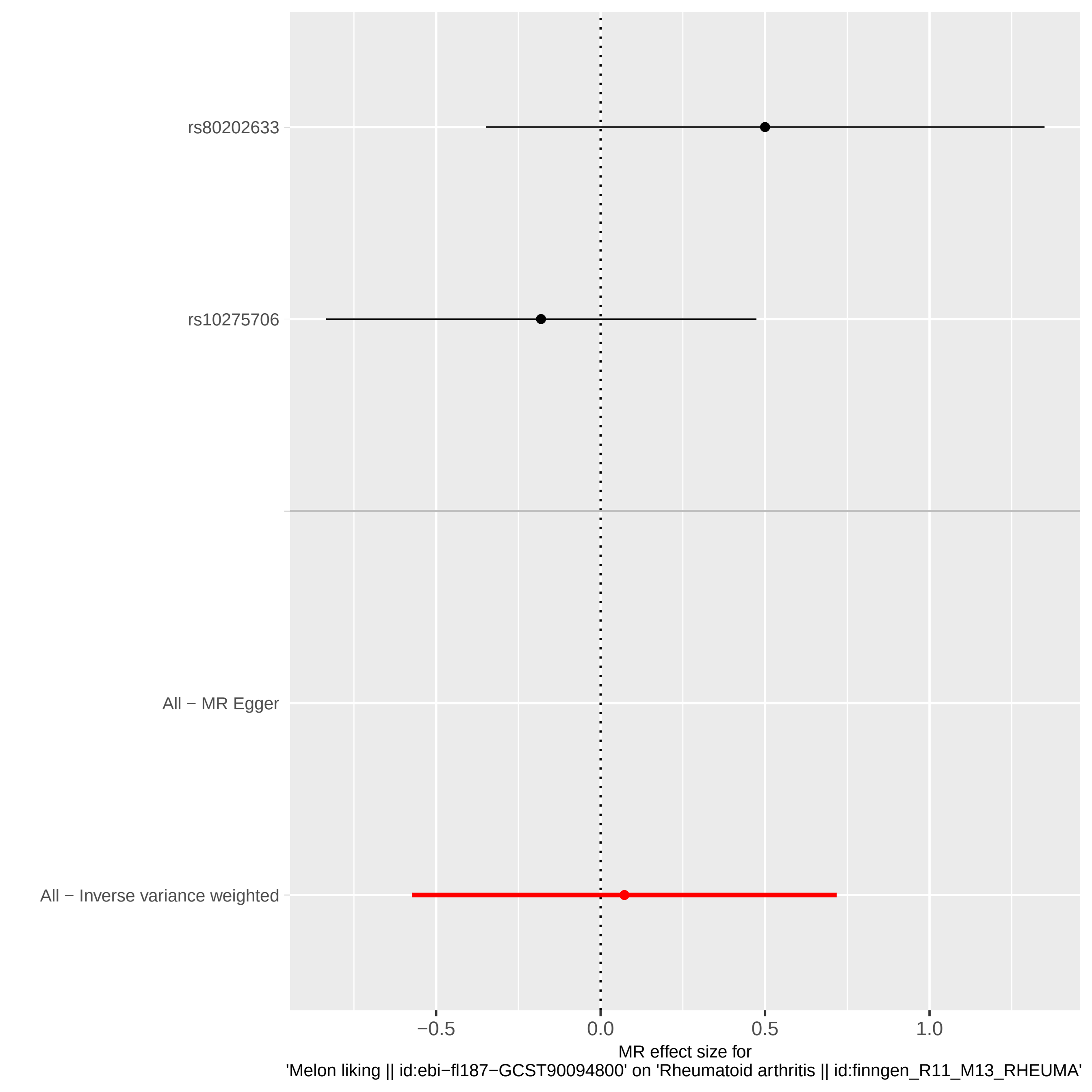
rs80202633
rs10275706
All − MR Egger
All − Inverse variance weighted
−0.5
0.0
0.5
1.0
MR effect size for
'Melon liking || id:ebi−fl187−GCST90094800' on 'Rheumatoid arthritis || id:finngen_R11_M13_RHEUMA'

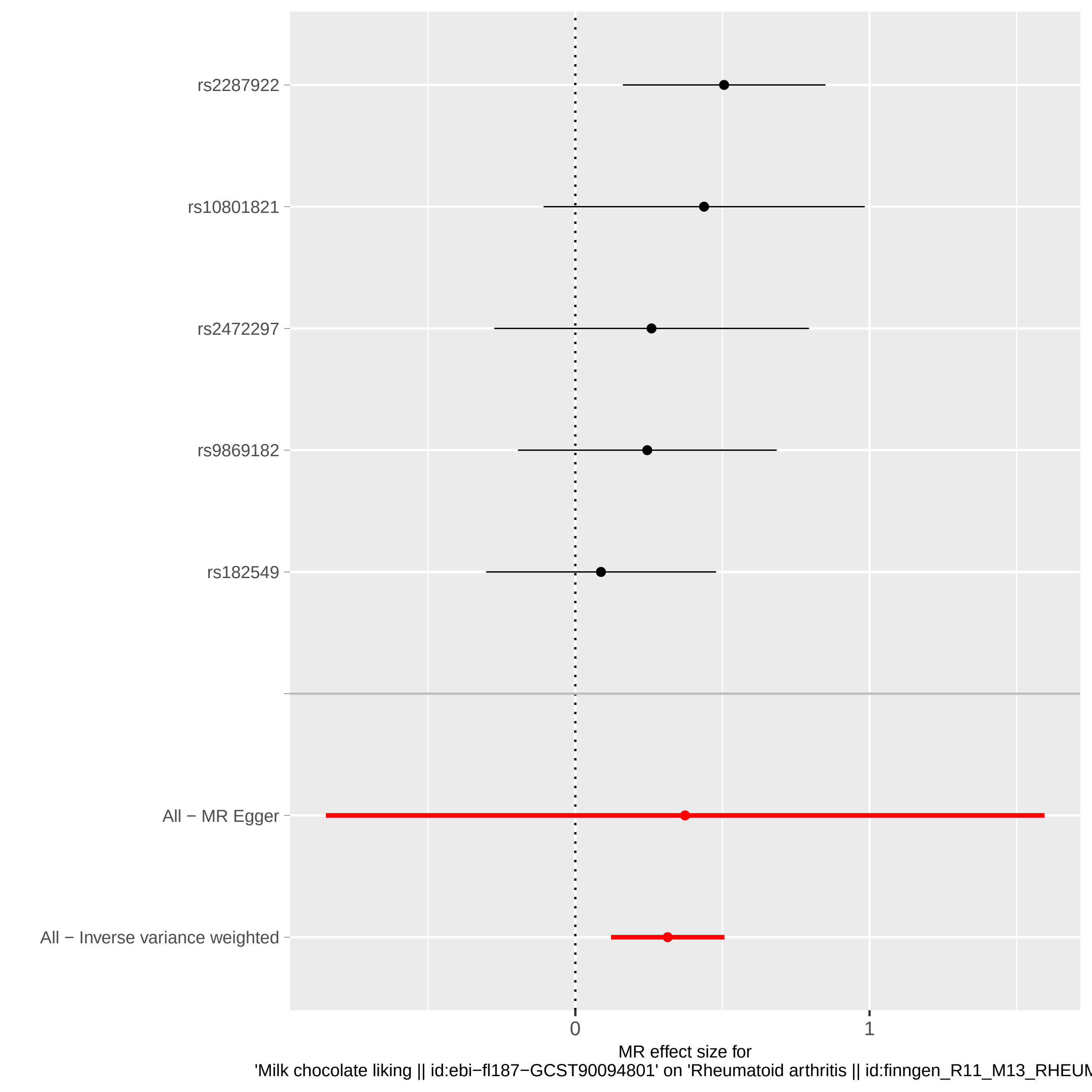

rs2287922
rs10801821
rs2472297
rs9869182
rs182549
All − MR Egger
All − Inverse variance weighted
0
1
MR effect size for
'Milk chocolate liking || id:ebi−fl187−GCST90094801' on 'Rheumatoid arthritis || id:finngen_R11_M13_RHEUM

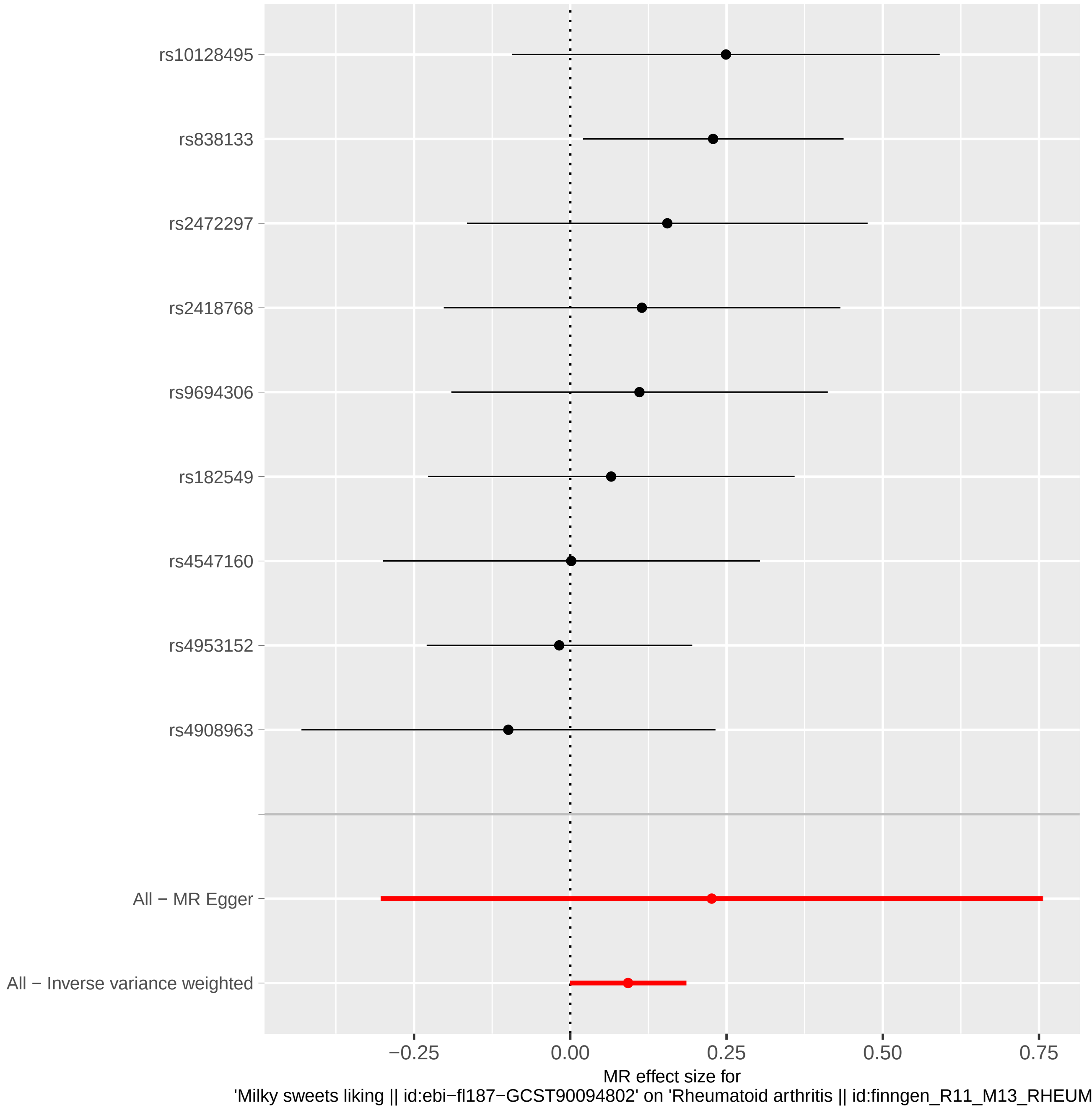
rs10128495
rs838133
rs2472297
rs2418768
rs9694306
rs182549
rs4547160
rs4953152
rs4908963
All − MR Egger
All − Inverse variance weighted
−0.25
0.00
0.25
0.50
0.75
MR effect size for
'Milky sweets liking || id:ebi−fl187−GCST90094802' on 'Rheumatoid arthritis || id:finngen_R11_M13_RHEUM

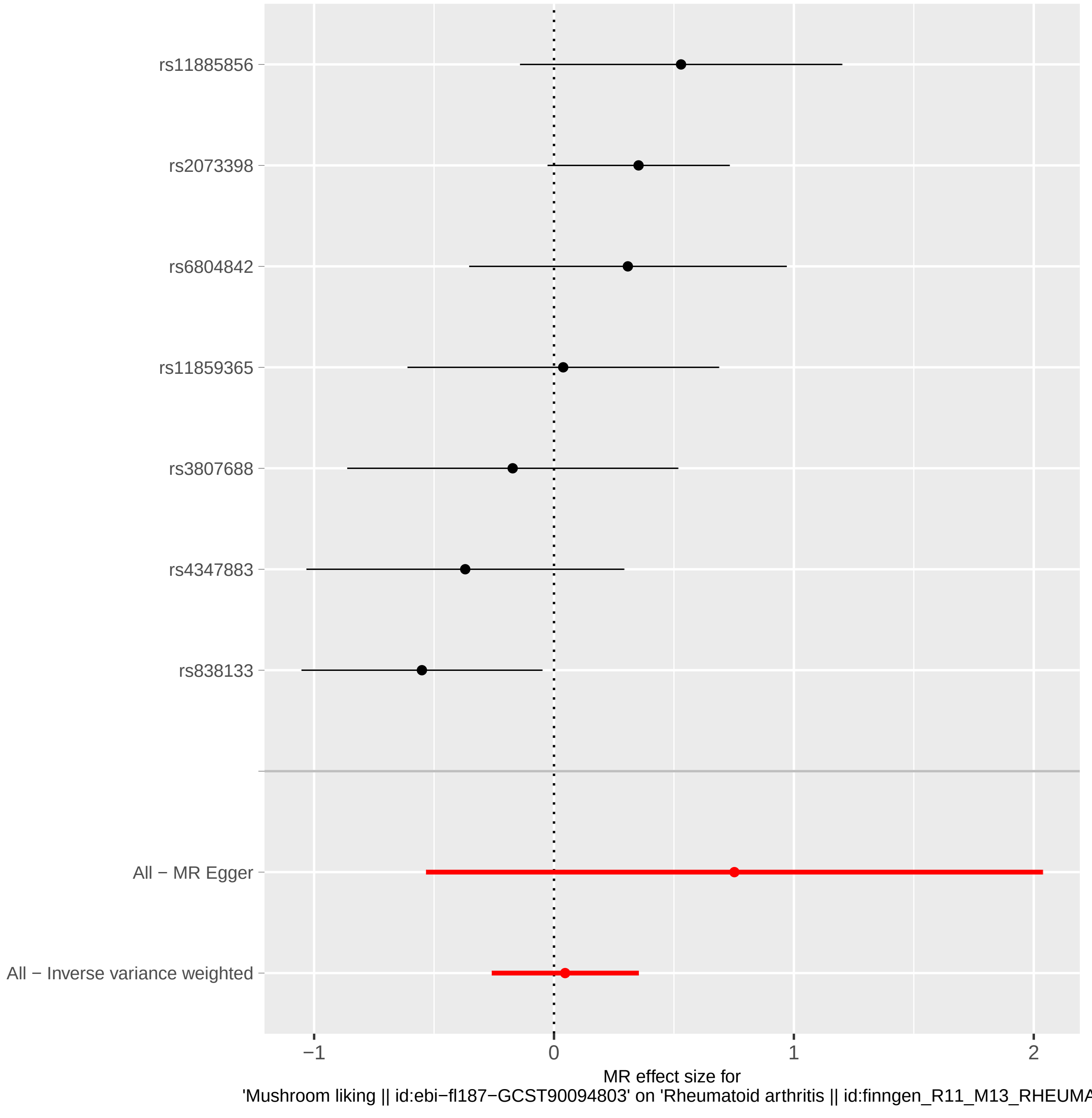

rs11885856
rs2073398
rs6804842
rs11859365
rs3807688
rs4347883
rs838133
All − MR Egger
All − Inverse variance weighted
−1
0
1
2
MR effect size for
'Mushroom liking || id:ebi−fl187−GCST90094803' on 'Rheumatoid arthritis || id:finngen_R11_M13_RHEUMA

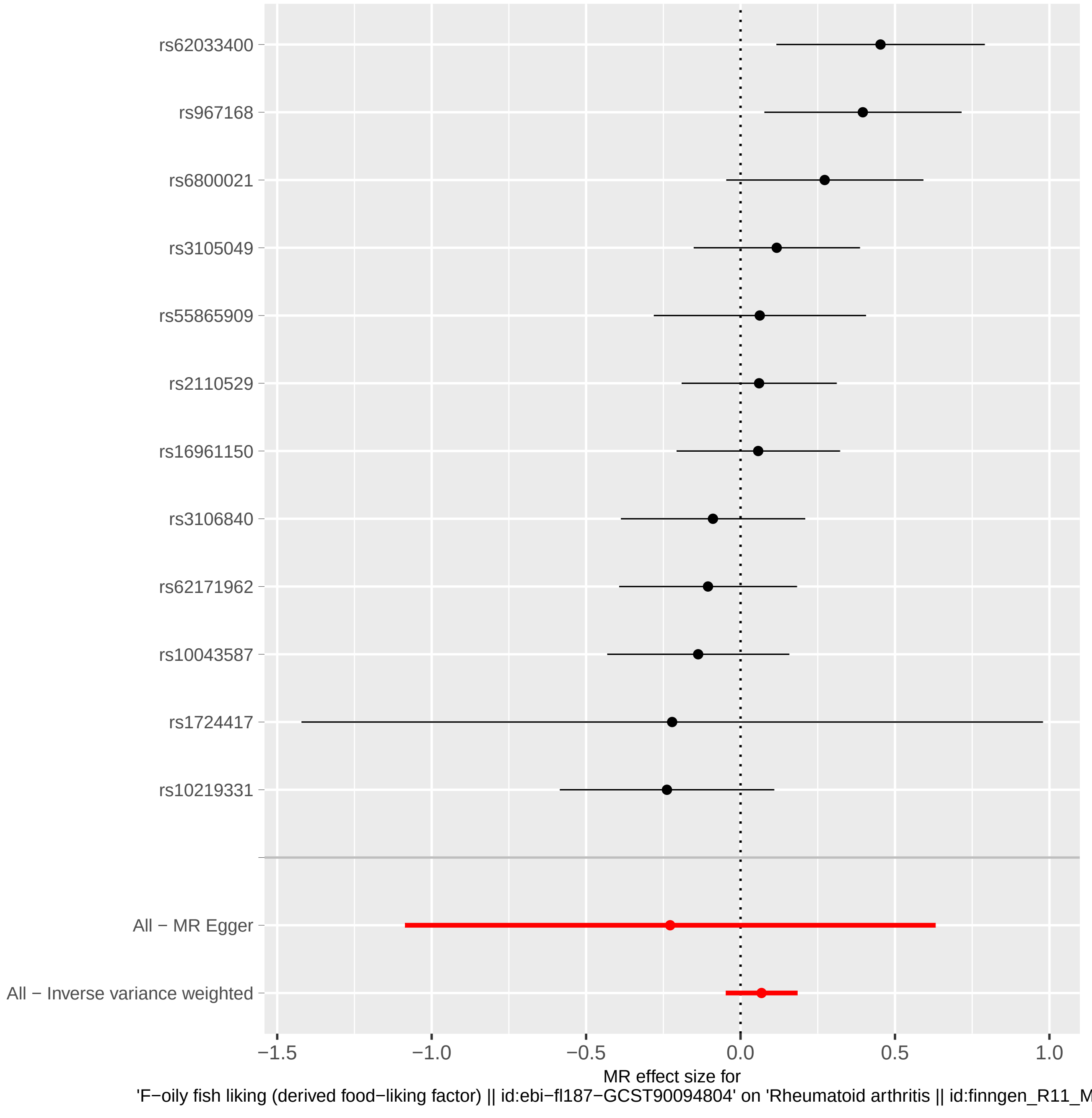
rs62033400
rs967168
rs6800021
rs3105049
rs55865909
rs2110529
rs16961150
rs3106840
rs62171962
rs10043587
rs1724417
rs10219331
All − MR Egger
All − Inverse variance weighted
−1.5
−1.0
−0.5
0.0
0.5
1.0
MR effect size for
'F−oily fish liking (derived food−liking factor) || id:ebi−fl187−GCST90094804' on 'Rheumatoid arthritis || id:finngen_R11_M

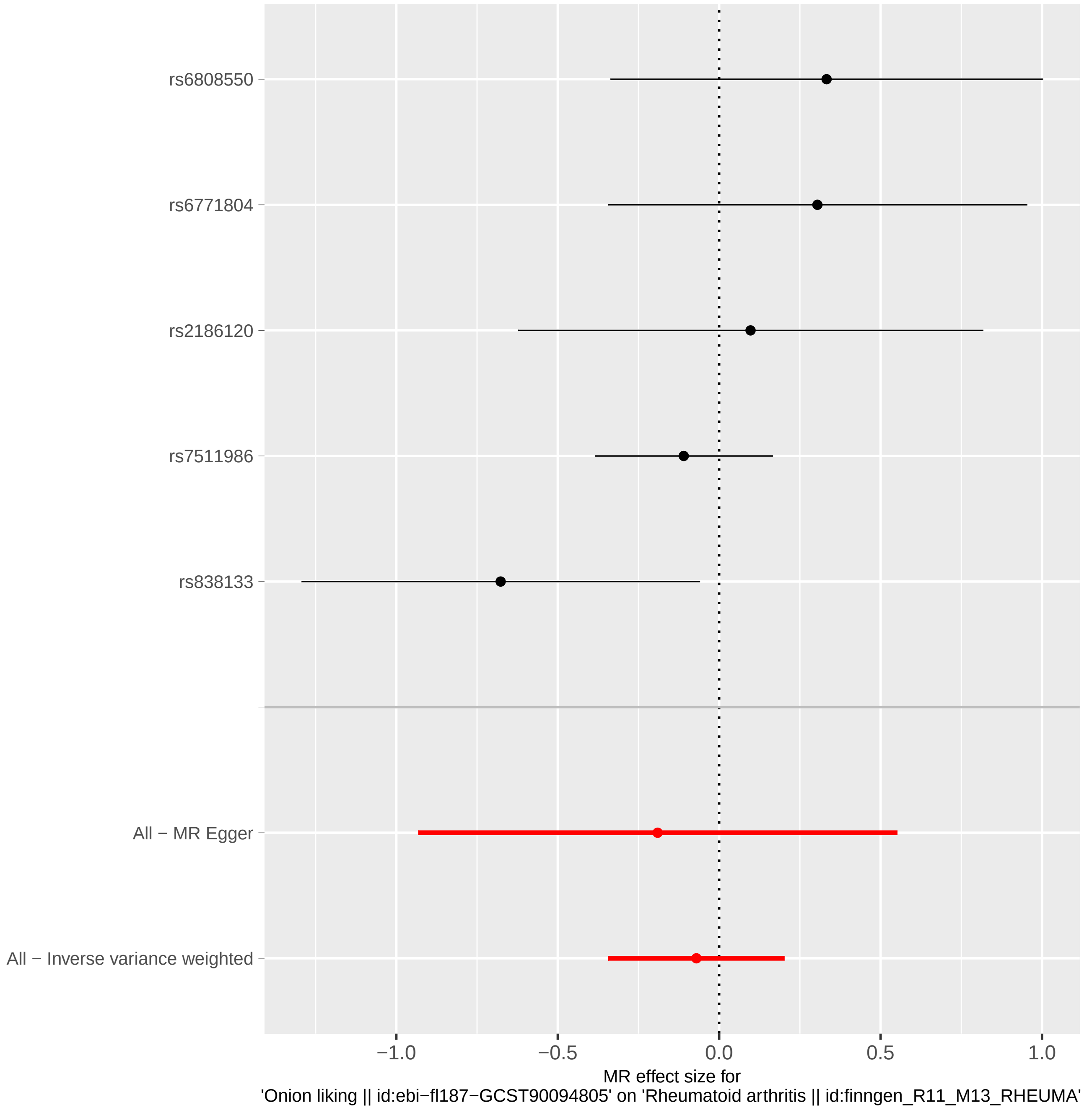
rs6808550
rs6771804
rs2186120
rs7511986
rs838133
All − MR Egger
All − Inverse variance weighted
−1.0
−0.5
0.0
0.5
1.0
MR effect size for
'Onion liking || id:ebi−fl187−GCST90094805' on 'Rheumatoid arthritis || id:finngen_R11_M13_RHEUMA'

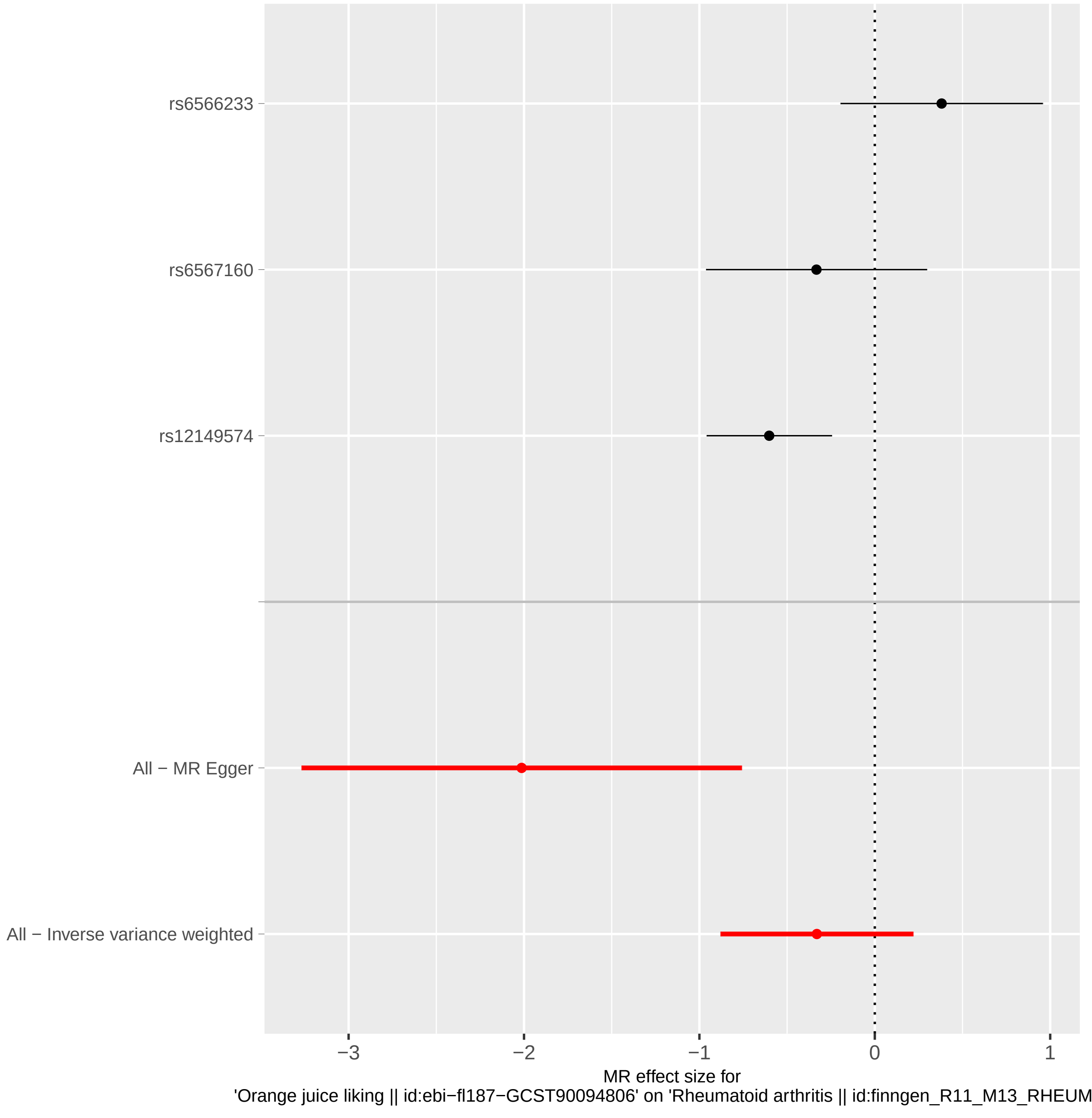
rs6566233
rs6567160
rs12149574
All − MR Egger
All − Inverse variance weighted
−3
−2
−1
0
1
MR effect size for
'Orange juice liking || id:ebi−fl187−GCST90094806' on 'Rheumatoid arthritis || id:finngen_R11_M13_RHEUM

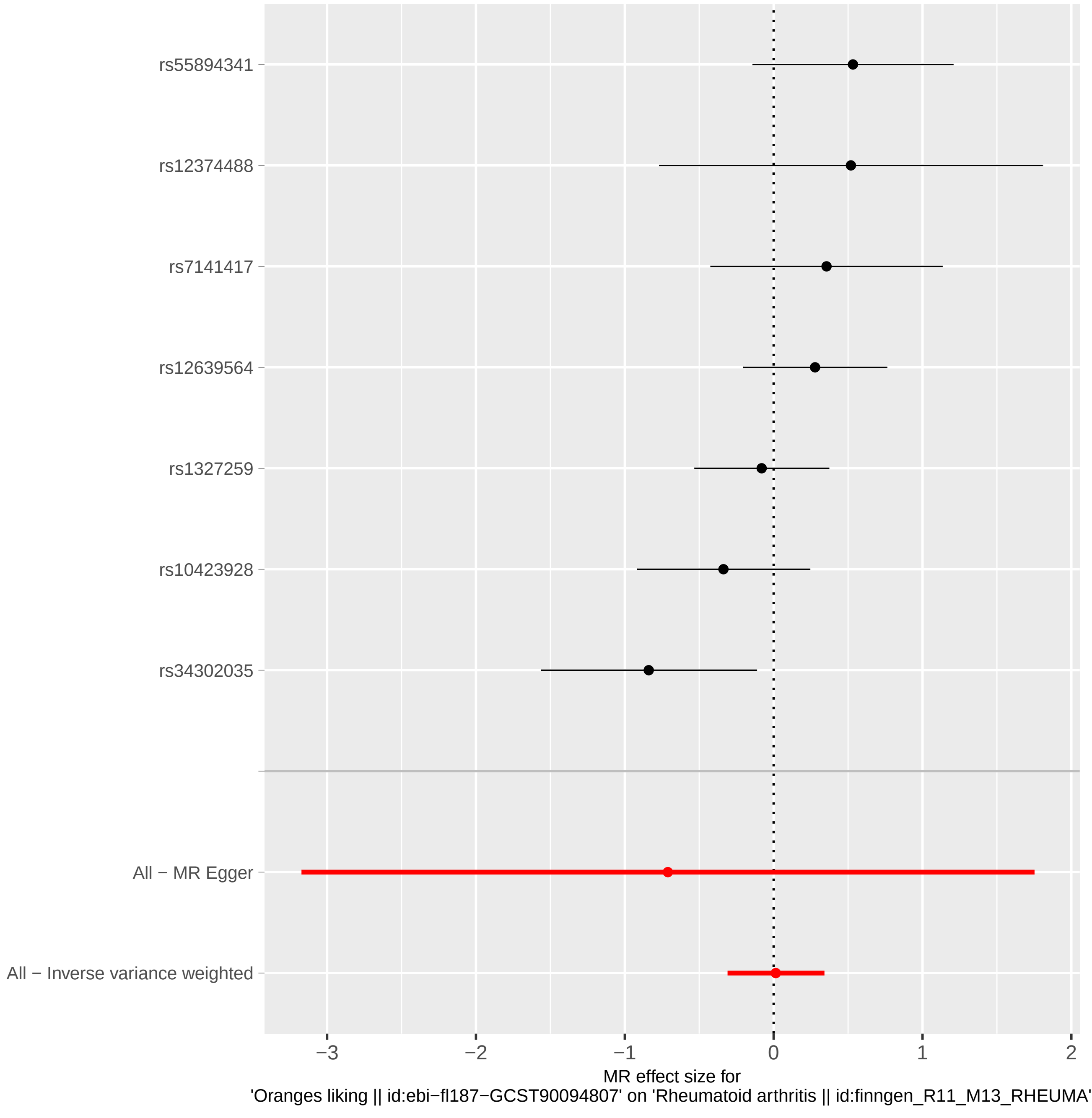

rs55894341
rs12374488
rs7141417
rs12639564
rs1327259
rs10423928
rs34302035
All − MR Egger
All − Inverse variance weighted
−3
−2
−1
0
1
2
MR effect size for
'Oranges liking || id:ebi−fl187−GCST90094807' on 'Rheumatoid arthritis || id:finngen_R11_M13_RHEUMA'

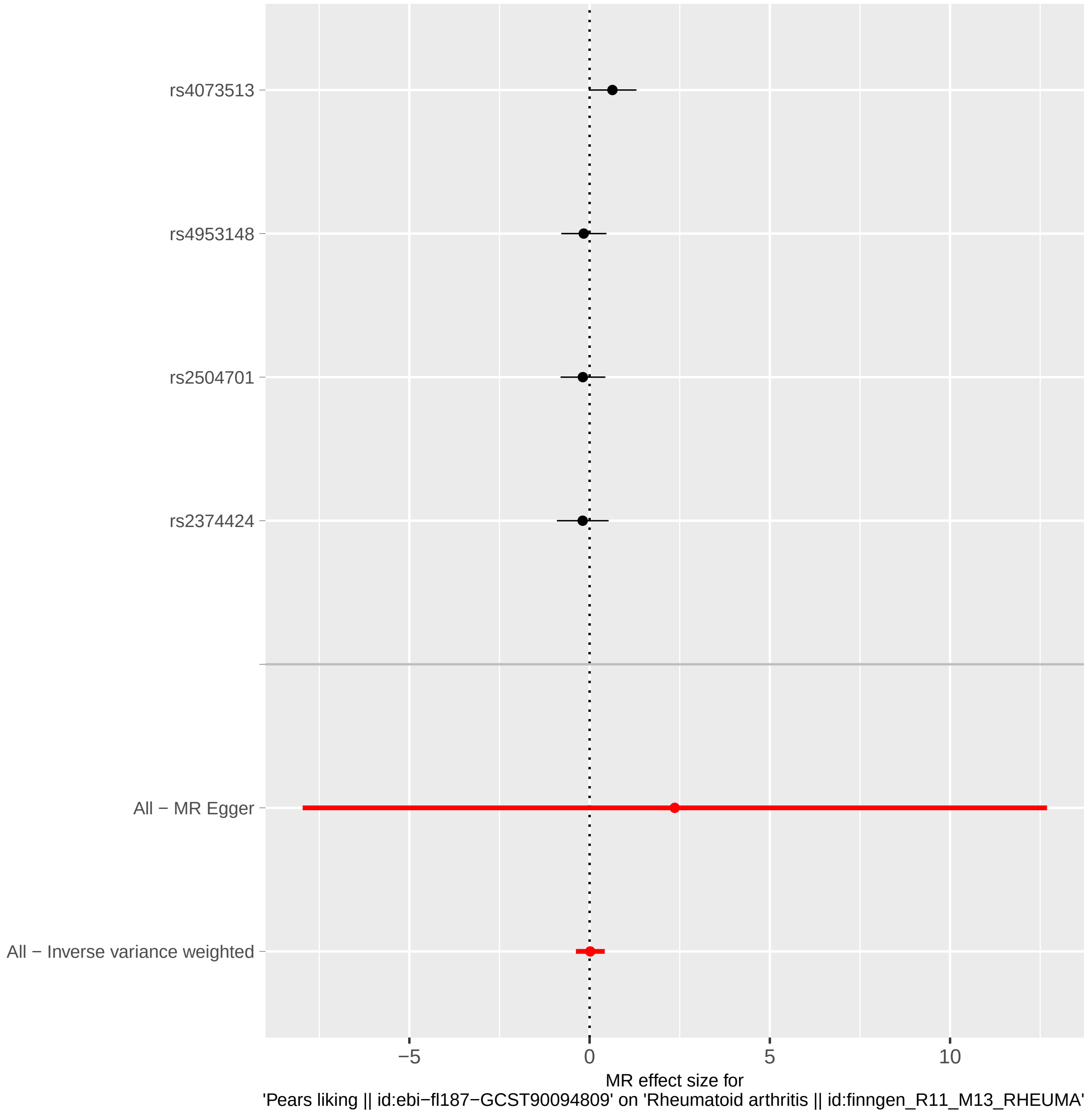

rs4073513
rs4953148
rs2504701
rs2374424
All − MR Egger
All − Inverse variance weighted
−5
0
5
10
MR effect size for
'Pears liking || id:ebi−fl187−GCST90094809' on 'Rheumatoid arthritis || id:finngen_R11_M13_RHEUMA'

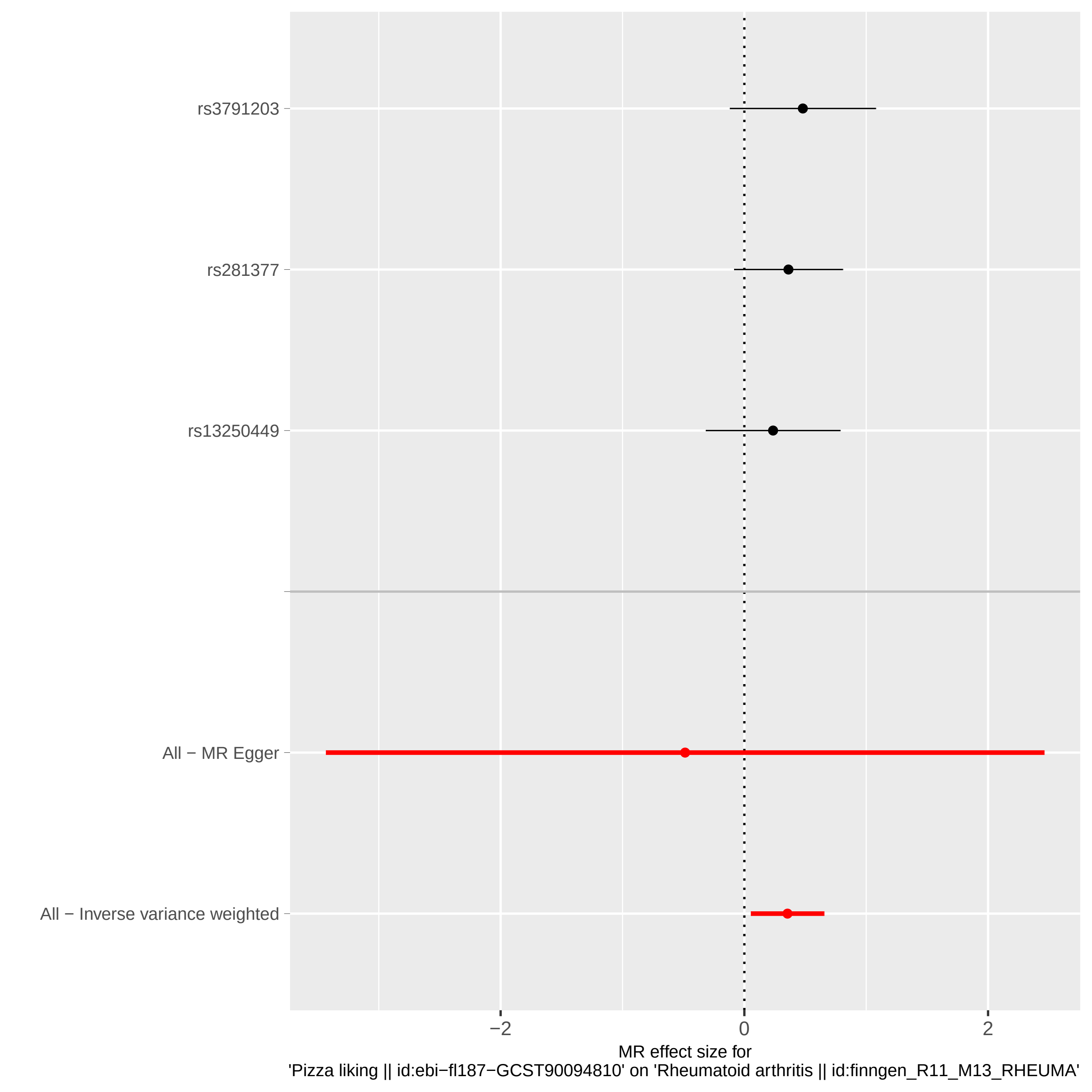

rs3791203
rs281377
rs13250449
All − MR Egger
All − Inverse variance weighted
−2
0
2
MR effect size for
'Pizza liking || id:ebi−fl187−GCST90094810' on 'Rheumatoid arthritis || id:finngen_R11_M13_RHEUMA'

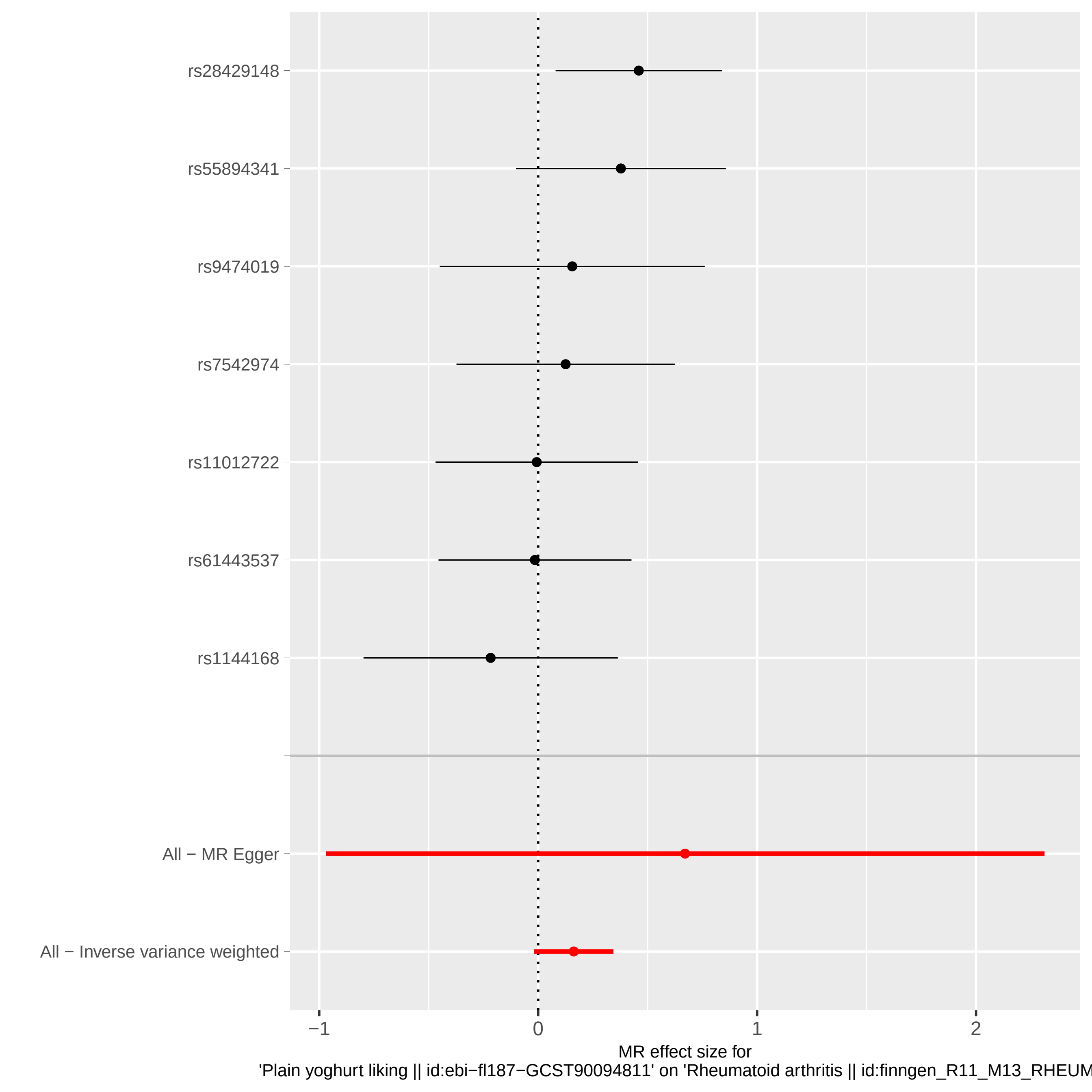
rs28429148
rs55894341
rs9474019
rs7542974
rs11012722
rs61443537
rs1144168
All − MR Egger
All − Inverse variance weighted
−1
0
1
2
MR effect size for
'Plain yoghurt liking || id:ebi−fl187−GCST90094811' on 'Rheumatoid arthritis || id:finngen_R11_M13_RHEUM

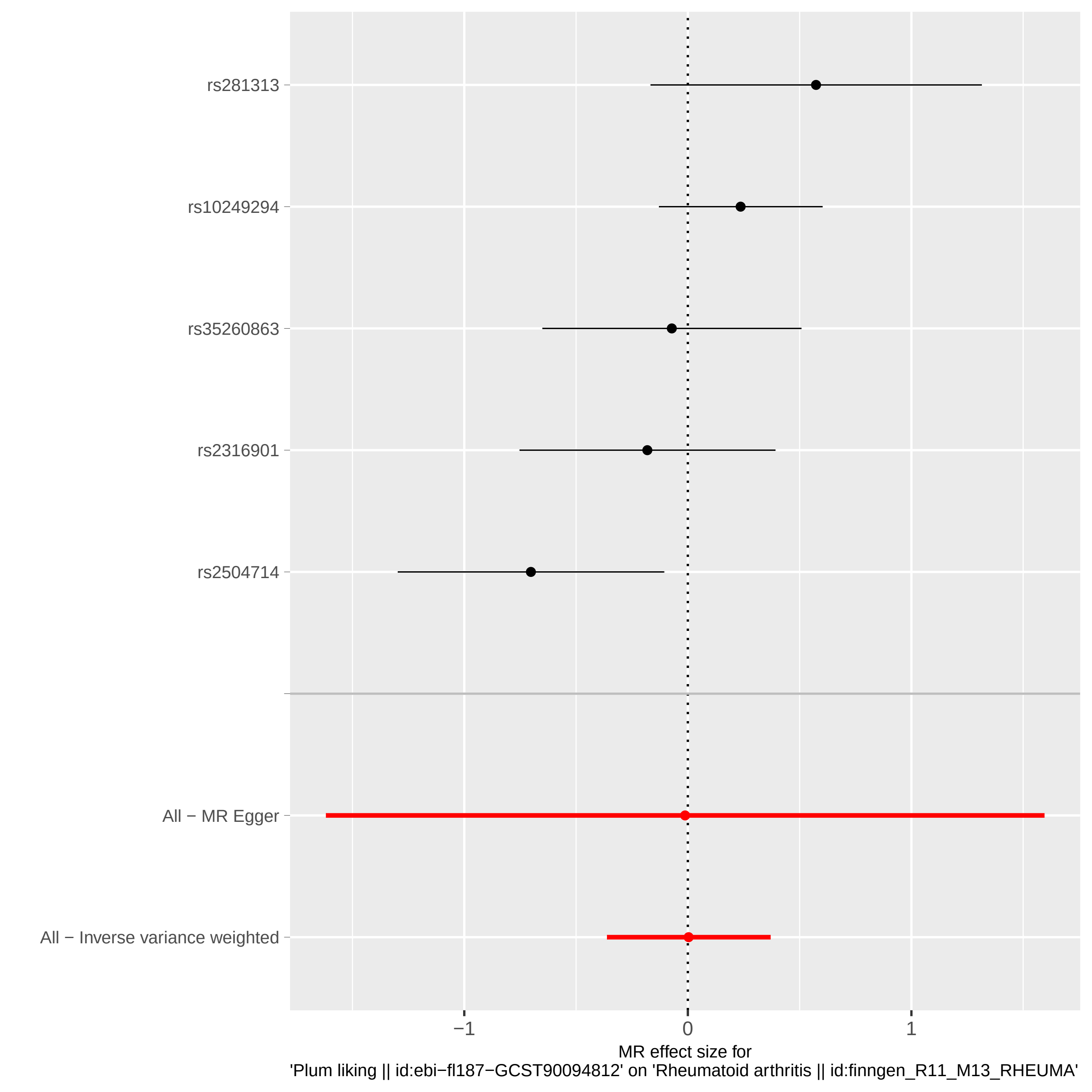

rs281313
rs10249294
rs35260863
rs2316901
rs2504714
All − MR Egger
All − Inverse variance weighted
−1
0
1
MR effect size for
'Plum liking || id:ebi−fl187−GCST90094812' on 'Rheumatoid arthritis || id:finngen_R11_M13_RHEUMA'

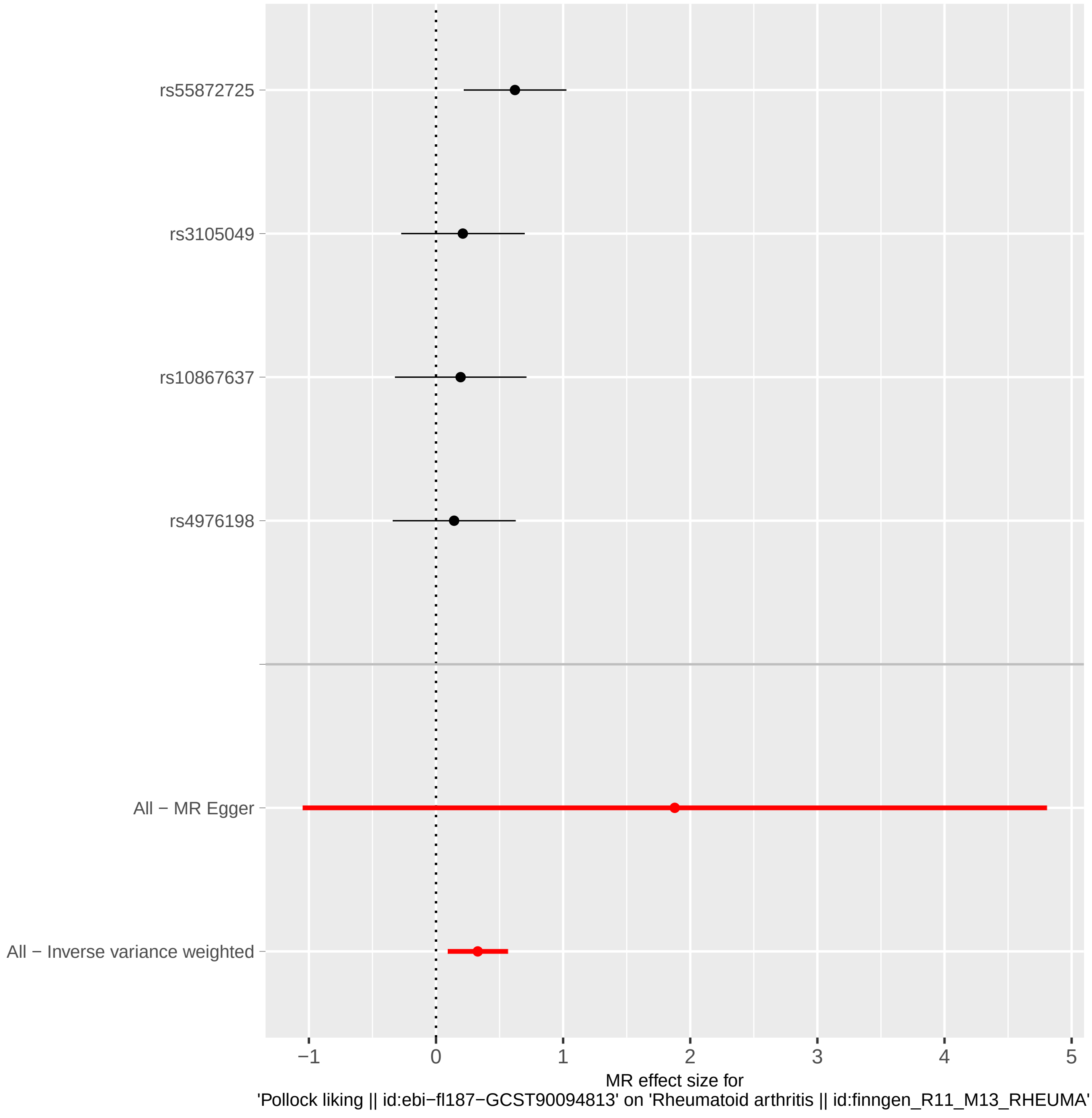
rs55872725
rs3105049
rs10867637
rs4976198
All − MR Egger
All − Inverse variance weighted
−1
0
1
2
3
4
5
MR effect size for
'Pollock liking || id:ebi−fl187−GCST90094813' on 'Rheumatoid arthritis || id:finngen_R11_M13_RHEUMA'

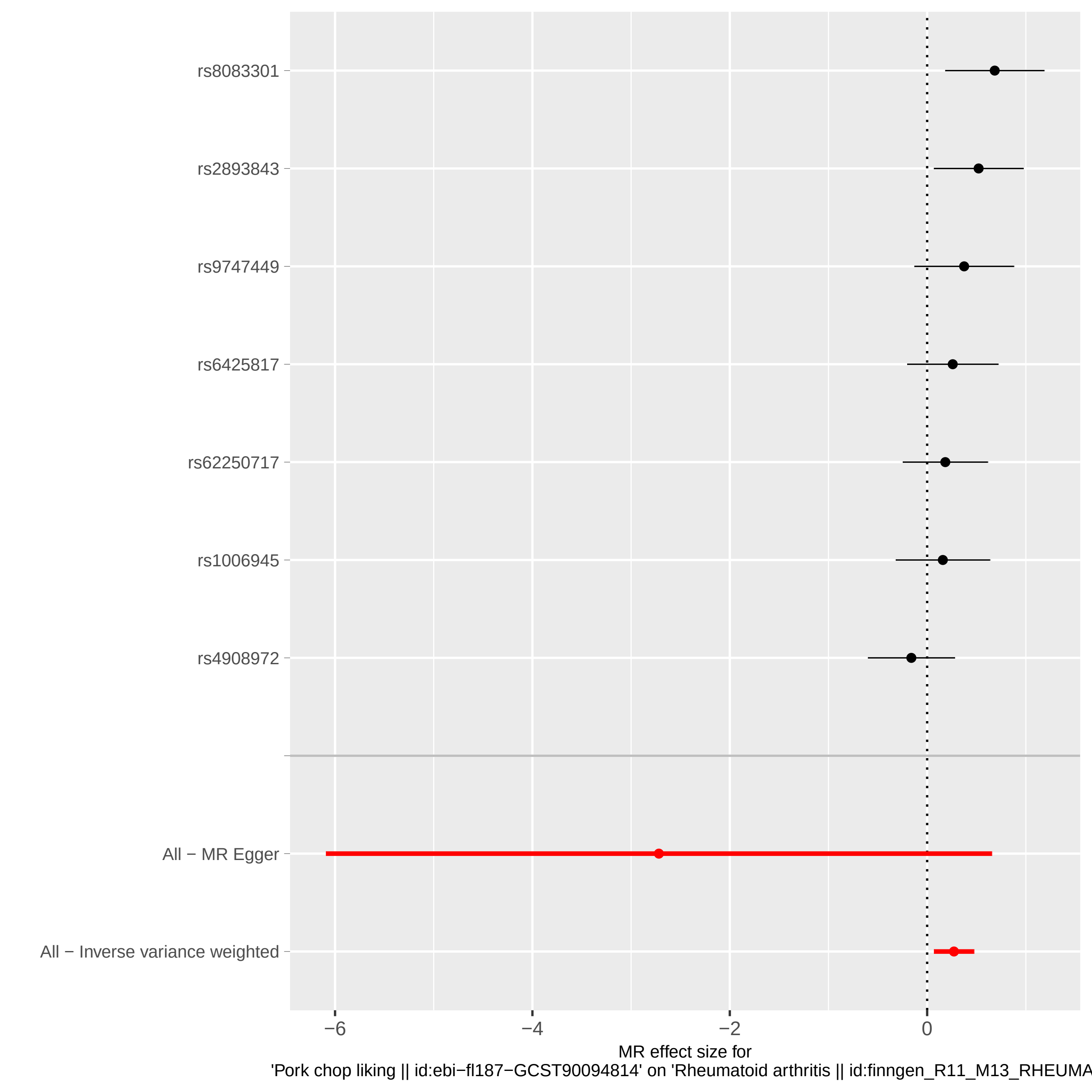

rs8083301
rs2893843
rs9747449
rs6425817
rs62250717
rs1006945
rs4908972
All − MR Egger
All − Inverse variance weighted
−6
−4
−2
0
MR effect size for
'Pork chop liking || id:ebi−fl187−GCST90094814' on 'Rheumatoid arthritis || id:finngen_R11_M13_RHEUMA

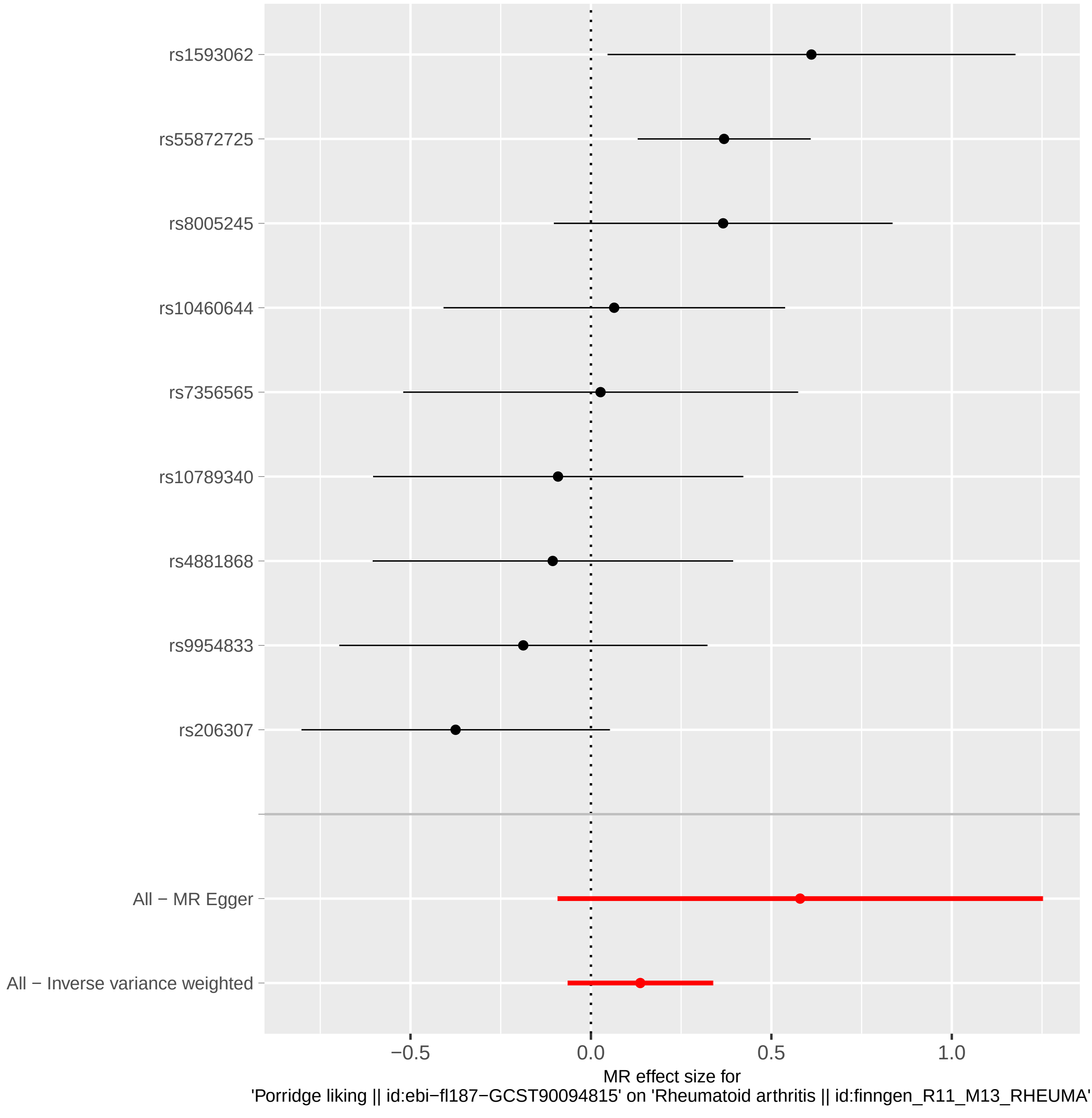

rs1593062
rs55872725
rs8005245
rs10460644
rs7356565
rs10789340
rs4881868
rs9954833
rs206307
All − MR Egger
All − Inverse variance weighted
−0.5
0.0
0.5
1.0
MR effect size for
'Porridge liking || id:ebi−fl187−GCST90094815' on 'Rheumatoid arthritis || id:finngen_R11_M13_RHEUMA'

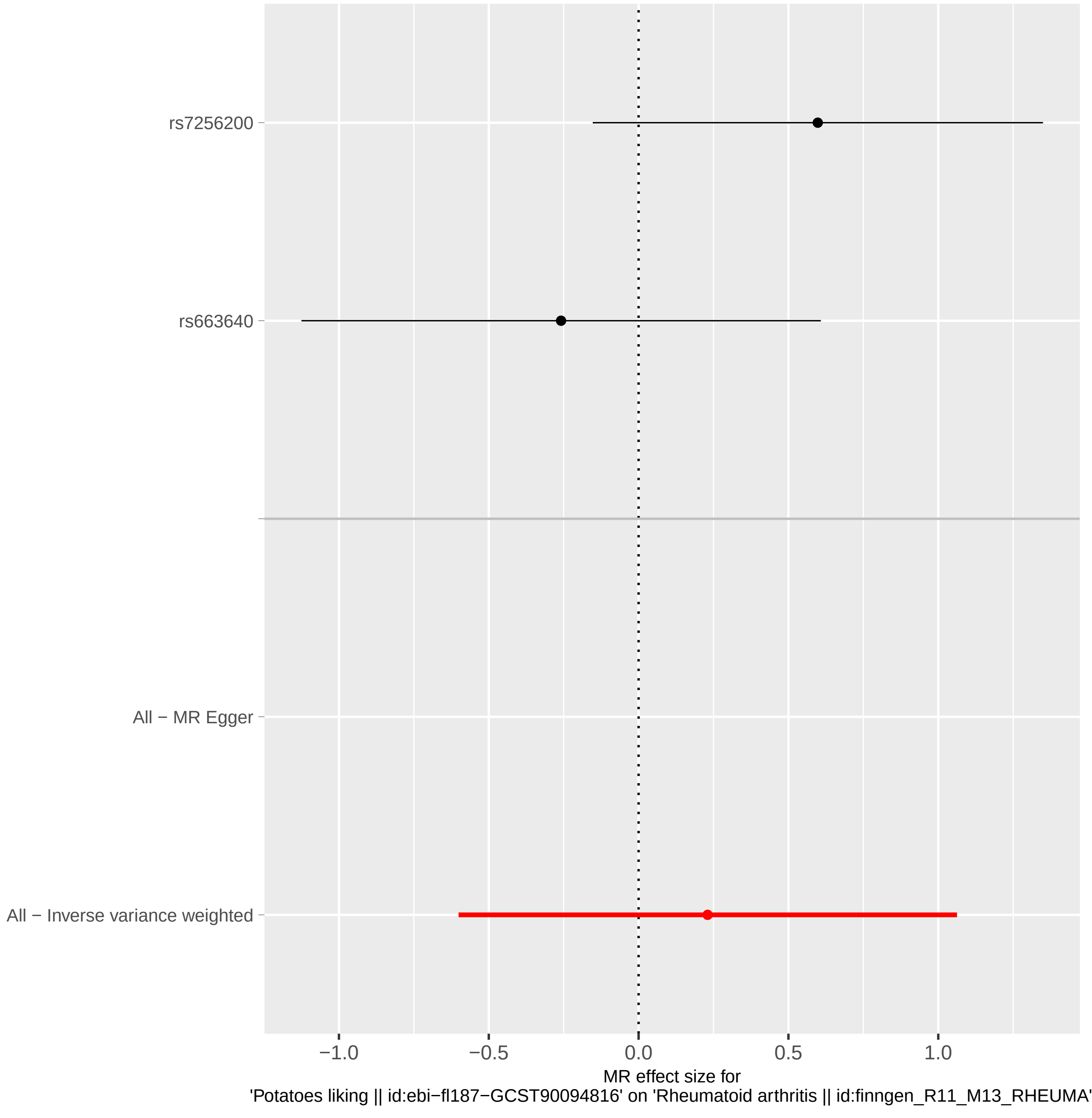
rs7256200
rs663640
All − MR Egger
All − Inverse variance weighted
−1.0
−0.5
0.0
0.5
1.0
MR effect size for
'Potatoes liking || id:ebi−fl187−GCST90094816' on 'Rheumatoid arthritis || id:finngen_R11_M13_RHEUMA'

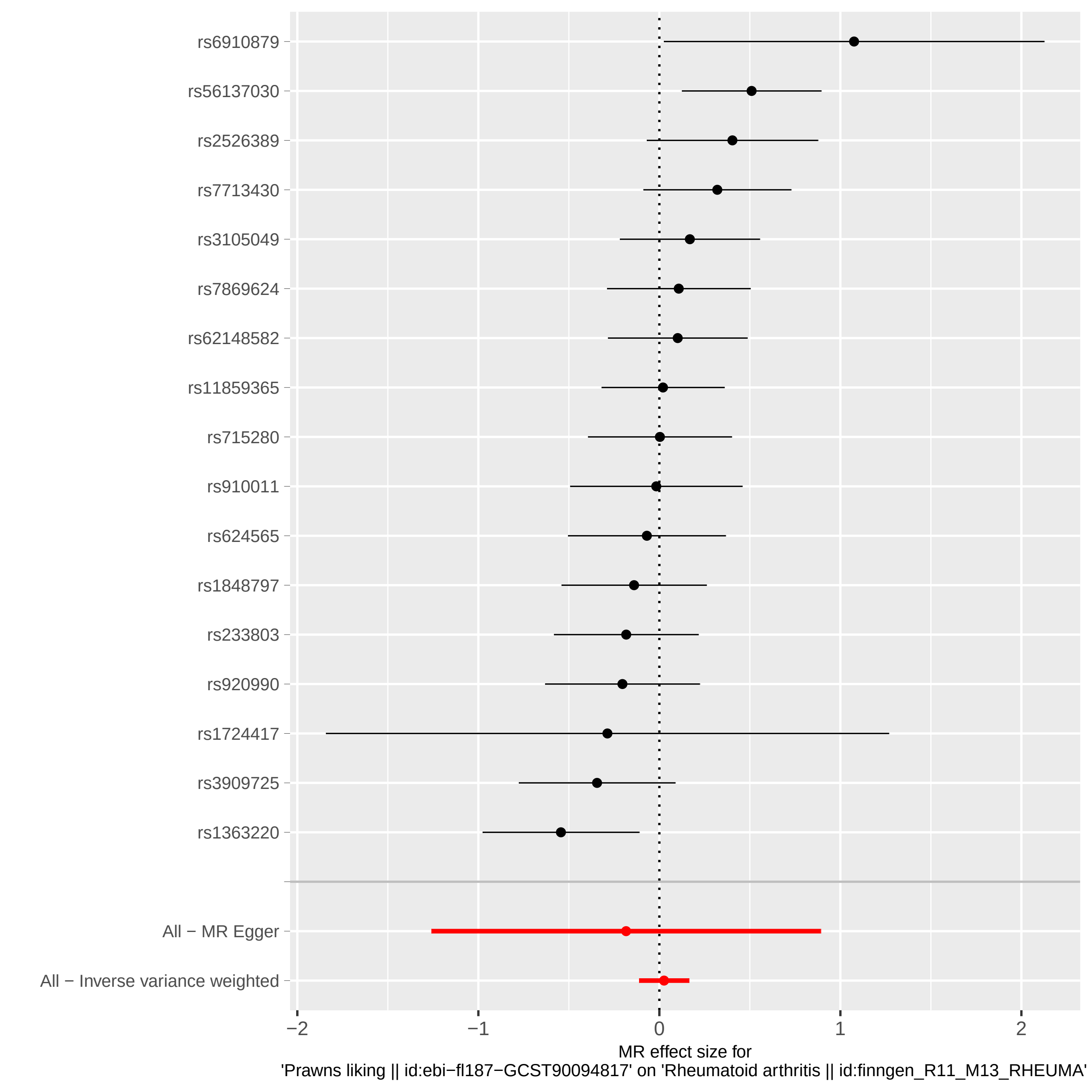
rs6910879
rs56137030
rs2526389
rs7713430
rs3105049
rs7869624
rs62148582
rs11859365
rs715280
rs910011
rs624565
rs1848797
rs233803
rs920990
rs1724417
rs3909725
rs1363220
All − MR Egger
All − Inverse variance weighted
−2
−1
0
1
2
MR effect size for
'Prawns liking || id:ebi−fl187−GCST90094817' on 'Rheumatoid arthritis || id:finngen_R11_M13_RHEUMA'

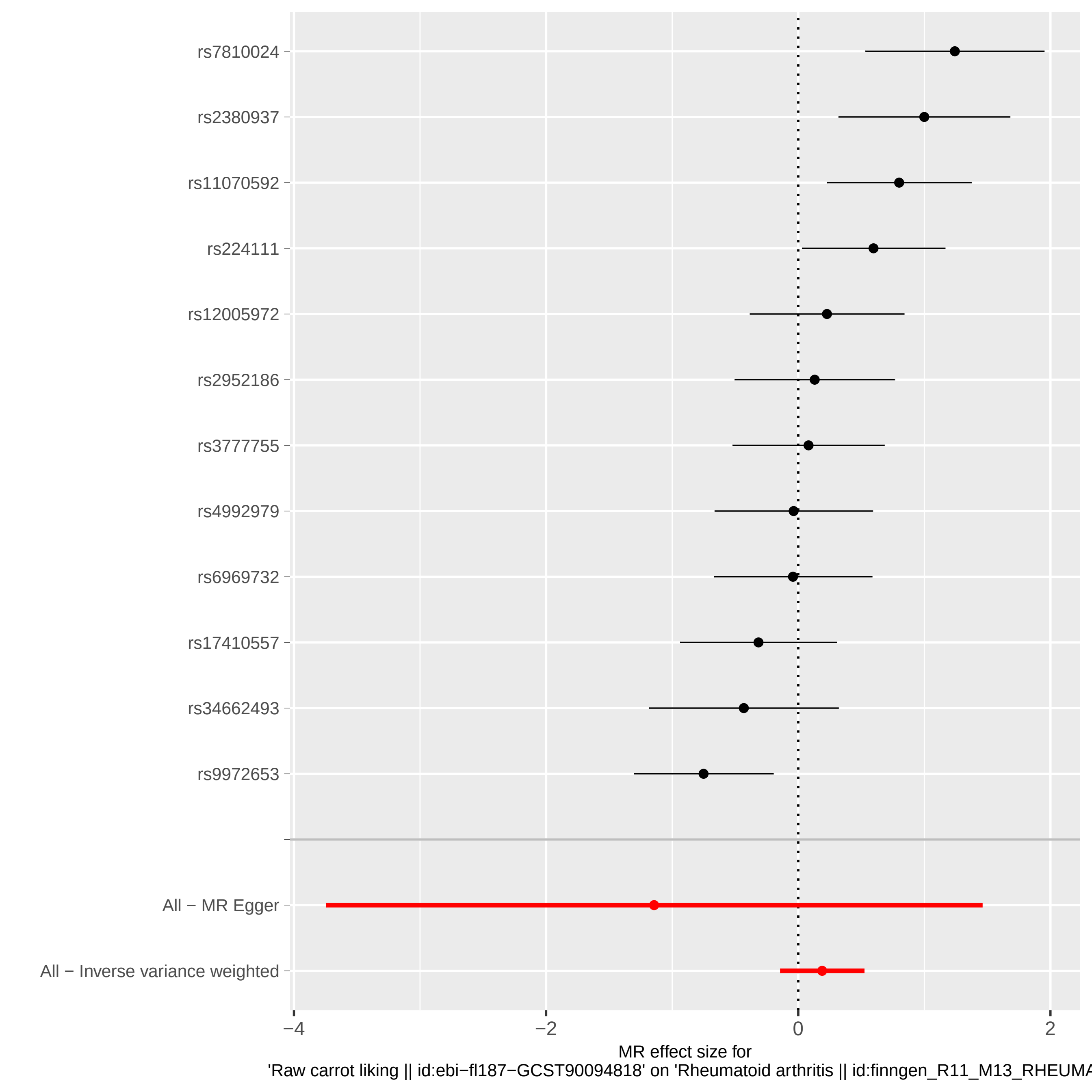
rs7810024
rs2380937
rs11070592
rs224111
rs12005972
rs2952186
rs3777755
rs4992979
rs6969732
rs17410557
rs34662493
rs9972653
All − MR Egger
All − Inverse variance weighted
−4
−2
0
2
MR effect size for
'Raw carrot liking || id:ebi−fl187−GCST90094818' on 'Rheumatoid arthritis || id:finngen_R11_M13_RHEUMA

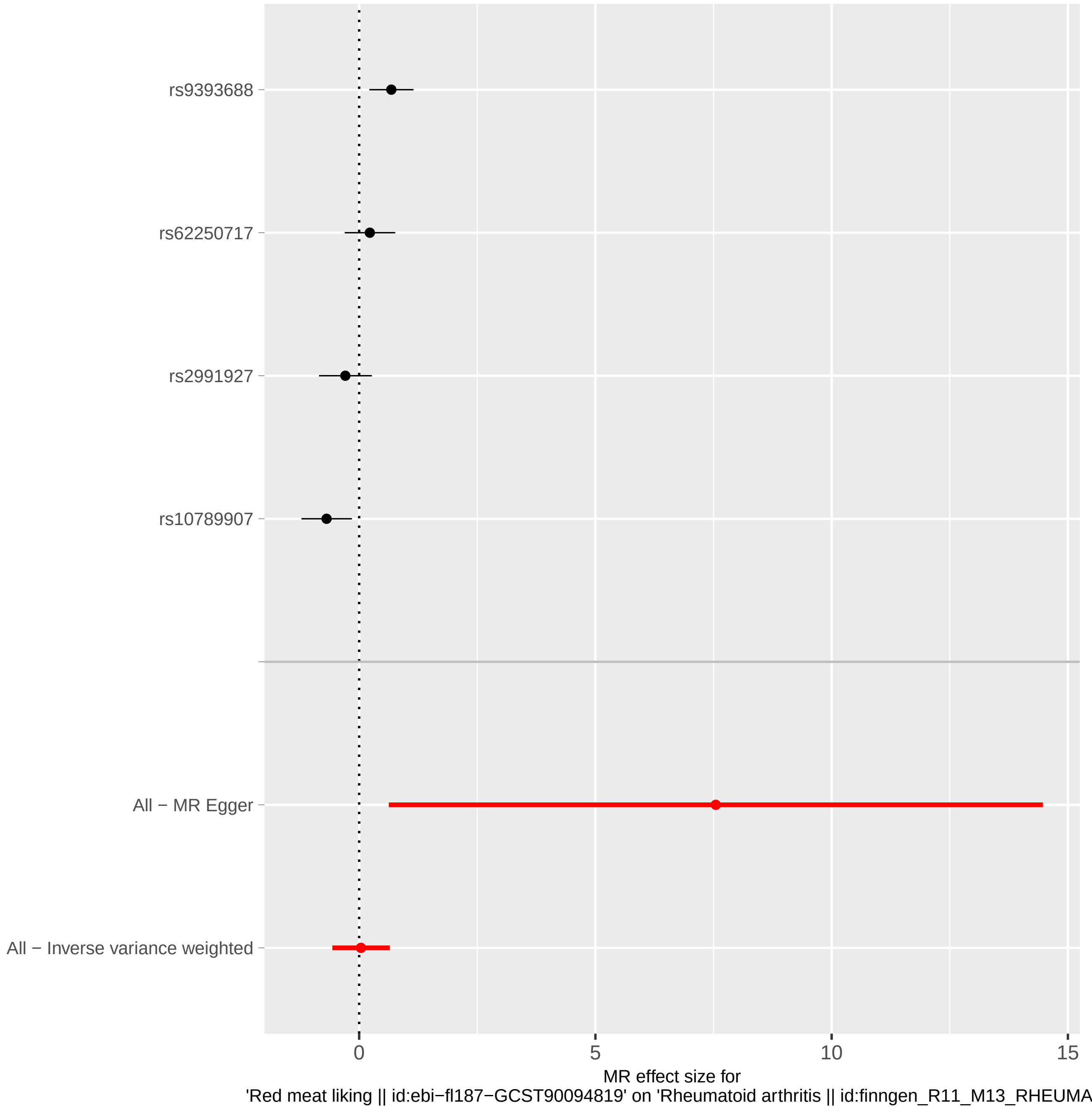
rs9393688
rs62250717
rs2991927
rs10789907
All − MR Egger
All − Inverse variance weighted
0
5
10
15
MR effect size for
'Red meat liking || id:ebi−fl187−GCST90094819' on 'Rheumatoid arthritis || id:finngen_R11_M13_RHEUMA

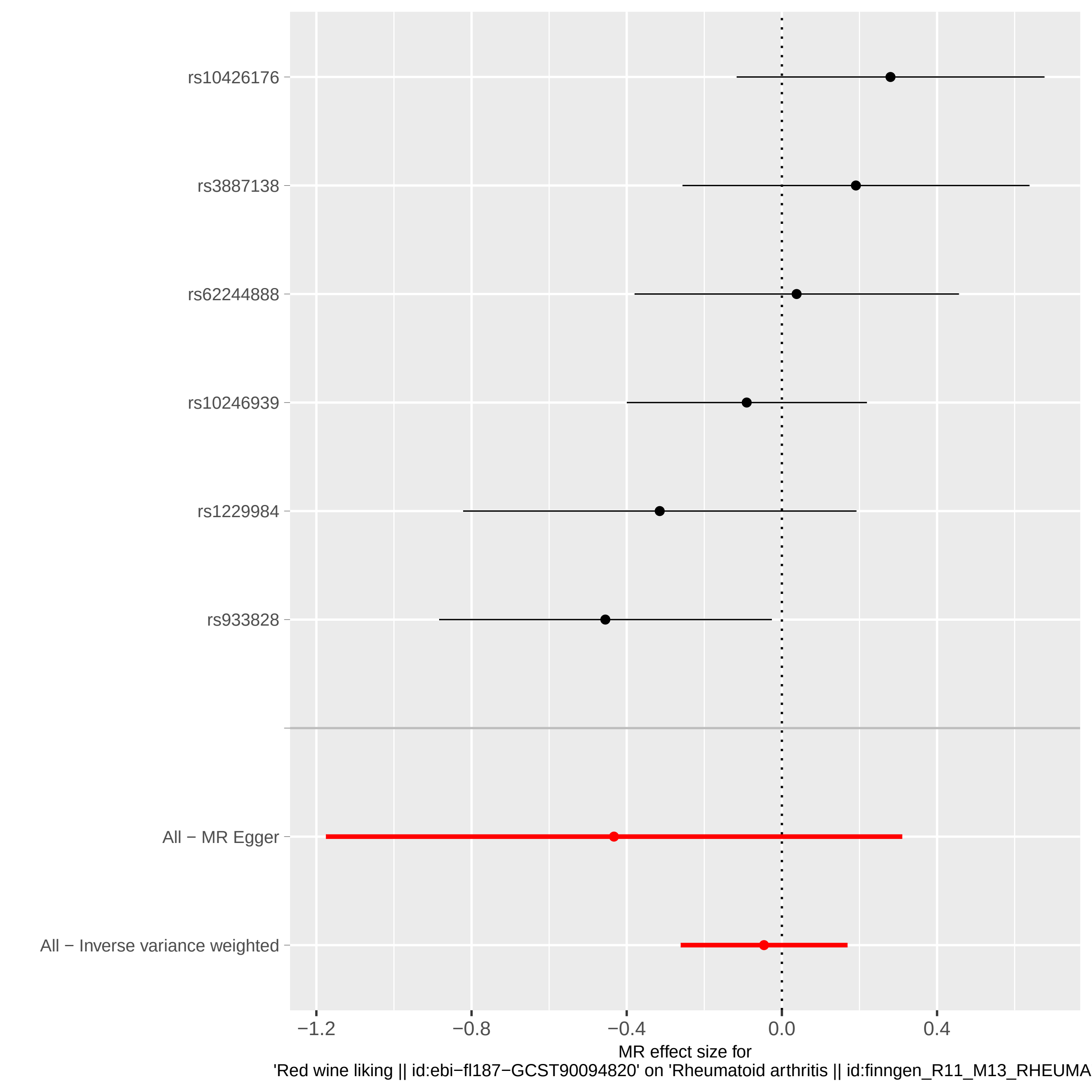
rs10426176
rs3887138
rs62244888
rs10246939
rs1229984
rs933828
All − MR Egger
All − Inverse variance weighted
−1.2
−0.8
−0.4
0.0
0.4
MR effect size for
'Red wine liking || id:ebi−fl187−GCST90094820' on 'Rheumatoid arthritis || id:finngen_R11_M13_RHEUMA

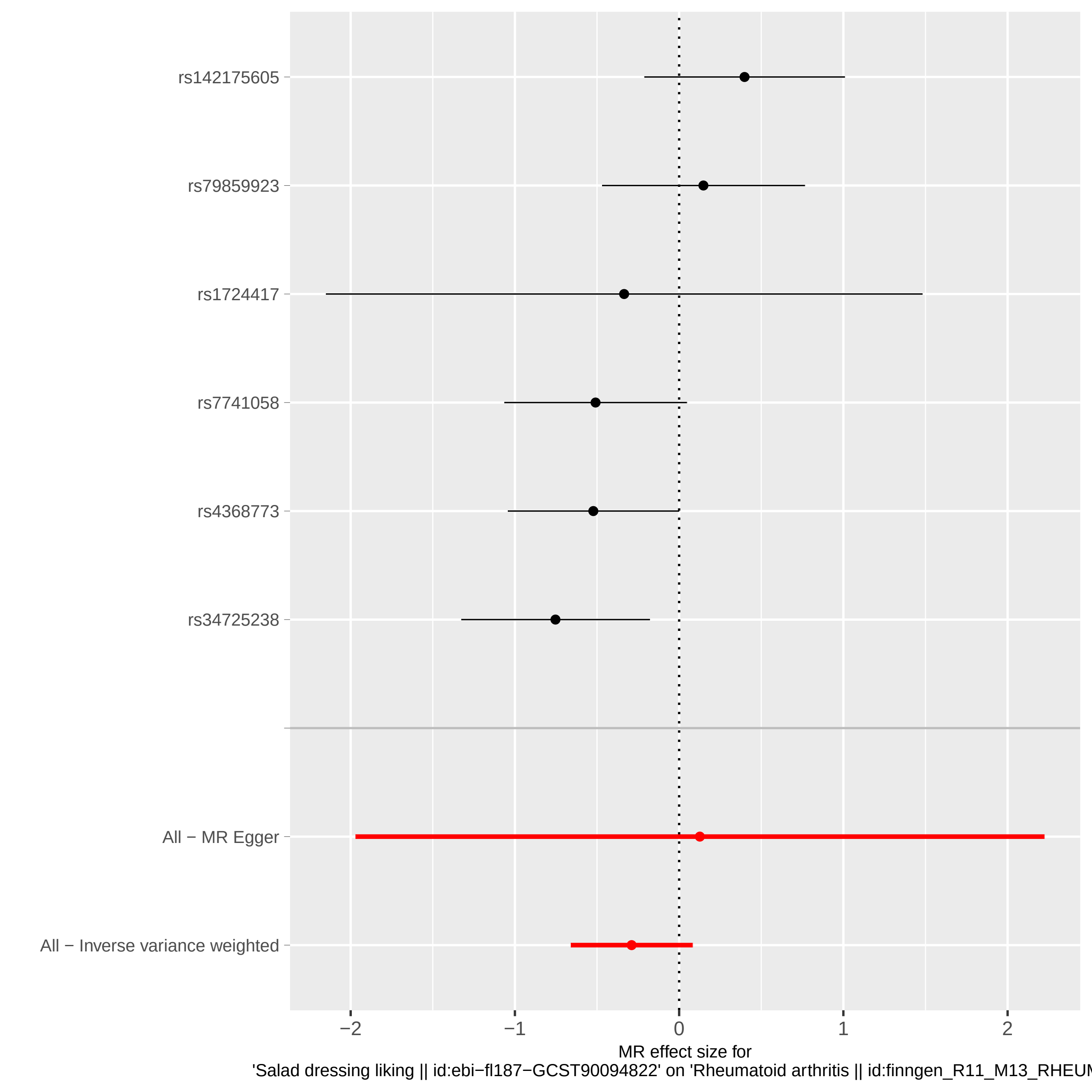
rs142175605
rs79859923
rs1724417
rs7741058
rs4368773
rs34725238
All − MR Egger
All − Inverse variance weighted
−2
−1
0
1
2
MR effect size for
'Salad dressing liking || id:ebi−fl187−GCST90094822' on 'Rheumatoid arthritis || id:finngen_R11_M13_RHEUM

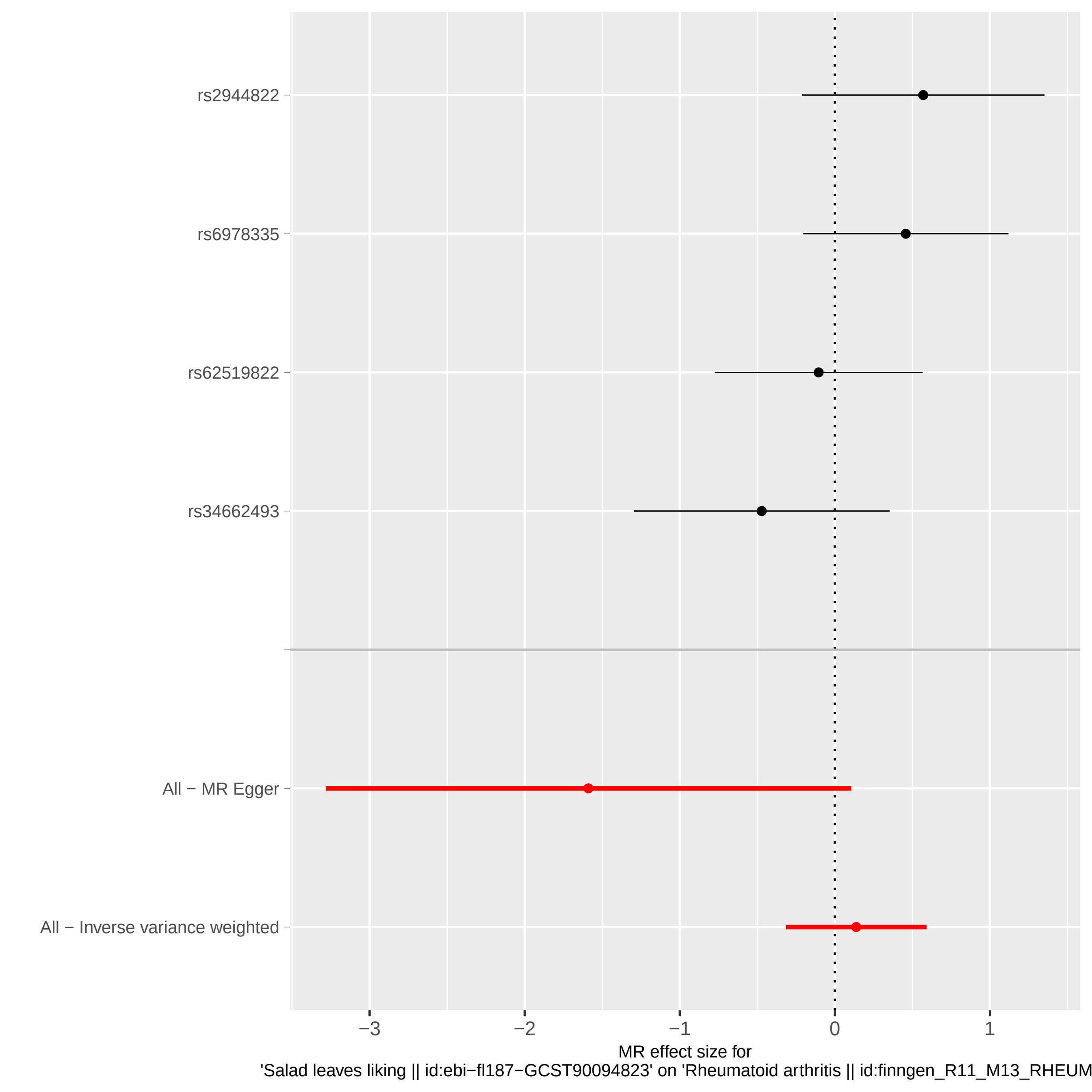
rs2944822
rs6978335
rs62519822
rs34662493
All − MR Egger
All − Inverse variance weighted
−3
−2
−1
0
1
MR effect size for
'Salad leaves liking || id:ebi−fl187−GCST90094823' on 'Rheumatoid arthritis || id:finngen_R11_M13_RHEUM

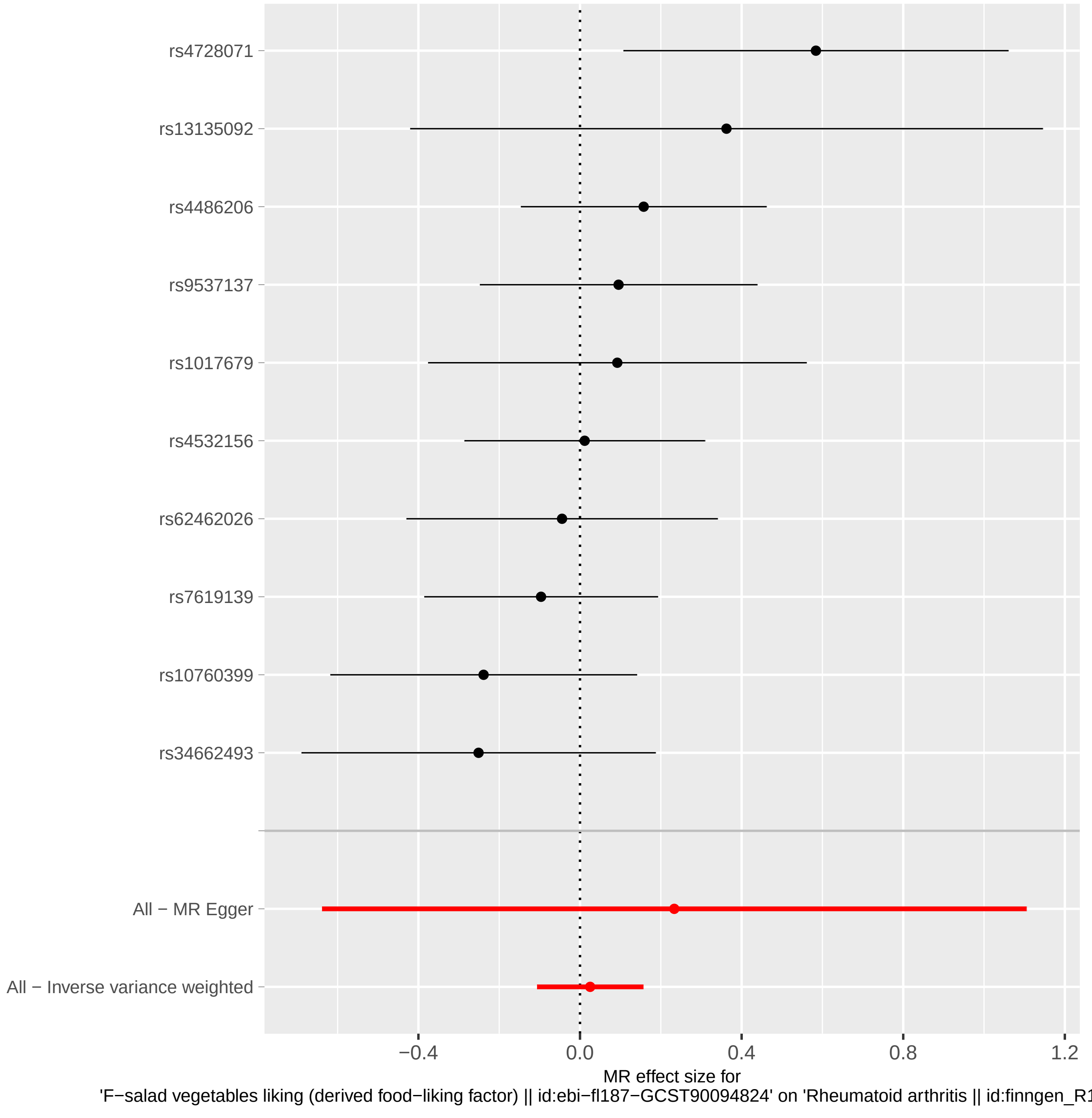
rs4728071
rs13135092
rs4486206
rs9537137
rs1017679
rs4532156
rs62462026
rs7619139
rs10760399
rs34662493
All − MR Egger
All − Inverse variance weighted
−0.4
0.0
0.4
0.8
1.2
MR effect size for
'F−salad vegetables liking (derived food−liking factor) || id:ebi−fl187−GCST90094824' on 'Rheumatoid arthritis || id:finngen_R1

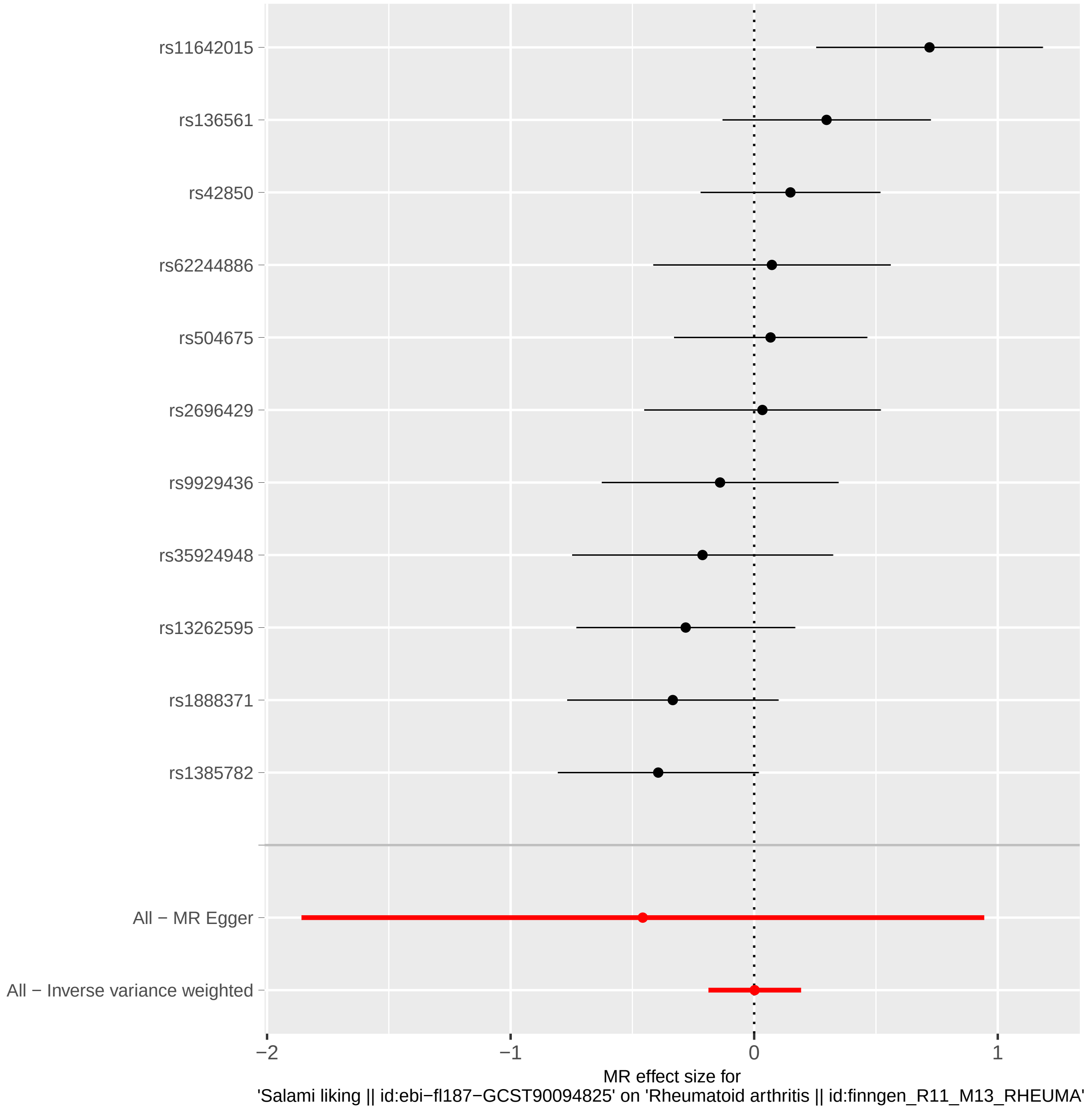
rs11642015
rs136561
rs42850
rs62244886
rs504675
rs2696429
rs9929436
rs35924948
rs13262595
rs1888371
rs1385782
All − MR Egger
All − Inverse variance weighted
−2
−1
0
1
MR effect size for
'Salami liking || id:ebi−fl187−GCST90094825' on 'Rheumatoid arthritis || id:finngen_R11_M13_RHEUMA'

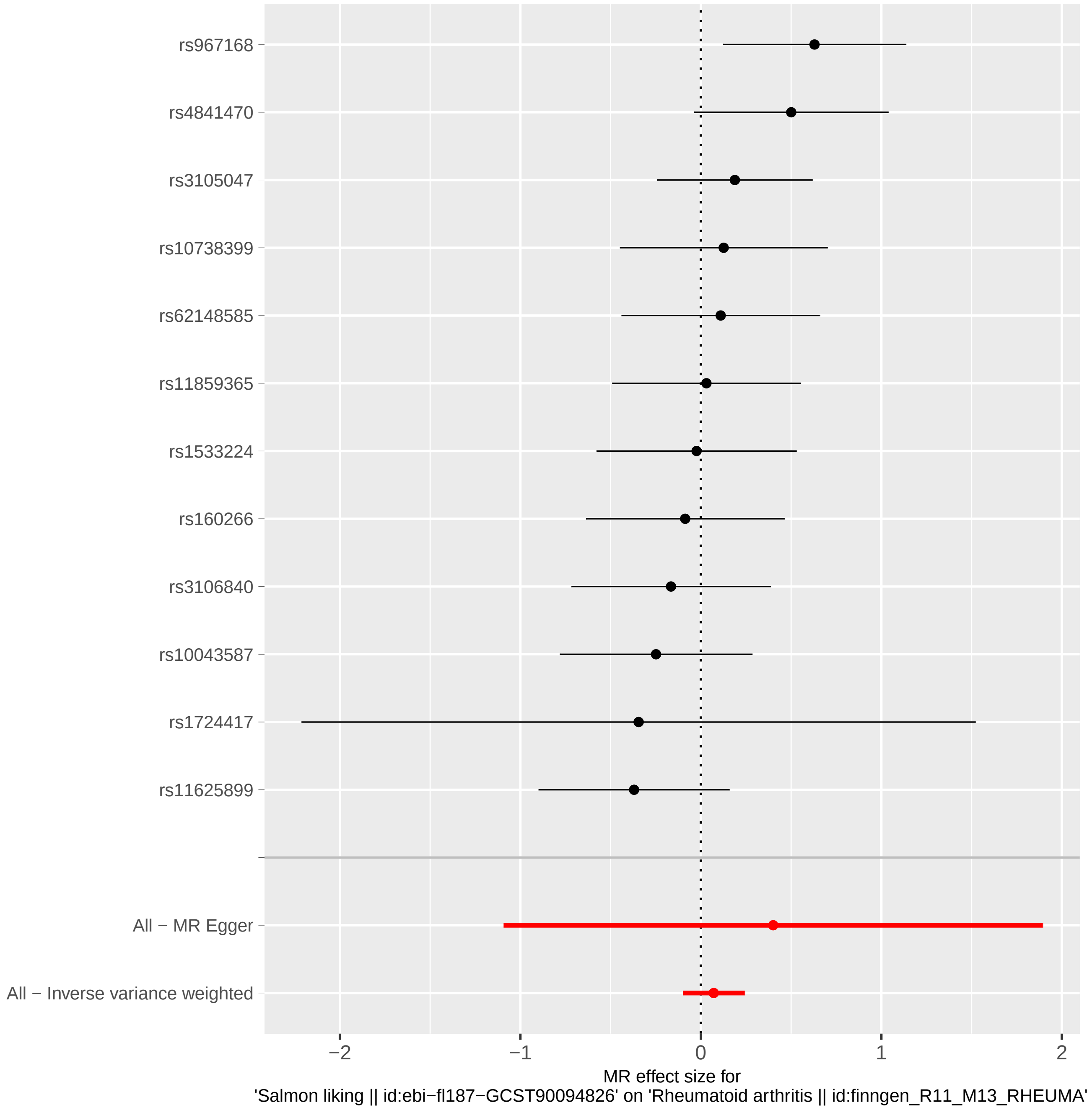
rs967168
rs4841470
rs3105047
rs10738399
rs62148585
rs11859365
rs1533224
rs160266
rs3106840
rs10043587
rs1724417
rs11625899
All − MR Egger
All − Inverse variance weighted
−2
−1
0
1
2
MR effect size for
'Salmon liking || id:ebi−fl187−GCST90094826' on 'Rheumatoid arthritis || id:finngen_R11_M13_RHEUMA'

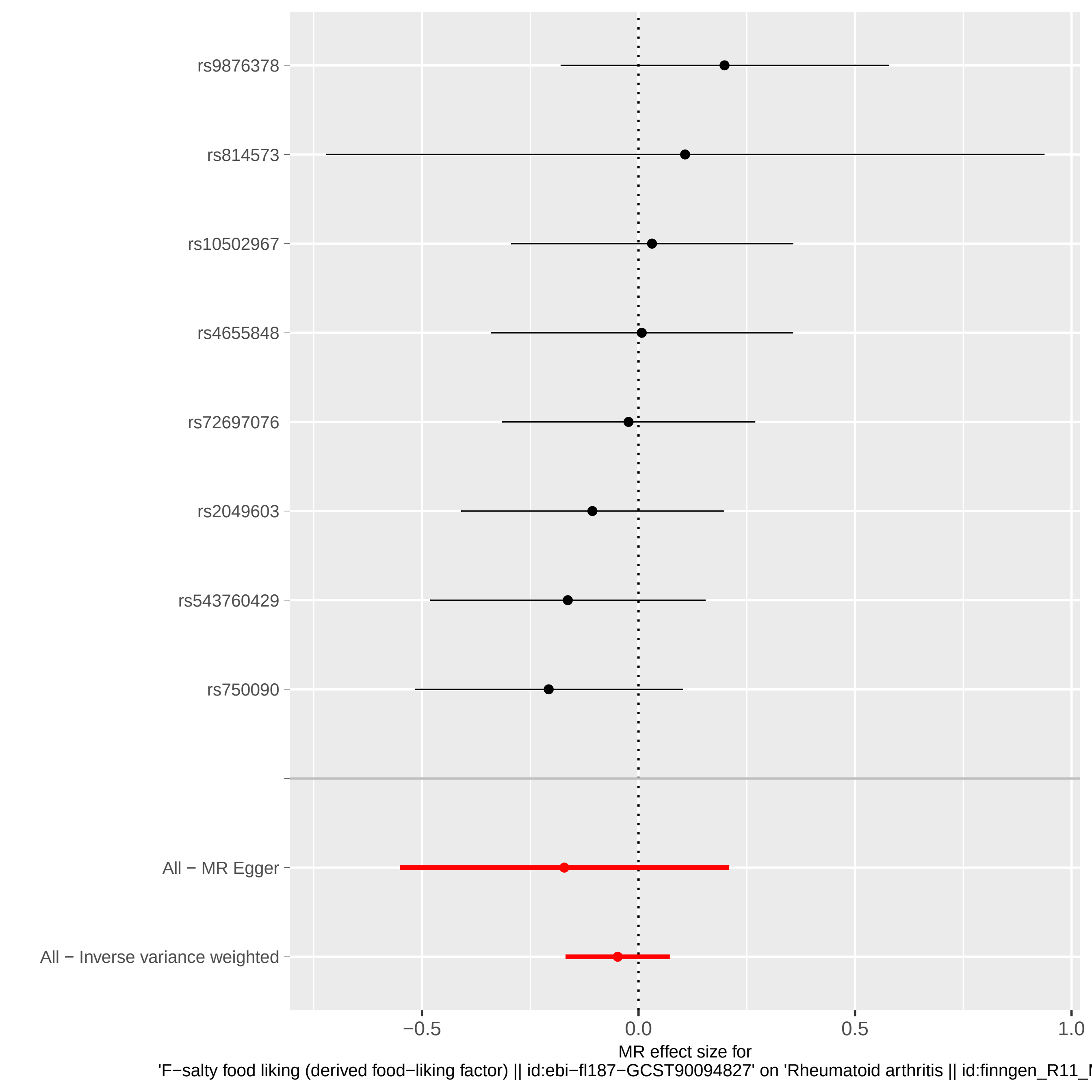

rs9876378
rs814573
rs10502967
rs4655848
rs72697076
rs2049603
rs543760429
rs750090
All − MR Egger
All − Inverse variance weighted
−0.5
0.0
0.5
1.0
MR effect size for
'F−salty food liking (derived food−liking factor) || id:ebi−fl187−GCST90094827' on 'Rheumatoid arthritis || id:finngen_R11_

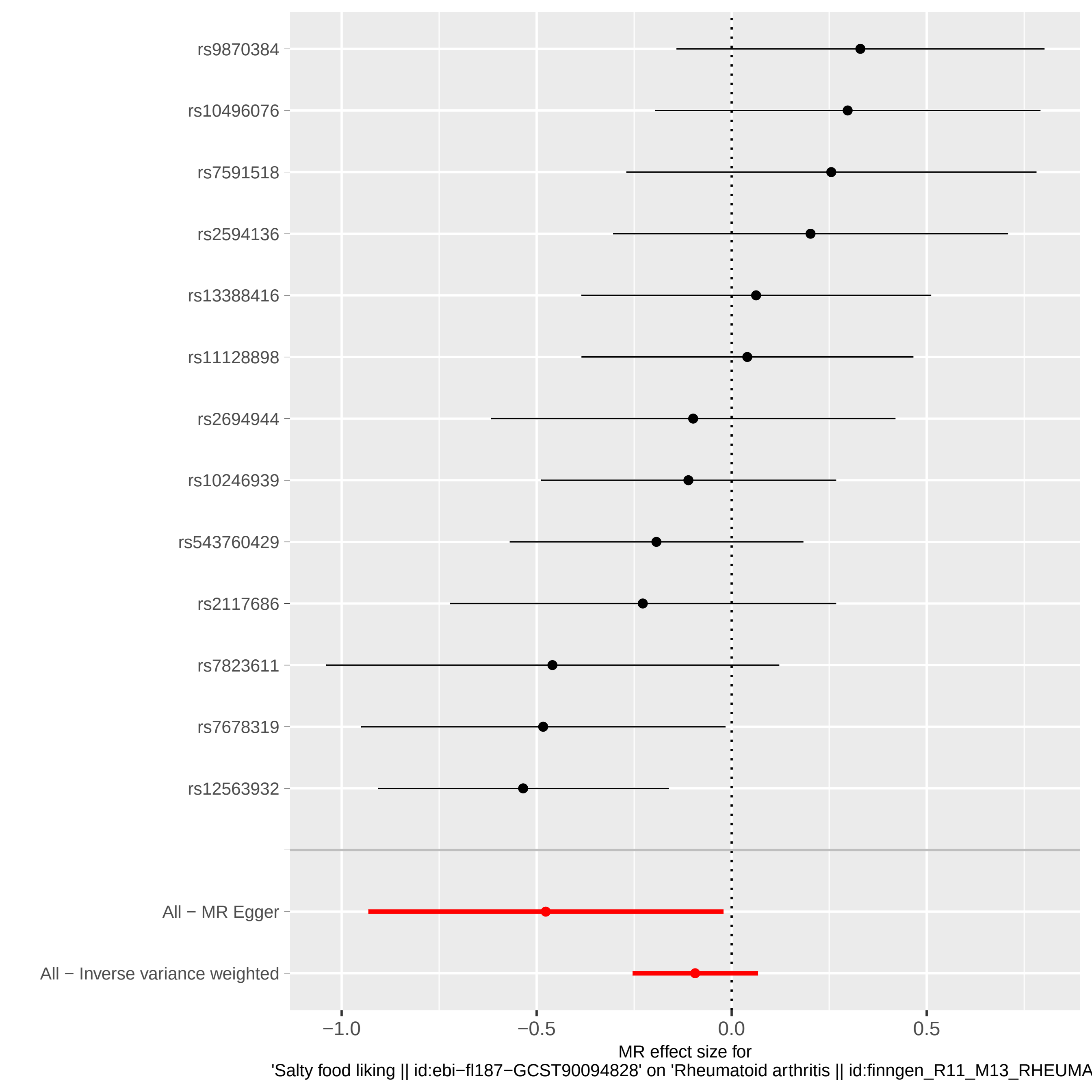

rs9870384
rs10496076
rs7591518
rs2594136
rs13388416
rs11128898
rs2694944
rs10246939
rs543760429
rs2117686
rs7823611
rs7678319
rs12563932
All − MR Egger
All − Inverse variance weighted
−1.0
−0.5
0.0
0.5
MR effect size for
'Salty food liking || id:ebi−fl187−GCST90094828' on 'Rheumatoid arthritis || id:finngen_R11_M13_RHEUMA

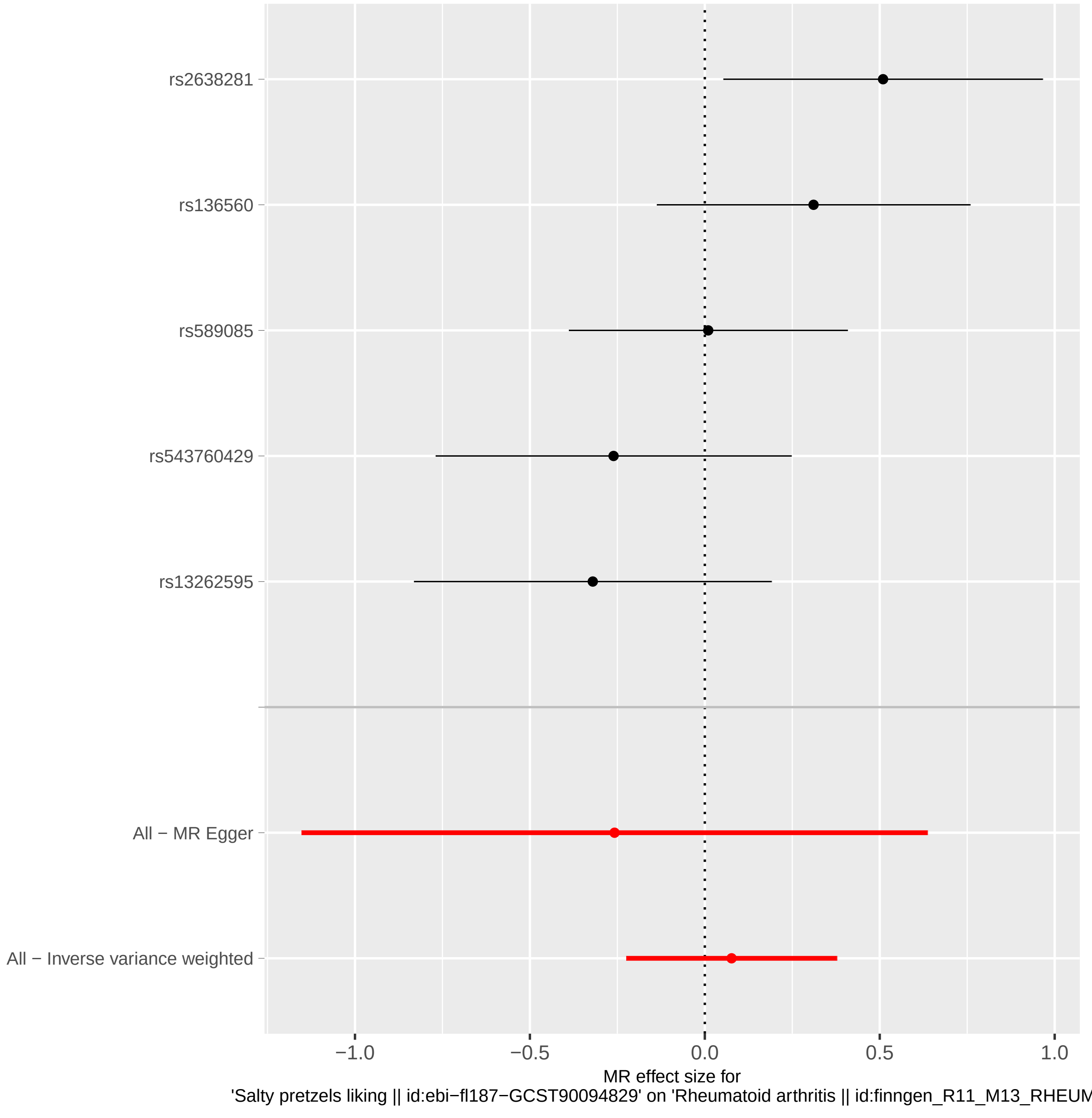
rs2638281
rs136560
rs589085
rs543760429
rs13262595
All − MR Egger
All − Inverse variance weighted
−1.0
−0.5
0.0
0.5
1.0
MR effect size for
'Salty pretzels liking || id:ebi−fl187−GCST90094829' on 'Rheumatoid arthritis || id:finngen_R11_M13_RHEUM

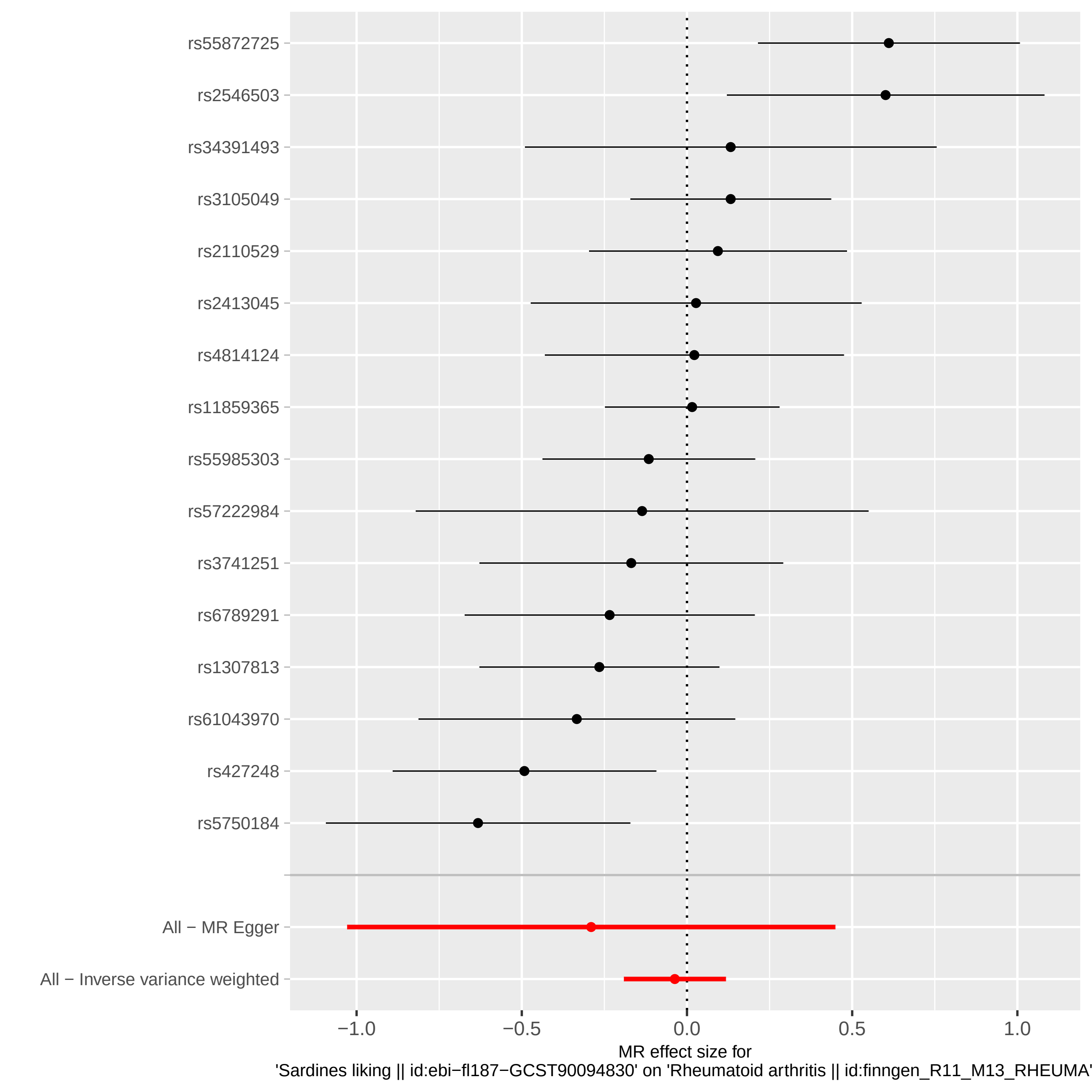
rs55872725
rs2546503
rs34391493
rs3105049
rs2110529
rs2413045
rs4814124
rs11859365
rs55985303
rs57222984
rs3741251
rs6789291
rs1307813
rs61043970
rs427248
rs5750184
All − MR Egger
All − Inverse variance weighted
−1.0
−0.5
0.0
0.5
1.0
MR effect size for
'Sardines liking || id:ebi−fl187−GCST90094830' on 'Rheumatoid arthritis || id:finngen_R11_M13_RHEUMA'

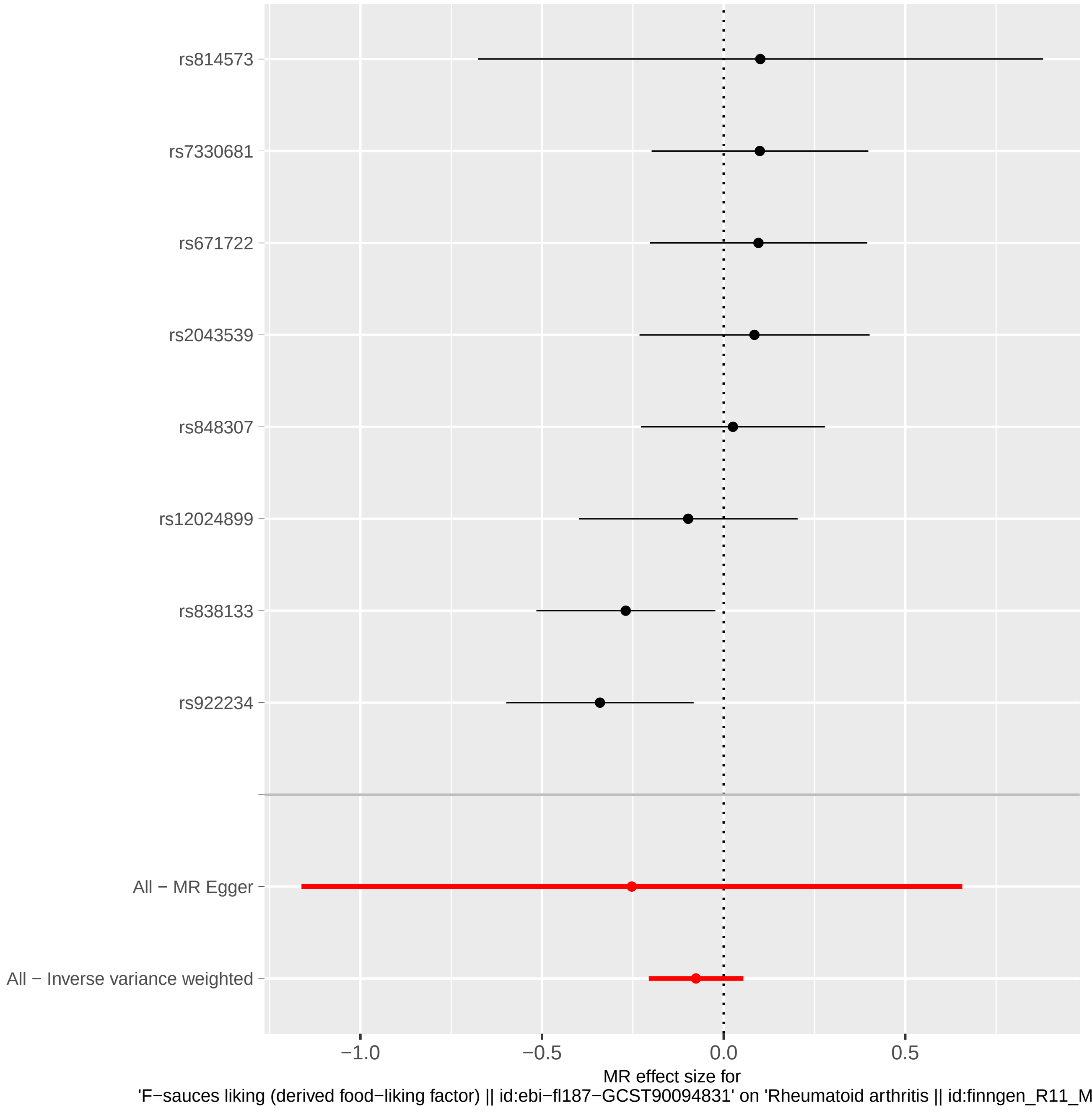
rs814573
rs7330681
rs671722
rs2043539
rs848307
rs12024899
rs838133
rs922234
All − MR Egger
All − Inverse variance weighted
−1.0
−0.5
0.0
0.5
MR effect size for
'F−sauces liking (derived food−liking factor) || id:ebi−fl187−GCST90094831' on 'Rheumatoid arthritis || id:finngen_R11_M

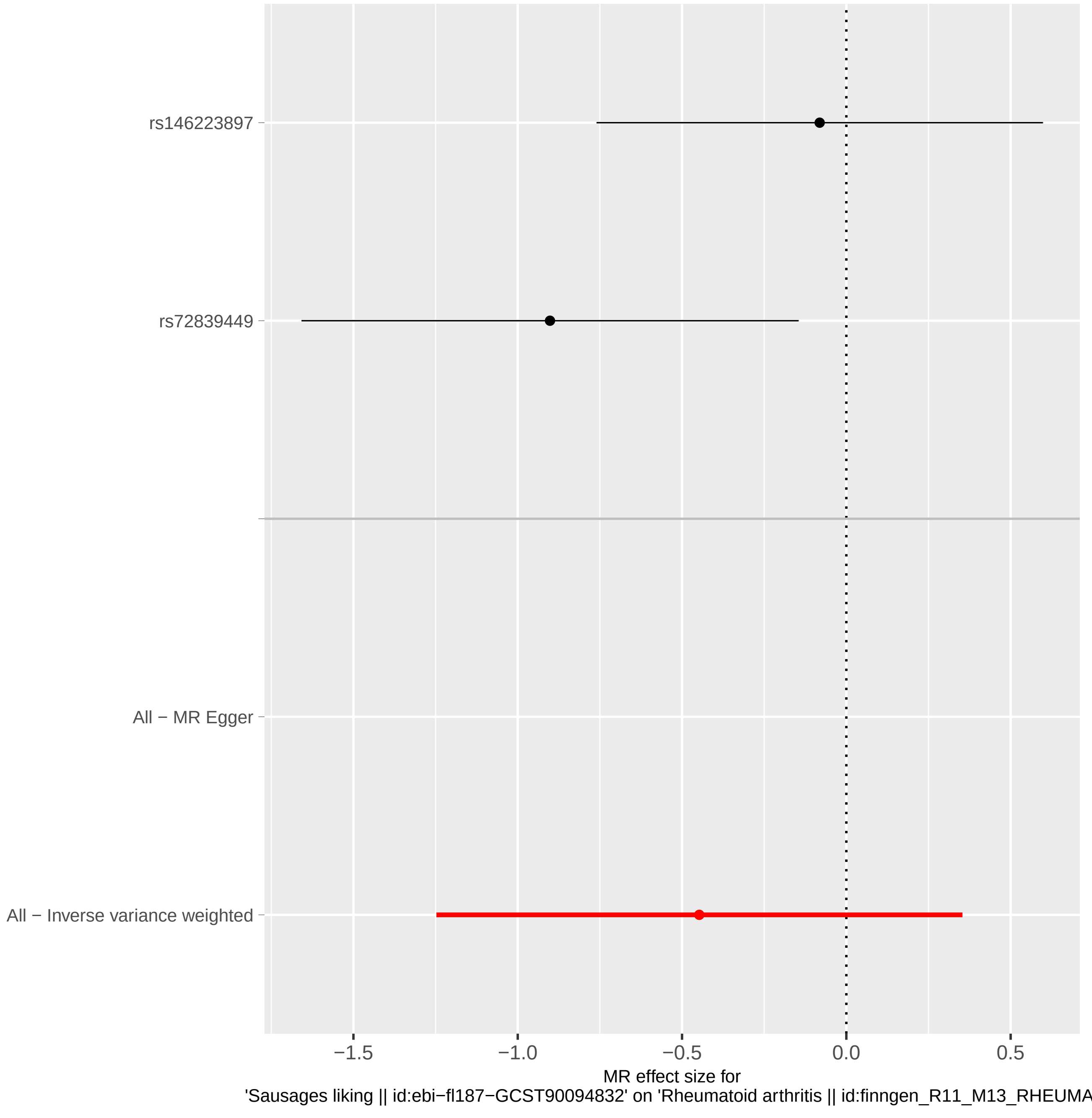

rs146223897
rs72839449
All − MR Egger
All − Inverse variance weighted
−1.5
−1.0
−0.5
0.0
0.5
MR effect size for
'Sausages liking || id:ebi−fl187−GCST90094832' on 'Rheumatoid arthritis || id:finngen_R11_M13_RHEUMA

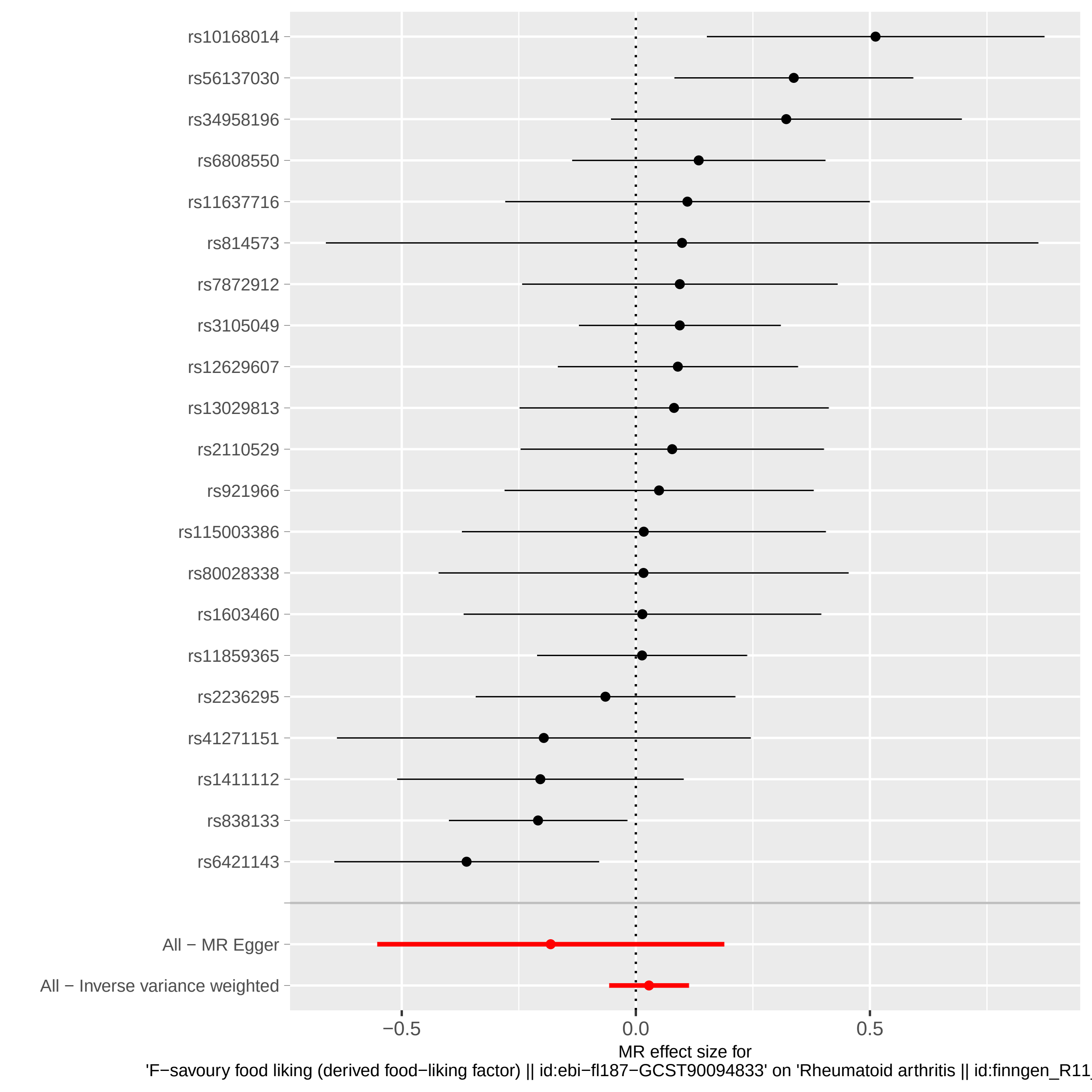

rs10168014
rs56137030
rs34958196
rs6808550
rs11637716
rs814573
rs7872912
rs3105049
rs12629607
rs13029813
rs2110529
rs921966
rs115003386
rs80028338
rs1603460
rs11859365
rs2236295
rs41271151
rs1411112
rs838133
rs6421143
All − MR Egger
All − Inverse variance weighted
−0.5
0.0
0.5
MR effect size for
'F−savoury food liking (derived food−liking factor) || id:ebi−fl187−GCST90094833' on 'Rheumatoid arthritis || id:finngen_R11_

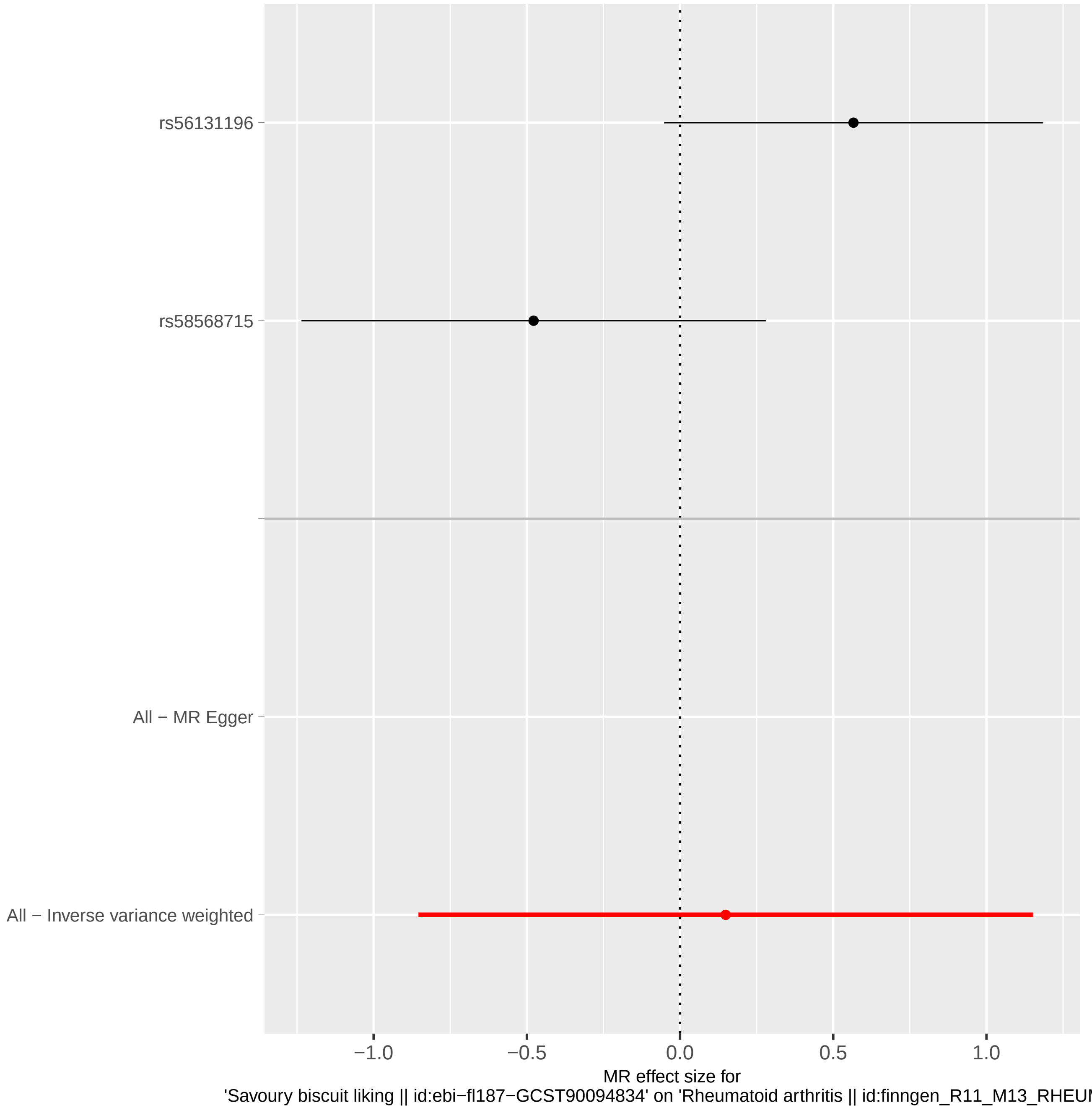
rs56131196
rs58568715
All − MR Egger
All − Inverse variance weighted
−1.0
−0.5
0.0
0.5
1.0
MR effect size for
'Savoury biscuit liking || id:ebi−fl187−GCST90094834' on 'Rheumatoid arthritis || id:finngen_R11_M13_RHEU

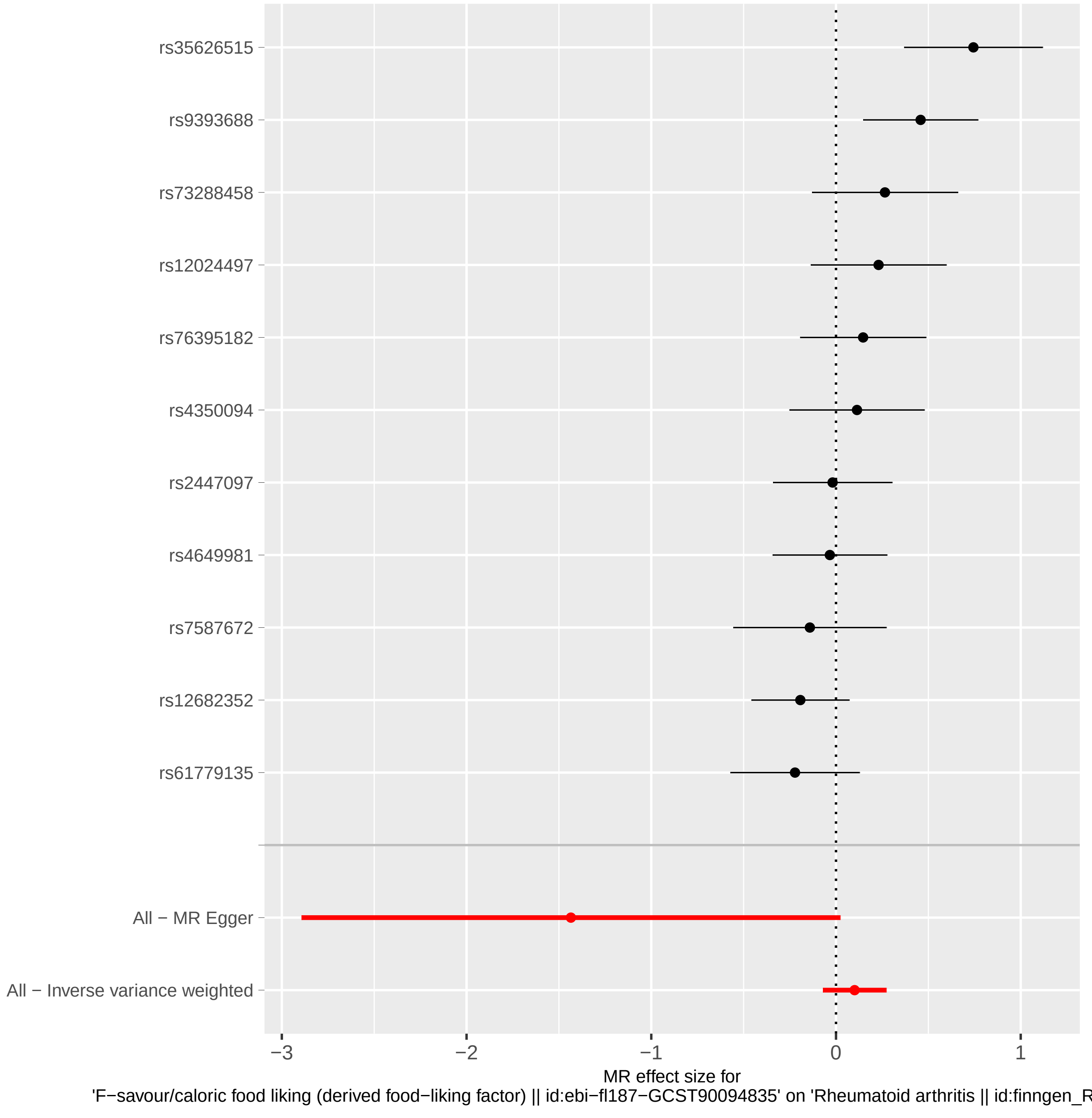
rs35626515
rs9393688
rs73288458
rs12024497
rs76395182
rs4350094
rs2447097
rs4649981
rs7587672
rs12682352
rs61779135
All − MR Egger
All − Inverse variance weighted
−3
−2
−1
0
1
MR effect size for
'F−savour/caloric food liking (derived food−liking factor) || id:ebi−fl187−GCST90094835' on 'Rheumatoid arthritis || id:finngen_R

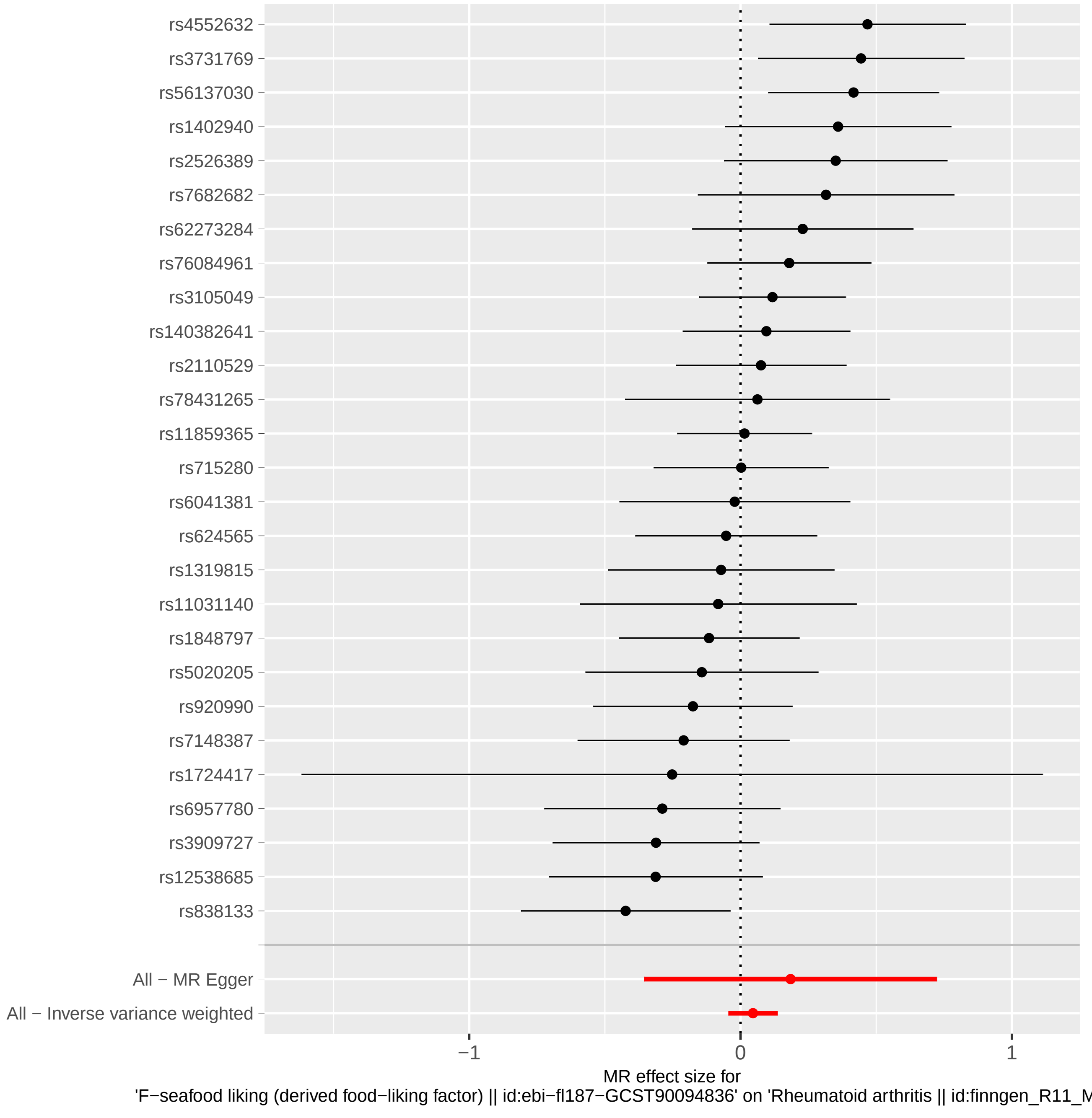
rs4552632
rs3731769
rs56137030
rs1402940
rs2526389
rs7682682
rs62273284
rs76084961
rs3105049
rs140382641
rs2110529
rs78431265
rs11859365
rs715280
rs6041381
rs624565
rs1319815
rs11031140
rs1848797
rs5020205
rs920990
rs7148387
rs1724417
rs6957780
rs3909727
rs12538685
rs838133
All − MR Egger
All − Inverse variance weighted
−1
0
1
MR effect size for
'F−seafood liking (derived food−liking factor) || id:ebi−fl187−GCST90094836' on 'Rheumatoid arthritis || id:finngen_R11_M

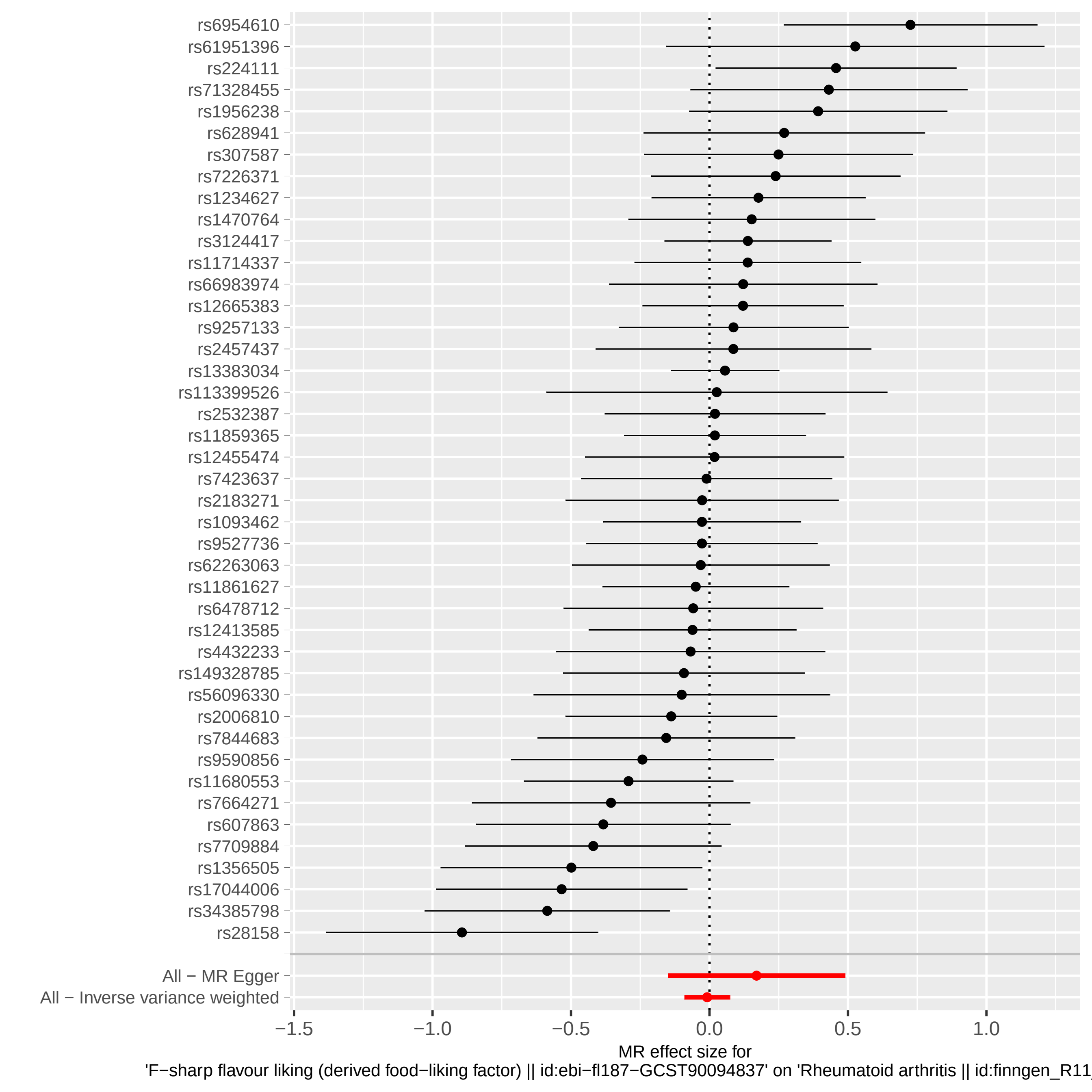
rs6954610
rs61951396
rs224111
rs71328455
rs1956238
rs628941
rs307587
rs7226371
rs1234627
rs1470764
rs3124417
rs11714337
rs66983974
rs12665383
rs9257133
rs2457437
rs13383034
rs113399526
rs2532387
rs11859365
rs12455474
rs7423637
rs2183271
rs1093462
rs9527736
rs62263063
rs11861627
rs6478712
rs12413585
rs4432233
rs149328785
rs56096330
rs2006810
rs7844683
rs9590856
rs11680553
rs7664271
rs607863
rs7709884
rs1356505
rs17044006
rs34385798
rs28158
All − MR Egger
All − Inverse variance weighted
−1.5
−1.0
−0.5
0.0
0.5
1.0
MR effect size for
'F−sharp flavour liking (derived food−liking factor) || id:ebi−fl187−GCST90094837' on 'Rheumatoid arthritis || id:finngen_R11_

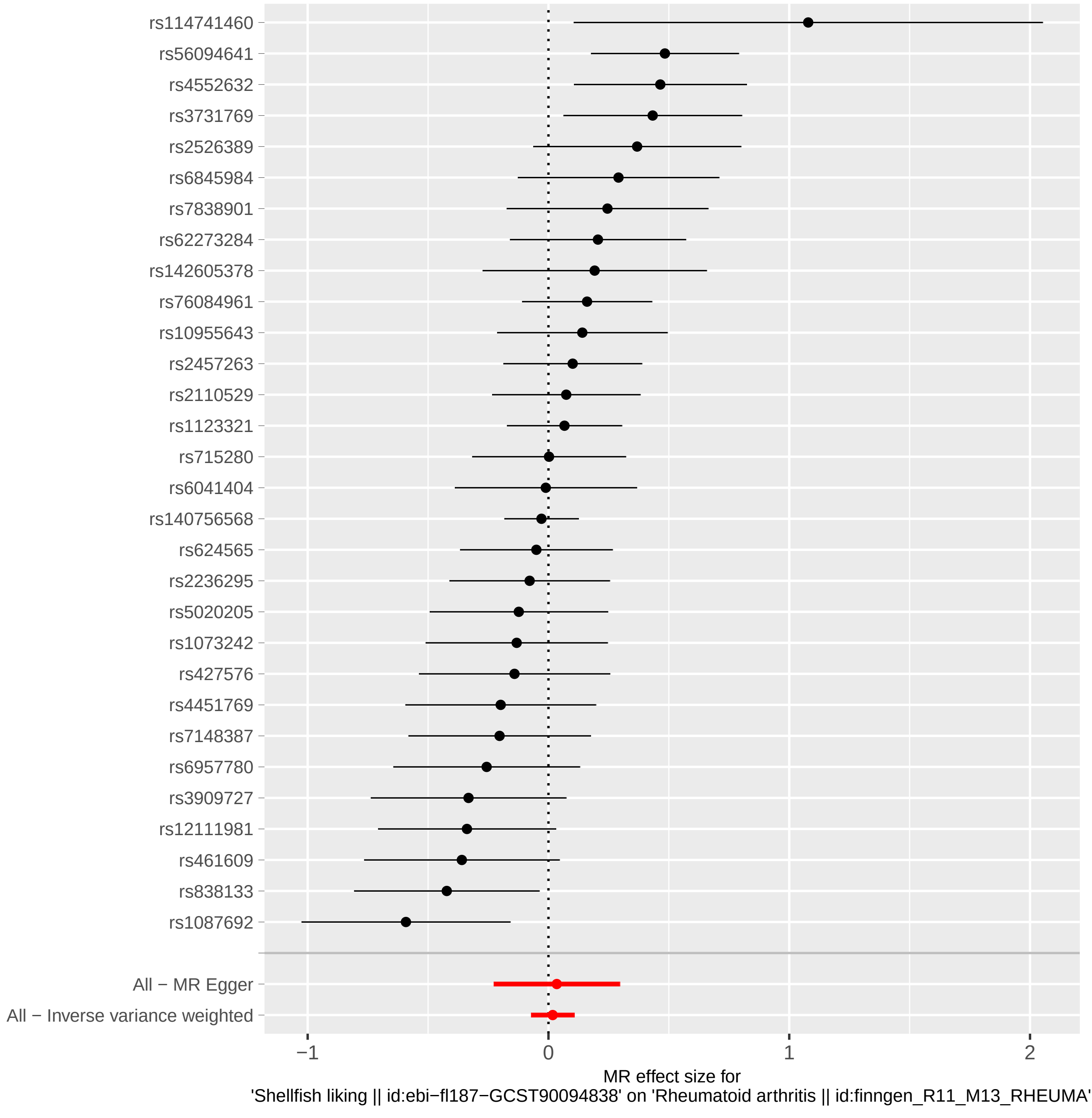
rs114741460
rs56094641
rs4552632
rs3731769
rs2526389
rs6845984
rs7838901
rs62273284
rs142605378
rs76084961
rs10955643
rs2457263
rs2110529
rs1123321
rs715280
rs6041404
rs140756568
rs624565
rs2236295
rs5020205
rs1073242
rs427576
rs4451769
rs7148387
rs6957780
rs3909727
rs12111981
rs461609
rs838133
rs1087692
All − MR Egger
All − Inverse variance weighted
−1
0
1
2
MR effect size for
'Shellfish liking || id:ebi−fl187−GCST90094838' on 'Rheumatoid arthritis || id:finngen_R11_M13_RHEUMA'

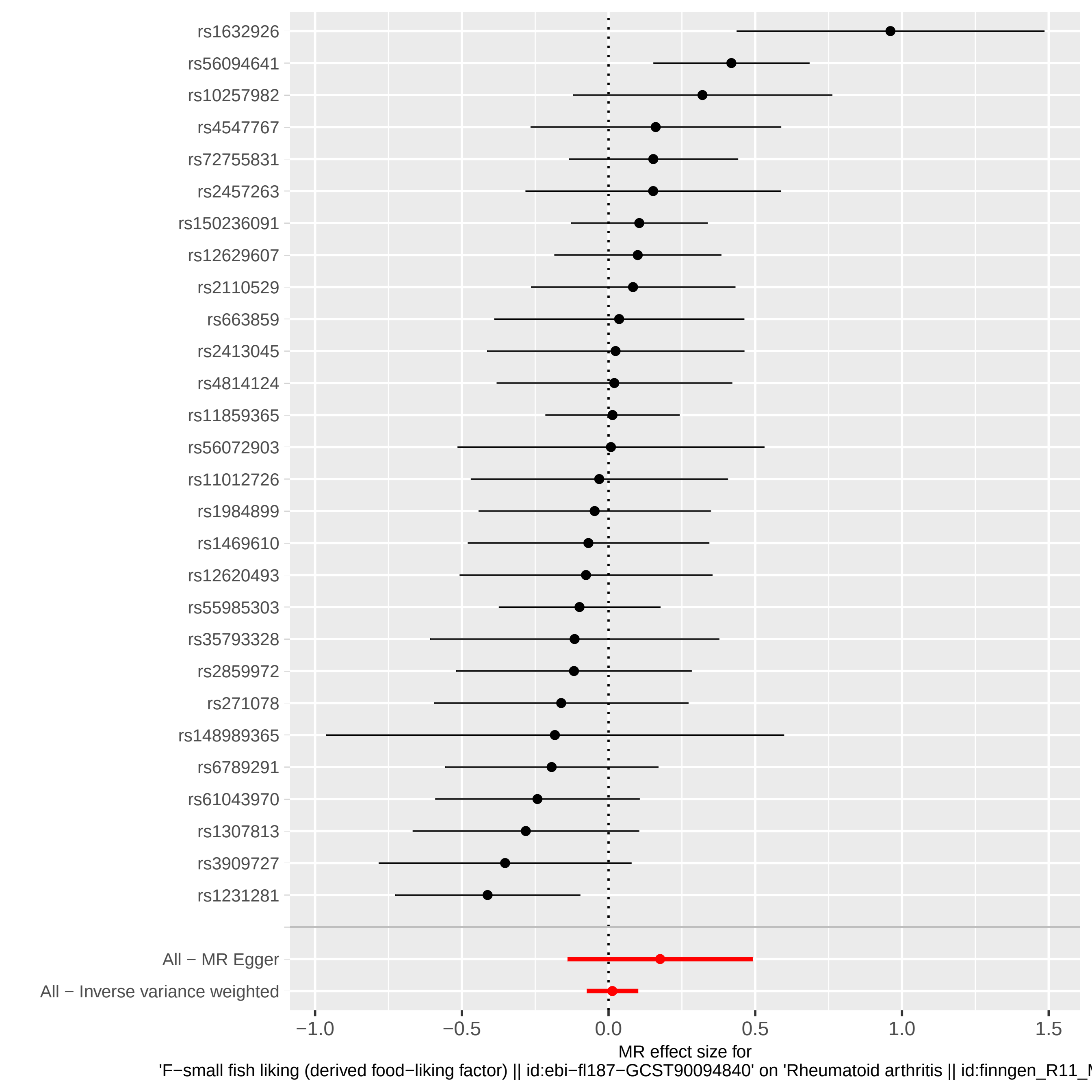

rs1632926
rs56094641
rs10257982
rs4547767
rs72755831
rs2457263
rs150236091
rs12629607
rs2110529
rs663859
rs2413045
rs4814124
rs11859365
rs56072903
rs11012726
rs1984899
rs1469610
rs12620493
rs55985303
rs35793328
rs2859972
rs271078
rs148989365
rs6789291
rs61043970
rs1307813
rs3909727
rs1231281
All − MR Egger
All − Inverse variance weighted
−1.0
−0.5
0.0
0.5
1.0
1.5
MR effect size for
'F−small fish liking (derived food−liking factor) || id:ebi−fl187−GCST90094840' on 'Rheumatoid arthritis || id:finngen_R11_M

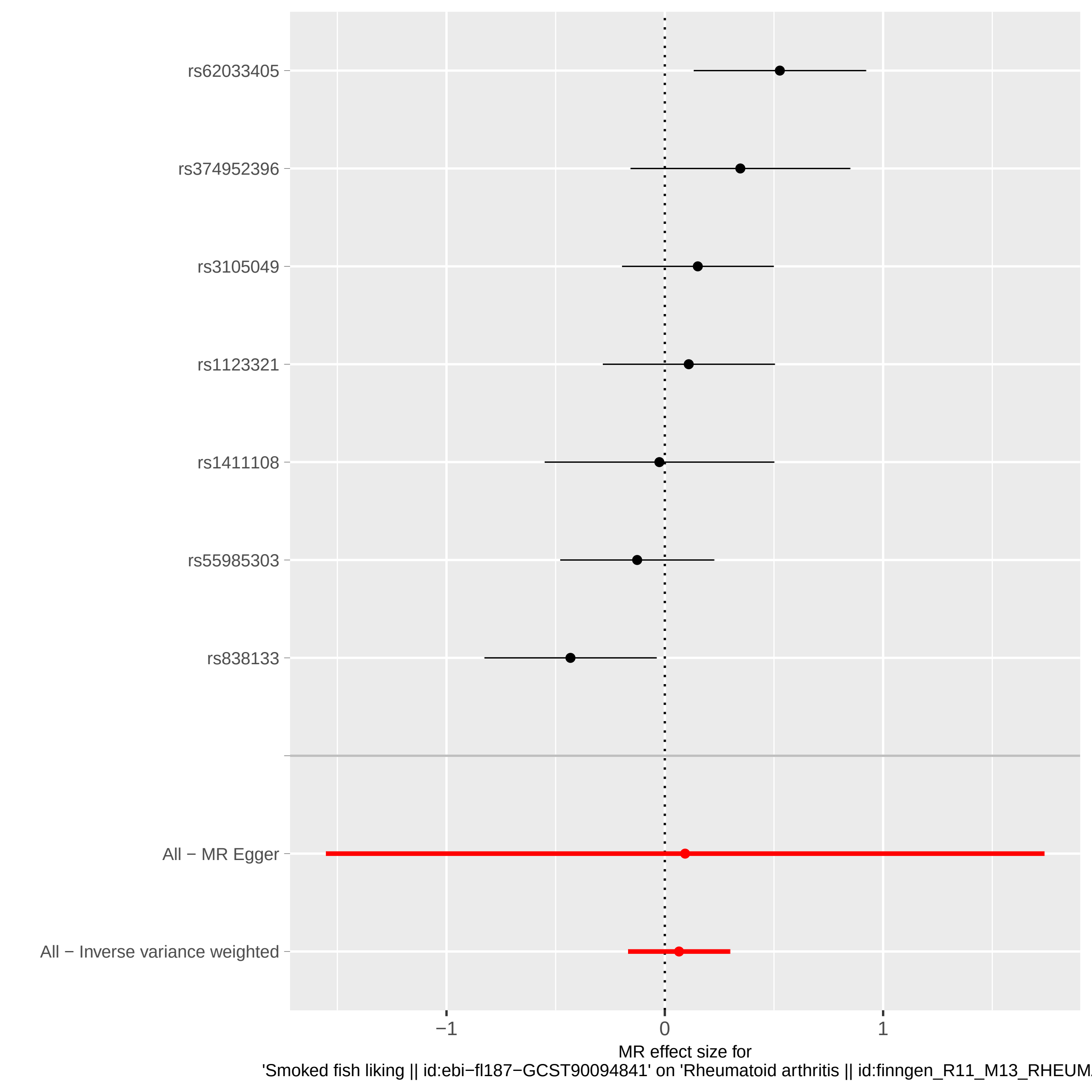
rs62033405
rs374952396
rs3105049
rs1123321
rs1411108
rs55985303
rs838133
All − MR Egger
All − Inverse variance weighted
−1
0
1
MR effect size for
'Smoked fish liking || id:ebi−fl187−GCST90094841' on 'Rheumatoid arthritis || id:finngen_R11_M13_RHEUM

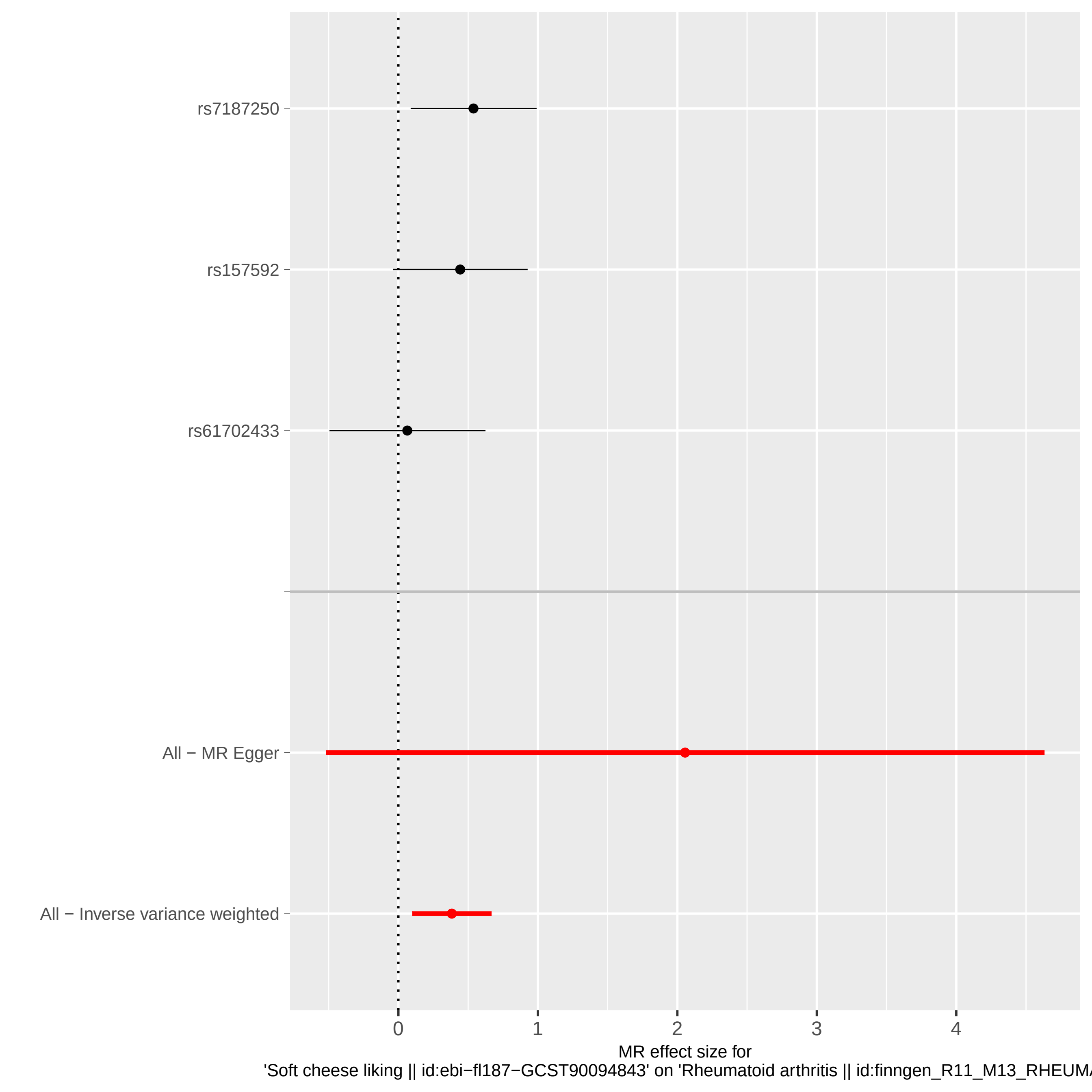
rs7187250
rs157592
rs61702433
All − MR Egger
All − Inverse variance weighted
0
1
2
3
4
MR effect size for
'Soft cheese liking || id:ebi−fl187−GCST90094843' on 'Rheumatoid arthritis || id:finngen_R11_M13_RHEUM

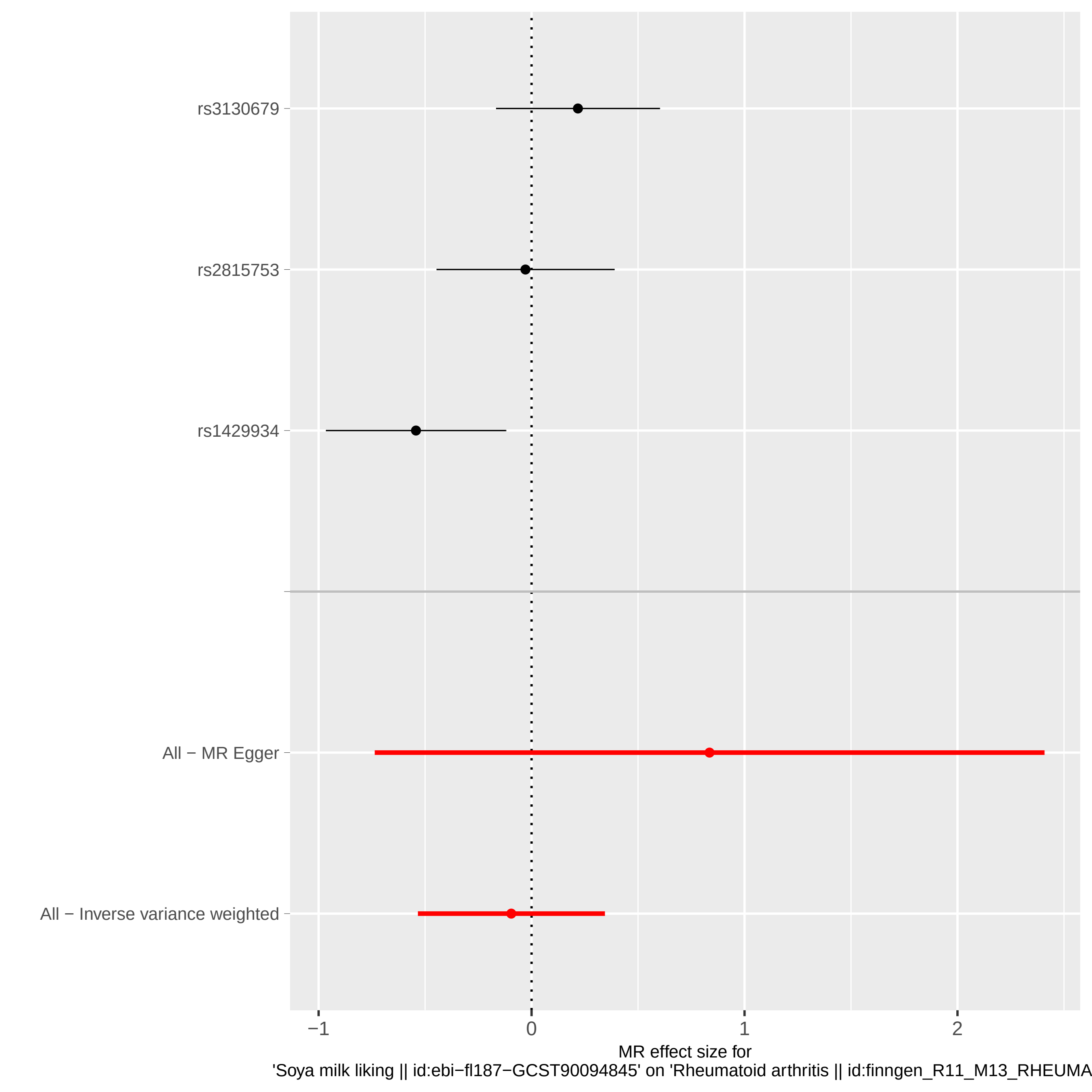
rs3130679
rs2815753
rs1429934
All − MR Egger
All − Inverse variance weighted
−1
0
1
2
MR effect size for
'Soya milk liking || id:ebi−fl187−GCST90094845' on 'Rheumatoid arthritis || id:finngen_R11_M13_RHEUMA

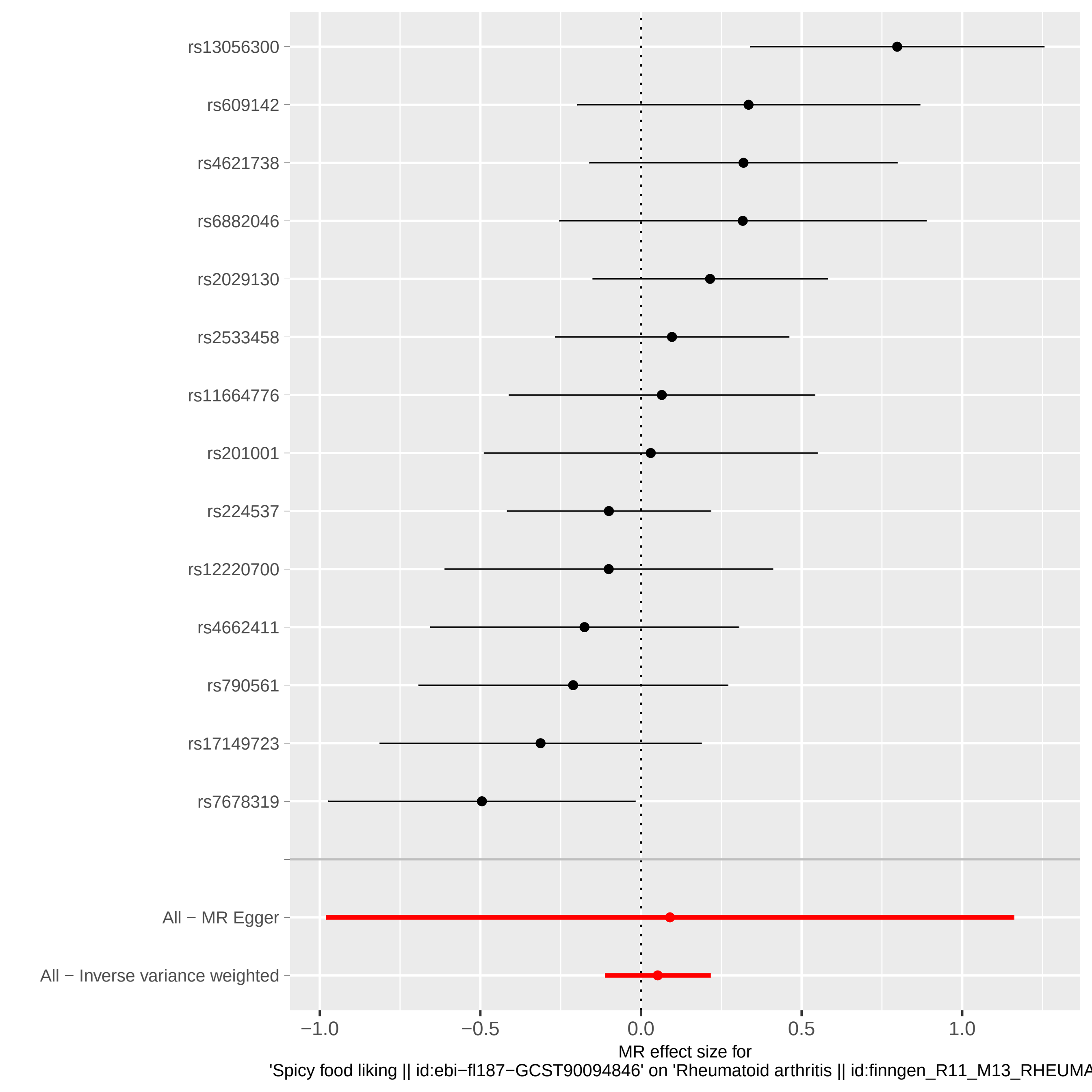

rs13056300
rs609142
rs4621738
rs6882046
rs2029130
rs2533458
rs11664776
rs201001
rs224537
rs12220700
rs4662411
rs790561
rs17149723
rs7678319
All − MR Egger
All − Inverse variance weighted
−1.0
−0.5
0.0
0.5
1.0
MR effect size for
'Spicy food liking || id:ebi−fl187−GCST90094846' on 'Rheumatoid arthritis || id:finngen_R11_M13_RHEUMA

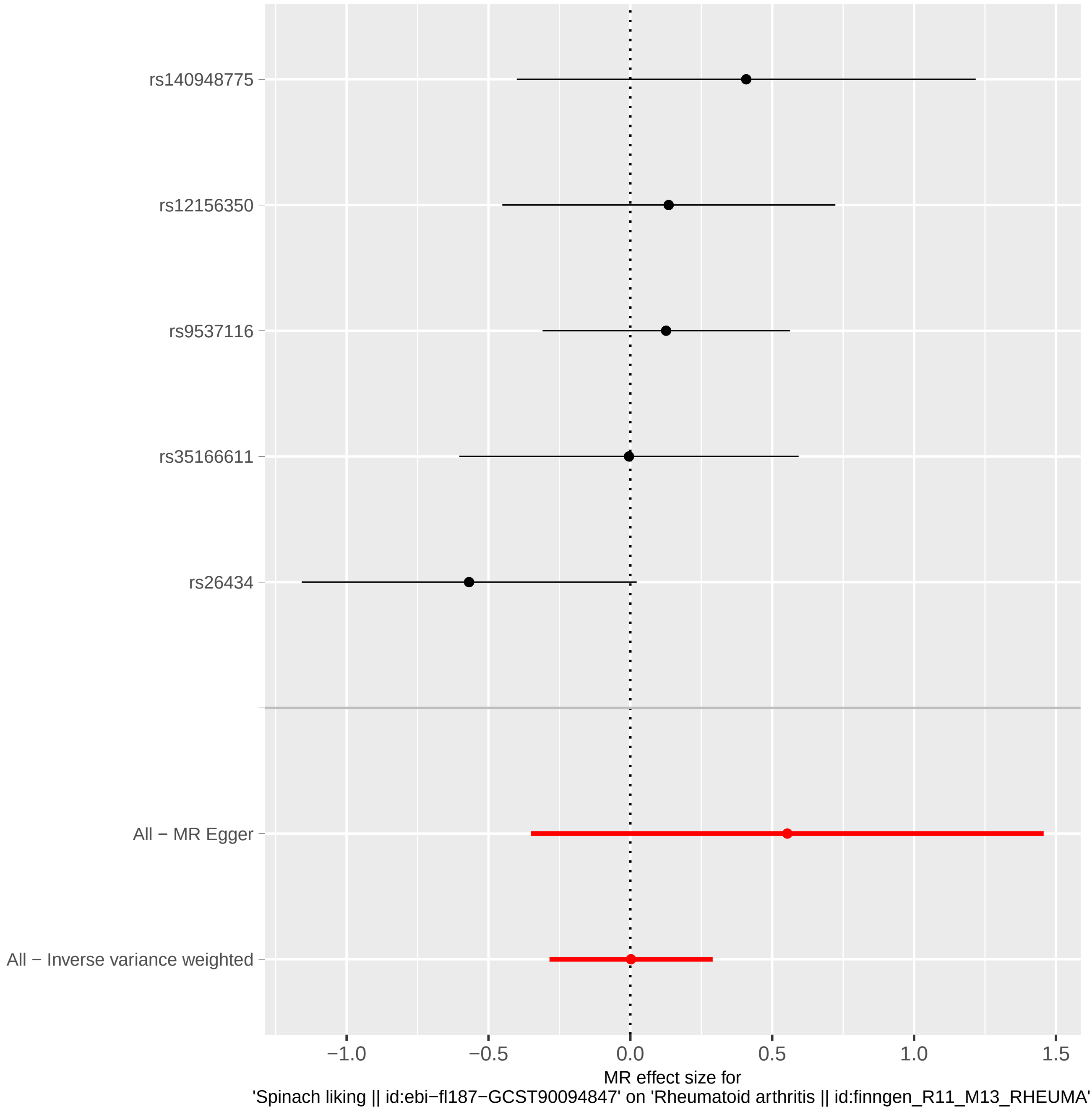
rs140948775
rs12156350
rs9537116
rs35166611
rs26434
All − MR Egger
All − Inverse variance weighted
−1.0
−0.5
0.0
0.5
1.0
1.5
MR effect size for
'Spinach liking || id:ebi−fl187−GCST90094847' on 'Rheumatoid arthritis || id:finngen_R11_M13_RHEUMA'

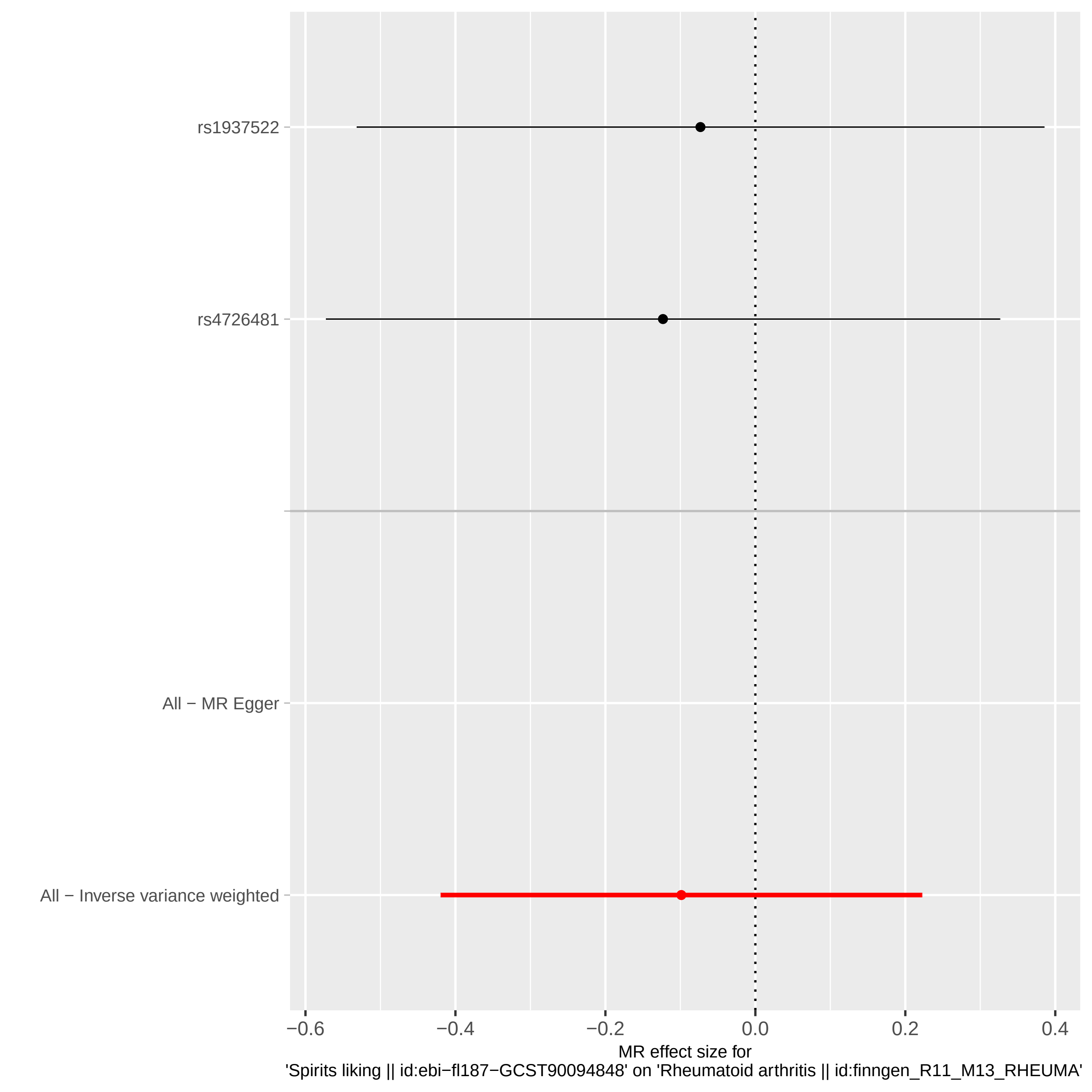
rs1937522
rs4726481
All − MR Egger
All − Inverse variance weighted
−0.6
−0.4
−0.2
0.0
0.2
0.4
MR effect size for
'Spirits liking || id:ebi−fl187−GCST90094848' on 'Rheumatoid arthritis || id:finngen_R11_M13_RHEUMA'

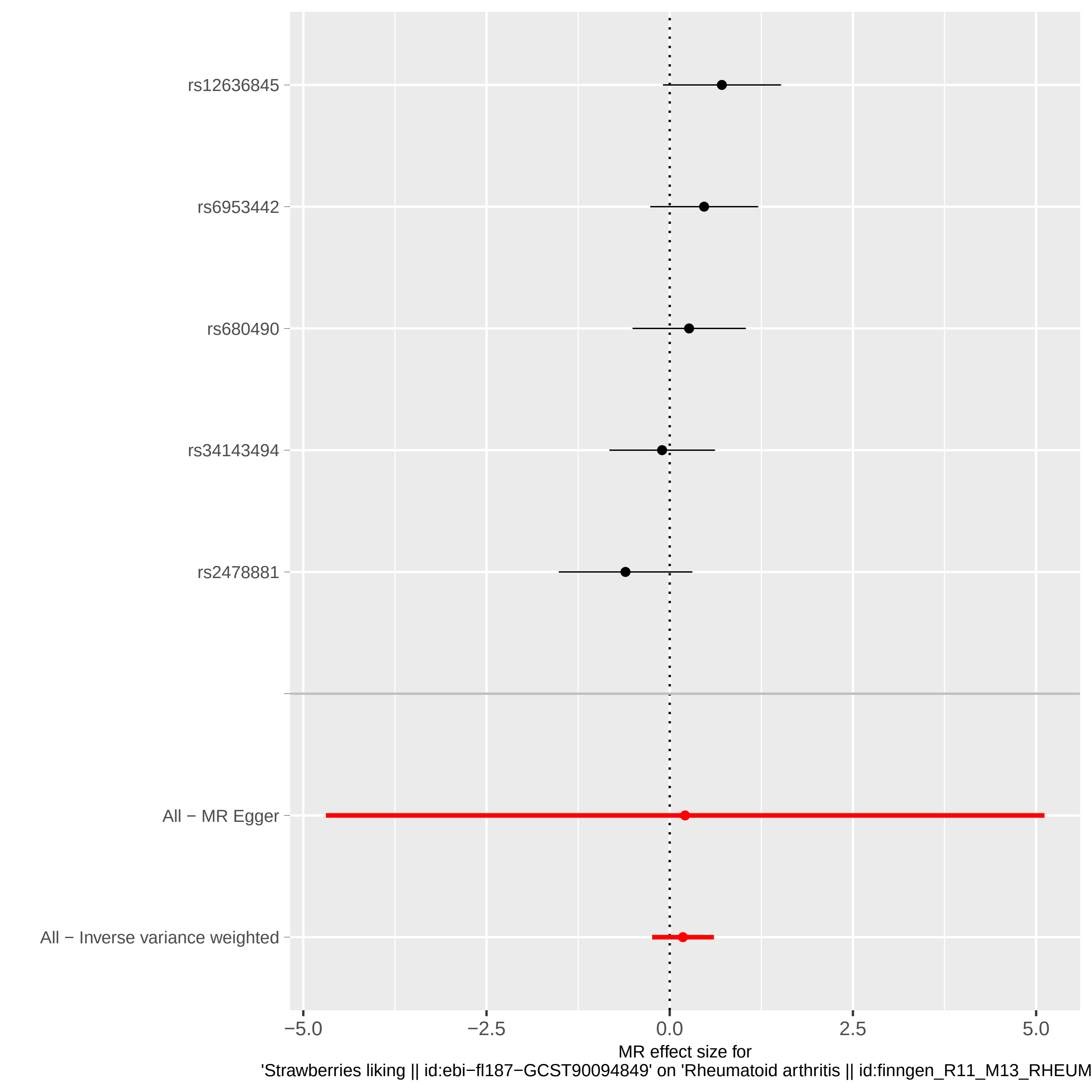
rs12636845
rs6953442
rs680490
rs34143494
rs2478881
All − MR Egger
All − Inverse variance weighted
−5.0
−2.5
0.0
2.5
5.0
MR effect size for
'Strawberries liking || id:ebi−fl187−GCST90094849' on 'Rheumatoid arthritis || id:finngen_R11_M13_RHEUM

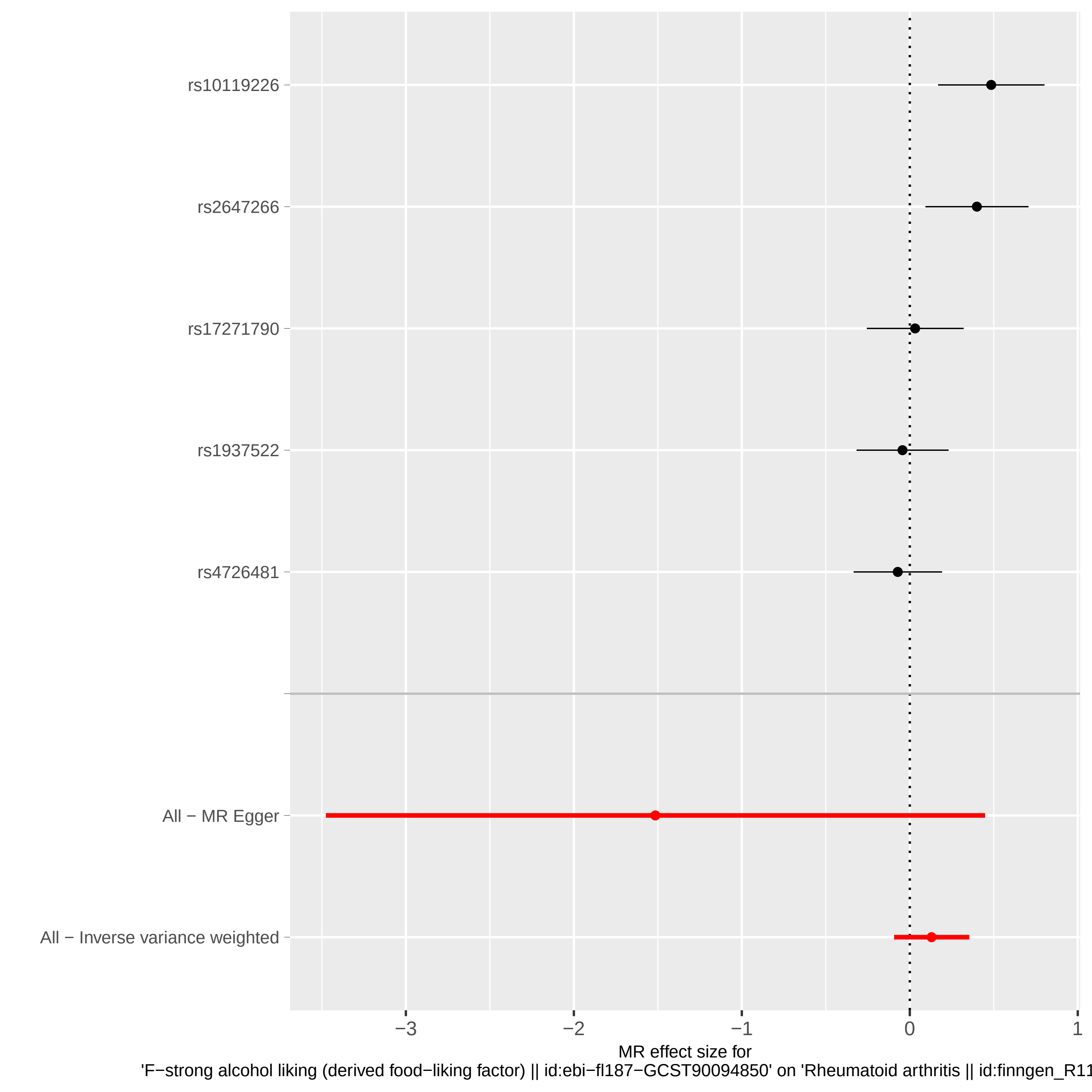
rs10119226
rs2647266
rs17271790
rs1937522
rs4726481
All − MR Egger
All − Inverse variance weighted
−3
−2
−1
0
1
MR effect size for
'F−strong alcohol liking (derived food−liking factor) || id:ebi−fl187−GCST90094850' on 'Rheumatoid arthritis || id:finngen_R11

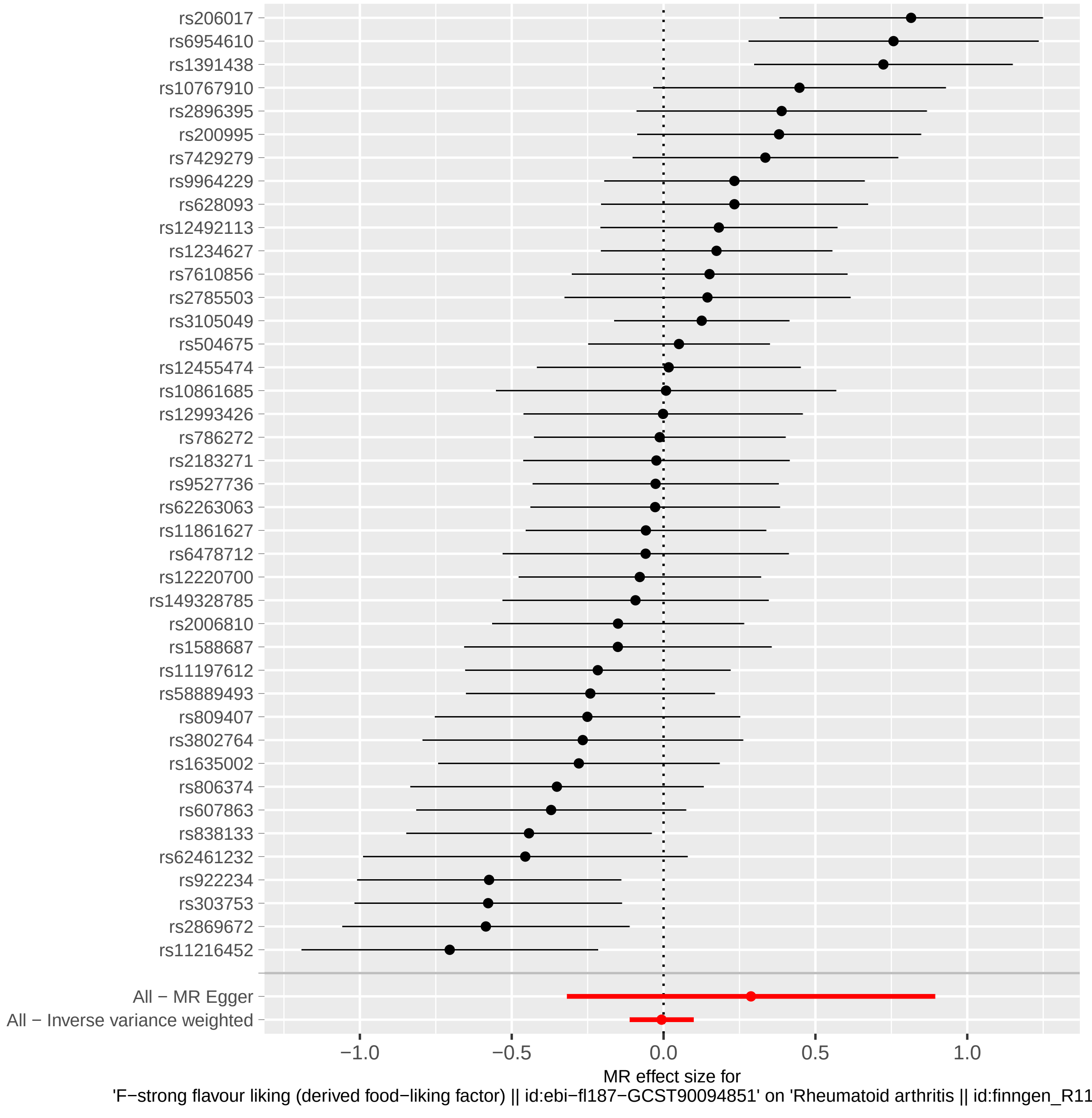

rs206017
rs6954610
rs1391438
rs10767910
rs2896395
rs200995
rs7429279
rs9964229
rs628093
rs12492113
rs1234627
rs7610856
rs2785503
rs3105049
rs504675
rs12455474
rs10861685
rs12993426
rs786272
rs2183271
rs9527736
rs62263063
rs11861627
rs6478712
rs12220700
rs149328785
rs2006810
rs1588687
rs11197612
rs58889493
rs809407
rs3802764
rs1635002
rs806374
rs607863
rs838133
rs62461232
rs922234
rs303753
rs2869672
rs11216452
All − MR Egger
All − Inverse variance weighted
−1.0
−0.5
0.0
0.5
1.0
MR effect size for
'F−strong flavour liking (derived food−liking factor) || id:ebi−fl187−GCST90094851' on 'Rheumatoid arthritis || id:finngen_R11

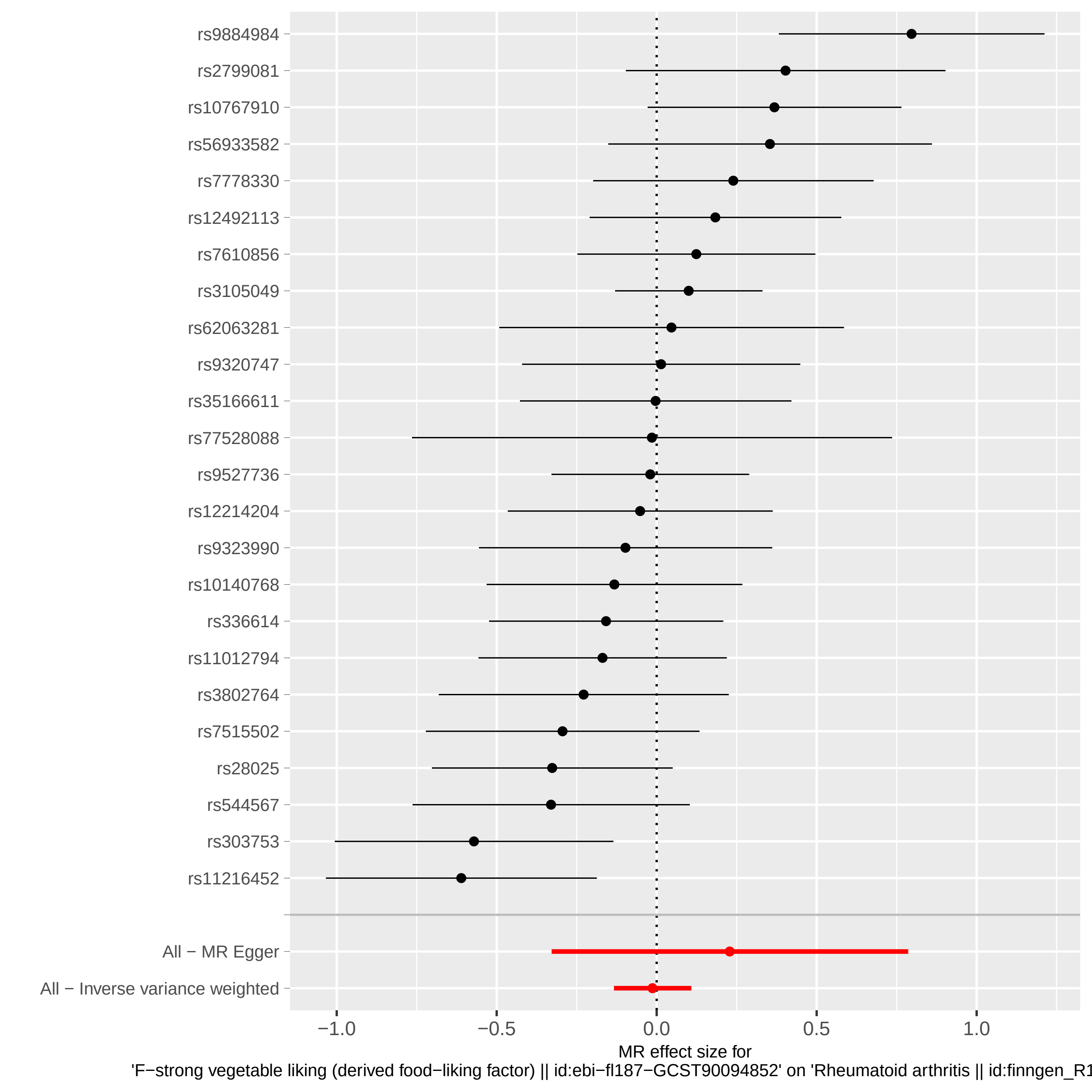

rs9884984
rs2799081
rs10767910
rs56933582
rs7778330
rs12492113
rs7610856
rs3105049
rs62063281
rs9320747
rs35166611
rs77528088
rs9527736
rs12214204
rs9323990
rs10140768
rs336614
rs11012794
rs3802764
rs7515502
rs28025
rs544567
rs303753
rs11216452
All − MR Egger
All − Inverse variance weighted
−1.0
−0.5
0.0
0.5
1.0
MR effect size for
'F−strong vegetable liking (derived food−liking factor) || id:ebi−fl187−GCST90094852' on 'Rheumatoid arthritis || id:finngen_R1

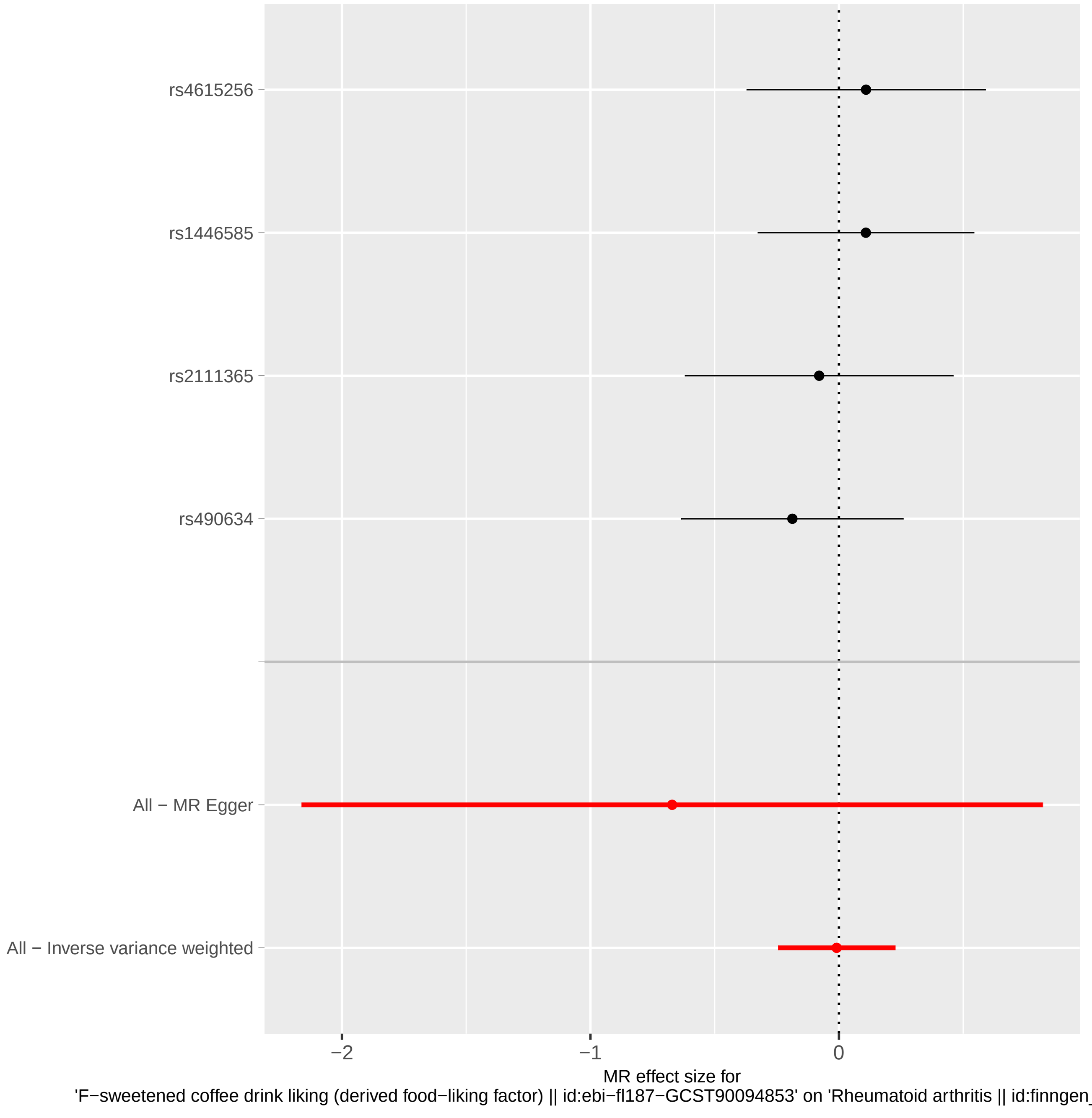

rs4615256
rs1446585
rs2111365
rs490634
All − MR Egger
All − Inverse variance weighted
−2
−1
0
MR effect size for
'F−sweetened coffee drink liking (derived food−liking factor) || id:ebi−fl187−GCST90094853' on 'Rheumatoid arthritis || id:finngen_

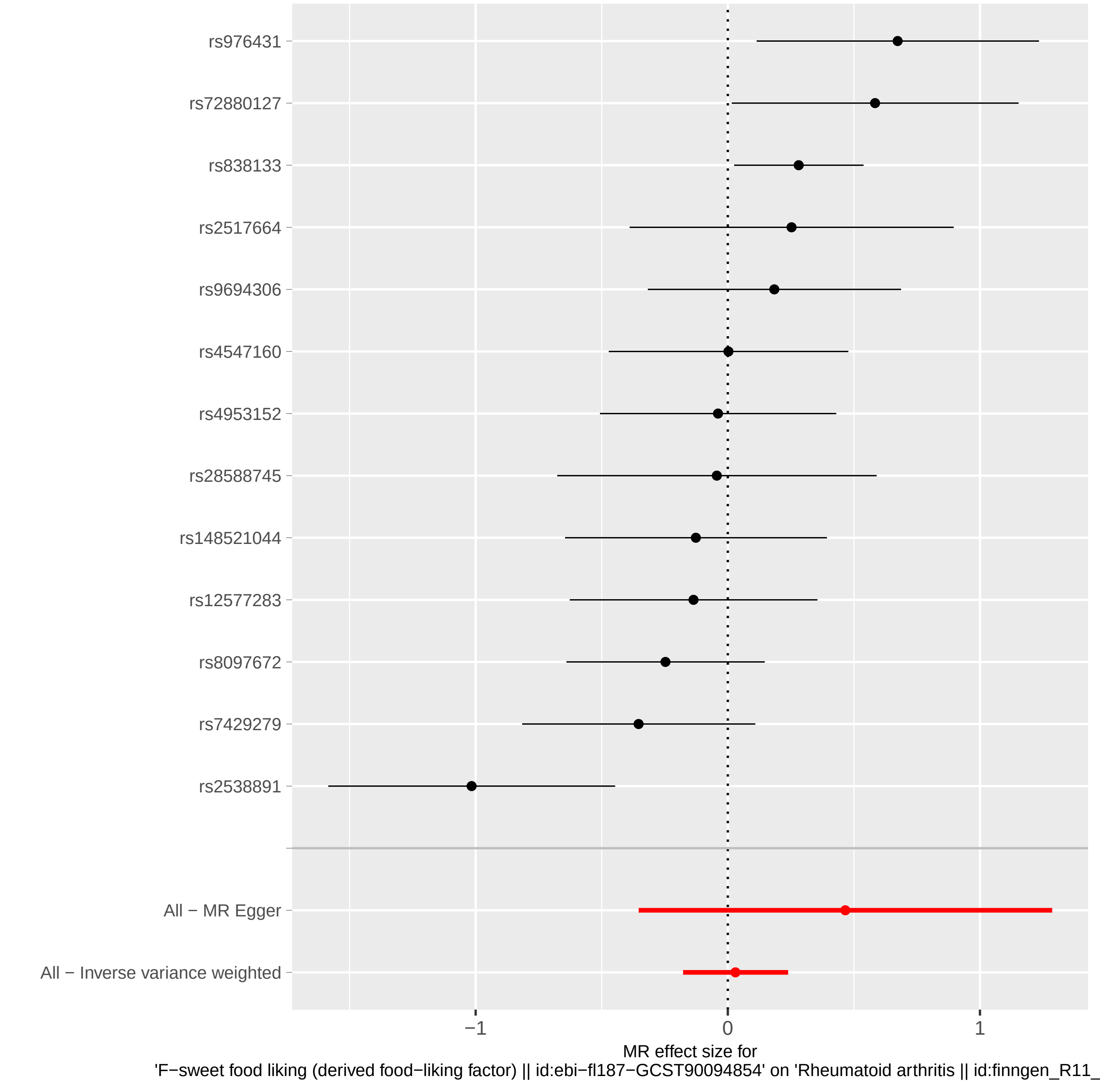
rs976431
rs72880127
rs838133
rs2517664
rs9694306
rs4547160
rs4953152
rs28588745
rs148521044
rs12577283
rs8097672
rs7429279
rs2538891
All − MR Egger
All − Inverse variance weighted
−1
0
1
MR effect size for
'F−sweet food liking (derived food−liking factor) || id:ebi−fl187−GCST90094854' on 'Rheumatoid arthritis || id:finngen_R11_

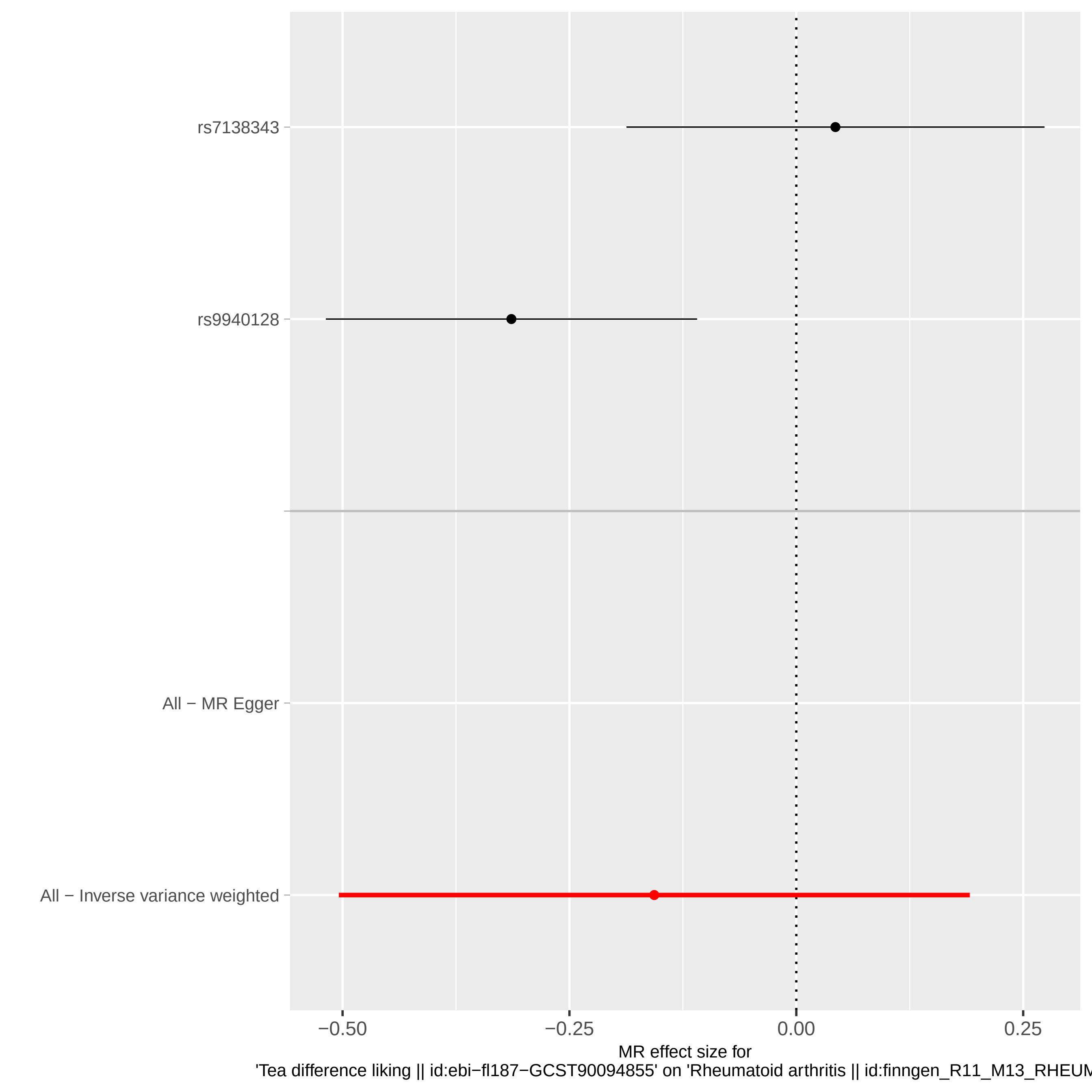
rs7138343
rs9940128
All − MR Egger
All − Inverse variance weighted
−0.50
−0.25
0.00
0.25
MR effect size for
'Tea difference liking || id:ebi−fl187−GCST90094855' on 'Rheumatoid arthritis || id:finngen_R11_M13_RHEUM

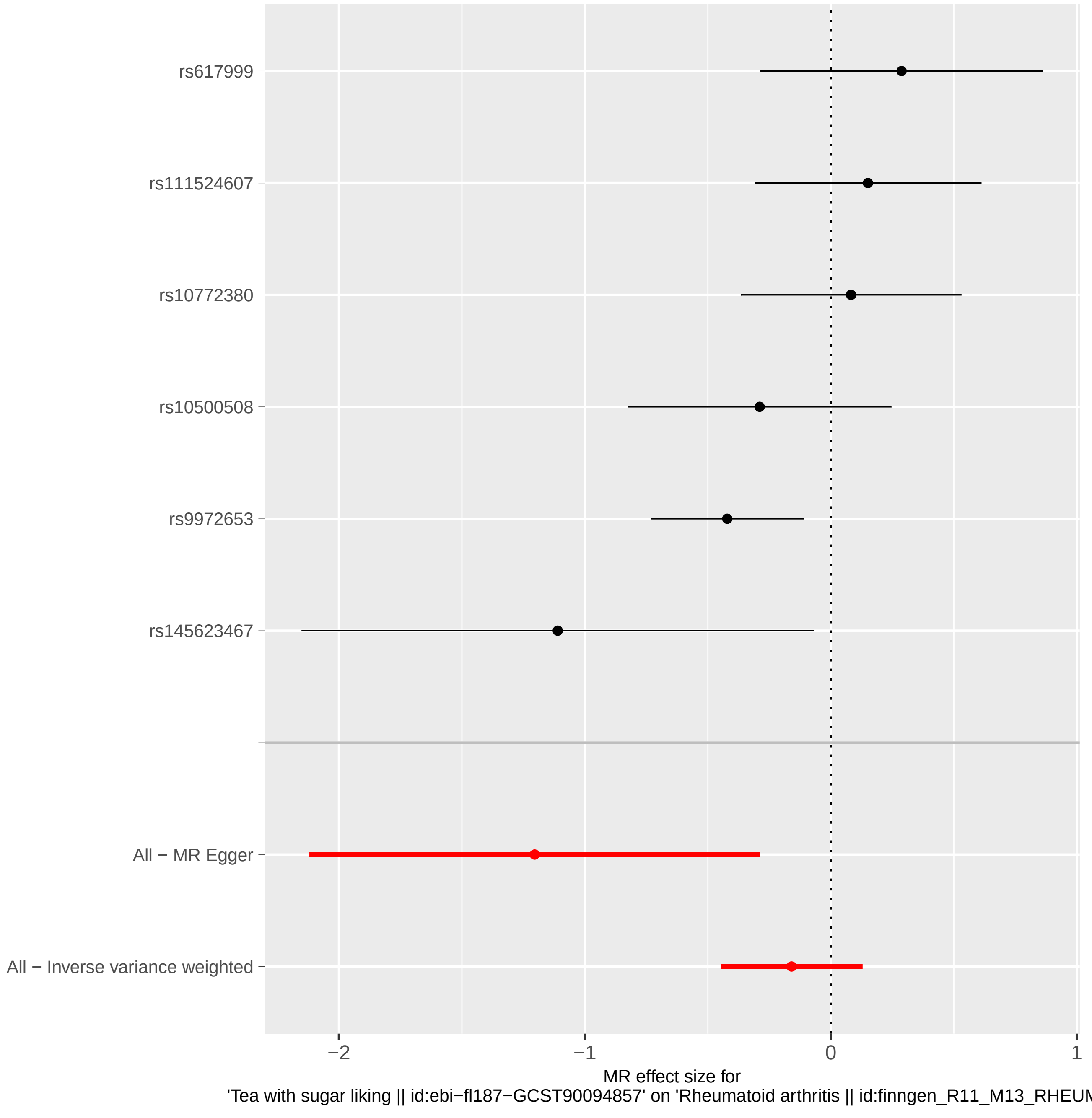

rs617999
rs111524607
rs10772380
rs10500508
rs9972653
rs145623467
All − MR Egger
All − Inverse variance weighted
−2
−1
0
1
MR effect size for
'Tea with sugar liking || id:ebi−fl187−GCST90094857' on 'Rheumatoid arthritis || id:finngen_R11_M13_RHEUM

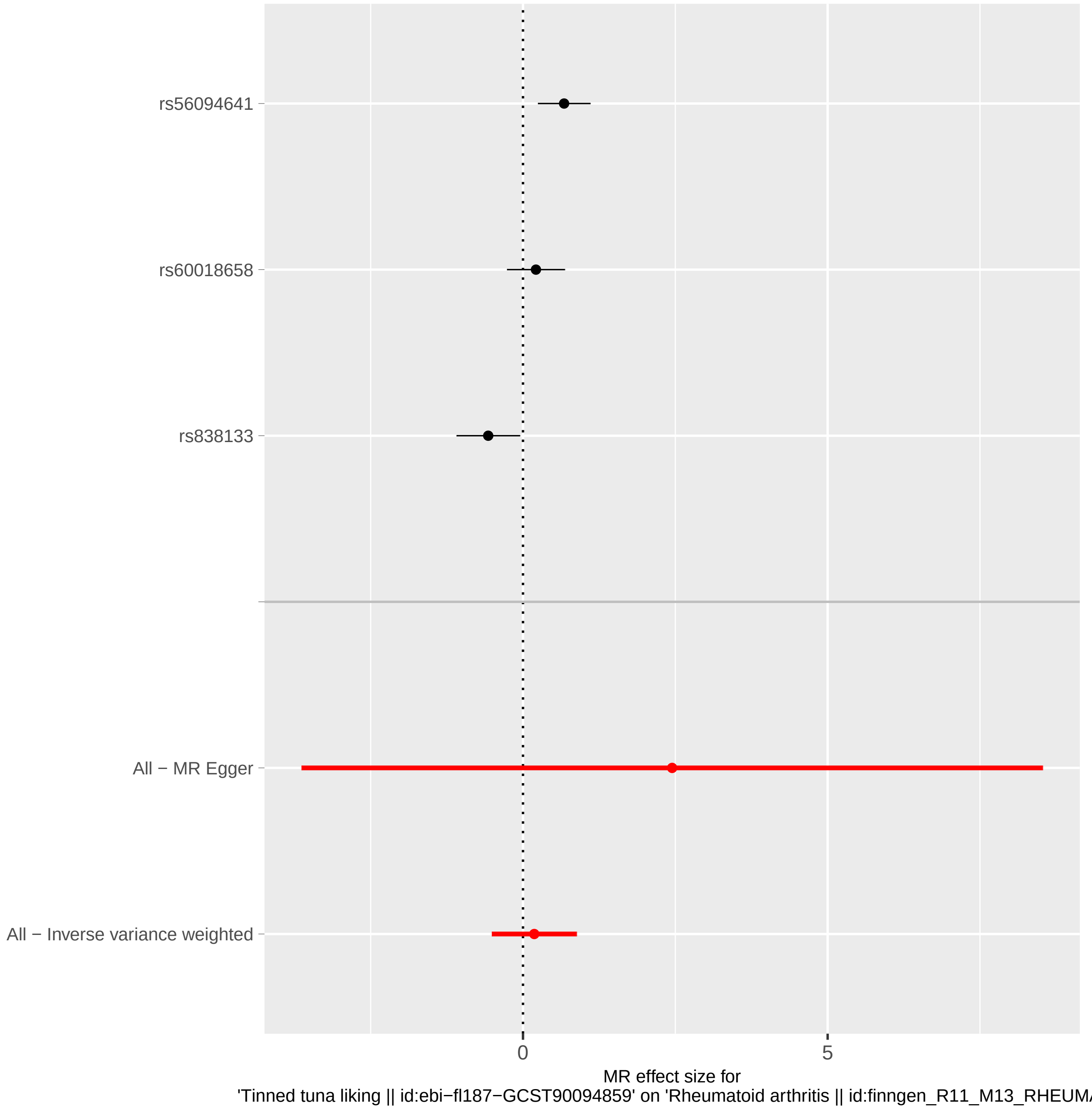
rs56094641
rs60018658
rs838133
All − MR Egger
All − Inverse variance weighted
0
5
MR effect size for
'Tinned tuna liking || id:ebi−fl187−GCST90094859' on 'Rheumatoid arthritis || id:finngen_R11_M13_RHEUM

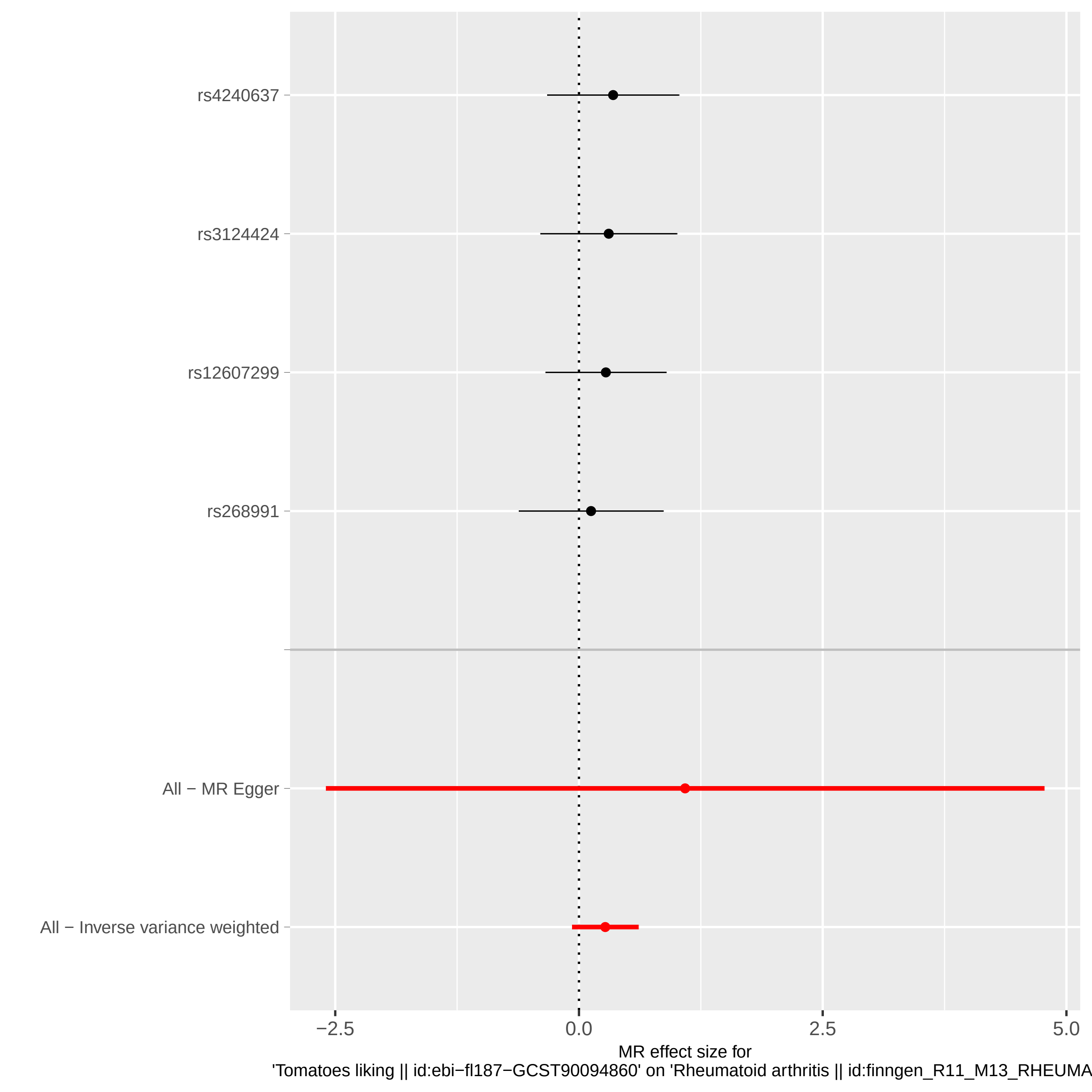
rs4240637
rs3124424
rs12607299
rs268991
All − MR Egger
All − Inverse variance weighted
−2.5
0.0
2.5
5.0
MR effect size for
'Tomatoes liking || id:ebi−fl187−GCST90094860' on 'Rheumatoid arthritis || id:finngen_R11_M13_RHEUMA

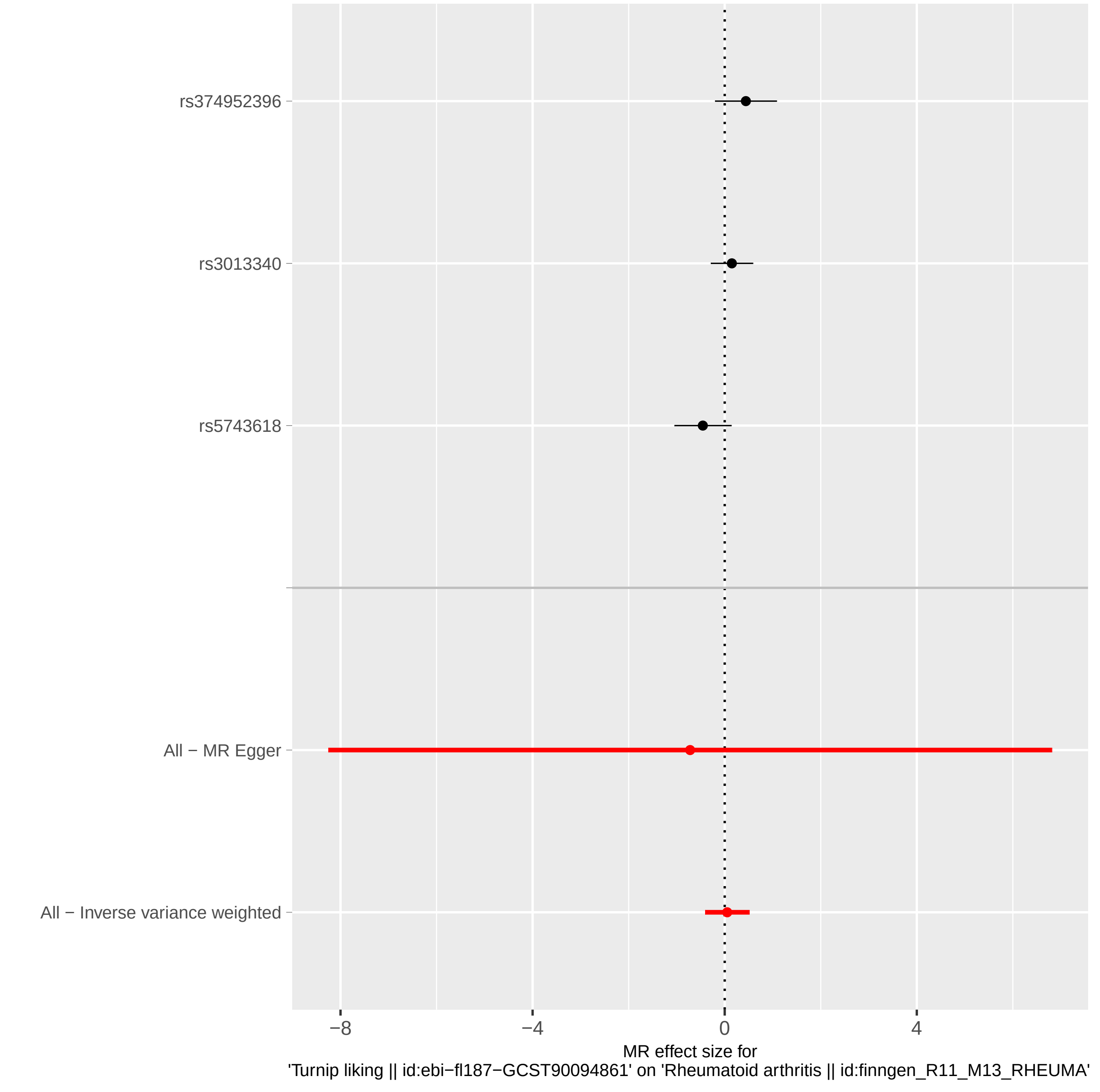

rs374952396
rs3013340
rs5743618
All − MR Egger
All − Inverse variance weighted
−8
−4
0
4
MR effect size for
'Turnip liking || id:ebi−fl187−GCST90094861' on 'Rheumatoid arthritis || id:finngen_R11_M13_RHEUMA'

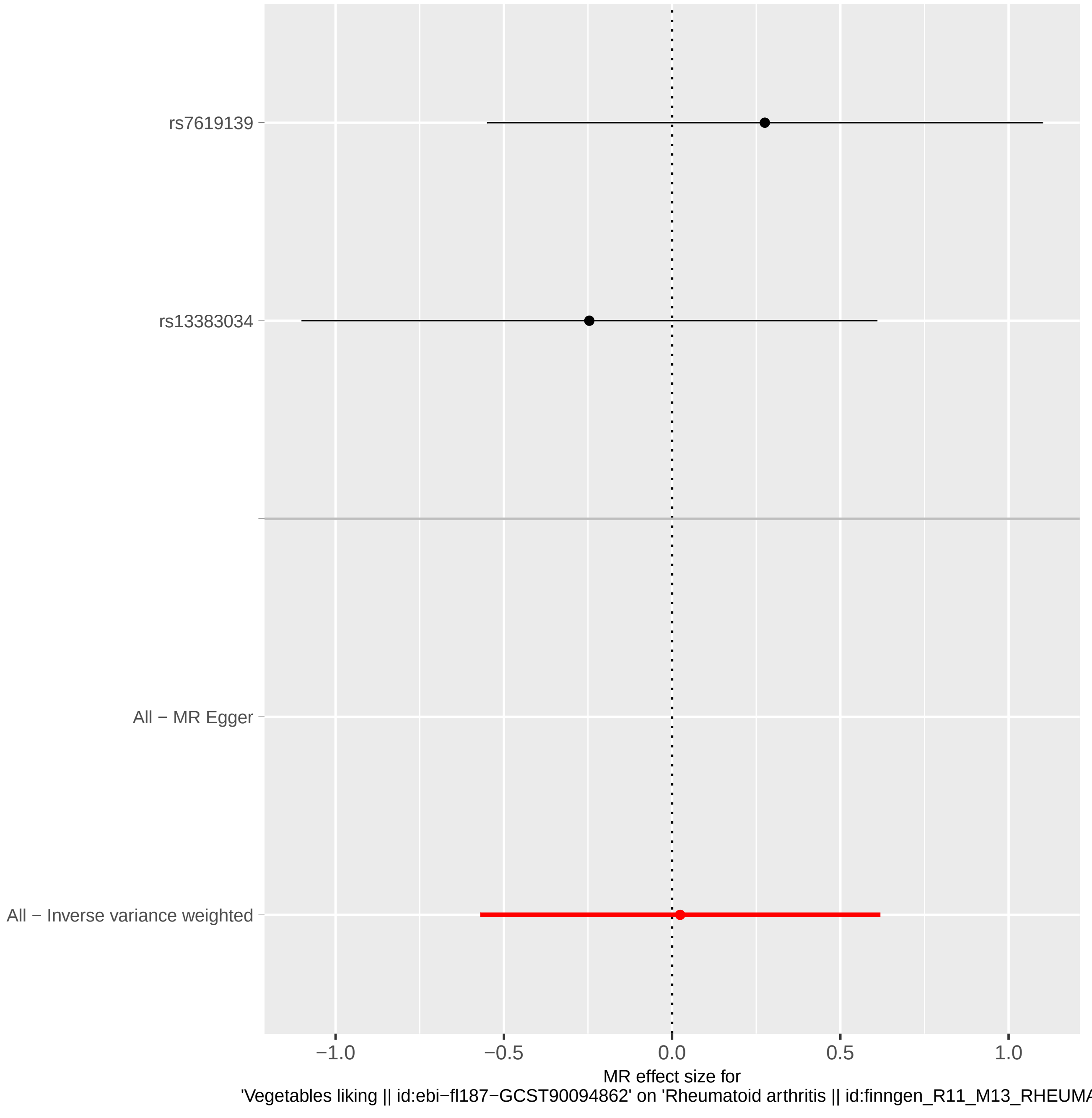
rs7619139
rs13383034
All − MR Egger
All − Inverse variance weighted
−1.0
−0.5
0.0
0.5
1.0
MR effect size for
'Vegetables liking || id:ebi−fl187−GCST90094862' on 'Rheumatoid arthritis || id:finngen_R11_M13_RHEUMA

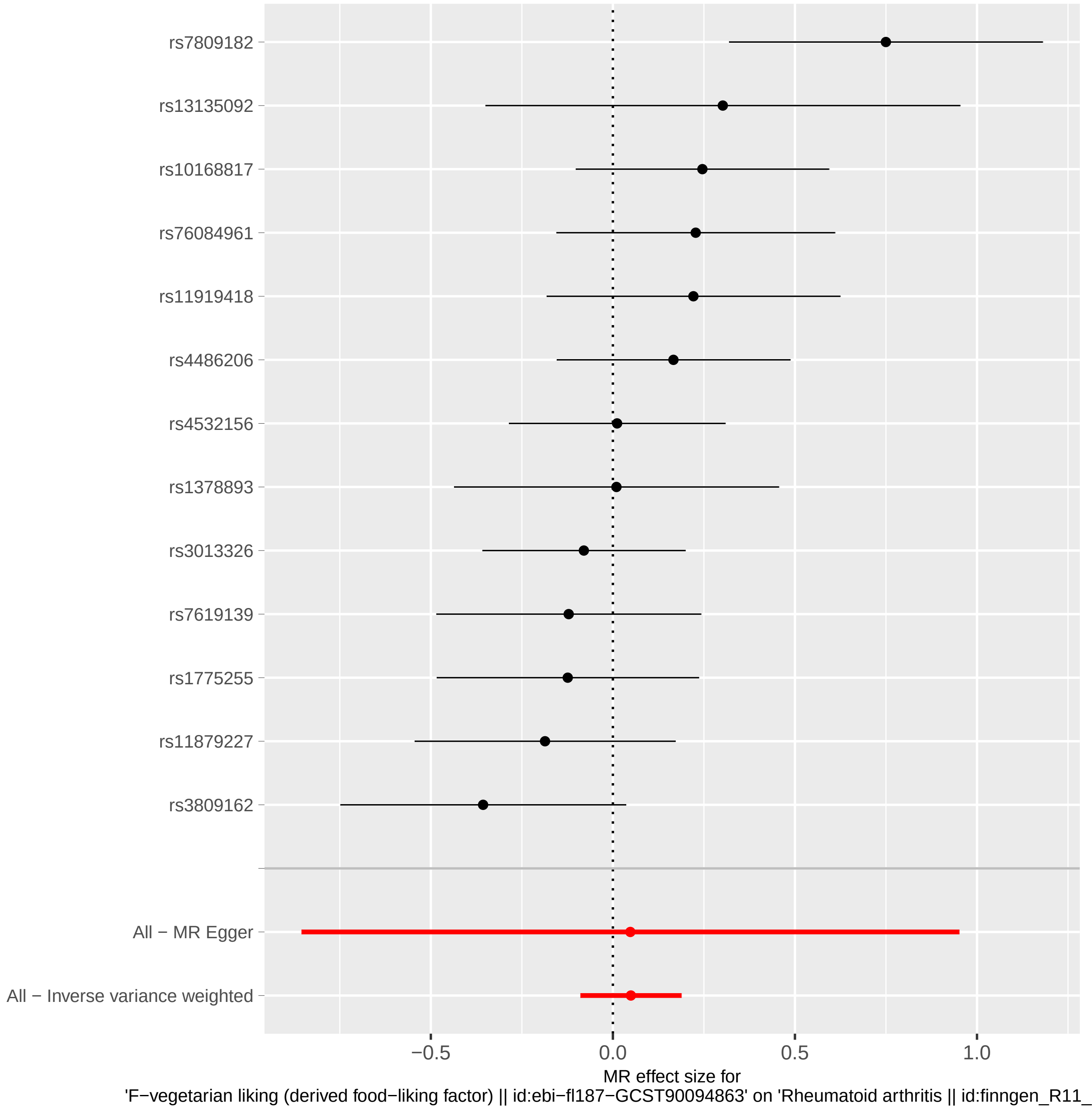

rs7809182
rs13135092
rs10168817
rs76084961
rs11919418
rs4486206
rs4532156
rs1378893
rs3013326
rs7619139
rs1775255
rs11879227
rs3809162
All − MR Egger
All − Inverse variance weighted
−0.5
0.0
0.5
1.0
MR effect size for
'F−vegetarian liking (derived food−liking factor) || id:ebi−fl187−GCST90094863' on 'Rheumatoid arthritis || id:finngen_R11_

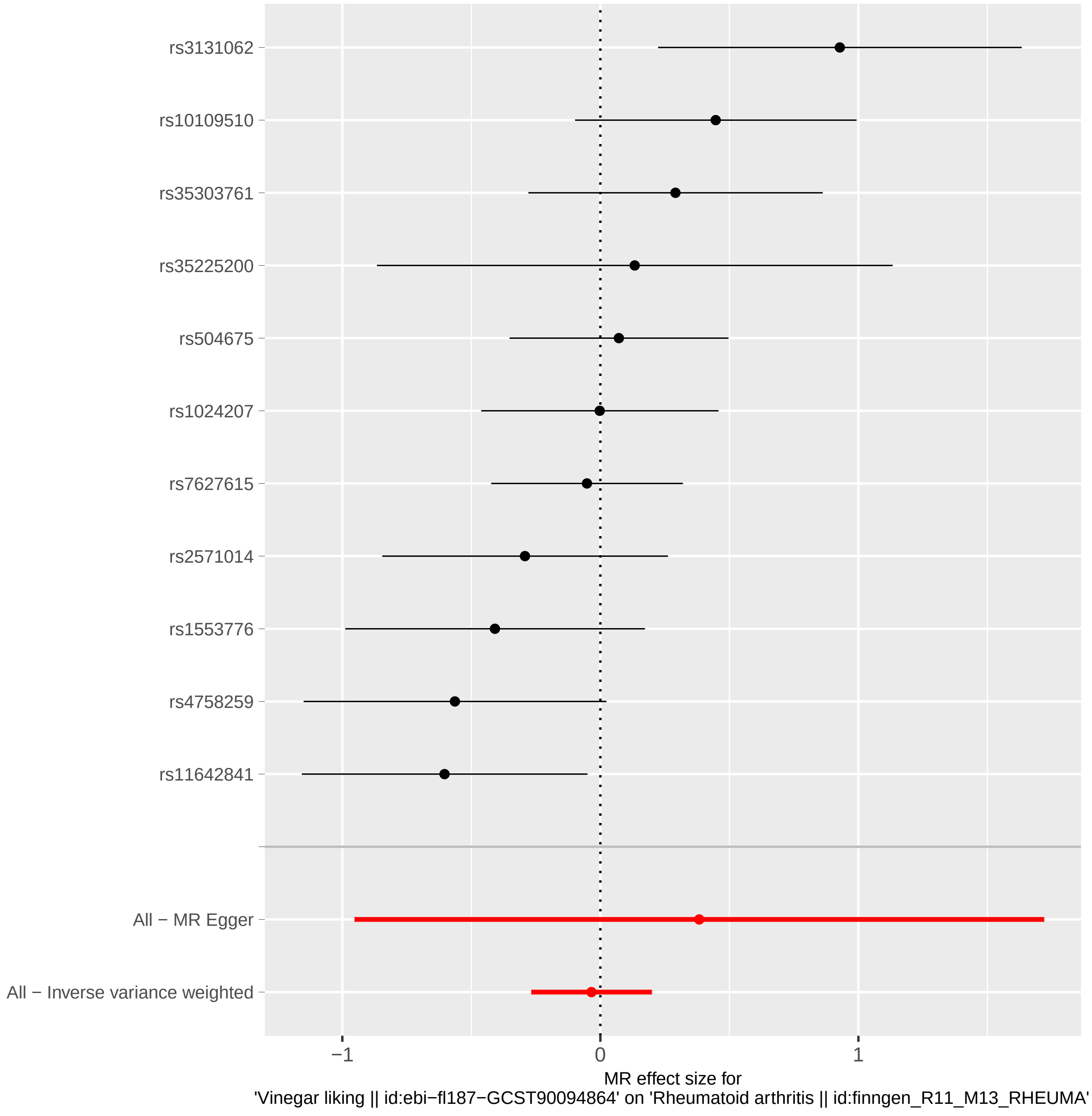
rs3131062
rs10109510
rs35303761
rs35225200
rs504675
rs1024207
rs7627615
rs2571014
rs1553776
rs4758259
rs11642841
All − MR Egger
All − Inverse variance weighted
−1
0
1
MR effect size for
'Vinegar liking || id:ebi−fl187−GCST90094864' on 'Rheumatoid arthritis || id:finngen_R11_M13_RHEUMA'

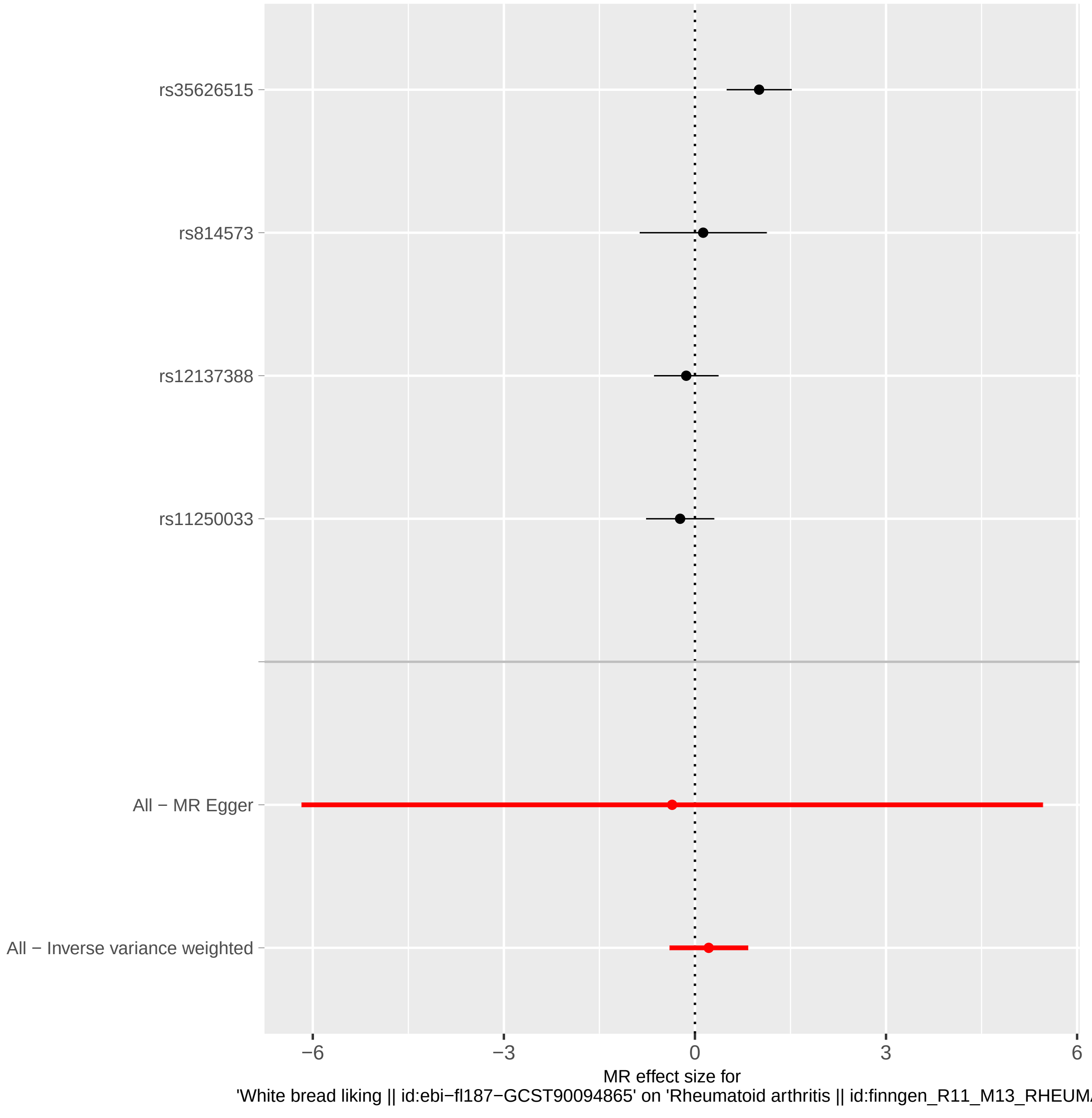
rs35626515
rs814573
rs12137388
rs11250033
All − MR Egger
All − Inverse variance weighted
−6
−3
0
3
6
MR effect size for
'White bread liking || id:ebi−fl187−GCST90094865' on 'Rheumatoid arthritis || id:finngen_R11_M13_RHEUM

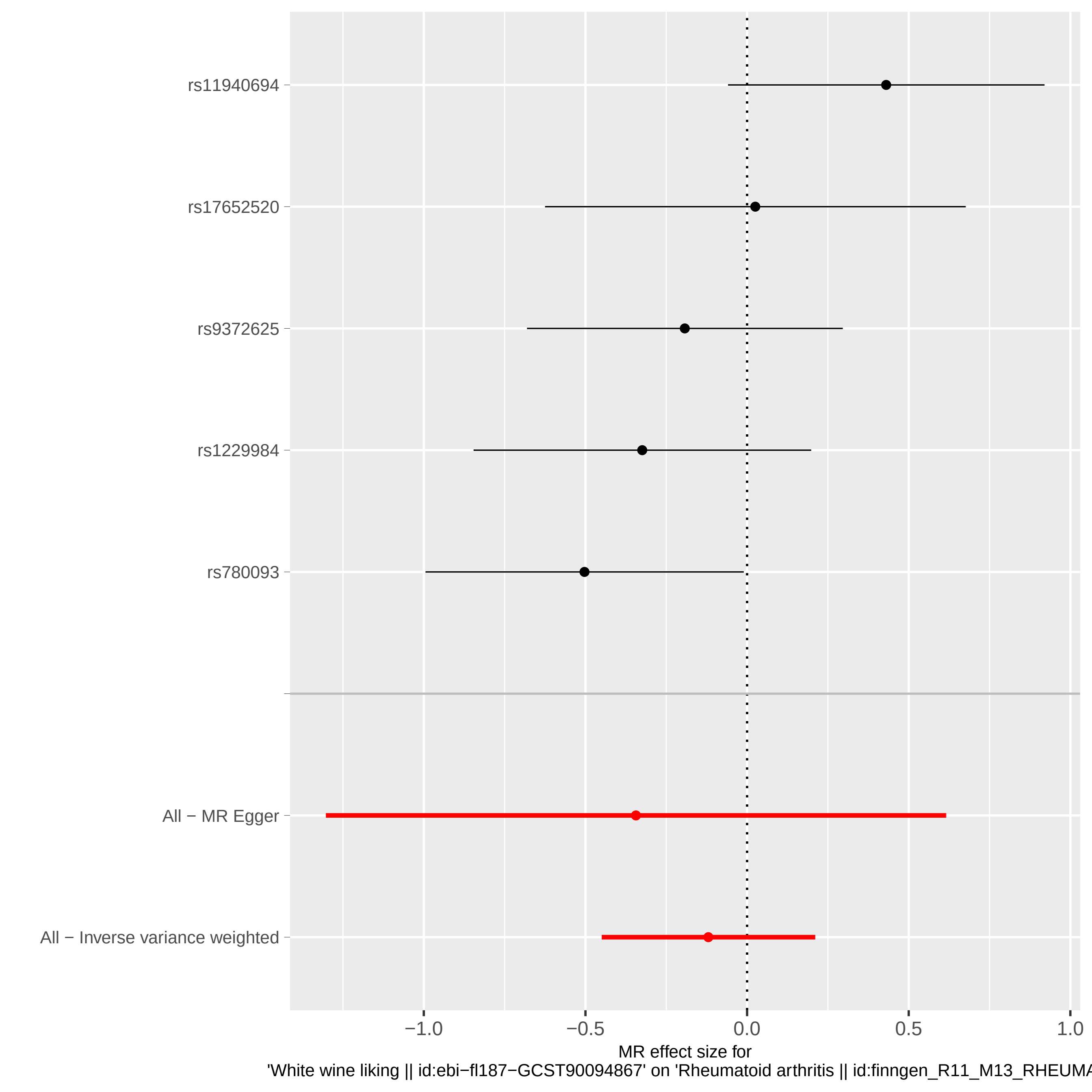
rs11940694
rs17652520
rs9372625
rs1229984
rs780093
All − MR Egger
All − Inverse variance weighted
−1.0
−0.5
0.0
0.5
1.0
MR effect size for
'White wine liking || id:ebi−fl187−GCST90094867' on 'Rheumatoid arthritis || id:finngen_R11_M13_RHEUMA

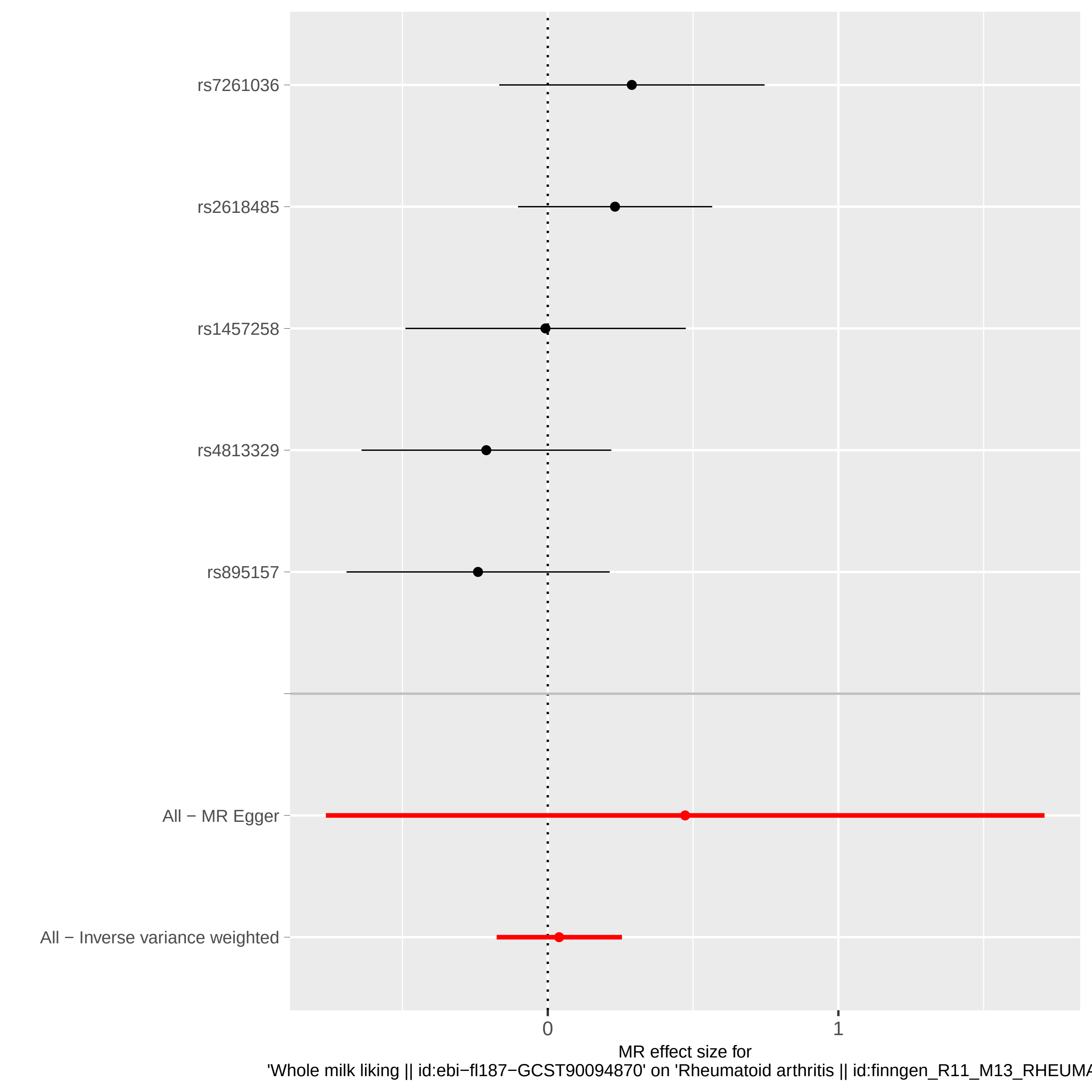
rs7261036
rs2618485
rs1457258
rs4813329
rs895157
All − MR Egger
All − Inverse variance weighted
0
1
MR effect size for
'Whole milk liking || id:ebi−fl187−GCST90094870' on 'Rheumatoid arthritis || id:finngen_R11_M13_RHEUMA

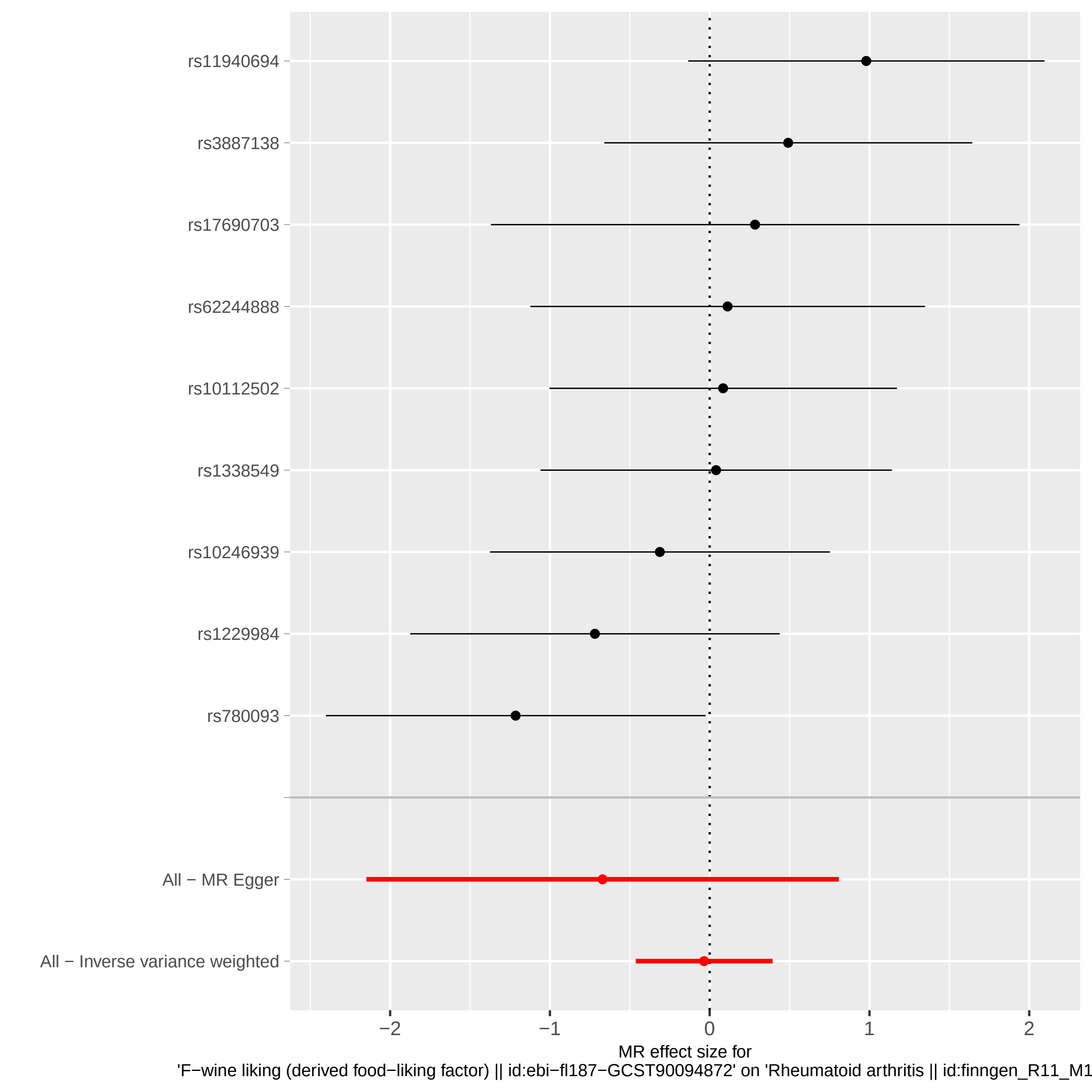
rs11940694
rs3887138
rs17690703
rs62244888
rs10112502
rs1338549
rs10246939
rs1229984
rs780093
All − MR Egger
All − Inverse variance weighted
−2
−1
0
1
2
MR effect size for
'F−wine liking (derived food−liking factor) || id:ebi−fl187−GCST90094872' on 'Rheumatoid arthritis || id:finngen_R11_M1

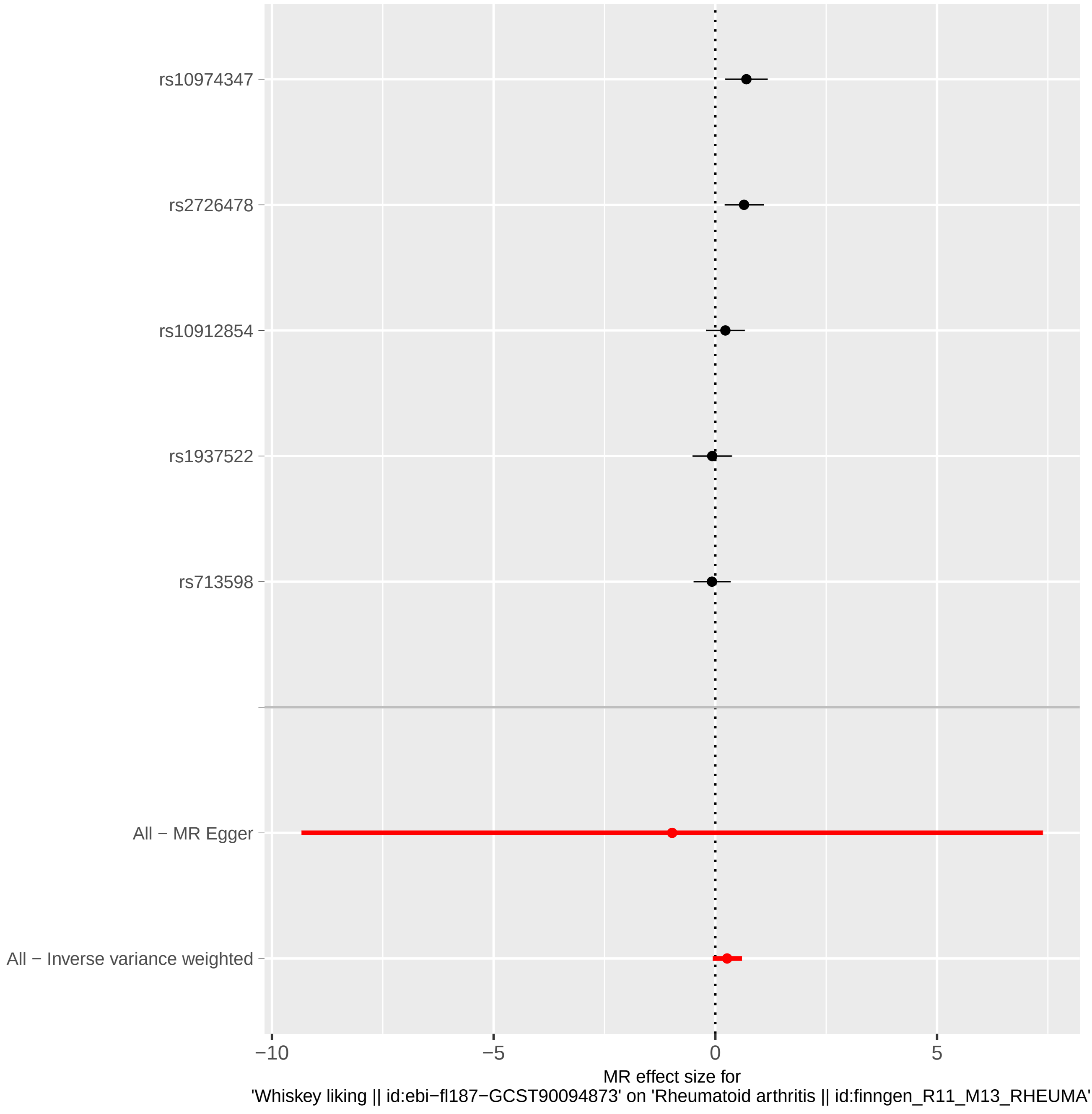
rs10974347
rs2726478
rs10912854
rs1937522
rs713598
All − MR Egger
All − Inverse variance weighted
−10
−5
0
5
MR effect size for
'Whiskey liking || id:ebi−fl187−GCST90094873' on 'Rheumatoid arthritis || id:finngen_R11_M13_RHEUMA'

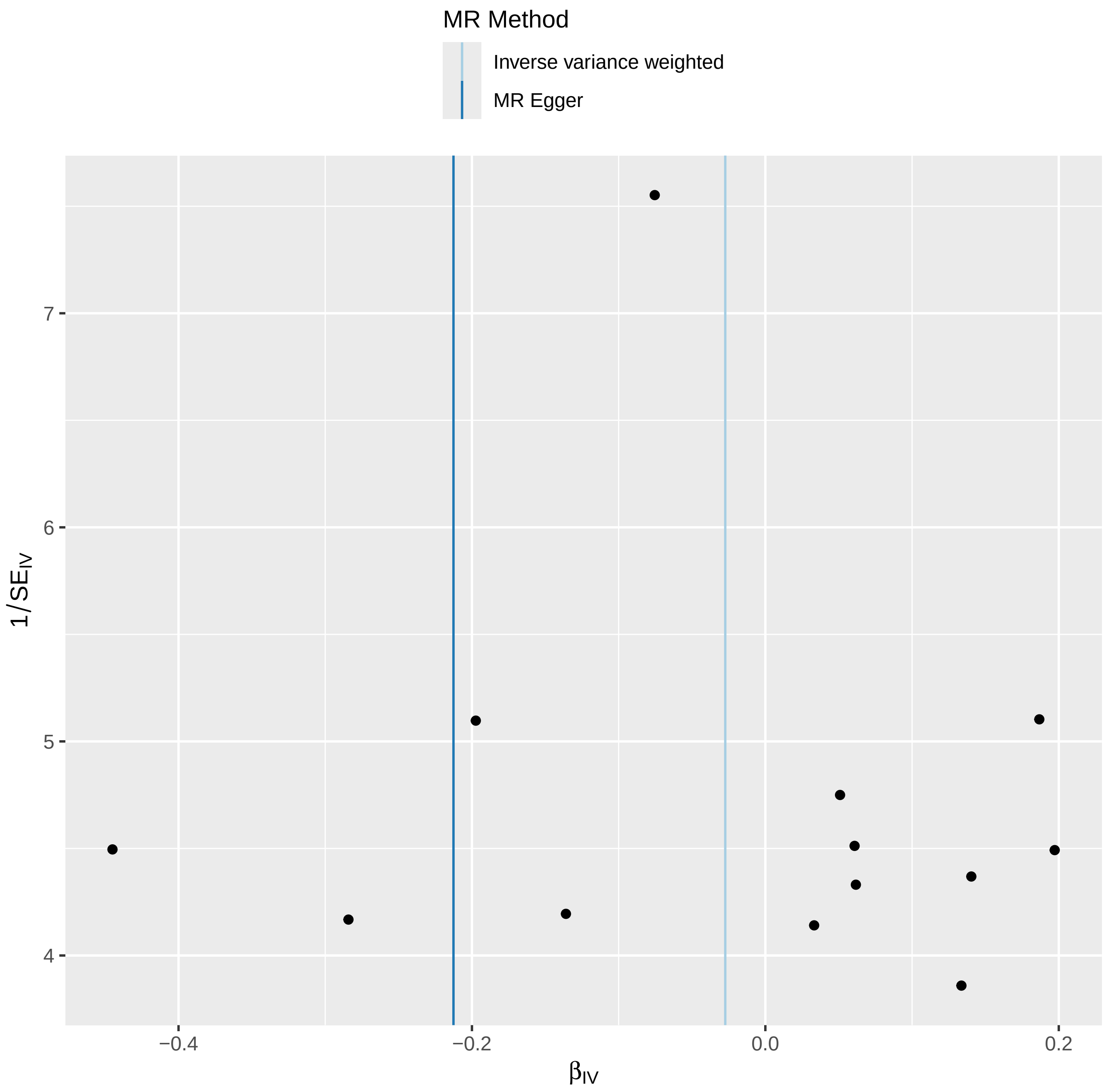

MR Method
Inverse variance weighted
MR Egger
7
6
5
4
$1/SE_{IV}$
−0.4
−0.2
0.0
0.2
β_{IV}

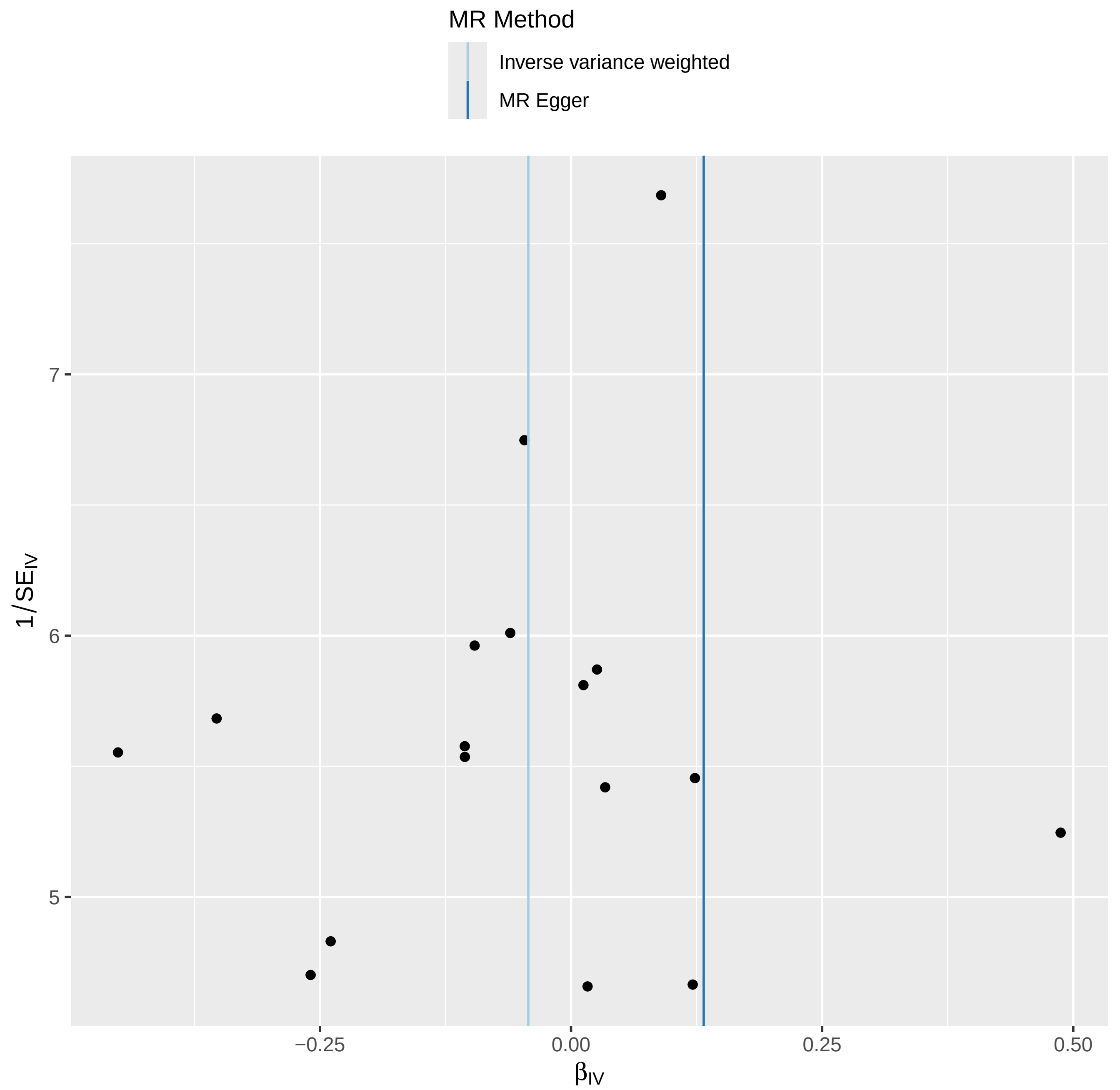

MR Method
Inverse variance weighted
MR Egger
7
6
5
1/SE_IV
−0.25
0.00
0.25
0.50
β_IV

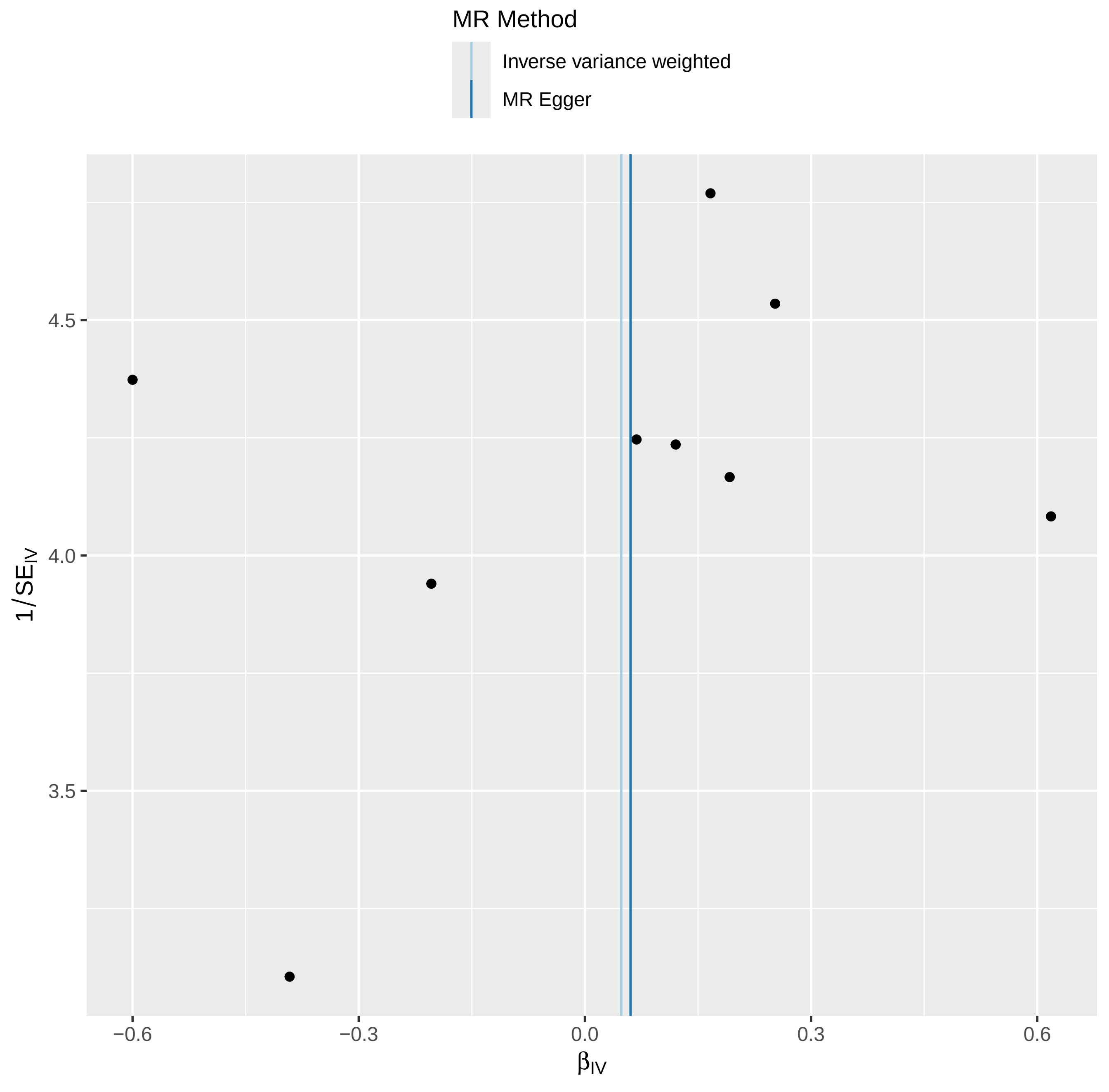

MR Method
Inverse variance weighted
MR Egger
4.5
4.0
3.5
1/SE_IV
−0.6
−0.3
0.0
0.3
0.6
β_IV

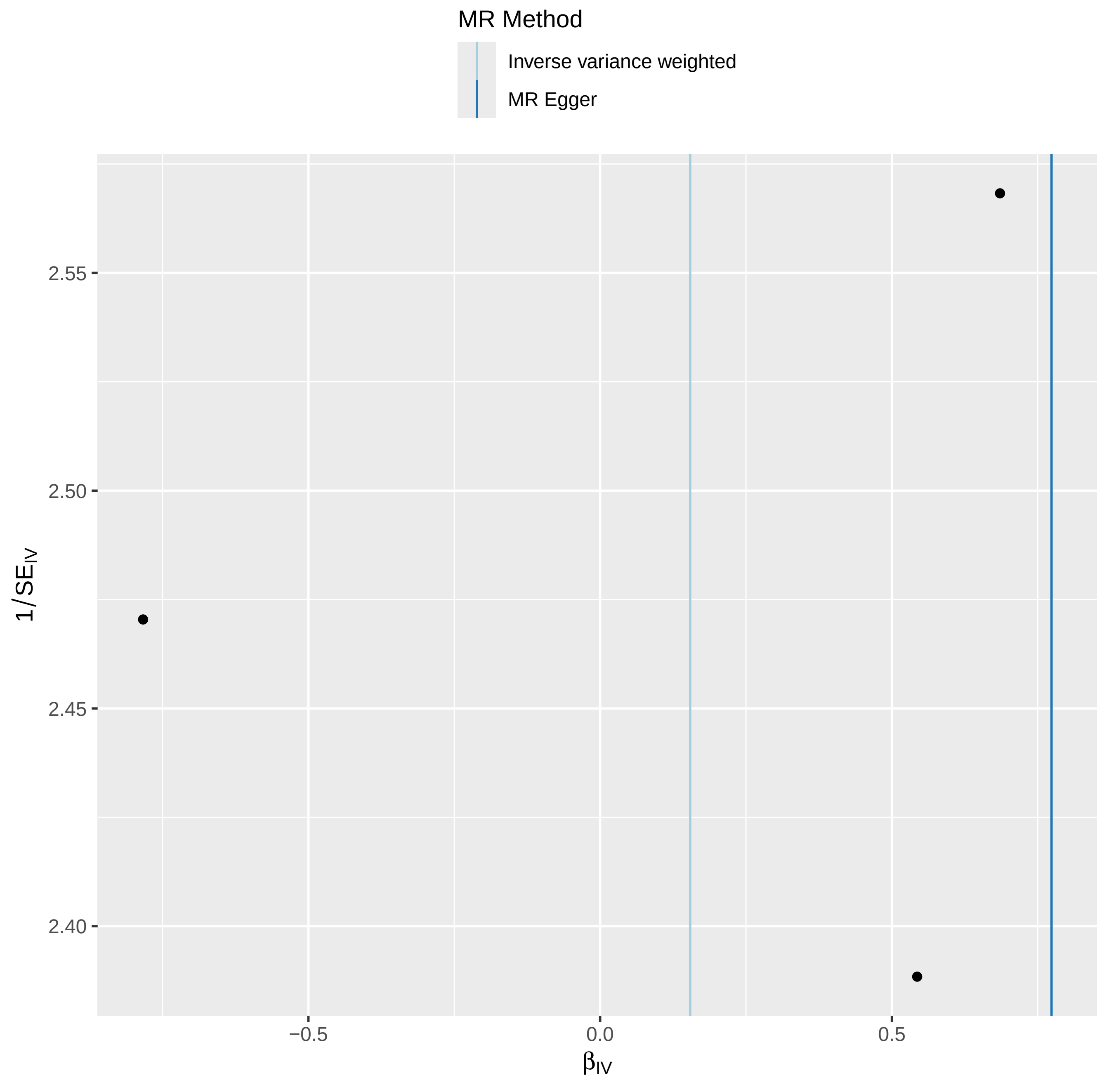
MR Method
Inverse variance weighted
MR Egger
2.55
2.50
2.45
2.40
1/SE_IV
−0.5
0.0
0.5
β_IV

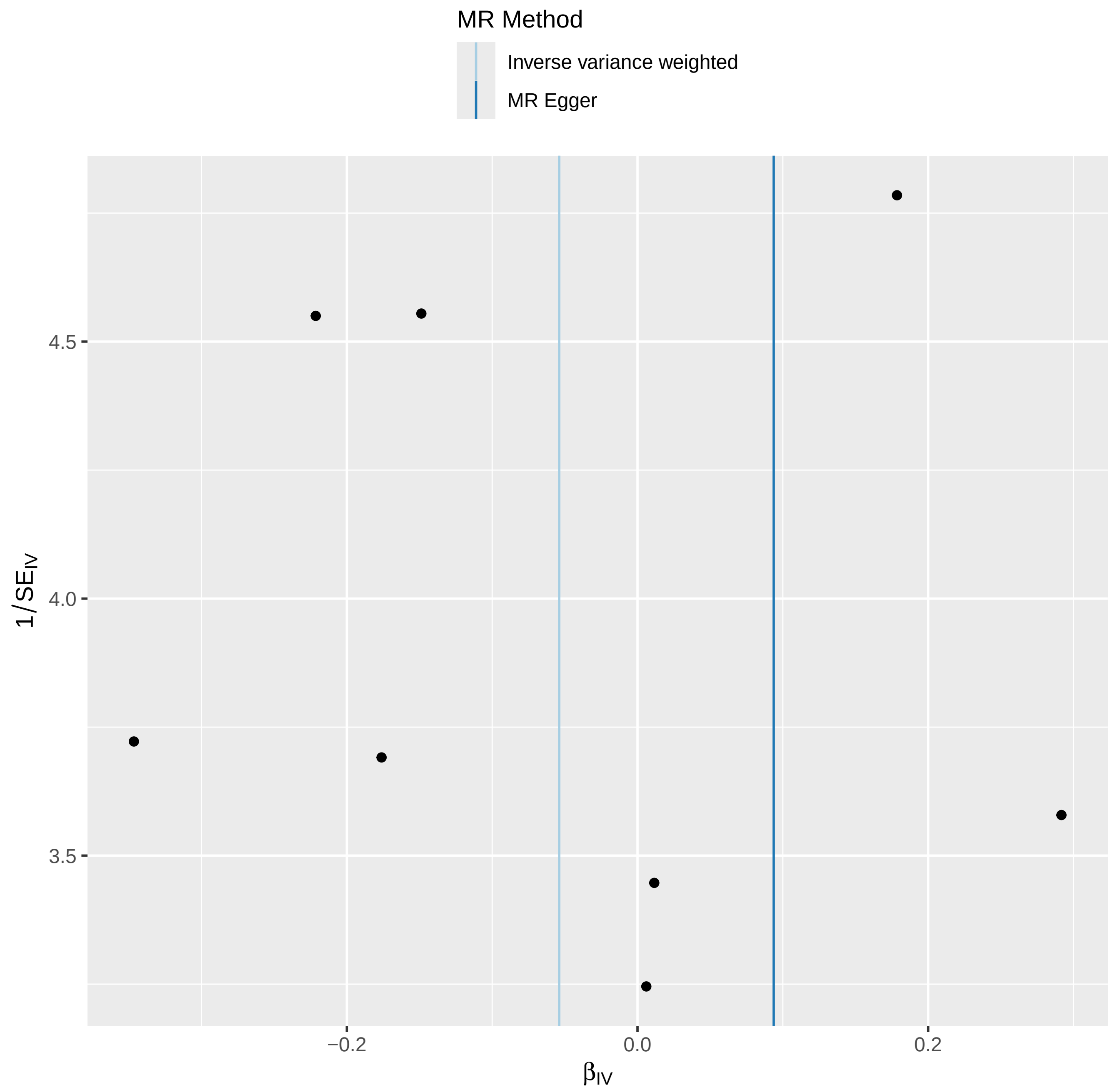
MR Method
Inverse variance weighted
MR Egger
4.5
4.0
3.5
1/SE_IV
−0.2
0.0
0.2
β_IV

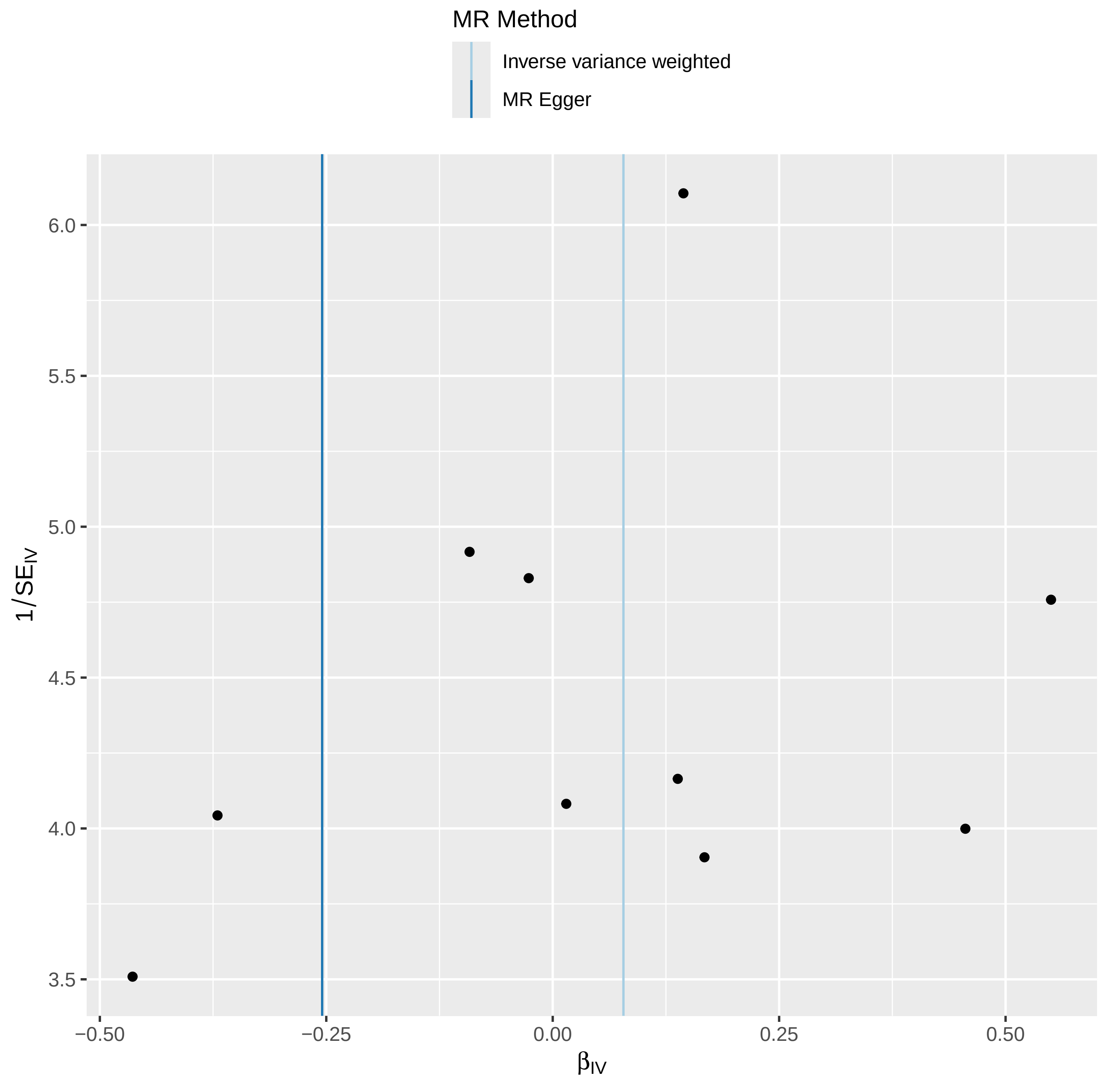
MR Method
Inverse variance weighted
MR Egger
6.0
5.5
5.0
4.5
4.0
3.5
1/SE$_{IV}$
−0.50
−0.25
0.00
0.25
0.50
β_{IV}

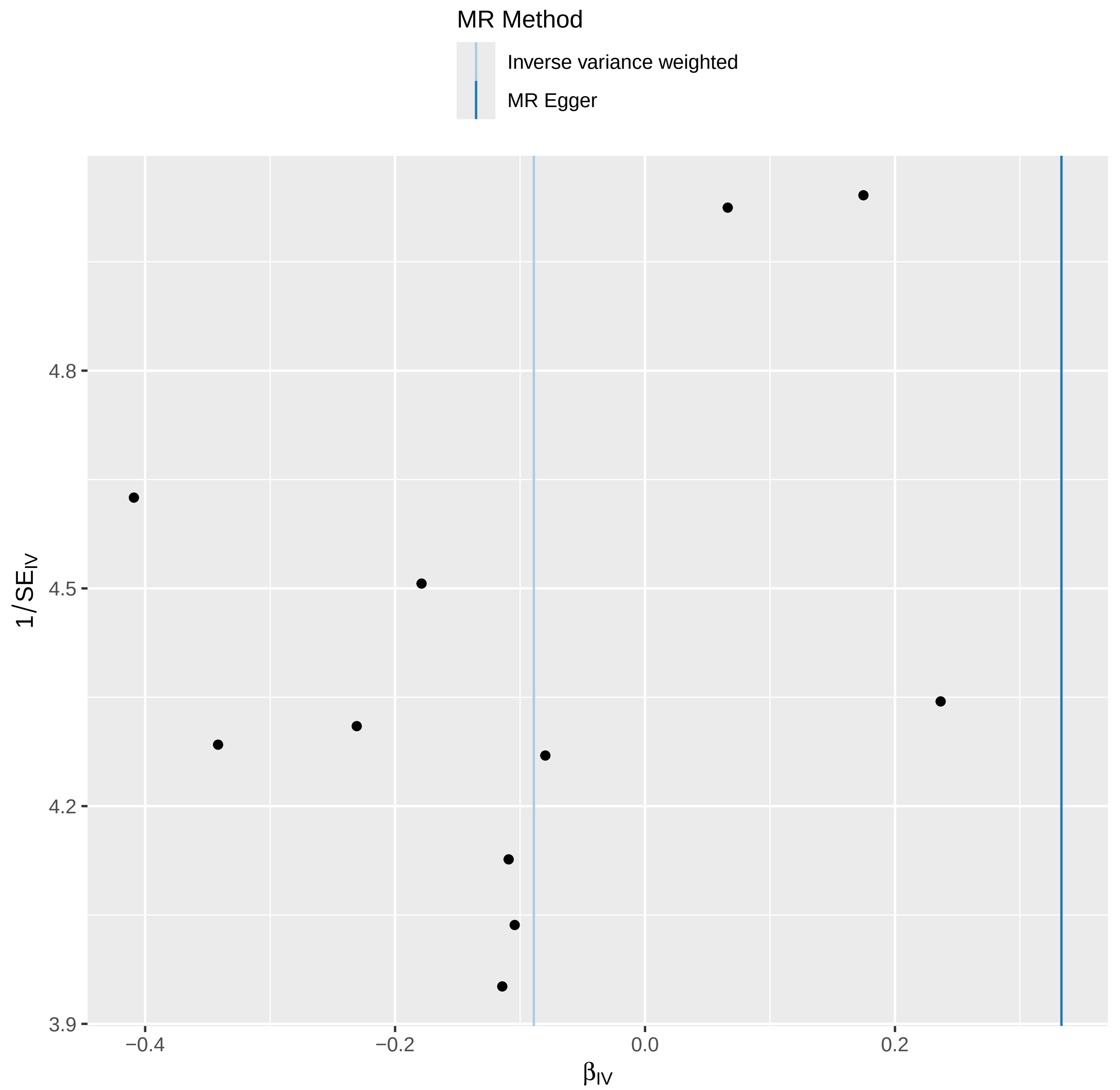
MR Method
Inverse variance weighted
MR Egger
4.8
4.5
4.2
3.9
1/SE_IV
−0.4
−0.2
0.0
0.2
β_IV

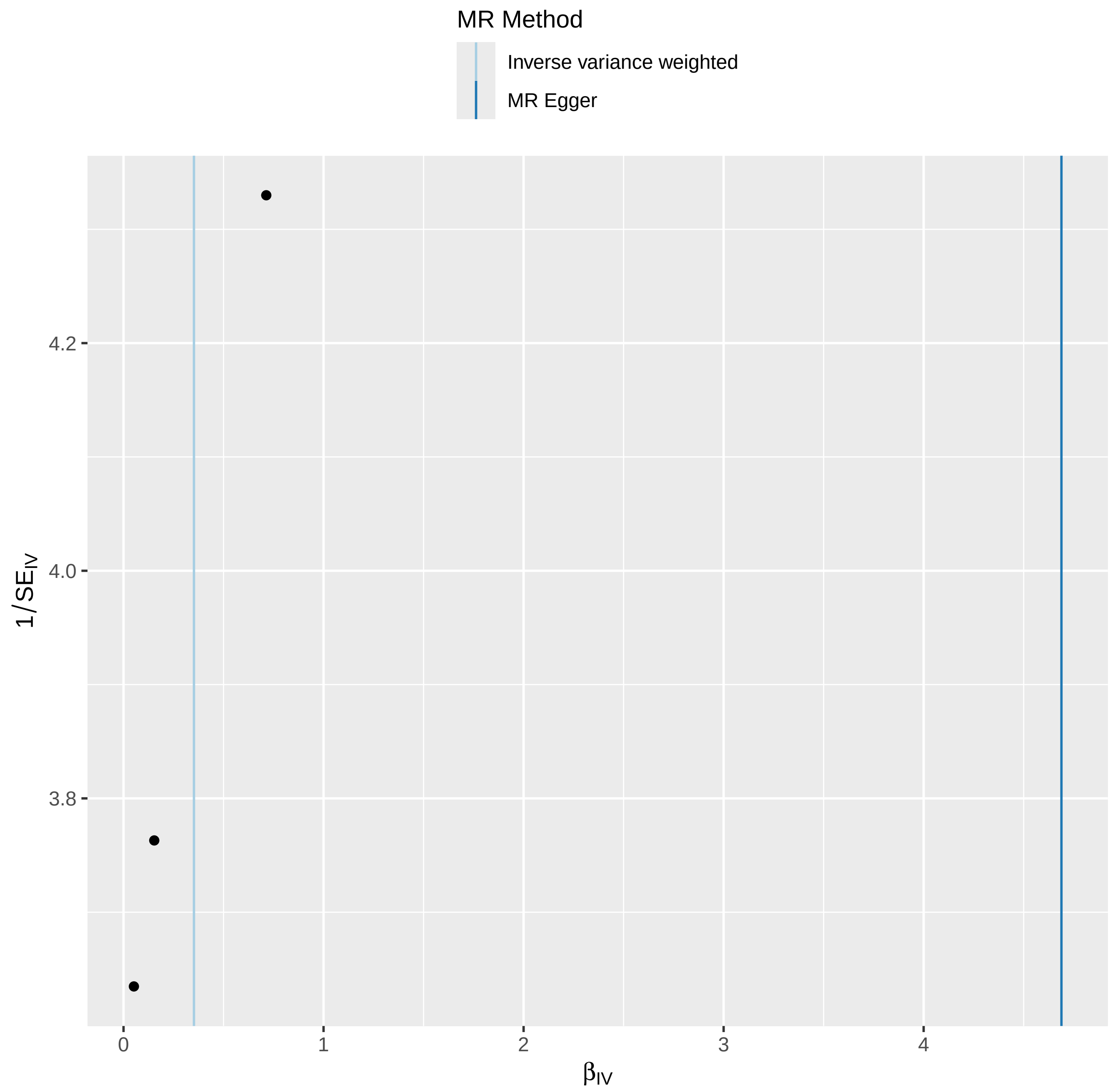
MR Method
Inverse variance weighted
MR Egger
4.2
4.0
3.8
1/SE_IV
0
1
2
3
4
β_IV

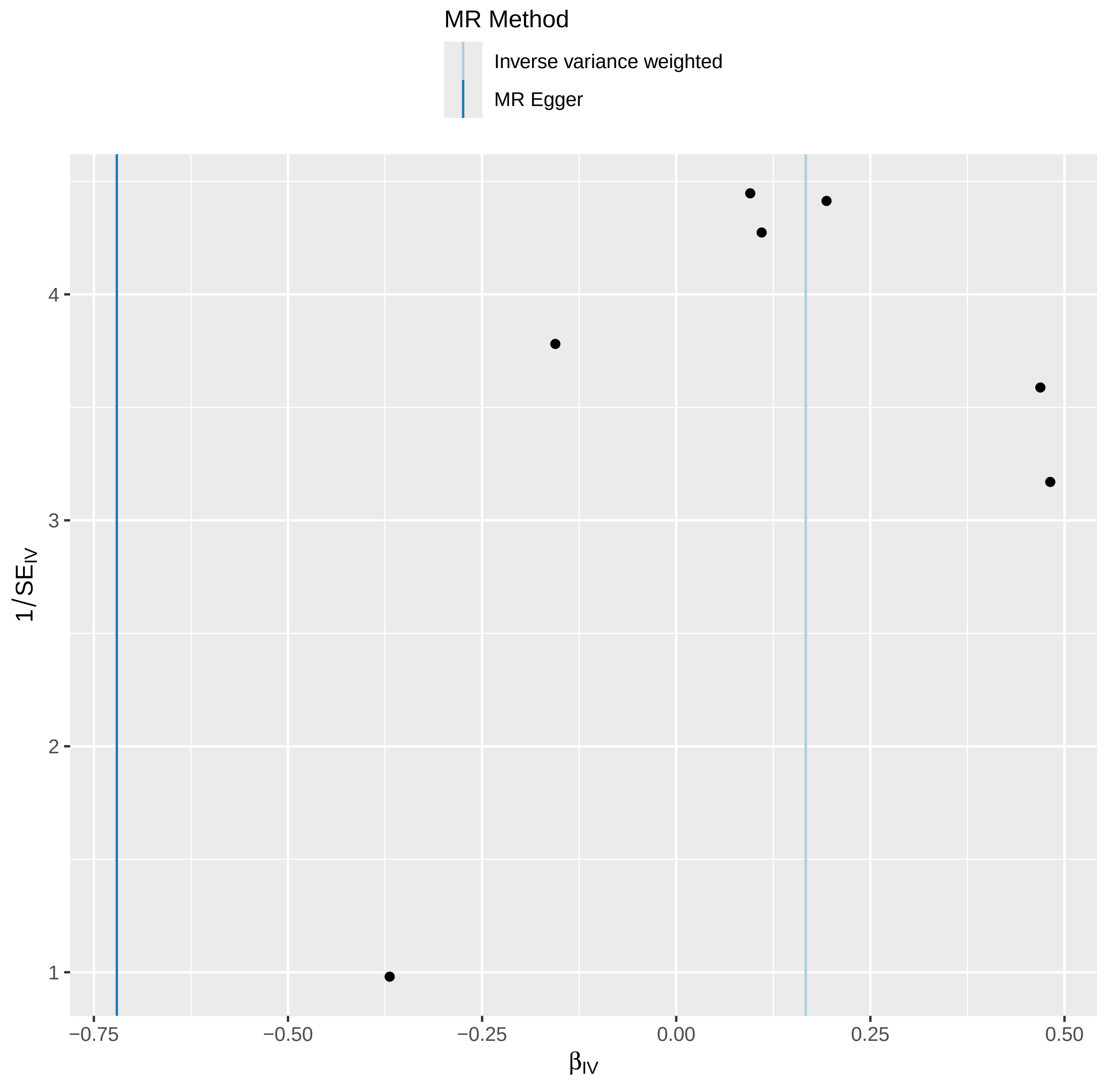
MR Method
Inverse variance weighted
MR Egger
4
3
2
1
1/SE_IV
−0.75
−0.50
−0.25
0.00
0.25
0.50
β_IV

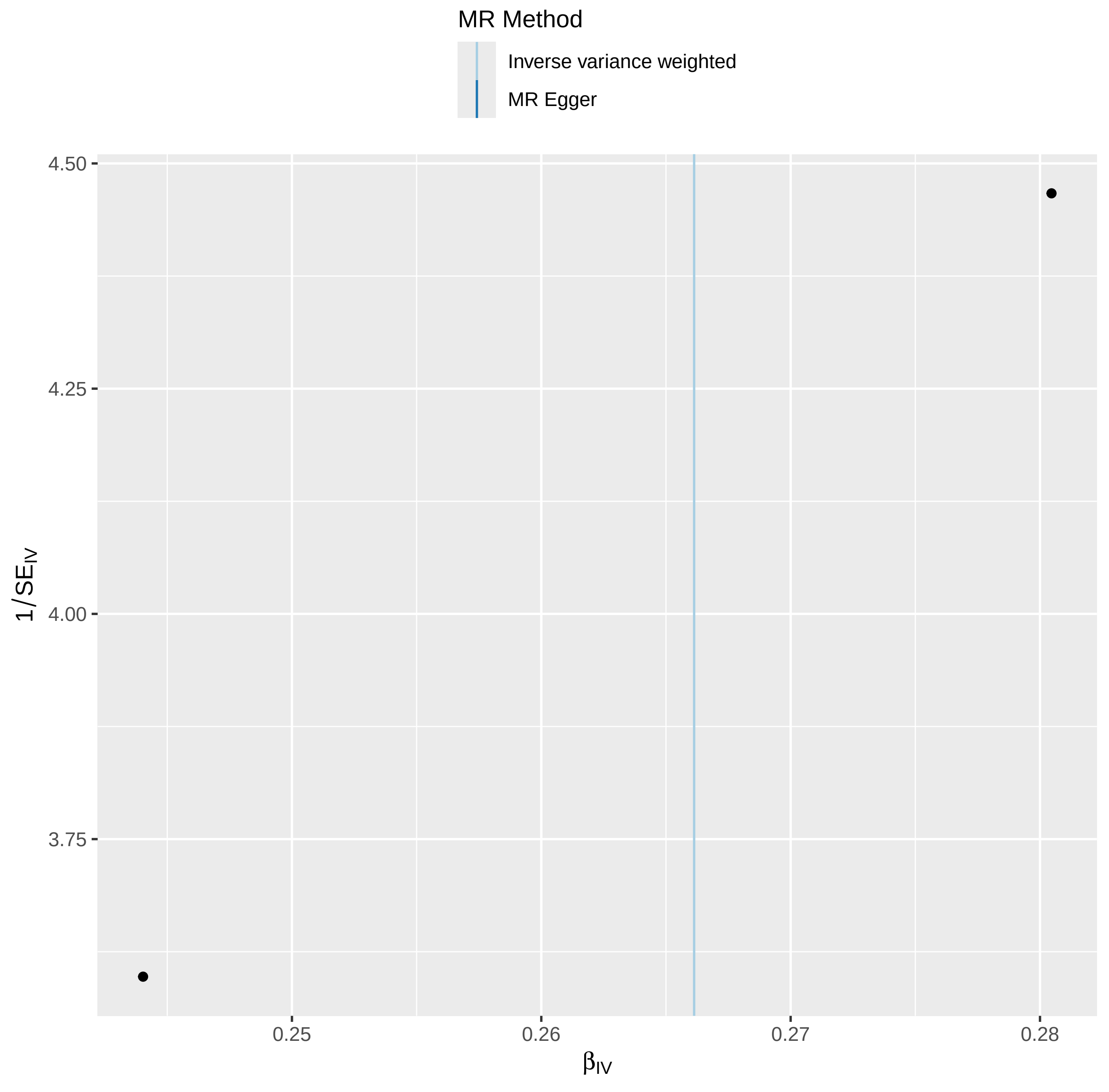

MR Method
Inverse variance weighted
MR Egger
4.50
4.25
4.00
3.75
1/SE_IV
0.25
0.26
0.27
0.28
β_IV

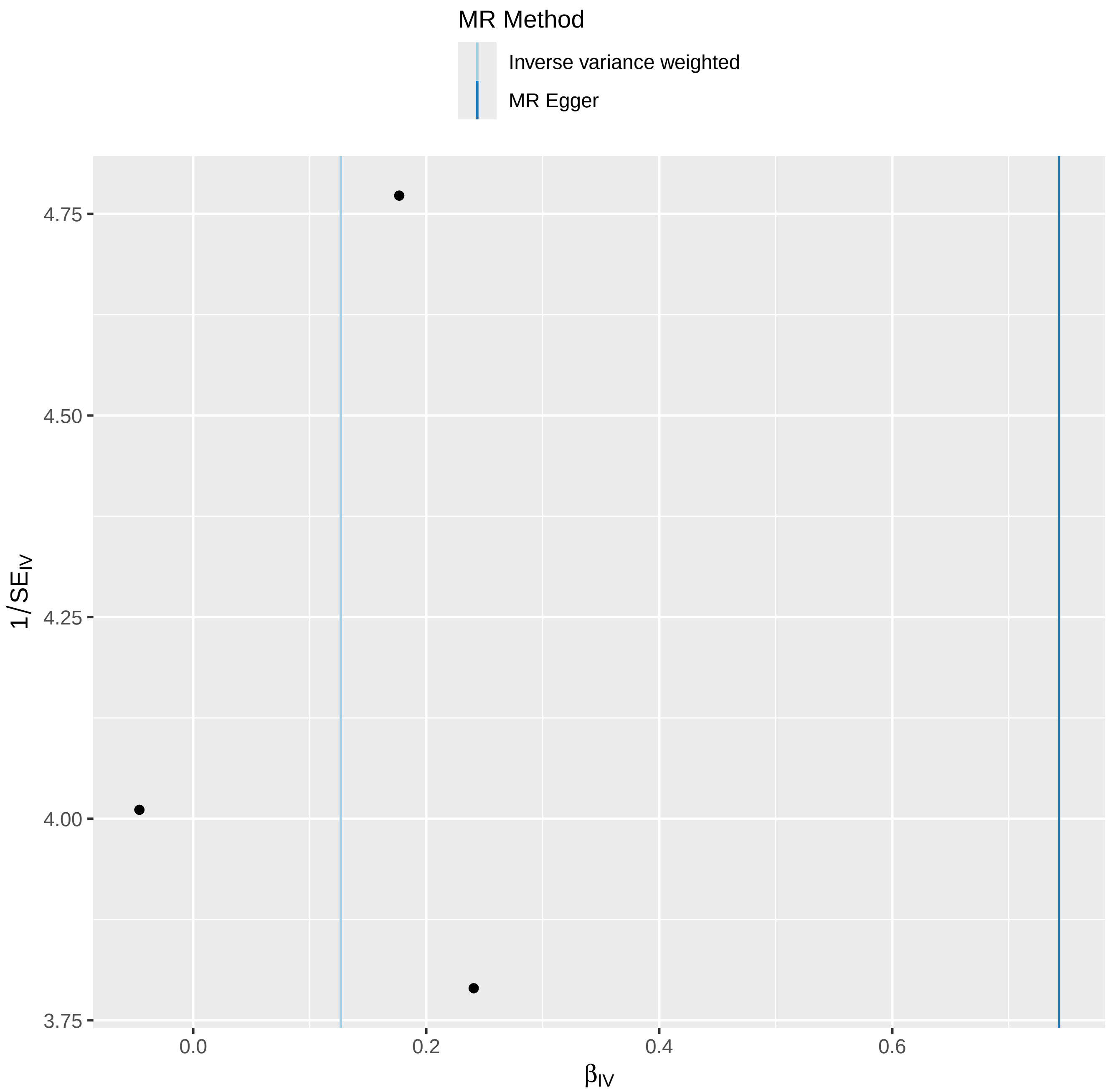
MR Method
Inverse variance weighted
MR Egger
1/SE_IV
4.75
4.50
4.25
4.00
3.75
0.0
0.2
0.4
0.6
β_IV

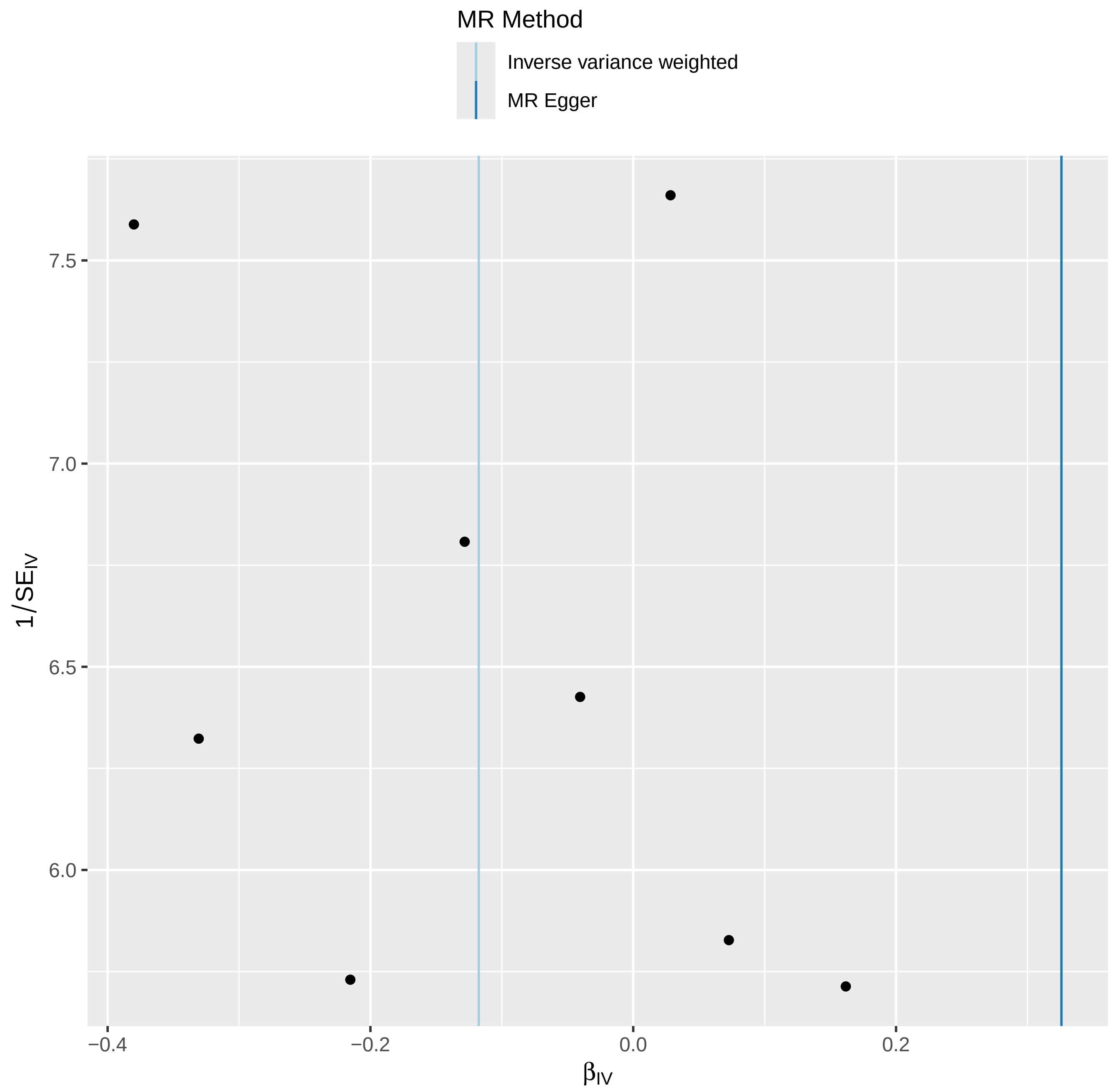
MR Method
Inverse variance weighted
MR Egger
7.5
7.0
6.5
6.0
1/SE_IV
−0.4
−0.2
0.0
0.2
β_IV

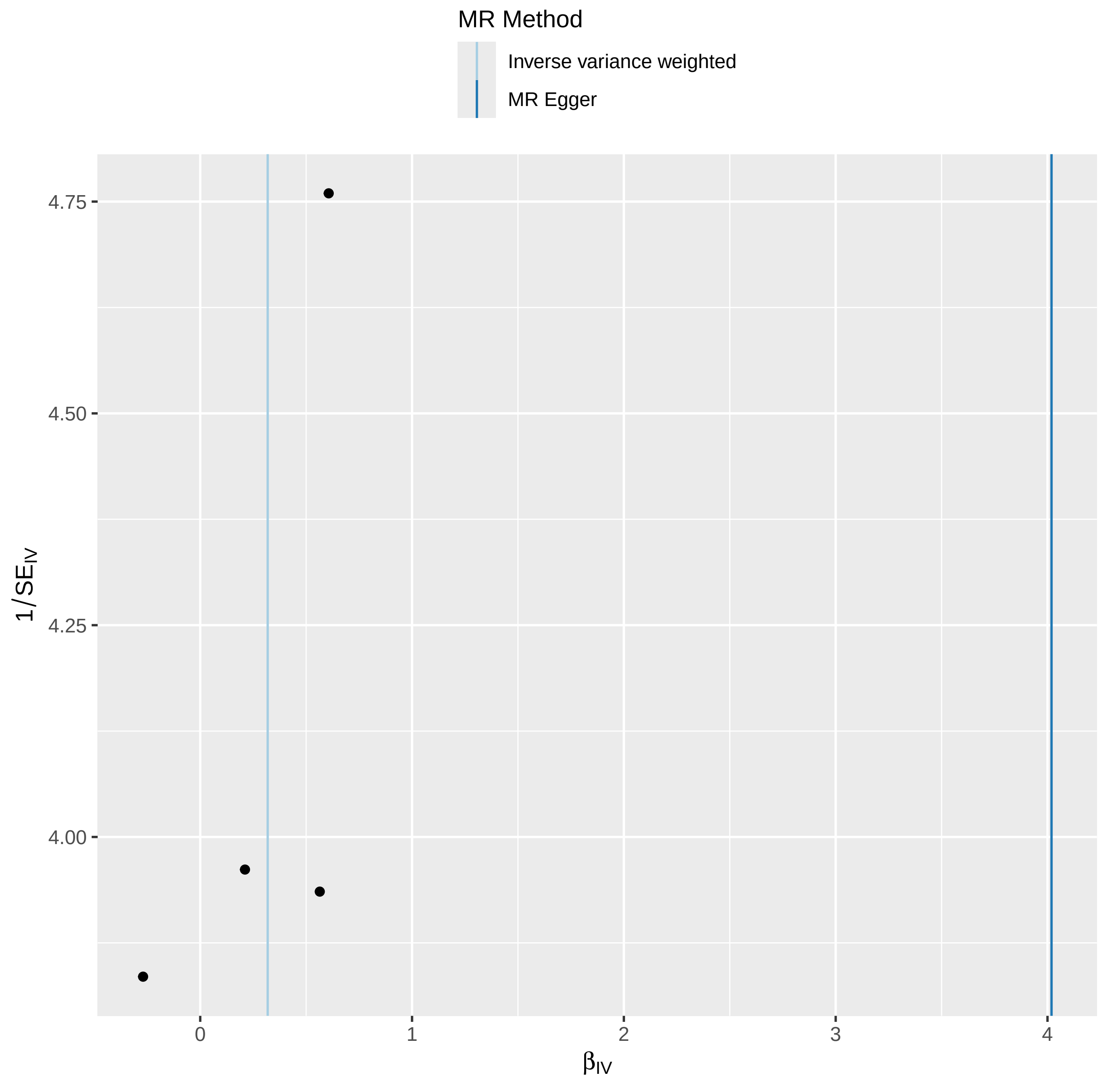

MR Method
Inverse variance weighted
MR Egger
4.75
4.50
4.25
4.00
1/SE_IV
0
1
2
3
4
β_IV

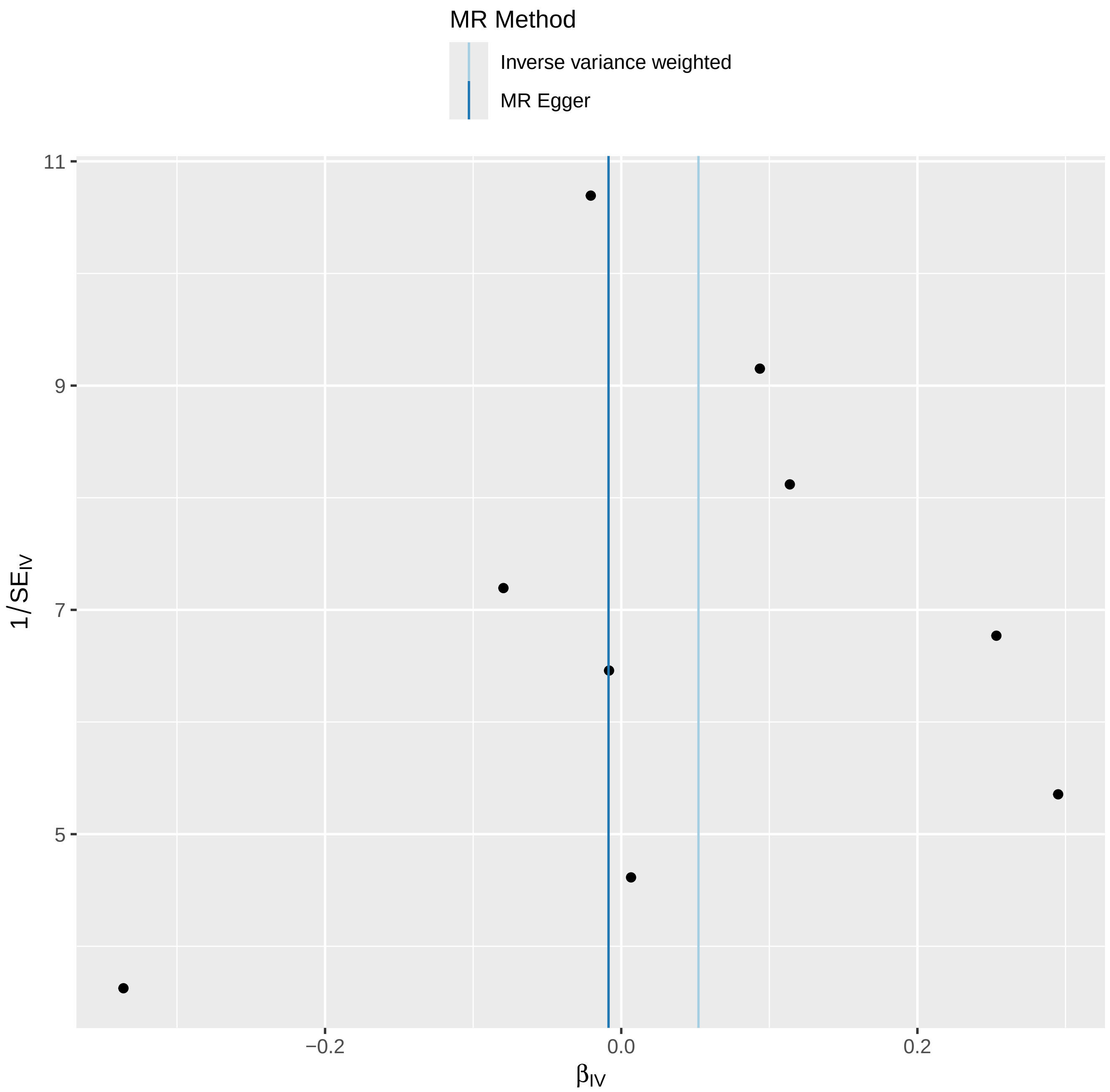

MR Method
Inverse variance weighted
MR Egger
11
9
7
5
$1/SE_{IV}$
−0.2
0.0
0.2
β_{IV}

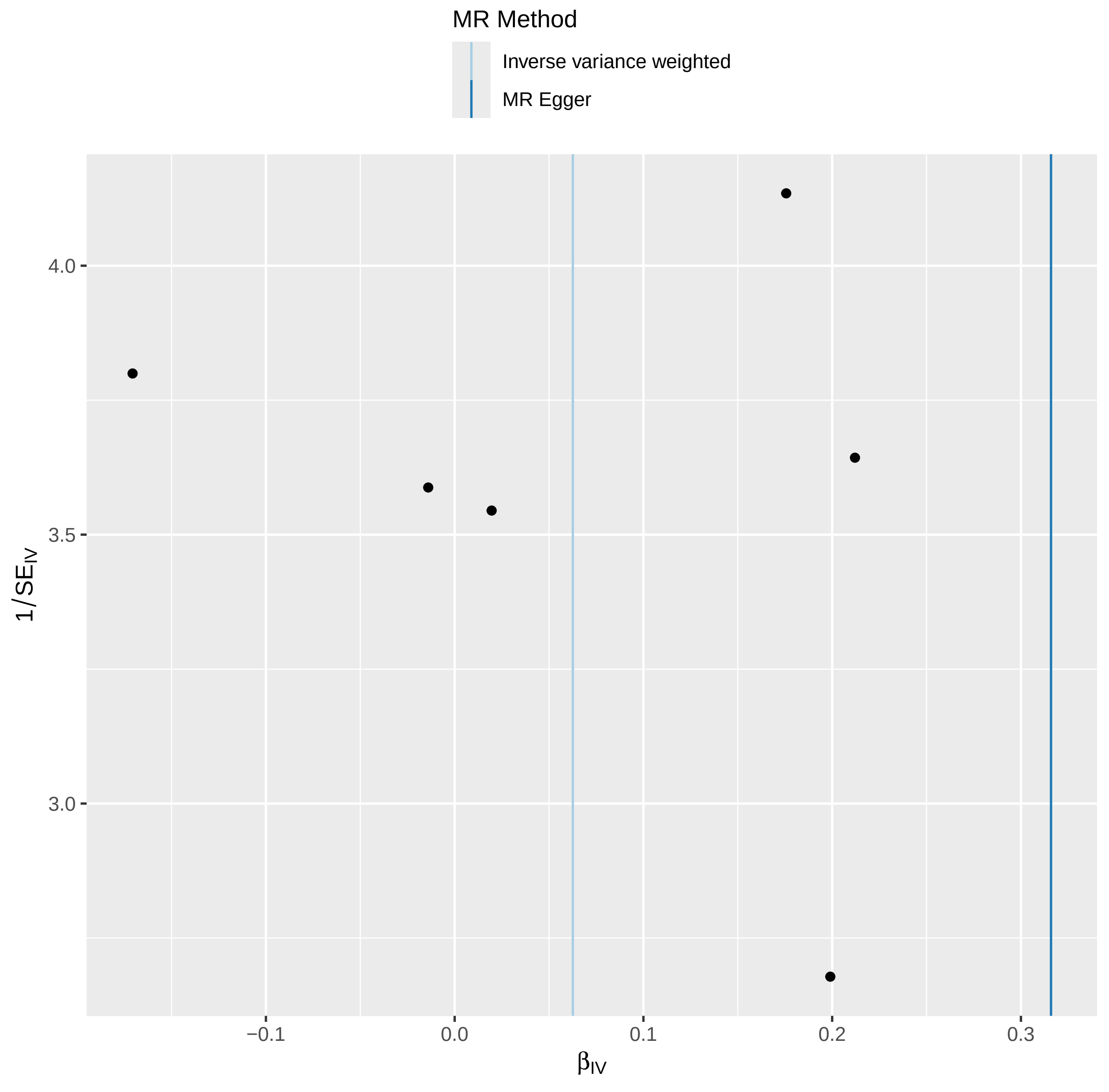
MR Method
Inverse variance weighted
MR Egger
4.0
3.5
3.0
1/SE_IV
−0.1
0.0
0.1
0.2
0.3
β_IV

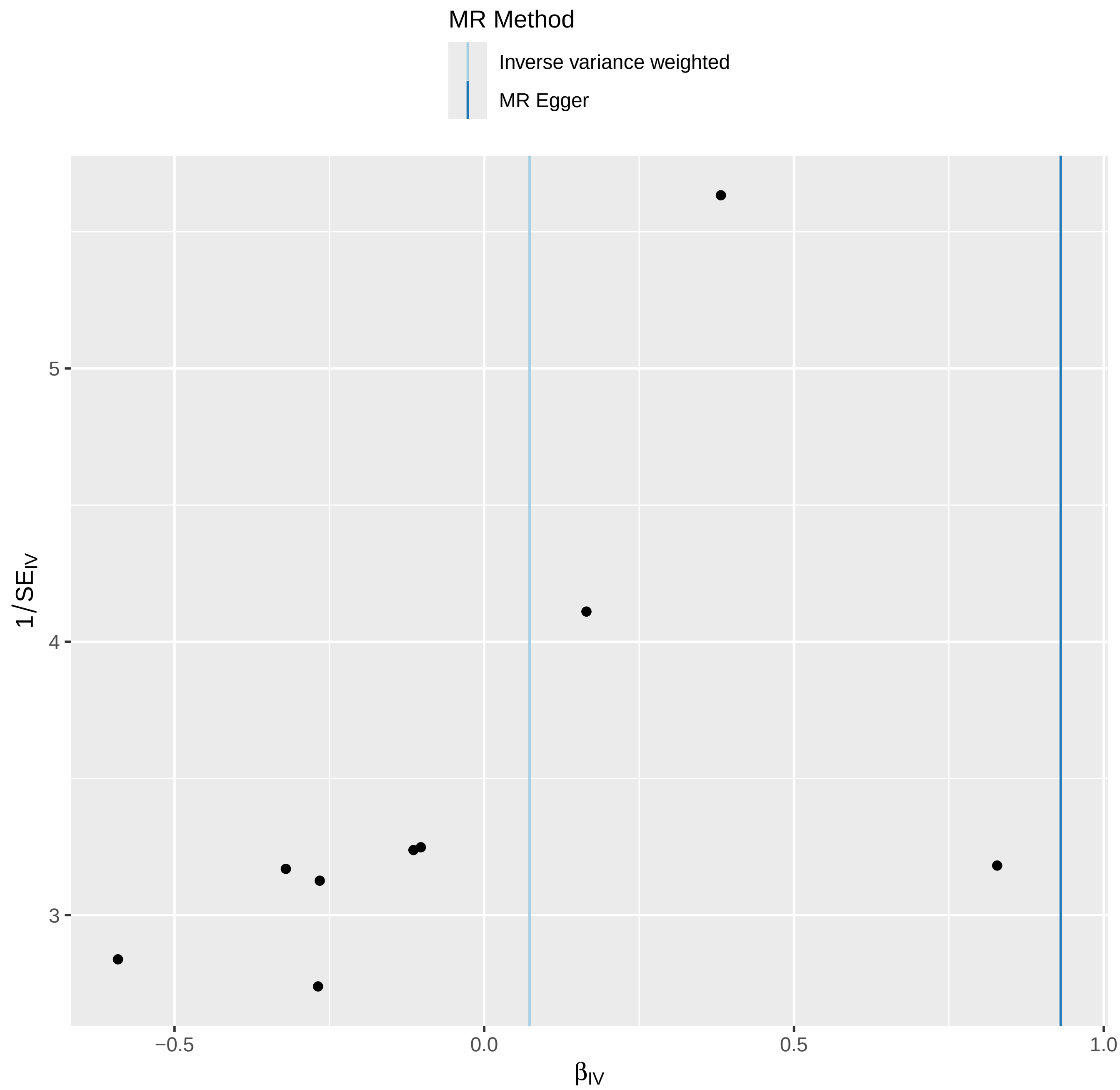

MR Method
Inverse variance weighted
MR Egger
5
4
3
$1/SE_{IV}$
−0.5
0.0
0.5
1.0
β_{IV}

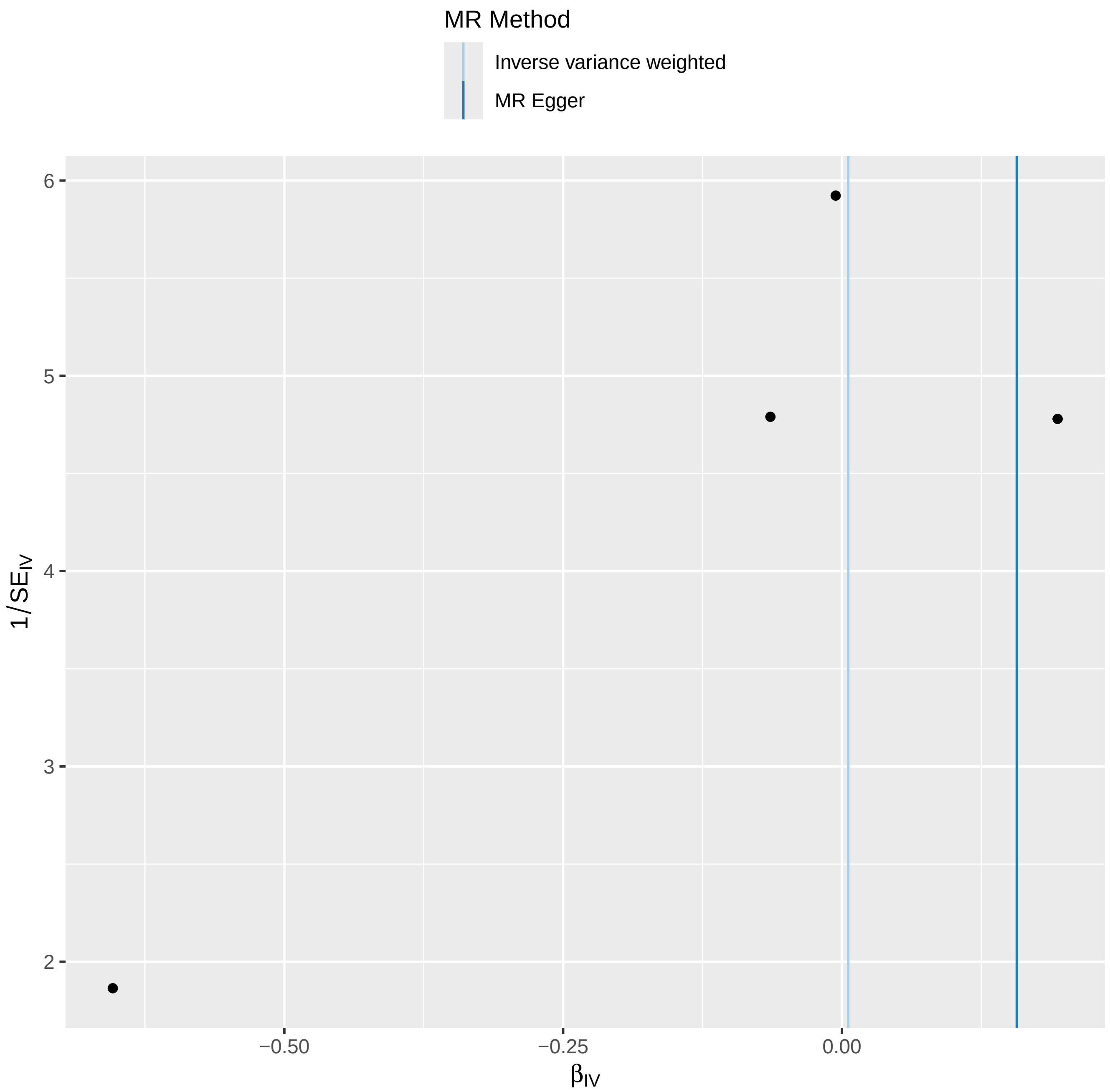
MR Method
Inverse variance weighted
MR Egger
6
5
4
3
2
1/SE_IV
−0.50
−0.25
0.00
β_IV

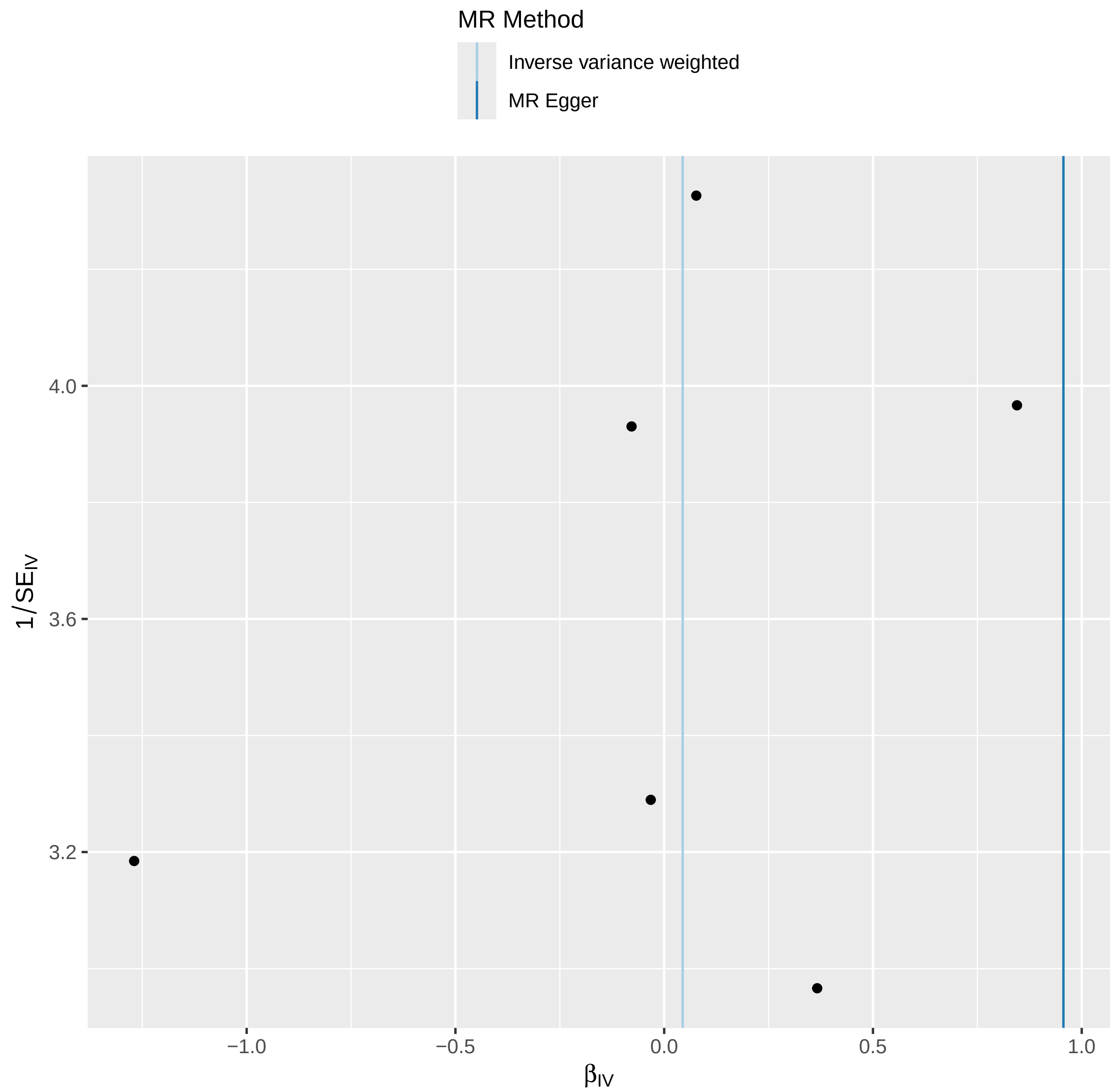
MR Method
Inverse variance weighted
MR Egger
4.0
3.6
3.2
1/SE_IV
−1.0
−0.5
0.0
0.5
1.0
β_IV

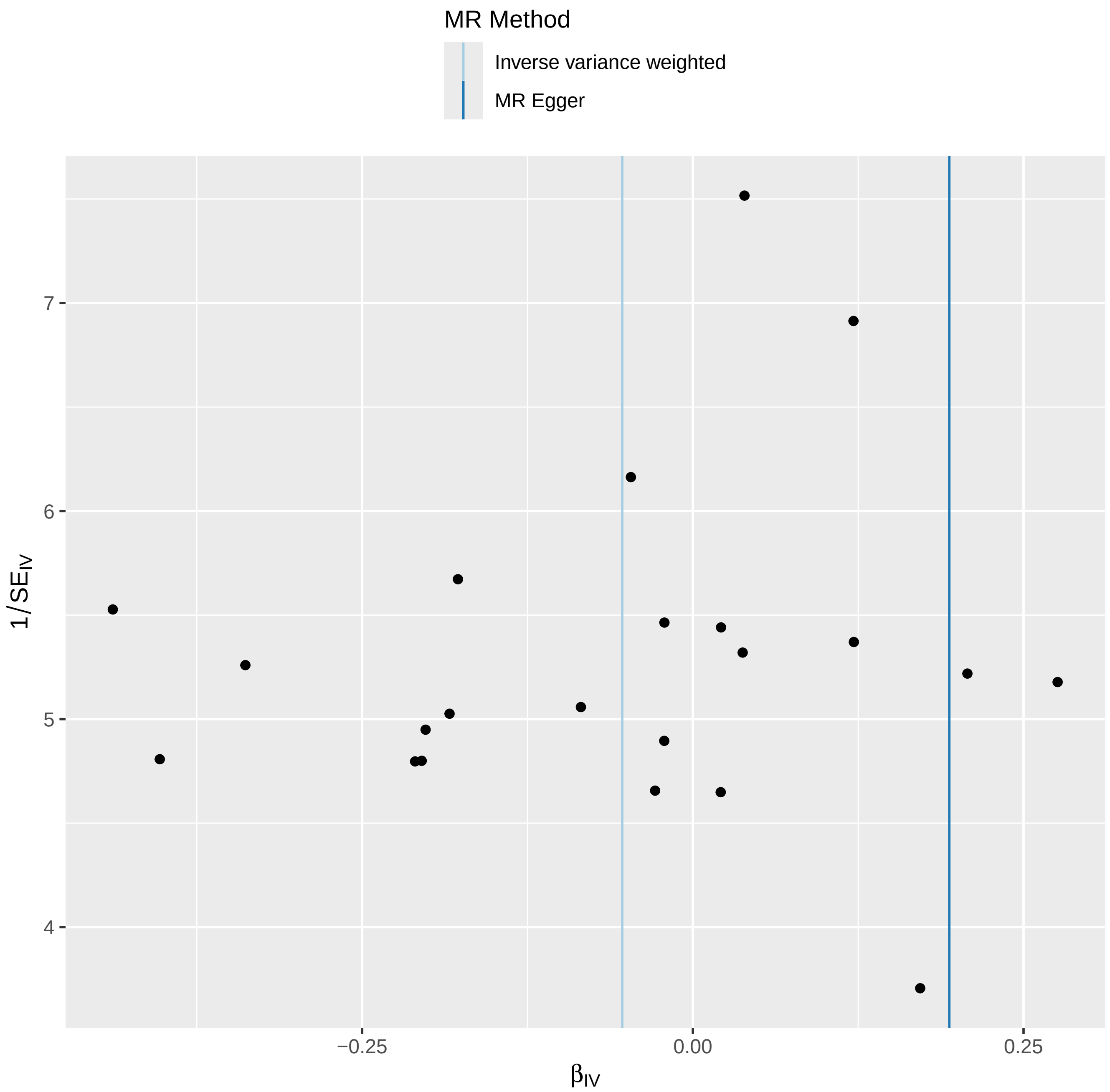
MR Method
Inverse variance weighted
MR Egger
7
6
5
4
−0.25
0.00
0.25
$1/SE_{IV}$
β_{IV}

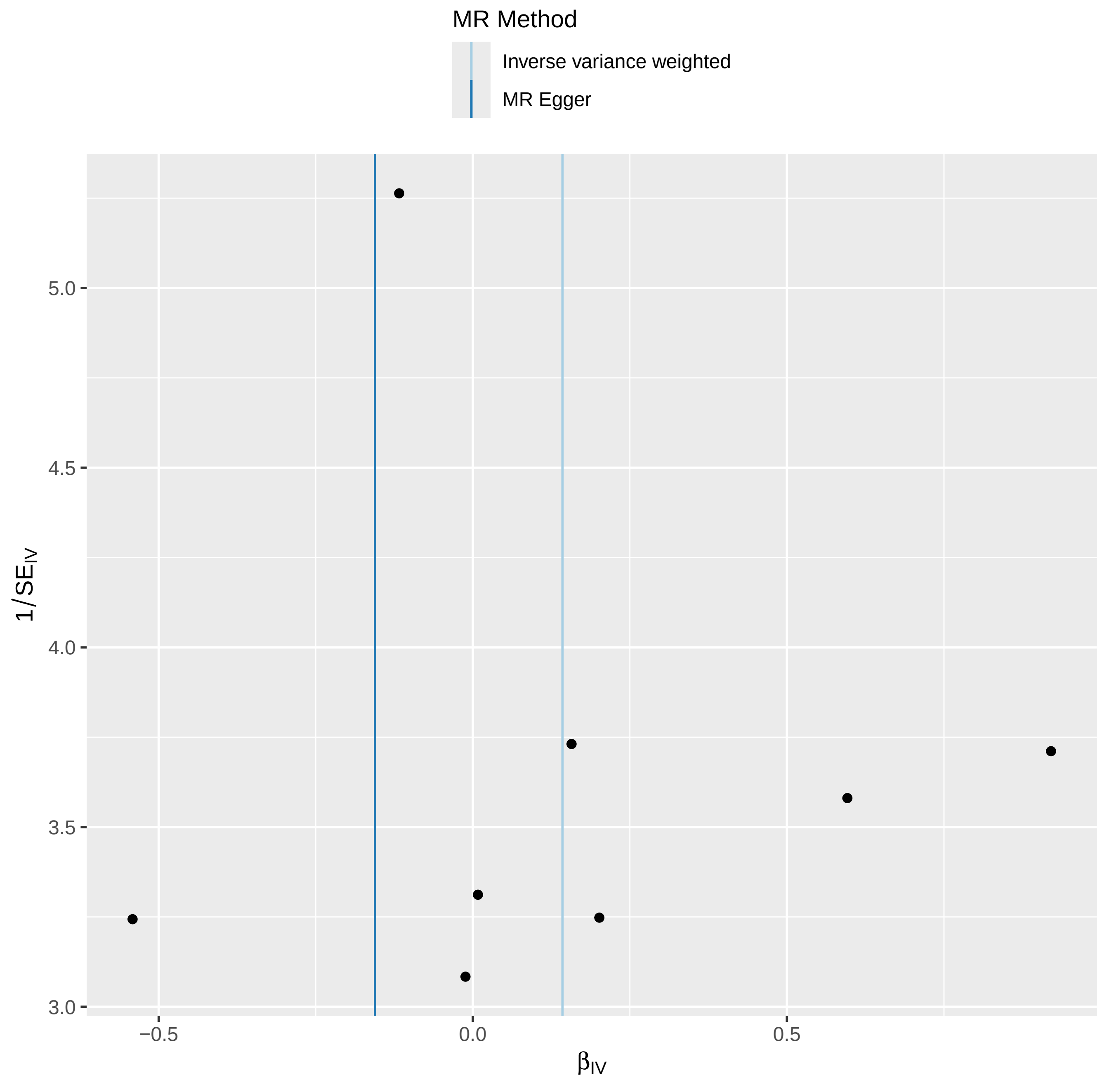

MR Method
Inverse variance weighted
MR Egger
5.0
4.5
4.0
3.5
3.0
1/SE_IV
−0.5
0.0
0.5
β_IV

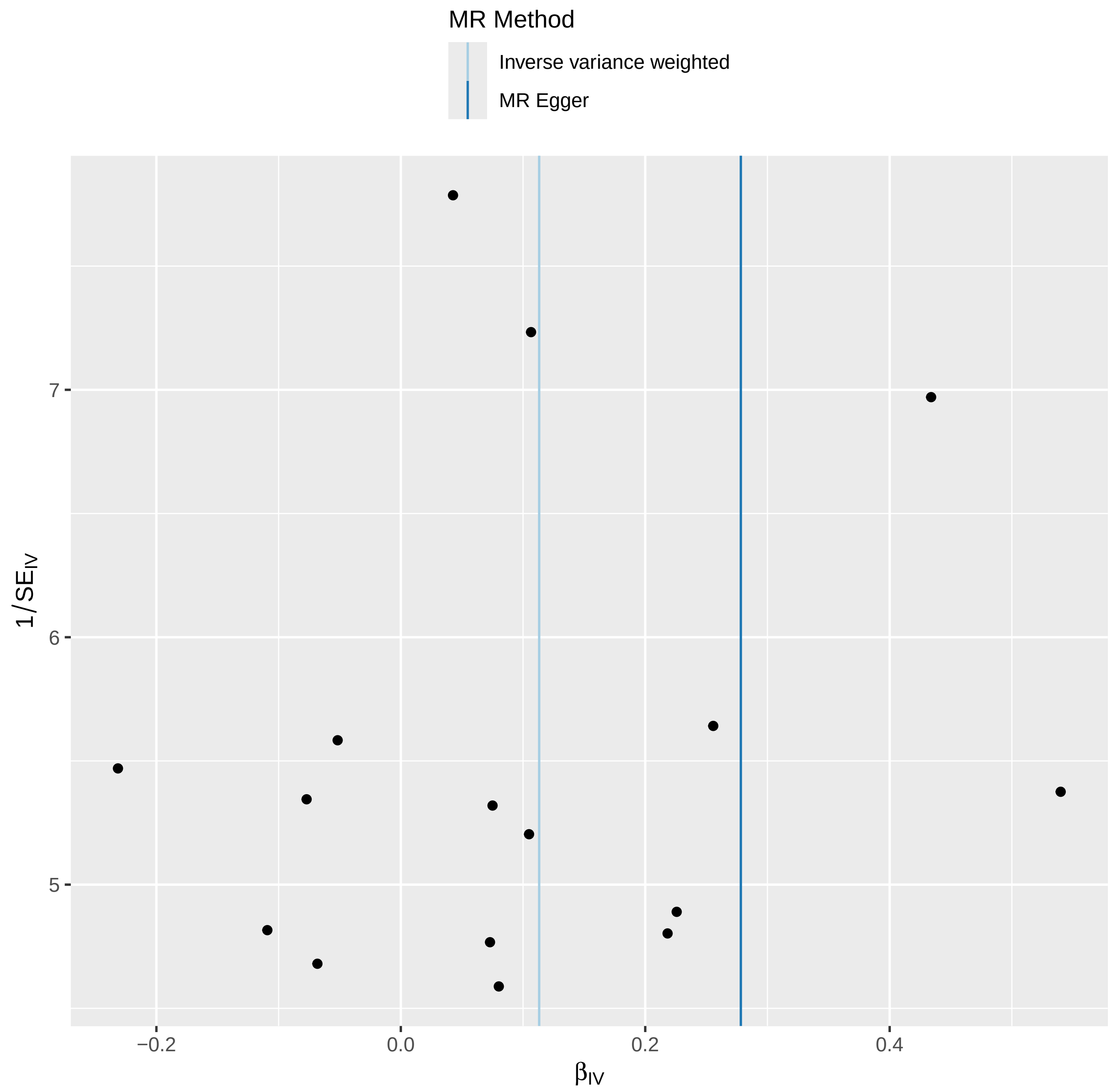
MR Method
Inverse variance weighted
MR Egger
1/SE_IV
7
6
5
−0.2
0.0
0.2
0.4
β_IV

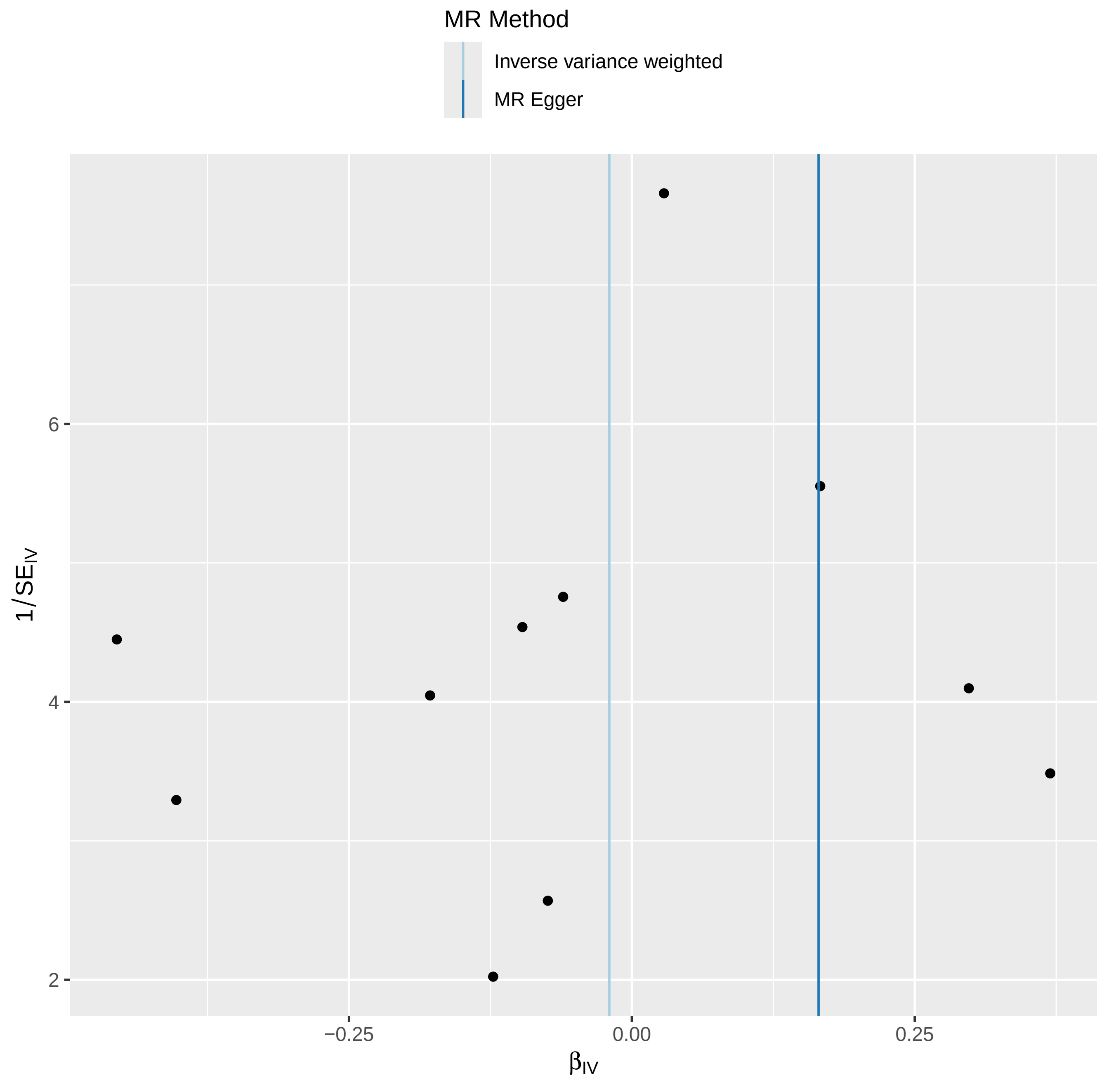

MR Method
Inverse variance weighted
MR Egger
6
4
2
1/SE_{IV}
−0.25
0.00
0.25
β_{IV}

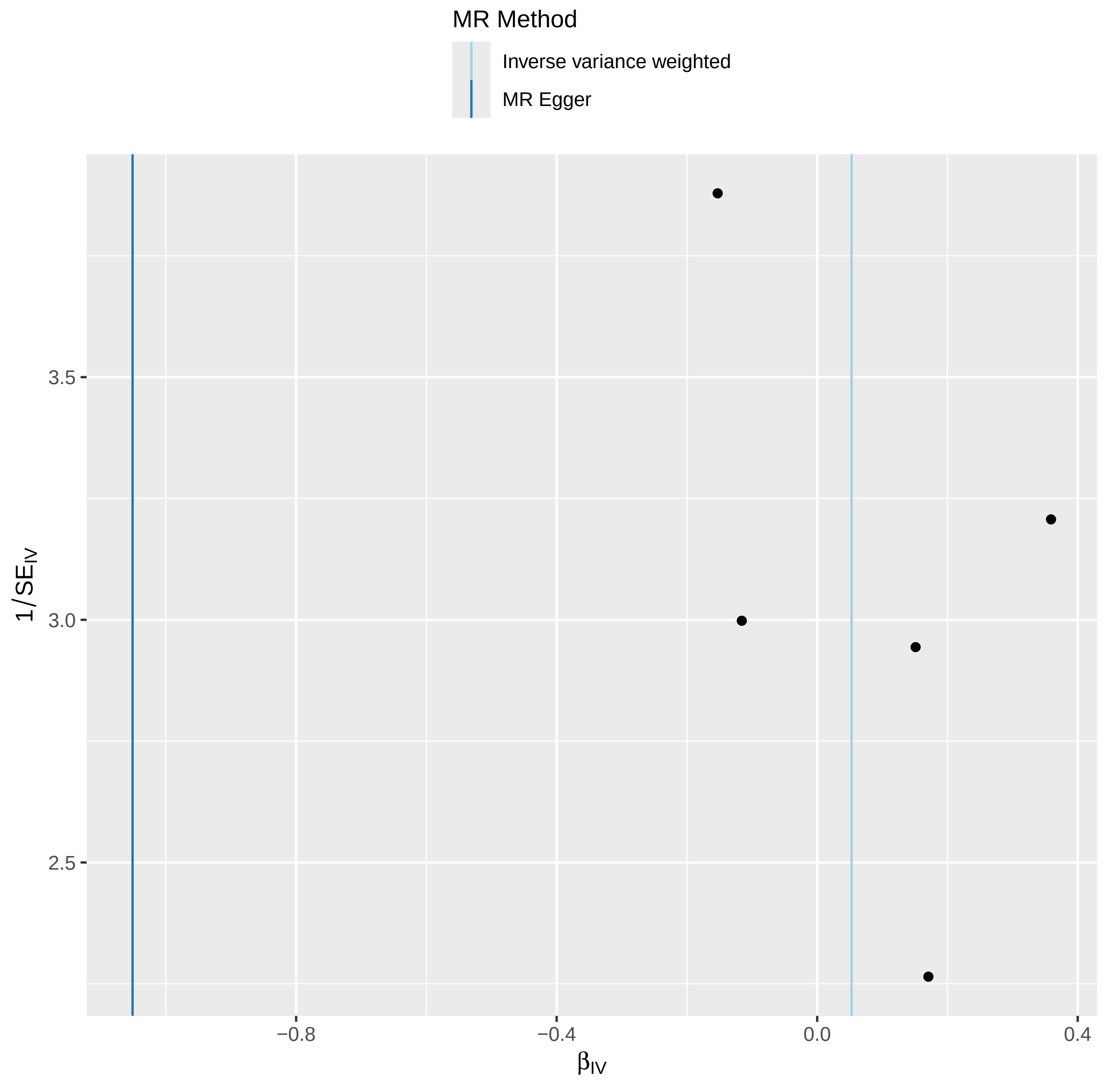

MR Method
Inverse variance weighted
MR Egger
3.5
3.0
2.5
1/SE_IV
−0.8
−0.4
0.0
0.4
β_IV

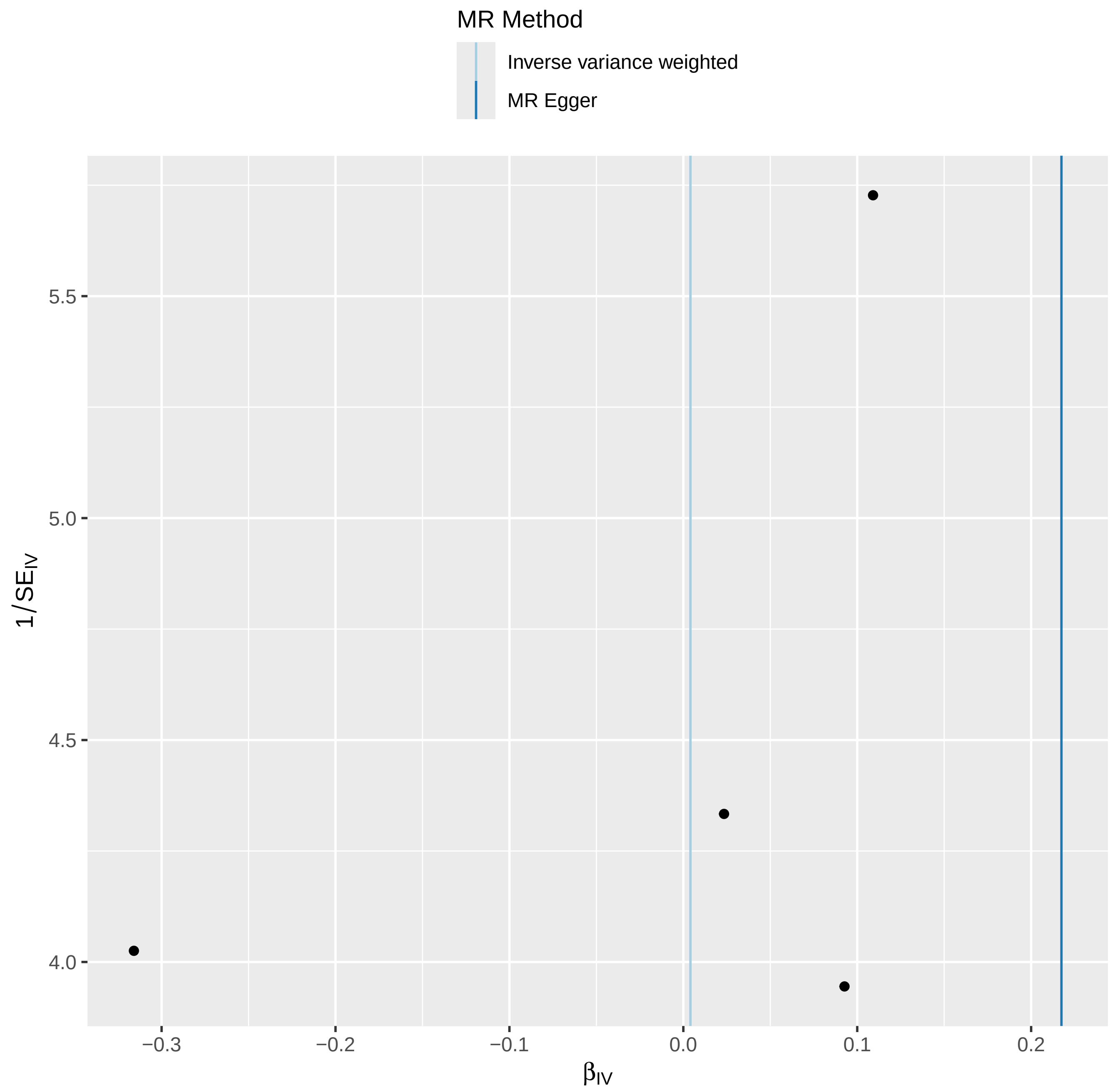
MR Method
Inverse variance weighted
MR Egger
5.5
5.0
4.5
4.0
$1/SE_{IV}$
−0.3
−0.2
−0.1
0.0
0.1
0.2
β_{IV}

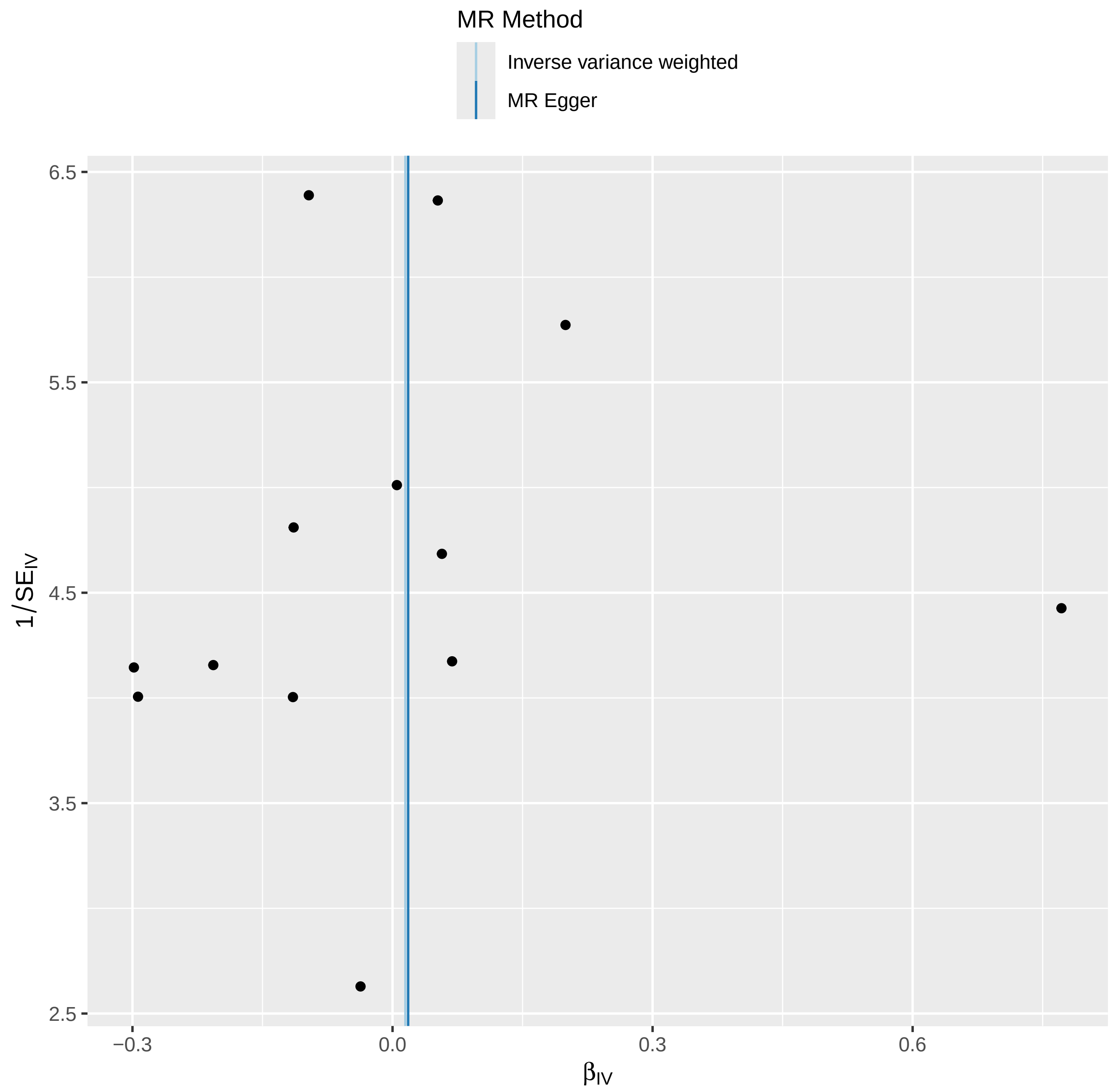
MR Method
Inverse variance weighted
MR Egger
6.5
5.5
4.5
3.5
2.5
$1/SE_{IV}$
−0.3
0.0
0.3
0.6
β_{IV}

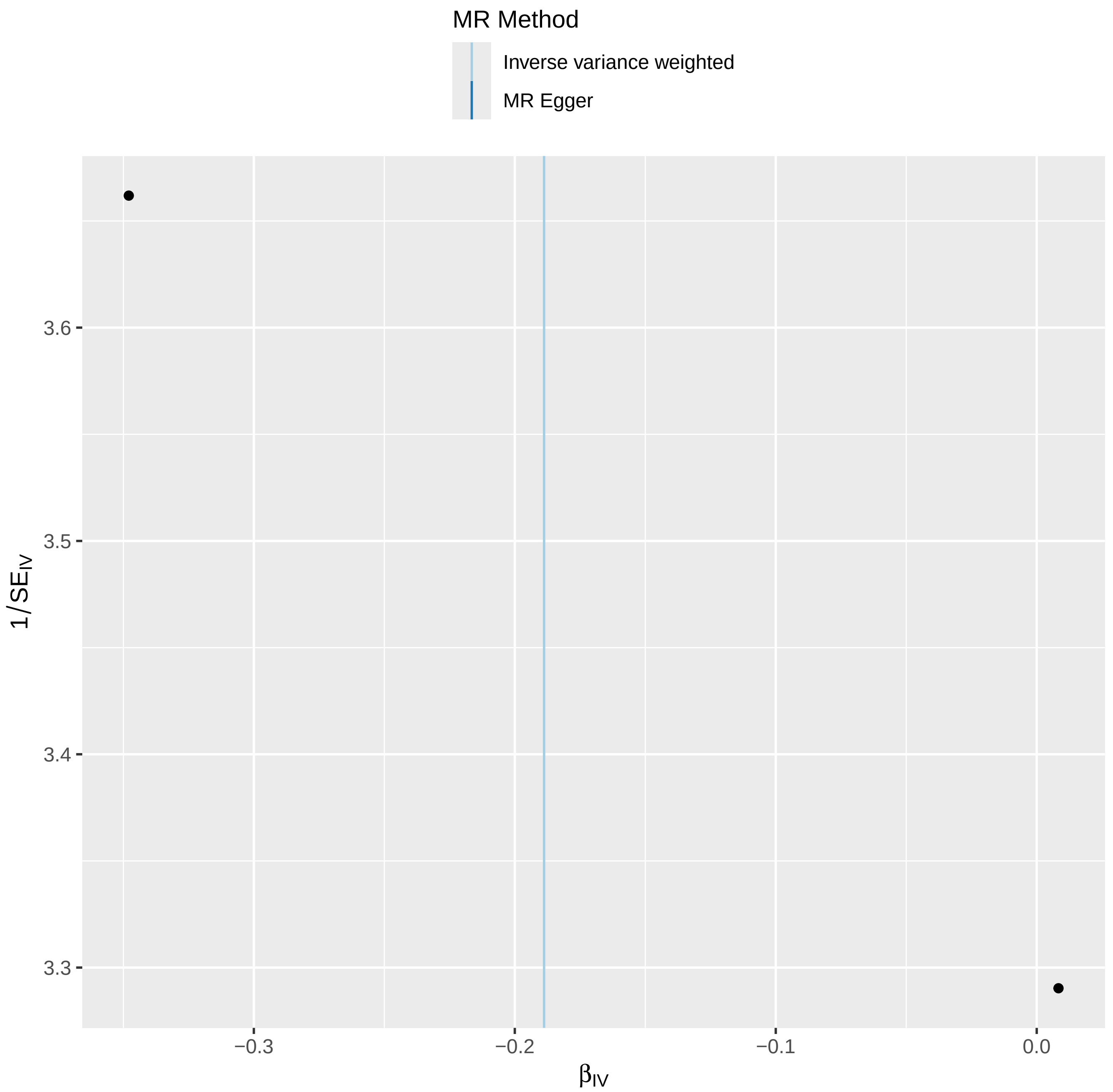
MR Method
Inverse variance weighted
MR Egger
3.6
3.5
3.4
3.3
1/SE_IV
−0.3
−0.2
−0.1
0.0
β_IV

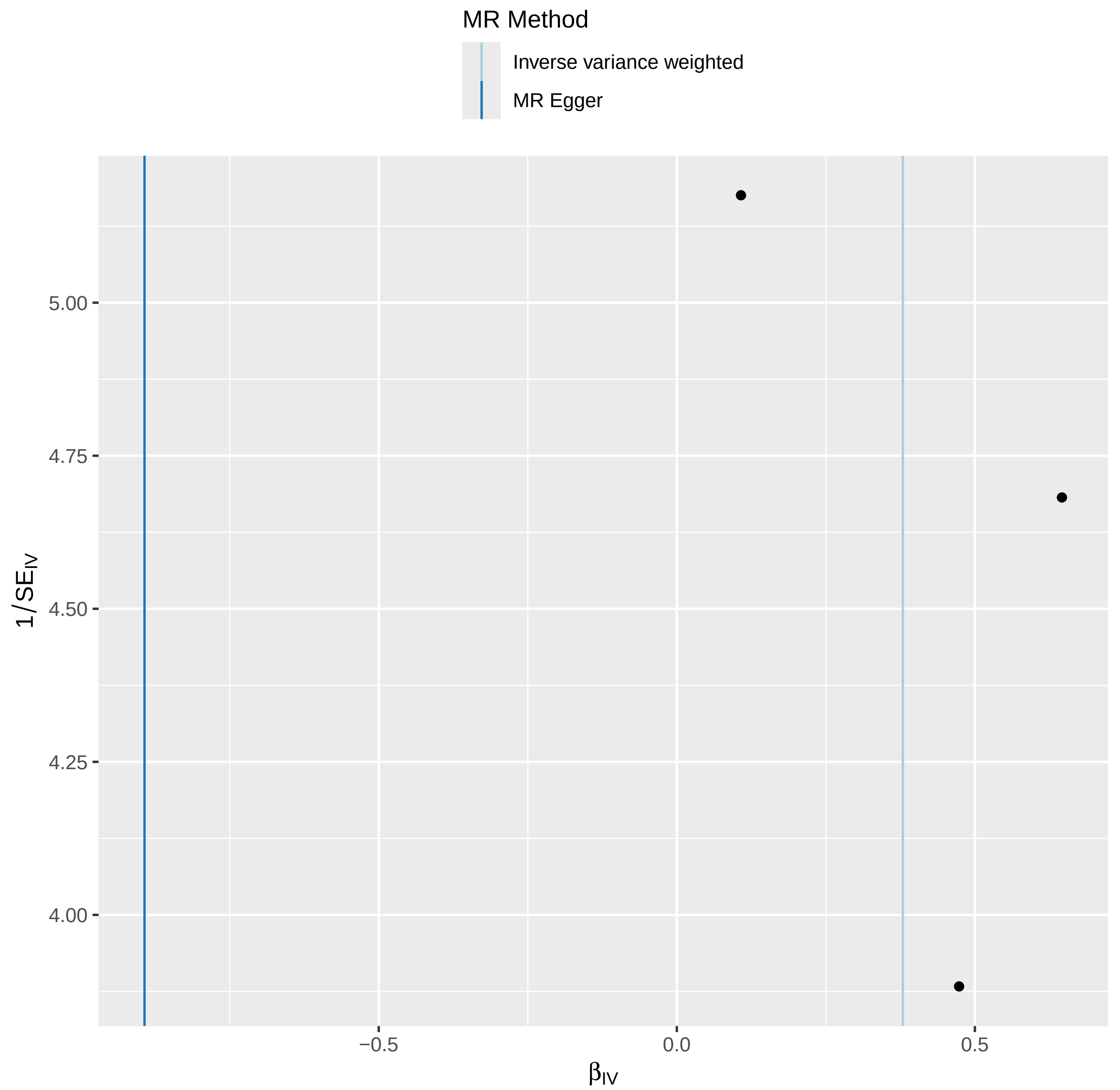
MR Method
Inverse variance weighted
MR Egger
5.00
4.75
4.50
4.25
4.00
1/SE_IV
−0.5
0.0
0.5
β_IV

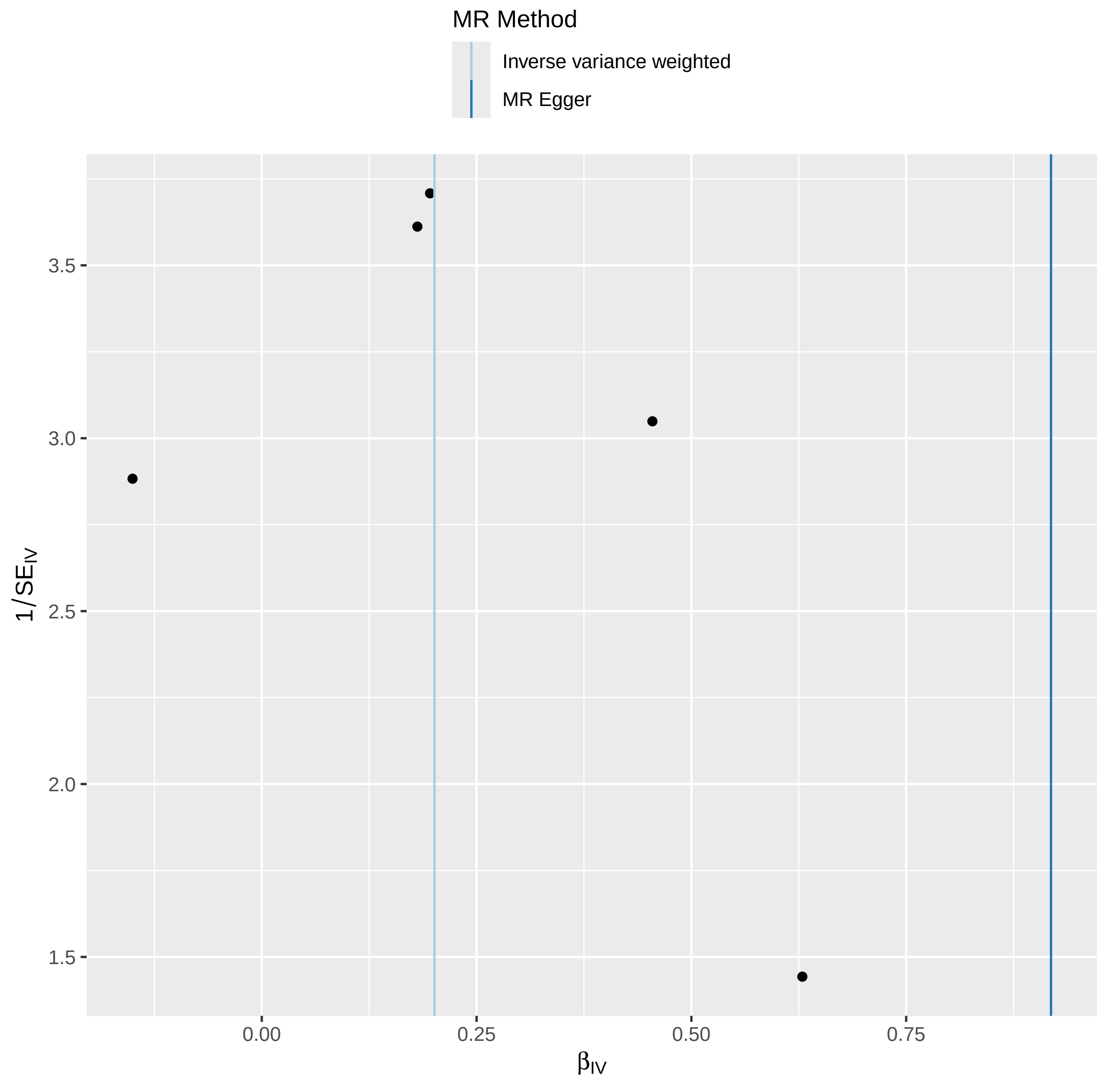
MR Method
Inverse variance weighted
MR Egger
3.5
3.0
2.5
2.0
1.5
$1/SE_{IV}$
0.00
0.25
0.50
0.75
β_{IV}

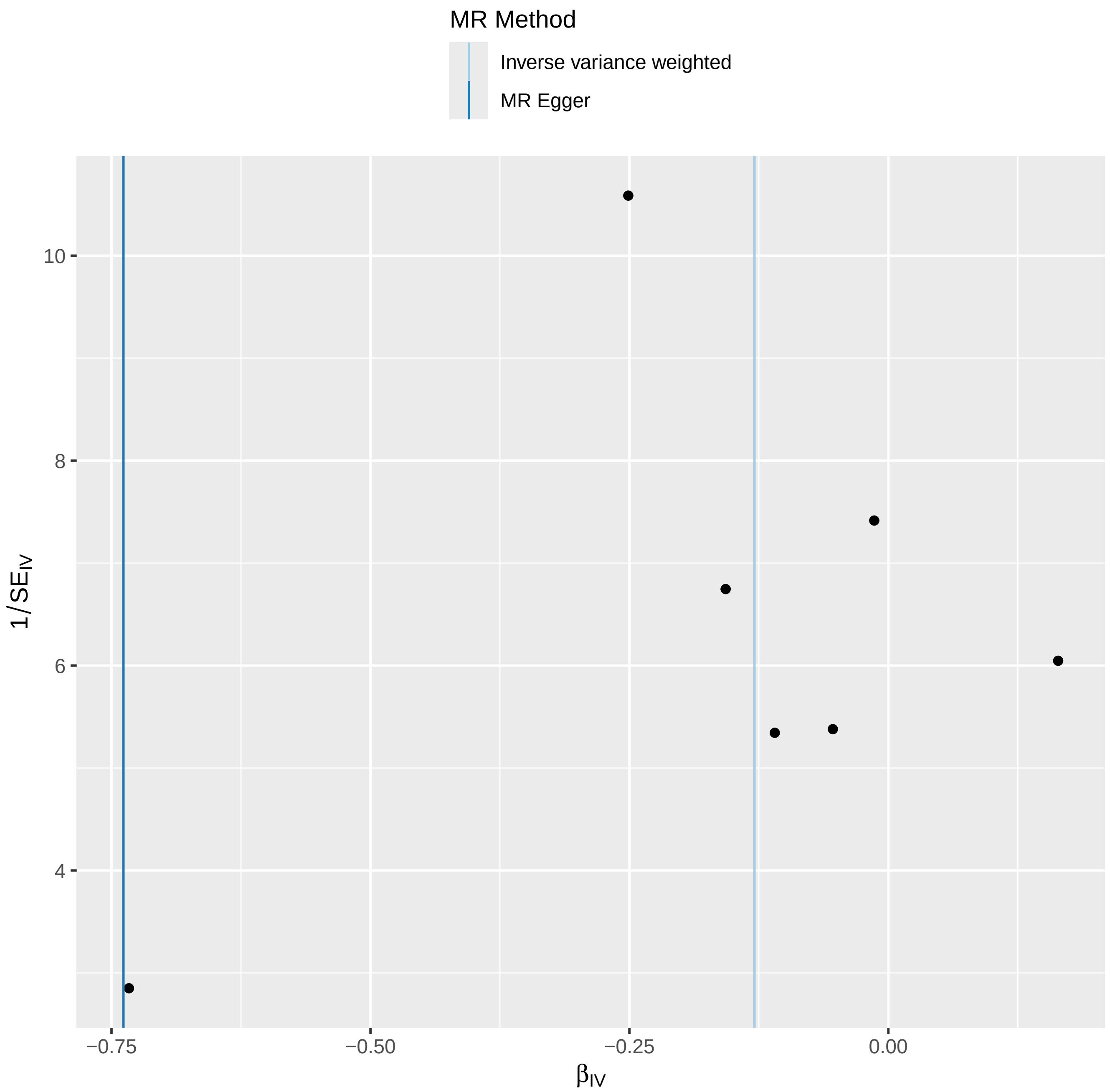

MR Method
Inverse variance weighted
MR Egger
10
8
6
4
1/SE_IV
−0.75
−0.50
−0.25
0.00
β_IV

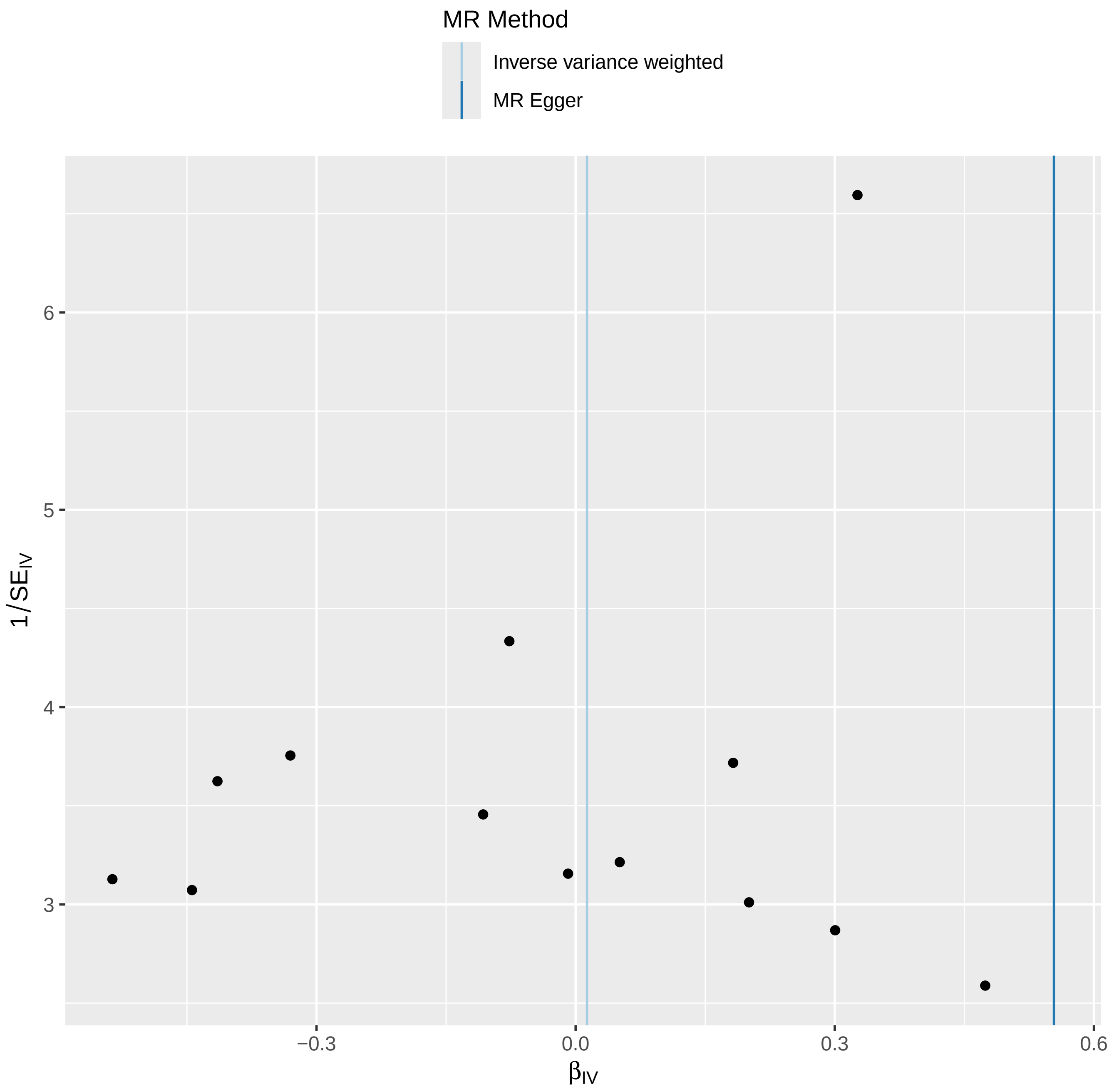

MR Method
Inverse variance weighted
MR Egger
6
5
4
3
1/SE$_{IV}$
−0.3
0.0
0.3
0.6
β$_{IV}$

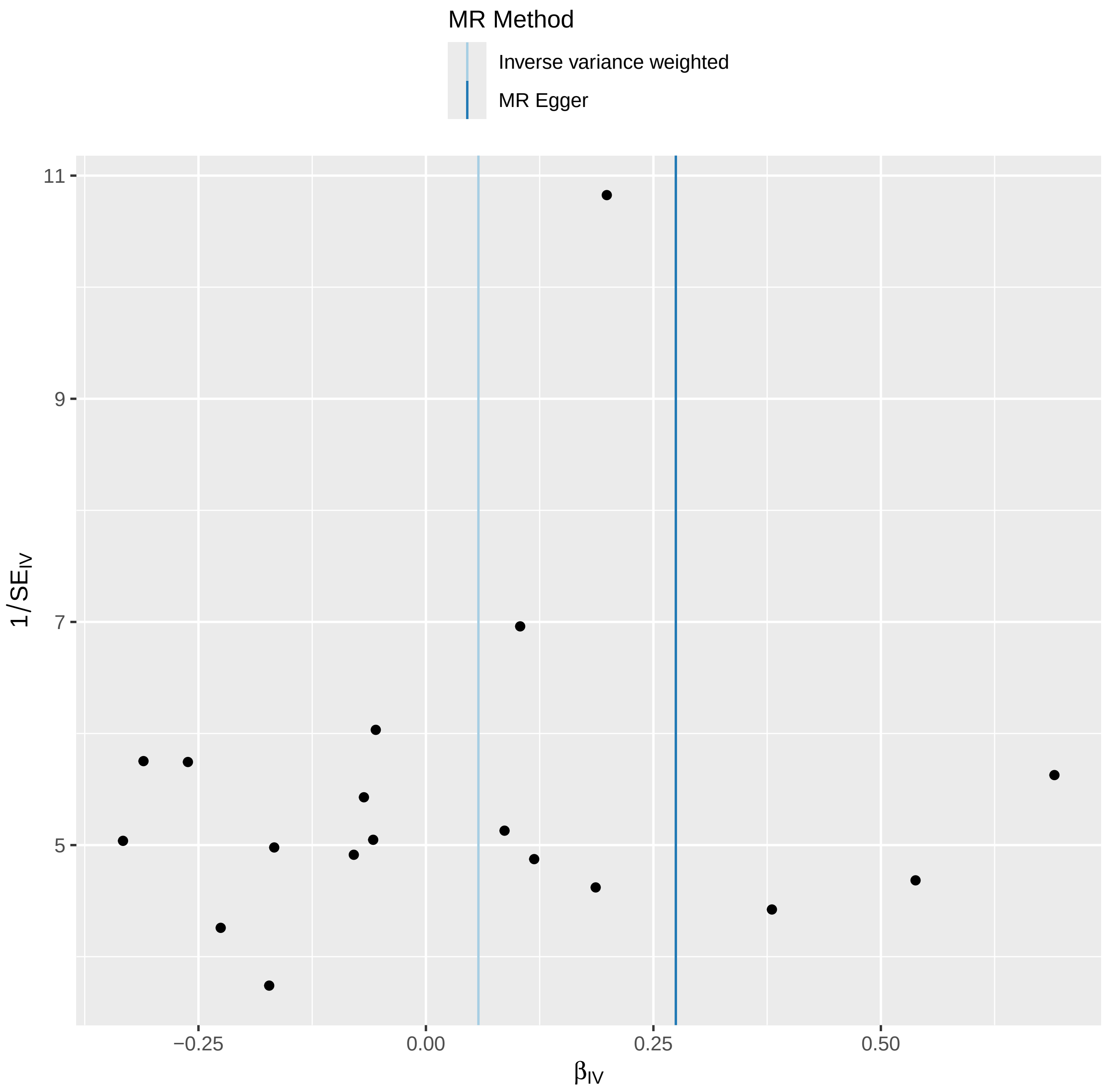
MR Method
Inverse variance weighted
MR Egger
11
9
7
5
$1/SE_{IV}$
−0.25
0.00
0.25
0.50
β_{IV}

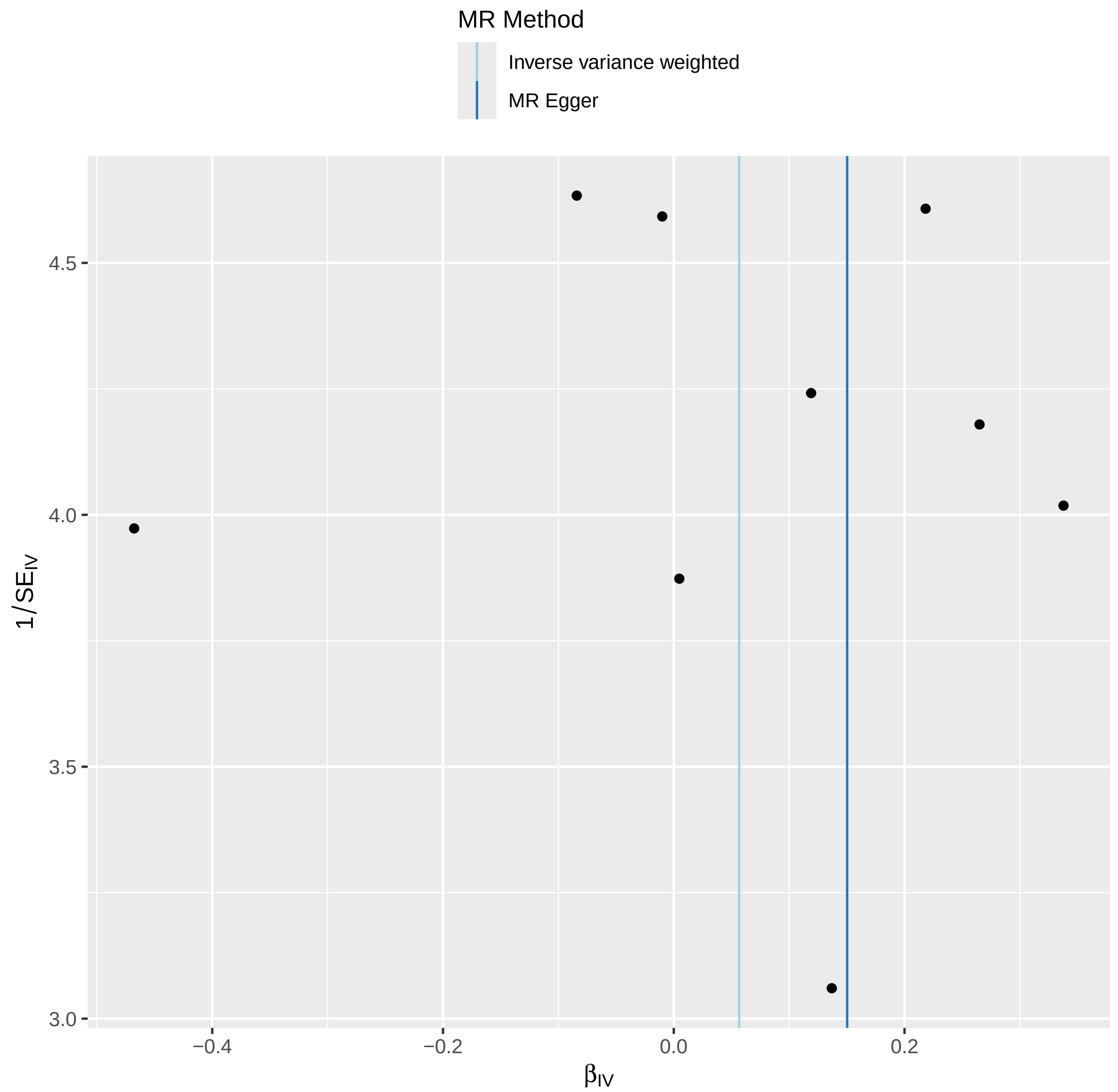

MR Method
Inverse variance weighted
MR Egger
4.5
4.0
3.5
3.0
1/SE_IV
−0.4
−0.2
0.0
0.2
β_IV

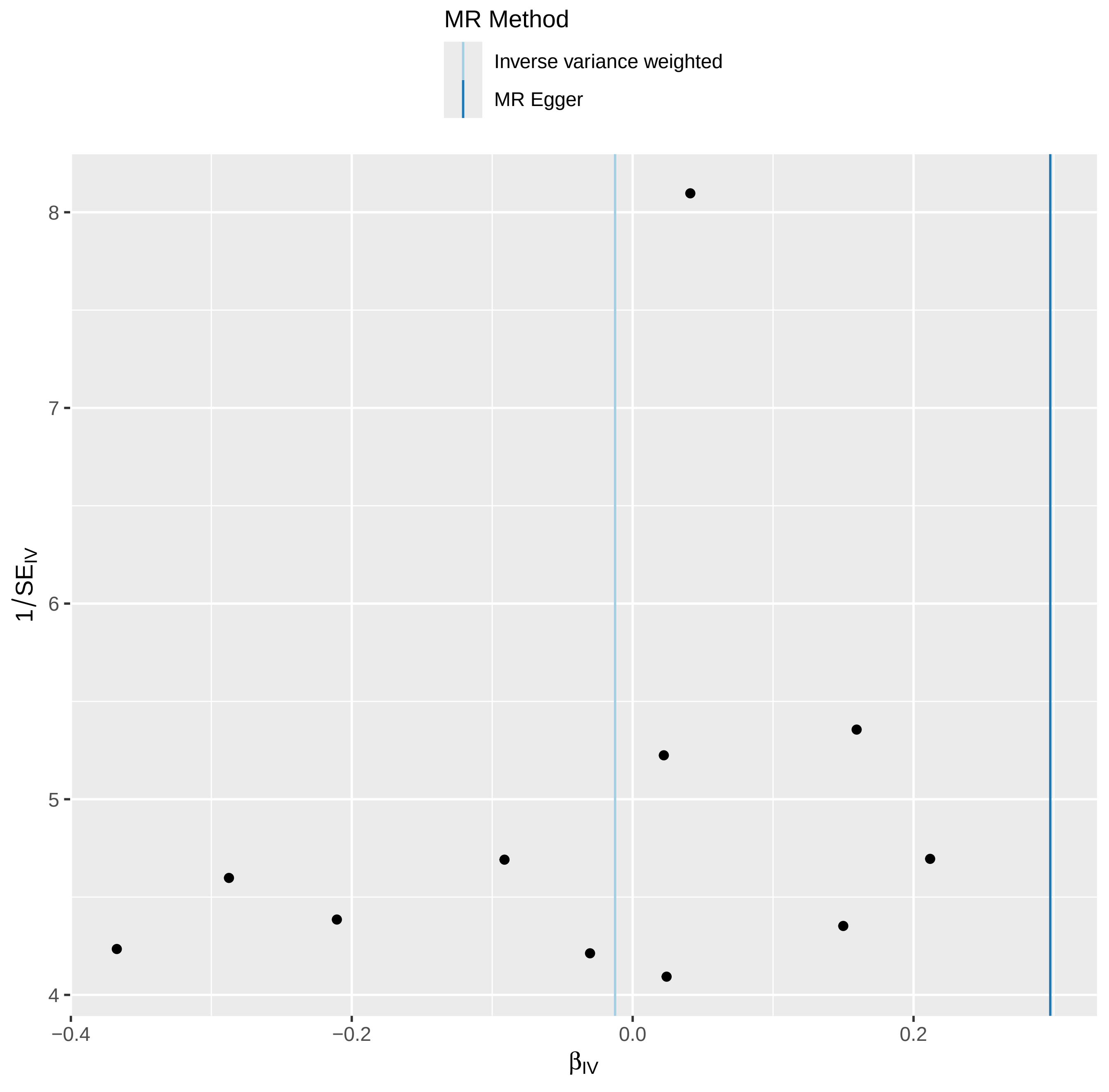
MR Method
Inverse variance weighted
MR Egger
1/SE_IV
β_IV
8
7
6
5
4
−0.4
−0.2
0.0
0.2

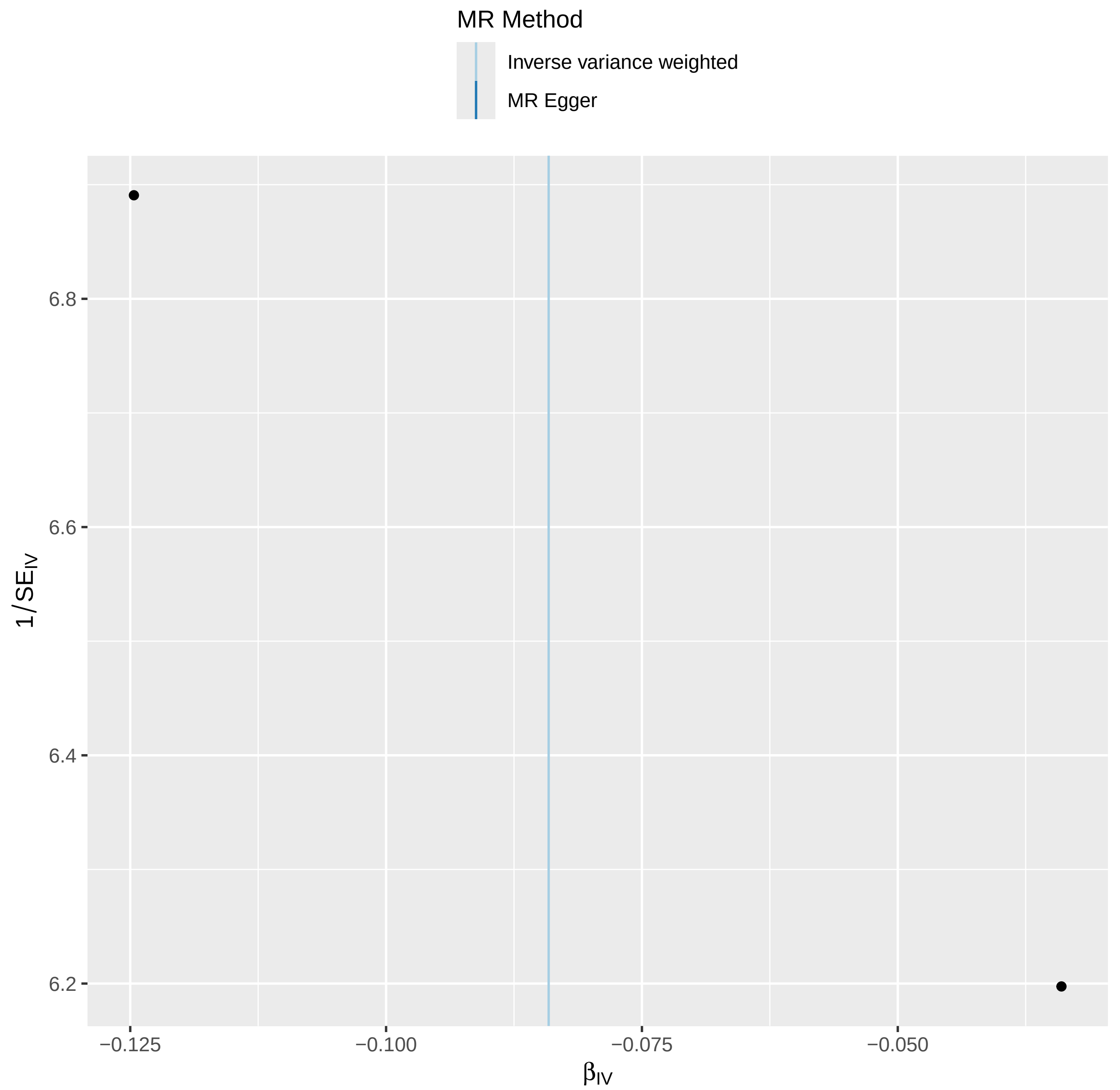
MR Method
Inverse variance weighted
MR Egger
6.8
6.6
6.4
6.2
$1/SE_{IV}$
−0.125
−0.100
−0.075
−0.050
β_{IV}

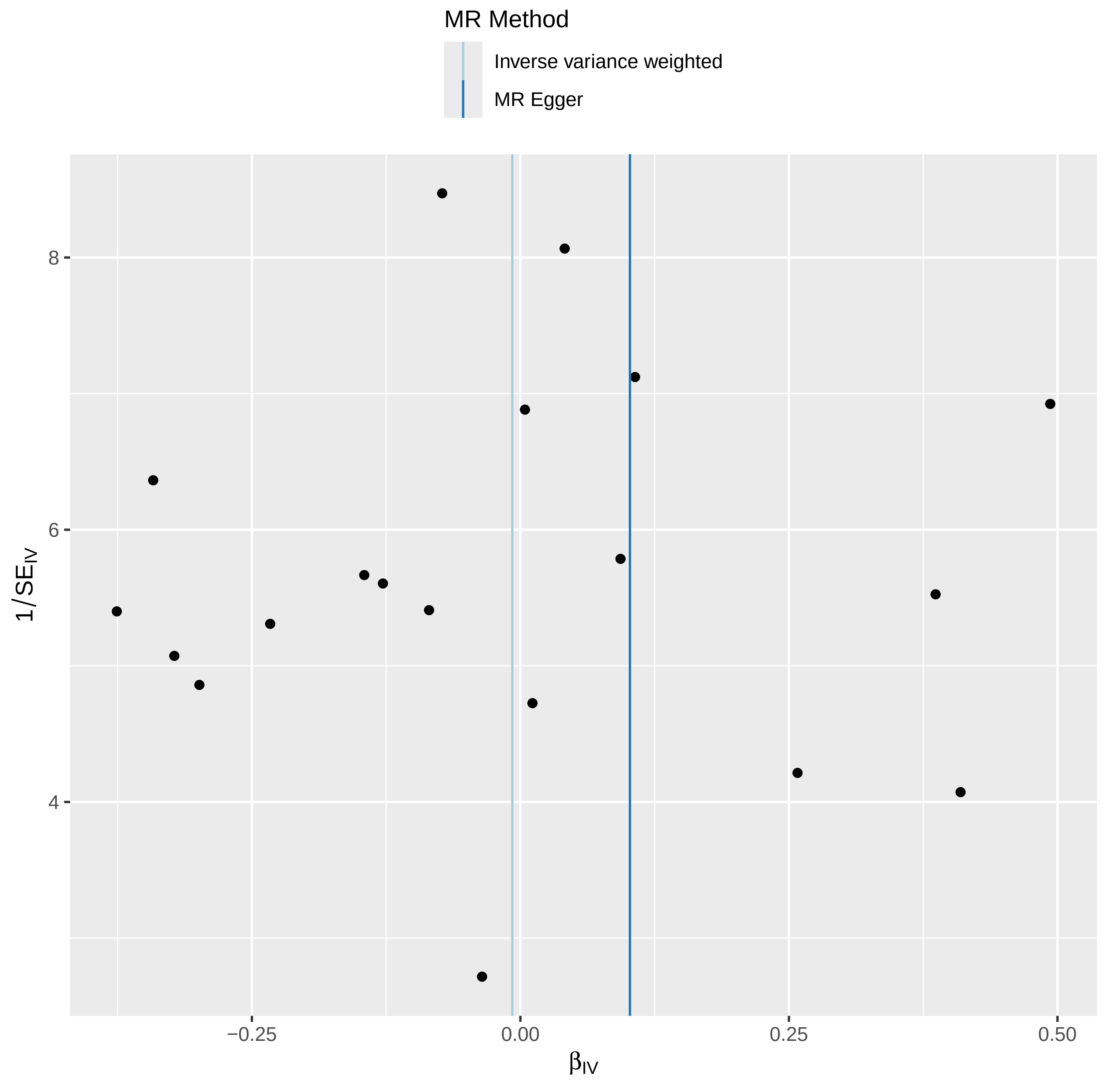
MR Method
Inverse variance weighted
MR Egger
8
6
4
1/SE_IV
−0.25
0.00
0.25
0.50
β_IV

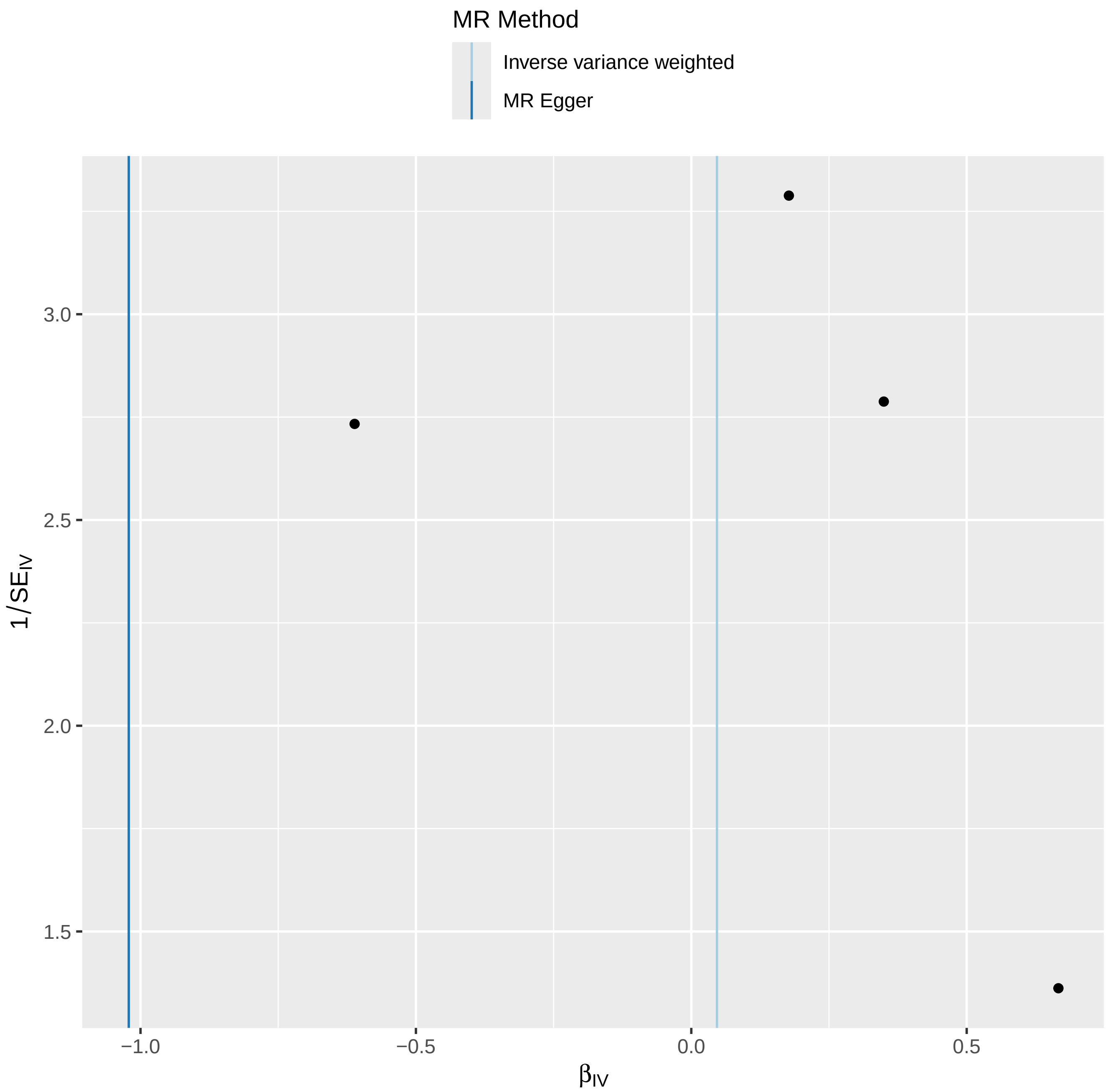
MR Method
Inverse variance weighted
MR Egger
3.0
2.5
2.0
1.5
1/SE_IV
−1.0
−0.5
0.0
0.5
β_IV

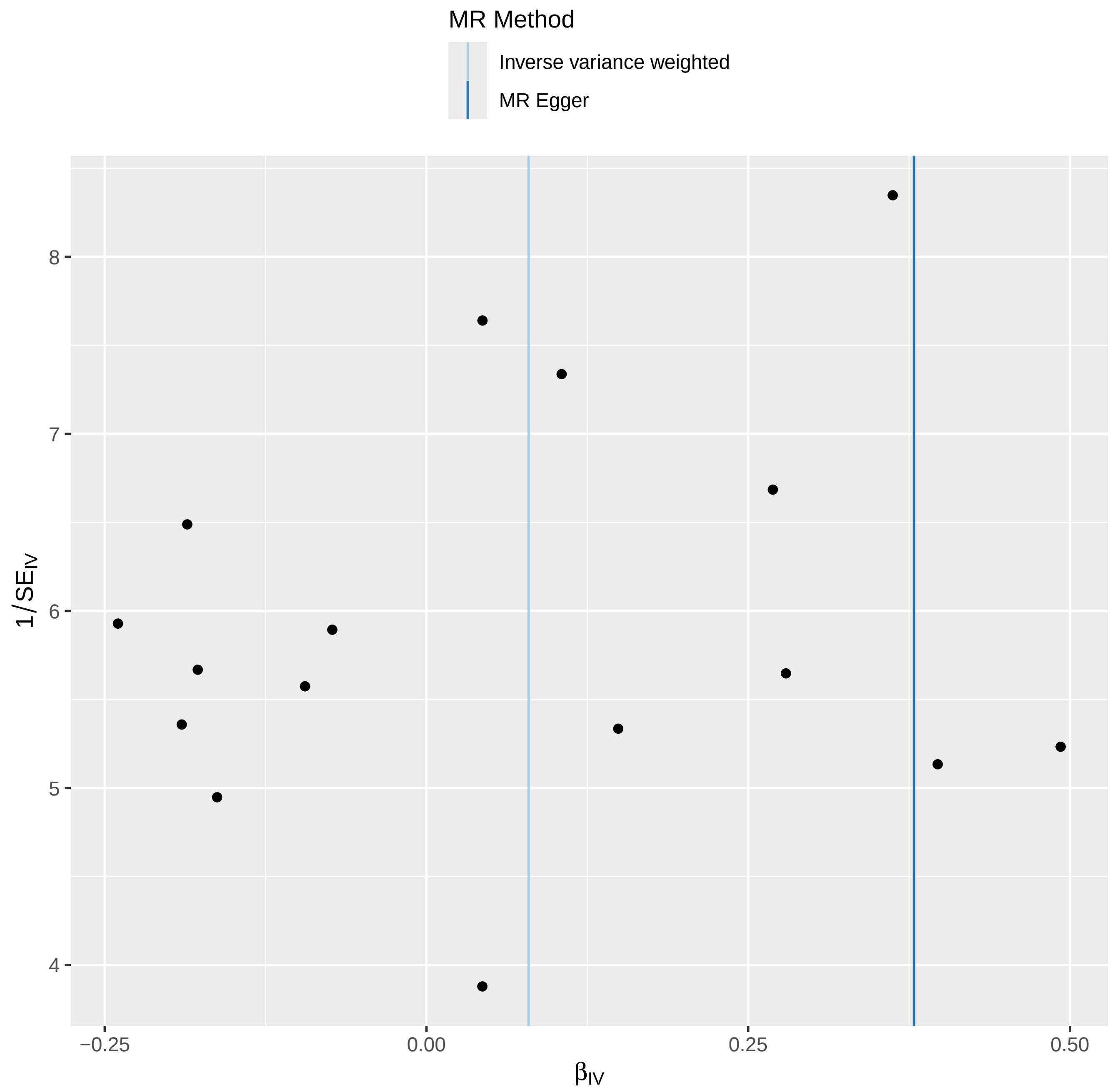
MR Method
Inverse variance weighted
MR Egger
8
7
6
5
4
1/SE_IV
−0.25
0.00
0.25
0.50
β_IV

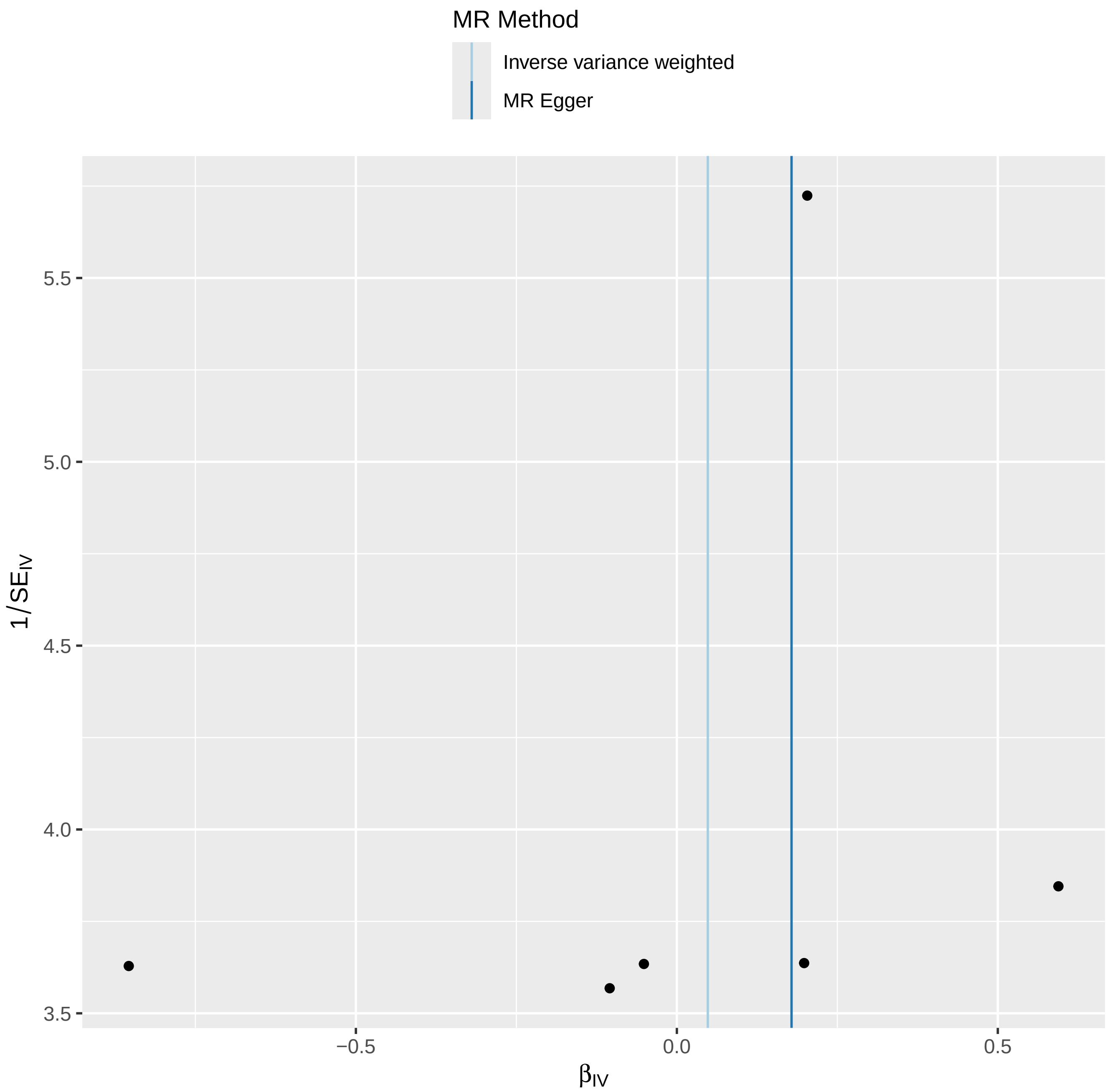

MR Method
Inverse variance weighted
MR Egger
5.5
5.0
4.5
4.0
3.5
1/SE_IV
−0.5
0.0
0.5
β_IV

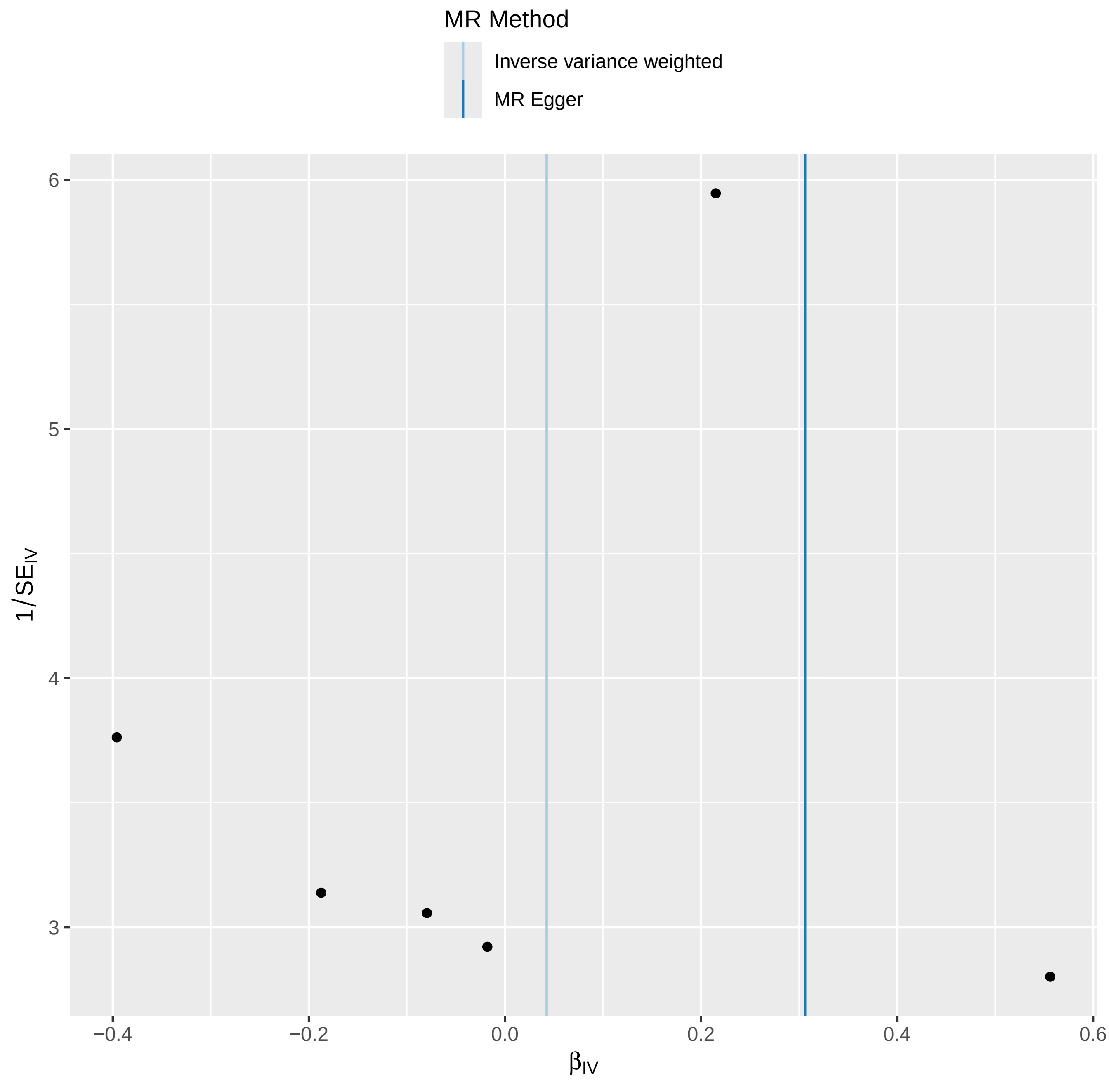

MR Method
Inverse variance weighted
MR Egger
6
5
4
3
1/SE_IV
−0.4
−0.2
0.0
0.2
0.4
0.6
β_IV

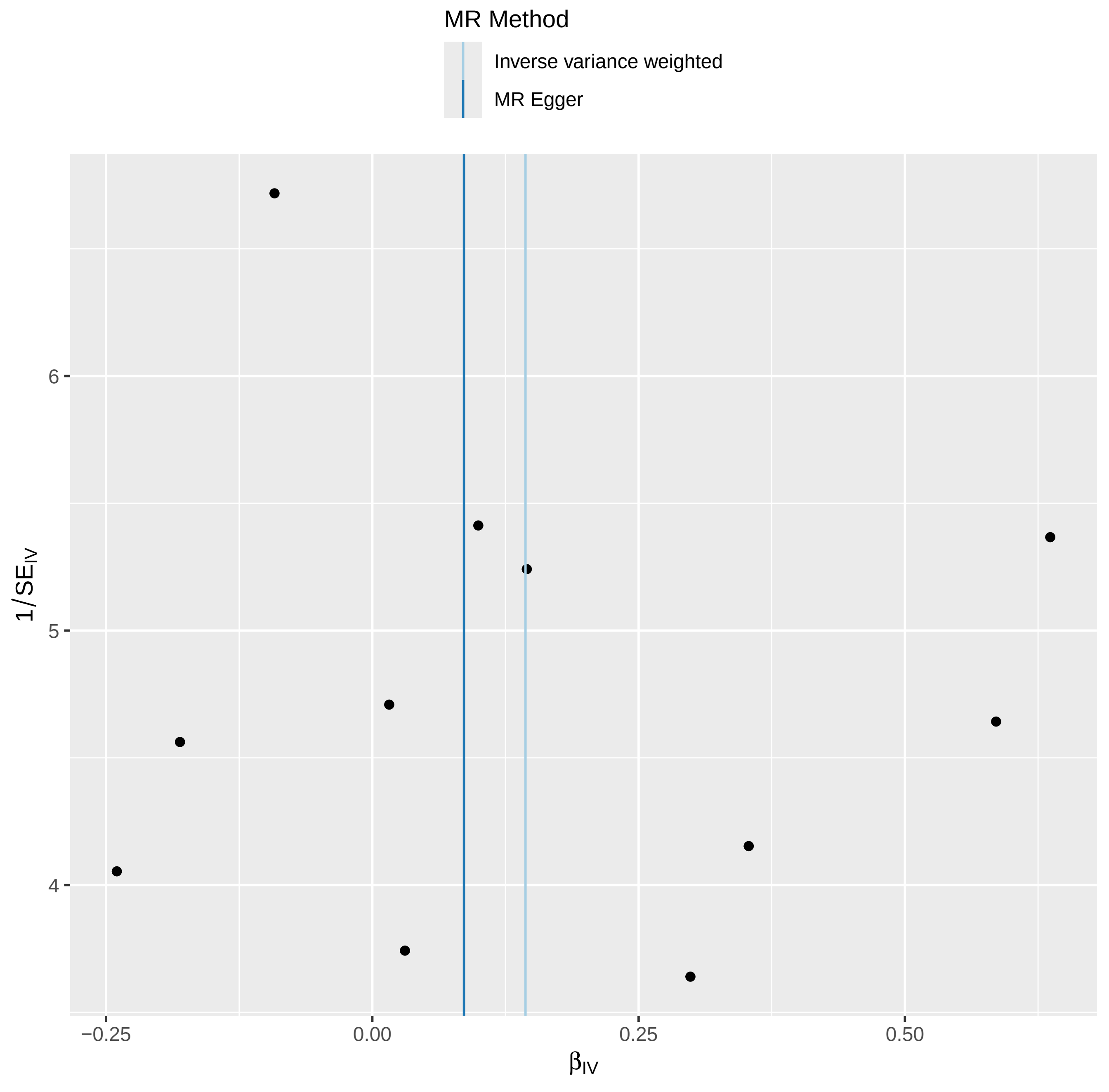
MR Method
Inverse variance weighted
MR Egger
1/SE_IV
6
5
4
−0.25
0.00
0.25
0.50
β_IV

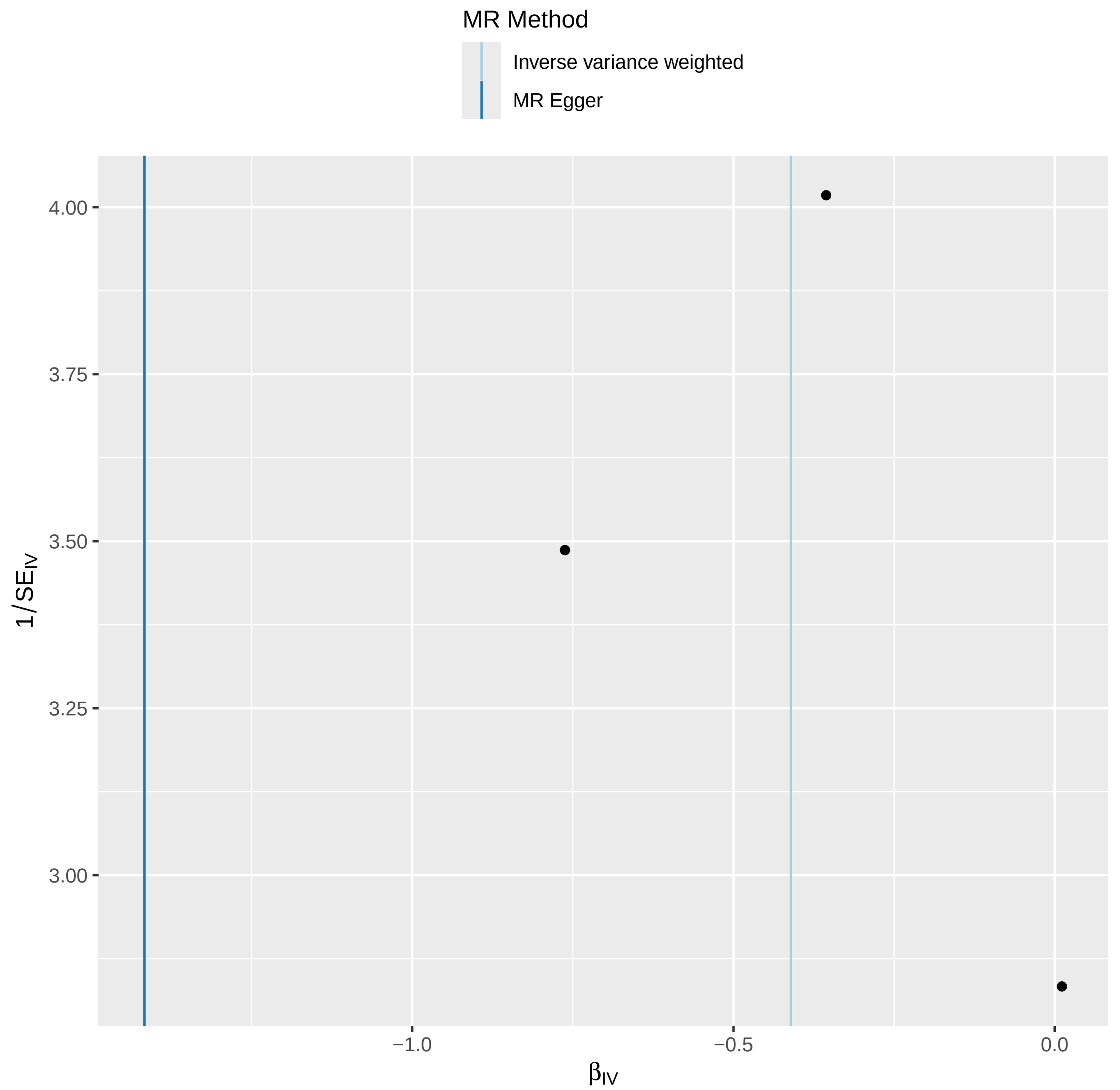
MR Method
Inverse variance weighted
MR Egger
4.00
3.75
3.50
3.25
3.00
1/SE_IV
−1.0
−0.5
0.0
β_IV

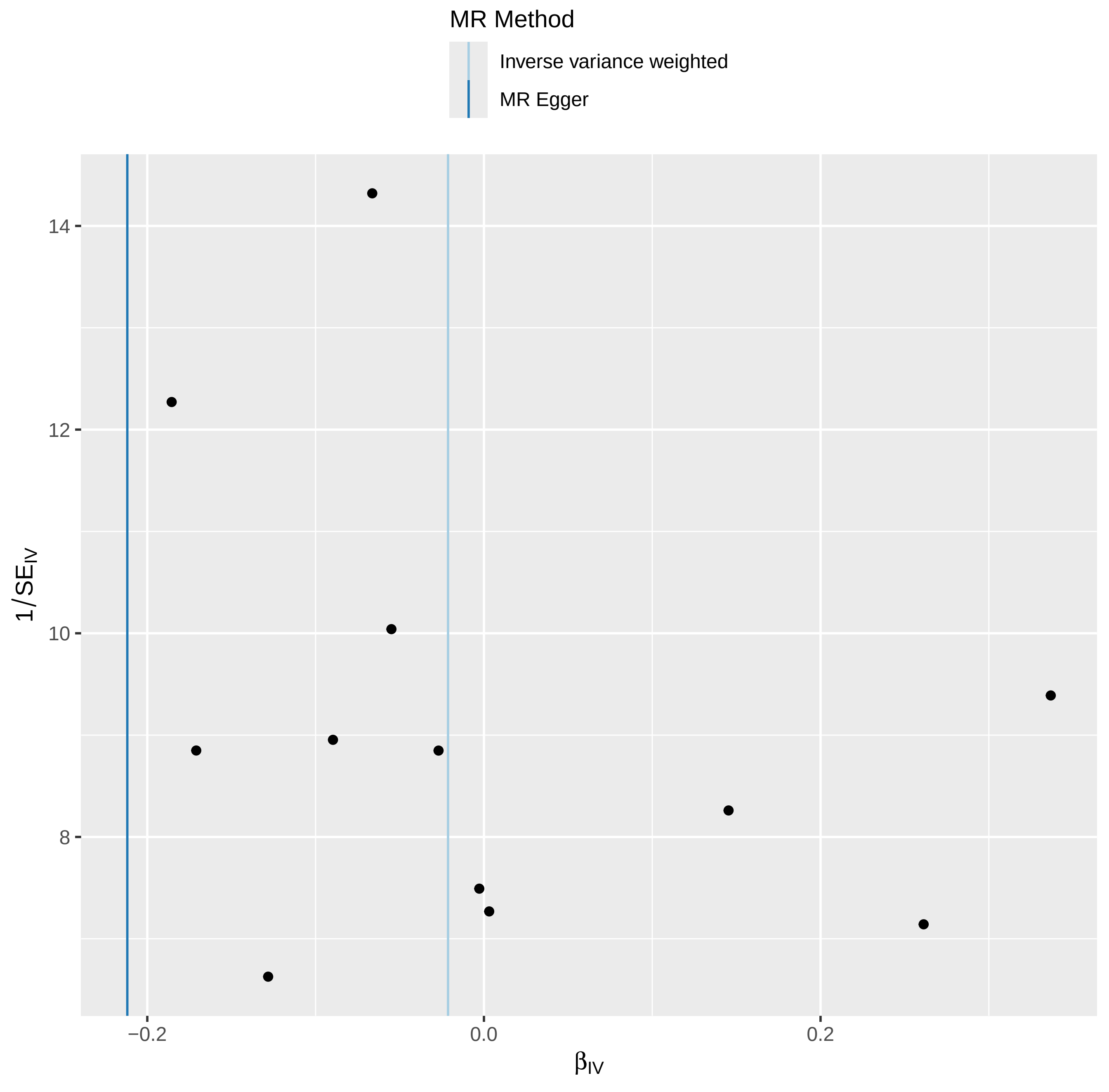
MR Method
Inverse variance weighted
MR Egger
14
12
10
8
1/SE_IV
−0.2
0.0
0.2
β_IV

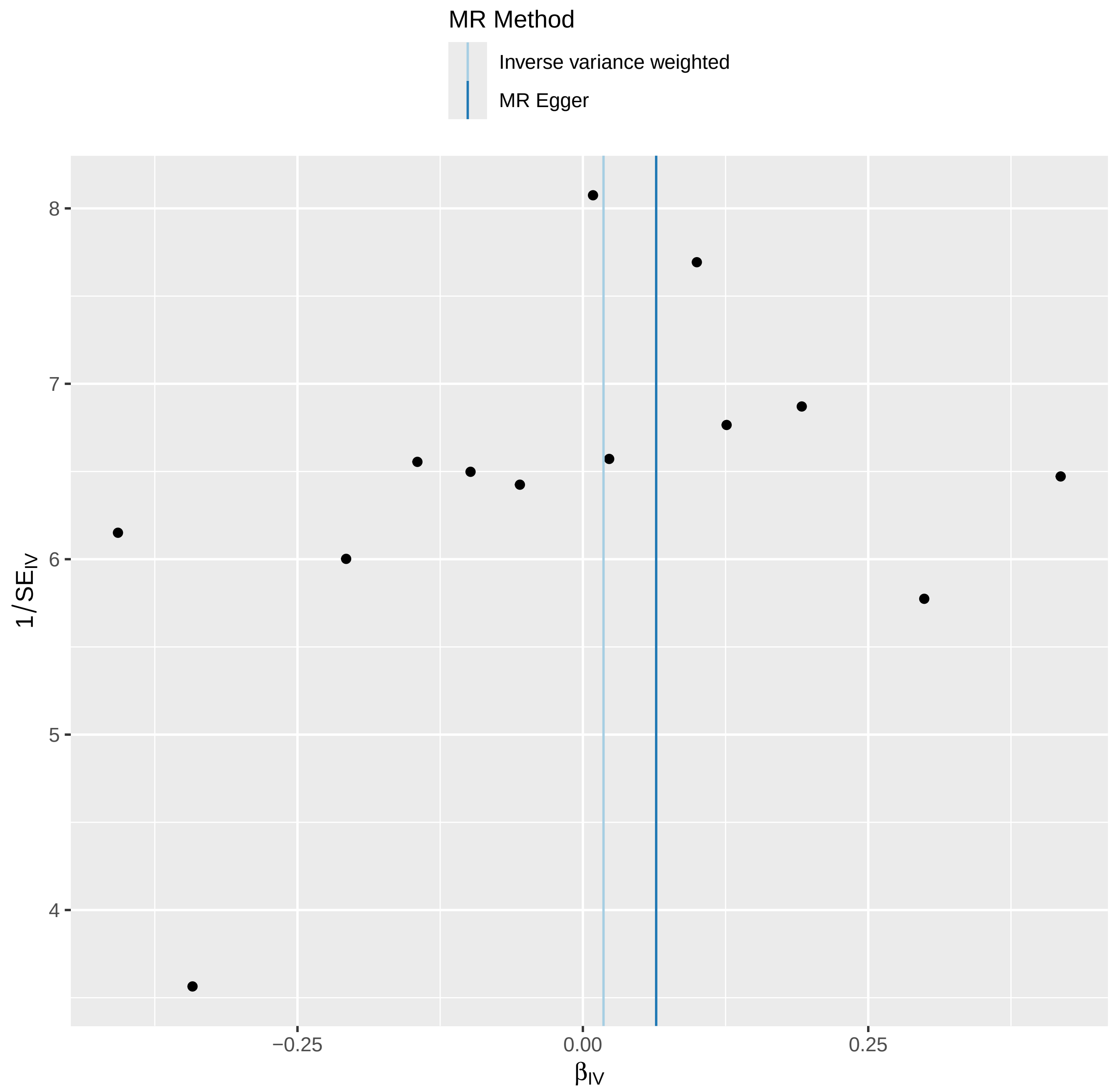
MR Method
Inverse variance weighted
MR Egger
8
7
6
5
4
1/SE$_{IV}$
−0.25
0.00
0.25
β_{IV}

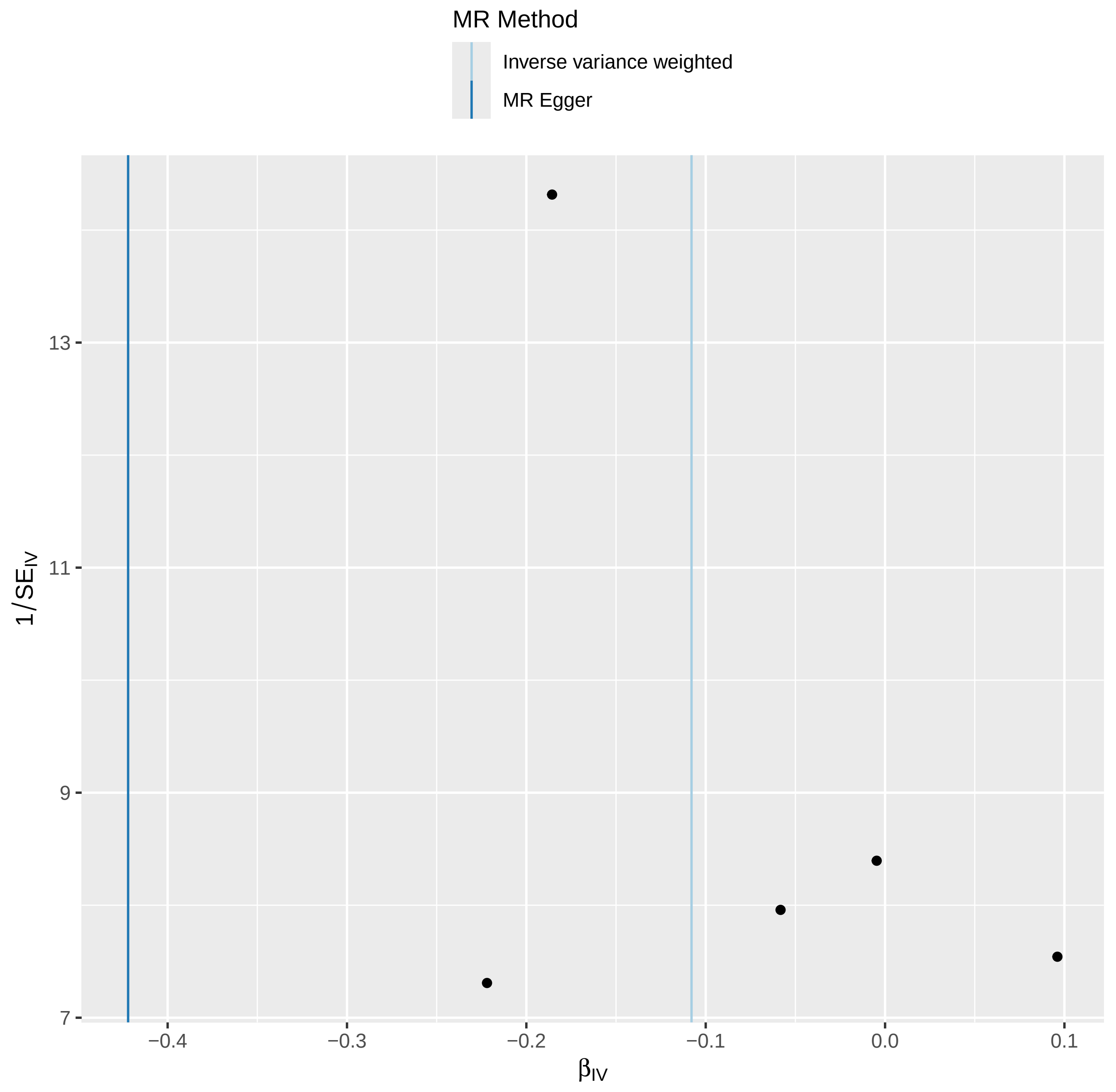
MR Method
Inverse variance weighted
MR Egger
13
11
9
7
−0.4
−0.3
−0.2
−0.1
0.0
0.1
β_{IV}
$1/SE_{IV}$

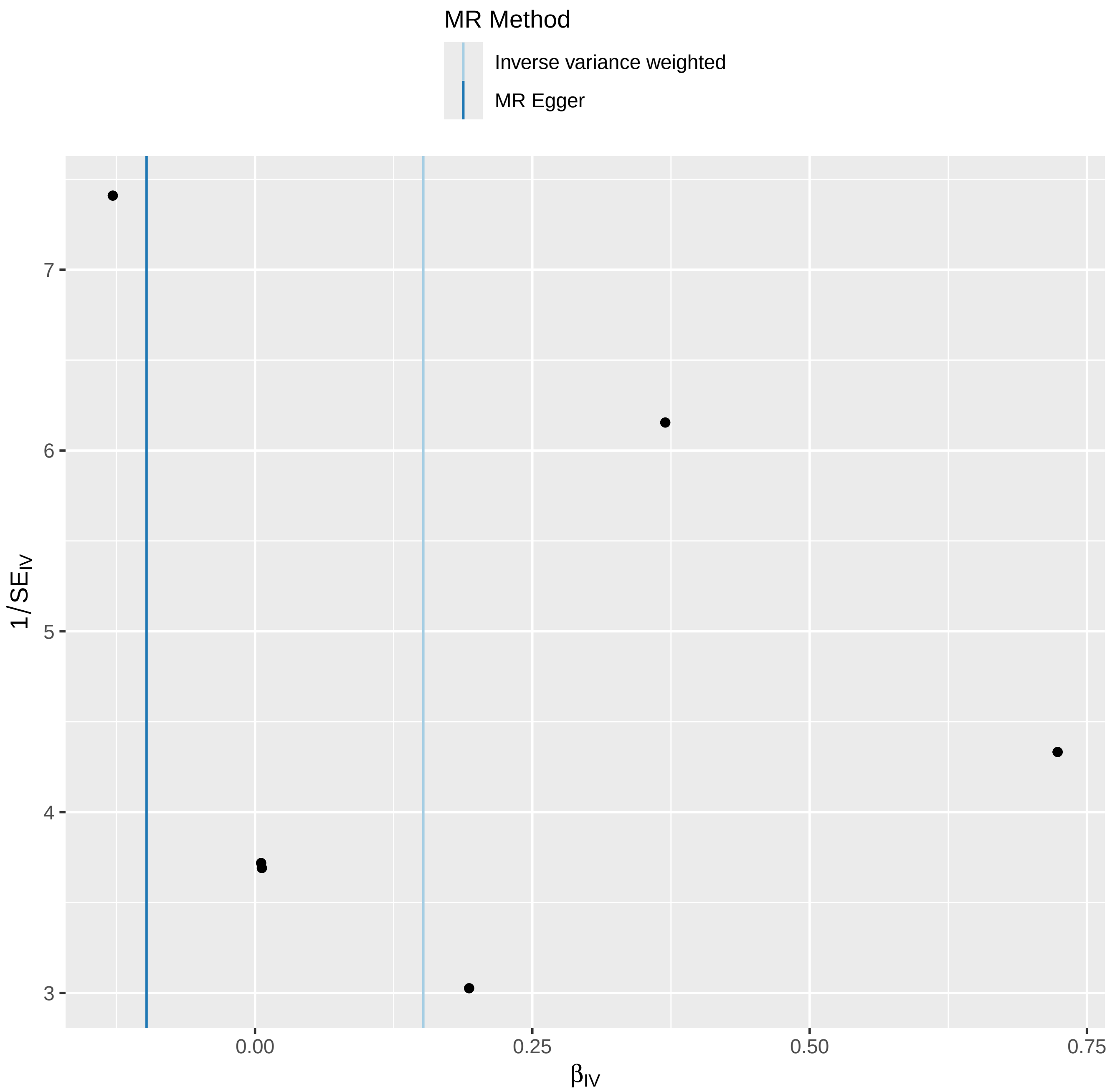

MR Method
Inverse variance weighted
MR Egger
7
6
5
4
3
1/SE_IV
0.00
0.25
0.50
0.75
β_IV

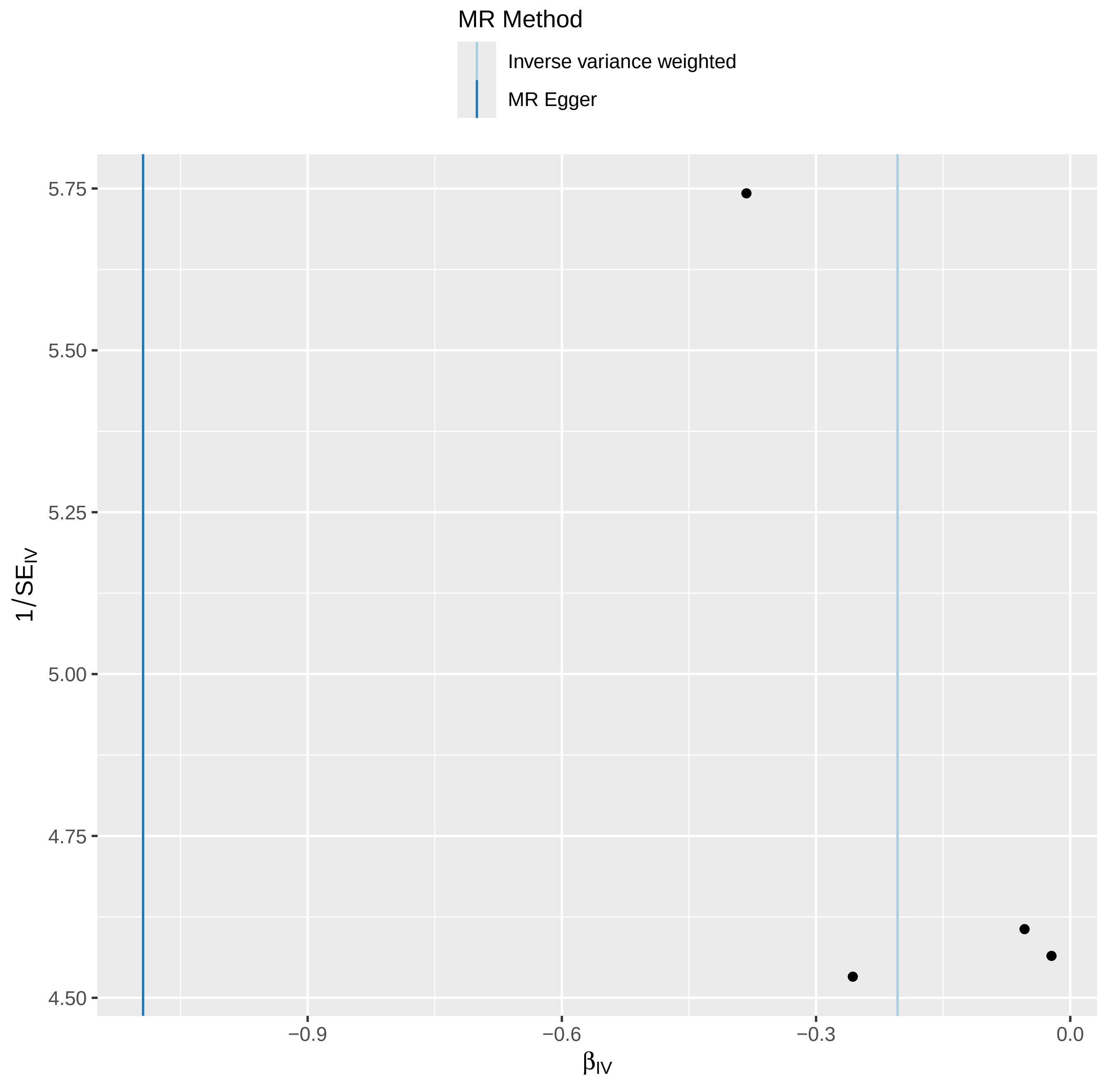
MR Method
Inverse variance weighted
MR Egger
5.75
5.50
5.25
5.00
4.75
4.50
1/SE_IV
−0.9
−0.6
−0.3
0.0
β_IV

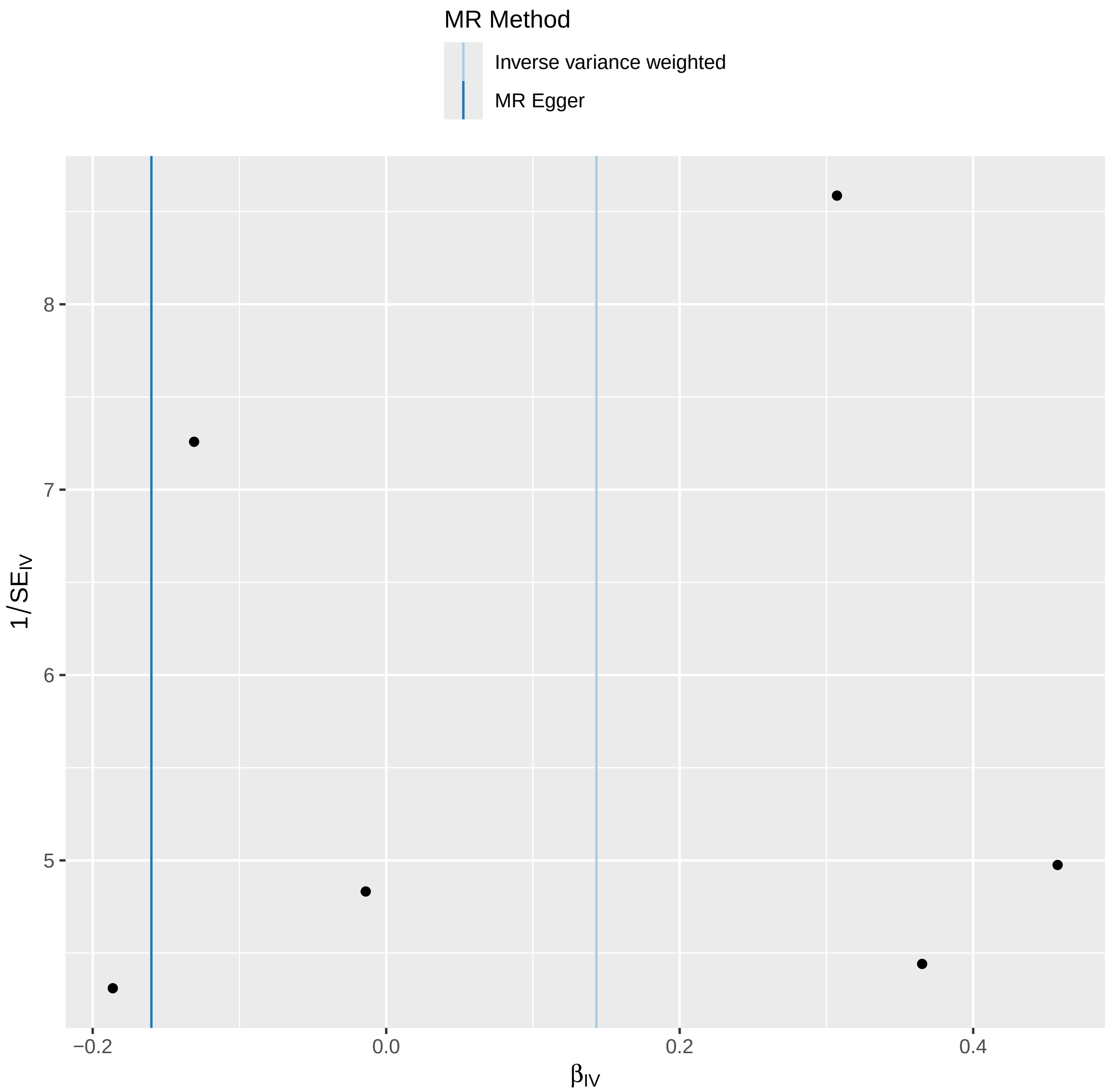

MR Method
Inverse variance weighted
MR Egger
8
7
6
5
1/SE$_{IV}$
−0.2
0.0
0.2
0.4
β$_{IV}$

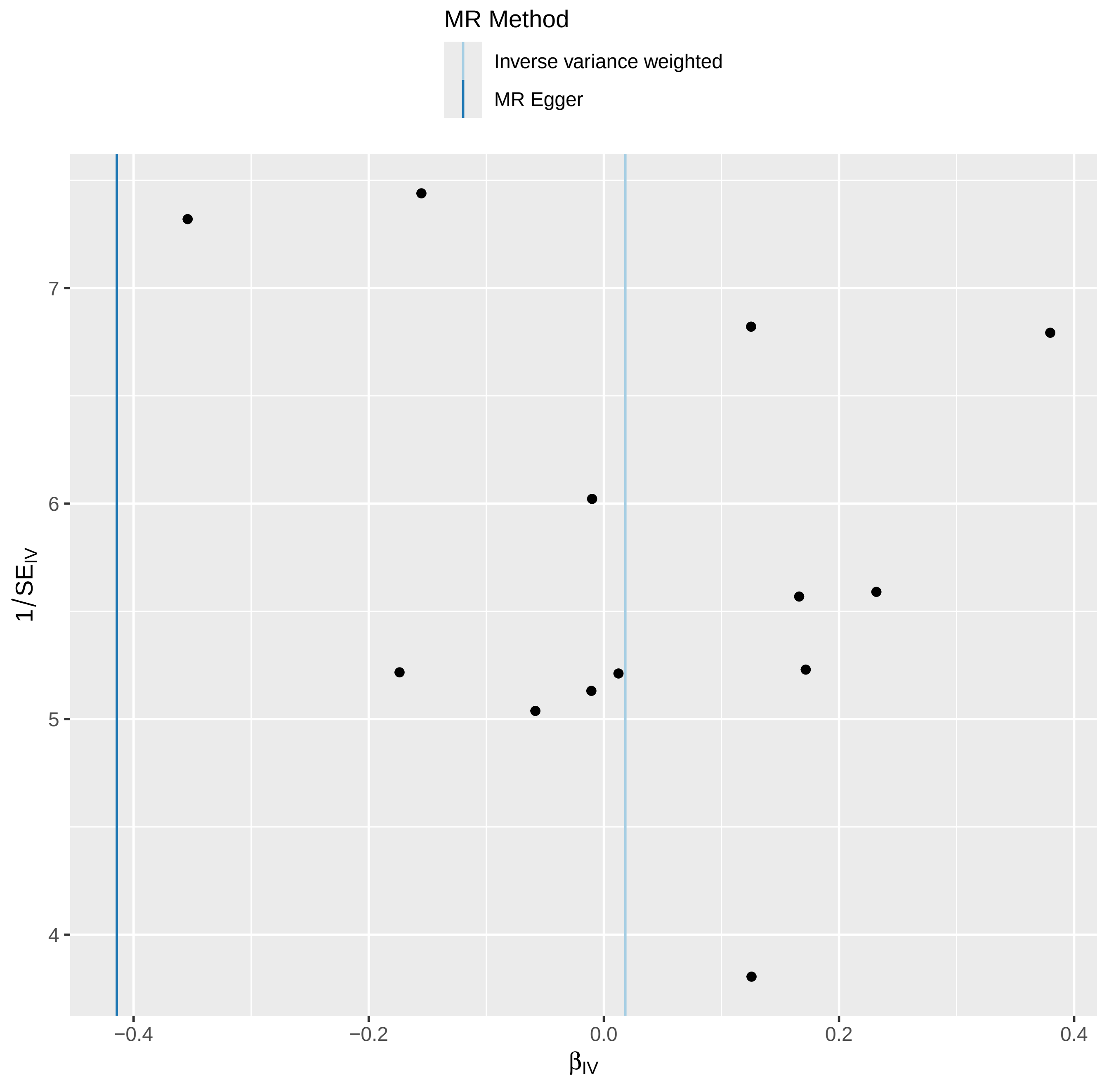
MR Method
Inverse variance weighted
MR Egger
7
6
5
4
1/SE_IV
−0.4
−0.2
0.0
0.2
0.4
β_IV

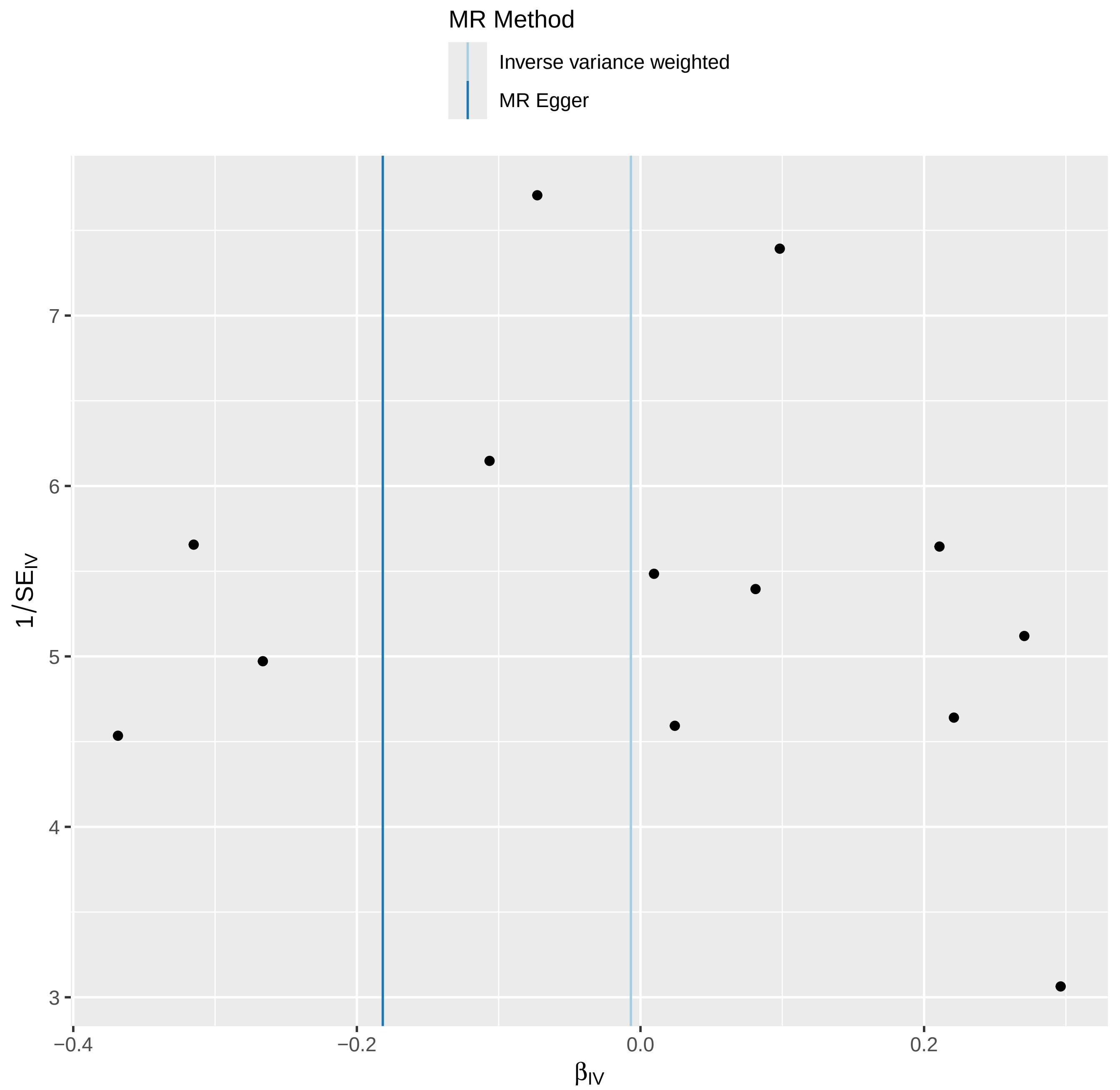
MR Method
Inverse variance weighted
MR Egger
1/SE_IV
β_IV
−0.4
−0.2
0.0
0.2
3
4
5
6
7

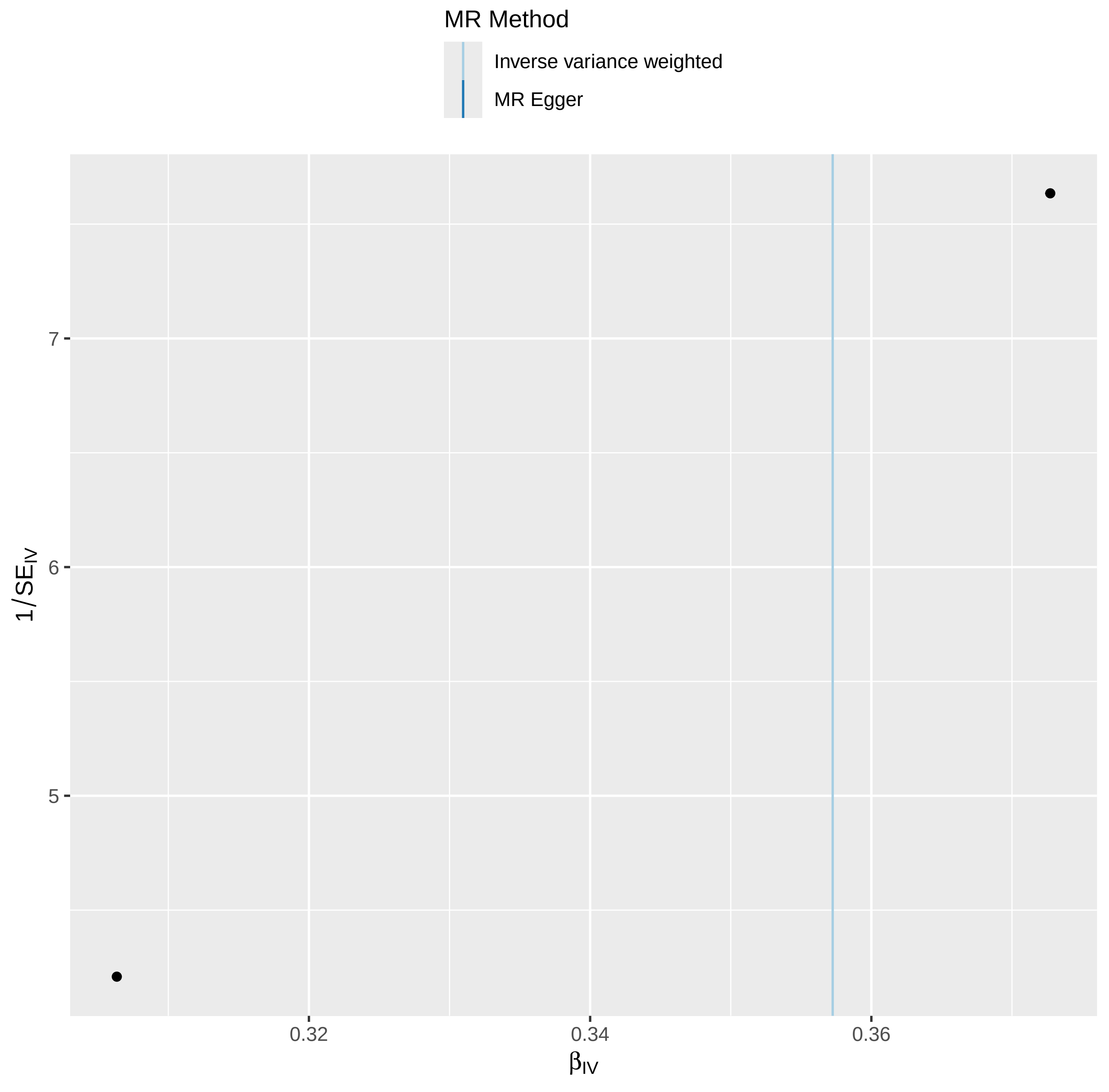

MR Method
Inverse variance weighted
MR Egger
7
6
5
0.32
0.34
0.36
1/SE_IV
β_IV

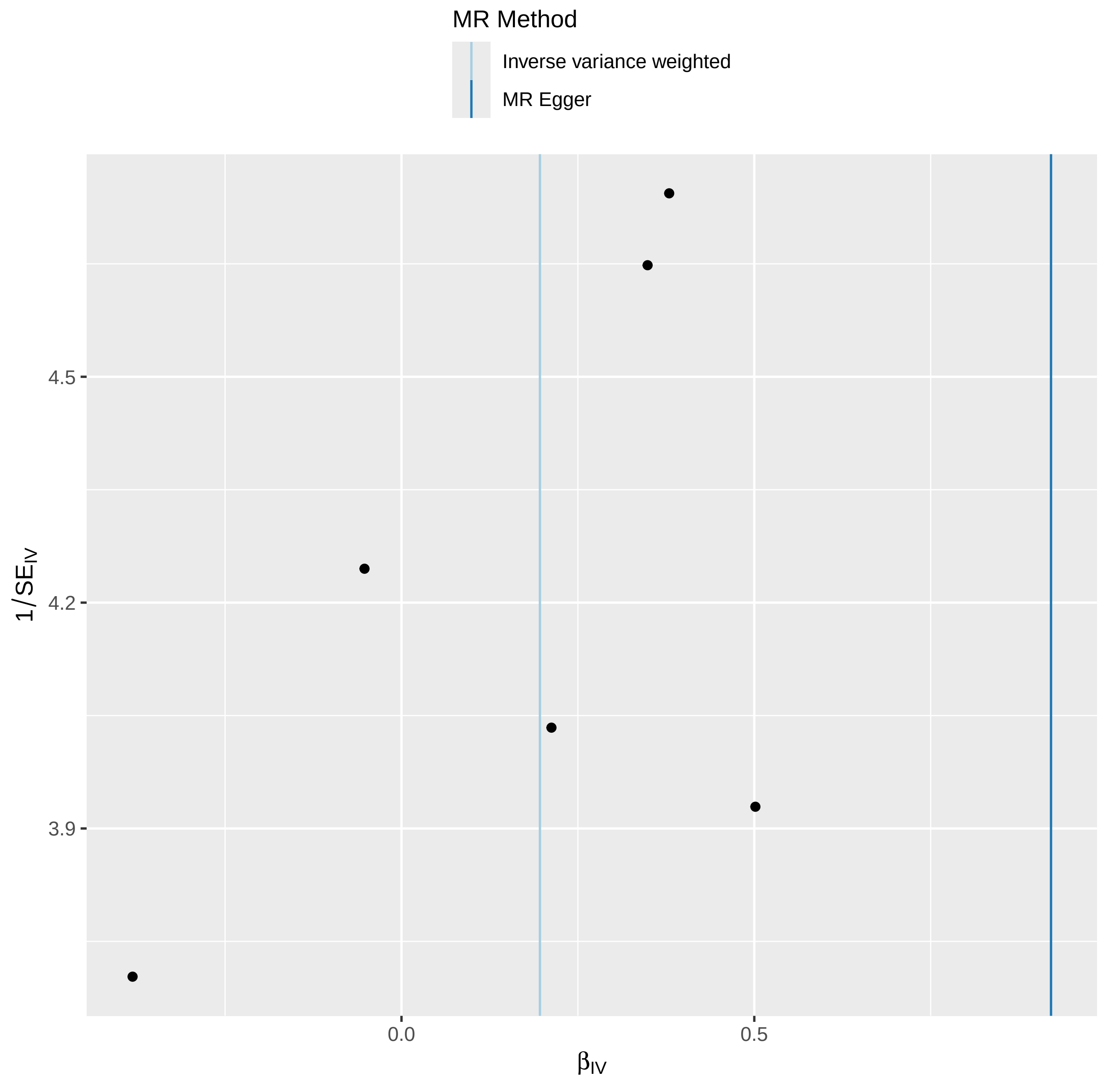

MR Method
Inverse variance weighted
MR Egger
4.5
4.2
3.9
$1/SE_{IV}$
0.0
0.5
β_{IV}

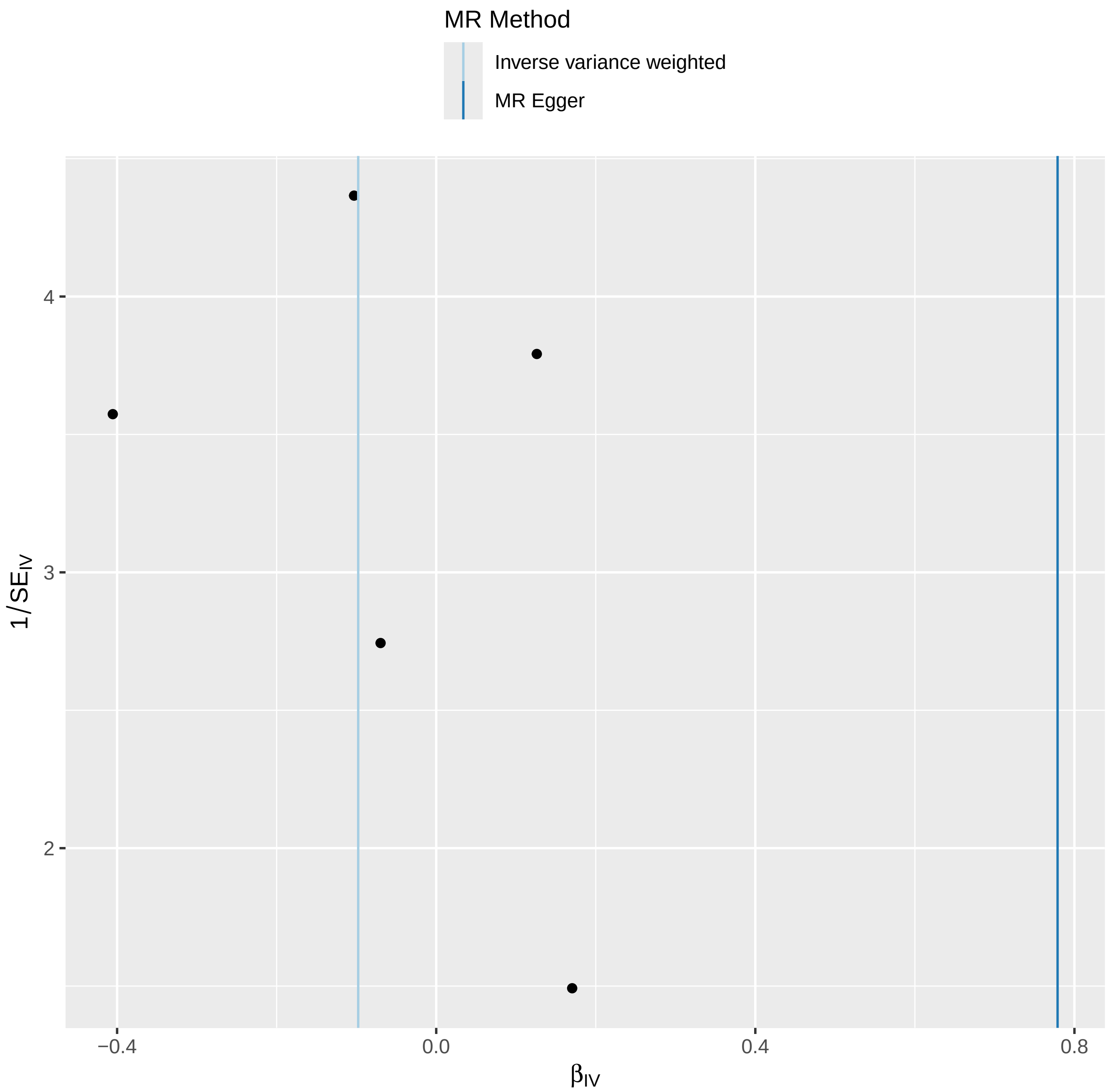

MR Method
Inverse variance weighted
MR Egger
1/SE_IV
4
3
2
−0.4
0.0
0.4
0.8
β_IV

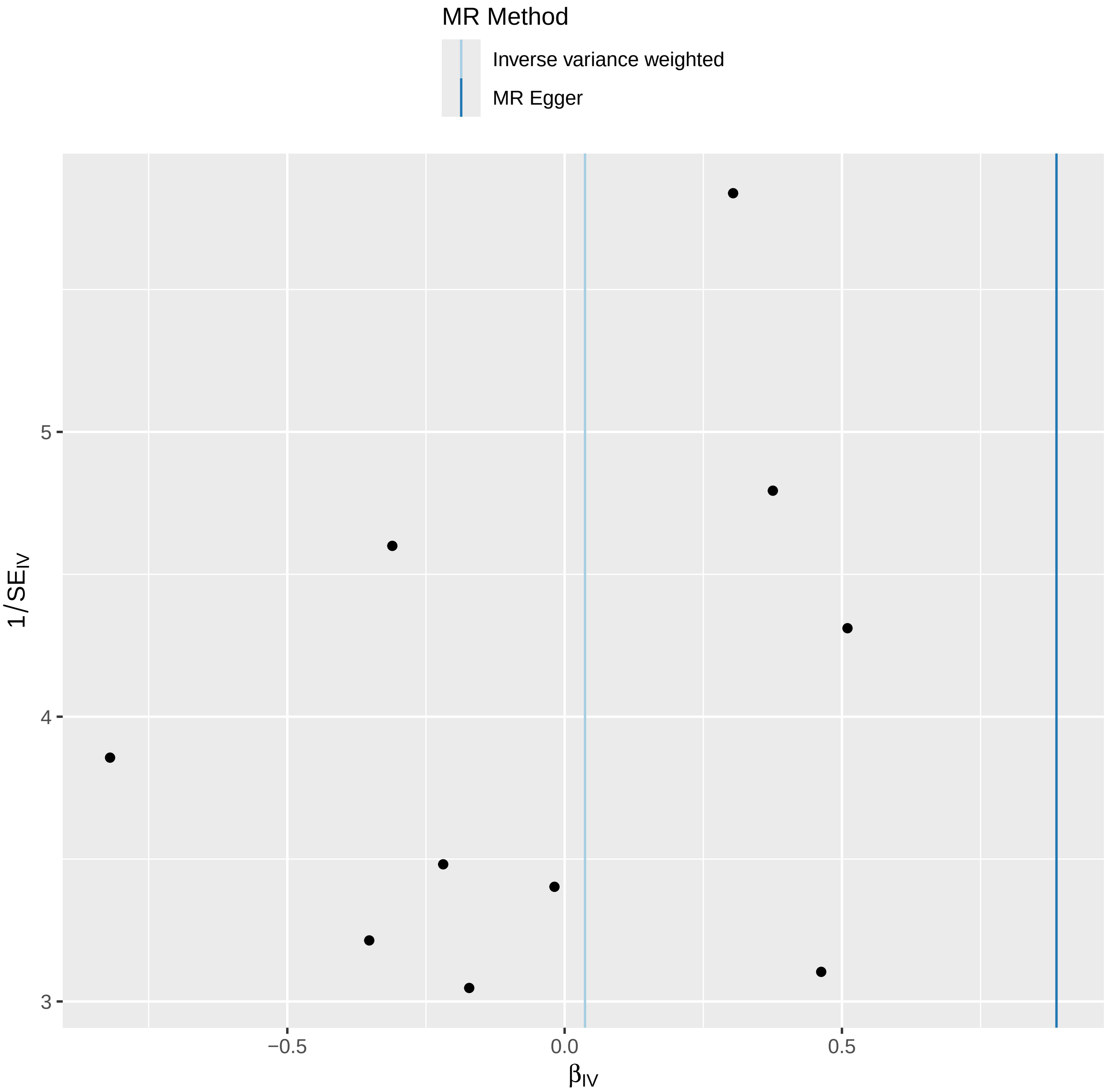

MR Method
Inverse variance weighted
MR Egger
5
4
3
1/SE_IV
−0.5
0.0
0.5
β_IV

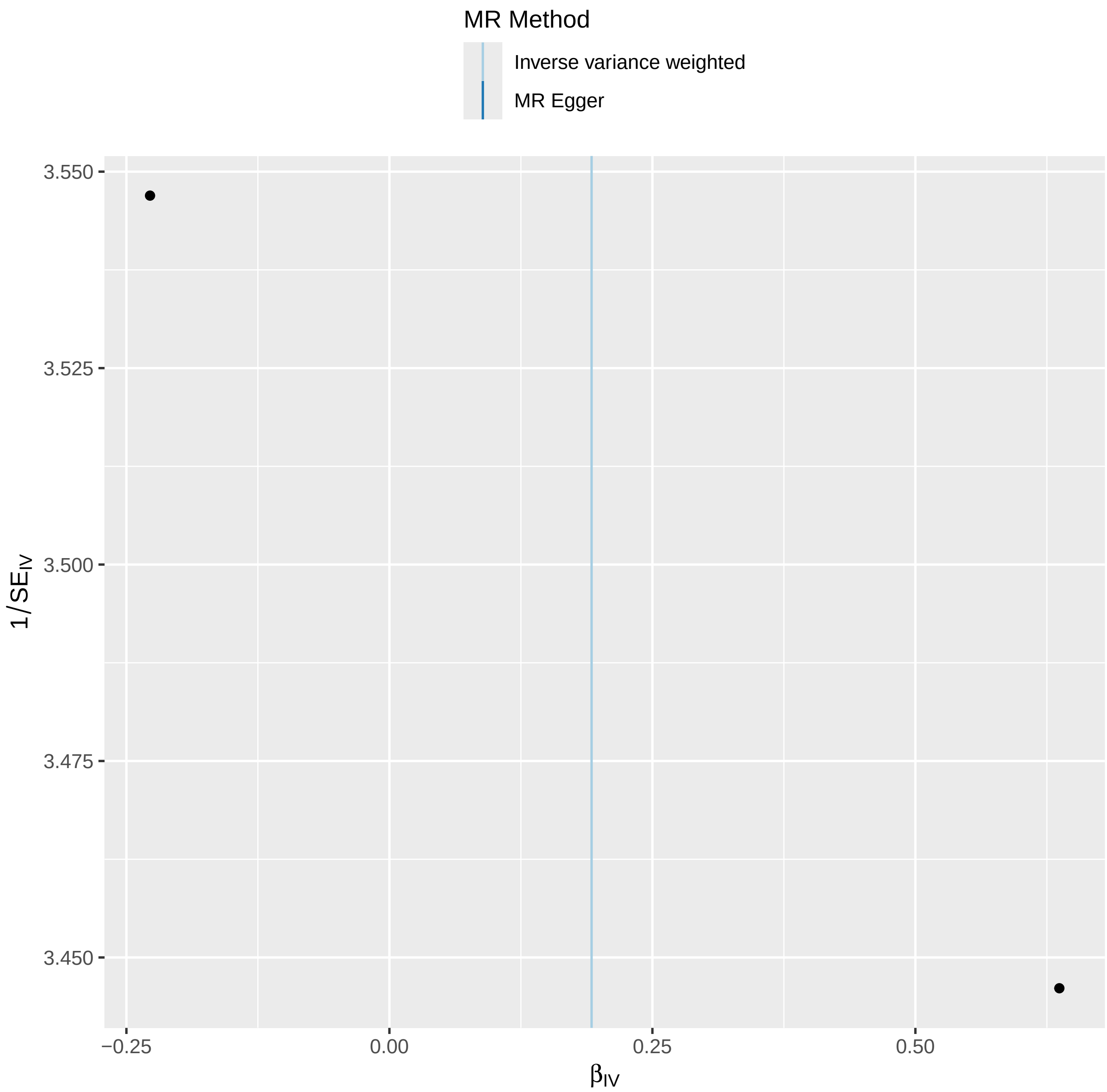
MR Method
Inverse variance weighted
MR Egger
3.550
3.525
3.500
3.475
3.450
1/SE_IV
−0.25
0.00
0.25
0.50
β_IV

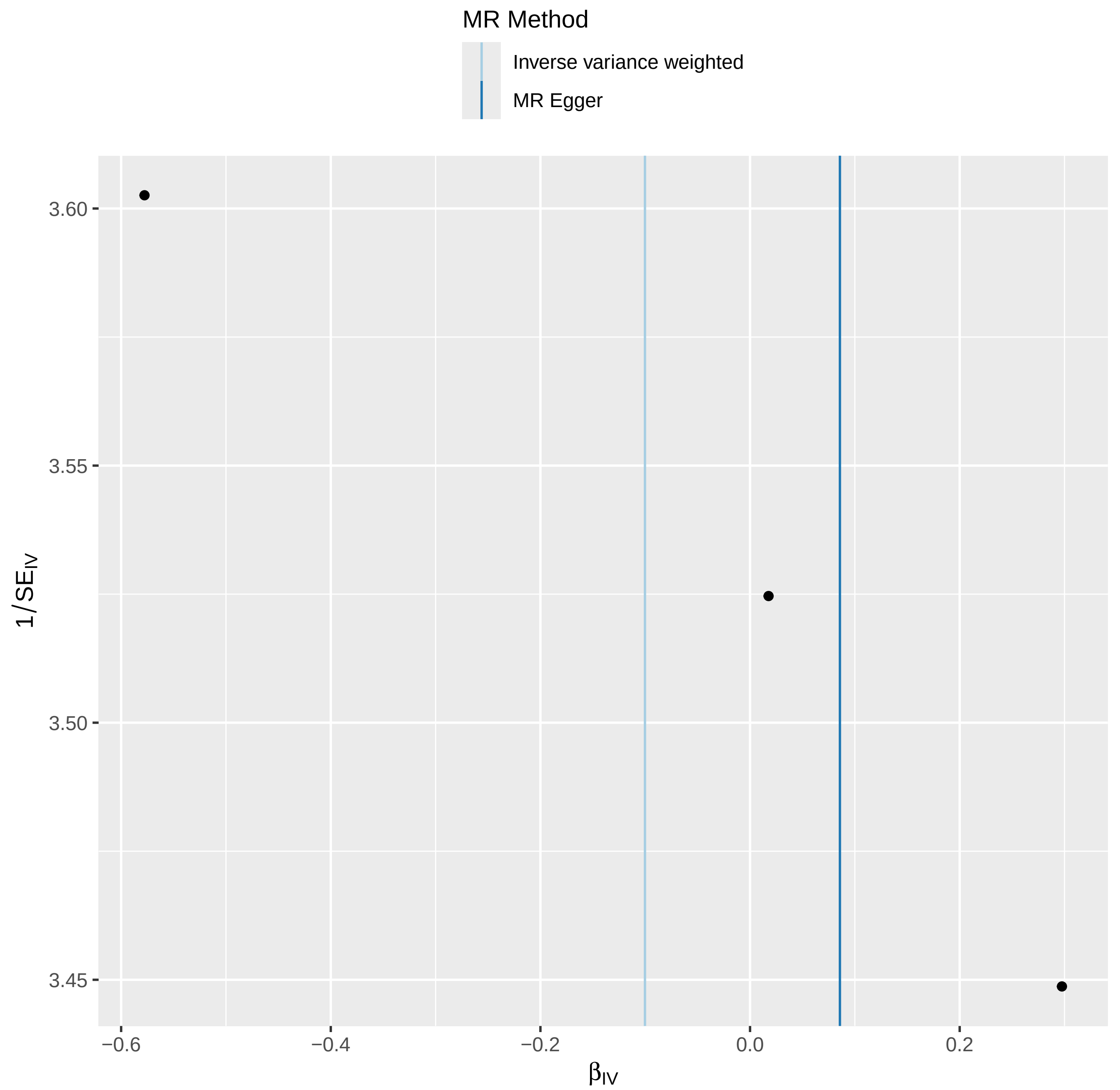
MR Method
Inverse variance weighted
MR Egger
3.60
3.55
3.50
3.45
1/SE_IV
−0.6
−0.4
−0.2
0.0
0.2
β_IV

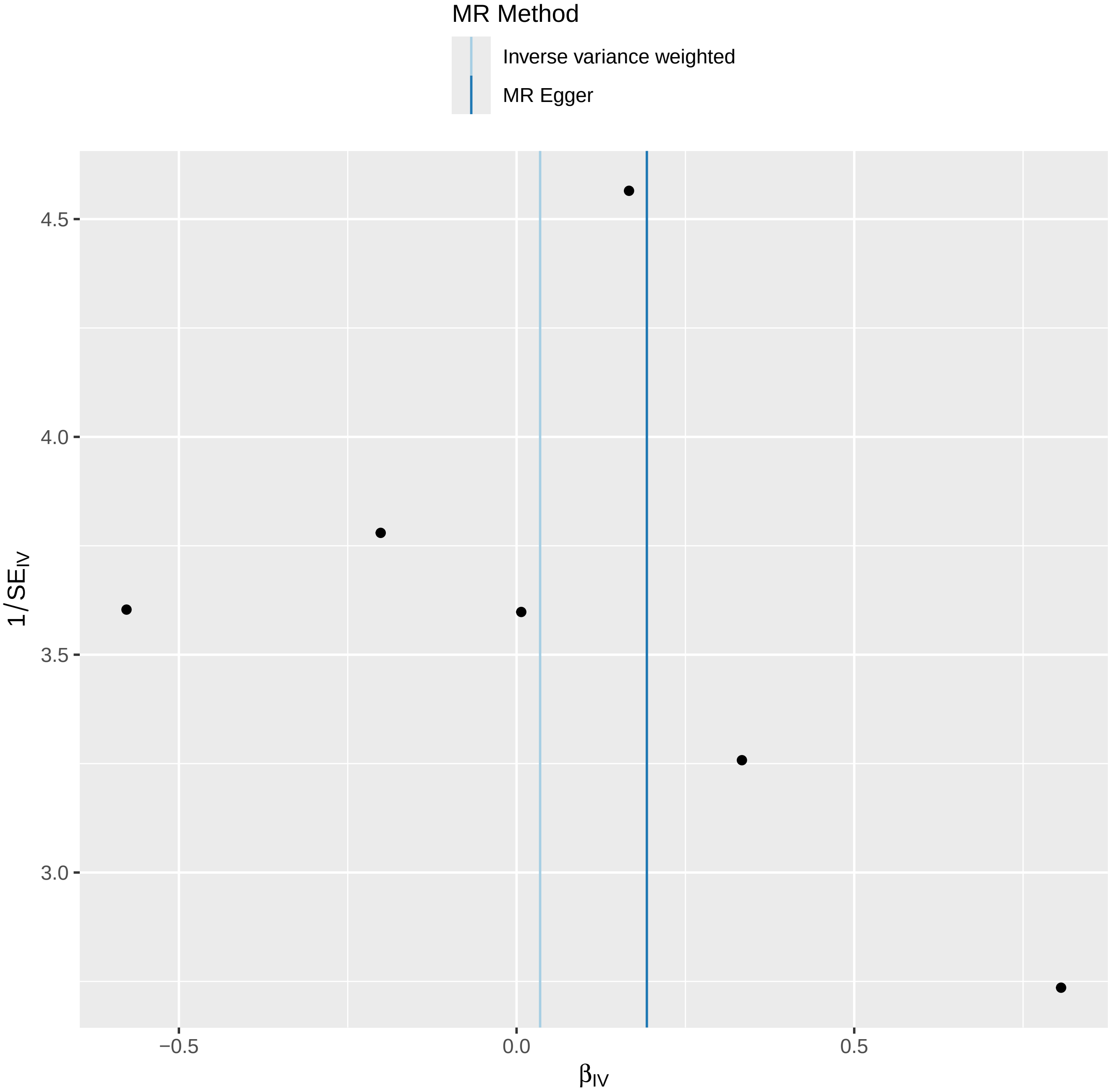

MR Method
Inverse variance weighted
MR Egger
4.5
4.0
3.5
3.0
1/SE_IV
−0.5
0.0
0.5
β_IV

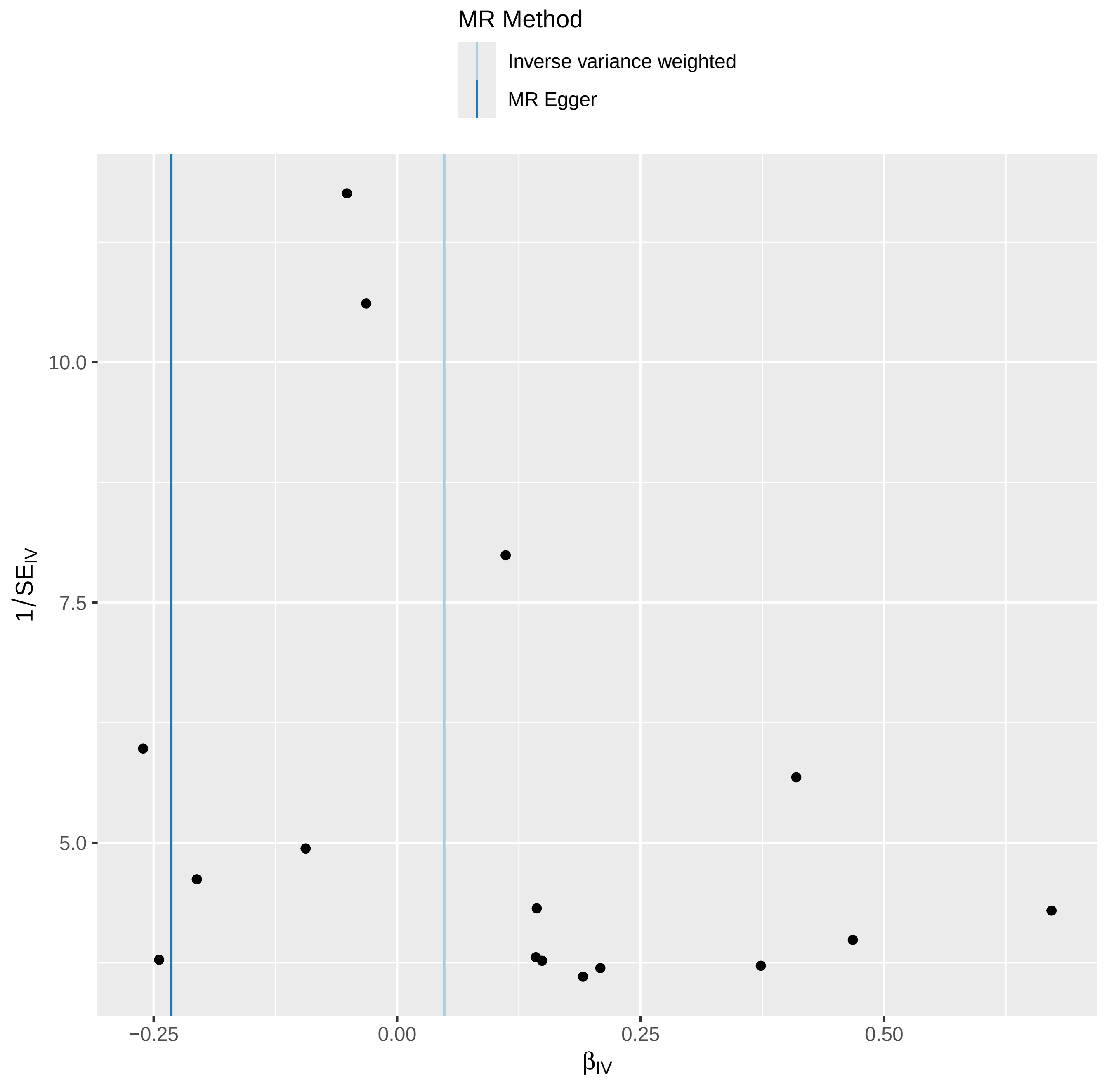
MR Method
Inverse variance weighted
MR Egger
10.0
7.5
5.0
1/SE$_{IV}$
−0.25
0.00
0.25
0.50
β$_{IV}$

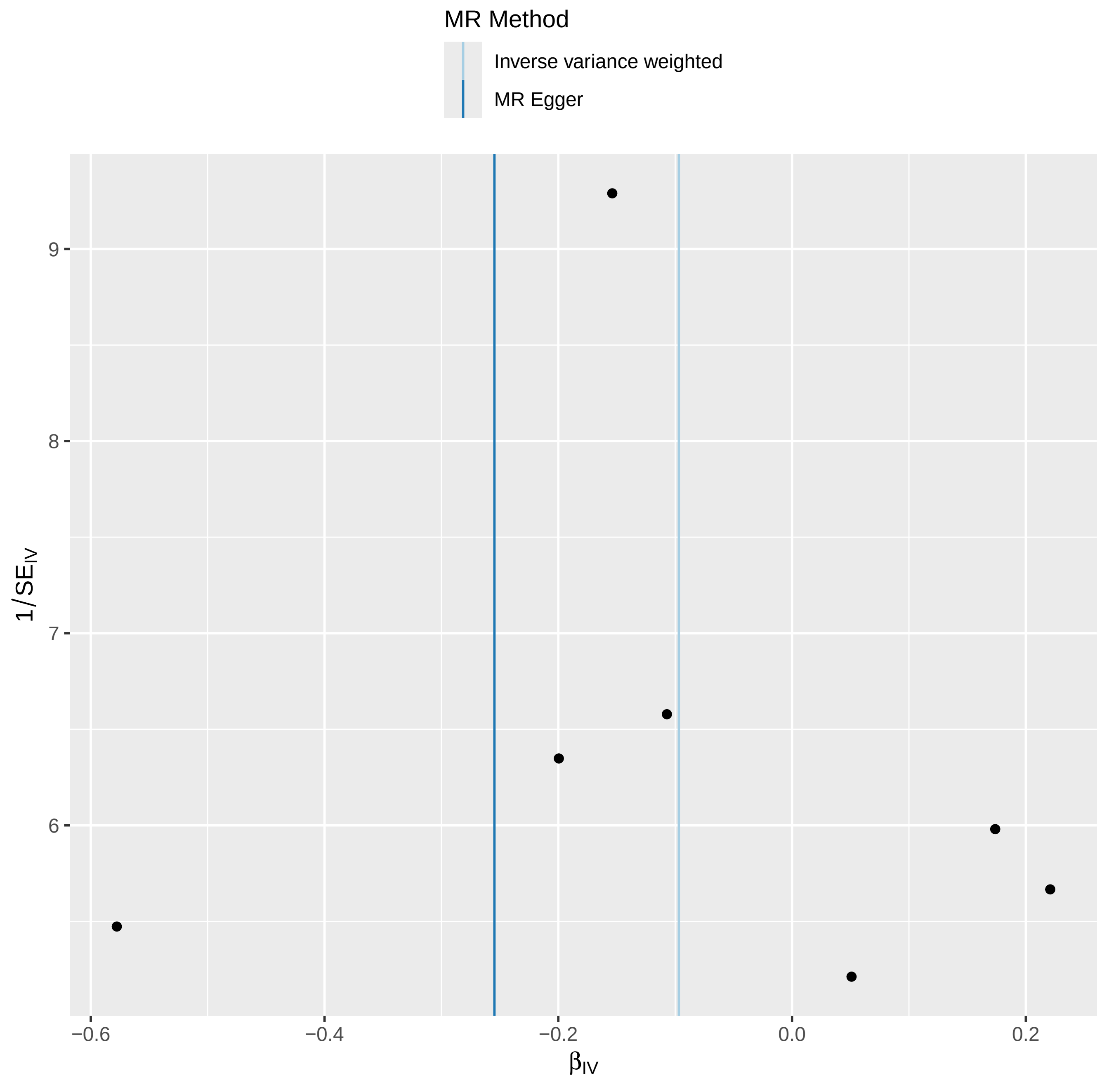
MR Method
Inverse variance weighted
MR Egger
1/SE_IV
9
8
7
6
−0.6
−0.4
−0.2
0.0
0.2
β_IV

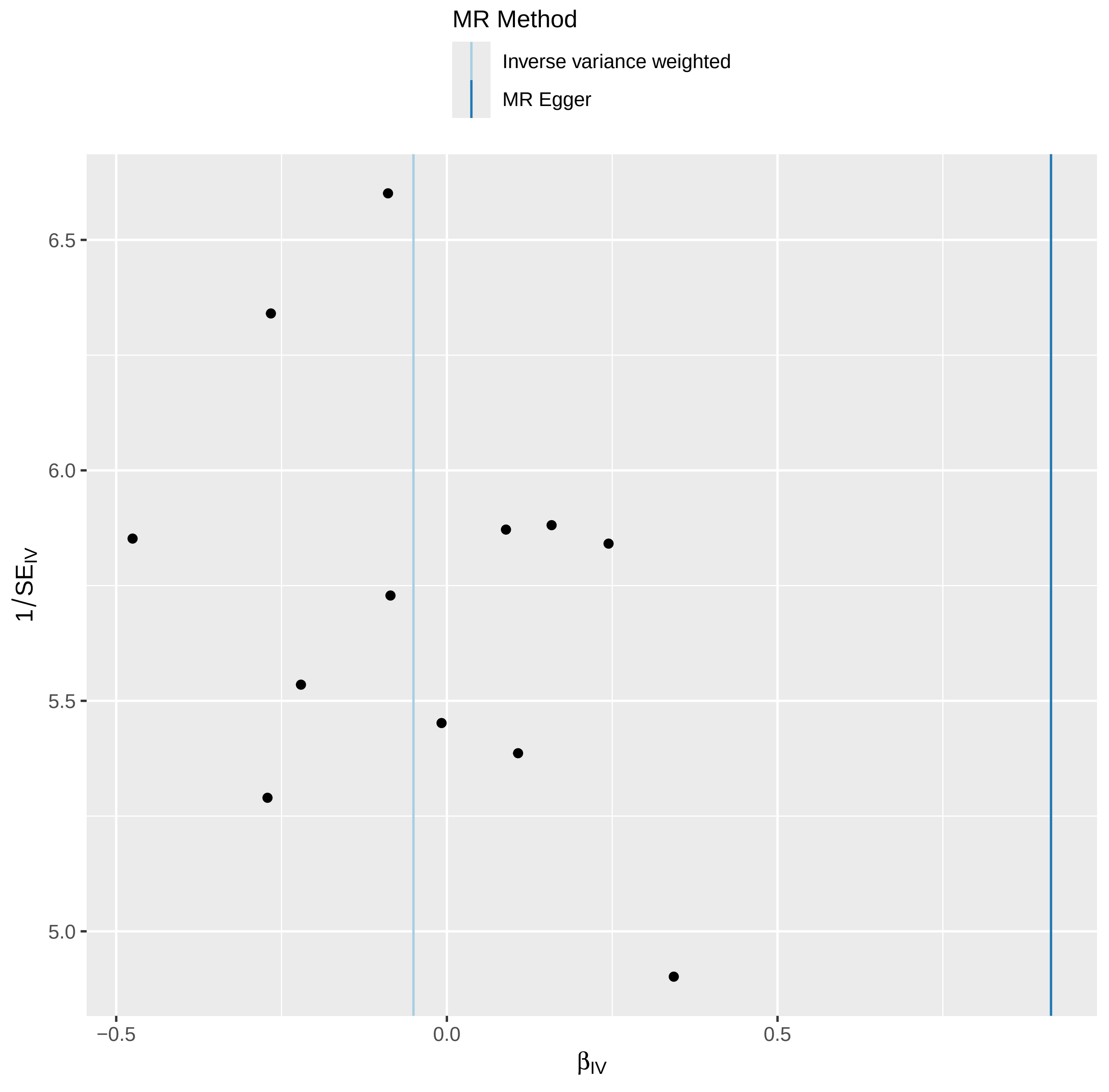

MR Method
Inverse variance weighted
MR Egger
6.5
6.0
5.5
5.0
1/SE_IV
−0.5
0.0
0.5
β_IV

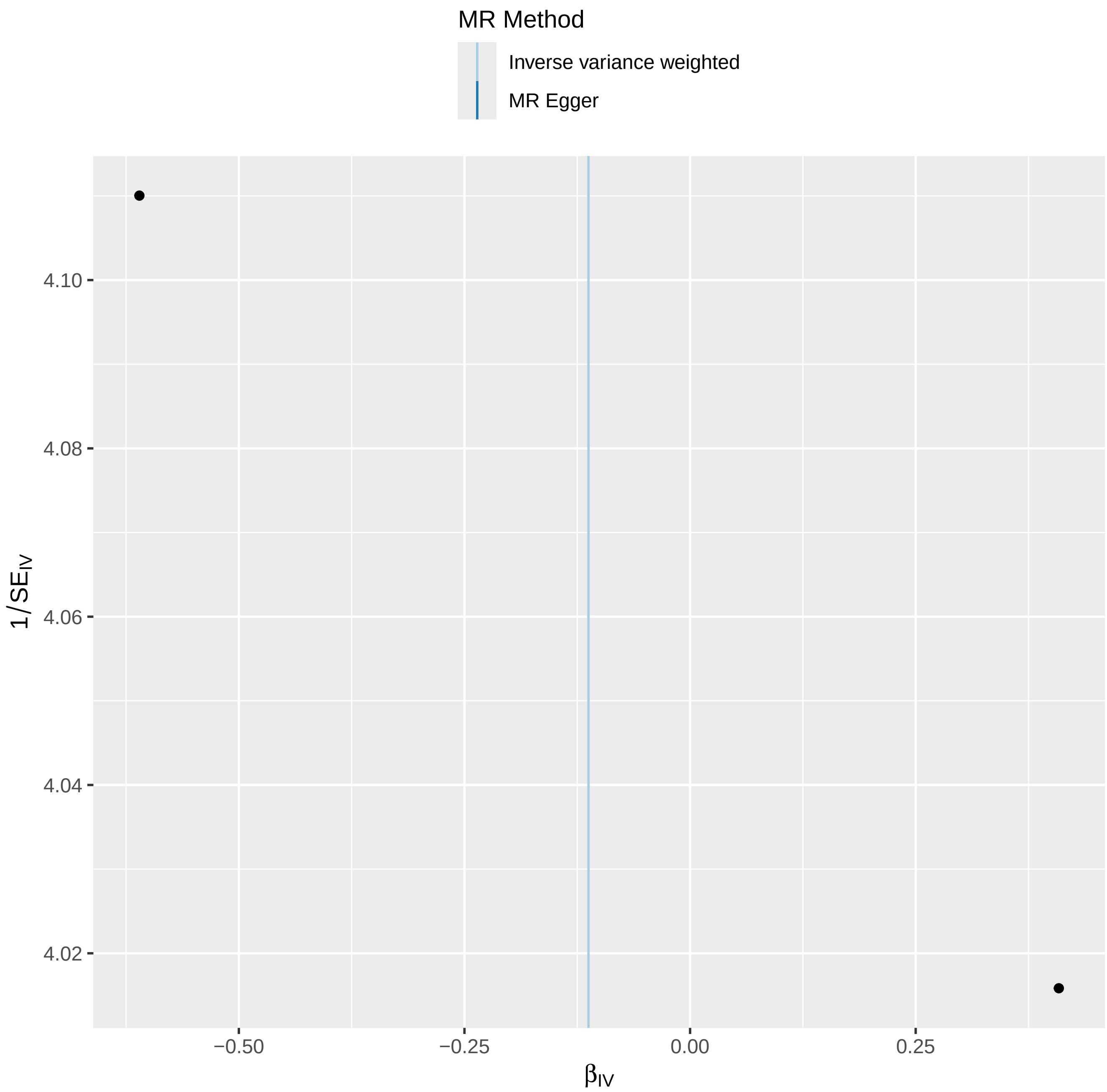
MR Method
Inverse variance weighted
MR Egger
4.10
4.08
4.06
4.04
4.02
1/SE_IV
−0.50
−0.25
0.00
0.25
β_IV

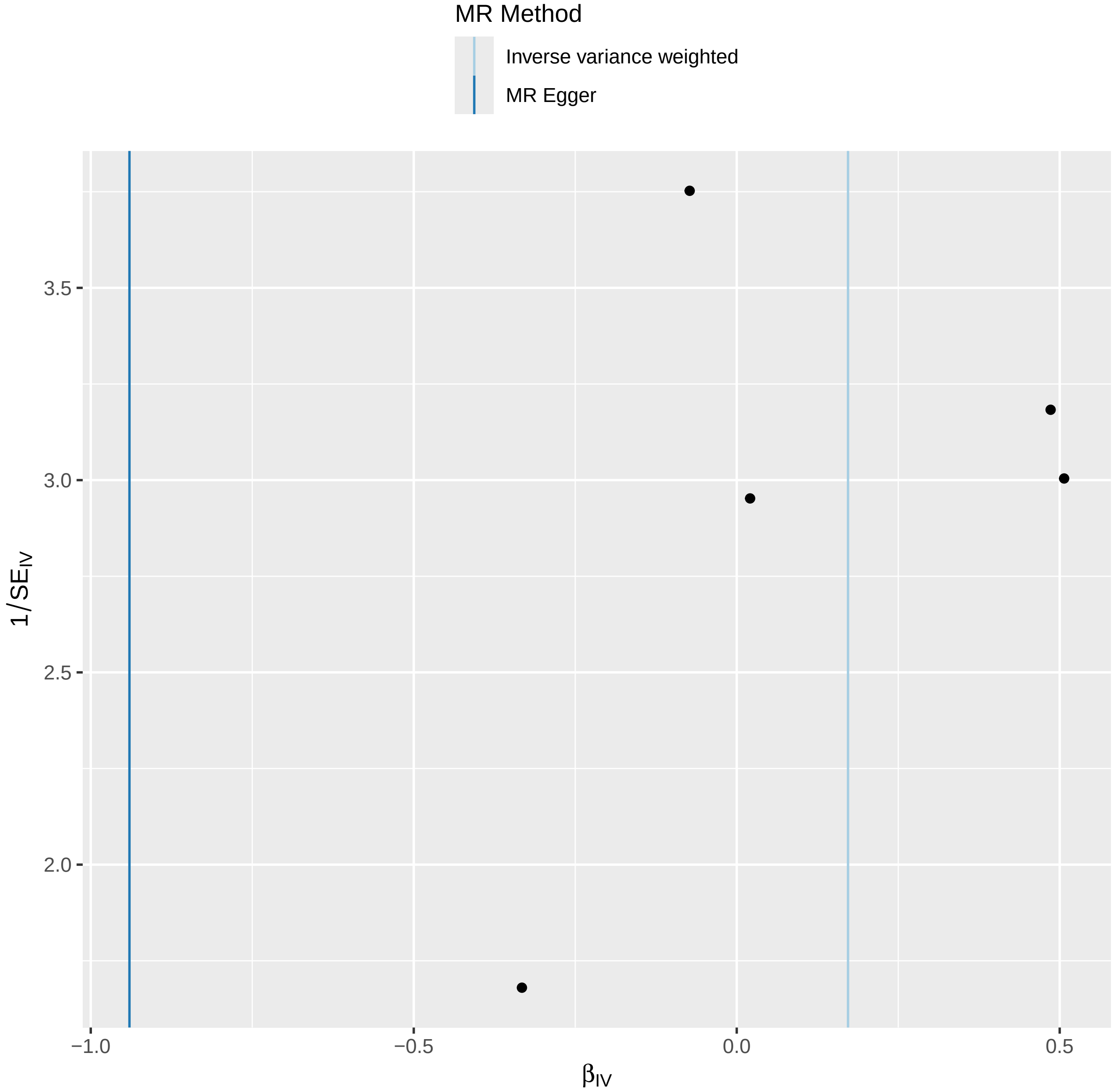
MR Method
Inverse variance weighted
MR Egger
3.5
3.0
2.5
2.0
1/SE_IV
−1.0
−0.5
0.0
0.5
β_IV

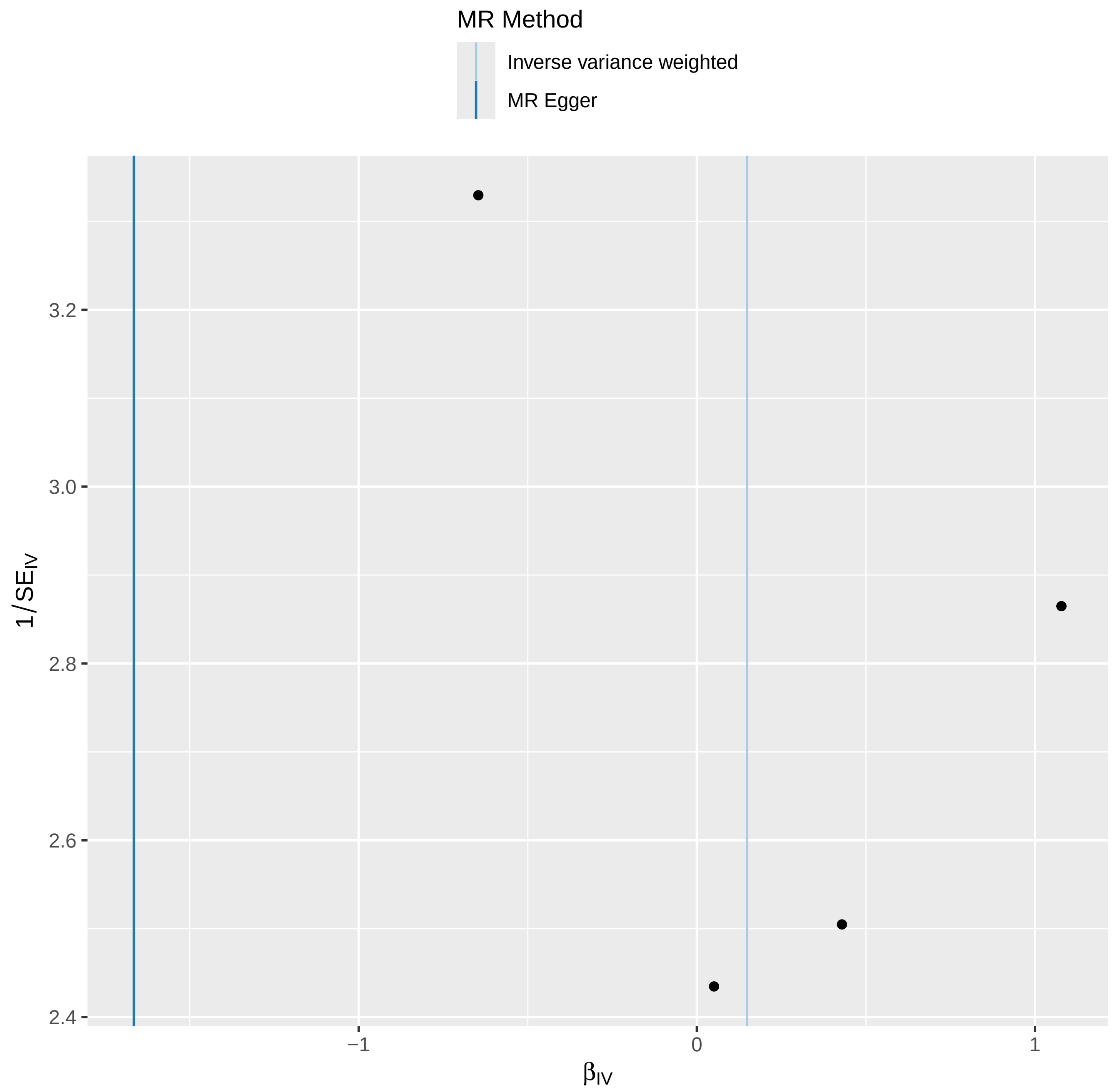

MR Method
Inverse variance weighted
MR Egger
3.2
3.0
2.8
2.6
2.4
1/SE_{IV}
−1
0
1
β_{IV}

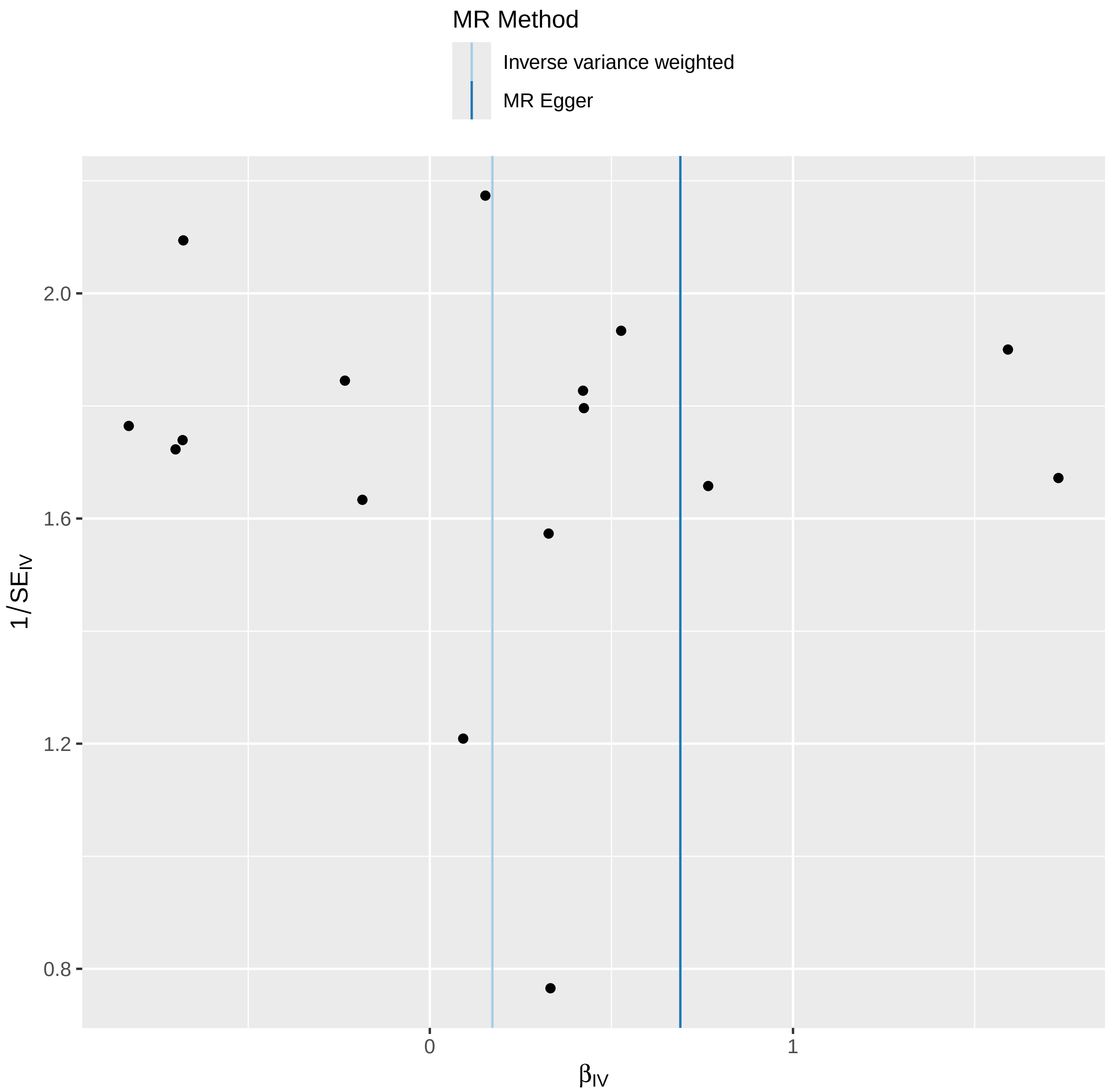

MR Method
Inverse variance weighted
MR Egger
2.0
1.6
1.2
0.8
1/SE_IV
0
1
β_IV

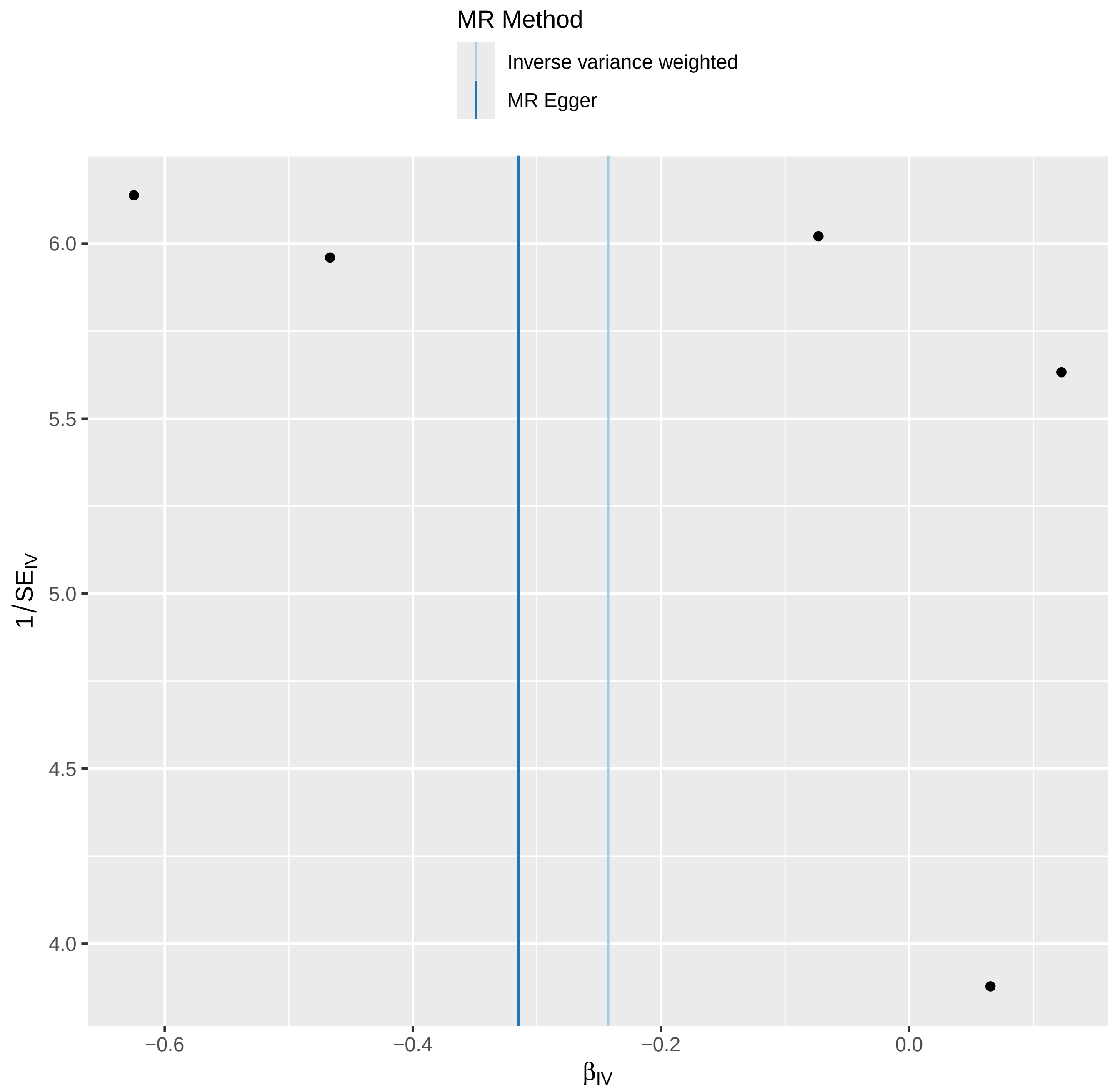

MR Method
Inverse variance weighted
MR Egger
6.0
5.5
5.0
4.5
4.0
1/SE_IV
−0.6
−0.4
−0.2
0.0
β_IV

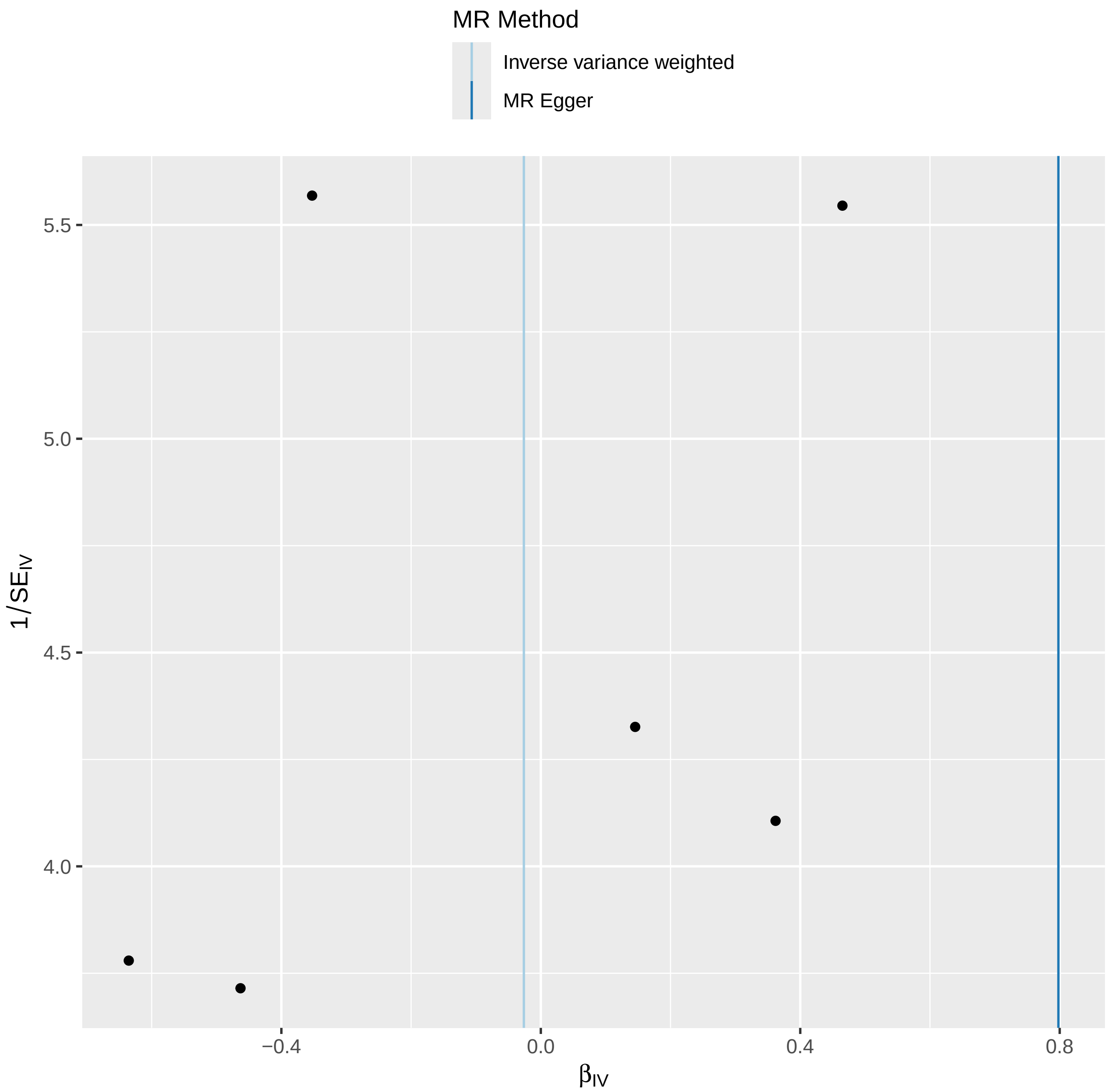
MR Method
Inverse variance weighted
MR Egger
5.5
5.0
4.5
4.0
$1/SE_{IV}$
−0.4
0.0
0.4
0.8
β_{IV}

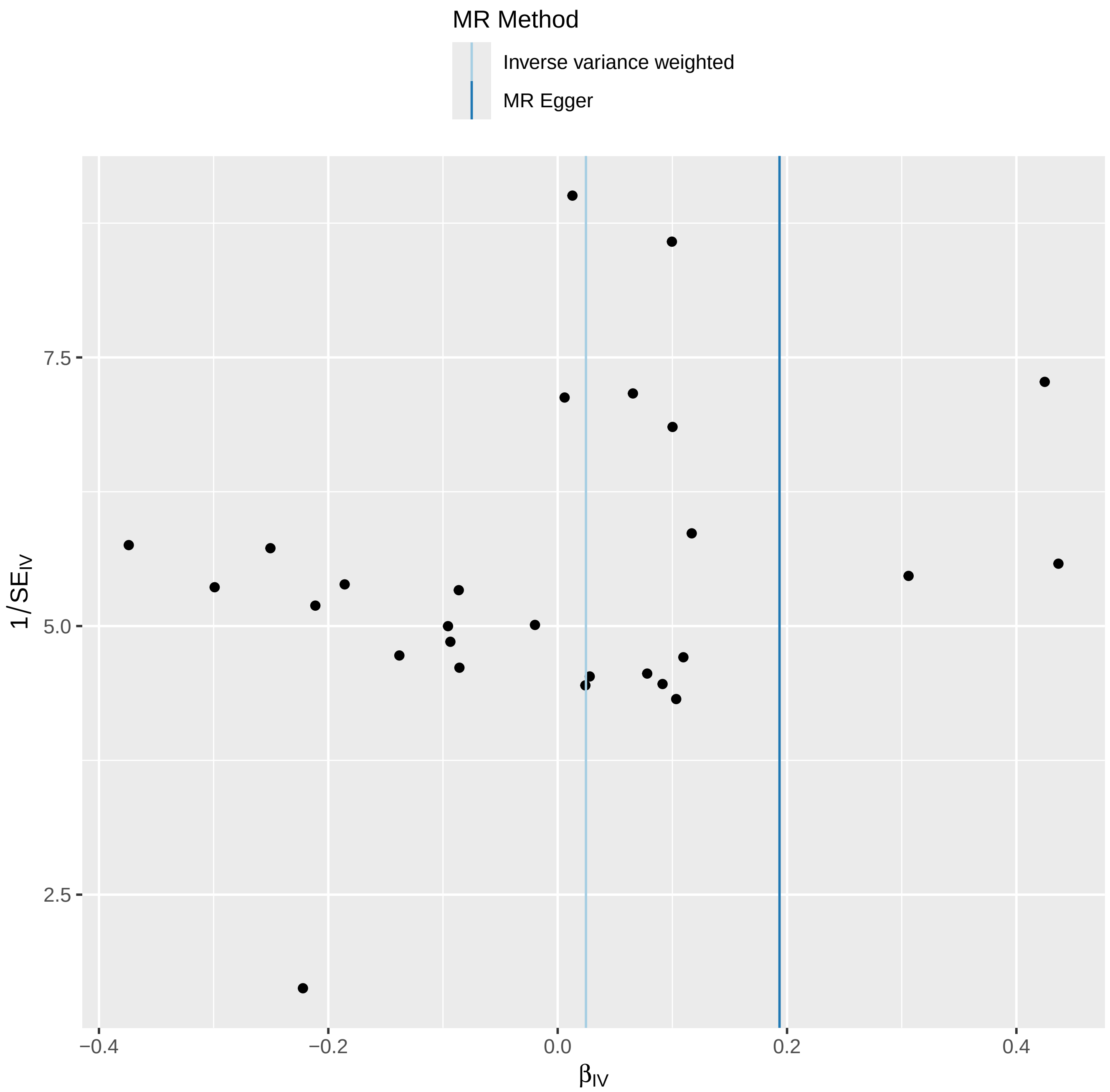
MR Method
Inverse variance weighted
MR Egger
7.5
5.0
2.5
1/SE_IV
−0.4
−0.2
0.0
0.2
0.4
β_IV

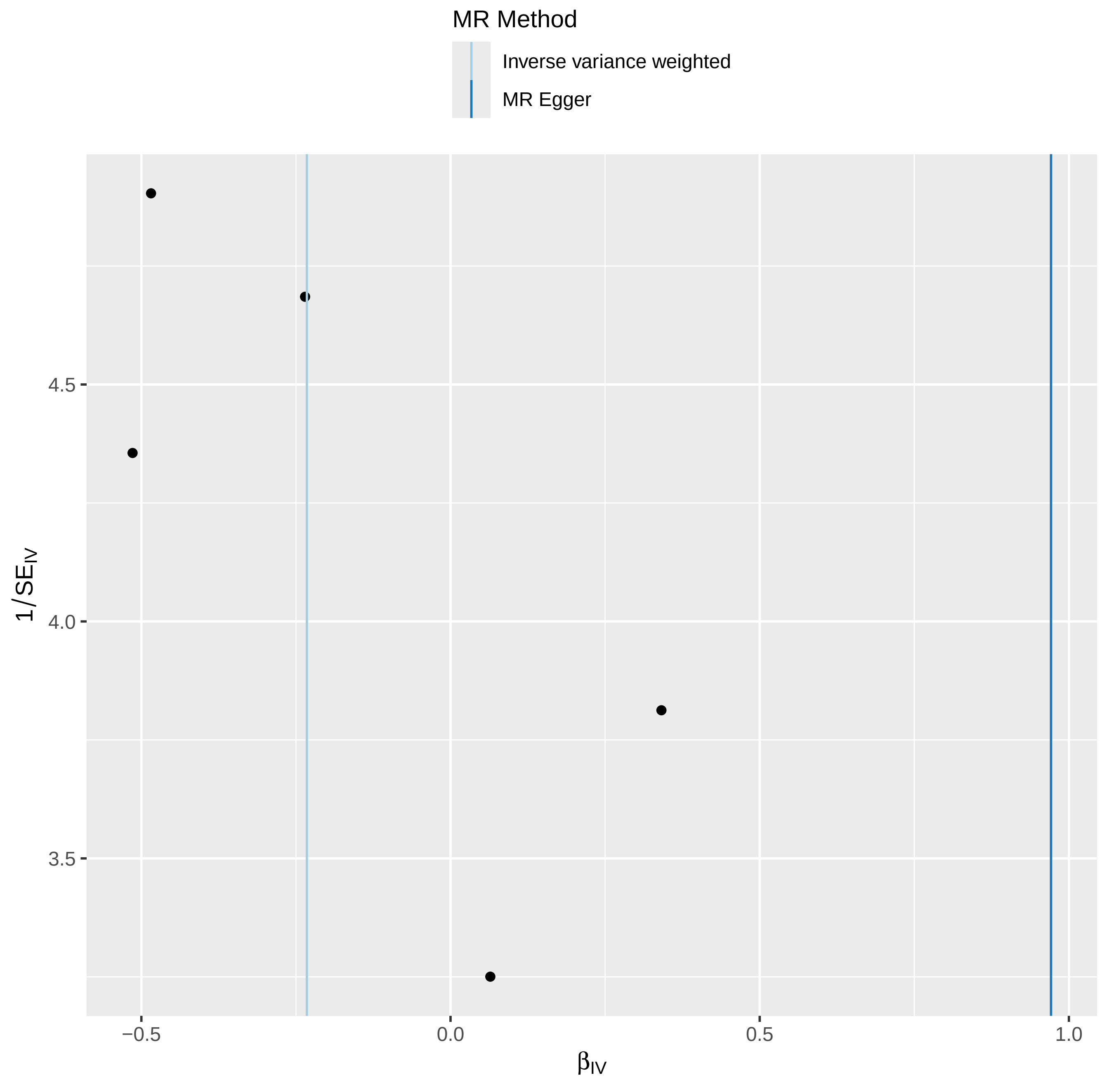
MR Method
Inverse variance weighted
MR Egger
4.5
4.0
3.5
1/SE_IV
−0.5
0.0
0.5
1.0
β_IV

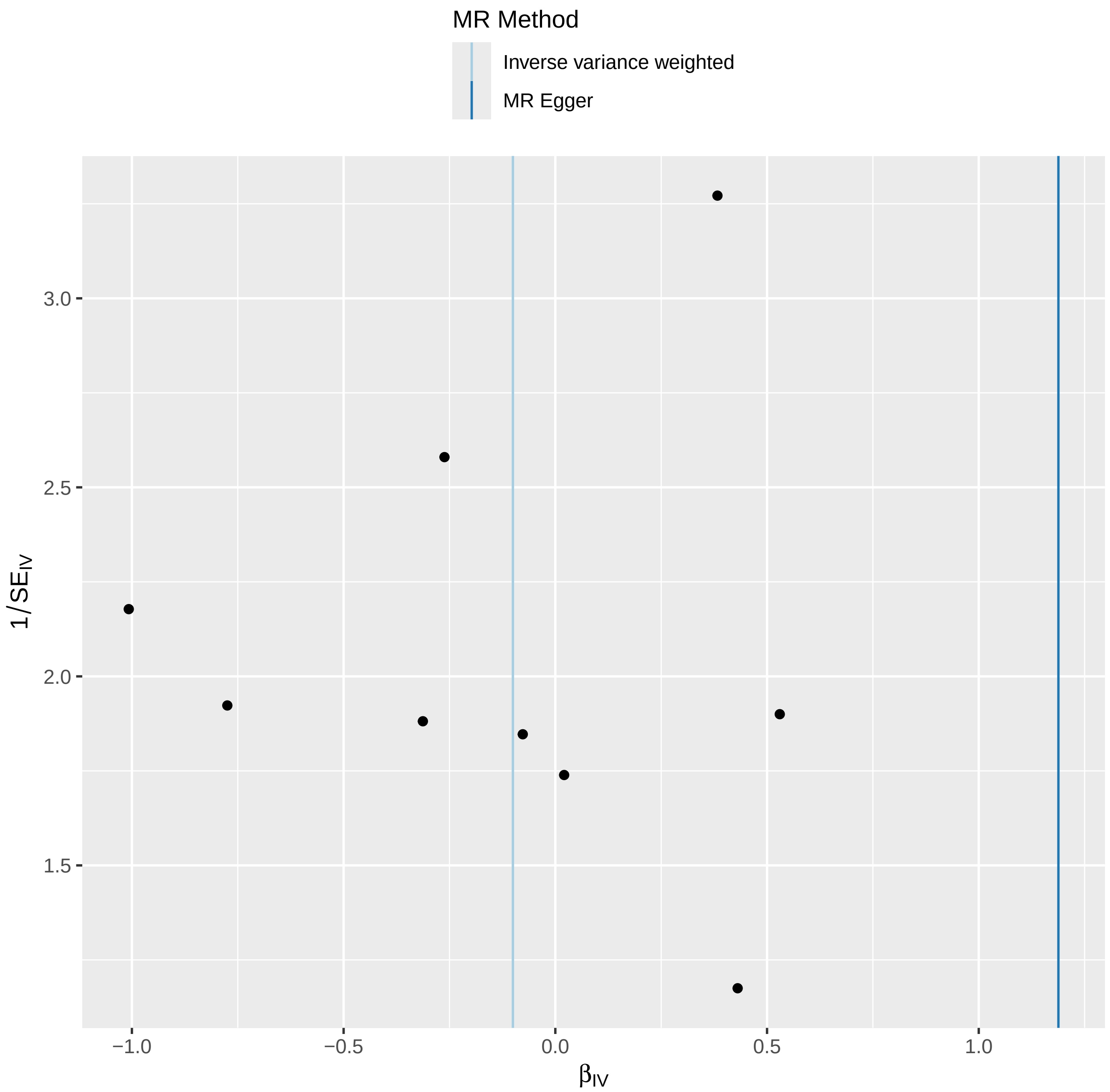

MR Method
Inverse variance weighted
MR Egger
3.0
2.5
2.0
1.5
1/SE$_{IV}$
−1.0
−0.5
0.0
0.5
1.0
β$_{IV}$

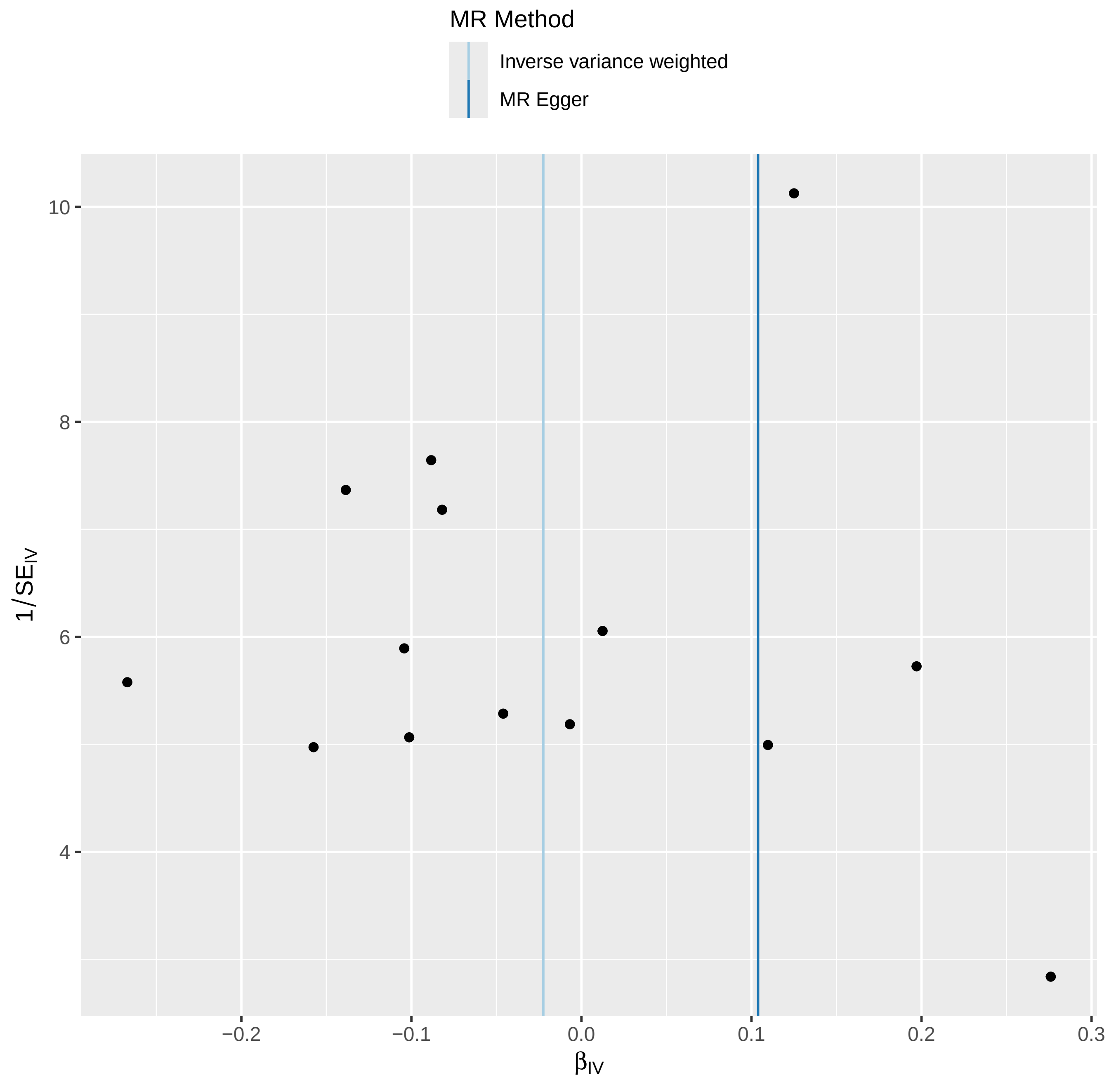

MR Method
Inverse variance weighted
MR Egger
10
8
6
4
1/SE_IV
−0.2
−0.1
0.0
0.1
0.2
0.3
β_IV

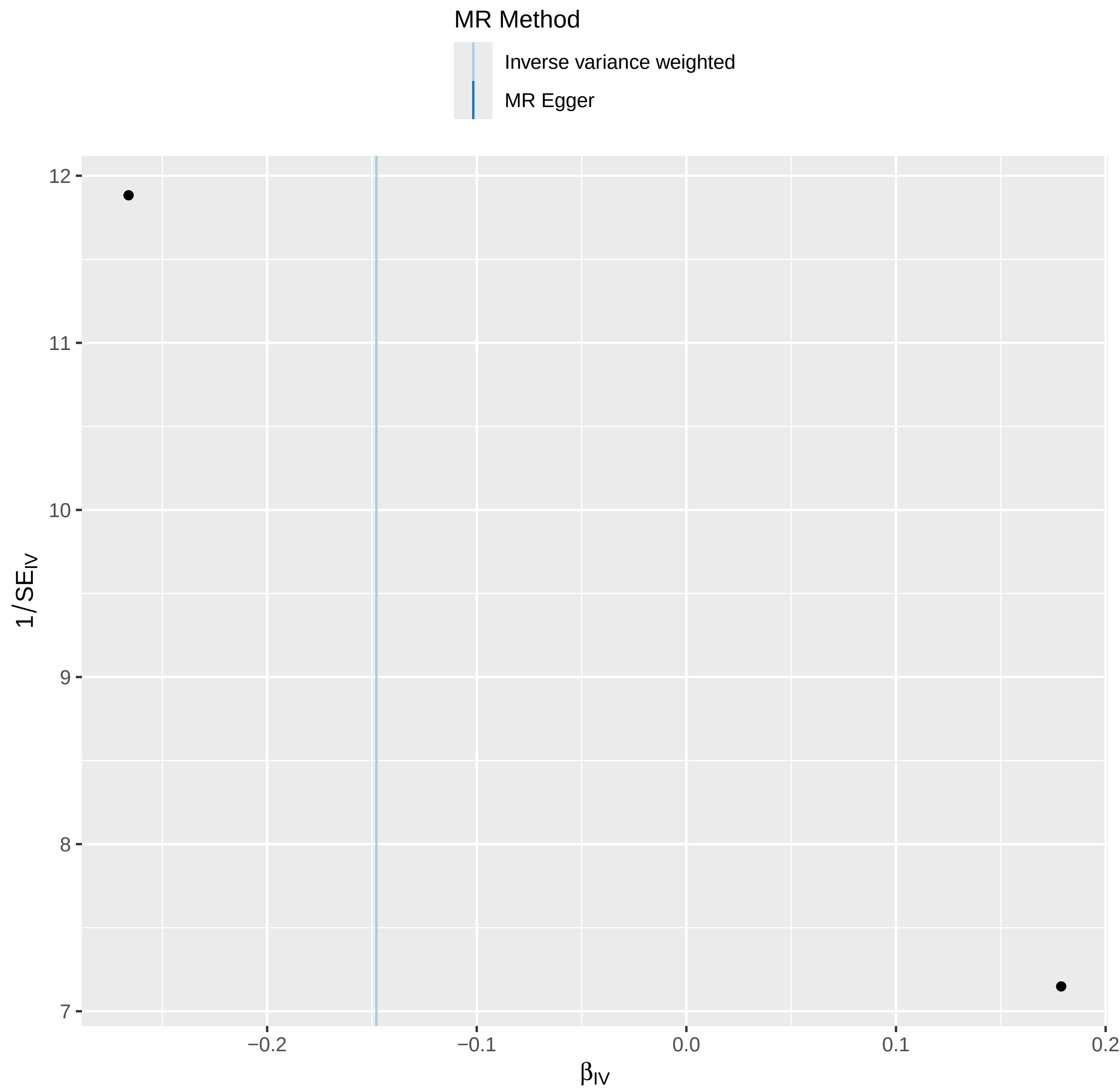

MR Method
Inverse variance weighted
MR Egger
12
11
10
9
8
7
1/SE_IV
−0.2
−0.1
0.0
0.1
0.2
β_IV

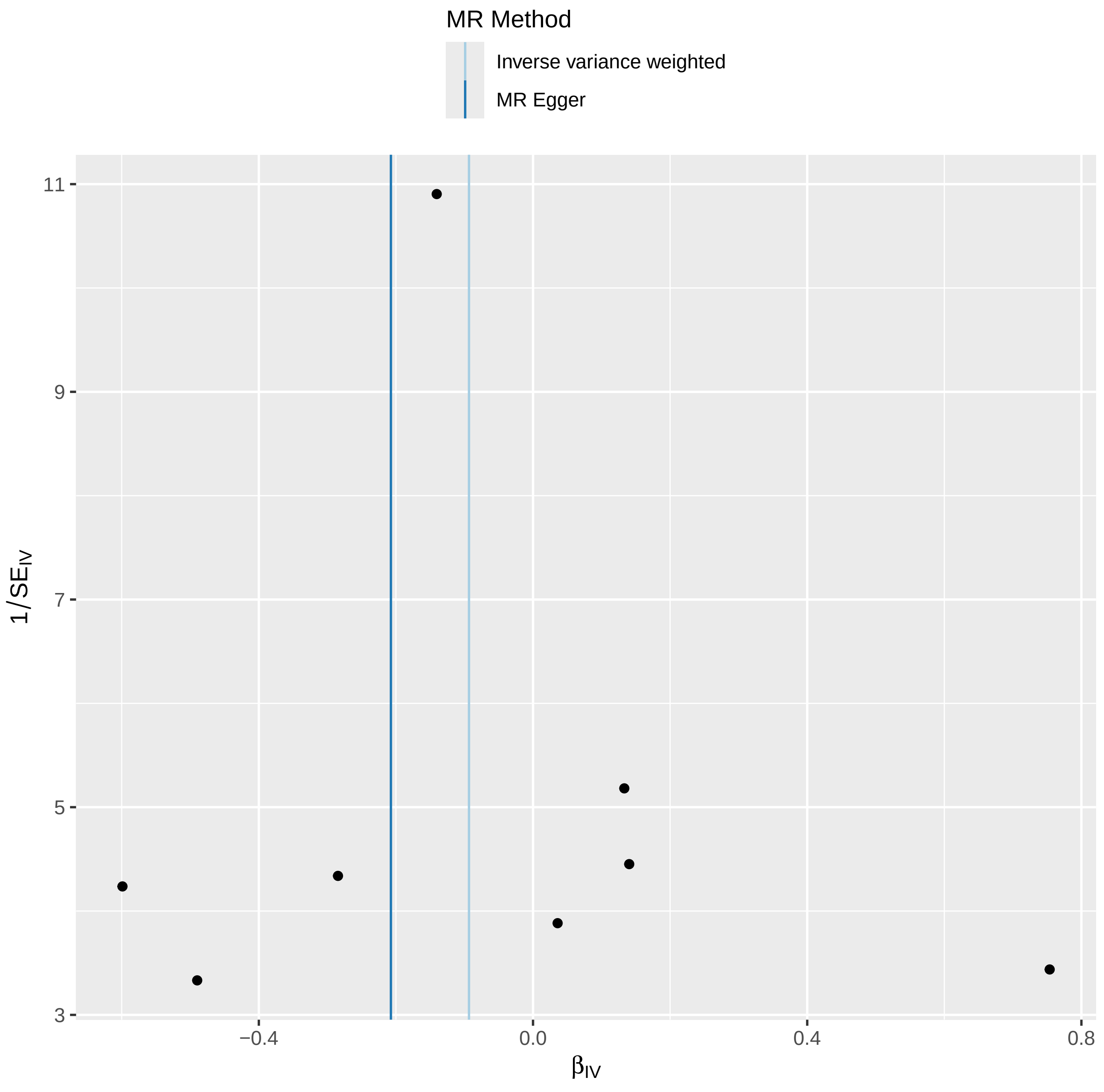
MR Method
Inverse variance weighted
MR Egger
11
9
7
5
3
$1/SE_{IV}$
−0.4
0.0
0.4
0.8
β_{IV}

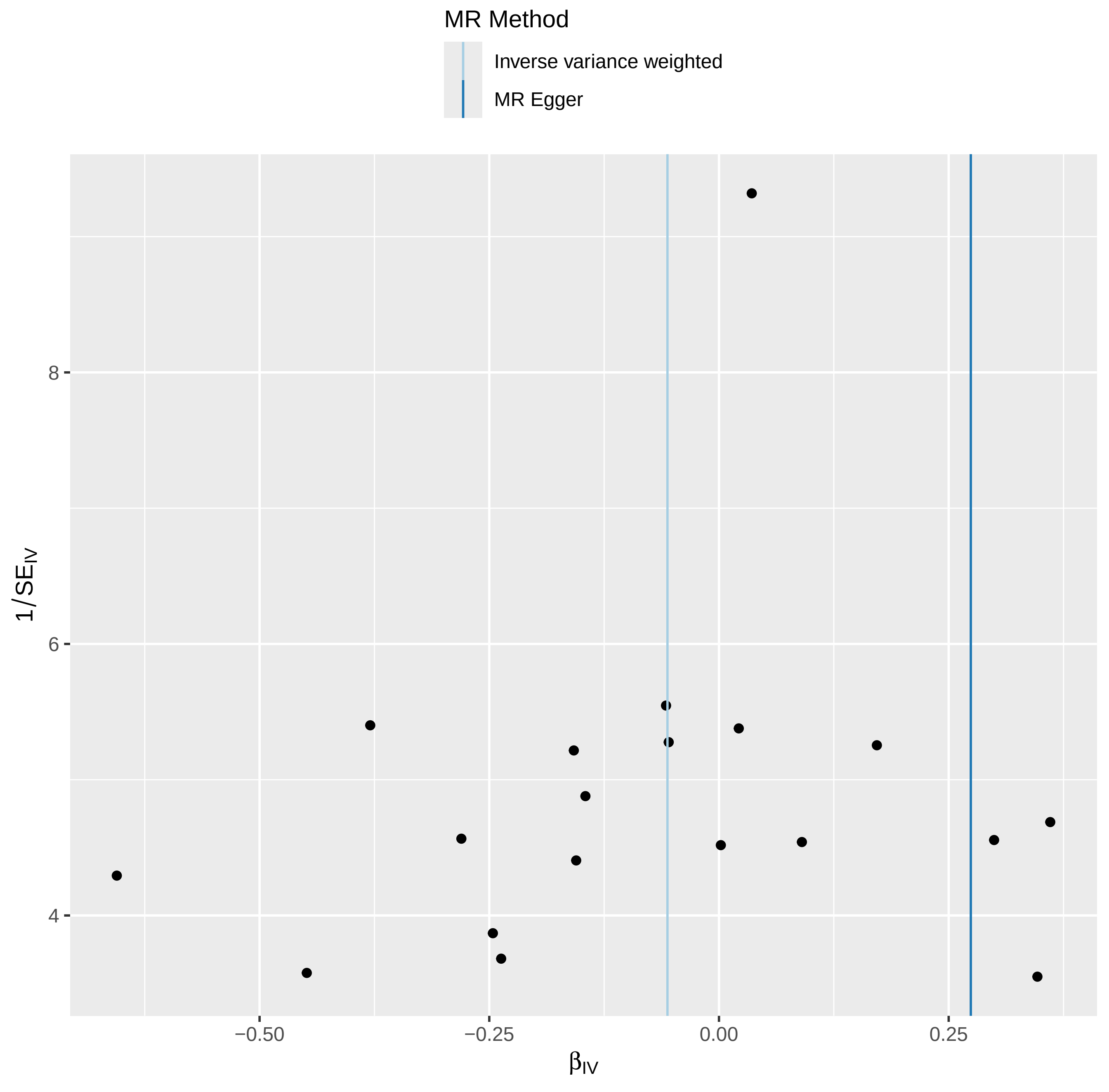

MR Method
Inverse variance weighted
MR Egger
8
6
4
1/SE_IV
−0.50
−0.25
0.00
0.25
β_IV

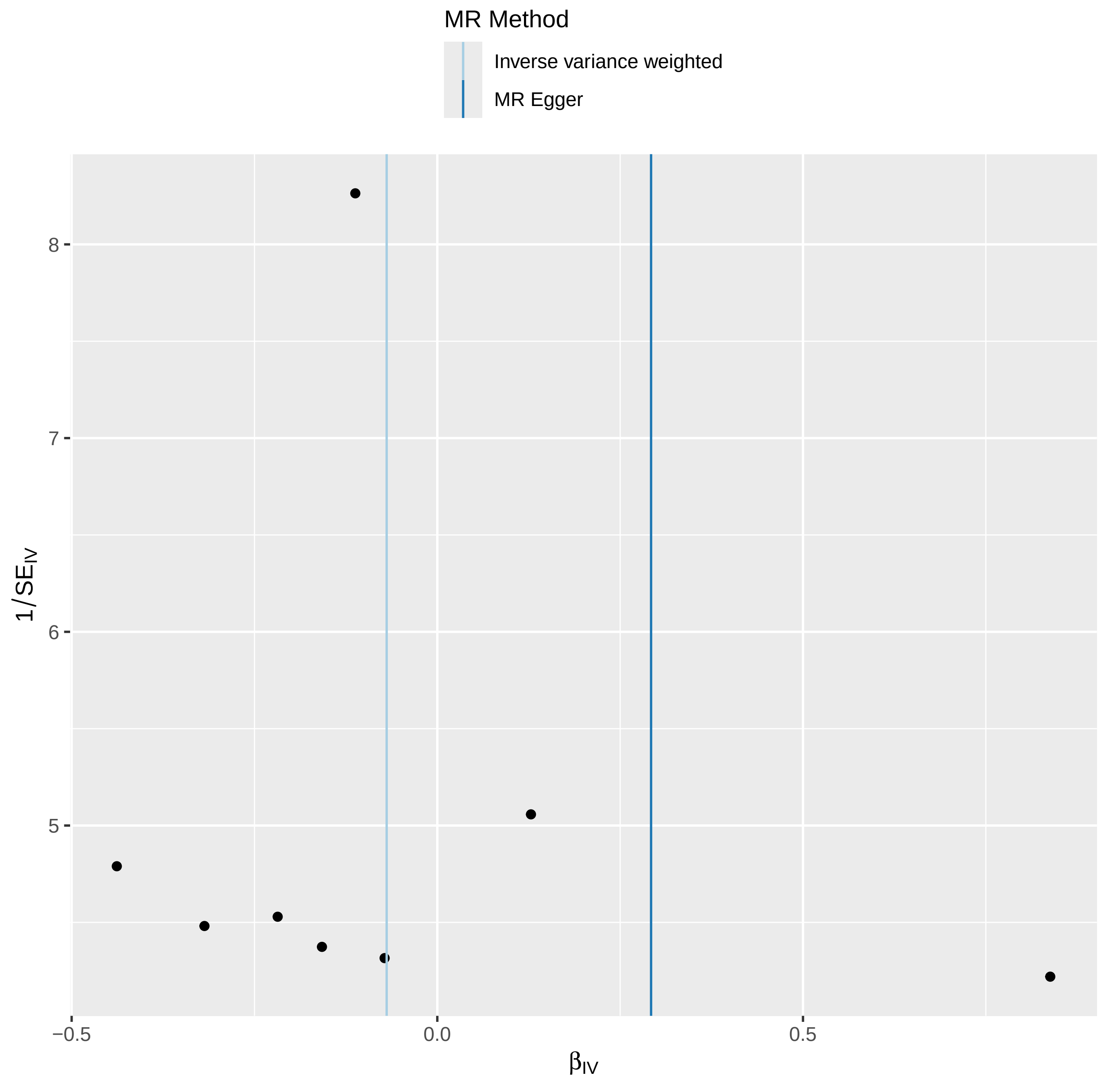
MR Method
Inverse variance weighted
MR Egger
8
7
6
5
1/SE$_{IV}$
−0.5
0.0
0.5
β_{IV}

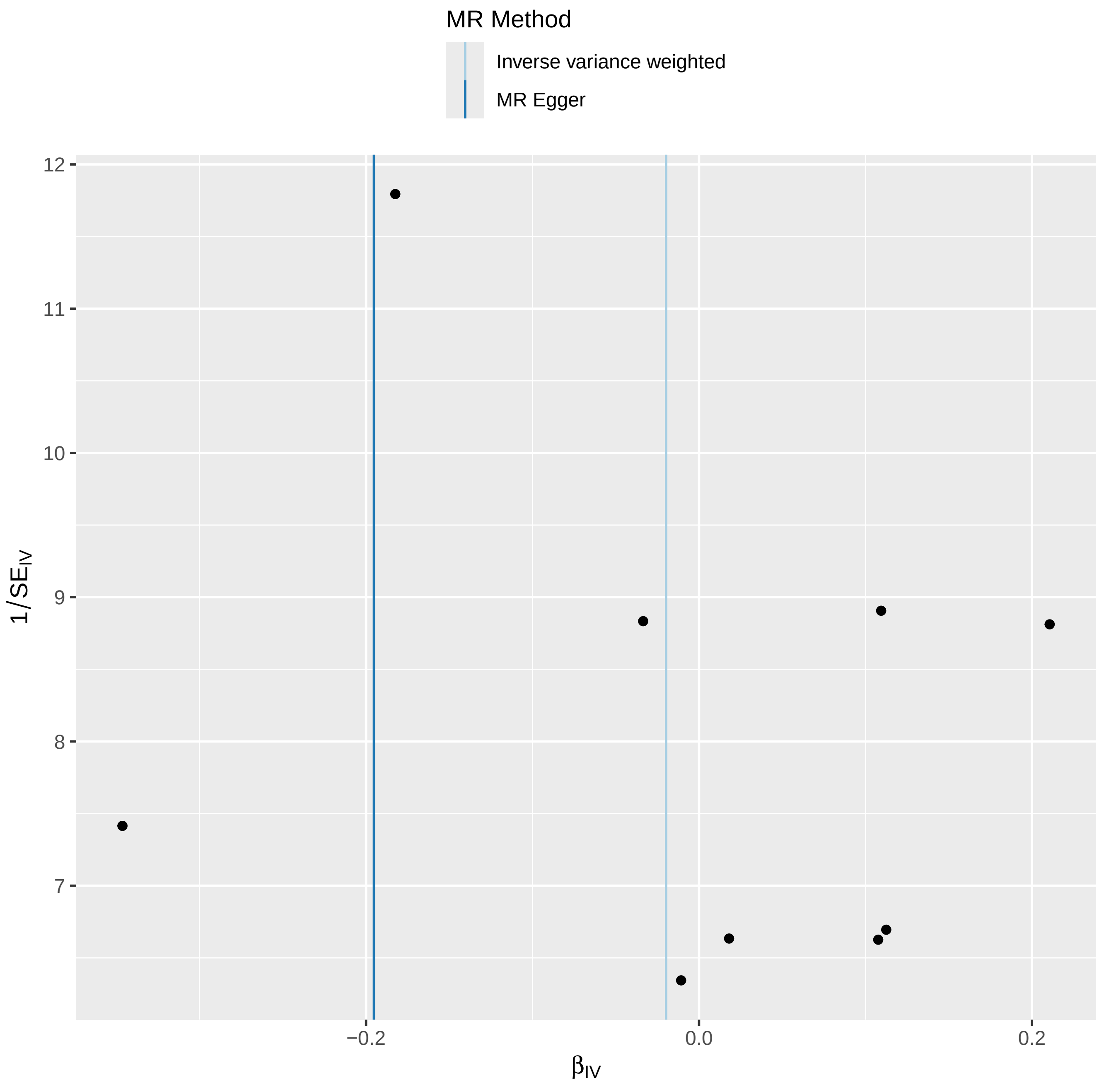

MR Method
Inverse variance weighted
MR Egger
12
11
10
9
8
7
$1/SE_{IV}$
−0.2
0.0
0.2
β_{IV}

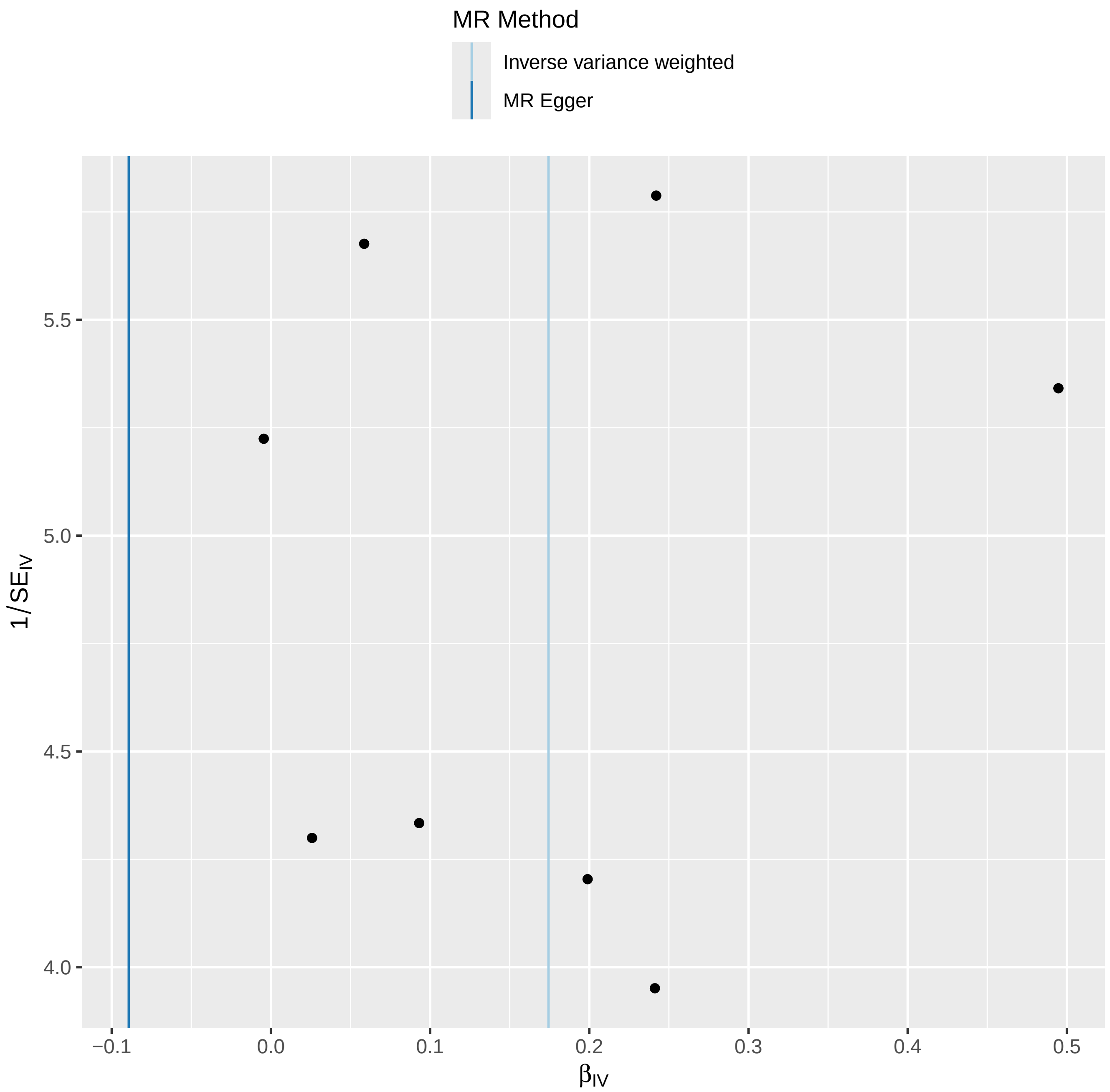

MR Method
Inverse variance weighted
MR Egger
5.5
5.0
4.5
4.0
1/SE$_{IV}$
−0.1
0.0
0.1
0.2
0.3
0.4
0.5
β$_{IV}$

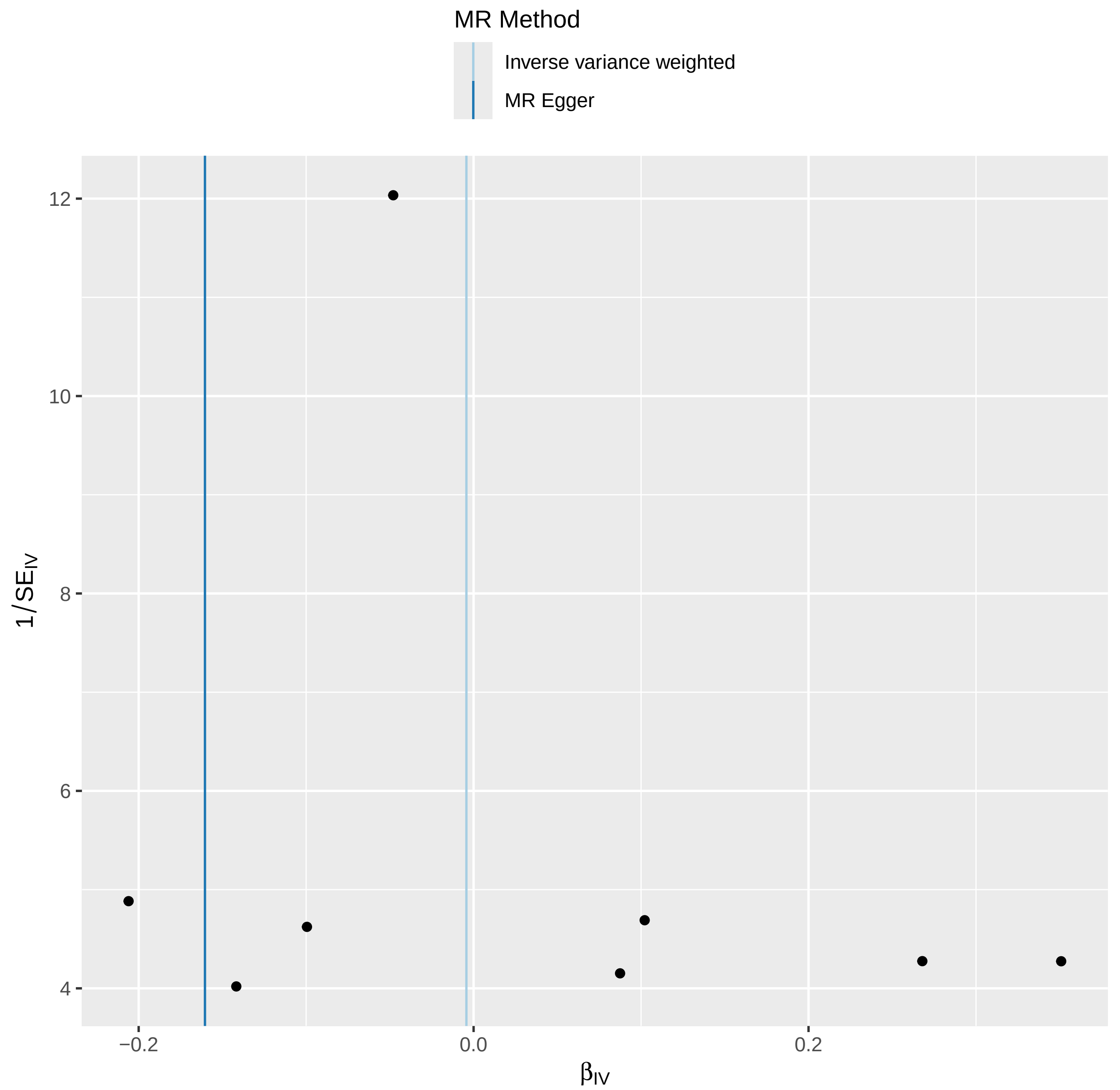

MR Method
Inverse variance weighted
MR Egger
12
10
8
6
4
$1/SE_{IV}$
−0.2
0.0
0.2
β_{IV}

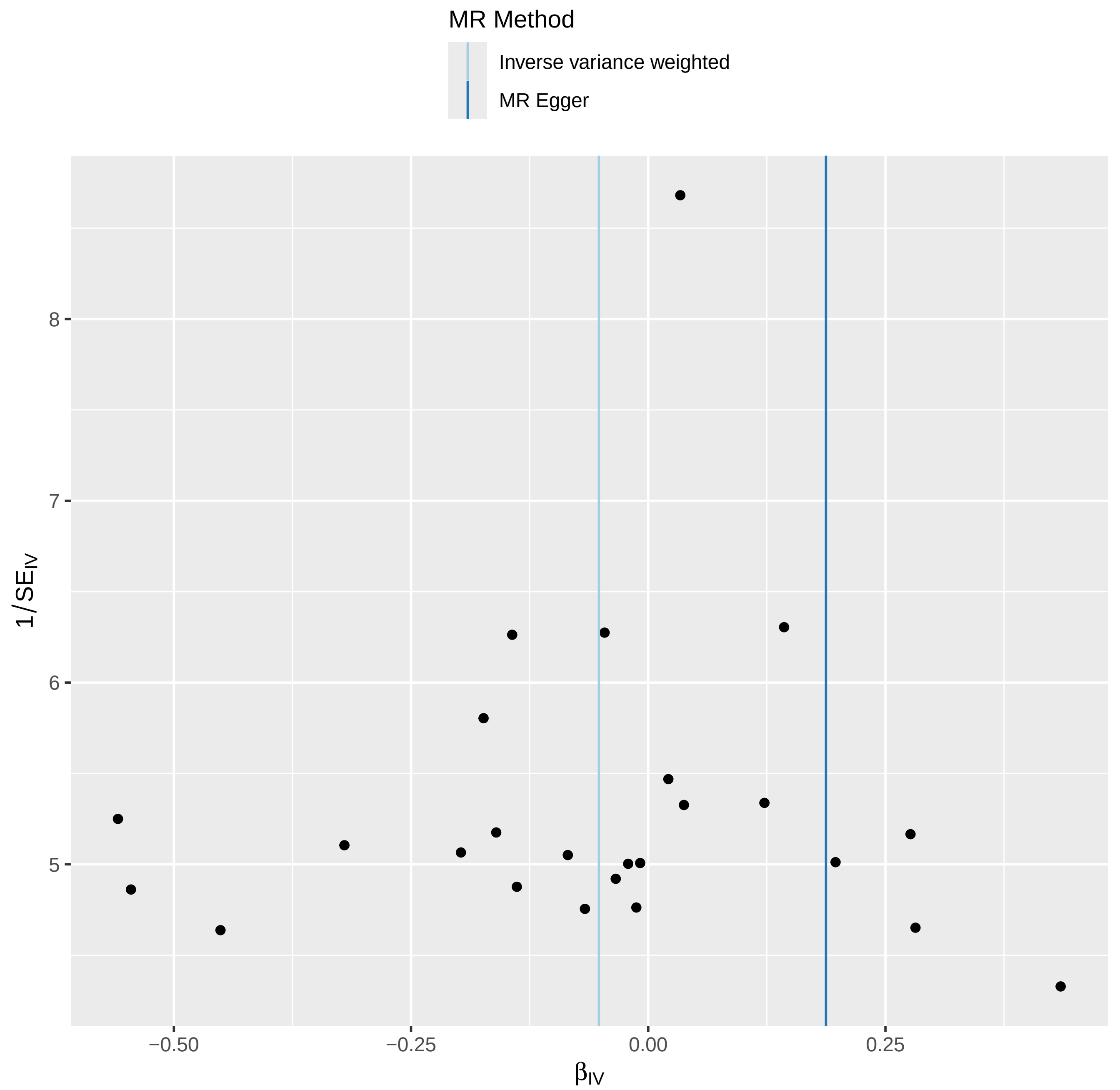

MR Method
Inverse variance weighted
MR Egger
8
7
6
5
1/SE_IV
−0.50
−0.25
0.00
0.25
β_IV

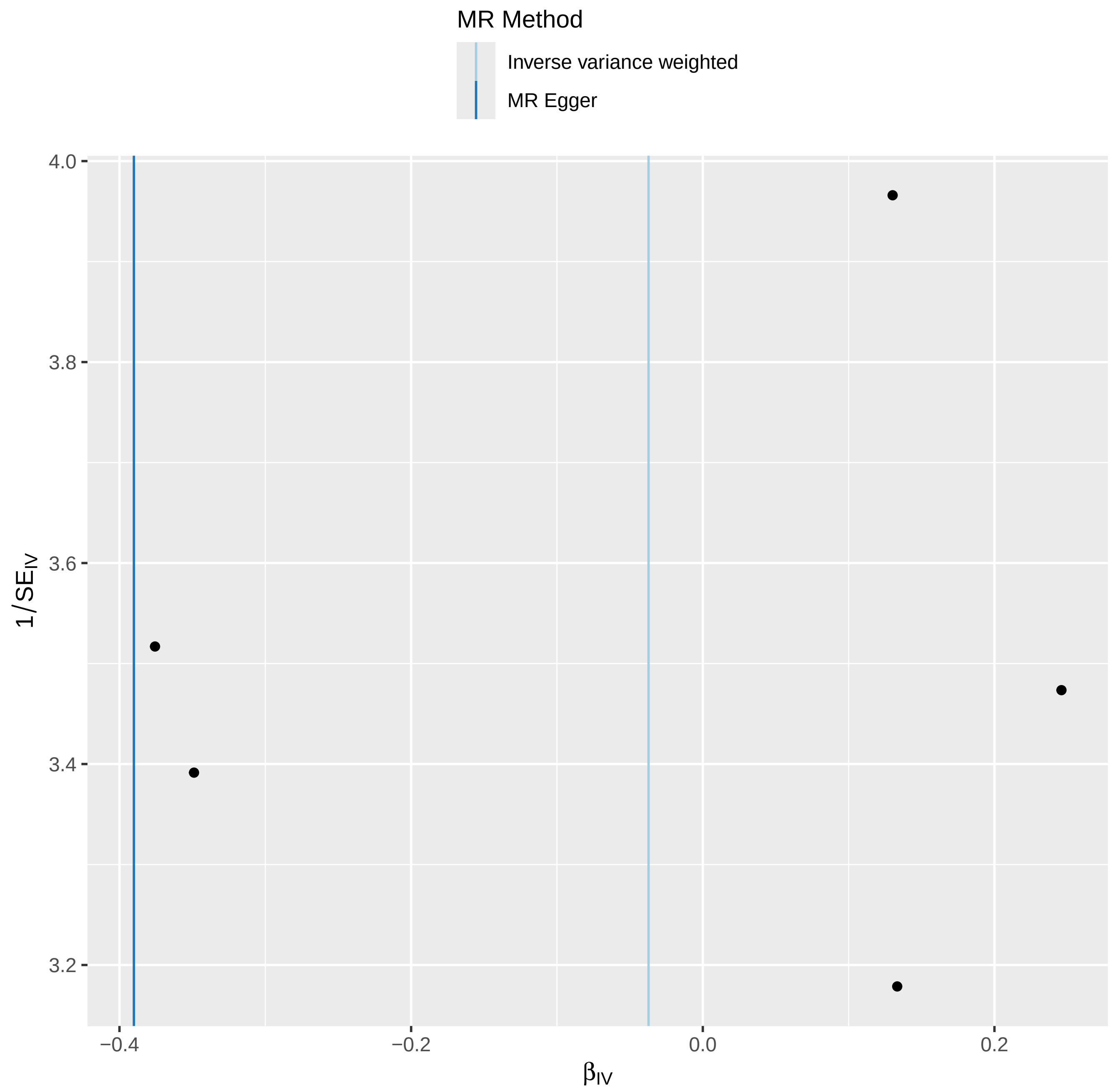
MR Method
Inverse variance weighted
MR Egger
4.0
3.8
3.6
3.4
3.2
1/SE_IV
−0.4
−0.2
0.0
0.2
β_IV

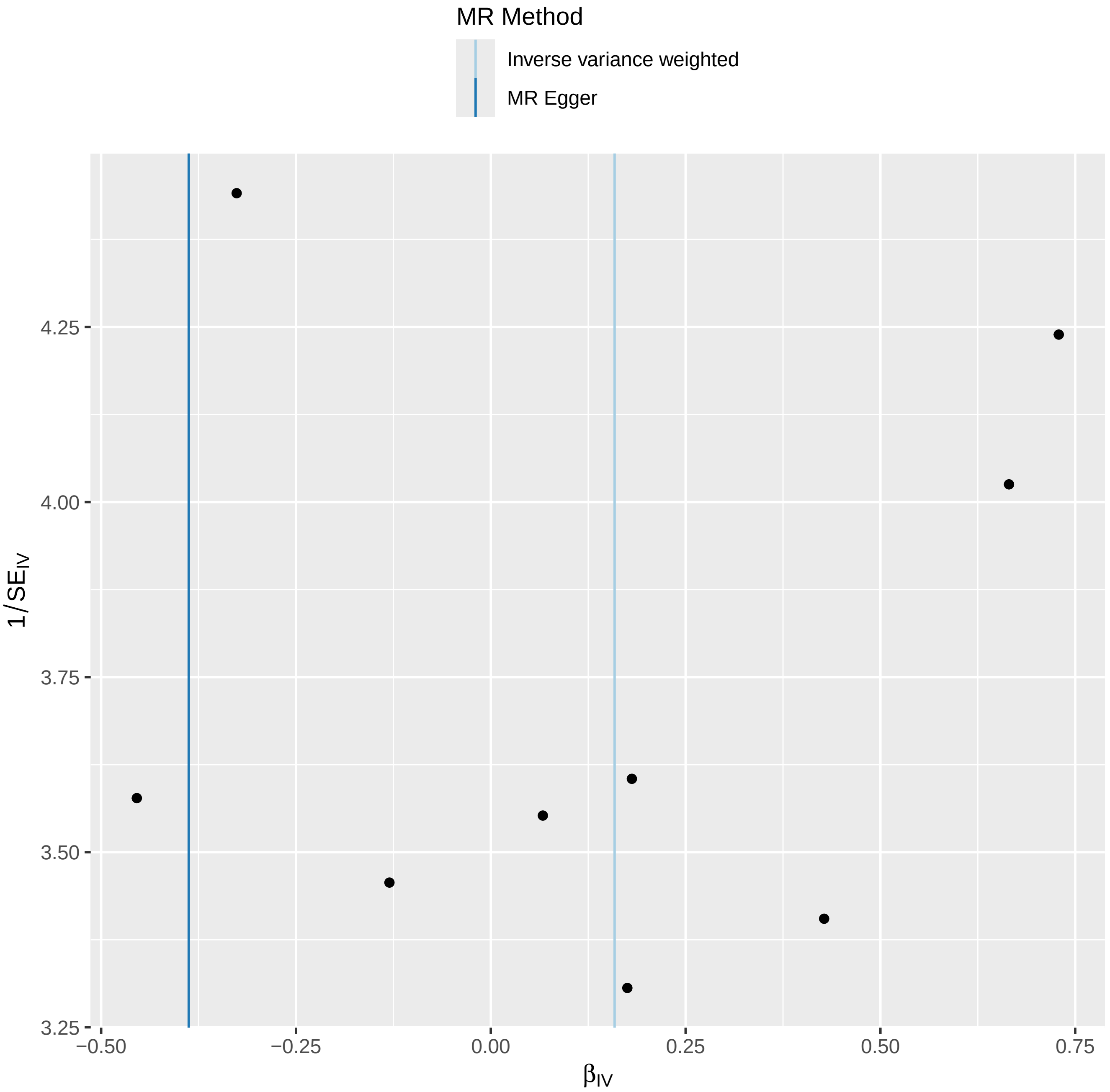

MR Method
Inverse variance weighted
MR Egger
4.25
4.00
3.75
3.50
3.25
$1/SE_{IV}$
−0.50
−0.25
0.00
0.25
0.50
0.75
β_{IV}

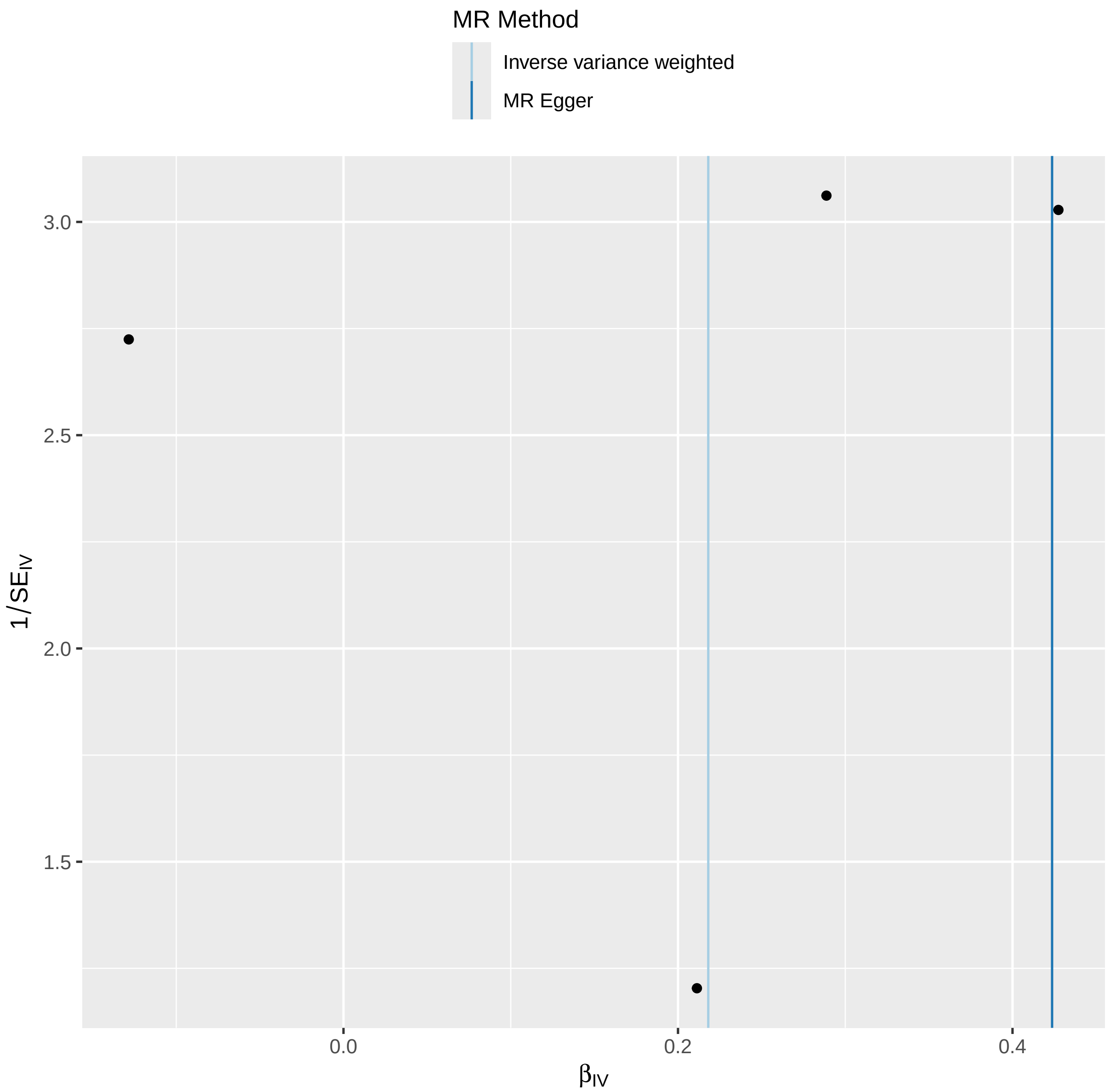
MR Method
Inverse variance weighted
MR Egger
3.0
2.5
2.0
1.5
1/SE$_{IV}$
0.0
0.2
0.4
β_{IV}

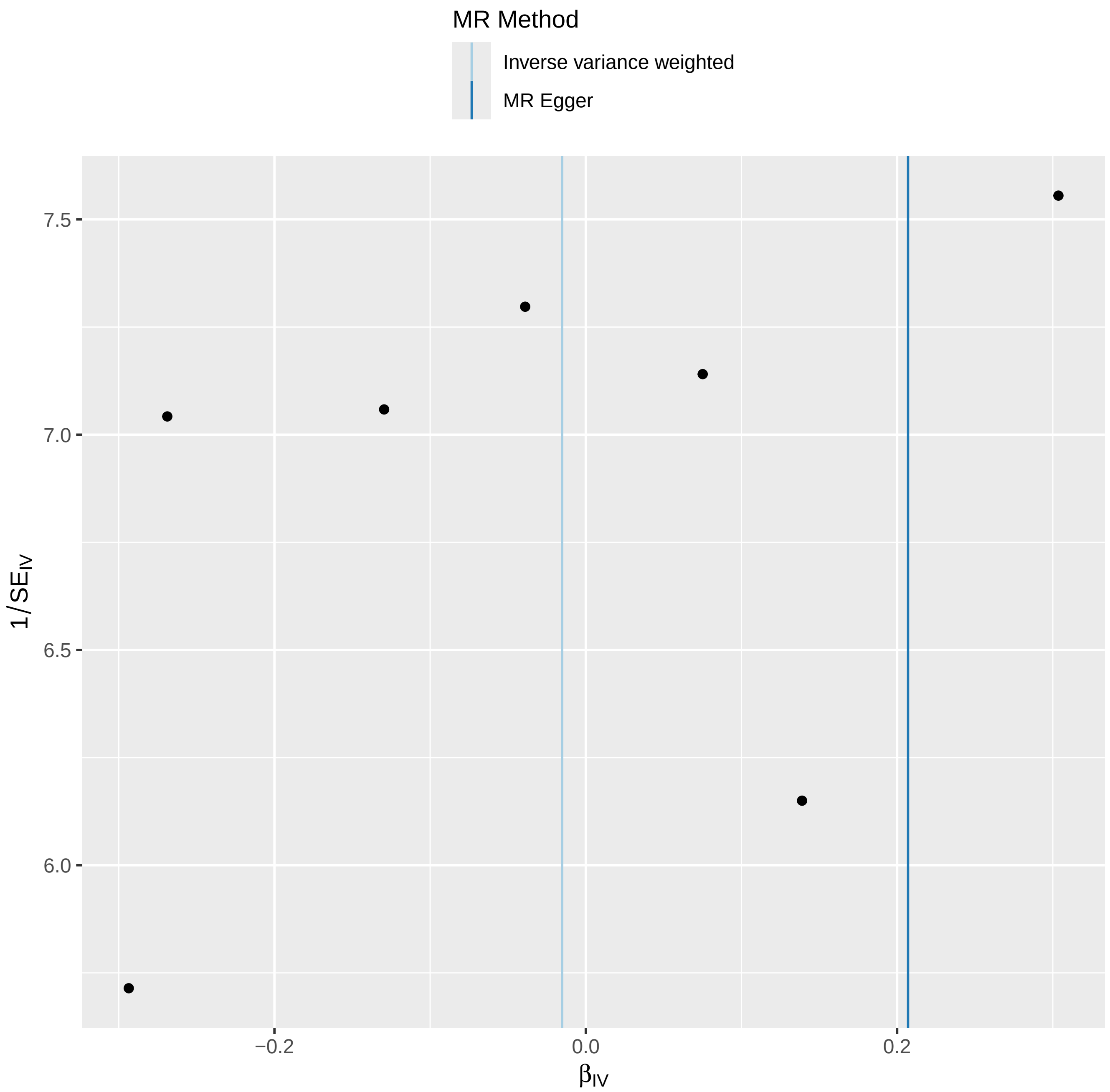
MR Method
Inverse variance weighted
MR Egger
7.5
7.0
6.5
6.0
1/SE_IV
−0.2
0.0
0.2
β_IV

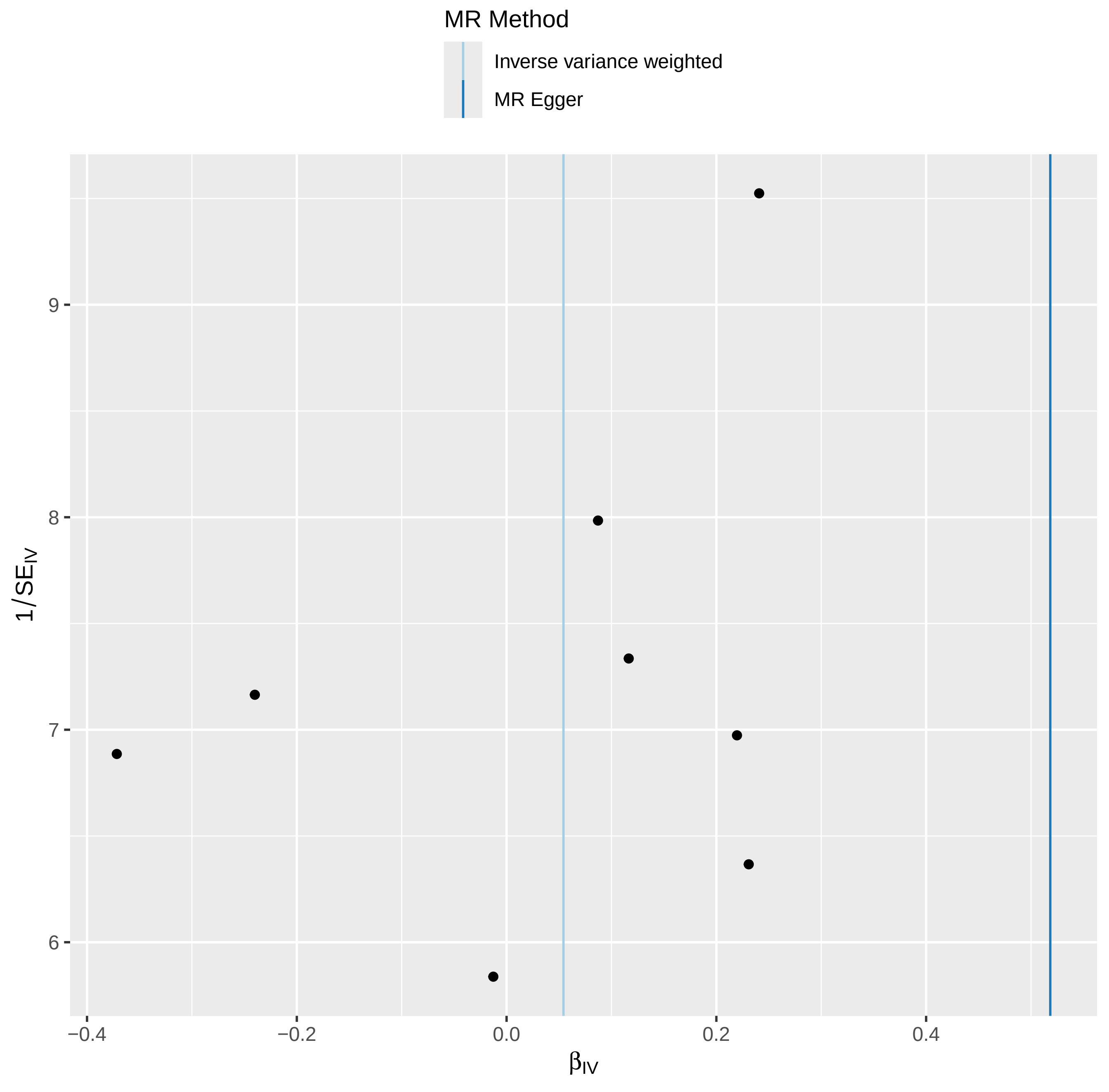

MR Method
Inverse variance weighted
MR Egger
9
8
7
6
1/SE$_{IV}$
−0.4
−0.2
0.0
0.2
0.4
β$_{IV}$

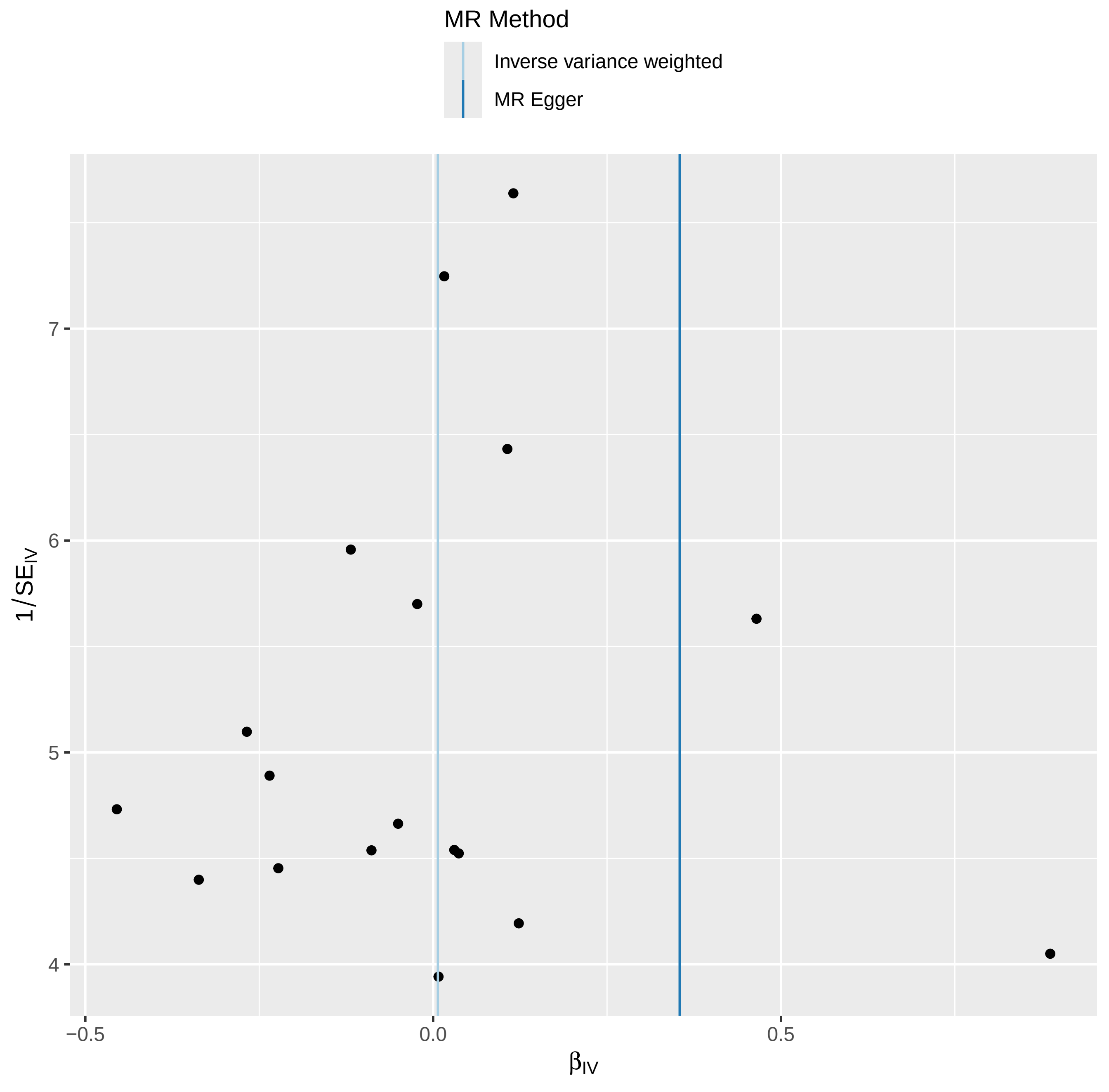
MR Method
Inverse variance weighted
MR Egger
7
6
5
4
1/SE_IV
−0.5
0.0
0.5
β_IV

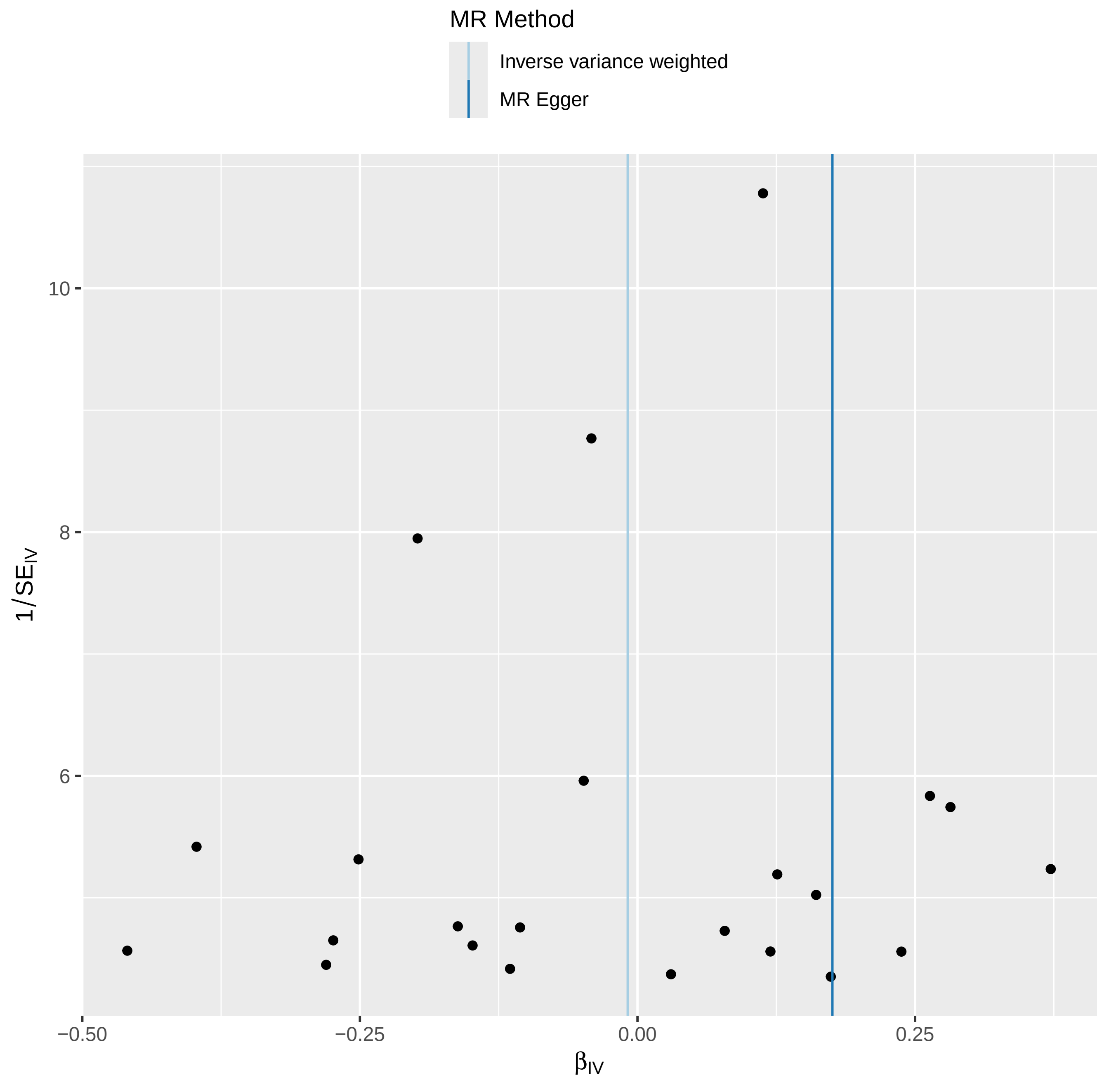

MR Method
Inverse variance weighted
MR Egger
10
8
6
1/SE_IV
−0.50
−0.25
0.00
0.25
β_IV

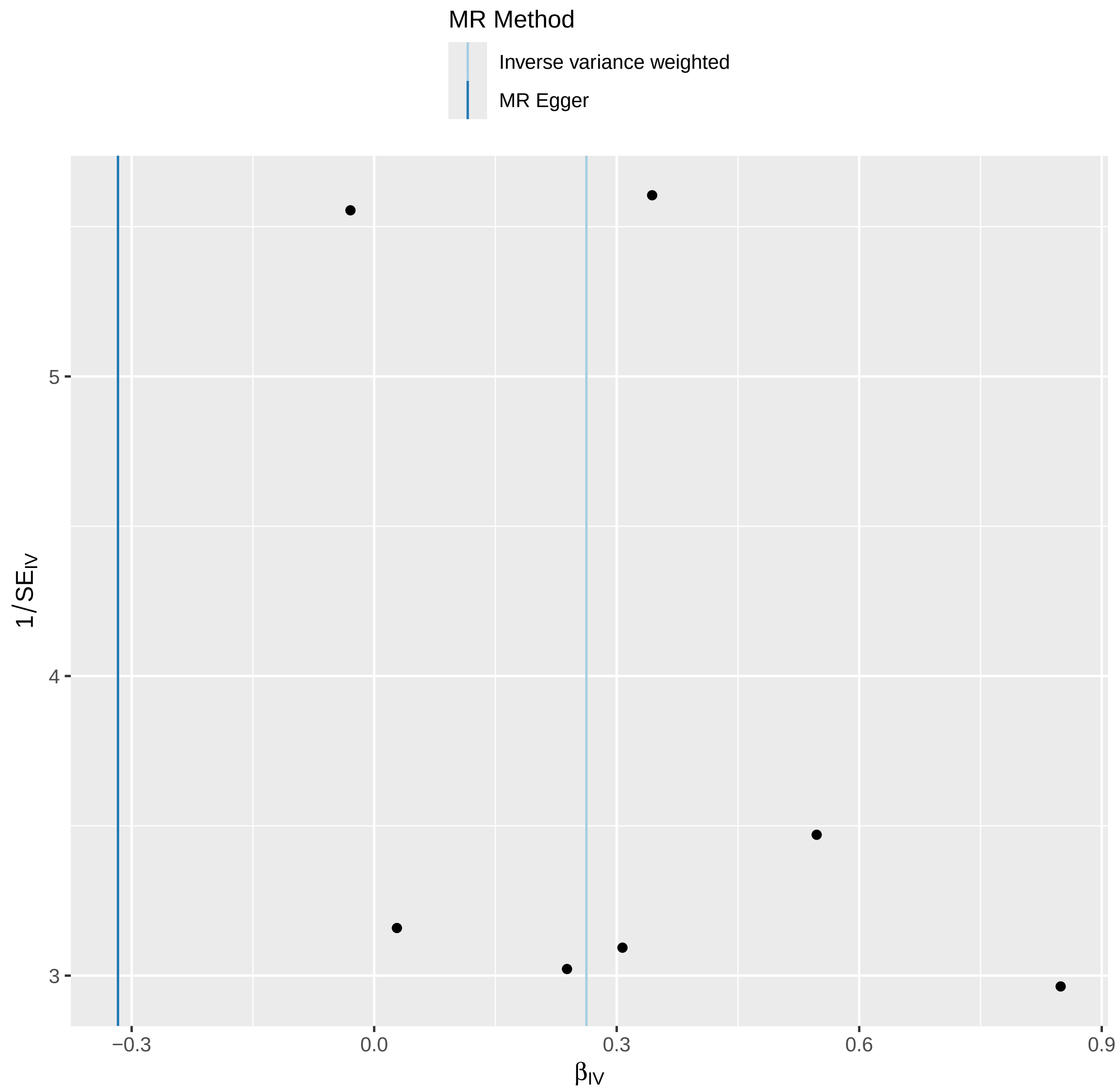
MR Method
Inverse variance weighted
MR Egger
5
4
3
$1/SE_{IV}$
−0.3
0.0
0.3
0.6
0.9
β_{IV}

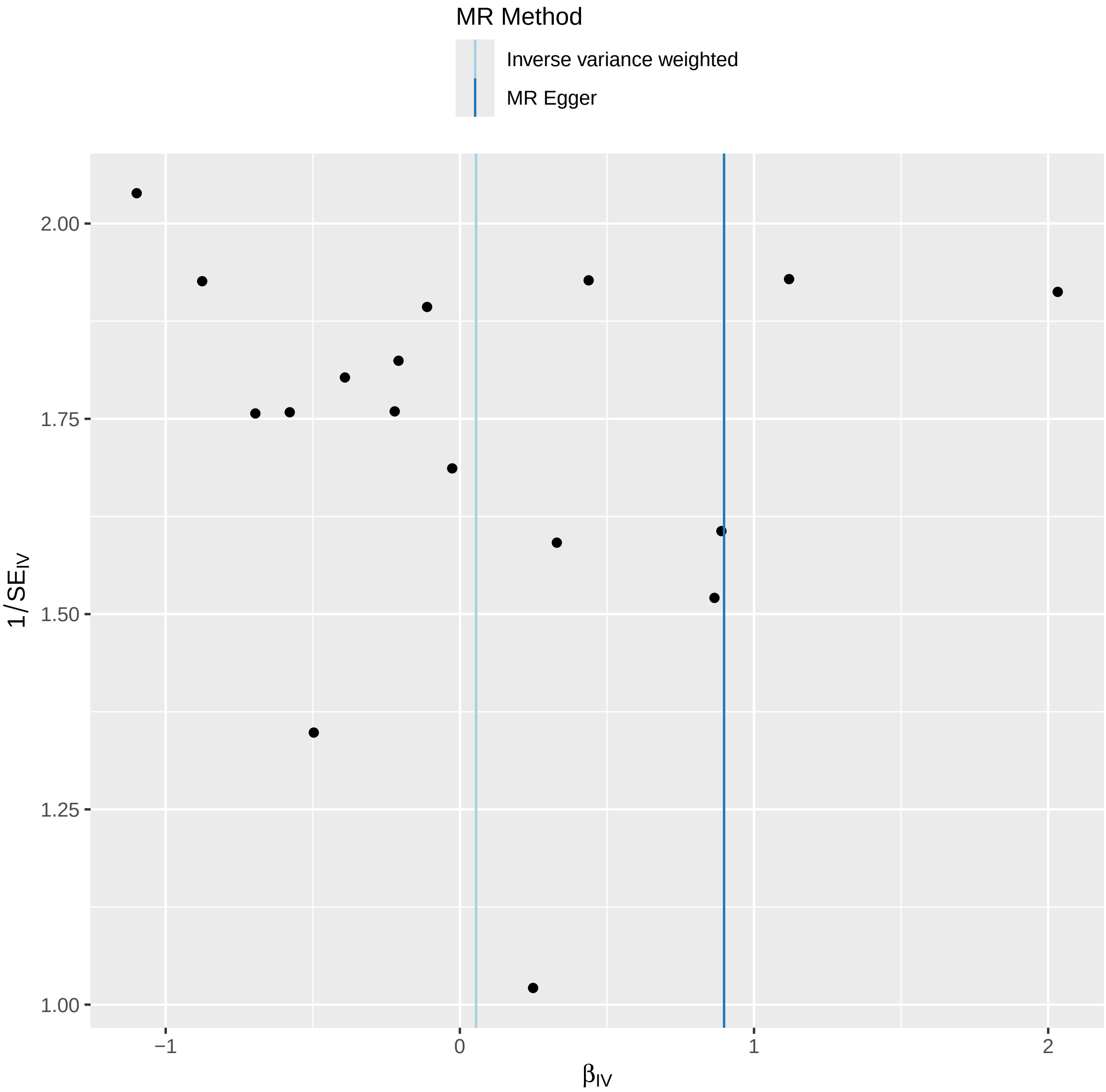

MR Method
Inverse variance weighted
MR Egger
2.00
1.75
1.50
1.25
1.00
$1/SE_{IV}$
−1
0
1
2
β_{IV}

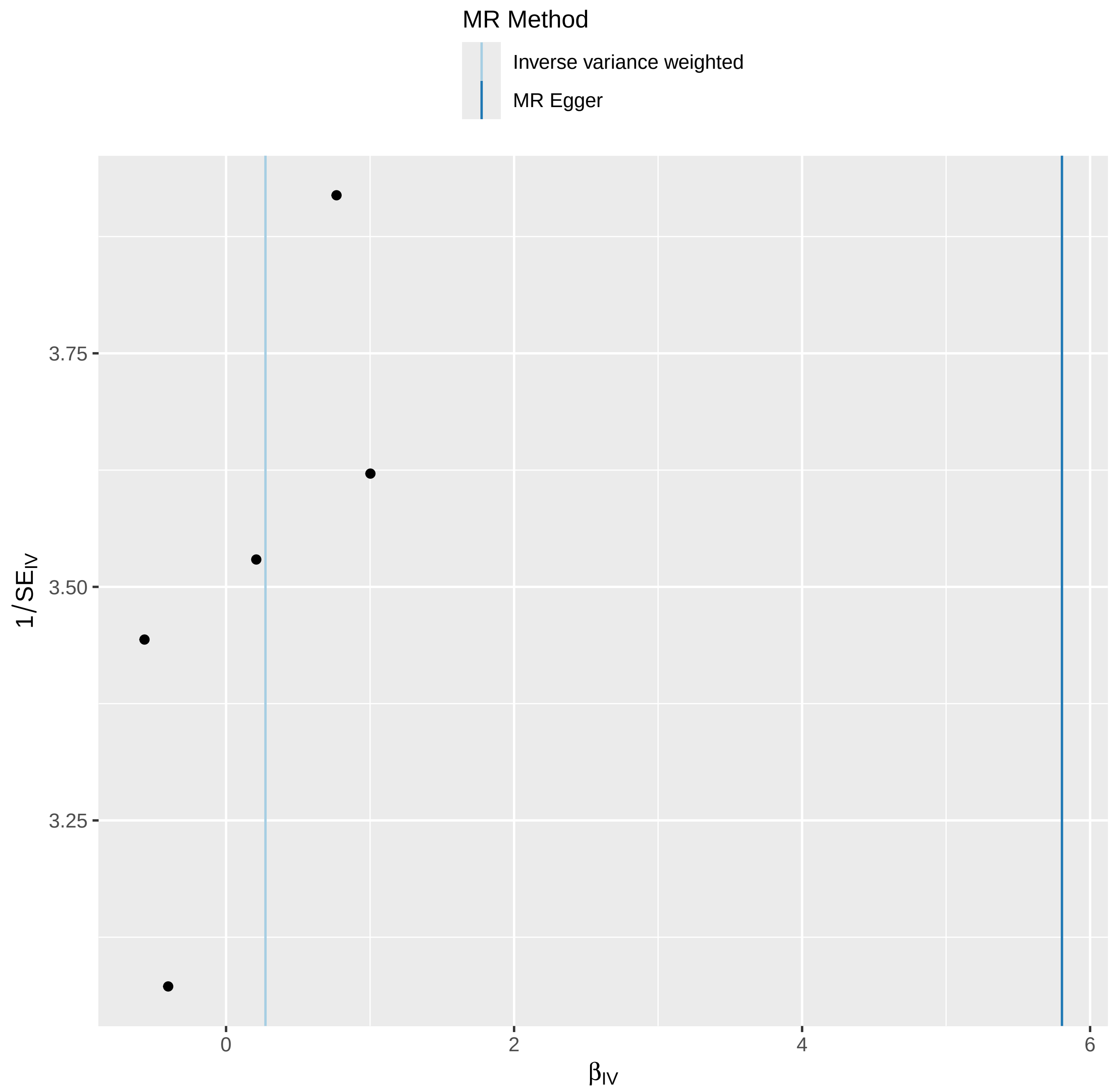
MR Method
Inverse variance weighted
MR Egger
3.75
3.50
3.25
$1/SE_{IV}$
0
2
4
6
β_{IV}

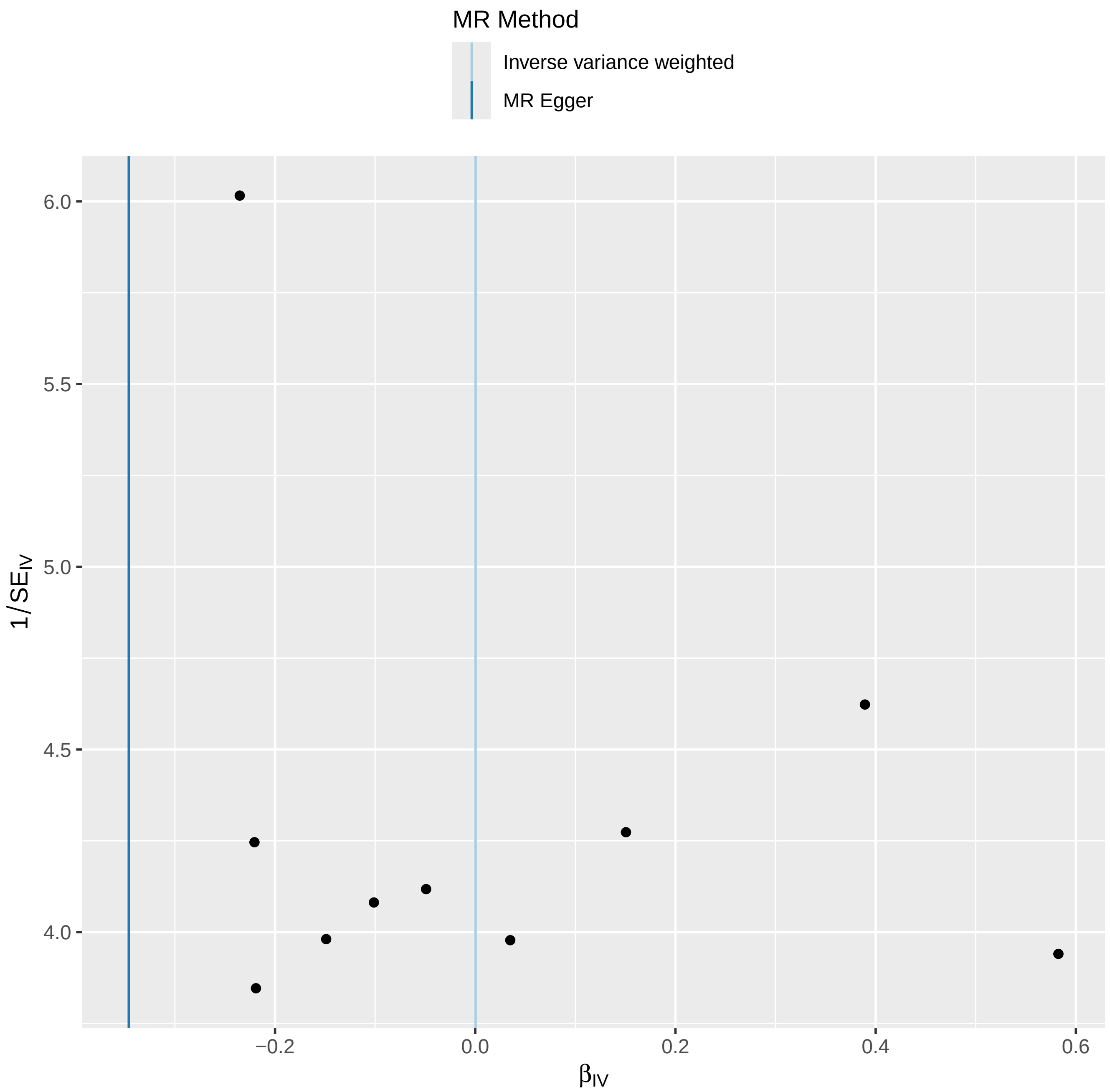

MR Method
Inverse variance weighted
MR Egger
6.0
5.5
5.0
4.5
4.0
1/SE$_{IV}$
−0.2
0.0
0.2
0.4
0.6
β$_{IV}$

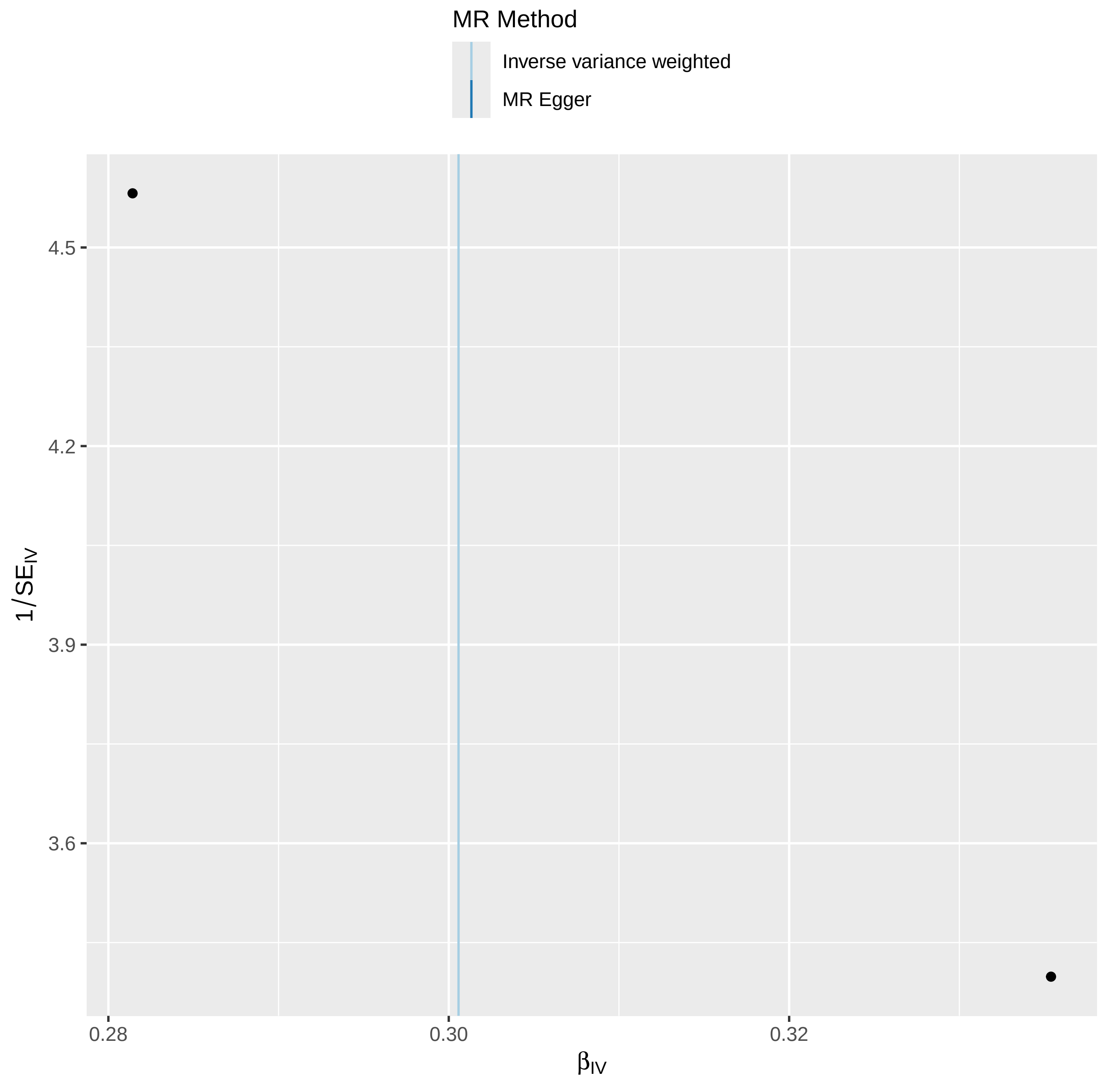
MR Method
Inverse variance weighted
MR Egger
4.5
4.2
3.9
3.6
$1/SE_{IV}$
0.28
0.30
0.32
β_{IV}

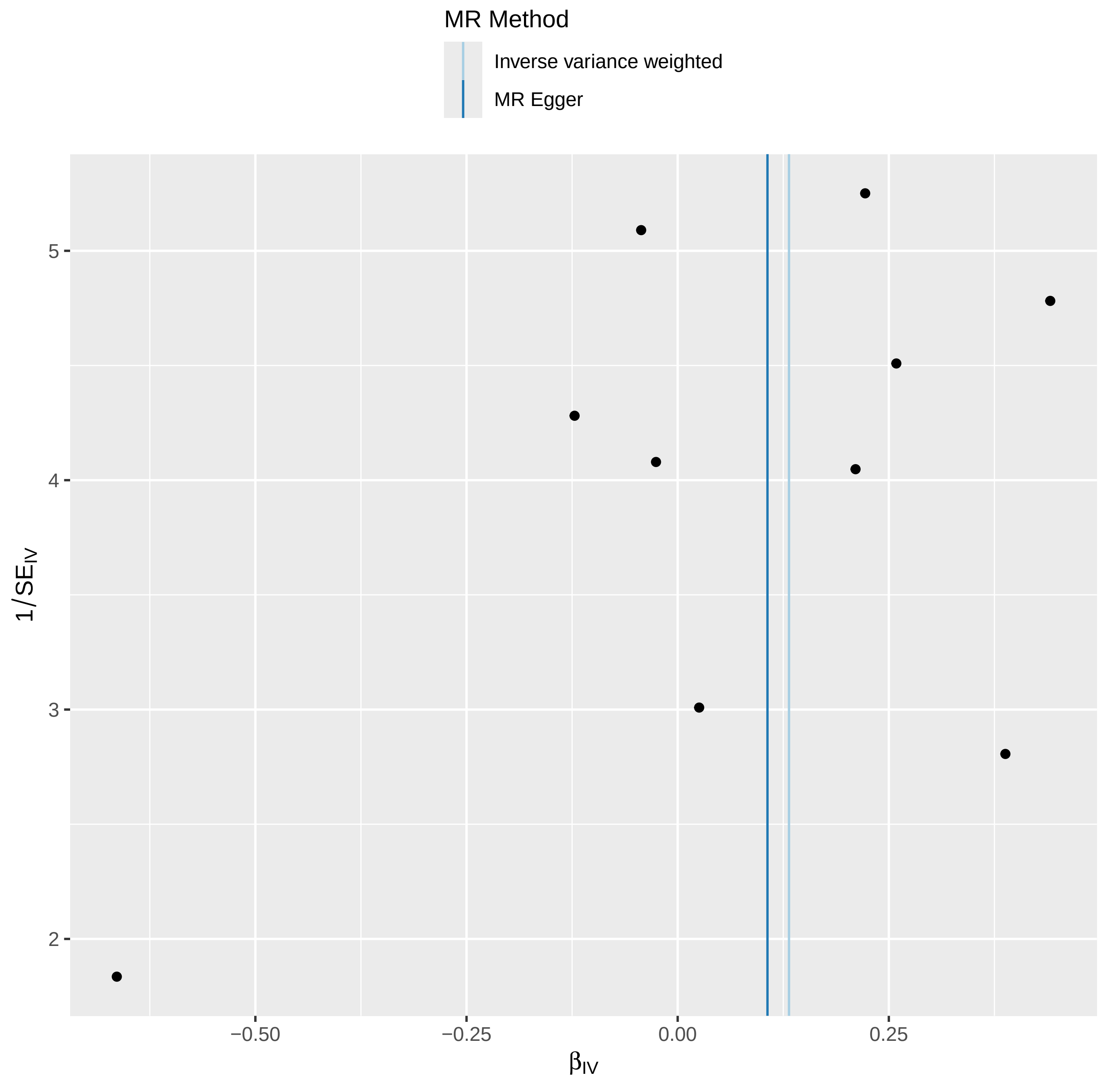

MR Method
Inverse variance weighted
MR Egger
5
4
3
2
−0.50
−0.25
0.00
0.25
$1/SE_{IV}$
β_{IV}

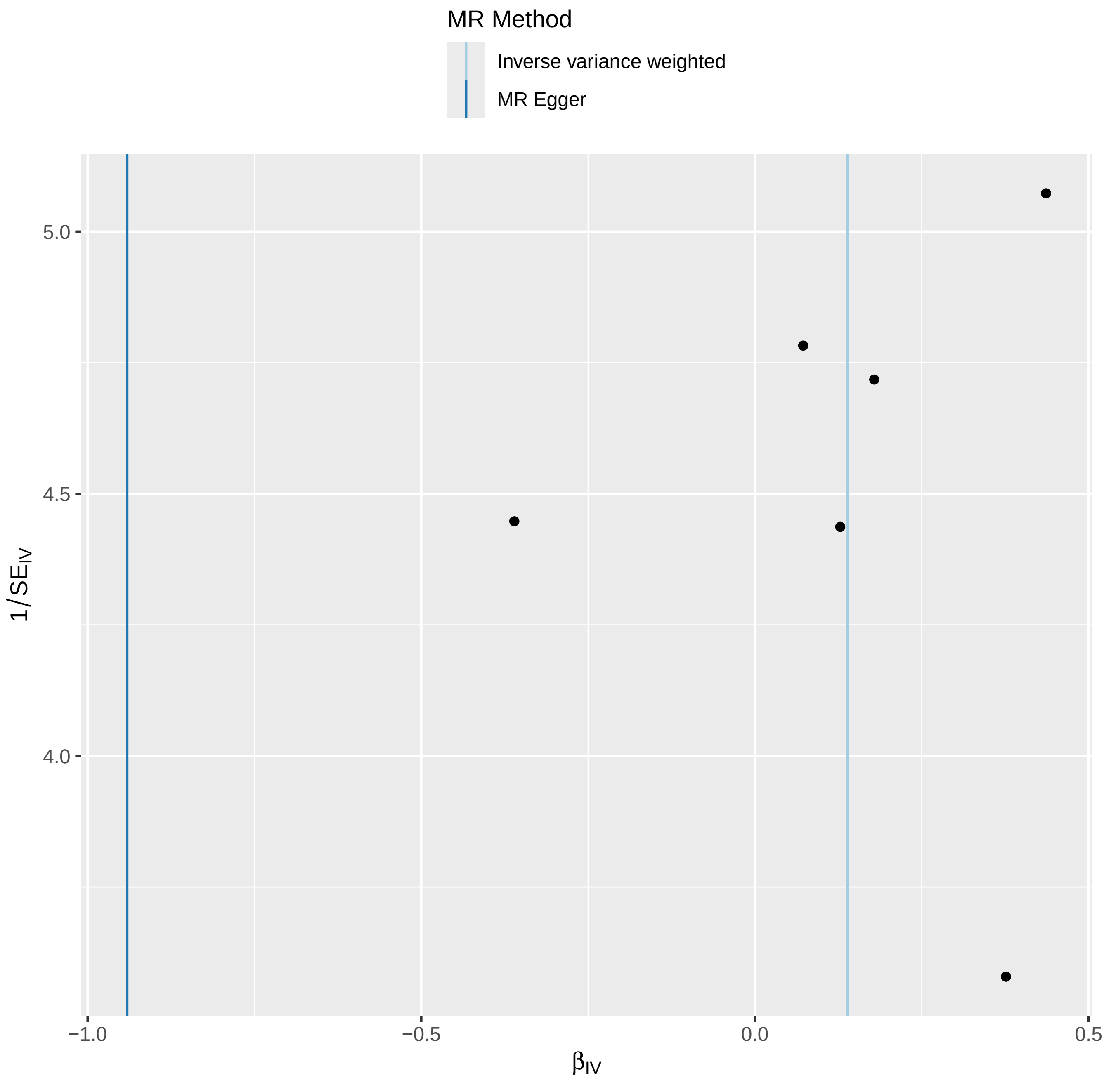
MR Method
Inverse variance weighted
MR Egger
5.0
4.5
4.0
1/SE_IV
−1.0
−0.5
0.0
0.5
β_IV

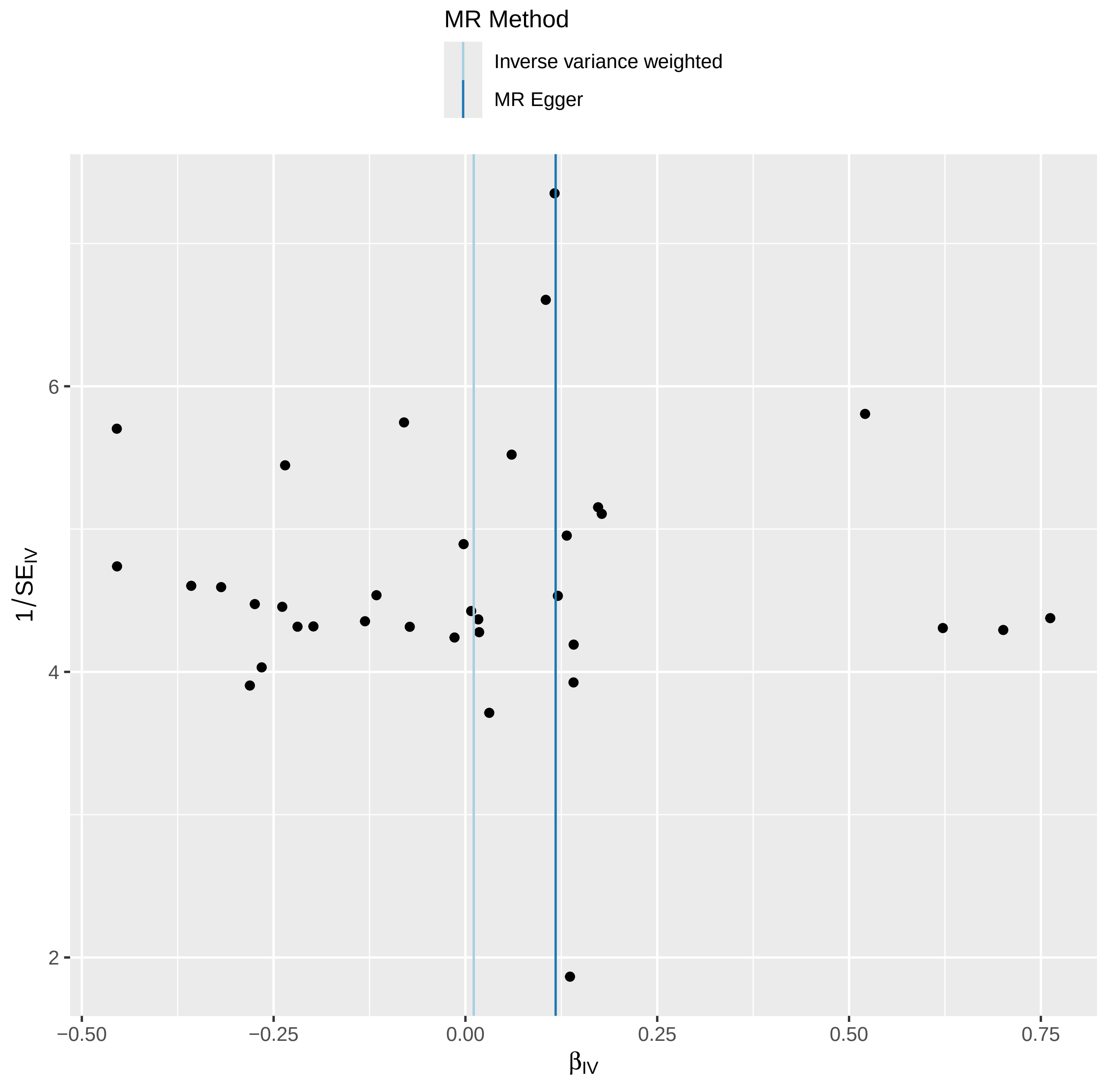

MR Method
Inverse variance weighted
MR Egger
1/SE$_{IV}$
6
4
2
−0.50
−0.25
0.00
0.25
0.50
0.75
β$_{IV}$

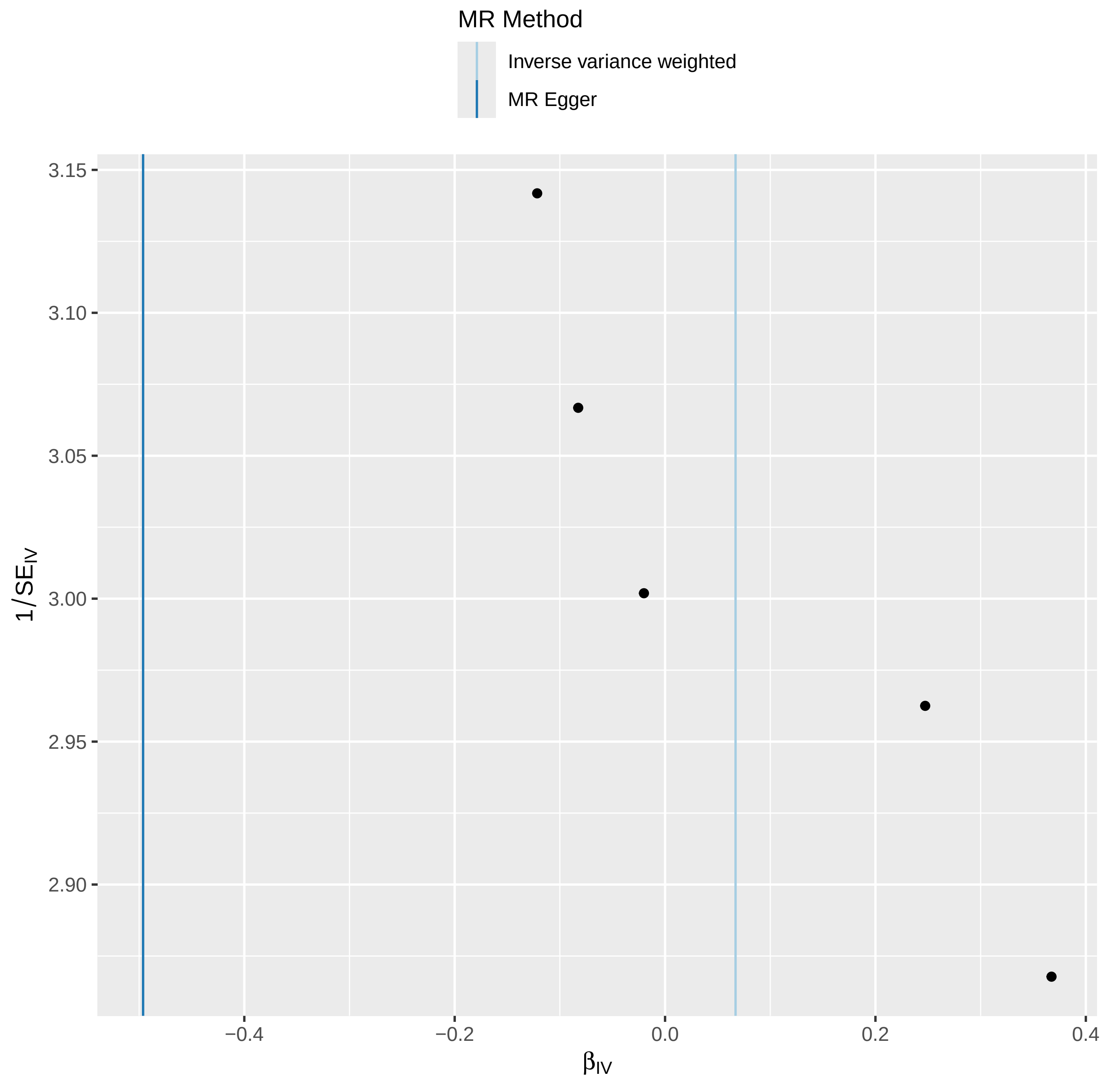
MR Method
Inverse variance weighted
MR Egger
3.15
3.10
3.05
3.00
2.95
2.90
1/SE_IV
−0.4
−0.2
0.0
0.2
0.4
β_IV

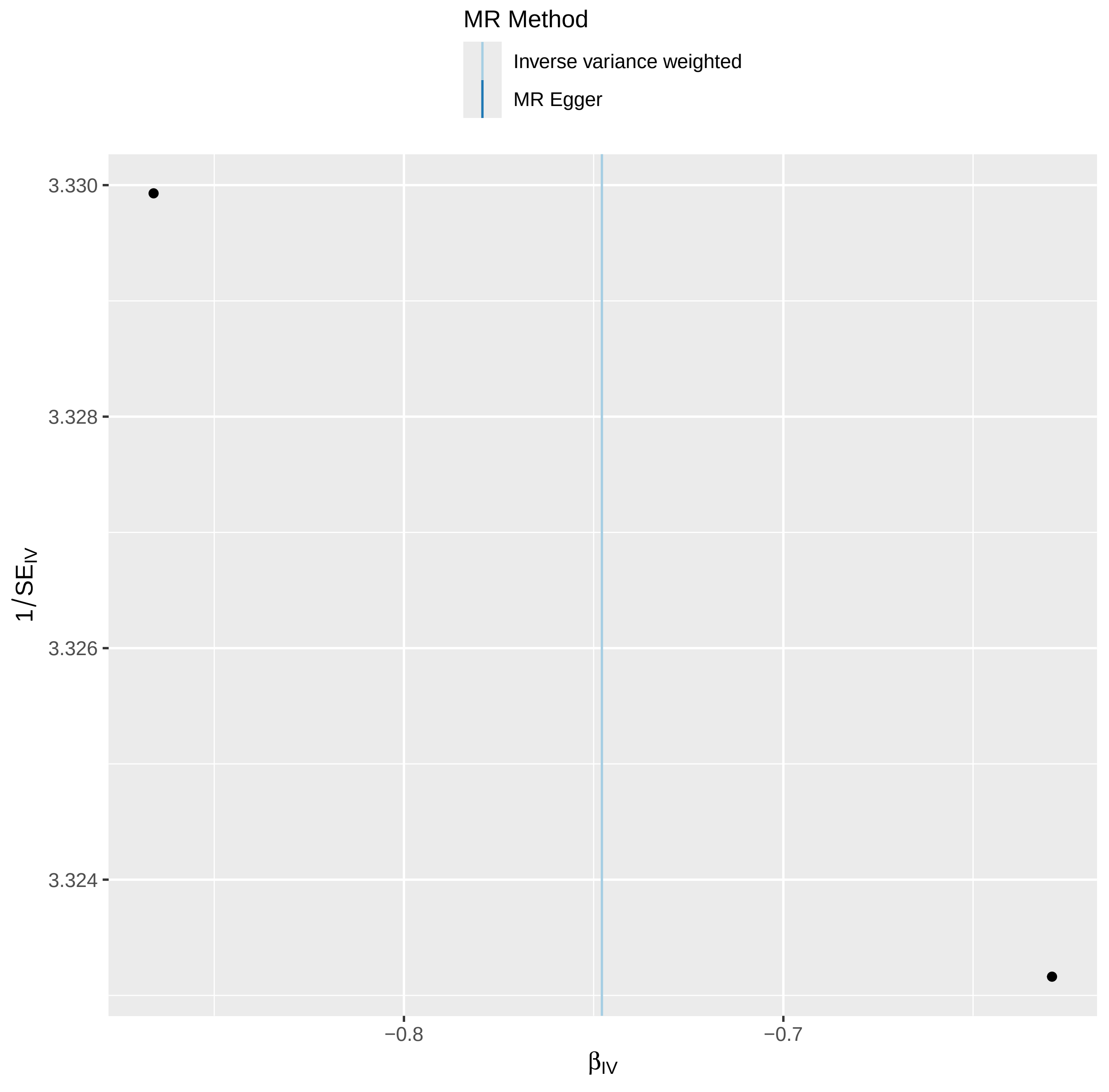

MR Method
Inverse variance weighted
MR Egger
3.330
3.328
3.326
3.324
1/SE_IV
−0.8
−0.7
β_IV

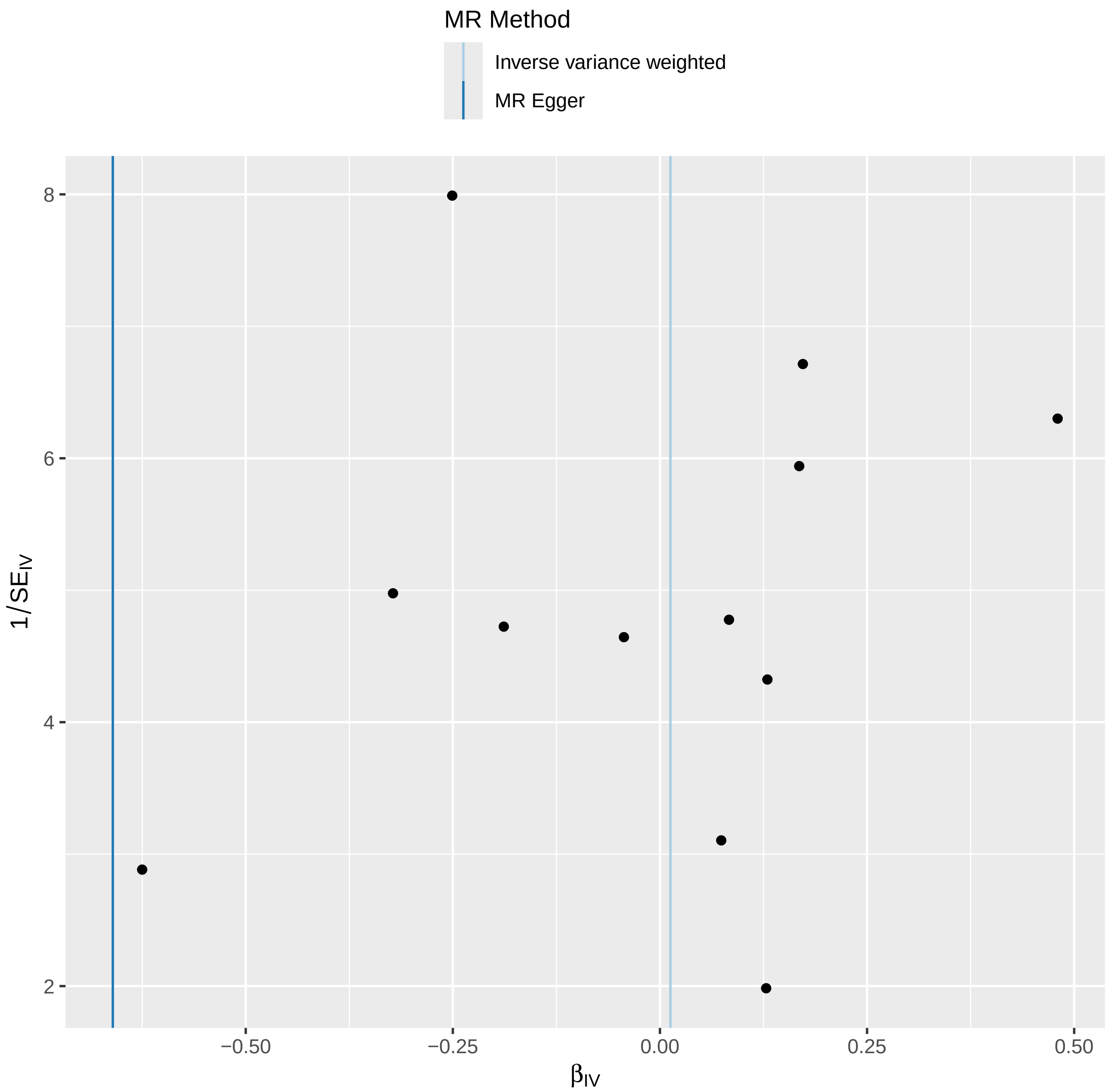

MR Method
Inverse variance weighted
MR Egger
8
6
4
2
$1/SE_{IV}$
−0.50
−0.25
0.00
0.25
0.50
β_{IV}

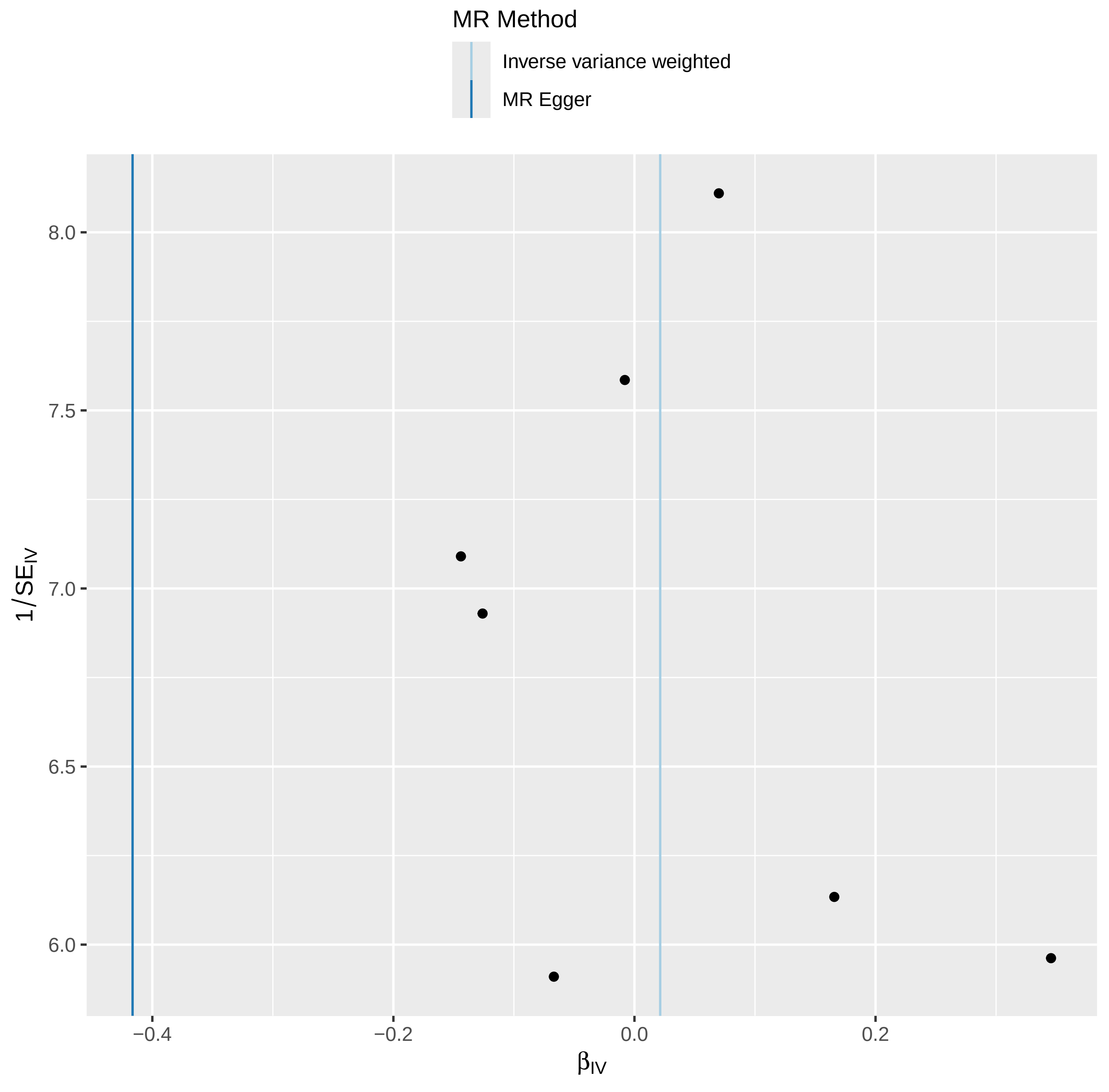
MR Method
Inverse variance weighted
MR Egger
8.0
7.5
7.0
6.5
6.0
$1/SE_{IV}$
−0.4
−0.2
0.0
0.2
β_{IV}

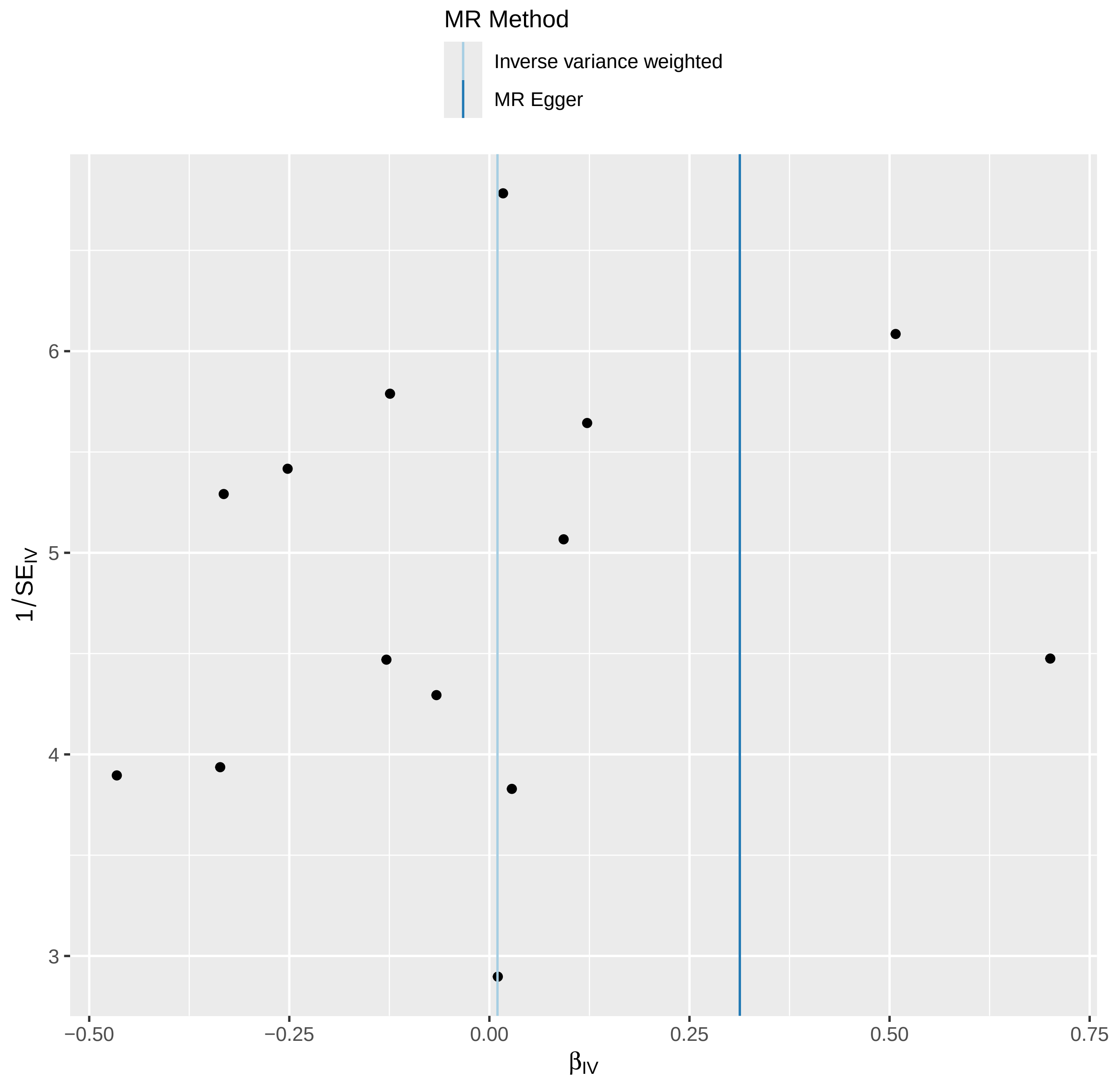

MR Method
Inverse variance weighted
MR Egger
6
5
4
3
$1/SE_{IV}$
−0.50
−0.25
0.00
0.25
0.50
0.75
β_{IV}

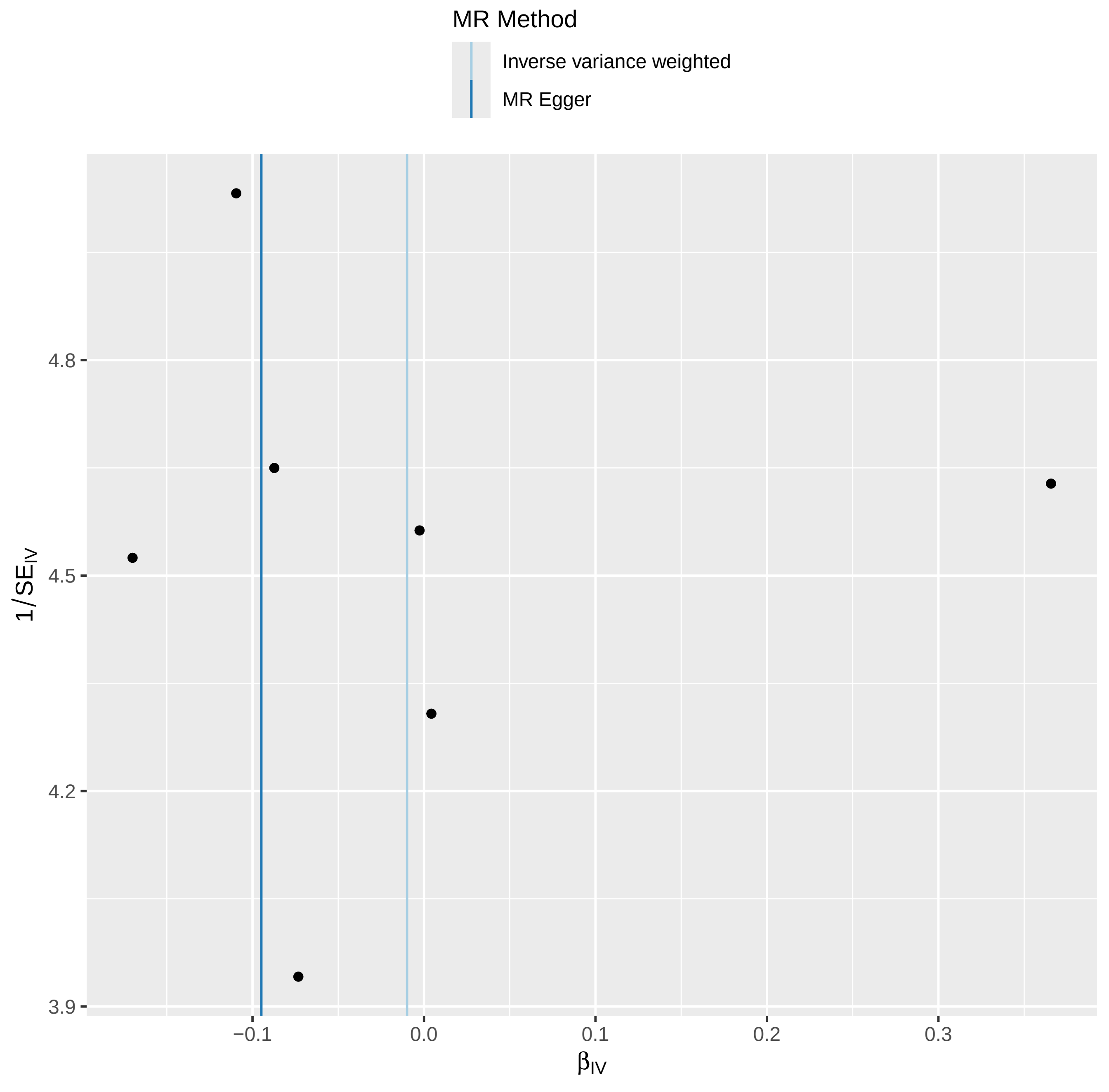

MR Method
Inverse variance weighted
MR Egger
4.8
4.5
4.2
3.9
$1/SE_{IV}$
−0.1
0.0
0.1
0.2
0.3
β_{IV}

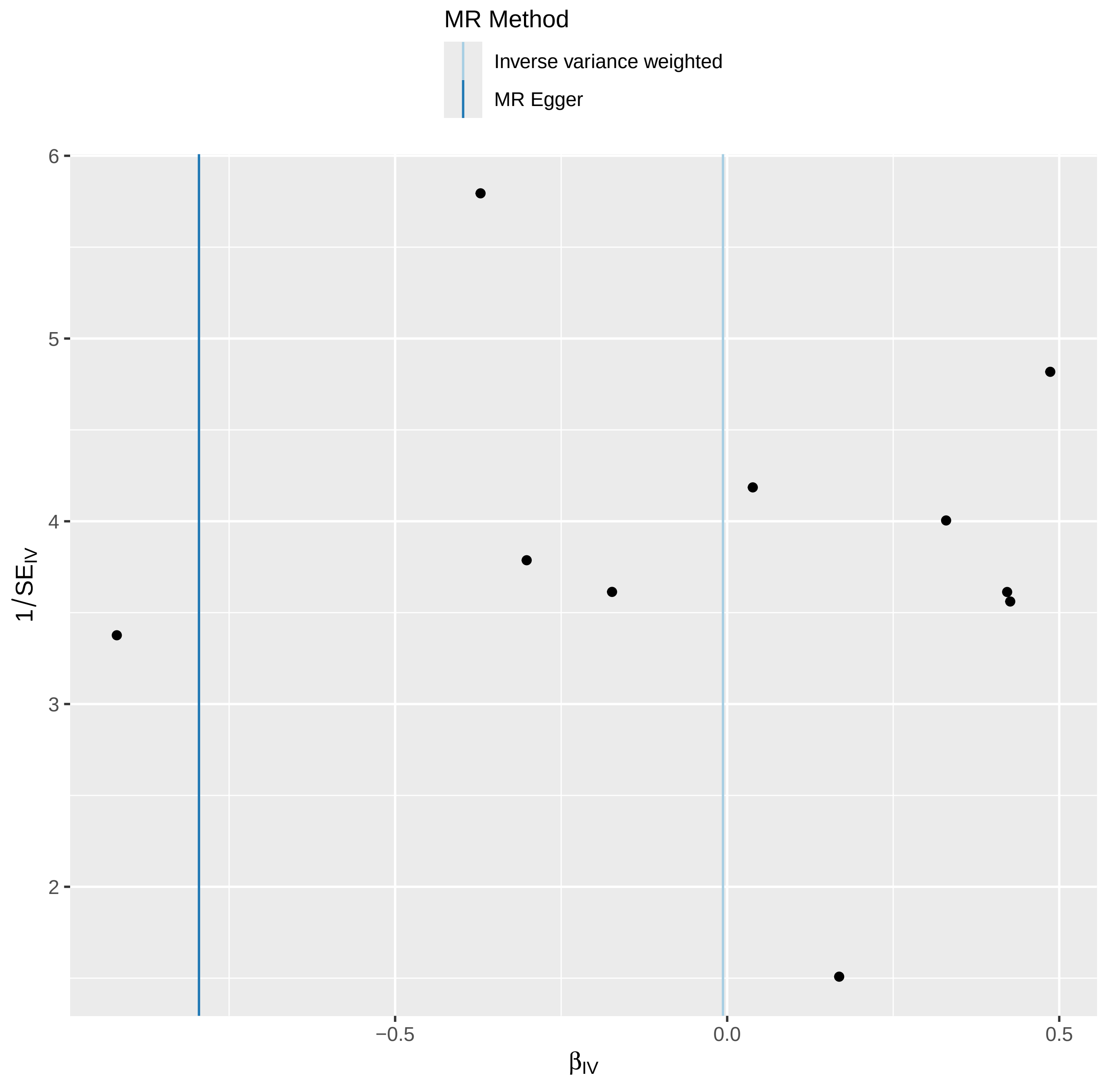
MR Method
Inverse variance weighted
MR Egger
6
5
4
3
2
1/SE_IV
−0.5
0.0
0.5
β_IV

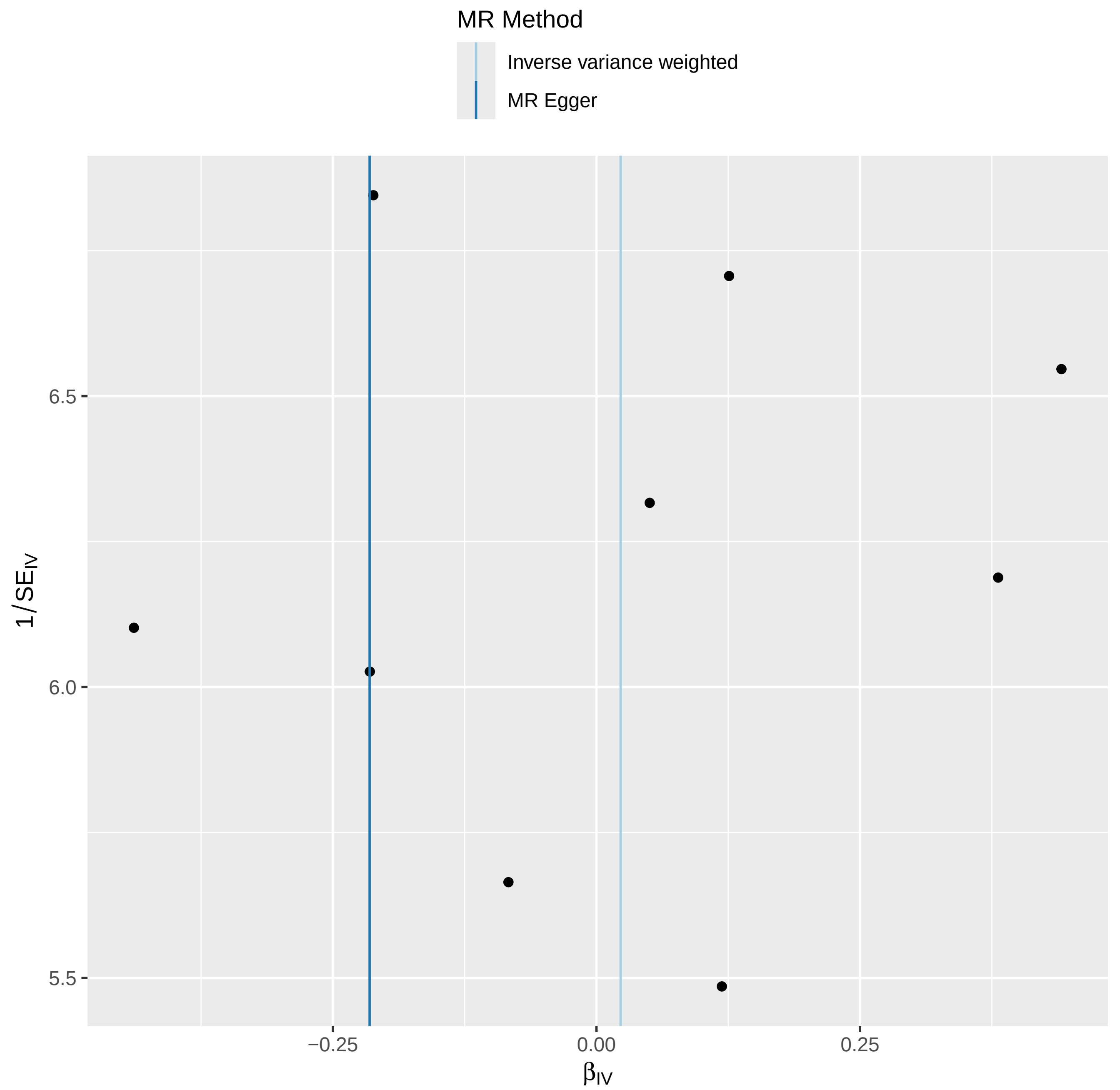

MR Method
Inverse variance weighted
MR Egger
6.5
6.0
5.5
−0.25
0.00
0.25
$1/SE_{IV}$
β_{IV}

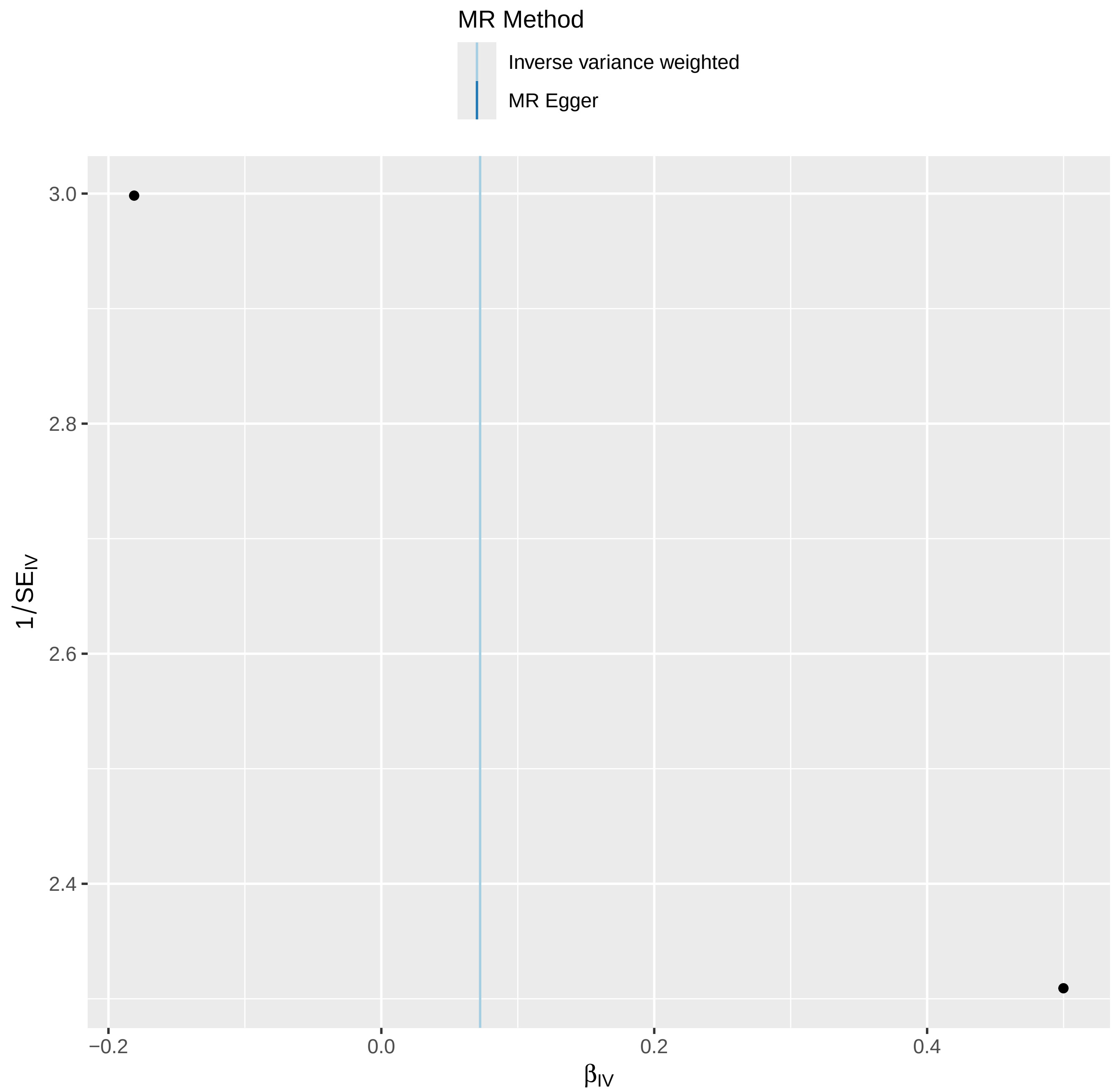
MR Method
Inverse variance weighted
MR Egger
3.0
2.8
2.6
2.4
1/SE_IV
−0.2
0.0
0.2
0.4
β_IV

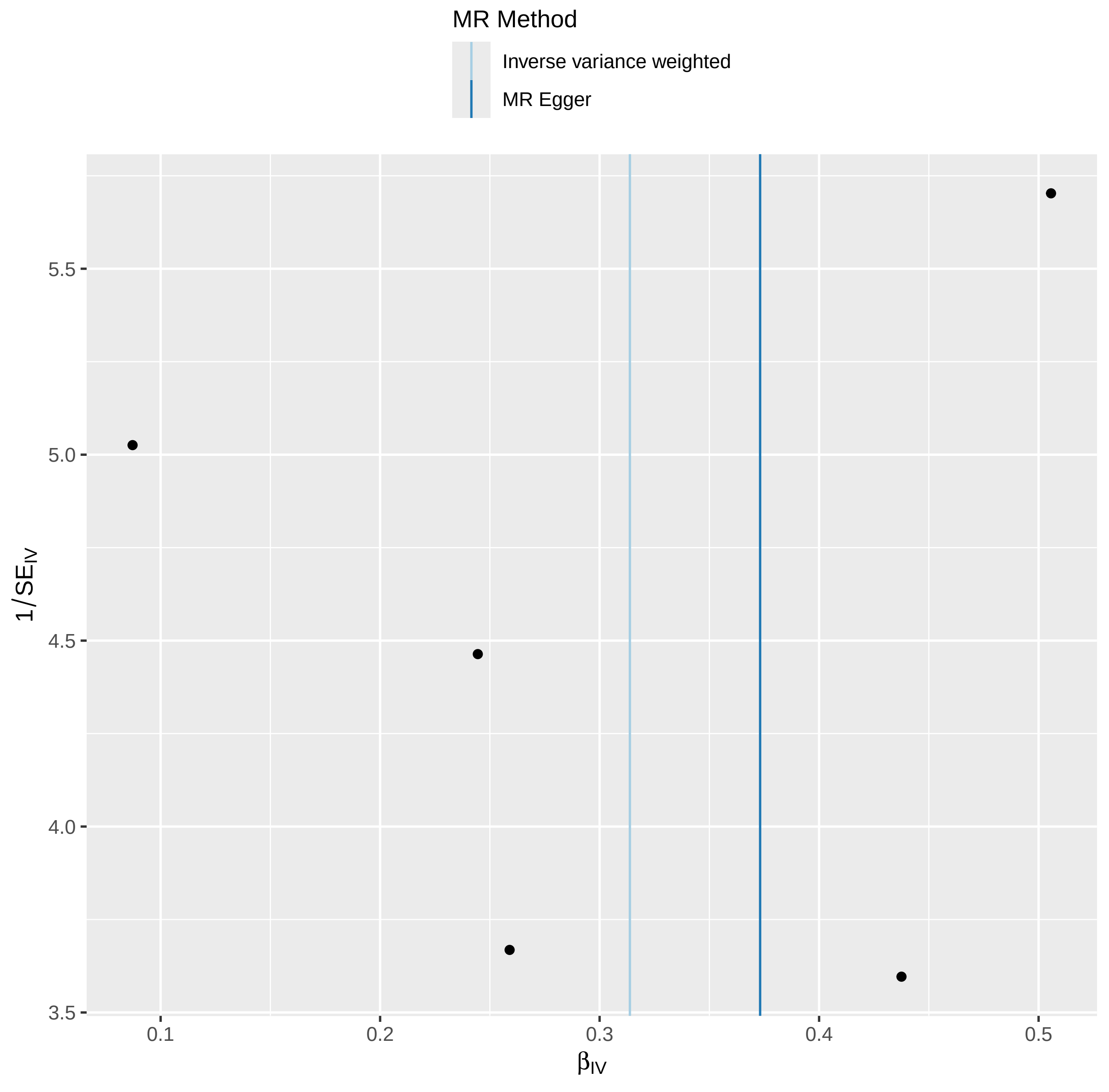

MR Method
Inverse variance weighted
MR Egger
5.5
5.0
4.5
4.0
3.5
1/SE_IV
0.1
0.2
0.3
0.4
0.5
β_IV

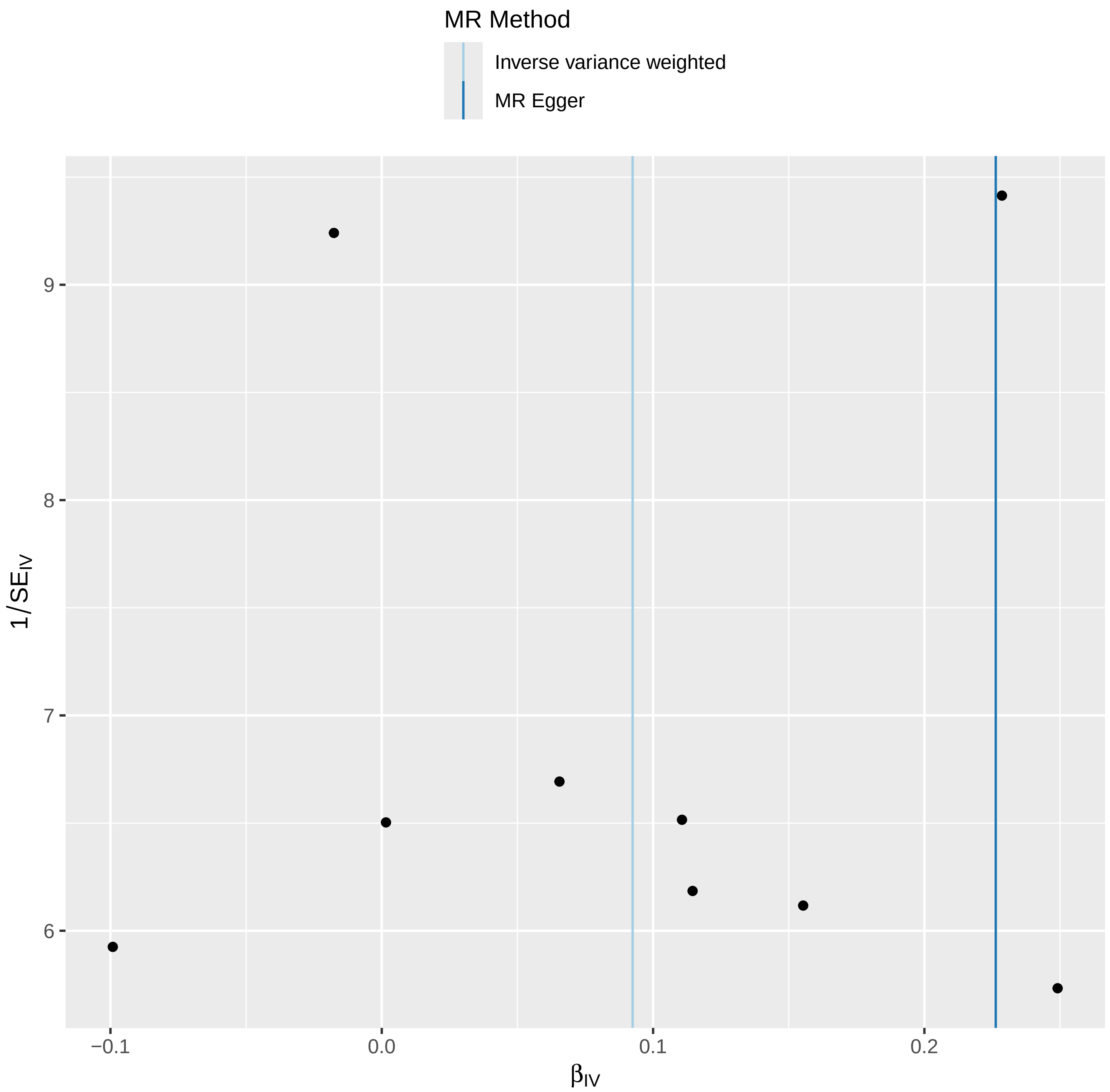
MR Method
Inverse variance weighted
MR Egger
1/SE_IV
β_IV
9
8
7
6
−0.1
0.0
0.1
0.2

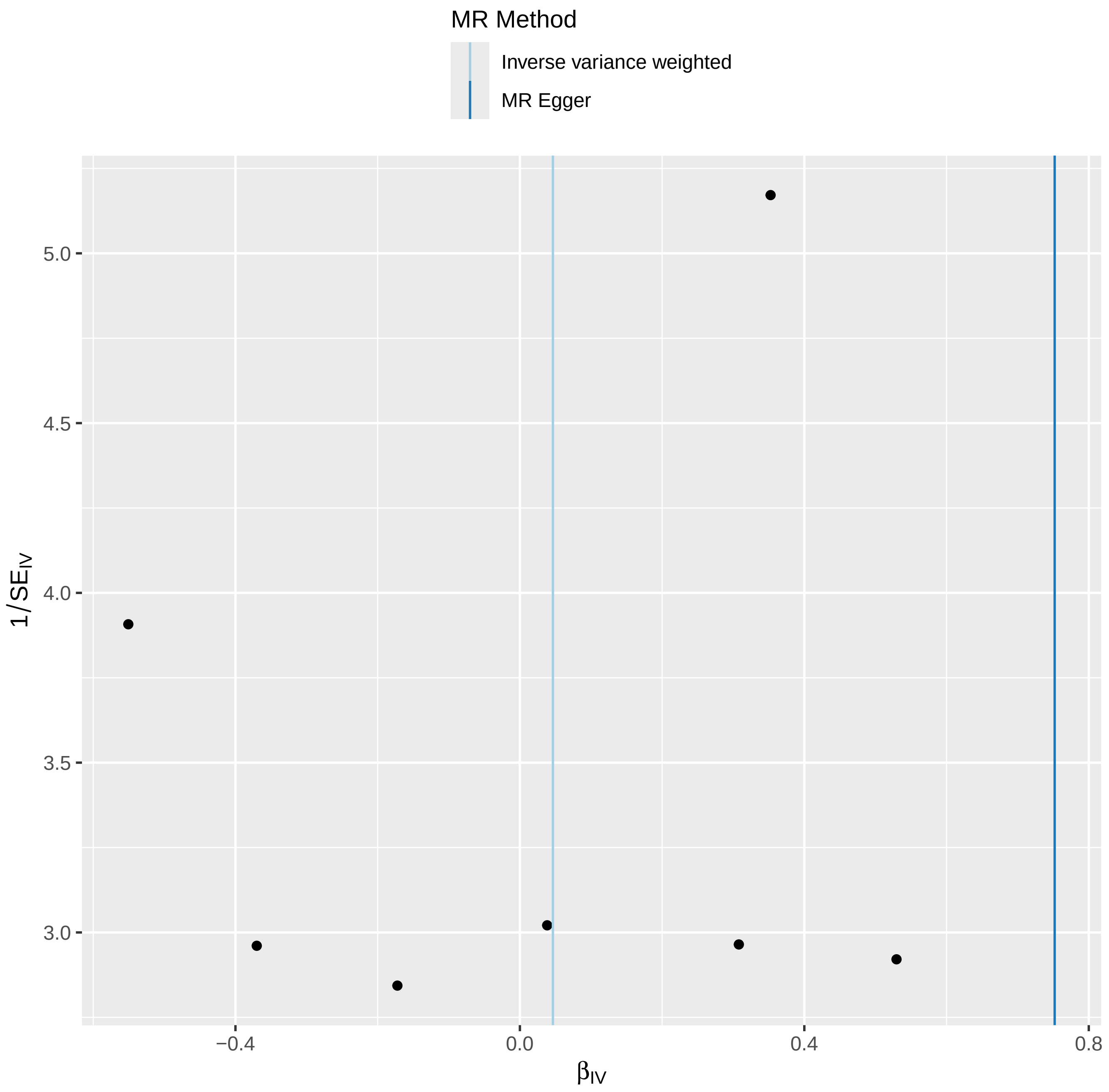

MR Method
Inverse variance weighted
MR Egger
5.0
4.5
4.0
3.5
3.0
$1/SE_{IV}$
−0.4
0.0
0.4
0.8
β_{IV}

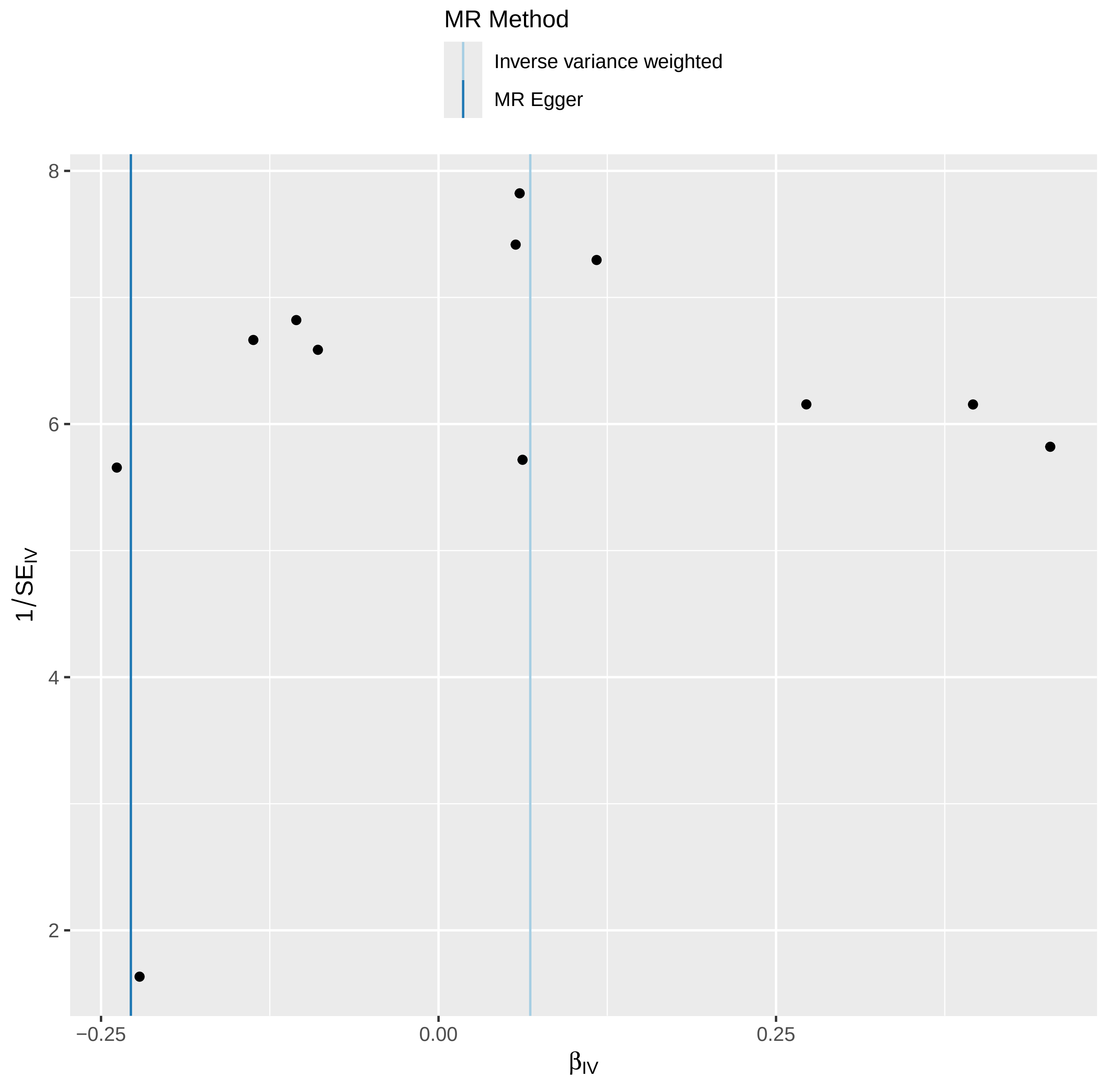

MR Method
Inverse variance weighted
MR Egger
8
6
4
2
1/SE_IV
−0.25
0.00
0.25
β_IV

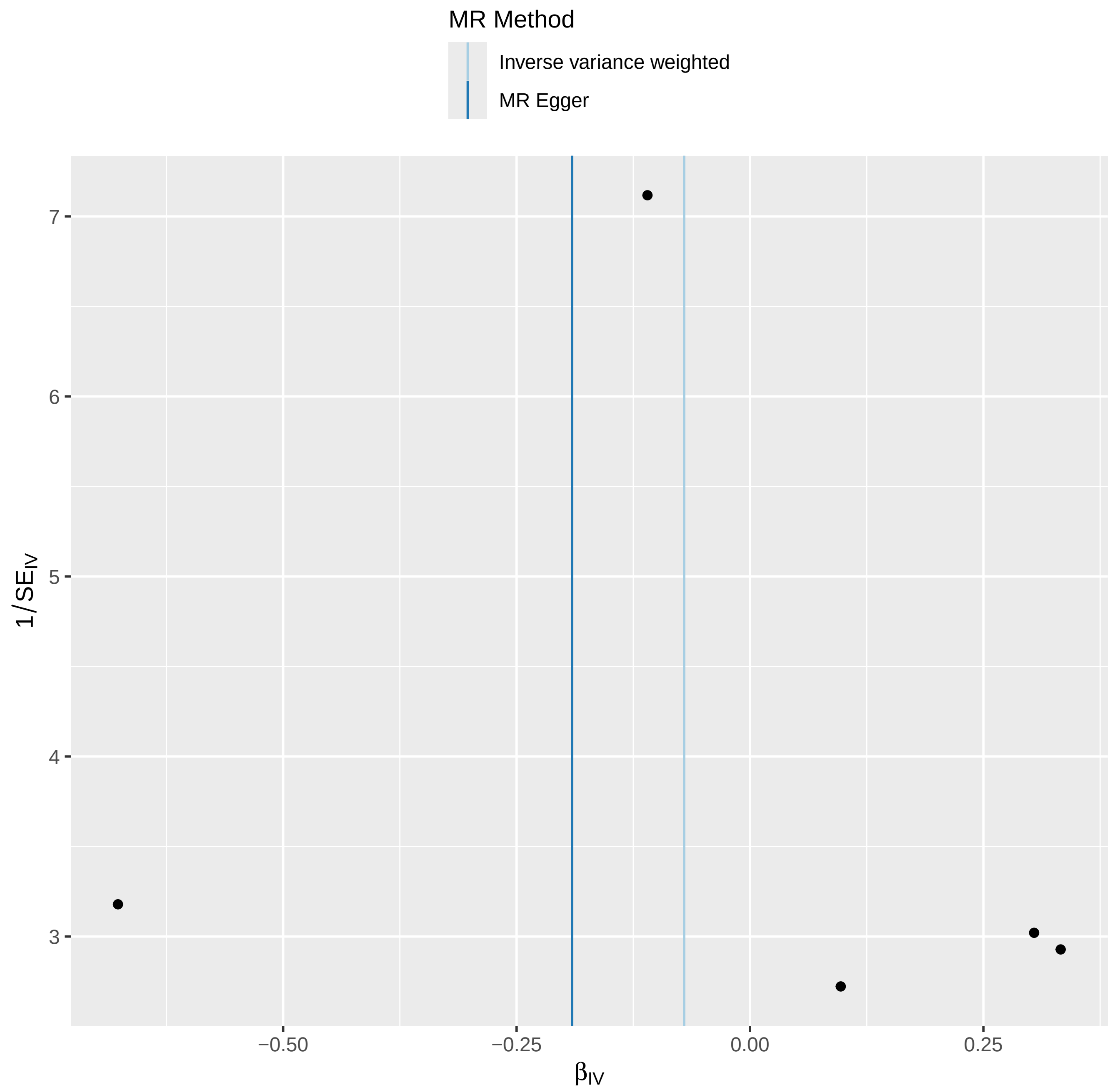
MR Method
Inverse variance weighted
MR Egger
7
6
5
4
3
1/SE$_{IV}$
−0.50
−0.25
0.00
0.25
β$_{IV}$

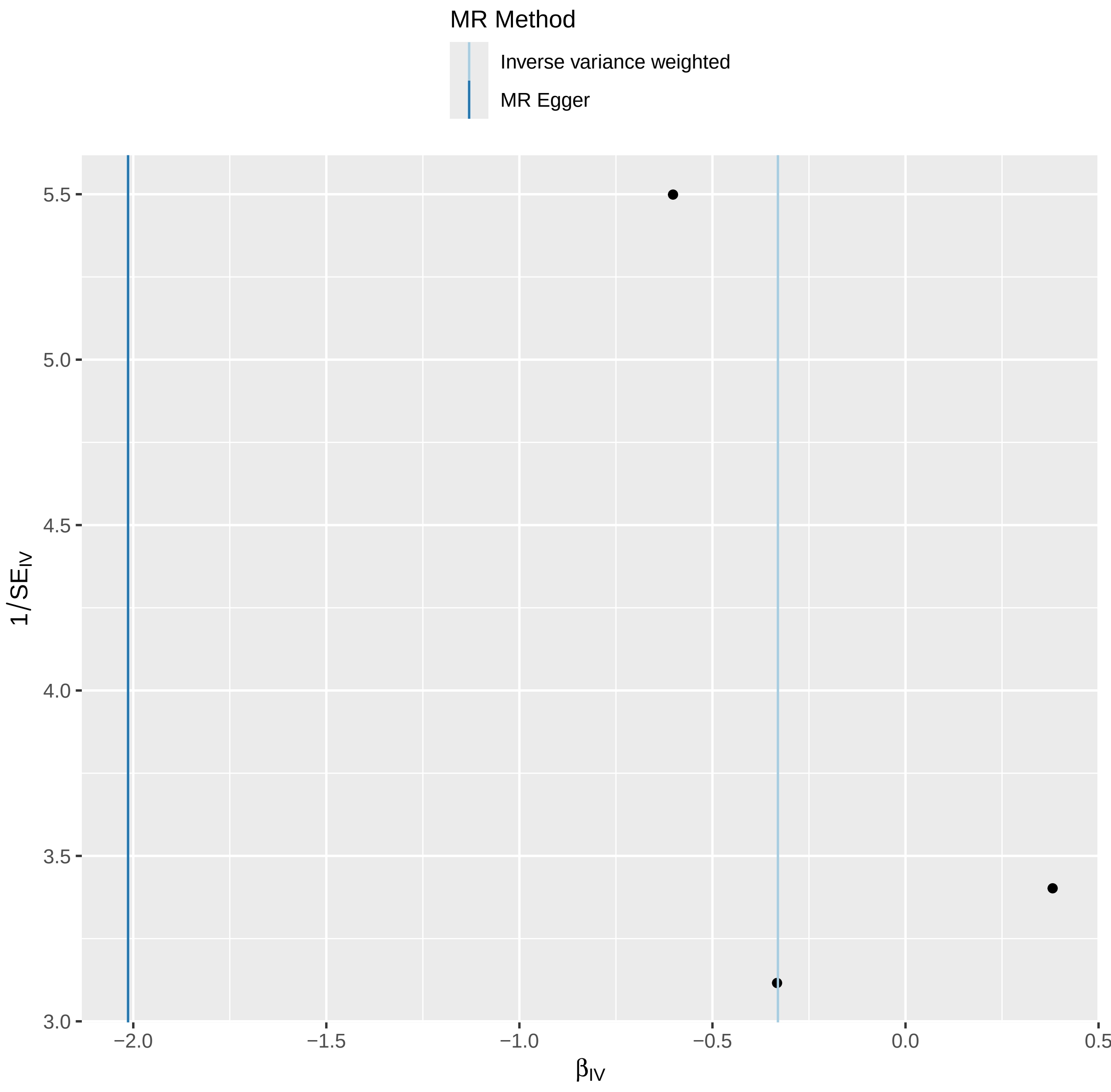

MR Method
Inverse variance weighted
MR Egger
5.5
5.0
4.5
4.0
3.5
3.0
$1/SE_{IV}$
−2.0
−1.5
−1.0
−0.5
0.0
0.5
β_{IV}

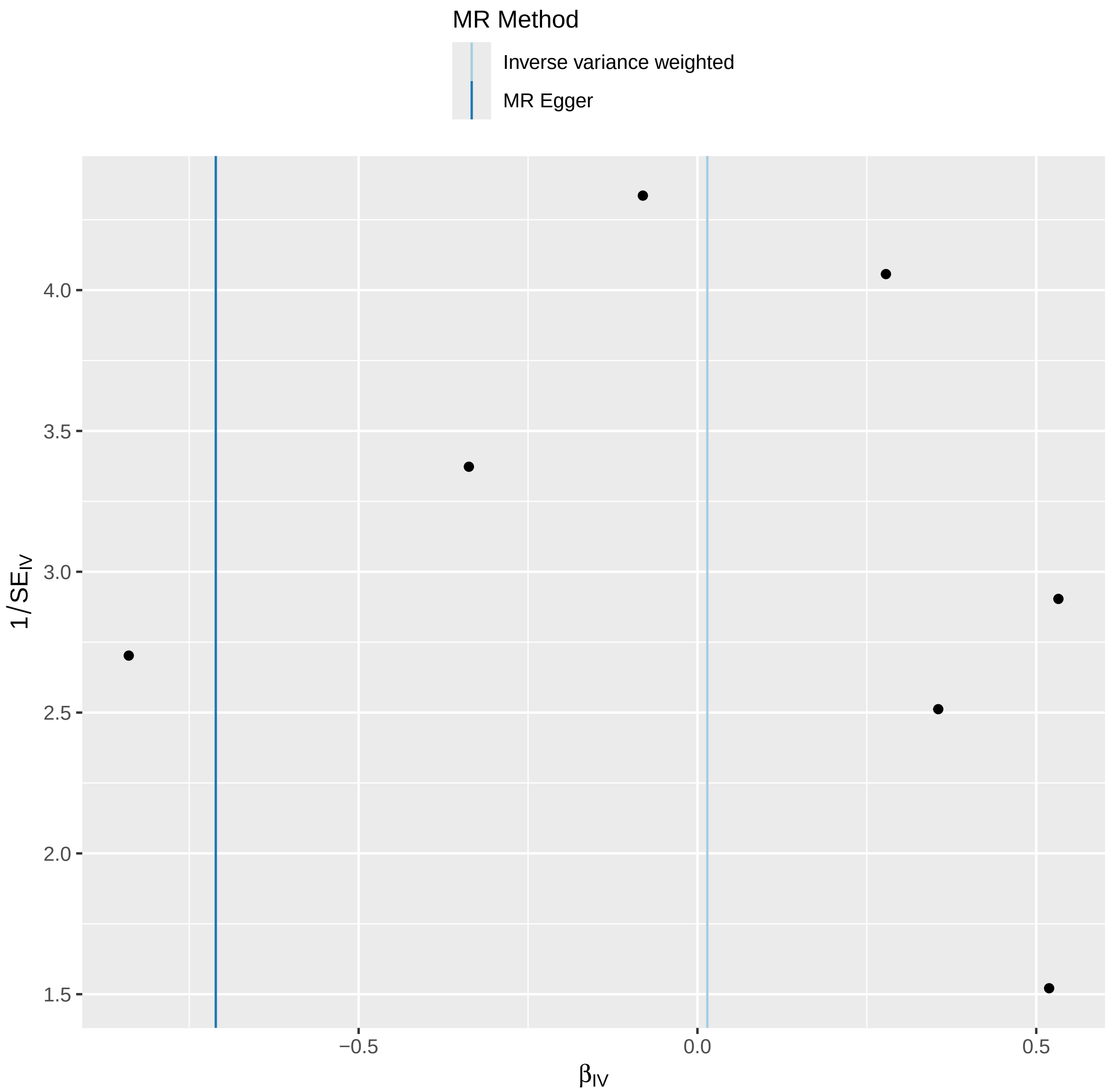

MR Method
Inverse variance weighted
MR Egger
4.0
3.5
3.0
2.5
2.0
1.5
$1/SE_{IV}$
−0.5
0.0
0.5
β_{IV}

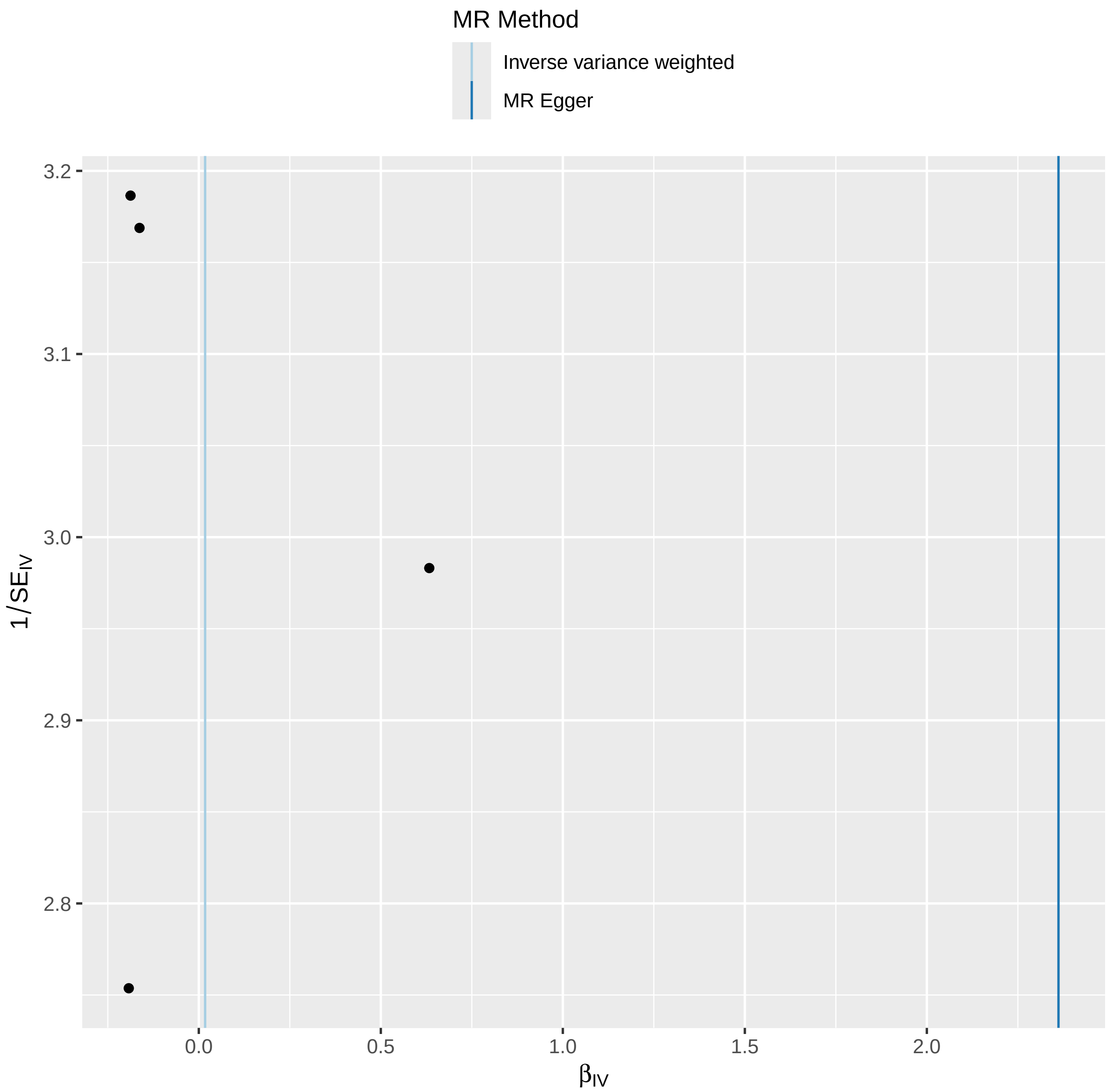

MR Method
Inverse variance weighted
MR Egger
3.2
3.1
3.0
2.9
2.8
1/SE$_{IV}$
0.0
0.5
1.0
1.5
2.0
β$_{IV}$

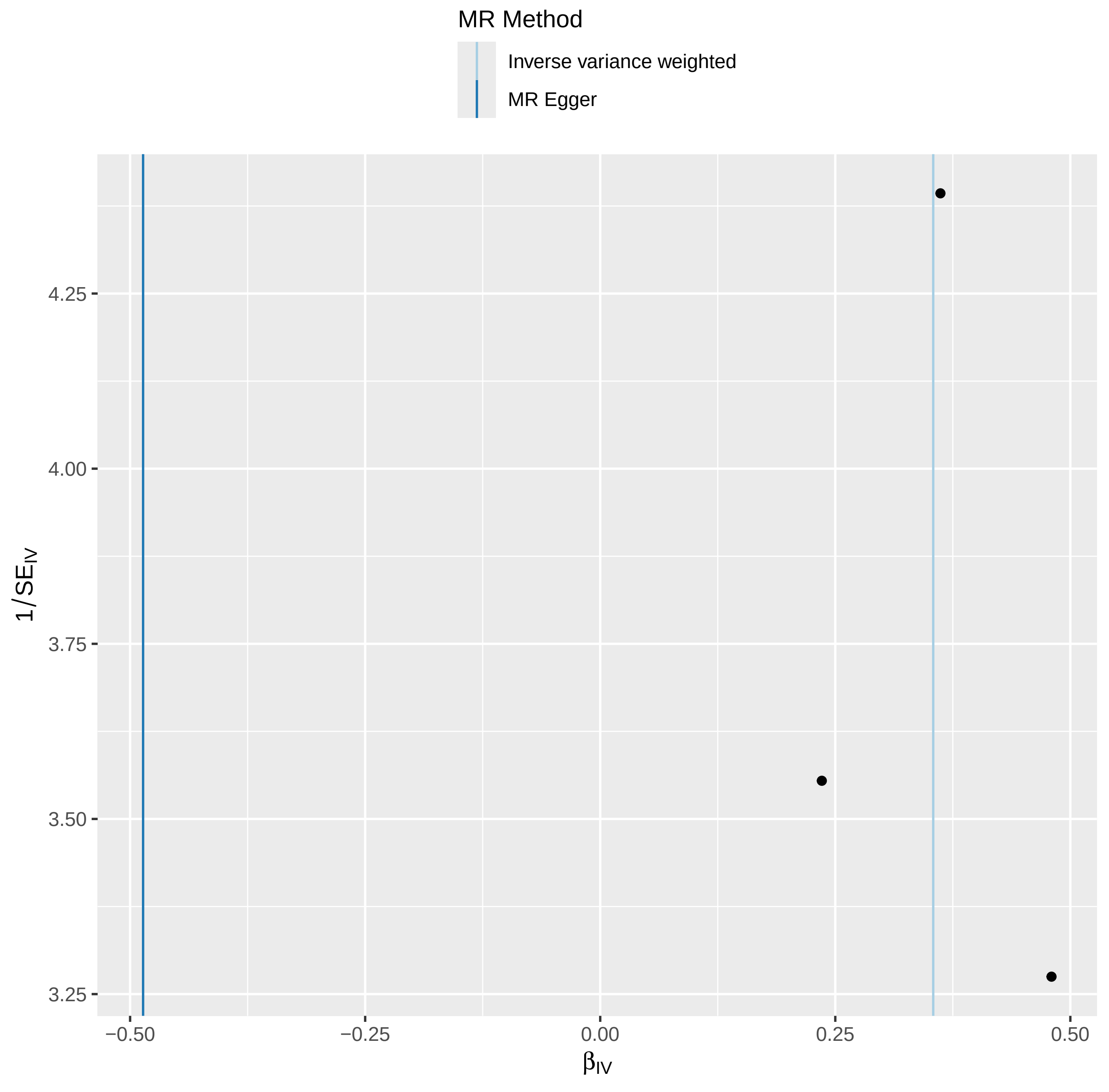
MR Method
Inverse variance weighted
MR Egger
4.25
4.00
3.75
3.50
3.25
1/SE_IV
−0.50
−0.25
0.00
0.25
0.50
β_IV

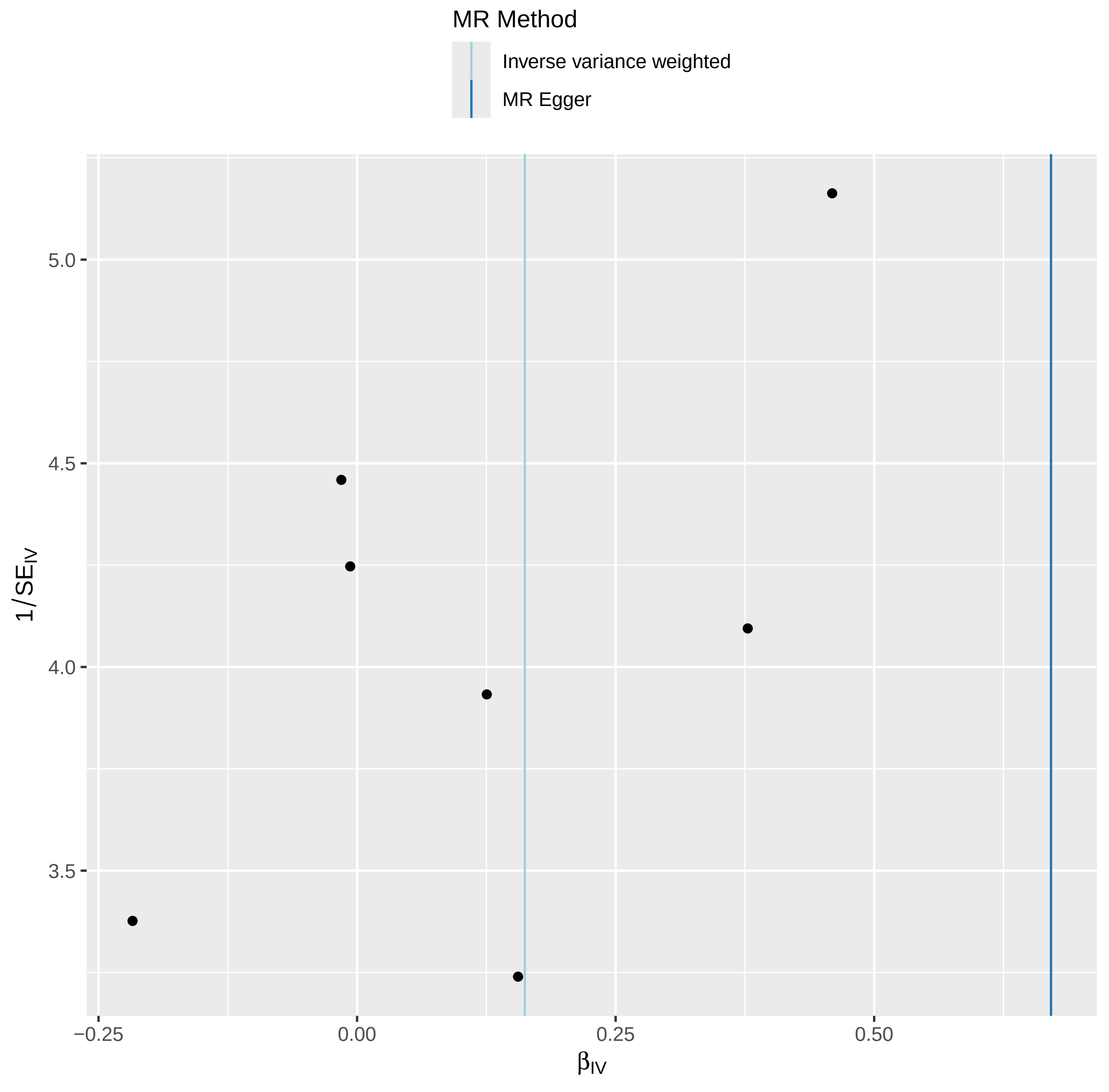
MR Method
Inverse variance weighted
MR Egger
5.0
4.5
4.0
3.5
1/SE$_{IV}$
−0.25
0.00
0.25
0.50
β$_{IV}$

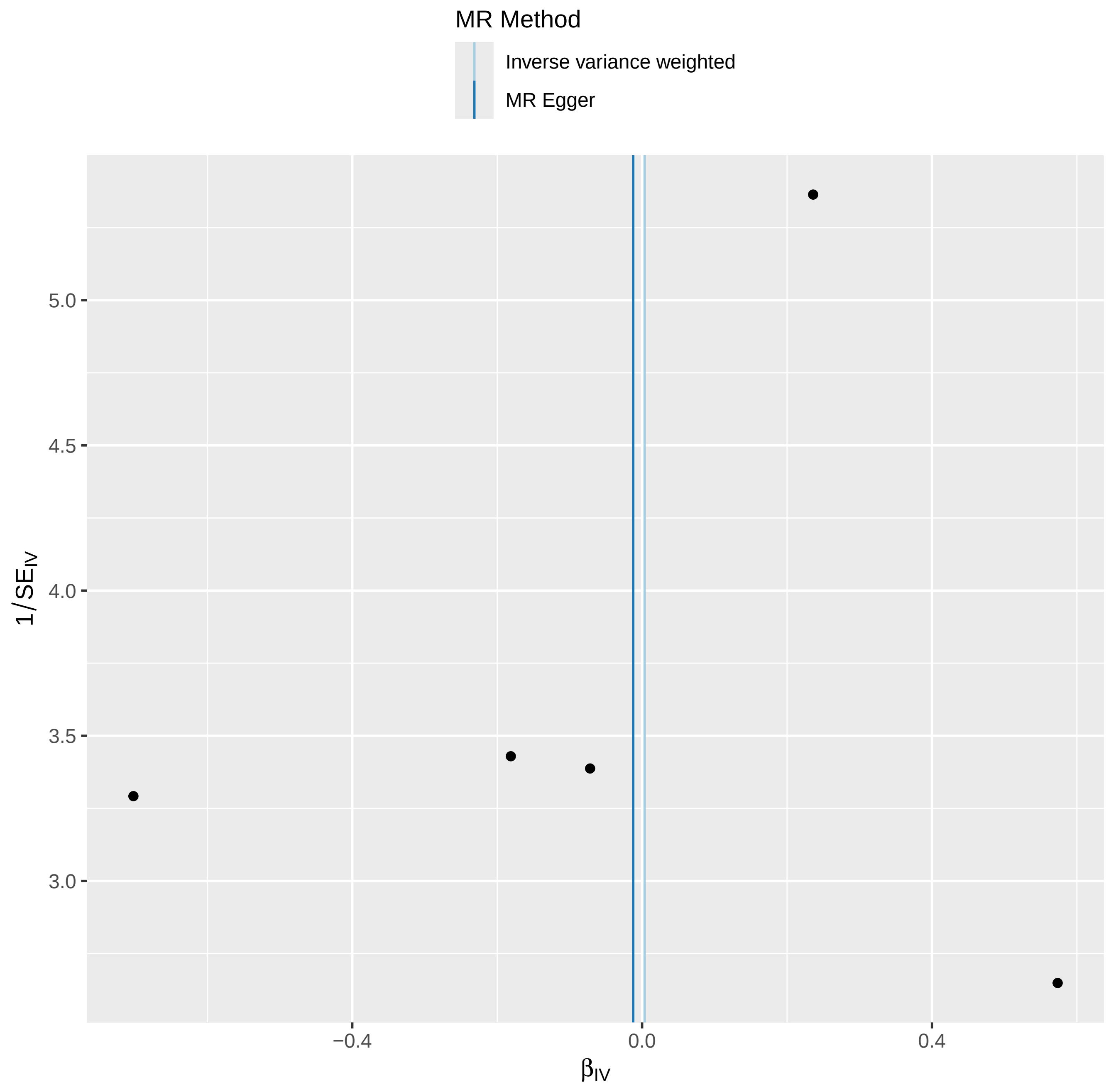
MR Method
Inverse variance weighted
MR Egger
5.0
4.5
4.0
3.5
3.0
1/SE_IV
−0.4
0.0
0.4
β_IV

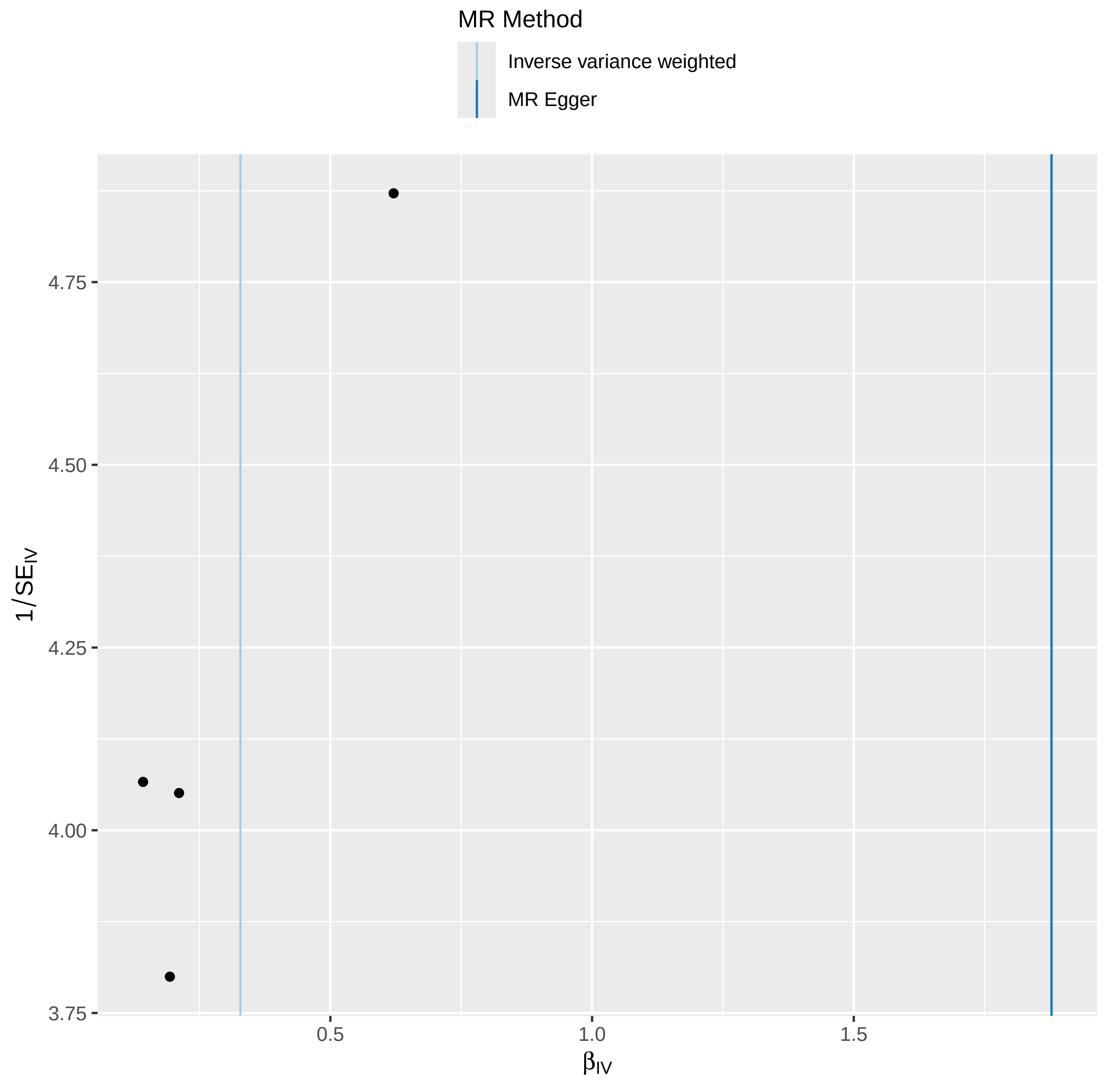

MR Method
Inverse variance weighted
MR Egger
4.75
4.50
4.25
4.00
3.75
1/SE_IV
0.5
1.0
1.5
β_IV

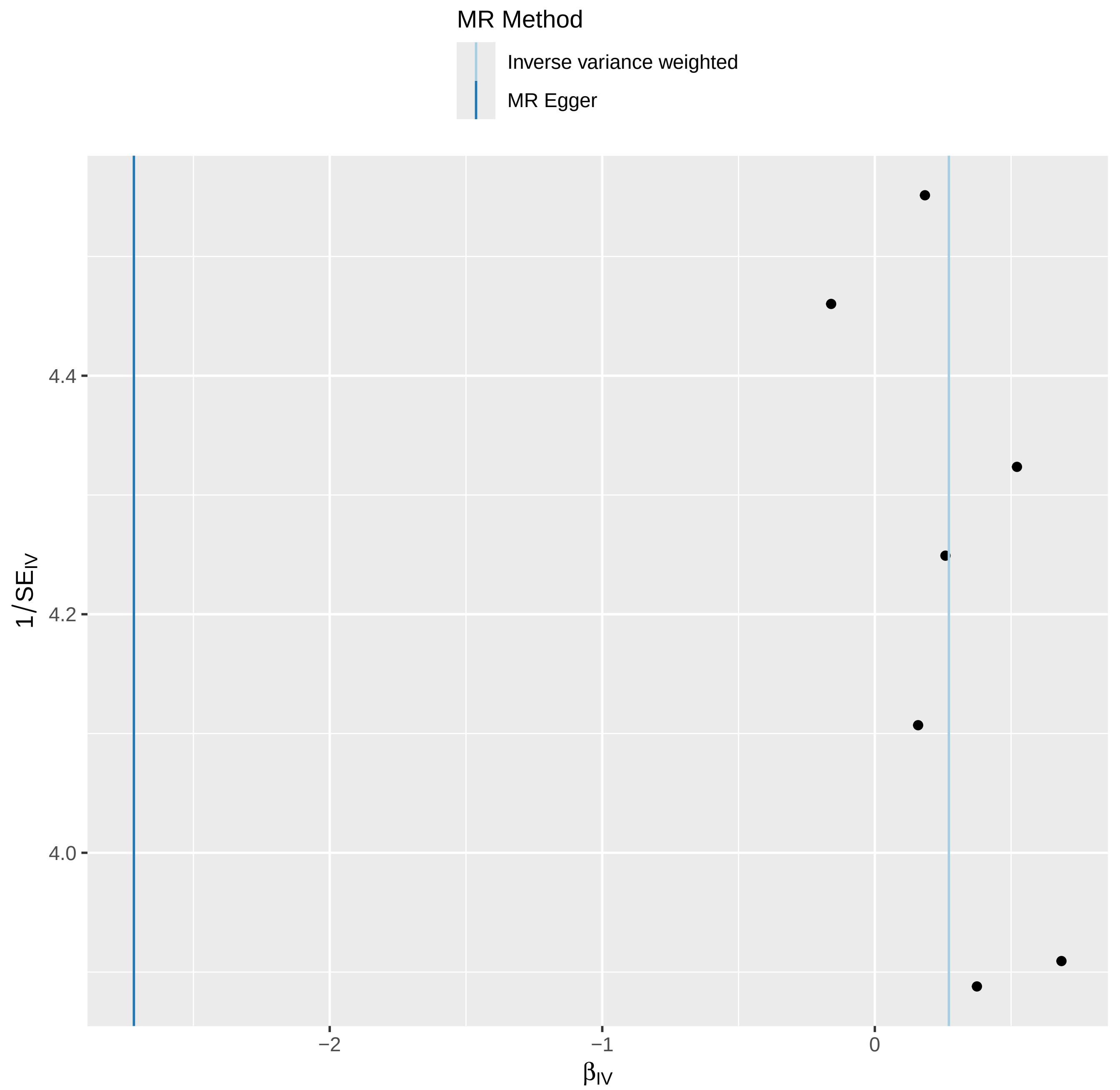

MR Method
Inverse variance weighted
MR Egger
4.4
4.2
4.0
1/SE_IV
−2
−1
0
β_IV

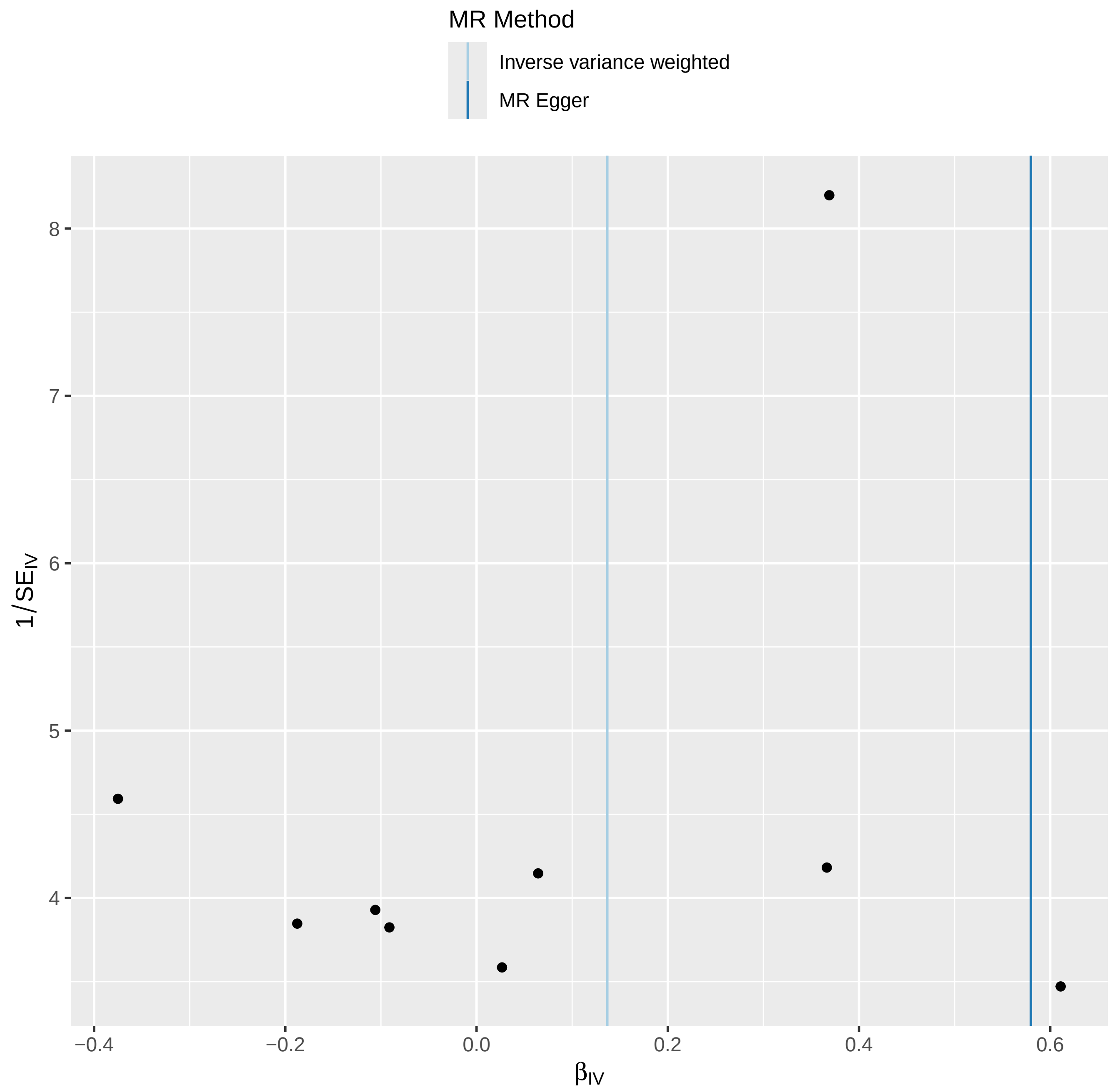

MR Method
Inverse variance weighted
MR Egger
8
7
6
5
4
$1/SE_{IV}$
−0.4
−0.2
0.0
0.2
0.4
0.6
β_{IV}

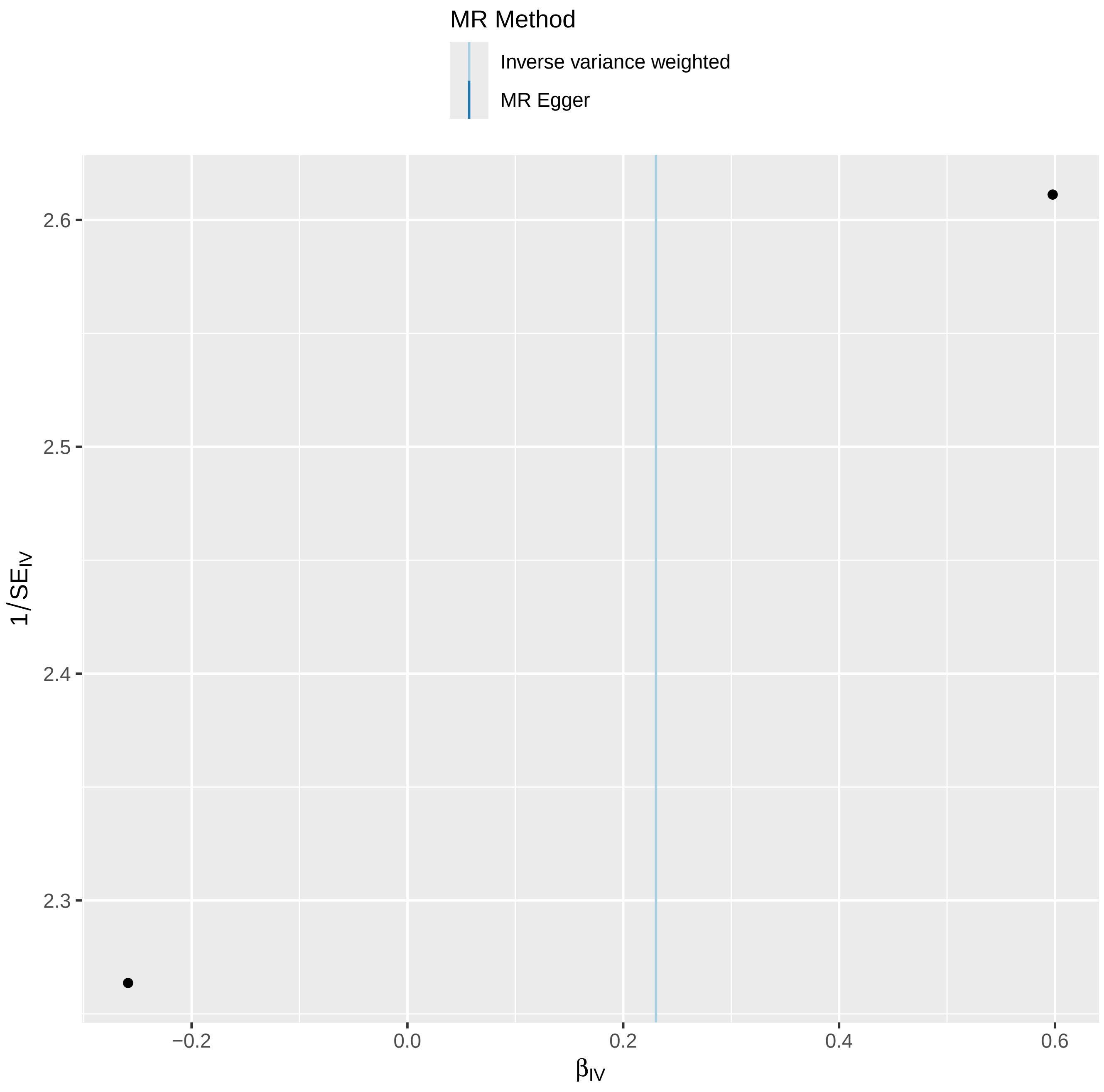
MR Method
Inverse variance weighted
MR Egger
2.6
2.5
2.4
2.3
1/SE$_{IV}$
−0.2
0.0
0.2
0.4
0.6
β$_{IV}$

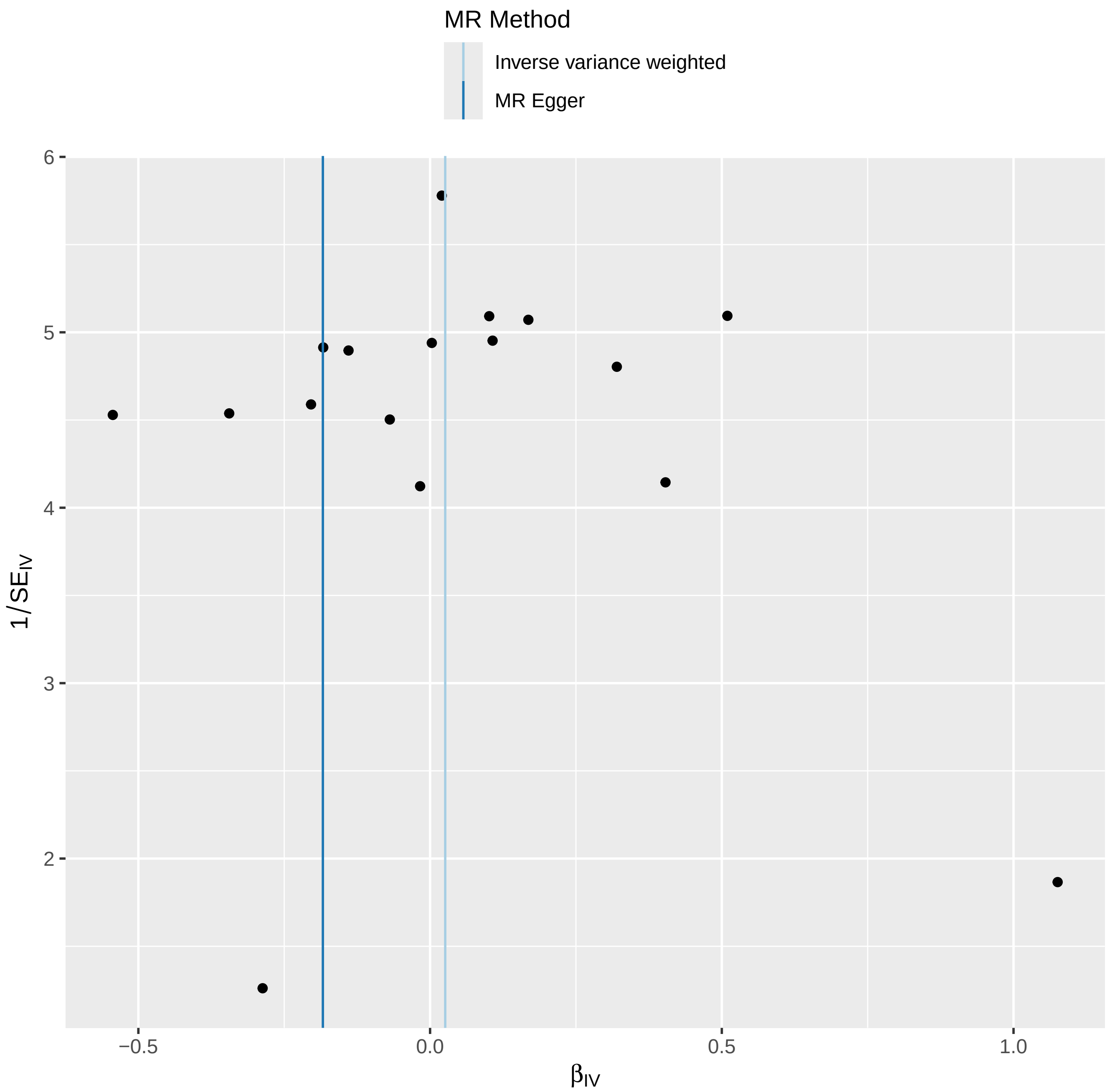
MR Method
Inverse variance weighted
MR Egger
1/SE_IV
β_IV
6
5
4
3
2
−0.5
0.0
0.5
1.0

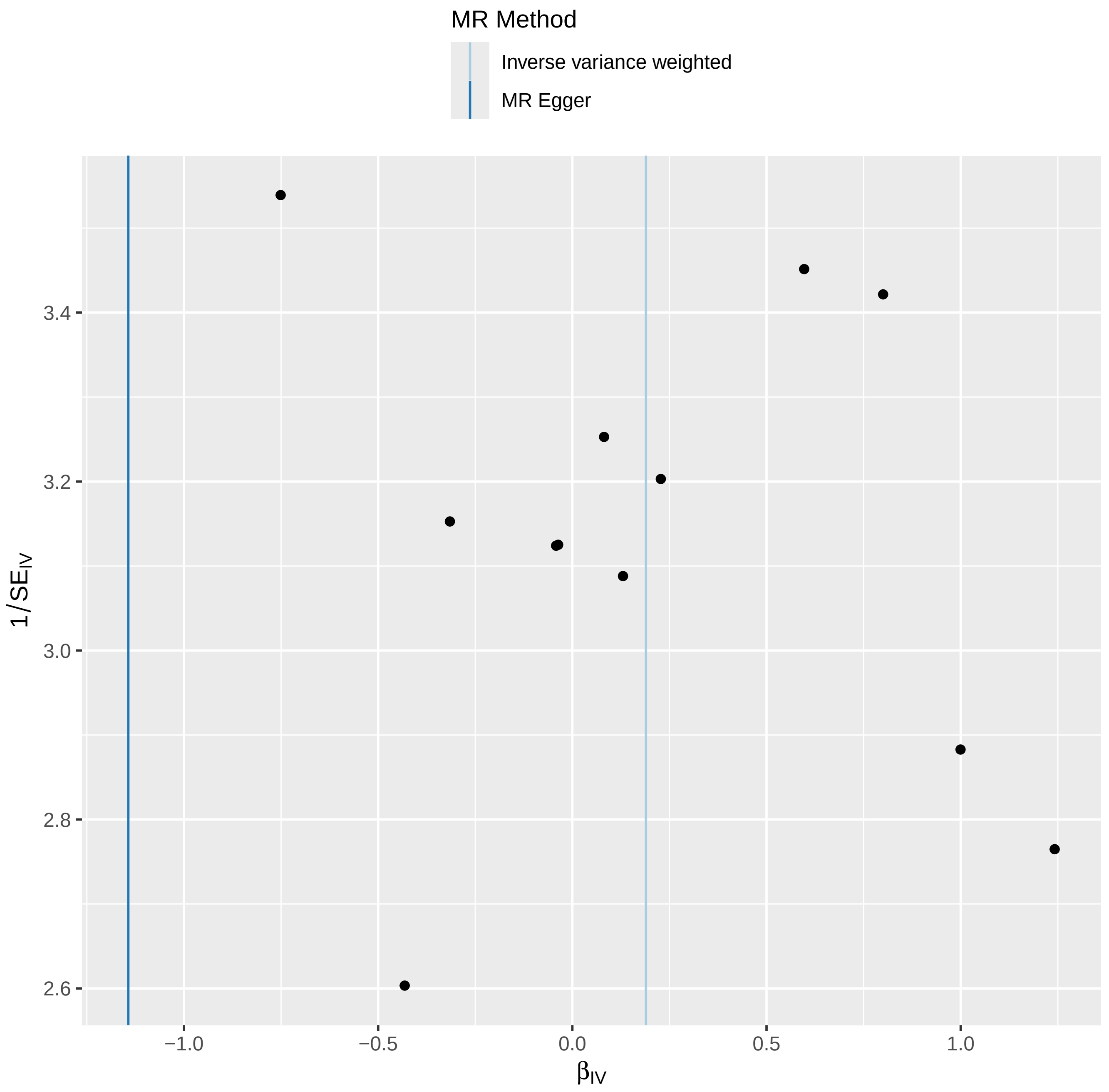
MR Method
Inverse variance weighted
MR Egger
3.4
3.2
3.0
2.8
2.6
1/SE_IV
−1.0
−0.5
0.0
0.5
1.0
β_IV

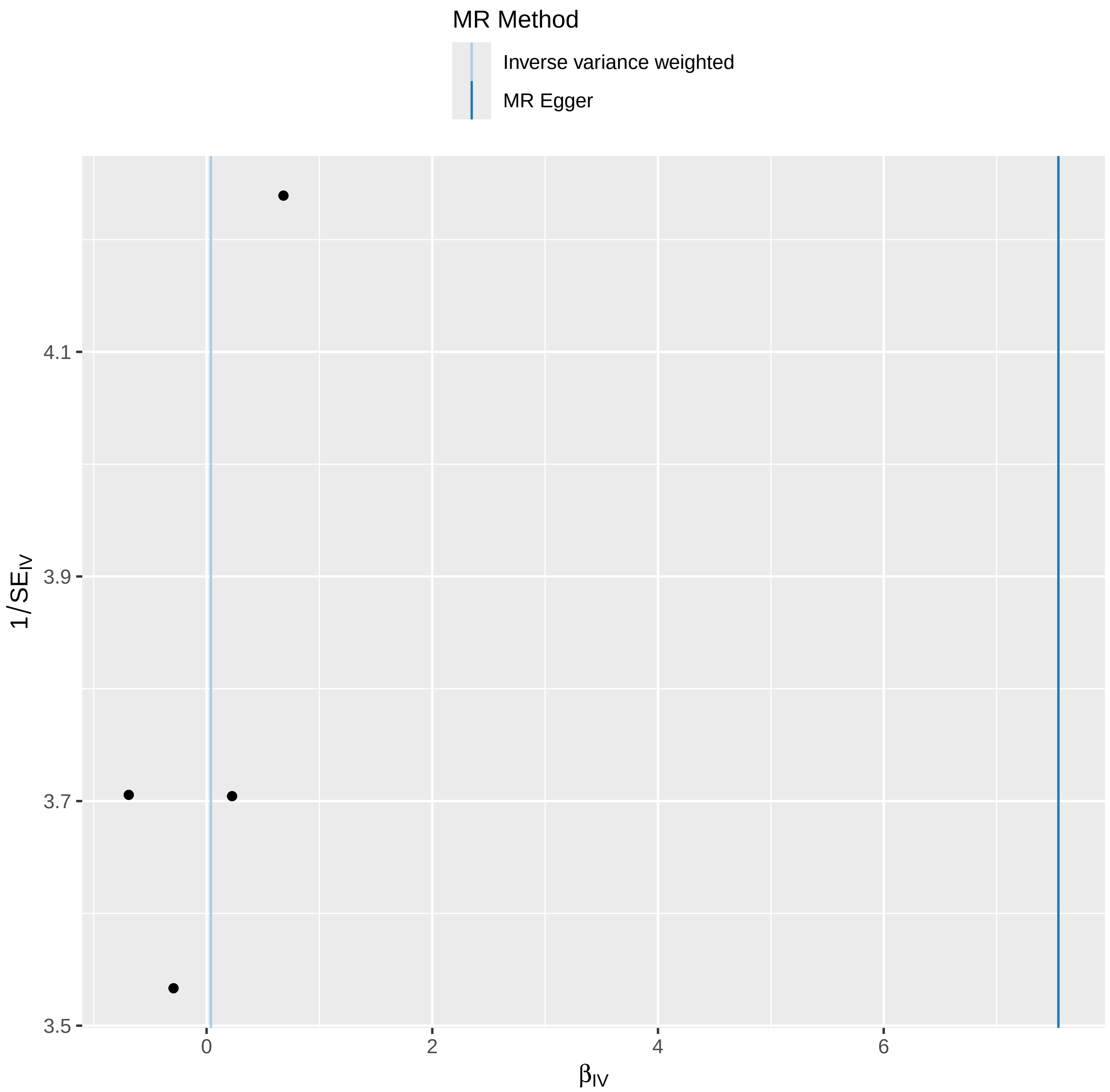

MR Method
Inverse variance weighted
MR Egger
4.1
3.9
3.7
3.5
1/SE_IV
0
2
4
6
β_IV

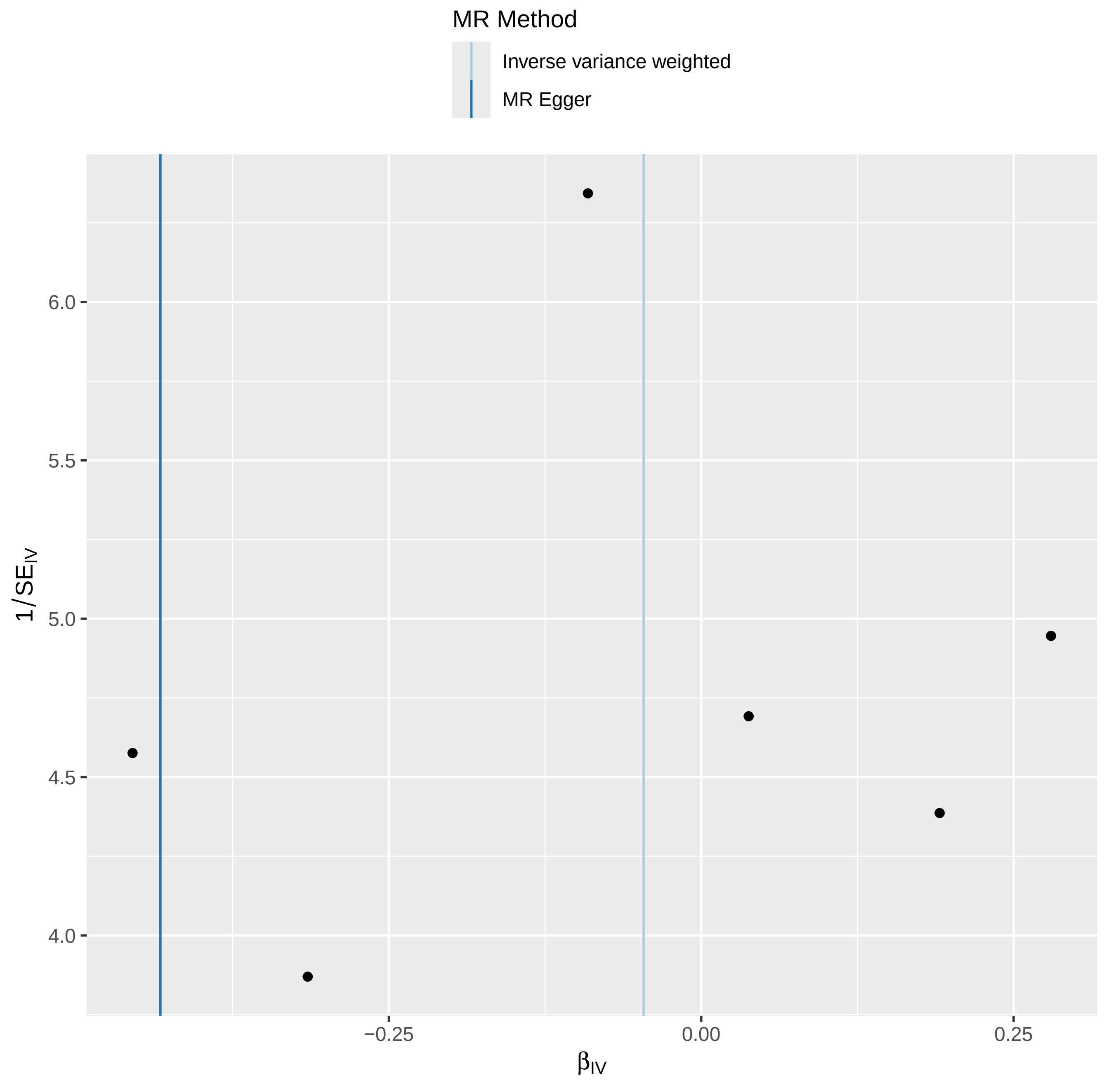

MR Method
Inverse variance weighted
MR Egger
6.0
5.5
5.0
4.5
4.0
−0.25
0.00
0.25
$1/SE_{IV}$
β_{IV}

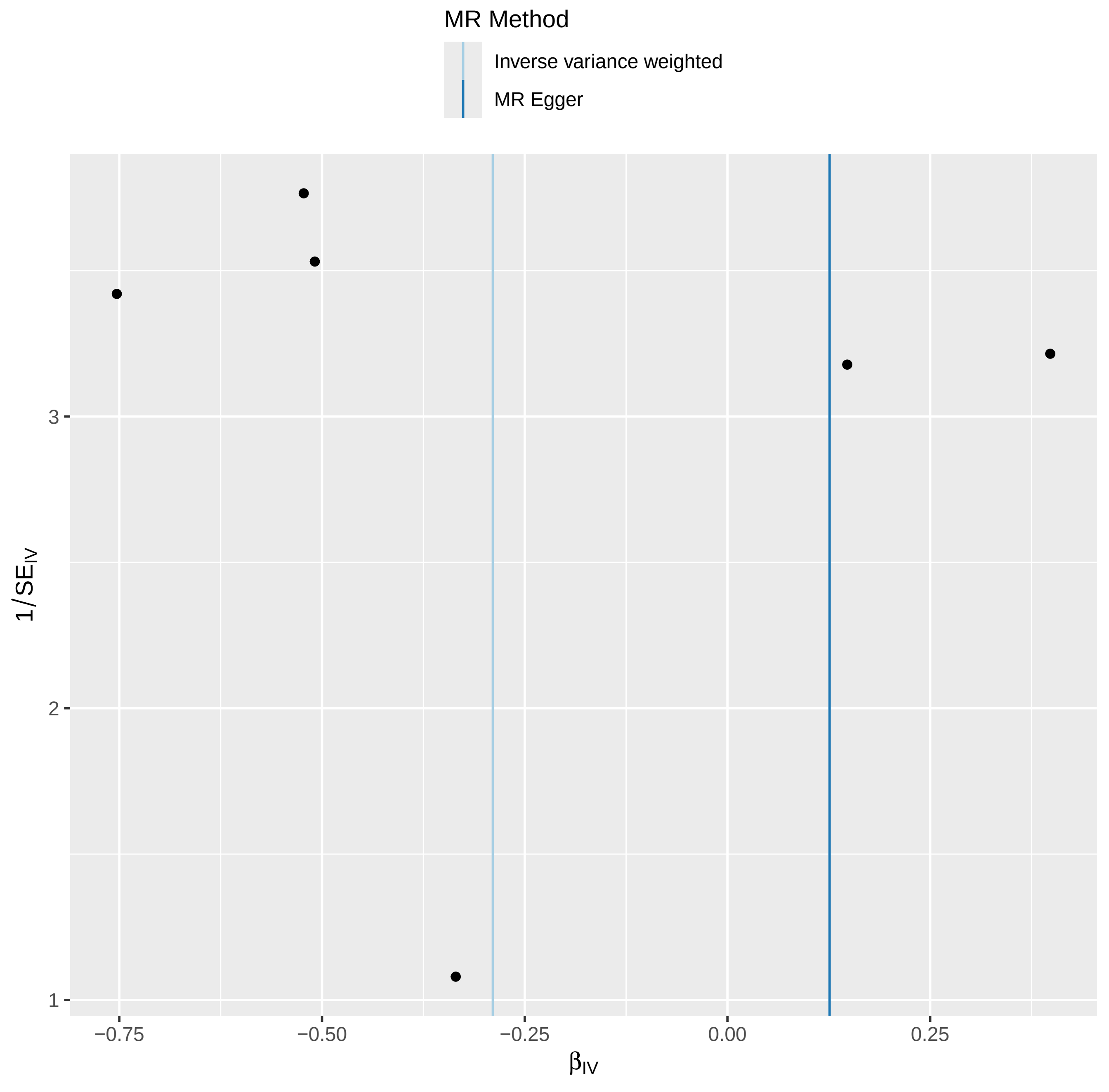

MR Method
Inverse variance weighted
MR Egger
1/SE_IV
3
2
1
−0.75
−0.50
−0.25
0.00
0.25
β_IV

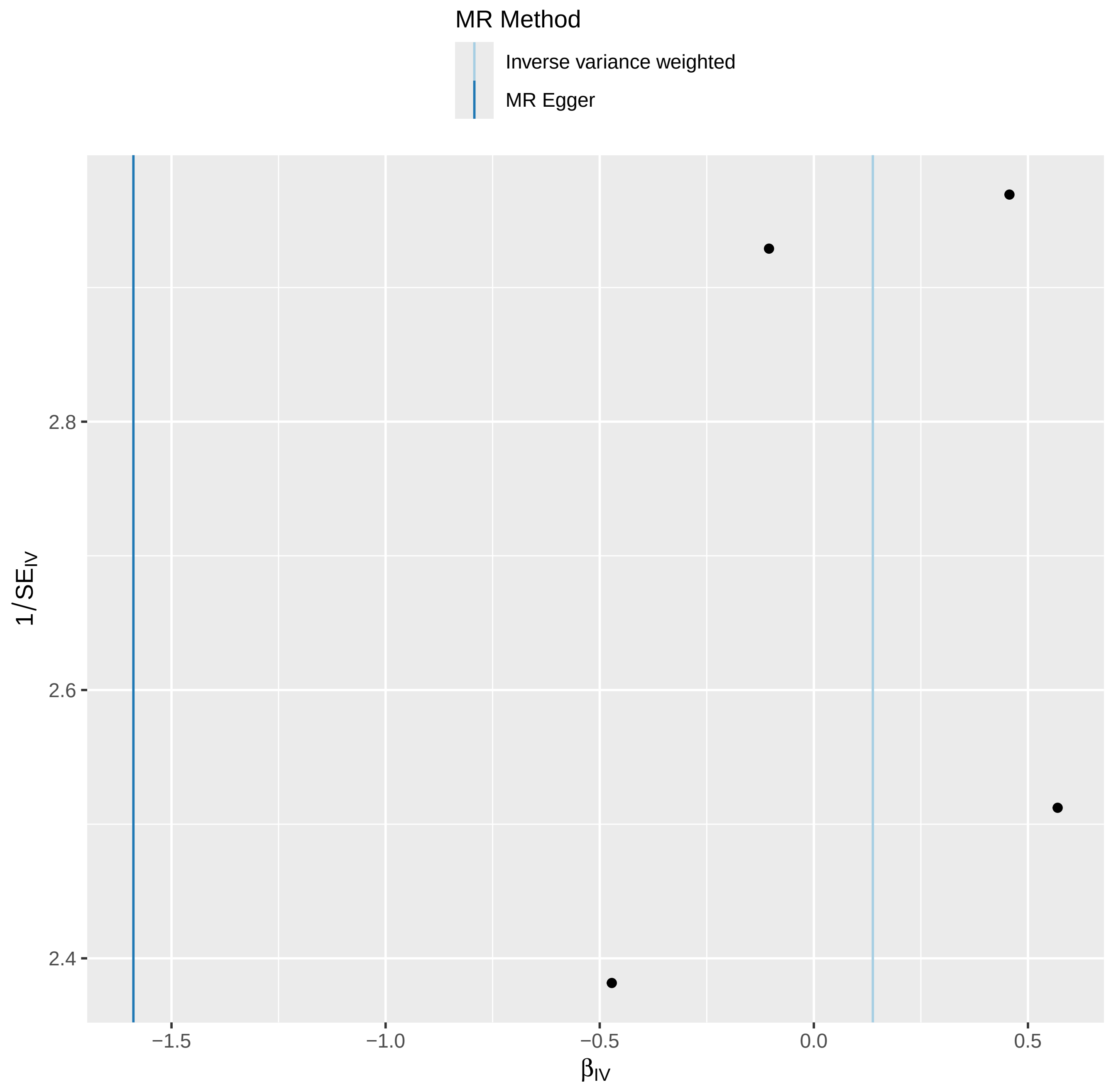

MR Method
Inverse variance weighted
MR Egger
2.8
2.6
2.4
1/SE$_{IV}$
−1.5
−1.0
−0.5
0.0
0.5
β$_{IV}$

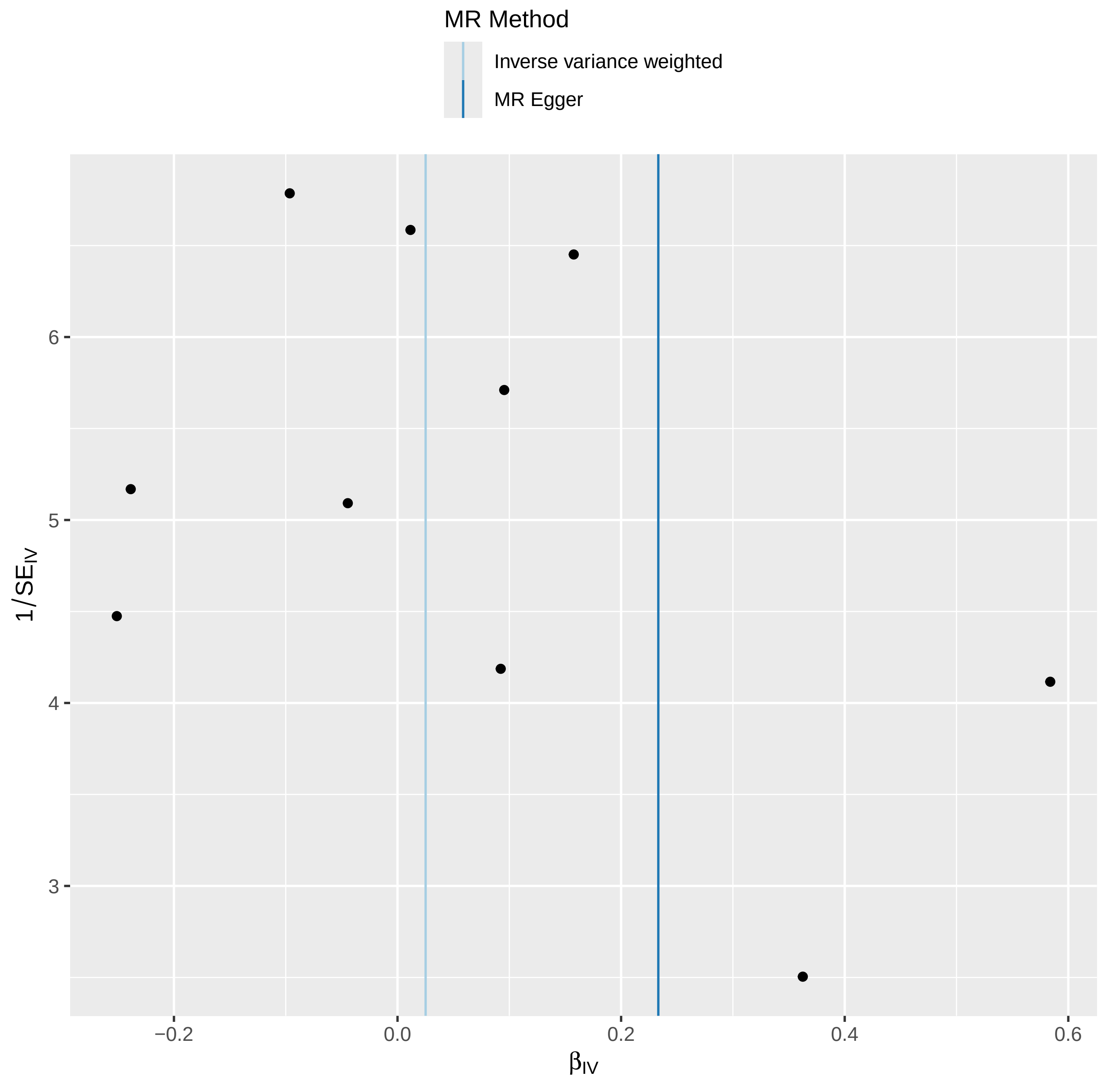
MR Method
Inverse variance weighted
MR Egger
6
5
4
3
$1/SE_{IV}$
−0.2
0.0
0.2
0.4
0.6
β_{IV}

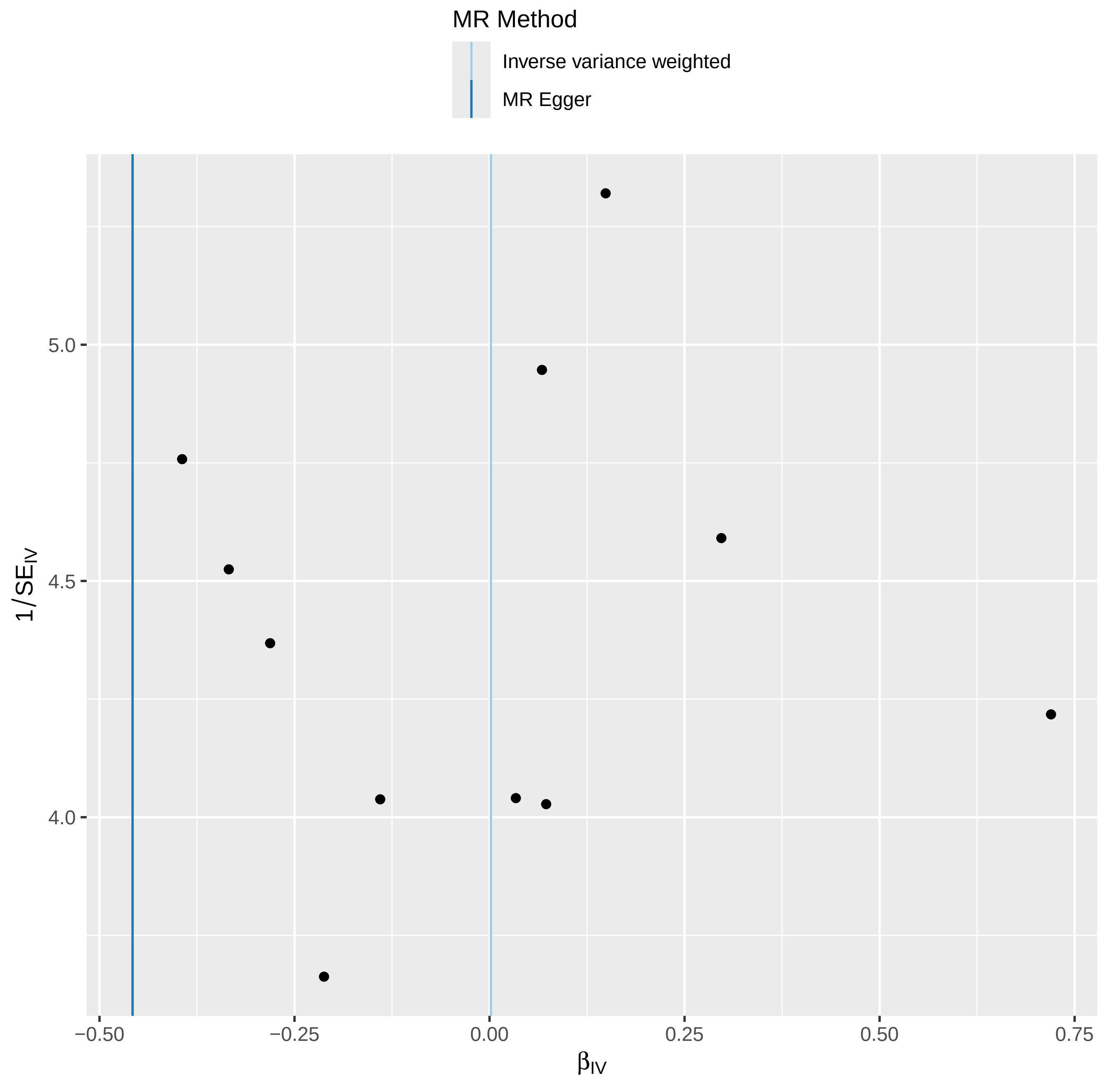
MR Method
Inverse variance weighted
MR Egger
5.0
4.5
4.0
1/SE_IV
−0.50
−0.25
0.00
0.25
0.50
0.75
β_IV

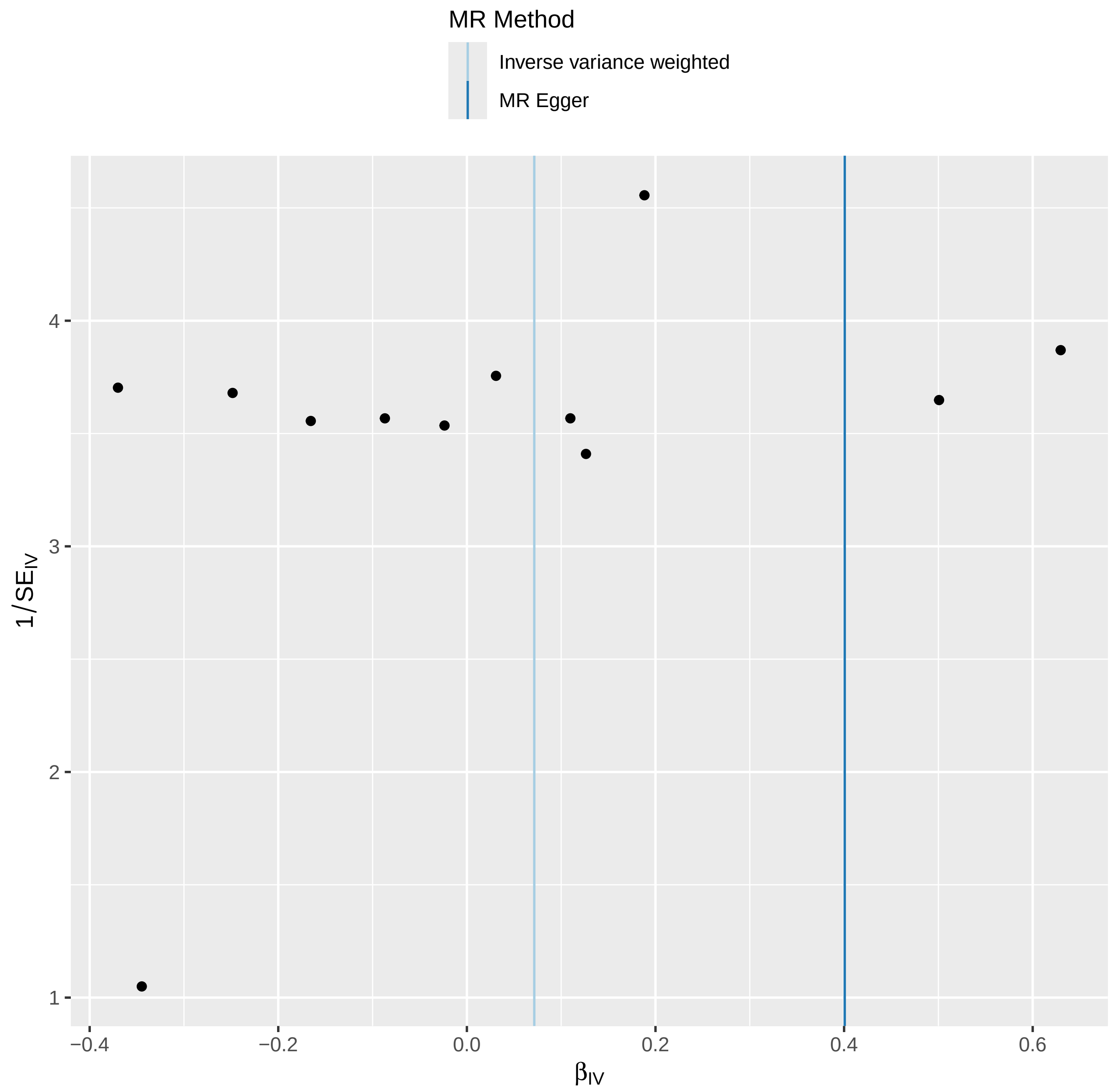
MR Method
Inverse variance weighted
MR Egger
1/SE_IV
β_IV
4
3
2
1
−0.4
−0.2
0.0
0.2
0.4
0.6

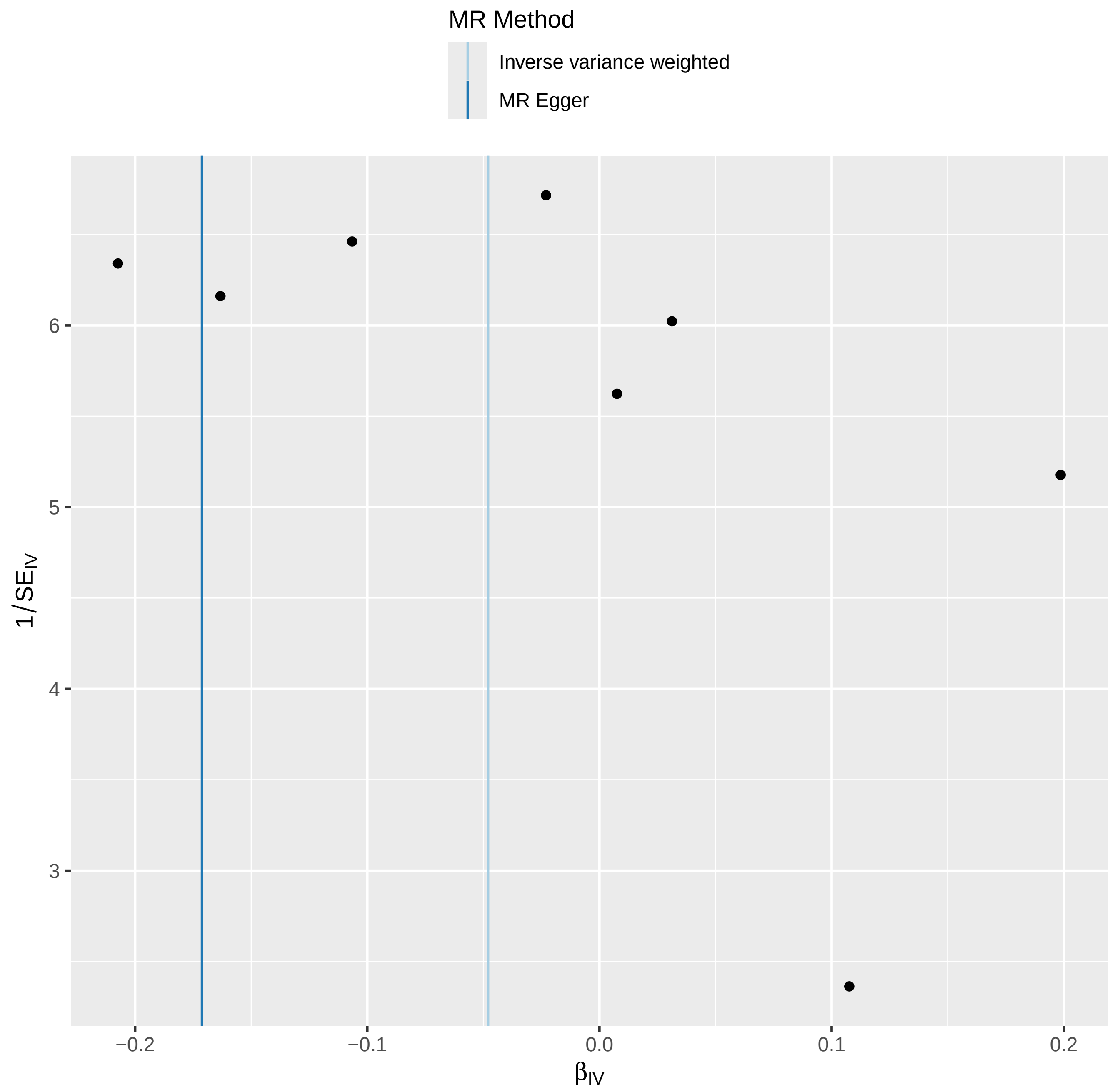
MR Method
Inverse variance weighted
MR Egger
1/SE_IV
β_IV
−0.2
−0.1
0.0
0.1
0.2
3
4
5
6

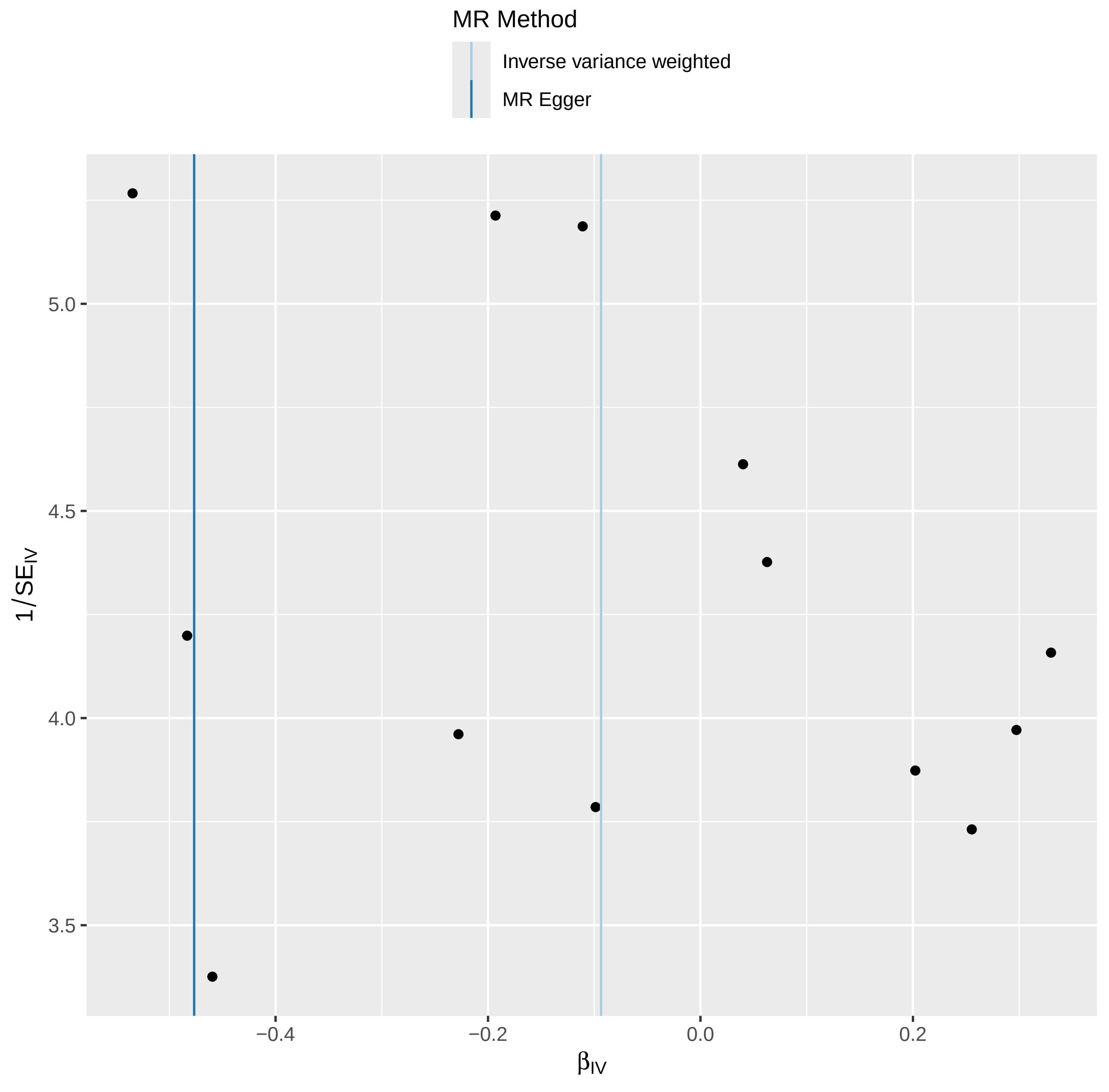
MR Method
Inverse variance weighted
MR Egger
5.0
4.5
4.0
3.5
$1/SE_{IV}$
−0.4
−0.2
0.0
0.2
β_{IV}

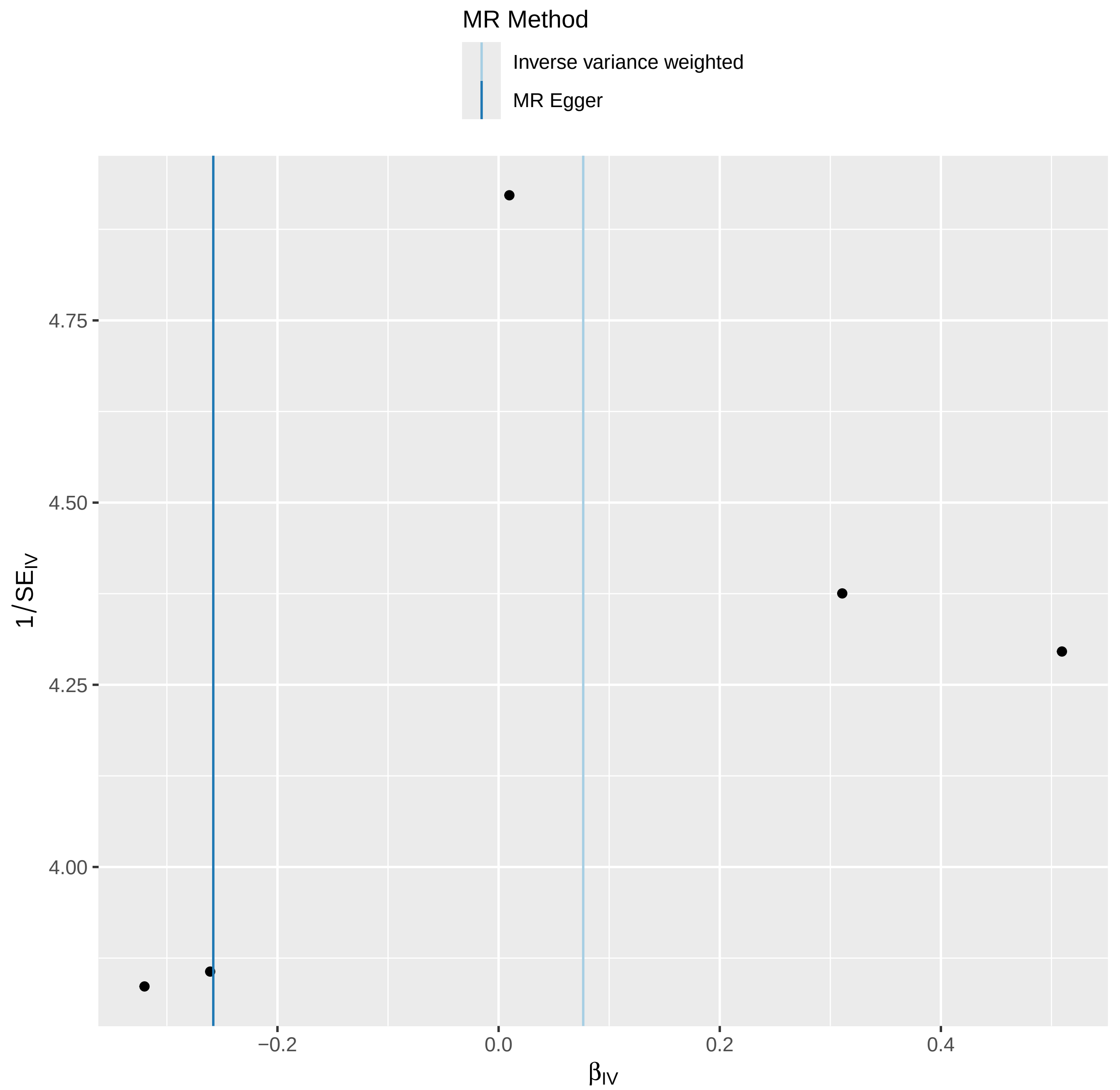

MR Method
Inverse variance weighted
MR Egger
4.75
4.50
4.25
4.00
1/SE_IV
−0.2
0.0
0.2
0.4
β_IV

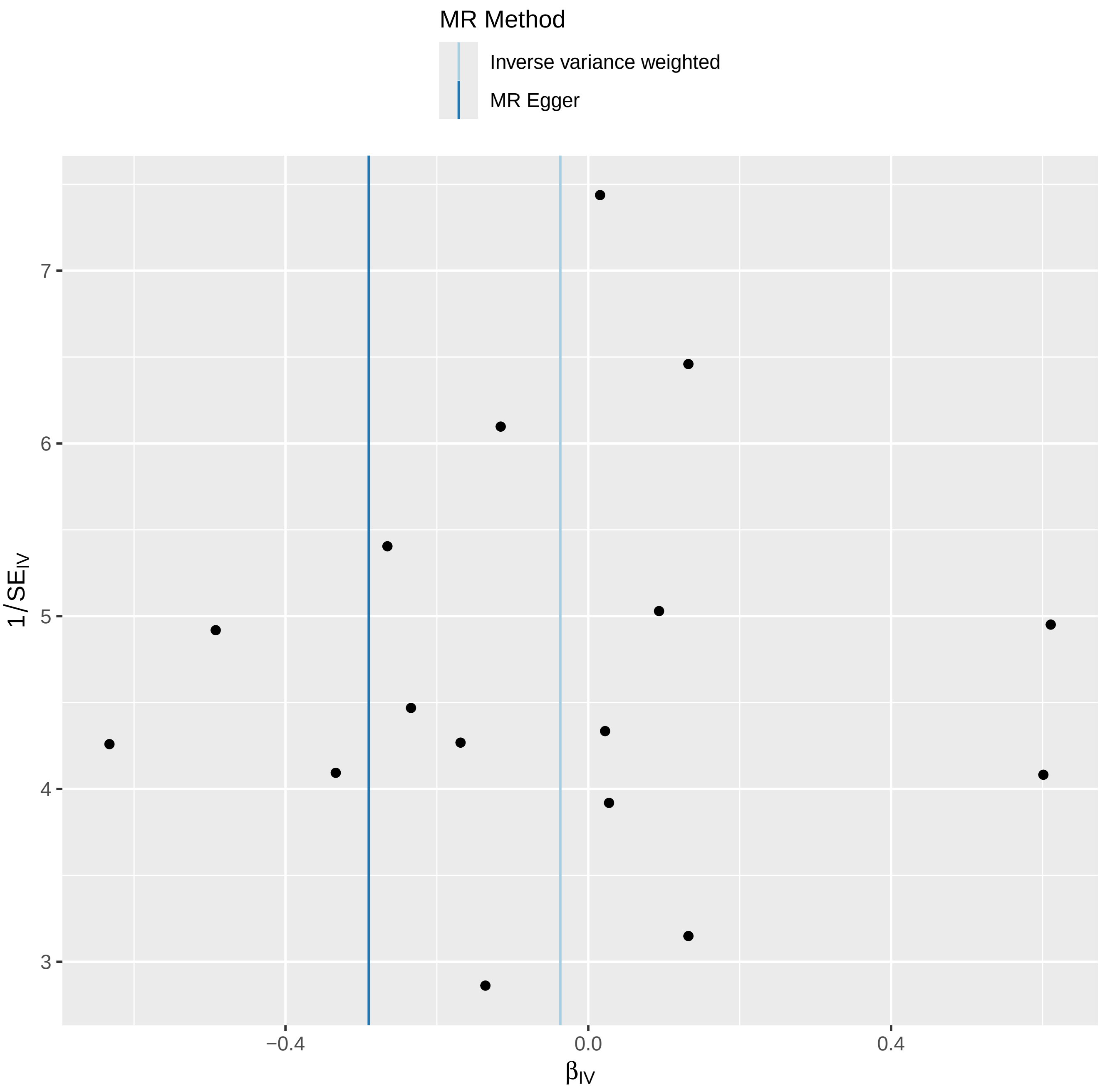

MR Method
Inverse variance weighted
MR Egger
7
6
5
4
3
1/SE_IV
−0.4
0.0
0.4
β_IV

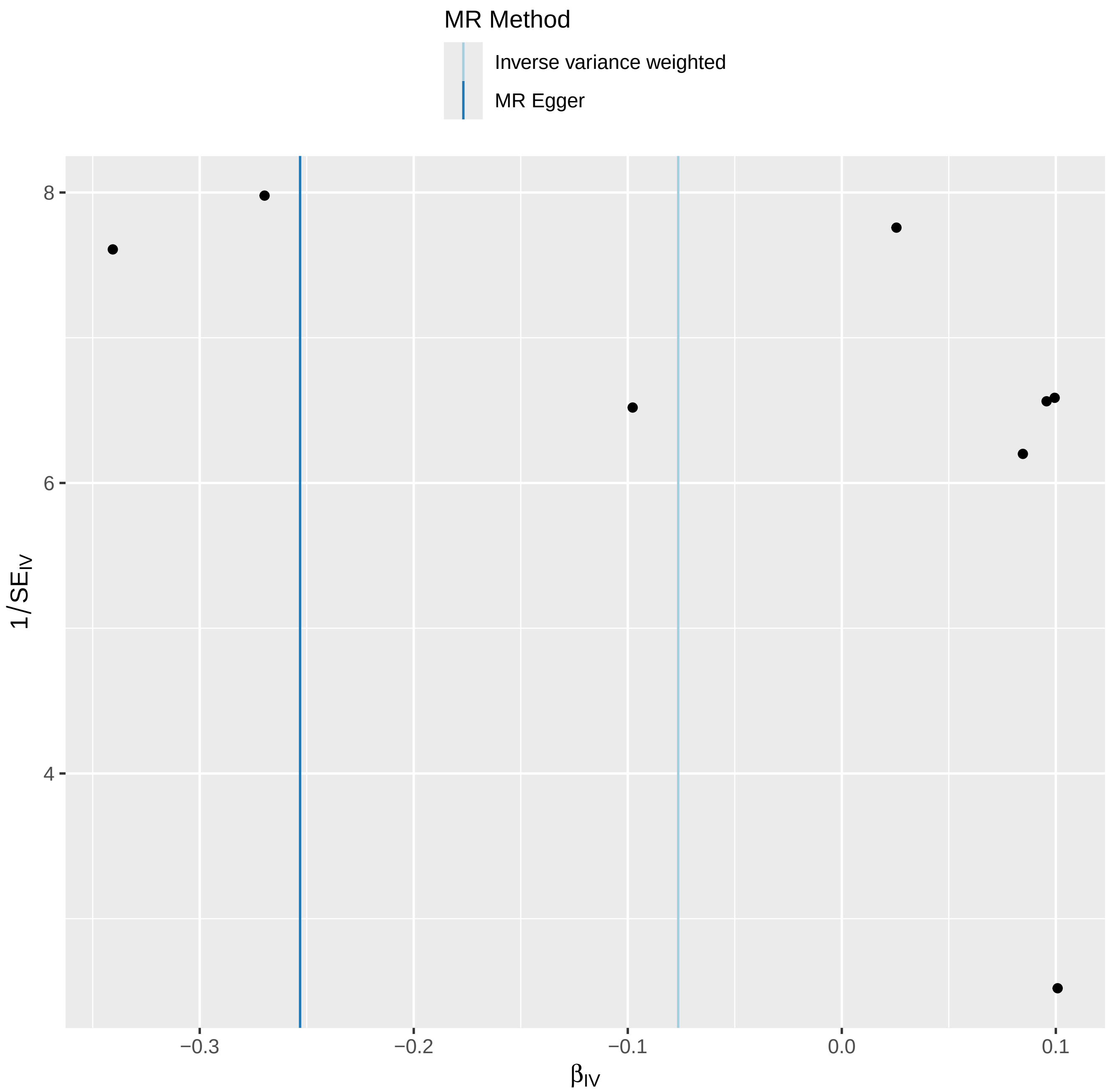

MR Method
Inverse variance weighted
MR Egger
8
6
4
1/SE_IV
−0.3
−0.2
−0.1
0.0
0.1
β_IV

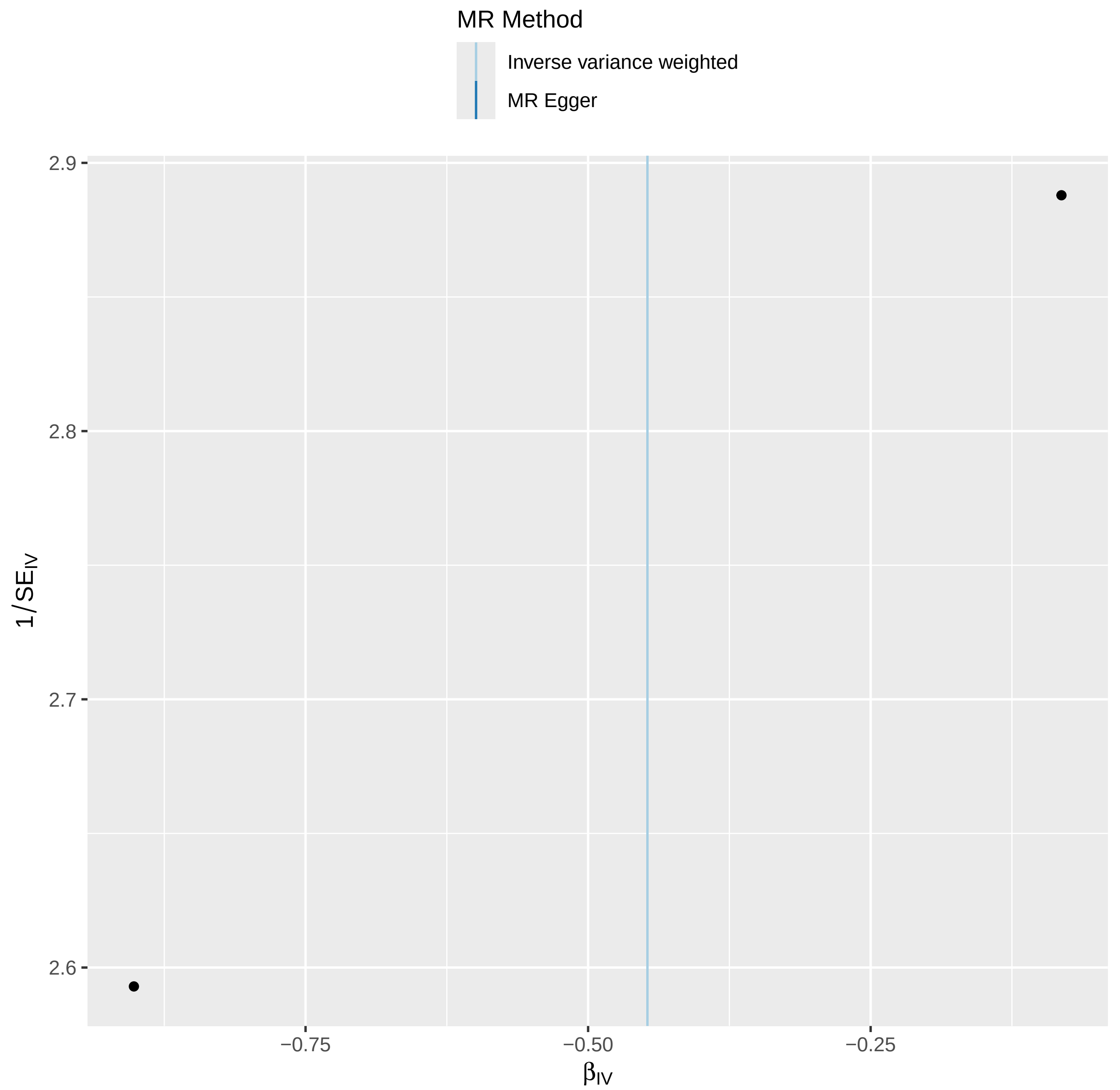

MR Method
Inverse variance weighted
MR Egger
2.9
2.8
2.7
2.6
−0.75
−0.50
−0.25
1/SE_IV
β_IV

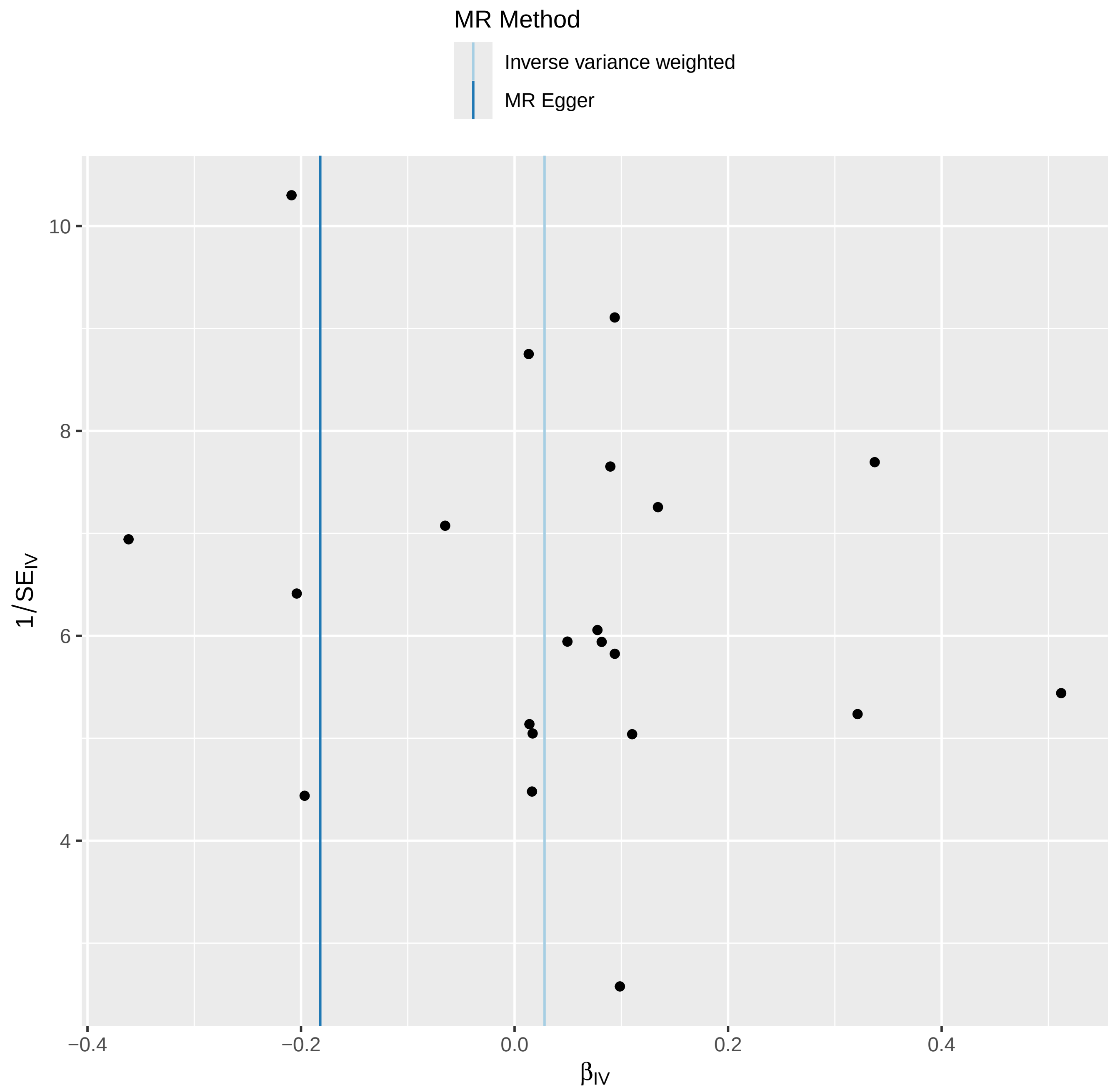
MR Method
Inverse variance weighted
MR Egger
10
8
6
4
1/SE_IV
−0.4
−0.2
0.0
0.2
0.4
β_IV

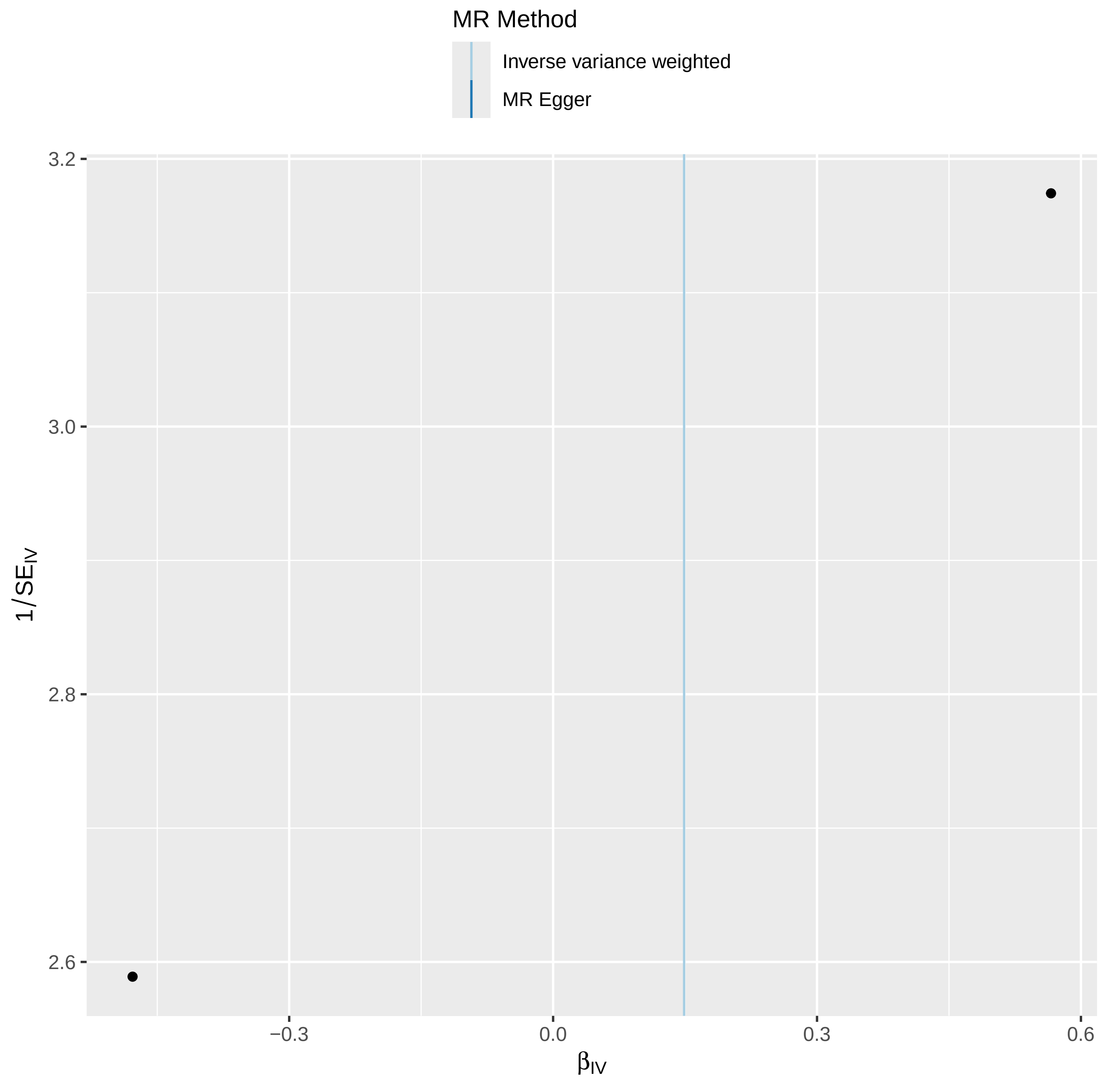

MR Method
Inverse variance weighted
MR Egger
3.2
3.0
2.8
2.6
$1/SE_{IV}$
−0.3
0.0
0.3
0.6
β_{IV}

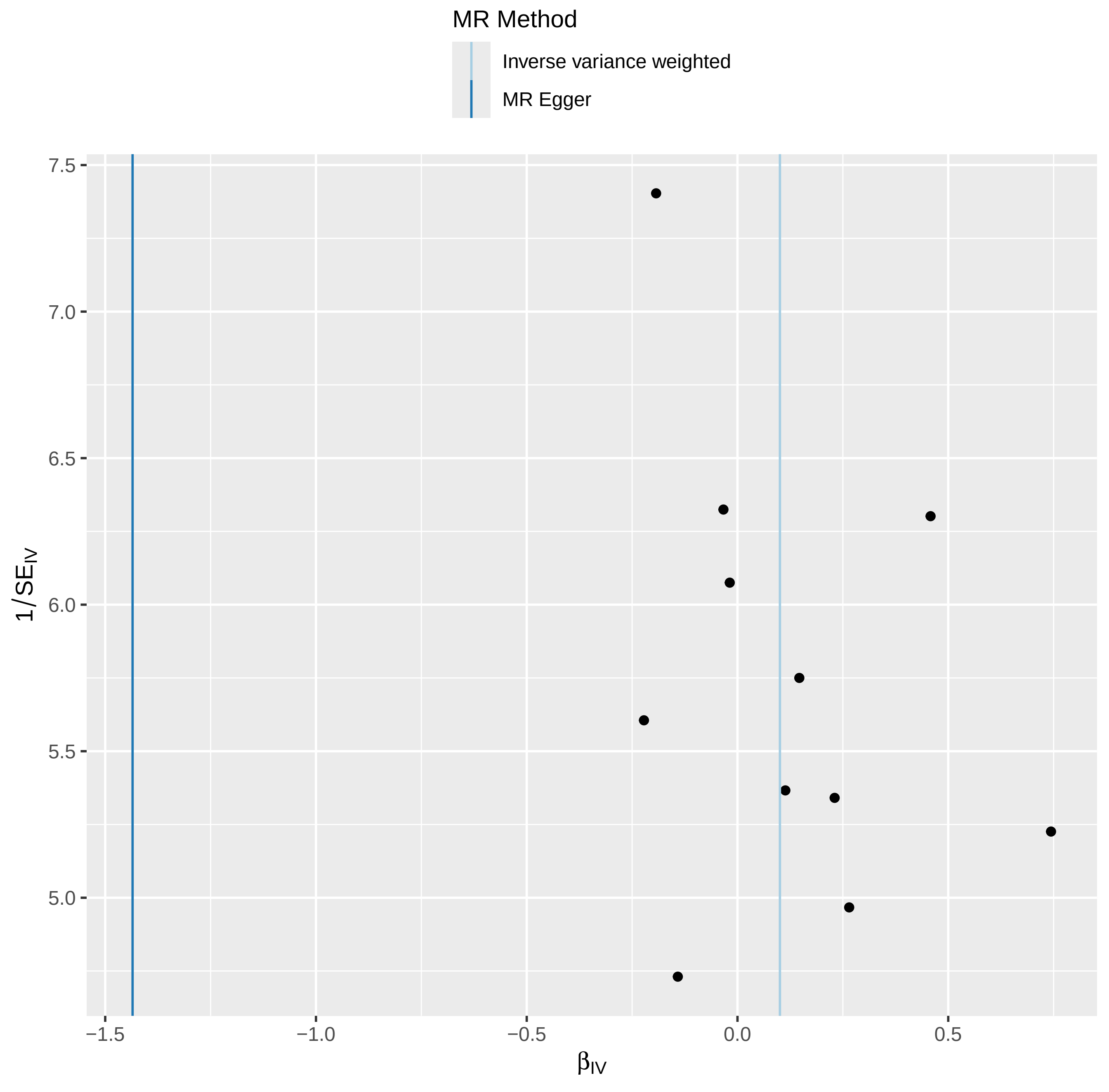
MR Method
Inverse variance weighted
MR Egger
7.5
7.0
6.5
6.0
5.5
5.0
1/SE_IV
−1.5
−1.0
−0.5
0.0
0.5
β_IV

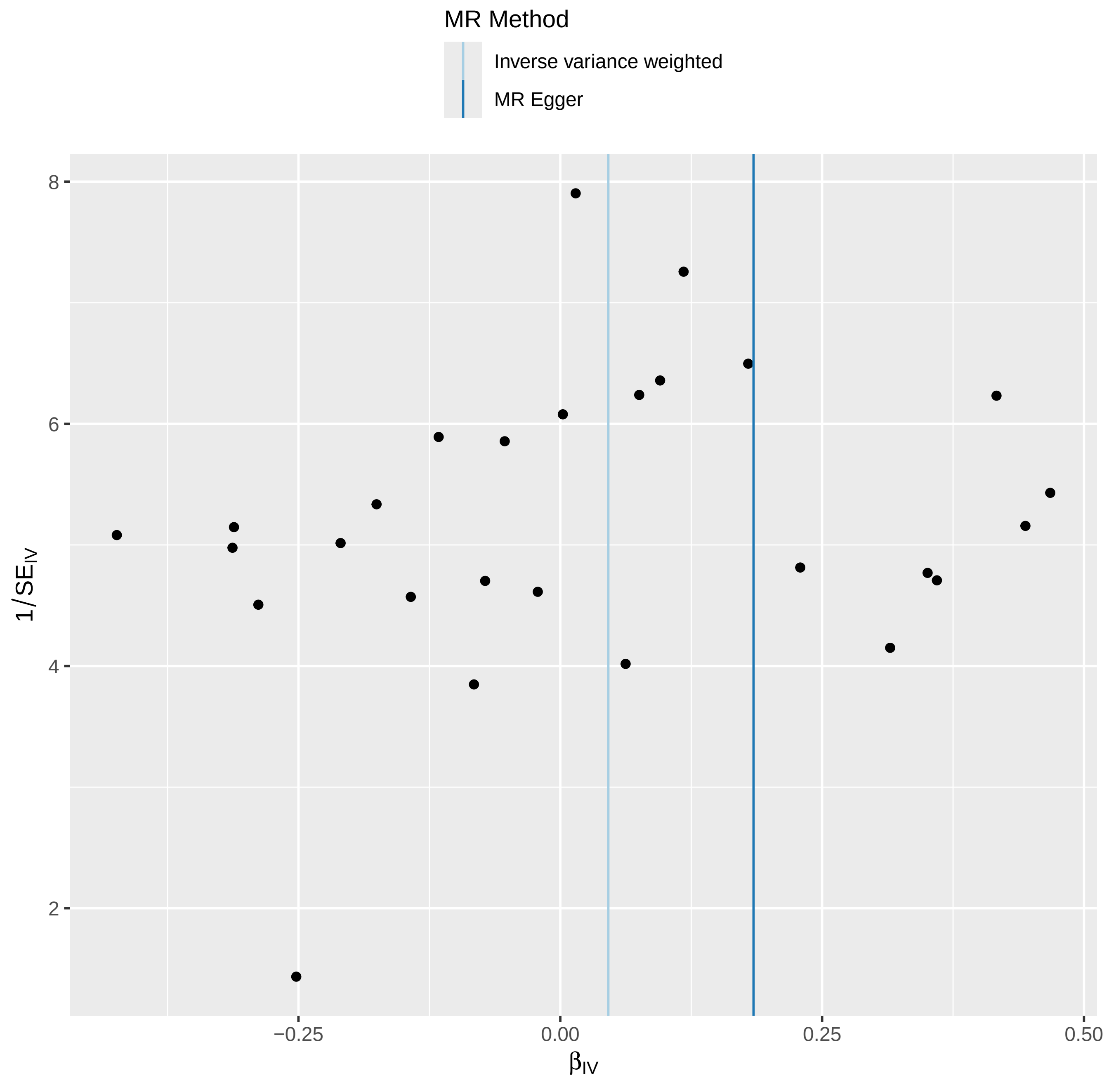

MR Method
Inverse variance weighted
MR Egger
8
6
4
2
1/SE_IV
−0.25
0.00
0.25
0.50
β_IV

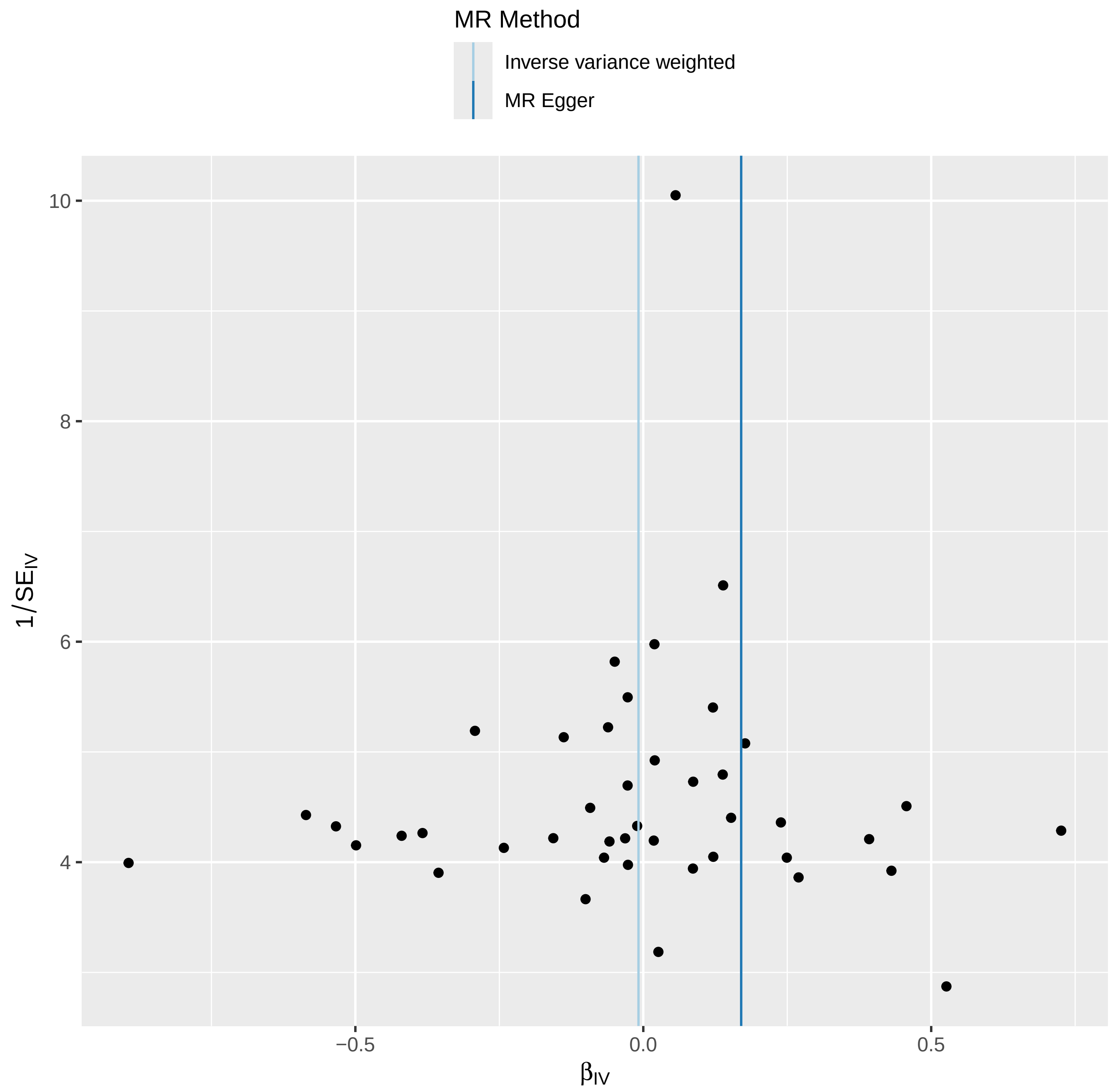

MR Method
Inverse variance weighted
MR Egger
10
8
6
4
1/SE$_{IV}$
−0.5
0.0
0.5
β$_{IV}$

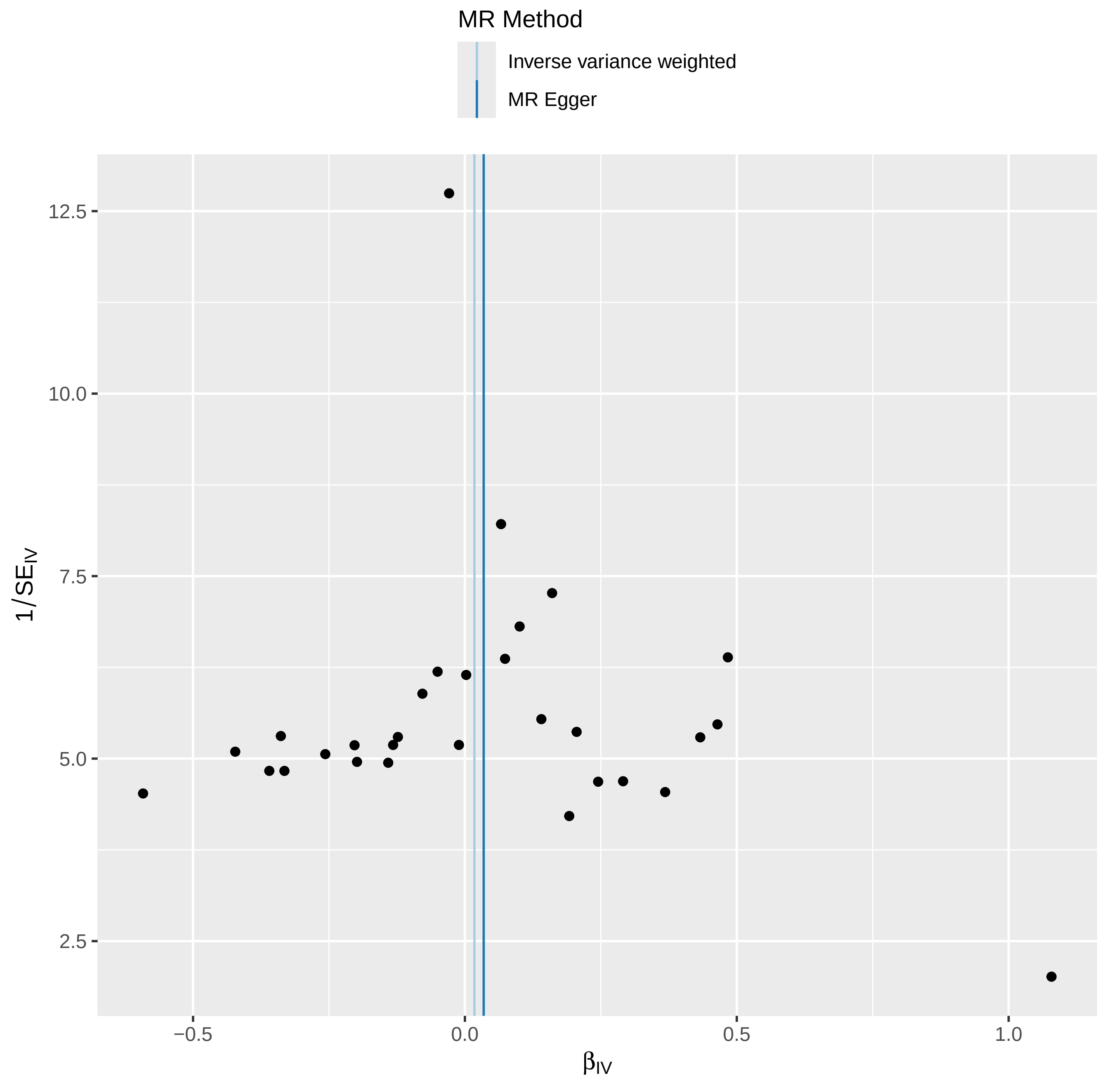

MR Method
Inverse variance weighted
MR Egger
12.5
10.0
7.5
5.0
2.5
1/SE_IV
−0.5
0.0
0.5
1.0
β_IV

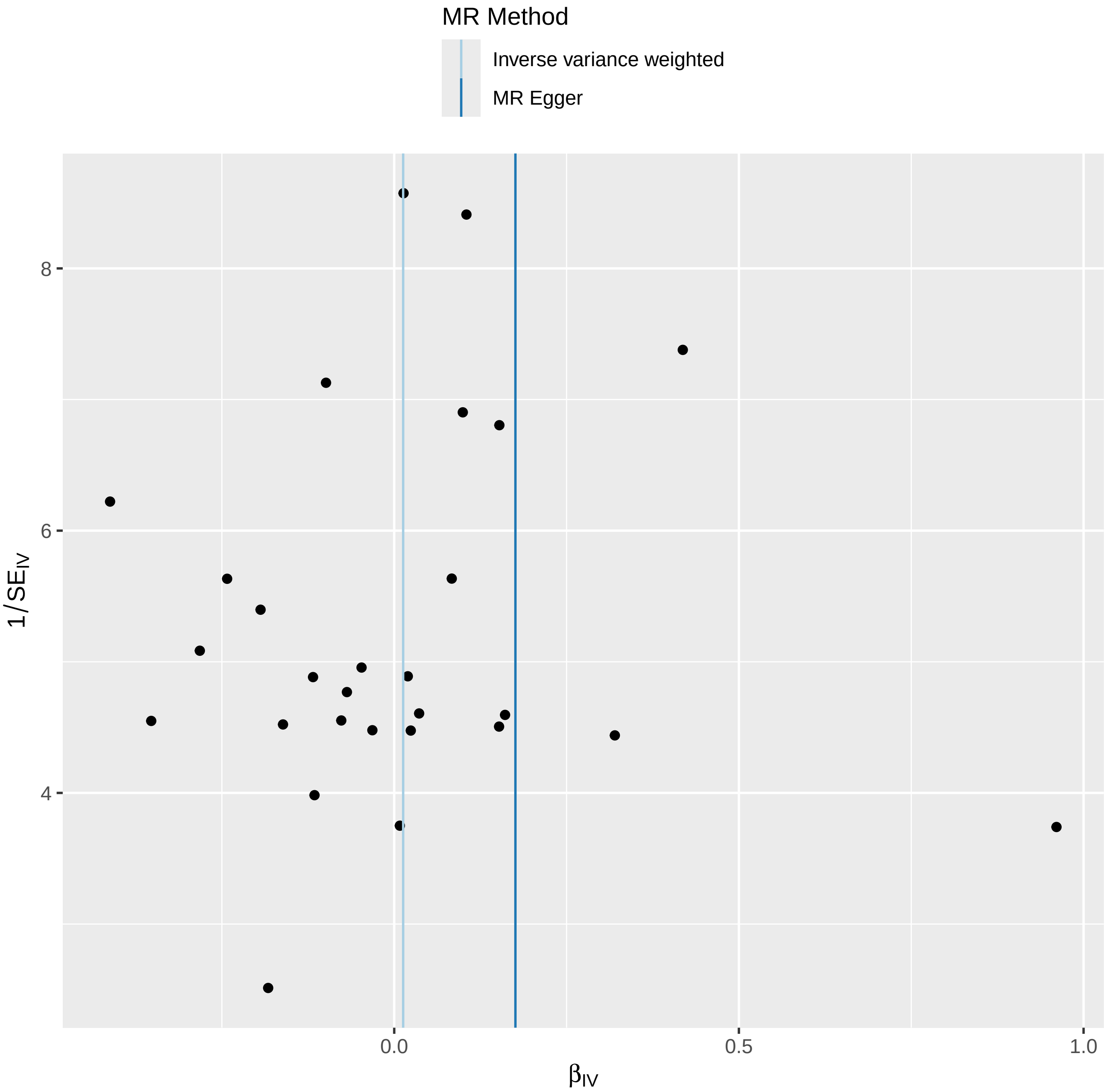

MR Method
Inverse variance weighted
MR Egger
8
6
4
1/SE_IV
0.0
0.5
1.0
β_IV

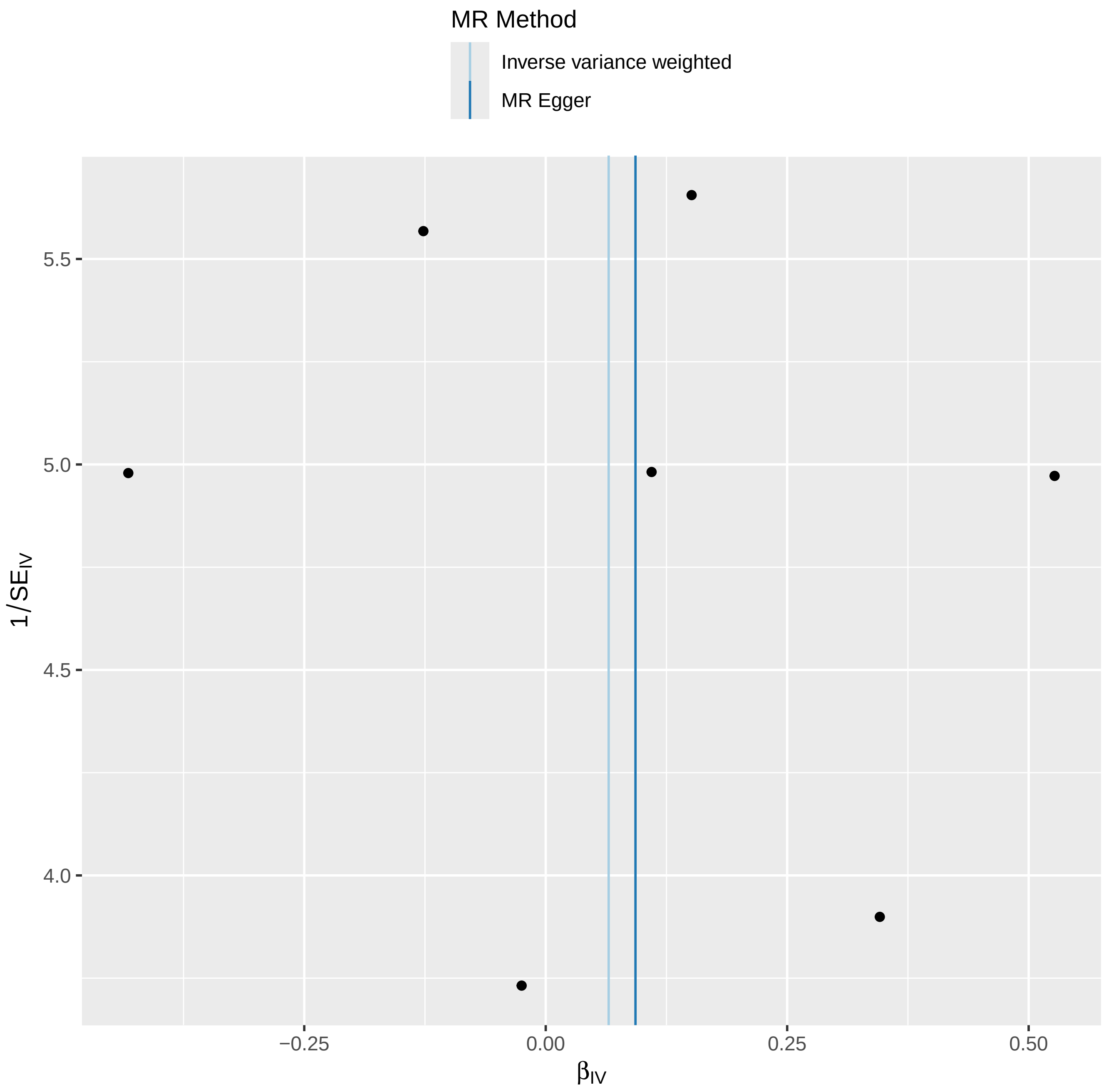
MR Method
Inverse variance weighted
MR Egger
5.5
5.0
4.5
4.0
1/SE_IV
−0.25
0.00
0.25
0.50
β_IV

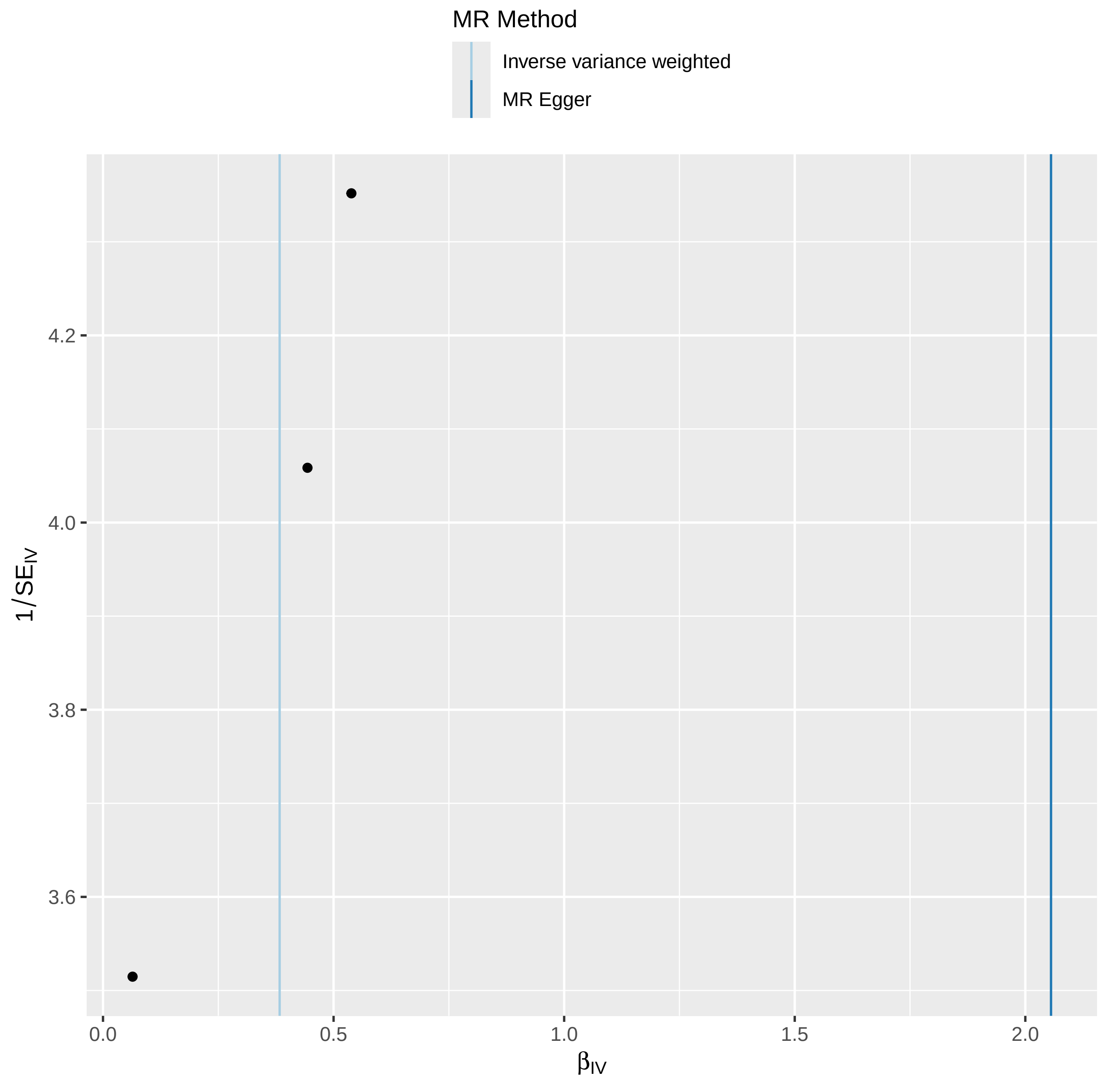
MR Method
Inverse variance weighted
MR Egger
4.2
4.0
3.8
3.6
1/SE_IV
0.0
0.5
1.0
1.5
2.0
β_IV

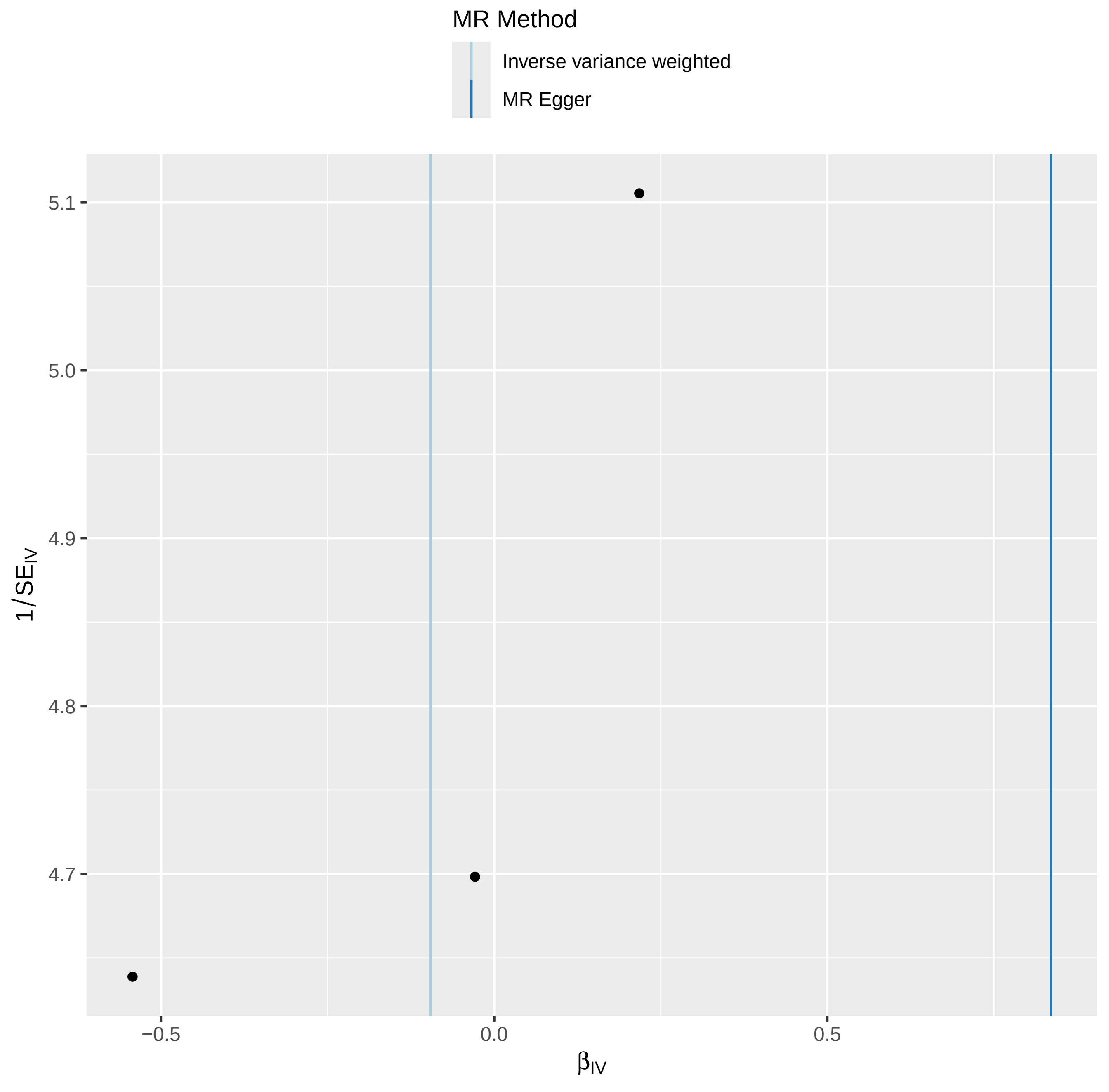
MR Method
Inverse variance weighted
MR Egger
5.1
5.0
4.9
4.8
4.7
1/SE_IV
−0.5
0.0
0.5
β_IV

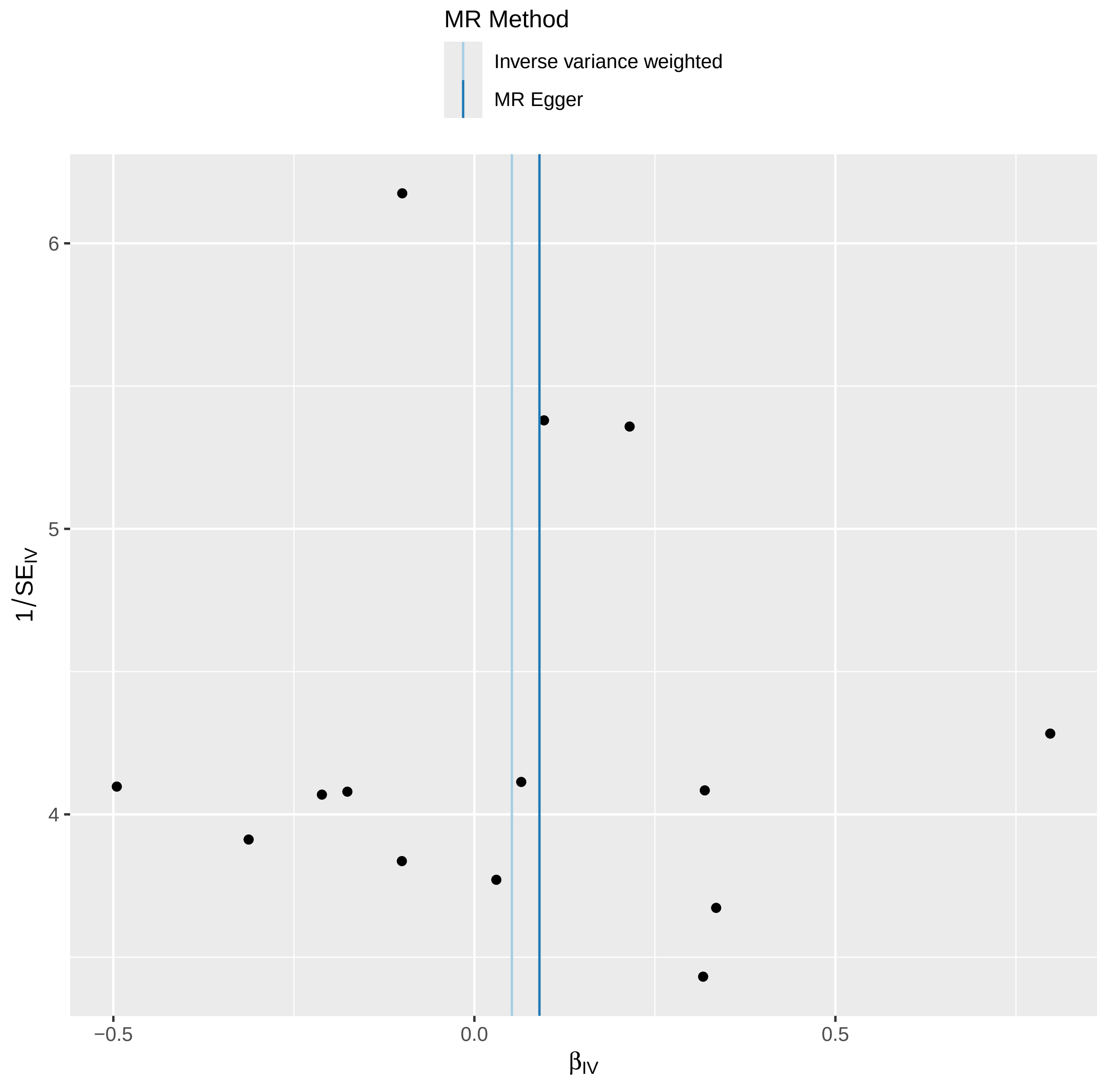
MR Method
Inverse variance weighted
MR Egger
6
5
4
1/SE_IV
−0.5
0.0
0.5
β_IV

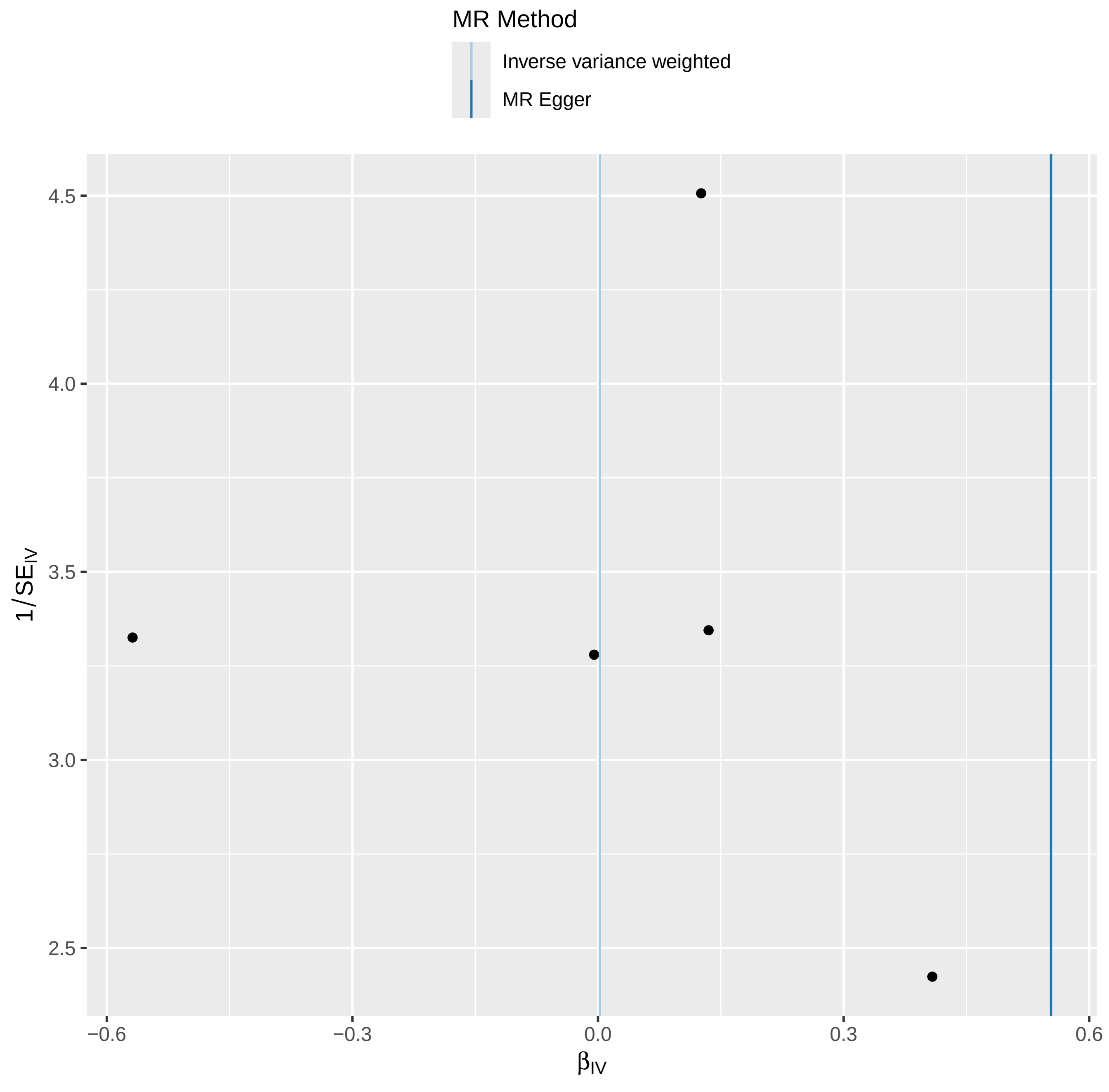

MR Method
Inverse variance weighted
MR Egger
4.5
4.0
3.5
3.0
2.5
$1/SE_{IV}$
−0.6
−0.3
0.0
0.3
0.6
β_{IV}

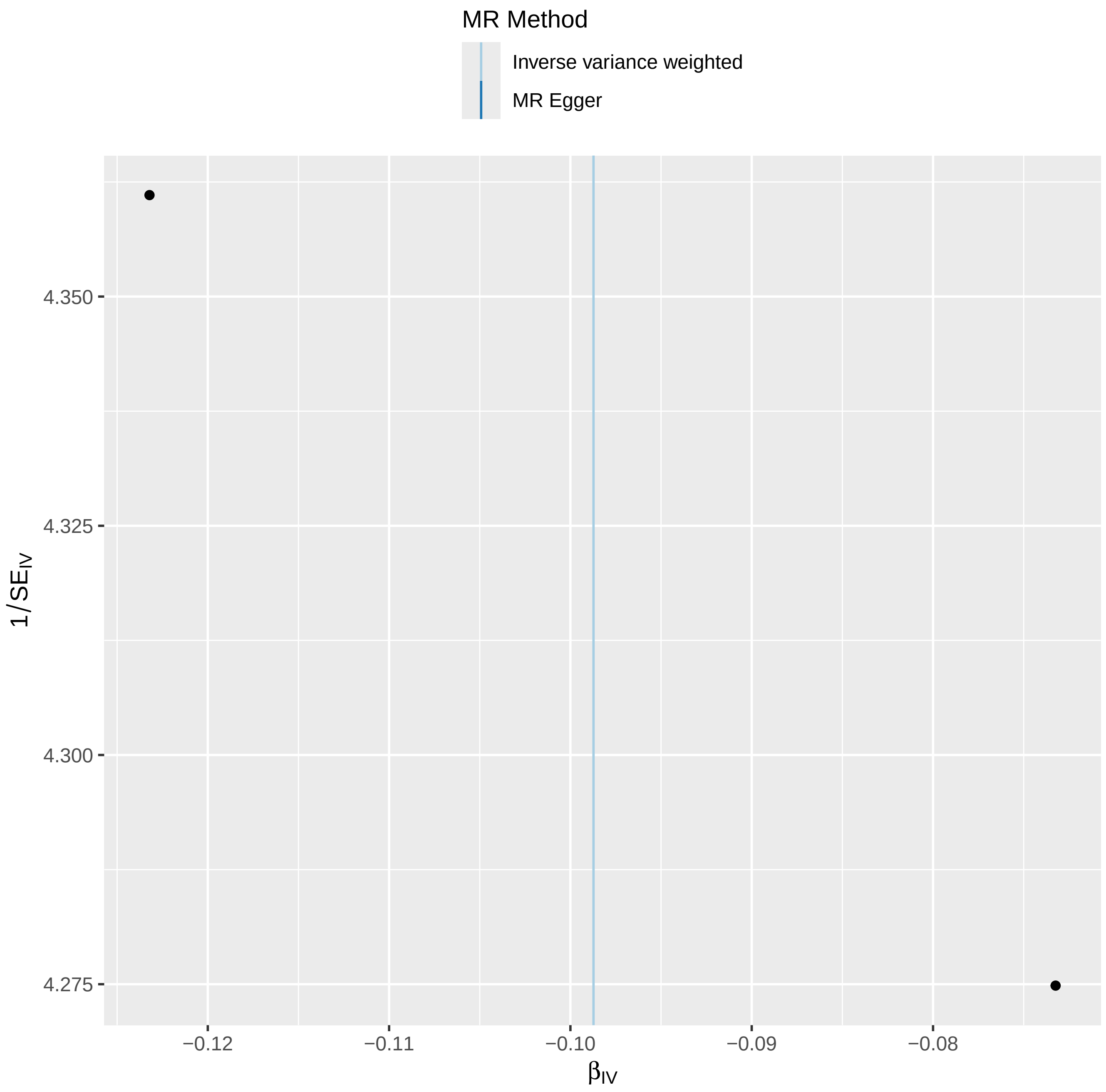
MR Method
Inverse variance weighted
MR Egger
4.350
4.325
4.300
4.275
1/SE_IV
−0.12
−0.11
−0.10
−0.09
−0.08
β_IV

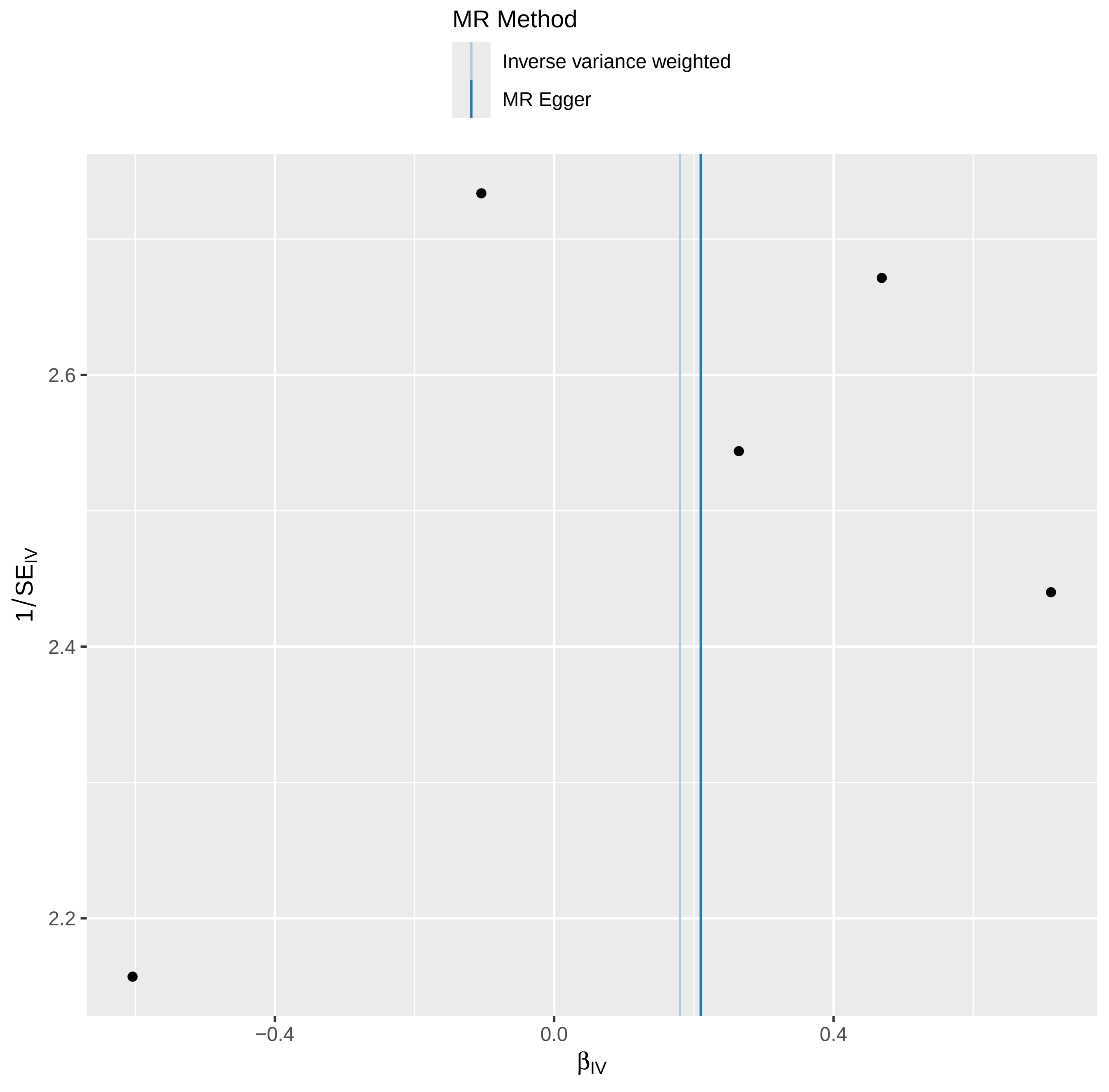

MR Method
Inverse variance weighted
MR Egger
2.6
2.4
2.2
1/SE_IV
−0.4
0.0
0.4
β_IV

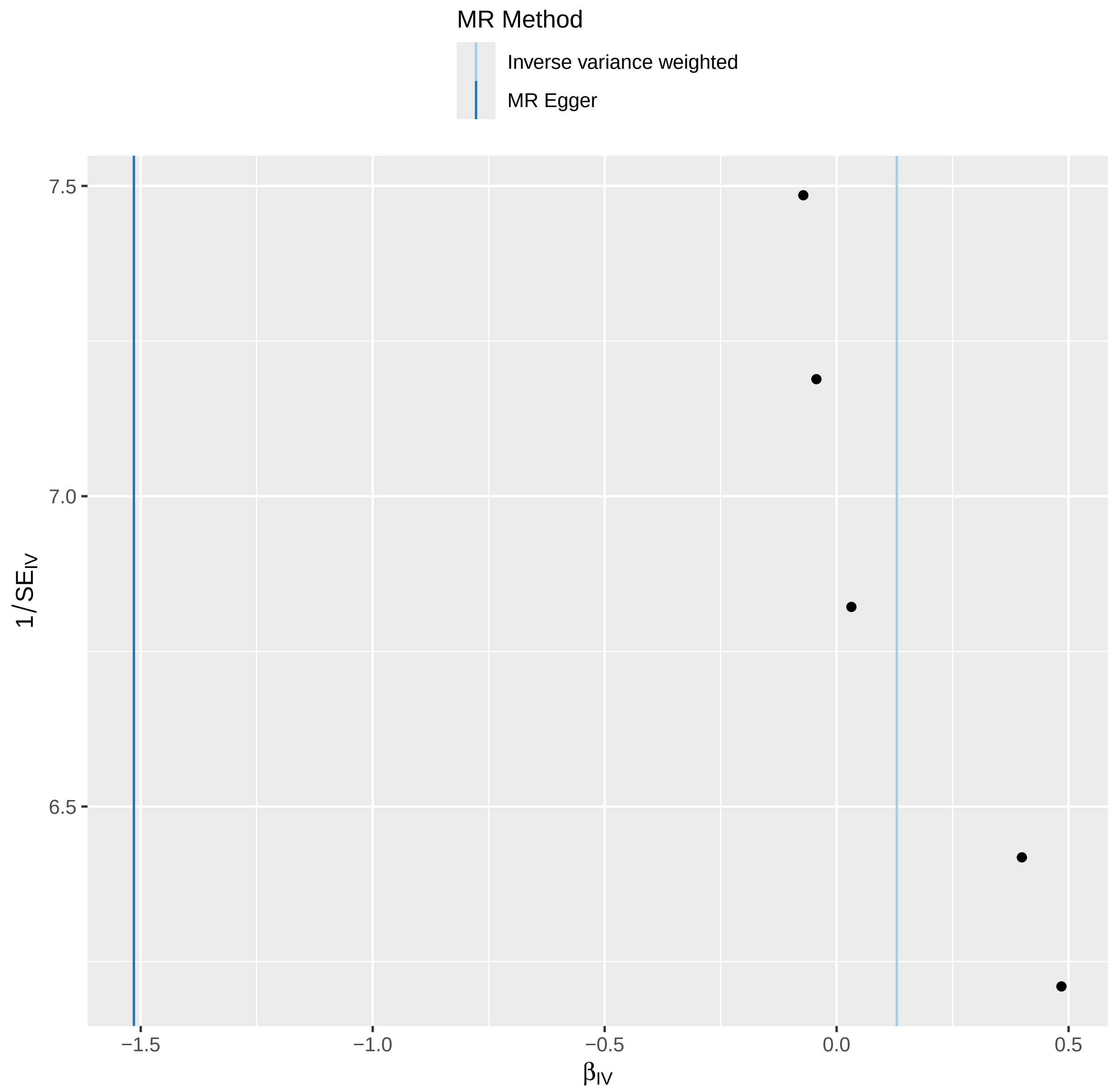

MR Method
Inverse variance weighted
MR Egger
7.5
7.0
6.5
1/SE_IV
−1.5
−1.0
−0.5
0.0
0.5
β_IV

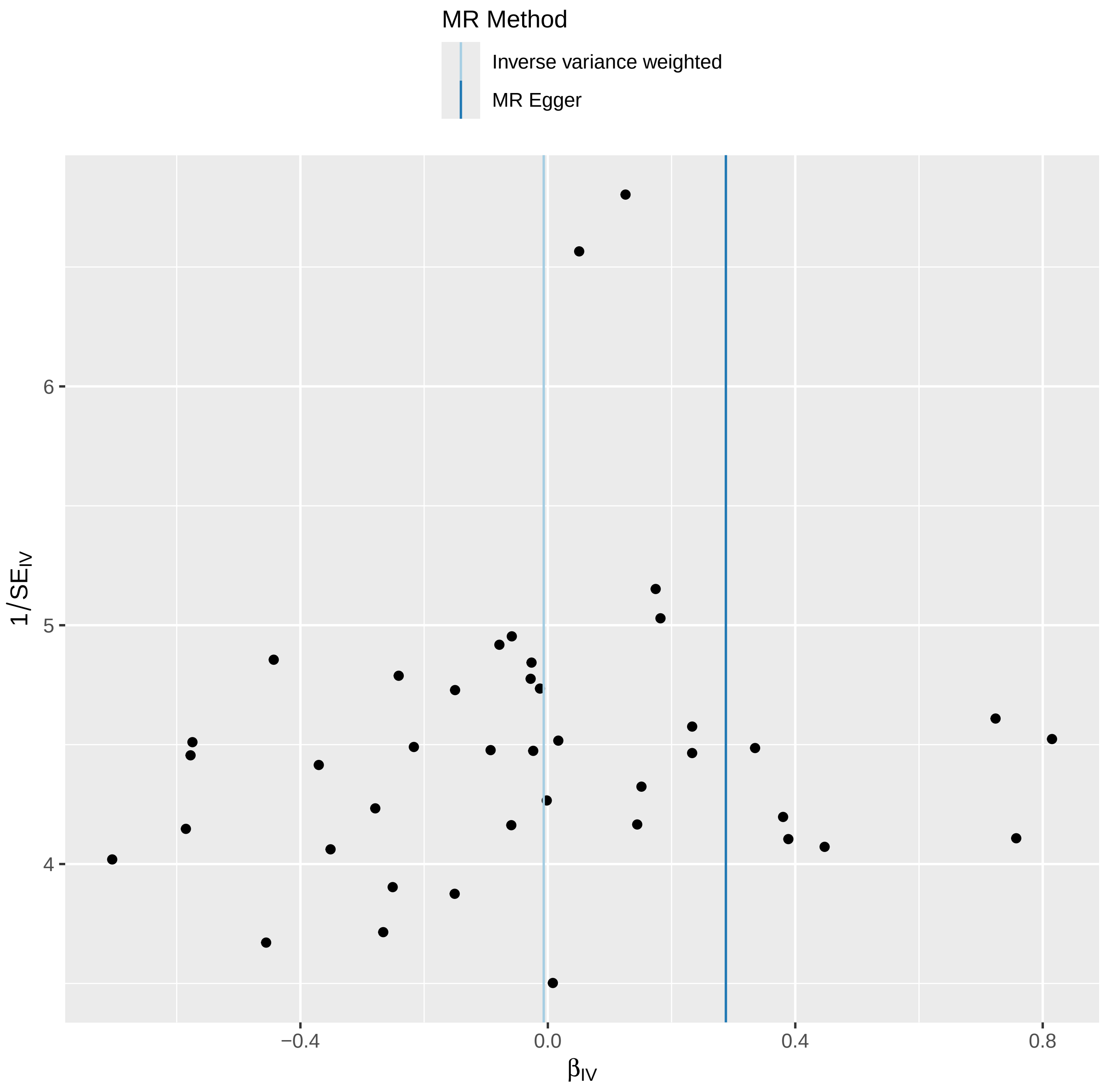
MR Method
Inverse variance weighted
MR Egger
1/SE_IV
6
5
4
−0.4
0.0
0.4
0.8
β_IV

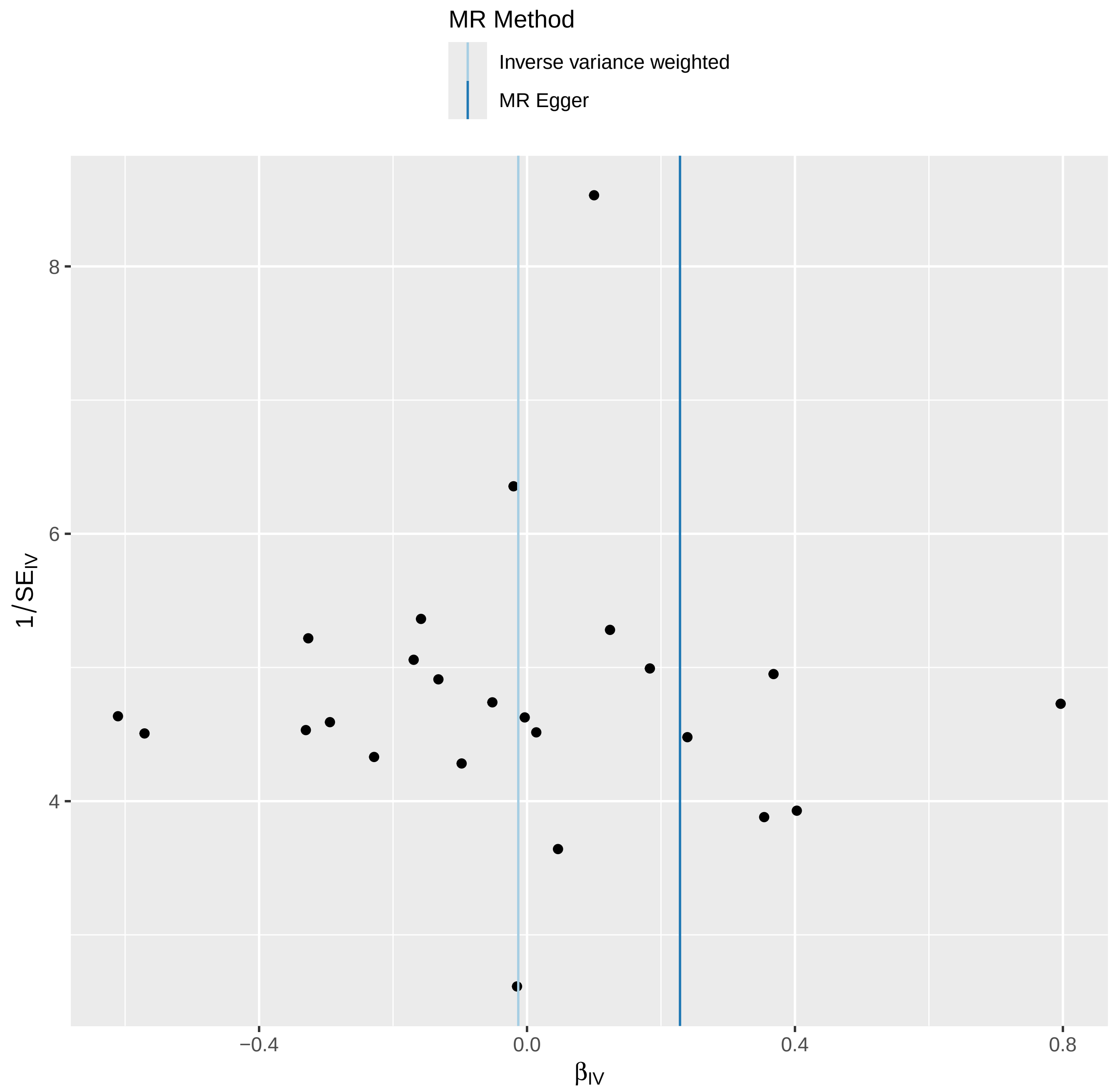

MR Method
Inverse variance weighted
MR Egger
8
6
4
1/SE_IV
−0.4
0.0
0.4
0.8
β_IV

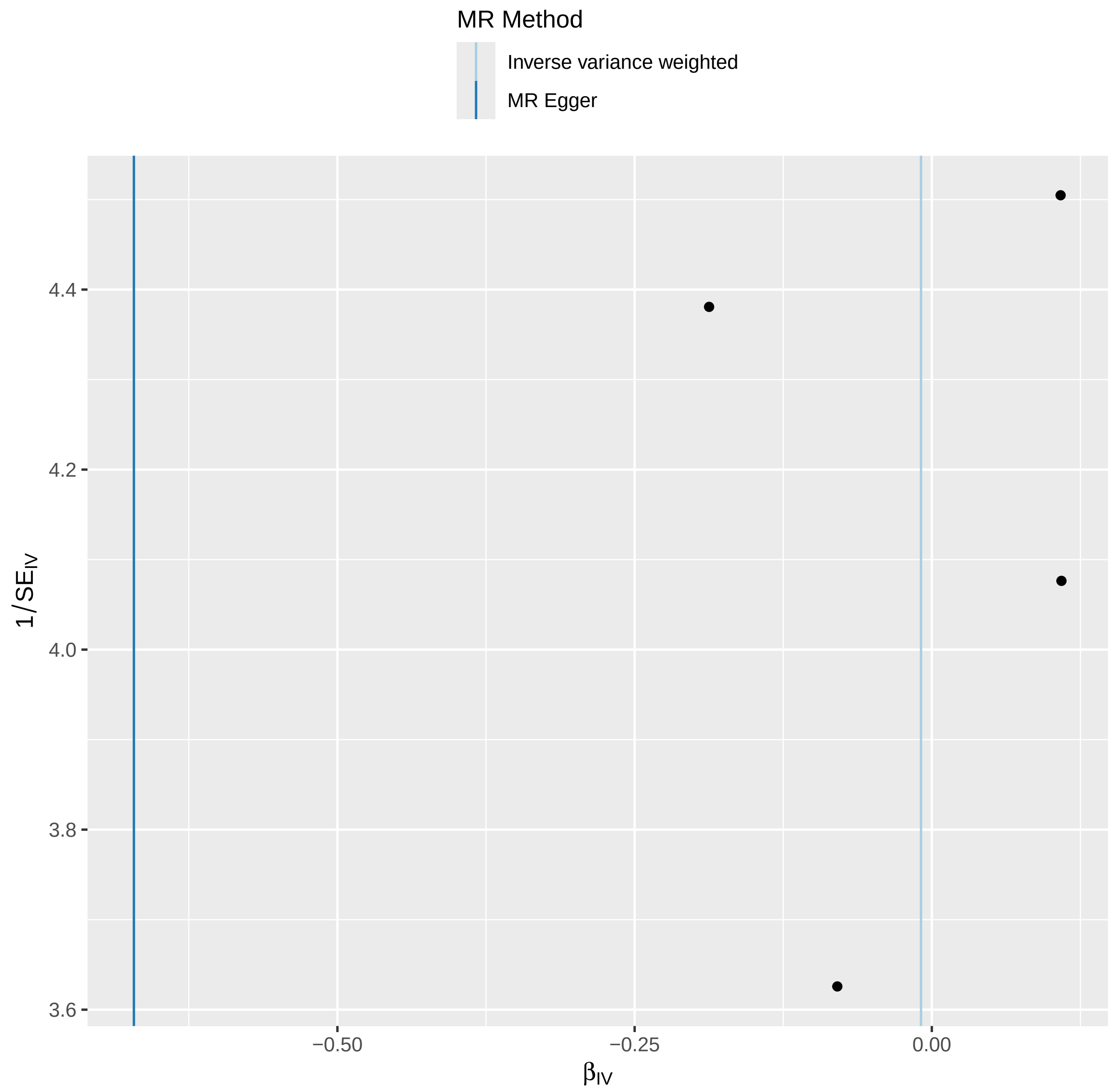
MR Method
Inverse variance weighted
MR Egger
4.4
4.2
4.0
3.8
3.6
1/SE_IV
−0.50
−0.25
0.00
β_IV

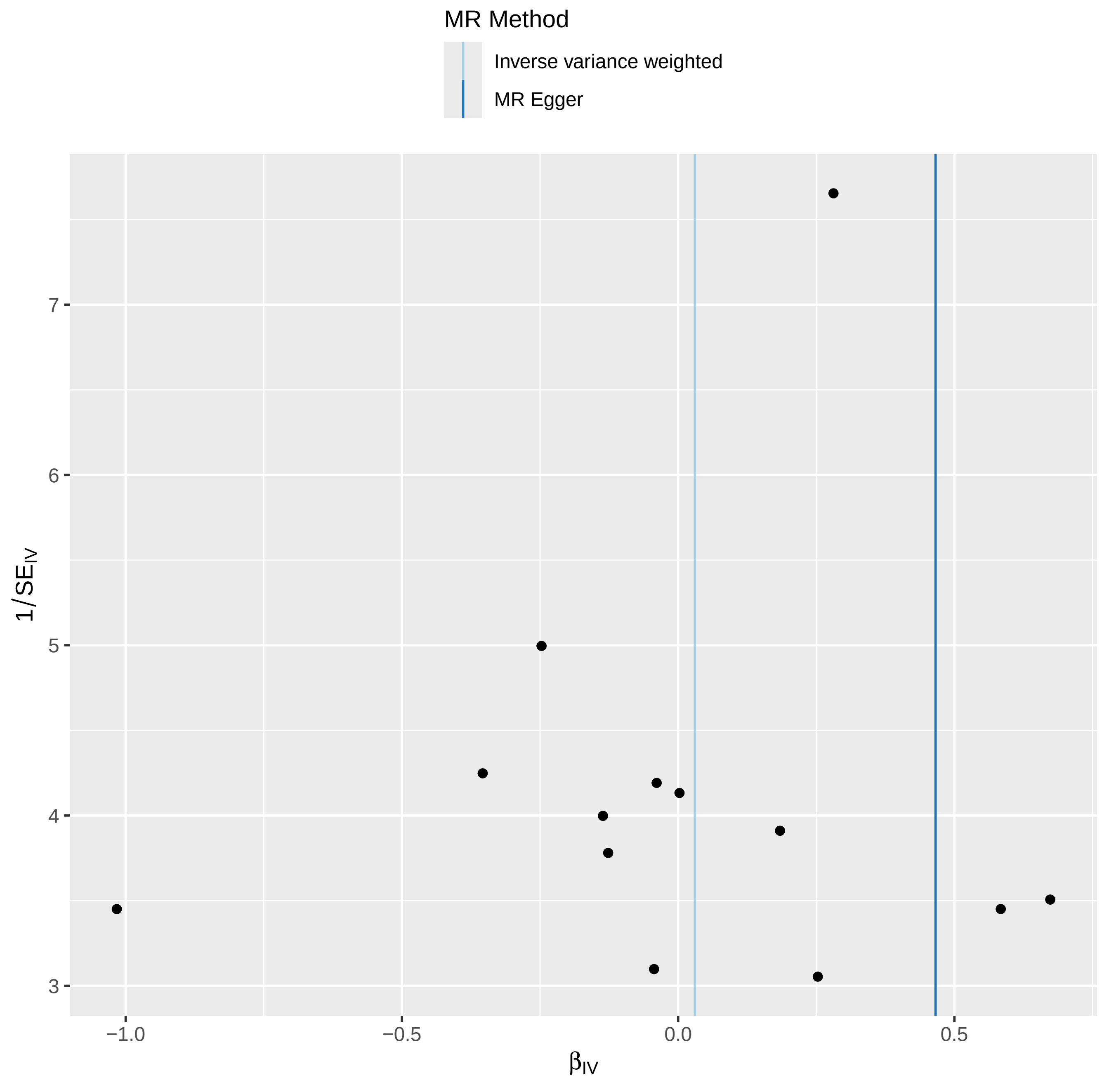

MR Method
Inverse variance weighted
MR Egger
7
6
5
4
3
1/SE$_{IV}$
−1.0
−0.5
0.0
0.5
β$_{IV}$

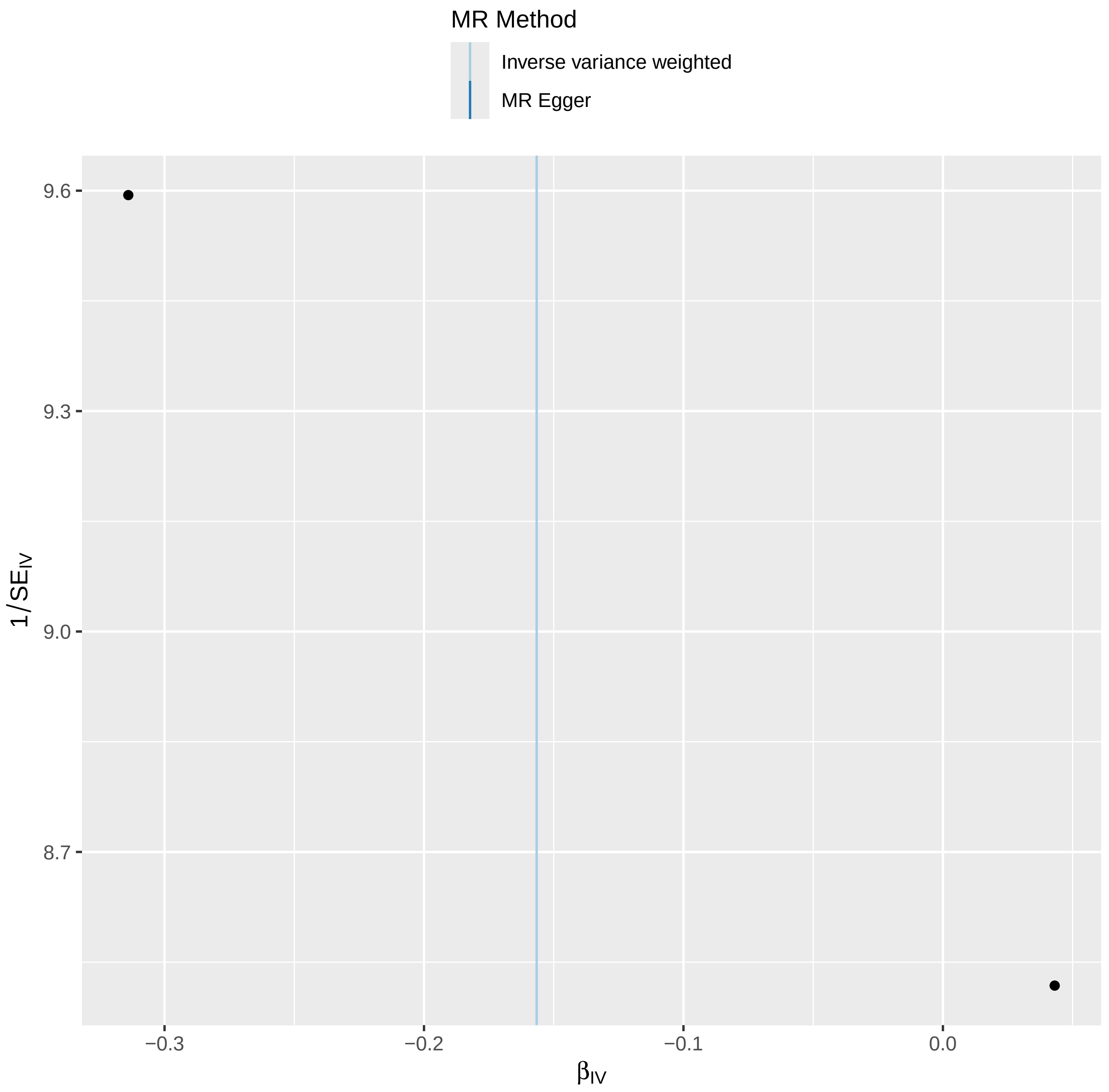
MR Method
Inverse variance weighted
MR Egger
9.6
9.3
9.0
8.7
1/SE$_{IV}$
−0.3
−0.2
−0.1
0.0
β$_{IV}$

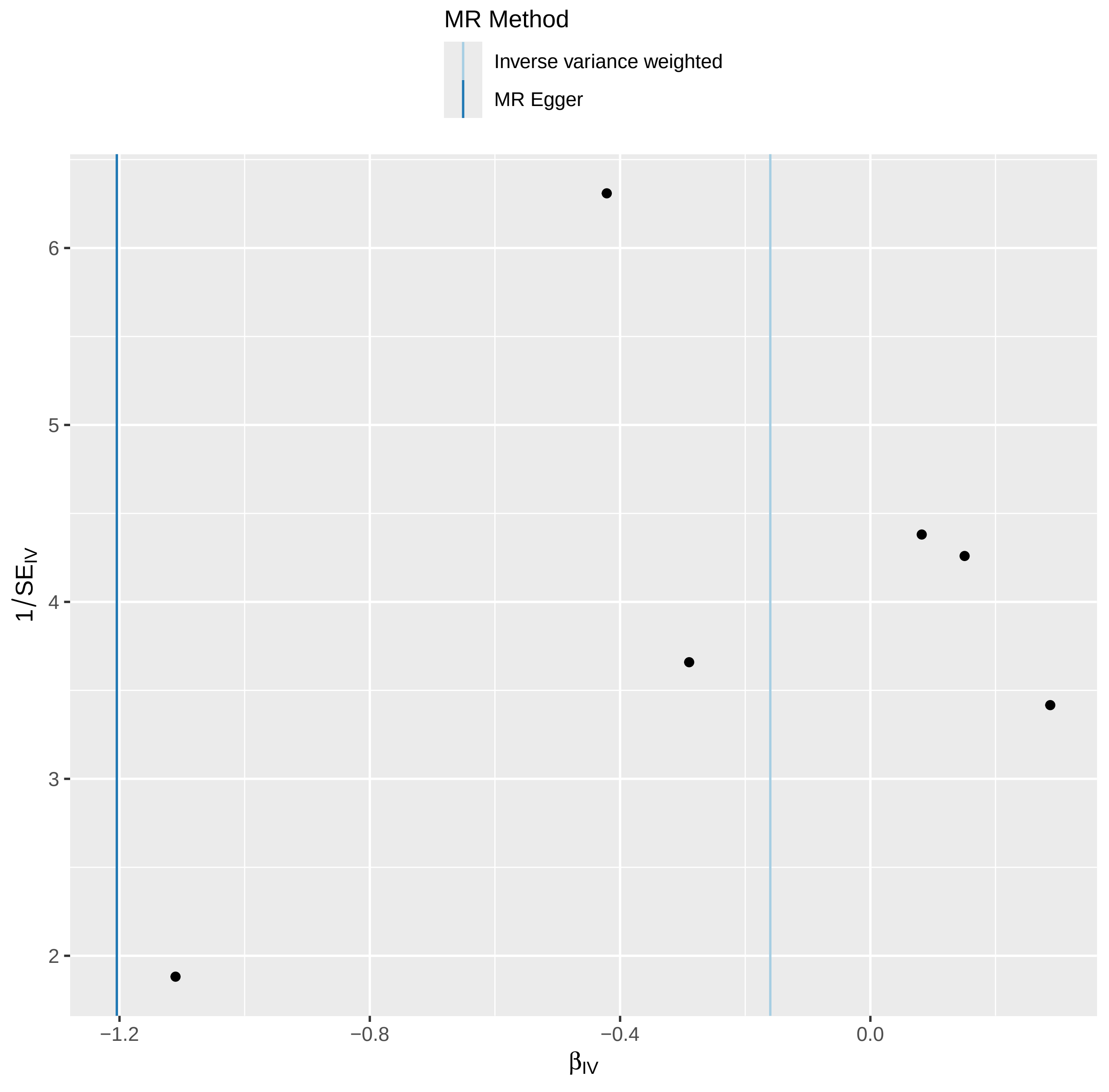
MR Method
Inverse variance weighted
MR Egger
1/SE_IV
β_IV
6
5
4
3
2
−1.2
−0.8
−0.4
0.0

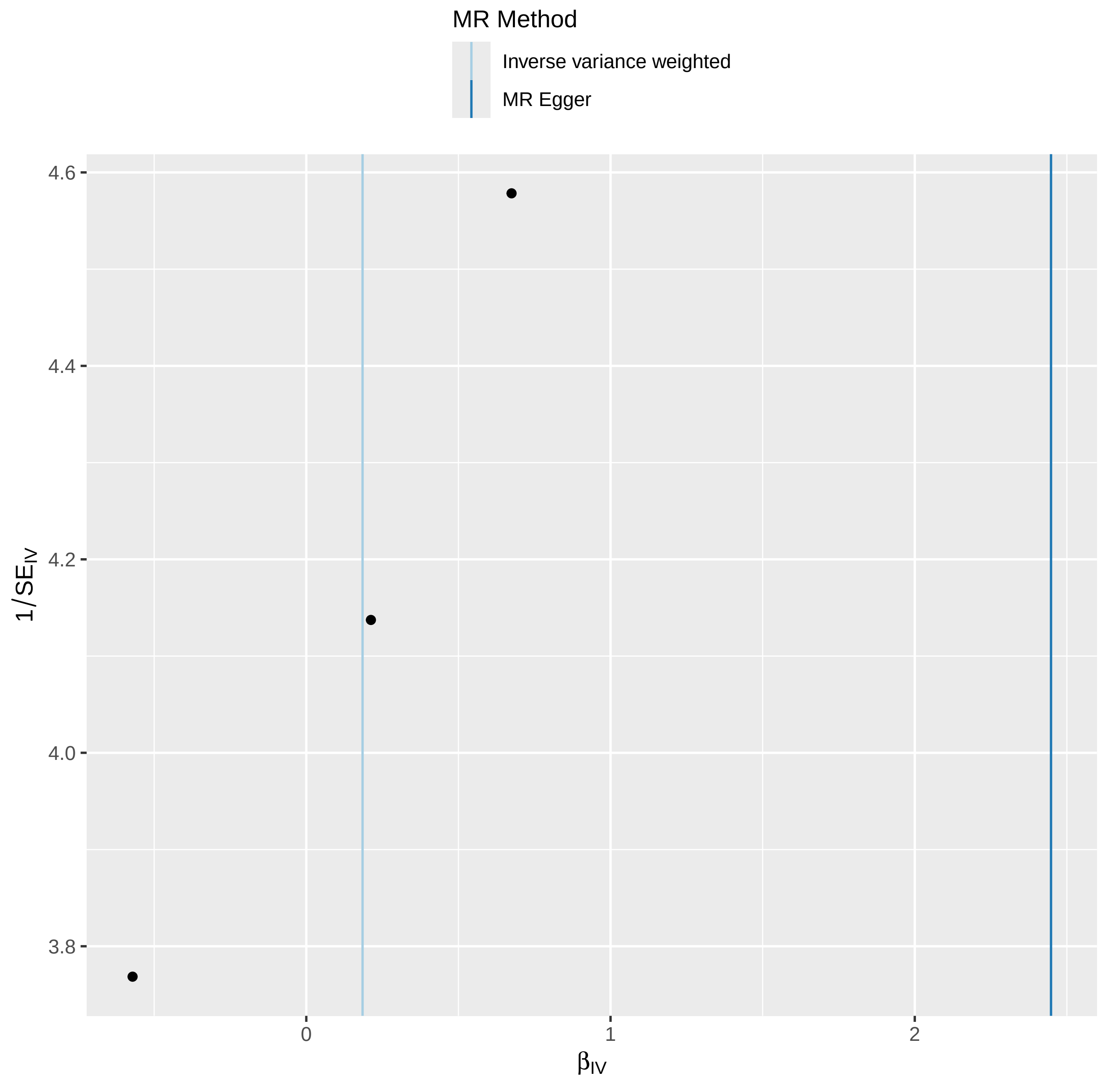

MR Method
Inverse variance weighted
MR Egger
4.6
4.4
4.2
4.0
3.8
1/SE_IV
0
1
2
β_IV

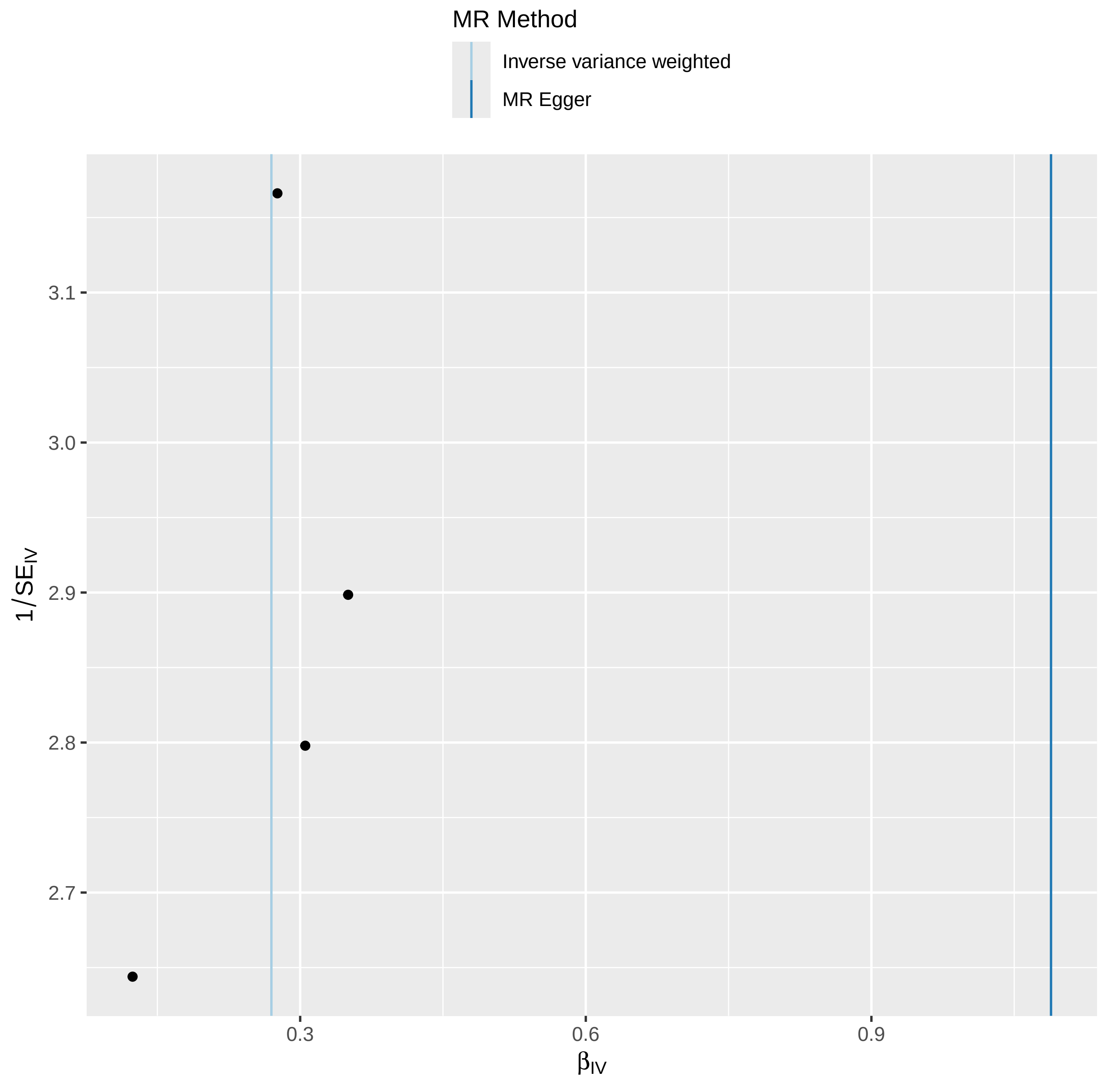
MR Method
Inverse variance weighted
MR Egger
3.1
3.0
2.9
2.8
2.7
1/SE_IV
0.3
0.6
0.9
β_IV

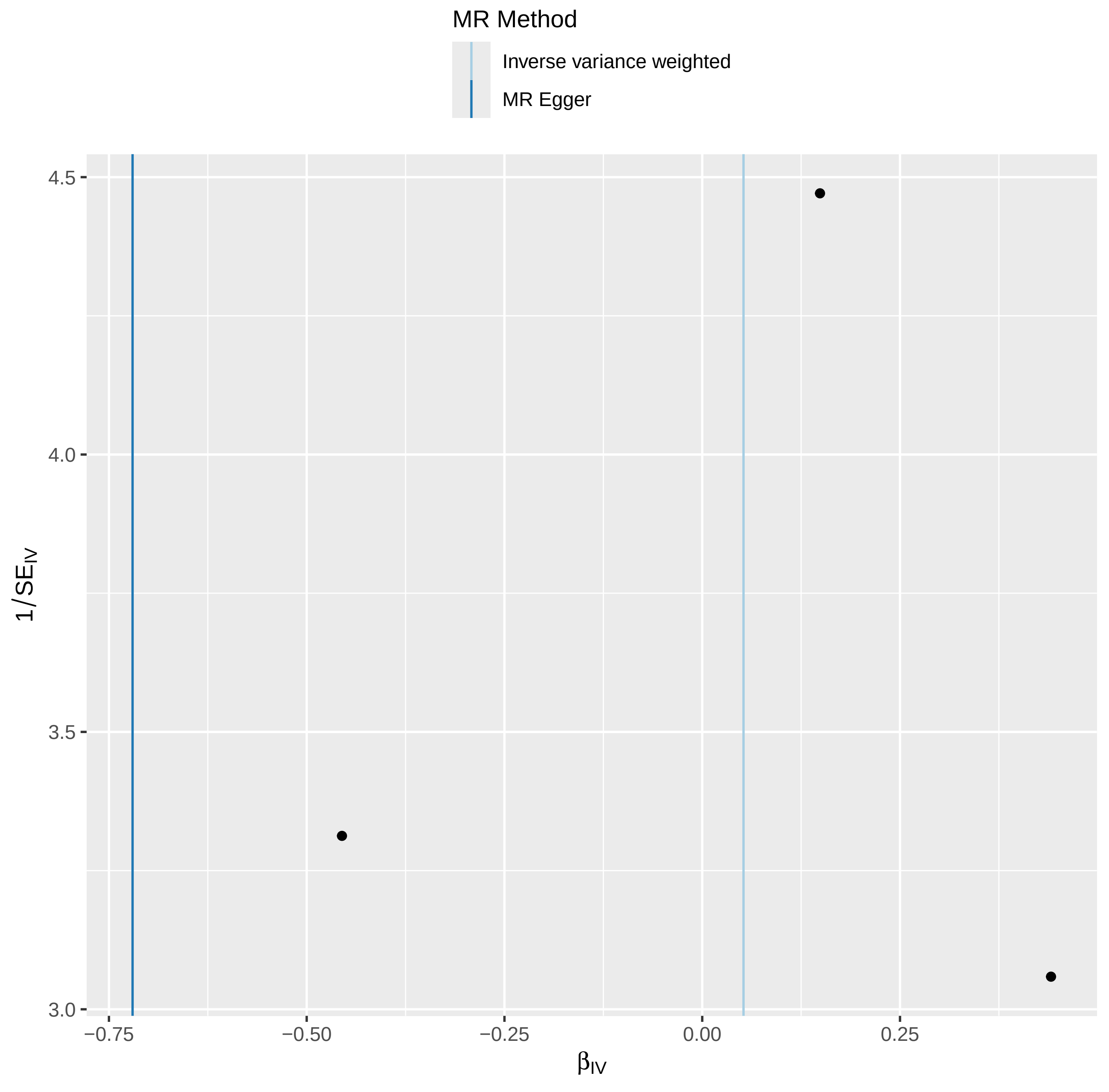

MR Method
Inverse variance weighted
MR Egger
4.5
4.0
3.5
3.0
−0.75
−0.50
−0.25
0.00
0.25
β_{IV}
$1/SE_{IV}$

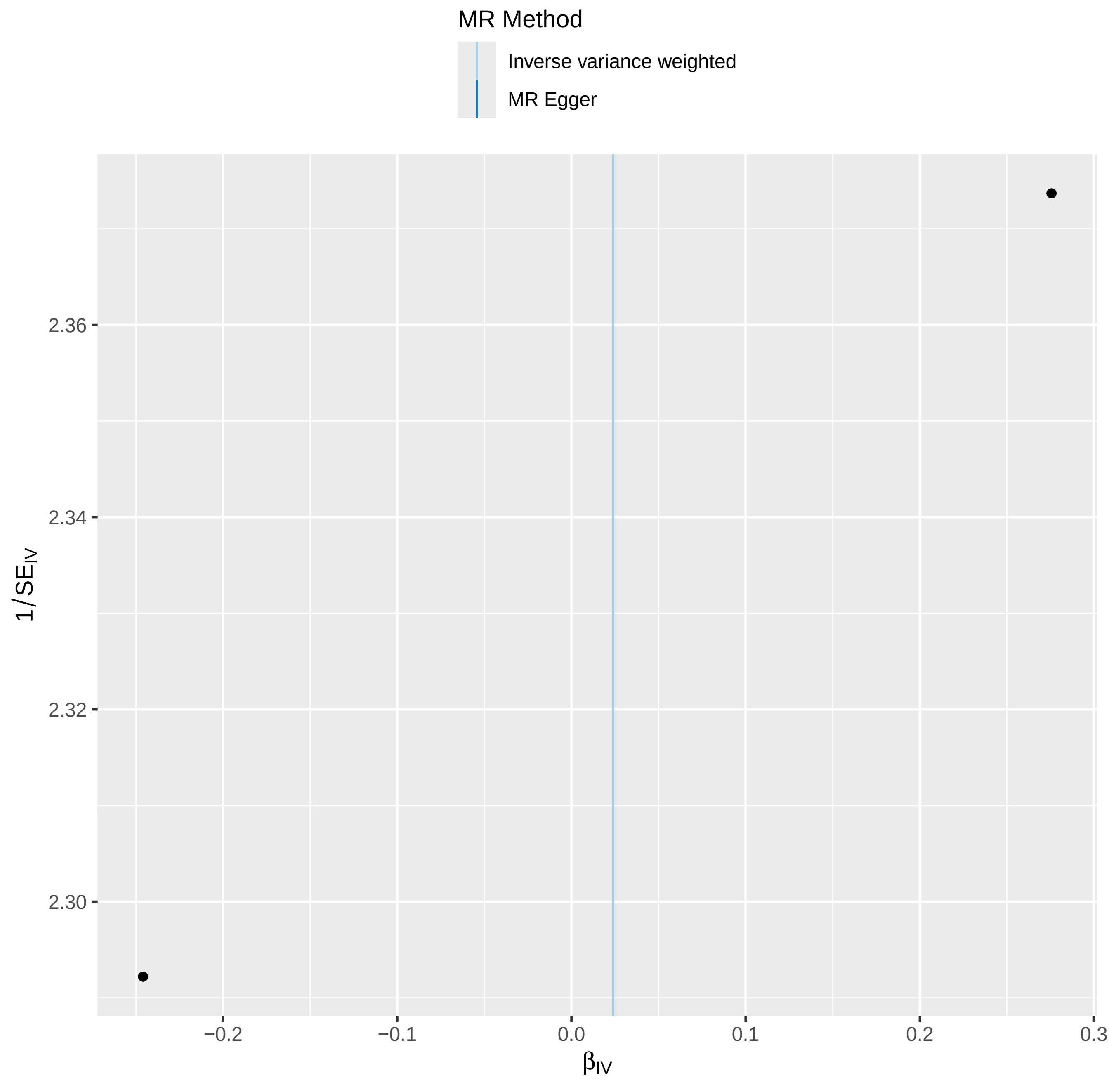
MR Method
Inverse variance weighted
MR Egger
2.36
2.34
2.32
2.30
1/SE_IV
−0.2
−0.1
0.0
0.1
0.2
0.3
β_IV

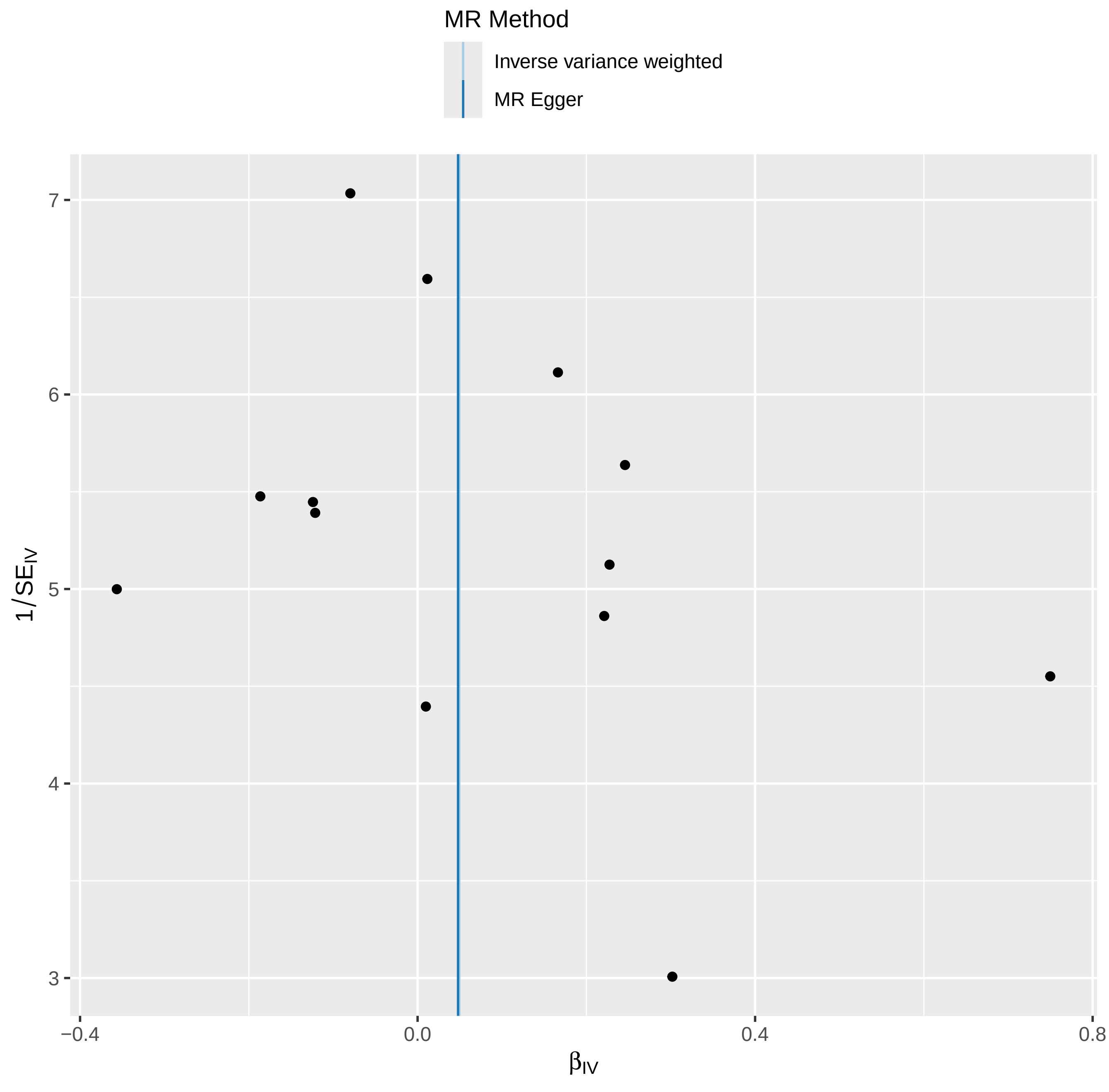
MR Method
Inverse variance weighted
MR Egger
7
6
5
4
3
1/SE$_{IV}$
−0.4
0.0
0.4
0.8
β$_{IV}$

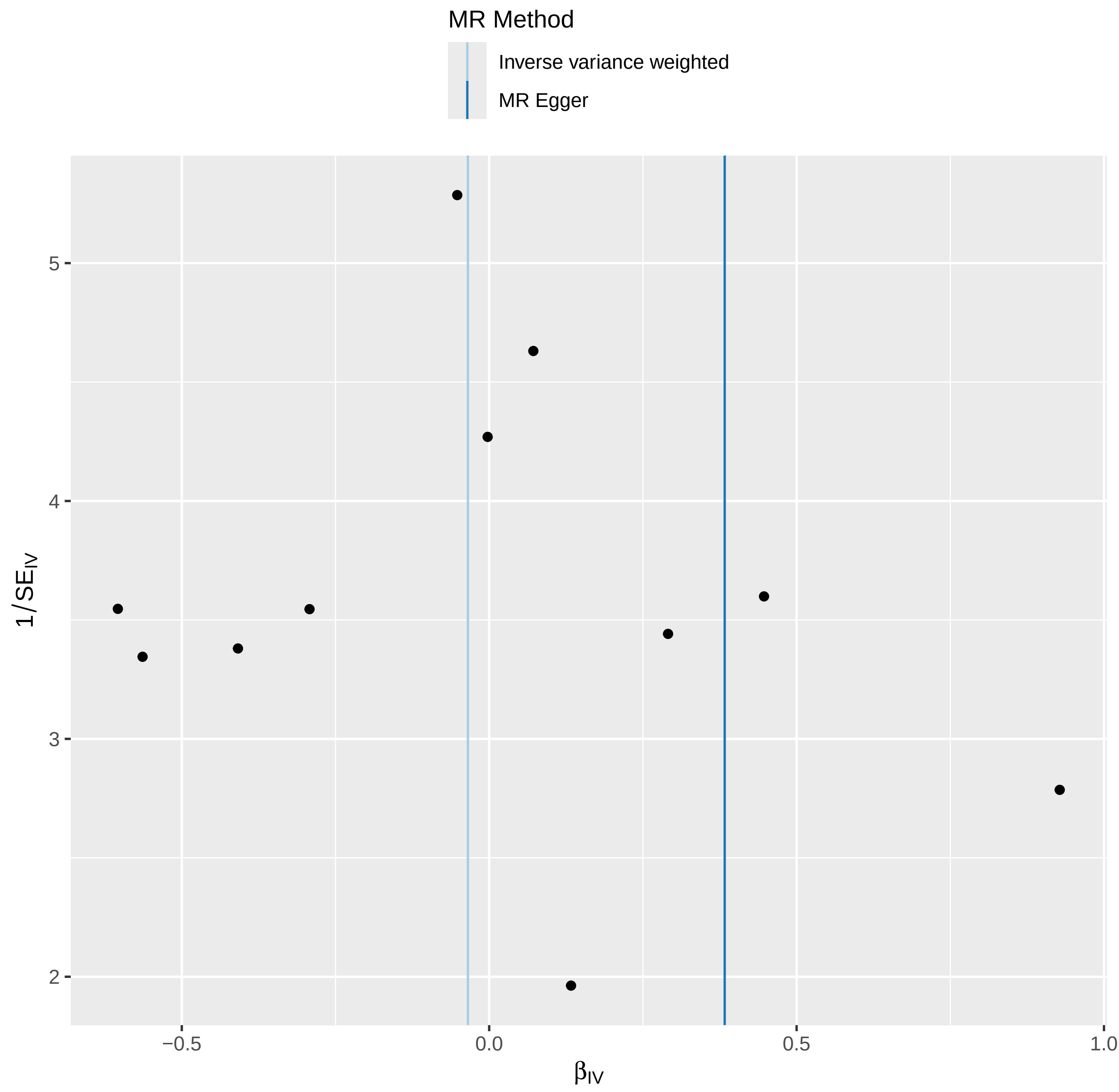

MR Method
Inverse variance weighted
MR Egger
5
4
3
2
$1/SE_{IV}$
−0.5
0.0
0.5
1.0
β_{IV}

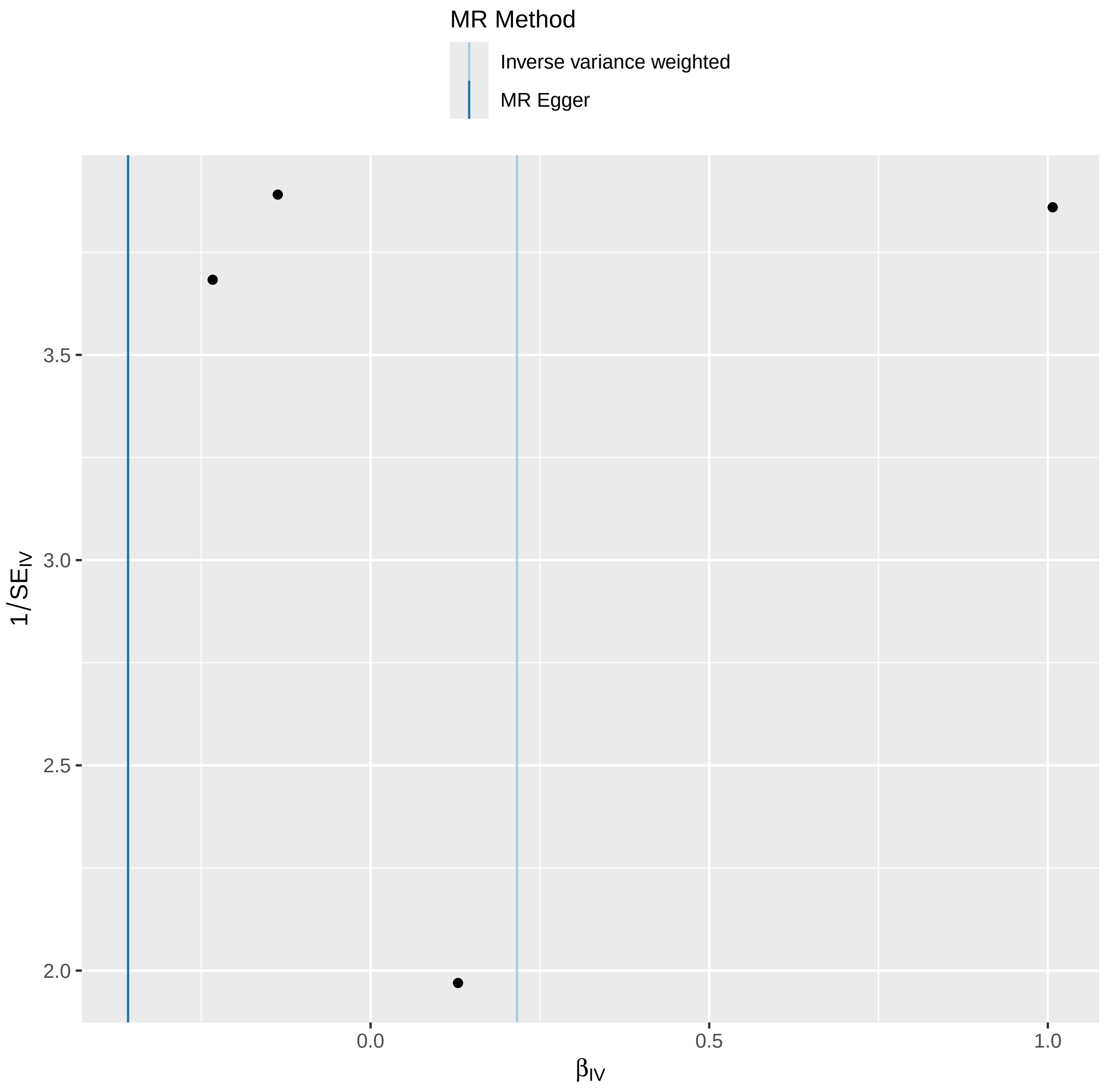
MR Method
Inverse variance weighted
MR Egger
3.5
3.0
2.5
2.0
$1/SE_{IV}$
0.0
0.5
1.0
β_{IV}

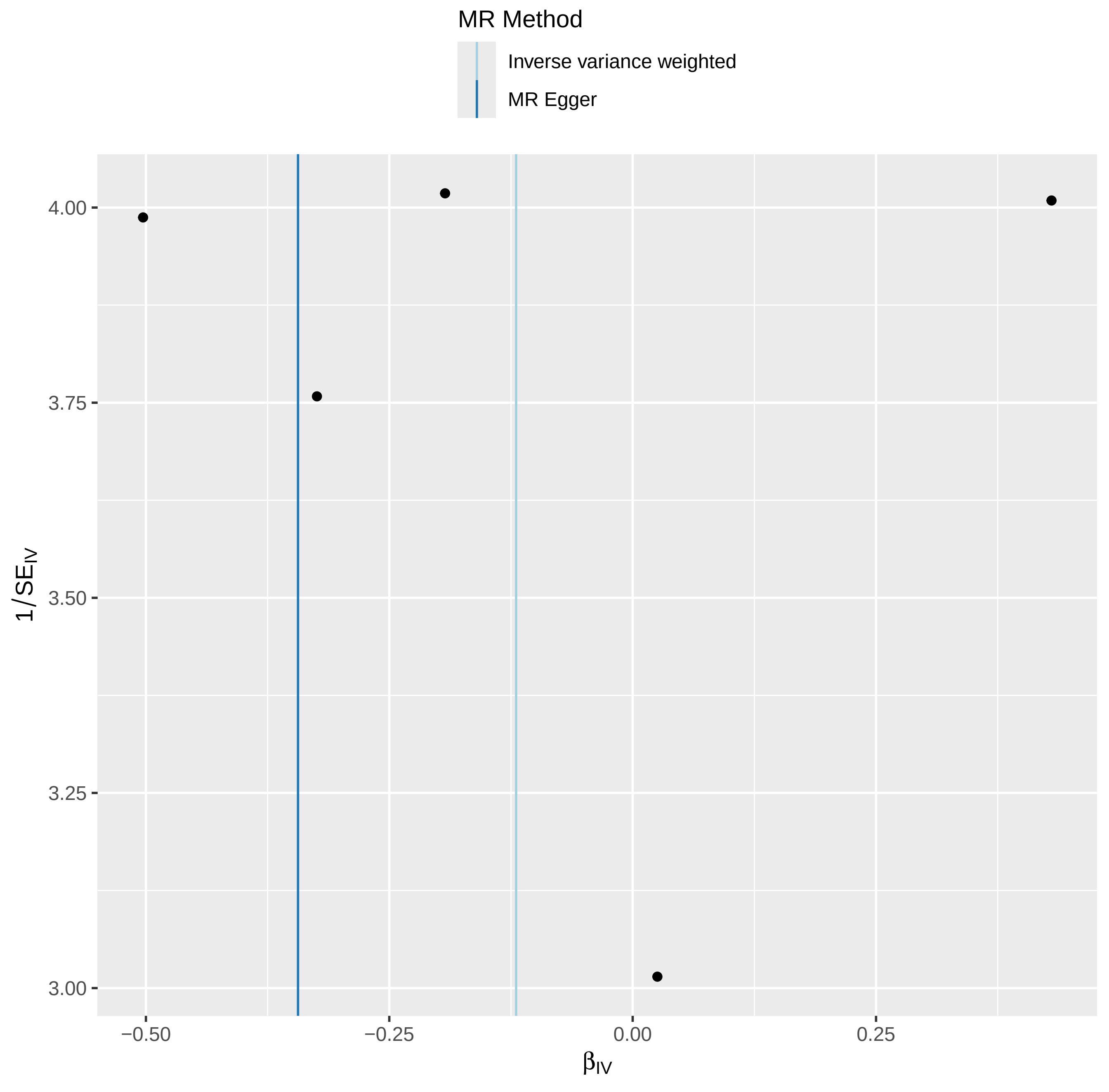

MR Method
Inverse variance weighted
MR Egger
4.00
3.75
3.50
3.25
3.00
1/SE$_{IV}$
−0.50
−0.25
0.00
0.25
β$_{IV}$

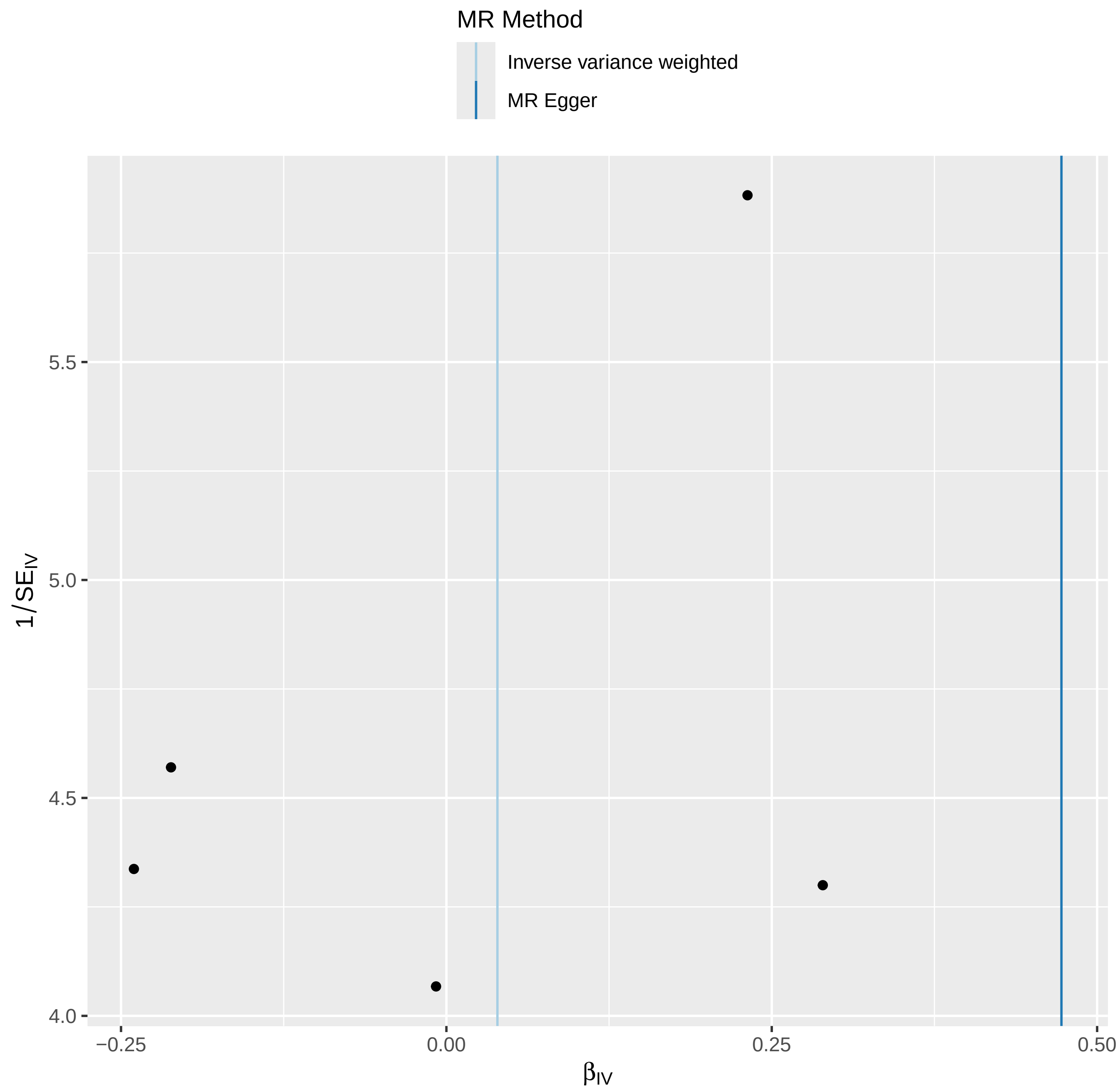
MR Method
Inverse variance weighted
MR Egger
5.5
5.0
4.5
4.0
$1/SE_{IV}$
−0.25
0.00
0.25
0.50
β_{IV}

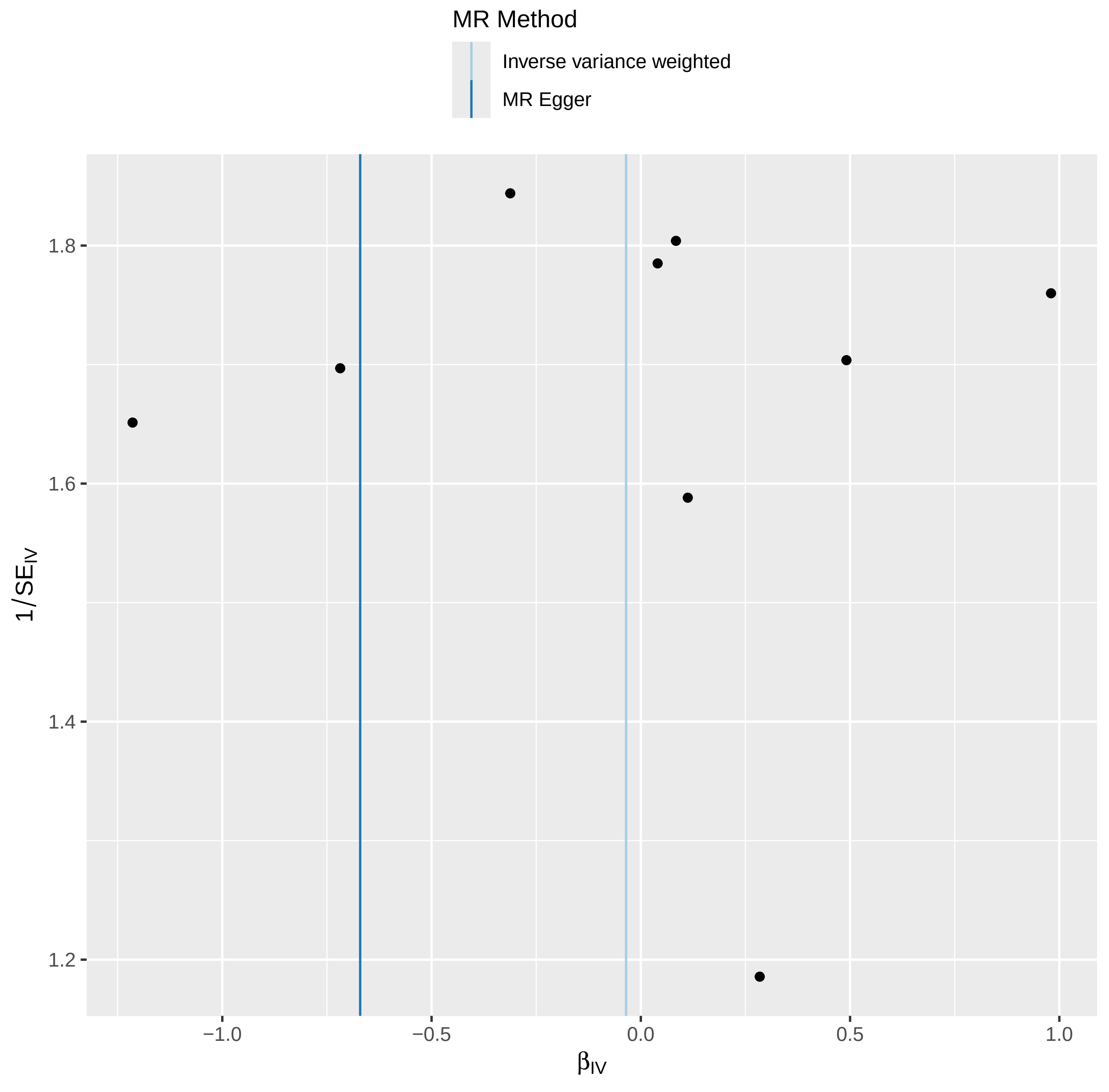

MR Method
Inverse variance weighted
MR Egger
1.8
1.6
1.4
1.2
$1/SE_{IV}$
−1.0
−0.5
0.0
0.5
1.0
β_{IV}

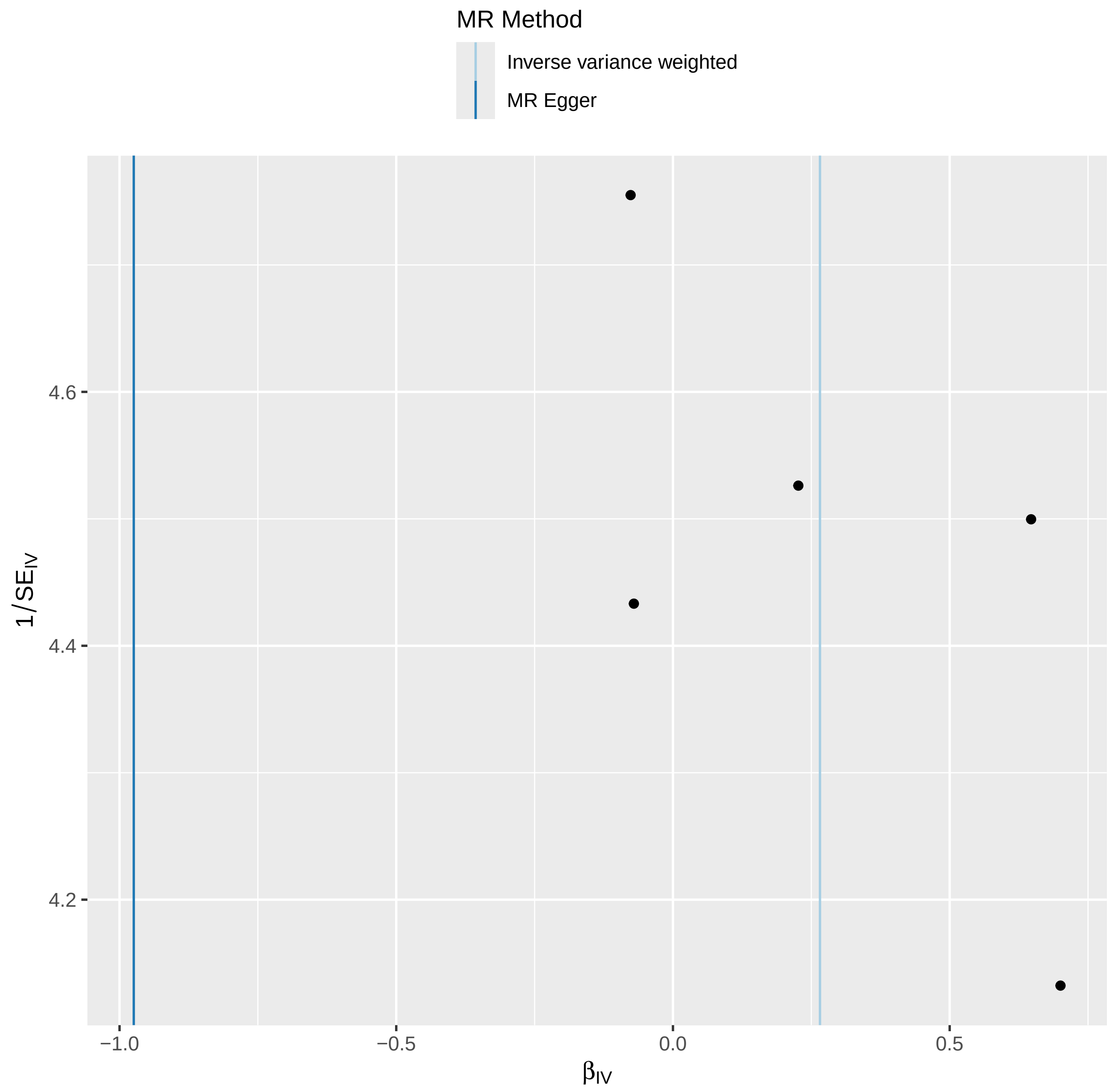
MR Method
Inverse variance weighted
MR Egger
4.6
4.4
4.2
1/SE_IV
−1.0
−0.5
0.0
0.5
β_IV
